# Data-Driven Discovery of Extravasation Pathway in Circulating Tumor Cells

**DOI:** 10.1038/srep43710

**Published:** 2017-03-06

**Authors:** S. Yadavalli, S. Jayaram, S. S. Manda, A. K. Madugundu, D. S. Nayakanti, T. Z. Tan, R. Bhat, A. Rangarajan, A. Chatterjee, H. Gowda, J. P. Thiery, P. Kumar

**Affiliations:** 1 Institute of Bioinformatics, International Technology Park, Whitefield, Bangalore, 560 066, India.; 2 Manipal University, Madhav Nagar, Manipal, 576104, India; 3 Center for Bioinformatics, Pondicherry University, Puducherry 605 014, India; 4 Cancer Science Institute of Singapore, National University of Singapore, Centre for Translational Medicine NUS Yong Loo Lin School of Medicine, Singapore 117599, Singapore; 5 Department of Molecular Reproduction, Development and Genetics, Indian Institute of Science, Bangalore, 560012, India; 6 YU-IOB Center for Systems Biology and Molecular Medicine, Yenepoya University, Mangalore, 575018, India; 7 Comprehensive Cancer Center, Institut Gustave Roussy, 114 Rue Edouard Vaillant, 94805 Villejuif, France.; 8 CNRS UMR 7057, Matter and Complex Systems, Université Paris Diderot, 10 rue Alice Domon et Léonie Duquet 75013 Paris, France; 9 Department of Biochemistry, National University of Singapore, Singapore 117597, Singapore.

## Abstract

Circulating tumor cells (CTCs) play a crucial role in cancer dissemination and provide a promising source of blood-based markers. Understanding the spectrum of transcriptional profiles of CTCs and their corresponding regulatory mechanisms will allow for a more robust analysis of CTC phenotypes. The current challenge in CTC research is the acquisition of useful clinical information from the multitude of high-throughput studies. To gain a deeper understanding of CTC heterogeneity and identify genes, pathways and processes that are consistently affected across tumors, we mined the literature for gene expression profiles in CTCs. Through in silico analysis and the integration of CTC-specific genes, we found highly significant biological mechanisms and regulatory processes acting in CTCs across various cancers, with a particular enrichment of the leukocyte extravasation pathway. This pathway appears to play a pivotal role in the migration of CTCs to distant metastatic sites. We find that CTCs from multiple cancers express both epithelial and mesenchymal markers in varying amounts, which is suggestive of dynamic and hybrid states along the epithelial-mesenchymal transition (EMT) spectrum. Targeting the specific molecular nodes to monitor disease and therapeutic control of CTCs in real time will likely improve the clinical management of cancer progression and metastases.

Nearly 90% of all cancer deaths are attributed to distant tumor metastasis^1^. Metastasis is a multi-step process involving cancer cell invasion into the stroma, intravasation into lymph and blood vessels, survival within the circulation, extravasation, and finally, colonization and proliferation at a secondary organ site^2^. Circulating tumor cells (CTCs), originating from primary or secondary tumors, are a key component of the metastatic cascade and are considered an important indicator of prognosis and cancer relapse. Recent findings suggest that CTCs can serve as non-invasive ‘liquid biopsies’, allowing clinicians to interrogate the tumor burden and early metastatic events, evaluate treatment response, predict patient sensitivity or resistance to therapies^3^, and provide a way to assess tumor heterogeneity; these collective benefits also point to a more personalized treatment strategy in early stages of the disease^4^,^5^. Understanding the role of CTCs in disease progression, however, is challenging, as CTCs are believed to exhibit a wide range of epithelial-mesenchymal transition (EMT) phenotypes^6^,^7^; albeit, there is only limited information available with respect to the role of EMT in CTCs^8^ and much of the work addressing EMT regulation has been carried out on cultured cells^7^.

It has been suggested that cancer cell extravasation may have been co-opted from leukocyte extravasation^9^, a well-documented phenomenon involving dynamic interactions between circulating leukocytes and endothelial cells lining the vasculature^10^; although, with some differences. Unlike leukocytes, which express the β2-integrins that bind directly to intercellular adhesion molecule 1 (ICAM1) receptors, cancer cells do not express β2-integrins but use leukocytes or other blood cells as bridge- or linker-cells^9^. Specifically, cancer cells are thought to colonize inflammatory pre-metastatic niches in secondary organs^11^. Extravasation at these sites may be controlled by E-selectins; cell adhesion molecules expressed on endothelial cells^12^. Further, cancer cell extravasation and possible heterotypic intercellular communication between cancer cells and endothelial cells may be influenced by interactions with molecules such as N-cadherin or galectin-3 on endothelial cells^9^.

Carcinoma cell invasion and migration during metastasis is also influenced by the transition of epithelial cells into motile mesenchymal cells, a process known as epithelial-mesenchymal transition (EMT)^13^. EMT is driven by conserved molecular and cellular developmental programs, which, too, appear to have been hijacked by carcinoma cells to initiate the metastatic cascade^14^. Primary tumors display considerable phenotypic heterogeneity, with carcinoma cells engaged in various stages of EMT. Recent work has confirmed the presence of CTCs with an intermediate or “hybrid” EMT status, and these are believed to confer cells their metastatic potential^15^,^16^,^17^. In our efforts to understand these intermediate states, we previously established an EMT scoring system to estimate EMT dynamics, which ranks the degree of EMT^18^. Yet, this scoring method is still unable to describe the intratumoral heterogeneity of individual cancer cells, including those destined to disseminate, such as CTCs. Experimental data and advanced bioinformatics analyses may help to uncover the role of EMT in systemic transport and extravasation. Few pathway-based approaches have characterized the differential expression of genes that promote metastasis in CTCs^19^,^20^,^21^,^22^. Knowledge of the gene expression signatures in CTCs and reconstruction of the inherent CTC signaling network could offer a valuable resource for further functional exploitation, such as in the design of targeted therapies, and in the identification of potential markers to monitor tumor progression and the initial lodging of CTCs in distant organs. This information may ultimately offer a new perspective to target CTCs to prevent tumor cell dissemination and metastasis^23^.

In the present study, we collected, reanalyzed and mapped the gene expression data from several published studies in CTCs to the signaling pathways and networks available to date in an attempt to reveal new insights into CTC signaling. We integrated the transcriptomic changes observed in CTCs across various cancer types to identify with high confidence putative candidate markers involved in extravasation of CTCs. The EMT spectrum was scored for the first time in CTCs from transcriptome datasets to reveal their EMT profile across cancers. The study unfolds some key biological processes that are inherent to these circulating cells.

## Results and Discussion

**Figure 1:**
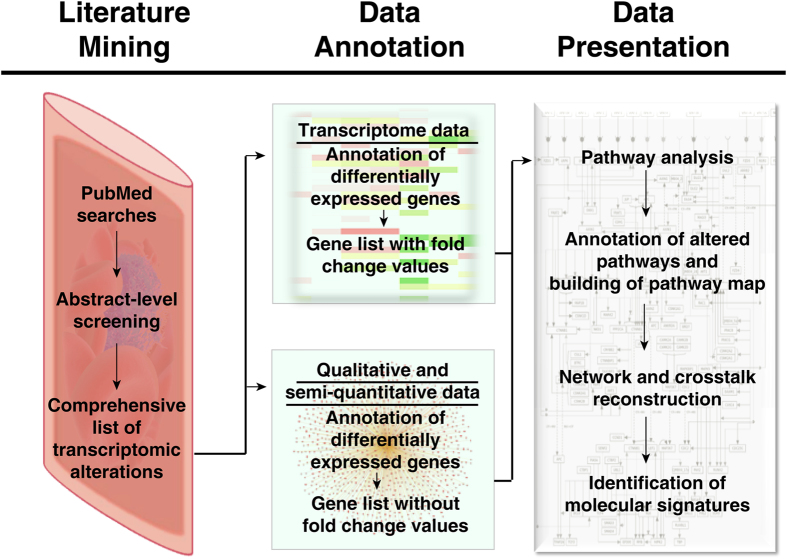
Schematic of the workflow employed for filtering and integration of data to identify molecular signatures in circulating tumor cells.

CTC phenotying and genotyping studies have expanded our knowledge on tumor progression and tumor evolution^24^,^25^. Though challenging, the emerging clinical applications of CTCs in cancer detection and disease management are indisputable^26^. We sought to explore unresolved biological and functional facets of CTCs and understand their molecular underpinnings in a clinical context. To this end, we collated and integrated data from gene expression profiles of CTCs within the existing literature, as depicted in the workflow in Fig. 1. Our literature survey using a relevant keyword search in PubMed resulted in 3,242 publications (Supplementary Fig. 1a,b). This was further screened and filtered to a total of 98 papers that were annotated for gene expression data in CTCs [see Methods]. Differentially expressed genes (DEGs), taken from qualitative and semi-quantitative experiments, resulted in a gene list of 7,572 molecules (denoted as ‘CTC_ALL’; Supplementary Table S1). Out of these, a list of 7,209 differentially expressed genes from large-scale transcriptomics studies from five cancers were compiled separately (denoted as ‘CTC_FC’; Supplementary Table S2; Supplementary Fig. 1c). Fold change (FC) values for gene expression in CTCs were available only for the CTC_FC list^19^,^20^,^27^,^28^,^29^ (Table 1). Despite major advances in CTC isolation techniques, obtaining pure CTCs from peripheral blood is still challenging. We leveraged expression data from large-scale transcriptomics studies that reported DEGs in CTCs and compiled our gene list for molecular characterization of CTCs predominantly from five such studies based on the quality of their isolation techniques. These five studies have used one or more advanced techniques to overcome the problem of contamination of CTC isolates with hematological and non-viable cells, almost successfully. The details of isolation techniques used to obtain relatively pure CTC populations for these five studies have been compiled in Supplementary Table S3. Improved isolation methods to yield pure populations of CTCs will unfold new opportunities for global gene expression analysis and molecular characterization.

**Figure 2:**
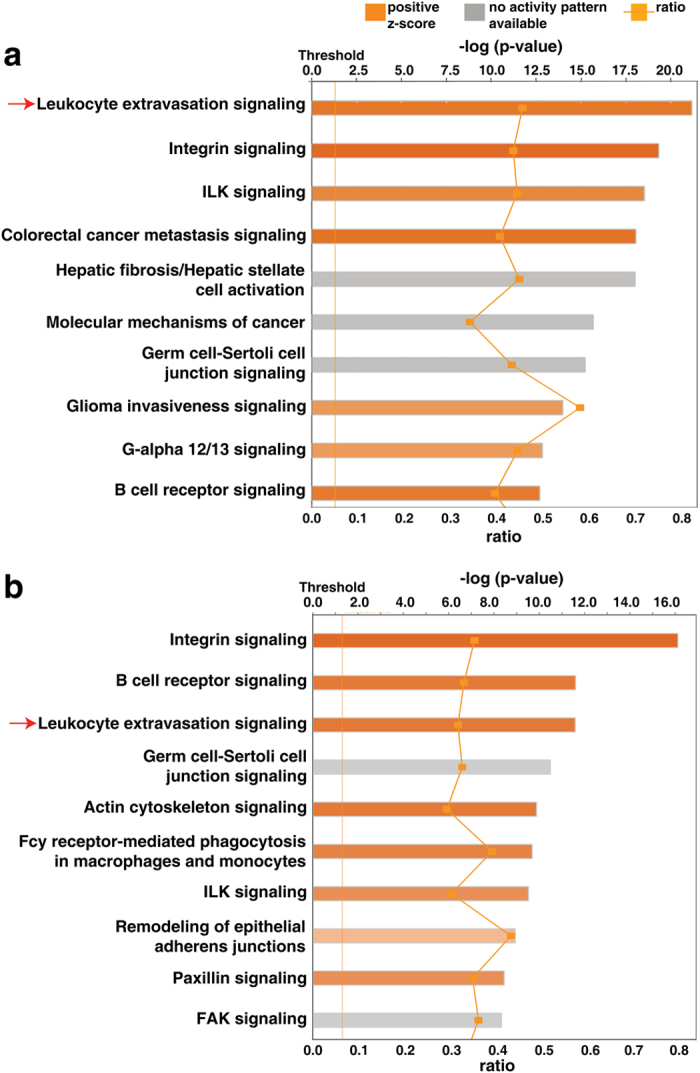
Ingenuity Pathway Analysis (IPA) to identify significant canonical pathways in CTCs. Top ten canonical pathways associated with genes from (**a**) CTC_ALL dataset and (**b**) CTC_FC dataset, as shown by IPA pathway analysis. Pathways identified are represented on the y-axis. The x-axis corresponds to the –log of the P-value (Fisher’s exact test) and the orange points on each pathway bar represent the ratio of the number of genes in a given pathway that meet the cutoff criteria, divided by the total number of genes that map to that pathway.

Ingenuity Pathway Analysis (IPA) of the overexpressed genes (*n* = 3,251) in the CTC_ALL dataset showed significant enrichment of the ‘Leukocyte Extravasation Pathway’ at 44.9% (92/205 molecules associated with ‘Leukocyte Extravasation Signaling’ in IPA; *p* = 6.81E-22; *z*-score = 7.180). The top canonical pathways with a positive *z*-score included ‘Integrin Signaling’ (*p* = 4.69E-20; *z*-score = 8.041), ‘ILK Signaling’ (*p* = 3E-19; *z*-score = 6.268), ‘Colorectal Cancer Metastasis Signaling’ (*p* = 8.96E-19; *z*-score = 7.507), ‘Glioma Invasiveness Signaling’ (*p* = 1.56E-12; *z*-score = 4.7), ‘Gα12/13 Signaling’ (*p* = 1.44E-13; *z*-score = 5.259) and ‘B Cell Receptor Signaling’ (*p* = 2E-13; z-score = 6.862) pathways. These pathways are involved in cell migration, adhesion, growth and differentiation, inhibiting apoptosis and enabling cell survival^30^,^31^,^32^,^33^,^34^,^35^,^36^. The top predicted upstream regulator was *TGFβ1(p* = 2.99E-39), and the IPA gene view indicated *TGFβ1* as the upstream regulator of many genes that play a vital role in cancer progression, such as *SERPINE1, ACTA2, CDKN1A, SMAD3, SMAD2, CTGF, FN1, CDH1, SMAD7, COL1A1, FOXP3, CDKN2B, COL1A2, VIM*. The top diseases were ‘Cancer’ (total no. of associated molecules = 2,871) and ‘Organismal Injury and Abnormalities’ (2,886), both with a *p*-value range of 2.53E-08 to 5.08E-33. The top molecular functions were ‘Cell Death and Survival’ (908; *p*-value range, 1.39E-08 to 1.42E-61) with a predicted decrease in cell death and predicted increase in cell survival, and ‘Cellular Growth and Proliferation’ (941; *p*-value range, 1.3E-08 to 7.19E-55). Similarly, IPA analysis of the CTC_FC dataset of overexpressed genes (*n* = 2,652) positioned the leukocyte extravasation pathway as third in the rank of enrichment (*p* = 2.6E-12; *z*-score: 6.260). The top ten enriched canonical pathways associated with each dataset are illustrated in Fig. 2.

### CTCs express leukocyte extravasation drivers

Although comparatively less is known about cancer cell extravasation as compared with that of leukocytes, previous studies suggest a similar process might be operating in CTCs^9^,^37^,^38^. Indeed, there is now ample evidence that CTCs may use similar localization/homing signals to that of leukocytes, such as chemokine receptors [stromal cell-derived factor (SDF1/*CXCL12*) and C-X-C motif chemokine receptor 4 (*CXCR4*)] and cytokines [suppressor of cytokine signaling 1 (*SOCS1*)], to guide them to distant metastatic sites to establish new tumors. Furthermore, the expression of these markers has been correlated with the presence of CTCs in peripheral blood and metastasis^39^,^40^,^41^.

**Figure 3:**
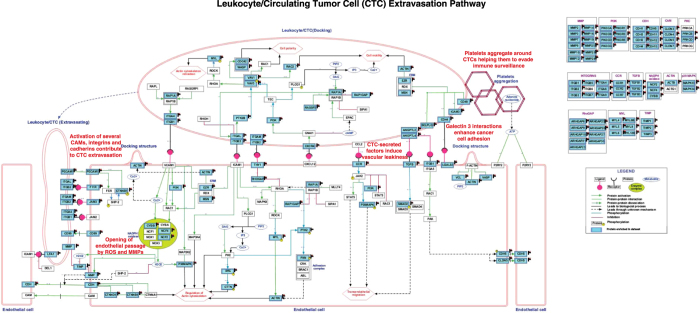
Enriched map of the leukocyte extravasation pathway in CTCs. The pathway diagram illustrates CTC/Leukocyte extravasation through the vascular endothelial lining. The upregulated molecules enriched among CTC_ALL dataset are highlighted (blue). The leukocyte extravasation pathway was built using the KEGG^96^,^97^ pathway (hsa04670) as a template and further appended with reactions represented in IPA (IPA, Qiagen, Redwood City, http://www.ingenuity.com/) pathway resource and extravasation-associated molecular interactions reported in the literature for CTCs. The interactions and their relevant references (PubMed IDs) are tabulated in Supplementary Table S4.

The leukocyte extravasation pathway (LEP) is a reasonably well characterized, multi-step process of leukocyte emigration from the blood into tissues through the vascular endothelium and is vital for immune surveillance and inflammation^42^. To explore this further, we expanded the annotated leukocyte extravasation pathway map and built a complete map of the leukocyte/CTC extravasation pathway by integrating information from current pathway resources, review articles and other literature on CTCs (Supplementary Table S4). The entities in our dataset were then mapped to the expanded pathway using the GeneSpring Pathway Architect software tool (version 13.0). The enriched map of the Leukocyte/CTC extravasation pathway generated is shown in Fig. 3. The pathway map illustrates all the upregulated molecules in our datasets (shown in blue colored boxes) that have been identified in CTCs across different cancers that may use a common mode of extravasation and metastatic colonization. The mechanistic roles of the different molecules identified and enriched in the integrated leukocyte/CTC pathway are highlighted and discussed below in greater detail.

Extravasation occurs via three sequential steps: (1) rolling, docking and adhesion via endothelial cell adhesion molecules (CAMs); (2) transmigration (extravasation) of leukocytes/CTCs; and (3) the subsequent extravasation of cells across vascular tight junctions^42^. A cancer cell moves along the endothelial lining by leading-edge protrusion and retraction of its tail. Selectins are known to mediate ‘rolling’ and tethering of leukocytes/tumor cells on the endothelium^12^. After ‘docking’, signaling is then driven by integrin-mediated interactions^12^. The resulting interactions between several selectins and integrin alpha/beta receptors on the surface of leukocytes/CTCs with the corresponding CAMs on the endothelial cells trigger a cascade of downstream signaling events in both cells^9^,^12^. In our combined pan-cancer datasets, we found upregulation of several integrins, including *ITGB1, ITGB2, ITGAL, ITGAM* and *ITGA4* and cell adhesion markers such as *ICAM3, PECAM1, JAM3* and *F11R*. The signal then travels via a chain of phosphorylation events that ultimately lead to phosphorylation and activation of the myosin light chain (MLC), and this causes retraction of the actin cytoskeleton in the tail-end of the migrating leukocyte or CTC^42^. We found several MLC molecules to be upregulated in our datasets (Fig. 3).

Cancer cells and leukocytes both use a group of adhesion receptors called galectins. Galectin-3 (*LGALS3*), known to be overexpressed in circulation of cancer patients, interacts with MUC1 resulting in exposure of cell surface adhesion molecule such as CD44 enhancing cancer cell adhesion^43^. Correspondingly, signaling events within the endothelial cell stimulate endothelial cell retraction followed by localized disassembly of the endothelial adherens junctions comprising cadherins and CAMs by the action of matrix metalloproteases (MMPs), actin remodeling and focal adhesion kinase (FAK) activation. This is consistent with the upregulation of several MMPs observed in our datasets and illustrated in Fig. 3. On the endothelial cell, *ICAM1* signals activate calcium flux and protein kinase C (PKC), which are required for leukocyte migration. Similarly, vascular cell adhesion molecule (*VCAM1*) ligation is required to open the “endothelial passage” by activating the production of reactive oxygen species (ROS) by NADPH oxidase. This in turn activates MMPs, which causes a loss of VE-cadherin-mediated adhesion and allows leukocytes or CTCs to extravasate^42^. Here, we find an upregulation of several components of this multi-subunit NADPH oxidase complex (Fig. 3).

Additionally, recent evidence suggests that platelets contribute to the survival and dissemination of cancer cells by interacting and aggregating around them, which allows cancer cells to evade immune assaults from natural killer cells^44^,^45^. Platelets release adenosine triphosphate (ATP), which in turn activates endogenous purinergic receptors (*P2Y2*) on cancer cells to mediate their transendothelial migration^46^. Tumor-secreted C-terminal fibrinogen-like domain of angiopoietin-like 4 (*ANGPTL4*) instigates vascular ‘leakiness’ and endothelial disruption by binding to integrins and TGFβ via the SMAD signaling pathway^47^,^48^. In our datasets, we found elevated expression of *ANGPTL2* and *ANGPTL4*. In sum, the enrichment observed in our analyses of the pan-cancer datasets will advance our current understanding of the biological mechanisms behind cancer cell dissemination and allow for a more targeted validation, with the hope to improve the management and prevention of metastatic progression.

Often pathways do not depict all the interconnections and cross-talk, hence in addition to pathways, we constructed a LEP-related network using available protein-protein interactions in STRING database (version 10.0; http://string-db.org/) to identify functional hubs with the most number of interactions [data not shown]. Some of the highly connected proteins included SRC, CDC42, PTK2, PXN, PIK3CA, MAPK14, VAV1, ACTB and ITGB2 among others. Earlier network studies on adhesome and EMT showed similar hubs of activity that can be used for targeted studies^49^,^50^. SRC, having the most connections in LEP network has been implicated in motility-regulating anoikis and microtentacle formation^51^,^52^, and disruption of SRC was found to suppress tumor cell extravasation^53^. These hubs may serve as prominent targets that can be investigated further in CTCs for therapeutic purposes.

While our observations center on gene expression changes in CTCs, we also found expression changes in several genes previously reported in endothelial cells (Fig. 3). We do realize that although care was taken in these studies to obtain a pure population of CTCs as mentioned previously, given the challenges involved in CTC isolation, some contamination with leukocytes and/or endothelial cells may occur, thus contributing to the observed changes in expression levels. However, other possibilities may exist wherein cancer cells can take on the functions of endothelial cells. Recently, a phenomenon called vascular mimicry (VM) has gained significance in tumor malignancies, including invasion and metastasis. VM in cancer cells leads to *de novo* vasculogenesis, wherein cancer cells express endothelial cell markers and an endothelial cell phenotype^54^,^55^, and permit the transfer of oxygen and nutrients^56^ in the tumor bed without coagulation events. Cancer cells endowed with VM harbor stem cell-like properties of chemoresistance and can develop into different cell types^57^,^58^. PIK2/Akt signaling pathway is activated in VM, which in turn activates MMPs. These proteases cleave Ln-5ϒ^2^ chains, and the resulting fragments create plasticity within the extracellular matrix (ECM)^59^. FAK, which we observed to be overexpressed in the pancreatic and melanoma CTC datasets, activates ERK1/2, which induces VM via MMPs^60^,^61^. We also observed overexpression of *HIF1A*—a master regulator of responses to hypoxia—in the prostate CTCs dataset. HIF1A activates VEGF expression, which in turn promotes VM by inducing EMT^62^,^63^. Further in-depth investigations and molecular characterizations of VM in tumor cells may reveal novel insights into the metastatic process.

### Unsupervised clustering of differentially expressed genes in CTCs

**Figure 4:**
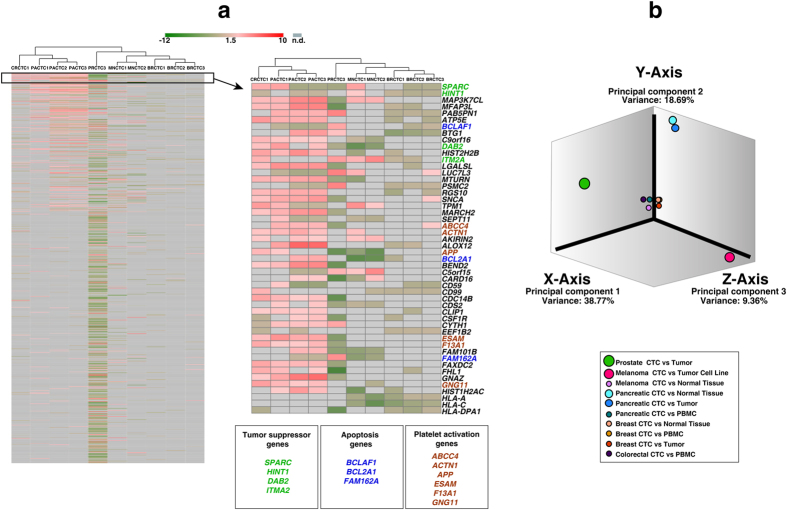
Unsupervised hierarchical clustering for differentially expressed genes in CTCs. (**a**) Heat map depicting hierarchical unsupervised clustering of gene expression profiles across the CTC subgroups from five cancers. Zoomed section of heat map shows the top 50 abundant genes across cancers; genes of interest are color-coded. Red in the heat map represents highly expressed genes, whereas green represents downregulated genes. (**b**) Principal component analysis showing the variance in gene expression datasets among CTC subgroups of five cancers.

To understand the gene expression profiles observed in CTCs among various cancers and identify signatures, we performed unsupervised clustering (using GENE-E version 3.0.206; http://www.broadinstitute.org/cancer/software/GENE-E/index.html) and principal component analysis (using GeneSpring GX software version 13.0) on differentially expressed gene datasets from the CTC_FC list (Fig. 4a,b, respectively). The top gene transcript, secreted protein acid and rich in cysteine (*SPARC*), codes for an important ECM protein reported to be differentially expressed and a modulator of several cellular processes required for metastasis initiation in the tumor microenvironment^64^,^65^. In colorectal, ovarian and prostate cancers, *SPARC* acts as a tumor suppressor by repressing angiogenesis by inhibiting vascular endothelial growth factor (VEGF); whereas, in pancreatic, breast, melanoma and glioblastoma cancers, its overexpression leads to the activation of MMPs, which in turn promote EMT^66^. *SPARC* has been shown to be necessary in pancreatic cancer metastasis^67^, and stromal *SPARC* overexpression is associated with poor survival^68^,^69^. It has therefore gained interest as a potential prognostic and therapeutic target in pancreatic cancer. We also observed downregulation of HIstidine triad NucleoTide-binding protein 1 (*HINT1*) in colorectal, pancreatic and breast CTCs (Fig. 4a). *HINT1* was recently considered a tumor suppressor gene^70^; although, its role in cancer metastasis is unclear^71^. We also found downregulation in genes involved in apoptosis in pancreatic (*BCLAF1, FAM162A*), breast (*BCLAF1*) and melanoma (*FAM162A*) CTCs.

Platelet activation and aggregation with CTCs not only protects cancer cells from natural killer cells and immune surveillance but can also prime them for metastasis^72^,^73^. Platelets play a major role in scaffolding the cancer cells and aid in their survival in circulation^74^. We found elevated expression of genes involved in platelet activation, such as *ABCC4, ACTN1, APP, ESAM, F13A1* and *GNG11*, in colorectal and pancreatic CTCs. Platelets secrete TGFβ1, which activates the TGFβ/Smad pathway in cancer cells and, at the same time, the platelet–cancer cell interaction activates the NF-kB pathway in cancer cells. These events trigger synergism between the pathways to promote metastasis^72^. They also assist in extravasation by releasing ATP molecules that activate the *P2Y2* receptors on cancer cells to permit transendothelial migration^46^.

Plastin 3 (*PLS3*) [data not shown in Fig. 4a but listed in Supplementary Table S1], a prominent CTC marker, was also differentially expressed in our screen. *PLS3* is strongly associated with prognosis of metastatic cancers; yet, others show that *PLS3* expression is unaffected during the EMT process^75^,^76^. Accordingly, we noted an overexpression of *PLS3* mRNA in breast and colorectal CTCs datasets but downregulation in prostate CTCs.

**Figure 5:**
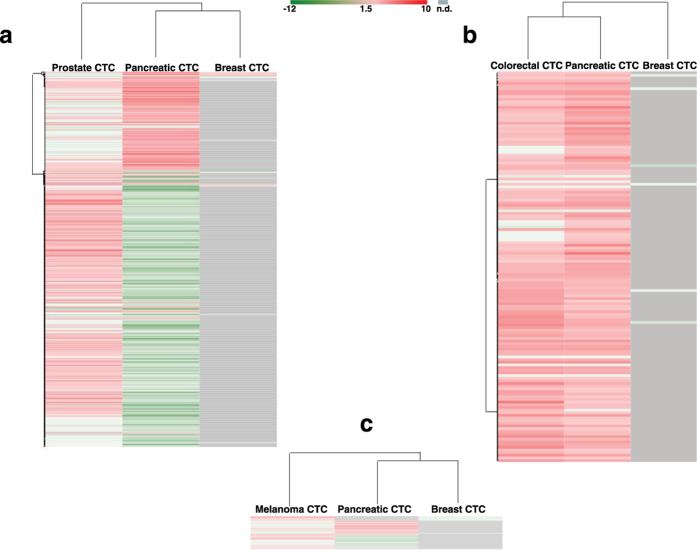
Unsupervised data-driven stratification of CTCs in relation to specific subgroups. (**a**) Heat map representing hierarchical unsupervised clustering of differentially expressed genes in CTCs when compared with primary tumors. (**b**) Unsupervised clustering of differentially expressed genes in CTCs when compared with peripheral blood mononuclear cells (PBMCs). (**c**) Unsupervised clustering of differentially expressed genes in CTCs when compared with normal tissue.

Patterns of differentially expressed genes in CTCs when compared to primary tumors or peripheral blood mononuclear cells (PBMCs) or normal tissue (Supplementary Fig. 2) were visualized by unsupervised clustering of DEGs for each sub-group (Fig. 5). We observed that colorectal CTCs and pancreatic CTCs showed similar gene expression profiles at the transcriptomic level and in their primary tumors. This could mean that CTC expression profiles could be used to reveal distinct expression patterns in some cancer types; understanding the diversities or similarities in CTC profiles across various cancer types may offer insights for the design of new therapeutic strategies^77^,^78^.

### Epithelial-Mesenchymal transition profile in CTCs

EMT is a key event involved in promoting the dissemination of carcinoma cells to distant organs^13^. We recently established a quantitative EMT scoring system across more than 15 cancers based on gene expression profiles^18^. This generic EMT signature for tumors and cell lines of different origins reflect not only the epithelial and mesenchymal states but also potential intermediate states that occur during the transition. Several studies have shown that CTCs exhibit a broad spectrum of EMT phenotypes, independent of carcinoma cell characteristics in primary tumors^17^,^79^. Epithelial markers (for example, EPCAM) are generally used to isolate CTCs from blood. However, CTCs exhibiting the full spectrum of EMT phenotypes will not be captured by this method, and CTCs thus isolated will not represent the entire population of CTCs in the blood.

In a benchmark study, circulating breast cancer cells were shown to express both epithelial and mesenchymal markers^17^. This observation was supported in a subsequent study by our group^80^, where we employed RNA-FISH to identify transcripts encoding various EMT markers (epithelial: E-cadherin, CK5, CK7, CK18, CK19, and *EPCAM*; and mesenchymal: *VIM* and *FSCN1*). We found heterogeneous expression of both epithelial and mesenchymal markers in the cultured CTCs. In concordance, others also suggest that CTCs from various cancer types may exhibit a dynamic EMT profile, and possibly showing an intermediate phenotype rather than a complete epithelial or mesenchymal state^6^.

**Figure 6:**
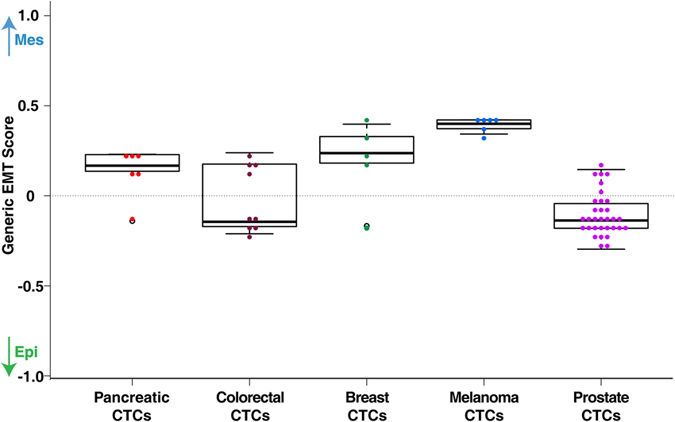
Epithelial-mesenchymal transition (EMT) scores across various cancers. Box plot-Dot plot gives the degree of EMT scores in a collection of CTCs from five cancers. EMT scores closer to +1.0 or −1.0 reflect more mesenchymal (Mes) or epithelial (Epi) states, respectively with potential intermediate states in between. Color code: red, pancreatic CTCs; maroon, colorectal CTCs; green, breast CTCs; blue, melanoma CTCs; purple, prostate CTCs.

The plasticity between epithelial and mesenchymal states in CTCs across cancers was assessed in two ways; 1) we employed the EMT scoring method^18^ [see Methods] to compute the generic EMT scores using transcriptome datasets from the five cancer studies (same studies used to generate CTC_FC dataset; Table 1). Figure 6 shows the distribution of the EMT scores in CTCs in each cancer type. Scores closer to +1.0 suggest a more mesenchymal-like (Mes) phenotype, and scores nearer to −1.0 reflect epithelial-like (Epi) phenotype. The EMT scores in our CTC datasets were distributed between −0.3 to +0.5, suggestive of intermediate phenotypes. The plot further shows a slightly higher expression of mesenchymal genes in melanoma and pancreatic cancer (Mean EMT scores - melanoma, +0.4; pancreatic, +0.2), which are suggestive of intermediate mesenchymal phenotypes and may reflect their ability to metastasize at an early stage^81^,^82^,^83^. In accordance with our previously reported intermediate EMT scores in a mixed population of breast cancer cell lines of Basal and Luminal phenotypes^18^, the breast CTCs in our dataset (subtyped as Basal and Luminal B^29^) also show intermediate mesenchymal scores; 2) we mapped the gene expression data from ‘CTC_FC’ dataset to the EMT signature genes (total = 418; epithelial = 228 and mesenchymal = 188) derived using gene expression profiles from tumor and cell lines from various cancers^18^,^84^,^85^. The distribution of expression values observed for the EMT signature genes in the CTC_FC dataset is shown in Supplementary Fig. 3. The swarm plot shows co-expression of epithelial and mesenchymal markers in CTCs derived from the five cancers. The observation from both analyses is in concordance with earlier studies based on experimental^86^ and mathematical models^87^, indicating an intermediate EMT phenotype in CTCs across cancers. The hybrid intermediate state may act as a crucial driver for tumor progression and metastatic colonization.

In addition, we computed the EMT scores for each cancer CTC dataset and compared them with their respective tumor and cancer cell lines EMT scores [see Methods]. The EMT score spectrum of CTCs exhibit similarities with our published^18^ cancer-specific EMT landscape of respective cell lines and tumors, albeit the average EMT score of CTCs is slightly higher in the case of colon, breast and pancreatic cancer datasets. As the EMT spectrum of CTCs resemble their tumor and cancer cell line counterparts more closely, this observation may add further evidence to substantiate that the gene expression data for the five cancer studies have been derived from a relatively pure population of CTC and not from cells of hematological origin. Future CTC expression studies with improved isolation techniques and comparison with expression profiles of different cell types will help to comprehensively characterize the molecular signatures of CTCs.

**Figure 7:**
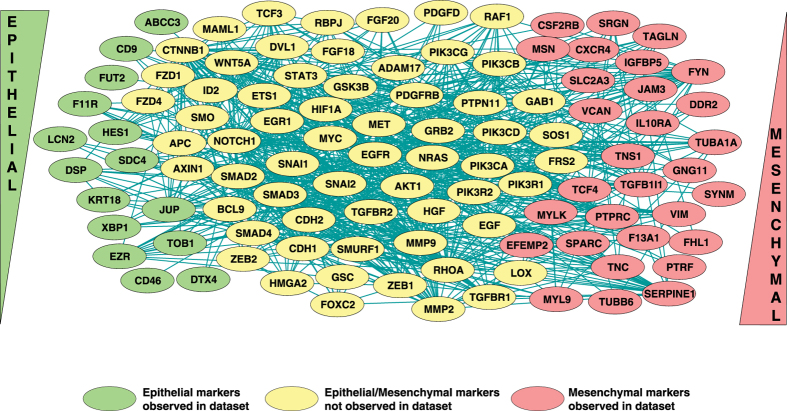
Integrated regulatory network for EMT in CTCs. Regulatory network depicting molecular interactions of EMT genes in CTCs across three cancers. The epithelial markers reported in our dataset are represented in green ovals; mesenchymal markers in red ovals. The yellow ovals represent other key EMT molecules reported in literature but not identified in our dataset.

Considering the disparate nature of the CTC_FC dataset, we built a gene interaction-based functional EMT network using a selected subset of gene expression data from colorectal, pancreatic and breast CTCs (DEGs when compared to PBMCs); this was done to acquire an accurate comparison of differential values and allow us to segregate high-confident EMT hub molecules from the network. The EMT network (Fig. 7) was built using Cytoscape^88^ (version 3.3.0; http://www.cytoscape.org/) by including key EMT regulators. The differential gene expression data from CTCs were then overlaid on this network. We noted an upregulation in *SPARC, MYLK, MYL6, GG11, F13A1* and *FHL1* (mesenchymal markers) and *CD9, LCN2*, and *ABCC3* (epithelial markers) in CTCs of at least two cancers. *SPARC* (also observed as top molecule in unsupervised clustering) is a well-known marker associated with invasion and an important mediator of bone metastasis^89^,^90^. Interestingly, *MYLK* and *CD9* have been implicated in lymph node metastasis in different cancers^91^,^92^, whereas *ABCC3* overexpression is reportedly involved in glycolysis, drug resistance and overall poor prognosis in bladder cancer [not in our dataset]^89^,^93^. However, some markers may play a tumor-specific role, as the loss of *FHL1* has been linked with increased metastasis in esophageal and gastric cancers^93^,^94^ [these cancers are not represented in our dataset]. Increased secretion of lipocalin-2 (*LCN2*), a tissue-specific factor not found in peripheral blood leukocytes, was reported in a CTC-derived cell line (BHGc7) from relapsed lung cancer patients^95^. Thus, CTC EMT phenotypes in different cancers may provide important, high-confident leads to monitor tumor-specific or CTC-specific events following therapeutic intervention.

## Concluding Remarks

This study has uncovered promising CTC-driven signaling nodes in the extravasation pathway and identified the key contributors that may determine favorable clinical outcomes. These CTC-associated signaling profiles will provide a valuable resource to identify downstream events crucial to the functioning of CTCs in the metastatic cascade. These findings bring us further toward understanding the mechanisms of various biological processing pathways within the circulating cell and pave the way for the identification of appropriate therapeutic targets against CTC, metastasis and cancer.

## Methods

We employed a multi-stage screening and filtering strategy to identify molecular signatures in CTCs. Briefly, we screened the literature for articles published on CTC expression. A list of all differentially expressed genes was prepared and used to identify significantly altered pathways and networks.

### Literature survey

PubMed was searched using keywords ‘circulating tumor cells signaling’ (November 9, 2015) which resulted in 1,073 publications. These publications were reviewed at the abstract level and 153 papers were narrowed down for further screening. Another PubMed search was carried out using keywords ‘circulating tumor cells gene expression’ (February 5, 2016), which resulted in 2,169 publications. Further review narrowed this down to 78 publications. Of the 231 (153 + 78) papers, 98 (42.4%) were thoroughly annotated for gene expression in CTCs [see Additional Supplementary Information and Supplementary Table S5]. Gene expression data were taken from semi-quantitative and qualitative experiments for gene annotation. This lenient but statistically significant (*p* < 0.05) gene list constituted genes reported to be overexpressed or downregulated in CTCs when compared to primary tumors, cancer cell lines, peripheral blood mononuclear cells (PBMCs), or normal tissue (each classified as CTC sub-group in the manuscript). Gene expression data were taken from studies that evaluated cancer patient CTC levels, cancer cells cultured *ex vivo* and spiked into the blood collected from healthy individuals, or CTCs isolated from blood of orthotopically grafted tumors in mice. From this extensive dataset compiled from 98 papers, data of differentially expressed genes from five large-scale transcriptomics studies across five different cancers was collated separately to generate a unique gene list with fold change values. Two of the five studies used RNA sequencing experiments and three used cDNA microarray to capture expression data. Details of the source for datasets are provided in Table 1.

### Transcriptomics dataset analysis

Transcriptome datasets were compiled from both high-throughput RNA-Seq experiments and microarray experiments (whichever available) related to CTCs. Supplementary results were compiled where available; otherwise data were processed to obtain the final set of genes. From the semi-quantitative experiments, sets of statistically significant, differentially expressed genes (DEG) with a log2 fold change cutoff of 1.5 and p-value < 0.05 were considered for downstream analysis. The colorectal and pancreatic microarray gene expression data (Gene Expression Omnibus repository; Accession Number: GSE31023 and GSE18670, respectively. https://www.ncbi.nlm.nih.gov/geo/query/acc.cgi?acc=GSE31023 and https://www.ncbi.nlm.nih.gov/geo/query/acc.cgi?acc=GSE18670) were reanalyzed using GEO2R (http://www.ncbi.nlm.nih.gov/geo/geo2r/) package to obtain the set of DEGs in CTCs. RNA-Seq data from the prostate cancer study were also reanalyzed to obtain genes that were differentially expressed with log2 fold change of 1.5 or higher using an in-house bioinformatics pipeline. Genes were filtered to include only the protein-coding genes for pathway analysis. Any pseudogenes, uncharacterized genes, non-coding RNA gene annotation were excluded. Entrez Gene IDs and gene biotype information were retrieved using the Ensemble Biomart (http://asia.ensembl.org/biomart/martview/) tool for human genome assembly; GRCh38p.5. Genes from various datasets were aggregated to form a master list of non-redundant DEGs.

For EMT scoring, transcriptome datasets of CTC from the five cancer studies (Table 1; Supplementary Table S6)—GSE18670, GSE31023, GSE38495, GSE45965, and GSE67980 were downloaded from GEO. The pre-processed data GSE45965 was also used. Dataset on Affymetrix platform (GSE18670) was RMA normalized using R version 3.3.1, Affy 1.50.0, whereas dataset on Agilent platform (GSE31023) was normalized using R limma 3.28.21. The RPM and RPKM normalized were extracted for RNA-Seq data GSE38495 and GSE67980. EMT score was computed using signatures and methods detailed in our previous study^18^.

Further, we computed the EMT scores for each of the five cancer CTC datasets using the gene expression data from genes overlapping across the datasets (n = 3167) and compared them to previously computed EMT scores for each tumor type and its cancer cell line collection^18^ (Supplementary Fig. 4)

### Pathway analysis

Two sets of data were used for pathway analysis: (1) the lenient gene list collated from 98 studies and (2) the differentially expressed genes list from the five high-throughput transcriptomics studies. (Denoted as CTC_ALL and CTC_FC (FC = fold-change), respectively for the purpose of differentiating between the datasets.)

The Ingenuity pathway analysis (IPA–Build: 377306 M; Version: 27216297; IPA, Qiagen, Redwood City, http://www.ingenuity.com/) tool was used for pathway analysis. Relationships of molecules differentially expressed in our dataset were inspected using the Core Analysis module in IPA Ingenuity Knowledge Base reference repository. Only the overexpressed genes from the CTC_ALL and CTC_FC datasets were uploaded into IPA, separately. Canonical pathway analyses identified the top canonical pathways that were significant in our datasets.

PathVisio (3.2.1; revision 4025; www.pathvisio.org), a free, open-source software for drawing and editing biological pathways, was used to manually depict the enrichment for Leukocyte Extravasation Signaling. Molecular interaction data were sourced from the KEGG pathway database (http://www.genome.jp/kegg/) and IPA pathways (IPA, Qiagen, Redwood City, http://www.ingenuity.com/). Literature mining was done for reported signaling patterns in CTCs, and only a handful of publications were available. Molecular interactions were annotated from these publications and appended onto the depicted pathway. An internal pathway review system was followed to carefully inspect for erroneous annotations. The pathway map was uploaded to GeneSpring software provided by Agilent Technologies (software version 13.0; http://www.genomics.agilent.com/en/Microarray-Data-Analysis-Software/GeneSpring-GX/?cid=AG-PT-130&tabId=AG-PR-1061) for enrichment. The gene list used for enrichment was identical to that used for IPA analysis (CTC_ALL, overexpressed genes n = 3,251).

STRING (version 10.0; http://string-db.org/) database was used for generating protein-protein interactions and Cytoscape (version 3.3.0; http://www.cytoscape.org/), an open-source platform, was used for visualizing the EMT biological pathways and molecular interaction network, and integrating the CTC gene expression data. In-house python scripts and the Adobe Illustrator (version CS5.1) graphics editor were used to draw or edit the figures.

## Additional Information

**How to cite this article:** Yadavalli, S. *et al*. Data-Driven Discovery of Extravasation Pathway in Circulating Tumor Cells. *Sci. Rep.* **7**, 43710; doi: 10.1038/srep43710 (2017).

**Publisher's note:** Springer Nature remains neutral with regard to jurisdictional claims in published maps and institutional affiliations.

## Supplementary Material

Supplementary Information

## Figures and Tables

**Table 1:** Details of CTC data from high-throughput transcriptomics studies of five cancers.

Cancer type	PubMed ID	Experiment type	Differential gene expression in CTCs compared to	Number of differentially expressed genes
Prostate	26383955	RNA sequencing	Primary tumors	4,203
Melanoma	22820318	RNA sequencing	Tumor cell lines	1,138
			Normal melanocytes	685
Breast	25432738	cDNA microarray	Normal epithelium	229
			PBMCs*	434
			Primary tumors	243
Colorectal	22811761	cDNA microarray	PBMCs*	736
Pancreatic	23157946	cDNA microarray	Normal tissue	1,571
			PBMCs*	666
			Primary tumors	1,490

^*^PBMCs – peripheral blood mononuclear cells.
