# Supplementary material for: Data-Driven Discovery of Extravasation Pathway in Circulating Tumor Cells
Source: Sci Rep. 2017 Mar 6;7:43710. doi: 10.1038/srep43710 (PMC5337960; doi:10.1038/srep43710)
Supplement: Supplementary Information [file srep43710-s1.pdf]

Data-Driven Discovery of Extravasation Pathway in Circulating Tumor Cells

Yadavalli S.[1], Jayaram S.[1,2], Manda SS.[1,3], Madugundu AK.[1,3], Nayakanti DS.[1], Tuan TZ.[6], Bhat R.[4], Rangarajan A.[4], Chatterjee A.[1,5], Gowda H.[1,5], Thiery JP.[6,7,8], Kumar P[1].

[1]Institute of Bioinformatics, International Technology Park, Whitefield, Bangalore, 560 066, India.
[2]Manipal University, Madhav Nagar, Manipal, 576104, India.
[3]Center for Bioinformatics, Pondicherry University, Puducherry 605 014, India.
[4]Department of Molecular Reproduction, Development and Genetics, Indian Institute of Science, Bangalore, 560012, India.
[5]YU-IOB Center for Systems Biology and Molecular Medicine, Yenepoya University, Mangalore, 575018, India.
[6]Cancer Science Institute of Singapore, National University of Singapore, Centre for Translational Medicine NUS Yong Loo Lin School of Medicine, Singapore 117599, Singapore.
[7]Comprehensive Cancer Center, Institut Gustave Roussy, 114 Rue Edouard Vaillant, 94805 Villejuif, France; CNRS UMR 7057, Matter and Complex Systems, Université Paris Diderot, 10 rue Alice Domon et Léonie Duquet 75013 Paris, France.
[8]Department of Biochemistry, National University of Singapore, Singapore 117597, Singapore.

Correspondence and requests for materials should be addressed to Kumar P. (email: prashant@ibioinformatics.org)

Supplementary Information

As shown in the supplementary figure 1a, the total number of papers that resulted from PubMed search with keyword 'circulating tumor cells signaling' is 1,073 (blue portion of the Venn diagram; 378+648+45+2 publications). This includes the papers that were common to results from keyword searches 'circulating tumor cells signaling and cancer' (648); 'circulating tumor cells signaling and epithelial mesenchymal transition' (2); and results common to all three keyword searches (45). The papers unique to the last two searches were excluded as they were not relevant. After abstract-level screening of these 1,073 papers, **153** were narrowed down for in-depth screening.

Similarly, as shown in the supplementary figure 1b, the total number of papers that resulted from PubMed search with keyword 'circulating tumor cells gene expression' is 2,169 (blue portion of the Venn diagram; 488+214+424+1,043 publications). This includes the papers that were common to results from keyword searches 'circulating tumor cells genes' (214); 'circulating tumor cells expression' (1,043); and results common to all three keyword searches (424). The papers unique to the last two searches were excluded due to non-relevance and large numbers. After abstract-level screening of these 2,169 papers, **78** were narrowed down for in-depth screening.

Out of these 231 papers (153+78), **98** papers were found to have gene expression data relevant to CTCs from which the gene list was compiled for our analysis. Of these, 93 papers were small-scale qualitative and semi-quantitative experiments that reported expression of a few genes in CTCs. The remaining five papers were based on large-scale transcriptomic experiments (RNA-Seq and Microarray) and reported differential expression of hundreds of genes in CTCs. The final gene list for our pathway analysis comprises mostly of genes from these five papers (7,209 out 7,572 total numbers of genes). These numbers are reported as CTC_ALL (7,572 genes from all 98 papers) out of which 7,209 (CTC_FC) were taken from the five large-scale transcriptomics experiment papers (Supplementary Table S1 and Supplementary Table S2, respectively). The reference information (PubMed IDs; PMIDs) for the 98 papers are listed in Supplementary Table S5.

Supplementary Figures

Supplementary Figure 1: Venn diagrams showing overlap between research articles when searched with keywords in Pubmed. (a) PubMed results with keywords containing ‘circulating tumor cells signaling’; (b) PubMed results with keywords containing ‘circulating tumor cells gene expression’; (c) Venn diagram showing number of differentially expressed genes in circulating tumor cells taken from semi-quantitative and qualitative experiments shown in CTC_ALL (n=7,572). Subset of differentially expressed genes from semi-quantitative experiments from five cancer studies is shown as CTC_FC (n=7,209).

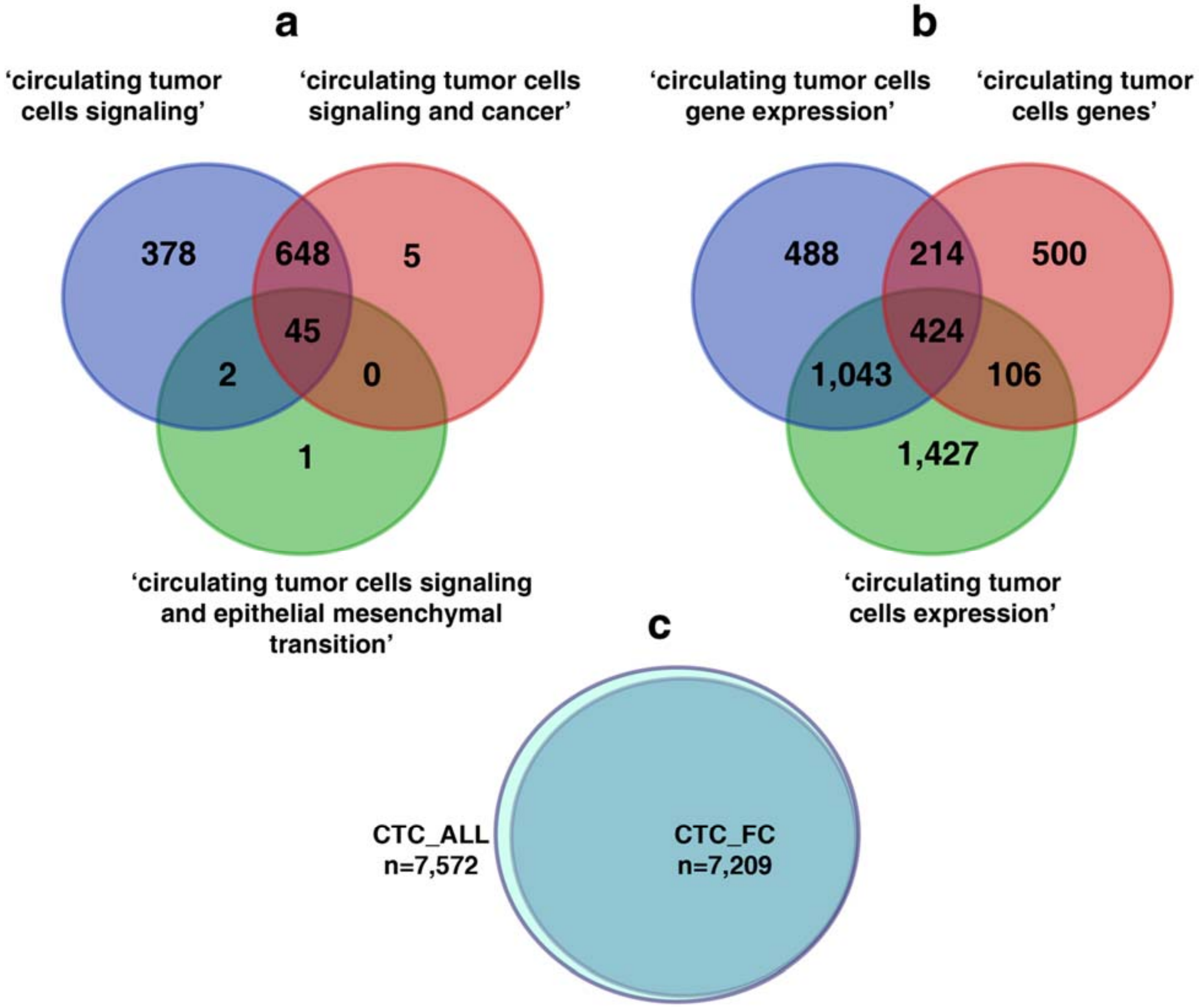

Supplementary Figure 2: Venn diagram showing the number of genes differentially expressed in CTCs in the five cancers. Number of genes overexpressed in CTCs when compared to primary tumors (a), peripheral blood mononuclear cells (PBMCs) (b), normal tissue (c). Number of genes downregulated in CTCs when compared to primary tumors (d), PBMCs (e), normal tissue (f).

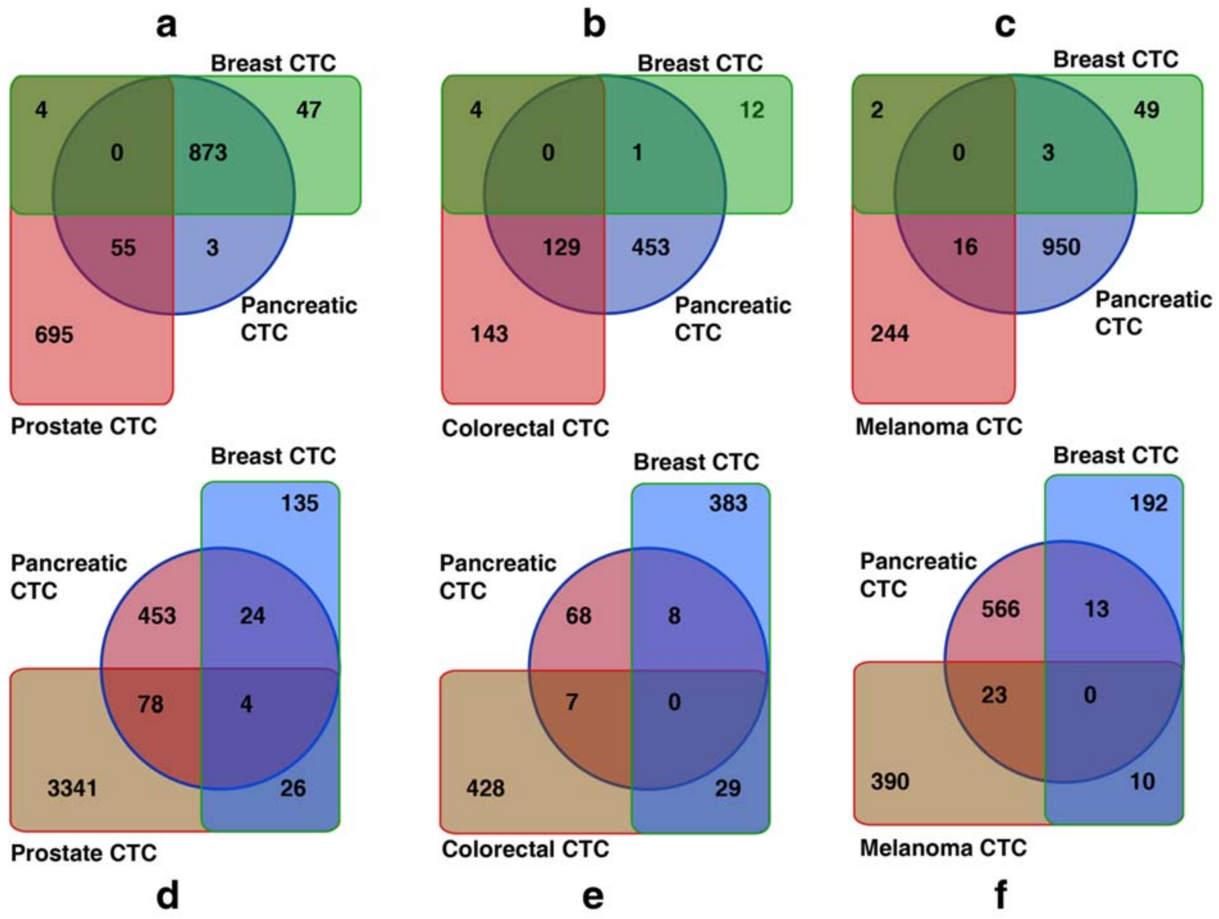

Supplementary Figure 3: Swarm plot shows the distribution of differentially expressed epithelial (green) and mesenchymal (blue) genes across the five cancer datasets. The genes are further segregated on their expression as epithelial or mesenchymal markers based on published literature. PRCTC1 denotes prostate CTCs gene expression when compared to primary tumors; CRCTC1– colorectal CTCs when compared to PBMCs; MNCTC1 – melanoma CTCs/melanoma cell lines; MNCTC2 – melanoma CTCs/primary melanocytes; PACTC1 – pancreatic CTCs/non-tumoral pancreatic tissue; PACTC2 – pancreatic CTCs/PBMCs; PACTC3 – pancreatic CTCs/primary tumors; BRCTC1 – breast CTCs/breast epithelium; BRCTC2 – breast CTCs/PBMCs; BRCTC3 – breast CTCs/primary tumors.

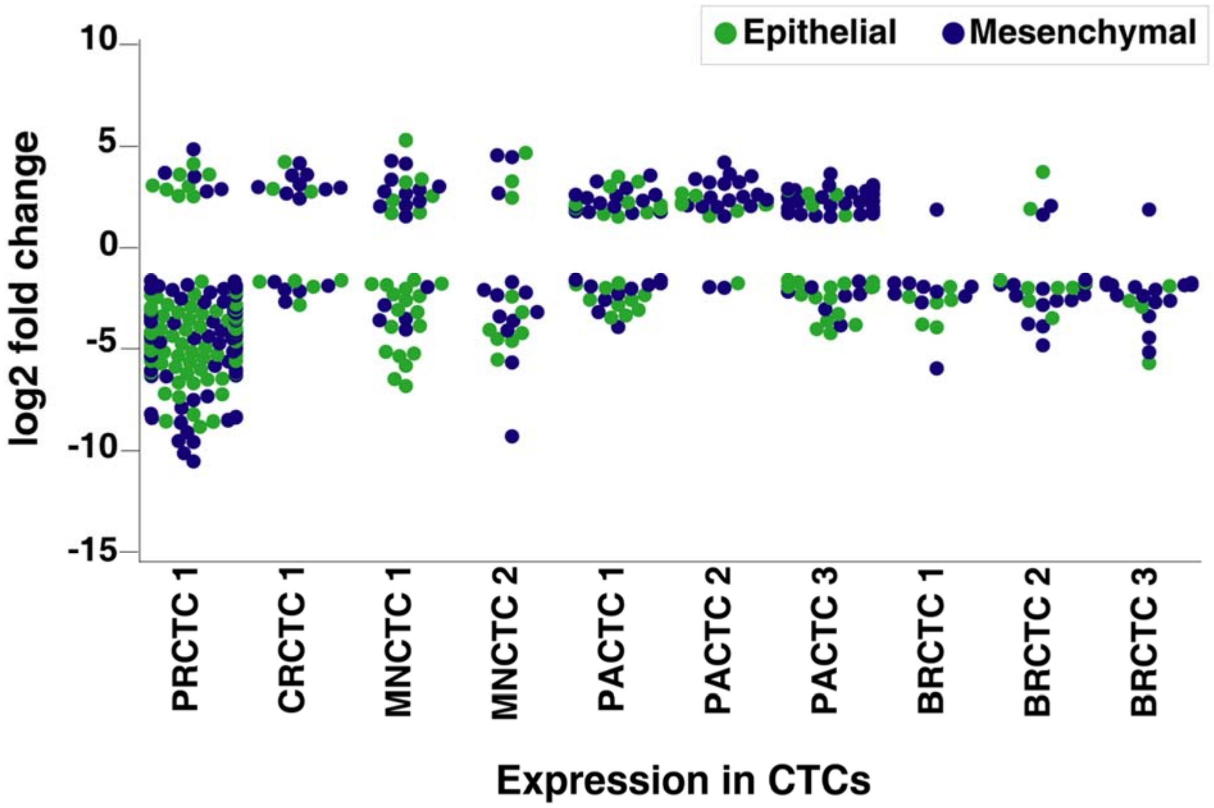

Supplementary Figure 4: Comparison of EMT scores of CTC gene expression data for the five cancers with a) EMT scores of tumors and b) EMT scores of cancer cell lines. The CTC dataset labels are highlighted in boxes.

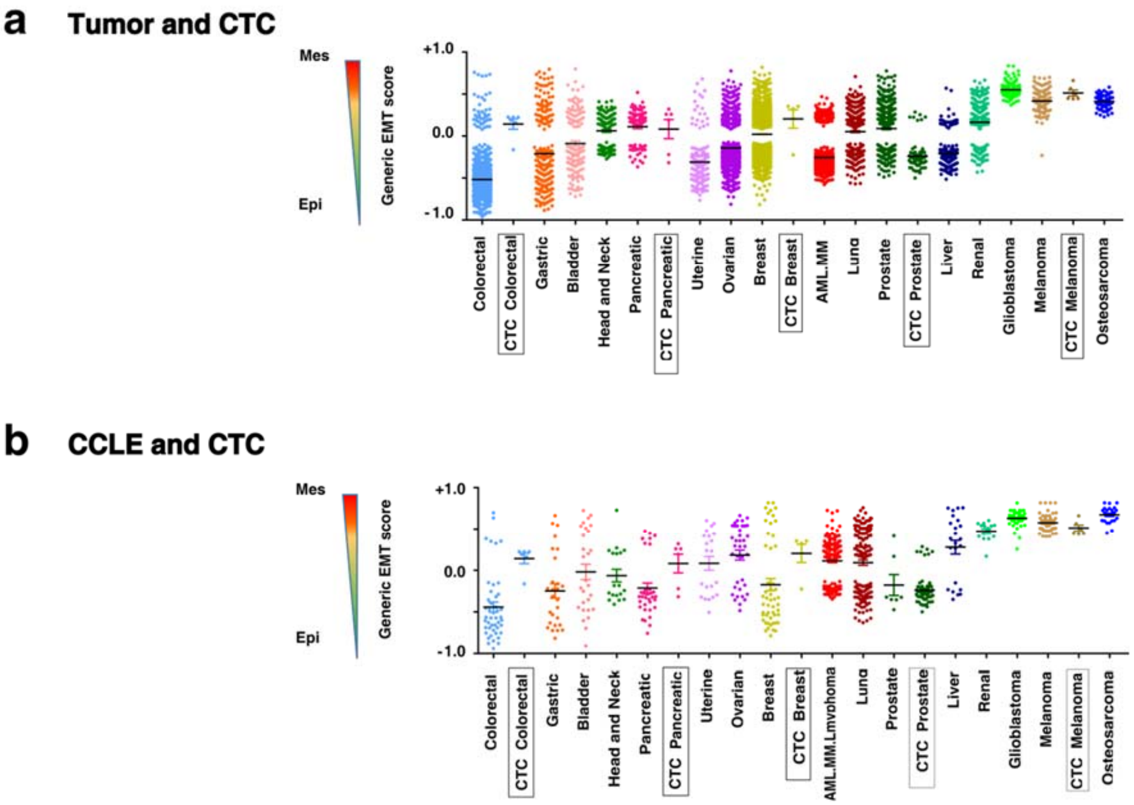

Supplementary Tables

Supplementary Table S1: CTC_ALL: Differentially expressed genes in circulating tumor cells when compared with primary tumors, PBMCs, normal tissue and tumor cell lines. Expression data for a total number of 7,572 genes were obtained from reported literature of semi-quantitative and qualitative experiments.

Supplementary Table S2: CTC_FC: Differentially expressed genes in circulating tumor cells when compared with primary tumors, PBMCs, normal tissue and tumor cell lines. Data for a total of 7,209 genes were obtained from reported literature of transcriptomics experiments from five different cancer studies of CTCs.

Supplementary Table S3: CTC isolation and purity confirmation techniques used by the five high-throughput experiments from which differentially expressed genes with fold change values (CTC_FC) was compiled for data analysis in our study.

Supplementary Table S4: List of molecular interactions reported in the literature (PubMed IDs included) and depicted in the leukocyte/CTC extravasation pathway.

Supplementary Table S5: PubMed IDs for 98 papers from which a gene list was compiled for molecular characterization of CTCs. This list includes the five large-scale transcriptomics experiment papers from which differentially expressed genes with fold change values were obtained (highlighted in yellow color).

Supplementary Table S6: EMT scores computed from transcriptome datasets of CTCs in five cancers—GSE18670, GSE31023, GSE38495, GSE45965, and GSE67980. The pre-processed data GSE45965 was also used.

Yadavalli et al., 2016. Data-Driven Discovery of Extravasation Pathway in Circulating Tumor Cells					
**Supplementary Table S1: CTC_ALL: Differentially expressed genes in circulating tumor cells when compared to primary tumors, PBMC, normal tissue and tumor cell lines. Expression data for a total number of 7,572 genes were obtained from reported literature of semi-quantitative and qualitative experiments.**					
	**Gene Symbol**	**Gene ID**	**Gene Description**	**Fold change (log2)**	**Arbitrary fold change *(for CTC_ALL analysis)***
1	*RPS4Y1*	6192	ribosomal protein S4, Y-linked 1 [Source:HGNC Symbol;Acc:HGNC:10425]	9.5	2
2	*COX7B2*	170712	cytochrome c oxidase subunit 7B2 [Source:HGNC Symbol;Acc:HGNC:24381]	9.3	2
3	*GTSF1*	121355	gametocyte specific factor 1 [Source:HGNC Symbol;Acc:HGNC:26565]	9.2	2
4	*SMUG1*	23583	single-strand-selective monofunctional uracil-DNA glycosylase 1 [Source:HGNC Symbol;Acc:HGNC:17148]	9.1	2
5	*PAGE5*	90737	PAGE family member 5 [Source:HGNC Symbol;Acc:HGNC:29992]	8.4	2
6	*TUBB1*	81027	tubulin beta 1 class VI [Source:HGNC Symbol;Acc:HGNC:16257]	8.3	2
7	*PPBP*	5473	pro-platelet basic protein [Source:HGNC Symbol;Acc:HGNC:9240]	8.3	2
8	*TMEFF2*	23671	transmembrane protein with EGF like and two follistatin like domains 2 [Source:HGNC Symbol;Acc:HGNC:11867]	7.7	2
9	*PF4*	5196	platelet factor 4 [Source:HGNC Symbol;Acc:HGNC:8861]	7.6	2
10	*ANKHD1*	54882	ankyrin repeat and KH domain containing 1 [Source:HGNC Symbol;Acc:HGNC:24714]	7.4	2
11	*ANKHD1-EIF4EBP3*	404734	ANKHD1-EIF4EBP3 readthrough [Source:HGNC Symbol;Acc:HGNC:33530]	7.4	2
12	*MLEC*	9761	malectin [Source:HGNC Symbol;Acc:HGNC:28973]	7.3	2
13	*S100A12*	6283	S100 calcium binding protein A12 [Source:HGNC Symbol;Acc:HGNC:10489]	7.0	2
14	*FCN1*	2219	ficolin 1 [Source:HGNC Symbol;Acc:HGNC:3623]	6.9	2
15	*ACRBP*	84519	acrosin binding protein [Source:HGNC Symbol;Acc:HGNC:17195]	6.9	2
16	*CCNB1*	891	cyclin B1 [Source:HGNC Symbol;Acc:HGNC:1579]	6.8	2
17	*NRGN*	4900	neurogranin [Source:HGNC Symbol;Acc:HGNC:8000]	6.8	2
18	*SREK1*	140890	splicing regulatory glutamic acid and lysine rich protein 1 [Source:HGNC Symbol;Acc:HGNC:17882]	6.7	2
19	*CENPM*	79019	centromere protein M [Source:HGNC Symbol;Acc:HGNC:18352]	6.6	2
20	*ANLN*	54443	anillin actin binding protein [Source:HGNC Symbol;Acc:HGNC:14082]	6.5	2
21	*KIAA0895*	23366	KIAA0895 [Source:HGNC Symbol;Acc:HGNC:22206]	6.5	2
22	*LYZ*	4069	lysozyme [Source:HGNC Symbol;Acc:HGNC:6740]	6.5	2
23	*CKAP4*	10970	cytoskeleton-associated protein 4 [Source:HGNC Symbol;Acc:HGNC:16991]	6.5	2
24	*ITGA2B*	3674	integrin subunit alpha 2b [Source:HGNC Symbol;Acc:HGNC:6138]	6.5	2
25	*TSG101*	7251	tumor susceptibility 101 [Source:HGNC Symbol;Acc:HGNC:15971]	6.4	2
26	*SRSF11*	9295	serine and arginine rich splicing factor 11 [Source:HGNC Symbol;Acc:HGNC:10782]	6.4	2
27	*SNX10*	29887	sorting nexin 10 [Source:HGNC Symbol;Acc:HGNC:14974]	6.4	2
28	*BMX*	660	BMX non-receptor tyrosine kinase [Source:HGNC Symbol;Acc:HGNC:1079]	6.3	2
29	*HIST1H3H*	8357	histone cluster 1, H3h [Source:HGNC Symbol;Acc:HGNC:4775]	6.3	2
30	*ECI2*	10455	enoyl-CoA delta isomerase 2 [Source:HGNC Symbol;Acc:HGNC:14601]	6.3	2
31	*RUVBL1*	8607	RuvB like AAA ATPase 1 [Source:HGNC Symbol;Acc:HGNC:10474]	6.3	2
32	*MRPL46*	26589	mitochondrial ribosomal protein L46 [Source:HGNC Symbol;Acc:HGNC:1192]	6.2	2
33	*MAF1*	84232	MAF1 homolog, negative regulator of RNA polymerase III [Source:HGNC Symbol;Acc:HGNC:24966]	6.0	2
34	*PF4V1*	5197	platelet factor 4 variant 1 [Source:HGNC Symbol;Acc:HGNC:8862]	6.0	2
35	*YY1AP1*	55249	YY1 associated protein 1 [Source:HGNC Symbol;Acc:HGNC:30935]	6.0	2
36	*TIMMDC1*	51300	translocase of inner mitochondrial membrane domain containing 1 [Source:HGNC Symbol;Acc:HGNC:1321]	5.9	2
37	*LYAR*	55646	Ly1 antibody reactive [Source:HGNC Symbol;Acc:HGNC:26021]	5.9	2
38	*TAL1*	6886	T-cell acute lymphocytic leukemia 1 [Source:HGNC Symbol;Acc:HGNC:11556]	5.9	2
39	*SF3B6*	51639	splicing factor 3b subunit 6 [Source:HGNC Symbol;Acc:HGNC:30096]	5.9	2
40	*BUD31*	8896	BUD31 homolog [Source:HGNC Symbol;Acc:HGNC:29629]	5.9	2
41	*RPL10L*	140801	ribosomal protein L10 like [Source:HGNC Symbol;Acc:HGNC:17976]	5.8	2
42	*MGAM*	8972	maltase-glucoamylase [Source:HGNC Symbol;Acc:HGNC:7043]	5.8	2
43	*ALOX12*	239	arachidonate 12-lipoxygenase, 12S type [Source:HGNC Symbol;Acc:HGNC:429]	5.8	2
44	*CCNB2*	9133	cyclin B2 [Source:HGNC Symbol;Acc:HGNC:1580]	5.8	2
45	*CYFIP2*	26999	cytoplasmic FMR1 interacting protein 2 [Source:HGNC Symbol;Acc:HGNC:13760]	5.8	2
46	*TREML1*	340205	triggering receptor expressed on myeloid cells like 1 [Source:HGNC Symbol;Acc:HGNC:20434]	5.8	2
47	*DNAJA4*	55466	DnaJ heat shock protein family (Hsp40) member A4 [Source:HGNC Symbol;Acc:HGNC:14885]	5.8	2
48	*HBG1*	3047	hemoglobin subunit gamma 1 [Source:HGNC Symbol;Acc:HGNC:4831]	5.8	2
49	*FUNDC2*	65991	FUN14 domain containing 2 [Source:HGNC Symbol;Acc:HGNC:24925]	5.7	2
50	*RGS18*	64407	regulator of G-protein signaling 18 [Source:HGNC Symbol;Acc:HGNC:14261]	5.7	2
51	*KRTAP19-1*	337882	keratin associated protein 19-1 [Source:HGNC Symbol;Acc:HGNC:18936]	5.7	2
52	*LPXN*	9404	leupaxin [Source:HGNC Symbol;Acc:HGNC:14061]	5.7	2
53	*SLC35B1*	10237	solute carrier family 35 member B1 [Source:HGNC Symbol;Acc:HGNC:20798]	5.7	2
54	*USP15*	9958	ubiquitin specific peptidase 15 [Source:HGNC Symbol;Acc:HGNC:12613]	5.7	2
55	*NAMPT*	10135	nicotinamide phosphoribosyltransferase [Source:HGNC Symbol;Acc:HGNC:30092]	5.7	2
56	*AHSP*	51327	alpha hemoglobin stabilizing protein [Source:HGNC Symbol;Acc:HGNC:18075]	5.7	2
57	*EWSR1*	2130	EWS RNA binding protein 1 [Source:HGNC Symbol;Acc:HGNC:3508]	5.7	2
58	*CELF1*	10658	CUGBP, Elav-like family member 1 [Source:HGNC Symbol;Acc:HGNC:2549]	5.7	2
59	*IFT57*	55081	intraflagellar transport 57 [Source:HGNC Symbol;Acc:HGNC:17367]	5.6	2
60	*SLA2*	84174	Src-like-adaptor 2 [Source:HGNC Symbol;Acc:HGNC:17329]	5.6	2
61	*SERPINA3*	12	serpin family A member 3 [Source:HGNC Symbol;Acc:HGNC:16]	5.6	2
62	*HMGN4*	10473	high mobility group nucleosomal binding domain 4 [Source:HGNC Symbol;Acc:HGNC:4989]	5.6	2
63	*TPP2*	7174	tripeptidyl peptidase 2 [Source:HGNC Symbol;Acc:HGNC:12016]	5.6	2
64	*NLRC5*	84166	NLR family CARD domain containing 5 [Source:HGNC Symbol;Acc:HGNC:29933]	5.6	2
65	*GNAZ*	2781	G protein subunit alpha z [Source:HGNC Symbol;Acc:HGNC:4395]	5.6	2
66	*SERPINB6*	5269	serpin family B member 6 [Source:HGNC Symbol;Acc:HGNC:8950]	5.6	2
67	*CDKN3*	1033	cyclin dependent kinase inhibitor 3 [Source:HGNC Symbol;Acc:HGNC:1791]	5.5	2
68	*PNN*	5411	pinin, desmosome associated protein [Source:HGNC Symbol;Acc:HGNC:9162]	5.5	2
69	*TSTD1*	100131187	thiosulfate sulfurtransferase like domain containing 1 [Source:HGNC Symbol;Acc:HGNC:35410]	5.5	2
70	*CST7*	8530	cystatin F [Source:HGNC Symbol;Acc:HGNC:2479]	5.4	2

71	*IGF2BP3*	10643	insulin like growth factor 2 mRNA binding protein 3 [Source:HGNC Symbol;Acc:HGNC:28868]	5.4	2
72	*CIAPIN1*	57019	cytokine induced apoptosis inhibitor 1 [Source:HGNC Symbol;Acc:HGNC:28050]	5.4	2
73	*RAB37*	326624	RAB37, member RAS oncogene family [Source:HGNC Symbol;Acc:HGNC:30268]	5.4	2
74	*SDPR*	8436	serum deprivation response [Source:HGNC Symbol;Acc:HGNC:10690]	5.4	2
75	*AP3D1*	8943	adaptor related protein complex 3 delta 1 subunit [Source:HGNC Symbol;Acc:HGNC:568]	5.4	2
76	*MSL1*	339287	male specific lethal 1 homolog [Source:HGNC Symbol;Acc:HGNC:27905]	5.4	2
77	*PSMB8*	5696	proteasome subunit beta 8 [Source:HGNC Symbol;Acc:HGNC:9545]	5.4	2
78	*HIST1H2AG*	8969	histone cluster 1, H2ag [Source:HGNC Symbol;Acc:HGNC:4737]	5.4	2
79	*HMMR*	3161	hyaluronan mediated motility receptor [Source:HGNC Symbol;Acc:HGNC:5012]	5.3	2
80	*IL2RB*	3560	interleukin 2 receptor subunit beta [Source:HGNC Symbol;Acc:HGNC:6009]	5.3	2
81	*RBM18*	92400	RNA binding motif protein 18 [Source:HGNC Symbol;Acc:HGNC:28413]	5.3	2
82	*BUB1*	699	BUB1 mitotic checkpoint serine/threonine kinase [Source:HGNC Symbol;Acc:HGNC:1148]	5.3	2
83	*MAGEA10*	4109	MAGE family member A10 [Source:HGNC Symbol;Acc:HGNC:6797]	5.3	2
84	*MAGEA10-MAGEA5*	100533997	MAGEA10-MAGEA5 Readthrough	5.3	2
85	*ANKRD17*	26057	ankyrin repeat domain 17 [Source:HGNC Symbol;Acc:HGNC:23575]	5.3	2
86	*TMEM40*	55287	transmembrane protein 40 [Source:HGNC Symbol;Acc:HGNC:25620]	5.3	2
87	*AFTPH*	54812	aftiphilin [Source:HGNC Symbol;Acc:HGNC:25951]	5.2	2
88	*ATP2B1*	490	ATPase plasma membrane Ca2+ transporting 1 [Source:HGNC Symbol;Acc:HGNC:814]	5.2	2
89	*DCAF13*	25879	DDB1 and CUL4 associated factor 13 [Source:HGNC Symbol;Acc:HGNC:24535]	5.2	2
90	*SLC25A32*	81034	solute carrier family 25 member 32 [Source:HGNC Symbol;Acc:HGNC:29683]	5.2	2
91	*GFI1B*	8328	growth factor independent 1B transcriptional repressor [Source:HGNC Symbol;Acc:HGNC:4238]	5.2	2
92	*S100A8*	6279	S100 calcium binding protein A8 [Source:HGNC Symbol;Acc:HGNC:10498]	5.2	2
93	*TMEM87A*	25963	transmembrane protein 87A [Source:HGNC Symbol;Acc:HGNC:24522]	5.2	2
94	*ANKRD10*	55608	ankyrin repeat domain 10 [Source:HGNC Symbol;Acc:HGNC:20265]	5.2	2
95	*PTN*	5764	pleiotrophin [Source:HGNC Symbol;Acc:HGNC:9630]	5.2	2
96	*TRIP12*	9320	thyroid hormone receptor interactor 12 [Source:HGNC Symbol;Acc:HGNC:12306]	5.1	2
97	*KRT15*	3866	keratin 15 [Source:HGNC Symbol;Acc:HGNC:6421]	5.1	2
98	*ITGB2*	3689	integrin subunit beta 2 [Source:HGNC Symbol;Acc:HGNC:6155]	5.1	2
99	*WDR45B*	56270	WD repeat domain 45B [Source:HGNC Symbol;Acc:HGNC:25072]	5.1	2
100	*IFT20*	90410	intraflagellar transport 20 [Source:HGNC Symbol;Acc:HGNC:30989]	5.1	2
101	*FGR*	2268	FGR proto-oncogene, Src family tyrosine kinase [Source:HGNC Symbol;Acc:HGNC:3697]	5.1	2
102	*NR2C2*	7182	nuclear receptor subfamily 2 group C member 2 [Source:HGNC Symbol;Acc:HGNC:7972]	5.1	2
103	*TRA2A*	29896	transformer 2 alpha homolog [Source:HGNC Symbol;Acc:HGNC:16645]	5.1	2
104	*MT-ND6*	4541	mitochondrially encoded NADH:ubiquinone oxidoreductase core subunit 6 [Source:HGNC Symbol;Acc:HGNC:7462]	5.1	2
105	*NUP93*	9688	nucleoporin 93 [Source:HGNC Symbol;Acc:HGNC:28958]	5.1	2
106	*MAGEB1*	4112	MAGE family member B1 [Source:HGNC Symbol;Acc:HGNC:6808]	5.1	2
107	*MAGEB4*	4115	MAGE family member B4 [Source:HGNC Symbol;Acc:HGNC:6811]	5.1	2
108	*ATP5S*	27109	ATP synthase, H+ transporting, mitochondrial Fo complex subunit s (factor B) [Source:HGNC Symbol;Acc:HGNC:18799]	5.1	2
109	*C2orf88*	84281	chromosome 2 open reading frame 88 [Source:HGNC Symbol;Acc:HGNC:28191]	5.1	2
110	*PAEP*	5047	progestagen associated endometrial protein [Source:HGNC Symbol;Acc:HGNC:8573]	5.1	2
111	*SAMD14*	201191	sterile alpha motif domain containing 14 [Source:HGNC Symbol;Acc:HGNC:27312]	5.1	2
112	*TMEM106C*	79022	transmembrane protein 106C [Source:HGNC Symbol;Acc:HGNC:28775]	5.1	2
113	*SPPL2B*	56928	signal peptide peptidase like 2B [Source:HGNC Symbol;Acc:HGNC:30627]	5.1	2
114	*CD247*	919	CD247 molecule [Source:HGNC Symbol;Acc:HGNC:1677]	5.1	2
115	*NUSAP1*	51203	nucleolar and spindle associated protein 1 [Source:HGNC Symbol;Acc:HGNC:18538]	5.1	2
116	*KIF5C*	3800	kinesin family member 5C [Source:HGNC Symbol;Acc:HGNC:6325]	5.1	2
117	*TRIM58*	25893	tripartite motif containing 58 [Source:HGNC Symbol;Acc:HGNC:24150]	5.1	2
118	*MKKS*	8195	McKusick-Kaufman syndrome [Source:HGNC Symbol;Acc:HGNC:7108]	5.1	2
119	*ERN2*	10595	endoplasmic reticulum to nucleus signaling 2 [Source:HGNC Symbol;Acc:HGNC:16942]	5.1	2
120	*PLK1*	5347	polo like kinase 1 [Source:HGNC Symbol;Acc:HGNC:9077]	5.1	2
121	*POM121*	9883	POM121 transmembrane nucleoporin [Source:HGNC Symbol;Acc:HGNC:19702]	5.0	2
122	*TMEM11*	8834	transmembrane protein 11 [Source:HGNC Symbol;Acc:HGNC:16823]	5.0	2
123	*SNRNP25*	79622	small nuclear ribonucleoprotein U11/U12 subunit 25 [Source:HGNC Symbol;Acc:HGNC:14161]	5.0	2
124	*PRAM1*	84106	PML-RARA regulated adaptor molecule 1 [Source:HGNC Symbol;Acc:HGNC:30091]	5.0	2
125	*GMPR2*	51292	guanosine monophosphate reductase 2 [Source:HGNC Symbol;Acc:HGNC:4377]	5.0	2
126	*ILF3*	3609	interleukin enhancer binding factor 3 [Source:HGNC Symbol;Acc:HGNC:6038]	5.0	2
127	*LUZP4*	51213	leucine zipper protein 4 [Source:HGNC Symbol;Acc:HGNC:24971]	5.0	2
128	*NUP35*	129401	nucleoporin 35 [Source:HGNC Symbol;Acc:HGNC:29797]	5.0	2
129	*SMPD1*	6609	sphingomyelin phosphodiesterase 1 [Source:HGNC Symbol;Acc:HGNC:11120]	5.0	2
130	*MBD6*	114785	methyl-CpG binding domain protein 6 [Source:HGNC Symbol;Acc:HGNC:20445]	5.0	2
131	*APOBEC3A*	200315	apolipoprotein B mRNA editing enzyme catalytic subunit 3A [Source:HGNC Symbol;Acc:HGNC:17343]	5.0	2
132	*UNC13B*	10497	unc-13 homolog B (C. elegans) [Source:HGNC Symbol;Acc:HGNC:12566]	5.0	2
133	*TIMM8B*	26521	translocase of inner mitochondrial membrane 8 homolog B (yeast) [Source:HGNC Symbol;Acc:HGNC:11818]	5.0	2
134	*C6orf62*	81688	chromosome 6 open reading frame 62 [Source:HGNC Symbol;Acc:HGNC:20998]	5.0	2
135	*YWHAG*	7532	tyrosine 3-monooxygenase/tryptophan 5-monooxygenase activation protein gamma [Source:HGNC Symbol;Acc:HGNC:12852]	5.0	2
136	*FAM133B*	257415	family with sequence similarity 133 member B [Source:HGNC Symbol;Acc:HGNC:28629]	5.0	2
137	*CKAP2*	26586	cytoskeleton associated protein 2 [Source:HGNC Symbol;Acc:HGNC:1990]	4.9	2
138	*MAGEC2*	51438	MAGE family member C2 [Source:HGNC Symbol;Acc:HGNC:13574]	4.9	2
139	*ARL6IP1*	23204	ADP ribosylation factor like GTPase 6 interacting protein 1 [Source:HGNC Symbol;Acc:HGNC:697]	4.9	2
140	*ALDH9A1*	223	aldehyde dehydrogenase 9 family member A1 [Source:HGNC Symbol;Acc:HGNC:412]	4.9	2
141	*UBE2V2*	7336	ubiquitin conjugating enzyme E2 V2 [Source:HGNC Symbol;Acc:HGNC:12495]	4.9	2
142	*LILRA2*	11027	leukocyte immunoglobulin like receptor A2 [Source:HGNC Symbol;Acc:HGNC:6603]	4.9	2
143	*GP9*	2815	glycoprotein IX platelet [Source:HGNC Symbol;Acc:HGNC:4444]	4.9	2
144	*PDZK1IP1*	10158	PDZK1 interacting protein 1 [Source:HGNC Symbol;Acc:HGNC:16887]	4.9	2

145	*MAGEB2*	4113	MAGE family member B2 [Source:HGNC Symbol;Acc:HGNC:6809]	4.9	2
146	*PPP2R2A*	5520	protein phosphatase 2 regulatory subunit Balpha [Source:HGNC Symbol;Acc:HGNC:9304]	4.9	2
147	*C12orf75*	387882	chromosome 12 open reading frame 75 [Source:HGNC Symbol;Acc:HGNC:35164]	4.9	2
148	*PRKAR2B*	5577	protein kinase cAMP-dependent type II regulatory subunit beta [Source:HGNC Symbol;Acc:HGNC:9392]	4.9	2
149	*CFP*	5199	complement factor properdin [Source:HGNC Symbol;Acc:HGNC:8864]	4.9	2
150	*DNAJB7*	150353	DnaJ heat shock protein family (Hsp40) member B7 [Source:HGNC Symbol;Acc:HGNC:24986]	4.9	2
151	*XPNPEP3*	63929	X-prolyl aminopeptidase 3, mitochondrial [Source:HGNC Symbol;Acc:HGNC:28052]	4.9	2
152	*SELENBP1*	8991	selenium binding protein 1 [Source:HGNC Symbol;Acc:HGNC:10719]	4.9	2
153	*SMU1*	55234	DNA replication regulator and spliceosomal factor [Source:HGNC Symbol;Acc:HGNC:18247]	4.9	2
154	*SUMO2*	6613	small ubiquitin-like modifier 2 [Source:HGNC Symbol;Acc:HGNC:11125]	4.9	2
155	*SEC61B*	10952	Sec61 translocon beta subunit [Source:HGNC Symbol;Acc:HGNC:16993]	4.9	2
156	*PEF1*	553115	penta-EF-hand domain containing 1 [Source:HGNC Symbol;Acc:HGNC:30009]	4.9	2
157	*PRKCB*	5579	protein kinase C beta [Source:HGNC Symbol;Acc:HGNC:9395]	4.9	2
158	*TOP2B*	7155	topoisomerase (DNA) II beta [Source:HGNC Symbol;Acc:HGNC:11990]	4.8	2
159	*HK3*	3101	hexokinase 3 [Source:HGNC Symbol;Acc:HGNC:4925]	4.8	2
160	*SFSWAP*	6433	splicing factor SWAP homolog [Source:HGNC Symbol;Acc:HGNC:10790]	4.8	2
161	*PRRC2C*	23215	proline rich coiled-coil 2C [Source:HGNC Symbol;Acc:HGNC:24903]	4.8	2
162	*COA1*	55744	cytochrome c oxidase assembly factor 1 homolog [Source:HGNC Symbol;Acc:HGNC:21868]	4.8	2
163	*LILRB2*	10288	leukocyte immunoglobulin like receptor B2 [Source:HGNC Symbol;Acc:HGNC:6606]	4.8	2
164	*CCT2*	10576	chaperonin containing TCP1 subunit 2 [Source:HGNC Symbol;Acc:HGNC:1615]	4.8	2
165	*MAGED2*	10916	MAGE family member D2 [Source:HGNC Symbol;Acc:HGNC:16353]	4.8	2
166	*CDC20*	991	cell division cycle 20 [Source:HGNC Symbol;Acc:HGNC:1723]	4.8	2
167	*SNRPA1*	6627	small nuclear ribonucleoprotein polypeptide A' [Source:HGNC Symbol;Acc:HGNC:11152]	4.8	2
168	*MAP3K7CL*	56911	MAP3K7 C-terminal like [Source:HGNC Symbol;Acc:HGNC:16457]	4.8	2
169	*PADI4*	23569	peptidyl arginine deiminase 4 [Source:HGNC Symbol;Acc:HGNC:18368]	4.8	2
170	*SPX*	80763	spexin hormone [Source:HGNC Symbol;Acc:HGNC:28139]	4.8	2
171	*GZMB*	3002	granzyme B [Source:HGNC Symbol;Acc:HGNC:4709]	4.8	2
172	*RASGRP2*	10235	RAS guanyl releasing protein 2 [Source:HGNC Symbol;Acc:HGNC:9879]	4.8	2
173	*FAM133A*	286499	family with sequence similarity 133 member A [Source:HGNC Symbol;Acc:HGNC:26748]	4.8	2
174	*MPL*	4352	MPL proto-oncogene, thrombopoietin receptor [Source:HGNC Symbol;Acc:HGNC:7217]	4.7	2
175	*APOBEC3B*	9582	apolipoprotein B mRNA editing enzyme catalytic subunit 3B [Source:HGNC Symbol;Acc:HGNC:17352]	4.7	2
176	*PTGS1*	5742	prostaglandin-endoperoxide synthase 1 [Source:HGNC Symbol;Acc:HGNC:9604]	4.7	2
177	*TIMM17A*	10440	translocase of inner mitochondrial membrane 17 homolog A (yeast) [Source:HGNC Symbol;Acc:HGNC:17315]	4.7	2
178	*NFE2*	4778	nuclear factor, erythroid 2 [Source:HGNC Symbol;Acc:HGNC:7780]	4.7	2
179	*MSN*	4478	moesin [Source:HGNC Symbol;Acc:HGNC:7373]	4.7	2
180	*CCNL1*	57018	cyclin L1 [Source:HGNC Symbol;Acc:HGNC:20569]	4.7	2
181	*NUP37*	79023	nucleoporin 37 [Source:HGNC Symbol;Acc:HGNC:29929]	4.7	2
182	*TMEM91*	641649	transmembrane protein 91 [Source:HGNC Symbol;Acc:HGNC:32393]	4.7	2
183	*ZNF185*	7739	zinc finger protein 185 (LIM domain) [Source:HGNC Symbol;Acc:HGNC:12976]	4.7	2
184	*PMM1*	5372	phosphomannomutase 1 [Source:HGNC Symbol;Acc:HGNC:9114]	4.7	2
185	*USP1*	7398	ubiquitin specific peptidase 1 [Source:HGNC Symbol;Acc:HGNC:12607]	4.7	2
186	*DLGAP5*	9787	DLG associated protein 5 [Source:HGNC Symbol;Acc:HGNC:16864]	4.7	2
187	*TROAP*	10024	trophinin associated protein [Source:HGNC Symbol;Acc:HGNC:12327]	4.7	2
188	*LUC7L3*	51747	LUC7 like 3 pre-mRNA splicing factor [Source:HGNC Symbol;Acc:HGNC:24309]	4.7	2
189	*SRPRB*	58477	SRP receptor beta subunit [Source:HGNC Symbol;Acc:HGNC:24085]	4.7	2
190	*NFIL3*	4783	nuclear factor, interleukin 3 regulated [Source:HGNC Symbol;Acc:HGNC:7787]	4.7	2
191	*ADD3*	120	adducin 3 [Source:HGNC Symbol;Acc:HGNC:245]	4.7	2
192	*TAP1*	6890	transporter 1, ATP-binding cassette, sub-family B (MDR/TAP) [Source:HGNC Symbol;Acc:HGNC:43]	4.7	2
193	*DLD*	1738	dihydrolipoamide dehydrogenase [Source:HGNC Symbol;Acc:HGNC:2898]	4.7	2
194	*SLC25A38*	54977	solute carrier family 25 member 38 [Source:HGNC Symbol;Acc:HGNC:26054]	4.7	2
195	*PLOD1*	5351	procollagen-lysine,2-oxoglutarate 5-dioxygenase 1 [Source:HGNC Symbol;Acc:HGNC:9081]	4.7	2
196	*PABPN1*	8106	poly(A) binding protein nuclear 1 [Source:HGNC Symbol;Acc:HGNC:8565]	4.6	2
197	*GAL3ST4*	79690	galactose-3-O-sulfotransferase 4 [Source:HGNC Symbol;Acc:HGNC:24145]	4.6	2
198	*DZIP3*	9666	DAZ interacting zinc finger protein 3 [Source:HGNC Symbol;Acc:HGNC:30938]	4.6	2
199	*KIAA1524*	57650	KIAA1524 [Source:HGNC Symbol;Acc:HGNC:29302]	4.6	2
200	*C10orf54*	64115	chromosome 10 open reading frame 54 [Source:HGNC Symbol;Acc:HGNC:30085]	4.6	2
201	*MFAP3L*	9848	microfibrillar associated protein 3 like [Source:HGNC Symbol;Acc:HGNC:29083]	4.6	2
202	*CTTN*	2017	cortactin [Source:HGNC Symbol;Acc:HGNC:3338]	4.6	2
203	*VPS28*	51160	VPS28, ESCRT-I subunit [Source:HGNC Symbol;Acc:HGNC:18178]	4.6	2
204	*DCTN3*	11258	dynactin subunit 3 [Source:HGNC Symbol;Acc:HGNC:2713]	4.6	2
205	*CCDC152*	100129792	coiled-coil domain containing 152 [Source:HGNC Symbol;Acc:HGNC:34438]	4.6	2
206	*SEPP1*	6414	selenoprotein P, plasma, 1 [Source:HGNC Symbol;Acc:HGNC:10751]	4.6	2
207	*PSMC2*	5701	proteasome 26S subunit, ATPase 2 [Source:HGNC Symbol;Acc:HGNC:9548]	4.6	2
208	*TMED2*	10959	transmembrane p24 trafficking protein 2 [Source:HGNC Symbol;Acc:HGNC:16996]	4.6	2
209	*EIF2S2*	8894	eukaryotic translation initiation factor 2 subunit beta [Source:HGNC Symbol;Acc:HGNC:3266]	4.6	2
210	*S100A9*	6280	S100 calcium binding protein A9 [Source:HGNC Symbol;Acc:HGNC:10499]	4.6	2
211	*SYTL5*	94122	synaptotagmin like 5 [Source:HGNC Symbol;Acc:HGNC:15589]	4.6	2
212	*RIN3*	79890	Ras and Rab interactor 3 [Source:HGNC Symbol;Acc:HGNC:18751]	4.6	2
213	*HNRNPH3*	3189	heterogeneous nuclear ribonucleoprotein H3 [Source:HGNC Symbol;Acc:HGNC:5043]	4.6	2
214	*CMTM5*	116173	CKLF like MARVEL transmembrane domain containing 5 [Source:HGNC Symbol;Acc:HGNC:19176]	4.6	2
215	*FAM136A*	84908	family with sequence similarity 136 member A [Source:HGNC Symbol;Acc:HGNC:25911]	4.6	2
216	*SYCP3*	50511	synaptonemal complex protein 3 [Source:HGNC Symbol;Acc:HGNC:18130]	4.6	2
217	*C11orf21*	29125	chromosome 11 open reading frame 21 [Source:HGNC Symbol;Acc:HGNC:13231]	4.6	2
218	*GNG10*	2790	G protein subunit gamma 10 [Source:HGNC Symbol;Acc:HGNC:4402]	4.6	2

219	*DNAJC25-GNG10*	552891	DNAJC25-GNG10 readthrough [Source:HGNC Symbol;Acc:HGNC:37501]	4.6	2
220	*DAPP1*	27071	dual adaptor of phosphotyrosine and 3-phosphoinositides 1 [Source:HGNC Symbol;Acc:HGNC:16500]	4.6	2
221	*MRPL22*	29093	mitochondrial ribosomal protein L22 [Source:HGNC Symbol;Acc:HGNC:14480]	4.6	2
222	*HJURP*	55355	Holliday junction recognition protein [Source:HGNC Symbol;Acc:HGNC:25444]	4.6	2
223	*ALDH3A2*	224	aldehyde dehydrogenase 3 family member A2 [Source:HGNC Symbol;Acc:HGNC:403]	4.6	2
224	*SPATA20*	64847	spermatogenesis associated 20 [Source:HGNC Symbol;Acc:HGNC:26125]	4.6	2
225	*C4orf19*	55286	chromosome 4 open reading frame 19 [Source:HGNC Symbol;Acc:HGNC:25618]	4.6	2
226	*RELL1*	768211	RELT like 1 [Source:HGNC Symbol;Acc:HGNC:27379]	4.6	2
227	*TSPAN33*	340348	tetraspanin 33 [Source:HGNC Symbol;Acc:HGNC:28743]	4.5	2
228	*CLEC12A*	160364	C-type lectin domain family 12 member A [Source:HGNC Symbol;Acc:HGNC:31713]	4.5	2
229	*C19orf33*	64073	chromosome 19 open reading frame 33 [Source:HGNC Symbol;Acc:HGNC:16668]	4.5	2
230	*UGP2*	7360	UDP-glucose pyrophosphorylase 2 [Source:HGNC Symbol;Acc:HGNC:12527]	4.5	2
231	*CCDC169*	728591	coiled-coil domain containing 169 [Source:HGNC Symbol;Acc:HGNC:34361]	4.5	2
232	*CCDC169-SOHLH2*	100526761	CCDC169-SOHLH2 readthrough [Source:HGNC Symbol;Acc:HGNC:38866]	4.5	2
233	*SOHLH2*	54937	spermatogenesis and oogenesis specific basic helix-loop-helix 2 [Source:HGNC Symbol;Acc:HGNC:26026]	4.5	2
234	*NOV*	4856	nephroblastoma overexpressed [Source:HGNC Symbol;Acc:HGNC:7885]	4.5	2
235	*ATF3*	467	activating transcription factor 3 [Source:HGNC Symbol;Acc:HGNC:785]	4.5	2
236	*PSMA5*	5686	proteasome subunit alpha 5 [Source:HGNC Symbol;Acc:HGNC:9534]	4.5	2
237	*AURKA*	6790	aurora kinase A [Source:HGNC Symbol;Acc:HGNC:11393]	4.5	2
238	*PRF1*	5551	perforin 1 [Source:HGNC Symbol;Acc:HGNC:9360]	4.5	2
239	*DNAJA2*	10294	DnaJ heat shock protein family (Hsp40) member A2 [Source:HGNC Symbol;Acc:HGNC:14884]	4.5	2
240	*CSF3R*	1441	colony stimulating factor 3 receptor [Source:HGNC Symbol;Acc:HGNC:2439]	4.5	2
241	*CHORDC1*	26973	cysteine and histidine rich domain containing 1 [Source:HGNC Symbol;Acc:HGNC:14525]	4.5	2
242	*NCF4*	4689	neutrophil cytosolic factor 4 [Source:HGNC Symbol;Acc:HGNC:7662]	4.5	2
243	*RACGAP1*	29127	Rac GTPase activating protein 1 [Source:HGNC Symbol;Acc:HGNC:9804]	4.5	2
244	*TCEAL8*	90843	transcription elongation factor A like 8 [Source:HGNC Symbol;Acc:HGNC:28683]	4.5	2
245	*S100A16*	140576	S100 calcium binding protein A16 [Source:HGNC Symbol;Acc:HGNC:20441]	4.5	2
246	*GFOD1*	54438	glucose-fructose oxidoreductase domain containing 1 [Source:HGNC Symbol;Acc:HGNC:21096]	4.5	2
247	*RUFY1*	80230	RUN and FYVE domain containing 1 [Source:HGNC Symbol;Acc:HGNC:19760]	4.5	2
248	*EIF5*	1983	eukaryotic translation initiation factor 5 [Source:HGNC Symbol;Acc:HGNC:3299]	4.5	2
249	*PGM2*	55276	phosphoglucomutase 2 [Source:HGNC Symbol;Acc:HGNC:8906]	4.5	2
250	*ASNS*	440	asparagine synthetase (glutamine-hydrolyzing) [Source:HGNC Symbol;Acc:HGNC:753]	4.5	2
251	*LILRA3*	11026	leukocyte immunoglobulin like receptor A3 [Source:HGNC Symbol;Acc:HGNC:6604]	4.5	2
252	*MRRF*	92399	mitochondrial ribosome recycling factor [Source:HGNC Symbol;Acc:HGNC:7234]	4.5	2
253	*YY1*	7528	YY1 transcription factor [Source:HGNC Symbol;Acc:HGNC:12856]	4.5	2
254	*TOP1*	7150	topoisomerase (DNA) I [Source:HGNC Symbol;Acc:HGNC:11986]	4.5	2
255	*FAM192A*	80011	family with sequence similarity 192 member A [Source:HGNC Symbol;Acc:HGNC:29856]	4.5	2
256	*DRAP1*	10589	DR1 associated protein 1 [Source:HGNC Symbol;Acc:HGNC:3019]	4.4	2
257	*OGT*	8473	O-linked N-acetylglucosamine (GlcNAc) transferase [Source:HGNC Symbol;Acc:HGNC:8127]	4.4	2
258	*TCAF1*	9747	TRPM8 channel associated factor 1 [Source:HGNC Symbol;Acc:HGNC:22201]	4.4	2
259	*ITM2A*	9452	integral membrane protein 2A [Source:HGNC Symbol;Acc:HGNC:6173]	4.4	2
260	*SLC25A36*	55186	solute carrier family 25 member 36 [Source:HGNC Symbol;Acc:HGNC:25554]	4.4	2
261	*RAP1GDS1*	5910	Rap1 GTPase-GDP dissociation stimulator 1 [Source:HGNC Symbol;Acc:HGNC:9859]	4.4	2
262	*PPM1A*	5494	protein phosphatase, Mg2+/Mn2+ dependent 1A [Source:HGNC Symbol;Acc:HGNC:9275]	4.4	2
263	*DPEP2*	64174	dipeptidase 2 [Source:HGNC Symbol;Acc:HGNC:23028]	4.4	2
264	*HBA2*	3040	hemoglobin subunit alpha 2 [Source:HGNC Symbol;Acc:HGNC:4824]	4.4	2
265	*UBE2E3*	10477	ubiquitin conjugating enzyme E2 E3 [Source:HGNC Symbol;Acc:HGNC:12479]	4.4	2
266	*WIPI1*	55062	WD repeat domain, phosphoinositide interacting 1 [Source:HGNC Symbol;Acc:HGNC:25471]	4.4	2
267	*CHD4*	1108	chromodomain helicase DNA binding protein 4 [Source:HGNC Symbol;Acc:HGNC:1919]	4.4	2
268	*GP1BA*	2811	glycoprotein Ib platelet alpha subunit [Source:HGNC Symbol;Acc:HGNC:4439]	4.4	2
269	*BEND2*	139105	BEN domain containing 2 [Source:HGNC Symbol;Acc:HGNC:28509]	4.4	2
270	*COMMD7*	149951	COMM domain containing 7 [Source:HGNC Symbol;Acc:HGNC:16223]	4.4	2
271	*FLII*	2314	FLII, actin remodeling protein [Source:HGNC Symbol;Acc:HGNC:3750]	4.4	2
272	*CHMP5*	51510	charged multivesicular body protein 5 [Source:HGNC Symbol;Acc:HGNC:26942]	4.4	2
273	*ADRM1*	11047	adhesion regulating molecule 1 [Source:HGNC Symbol;Acc:HGNC:15759]	4.4	2
274	*GZMH*	2999	granzyme H [Source:HGNC Symbol;Acc:HGNC:4710]	4.4	2
275	*RABIF*	5877	RAB interacting factor [Source:HGNC Symbol;Acc:HGNC:9797]	4.4	2
276	*PBK*	55872	PDZ binding kinase [Source:HGNC Symbol;Acc:HGNC:18282]	4.4	2
277	*PROK2*	60675	prokineticin 2 [Source:HGNC Symbol;Acc:HGNC:18455]	4.4	2
278	*ANKRD55*	79722	ankyrin repeat domain 55 [Source:HGNC Symbol;Acc:HGNC:25681]	4.4	2
279	*PAGE2B*	389860	PAGE family member 2B [Source:HGNC Symbol;Acc:HGNC:31805]	4.4	2
280	*GNLY*	10578	granulysin [Source:HGNC Symbol;Acc:HGNC:4414]	4.4	2
281	*SSB*	6741	Sjogren syndrome antigen B [Source:HGNC Symbol;Acc:HGNC:11316]	4.4	2
282	*RBM22*	55696	RNA binding motif protein 22 [Source:HGNC Symbol;Acc:HGNC:25503]	4.4	2
283	*MCL1*	4170	myeloid cell leukemia 1 [Source:HGNC Symbol;Acc:HGNC:6943]	4.4	2
284	*OSTC*	58505	oligosaccharyltransferase complex subunit (non-catalytic) [Source:HGNC Symbol;Acc:HGNC:24448]	4.4	2
285	*PILRB*	29990	paired immunoglobin-like type 2 receptor beta [Source:HGNC Symbol;Acc:HGNC:18297]	4.4	2
286	*SPDYE3*	441272	speedy/RINGO cell cycle regulator family member E3 [Source:HGNC Symbol;Acc:HGNC:35462]	4.4	2
287	*ZNF467*	168544	zinc finger protein 467 [Source:HGNC Symbol;Acc:HGNC:23154]	4.4	2
288	*SRPX*	8406	sushi repeat containing protein, X-linked [Source:HGNC Symbol;Acc:HGNC:11309]	4.4	2
289	*CCDC73*	493860	coiled-coil domain containing 73 [Source:HGNC Symbol;Acc:HGNC:23261]	4.3	2
290	*TTK*	7272	TTK protein kinase [Source:HGNC Symbol;Acc:HGNC:12401]	4.3	2
291	*AKIRIN1*	79647	akirin 1 [Source:HGNC Symbol;Acc:HGNC:25744]	4.3	2
292	*IST1*	9798	IST1, ESCRT-III associated factor [Source:HGNC Symbol;Acc:HGNC:28977]	4.3	2
293	*SRP72*	6731	signal recognition particle 72kDa [Source:HGNC Symbol;Acc:HGNC:11303]	4.3	2
294	*CLUL1*	27098	clusterin like 1 [Source:HGNC Symbol;Acc:HGNC:2096]	4.3	2

295	*ENOSF1*	55556	enolase superfamily member 1 [Source:HGNC Symbol;Acc:HGNC:30365]	4.3	2
296	*TYMS*	7298	thymidylate synthetase [Source:HGNC Symbol;Acc:HGNC:12441]	4.3	2
297	*TYMSOS*	494514	TYMS opposite strand [Source:HGNC Symbol;Acc:HGNC:29553]	4.3	2
298	*APOD*	347	apolipoprotein D [Source:HGNC Symbol;Acc:HGNC:612]	4.3	2
299	*CENPK*	64105	centromere protein K [Source:HGNC Symbol;Acc:HGNC:29479]	4.3	2
300	*SURF2*	6835	surfeit 2 [Source:HGNC Symbol;Acc:HGNC:11475]	4.3	2
301	*C5orf15*	56951	chromosome 5 open reading frame 15 [Source:HGNC Symbol;Acc:HGNC:20656]	4.3	2
302	*LARP4*	113251	La ribonucleoprotein domain family member 4 [Source:HGNC Symbol;Acc:HGNC:24320]	4.3	2
303	*FRMD3*	257019	FERM domain containing 3 [Source:HGNC Symbol;Acc:HGNC:24125]	4.3	2
304	*CTNNAL1*	8727	catenin alpha like 1 [Source:HGNC Symbol;Acc:HGNC:2512]	4.3	2
305	*LGMN*	5641	legumain [Source:HGNC Symbol;Acc:HGNC:9472]	4.3	2
306	*MMP9*	4318	matrix metallopeptidase 9 [Source:HGNC Symbol;Acc:HGNC:7176]	4.3	2
307	*C8orf76*	84933	chromosome 8 open reading frame 76 [Source:HGNC Symbol;Acc:HGNC:25924]	4.3	2
308	*ZHX1*	11244	zinc fingers and homeoboxes 1 [Source:HGNC Symbol;Acc:HGNC:12871]	4.3	2
309	*ZHX1-C8orf76*	100533106	ZHX1-C8orf76 readthrough [Source:HGNC Symbol;Acc:HGNC:42975]	4.3	2
310	*HN1*	51155	hematological and neurological expressed 1 [Source:HGNC Symbol;Acc:HGNC:14569]	4.3	2
311	*TNFRSF1B*	7133	tumor necrosis factor receptor superfamily member 1B [Source:HGNC Symbol;Acc:HGNC:11917]	4.3	2
312	*MSMO1*	6307	methylsterol monooxygenase 1 [Source:HGNC Symbol;Acc:HGNC:10545]	4.3	2
313	*ARL4C*	10123	ADP ribosylation factor like GTPase 4C [Source:HGNC Symbol;Acc:HGNC:698]	4.3	2
314	*LTB4R*	1241	leukotriene B4 receptor [Source:HGNC Symbol;Acc:HGNC:6713]	4.3	2
315	*TTC1*	7265	tetratricopeptide repeat domain 1 [Source:HGNC Symbol;Acc:HGNC:12391]	4.3	2
316	*HMGB2*	3148	high mobility group box 2 [Source:HGNC Symbol;Acc:HGNC:5000]	4.3	2
317	*TMEM170B*	100113407	transmembrane protein 170B [Source:HGNC Symbol;Acc:HGNC:34244]	4.3	2
318	*PUM3*	9933	pumilio RNA binding family member 3 [Source:HGNC Symbol;Acc:HGNC:29676]	4.3	2
319	*HOMER2*	9455	homer scaffolding protein 2 [Source:HGNC Symbol;Acc:HGNC:17513]	4.3	2
320	*XRRA1*	143570	X-ray radiation resistance associated 1 [Source:HGNC Symbol;Acc:HGNC:18868]	4.3	2
321	*SNN*	8303	stannin [Source:HGNC Symbol;Acc:HGNC:11149]	4.3	2
322	*MTERF2*	80298	mitochondrial transcription termination factor 2 [Source:HGNC Symbol;Acc:HGNC:30779]	4.3	2
323	*FGD3*	89846	FYVE, RhoGEF and PH domain containing 3 [Source:HGNC Symbol;Acc:HGNC:16027]	4.3	2
324	*CCDC174*	51244	coiled-coil domain containing 174 [Source:HGNC Symbol;Acc:HGNC:28033]	4.3	2
325	*GLT1D1*	144423	glycosyltransferase 1 domain containing 1 [Source:HGNC Symbol;Acc:HGNC:26483]	4.3	2
326	*NKIRAS1*	28512	NFKB inhibitor interacting Ras like 1 [Source:HGNC Symbol;Acc:HGNC:17899]	4.3	2
327	*FERMT3*	83706	fermitin family member 3 [Source:HGNC Symbol;Acc:HGNC:23151]	4.3	2
328	*EGLN2*	112398	egl-9 family hypoxia inducible factor 2 [Source:HGNC Symbol;Acc:HGNC:14660]	4.3	2
329	*USP48*	84196	ubiquitin specific peptidase 48 [Source:HGNC Symbol;Acc:HGNC:18533]	4.2	2
330	*CNDP1*	84735	carnosine dipeptidase 1 [Source:HGNC Symbol;Acc:HGNC:20675]	4.2	2
331	*FAM65B*	9750	family with sequence similarity 65 member B [Source:HGNC Symbol;Acc:HGNC:13872]	4.2	2
332	*LARP1*	23367	La ribonucleoprotein domain family member 1 [Source:HGNC Symbol;Acc:HGNC:29531]	4.2	2
333	*AQP10*	89872	aquaporin 10 [Source:HGNC Symbol;Acc:HGNC:16029]	4.2	2
334	*FAM168B*	130074	family with sequence similarity 168 member B [Source:HGNC Symbol;Acc:HGNC:27016]	4.2	2
335	*TMEM200B*	399474	transmembrane protein 200B [Source:HGNC Symbol;Acc:HGNC:33785]	4.2	2
336	*P2RY12*	64805	purinergic receptor P2Y12 [Source:HGNC Symbol;Acc:HGNC:18124]	4.2	2
337	*CLU*	1191	clusterin [Source:HGNC Symbol;Acc:HGNC:2095]	4.2	2
338	*DDX3Y*	8653	DEAD-box helicase 3, Y-linked [Source:HGNC Symbol;Acc:HGNC:2699]	4.2	2
339	*STXBP2*	6813	syntaxin binding protein 2 [Source:HGNC Symbol;Acc:HGNC:11445]	4.2	2
340	*AK4*	205	adenylate kinase 4 [Source:HGNC Symbol;Acc:HGNC:363]	4.2	2
341	*BST2*	684	bone marrow stromal cell antigen 2 [Source:HGNC Symbol;Acc:HGNC:1119]	4.2	2
342	*SRRM1*	10250	serine and arginine repetitive matrix 1 [Source:HGNC Symbol;Acc:HGNC:16638]	4.2	2
343	*KXD1*	79036	KxDL motif containing 1 [Source:HGNC Symbol;Acc:HGNC:28420]	4.2	2
344	*PABPC3*	5042	poly(A) binding protein cytoplasmic 3 [Source:HGNC Symbol;Acc:HGNC:8556]	4.2	2
345	*PSMD4*	5710	proteasome 26S subunit, non-ATPase 4 [Source:HGNC Symbol;Acc:HGNC:9561]	4.2	2
346	*TBXA2R*	6915	thromboxane A2 receptor [Source:HGNC Symbol;Acc:HGNC:11608]	4.2	2
347	*ZWINT*	11130	ZW10 interacting kinetochore protein [Source:HGNC Symbol;Acc:HGNC:13195]	4.2	2
348	*VAMP1*	6843	vesicle associated membrane protein 1 [Source:HGNC Symbol;Acc:HGNC:12642]	4.2	2
349	*HMHA1*	23526	Mitochondrial Calcium Uniporter	4.2	2
350	*RAD23A*	5886	RAD23 homolog A, nucleotide excision repair protein [Source:HGNC Symbol;Acc:HGNC:9812]	4.2	2
351	*PIN4*	5303	peptidylprolyl cis/trans isomerase, NIMA-interacting 4 [Source:HGNC Symbol;Acc:HGNC:8992]	4.2	2
352	*DGAT1*	8694	diacylglycerol O-acyltransferase 1 [Source:HGNC Symbol;Acc:HGNC:2843]	4.2	2
353	*OVOS2*	144203	Ovostatin 2	4.2	2
354	*IQCJ*	654502	IQ motif containing J [Source:HGNC Symbol;Acc:HGNC:32406]	4.2	2
355	*IQCJ-SCHIP1*	100505385	IQCJ-SCHIP1 readthrough [Source:HGNC Symbol;Acc:HGNC:38842]	4.2	2
356	*SCHIP1*	29970	schwannomin interacting protein 1 [Source:HGNC Symbol;Acc:HGNC:15678]	4.2	2
357	*PTPRJ*	5795	protein tyrosine phosphatase, receptor type J [Source:HGNC Symbol;Acc:HGNC:9673]	4.2	2
358	*DGAT2*	84649	diacylglycerol O-acyltransferase 2 [Source:HGNC Symbol;Acc:HGNC:16940]	4.2	2
359	*KCNN2*	3781	potassium calcium-activated channel subfamily N member 2 [Source:HGNC Symbol;Acc:HGNC:6291]	4.2	2
360	*CORO1A*	11151	coronin 1A [Source:HGNC Symbol;Acc:HGNC:2252]	4.2	2
361	*UBE2N*	7334	ubiquitin conjugating enzyme E2 N [Source:HGNC Symbol;Acc:HGNC:12492]	4.2	2
362	*TMEM97*	27346	transmembrane protein 97 [Source:HGNC Symbol;Acc:HGNC:28106]	4.2	2
363	*GAS2L1*	10634	growth arrest specific 2 like 1 [Source:HGNC Symbol;Acc:HGNC:16955]	4.2	2
364	*TNC*	3371	tenascin C [Source:HGNC Symbol;Acc:HGNC:5318]	4.2	2
365	*SLC25A5*	292	solute carrier family 25 member 5 [Source:HGNC Symbol;Acc:HGNC:10991]	4.2	2
366	*DNM1L*	10059	dynamin 1-like [Source:HGNC Symbol;Acc:HGNC:2973]	4.2	2
367	*EIF1AY*	9086	eukaryotic translation initiation factor 1A, Y-linked [Source:HGNC Symbol;Acc:HGNC:3252]	4.2	2
368	*HMOX1*	3162	heme oxygenase 1 [Source:HGNC Symbol;Acc:HGNC:5013]	4.2	2
369	*FKBP3*	2287	FK506 binding protein 3 [Source:HGNC Symbol;Acc:HGNC:3719]	4.2	2
370	*CD36*	948	CD36 molecule [Source:HGNC Symbol;Acc:HGNC:1663]	4.2	2
371	*MAEA*	10296	macrophage erythroblast attacher [Source:HGNC Symbol;Acc:HGNC:13731]	4.2	2
372	*DOK3*	79930	docking protein 3 [Source:HGNC Symbol;Acc:HGNC:24583]	4.2	2
373	*PXN*	5829	paxillin [Source:HGNC Symbol;Acc:HGNC:9718]	4.2	2

374	*NUDT1*	4521	nudix hydrolase 1 [Source:HGNC Symbol;Acc:HGNC:8048]	4.2	2
375	*PCGF3*	10336	polycomb group ring finger 3 [Source:HGNC Symbol;Acc:HGNC:10066]	4.2	2
376	*SDHB*	6390	succinate dehydrogenase complex iron sulfur subunit B [Source:HGNC Symbol;Acc:HGNC:10681]	4.2	2
377	*SEMA4D*	10507	semaphorin 4D [Source:HGNC Symbol;Acc:HGNC:10732]	4.1	2
378	*RNF5*	6048	ring finger protein 5 [Source:HGNC Symbol;Acc:HGNC:10068]	4.1	2
379	*HIST2H2BE*	8349	histone cluster 2, H2be [Source:HGNC Symbol;Acc:HGNC:4760]	4.1	2
380	*IQGAP3*	128239	IQ motif containing GTPase activating protein 3 [Source:HGNC Symbol;Acc:HGNC:20669]	4.1	2
381	*PRPF31*	26121	pre-mRNA processing factor 31 [Source:HGNC Symbol;Acc:HGNC:15446]	4.1	2
382	*FNTA*	2339	farnesyltransferase, CAAX box, alpha [Source:HGNC Symbol;Acc:HGNC:3782]	4.1	2
383	*CCR7*	1236	C-C motif chemokine receptor 7 [Source:HGNC Symbol;Acc:HGNC:1608]	4.1	2
384	*RSL24D1*	51187	ribosomal L24 domain containing 1 [Source:HGNC Symbol;Acc:HGNC:18479]	4.1	2
385	*UBE2L6*	9246	ubiquitin conjugating enzyme E2 L6 [Source:HGNC Symbol;Acc:HGNC:12490]	4.1	2
386	*ITGB3*	3690	integrin subunit beta 3 [Source:HGNC Symbol;Acc:HGNC:6156]	4.1	2
387	*PAGE2*	203569	PAGE family member 2 [Source:HGNC Symbol;Acc:HGNC:31804]	4.1	2
388	*PTCRA*	171558	pre T-cell antigen receptor alpha [Source:HGNC Symbol;Acc:HGNC:21290]	4.1	2
389	*LCN2*	3934	lipocalin 2 [Source:HGNC Symbol;Acc:HGNC:6526]	4.1	2
390	*NBEAL2*	23218	neurobeachin like 2 [Source:HGNC Symbol;Acc:HGNC:31928]	4.1	2
391	*SUV420H1*	51111	Lysine Methyltransferase 5B	4.1	2
392	*ANKRD28*	23243	ankyrin repeat domain 28 [Source:HGNC Symbol;Acc:HGNC:29024]	4.1	2
393	*SCLY*	51540	selenocysteine lyase [Source:HGNC Symbol;Acc:HGNC:18161]	4.1	2
394	*ALDH18A1*	5832	aldehyde dehydrogenase 18 family member A1 [Source:HGNC Symbol;Acc:HGNC:9722]	4.1	2
395	*SERPINE1*	5054	serpin family E member 1 [Source:HGNC Symbol;Acc:HGNC:8583]	4.1	2
396	*CEP57*	9702	centrosomal protein 57 [Source:HGNC Symbol;Acc:HGNC:30794]	4.1	2
397	*ZNF263*	10127	zinc finger protein 263 [Source:HGNC Symbol;Acc:HGNC:13056]	4.1	2
398	*TCTN3*	26123	tectonic family member 3 [Source:HGNC Symbol;Acc:HGNC:24519]	4.1	2
399	*SLC25A10*	1468	solute carrier family 25 member 10 [Source:HGNC Symbol;Acc:HGNC:10980]	4.1	2
400	*CRIM1*	51232	cysteine rich transmembrane BMP regulator 1 (chordin-like) [Source:HGNC Symbol;Acc:HGNC:2359]	4.1	2
401	*SLC11A1*	6556	solute carrier family 11 member 1 [Source:HGNC Symbol;Acc:HGNC:10907]	4.1	2
402	*FAM110A*	83541	family with sequence similarity 110 member A [Source:HGNC Symbol;Acc:HGNC:16188]	4.1	2
403	*CCDC58*	131076	coiled-coil domain containing 58 [Source:HGNC Symbol;Acc:HGNC:31136]	4.1	2
404	*TMCO1*	54499	transmembrane and coiled-coil domains 1 [Source:HGNC Symbol;Acc:HGNC:18188]	4.1	2
405	*LGALSL*	29094	galectin like [Source:HGNC Symbol;Acc:HGNC:25012]	4.1	2
406	*XRCC4*	7518	X-ray repair cross complementing 4 [Source:HGNC Symbol;Acc:HGNC:12831]	4.1	2
407	*TPM1*	7168	tropomyosin 1 (alpha) [Source:HGNC Symbol;Acc:HGNC:12010]	4.1	2
408	*CXCR2*	3579	C-X-C motif chemokine receptor 2 [Source:HGNC Symbol;Acc:HGNC:6027]	4.1	2
409	*MYL9*	10398	myosin light chain 9 [Source:HGNC Symbol;Acc:HGNC:15754]	4.1	2
410	*KRTCAP3*	200634	keratinocyte associated protein 3 [Source:HGNC Symbol;Acc:HGNC:28943]	4.1	2
411	*PHF6*	84295	PHD finger protein 6 [Source:HGNC Symbol;Acc:HGNC:18145]	4.1	2
412	*FPR1*	2357	formyl peptide receptor 1 [Source:HGNC Symbol;Acc:HGNC:3826]	4.1	2
413	*ZBTB44*	29068	zinc finger and BTB domain containing 44 [Source:HGNC Symbol;Acc:HGNC:25001]	4.1	2
414	*CD37*	951	CD37 molecule [Source:HGNC Symbol;Acc:HGNC:1666]	4.1	2
415	*FAM162A*	26355	family with sequence similarity 162 member A [Source:HGNC Symbol;Acc:HGNC:17865]	4.0	2
416	*MARCH2*	51257	membrane associated ring-CH-type finger 2 [Source:HGNC Symbol;Acc:HGNC:28038]	4.0	2
417	*STX11*	8676	syntaxin 11 [Source:HGNC Symbol;Acc:HGNC:11429]	4.0	2
418	*NOP56*	10528	NOP56 ribonucleoprotein [Source:HGNC Symbol;Acc:HGNC:15911]	4.0	2
419	*CLEC2B*	9976	C-type lectin domain family 2 member B [Source:HGNC Symbol;Acc:HGNC:2053]	4.0	2
420	*RBCK1*	10616	RANBP2-type and C3HC4-type zinc finger containing 1 [Source:HGNC Symbol;Acc:HGNC:15864]	4.0	2
421	*CYB5A*	1528	cytochrome b5 type A [Source:HGNC Symbol;Acc:HGNC:2570]	4.0	2
422	*METAP2*	10988	methionyl aminopeptidase 2 [Source:HGNC Symbol;Acc:HGNC:16672]	4.0	2
423	*ADCY7*	113	adenylate cyclase 7 [Source:HGNC Symbol;Acc:HGNC:238]	4.0	2
424	*ESAM*	90952	endothelial cell adhesion molecule [Source:HGNC Symbol;Acc:HGNC:17474]	4.0	2
425	*EPCAM*	4072	epithelial cell adhesion molecule [Source:HGNC Symbol;Acc:HGNC:11529]	4.0	2
426	*BIRC5*	332	baculoviral IAP repeat containing 5 [Source:HGNC Symbol;Acc:HGNC:593]	4.0	2
427	*MEGF9*	1955	multiple EGF like domains 9 [Source:HGNC Symbol;Acc:HGNC:3234]	4.0	2
428	*DECR1*	1666	2,4-dienoyl-CoA reductase 1, mitochondrial [Source:HGNC Symbol;Acc:HGNC:2753]	4.0	2
429	*RNF34*	80196	ring finger protein 34 [Source:HGNC Symbol;Acc:HGNC:17297]	4.0	2
430	*PRPF40A*	55660	pre-mRNA processing factor 40 homolog A [Source:HGNC Symbol;Acc:HGNC:16463]	4.0	2
431	*CCL5*	6352	C-C motif chemokine ligand 5 [Source:HGNC Symbol;Acc:HGNC:10632]	4.0	2
432	*NFIC*	4782	nuclear factor I C [Source:HGNC Symbol;Acc:HGNC:7786]	4.0	2
433	*SMIM5*	643008	small integral membrane protein 5 [Source:HGNC Symbol;Acc:HGNC:40030]	4.0	2
434	*PLAA*	9373	phospholipase A2 activating protein [Source:HGNC Symbol;Acc:HGNC:9043]	4.0	2
435	*CR1*	1378	complement component 3b/4b receptor 1 (Knops blood group) [Source:HGNC Symbol;Acc:HGNC:2334]	4.0	2
436	*VNN2*	8875	vanin 2 [Source:HGNC Symbol;Acc:HGNC:12706]	4.0	2
437	*CENPH*	64946	centromere protein H [Source:HGNC Symbol;Acc:HGNC:17268]	4.0	2
438	*VAPB*	9217	VAMP (vesicle-associated membrane protein)-associated protein B and C [Source:HGNC Symbol;Acc:HGNC:12649]	4.0	2
439	*HBG2*	3048	hemoglobin subunit gamma 2 [Source:HGNC Symbol;Acc:HGNC:4832]	4.0	2
440	*DOK2*	9046	docking protein 2 [Source:HGNC Symbol;Acc:HGNC:2991]	4.0	2
441	*PCSK6*	5046	proprotein convertase subtilisin/kexin type 6 [Source:HGNC Symbol;Acc:HGNC:8569]	4.0	2
442	*HGD*	3081	homogentisate 1,2-dioxygenase [Source:HGNC Symbol;Acc:HGNC:4892]	4.0	2
443	*GRINA*	2907	glutamate ionotropic receptor NMDA type subunit associated protein 1 [Source:HGNC Symbol;Acc:HGNC:4589]	4.0	2
444	*PPP1R10*	5514	protein phosphatase 1 regulatory subunit 10 [Source:HGNC Symbol;Acc:HGNC:9284]	4.0	2
445	*ARHGAP6*	395	Rho GTPase activating protein 6 [Source:HGNC Symbol;Acc:HGNC:676]	4.0	2
446	*ZDHHC9*	51114	zinc finger DHHC-type containing 9 [Source:HGNC Symbol;Acc:HGNC:18475]	4.0	2
447	*AKAP1*	8165	A-kinase anchoring protein 1 [Source:HGNC Symbol;Acc:HGNC:367]	4.0	2
448	*UNC119B*	84747	unc-119 lipid binding chaperone B [Source:HGNC Symbol;Acc:HGNC:16488]	4.0	2
449	*VPS37C*	55048	VPS37C, ESCRT-I subunit [Source:HGNC Symbol;Acc:HGNC:26097]	4.0	2

450	*LYL1*	4066	lymphoblastic leukemia associated hematopoiesis regulator 1 [Source:HGNC Symbol;Acc:HGNC:6734]	4.0	2
451	*ERAP2*	64167	endoplasmic reticulum aminopeptidase 2 [Source:HGNC Symbol;Acc:HGNC:29499]	4.0	2
452	*IKZF1*	10320	IKAROS family zinc finger 1 [Source:HGNC Symbol;Acc:HGNC:13176]	3.9	2
453	*SELL*	6402	selectin L [Source:HGNC Symbol;Acc:HGNC:10720]	3.9	2
454	*CBR4*	84869	carbonyl reductase 4 [Source:HGNC Symbol;Acc:HGNC:25891]	3.9	2
455	*CMTM2*	146225	CKLF like MARVEL transmembrane domain containing 2 [Source:HGNC Symbol;Acc:HGNC:19173]	3.9	2
456	*NAA50*	80218	N(alpha)-acetyltransferase 50, NatE catalytic subunit [Source:HGNC Symbol;Acc:HGNC:29533]	3.9	2
457	*LAMTOR5*	10542	late endosomal/lysosomal adaptor, MAPK and MTOR activator 5 [Source:HGNC Symbol;Acc:HGNC:17955]	3.9	2
458	*CCDC86*	79080	coiled-coil domain containing 86 [Source:HGNC Symbol;Acc:HGNC:28359]	3.9	2
459	*HDAC3*	8841	histone deacetylase 3 [Source:HGNC Symbol;Acc:HGNC:4854]	3.9	2
460	*NPFFR2*	10886	neuropeptide FF receptor 2 [Source:HGNC Symbol;Acc:HGNC:4525]	3.9	2
461	*MLH3*	27030	mutL homolog 3 [Source:HGNC Symbol;Acc:HGNC:7128]	3.9	2
462	*CCDC53*	51019	coiled-coil domain containing 53 [Source:HGNC Symbol;Acc:HGNC:24256]	3.9	2
463	*GRB10*	2887	growth factor receptor bound protein 10 [Source:HGNC Symbol;Acc:HGNC:4564]	3.9	2
464	*EHD3*	30845	EH domain containing 3 [Source:HGNC Symbol;Acc:HGNC:3244]	3.9	2
465	*PREX1*	57580	phosphatidylinositol-3,4,5-trisphosphate dependent Rac exchange factor 1 [Source:HGNC Symbol;Acc:HGNC:32594]	3.9	2
466	*GP6*	51206	glycoprotein VI platelet [Source:HGNC Symbol;Acc:HGNC:14388]	3.9	2
467	*HBS1L*	10767	HBS1 like translational GTPase [Source:HGNC Symbol;Acc:HGNC:4834]	3.9	2
468	*HIPK2*	28996	homeodomain interacting protein kinase 2 [Source:HGNC Symbol;Acc:HGNC:14402]	3.9	2
469	*CBR1*	873	carbonyl reductase 1 [Source:HGNC Symbol;Acc:HGNC:1548]	3.9	2
470	*BAZ1B*	9031	bromodomain adjacent to zinc finger domain 1B [Source:HGNC Symbol;Acc:HGNC:961]	3.9	2
471	*SMIM15*	643155	small integral membrane protein 15 [Source:HGNC Symbol;Acc:HGNC:33861]	3.9	2
472	*UBE2K*	3093	ubiquitin conjugating enzyme E2 K [Source:HGNC Symbol;Acc:HGNC:4914]	3.9	2
473	*PPFIA1*	8500	PTPRF interacting protein alpha 1 [Source:HGNC Symbol;Acc:HGNC:9245]	3.9	2
474	*MAP2K3*	5606	mitogen-activated protein kinase kinase 3 [Source:HGNC Symbol;Acc:HGNC:6843]	3.9	2
475	*PREPL*	9581	prolyl endopeptidase-like [Source:HGNC Symbol;Acc:HGNC:30228]	3.9	2
476	*ARHGAP9*	64333	Rho GTPase activating protein 9 [Source:HGNC Symbol;Acc:HGNC:14130]	3.9	2
477	*OCIAD2*	132299	OCIA domain containing 2 [Source:HGNC Symbol;Acc:HGNC:28685]	3.9	2
478	*BRD9*	65980	bromodomain containing 9 [Source:HGNC Symbol;Acc:HGNC:25818]	3.9	2
479	*NUDT5*	11164	nudix hydrolase 5 [Source:HGNC Symbol;Acc:HGNC:8052]	3.9	2
480	*DEPDC1B*	55789	DEP domain containing 1B [Source:HGNC Symbol;Acc:HGNC:24902]	3.9	2
481	*TNFRSF10C*	8794	tumor necrosis factor receptor superfamily member 10c [Source:HGNC Symbol;Acc:HGNC:11906]	3.9	2
482	*CT45A1*	541466	cancer/testis antigen family 45, member A1 [Source:HGNC Symbol;Acc:HGNC:33267]	3.9	2
483	*PIM3*	415116	Pim-3 proto-oncogene, serine/threonine kinase [Source:HGNC Symbol;Acc:HGNC:19310]	3.9	2
484	*SLC24A4*	123041	solute carrier family 24 member 4 [Source:HGNC Symbol;Acc:HGNC:10978]	3.9	2
485	*TCERG1*	10915	transcription elongation regulator 1 [Source:HGNC Symbol;Acc:HGNC:15630]	3.9	2
486	*HCAR3*	8843	hydroxycarboxylic acid receptor 3 [Source:HGNC Symbol;Acc:HGNC:16824]	3.9	2
487	*UBP1*	7342	upstream binding protein 1 (LBP-1a) [Source:HGNC Symbol;Acc:HGNC:12507]	3.9	2
488	*BET1*	10282	Bet1 golgi vesicular membrane trafficking protein [Source:HGNC Symbol;Acc:HGNC:14562]	3.9	2
489	*GAB1*	2549	GRB2 associated binding protein 1 [Source:HGNC Symbol;Acc:HGNC:4066]	3.9	2
490	*ATG16L2*	89849	autophagy related 16 like 2 [Source:HGNC Symbol;Acc:HGNC:25464]	3.9	2
491	*GSK3B*	2932	glycogen synthase kinase 3 beta [Source:HGNC Symbol;Acc:HGNC:4617]	3.9	2
492	*IQSEC1*	9922	IQ motif and Sec7 domain 1 [Source:HGNC Symbol;Acc:HGNC:29112]	3.9	2
493	*CAV2*	858	caveolin 2 [Source:HGNC Symbol;Acc:HGNC:1528]	3.9	2
494	*DNM3*	26052	dynamin 3 [Source:HGNC Symbol;Acc:HGNC:29125]	3.9	2
495	*BIN2*	51411	bridging integrator 2 [Source:HGNC Symbol;Acc:HGNC:1053]	3.9	2
496	*IL6R*	3570	interleukin 6 receptor [Source:HGNC Symbol;Acc:HGNC:6019]	3.8	2
497	*VPS13A*	23230	vacuolar protein sorting 13 homolog A [Source:HGNC Symbol;Acc:HGNC:1908]	3.8	2
498	*EIF5A*	1984	eukaryotic translation initiation factor 5A [Source:HGNC Symbol;Acc:HGNC:3300]	3.8	2
499	*NUFIP2*	57532	NUFIP2, FMR1 interacting protein 2 [Source:HGNC Symbol;Acc:HGNC:17634]	3.8	2
500	*PNRC2*	55629	proline rich nuclear receptor coactivator 2 [Source:HGNC Symbol;Acc:HGNC:23158]	3.8	2
501	*SMOX*	54498	spermine oxidase [Source:HGNC Symbol;Acc:HGNC:15862]	3.8	2
502	*G6PD*	2539	glucose-6-phosphate dehydrogenase [Source:HGNC Symbol;Acc:HGNC:4057]	3.8	2
503	*TWISTNB*	221830	TWIST neighbor [Source:HGNC Symbol;Acc:HGNC:18027]	3.8	2
504	*MLXIP*	22877	MLX interacting protein [Source:HGNC Symbol;Acc:HGNC:17055]	3.8	2
505	*DCAF5*	8816	DDB1 and CUL4 associated factor 5 [Source:HGNC Symbol;Acc:HGNC:20224]	3.8	2
506	*DDX19A*	55308	DEAD-box helicase 19A [Source:HGNC Symbol;Acc:HGNC:25628]	3.8	2
507	*MYO1F*	4542	myosin IF [Source:HGNC Symbol;Acc:HGNC:7600]	3.8	2
508	*MRPL43*	84545	mitochondrial ribosomal protein L43 [Source:HGNC Symbol;Acc:HGNC:14517]	3.8	2
509	*C6orf25*	80739	chromosome 6 open reading frame 25 [Source:HGNC Symbol;Acc:HGNC:13937]	3.8	2
510	*TMEM147*	10430	transmembrane protein 147 [Source:HGNC Symbol;Acc:HGNC:30414]	3.8	2
511	*BCAS3*	54828	breast carcinoma amplified sequence 3 [Source:HGNC Symbol;Acc:HGNC:14347]	3.8	2
512	*TRIB1*	10221	tribbles pseudokinase 1 [Source:HGNC Symbol;Acc:HGNC:16891]	3.8	2
513	*KIF2A*	3796	kinesin heavy chain member 2A [Source:HGNC Symbol;Acc:HGNC:6318]	3.8	2
514	*TGFB1*	7040	transforming growth factor beta 1 [Source:HGNC Symbol;Acc:HGNC:11766]	3.8	2
515	*SUPT16H*	11198	SPT16 homolog, facilitates chromatin remodeling subunit [Source:HGNC Symbol;Acc:HGNC:11465]	3.8	2
516	*ERP44*	23071	endoplasmic reticulum protein 44 [Source:HGNC Symbol;Acc:HGNC:18311]	3.8	2
517	*MYZAP*	100820829	myocardial zonula adherens protein [Source:HGNC Symbol;Acc:HGNC:43444]	3.8	2
518	*ARID1A*	8289	AT-rich interaction domain 1A [Source:HGNC Symbol;Acc:HGNC:11110]	3.8	2
519	*TIMM10*	26519	translocase of inner mitochondrial membrane 10 homolog (yeast) [Source:HGNC Symbol;Acc:HGNC:11814]	3.8	2
520	*TRAPPC6B*	122553	trafficking protein particle complex 6B [Source:HGNC Symbol;Acc:HGNC:23066]	3.8	2
521	*NOP16*	51491	NOP16 nucleolar protein [Source:HGNC Symbol;Acc:HGNC:26934]	3.8	2
522	*CD96*	10225	CD96 molecule [Source:HGNC Symbol;Acc:HGNC:16892]	3.8	2
523	*HAL*	3034	histidine ammonia-lyase [Source:HGNC Symbol;Acc:HGNC:4806]	3.8	2
524	*SSX2*	6757	SSX family member 2 [Source:HGNC Symbol;Acc:HGNC:11336]	3.8	2

525	*SSX2B*	727837	SSX family member 2B [Source:HGNC Symbol;Acc:HGNC:22263]	3.8	2
526	*P2RX1*	5023	purinergic receptor P2X 1 [Source:HGNC Symbol;Acc:HGNC:8533]	3.8	2
527	*USO1*	8615	USO1 vesicle transport factor [Source:HGNC Symbol;Acc:HGNC:30904]	3.8	2
528	*RBM25*	58517	RNA binding motif protein 25 [Source:HGNC Symbol;Acc:HGNC:23244]	3.8	2
529	*FAM193A*	8603	family with sequence similarity 193 member A [Source:HGNC Symbol;Acc:HGNC:16822]	3.8	2
530	*ZDHHC18*	84243	zinc finger DHHC-type containing 18 [Source:HGNC Symbol;Acc:HGNC:20712]	3.8	2
531	*STON2*	85439	stonin 2 [Source:HGNC Symbol;Acc:HGNC:30652]	3.8	2
532	*APTX*	54840	aprataxin [Source:HGNC Symbol;Acc:HGNC:15984]	3.8	2
533	*MS4A1*	931	membrane spanning 4-domains A1 [Source:HGNC Symbol;Acc:HGNC:7315]	3.8	2
534	*CLEC4D*	338339	C-type lectin domain family 4 member D [Source:HGNC Symbol;Acc:HGNC:14554]	3.8	2
535	*SELP*	6403	selectin P [Source:HGNC Symbol;Acc:HGNC:10721]	3.8	2
536	*PRTFDC1*	56952	phosphoribosyl transferase domain containing 1 [Source:HGNC Symbol;Acc:HGNC:23333]	3.8	2
537	*C21orf33*	8209	chromosome 21 open reading frame 33 [Source:HGNC Symbol;Acc:HGNC:1273]	3.8	2
538	*CT45A4*	441520	cancer/testis antigen family 45, member A4	3.8	2
539	*CSE1L*	1434	chromosome segregation 1 like [Source:HGNC Symbol;Acc:HGNC:2431]	3.8	2
540	*ZDHHC16*	84287	zinc finger DHHC-type containing 16 [Source:HGNC Symbol;Acc:HGNC:20714]	3.8	2
541	*SEPT5*	5413	septin 5 [Source:HGNC Symbol;Acc:HGNC:9164]	3.8	2
542	*MRPS30*	10884	mitochondrial ribosomal protein S30 [Source:HGNC Symbol;Acc:HGNC:8769]	3.7	2
543	*CT45A2*	728911	cancer/testis antigen family 45, member A2 [Source:HGNC Symbol;Acc:HGNC:28400]	3.7	2
544	*CT45A3*	441519	cancer/testis antigen family 45, member A3 [Source:HGNC Symbol;Acc:HGNC:33268]	3.7	2
545	*TP53BP1*	7158	tumor protein p53 binding protein 1 [Source:HGNC Symbol;Acc:HGNC:11999]	3.7	2
546	*SEC13*	6396	SEC13 homolog, nuclear pore and COPII coat complex component [Source:HGNC Symbol;Acc:HGNC:10697]	3.7	2
547	*CAPZB*	832	capping actin protein of muscle Z-line beta subunit [Source:HGNC Symbol;Acc:HGNC:1491]	3.7	2
548	*S100B*	6285	S100 calcium binding protein B [Source:HGNC Symbol;Acc:HGNC:10500]	3.7	2
549	*COPS2*	9318	COP9 signalosome subunit 2 [Source:HGNC Symbol;Acc:HGNC:30747]	3.7	2
550	*KRT1*	3848	keratin 1 [Source:HGNC Symbol;Acc:HGNC:6412]	3.7	2
551	*SNX6*	58533	sorting nexin 6 [Source:HGNC Symbol;Acc:HGNC:14970]	3.7	2
552	*SNCA*	6622	synuclein alpha [Source:HGNC Symbol;Acc:HGNC:11138]	3.7	2
553	*ECHDC2*	55268	enoyl-CoA hydratase domain containing 2 [Source:HGNC Symbol;Acc:HGNC:23408]	3.7	2
554	*KHDRBS1*	10657	KH RNA binding domain containing, signal transduction associated 1 [Source:HGNC Symbol;Acc:HGNC:18116]	3.7	2
555	*ACSBG1*	23205	acyl-CoA synthetase bubblegum family member 1 [Source:HGNC Symbol;Acc:HGNC:29567]	3.7	2
556	*ZNF644*	84146	zinc finger protein 644 [Source:HGNC Symbol;Acc:HGNC:29222]	3.7	2
557	*ATP5E*	514	ATP synthase, H+ transporting, mitochondrial F1 complex, epsilon subunit [Source:HGNC Symbol;Acc:HGNC:838]	3.7	2
558	*TCOF1*	6949	treacle ribosome biogenesis factor 1 [Source:HGNC Symbol;Acc:HGNC:11654]	3.7	2
559	*LST1*	7940	leukocyte specific transcript 1 [Source:HGNC Symbol;Acc:HGNC:14189]	3.7	2
560	*XK*	7504	X-linked Kx blood group [Source:HGNC Symbol;Acc:HGNC:12811]	3.7	2
561	*CDC25C*	995	cell division cycle 25C [Source:HGNC Symbol;Acc:HGNC:1727]	3.7	2
562	*AGBL5*	60509	ATP/GTP binding protein-like 5 [Source:HGNC Symbol;Acc:HGNC:26147]	3.7	2
563	*MYO1G*	64005	myosin IG [Source:HGNC Symbol;Acc:HGNC:13880]	3.7	2
564	*MRPL42*	28977	mitochondrial ribosomal protein L42 [Source:HGNC Symbol;Acc:HGNC:14493]	3.7	2
565	*AIMP2*	7965	aminoacyl tRNA synthetase complex interacting multifunctional protein 2 [Source:HGNC Symbol;Acc:HGNC:20609]	3.7	2
566	*AQP9*	366	aquaporin 9 [Source:HGNC Symbol;Acc:HGNC:643]	3.7	2
567	*CASP7*	840	caspase 7 [Source:HGNC Symbol;Acc:HGNC:1508]	3.7	2
568	*MYL12B*	103910	myosin light chain 12B [Source:HGNC Symbol;Acc:HGNC:29827]	3.7	2
569	*CYTH2*	9266	cytohesin 2 [Source:HGNC Symbol;Acc:HGNC:9502]	3.7	2
570	*C14orf119*	55017	chromosome 14 open reading frame 119 [Source:HGNC Symbol;Acc:HGNC:20270]	3.7	2
571	*LILRA1*	11024	leukocyte immunoglobulin like receptor A1 [Source:HGNC Symbol;Acc:HGNC:6602]	3.7	2
572	*GATAD1*	57798	GATA zinc finger domain containing 1 [Source:HGNC Symbol;Acc:HGNC:29941]	3.7	2
573	*NRBP2*	340371	nuclear receptor binding protein 2 [Source:HGNC Symbol;Acc:HGNC:19339]	3.7	2
574	*UBE2A*	7319	ubiquitin conjugating enzyme E2 A [Source:HGNC Symbol;Acc:HGNC:12472]	3.7	2
575	*BCAS2*	10286	breast carcinoma amplified sequence 2 [Source:HGNC Symbol;Acc:HGNC:975]	3.7	2
576	*PLP2*	5355	proteolipid protein 2 [Source:HGNC Symbol;Acc:HGNC:9087]	3.7	2
577	*ARHGAP30*	257106	Rho GTPase activating protein 30 [Source:HGNC Symbol;Acc:HGNC:27414]	3.7	2
578	*LSM1*	27257	LSM1 homolog, mRNA degradation associated [Source:HGNC Symbol;Acc:HGNC:20472]	3.7	2
579	*ID3*	3399	inhibitor of DNA binding 3, HLH protein [Source:HGNC Symbol;Acc:HGNC:5362]	3.7	2
580	*CYB5B*	80777	cytochrome b5 type B [Source:HGNC Symbol;Acc:HGNC:24374]	3.7	2
581	*SLC35F2*	54733	solute carrier family 35 member F2 [Source:HGNC Symbol;Acc:HGNC:23615]	3.7	2
582	*DYSF*	8291	dysferlin [Source:HGNC Symbol;Acc:HGNC:3097]	3.7	2
583	*GTF2E2*	2961	general transcription factor IIE subunit 2 [Source:HGNC Symbol;Acc:HGNC:4651]	3.7	2
584	*FOXK2*	3607	forkhead box K2 [Source:HGNC Symbol;Acc:HGNC:6036]	3.7	2
585	*IL17RA*	23765	interleukin 17 receptor A [Source:HGNC Symbol;Acc:HGNC:5985]	3.7	2
586	*LPIN2*	9663	lipin 2 [Source:HGNC Symbol;Acc:HGNC:14450]	3.7	2
587	*CADPS2*	93664	calcium dependent secretion activator 2 [Source:HGNC Symbol;Acc:HGNC:16018]	3.7	2
588	*ACTR1A*	10121	ARP1 actin-related protein 1 homolog A, centractin alpha [Source:HGNC Symbol;Acc:HGNC:167]	3.7	2
589	*KIF22*	3835	kinesin family member 22 [Source:HGNC Symbol;Acc:HGNC:6391]	3.7	2
590	*MRPL12*	6182	mitochondrial ribosomal protein L12 [Source:HGNC Symbol;Acc:HGNC:10378]	3.7	2
591	*HBA1*	3039	hemoglobin subunit alpha 1 [Source:HGNC Symbol;Acc:HGNC:4823]	3.7	2
592	*MAST4*	375449	microtubule associated serine/threonine kinase family member 4 [Source:HGNC Symbol;Acc:HGNC:19037]	3.7	2
593	*TARS*	6897	threonyl-tRNA synthetase [Source:HGNC Symbol;Acc:HGNC:11572]	3.7	2
594	*ST13*	6767	suppression of tumorigenicity 13 (colon carcinoma) (Hsp70 interacting protein) [Source:HGNC Symbol;Acc:HGNC:11343]	3.7	2
595	*KIAA0232*	9778	KIAA0232 [Source:HGNC Symbol;Acc:HGNC:28992]	3.7	2
596	*ZC3H13*	23091	zinc finger CCCH-type containing 13 [Source:HGNC Symbol;Acc:HGNC:20368]	3.7	2
597	*DYNLT3*	6990	dynein light chain Tctex-type 3 [Source:HGNC Symbol;Acc:HGNC:11694]	3.7	2
598	*ARHGEF37*	389337	Rho guanine nucleotide exchange factor 37 [Source:HGNC Symbol;Acc:HGNC:34430]	3.7	2
599	*BTG1*	694	B-cell translocation gene 1, anti-proliferative [Source:HGNC Symbol;Acc:HGNC:1130]	3.7	2

600	*SH3BGRL2*	83699	SH3 domain binding glutamate rich protein like 2 [Source:HGNC Symbol;Acc:HGNC:15567]	3.7	2
601	*LILRB3*	11025	leukocyte immunoglobulin like receptor B3 [Source:HGNC Symbol;Acc:HGNC:6607]	3.7	2
602	*SEC14L5*	9717	SEC14 like lipid binding 5 [Source:HGNC Symbol;Acc:HGNC:29032]	3.6	2
603	*CDC23*	8697	cell division cycle 23 [Source:HGNC Symbol;Acc:HGNC:1724]	3.6	2
604	*RAB40B*	10966	RAB40B, member RAS oncogene family [Source:HGNC Symbol;Acc:HGNC:18284]	3.6	2
605	*NCOA1*	8648	nuclear receptor coactivator 1 [Source:HGNC Symbol;Acc:HGNC:7668]	3.6	2
606	*DSP*	1832	desmoplakin [Source:HGNC Symbol;Acc:HGNC:3052]	3.6	2
607	*HIST1H2BG*	8339	histone cluster 1, H2bg [Source:HGNC Symbol;Acc:HGNC:4746]	3.6	2
608	*HIST1H2AC*	8334	histone cluster 1, H2ac [Source:HGNC Symbol;Acc:HGNC:4733]	3.6	2
609	*MNDA*	4332	myeloid cell nuclear differentiation antigen [Source:HGNC Symbol;Acc:HGNC:7183]	3.6	2
610	*MALSU1*	115416	mitochondrial assembly of ribosomal large subunit 1 [Source:HGNC Symbol;Acc:HGNC:21721]	3.6	2
611	*RTCB*	51493	RNA 2',3'-cyclic phosphate and 5'-OH ligase [Source:HGNC Symbol;Acc:HGNC:26935]	3.6	2
612	*KLF10*	7071	Kruppel like factor 10 [Source:HGNC Symbol;Acc:HGNC:11810]	3.6	2
613	*TLE3*	7090	transducin like enhancer of split 3 [Source:HGNC Symbol;Acc:HGNC:11839]	3.6	2
614	*KLHL18*	23276	kelch like family member 18 [Source:HGNC Symbol;Acc:HGNC:29120]	3.6	2
615	*PIK3CD*	5293	phosphatidylinositol-4,5-bisphosphate 3-kinase catalytic subunit delta [Source:HGNC Symbol;Acc:HGNC:8977]	3.6	2
616	*FAM120A*	23196	family with sequence similarity 120A [Source:HGNC Symbol;Acc:HGNC:13247]	3.6	2
617	*TADA1*	117143	transcriptional adaptor 1 [Source:HGNC Symbol;Acc:HGNC:30631]	3.6	2
618	*TSPAN32*	10077	tetraspanin 32 [Source:HGNC Symbol;Acc:HGNC:13410]	3.6	2
619	*UBE2V1*	7335	ubiquitin conjugating enzyme E2 V1 [Source:HGNC Symbol;Acc:HGNC:12494]	3.6	2
620	*VCL*	7414	vinculin [Source:HGNC Symbol;Acc:HGNC:12665]	3.6	2
621	*SYT16*	83851	synaptotagmin 16 [Source:HGNC Symbol;Acc:HGNC:23142]	3.6	2
622	*PSMG3*	84262	proteasome assembly chaperone 3 [Source:HGNC Symbol;Acc:HGNC:22420]	3.6	2
623	*PQBP1*	10084	polyglutamine binding protein 1 [Source:HGNC Symbol;Acc:HGNC:9330]	3.6	2
624	*ADGRE1*	2015	adhesion G protein-coupled receptor E1 [Source:HGNC Symbol;Acc:HGNC:3336]	3.6	2
625	*CTSW*	1521	cathepsin W [Source:HGNC Symbol;Acc:HGNC:2546]	3.6	2
626	*TRMT112*	51504	tRNA methyltransferase 11-2 homolog (S. cerevisiae) [Source:HGNC Symbol;Acc:HGNC:26940]	3.6	2
627	*IL18RAP*	8807	interleukin 18 receptor accessory protein [Source:HGNC Symbol;Acc:HGNC:5989]	3.6	2
628	*THEMIS2*	9473	thymocyte selection associated family member 2 [Source:HGNC Symbol;Acc:HGNC:16839]	3.6	2
629	*NFKBIA*	4792	NFKB inhibitor alpha [Source:HGNC Symbol;Acc:HGNC:7797]	3.6	2
630	*MGLL*	11343	monoglyceride lipase [Source:HGNC Symbol;Acc:HGNC:17038]	3.6	2
631	*IDI1*	3422	isopentenyl-diphosphate delta isomerase 1 [Source:HGNC Symbol;Acc:HGNC:5387]	3.6	2
632	*IDI2*	91734	isopentenyl-diphosphate delta isomerase 2 [Source:HGNC Symbol;Acc:HGNC:23487]	3.6	2
633	*DAB2*	1601	DAB2, clathrin adaptor protein [Source:HGNC Symbol;Acc:HGNC:2662]	3.6	2
634	*NKG7*	4818	natural killer cell granule protein 7 [Source:HGNC Symbol;Acc:HGNC:7830]	3.6	2
635	*COMMD5*	28991	COMM domain containing 5 [Source:HGNC Symbol;Acc:HGNC:17902]	3.6	2
636	*RER1*	11079	retention in endoplasmic reticulum sorting receptor 1 [Source:HGNC Symbol;Acc:HGNC:30309]	3.6	2
637	*GYG1*	2992	glycogenin 1 [Source:HGNC Symbol;Acc:HGNC:4699]	3.6	2
638	*SPAG7*	9552	sperm associated antigen 7 [Source:HGNC Symbol;Acc:HGNC:11216]	3.6	2
639	*RNF169*	254225	ring finger protein 169 [Source:HGNC Symbol;Acc:HGNC:26961]	3.6	2
640	*DROSHA*	29102	drosha ribonuclease III [Source:HGNC Symbol;Acc:HGNC:17904]	3.6	2
641	*MOB3A*	126308	MOB kinase activator 3A [Source:HGNC Symbol;Acc:HGNC:29802]	3.6	2
642	*PTP4A1*	7803	protein tyrosine phosphatase type IVA, member 1 [Source:HGNC Symbol;Acc:HGNC:9634]	3.6	2
643	*TCHP*	84260	trichoplein keratin filament binding [Source:HGNC Symbol;Acc:HGNC:28135]	3.6	2
644	*CKS2*	1164	CDC28 protein kinase regulatory subunit 2 [Source:HGNC Symbol;Acc:HGNC:2000]	3.6	2
645	*GUSB*	2990	glucuronidase beta [Source:HGNC Symbol;Acc:HGNC:4696]	3.6	2
646	*SHCBP1*	79801	SHC binding and spindle associated 1 [Source:HGNC Symbol;Acc:HGNC:29547]	3.6	2
647	*MPP1*	4354	membrane palmitoylated protein 1 [Source:HGNC Symbol;Acc:HGNC:7219]	3.6	2
648	*DLG3*	1741	discs large MAGUK scaffold protein 3 [Source:HGNC Symbol;Acc:HGNC:2902]	3.6	2
649	*PDCD2*	5134	programmed cell death 2 [Source:HGNC Symbol;Acc:HGNC:8762]	3.6	2
650	*MTAP*	4507	methylthioadenosine phosphorylase [Source:HGNC Symbol;Acc:HGNC:7413]	3.6	2
651	*RBFOX2*	23543	RNA binding protein, fox-1 homolog 2 [Source:HGNC Symbol;Acc:HGNC:9906]	3.6	2
652	*SNRPF*	6636	small nuclear ribonucleoprotein polypeptide F [Source:HGNC Symbol;Acc:HGNC:11162]	3.6	2
653	*ACTR2*	10097	ARP2 actin-related protein 2 homolog (yeast) [Source:HGNC Symbol;Acc:HGNC:169]	3.6	2
654	*LILRB1*	10859	leukocyte immunoglobulin like receptor B1 [Source:HGNC Symbol;Acc:HGNC:6605]	3.6	2
655	*MEAF6*	64769	MYST/Esa1 associated factor 6 [Source:HGNC Symbol;Acc:HGNC:25674]	3.6	2
656	*ITGAL*	3683	integrin subunit alpha L [Source:HGNC Symbol;Acc:HGNC:6148]	3.6	2
657	*THUMPD3*	25917	THUMP domain containing 3 [Source:HGNC Symbol;Acc:HGNC:24493]	3.6	2
658	*FNBP4*	23360	formin binding protein 4 [Source:HGNC Symbol;Acc:HGNC:19752]	3.6	2
659	*LMNA*	4000	lamin A/C [Source:HGNC Symbol;Acc:HGNC:6636]	3.6	2
660	*TMEM248*	55069	transmembrane protein 248 [Source:HGNC Symbol;Acc:HGNC:25476]	3.6	2
661	*PLEKHO1*	51177	pleckstrin homology domain containing O1 [Source:HGNC Symbol;Acc:HGNC:24310]	3.6	2
662	*PPP6C*	5537	protein phosphatase 6 catalytic subunit [Source:HGNC Symbol;Acc:HGNC:9323]	3.5	2
663	*ABLIM3*	22885	actin binding LIM protein family member 3 [Source:HGNC Symbol;Acc:HGNC:29132]	3.5	2
664	*TMEM158*	25907	transmembrane protein 158 (gene/pseudogene) [Source:HGNC Symbol;Acc:HGNC:30293]	3.5	2
665	*NOSIP*	51070	nitric oxide synthase interacting protein [Source:HGNC Symbol;Acc:HGNC:17946]	3.5	2
666	*LIMCH1*	22998	LIM and calponin homology domains 1 [Source:HGNC Symbol;Acc:HGNC:29191]	3.5	2
667	*TOMM5*	401505	translocase of outer mitochondrial membrane 5 [Source:HGNC Symbol;Acc:HGNC:31369]	3.5	2
668	*IL12RB1*	3594	interleukin 12 receptor subunit beta 1 [Source:HGNC Symbol;Acc:HGNC:5971]	3.5	2
669	*PDGFA*	5154	platelet derived growth factor subunit A [Source:HGNC Symbol;Acc:HGNC:8799]	3.5	2
670	*UBE2C*	11065	ubiquitin conjugating enzyme E2 C [Source:HGNC Symbol;Acc:HGNC:15937]	3.5	2
671	*SCYL1*	57410	SCY1 like pseudokinase 1 [Source:HGNC Symbol;Acc:HGNC:14372]	3.5	2
672	*FOXN3*	1112	forkhead box N3 [Source:HGNC Symbol;Acc:HGNC:1928]	3.5	2
673	*ACO2*	50	aconitase 2 [Source:HGNC Symbol;Acc:HGNC:118]	3.5	2
674	*HSF1*	3297	heat shock transcription factor 1 [Source:HGNC Symbol;Acc:HGNC:5224]	3.5	2
675	*NSUN6*	221078	NOP2/Sun RNA methyltransferase family member 6 [Source:HGNC Symbol;Acc:HGNC:23529]	3.5	2

676	*TSPAN8*	7103	tetraspanin 8 [Source:HGNC Symbol;Acc:HGNC:11855]	3.5	2
677	*PARVB*	29780	parvin beta [Source:HGNC Symbol;Acc:HGNC:14653]	3.5	2
678	*SHQ1*	55164	SHQ1, H/ACA ribonucleoprotein assembly factor [Source:HGNC Symbol;Acc:HGNC:25543]	3.5	2
679	*AP1M2*	10053	adaptor related protein complex 1 mu 2 subunit [Source:HGNC Symbol;Acc:HGNC:558]	3.5	2
680	*MTURN*	222166	maturin, neural progenitor differentiation regulator homolog (Xenopus) [Source:HGNC Symbol;Acc:HGNC:25457]	3.5	2
681	*INF2*	64423	inverted formin, FH2 and WH2 domain containing [Source:HGNC Symbol;Acc:HGNC:23791]	3.5	2
682	*CARS2*	79587	cysteinyl-tRNA synthetase 2, mitochondrial (putative) [Source:HGNC Symbol;Acc:HGNC:25695]	3.5	2
683	*ORMDL2*	29095	ORMDL sphingolipid biosynthesis regulator 2 [Source:HGNC Symbol;Acc:HGNC:16037]	3.5	2
684	*SASH3*	54440	SAM and SH3 domain containing 3 [Source:HGNC Symbol;Acc:HGNC:15975]	3.5	2
685	*GNG11*	2791	G protein subunit gamma 11 [Source:HGNC Symbol;Acc:HGNC:4403]	3.5	2
686	*TMEM80*	283232	transmembrane protein 80 [Source:HGNC Symbol;Acc:HGNC:27453]	3.5	2
687	*SIRPB1*	10326	signal regulatory protein beta 1 [Source:HGNC Symbol;Acc:HGNC:15928]	3.5	2
688	*BBX*	56987	BBX, HMG-box containing [Source:HGNC Symbol;Acc:HGNC:14422]	3.5	2
689	*DMTN*	2039	dematin actin binding protein [Source:HGNC Symbol;Acc:HGNC:3382]	3.5	2
690	*ARPC1A*	10552	actin related protein 2/3 complex subunit 1A [Source:HGNC Symbol;Acc:HGNC:703]	3.5	2
691	*FANCD2*	2177	Fanconi anemia complementation group D2 [Source:HGNC Symbol;Acc:HGNC:3585]	3.5	2
692	*FANCD2OS*	115795	FANCD2 opposite strand [Source:HGNC Symbol;Acc:HGNC:28623]	3.5	2
693	*VEPH1*	79674	ventricular zone expressed PH domain containing 1 [Source:HGNC Symbol;Acc:HGNC:25735]	3.5	2
694	*SEL1L*	6400	SEL1L ERAD E3 ligase adaptor subunit [Source:HGNC Symbol;Acc:HGNC:10717]	3.5	2
695	*PROSER2*	254427	proline and serine rich 2 [Source:HGNC Symbol;Acc:HGNC:23728]	3.5	2
696	*DCTD*	1635	dCMP deaminase [Source:HGNC Symbol;Acc:HGNC:2710]	3.5	2
697	*PLRG1*	5356	pleiotropic regulator 1 [Source:HGNC Symbol;Acc:HGNC:9089]	3.5	2
698	*METTL5*	29081	methyltransferase like 5 [Source:HGNC Symbol;Acc:HGNC:25006]	3.5	2
699	*SERPINA1*	5265	serpin family A member 1 [Source:HGNC Symbol;Acc:HGNC:8941]	3.5	2
700	*SSX1*	6756	SSX family member 1 [Source:HGNC Symbol;Acc:HGNC:11335]	3.5	2
701	*KDM4B*	23030	lysine demethylase 4B [Source:HGNC Symbol;Acc:HGNC:29136]	3.5	2
702	*MYLK*	4638	myosin light chain kinase [Source:HGNC Symbol;Acc:HGNC:7590]	3.5	2
703	*GMPR*	2766	guanosine monophosphate reductase [Source:HGNC Symbol;Acc:HGNC:4376]	3.5	2
704	*AKNA*	80709	AT-hook transcription factor [Source:HGNC Symbol;Acc:HGNC:24108]	3.5	2
705	*ADPRHL2*	54936	ADP-ribosylhydrolase like 2 [Source:HGNC Symbol;Acc:HGNC:21304]	3.5	2
706	*CD1D*	912	CD1d molecule [Source:HGNC Symbol;Acc:HGNC:1637]	3.5	2
707	*NCAPH*	23397	non-SMC condensin I complex subunit H [Source:HGNC Symbol;Acc:HGNC:1112]	3.5	2
708	*KIF4A*	24137	kinesin family member 4A [Source:HGNC Symbol;Acc:HGNC:13339]	3.5	2
709	*MARCKSL1*	65108	MARCKS like 1 [Source:HGNC Symbol;Acc:HGNC:7142]	3.5	2
710	*CT45A6*	541465	cancer/testis antigen family 45, member A6 [Source:HGNC Symbol;Acc:HGNC:33271]	3.5	2
711	*PHTF2*	57157	putative homeodomain transcription factor 2 [Source:HGNC Symbol;Acc:HGNC:13411]	3.5	2
712	*XRN2*	22803	5'-3' exoribonuclease 2 [Source:HGNC Symbol;Acc:HGNC:12836]	3.5	2
713	*ALDH1B1*	219	aldehyde dehydrogenase 1 family member B1 [Source:HGNC Symbol;Acc:HGNC:407]	3.5	2
714	*VAMP5*	10791	vesicle associated membrane protein 5 [Source:HGNC Symbol;Acc:HGNC:12646]	3.5	2
715	*CDC5L*	988	cell division cycle 5 like [Source:HGNC Symbol;Acc:HGNC:1743]	3.5	2
716	*ADCY3*	109	adenylate cyclase 3 [Source:HGNC Symbol;Acc:HGNC:234]	3.5	2
717	*CENPO*	79172	centromere protein O [Source:HGNC Symbol;Acc:HGNC:28152]	3.5	2
718	*PTRHD1*	391356	peptidyl-tRNA hydrolase domain containing 1 [Source:HGNC Symbol;Acc:HGNC:33782]	3.5	2
719	*DNAJC5*	80331	DnaJ heat shock protein family (Hsp40) member C5 [Source:HGNC Symbol;Acc:HGNC:16235]	3.5	2
720	*TMEM59*	9528	transmembrane protein 59 [Source:HGNC Symbol;Acc:HGNC:1239]	3.4	2
721	*TOB2*	10766	transducer of ERBB2, 2 [Source:HGNC Symbol;Acc:HGNC:11980]	3.4	2
722	*GDI1*	2664	GDP dissociation inhibitor 1 [Source:HGNC Symbol;Acc:HGNC:4226]	3.4	2
723	*MAN1A2*	10905	mannosidase alpha class 1A member 2 [Source:HGNC Symbol;Acc:HGNC:6822]	3.4	2
724	*LRRC42*	115353	leucine rich repeat containing 42 [Source:HGNC Symbol;Acc:HGNC:28792]	3.4	2
725	*CNDP2*	55748	CNDP dipeptidase 2 (metallopeptidase M20 family) [Source:HGNC Symbol;Acc:HGNC:24437]	3.4	2
726	*ICAM3*	3385	intercellular adhesion molecule 3 [Source:HGNC Symbol;Acc:HGNC:5346]	3.4	2
727	*POLR3H*	171568	polymerase (RNA) III subunit H [Source:HGNC Symbol;Acc:HGNC:30349]	3.4	2
728	*MME*	4311	membrane metallo-endopeptidase [Source:HGNC Symbol;Acc:HGNC:7154]	3.4	2
729	*PRPF18*	8559	pre-mRNA processing factor 18 [Source:HGNC Symbol;Acc:HGNC:17351]	3.4	2
730	*ATP9A*	10079	ATPase phospholipid transporting 9A (putative) [Source:HGNC Symbol;Acc:HGNC:13540]	3.4	2
731	*SEMA4G*	57715	semaphorin 4G [Source:HGNC Symbol;Acc:HGNC:10735]	3.4	2
732	*OIP5*	11339	Opa interacting protein 5 [Source:HGNC Symbol;Acc:HGNC:20300]	3.4	2
733	*SCRIB*	23513	scribbled planar cell polarity protein [Source:HGNC Symbol;Acc:HGNC:30377]	3.4	2
734	*PIGS*	94005	phosphatidylinositol glycan anchor biosynthesis class S [Source:HGNC Symbol;Acc:HGNC:14937]	3.4	2
735	*MEN1*	4221	menin 1 [Source:HGNC Symbol;Acc:HGNC:7010]	3.4	2
736	*ITSN2*	50618	intersectin 2 [Source:HGNC Symbol;Acc:HGNC:6184]	3.4	2
737	*EIF3M*	10480	eukaryotic translation initiation factor 3 subunit M [Source:HGNC Symbol;Acc:HGNC:24460]	3.4	2
738	*ARHGAP18*	93663	Rho GTPase activating protein 18 [Source:HGNC Symbol;Acc:HGNC:21035]	3.4	2
739	*MAP1B*	4131	microtubule associated protein 1B [Source:HGNC Symbol;Acc:HGNC:6836]	3.4	2
740	*COX17*	10063	COX17 cytochrome c oxidase copper chaperone [Source:HGNC Symbol;Acc:HGNC:2264]	3.4	2
741	*PGM1*	5236	phosphoglucomutase 1 [Source:HGNC Symbol;Acc:HGNC:8905]	3.4	2
742	*NQO2*	4835	NAD(P)H quinone dehydrogenase 2 [Source:HGNC Symbol;Acc:HGNC:7856]	3.4	2
743	*CYP4F3*	4051	cytochrome P450 family 4 subfamily F member 3 [Source:HGNC Symbol;Acc:HGNC:2646]	3.4	2
744	*LCOR*	84458	ligand dependent nuclear receptor corepressor [Source:HGNC Symbol;Acc:HGNC:29503]	3.4	2
745	*CCDC69*	26112	coiled-coil domain containing 69 [Source:HGNC Symbol;Acc:HGNC:24487]	3.4	2
746	*ZNF268*	10795	zinc finger protein 268 [Source:HGNC Symbol;Acc:HGNC:13061]	3.4	2
747	*ESRRA*	2101	estrogen related receptor alpha [Source:HGNC Symbol;Acc:HGNC:3471]	3.4	2
748	*C5AR1*	728	complement component 5a receptor 1 [Source:HGNC Symbol;Acc:HGNC:1338]	3.4	2

749	*ANKRD9*	122416	ankyrin repeat domain 9 [Source:HGNC Symbol;Acc:HGNC:20096]	3.4	2
750	*AMZ2*	51321	archaelysin family metallopeptidase 2 [Source:HGNC Symbol;Acc:HGNC:28041]	3.4	2
751	*CPNE5*	57699	copine 5 [Source:HGNC Symbol;Acc:HGNC:2318]	3.4	2
752	*GP5*	2814	glycoprotein V platelet [Source:HGNC Symbol;Acc:HGNC:4443]	3.4	2
753	*H2AFV*	94239	H2A histone family member V [Source:HGNC Symbol;Acc:HGNC:20664]	3.4	2
754	*PPP2R5E*	5529	protein phosphatase 2 regulatory subunit B'epsilon [Source:HGNC Symbol;Acc:HGNC:9313]	3.4	2
755	*ITGB5*	3693	integrin subunit beta 5 [Source:HGNC Symbol;Acc:HGNC:6160]	3.4	2
756	*RHOT2*	89941	ras homolog family member T2 [Source:HGNC Symbol;Acc:HGNC:21169]	3.4	2
757	*CLEC4E*	26253	C-type lectin domain family 4 member E [Source:HGNC Symbol;Acc:HGNC:14555]	3.4	2
758	*ASAP1*	50807	ArfGAP with SH3 domain, ankyrin repeat and PH domain 1 [Source:HGNC Symbol;Acc:HGNC:2720]	3.4	2
759	*CHRNE*	1145	cholinergic receptor nicotinic epsilon subunit [Source:HGNC Symbol;Acc:HGNC:1966]	3.4	2
760	*TNIK*	23043	TRAF2 and NCK interacting kinase [Source:HGNC Symbol;Acc:HGNC:30765]	3.4	2
761	*ABTB1*	80325	ankyrin repeat and BTB domain containing 1 [Source:HGNC Symbol;Acc:HGNC:18275]	3.4	2
762	*STRAP*	11171	serine/threonine kinase receptor associated protein [Source:HGNC Symbol;Acc:HGNC:30796]	3.4	2
763	*POM121C*	100101267	POM121 transmembrane nucleoporin C [Source:HGNC Symbol;Acc:HGNC:34005]	3.4	2
764	*TSC22D1*	8848	TSC22 domain family member 1 [Source:HGNC Symbol;Acc:HGNC:16826]	3.4	2
765	*CD53*	963	CD53 molecule [Source:HGNC Symbol;Acc:HGNC:1686]	3.4	2
766	*PILRA*	29992	paired immunoglobin like type 2 receptor alpha [Source:HGNC Symbol;Acc:HGNC:20396]	3.4	2
767	*MEIS1*	4211	Meis homeobox 1 [Source:HGNC Symbol;Acc:HGNC:7000]	3.4	2
768	*FOS*	2353	Fos proto-oncogene, AP-1 transcription factor subunit [Source:HGNC Symbol;Acc:HGNC:3796]	3.4	2
769	*MRPL36*	64979	mitochondrial ribosomal protein L36 [Source:HGNC Symbol;Acc:HGNC:14490]	3.4	2
770	*AGPAT3*	56894	1-acylglycerol-3-phosphate O-acyltransferase 3 [Source:HGNC Symbol;Acc:HGNC:326]	3.4	2
771	*MRPS27*	23107	mitochondrial ribosomal protein S27 [Source:HGNC Symbol;Acc:HGNC:14512]	3.4	2
772	*HLA-F*	3134	major histocompatibility complex, class I, F [Source:HGNC Symbol;Acc:HGNC:4963]	3.4	2
773	*DEPDC1*	55635	DEP domain containing 1 [Source:HGNC Symbol;Acc:HGNC:22949]	3.4	2
774	*ELP3*	55140	elongator acetyltransferase complex subunit 3 [Source:HGNC Symbol;Acc:HGNC:20696]	3.4	2
775	*NGFRAP1*	27018	Brain Expressed X-Linked 3	3.4	2
776	*TMEM164*	84187	transmembrane protein 164 [Source:HGNC Symbol;Acc:HGNC:26217]	3.4	2
777	*P2RY8*	286530	purinergic receptor P2Y8 [Source:HGNC Symbol;Acc:HGNC:15524]	3.4	2
778	*LCORL*	254251	ligand dependent nuclear receptor corepressor like [Source:HGNC Symbol;Acc:HGNC:30776]	3.4	2
779	*NCAPG*	64151	non-SMC condensin I complex subunit G [Source:HGNC Symbol;Acc:HGNC:24304]	3.4	2
780	*NDUFAF2*	91942	NADH:ubiquinone oxidoreductase complex assembly factor 2 [Source:HGNC Symbol;Acc:HGNC:28086]	3.4	2
781	*BRD8*	10902	bromodomain containing 8 [Source:HGNC Symbol;Acc:HGNC:19874]	3.4	2
782	*RAB14*	51552	RAB14, member RAS oncogene family [Source:HGNC Symbol;Acc:HGNC:16524]	3.4	2
783	*NUP210*	23225	nucleoporin 210 [Source:HGNC Symbol;Acc:HGNC:30052]	3.4	2
784	*MRPL15*	29088	mitochondrial ribosomal protein L15 [Source:HGNC Symbol;Acc:HGNC:14054]	3.4	2
785	*SMN1*	6606	survival of motor neuron 1, telomeric [Source:HGNC Symbol;Acc:HGNC:11117]	3.4	2
786	*CEP350*	9857	centrosomal protein 350 [Source:HGNC Symbol;Acc:HGNC:24238]	3.4	2
787	*MAD2L1*	4085	MAD2 mitotic arrest deficient-like 1 (yeast) [Source:HGNC Symbol;Acc:HGNC:6763]	3.4	2
788	*TSPAN5*	10098	tetraspanin 5 [Source:HGNC Symbol;Acc:HGNC:17753]	3.4	2
789	*FANCI*	55215	Fanconi anemia complementation group I [Source:HGNC Symbol;Acc:HGNC:25568]	3.4	2
790	*POLG*	5428	polymerase (DNA) gamma, catalytic subunit [Source:HGNC Symbol;Acc:HGNC:9179]	3.4	2
791	*CCBL2*	56267	Cysteine Conjugate-Beta Lyase 2	3.3	2
792	*ARHGAP27*	201176	Rho GTPase activating protein 27 [Source:HGNC Symbol;Acc:HGNC:31813]	3.3	2
793	*HCK*	3055	HCK proto-oncogene, Src family tyrosine kinase [Source:HGNC Symbol;Acc:HGNC:4840]	3.3	2
794	*KNSTRN*	90417	kinetochore-localized astrin/SPAG5 binding protein [Source:HGNC Symbol;Acc:HGNC:30767]	3.3	2
795	*TNFAIP3*	7128	TNF alpha induced protein 3 [Source:HGNC Symbol;Acc:HGNC:11896]	3.3	2
796	*KIF20B*	9585	kinesin family member 20B [Source:HGNC Symbol;Acc:HGNC:7212]	3.3	2
797	*PSTPIP2*	9050	proline-serine-threonine phosphatase interacting protein 2 [Source:HGNC Symbol;Acc:HGNC:9581]	3.3	2
798	*MMP16*	4325	matrix metallopeptidase 16 [Source:HGNC Symbol;Acc:HGNC:7162]	3.3	2
799	*PBX1*	5087	PBX homeobox 1 [Source:HGNC Symbol;Acc:HGNC:8632]	3.3	2
800	*DAP*	1611	death-associated protein [Source:HGNC Symbol;Acc:HGNC:2672]	3.3	2
801	*GSE1*	23199	Gse1 coiled-coil protein [Source:HGNC Symbol;Acc:HGNC:28979]	3.3	2
802	*RCC1*	1104	regulator of chromosome condensation 1 [Source:HGNC Symbol;Acc:HGNC:1913]	3.3	2
803	*RNF17*	56163	ring finger protein 17 [Source:HGNC Symbol;Acc:HGNC:10060]	3.3	2
804	*COX7A2L*	9167	cytochrome c oxidase subunit 7A2 like [Source:HGNC Symbol;Acc:HGNC:2289]	3.3	2
805	*HIST1H2BK*	85236	histone cluster 1, H2bk [Source:HGNC Symbol;Acc:HGNC:13954]	3.3	2
806	*CDC14B*	8555	cell division cycle 14B [Source:HGNC Symbol;Acc:HGNC:1719]	3.3	2
807	*HBE1*	3046	hemoglobin subunit epsilon 1 [Source:HGNC Symbol;Acc:HGNC:4830]	3.3	2
808	*PSTPIP1*	9051	proline-serine-threonine phosphatase interacting protein 1 [Source:HGNC Symbol;Acc:HGNC:9580]	3.3	2
809	*LSM10*	84967	LSM10, U7 small nuclear RNA associated [Source:HGNC Symbol;Acc:HGNC:17562]	3.3	2
810	*ANKRD33B*	651746	ankyrin repeat domain 33B [Source:HGNC Symbol;Acc:HGNC:35240]	3.3	2
811	*CCDC127*	133957	coiled-coil domain containing 127 [Source:HGNC Symbol;Acc:HGNC:30520]	3.3	2
812	*NONO*	4841	non-POU domain containing, octamer-binding [Source:HGNC Symbol;Acc:HGNC:7871]	3.3	2
813	*MPZL3*	196264	myelin protein zero like 3 [Source:HGNC Symbol;Acc:HGNC:27279]	3.3	2
814	*EAF2*	55840	ELL associated factor 2 [Source:HGNC Symbol;Acc:HGNC:23115]	3.3	2
815	*SLC44A2*	57153	solute carrier family 44 member 2 [Source:HGNC Symbol;Acc:HGNC:17292]	3.3	2
816	*SPARC*	6678	secreted protein acidic and cysteine rich [Source:HGNC Symbol;Acc:HGNC:11219]	3.3	2
817	*MAX*	4149	MYC associated factor X [Source:HGNC Symbol;Acc:HGNC:6913]	3.3	2
818	*C16orf72*	29035	chromosome 16 open reading frame 72 [Source:HGNC Symbol;Acc:HGNC:30103]	3.3	2
819	*ZNF367*	195828	zinc finger protein 367 [Source:HGNC Symbol;Acc:HGNC:18320]	3.3	2
820	*NUDT4*	11163	nudix hydrolase 4 [Source:HGNC Symbol;Acc:HGNC:8051]	3.3	2
821	*C1orf162*	128346	chromosome 1 open reading frame 162 [Source:HGNC Symbol;Acc:HGNC:28344]	3.3	2
822	*BRD4*	23476	bromodomain containing 4 [Source:HGNC Symbol;Acc:HGNC:13575]	3.3	2
823	*PLLP*	51090	plasmolipin [Source:HGNC Symbol;Acc:HGNC:18553]	3.3	2

824	*GLRX2*	51022	glutaredoxin 2 [Source:HGNC Symbol;Acc:HGNC:16065]	3.3	2
825	*HDAC1*	3065	histone deacetylase 1 [Source:HGNC Symbol;Acc:HGNC:4852]	3.3	2
826	*CARD8*	22900	caspase recruitment domain family member 8 [Source:HGNC Symbol;Acc:HGNC:17057]	3.3	2
827	*CTDSP2*	10106	CTD small phosphatase 2 [Source:HGNC Symbol;Acc:HGNC:17077]	3.3	2
828	*ABCF3*	55324	ATP binding cassette subfamily F member 3 [Source:HGNC Symbol;Acc:HGNC:72]	3.3	2
829	*CTDSPL*	10217	CTD small phosphatase like [Source:HGNC Symbol;Acc:HGNC:16890]	3.3	2
830	*SHMT2*	6472	serine hydroxymethyltransferase 2 [Source:HGNC Symbol;Acc:HGNC:10852]	3.3	2
831	*REV1*	51455	REV1, DNA directed polymerase [Source:HGNC Symbol;Acc:HGNC:14060]	3.3	2
832	*KIF5B*	3799	kinesin family member 5B [Source:HGNC Symbol;Acc:HGNC:6324]	3.3	2
833	*SELK*	58515	Selenoprotein K	3.3	2
834	*LBHD1*	79081	LBH domain containing 1 [Source:HGNC Symbol;Acc:HGNC:28351]	3.3	2
835	*NT5M*	56953	5',3'-nucleotidase, mitochondrial [Source:HGNC Symbol;Acc:HGNC:15769]	3.3	2
836	*MRPS25*	64432	mitochondrial ribosomal protein S25 [Source:HGNC Symbol;Acc:HGNC:14511]	3.3	2
837	*ABCG5*	64240	ATP binding cassette subfamily G member 5 [Source:HGNC Symbol;Acc:HGNC:13886]	3.3	2
838	*LSM5*	23658	LSM5 homolog, U6 small nuclear RNA and mRNA degradation associated [Source:HGNC Symbol;Acc:HGNC:17162]	3.3	2
839	*CD151*	977	CD151 molecule (Raph blood group) [Source:HGNC Symbol;Acc:HGNC:1630]	3.3	2
840	*ZMAT2*	153527	zinc finger matrin-type 2 [Source:HGNC Symbol;Acc:HGNC:26433]	3.3	2
841	*MRPL37*	51253	mitochondrial ribosomal protein L37 [Source:HGNC Symbol;Acc:HGNC:14034]	3.3	2
842	*LSM6*	11157	LSM6 homolog, U6 small nuclear RNA and mRNA degradation associated [Source:HGNC Symbol;Acc:HGNC:17017]	3.3	2
843	*CYB5D1*	124637	cytochrome b5 domain containing 1 [Source:HGNC Symbol;Acc:HGNC:26516]	3.3	2
844	*PLOD3*	8985	procollagen-lysine,2-oxoglutarate 5-dioxygenase 3 [Source:HGNC Symbol;Acc:HGNC:9083]	3.3	2
845	*DDX49*	54555	DEAD-box helicase 49 [Source:HGNC Symbol;Acc:HGNC:18684]	3.3	2
846	*DOCK2*	1794	dedicator of cytokinesis 2 [Source:HGNC Symbol;Acc:HGNC:2988]	3.3	2
847	*ATP2A3*	489	ATPase sarcoplasmic/endoplasmic reticulum Ca2+ transporting 3 [Source:HGNC Symbol;Acc:HGNC:813]	3.3	2
848	*PSMD5*	5711	proteasome 26S subunit, non-ATPase 5 [Source:HGNC Symbol;Acc:HGNC:9563]	3.3	2
849	*SAPCD2*	89958	suppressor APC domain containing 2 [Source:HGNC Symbol;Acc:HGNC:28055]	3.3	2
850	*PDE5A*	8654	phosphodiesterase 5A [Source:HGNC Symbol;Acc:HGNC:8784]	3.3	2
851	*ANKLE2*	23141	ankyrin repeat and LEM domain containing 2 [Source:HGNC Symbol;Acc:HGNC:29101]	3.3	2
852	*TUBA4A*	7277	tubulin alpha 4a [Source:HGNC Symbol;Acc:HGNC:12407]	3.3	2
853	*GGNBP2*	79893	gametogenetin binding protein 2 [Source:HGNC Symbol;Acc:HGNC:19357]	3.3	2
854	*SEC61G*	23480	Sec61 translocon gamma subunit [Source:HGNC Symbol;Acc:HGNC:18277]	3.3	2
855	*SLAIN2*	57606	SLAIN motif family member 2 [Source:HGNC Symbol;Acc:HGNC:29282]	3.3	2
856	*KCNAB2*	8514	potassium voltage-gated channel subfamily A regulatory beta subunit 2 [Source:HGNC Symbol;Acc:HGNC:6229]	3.3	2
857	*CAB39L*	81617	calcium binding protein 39 like [Source:HGNC Symbol;Acc:HGNC:20290]	3.3	2
858	*ZG16B*	124220	zymogen granule protein 16B [Source:HGNC Symbol;Acc:HGNC:30456]	3.2	2
859	*CSGALNACT1*	55790	chondroitin sulfate N-acetylgalactosaminyltransferase 1 [Source:HGNC Symbol;Acc:HGNC:24290]	3.2	2
860	*MRPL17*	63875	mitochondrial ribosomal protein L17 [Source:HGNC Symbol;Acc:HGNC:14053]	3.2	2
861	*SARS*	6301	seryl-tRNA synthetase [Source:HGNC Symbol;Acc:HGNC:10537]	3.2	2
862	*HSPA6*	3310	heat shock protein family A (Hsp70) member 6 [Source:HGNC Symbol;Acc:HGNC:5239]	3.2	2
863	*LRRK2*	120892	leucine rich repeat kinase 2 [Source:HGNC Symbol;Acc:HGNC:18618]	3.2	2
864	*CLPP*	8192	caseinolytic mitochondrial matrix peptidase proteolytic subunit [Source:HGNC Symbol;Acc:HGNC:2084]	3.2	2
865	*SPCS2*	9789	signal peptidase complex subunit 2 [Source:HGNC Symbol;Acc:HGNC:28962]	3.2	2
866	*PWP1*	11137	PWP1 homolog, endonuclein [Source:HGNC Symbol;Acc:HGNC:17015]	3.2	2
867	*COG8*	84342	component of oligomeric golgi complex 8 [Source:HGNC Symbol;Acc:HGNC:18623]	3.2	2
868	*CYTH1*	9267	cytohesin 1 [Source:HGNC Symbol;Acc:HGNC:9501]	3.2	2
869	*MPEG1*	219972	macrophage expressed 1 [Source:HGNC Symbol;Acc:HGNC:29619]	3.2	2
870	*ARRB1*	408	arrestin beta 1 [Source:HGNC Symbol;Acc:HGNC:711]	3.2	2
871	*DBI*	1622	diazepam binding inhibitor, acyl-CoA binding protein [Source:HGNC Symbol;Acc:HGNC:2690]	3.2	2
872	*CSNK1D*	1453	casein kinase 1 delta [Source:HGNC Symbol;Acc:HGNC:2452]	3.2	2
873	*KRI1*	65095	KRI1 homolog [Source:HGNC Symbol;Acc:HGNC:25769]	3.2	2
874	*SEPHS1*	22929	selenophosphate synthetase 1 [Source:HGNC Symbol;Acc:HGNC:19685]	3.2	2
875	*LAMC1*	3915	laminin subunit gamma 1 [Source:HGNC Symbol;Acc:HGNC:6492]	3.2	2
876	*CLEC1B*	51266	C-type lectin domain family 1 member B [Source:HGNC Symbol;Acc:HGNC:24356]	3.2	2
877	*ITGAX*	3687	integrin subunit alpha X [Source:HGNC Symbol;Acc:HGNC:6152]	3.2	2
878	*TUBA8*	51807	tubulin alpha 8 [Source:HGNC Symbol;Acc:HGNC:12410]	3.2	2
879	*DDC*	1644	dopa decarboxylase [Source:HGNC Symbol;Acc:HGNC:2719]	3.2	2
880	*RHOBTB1*	9886	Rho related BTB domain containing 1 [Source:HGNC Symbol;Acc:HGNC:18738]	3.2	2
881	*IDH2*	3418	isocitrate dehydrogenase (NADP(+)) 2, mitochondrial [Source:HGNC Symbol;Acc:HGNC:5383]	3.2	2
882	*SSX2IP*	117178	SSX family member 2 interacting protein [Source:HGNC Symbol;Acc:HGNC:16509]	3.2	2
883	*PYCR1*	5831	pyrroline-5-carboxylate reductase 1 [Source:HGNC Symbol;Acc:HGNC:9721]	3.2	2
884	*AGPAT1*	10554	1-acylglycerol-3-phosphate O-acyltransferase 1 [Source:HGNC Symbol;Acc:HGNC:324]	3.2	2
885	*CRBN*	51185	cereblon [Source:HGNC Symbol;Acc:HGNC:30185]	3.2	2
886	*FAM209B*	388799	family with sequence similarity 209 member B [Source:HGNC Symbol;Acc:HGNC:16101]	3.2	2
887	*MBOAT1*	154141	membrane bound O-acyltransferase domain containing 1 [Source:HGNC Symbol;Acc:HGNC:21579]	3.2	2
888	*CDCA8*	55143	cell division cycle associated 8 [Source:HGNC Symbol;Acc:HGNC:14629]	3.2	2
889	*NR2F2*	7026	nuclear receptor subfamily 2 group F member 2 [Source:HGNC Symbol;Acc:HGNC:7976]	3.2	2
890	*ASPM*	259266	abnormal spindle microtubule assembly [Source:HGNC Symbol;Acc:HGNC:19048]	3.2	2
891	*C12orf76*	400073	chromosome 12 open reading frame 76 [Source:HGNC Symbol;Acc:HGNC:33790]	3.2	2
892	*ARF3*	377	ADP ribosylation factor 3 [Source:HGNC Symbol;Acc:HGNC:654]	3.2	2
893	*BUB3*	9184	BUB3, mitotic checkpoint protein [Source:HGNC Symbol;Acc:HGNC:1151]	3.2	2
894	*C14orf1*	11161	chromosome 14 open reading frame 1 [Source:HGNC Symbol;Acc:HGNC:1187]	3.2	2
895	*MORN2*	729967	MORN repeat containing 2 [Source:HGNC Symbol;Acc:HGNC:30166]	3.2	2
896	*CLMN*	79789	calmin (calponin-like, transmembrane) [Source:HGNC Symbol;Acc:HGNC:19972]	3.2	2
897	*PDIA5*	10954	protein disulfide isomerase family A member 5 [Source:HGNC Symbol;Acc:HGNC:24811]	3.2	2

898	*AKT1*	207	AKT serine/threonine kinase 1 [Source:HGNC Symbol;Acc:HGNC:391]	3.2	2
899	*PLEK*	5341	pleckstrin [Source:HGNC Symbol;Acc:HGNC:9070]	3.2	2
900	*NUCKS1*	64710	nuclear casein kinase and cyclin dependent kinase substrate 1 [Source:HGNC Symbol;Acc:HGNC:29923]	3.2	2
901	*LRBA*	987	LPS responsive beige-like anchor protein [Source:HGNC Symbol;Acc:HGNC:1742]	3.2	2
902	*AKAP9*	10142	A-kinase anchoring protein 9 [Source:HGNC Symbol;Acc:HGNC:379]	3.2	2
903	*EZR*	7430	ezrin [Source:HGNC Symbol;Acc:HGNC:12691]	3.2	2
904	*APBB1IP*	54518	amyloid beta precursor protein binding family B member 1 interacting protein [Source:HGNC Symbol;Acc:HGNC:17379]	3.2	2
905	*FAXDC2*	10826	fatty acid hydroxylase domain containing 2 [Source:HGNC Symbol;Acc:HGNC:1334]	3.2	2
906	*ARF4*	378	ADP ribosylation factor 4 [Source:HGNC Symbol;Acc:HGNC:655]	3.2	2
907	*WIPF1*	7456	WAS/WASL interacting protein family member 1 [Source:HGNC Symbol;Acc:HGNC:12736]	3.2	2
908	*SCO2*	9997	SCO2 cytochrome c oxidase assembly protein [Source:HGNC Symbol;Acc:HGNC:10604]	3.2	2
909	*GUCY1A3*	2982	guanylate cyclase 1, soluble, alpha 3 [Source:HGNC Symbol;Acc:HGNC:4685]	3.2	2
910	*SMG7*	9887	SMG7, nonsense mediated mRNA decay factor [Source:HGNC Symbol;Acc:HGNC:16792]	3.2	2
911	*LARP7*	51574	La ribonucleoprotein domain family member 7 [Source:HGNC Symbol;Acc:HGNC:24912]	3.2	2
912	*ZNF426*	79088	zinc finger protein 426 [Source:HGNC Symbol;Acc:HGNC:20725]	3.2	2
913	*SMPD4*	55627	sphingomyelin phosphodiesterase 4 [Source:HGNC Symbol;Acc:HGNC:32949]	3.2	2
914	*ALOX5*	240	arachidonate 5-lipoxygenase [Source:HGNC Symbol;Acc:HGNC:435]	3.2	2
915	*IRAK3*	11213	interleukin 1 receptor associated kinase 3 [Source:HGNC Symbol;Acc:HGNC:17020]	3.2	2
916	*BCLAF1*	9774	BCL2 associated transcription factor 1 [Source:HGNC Symbol;Acc:HGNC:16863]	3.2	2
917	*DENND2C*	163259	DENN domain containing 2C [Source:HGNC Symbol;Acc:HGNC:24748]	3.2	2
918	*ADRBK2*	157	adrenoceptor beta 2	3.2	2
919	*PCF11*	51585	PCF11 cleavage and polyadenylation factor subunit [Source:HGNC Symbol;Acc:HGNC:30097]	3.2	2
920	*CYBB*	1536	cytochrome b-245 beta chain [Source:HGNC Symbol;Acc:HGNC:2578]	3.2	2
921	*SCN1B*	6324	sodium voltage-gated channel beta subunit 1 [Source:HGNC Symbol;Acc:HGNC:10586]	3.2	2
922	*LCTL*	197021	lactase like [Source:HGNC Symbol;Acc:HGNC:15583]	3.2	2
923	*ZWILCH*	55055	zwilch kinetochore protein [Source:HGNC Symbol;Acc:HGNC:25468]	3.2	2
924	*ASNA1*	439	arsA arsenite transporter, ATP-binding, homolog 1 (bacterial) [Source:HGNC Symbol;Acc:HGNC:752]	3.2	2
925	*DENND1C*	79958	DENN domain containing 1C [Source:HGNC Symbol;Acc:HGNC:26225]	3.2	2
926	*NAA10*	8260	N(alpha)-acetyltransferase 10, NatA catalytic subunit [Source:HGNC Symbol;Acc:HGNC:18704]	3.2	2
927	*AZI2*	64343	5-azacytidine induced 2 [Source:HGNC Symbol;Acc:HGNC:24002]	3.2	2
928	*PCBP2*	5094	poly(rC) binding protein 2 [Source:HGNC Symbol;Acc:HGNC:8648]	3.2	2
929	*TUSC2*	11334	tumor suppressor candidate 2 [Source:HGNC Symbol;Acc:HGNC:17034]	3.2	2
930	*RHOF*	54509	ras homolog family member F (in filopodia) [Source:HGNC Symbol;Acc:HGNC:15703]	3.2	2
931	*TMC8*	147138	transmembrane channel like 8 [Source:HGNC Symbol;Acc:HGNC:20474]	3.2	2
932	*PDLIM1*	9124	PDZ and LIM domain 1 [Source:HGNC Symbol;Acc:HGNC:2067]	3.2	2
933	*SEP15*	9403	15 KDa Selenoprotein	3.2	2
934	*NAGA*	4668	alpha-N-acetylgalactosaminidase [Source:HGNC Symbol;Acc:HGNC:7631]	3.2	2
935	*WNK2*	65268	WNK lysine deficient protein kinase 2 [Source:HGNC Symbol;Acc:HGNC:14542]	3.2	2
936	*GCOM1*	145781	GRINL1A complex locus 1 [Source:HGNC Symbol;Acc:HGNC:26424]	3.2	2
937	*SYNM*	23336	synemin [Source:HGNC Symbol;Acc:HGNC:24466]	3.2	2
938	*TCEA1*	6917	transcription elongation factor A1 [Source:HGNC Symbol;Acc:HGNC:11612]	3.2	2
939	*ZYX*	7791	zyxin [Source:HGNC Symbol;Acc:HGNC:13200]	3.2	2
940	*UBE2S*	27338	ubiquitin conjugating enzyme E2 S [Source:HGNC Symbol;Acc:HGNC:17895]	3.2	2
941	*DHCR24*	1718	24-dehydrocholesterol reductase [Source:HGNC Symbol;Acc:HGNC:2859]	3.1	2
942	*HRAS*	3265	Harvey rat sarcoma viral oncogene homolog [Source:HGNC Symbol;Acc:HGNC:5173]	3.1	2
943	*PSMD13*	5719	proteasome 26S subunit, non-ATPase 13 [Source:HGNC Symbol;Acc:HGNC:9558]	3.1	2
944	*NOL6*	65083	nucleolar protein 6 [Source:HGNC Symbol;Acc:HGNC:19910]	3.1	2
945	*TGFB1I1*	7041	transforming growth factor beta 1 induced transcript 1 [Source:HGNC Symbol;Acc:HGNC:11767]	3.1	2
946	*MMRN1*	22915	multimerin 1 [Source:HGNC Symbol;Acc:HGNC:7178]	3.1	2
947	*PPIG*	9360	peptidylprolyl isomerase G [Source:HGNC Symbol;Acc:HGNC:14650]	3.1	2
948	*MORF4L2*	9643	mortality factor 4 like 2 [Source:HGNC Symbol;Acc:HGNC:16849]	3.1	2
949	*RBP7*	116362	retinol binding protein 7 [Source:HGNC Symbol;Acc:HGNC:30316]	3.1	2
950	*SELPLG*	6404	selectin P ligand [Source:HGNC Symbol;Acc:HGNC:10722]	3.1	2
951	*WASL*	8976	Wiskott-Aldrich syndrome like [Source:HGNC Symbol;Acc:HGNC:12735]	3.1	2
952	*TMEM150C*	441027	transmembrane protein 150C [Source:HGNC Symbol;Acc:HGNC:37263]	3.1	2
953	*FOLR3*	2352	folate receptor 3 (gamma) [Source:HGNC Symbol;Acc:HGNC:3795]	3.1	2
954	*SDR39U1*	56948	short chain dehydrogenase/reductase family 39U member 1 [Source:HGNC Symbol;Acc:HGNC:20275]	3.1	2
955	*LRRFIP1*	9208	leucine rich repeat (in FLII) interacting protein 1 [Source:HGNC Symbol;Acc:HGNC:6702]	3.1	2
956	*DIAPH1*	1729	diaphanous related formin 1 [Source:HGNC Symbol;Acc:HGNC:2876]	3.1	2
957	*ELOF1*	84337	elongation factor 1 homolog [Source:HGNC Symbol;Acc:HGNC:28691]	3.1	2
958	*SETD8*	387893	SET domain containing lysine methyltransferase 8	3.1	2
959	*GIMAP1*	170575	GTPase, IMAP family member 1 [Source:HGNC Symbol;Acc:HGNC:23237]	3.1	2
960	*GRPEL1*	80273	GrpE like 1, mitochondrial [Source:HGNC Symbol;Acc:HGNC:19696]	3.1	2
961	*MMP25*	64386	matrix metallopeptidase 25 [Source:HGNC Symbol;Acc:HGNC:14246]	3.1	2
962	*MXD1*	4084	MAX dimerization protein 1 [Source:HGNC Symbol;Acc:HGNC:6761]	3.1	2
963	*EIF4EBP3*	8637	eukaryotic translation initiation factor 4E binding protein 3 [Source:HGNC Symbol;Acc:HGNC:3290]	3.1	2
964	*DCAF12*	25853	DDB1 and CUL4 associated factor 12 [Source:HGNC Symbol;Acc:HGNC:19911]	3.1	2
965	*RYBP*	23429	RING1 and YY1 binding protein [Source:HGNC Symbol;Acc:HGNC:10480]	3.1	2
966	*NDUFB10*	4716	NADH:ubiquinone oxidoreductase subunit B10 [Source:HGNC Symbol;Acc:HGNC:7696]	3.1	2
967	*FUT8*	2530	fucosyltransferase 8 [Source:HGNC Symbol;Acc:HGNC:4019]	3.1	2
968	*OSER1*	51526	oxidative stress responsive serine rich 1 [Source:HGNC Symbol;Acc:HGNC:16105]	3.1	2
969	*BZW1*	9689	basic leucine zipper and W2 domains 1 [Source:HGNC Symbol;Acc:HGNC:18380]	3.1	2
970	*CTPS1*	1503	CTP synthase 1 [Source:HGNC Symbol;Acc:HGNC:2519]	3.1	2
971	*TRIM28*	10155	tripartite motif containing 28 [Source:HGNC Symbol;Acc:HGNC:16384]	3.1	2
972	*CHKA*	1119	choline kinase alpha [Source:HGNC Symbol;Acc:HGNC:1937]	3.1	2

973	*F5*	2153	coagulation factor V [Source:HGNC Symbol;Acc:HGNC:3542]	3.1	2
974	*GRAP2*	9402	GRB2-related adaptor protein 2 [Source:HGNC Symbol;Acc:HGNC:4563]	3.1	2
975	*CCDC167*	154467	coiled-coil domain containing 167 [Source:HGNC Symbol;Acc:HGNC:21239]	3.1	2
976	*CELF2*	10659	CUGBP, Elav-like family member 2 [Source:HGNC Symbol;Acc:HGNC:2550]	3.1	2
977	*RNASE4*	6038	ribonuclease A family member 4 [Source:HGNC Symbol;Acc:HGNC:10047]	3.1	2
978	*VARS*	7407	valyl-tRNA synthetase [Source:HGNC Symbol;Acc:HGNC:12651]	3.1	2
979	*HIST1H2BD*	3017	histone cluster 1, H2bd [Source:HGNC Symbol;Acc:HGNC:4747]	3.1	2
980	*ZNF410*	57862	zinc finger protein 410 [Source:HGNC Symbol;Acc:HGNC:20144]	3.1	2
981	*SF3B2*	10992	splicing factor 3b subunit 2 [Source:HGNC Symbol;Acc:HGNC:10769]	3.1	2
982	*EPB42*	2038	erythrocyte membrane protein band 4.2 [Source:HGNC Symbol;Acc:HGNC:3381]	3.1	2
983	*ATP6V1B2*	526	ATPase H+ transporting V1 subunit B2 [Source:HGNC Symbol;Acc:HGNC:854]	3.1	2
984	*RASSF2*	9770	Ras association domain family member 2 [Source:HGNC Symbol;Acc:HGNC:9883]	3.1	2
985	*PRKCD*	5580	protein kinase C delta [Source:HGNC Symbol;Acc:HGNC:9399]	3.1	2
986	*MED24*	9862	mediator complex subunit 24 [Source:HGNC Symbol;Acc:HGNC:22963]	3.1	2
987	*LIMD2*	80774	LIM domain containing 2 [Source:HGNC Symbol;Acc:HGNC:28142]	3.1	2
988	*DCLRE1A*	9937	DNA cross-link repair 1A [Source:HGNC Symbol;Acc:HGNC:17660]	3.1	2
989	*USP32*	84669	ubiquitin specific peptidase 32 [Source:HGNC Symbol;Acc:HGNC:19143]	3.1	2
990	*RNF125*	54941	ring finger protein 125 [Source:HGNC Symbol;Acc:HGNC:21150]	3.1	2
991	*ARL14EP*	120534	ADP ribosylation factor like GTPase 14 effector protein [Source:HGNC Symbol;Acc:HGNC:26798]	3.1	2
992	*VAV1*	7409	vav guanine nucleotide exchange factor 1 [Source:HGNC Symbol;Acc:HGNC:12657]	3.1	2
993	*CCR2*	729230	C-C motif chemokine receptor 2 [Source:HGNC Symbol;Acc:HGNC:1603]	3.1	2
994	*PSMG2*	56984	proteasome assembly chaperone 2 [Source:HGNC Symbol;Acc:HGNC:24929]	3.1	2
995	*ITGAM*	3684	integrin subunit alpha M [Source:HGNC Symbol;Acc:HGNC:6149]	3.1	2
996	*SLC43A3*	29015	solute carrier family 43 member 3 [Source:HGNC Symbol;Acc:HGNC:17466]	3.1	2
997	*TMEM8A*	58986	transmembrane protein 8A [Source:HGNC Symbol;Acc:HGNC:17205]	3.1	2
998	*ARL4A*	10124	ADP ribosylation factor like GTPase 4A [Source:HGNC Symbol;Acc:HGNC:695]	3.1	2
999	*RBM28*	55131	RNA binding motif protein 28 [Source:HGNC Symbol;Acc:HGNC:21863]	3.1	2
1000	*ORAI2*	80228	ORAI calcium release-activated calcium modulator 2 [Source:HGNC Symbol;Acc:HGNC:21667]	3.1	2
1001	*RARS2*	57038	arginyl-tRNA synthetase 2, mitochondrial [Source:HGNC Symbol;Acc:HGNC:21406]	3.1	2
1002	*CXorf56*	63932	chromosome X open reading frame 56 [Source:HGNC Symbol;Acc:HGNC:26239]	3.1	2
1003	*BRI3*	25798	brain protein I3 [Source:HGNC Symbol;Acc:HGNC:1109]	3.1	2
1004	*THAP3*	90326	THAP domain containing 3 [Source:HGNC Symbol;Acc:HGNC:20855]	3.1	2
1005	*TACC3*	10460	transforming acidic coiled-coil containing protein 3 [Source:HGNC Symbol;Acc:HGNC:11524]	3.1	2
1006	*LSMEM1*	286006	leucine rich single-pass membrane protein 1 [Source:HGNC Symbol;Acc:HGNC:22036]	3.1	2
1007	*JAM3*	83700	junctional adhesion molecule 3 [Source:HGNC Symbol;Acc:HGNC:15532]	3.1	2
1008	*HCST*	10870	hematopoietic cell signal transducer [Source:HGNC Symbol;Acc:HGNC:16977]	3.1	2
1009	*SRSF9*	8683	serine and arginine rich splicing factor 9 [Source:HGNC Symbol;Acc:HGNC:10791]	3.1	2
1010	*PTK2B*	2185	protein tyrosine kinase 2 beta [Source:HGNC Symbol;Acc:HGNC:9612]	3.1	2
1011	*CALU*	813	calumenin [Source:HGNC Symbol;Acc:HGNC:1458]	3.1	2
1012	*ACTN1*	87	actinin alpha 1 [Source:HGNC Symbol;Acc:HGNC:163]	3.1	2
1013	*RSRP1*	57035	arginine and serine rich protein 1 [Source:HGNC Symbol;Acc:HGNC:25234]	3.1	2
1014	*ATRX*	546	ATRX, chromatin remodeler [Source:HGNC Symbol;Acc:HGNC:886]	3.1	2
1015	*PRMT5*	10419	protein arginine methyltransferase 5 [Source:HGNC Symbol;Acc:HGNC:10894]	3.1	2
1016	*VWA5B1*	127731	von Willebrand factor A domain containing 5B1 [Source:HGNC Symbol;Acc:HGNC:26538]	3.1	2
1017	*ATP6V0E1*	8992	ATPase H+ transporting V0 subunit e1 [Source:HGNC Symbol;Acc:HGNC:863]	3.1	2
1018	*PRKCDBP*	112464	protein kinase C delta binding protein [Source:HGNC Symbol;Acc:HGNC:9400]	3.1	2
1019	*CLEC7A*	64581	C-type lectin domain family 7 member A [Source:HGNC Symbol;Acc:HGNC:14558]	3.1	2
1020	*POLR1D*	51082	polymerase (RNA) I subunit D [Source:HGNC Symbol;Acc:HGNC:20422]	3.1	2
1021	*COTL1*	23406	coactosin like F-actin binding protein 1 [Source:HGNC Symbol;Acc:HGNC:18304]	3.1	2
1022	*USP8*	9101	ubiquitin specific peptidase 8 [Source:HGNC Symbol;Acc:HGNC:12631]	3.1	2
1023	*CBX6*	23466	chromobox 6 [Source:HGNC Symbol;Acc:HGNC:1556]	3.1	2
1024	*TBC1D10C*	374403	TBC1 domain family member 10C [Source:HGNC Symbol;Acc:HGNC:24702]	3.1	2
1025	*LY6G6F*	259215	lymphocyte antigen 6 complex, locus G6F [Source:HGNC Symbol;Acc:HGNC:13933]	3.1	2
1026	*CXCL5*	6374	C-X-C motif chemokine ligand 5 [Source:HGNC Symbol;Acc:HGNC:10642]	3.1	2
1027	*C6orf106*	64771	chromosome 6 open reading frame 106 [Source:HGNC Symbol;Acc:HGNC:21215]	3.0	2
1028	*POLR2A*	5430	polymerase (RNA) II subunit A [Source:HGNC Symbol;Acc:HGNC:9187]	3.0	2
1029	*SPPL3*	121665	signal peptide peptidase like 3 [Source:HGNC Symbol;Acc:HGNC:30424]	3.0	2
1030	*F13A1*	2162	coagulation factor XIII A chain [Source:HGNC Symbol;Acc:HGNC:3531]	3.0	2
1031	*CCDC14*	64770	coiled-coil domain containing 14 [Source:HGNC Symbol;Acc:HGNC:25766]	3.0	2
1032	*TMX2*	51075	thioredoxin related transmembrane protein 2 [Source:HGNC Symbol;Acc:HGNC:30739]	3.0	2
1033	*ATP5F1*	515	ATP synthase, H+ transporting, mitochondrial Fo complex subunit B1 [Source:HGNC Symbol;Acc:HGNC:840]	3.0	2
1034	*RINT1*	60561	RAD50 interactor 1 [Source:HGNC Symbol;Acc:HGNC:21876]	3.0	2
1035	*TUBA1C*	84790	tubulin alpha 1c [Source:HGNC Symbol;Acc:HGNC:20768]	3.0	2
1036	*CIRBP*	1153	cold inducible RNA binding protein [Source:HGNC Symbol;Acc:HGNC:1982]	3.0	2
1037	*PELI1*	57162	pellino E3 ubiquitin protein ligase 1 [Source:HGNC Symbol;Acc:HGNC:8827]	3.0	2
1038	*PCYOX1L*	78991	prenylcysteine oxidase 1 like [Source:HGNC Symbol;Acc:HGNC:28477]	3.0	2
1039	*PLIN5*	440503	perilipin 5 [Source:HGNC Symbol;Acc:HGNC:33196]	3.0	2
1040	*PIM2*	11040	Pim-2 proto-oncogene, serine/threonine kinase [Source:HGNC Symbol;Acc:HGNC:8987]	3.0	2
1041	*PPP1R15B*	84919	protein phosphatase 1 regulatory subunit 15B [Source:HGNC Symbol;Acc:HGNC:14951]	3.0	2
1042	*RNASEH2A*	10535	ribonuclease H2 subunit A [Source:HGNC Symbol;Acc:HGNC:18518]	3.0	2
1043	*APOBR*	55911	apolipoprotein B receptor [Source:HGNC Symbol;Acc:HGNC:24087]	3.0	2
1044	*PTPN18*	26469	protein tyrosine phosphatase, non-receptor type 18 [Source:HGNC Symbol;Acc:HGNC:9649]	3.0	2
1045	*FES*	2242	FES proto-oncogene, tyrosine kinase [Source:HGNC Symbol;Acc:HGNC:3657]	3.0	2
1046	*SYAP1*	94056	synapse associated protein 1 [Source:HGNC Symbol;Acc:HGNC:16273]	3.0	2
1047	*FAF2*	23197	Fas associated factor family member 2 [Source:HGNC Symbol;Acc:HGNC:24666]	3.0	2
1048	*UBALD2*	283991	UBA like domain containing 2 [Source:HGNC Symbol;Acc:HGNC:28438]	3.0	2
1049	*MINK1*	50488	misshapen like kinase 1 [Source:HGNC Symbol;Acc:HGNC:17565]	3.0	2
1050	*GDF11*	10220	growth differentiation factor 11 [Source:HGNC Symbol;Acc:HGNC:4216]	3.0	2

1051	*PLA2G12A*	81579	phospholipase A2 group XIIA [Source:HGNC Symbol;Acc:HGNC:18554]	3.0	2
1052	*NADSYN1*	55191	NAD synthetase 1 [Source:HGNC Symbol;Acc:HGNC:29832]	3.0	2
1053	*WSB1*	26118	WD repeat and SOCS box containing 1 [Source:HGNC Symbol;Acc:HGNC:19221]	3.0	2
1054	*FYB*	2533	FYN binding protein [Source:HGNC Symbol;Acc:HGNC:4036]	3.0	2
1055	*ADAM19*	8728	ADAM metallopeptidase domain 19 [Source:HGNC Symbol;Acc:HGNC:197]	3.0	2
1056	*FASN*	2194	fatty acid synthase [Source:HGNC Symbol;Acc:HGNC:3594]	3.0	2
1057	*ZEB2*	9839	zinc finger E-box binding homeobox 2 [Source:HGNC Symbol;Acc:HGNC:14881]	3.0	2
1058	*MAPK1*	5594	mitogen-activated protein kinase 1 [Source:HGNC Symbol;Acc:HGNC:6871]	3.0	2
1059	*SIK2*	23235	salt inducible kinase 2 [Source:HGNC Symbol;Acc:HGNC:21680]	3.0	2
1060	*DDX39A*	10212	DEAD-box helicase 39A [Source:HGNC Symbol;Acc:HGNC:17821]	3.0	2
1061	*MIDN*	90007	midnolin [Source:HGNC Symbol;Acc:HGNC:16298]	3.0	2
1062	*GOLPH3*	64083	golgi phosphoprotein 3 [Source:HGNC Symbol;Acc:HGNC:15452]	3.0	2
1063	*SLC25A33*	84275	solute carrier family 25 member 33 [Source:HGNC Symbol;Acc:HGNC:29681]	3.0	2
1064	*OLIG1*	116448	oligodendrocyte transcription factor 1 [Source:HGNC Symbol;Acc:HGNC:16983]	3.0	2
1065	*PCMT1*	5110	protein-L-isoaspartate (D-aspartate) O-methyltransferase [Source:HGNC Symbol;Acc:HGNC:8728]	3.0	2
1066	*DPM3*	54344	dolichyl-phosphate mannosyltransferase subunit 3 [Source:HGNC Symbol;Acc:HGNC:3007]	3.0	2
1067	*HLX*	3142	H2.0 like homeobox [Source:HGNC Symbol;Acc:HGNC:4978]	3.0	2
1068	*CDC42BPB*	9578	CDC42 binding protein kinase beta [Source:HGNC Symbol;Acc:HGNC:1738]	3.0	2
1069	*ADAM8*	101	ADAM metallopeptidase domain 8 [Source:HGNC Symbol;Acc:HGNC:215]	3.0	2
1070	*DCTN6*	10671	dynactin subunit 6 [Source:HGNC Symbol;Acc:HGNC:16964]	3.0	2
1071	*HSD17B11*	51170	hydroxysteroid 17-beta dehydrogenase 11 [Source:HGNC Symbol;Acc:HGNC:22960]	3.0	2
1072	*FRAT2*	23401	frequently rearranged in advanced T-cell lymphomas 2 [Source:HGNC Symbol;Acc:HGNC:16048]	3.0	2
1073	*MINA*	84864	MYC induced nuclear antigen [Source:HGNC Symbol;Acc:HGNC:19441]	3.0	2
1074	*OASL*	8638	2'-5'-oligoadenylate synthetase like [Source:HGNC Symbol;Acc:HGNC:8090]	3.0	2
1075	*SRSF1*	6426	serine and arginine rich splicing factor 1 [Source:HGNC Symbol;Acc:HGNC:10780]	3.0	2
1076	*CDC42*	998	cell division cycle 42 [Source:HGNC Symbol;Acc:HGNC:1736]	3.0	2
1077	*PARVG*	64098	parvin gamma [Source:HGNC Symbol;Acc:HGNC:14654]	3.0	2
1078	*DEF8*	54849	differentially expressed in FDCP 8 homolog (mouse) [Source:HGNC Symbol;Acc:HGNC:25969]	3.0	2
1079	*KLF6*	1316	Kruppel like factor 6 [Source:HGNC Symbol;Acc:HGNC:2235]	3.0	2
1080	*TATDN1*	83940	TatD DNase domain containing 1 [Source:HGNC Symbol;Acc:HGNC:24220]	3.0	2
1081	*KIFC1*	3833	kinesin family member C1 [Source:HGNC Symbol;Acc:HGNC:6389]	3.0	2
1082	*CDS1*	1040	CDP-diacylglycerol synthase 1 [Source:HGNC Symbol;Acc:HGNC:1800]	3.0	2
1083	*TMEM179B*	374395	transmembrane protein 179B [Source:HGNC Symbol;Acc:HGNC:33744]	3.0	2
1084	*EFCAB14*	9813	EF-hand calcium binding domain 14 [Source:HGNC Symbol;Acc:HGNC:29051]	3.0	2
1085	*PVRL2*	5819	Nectin Cell Adhesion Molecule 2	3.0	2
1086	*JARID2*	3720	jumonji and AT-rich interaction domain containing 2 [Source:HGNC Symbol;Acc:HGNC:6196]	3.0	2
1087	*BTK*	695	Bruton tyrosine kinase [Source:HGNC Symbol;Acc:HGNC:1133]	3.0	2
1088	*SH3KBP1*	30011	SH3 domain containing kinase binding protein 1 [Source:HGNC Symbol;Acc:HGNC:13867]	3.0	2
1089	*RGS10*	6001	regulator of G-protein signaling 10 [Source:HGNC Symbol;Acc:HGNC:9992]	3.0	2
1090	*PNKD*	25953	paroxysmal nonkinesigenic dyskinesia [Source:HGNC Symbol;Acc:HGNC:9153]	3.0	2
1091	*BCL2A1*	597	BCL2 related protein A1 [Source:HGNC Symbol;Acc:HGNC:991]	3.0	2
1092	*MGME1*	92667	mitochondrial genome maintenance exonuclease 1 [Source:HGNC Symbol;Acc:HGNC:16205]	3.0	2
1093	*PRDM4*	11108	PR domain 4 [Source:HGNC Symbol;Acc:HGNC:9348]	3.0	2
1094	*PHF19*	26147	PHD finger protein 19 [Source:HGNC Symbol;Acc:HGNC:24566]	3.0	2
1095	*POLR1C*	9533	polymerase (RNA) I subunit C [Source:HGNC Symbol;Acc:HGNC:20194]	3.0	2
1096	*DDX11*	1663	DEAD/H-box helicase 11 [Source:HGNC Symbol;Acc:HGNC:2736]	3.0	2
1097	*FAM110B*	90362	family with sequence similarity 110 member B [Source:HGNC Symbol;Acc:HGNC:28587]	3.0	2
1098	*HIF1AN*	55662	hypoxia inducible factor 1 alpha subunit inhibitor [Source:HGNC Symbol;Acc:HGNC:17113]	3.0	2
1099	*PLEC*	5339	plectin [Source:HGNC Symbol;Acc:HGNC:9069]	3.0	2
1100	*STOM*	2040	stomatin [Source:HGNC Symbol;Acc:HGNC:3383]	3.0	2
1101	*PITHD1*	57095	PITH domain containing 1 [Source:HGNC Symbol;Acc:HGNC:25022]	3.0	2
1102	*WEE1*	7465	WEE1 G2 checkpoint kinase [Source:HGNC Symbol;Acc:HGNC:12761]	3.0	2
1103	*FAM177A1*	283635	family with sequence similarity 177 member A1 [Source:HGNC Symbol;Acc:HGNC:19829]	3.0	2
1104	*SLA*	6503	Src-like-adaptor [Source:HGNC Symbol;Acc:HGNC:10902]	3.0	2
1105	*TXNDC11*	51061	thioredoxin domain containing 11 [Source:HGNC Symbol;Acc:HGNC:28030]	3.0	2
1106	*RNASEH1*	246243	ribonuclease H1 [Source:HGNC Symbol;Acc:HGNC:18466]	3.0	2
1107	*TECPR2*	9895	tectonin beta-propeller repeat containing 2 [Source:HGNC Symbol;Acc:HGNC:19957]	3.0	2
1108	*SWT1*	54823	SWT1, RNA endoribonuclease homolog [Source:HGNC Symbol;Acc:HGNC:16785]	3.0	2
1109	*C7orf25*	79020	chromosome 7 open reading frame 25 [Source:HGNC Symbol;Acc:HGNC:21703]	3.0	2
1110	*CAPN3*	825	calpain 3 [Source:HGNC Symbol;Acc:HGNC:1480]	3.0	2
1111	*STX8*	9482	syntaxin 8 [Source:HGNC Symbol;Acc:HGNC:11443]	3.0	2
1112	*HARS*	3035	histidyl-tRNA synthetase [Source:HGNC Symbol;Acc:HGNC:4816]	3.0	2
1113	*MED16*	10025	mediator complex subunit 16 [Source:HGNC Symbol;Acc:HGNC:17556]	3.0	2
1114	*STARD3NL*	83930	STARD3 N-terminal like [Source:HGNC Symbol;Acc:HGNC:19169]	3.0	2
1115	*MBD4*	8930	methyl-CpG binding domain 4, DNA glycosylase [Source:HGNC Symbol;Acc:HGNC:6919]	3.0	2
1116	*SPOCD1*	90853	SPOC domain containing 1 [Source:HGNC Symbol;Acc:HGNC:26338]	3.0	2
1117	*MED27*	9442	mediator complex subunit 27 [Source:HGNC Symbol;Acc:HGNC:2377]	3.0	2
1118	*IL27RA*	9466	interleukin 27 receptor subunit alpha [Source:HGNC Symbol;Acc:HGNC:17290]	3.0	2
1119	*FOXH1*	8928	forkhead box H1 [Source:HGNC Symbol;Acc:HGNC:3814]	3.0	2
1120	*C1orf198*	84886	chromosome 1 open reading frame 198 [Source:HGNC Symbol;Acc:HGNC:25900]	3.0	2
1121	*CBX4*	8535	chromobox 4 [Source:HGNC Symbol;Acc:HGNC:1554]	3.0	2
1122	*KRT10*	3858	keratin 10 [Source:HGNC Symbol;Acc:HGNC:6413]	3.0	2
1123	*BSPRY*	54836	B-box and SPRY domain containing [Source:HGNC Symbol;Acc:HGNC:18232]	3.0	2
1124	*GAB3*	139716	GRB2 associated binding protein 3 [Source:HGNC Symbol;Acc:HGNC:17515]	3.0	2
1125	*SESN2*	83667	sestrin 2 [Source:HGNC Symbol;Acc:HGNC:20746]	3.0	2
1126	*GNL3*	26354	G protein nucleolar 3 [Source:HGNC Symbol;Acc:HGNC:29931]	3.0	2

1127	*RBPJ*	3516	recombination signal binding protein for immunoglobulin kappa J region [Source:HGNC Symbol;Acc:HGNC:5724]	3.0	2
1128	*OAS1*	4938	2'-5'-oligoadenylate synthetase 1 [Source:HGNC Symbol;Acc:HGNC:8086]	3.0	2
1129	*CAMSAP1*	157922	calmodulin regulated spectrin associated protein 1 [Source:HGNC Symbol;Acc:HGNC:19946]	3.0	2
1130	*CMIP*	80790	c-Maf inducing protein [Source:HGNC Symbol;Acc:HGNC:24319]	2.9	2
1131	*SLC19A1*	6573	solute carrier family 19 member 1 [Source:HGNC Symbol;Acc:HGNC:10937]	2.9	2
1132	*TWIST1*	7291	twist family bHLH transcription factor 1 [Source:HGNC Symbol;Acc:HGNC:12428]	2.9	2
1133	*FSTL5*	56884	follistatin like 5 [Source:HGNC Symbol;Acc:HGNC:21386]	2.9	2
1134	*SLC20A1*	6574	solute carrier family 20 member 1 [Source:HGNC Symbol;Acc:HGNC:10946]	2.9	2
1135	*FSCN3*	29999	fascin actin-bundling protein 3 [Source:HGNC Symbol;Acc:HGNC:3961]	2.9	2
1136	*NIN*	51199	ninein [Source:HGNC Symbol;Acc:HGNC:14906]	2.9	2
1137	*METTL21A*	151194	methyltransferase like 21A [Source:HGNC Symbol;Acc:HGNC:30476]	2.9	2
1138	*XPC*	7508	XPC complex subunit, DNA damage recognition and repair factor [Source:HGNC Symbol;Acc:HGNC:12816]	2.9	2
1139	*C8orf82*	414919	chromosome 8 open reading frame 82 [Source:HGNC Symbol;Acc:HGNC:33826]	2.9	2
1140	*LYN*	4067	LYN proto-oncogene, Src family tyrosine kinase [Source:HGNC Symbol;Acc:HGNC:6735]	2.9	2
1141	*CD300LF*	146722	CD300 molecule like family member f [Source:HGNC Symbol;Acc:HGNC:29883]	2.9	2
1142	*PTS*	5805	6-pyruvoyltetrahydropterin synthase [Source:HGNC Symbol;Acc:HGNC:9689]	2.9	2
1143	*SERPIND1*	3053	serpin family D member 1 [Source:HGNC Symbol;Acc:HGNC:4838]	2.9	2
1144	*GP1BB*	2812	glycoprotein Ib platelet beta subunit [Source:HGNC Symbol;Acc:HGNC:4440]	2.9	2
1145	*CLDN5*	7122	claudin 5 [Source:HGNC Symbol;Acc:HGNC:2047]	2.9	2
1146	*SMAP2*	64744	small ArfGAP2 [Source:HGNC Symbol;Acc:HGNC:25082]	2.9	2
1147	*FBN2*	2201	fibrillin 2 [Source:HGNC Symbol;Acc:HGNC:3604]	2.9	2
1148	*CSNK1A1L*	122011	casein kinase 1 alpha 1 like [Source:HGNC Symbol;Acc:HGNC:20289]	2.9	2
1149	*GART*	2618	phosphoribosylglycinamide formyltransferase, phosphoribosylglycinamide synthetase, phosphoribosylaminoimidazole synthetase [Source:HGNC Symbol;Acc:HGNC:4163]	2.9	2
1150	*SH3TC1*	54436	SH3 domain and tetratricopeptide repeats 1 [Source:HGNC Symbol;Acc:HGNC:26009]	2.9	2
1151	*COX6A1*	1337	cytochrome c oxidase subunit 6A1 [Source:HGNC Symbol;Acc:HGNC:2277]	2.9	2
1152	*TTC7B*	145567	tetratricopeptide repeat domain 7B [Source:HGNC Symbol;Acc:HGNC:19858]	2.9	2
1153	*LYPLA2*	11313	lysophospholipase II [Source:HGNC Symbol;Acc:HGNC:6738]	2.9	2
1154	*GYPA*	2993	glycophorin A (MNS blood group) [Source:HGNC Symbol;Acc:HGNC:4702]	2.9	2
1155	*MCM3*	4172	minichromosome maintenance complex component 3 [Source:HGNC Symbol;Acc:HGNC:6945]	2.9	2
1156	*IL1R2*	7850	interleukin 1 receptor type 2 [Source:HGNC Symbol;Acc:HGNC:5994]	2.9	2
1157	*SLC2A8*	29988	solute carrier family 2 member 8 [Source:HGNC Symbol;Acc:HGNC:13812]	2.9	2
1158	*SUZ12*	23512	SUZ12 polycomb repressive complex 2 subunit [Source:HGNC Symbol;Acc:HGNC:17101]	2.9	2
1159	*HCFC1R1*	54985	host cell factor C1 regulator 1 [Source:HGNC Symbol;Acc:HGNC:21198]	2.9	2
1160	*THOC6*	79228	THO complex 6 [Source:HGNC Symbol;Acc:HGNC:28369]	2.9	2
1161	*BRD7*	29117	bromodomain containing 7 [Source:HGNC Symbol;Acc:HGNC:14310]	2.9	2
1162	*HNRNPA0*	10949	heterogeneous nuclear ribonucleoprotein A0 [Source:HGNC Symbol;Acc:HGNC:5030]	2.9	2
1163	*PMPCB*	9512	peptidase, mitochondrial processing beta subunit [Source:HGNC Symbol;Acc:HGNC:9119]	2.9	2
1164	*CNPPD1*	27013	cyclin Pas1/PHO80 domain containing 1 [Source:HGNC Symbol;Acc:HGNC:25220]	2.9	2
1165	*UPP1*	7378	uridine phosphorylase 1 [Source:HGNC Symbol;Acc:HGNC:12576]	2.9	2
1166	*NAGK*	55577	N-acetylglucosamine kinase [Source:HGNC Symbol;Acc:HGNC:17174]	2.9	2
1167	*E2F6*	1876	E2F transcription factor 6 [Source:HGNC Symbol;Acc:HGNC:3120]	2.9	2
1168	*AIMP1*	9255	aminoacyl tRNA synthetase complex interacting multifunctional protein 1 [Source:HGNC Symbol;Acc:HGNC:10648]	2.9	2
1169	*LDLRAP1*	26119	low density lipoprotein receptor adaptor protein 1 [Source:HGNC Symbol;Acc:HGNC:18640]	2.9	2
1170	*EDARADD*	128178	EDAR-associated death domain [Source:HGNC Symbol;Acc:HGNC:14341]	2.9	2
1171	*KIF9*	64147	kinesin family member 9 [Source:HGNC Symbol;Acc:HGNC:16666]	2.9	2
1172	*TRAM1*	23471	translocation associated membrane protein 1 [Source:HGNC Symbol;Acc:HGNC:20568]	2.9	2
1173	*TMEM189-UBE2V1*	387522	TMEM189-UBE2V1 readthrough [Source:HGNC Symbol;Acc:HGNC:33521]	2.9	2
1174	*MYL6B*	140465	myosin light chain 6B [Source:HGNC Symbol;Acc:HGNC:29823]	2.9	2
1175	*PROS1*	5627	protein S (alpha) [Source:HGNC Symbol;Acc:HGNC:9456]	2.9	2
1176	*CMTM4*	146223	CKLF like MARVEL transmembrane domain containing 4 [Source:HGNC Symbol;Acc:HGNC:19175]	2.9	2
1177	*TLR8*	51311	toll like receptor 8 [Source:HGNC Symbol;Acc:HGNC:15632]	2.9	2
1178	*HABP4*	22927	hyaluronan binding protein 4 [Source:HGNC Symbol;Acc:HGNC:17062]	2.9	2
1179	*ENDOD1*	23052	endonuclease domain containing 1 [Source:HGNC Symbol;Acc:HGNC:29129]	2.9	2
1180	*SLC25A23*	79085	solute carrier family 25 member 23 [Source:HGNC Symbol;Acc:HGNC:19375]	2.9	2
1181	*EYA1*	2138	EYA transcriptional coactivator and phosphatase 1 [Source:HGNC Symbol;Acc:HGNC:3519]	2.9	2
1182	*PEAR1*	375033	platelet endothelial aggregation receptor 1 [Source:HGNC Symbol;Acc:HGNC:33631]	2.9	2
1183	*FGGY*	55277	FGGY carbohydrate kinase domain containing [Source:HGNC Symbol;Acc:HGNC:25610]	2.9	2
1184	*LPAR5*	57121	lysophosphatidic acid receptor 5 [Source:HGNC Symbol;Acc:HGNC:13307]	2.9	2
1185	*DHRS3*	9249	dehydrogenase/reductase 3 [Source:HGNC Symbol;Acc:HGNC:17693]	2.9	2
1186	*RASA3*	22821	RAS p21 protein activator 3 [Source:HGNC Symbol;Acc:HGNC:20331]	2.9	2
1187	*CAPG*	822	capping actin protein, gelsolin like [Source:HGNC Symbol;Acc:HGNC:1474]	2.9	2
1188	*CD33*	945	CD33 molecule [Source:HGNC Symbol;Acc:HGNC:1659]	2.9	2
1189	*KRR1*	11103	KRR1, small subunit processome component homolog [Source:HGNC Symbol;Acc:HGNC:5176]	2.9	2
1190	*FHL1*	2273	four and a half LIM domains 1 [Source:HGNC Symbol;Acc:HGNC:3702]	2.9	2
1191	*R3HDM4*	91300	R3H domain containing 4 [Source:HGNC Symbol;Acc:HGNC:28270]	2.9	2
1192	*PDCD11*	22984	programmed cell death 11 [Source:HGNC Symbol;Acc:HGNC:13408]	2.9	2
1193	*NLRC4*	58484	NLR family CARD domain containing 4 [Source:HGNC Symbol;Acc:HGNC:16412]	2.9	2
1194	*PWP2*	5822	PWP2 periodic tryptophan protein homolog (yeast) [Source:HGNC Symbol;Acc:HGNC:9711]	2.9	2
1195	*GNA15*	2769	G protein subunit alpha 15 [Source:HGNC Symbol;Acc:HGNC:4383]	2.9	2
1196	*GOLGA4*	2803	golgin A4 [Source:HGNC Symbol;Acc:HGNC:4427]	2.9	2
1197	*RNF24*	11237	ring finger protein 24 [Source:HGNC Symbol;Acc:HGNC:13779]	2.9	2
1198	*EPB41*	2035	erythrocyte membrane protein band 4.1 [Source:HGNC Symbol;Acc:HGNC:3377]	2.9	2
1199	*NEXN*	91624	nexilin F-actin binding protein [Source:HGNC Symbol;Acc:HGNC:29557]	2.9	2

1200	*KIAA1324*	57535	KIAA1324 [Source:HGNC Symbol;Acc:HGNC:29618]	2.9	2
1201	*CDC37L1*	55664	cell division cycle 37 like 1 [Source:HGNC Symbol;Acc:HGNC:17179]	2.9	2
1202	*FLI1*	2313	Fli-1 proto-oncogene, ETS transcription factor [Source:HGNC Symbol;Acc:HGNC:3749]	2.9	2
1203	*SIRT7*	51547	sirtuin 7 [Source:HGNC Symbol;Acc:HGNC:14935]	2.9	2
1204	*PPDPF*	79144	pancreatic progenitor cell differentiation and proliferation factor [Source:HGNC Symbol;Acc:HGNC:16142]	2.9	2
1205	*GLA*	2717	galactosidase alpha [Source:HGNC Symbol;Acc:HGNC:4296]	2.9	2
1206	*PHB2*	11331	prohibitin 2 [Source:HGNC Symbol;Acc:HGNC:30306]	2.9	2
1207	*STK17B*	9262	serine/threonine kinase 17b [Source:HGNC Symbol;Acc:HGNC:11396]	2.9	2
1208	*GTF3C4*	9329	general transcription factor IIIC subunit 4 [Source:HGNC Symbol;Acc:HGNC:4667]	2.9	2
1209	*CEP55*	55165	centrosomal protein 55 [Source:HGNC Symbol;Acc:HGNC:1161]	2.9	2
1210	*MYL5*	4636	myosin light chain 5 [Source:HGNC Symbol;Acc:HGNC:7586]	2.9	2
1211	*BPTF*	2186	bromodomain PHD finger transcription factor [Source:HGNC Symbol;Acc:HGNC:3581]	2.9	2
1212	*UCP2*	7351	uncoupling protein 2 [Source:HGNC Symbol;Acc:HGNC:12518]	2.9	2
1213	*CALM3*	808	calmodulin 3 (phosphorylase kinase, delta) [Source:HGNC Symbol;Acc:HGNC:1449]	2.9	2
1214	*NTNG2*	84628	netrin G2 [Source:HGNC Symbol;Acc:HGNC:14288]	2.9	2
1215	*LSP1*	4046	lymphocyte-specific protein 1 [Source:HGNC Symbol;Acc:HGNC:6707]	2.9	2
1216	*RFC3*	5983	replication factor C subunit 3 [Source:HGNC Symbol;Acc:HGNC:9971]	2.9	2
1217	*TMEM2*	23670	transmembrane protein 2 [Source:HGNC Symbol;Acc:HGNC:11869]	2.9	2
1218	*SIRPB2*	284759	signal regulatory protein beta 2 [Source:HGNC Symbol;Acc:HGNC:16247]	2.9	2
1219	*TTF1*	7270	transcription termination factor 1 [Source:HGNC Symbol;Acc:HGNC:12397]	2.9	2
1220	*ASAP2*	8853	ArfGAP with SH3 domain, ankyrin repeat and PH domain 2 [Source:HGNC Symbol;Acc:HGNC:2721]	2.9	2
1221	*ST6GAL1*	6480	ST6 beta-galactoside alpha-2,6-sialyltransferase 1 [Source:HGNC Symbol;Acc:HGNC:10860]	2.9	2
1222	*ZFYVE9*	9372	zinc finger FYVE-type containing 9 [Source:HGNC Symbol;Acc:HGNC:6775]	2.9	2
1223	*GINS2*	51659	GINS complex subunit 2 [Source:HGNC Symbol;Acc:HGNC:24575]	2.9	2
1224	*ACAT2*	39	acetyl-CoA acetyltransferase 2 [Source:HGNC Symbol;Acc:HGNC:94]	2.9	2
1225	*TCP1*	6950	t-complex 1 [Source:HGNC Symbol;Acc:HGNC:11655]	2.9	2
1226	*EIF4E2*	9470	eukaryotic translation initiation factor 4E family member 2 [Source:HGNC Symbol;Acc:HGNC:3293]	2.9	2
1227	*BABAM1*	29086	BRISC and BRCA1 A complex member 1 [Source:HGNC Symbol;Acc:HGNC:25008]	2.9	2
1228	*UBE2H*	7328	ubiquitin conjugating enzyme E2 H [Source:HGNC Symbol;Acc:HGNC:12484]	2.9	2
1229	*TSPAN9*	10867	tetraspanin 9 [Source:HGNC Symbol;Acc:HGNC:21640]	2.9	2
1230	*FGD2*	221472	FYVE, RhoGEF and PH domain containing 2 [Source:HGNC Symbol;Acc:HGNC:3664]	2.9	2
1231	*ASB3*	51130	ankyrin repeat and SOCS box containing 3 [Source:HGNC Symbol;Acc:HGNC:16013]	2.8	2
1232	*GIT2*	9815	GIT ArfGAP 2 [Source:HGNC Symbol;Acc:HGNC:4273]	2.8	2
1233	*HSPB1*	3315	heat shock protein family B (small) member 1 [Source:HGNC Symbol;Acc:HGNC:5246]	2.8	2
1234	*NCAM2*	4685	neural cell adhesion molecule 2 [Source:HGNC Symbol;Acc:HGNC:7657]	2.8	2
1235	*SH3BGRL3*	83442	SH3 domain binding glutamate rich protein like 3 [Source:HGNC Symbol;Acc:HGNC:15568]	2.8	2
1236	*MAN2A2*	4122	mannosidase alpha class 2A member 2 [Source:HGNC Symbol;Acc:HGNC:6825]	2.8	2
1237	*ENSA*	2029	endosulfine alpha [Source:HGNC Symbol;Acc:HGNC:3360]	2.8	2
1238	*GADD45A*	1647	growth arrest and DNA damage inducible alpha [Source:HGNC Symbol;Acc:HGNC:4095]	2.8	2
1239	*MFSD5*	84975	major facilitator superfamily domain containing 5 [Source:HGNC Symbol;Acc:HGNC:28156]	2.8	2
1240	*KDM6B*	23135	lysine demethylase 6B [Source:HGNC Symbol;Acc:HGNC:29012]	2.8	2
1241	*MRPL35*	51318	mitochondrial ribosomal protein L35 [Source:HGNC Symbol;Acc:HGNC:14489]	2.8	2
1242	*CBX3*	11335	chromobox 3 [Source:HGNC Symbol;Acc:HGNC:1553]	2.8	2
1243	*HTATIP2*	10553	HIV-1 Tat interactive protein 2 [Source:HGNC Symbol;Acc:HGNC:16637]	2.8	2
1244	*GAS2L3*	283431	growth arrest specific 2 like 3 [Source:HGNC Symbol;Acc:HGNC:27475]	2.8	2
1245	*SRP68*	6730	signal recognition particle 68 [Source:HGNC Symbol;Acc:HGNC:11302]	2.8	2
1246	*ARAP1*	116985	ArfGAP with RhoGAP domain, ankyrin repeat and PH domain 1 [Source:HGNC Symbol;Acc:HGNC:16925]	2.8	2
1247	*PYCR2*	29920	pyrroline-5-carboxylate reductase family member 2 [Source:HGNC Symbol;Acc:HGNC:30262]	2.8	2
1248	*TIMM21*	29090	translocase of inner mitochondrial membrane 21 [Source:HGNC Symbol;Acc:HGNC:25010]	2.8	2
1249	*PPP6R1*	22870	protein phosphatase 6 regulatory subunit 1 [Source:HGNC Symbol;Acc:HGNC:29195]	2.8	2
1250	*EGLN3*	112399	egl-9 family hypoxia inducible factor 3 [Source:HGNC Symbol;Acc:HGNC:14661]	2.8	2
1251	*SRSF4*	6429	serine and arginine rich splicing factor 4 [Source:HGNC Symbol;Acc:HGNC:10786]	2.8	2
1252	*ZNF592*	9640	zinc finger protein 592 [Source:HGNC Symbol;Acc:HGNC:28986]	2.8	2
1253	*AMFR*	267	autocrine motility factor receptor [Source:HGNC Symbol;Acc:HGNC:463]	2.8	2
1254	*UFC1*	51506	ubiquitin-fold modifier conjugating enzyme 1 [Source:HGNC Symbol;Acc:HGNC:26941]	2.8	2
1255	*NOB1*	28987	NIN1/PSMD8 binding protein 1 homolog [Source:HGNC Symbol;Acc:HGNC:29540]	2.8	2
1256	*TLR4*	7099	toll like receptor 4 [Source:HGNC Symbol;Acc:HGNC:11850]	2.8	2
1257	*CD9*	928	CD9 molecule [Source:HGNC Symbol;Acc:HGNC:1709]	2.8	2
1258	*IL10RA*	3587	interleukin 10 receptor subunit alpha [Source:HGNC Symbol;Acc:HGNC:5964]	2.8	2
1259	*S100Z*	170591	S100 calcium binding protein Z [Source:HGNC Symbol;Acc:HGNC:30367]	2.8	2
1260	*ARID3B*	10620	AT-rich interaction domain 3B [Source:HGNC Symbol;Acc:HGNC:14350]	2.8	2
1261	*SLC2A3*	6515	solute carrier family 2 member 3 [Source:HGNC Symbol;Acc:HGNC:11007]	2.8	2
1262	*ATL2*	64225	atlastin GTPase 2 [Source:HGNC Symbol;Acc:HGNC:24047]	2.8	2
1263	*CTNND1*	1500	catenin delta 1 [Source:HGNC Symbol;Acc:HGNC:2515]	2.8	2
1264	*TMSB4X*	7114	thymosin beta 4, X-linked [Source:HGNC Symbol;Acc:HGNC:11881]	2.8	2
1265	*CPD*	1362	carboxypeptidase D [Source:HGNC Symbol;Acc:HGNC:2301]	2.8	2
1266	*FRYL*	285527	FRY like transcription coactivator [Source:HGNC Symbol;Acc:HGNC:29127]	2.8	2
1267	*THBS1*	7057	thrombospondin 1 [Source:HGNC Symbol;Acc:HGNC:11785]	2.8	2
1268	*H1F0*	3005	H1 histone family member 0 [Source:HGNC Symbol;Acc:HGNC:4714]	2.8	2
1269	*RAD21*	5885	RAD21 cohesin complex component [Source:HGNC Symbol;Acc:HGNC:9811]	2.8	2
1270	*HDAC8*	55869	histone deacetylase 8 [Source:HGNC Symbol;Acc:HGNC:13315]	2.8	2
1271	*FAF1*	11124	Fas associated factor 1 [Source:HGNC Symbol;Acc:HGNC:3578]	2.8	2
1272	*MICAL1*	64780	microtubule associated monooxygenase, calponin and LIM domain containing 1 [Source:HGNC Symbol;Acc:HGNC:20619]	2.8	2
1273	*RNASE2*	6036	ribonuclease A family member 2 [Source:HGNC Symbol;Acc:HGNC:10045]	2.8	2

1274	*PTPRC*	5788	protein tyrosine phosphatase, receptor type C [Source:HGNC Symbol;Acc:HGNC:9666]	2.8	2
1275	*SP110*	3431	SP110 nuclear body protein [Source:HGNC Symbol;Acc:HGNC:5401]	2.8	2
1276	*VEZF1*	7716	vascular endothelial zinc finger 1 [Source:HGNC Symbol;Acc:HGNC:12949]	2.8	2
1277	*EIF4G1*	1981	eukaryotic translation initiation factor 4 gamma 1 [Source:HGNC Symbol;Acc:HGNC:3296]	2.8	2
1278	*STK10*	6793	serine/threonine kinase 10 [Source:HGNC Symbol;Acc:HGNC:11388]	2.8	2
1279	*GUCY1B3*	2983	guanylate cyclase 1, soluble, beta 3 [Source:HGNC Symbol;Acc:HGNC:4687]	2.8	2
1280	*KIF3C*	3797	kinesin family member 3C [Source:HGNC Symbol;Acc:HGNC:6321]	2.8	2
1281	*SVIP*	258010	small VCP/p97-interacting protein [Source:HGNC Symbol;Acc:HGNC:25238]	2.8	2
1282	*MLX*	6945	MLX, MAX dimerization protein [Source:HGNC Symbol;Acc:HGNC:11645]	2.8	2
1283	*MAPK13*	5603	mitogen-activated protein kinase 13 [Source:HGNC Symbol;Acc:HGNC:6875]	2.8	2
1284	*RAC2*	5880	ras-related C3 botulinum toxin substrate 2 (rho family, small GTP binding protein Rac2) [Source:HGNC Symbol;Acc:HGNC:9802]	2.8	2
1285	*MGAT4A*	11320	mannosyl (alpha-1,3-)-glycoprotein beta-1,4-N-acetylglucosaminyltransferase, isozyme A [Source:HGNC Symbol;Acc:HGNC:7047]	2.8	2
1286	*TLN1*	7094	talin 1 [Source:HGNC Symbol;Acc:HGNC:11845]	2.8	2
1287	*CHCHD4*	131474	coiled-coil-helix-coiled-coil-helix domain containing 4 [Source:HGNC Symbol;Acc:HGNC:26467]	2.8	2
1288	*CRISPLD1*	83690	cysteine rich secretory protein LCCL domain containing 1 [Source:HGNC Symbol;Acc:HGNC:18206]	2.8	2
1289	*AXL*	558	AXL receptor tyrosine kinase [Source:HGNC Symbol;Acc:HGNC:905]	2.8	2
1290	*GRK6*	2870	G protein-coupled receptor kinase 6 [Source:HGNC Symbol;Acc:HGNC:4545]	2.8	2
1291	*MED22*	6837	mediator complex subunit 22 [Source:HGNC Symbol;Acc:HGNC:11477]	2.8	2
1292	*ADGRG3*	222487	adhesion G protein-coupled receptor G3 [Source:HGNC Symbol;Acc:HGNC:13728]	2.8	2
1293	*C1orf174*	339448	chromosome 1 open reading frame 174 [Source:HGNC Symbol;Acc:HGNC:27915]	2.8	2
1294	*RAB8A*	4218	RAB8A, member RAS oncogene family [Source:HGNC Symbol;Acc:HGNC:7007]	2.8	2
1295	*PCED1A*	64773	PC-esterase domain containing 1A [Source:HGNC Symbol;Acc:HGNC:16212]	2.8	2
1296	*MCEMP1*	199675	mast cell-expressed membrane protein 1 [Source:HGNC Symbol;Acc:HGNC:27291]	2.8	2
1297	*TMEM71*	137835	transmembrane protein 71 [Source:HGNC Symbol;Acc:HGNC:26572]	2.8	2
1298	*NLRP3*	114548	NLR family pyrin domain containing 3 [Source:HGNC Symbol;Acc:HGNC:16400]	2.8	2
1299	*CABP5*	56344	calcium binding protein 5 [Source:HGNC Symbol;Acc:HGNC:13714]	2.8	2
1300	*P2RY13*	53829	purinergic receptor P2Y13 [Source:HGNC Symbol;Acc:HGNC:4537]	2.8	2
1301	*INPP5D*	3635	inositol polyphosphate-5-phosphatase D [Source:HGNC Symbol;Acc:HGNC:6079]	2.8	2
1302	*PCGF5*	84333	polycomb group ring finger 5 [Source:HGNC Symbol;Acc:HGNC:28264]	2.8	2
1303	*AEN*	64782	apoptosis enhancing nuclease [Source:HGNC Symbol;Acc:HGNC:25722]	2.8	2
1304	*EXOSC1*	51013	exosome component 1 [Source:HGNC Symbol;Acc:HGNC:17286]	2.8	2
1305	*C15orf52*	388115	chromosome 15 open reading frame 52 [Source:HGNC Symbol;Acc:HGNC:33488]	2.8	2
1306	*MIA*	8190	melanoma inhibitory activity [Source:HGNC Symbol;Acc:HGNC:7076]	2.8	2
1307	*RAB4B*	53916	RAB4B, member RAS oncogene family [Source:HGNC Symbol;Acc:HGNC:9782]	2.8	2
1308	*DARS2*	55157	aspartyl-tRNA synthetase 2, mitochondrial [Source:HGNC Symbol;Acc:HGNC:25538]	2.8	2
1309	*HIST1H3B*	8358	histone cluster 1, H3b [Source:HGNC Symbol;Acc:HGNC:4776]	2.8	2
1310	*PTPRS*	5802	protein tyrosine phosphatase, receptor type S [Source:HGNC Symbol;Acc:HGNC:9681]	2.8	2
1311	*FAM227B*	196951	family with sequence similarity 227 member B [Source:HGNC Symbol;Acc:HGNC:26543]	2.8	2
1312	*GALK2*	2585	galactokinase 2 [Source:HGNC Symbol;Acc:HGNC:4119]	2.8	2
1313	*ATP13A1*	57130	ATPase 13A1 [Source:HGNC Symbol;Acc:HGNC:24215]	2.8	2
1314	*MED21*	9412	mediator complex subunit 21 [Source:HGNC Symbol;Acc:HGNC:11473]	2.8	2
1315	*LOXL3*	84695	lysyl oxidase like 3 [Source:HGNC Symbol;Acc:HGNC:13869]	2.8	2
1316	*MTHFR*	4524	methylenetetrahydrofolate reductase (NAD(P)H) [Source:HGNC Symbol;Acc:HGNC:7436]	2.8	2
1317	*GNL1*	2794	G protein nucleolar 1 (putative) [Source:HGNC Symbol;Acc:HGNC:4413]	2.8	2
1318	*SRGN*	5552	serglycin [Source:HGNC Symbol;Acc:HGNC:9361]	2.8	2
1319	*GNE*	10020	glucosamine (UDP-N-acetyl)-2-epimerase/N-acetylmannosamine kinase [Source:HGNC Symbol;Acc:HGNC:23657]	2.8	2
1320	*KPNB1*	3837	karyopherin subunit beta 1 [Source:HGNC Symbol;Acc:HGNC:6400]	2.8	2
1321	*SLCO4A1*	28231	solute carrier organic anion transporter family member 4A1 [Source:HGNC Symbol;Acc:HGNC:10953]	2.8	2
1322	*PFDN4*	5203	prefoldin subunit 4 [Source:HGNC Symbol;Acc:HGNC:8868]	2.8	2
1323	*SLC37A4*	2542	solute carrier family 37 member 4 [Source:HGNC Symbol;Acc:HGNC:4061]	2.8	2
1324	*MIER1*	57708	MIER1 transcriptional regulator [Source:HGNC Symbol;Acc:HGNC:29657]	2.8	2
1325	*AGER*	177	advanced glycosylation end product-specific receptor [Source:HGNC Symbol;Acc:HGNC:320]	2.8	2
1326	*RXRA*	6256	retinoid X receptor alpha [Source:HGNC Symbol;Acc:HGNC:10477]	2.8	2
1327	*CTSA*	5476	cathepsin A [Source:HGNC Symbol;Acc:HGNC:9251]	2.8	2
1328	*PIK3R5*	23533	phosphoinositide-3-kinase regulatory subunit 5 [Source:HGNC Symbol;Acc:HGNC:30035]	2.8	2
1329	*C15orf39*	56905	chromosome 15 open reading frame 39 [Source:HGNC Symbol;Acc:HGNC:24497]	2.8	2
1330	*FKBP1B*	2281	FK506 binding protein 1B [Source:HGNC Symbol;Acc:HGNC:3712]	2.8	2
1331	*MCTP1*	79772	multiple C2 and transmembrane domain containing 1 [Source:HGNC Symbol;Acc:HGNC:26183]	2.8	2
1332	*ZC3HAV1L*	92092	zinc finger CCCH-type containing, antiviral 1 like [Source:HGNC Symbol;Acc:HGNC:22423]	2.8	2
1333	*SSSCA1*	10534	Sjogren syndrome/scleroderma autoantigen 1 [Source:HGNC Symbol;Acc:HGNC:11328]	2.8	2
1334	*MXD4*	10608	MAX dimerization protein 4 [Source:HGNC Symbol;Acc:HGNC:13906]	2.8	2
1335	*MAP3K3*	4215	mitogen-activated protein kinase kinase kinase 3 [Source:HGNC Symbol;Acc:HGNC:6855]	2.8	2
1336	*C6orf201*	404220	chromosome 6 open reading frame 201 [Source:HGNC Symbol;Acc:HGNC:21620]	2.8	2
1337	*ABCA7*	10347	ATP binding cassette subfamily A member 7 [Source:HGNC Symbol;Acc:HGNC:37]	2.8	2
1338	*SCN11A*	11280	sodium voltage-gated channel alpha subunit 11 [Source:HGNC Symbol;Acc:HGNC:10583]	2.7	2
1339	*B4GALT1*	2683	beta-1,4-galactosyltransferase 1 [Source:HGNC Symbol;Acc:HGNC:924]	2.7	2
1340	*AOAH*	313	acyloxyacyl hydrolase [Source:HGNC Symbol;Acc:HGNC:548]	2.7	2
1341	*SRSF3*	6428	serine and arginine rich splicing factor 3 [Source:HGNC Symbol;Acc:HGNC:10785]	2.7	2
1342	*BUB1B*	701	BUB1 mitotic checkpoint serine/threonine kinase B [Source:HGNC Symbol;Acc:HGNC:1149]	2.7	2
1343	*PAK6*	56924	p21 (RAC1) activated kinase 6 [Source:HGNC Symbol;Acc:HGNC:16061]	2.7	2
1344	*SOD2*	6648	superoxide dismutase 2, mitochondrial [Source:HGNC Symbol;Acc:HGNC:11180]	2.7	2
1345	*GIMAP8*	155038	GTPase, IMAP family member 8 [Source:HGNC Symbol;Acc:HGNC:21792]	2.7	2

1346	*ZNF445*	353274	zinc finger protein 445 [Source:HGNC Symbol;Acc:HGNC:21018]	2.7	2
1347	*SLC39A10*	57181	solute carrier family 39 member 10 [Source:HGNC Symbol;Acc:HGNC:20861]	2.7	2
1348	*NADK*	65220	NAD kinase [Source:HGNC Symbol;Acc:HGNC:29831]	2.7	2
1349	*ANG*	283	angiogenin [Source:HGNC Symbol;Acc:HGNC:483]	2.7	2
1350	*DPYSL2*	1808	dihydropyrimidinase like 2 [Source:HGNC Symbol;Acc:HGNC:3014]	2.7	2
1351	*PIP4K2C*	79837	phosphatidylinositol-5-phosphate 4-kinase type 2 gamma [Source:HGNC Symbol;Acc:HGNC:23786]	2.7	2
1352	*WDR41*	55255	WD repeat domain 41 [Source:HGNC Symbol;Acc:HGNC:25601]	2.7	2
1353	*POLR3GL*	84265	polymerase (RNA) III subunit G like [Source:HGNC Symbol;Acc:HGNC:28466]	2.7	2
1354	*ITPKC*	80271	inositol-trisphosphate 3-kinase C [Source:HGNC Symbol;Acc:HGNC:14897]	2.7	2
1355	*HMG20B*	10362	high mobility group 20B [Source:HGNC Symbol;Acc:HGNC:5002]	2.7	2
1356	*C2*	717	complement component 2 [Source:HGNC Symbol;Acc:HGNC:1248]	2.7	2
1357	*STBD1*	8987	starch binding domain 1 [Source:HGNC Symbol;Acc:HGNC:24854]	2.7	2
1358	*ACP1*	52	acid phosphatase 1, soluble [Source:HGNC Symbol;Acc:HGNC:122]	2.7	2
1359	*NOMO1*	23420	NODAL modulator 1 [Source:HGNC Symbol;Acc:HGNC:30060]	2.7	2
1360	*HIST1H2BF*	8343	histone cluster 1, H2bf [Source:HGNC Symbol;Acc:HGNC:4752]	2.7	2
1361	*PGD*	5226	phosphogluconate dehydrogenase [Source:HGNC Symbol;Acc:HGNC:8891]	2.7	2
1362	*HIST1H2BE*	8344	histone cluster 1, H2be [Source:HGNC Symbol;Acc:HGNC:4753]	2.7	2
1363	*CCDC71L*	168455	coiled-coil domain containing 71-like [Source:HGNC Symbol;Acc:HGNC:26685]	2.7	2
1364	*MDH2*	4191	malate dehydrogenase 2 [Source:HGNC Symbol;Acc:HGNC:6971]	2.7	2
1365	*RGS2*	5997	regulator of G-protein signaling 2 [Source:HGNC Symbol;Acc:HGNC:9998]	2.7	2
1366	*MGAT4B*	11282	mannosyl (alpha-1,3-)-glycoprotein beta-1,4-N-acetylglucosaminyltransferase, isozyme B [Source:HGNC Symbol;Acc:HGNC:7048]	2.7	2
1367	*SLC5A3*	6526	solute carrier family 5 member 3 [Source:HGNC Symbol;Acc:HGNC:11038]	2.7	2
1368	*PSMD1*	5707	proteasome 26S subunit, non-ATPase 1 [Source:HGNC Symbol;Acc:HGNC:9554]	2.7	2
1369	*HMBOX1*	79618	homeobox containing 1 [Source:HGNC Symbol;Acc:HGNC:26137]	2.7	2
1370	*AKAP12*	9590	A-kinase anchoring protein 12 [Source:HGNC Symbol;Acc:HGNC:370]	2.7	2
1371	*NOTCH1*	4851	notch 1 [Source:HGNC Symbol;Acc:HGNC:7881]	2.7	2
1372	*KIAA0513*	9764	KIAA0513 [Source:HGNC Symbol;Acc:HGNC:29058]	2.7	2
1373	*IGF2R*	3482	insulin like growth factor 2 receptor [Source:HGNC Symbol;Acc:HGNC:5467]	2.7	2
1374	*RPS6KB1*	6198	ribosomal protein S6 kinase B1 [Source:HGNC Symbol;Acc:HGNC:10436]	2.7	2
1375	*TIMP1*	7076	TIMP metallopeptidase inhibitor 1 [Source:HGNC Symbol;Acc:HGNC:11820]	2.7	2
1376	*ABCC3*	8714	ATP binding cassette subfamily C member 3 [Source:HGNC Symbol;Acc:HGNC:54]	2.7	2
1377	*DCTN2*	10540	dynactin subunit 2 [Source:HGNC Symbol;Acc:HGNC:2712]	2.7	2
1378	*EAF1*	85403	ELL associated factor 1 [Source:HGNC Symbol;Acc:HGNC:20907]	2.7	2
1379	*PGRMC1*	10857	progesterone receptor membrane component 1 [Source:HGNC Symbol;Acc:HGNC:16090]	2.7	2
1380	*RUNX3*	864	runt related transcription factor 3 [Source:HGNC Symbol;Acc:HGNC:10473]	2.7	2
1381	*ITGB7*	3695	integrin subunit beta 7 [Source:HGNC Symbol;Acc:HGNC:6162]	2.7	2
1382	*GAS7*	8522	growth arrest specific 7 [Source:HGNC Symbol;Acc:HGNC:4169]	2.7	2
1383	*CRTC3*	64784	CREB regulated transcription coactivator 3 [Source:HGNC Symbol;Acc:HGNC:26148]	2.7	2
1384	*RFX5*	5993	regulatory factor X5 [Source:HGNC Symbol;Acc:HGNC:9986]	2.7	2
1385	*CCDC91*	55297	coiled-coil domain containing 91 [Source:HGNC Symbol;Acc:HGNC:24855]	2.7	2
1386	*CYTH4*	27128	cytohesin 4 [Source:HGNC Symbol;Acc:HGNC:9505]	2.7	2
1387	*MARCH1*	55016	membrane associated ring-CH-type finger 1 [Source:HGNC Symbol;Acc:HGNC:26077]	2.7	2
1388	*ZBTB7A*	51341	zinc finger and BTB domain containing 7A [Source:HGNC Symbol;Acc:HGNC:18078]	2.7	2
1389	*NRBF2*	29982	nuclear receptor binding factor 2 [Source:HGNC Symbol;Acc:HGNC:19692]	2.7	2
1390	*LCP2*	3937	lymphocyte cytosolic protein 2 [Source:HGNC Symbol;Acc:HGNC:6529]	2.7	2
1391	*MRPL33*	9553	mitochondrial ribosomal protein L33 [Source:HGNC Symbol;Acc:HGNC:14487]	2.7	2
1392	*TXNDC16*	57544	thioredoxin domain containing 16 [Source:HGNC Symbol;Acc:HGNC:19965]	2.7	2
1393	*PXK*	54899	PX domain containing serine/threonine kinase like [Source:HGNC Symbol;Acc:HGNC:23326]	2.7	2
1394	*PPP1R15A*	23645	protein phosphatase 1 regulatory subunit 15A [Source:HGNC Symbol;Acc:HGNC:14375]	2.7	2
1395	*RRM2*	6241	ribonucleotide reductase regulatory subunit M2 [Source:HGNC Symbol;Acc:HGNC:10452]	2.7	2
1396	*MT1X*	4501	metallothionein 1X [Source:HGNC Symbol;Acc:HGNC:7405]	2.7	2
1397	*PPM1L*	151742	protein phosphatase, Mg2+/Mn2+ dependent 1L [Source:HGNC Symbol;Acc:HGNC:16381]	2.7	2
1398	*RCSD1*	92241	RCSD domain containing 1 [Source:HGNC Symbol;Acc:HGNC:28310]	2.7	2
1399	*ADGRE2*	30817	adhesion G protein-coupled receptor E2 [Source:HGNC Symbol;Acc:HGNC:3337]	2.7	2
1400	*IL16*	3603	interleukin 16 [Source:HGNC Symbol;Acc:HGNC:5980]	2.7	2
1401	*TAX1BP1*	8887	Tax1 binding protein 1 [Source:HGNC Symbol;Acc:HGNC:11575]	2.7	2
1402	*TMEM261*	90871	transmembrane protein 261 [Source:HGNC Symbol;Acc:HGNC:30536]	2.7	2
1403	*AP1S2*	8905	adaptor related protein complex 1 sigma 2 subunit [Source:HGNC Symbol;Acc:HGNC:560]	2.7	2
1404	*KMT2E*	55904	lysine methyltransferase 2E [Source:HGNC Symbol;Acc:HGNC:18541]	2.7	2
1405	*PIP4K2A*	5305	phosphatidylinositol-5-phosphate 4-kinase type 2 alpha [Source:HGNC Symbol;Acc:HGNC:8997]	2.7	2
1406	*ZBED3*	84327	zinc finger BED-type containing 3 [Source:HGNC Symbol;Acc:HGNC:20711]	2.7	2
1407	*TRA2B*	6434	transformer 2 beta homolog (Drosophila) [Source:HGNC Symbol;Acc:HGNC:10781]	2.7	2
1408	*SLCO3A1*	28232	solute carrier organic anion transporter family member 3A1 [Source:HGNC Symbol;Acc:HGNC:10952]	2.7	2
1409	*SRP19*	6728	signal recognition particle 19kDa [Source:HGNC Symbol;Acc:HGNC:11300]	2.7	2
1410	*LIMS1*	3987	LIM Zinc Finger Domain Containing 1	2.7	2
1411	*SGOL2*	151246	Shugoshin 2	2.7	2
1412	*SMC2*	10592	structural maintenance of chromosomes 2 [Source:HGNC Symbol;Acc:HGNC:14011]	2.7	2
1413	*EFCAB10*	100130771	EF-hand calcium binding domain 10 [Source:HGNC Symbol;Acc:HGNC:34531]	2.7	2
1414	*CCND3*	896	cyclin D3 [Source:HGNC Symbol;Acc:HGNC:1585]	2.7	2
1415	*ATP6AP1*	537	ATPase H+ transporting accessory protein 1 [Source:HGNC Symbol;Acc:HGNC:868]	2.7	2
1416	*DNAJC11*	55735	DnaJ heat shock protein family (Hsp40) member C11 [Source:HGNC Symbol;Acc:HGNC:25570]	2.7	2
1417	*TFPI*	7035	tissue factor pathway inhibitor [Source:HGNC Symbol;Acc:HGNC:11760]	2.7	2
1418	*MIPEP*	4285	mitochondrial intermediate peptidase [Source:HGNC Symbol;Acc:HGNC:7104]	2.7	2
1419	*SLC25A37*	51312	solute carrier family 25 member 37 [Source:HGNC Symbol;Acc:HGNC:29786]	2.7	2
1420	*MKI67*	4288	marker of proliferation Ki-67 [Source:HGNC Symbol;Acc:HGNC:7107]	2.7	2
1421	*MFNG*	4242	MFNG O-fucosylpeptide 3-beta-N-acetylglucosaminyltransferase [Source:HGNC Symbol;Acc:HGNC:7038]	2.7	2

1422	*NAT9*	26151	N-acetyltransferase 9 (putative) [Source:HGNC Symbol;Acc:HGNC:23133]	2.7	2
1423	*FRAT1*	10023	frequently rearranged in advanced T-cell lymphomas 1 [Source:HGNC Symbol;Acc:HGNC:3944]	2.7	2
1424	*LBR*	3930	lamin B receptor [Source:HGNC Symbol;Acc:HGNC:6518]	2.7	2
1425	*NFKB1*	4790	nuclear factor kappa B subunit 1 [Source:HGNC Symbol;Acc:HGNC:7794]	2.7	2
1426	*TRAPPC6A*	79090	trafficking protein particle complex 6A [Source:HGNC Symbol;Acc:HGNC:23069]	2.7	2
1427	*ABCC4*	10257	ATP binding cassette subfamily C member 4 [Source:HGNC Symbol;Acc:HGNC:55]	2.7	2
1428	*HNRNPA1*	3178	heterogeneous nuclear ribonucleoprotein A1 [Source:HGNC Symbol;Acc:HGNC:5031]	2.7	2
1429	*GLIPR2*	152007	GLI pathogenesis related 2 [Source:HGNC Symbol;Acc:HGNC:18007]	2.6	2
1430	*IRF7*	3665	interferon regulatory factor 7 [Source:HGNC Symbol;Acc:HGNC:6122]	2.6	2
1431	*TMEM140*	55281	transmembrane protein 140 [Source:HGNC Symbol;Acc:HGNC:21870]	2.6	2
1432	*MCUR1*	63933	mitochondrial calcium uniporter regulator 1 [Source:HGNC Symbol;Acc:HGNC:21097]	2.6	2
1433	*MMD*	23531	monocyte to macrophage differentiation associated [Source:HGNC Symbol;Acc:HGNC:7153]	2.6	2
1434	*USP31*	57478	ubiquitin specific peptidase 31 [Source:HGNC Symbol;Acc:HGNC:20060]	2.6	2
1435	*ZNF330*	27309	zinc finger protein 330 [Source:HGNC Symbol;Acc:HGNC:15462]	2.6	2
1436	*DNAJC22*	79962	DnaJ heat shock protein family (Hsp40) member C22 [Source:HGNC Symbol;Acc:HGNC:25802]	2.6	2
1437	*ZAP70*	7535	zeta chain of T cell receptor associated protein kinase 70kDa [Source:HGNC Symbol;Acc:HGNC:12858]	2.6	2
1438	*UBA3*	9039	ubiquitin like modifier activating enzyme 3 [Source:HGNC Symbol;Acc:HGNC:12470]	2.6	2
1439	*MON1A*	84315	MON1 homolog A, secretory trafficking associated [Source:HGNC Symbol;Acc:HGNC:28207]	2.6	2
1440	*TREM1*	54210	triggering receptor expressed on myeloid cells 1 [Source:HGNC Symbol;Acc:HGNC:17760]	2.6	2
1441	*NIPSNAP1*	8508	nipsnap homolog 1 (C. elegans) [Source:HGNC Symbol;Acc:HGNC:7827]	2.6	2
1442	*SLC35C1*	55343	solute carrier family 35 member C1 [Source:HGNC Symbol;Acc:HGNC:20197]	2.6	2
1443	*EVI2B*	2124	ecotropic viral integration site 2B [Source:HGNC Symbol;Acc:HGNC:3500]	2.6	2
1444	*SAC3D1*	29901	SAC3 domain containing 1 [Source:HGNC Symbol;Acc:HGNC:30179]	2.6	2
1445	*PAPSS1*	9061	3'-phosphoadenosine 5'-phosphosulfate synthase 1 [Source:HGNC Symbol;Acc:HGNC:8603]	2.6	2
1446	*AK2*	204	adenylate kinase 2 [Source:HGNC Symbol;Acc:HGNC:362]	2.6	2
1447	*ULK4*	54986	unc-51 like kinase 4 [Source:HGNC Symbol;Acc:HGNC:15784]	2.6	2
1448	*FXYD5*	53827	FXYD domain containing ion transport regulator 5 [Source:HGNC Symbol;Acc:HGNC:4029]	2.6	2
1449	*ZC3H12A*	80149	zinc finger CCCH-type containing 12A [Source:HGNC Symbol;Acc:HGNC:26259]	2.6	2
1450	*ST3GAL1*	6482	ST3 beta-galactoside alpha-2,3-sialyltransferase 1 [Source:HGNC Symbol;Acc:HGNC:10862]	2.6	2
1451	*ETV1*	2115	ETS variant 1 [Source:HGNC Symbol;Acc:HGNC:3490]	2.6	2
1452	*PTPN6*	5777	protein tyrosine phosphatase, non-receptor type 6 [Source:HGNC Symbol;Acc:HGNC:9658]	2.6	2
1453	*ELOVL7*	79993	ELOVL fatty acid elongase 7 [Source:HGNC Symbol;Acc:HGNC:26292]	2.6	2
1454	*ZNF473*	25888	zinc finger protein 473 [Source:HGNC Symbol;Acc:HGNC:23239]	2.6	2
1455	*BICD2*	23299	BICD cargo adaptor 2 [Source:HGNC Symbol;Acc:HGNC:17208]	2.6	2
1456	*LAT2*	7462	linker for activation of T-cells family member 2 [Source:HGNC Symbol;Acc:HGNC:12749]	2.6	2
1457	*DOK1*	1796	docking protein 1 [Source:HGNC Symbol;Acc:HGNC:2990]	2.6	2
1458	*CALML3*	810	calmodulin like 3 [Source:HGNC Symbol;Acc:HGNC:1452]	2.6	2
1459	*ETF1*	2107	eukaryotic translation termination factor 1 [Source:HGNC Symbol;Acc:HGNC:3477]	2.6	2
1460	*IFI27L1*	122509	interferon alpha inducible protein 27 like 1 [Source:HGNC Symbol;Acc:HGNC:19754]	2.6	2
1461	*IRS2*	8660	insulin receptor substrate 2 [Source:HGNC Symbol;Acc:HGNC:6126]	2.6	2
1462	*HSPA2*	3306	heat shock protein family A (Hsp70) member 2 [Source:HGNC Symbol;Acc:HGNC:5235]	2.6	2
1463	*LRMP*	4033	lymphoid restricted membrane protein [Source:HGNC Symbol;Acc:HGNC:6690]	2.6	2
1464	*HES1*	3280	hes family bHLH transcription factor 1 [Source:HGNC Symbol;Acc:HGNC:5192]	2.6	2
1465	*TM4SF1*	4071	transmembrane 4 L six family member 1 [Source:HGNC Symbol;Acc:HGNC:11853]	2.6	2
1466	*TFAP2C*	7022	transcription factor AP-2 gamma [Source:HGNC Symbol;Acc:HGNC:11744]	2.6	2
1467	*MAPKAPK3*	7867	mitogen-activated protein kinase-activated protein kinase 3 [Source:HGNC Symbol;Acc:HGNC:6888]	2.6	2
1468	*KLHDC8B*	200942	kelch domain containing 8B [Source:HGNC Symbol;Acc:HGNC:28557]	2.6	2
1469	*CREBRF*	153222	CREB3 regulatory factor [Source:HGNC Symbol;Acc:HGNC:24050]	2.6	2
1470	*NUDCD2*	134492	NudC domain containing 2 [Source:HGNC Symbol;Acc:HGNC:30535]	2.6	2
1471	*CPPED1*	55313	calcineurin like phosphoesterase domain containing 1 [Source:HGNC Symbol;Acc:HGNC:25632]	2.6	2
1472	*CD99*	4267	CD99 molecule [Source:HGNC Symbol;Acc:HGNC:7082]	2.6	2
1473	*RAB4A*	5867	RAB4A, member RAS oncogene family [Source:HGNC Symbol;Acc:HGNC:9781]	2.6	2
1474	*LTBP1*	4052	latent transforming growth factor beta binding protein 1 [Source:HGNC Symbol;Acc:HGNC:6714]	2.6	2
1475	*RABGAP1L*	9910	RAB GTPase activating protein 1-like [Source:HGNC Symbol;Acc:HGNC:24663]	2.6	2
1476	*BANK1*	55024	B-cell scaffold protein with ankyrin repeats 1 [Source:HGNC Symbol;Acc:HGNC:18233]	2.6	2
1477	*PAXIP1*	22976	PAX interacting protein 1 [Source:HGNC Symbol;Acc:HGNC:8624]	2.6	2
1478	*TMEM154*	201799	transmembrane protein 154 [Source:HGNC Symbol;Acc:HGNC:26489]	2.6	2
1479	*KCTD20*	222658	potassium channel tetramerization domain containing 20 [Source:HGNC Symbol;Acc:HGNC:21052]	2.6	2
1480	*HIST1H2BC*	8347	histone cluster 1, H2bc [Source:HGNC Symbol;Acc:HGNC:4757]	2.6	2
1481	*TTYH2*	94015	tweety family member 2 [Source:HGNC Symbol;Acc:HGNC:13877]	2.6	2
1482	*TARBP2*	6895	TARBP2, RISC loading complex RNA binding subunit [Source:HGNC Symbol;Acc:HGNC:11569]	2.6	2
1483	*ARRB2*	409	arrestin beta 2 [Source:HGNC Symbol;Acc:HGNC:712]	2.6	2
1484	*CCT6A*	908	chaperonin containing TCP1 subunit 6A [Source:HGNC Symbol;Acc:HGNC:1620]	2.6	2
1485	*STK4*	6789	serine/threonine kinase 4 [Source:HGNC Symbol;Acc:HGNC:11408]	2.6	2
1486	*EHBP1L1*	254102	EH domain binding protein 1 like 1 [Source:HGNC Symbol;Acc:HGNC:30682]	2.6	2
1487	*FRS2*	10818	fibroblast growth factor receptor substrate 2 [Source:HGNC Symbol;Acc:HGNC:16971]	2.6	2
1488	*CECR1*	51816	cat eye syndrome chromosome region, candidate 1 [Source:HGNC Symbol;Acc:HGNC:1839]	2.6	2
1489	*GLRX5*	51218	glutaredoxin 5 [Source:HGNC Symbol;Acc:HGNC:20134]	2.6	2
1490	*ZDHHC4*	55146	zinc finger DHHC-type containing 4 [Source:HGNC Symbol;Acc:HGNC:18471]	2.6	2

1491	*DOCK8*	81704	dedicator of cytokinesis 8 [Source:HGNC Symbol;Acc:HGNC:19191]	2.6	2
1492	*TBXAS1*	6916	thromboxane A synthase 1 [Source:HGNC Symbol;Acc:HGNC:11609]	2.6	2
1493	*CAMTA2*	23125	calmodulin binding transcription activator 2 [Source:HGNC Symbol;Acc:HGNC:18807]	2.6	2
1494	*FYN*	2534	FYN proto-oncogene, Src family tyrosine kinase [Source:HGNC Symbol;Acc:HGNC:4037]	2.6	2
1495	*FAM213A*	84293	family with sequence similarity 213 member A [Source:HGNC Symbol;Acc:HGNC:28651]	2.6	2
1496	*SPTLC2*	9517	serine palmitoyltransferase long chain base subunit 2 [Source:HGNC Symbol;Acc:HGNC:11278]	2.6	2
1497	*TIMM23*	100287932	translocase of inner mitochondrial membrane 23 [Source:HGNC Symbol;Acc:HGNC:17312]	2.6	2
1498	*POLD3*	10714	polymerase (DNA) delta 3, accessory subunit [Source:HGNC Symbol;Acc:HGNC:20932]	2.6	2
1499	*PLK2*	10769	polo like kinase 2 [Source:HGNC Symbol;Acc:HGNC:19699]	2.6	2
1500	*CD300A*	11314	CD300a molecule [Source:HGNC Symbol;Acc:HGNC:19319]	2.6	2
1501	*KLHL6*	89857	kelch like family member 6 [Source:HGNC Symbol;Acc:HGNC:18653]	2.6	2
1502	*RPL22L1*	200916	ribosomal protein L22 like 1 [Source:HGNC Symbol;Acc:HGNC:27610]	2.6	2
1503	*TYMP*	1890	thymidine phosphorylase [Source:HGNC Symbol;Acc:HGNC:3148]	2.6	2
1504	*GCH1*	2643	GTP cyclohydrolase 1 [Source:HGNC Symbol;Acc:HGNC:4193]	2.6	2
1505	*HSPA14*	51182	heat shock protein family A (Hsp70) member 14 [Source:HGNC Symbol;Acc:HGNC:29526]	2.6	2
1506	*DLC1*	10395	DLC1 Rho GTPase activating protein [Source:HGNC Symbol;Acc:HGNC:2897]	2.6	2
1507	*RPL39L*	116832	ribosomal protein L39 like [Source:HGNC Symbol;Acc:HGNC:17094]	2.6	2
1508	*CPED1*	79974	cadherin like and PC-esterase domain containing 1 [Source:HGNC Symbol;Acc:HGNC:26159]	2.6	2
1509	*VIM*	7431	vimentin [Source:HGNC Symbol;Acc:HGNC:12692]	2.6	2
1510	*XPO6*	23214	exportin 6 [Source:HGNC Symbol;Acc:HGNC:19733]	2.6	2
1511	*FAM208B*	54906	family with sequence similarity 208 member B [Source:HGNC Symbol;Acc:HGNC:23484]	2.6	2
1512	*SESTD1*	91404	SEC14 and spectrin domain containing 1 [Source:HGNC Symbol;Acc:HGNC:18379]	2.6	2
1513	*PSMD11*	5717	proteasome 26S subunit, non-ATPase 11 [Source:HGNC Symbol;Acc:HGNC:9556]	2.6	2
1514	*ITGA6*	3655	integrin subunit alpha 6 [Source:HGNC Symbol;Acc:HGNC:6142]	2.6	2
1515	*CCR1*	1230	C-C motif chemokine receptor 1 [Source:HGNC Symbol;Acc:HGNC:1602]	2.6	2
1516	*CFAP20*	29105	cilia and flagella associated protein 20 [Source:HGNC Symbol;Acc:HGNC:29523]	2.6	2
1517	*SMARCA1*	6594	SWI/SNF related, matrix associated, actin dependent regulator of chromatin, subfamily a, member 1 [Source:HGNC Symbol;Acc:HGNC:11097]	2.6	2
1518	*GPSM3*	63940	G-protein signaling modulator 3 [Source:HGNC Symbol;Acc:HGNC:13945]	2.6	2
1519	*GPBAR1*	151306	G protein-coupled bile acid receptor 1 [Source:HGNC Symbol;Acc:HGNC:19680]	2.6	2
1520	*XRCC1*	7515	X-ray repair cross complementing 1 [Source:HGNC Symbol;Acc:HGNC:12828]	2.6	2
1521	*UBL7*	84993	ubiquitin like 7 [Source:HGNC Symbol;Acc:HGNC:28221]	2.6	2
1522	*PRKX*	5613	protein kinase, X-linked [Source:HGNC Symbol;Acc:HGNC:9441]	2.6	2
1523	*SNURF*	8926	SNRPN upstream reading frame [Source:HGNC Symbol;Acc:HGNC:11171]	2.5	2
1524	*DOK4*	55715	docking protein 4 [Source:HGNC Symbol;Acc:HGNC:19868]	2.5	2
1525	*FAM101B*	359845	family with sequence similarity 101 member B [Source:HGNC Symbol;Acc:HGNC:28705]	2.5	2
1526	*FLNB*	2317	filamin B [Source:HGNC Symbol;Acc:HGNC:3755]	2.5	2
1527	*CPM*	1368	carboxypeptidase M [Source:HGNC Symbol;Acc:HGNC:2311]	2.5	2
1528	*HMGB3*	3149	high mobility group box 3 [Source:HGNC Symbol;Acc:HGNC:5004]	2.5	2
1529	*NCF2*	4688	neutrophil cytosolic factor 2 [Source:HGNC Symbol;Acc:HGNC:7661]	2.5	2
1530	*CA2*	760	carbonic anhydrase 2 [Source:HGNC Symbol;Acc:HGNC:1373]	2.5	2
1531	*CCNI*	10983	cyclin I [Source:HGNC Symbol;Acc:HGNC:1595]	2.5	2
1532	*SIGLEC10*	89790	sialic acid binding Ig like lectin 10 [Source:HGNC Symbol;Acc:HGNC:15620]	2.5	2
1533	*SPNS1*	83985	sphingolipid transporter 1 (putative) [Source:HGNC Symbol;Acc:HGNC:30621]	2.5	2
1534	*IPCEF1*	26034	interaction protein for cytohesin exchange factors 1 [Source:HGNC Symbol;Acc:HGNC:21204]	2.5	2
1535	*IFRD1*	3475	interferon related developmental regulator 1 [Source:HGNC Symbol;Acc:HGNC:5456]	2.5	2
1536	*ARHGAP25*	9938	Rho GTPase activating protein 25 [Source:HGNC Symbol;Acc:HGNC:28951]	2.5	2
1537	*PSME4*	23198	proteasome activator subunit 4 [Source:HGNC Symbol;Acc:HGNC:20635]	2.5	2
1538	*XRCC6*	2547	X-ray repair cross complementing 6 [Source:HGNC Symbol;Acc:HGNC:4055]	2.5	2
1539	*TNK2*	10188	tyrosine kinase non receptor 2 [Source:HGNC Symbol;Acc:HGNC:19297]	2.5	2
1540	*COPZ1*	22818	coatomer protein complex subunit zeta 1 [Source:HGNC Symbol;Acc:HGNC:2243]	2.5	2
1541	*PTTG1*	9232	pituitary tumor-transforming 1 [Source:HGNC Symbol;Acc:HGNC:9690]	2.5	2
1542	*DKK1*	22943	dickkopf WNT signaling pathway inhibitor 1 [Source:HGNC Symbol;Acc:HGNC:2891]	2.5	2
1543	*CFD*	1675	complement factor D [Source:HGNC Symbol;Acc:HGNC:2771]	2.5	2
1544	*EHD1*	10938	EH domain containing 1 [Source:HGNC Symbol;Acc:HGNC:3242]	2.5	2
1545	*ABCE1*	6059	ATP binding cassette subfamily E member 1 [Source:HGNC Symbol;Acc:HGNC:69]	2.5	2
1546	*ANAPC10*	10393	anaphase promoting complex subunit 10 [Source:HGNC Symbol;Acc:HGNC:24077]	2.5	2
1547	*FABP5*	2171	fatty acid binding protein 5 [Source:HGNC Symbol;Acc:HGNC:3560]	2.5	2
1548	*TRAF3IP3*	80342	TRAF3 interacting protein 3 [Source:HGNC Symbol;Acc:HGNC:30766]	2.5	2
1549	*PLA2G7*	7941	phospholipase A2 group VII [Source:HGNC Symbol;Acc:HGNC:9040]	2.5	2
1550	*LYST*	1130	lysosomal trafficking regulator [Source:HGNC Symbol;Acc:HGNC:1968]	2.5	2
1551	*PBXIP1*	57326	PBX homeobox interacting protein 1 [Source:HGNC Symbol;Acc:HGNC:21199]	2.5	2
1552	*SPRY4*	81848	sprouty RTK signaling antagonist 4 [Source:HGNC Symbol;Acc:HGNC:15533]	2.5	2
1553	*SLC38A9*	153129	solute carrier family 38 member 9 [Source:HGNC Symbol;Acc:HGNC:26907]	2.5	2
1554	*PGGT1B*	5229	protein geranylgeranyltransferase type I subunit beta [Source:HGNC Symbol;Acc:HGNC:8895]	2.5	2
1555	*NET1*	10276	neuroepithelial cell transforming 1 [Source:HGNC Symbol;Acc:HGNC:14592]	2.5	2
1556	*HSPA13*	6782	heat shock protein family A (Hsp70) member 13 [Source:HGNC Symbol;Acc:HGNC:11375]	2.5	2
1557	*SVBP*	374969	small vasohibin binding protein [Source:HGNC Symbol;Acc:HGNC:29204]	2.5	2
1558	*RBM26*	64062	RNA binding motif protein 26 [Source:HGNC Symbol;Acc:HGNC:20327]	2.5	2
1559	*DAAM1*	23002	dishevelled associated activator of morphogenesis 1 [Source:HGNC Symbol;Acc:HGNC:18142]	2.5	2
1560	*PIGG*	54872	phosphatidylinositol glycan anchor biosynthesis class G [Source:HGNC Symbol;Acc:HGNC:25985]	2.5	2
1561	*TUSC1*	286319	tumor suppressor candidate 1 [Source:HGNC Symbol;Acc:HGNC:31010]	2.5	2
1562	*BCL6*	604	B-cell CLL/lymphoma 6 [Source:HGNC Symbol;Acc:HGNC:1001]	2.5	2
1563	*ANKRD11*	29123	ankyrin repeat domain 11 [Source:HGNC Symbol;Acc:HGNC:21316]	2.5	2
1564	*SNX20*	124460	sorting nexin 20 [Source:HGNC Symbol;Acc:HGNC:30390]	2.5	2

1565	*NFKBIE*	4794	NFKB inhibitor epsilon [Source:HGNC Symbol;Acc:HGNC:7799]	2.5	2
1566	*SSH3*	54961	slingshot protein phosphatase 3 [Source:HGNC Symbol;Acc:HGNC:30581]	2.5	2
1567	*FHL2*	2274	four and a half LIM domains 2 [Source:HGNC Symbol;Acc:HGNC:3703]	2.5	2
1568	*OSCAR*	126014	osteoclast associated, immunoglobulin-like receptor [Source:HGNC Symbol;Acc:HGNC:29960]	2.5	2
1569	*FAM57A*	79850	family with sequence similarity 57 member A [Source:HGNC Symbol;Acc:HGNC:29646]	2.5	2
1570	*NT5C3A*	51251	5'-nucleotidase, cytosolic IIIA [Source:HGNC Symbol;Acc:HGNC:17820]	2.5	2
1571	*MFSD3*	113655	major facilitator superfamily domain containing 3 [Source:HGNC Symbol;Acc:HGNC:25157]	2.5	2
1572	*PRRG4*	79056	proline rich and Gla domain 4 [Source:HGNC Symbol;Acc:HGNC:30799]	2.5	2
1573	*HEXIM2*	124790	hexamethylene bisacetamide inducible 2 [Source:HGNC Symbol;Acc:HGNC:28591]	2.5	2
1574	*LEPROT*	54741	leptin receptor overlapping transcript [Source:HGNC Symbol;Acc:HGNC:29477]	2.5	2
1575	*KLF2*	10365	Kruppel like factor 2 [Source:HGNC Symbol;Acc:HGNC:6347]	2.5	2
1576	*GNB5*	10681	G protein subunit beta 5 [Source:HGNC Symbol;Acc:HGNC:4401]	2.5	2
1577	*CCAR1*	55749	cell division cycle and apoptosis regulator 1 [Source:HGNC Symbol;Acc:HGNC:24236]	2.5	2
1578	*C9orf89*	84270	chromosome 9 open reading frame 89	2.5	2
1579	*RAP1GAP*	5909	RAP1 GTPase activating protein [Source:HGNC Symbol;Acc:HGNC:9858]	2.5	2
1580	*C7orf26*	79034	chromosome 7 open reading frame 26 [Source:HGNC Symbol;Acc:HGNC:21702]	2.5	2
1581	*HEMGN*	55363	hemogen [Source:HGNC Symbol;Acc:HGNC:17509]	2.5	2
1582	*RNF166*	115992	ring finger protein 166 [Source:HGNC Symbol;Acc:HGNC:28856]	2.5	2
1583	*EMC7*	56851	ER membrane protein complex subunit 7 [Source:HGNC Symbol;Acc:HGNC:24301]	2.5	2
1584	*TUBB2A*	7280	tubulin beta 2A class IIa [Source:HGNC Symbol;Acc:HGNC:12412]	2.5	2
1585	*SCFD2*	152579	sec1 family domain containing 2 [Source:HGNC Symbol;Acc:HGNC:30676]	2.5	2
1586	*CDK5RAP2*	55755	CDK5 regulatory subunit associated protein 2 [Source:HGNC Symbol;Acc:HGNC:18672]	2.5	2
1587	*LMF1*	64788	lipase maturation factor 1 [Source:HGNC Symbol;Acc:HGNC:14154]	2.5	2
1588	*SHKBP1*	92799	SH3KBP1 binding protein 1 [Source:HGNC Symbol;Acc:HGNC:19214]	2.5	2
1589	*GMFG*	9535	glia maturation factor gamma [Source:HGNC Symbol;Acc:HGNC:4374]	2.5	2
1590	*PIK3R3*	8503	phosphoinositide-3-kinase regulatory subunit 3 [Source:HGNC Symbol;Acc:HGNC:8981]	2.5	2
1591	*NDUFA6*	4700	NADH:ubiquinone oxidoreductase subunit A6 [Source:HGNC Symbol;Acc:HGNC:7690]	2.5	2
1592	*SP3*	6670	Sp3 transcription factor [Source:HGNC Symbol;Acc:HGNC:11208]	2.5	2
1593	*TAF3*	83860	TATA-box binding protein associated factor 3 [Source:HGNC Symbol;Acc:HGNC:17303]	2.5	2
1594	*ELF4*	2000	E74 like ETS transcription factor 4 [Source:HGNC Symbol;Acc:HGNC:3319]	2.5	2
1595	*PECAM1*	5175	platelet and endothelial cell adhesion molecule 1 [Source:HGNC Symbol;Acc:HGNC:8823]	2.5	2
1596	*CEBPB*	1051	CCAAT/enhancer binding protein beta [Source:HGNC Symbol;Acc:HGNC:1834]	2.5	2
1597	*FBXO41*	150726	F-box protein 41 [Source:HGNC Symbol;Acc:HGNC:29409]	2.5	2
1598	*GRK5*	2869	G protein-coupled receptor kinase 5 [Source:HGNC Symbol;Acc:HGNC:4544]	2.5	2
1599	*HAUS6*	54801	HAUS augmin like complex subunit 6 [Source:HGNC Symbol;Acc:HGNC:25948]	2.5	2
1600	*GALNT3*	2591	polypeptide N-acetylgalactosaminyltransferase 3 [Source:HGNC Symbol;Acc:HGNC:4125]	2.5	2
1601	*IMPDH1*	3614	IMP (inosine 5'-monophosphate) dehydrogenase 1 [Source:HGNC Symbol;Acc:HGNC:6052]	2.5	2
1602	*KIAA1033*	23325	KIAA1033 [Source:HGNC Symbol;Acc:HGNC:29174]	2.5	2
1603	*SUMO3*	6612	small ubiquitin-like modifier 3 [Source:HGNC Symbol;Acc:HGNC:11124]	2.5	2
1604	*GOLGA3*	2802	golgin A3 [Source:HGNC Symbol;Acc:HGNC:4426]	2.5	2
1605	*NUMB*	8650	NUMB, endocytic adaptor protein [Source:HGNC Symbol;Acc:HGNC:8060]	2.5	2
1606	*RRS1*	23212	ribosome biogenesis regulator homolog [Source:HGNC Symbol;Acc:HGNC:17083]	2.5	2
1607	*ASGR2*	433	asialoglycoprotein receptor 2 [Source:HGNC Symbol;Acc:HGNC:743]	2.5	2
1608	*FIP1L1*	81608	factor interacting with PAPOLA and CPSF1 [Source:HGNC Symbol;Acc:HGNC:19124]	2.5	2
1609	*RGS19*	10287	regulator of G-protein signaling 19 [Source:HGNC Symbol;Acc:HGNC:13735]	2.5	2
1610	*FAM107B*	83641	family with sequence similarity 107 member B [Source:HGNC Symbol;Acc:HGNC:23726]	2.5	2
1611	*UBA6*	55236	ubiquitin like modifier activating enzyme 6 [Source:HGNC Symbol;Acc:HGNC:25581]	2.5	2
1612	*NCK2*	8440	NCK adaptor protein 2 [Source:HGNC Symbol;Acc:HGNC:7665]	2.5	2
1613	*TAF15*	8148	TATA-box binding protein associated factor 15 [Source:HGNC Symbol;Acc:HGNC:11547]	2.5	2
1614	*FGL1*	2267	fibrinogen like 1 [Source:HGNC Symbol;Acc:HGNC:3695]	2.5	2
1615	*IRX3*	79191	iroquois homeobox 3 [Source:HGNC Symbol;Acc:HGNC:14360]	2.5	2
1616	*TM2D1*	83941	TM2 domain containing 1 [Source:HGNC Symbol;Acc:HGNC:24142]	2.5	2
1617	*IL6ST*	3572	interleukin 6 signal transducer [Source:HGNC Symbol;Acc:HGNC:6021]	2.5	2
1618	*STK40*	83931	serine/threonine kinase 40 [Source:HGNC Symbol;Acc:HGNC:21373]	2.5	2
1619	*WBP5*	51186	WW domain binding protein 5	2.5	2
1620	*CCDC92*	80212	coiled-coil domain containing 92 [Source:HGNC Symbol;Acc:HGNC:29563]	2.5	2
1621	*SRRD*	402055	SRR1 domain containing [Source:HGNC Symbol;Acc:HGNC:33910]	2.5	2
1622	*TSPAN14*	81619	tetraspanin 14 [Source:HGNC Symbol;Acc:HGNC:23303]	2.5	2
1623	*SKA1*	220134	spindle and kinetochore associated complex subunit 1 [Source:HGNC Symbol;Acc:HGNC:28109]	2.5	2
1624	*TP53INP2*	58476	tumor protein p53 inducible nuclear protein 2 [Source:HGNC Symbol;Acc:HGNC:16104]	2.5	2
1625	*C9orf16*	79095	chromosome 9 open reading frame 16 [Source:HGNC Symbol;Acc:HGNC:17823]	2.5	2
1626	*PRAC2*	360205	prostate cancer susceptibility candidate 2 [Source:HGNC Symbol;Acc:HGNC:30143]	2.5	2
1627	*NDUFB2*	4708	NADH:ubiquinone oxidoreductase subunit B2 [Source:HGNC Symbol;Acc:HGNC:7697]	2.5	2
1628	*BIN1*	274	bridging integrator 1 [Source:HGNC Symbol;Acc:HGNC:1052]	2.5	2
1629	*SCCPDH*	51097	saccharopine dehydrogenase (putative) [Source:HGNC Symbol;Acc:HGNC:24275]	2.5	2
1630	*ALPI*	248	alkaline phosphatase, intestinal [Source:HGNC Symbol;Acc:HGNC:437]	2.5	2
1631	*FAM63A*	55793	family with sequence similarity 63 member A [Source:HGNC Symbol;Acc:HGNC:25648]	2.5	2
1632	*NAALADL2*	254827	N-acetylated alpha-linked acidic dipeptidase-like 2 [Source:HGNC Symbol;Acc:HGNC:23219]	2.4	2
1633	*CNST*	163882	consortin, connexin sorting protein [Source:HGNC Symbol;Acc:HGNC:26486]	2.4	2
1634	*CDKL1*	8814	cyclin dependent kinase like 1 [Source:HGNC Symbol;Acc:HGNC:1781]	2.4	2
1635	*EMB*	133418	embigin [Source:HGNC Symbol;Acc:HGNC:30465]	2.4	2
1636	*FAM209A*	200232	family with sequence similarity 209 member A [Source:HGNC Symbol;Acc:HGNC:16100]	2.4	2
1637	*PNMA1*	9240	paraneoplastic Ma antigen 1 [Source:HGNC Symbol;Acc:HGNC:9158]	2.4	2
1638	*MAST3*	23031	microtubule associated serine/threonine kinase 3 [Source:HGNC Symbol;Acc:HGNC:19036]	2.4	2
1639	*BOLA2*	552900	bolA family member 2 [Source:HGNC Symbol;Acc:HGNC:29488]	2.4	2
1640	*C9orf72*	203228	chromosome 9 open reading frame 72 [Source:HGNC Symbol;Acc:HGNC:28337]	2.4	2
1641	*PLPP4*	196051	phospholipid phosphatase 4 [Source:HGNC Symbol;Acc:HGNC:23531]	2.4	2
1642	*NECAP1*	25977	NECAP endocytosis associated 1 [Source:HGNC Symbol;Acc:HGNC:24539]	2.4	2

1643	*FAM134C*	162427	family with sequence similarity 134 member C [Source:HGNC Symbol;Acc:HGNC:27258]	2.4	2
1644	*TUBG1*	7283	tubulin gamma 1 [Source:HGNC Symbol;Acc:HGNC:12417]	2.4	2
1645	*MAP1S*	55201	microtubule associated protein 1S [Source:HGNC Symbol;Acc:HGNC:15715]	2.4	2
1646	*AR*	367	androgen receptor [Source:HGNC Symbol;Acc:HGNC:644]	2.4	2
1647	*TADA2B*	93624	transcriptional adaptor 2B [Source:HGNC Symbol;Acc:HGNC:30781]	2.4	2
1648	*CBS*	875	cystathionine-beta-synthase [Source:HGNC Symbol;Acc:HGNC:1550]	2.4	2
1649	*FKSG49*	400949	Hepatocellular Carcinoma-Associated Antigen HCA25b	2.4	2
1650	*NPIPB3*	23117	nuclear pore complex interacting protein family member B3 [Source:HGNC Symbol;Acc:HGNC:28989]	2.4	2
1651	*IGF2BP2*	10644	insulin like growth factor 2 mRNA binding protein 2 [Source:HGNC Symbol;Acc:HGNC:28867]	2.4	2
1652	*TPM4*	7171	tropomyosin 4 [Source:HGNC Symbol;Acc:HGNC:12013]	2.4	2
1653	*ANGPTL4*	51129	angiopoietin like 4 [Source:HGNC Symbol;Acc:HGNC:16039]	2.4	2
1654	*ARHGEF2*	9181	Rho/Rac guanine nucleotide exchange factor 2 [Source:HGNC Symbol;Acc:HGNC:682]	2.4	2
1655	*RDH11*	51109	retinol dehydrogenase 11 (all-trans/9-cis/11-cis) [Source:HGNC Symbol;Acc:HGNC:17964]	2.4	2
1656	*APP*	351	amyloid beta precursor protein [Source:HGNC Symbol;Acc:HGNC:620]	2.4	2
1657	*ATP5SL*	55101	ATP5S like [Source:HGNC Symbol;Acc:HGNC:25496]	2.4	2
1658	*AKIRIN2*	55122	akirin 2 [Source:HGNC Symbol;Acc:HGNC:21407]	2.4	2
1659	*VIL1*	7429	villin 1 [Source:HGNC Symbol;Acc:HGNC:12690]	2.4	2
1660	*TNS1*	7145	tensin 1 [Source:HGNC Symbol;Acc:HGNC:11973]	2.4	2
1661	*PRCC*	5546	papillary renal cell carcinoma (translocation-associated) [Source:HGNC Symbol;Acc:HGNC:9343]	2.4	2
1662	*MARCO*	8685	macrophage receptor with collagenous structure [Source:HGNC Symbol;Acc:HGNC:6895]	2.4	2
1663	*TUBA3C*	7278	tubulin alpha 3c [Source:HGNC Symbol;Acc:HGNC:12408]	2.4	2
1664	*FAM49A*	81553	family with sequence similarity 49 member A [Source:HGNC Symbol;Acc:HGNC:25373]	2.4	2
1665	*AKR1B1*	231	aldo-keto reductase family 1 member B [Source:HGNC Symbol;Acc:HGNC:381]	2.4	2
1666	*SSH2*	85464	slingshot protein phosphatase 2 [Source:HGNC Symbol;Acc:HGNC:30580]	2.4	2
1667	*CSF2RB*	1439	colony stimulating factor 2 receptor beta common subunit [Source:HGNC Symbol;Acc:HGNC:2436]	2.4	2
1668	*KBTBD11*	9920	kelch repeat and BTB domain containing 11 [Source:HGNC Symbol;Acc:HGNC:29104]	2.4	2
1669	*OTUD1*	220213	OTU deubiquitinase 1 [Source:HGNC Symbol;Acc:HGNC:27346]	2.4	2
1670	*CD52*	1043	CD52 molecule [Source:HGNC Symbol;Acc:HGNC:1804]	2.4	2
1671	*SF3B4*	10262	splicing factor 3b subunit 4 [Source:HGNC Symbol;Acc:HGNC:10771]	2.4	2
1672	*ACER2*	340485	alkaline ceramidase 2 [Source:HGNC Symbol;Acc:HGNC:23675]	2.4	2
1673	*EGF*	1950	epidermal growth factor [Source:HGNC Symbol;Acc:HGNC:3229]	2.4	2
1674	*CHMP6*	79643	charged multivesicular body protein 6 [Source:HGNC Symbol;Acc:HGNC:25675]	2.4	2
1675	*MND1*	84057	meiotic nuclear divisions 1 [Source:HGNC Symbol;Acc:HGNC:24839]	2.4	2
1676	*CLDN7*	1366	claudin 7 [Source:HGNC Symbol;Acc:HGNC:2049]	2.4	2
1677	*ELP5*	23587	elongator acetyltransferase complex subunit 5 [Source:HGNC Symbol;Acc:HGNC:30617]	2.4	2
1678	*SPECC1*	92521	sperm antigen with calponin homology and coiled-coil domains 1 [Source:HGNC Symbol;Acc:HGNC:30615]	2.4	2
1679	*EIF2AK1*	27102	eukaryotic translation initiation factor 2 alpha kinase 1 [Source:HGNC Symbol;Acc:HGNC:24921]	2.4	2
1680	*SEC14L1*	6397	SEC14 like lipid binding 1 [Source:HGNC Symbol;Acc:HGNC:10698]	2.4	2
1681	*SPTBN1*	6711	spectrin beta, non-erythrocytic 1 [Source:HGNC Symbol;Acc:HGNC:11275]	2.4	2
1682	*BRMS1*	25855	breast cancer metastasis suppressor 1 [Source:HGNC Symbol;Acc:HGNC:17262]	2.4	2
1683	*NLK*	51701	nemo like kinase [Source:HGNC Symbol;Acc:HGNC:29858]	2.4	2
1684	*BTBD11*	121551	BTB domain containing 11 [Source:HGNC Symbol;Acc:HGNC:23844]	2.4	2
1685	*LILRA5*	353514	leukocyte immunoglobulin like receptor A5 [Source:HGNC Symbol;Acc:HGNC:16309]	2.4	2
1686	*MBNL3*	55796	muscleblind like splicing regulator 3 [Source:HGNC Symbol;Acc:HGNC:20564]	2.4	2
1687	*HPSE*	10855	heparanase [Source:HGNC Symbol;Acc:HGNC:5164]	2.4	2
1688	*SCAF8*	22828	SR-related CTD associated factor 8 [Source:HGNC Symbol;Acc:HGNC:20959]	2.4	2
1689	*SLC25A17*	10478	solute carrier family 25 member 17 [Source:HGNC Symbol;Acc:HGNC:10987]	2.4	2
1690	*LRCH4*	4034	leucine rich repeats and calponin homology domain containing 4 [Source:HGNC Symbol;Acc:HGNC:6691]	2.4	2
1691	*ARHGEF1*	9138	Rho guanine nucleotide exchange factor 1 [Source:HGNC Symbol;Acc:HGNC:681]	2.4	2
1692	*SLC10A3*	8273	solute carrier family 10 member 3 [Source:HGNC Symbol;Acc:HGNC:22979]	2.4	2
1693	*COQ4*	51117	coenzyme Q4 [Source:HGNC Symbol;Acc:HGNC:19693]	2.4	2
1694	*CNPY3*	10695	canopy FGF signaling regulator 3 [Source:HGNC Symbol;Acc:HGNC:11968]	2.4	2
1695	*NECAP2*	55707	NECAP endocytosis associated 2 [Source:HGNC Symbol;Acc:HGNC:25528]	2.4	2
1696	*SMIM3*	85027	small integral membrane protein 3 [Source:HGNC Symbol;Acc:HGNC:30248]	2.4	2
1697	*CMPK1*	51727	cytidine/uridine monophosphate kinase 1 [Source:HGNC Symbol;Acc:HGNC:18170]	2.4	2
1698	*CFLAR*	8837	CASP8 and FADD like apoptosis regulator [Source:HGNC Symbol;Acc:HGNC:1876]	2.4	2
1699	*RBM34*	23029	RNA binding motif protein 34 [Source:HGNC Symbol;Acc:HGNC:28965]	2.4	2
1700	*PPP1R9A*	55607	protein phosphatase 1 regulatory subunit 9A [Source:HGNC Symbol;Acc:HGNC:14946]	2.4	2
1701	*ERICH1*	157697	glutamate rich 1 [Source:HGNC Symbol;Acc:HGNC:27234]	2.4	2
1702	*LMNB1*	4001	lamin B1 [Source:HGNC Symbol;Acc:HGNC:6637]	2.4	2
1703	*KLHL9*	55958	kelch like family member 9 [Source:HGNC Symbol;Acc:HGNC:18732]	2.4	2
1704	*DENND3*	22898	DENN domain containing 3 [Source:HGNC Symbol;Acc:HGNC:29134]	2.4	2
1705	*HACD4*	401494	3-hydroxyacyl-CoA dehydratase 4 [Source:HGNC Symbol;Acc:HGNC:20920]	2.4	2
1706	*PRPS2*	5634	phosphoribosyl pyrophosphate synthetase 2 [Source:HGNC Symbol;Acc:HGNC:9465]	2.4	2
1707	*DHX38*	9785	DEAH-box helicase 38 [Source:HGNC Symbol;Acc:HGNC:17211]	2.4	2
1708	*ANKRD44*	91526	ankyrin repeat domain 44 [Source:HGNC Symbol;Acc:HGNC:25259]	2.4	2
1709	*HLA-DOA*	3111	major histocompatibility complex, class II, DO alpha [Source:HGNC Symbol;Acc:HGNC:4936]	2.4	2
1710	*TMCC3*	57458	transmembrane and coiled-coil domain family 3 [Source:HGNC Symbol;Acc:HGNC:29199]	2.4	2
1711	*MATN2*	4147	matrilin 2 [Source:HGNC Symbol;Acc:HGNC:6908]	2.4	2
1712	*PIGK*	10026	phosphatidylinositol glycan anchor biosynthesis class K [Source:HGNC Symbol;Acc:HGNC:8965]	2.4	2
1713	*NIPAL1*	152519	NIPA like domain containing 1 [Source:HGNC Symbol;Acc:HGNC:27194]	2.4	2
1714	*RNF144B*	255488	ring finger protein 144B [Source:HGNC Symbol;Acc:HGNC:21578]	2.4	2
1715	*GDAP2*	54834	ganglioside induced differentiation associated protein 2 [Source:HGNC Symbol;Acc:HGNC:18010]	2.4	2
1716	*E2F3*	1871	E2F transcription factor 3 [Source:HGNC Symbol;Acc:HGNC:3115]	2.4	2

1717	*ZZEF1*	23140	zinc finger ZZ-type and EF-hand domain containing 1 [Source:HGNC Symbol;Acc:HGNC:29027]	2.4	2
1718	*TRIP13*	9319	thyroid hormone receptor interactor 13 [Source:HGNC Symbol;Acc:HGNC:12307]	2.4	2
1719	*RNF149*	284996	ring finger protein 149 [Source:HGNC Symbol;Acc:HGNC:23137]	2.4	2
1720	*ARHGAP21*	57584	Rho GTPase activating protein 21 [Source:HGNC Symbol;Acc:HGNC:23725]	2.4	2
1721	*GCA*	25801	grancalcin [Source:HGNC Symbol;Acc:HGNC:15990]	2.4	2
1722	*RASAL3*	64926	RAS protein activator like 3 [Source:HGNC Symbol;Acc:HGNC:26129]	2.4	2
1723	*TPGS1*	91978	tubulin polyglutamylase complex subunit 1 [Source:HGNC Symbol;Acc:HGNC:25058]	2.4	2
1724	*FAM26F*	441168	family with sequence similarity 26 member F [Source:HGNC Symbol;Acc:HGNC:33391]	2.4	2
1725	*OSBP2*	23762	oxysterol binding protein 2 [Source:HGNC Symbol;Acc:HGNC:8504]	2.4	2
1726	*SEPT8*	23176	septin 8 [Source:HGNC Symbol;Acc:HGNC:16511]	2.4	2
1727	*GNG8*	94235	G protein subunit gamma 8 [Source:HGNC Symbol;Acc:HGNC:19664]	2.4	2
1728	*CENPU*	79682	centromere protein U [Source:HGNC Symbol;Acc:HGNC:21348]	2.4	2
1729	*PRIMPOL*	201973	primase and DNA directed polymerase [Source:HGNC Symbol;Acc:HGNC:26575]	2.4	2
1730	*NENF*	29937	neudesin neurotrophic factor [Source:HGNC Symbol;Acc:HGNC:30384]	2.4	2
1731	*WHAMM*	123720	WAS protein homolog associated with actin, golgi membranes and microtubules [Source:HGNC Symbol;Acc:HGNC:30493]	2.4	2
1732	*SPN*	6693	sialophorin [Source:HGNC Symbol;Acc:HGNC:11249]	2.4	2
1733	*EEF1D*	1936	eukaryotic translation elongation factor 1 delta [Source:HGNC Symbol;Acc:HGNC:3211]	2.4	2
1734	*FANCL*	55120	Fanconi anemia complementation group L [Source:HGNC Symbol;Acc:HGNC:20748]	2.4	2
1735	*ELOVL5*	60481	ELOVL fatty acid elongase 5 [Source:HGNC Symbol;Acc:HGNC:21308]	2.4	2
1736	*DGKD*	8527	diacylglycerol kinase delta [Source:HGNC Symbol;Acc:HGNC:2851]	2.4	2
1737	*ANKRD36B*	57730	ankyrin repeat domain 36B [Source:HGNC Symbol;Acc:HGNC:29333]	2.4	2
1738	*FLNA*	2316	filamin A [Source:HGNC Symbol;Acc:HGNC:3754]	2.4	2
1739	*CPNE8*	144402	copine 8 [Source:HGNC Symbol;Acc:HGNC:23498]	2.4	2
1740	*RBMX*	27316	RNA binding motif protein, X-linked [Source:HGNC Symbol;Acc:HGNC:9910]	2.3	2
1741	*ENKUR*	219670	enkurin, TRPC channel interacting protein [Source:HGNC Symbol;Acc:HGNC:28388]	2.3	2
1742	*FCRLA*	84824	Fc receptor like A [Source:HGNC Symbol;Acc:HGNC:18504]	2.3	2
1743	*SH2D3C*	10044	SH2 domain containing 3C [Source:HGNC Symbol;Acc:HGNC:16884]	2.3	2
1744	*SLC24A3*	57419	solute carrier family 24 member 3 [Source:HGNC Symbol;Acc:HGNC:10977]	2.3	2
1745	*TNNC2*	7125	troponin C2, fast skeletal type [Source:HGNC Symbol;Acc:HGNC:11944]	2.3	2
1746	*SOS1*	6654	SOS Ras/Rac guanine nucleotide exchange factor 1 [Source:HGNC Symbol;Acc:HGNC:11187]	2.3	2
1747	*BCR*	613	BCR, RhoGEF and GTPase activating protein [Source:HGNC Symbol;Acc:HGNC:1014]	2.3	2
1748	*YWHAH*	7533	tyrosine 3-monooxygenase/tryptophan 5-monooxygenase activation protein eta [Source:HGNC Symbol;Acc:HGNC:12853]	2.3	2
1749	*MBOAT7*	79143	membrane bound O-acyltransferase domain containing 7 [Source:HGNC Symbol;Acc:HGNC:15505]	2.3	2
1750	*FAM78A*	286336	family with sequence similarity 78 member A [Source:HGNC Symbol;Acc:HGNC:25465]	2.3	2
1751	*GPR65*	8477	G protein-coupled receptor 65 [Source:HGNC Symbol;Acc:HGNC:4517]	2.3	2
1752	*ANKRD13D*	338692	ankyrin repeat domain 13D [Source:HGNC Symbol;Acc:HGNC:27880]	2.3	2
1753	*GATAD2A*	54815	GATA zinc finger domain containing 2A [Source:HGNC Symbol;Acc:HGNC:29989]	2.3	2
1754	*RSL1D1*	26156	ribosomal L1 domain containing 1 [Source:HGNC Symbol;Acc:HGNC:24534]	2.3	2
1755	*SIGLEC7*	27036	sialic acid binding Ig like lectin 7 [Source:HGNC Symbol;Acc:HGNC:10876]	2.3	2
1756	*SMDT1*	91689	single-pass membrane protein with aspartate rich tail 1 [Source:HGNC Symbol;Acc:HGNC:25055]	2.3	2
1757	*IRF5*	3663	interferon regulatory factor 5 [Source:HGNC Symbol;Acc:HGNC:6120]	2.3	2
1758	*MGRN1*	23295	mahogunin ring finger 1 [Source:HGNC Symbol;Acc:HGNC:20254]	2.3	2
1759	*RAP2B*	5912	RAP2B, member of RAS oncogene family [Source:HGNC Symbol;Acc:HGNC:9862]	2.3	2
1760	*CLN8*	2055	ceroid-lipofuscinosis, neuronal 8 [Source:HGNC Symbol;Acc:HGNC:2079]	2.3	2
1761	*PHYKPL*	85007	5-phosphohydroxy-L-lysine phospho-lyase [Source:HGNC Symbol;Acc:HGNC:28249]	2.3	2
1762	*CACYBP*	27101	calcyclin binding protein [Source:HGNC Symbol;Acc:HGNC:30423]	2.3	2
1763	*TNFAIP8L2*	79626	TNF alpha induced protein 8 like 2 [Source:HGNC Symbol;Acc:HGNC:26277]	2.3	2
1764	*CBL*	867	Cbl proto-oncogene [Source:HGNC Symbol;Acc:HGNC:1541]	2.3	2
1765	*ZNF133*	7692	zinc finger protein 133 [Source:HGNC Symbol;Acc:HGNC:12917]	2.3	2
1766	*NLRP12*	91662	NLR family pyrin domain containing 12 [Source:HGNC Symbol;Acc:HGNC:22938]	2.3	2
1767	*BTF3L4*	91408	basic transcription factor 3-like 4 [Source:HGNC Symbol;Acc:HGNC:30547]	2.3	2
1768	*PTPN21*	11099	protein tyrosine phosphatase, non-receptor type 21 [Source:HGNC Symbol;Acc:HGNC:9651]	2.3	2
1769	*C19orf43*	79002	chromosome 19 open reading frame 43 [Source:HGNC Symbol;Acc:HGNC:28424]	2.3	2
1770	*TRAF1*	7185	TNF receptor associated factor 1 [Source:HGNC Symbol;Acc:HGNC:12031]	2.3	2
1771	*CCDC88A*	55704	coiled-coil domain containing 88A [Source:HGNC Symbol;Acc:HGNC:25523]	2.3	2
1772	*MBP*	4155	myelin basic protein [Source:HGNC Symbol;Acc:HGNC:6925]	2.3	2
1773	*MTPAP*	55149	mitochondrial poly(A) polymerase [Source:HGNC Symbol;Acc:HGNC:25532]	2.3	2
1774	*HINT3*	135114	histidine triad nucleotide binding protein 3 [Source:HGNC Symbol;Acc:HGNC:18468]	2.3	2
1775	*TNFAIP8L1*	126282	TNF alpha induced protein 8 like 1 [Source:HGNC Symbol;Acc:HGNC:28279]	2.3	2
1776	*RSU1*	6251	Ras suppressor protein 1 [Source:HGNC Symbol;Acc:HGNC:10464]	2.3	2
1777	*PFKFB4*	5210	6-phosphofructo-2-kinase/fructose-2,6-biphosphatase 4 [Source:HGNC Symbol;Acc:HGNC:8875]	2.3	2
1778	*MCM4*	4173	minichromosome maintenance complex component 4 [Source:HGNC Symbol;Acc:HGNC:6947]	2.3	2
1779	*GPR27*	2850	G protein-coupled receptor 27 [Source:HGNC Symbol;Acc:HGNC:4482]	2.3	2
1780	*NAE1*	8883	NEDD8 activating enzyme E1 subunit 1 [Source:HGNC Symbol;Acc:HGNC:621]	2.3	2
1781	*RAB27A*	5873	RAB27A, member RAS oncogene family [Source:HGNC Symbol;Acc:HGNC:9766]	2.3	2
1782	*MESDC1*	59274	mesoderm development candidate 1 [Source:HGNC Symbol;Acc:HGNC:13519]	2.3	2
1783	*PEBP1*	5037	phosphatidylethanolamine binding protein 1 [Source:HGNC Symbol;Acc:HGNC:8630]	2.3	2
1784	*TUBB6*	84617	tubulin beta 6 class V [Source:HGNC Symbol;Acc:HGNC:20776]	2.3	2
1785	*AGR3*	155465	anterior gradient 3, protein disulphide isomerase family member [Source:HGNC Symbol;Acc:HGNC:24167]	2.3	2
1786	*CRAT*	1384	carnitine O-acetyltransferase [Source:HGNC Symbol;Acc:HGNC:2342]	2.3	2
1787	*PPP1R12A*	4659	protein phosphatase 1 regulatory subunit 12A [Source:HGNC Symbol;Acc:HGNC:7618]	2.3	2
1788	*APOPT1*	84334	apoptogenic 1, mitochondrial [Source:HGNC Symbol;Acc:HGNC:20492]	2.3	2
1789	*C14orf142*	84520	chromosome 14 open reading frame 142 [Source:HGNC Symbol;Acc:HGNC:20356]	2.3	2

1790	*UBR7*	55148	ubiquitin protein ligase E3 component n-recognin 7 (putative) [Source:HGNC Symbol;Acc:HGNC:20344]	2.3	2
1791	*ADGRE5*	976	adhesion G protein-coupled receptor E5 [Source:HGNC Symbol;Acc:HGNC:1711]	2.3	2
1792	*TAGLN2*	8407	transgelin 2 [Source:HGNC Symbol;Acc:HGNC:11554]	2.3	2
1793	*FAAH*	2166	fatty acid amide hydrolase [Source:HGNC Symbol;Acc:HGNC:3553]	2.3	2
1794	*MAP4K2*	5871	mitogen-activated protein kinase kinase kinase kinase 2 [Source:HGNC Symbol;Acc:HGNC:6864]	2.3	2
1795	*CSK*	1445	c-src tyrosine kinase [Source:HGNC Symbol;Acc:HGNC:2444]	2.3	2
1796	*SPEN*	23013	spen family transcriptional repressor [Source:HGNC Symbol;Acc:HGNC:17575]	2.3	2
1797	*POLR3C*	10623	polymerase (RNA) III subunit C [Source:HGNC Symbol;Acc:HGNC:30076]	2.3	2
1798	*CUX1*	1523	cut like homeobox 1 [Source:HGNC Symbol;Acc:HGNC:2557]	2.3	2
1799	*LIMS3*	96626	LIM zinc finger domain containing 3 [Source:HGNC Symbol;Acc:HGNC:30047]	2.3	2
1800	*FBXW2*	26190	F-box and WD repeat domain containing 2 [Source:HGNC Symbol;Acc:HGNC:13608]	2.3	2
1801	*CD48*	962	CD48 molecule [Source:HGNC Symbol;Acc:HGNC:1683]	2.3	2
1802	*STARD8*	9754	StAR related lipid transfer domain containing 8 [Source:HGNC Symbol;Acc:HGNC:19161]	2.3	2
1803	*FGL2*	10875	fibrinogen like 2 [Source:HGNC Symbol;Acc:HGNC:3696]	2.3	2
1804	*BMPR2*	659	bone morphogenetic protein receptor type 2 [Source:HGNC Symbol;Acc:HGNC:1078]	2.3	2
1805	*RAB27B*	5874	RAB27B, member RAS oncogene family [Source:HGNC Symbol;Acc:HGNC:9767]	2.3	2
1806	*IKBKG*	8517	inhibitor of kappa light polypeptide gene enhancer in B-cells, kinase gamma [Source:HGNC Symbol;Acc:HGNC:5961]	2.3	2
1807	*BORCS8*	729991	BLOC-1 related complex subunit 8 [Source:HGNC Symbol;Acc:HGNC:37247]	2.3	2
1808	*MSANTD3*	91283	Myb/SANT DNA binding domain containing 3 [Source:HGNC Symbol;Acc:HGNC:23370]	2.2	2
1809	*ITIH2*	3698	inter-alpha-trypsin inhibitor heavy chain 2 [Source:HGNC Symbol;Acc:HGNC:6167]	2.2	2
1810	*EPS15L1*	58513	epidermal growth factor receptor pathway substrate 15 like 1 [Source:HGNC Symbol;Acc:HGNC:24634]	2.2	2
1811	*PBLD*	64081	phenazine biosynthesis like protein domain containing [Source:HGNC Symbol;Acc:HGNC:23301]	2.2	2
1812	*CREB5*	9586	cAMP responsive element binding protein 5 [Source:HGNC Symbol;Acc:HGNC:16844]	2.2	2
1813	*CD93*	22918	CD93 molecule [Source:HGNC Symbol;Acc:HGNC:15855]	2.2	2
1814	*KIAA1841*	84542	KIAA1841 [Source:HGNC Symbol;Acc:HGNC:29387]	2.2	2
1815	*RBBP7*	5931	RB binding protein 7, chromatin remodeling factor [Source:HGNC Symbol;Acc:HGNC:9890]	2.2	2
1816	*DUSP22*	56940	dual specificity phosphatase 22 [Source:HGNC Symbol;Acc:HGNC:16077]	2.2	2
1817	*ELMO1*	9844	engulfment and cell motility 1 [Source:HGNC Symbol;Acc:HGNC:16286]	2.2	2
1818	*SEPT6*	23157	septin 6 [Source:HGNC Symbol;Acc:HGNC:15848]	2.2	2
1819	*TCIRG1*	10312	T-cell immune regulator 1, ATPase H+ transporting V0 subunit a3 [Source:HGNC Symbol;Acc:HGNC:11647]	2.2	2
1820	*ASCL2*	430	achaete-scute family bHLH transcription factor 2 [Source:HGNC Symbol;Acc:HGNC:739]	2.2	2
1821	*KIAA1551*	55196	KIAA1551 [Source:HGNC Symbol;Acc:HGNC:25559]	2.2	2
1822	*SH3BP5*	9467	SH3 domain binding protein 5 [Source:HGNC Symbol;Acc:HGNC:10827]	2.2	2
1823	*UBE2J1*	51465	ubiquitin conjugating enzyme E2 J1 [Source:HGNC Symbol;Acc:HGNC:17598]	2.2	2
1824	*EMC9*	51016	ER membrane protein complex subunit 9 [Source:HGNC Symbol;Acc:HGNC:20273]	2.2	2
1825	*PSME1*	5720	proteasome activator subunit 1 [Source:HGNC Symbol;Acc:HGNC:9568]	2.2	2
1826	*AMMECR1*	9949	Alport syndrome, mental retardation, midface hypoplasia and elliptocytosis chromosomal region gene 1 [Source:HGNC Symbol;Acc:HGNC:467]	2.2	2
1827	*TRANK1*	9881	tetratricopeptide repeat and ankyrin repeat containing 1 [Source:HGNC Symbol;Acc:HGNC:29011]	2.2	2
1828	*ETFA*	2108	electron transfer flavoprotein alpha subunit [Source:HGNC Symbol;Acc:HGNC:3481]	2.2	2
1829	*OAS2*	4939	2'-5'-oligoadenylate synthetase 2 [Source:HGNC Symbol;Acc:HGNC:8087]	2.2	2
1830	*MTCH2*	23788	mitochondrial carrier 2 [Source:HGNC Symbol;Acc:HGNC:17587]	2.2	2
1831	*RPS11*	6205	ribosomal protein S11 [Source:HGNC Symbol;Acc:HGNC:10384]	2.2	2
1832	*FGFR1*	2260	fibroblast growth factor receptor 1 [Source:HGNC Symbol;Acc:HGNC:3688]	2.2	2
1833	*NFKBIZ*	64332	NFKB inhibitor zeta [Source:HGNC Symbol;Acc:HGNC:29805]	2.2	2
1834	*GAS6*	2621	growth arrest specific 6 [Source:HGNC Symbol;Acc:HGNC:4168]	2.2	2
1835	*WWP2*	11060	WW domain containing E3 ubiquitin protein ligase 2 [Source:HGNC Symbol;Acc:HGNC:16804]	2.2	2
1836	*ABHD12*	26090	abhydrolase domain containing 12 [Source:HGNC Symbol;Acc:HGNC:15868]	2.2	2
1837	*GAK*	2580	cyclin G associated kinase [Source:HGNC Symbol;Acc:HGNC:4113]	2.2	2
1838	*MAP1A*	4130	microtubule associated protein 1A [Source:HGNC Symbol;Acc:HGNC:6835]	2.2	2
1839	*RNF11*	26994	ring finger protein 11 [Source:HGNC Symbol;Acc:HGNC:10056]	2.2	2
1840	*SPATA24*	202051	spermatogenesis associated 24 [Source:HGNC Symbol;Acc:HGNC:27322]	2.2	2
1841	*RHBDD1*	84236	rhomboid domain containing 1 [Source:HGNC Symbol;Acc:HGNC:23081]	2.2	2
1842	*TMED8*	283578	transmembrane p24 trafficking protein family member 8 [Source:HGNC Symbol;Acc:HGNC:18633]	2.2	2
1843	*ZCCHC6*	79670	zinc finger CCHC-type containing 6 [Source:HGNC Symbol;Acc:HGNC:25817]	2.2	2
1844	*CD55*	1604	CD55 molecule (Cromer blood group) [Source:HGNC Symbol;Acc:HGNC:2665]	2.2	2
1845	*GPS2*	2874	G protein pathway suppressor 2 [Source:HGNC Symbol;Acc:HGNC:4550]	2.2	2
1846	*SLC12A2*	6558	solute carrier family 12 member 2 [Source:HGNC Symbol;Acc:HGNC:10911]	2.2	2
1847	*ZNF75D*	7626	zinc finger protein 75D [Source:HGNC Symbol;Acc:HGNC:13145]	2.2	2
1848	*ZGPAT*	84619	zinc finger CCCH-type and G-patch domain containing [Source:HGNC Symbol;Acc:HGNC:15948]	2.2	2
1849	*FKBP5*	2289	FK506 binding protein 5 [Source:HGNC Symbol;Acc:HGNC:3721]	2.2	2
1850	*SEC24C*	9632	SEC24 homolog C, COPII coat complex component [Source:HGNC Symbol;Acc:HGNC:10705]	2.2	2
1851	*PHF21A*	51317	PHD finger protein 21A [Source:HGNC Symbol;Acc:HGNC:24156]	2.2	2
1852	*LYRM2*	57226	LYR motif containing 2 [Source:HGNC Symbol;Acc:HGNC:25229]	2.2	2
1853	*MVP*	9961	major vault protein [Source:HGNC Symbol;Acc:HGNC:7531]	2.2	2
1854	*PAGR1*	79447	PAXIP1 associated glutamate rich protein 1 [Source:HGNC Symbol;Acc:HGNC:28707]	2.2	2
1855	*MAT2B*	27430	methionine adenosyltransferase 2B [Source:HGNC Symbol;Acc:HGNC:6905]	2.2	2
1856	*RNF44*	22838	ring finger protein 44 [Source:HGNC Symbol;Acc:HGNC:19180]	2.2	2
1857	*NBPF10*	100132406	neuroblastoma breakpoint family member 10 [Source:HGNC Symbol;Acc:HGNC:31992]	2.2	2
1858	*PSMD10*	5716	proteasome 26S subunit, non-ATPase 10 [Source:HGNC Symbol;Acc:HGNC:9555]	2.2	2
1859	*FNBP1L*	54874	formin binding protein 1 like [Source:HGNC Symbol;Acc:HGNC:20851]	2.2	2
1860	*PARP9*	83666	poly(ADP-ribose) polymerase family member 9 [Source:HGNC Symbol;Acc:HGNC:24118]	2.2	2

1861	*FCRL5*	83416	Fc receptor like 5 [Source:HGNC Symbol;Acc:HGNC:18508]	2.2	2
1862	*ASS1*	445	argininosuccinate synthase 1 [Source:HGNC Symbol;Acc:HGNC:758]	2.2	2
1863	*CKLF*	51192	chemokine-like factor [Source:HGNC Symbol;Acc:HGNC:13253]	2.2	2
1864	*CKLF-CMTM1*	100529251	CKLF-CMTM1 readthrough [Source:HGNC Symbol;Acc:HGNC:39977]	2.2	2
1865	*CMTM1*	113540	CKLF like MARVEL transmembrane domain containing 1 [Source:HGNC Symbol;Acc:HGNC:19172]	2.2	2
1866	*H2AFY*	9555	H2A histone family member Y [Source:HGNC Symbol;Acc:HGNC:4740]	2.2	2
1867	*HES6*	55502	hes family bHLH transcription factor 6 [Source:HGNC Symbol;Acc:HGNC:18254]	2.2	2
1868	*CACNA2D4*	93589	calcium voltage-gated channel auxiliary subunit alpha2delta 4 [Source:HGNC Symbol;Acc:HGNC:20202]	2.2	2
1869	*HYI*	81888	hydroxypyruvate isomerase (putative) [Source:HGNC Symbol;Acc:HGNC:26948]	2.2	2
1870	*AIF1*	199	allograft inflammatory factor 1 [Source:HGNC Symbol;Acc:HGNC:352]	2.2	2
1871	*PSMB4*	5692	proteasome subunit beta 4 [Source:HGNC Symbol;Acc:HGNC:9541]	2.2	2
1872	*RNF182*	221687	ring finger protein 182 [Source:HGNC Symbol;Acc:HGNC:28522]	2.2	2
1873	*TMEM222*	84065	transmembrane protein 222 [Source:HGNC Symbol;Acc:HGNC:25363]	2.2	2
1874	*LRP1*	4035	LDL receptor related protein 1 [Source:HGNC Symbol;Acc:HGNC:6692]	2.2	2
1875	*NINJ1*	4814	ninjurin 1 [Source:HGNC Symbol;Acc:HGNC:7824]	2.2	2
1876	*FGF13*	2258	fibroblast growth factor 13 [Source:HGNC Symbol;Acc:HGNC:3670]	2.2	2
1877	*ICAM2*	3384	intercellular adhesion molecule 2 [Source:HGNC Symbol;Acc:HGNC:5345]	2.2	2
1878	*LRG1*	116844	leucine rich alpha-2-glycoprotein 1 [Source:HGNC Symbol;Acc:HGNC:29480]	2.2	2
1879	*ZMIZ2*	83637	zinc finger MIZ-type containing 2 [Source:HGNC Symbol;Acc:HGNC:22229]	2.2	2
1880	*UBA7*	7318	ubiquitin like modifier activating enzyme 7 [Source:HGNC Symbol;Acc:HGNC:12471]	2.2	2
1881	*RPS4Y2*	140032	ribosomal protein S4, Y-linked 2 [Source:HGNC Symbol;Acc:HGNC:18501]	2.2	2
1882	*UBL4A*	8266	ubiquitin like 4A [Source:HGNC Symbol;Acc:HGNC:12505]	2.2	2
1883	*SUB1*	10923	SUB1 homolog, transcriptional regulator [Source:HGNC Symbol;Acc:HGNC:19985]	2.2	2
1884	*FCER1G*	2207	Fc fragment of IgE receptor Ig [Source:HGNC Symbol;Acc:HGNC:3611]	2.2	2
1885	*NAIP*	4671	NLR family apoptosis inhibitory protein [Source:HGNC Symbol;Acc:HGNC:7634]	2.2	2
1886	*MAPK7*	5598	mitogen-activated protein kinase 7 [Source:HGNC Symbol;Acc:HGNC:6880]	2.2	2
1887	*WDR1*	9948	WD repeat domain 1 [Source:HGNC Symbol;Acc:HGNC:12754]	2.2	2
1888	*STMN1*	3925	stathmin 1 [Source:HGNC Symbol;Acc:HGNC:6510]	2.2	2
1889	*COMMD10*	51397	COMM domain containing 10 [Source:HGNC Symbol;Acc:HGNC:30201]	2.2	2
1890	*PTK2*	5747	protein tyrosine kinase 2 [Source:HGNC Symbol;Acc:HGNC:9611]	2.2	2
1891	*CASC5*	57082	cancer susceptibility candidate 5 [Source:HGNC Symbol;Acc:HGNC:24054]	2.2	2
1892	*NOD2*	64127	nucleotide binding oligomerization domain containing 2 [Source:HGNC Symbol;Acc:HGNC:5331]	2.2	2
1893	*S100A10*	6281	S100 calcium binding protein A10 [Source:HGNC Symbol;Acc:HGNC:10487]	2.2	2
1894	*YWHAZ*	7534	tyrosine 3-monooxygenase/tryptophan 5-monooxygenase activation protein zeta [Source:HGNC Symbol;Acc:HGNC:12855]	2.2	2
1895	*BEND7*	222389	BEN domain containing 7 [Source:HGNC Symbol;Acc:HGNC:23514]	2.2	2
1896	*AGTPBP1*	23287	ATP/GTP binding protein 1 [Source:HGNC Symbol;Acc:HGNC:17258]	2.2	2
1897	*SKA2*	348235	spindle and kinetochore associated complex subunit 2 [Source:HGNC Symbol;Acc:HGNC:28006]	2.2	2
1898	*CX3CR1*	1524	C-X3-C motif chemokine receptor 1 [Source:HGNC Symbol;Acc:HGNC:2558]	2.2	2
1899	*PYGB*	5834	phosphorylase, glycogen; brain [Source:HGNC Symbol;Acc:HGNC:9723]	2.2	2
1900	*FDFT1*	2222	farnesyl-diphosphate farnesyltransferase 1 [Source:HGNC Symbol;Acc:HGNC:3629]	2.2	2
1901	*MYCT1*	80177	myc target 1 [Source:HGNC Symbol;Acc:HGNC:23172]	2.2	2
1902	*PPP1R14A*	94274	protein phosphatase 1 regulatory inhibitor subunit 14A [Source:HGNC Symbol;Acc:HGNC:14871]	2.2	2
1903	*GPCPD1*	56261	glycerophosphocholine phosphodiesterase 1 [Source:HGNC Symbol;Acc:HGNC:26957]	2.2	2
1904	*RAB32*	10981	RAB32, member RAS oncogene family [Source:HGNC Symbol;Acc:HGNC:9772]	2.2	2
1905	*CAMKK1*	84254	calcium/calmodulin dependent protein kinase kinase 1 [Source:HGNC Symbol;Acc:HGNC:1469]	2.2	2
1906	*EFHD2*	79180	EF-hand domain family member D2 [Source:HGNC Symbol;Acc:HGNC:28670]	2.2	2
1907	*DDIT3*	1649	DNA damage inducible transcript 3 [Source:HGNC Symbol;Acc:HGNC:2726]	2.2	2
1908	*GSDMD*	79792	gasdermin D [Source:HGNC Symbol;Acc:HGNC:25697]	2.2	2
1909	*RILPL2*	196383	Rab interacting lysosomal protein like 2 [Source:HGNC Symbol;Acc:HGNC:28787]	2.2	2
1910	*NUP43*	348995	nucleoporin 43 [Source:HGNC Symbol;Acc:HGNC:21182]	2.2	2
1911	*PRKRIP1*	79706	PRKR interacting protein 1 (IL11 inducible) [Source:HGNC Symbol;Acc:HGNC:21894]	2.1	2
1912	*MCTS1*	28985	malignant T-cell amplified sequence 1 [Source:HGNC Symbol;Acc:HGNC:23357]	2.1	2
1913	*NAAA*	27163	N-acylethanolamine acid amidase [Source:HGNC Symbol;Acc:HGNC:736]	2.1	2
1914	*ARMCX6*	54470	armadillo repeat containing, X-linked 6 [Source:HGNC Symbol;Acc:HGNC:26094]	2.1	2
1915	*COQ7*	10229	coenzyme Q7, hydroxylase [Source:HGNC Symbol;Acc:HGNC:2244]	2.1	2
1916	*MADD*	8567	MAP kinase activating death domain [Source:HGNC Symbol;Acc:HGNC:6766]	2.1	2
1917	*CCDC59*	29080	coiled-coil domain containing 59 [Source:HGNC Symbol;Acc:HGNC:25005]	2.1	2
1918	*METTL25*	84190	methyltransferase like 25 [Source:HGNC Symbol;Acc:HGNC:26228]	2.1	2
1919	*LAMTOR3*	8649	late endosomal/lysosomal adaptor, MAPK and MTOR activator 3 [Source:HGNC Symbol;Acc:HGNC:15606]	2.1	2
1920	*ADAP2*	55803	ArfGAP with dual PH domains 2 [Source:HGNC Symbol;Acc:HGNC:16487]	2.1	2
1921	*CLDND1*	56650	claudin domain containing 1 [Source:HGNC Symbol;Acc:HGNC:1322]	2.1	2
1922	*SMCHD1*	23347	structural maintenance of chromosomes flexible hinge domain containing 1 [Source:HGNC Symbol;Acc:HGNC:29090]	2.1	2
1923	*HCLS1*	3059	hematopoietic cell-specific Lyn substrate 1 [Source:HGNC Symbol;Acc:HGNC:4844]	2.1	2
1924	*IL15*	3600	interleukin 15 [Source:HGNC Symbol;Acc:HGNC:5977]	2.1	2
1925	*NAGPA*	51172	N-acetylglucosamine-1-phosphodiester alpha-N-acetylglucosaminidase [Source:HGNC Symbol;Acc:HGNC:17378]	2.1	2
1926	*TIMM13*	26517	translocase of inner mitochondrial membrane 13 [Source:HGNC Symbol;Acc:HGNC:11816]	2.1	2
1927	*TMPRSS9*	360200	transmembrane protease, serine 9 [Source:HGNC Symbol;Acc:HGNC:30079]	2.1	2
1928	*C2CD2L*	9854	C2CD2 like [Source:HGNC Symbol;Acc:HGNC:29000]	2.1	2
1929	*ORC5*	5001	origin recognition complex subunit 5 [Source:HGNC Symbol;Acc:HGNC:8491]	2.1	2
1930	*POU2F2*	5452	POU class 2 homeobox 2 [Source:HGNC Symbol;Acc:HGNC:9213]	2.1	2
1931	*SPIDR*	23514	scaffolding protein involved in DNA repair [Source:HGNC Symbol;Acc:HGNC:28971]	2.1	2
1932	*PTMA*	5757	prothymosin, alpha [Source:HGNC Symbol;Acc:HGNC:9623]	2.1	2
1933	*ZSCAN29*	146050	zinc finger and SCAN domain containing 29 [Source:HGNC Symbol;Acc:HGNC:26673]	2.1	2

1934	*IFT74*	80173	intraflagellar transport 74 [Source:HGNC Symbol;Acc:HGNC:21424]	2.1	2
1935	*EPOR*	2057	erythropoietin receptor [Source:HGNC Symbol;Acc:HGNC:3416]	2.1	2
1936	*NGDN*	25983	neuroguidin [Source:HGNC Symbol;Acc:HGNC:20271]	2.1	2
1937	*MX2*	4600	MX dynamin like GTPase 2 [Source:HGNC Symbol;Acc:HGNC:7533]	2.1	2
1938	*CDC42EP3*	10602	CDC42 effector protein 3 [Source:HGNC Symbol;Acc:HGNC:16943]	2.1	2
1939	*LAMB1*	3912	laminin subunit beta 1 [Source:HGNC Symbol;Acc:HGNC:6486]	2.1	2
1940	*CSF1R*	1436	colony stimulating factor 1 receptor [Source:HGNC Symbol;Acc:HGNC:2433]	2.1	2
1941	*F11R*	50848	F11 receptor [Source:HGNC Symbol;Acc:HGNC:14685]	2.1	2
1942	*ZNF548*	147694	zinc finger protein 548 [Source:HGNC Symbol;Acc:HGNC:26561]	2.1	2
1943	*RAB12*	201475	RAB12, member RAS oncogene family [Source:HGNC Symbol;Acc:HGNC:31332]	2.1	2
1944	*GTPBP2*	54676	GTP binding protein 2 [Source:HGNC Symbol;Acc:HGNC:4670]	2.1	2
1945	*GARS*	2617	glycyl-tRNA synthetase [Source:HGNC Symbol;Acc:HGNC:4162]	2.1	2
1946	*CLEC2D*	29121	C-type lectin domain family 2 member D [Source:HGNC Symbol;Acc:HGNC:14351]	2.1	2
1947	*ZRSR2*	8233	zinc finger CCCH-type, RNA binding motif and serine/arginine rich 2 [Source:HGNC Symbol;Acc:HGNC:23019]	2.1	2
1948	*MT-ATP6*	4508	mitochondrially encoded ATP synthase 6 [Source:HGNC Symbol;Acc:HGNC:7414]	2.1	2
1949	*ME2*	4200	malic enzyme 2 [Source:HGNC Symbol;Acc:HGNC:6984]	2.1	2
1950	*AGTRAP*	57085	angiotensin II receptor associated protein [Source:HGNC Symbol;Acc:HGNC:13539]	2.1	2
1951	*DPPA4*	55211	developmental pluripotency associated 4 [Source:HGNC Symbol;Acc:HGNC:19200]	2.1	2
1952	*TMC6*	11322	transmembrane channel like 6 [Source:HGNC Symbol;Acc:HGNC:18021]	2.1	2
1953	*DENND4B*	9909	DENN domain containing 4B [Source:HGNC Symbol;Acc:HGNC:29044]	2.1	2
1954	*TMPO*	7112	thymopoietin [Source:HGNC Symbol;Acc:HGNC:11875]	2.1	2
1955	*GPX2*	2877	glutathione peroxidase 2 [Source:HGNC Symbol;Acc:HGNC:4554]	2.1	2
1956	*MTG2*	26164	mitochondrial ribosome associated GTPase 2 [Source:HGNC Symbol;Acc:HGNC:16239]	2.1	2
1957	*CARD16*	114769	caspase recruitment domain family member 16 [Source:HGNC Symbol;Acc:HGNC:33701]	2.1	2
1958	*NPEPL1*	79716	aminopeptidase-like 1 [Source:HGNC Symbol;Acc:HGNC:16244]	2.1	2
1959	*SDC4*	6385	syndecan 4 [Source:HGNC Symbol;Acc:HGNC:10661]	2.1	2
1960	*TMEM129*	92305	transmembrane protein 129 [Source:HGNC Symbol;Acc:HGNC:25137]	2.1	2
1961	*RARA*	5914	retinoic acid receptor alpha [Source:HGNC Symbol;Acc:HGNC:9864]	2.1	2
1962	*PDLIM5*	10611	PDZ and LIM domain 5 [Source:HGNC Symbol;Acc:HGNC:17468]	2.1	2
1963	*ZXDC*	79364	ZXD family zinc finger C [Source:HGNC Symbol;Acc:HGNC:28160]	2.1	2
1964	*PPIP5K1*	9677	diphosphoinositol pentakisphosphate kinase 1 [Source:HGNC Symbol;Acc:HGNC:29023]	2.1	2
1965	*TRAFD1*	10906	TRAF-type zinc finger domain containing 1 [Source:HGNC Symbol;Acc:HGNC:24808]	2.1	2
1966	*SIPA1L2*	57568	signal induced proliferation associated 1 like 2 [Source:HGNC Symbol;Acc:HGNC:23800]	2.1	2
1967	*SAR1A*	56681	secretion associated Ras related GTPase 1A [Source:HGNC Symbol;Acc:HGNC:10534]	2.1	2
1968	*SLC12A9*	56996	solute carrier family 12 member 9 [Source:HGNC Symbol;Acc:HGNC:17435]	2.1	2
1969	*PPP3CA*	5530	protein phosphatase 3 catalytic subunit alpha [Source:HGNC Symbol;Acc:HGNC:9314]	2.1	2
1970	*SWI5*	375757	SWI5 homologous recombination repair protein [Source:HGNC Symbol;Acc:HGNC:31412]	2.1	2
1971	*SMCR8*	140775	Smith-Magenis syndrome chromosome region, candidate 8 [Source:HGNC Symbol;Acc:HGNC:17921]	2.1	2
1972	*LPAR2*	9170	lysophosphatidic acid receptor 2 [Source:HGNC Symbol;Acc:HGNC:3168]	2.1	2
1973	*RHOG*	391	ras homolog family member G [Source:HGNC Symbol;Acc:HGNC:672]	2.1	2
1974	*RAB24*	53917	RAB24, member RAS oncogene family [Source:HGNC Symbol;Acc:HGNC:9765]	2.1	2
1975	*FGFR1OP2*	26127	FGFR1 oncogene partner 2 [Source:HGNC Symbol;Acc:HGNC:23098]	2.1	2
1976	*TNFAIP2*	7127	TNF alpha induced protein 2 [Source:HGNC Symbol;Acc:HGNC:11895]	2.1	2
1977	*ZNF135*	7694	zinc finger protein 135 [Source:HGNC Symbol;Acc:HGNC:12919]	2.1	2
1978	*TAX1BP3*	30851	Tax1 binding protein 3 [Source:HGNC Symbol;Acc:HGNC:30684]	2.1	2
1979	*CYB5R3*	1727	cytochrome b5 reductase 3 [Source:HGNC Symbol;Acc:HGNC:2873]	2.1	2
1980	*VGLL4*	9686	vestigial like family member 4 [Source:HGNC Symbol;Acc:HGNC:28966]	2.1	2
1981	*HLA-E*	3133	major histocompatibility complex, class I, E [Source:HGNC Symbol;Acc:HGNC:4962]	2.1	2
1982	*MXD3*	83463	MAX dimerization protein 3 [Source:HGNC Symbol;Acc:HGNC:14008]	2.1	2
1983	*PRELID1*	27166	PRELI domain containing 1 [Source:HGNC Symbol;Acc:HGNC:30255]	2.1	2
1984	*CACNG6*	59285	calcium voltage-gated channel auxiliary subunit gamma 6 [Source:HGNC Symbol;Acc:HGNC:13625]	2.1	2
1985	*CHD9*	80205	chromodomain helicase DNA binding protein 9 [Source:HGNC Symbol;Acc:HGNC:25701]	2.1	2
1986	*ARMCX3*	51566	armadillo repeat containing, X-linked 3 [Source:HGNC Symbol;Acc:HGNC:24065]	2.1	2
1987	*DENR*	8562	density-regulated protein [Source:HGNC Symbol;Acc:HGNC:2769]	2.1	2
1988	*PAPOLA*	10914	poly(A) polymerase alpha [Source:HGNC Symbol;Acc:HGNC:14981]	2.1	2
1989	*SLC16A6*	9120	solute carrier family 16 member 6 [Source:HGNC Symbol;Acc:HGNC:10927]	2.1	2
1990	*NKIRAS2*	28511	NFKB inhibitor interacting Ras like 2 [Source:HGNC Symbol;Acc:HGNC:17898]	2.1	2
1991	*F2R*	2149	coagulation factor II thrombin receptor [Source:HGNC Symbol;Acc:HGNC:3537]	2.1	2
1992	*ATHL1*	80162	ATH1, acid trehalase-like 1 (yeast) [Source:HGNC Symbol;Acc:HGNC:26210]	2.1	2
1993	*TRIM25*	7706	tripartite motif containing 25 [Source:HGNC Symbol;Acc:HGNC:12932]	2.1	2
1994	*CHD1L*	9557	chromodomain helicase DNA binding protein 1 like [Source:HGNC Symbol;Acc:HGNC:1916]	2.1	2
1995	*SYTL1*	84958	synaptotagmin like 1 [Source:HGNC Symbol;Acc:HGNC:15584]	2.1	2
1996	*MOB1B*	92597	MOB kinase activator 1B [Source:HGNC Symbol;Acc:HGNC:29801]	2.1	2
1997	*ZNF593*	51042	zinc finger protein 593 [Source:HGNC Symbol;Acc:HGNC:30943]	2.1	2
1998	*RAPGEF2*	9693	Rap guanine nucleotide exchange factor 2 [Source:HGNC Symbol;Acc:HGNC:16854]	2.1	2
1999	*COL1A1*	1277	collagen type I alpha 1 [Source:HGNC Symbol;Acc:HGNC:2197]	2.1	2
2000	*ID1*	3397	inhibitor of DNA binding 1, HLH protein [Source:HGNC Symbol;Acc:HGNC:5360]	2.1	2
2001	*S100P*	6286	S100 calcium binding protein P [Source:HGNC Symbol;Acc:HGNC:10504]	2.1	2
2002	*YIF1B*	90522	Yip1 interacting factor homolog B, membrane trafficking protein [Source:HGNC Symbol;Acc:HGNC:30511]	2.1	2
2003	*VAV3*	10451	vav guanine nucleotide exchange factor 3 [Source:HGNC Symbol;Acc:HGNC:12659]	2.1	2
2004	*APOBEC3D*	140564	apolipoprotein B mRNA editing enzyme catalytic subunit 3D [Source:HGNC Symbol;Acc:HGNC:17354]	2.1	2
2005	*PHF5A*	84844	PHD finger protein 5A [Source:HGNC Symbol;Acc:HGNC:18000]	2.1	2
2006	*TTC7A*	57217	tetratricopeptide repeat domain 7A [Source:HGNC Symbol;Acc:HGNC:19750]	2.1	2
2007	*TMEM259*	91304	transmembrane protein 259 [Source:HGNC Symbol;Acc:HGNC:17039]	2.1	2
2008	*AXIN1*	8312	axin 1 [Source:HGNC Symbol;Acc:HGNC:903]	2.1	2
2009	*SERPINC1*	462	serpin family C member 1 [Source:HGNC Symbol;Acc:HGNC:775]	2.1	2
2010	*MEF2D*	4209	myocyte enhancer factor 2D [Source:HGNC Symbol;Acc:HGNC:6997]	2.0	2
2011	*FCRL3*	115352	Fc receptor like 3 [Source:HGNC Symbol;Acc:HGNC:18506]	2.0	2

2012	*ATP2C1*	27032	ATPase secretory pathway Ca2+ transporting 1 [Source:HGNC Symbol;Acc:HGNC:13211]	2.0	2
2013	*NAP1L1*	4673	nucleosome assembly protein 1 like 1 [Source:HGNC Symbol;Acc:HGNC:7637]	2.0	2
2014	*INA*	9118	internexin neuronal intermediate filament protein alpha [Source:HGNC Symbol;Acc:HGNC:6057]	2.0	2
2015	*EFEMP2*	30008	EGF containing fibulin like extracellular matrix protein 2 [Source:HGNC Symbol;Acc:HGNC:3219]	2.0	2
2016	*HVCN1*	84329	hydrogen voltage gated channel 1 [Source:HGNC Symbol;Acc:HGNC:28240]	2.0	2
2017	*UBN1*	29855	ubinuclein 1 [Source:HGNC Symbol;Acc:HGNC:12506]	2.0	2
2018	*TOR4A*	54863	torsin family 4 member A [Source:HGNC Symbol;Acc:HGNC:25981]	2.0	2
2019	*LGALS1*	3956	galectin 1 [Source:HGNC Symbol;Acc:HGNC:6561]	2.0	2
2020	*RNF10*	9921	ring finger protein 10 [Source:HGNC Symbol;Acc:HGNC:10055]	2.0	2
2021	*RNF130*	55819	ring finger protein 130 [Source:HGNC Symbol;Acc:HGNC:18280]	2.0	2
2022	*CASP1*	834	caspase 1 [Source:HGNC Symbol;Acc:HGNC:1499]	2.0	2
2023	*ST6GAL2*	84620	ST6 beta-galactoside alpha-2,6-sialyltransferase 2 [Source:HGNC Symbol;Acc:HGNC:10861]	2.0	2
2024	*ZIC2*	7546	Zic family member 2 [Source:HGNC Symbol;Acc:HGNC:12873]	2.0	2
2025	*EMILIN2*	84034	elastin microfibril interfacer 2 [Source:HGNC Symbol;Acc:HGNC:19881]	2.0	2
2026	*CHST2*	9435	carbohydrate sulfotransferase 2 [Source:HGNC Symbol;Acc:HGNC:1970]	2.0	2
2027	*CD276*	80381	CD276 molecule [Source:HGNC Symbol;Acc:HGNC:19137]	2.0	2
2028	*EVI2A*	2123	ecotropic viral integration site 2A [Source:HGNC Symbol;Acc:HGNC:3499]	2.0	2
2029	*MSL2*	55167	male-specific lethal 2 homolog (Drosophila) [Source:HGNC Symbol;Acc:HGNC:25544]	2.0	2
2030	*LITAF*	9516	lipopolysaccharide induced TNF factor [Source:HGNC Symbol;Acc:HGNC:16841]	2.0	2
2031	*SRRM2*	23524	serine/arginine repetitive matrix 2 [Source:HGNC Symbol;Acc:HGNC:16639]	2.0	2
2032	*VASP*	7408	vasodilator-stimulated phosphoprotein [Source:HGNC Symbol;Acc:HGNC:12652]	2.0	2
2033	*RPH3A*	22895	rabphilin 3A [Source:HGNC Symbol;Acc:HGNC:17056]	2.0	2
2034	*BID*	637	BH3 interacting domain death agonist [Source:HGNC Symbol;Acc:HGNC:1050]	2.0	2
2035	*CSTA*	1475	cystatin A [Source:HGNC Symbol;Acc:HGNC:2481]	2.0	2
2036	*BRCA1*	672	BRCA1, DNA repair associated [Source:HGNC Symbol;Acc:HGNC:1100]	2.0	2
2037	*EMC3*	55831	ER membrane protein complex subunit 3 [Source:HGNC Symbol;Acc:HGNC:23999]	2.0	2
2038	*ATXN7*	6314	ataxin 7 [Source:HGNC Symbol;Acc:HGNC:10560]	2.0	2
2039	*PNPLA6*	10908	patatin like phospholipase domain containing 6 [Source:HGNC Symbol;Acc:HGNC:16268]	2.0	2
2040	*SECTM1*	6398	secreted and transmembrane 1 [Source:HGNC Symbol;Acc:HGNC:10707]	2.0	2
2041	*HP*	3240	haptoglobin [Source:HGNC Symbol;Acc:HGNC:5141]	2.0	2
2042	*TKT*	7086	transketolase [Source:HGNC Symbol;Acc:HGNC:11834]	2.0	2
2043	*SEMA4A*	64218	semaphorin 4A [Source:HGNC Symbol;Acc:HGNC:10729]	2.0	2
2044	*VDAC2*	7417	voltage dependent anion channel 2 [Source:HGNC Symbol;Acc:HGNC:12672]	2.0	2
2045	*POLR1B*	84172	polymerase (RNA) I subunit B [Source:HGNC Symbol;Acc:HGNC:20454]	2.0	2
2046	*GCLM*	2730	glutamate-cysteine ligase modifier subunit [Source:HGNC Symbol;Acc:HGNC:4312]	2.0	2
2047	*ISCA1*	81689	iron-sulfur cluster assembly 1 [Source:HGNC Symbol;Acc:HGNC:28660]	2.0	2
2048	*PITPNA*	5306	phosphatidylinositol transfer protein alpha [Source:HGNC Symbol;Acc:HGNC:9001]	2.0	2
2049	*EIF4EBP2*	1979	eukaryotic translation initiation factor 4E binding protein 2 [Source:HGNC Symbol;Acc:HGNC:3289]	2.0	2
2050	*THEM5*	284486	thioesterase superfamily member 5 [Source:HGNC Symbol;Acc:HGNC:26755]	2.0	2
2051	*SNX24*	28966	sorting nexin 24 [Source:HGNC Symbol;Acc:HGNC:21533]	2.0	2
2052	*RHBDF2*	79651	rhomboid 5 homolog 2 [Source:HGNC Symbol;Acc:HGNC:20788]	2.0	2
2053	*VDR*	7421	vitamin D (1,25- dihydroxyvitamin D3) receptor [Source:HGNC Symbol;Acc:HGNC:12679]	2.0	2
2054	*H2AFZ*	3015	H2A histone family member Z [Source:HGNC Symbol;Acc:HGNC:4741]	2.0	2
2055	*RNPEPL1*	57140	arginyl aminopeptidase (aminopeptidase B)-like 1 [Source:HGNC Symbol;Acc:HGNC:10079]	2.0	2
2056	*GIMAP7*	168537	GTPase, IMAP family member 7 [Source:HGNC Symbol;Acc:HGNC:22404]	2.0	2
2057	*CKS1B*	1163	CDC28 protein kinase regulatory subunit 1B [Source:HGNC Symbol;Acc:HGNC:19083]	2.0	2
2058	*PFKM*	5213	phosphofructokinase, muscle [Source:HGNC Symbol;Acc:HGNC:8877]	2.0	2
2059	*MYO9B*	4650	myosin IXB [Source:HGNC Symbol;Acc:HGNC:7609]	2.0	2
2060	*ARPC1B*	10095	actin related protein 2/3 complex subunit 1B [Source:HGNC Symbol;Acc:HGNC:704]	2.0	2
2061	*ANGEL1*	23357	angel homolog 1 (Drosophila) [Source:HGNC Symbol;Acc:HGNC:19961]	2.0	2
2062	*RPS6KA1*	6195	ribosomal protein S6 kinase A1 [Source:HGNC Symbol;Acc:HGNC:10430]	2.0	2
2063	*CAMK1*	8536	calcium/calmodulin dependent protein kinase I [Source:HGNC Symbol;Acc:HGNC:1459]	2.0	2
2064	*TNPO3*	23534	transportin 3 [Source:HGNC Symbol;Acc:HGNC:17103]	2.0	2
2065	*BAZ2A*	11176	bromodomain adjacent to zinc finger domain 2A [Source:HGNC Symbol;Acc:HGNC:962]	2.0	2
2066	*OPHN1*	4983	oligophrenin 1 [Source:HGNC Symbol;Acc:HGNC:8148]	2.0	2
2067	*H2AFJ*	55766	H2A histone family member J [Source:HGNC Symbol;Acc:HGNC:14456]	2.0	2
2068	*C1QB*	713	complement component 1, q subcomponent, B chain [Source:HGNC Symbol;Acc:HGNC:1242]	2.0	2
2069	*CTSS*	1520	cathepsin S [Source:HGNC Symbol;Acc:HGNC:2545]	2.0	2
2070	*NHP2*	55651	NHP2 ribonucleoprotein [Source:HGNC Symbol;Acc:HGNC:14377]	2.0	2
2071	*FCRL1*	115350	Fc receptor like 1 [Source:HGNC Symbol;Acc:HGNC:18509]	2.0	2
2072	*FNBP1*	23048	formin binding protein 1 [Source:HGNC Symbol;Acc:HGNC:17069]	2.0	2
2073	*VDAC3*	7419	voltage dependent anion channel 3 [Source:HGNC Symbol;Acc:HGNC:12674]	2.0	2
2074	*TMEM241*	85019	transmembrane protein 241 [Source:HGNC Symbol;Acc:HGNC:31723]	2.0	2
2075	*NPLOC4*	55666	NPL4 homolog, ubiquitin recognition factor [Source:HGNC Symbol;Acc:HGNC:18261]	2.0	2
2076	*ADGRE3*	84658	adhesion G protein-coupled receptor E3 [Source:HGNC Symbol;Acc:HGNC:23647]	2.0	2
2077	*CD86*	942	CD86 molecule [Source:HGNC Symbol;Acc:HGNC:1705]	2.0	2
2078	*TOM1*	10043	target of myb1 membrane trafficking protein [Source:HGNC Symbol;Acc:HGNC:11982]	2.0	2
2079	*SEPT11*	55752	septin 11 [Source:HGNC Symbol;Acc:HGNC:25589]	2.0	2
2080	*HIST1H2AE*	3012	histone cluster 1, H2ae [Source:HGNC Symbol;Acc:HGNC:4724]	2.0	2
2081	*GPR137C*	283554	G protein-coupled receptor 137C [Source:HGNC Symbol;Acc:HGNC:25445]	2.0	2
2082	*TCF4*	6925	transcription factor 4 [Source:HGNC Symbol;Acc:HGNC:11634]	2.0	2
2083	*RBX1*	9978	ring-box 1 [Source:HGNC Symbol;Acc:HGNC:9928]	2.0	2
2084	*WTAP*	9589	Wilms tumor 1 associated protein [Source:HGNC Symbol;Acc:HGNC:16846]	2.0	2
2085	*CIC*	23152	capicua transcriptional repressor [Source:HGNC Symbol;Acc:HGNC:14214]	2.0	2
2086	*SDC3*	9672	syndecan 3 [Source:HGNC Symbol;Acc:HGNC:10660]	2.0	2
2087	*RAP1A*	5906	RAP1A, member of RAS oncogene family [Source:HGNC Symbol;Acc:HGNC:9855]	2.0	2
2088	*TLR5*	7100	toll like receptor 5 [Source:HGNC Symbol;Acc:HGNC:11851]	2.0	2

2089	*SCARB2*	950	scavenger receptor class B member 2 [Source:HGNC Symbol;Acc:HGNC:1665]	2.0	2
2090	*VPS37B*	79720	VPS37B, ESCRT-I subunit [Source:HGNC Symbol;Acc:HGNC:25754]	2.0	2
2091	*NHLRC2*	374354	NHL repeat containing 2 [Source:HGNC Symbol;Acc:HGNC:24731]	2.0	2
2092	*MAP3K11*	4296	mitogen-activated protein kinase kinase kinase 11 [Source:HGNC Symbol;Acc:HGNC:6850]	2.0	2
2093	*MAP4K1*	11184	mitogen-activated protein kinase kinase kinase kinase 1 [Source:HGNC Symbol;Acc:HGNC:6863]	2.0	2
2094	*PRRC2B*	84726	proline rich coiled-coil 2B [Source:HGNC Symbol;Acc:HGNC:28121]	2.0	2
2095	*BRK1*	55845	BRICK1, SCAR/WAVE actin nucleating complex subunit [Source:HGNC Symbol;Acc:HGNC:23057]	2.0	2
2096	*BASP1*	10409	brain abundant membrane attached signal protein 1 [Source:HGNC Symbol;Acc:HGNC:957]	2.0	2
2097	*PDSS1*	23590	prenyl (decaprenyl) diphosphate synthase, subunit 1 [Source:HGNC Symbol;Acc:HGNC:17759]	2.0	2
2098	*PRAF2*	11230	PRA1 domain family member 2 [Source:HGNC Symbol;Acc:HGNC:28911]	2.0	2
2099	*NPRL3*	8131	NPR3 like, GATOR1 complex subunit [Source:HGNC Symbol;Acc:HGNC:14124]	2.0	2
2100	*DNAH3*	55567	dynein axonemal heavy chain 3 [Source:HGNC Symbol;Acc:HGNC:2949]	2.0	2
2101	*CEACAM4*	1089	carcinoembryonic antigen related cell adhesion molecule 4 [Source:HGNC Symbol;Acc:HGNC:1816]	2.0	2
2102	*CERS1*	10715	ceramide synthase 1 [Source:HGNC Symbol;Acc:HGNC:14253]	2.0	2
2103	*BAHCC1*	57597	BAH domain and coiled-coil containing 1 [Source:HGNC Symbol;Acc:HGNC:29279]	2.0	2
2104	*ATF4*	468	activating transcription factor 4 [Source:HGNC Symbol;Acc:HGNC:786]	2.0	2
2105	*GTPBP1*	9567	GTP binding protein 1 [Source:HGNC Symbol;Acc:HGNC:4669]	2.0	2
2106	*RAB29*	8934	RAB29, member RAS oncogene family [Source:HGNC Symbol;Acc:HGNC:9789]	2.0	2
2107	*CD14*	929	CD14 molecule [Source:HGNC Symbol;Acc:HGNC:1628]	2.0	2
2108	*TRIM38*	10475	tripartite motif containing 38 [Source:HGNC Symbol;Acc:HGNC:10059]	2.0	2
2109	*LAMTOR1*	55004	late endosomal/lysosomal adaptor, MAPK and MTOR activator 1 [Source:HGNC Symbol;Acc:HGNC:26068]	2.0	2
2110	*SLC35A4*	113829	solute carrier family 35 member A4 [Source:HGNC Symbol;Acc:HGNC:20753]	2.0	2
2111	*FCGR3B*	2215	Fc fragment of IgG receptor IIIb [Source:HGNC Symbol;Acc:HGNC:3620]	2.0	2
2112	*CYB5R1*	51706	cytochrome b5 reductase 1 [Source:HGNC Symbol;Acc:HGNC:13397]	2.0	2
2113	*PISD*	23761	phosphatidylserine decarboxylase [Source:HGNC Symbol;Acc:HGNC:8999]	2.0	2
2114	*ANKRD36*	375248	ankyrin repeat domain 36 [Source:HGNC Symbol;Acc:HGNC:24079]	2.0	2
2115	*C17orf62*	79415	chromosome 17 open reading frame 62 [Source:HGNC Symbol;Acc:HGNC:28672]	2.0	2
2116	*STAB1*	23166	stabilin 1 [Source:HGNC Symbol;Acc:HGNC:18628]	1.9	2
2117	*ARPC2*	10109	actin related protein 2/3 complex subunit 2 [Source:HGNC Symbol;Acc:HGNC:705]	1.9	2
2118	*USP12*	219333	ubiquitin specific peptidase 12 [Source:HGNC Symbol;Acc:HGNC:20485]	1.9	2
2119	*LCP1*	3936	lymphocyte cytosolic protein 1 [Source:HGNC Symbol;Acc:HGNC:6528]	1.9	2
2120	*DDX17*	10521	DEAD-box helicase 17 [Source:HGNC Symbol;Acc:HGNC:2740]	1.9	2
2121	*SP1*	6667	Sp1 transcription factor [Source:HGNC Symbol;Acc:HGNC:11205]	1.9	2
2122	*SH2B3*	10019	SH2B adaptor protein 3 [Source:HGNC Symbol;Acc:HGNC:29605]	1.9	2
2123	*RIT1*	6016	Ras like without CAAX 1 [Source:HGNC Symbol;Acc:HGNC:10023]	1.9	2
2124	*OGFRL1*	79627	opioid growth factor receptor-like 1 [Source:HGNC Symbol;Acc:HGNC:21378]	1.9	2
2125	*MATR3*	9782	matrin 3 [Source:HGNC Symbol;Acc:HGNC:6912]	1.9	2
2126	*PRKAR1B*	5575	protein kinase cAMP-dependent type I regulatory subunit beta [Source:HGNC Symbol;Acc:HGNC:9390]	1.9	2
2127	*TLR2*	7097	toll like receptor 2 [Source:HGNC Symbol;Acc:HGNC:11848]	1.9	2
2128	*ZBTB45*	84878	zinc finger and BTB domain containing 45 [Source:HGNC Symbol;Acc:HGNC:23715]	1.9	2
2129	*VPS37A*	137492	VPS37A, ESCRT-I subunit [Source:HGNC Symbol;Acc:HGNC:24928]	1.9	2
2130	*TM4SF18*	116441	transmembrane 4 L six family member 18 [Source:HGNC Symbol;Acc:HGNC:25181]	1.9	2
2131	*STAT5B*	6777	signal transducer and activator of transcription 5B [Source:HGNC Symbol;Acc:HGNC:11367]	1.9	2
2132	*SLC38A7*	55238	solute carrier family 38 member 7 [Source:HGNC Symbol;Acc:HGNC:25582]	1.9	2
2133	*PSIP1*	11168	PC4 and SFRS1 interacting protein 1 [Source:HGNC Symbol;Acc:HGNC:9527]	1.9	2
2134	*DUSP6*	1848	dual specificity phosphatase 6 [Source:HGNC Symbol;Acc:HGNC:3072]	1.9	2
2135	*ERC1*	23085	ELKS/RAB6-interacting/CAST family member 1 [Source:HGNC Symbol;Acc:HGNC:17072]	1.9	2
2136	*NUDT19*	390916	nudix hydrolase 19 [Source:HGNC Symbol;Acc:HGNC:32036]	1.9	2
2137	*MRTO4*	51154	MRT4 homolog, ribosome maturation factor [Source:HGNC Symbol;Acc:HGNC:18477]	1.9	2
2138	*SKAP2*	8935	src kinase associated phosphoprotein 2 [Source:HGNC Symbol;Acc:HGNC:15687]	1.9	2
2139	*JMJD6*	23210	arginine demethylase and lysine hydroxylase [Source:HGNC Symbol;Acc:HGNC:19355]	1.9	2
2140	*SLC22A18*	5002	solute carrier family 22 member 18 [Source:HGNC Symbol;Acc:HGNC:10964]	1.9	2
2141	*MS4A6A*	64231	membrane spanning 4-domains A6A [Source:HGNC Symbol;Acc:HGNC:13375]	1.9	2
2142	*RALB*	5899	RALB Ras like proto-oncogene B [Source:HGNC Symbol;Acc:HGNC:9840]	1.9	2
2143	*NOTCH2NL*	388677	notch 2 N-terminal like [Source:HGNC Symbol;Acc:HGNC:31862]	1.9	2
2144	*PFN1*	5216	profilin 1 [Source:HGNC Symbol;Acc:HGNC:8881]	1.9	2
2145	*PIK3CG*	5294	phosphatidylinositol-4,5-bisphosphate 3-kinase catalytic subunit gamma [Source:HGNC Symbol;Acc:HGNC:8978]	1.9	2
2146	*NFAM1*	150372	NFAT activating protein with ITAM motif 1 [Source:HGNC Symbol;Acc:HGNC:29872]	1.9	2
2147	*MAPRE2*	10982	microtubule associated protein RP/EB family member 2 [Source:HGNC Symbol;Acc:HGNC:6891]	1.9	2
2148	*ENGASE*	64772	endo-beta-N-acetylglucosaminidase [Source:HGNC Symbol;Acc:HGNC:24622]	1.9	2
2149	*PKP2*	5318	plakophilin 2 [Source:HGNC Symbol;Acc:HGNC:9024]	1.9	2
2150	*TNFRSF14*	8764	tumor necrosis factor receptor superfamily member 14 [Source:HGNC Symbol;Acc:HGNC:11912]	1.9	2
2151	*SIRPA*	140885	signal regulatory protein alpha [Source:HGNC Symbol;Acc:HGNC:9662]	1.9	2
2152	*USF3*	205717	upstream transcription factor family member 3 [Source:HGNC Symbol;Acc:HGNC:30494]	1.9	2
2153	*MEPCE*	56257	methylphosphate capping enzyme [Source:HGNC Symbol;Acc:HGNC:20247]	1.9	2
2154	*S100A4*	6275	S100 calcium binding protein A4 [Source:HGNC Symbol;Acc:HGNC:10494]	1.9	2
2155	*STRN4*	29888	striatin 4 [Source:HGNC Symbol;Acc:HGNC:15721]	1.9	2
2156	*RHAG*	6005	Rh-associated glycoprotein [Source:HGNC Symbol;Acc:HGNC:10006]	1.9	2
2157	*RNF123*	63891	ring finger protein 123 [Source:HGNC Symbol;Acc:HGNC:21148]	1.9	2
2158	*AFAP1L1*	134265	actin filament associated protein 1 like 1 [Source:HGNC Symbol;Acc:HGNC:26714]	1.9	2
2159	*ZFYVE27*	118813	zinc finger FYVE-type containing 27 [Source:HGNC Symbol;Acc:HGNC:26559]	1.9	2

2160	*CMC2*	56942	C-X9-C motif containing 2 [Source:HGNC Symbol;Acc:HGNC:24447]	1.9	2
2161	*CLIP1*	6249	CAP-Gly domain containing linker protein 1 [Source:HGNC Symbol;Acc:HGNC:10461]	1.9	2
2162	*BATF3*	55509	basic leucine zipper ATF-like transcription factor 3 [Source:HGNC Symbol;Acc:HGNC:28915]	1.9	2
2163	*SHOC2*	8036	SHOC2, leucine rich repeat scaffold protein [Source:HGNC Symbol;Acc:HGNC:15454]	1.9	2
2164	*PSMB3*	5691	proteasome subunit beta 3 [Source:HGNC Symbol;Acc:HGNC:9540]	1.9	2
2165	*KPNA3*	3839	karyopherin subunit alpha 3 [Source:HGNC Symbol;Acc:HGNC:6396]	1.9	2
2166	*SLC7A7*	9056	solute carrier family 7 member 7 [Source:HGNC Symbol;Acc:HGNC:11065]	1.9	2
2167	*PRRC2A*	7916	proline rich coiled-coil 2A [Source:HGNC Symbol;Acc:HGNC:13918]	1.9	2
2168	*SLC25A40*	55972	solute carrier family 25 member 40 [Source:HGNC Symbol;Acc:HGNC:29680]	1.9	2
2169	*ARHGAP26*	23092	Rho GTPase activating protein 26 [Source:HGNC Symbol;Acc:HGNC:17073]	1.9	2
2170	*NDE1*	54820	nudE neurodevelopment protein 1 [Source:HGNC Symbol;Acc:HGNC:17619]	1.9	2
2171	*ITGB1*	3688	integrin subunit beta 1 [Source:HGNC Symbol;Acc:HGNC:6153]	1.9	2
2172	*SRSF8*	10929	serine and arginine rich splicing factor 8 [Source:HGNC Symbol;Acc:HGNC:16988]	1.9	2
2173	*ETFB*	2109	electron transfer flavoprotein beta subunit [Source:HGNC Symbol;Acc:HGNC:3482]	1.9	2
2174	*GUK1*	2987	guanylate kinase 1 [Source:HGNC Symbol;Acc:HGNC:4693]	1.9	2
2175	*KDM2B*	84678	lysine demethylase 2B [Source:HGNC Symbol;Acc:HGNC:13610]	1.9	2
2176	*BTF3*	689	basic transcription factor 3 [Source:HGNC Symbol;Acc:HGNC:1125]	1.9	2
2177	*PKP4*	8502	plakophilin 4 [Source:HGNC Symbol;Acc:HGNC:9026]	1.9	2
2178	*C1orf106*	55765	chromosome 1 open reading frame 106 [Source:HGNC Symbol;Acc:HGNC:25599]	1.9	2
2179	*RIN1*	9610	Ras and Rab interactor 1 [Source:HGNC Symbol;Acc:HGNC:18749]	1.9	2
2180	*RAB13*	5872	RAB13, member RAS oncogene family [Source:HGNC Symbol;Acc:HGNC:9762]	1.9	2
2181	*SAV1*	60485	salvador family WW domain containing protein 1 [Source:HGNC Symbol;Acc:HGNC:17795]	1.9	2
2182	*PYCARD*	29108	PYD and CARD domain containing [Source:HGNC Symbol;Acc:HGNC:16608]	1.9	2
2183	*KTN1*	3895	kinectin 1 [Source:HGNC Symbol;Acc:HGNC:6467]	1.9	2
2184	*INSR*	3643	insulin receptor [Source:HGNC Symbol;Acc:HGNC:6091]	1.9	2
2185	*BMP6*	654	bone morphogenetic protein 6 [Source:HGNC Symbol;Acc:HGNC:1073]	1.9	2
2186	*FBRSL1*	57666	fibrosin like 1 [Source:HGNC Symbol;Acc:HGNC:29308]	1.9	2
2187	*CCDC7*	221016	coiled-coil domain containing 7 [Source:HGNC Symbol;Acc:HGNC:26533]	1.9	2
2188	*ITGA4*	3676	integrin subunit alpha 4 [Source:HGNC Symbol;Acc:HGNC:6140]	1.9	2
2189	*CDS2*	8760	CDP-diacylglycerol synthase 2 [Source:HGNC Symbol;Acc:HGNC:1801]	1.9	2
2190	*ACOT7*	11332	acyl-CoA thioesterase 7 [Source:HGNC Symbol;Acc:HGNC:24157]	1.9	2
2191	*CYTIP*	9595	cytohesin 1 interacting protein [Source:HGNC Symbol;Acc:HGNC:9506]	1.9	2
2192	*HIST1H2AI*	8329	histone cluster 1, H2ai [Source:HGNC Symbol;Acc:HGNC:4725]	1.9	2
2193	*TIMM8A*	1678	translocase of inner mitochondrial membrane 8 homolog A (yeast) [Source:HGNC Symbol;Acc:HGNC:11817]	1.9	2
2194	*TSR1*	55720	TSR1, ribosome maturation factor [Source:HGNC Symbol;Acc:HGNC:25542]	1.9	2
2195	*PFDN6*	10471	prefoldin subunit 6 [Source:HGNC Symbol;Acc:HGNC:4926]	1.9	2
2196	*MED15*	51586	mediator complex subunit 15 [Source:HGNC Symbol;Acc:HGNC:14248]	1.9	2
2197	*KIF20A*	10112	kinesin family member 20A [Source:HGNC Symbol;Acc:HGNC:9787]	1.9	2
2198	*RASSF3*	283349	Ras association domain family member 3 [Source:HGNC Symbol;Acc:HGNC:14271]	1.9	2
2199	*SYDE1*	85360	synapse defective Rho GTPase homolog 1 [Source:HGNC Symbol;Acc:HGNC:25824]	1.9	2
2200	*MYL6*	4637	myosin light chain 6 [Source:HGNC Symbol;Acc:HGNC:7587]	1.9	2
2201	*LRP8*	7804	LDL receptor related protein 8 [Source:HGNC Symbol;Acc:HGNC:6700]	1.9	2
2202	*CLK3*	1198	CDC like kinase 3 [Source:HGNC Symbol;Acc:HGNC:2071]	1.9	2
2203	*ZNF221*	7638	zinc finger protein 221 [Source:HGNC Symbol;Acc:HGNC:13014]	1.9	2
2204	*FAM229B*	619208	family with sequence similarity 229 member B [Source:HGNC Symbol;Acc:HGNC:33858]	1.9	2
2205	*TMEM173*	340061	transmembrane protein 173 [Source:HGNC Symbol;Acc:HGNC:27962]	1.9	2
2206	*CA4*	762	carbonic anhydrase 4 [Source:HGNC Symbol;Acc:HGNC:1375]	1.9	2
2207	*HDAC2*	3066	histone deacetylase 2 [Source:HGNC Symbol;Acc:HGNC:4853]	1.9	2
2208	*ADH4*	127	alcohol dehydrogenase 4 (class II), pi polypeptide [Source:HGNC Symbol;Acc:HGNC:252]	1.9	2
2209	*REL*	5966	REL proto-oncogene, NF-kB subunit [Source:HGNC Symbol;Acc:HGNC:9954]	1.9	2
2210	*MON1B*	22879	MON1 homolog B, secretory trafficking associated [Source:HGNC Symbol;Acc:HGNC:25020]	1.9	2
2211	*ARHGEF18*	23370	Rho/Rac guanine nucleotide exchange factor 18 [Source:HGNC Symbol;Acc:HGNC:17090]	1.9	2
2212	*DBN1*	1627	drebrin 1 [Source:HGNC Symbol;Acc:HGNC:2695]	1.9	2
2213	*CLCN7*	1186	chloride voltage-gated channel 7 [Source:HGNC Symbol;Acc:HGNC:2025]	1.9	2
2214	*SLC39A3*	29985	solute carrier family 39 member 3 [Source:HGNC Symbol;Acc:HGNC:17128]	1.9	2
2215	*ZFPM1*	161882	zinc finger protein, FOG family member 1 [Source:HGNC Symbol;Acc:HGNC:19762]	1.8	2
2216	*TPST2*	8459	tyrosylprotein sulfotransferase 2 [Source:HGNC Symbol;Acc:HGNC:12021]	1.8	2
2217	*PYGL*	5836	phosphorylase, glycogen, liver [Source:HGNC Symbol;Acc:HGNC:9725]	1.8	2
2218	*FAM188A*	80013	family with sequence similarity 188 member A [Source:HGNC Symbol;Acc:HGNC:23578]	1.8	2
2219	*CDH2*	1000	cadherin 2 [Source:HGNC Symbol;Acc:HGNC:1759]	1.8	2
2220	*PSMC4*	5704	proteasome 26S subunit, ATPase 4 [Source:HGNC Symbol;Acc:HGNC:9551]	1.8	2
2221	*PCDHB16*	57717	protocadherin beta 16 [Source:HGNC Symbol;Acc:HGNC:14546]	1.8	2
2222	*PBX2*	5089	PBX homeobox 2 [Source:HGNC Symbol;Acc:HGNC:8633]	1.8	2
2223	*APLP2*	334	amyloid beta precursor like protein 2 [Source:HGNC Symbol;Acc:HGNC:598]	1.8	2
2224	*MAP1LC3B*	81631	microtubule associated protein 1 light chain 3 beta [Source:HGNC Symbol;Acc:HGNC:13352]	1.8	2
2225	*KDELC1*	79070	KDEL motif containing 1 [Source:HGNC Symbol;Acc:HGNC:19350]	1.8	2
2226	*CEP85L*	387119	centrosomal protein 85 like [Source:HGNC Symbol;Acc:HGNC:21638]	1.8	2
2227	*IPO13*	9670	importin 13 [Source:HGNC Symbol;Acc:HGNC:16853]	1.8	2
2228	*CA3*	761	carbonic anhydrase 3 [Source:HGNC Symbol;Acc:HGNC:1374]	1.8	2
2229	*GPR34*	2857	G protein-coupled receptor 34 [Source:HGNC Symbol;Acc:HGNC:4490]	1.8	2
2230	*HRASLS*	57110	HRAS like suppressor [Source:HGNC Symbol;Acc:HGNC:14922]	1.8	2
2231	*ST3GAL6*	10402	ST3 beta-galactoside alpha-2,3-sialyltransferase 6 [Source:HGNC Symbol;Acc:HGNC:18080]	1.8	2
2232	*CES1*	1066	carboxylesterase 1 [Source:HGNC Symbol;Acc:HGNC:1863]	1.8	2
2233	*CPB2*	1361	carboxypeptidase B2 [Source:HGNC Symbol;Acc:HGNC:2300]	1.8	2
2234	*TRIP6*	7205	thyroid hormone receptor interactor 6 [Source:HGNC Symbol;Acc:HGNC:12311]	1.8	2
2235	*PLEKHG2*	64857	pleckstrin homology and RhoGEF domain containing G2 [Source:HGNC Symbol;Acc:HGNC:29515]	1.8	2

2236	*METTL2B*	55798	methyltransferase like 2B [Source:HGNC Symbol;Acc:HGNC:18272]	1.8	2
2237	*CPEB4*	80315	cytoplasmic polyadenylation element binding protein 4 [Source:HGNC Symbol;Acc:HGNC:21747]	1.8	2
2238	*NUP98*	4928	nucleoporin 98 [Source:HGNC Symbol;Acc:HGNC:8068]	1.8	2
2239	*PGAP2*	27315	post-GPI attachment to proteins 2 [Source:HGNC Symbol;Acc:HGNC:17893]	1.8	2
2240	*INPP5F*	22876	inositol polyphosphate-5-phosphatase F [Source:HGNC Symbol;Acc:HGNC:17054]	1.8	2
2241	*MTA3*	57504	metastasis associated 1 family member 3 [Source:HGNC Symbol;Acc:HGNC:23784]	1.8	2
2242	*MYH9*	4627	myosin, heavy chain 9, non-muscle [Source:HGNC Symbol;Acc:HGNC:7579]	1.8	2
2243	*TRAPPC1*	58485	trafficking protein particle complex 1 [Source:HGNC Symbol;Acc:HGNC:19894]	1.8	2
2244	*ARG2*	384	arginase 2 [Source:HGNC Symbol;Acc:HGNC:664]	1.8	2
2245	*YWHAE*	7531	tyrosine 3-monooxygenase/tryptophan 5-monooxygenase activation protein epsilon [Source:HGNC Symbol;Acc:HGNC:12851]	1.8	2
2246	*JAK3*	3718	Janus kinase 3 [Source:HGNC Symbol;Acc:HGNC:6193]	1.8	2
2247	*SMPDL3A*	10924	sphingomyelin phosphodiesterase acid like 3A [Source:HGNC Symbol;Acc:HGNC:17389]	1.8	2
2248	*SMC4*	10051	structural maintenance of chromosomes 4 [Source:HGNC Symbol;Acc:HGNC:14013]	1.8	2
2249	*NCAPD2*	9918	non-SMC condensin I complex subunit D2 [Source:HGNC Symbol;Acc:HGNC:24305]	1.8	2
2250	*SLC50A1*	55974	solute carrier family 50 member 1 [Source:HGNC Symbol;Acc:HGNC:30657]	1.8	2
2251	*SENP6*	26054	SUMO1/sentrin specific peptidase 6 [Source:HGNC Symbol;Acc:HGNC:20944]	1.8	2
2252	*INTS9*	55756	integrator complex subunit 9 [Source:HGNC Symbol;Acc:HGNC:25592]	1.8	2
2253	*SIAE*	54414	sialic acid acetylesterase [Source:HGNC Symbol;Acc:HGNC:18187]	1.8	2
2254	*LEPR*	3953	leptin receptor [Source:HGNC Symbol;Acc:HGNC:6554]	1.8	2
2255	*TNFRSF1A*	7132	tumor necrosis factor receptor superfamily member 1A [Source:HGNC Symbol;Acc:HGNC:11916]	1.8	2
2256	*SLC25A16*	8034	solute carrier family 25 member 16 [Source:HGNC Symbol;Acc:HGNC:10986]	1.8	2
2257	*HBB*	3043	hemoglobin subunit beta [Source:HGNC Symbol;Acc:HGNC:4827]	1.8	2
2258	*CFL2*	1073	cofilin 2 [Source:HGNC Symbol;Acc:HGNC:1875]	1.8	2
2259	*AGFG1*	3267	ArfGAP with FG repeats 1 [Source:HGNC Symbol;Acc:HGNC:5175]	1.8	2
2260	*RASSF4*	83937	Ras association domain family member 4 [Source:HGNC Symbol;Acc:HGNC:20793]	1.8	2
2261	*CAPZA2*	830	capping actin protein of muscle Z-line alpha subunit 2 [Source:HGNC Symbol;Acc:HGNC:1490]	1.8	2
2262	*PLXNA1*	5361	plexin A1 [Source:HGNC Symbol;Acc:HGNC:9099]	1.8	2
2263	*ZFP36L2*	678	ZFP36 ring finger protein-like 2 [Source:HGNC Symbol;Acc:HGNC:1108]	1.8	2
2264	*LUZP6*	767558	leucine zipper protein 6 [Source:HGNC Symbol;Acc:HGNC:33955]	1.8	2
2265	*MAN2B1*	4125	mannosidase alpha class 2B member 1 [Source:HGNC Symbol;Acc:HGNC:6826]	1.8	2
2266	*CLCN3*	1182	chloride voltage-gated channel 3 [Source:HGNC Symbol;Acc:HGNC:2021]	1.8	2
2267	*CT45A5*	441521	cancer/testis antigen family 45, member A5 [Source:HGNC Symbol;Acc:HGNC:33270]	1.8	2
2268	*PPP1R18*	170954	protein phosphatase 1 regulatory subunit 18 [Source:HGNC Symbol;Acc:HGNC:29413]	1.8	2
2269	*ALDOA*	226	aldolase, fructose-bisphosphate A [Source:HGNC Symbol;Acc:HGNC:414]	1.8	2
2270	*BCL2L1*	598	BCL2 like 1 [Source:HGNC Symbol;Acc:HGNC:992]	1.8	2
2271	*FAM72A*	554282	family with sequence similarity 72 member A [Source:HGNC Symbol;Acc:HGNC:24044]	1.8	2
2272	*SIAH2*	6478	siah E3 ubiquitin protein ligase 2 [Source:HGNC Symbol;Acc:HGNC:10858]	1.8	2
2273	*WBP2*	23558	WW domain binding protein 2 [Source:HGNC Symbol;Acc:HGNC:12738]	1.8	2
2274	*ALDH1A2*	8854	aldehyde dehydrogenase 1 family member A2 [Source:HGNC Symbol;Acc:HGNC:15472]	1.8	2
2275	*CALM2*	805	calmodulin 2 (phosphorylase kinase, delta) [Source:HGNC Symbol;Acc:HGNC:1445]	1.8	2
2276	*PPM1F*	9647	protein phosphatase, Mg2+/Mn2+ dependent 1F [Source:HGNC Symbol;Acc:HGNC:19388]	1.8	2
2277	*ECE1*	1889	endothelin converting enzyme 1 [Source:HGNC Symbol;Acc:HGNC:3146]	1.8	2
2278	*CTNS*	1497	cystinosin, lysosomal cystine transporter [Source:HGNC Symbol;Acc:HGNC:2518]	1.8	2
2279	*SYCP2L*	221711	synaptonemal complex protein 2 like [Source:HGNC Symbol;Acc:HGNC:21537]	1.8	2
2280	*NDST1*	3340	N-deacetylase/N-sulfotransferase 1 [Source:HGNC Symbol;Acc:HGNC:7680]	1.8	2
2281	*IGSF6*	10261	immunoglobulin superfamily member 6 [Source:HGNC Symbol;Acc:HGNC:5953]	1.8	2
2282	*SETX*	23064	senataxin [Source:HGNC Symbol;Acc:HGNC:445]	1.8	2
2283	*C9orf91*	203197	chromosome 9 open reading frame 91 [Source:HGNC Symbol;Acc:HGNC:24513]	1.8	2
2284	*UACA*	55075	uveal autoantigen with coiled-coil domains and ankyrin repeats [Source:HGNC Symbol;Acc:HGNC:15947]	1.8	2
2285	*HMGCS1*	3157	3-hydroxy-3-methylglutaryl-CoA synthase 1 [Source:HGNC Symbol;Acc:HGNC:5007]	1.8	2
2286	*SLC16A1*	6566	solute carrier family 16 member 1 [Source:HGNC Symbol;Acc:HGNC:10922]	1.8	2
2287	*ATP1B1*	481	ATPase Na+/K+ transporting subunit beta 1 [Source:HGNC Symbol;Acc:HGNC:804]	1.8	2
2288	*ACSL1*	2180	acyl-CoA synthetase long-chain family member 1 [Source:HGNC Symbol;Acc:HGNC:3569]	1.8	2
2289	*STAG2*	10735	stromal antigen 2 [Source:HGNC Symbol;Acc:HGNC:11355]	1.8	2
2290	*PSME2*	5721	proteasome activator subunit 2 [Source:HGNC Symbol;Acc:HGNC:9569]	1.8	2
2291	*SLC43A2*	124935	solute carrier family 43 member 2 [Source:HGNC Symbol;Acc:HGNC:23087]	1.8	2
2292	*LILRB4*	11006	leukocyte immunoglobulin like receptor B4 [Source:HGNC Symbol;Acc:HGNC:6608]	1.8	2
2293	*ZFAND5*	7763	zinc finger AN1-type containing 5 [Source:HGNC Symbol;Acc:HGNC:13008]	1.8	2
2294	*MTFR1L*	56181	mitochondrial fission regulator 1 like [Source:HGNC Symbol;Acc:HGNC:28836]	1.8	2
2295	*MAP4*	4134	microtubule associated protein 4 [Source:HGNC Symbol;Acc:HGNC:6862]	1.8	2
2296	*CLIC2*	1193	chloride intracellular channel 2 [Source:HGNC Symbol;Acc:HGNC:2063]	1.8	2
2297	*N4BP2L1*	90634	NEDD4 binding protein 2-like 1 [Source:HGNC Symbol;Acc:HGNC:25037]	1.8	2
2298	*GK*	2710	glycerol kinase [Source:HGNC Symbol;Acc:HGNC:4289]	1.8	2
2299	*DHRS13*	147015	dehydrogenase/reductase 13 [Source:HGNC Symbol;Acc:HGNC:28326]	1.8	2
2300	*ARMC10*	83787	armadillo repeat containing 10 [Source:HGNC Symbol;Acc:HGNC:21706]	1.8	2
2301	*NAPEPLD*	222236	N-acyl phosphatidylethanolamine phospholipase D [Source:HGNC Symbol;Acc:HGNC:21683]	1.8	2
2302	*GLIPR1*	11010	GLI pathogenesis related 1 [Source:HGNC Symbol;Acc:HGNC:17001]	1.8	2
2303	*ADI1*	55256	acireductone dioxygenase 1 [Source:HGNC Symbol;Acc:HGNC:30576]	1.8	2
2304	*SERPINB1*	1992	serpin family B member 1 [Source:HGNC Symbol;Acc:HGNC:3311]	1.8	2
2305	*RAB5C*	5878	RAB5C, member RAS oncogene family [Source:HGNC Symbol;Acc:HGNC:9785]	1.8	2
2306	*TNFSF13B*	10673	tumor necrosis factor superfamily member 13b [Source:HGNC Symbol;Acc:HGNC:11929]	1.8	2
2307	*NPTN*	27020	neuroplastin [Source:HGNC Symbol;Acc:HGNC:17867]	1.8	2
2308	*ATAD3B*	83858	ATPase family, AAA domain containing 3B [Source:HGNC Symbol;Acc:HGNC:24007]	1.8	2
2309	*IER2*	9592	immediate early response 2 [Source:HGNC Symbol;Acc:HGNC:28871]	1.8	2
2310	*MTF1*	4520	metal-regulatory transcription factor 1 [Source:HGNC Symbol;Acc:HGNC:7428]	1.8	2

2311	*KCNMB4*	27345	potassium calcium-activated channel subfamily M regulatory beta subunit 4 [Source:HGNC Symbol;Acc:HGNC:6289]	1.8	2
2312	*EMP2*	2013	epithelial membrane protein 2 [Source:HGNC Symbol;Acc:HGNC:3334]	1.8	2
2313	*EIF4G3*	8672	eukaryotic translation initiation factor 4 gamma 3 [Source:HGNC Symbol;Acc:HGNC:3298]	1.8	2
2314	*MYL4*	4635	myosin light chain 4 [Source:HGNC Symbol;Acc:HGNC:7585]	1.8	2
2315	*SH3BGRL*	6451	SH3 domain binding glutamate rich protein like [Source:HGNC Symbol;Acc:HGNC:10823]	1.8	2
2316	*RHOBTB3*	22836	Rho related BTB domain containing 3 [Source:HGNC Symbol;Acc:HGNC:18757]	1.8	2
2317	*BRD2*	6046	bromodomain containing 2 [Source:HGNC Symbol;Acc:HGNC:1103]	1.8	2
2318	*IGSF10*	285313	immunoglobulin superfamily member 10 [Source:HGNC Symbol;Acc:HGNC:26384]	1.8	2
2319	*CDKL5*	6792	cyclin dependent kinase like 5 [Source:HGNC Symbol;Acc:HGNC:11411]	1.8	2
2320	*TLR1*	7096	toll like receptor 1 [Source:HGNC Symbol;Acc:HGNC:11847]	1.8	2
2321	*P2RY1*	5028	purinergic receptor P2Y1 [Source:HGNC Symbol;Acc:HGNC:8539]	1.8	2
2322	*BCL10*	8915	B-cell CLL/lymphoma 10 [Source:HGNC Symbol;Acc:HGNC:989]	1.8	2
2323	*PGS1*	9489	phosphatidylglycerophosphate synthase 1 [Source:HGNC Symbol;Acc:HGNC:30029]	1.8	2
2324	*TRIM13*	10206	tripartite motif containing 13 [Source:HGNC Symbol;Acc:HGNC:9976]	1.8	2
2325	*SLC25A39*	51629	solute carrier family 25 member 39 [Source:HGNC Symbol;Acc:HGNC:24279]	1.8	2
2326	*ORC6*	23594	origin recognition complex subunit 6 [Source:HGNC Symbol;Acc:HGNC:17151]	1.8	2
2327	*PTAFR*	5724	platelet activating factor receptor [Source:HGNC Symbol;Acc:HGNC:9582]	1.8	2
2328	*CD59*	966	CD59 molecule [Source:HGNC Symbol;Acc:HGNC:1689]	1.7	2
2329	*IRAK4*	51135	interleukin 1 receptor associated kinase 4 [Source:HGNC Symbol;Acc:HGNC:17967]	1.7	2
2330	*FADD*	8772	Fas associated via death domain [Source:HGNC Symbol;Acc:HGNC:3573]	1.7	2
2331	*DNAJC16*	23341	DnaJ heat shock protein family (Hsp40) member C16 [Source:HGNC Symbol;Acc:HGNC:29157]	1.7	2
2332	*TBC1D2*	55357	TBC1 domain family member 2 [Source:HGNC Symbol;Acc:HGNC:18026]	1.7	2
2333	*GLRX3*	10539	glutaredoxin 3 [Source:HGNC Symbol;Acc:HGNC:15987]	1.7	2
2334	*SMAD2*	4087	SMAD family member 2 [Source:HGNC Symbol;Acc:HGNC:6768]	1.7	2
2335	*RPL26L1*	51121	ribosomal protein L26 like 1 [Source:HGNC Symbol;Acc:HGNC:17050]	1.7	2
2336	*GRB14*	2888	growth factor receptor bound protein 14 [Source:HGNC Symbol;Acc:HGNC:4565]	1.7	2
2337	*CHST3*	9469	carbohydrate sulfotransferase 3 [Source:HGNC Symbol;Acc:HGNC:1971]	1.7	2
2338	*AMPD2*	271	adenosine monophosphate deaminase 2 [Source:HGNC Symbol;Acc:HGNC:469]	1.7	2
2339	*RGL2*	5863	ral guanine nucleotide dissociation stimulator like 2 [Source:HGNC Symbol;Acc:HGNC:9769]	1.7	2
2340	*CDK6*	1021	cyclin dependent kinase 6 [Source:HGNC Symbol;Acc:HGNC:1777]	1.7	2
2341	*HEXDC*	284004	hexosaminidase D [Source:HGNC Symbol;Acc:HGNC:26307]	1.7	2
2342	*DNMT3A*	1788	DNA (cytosine-5-)-methyltransferase 3 alpha [Source:HGNC Symbol;Acc:HGNC:2978]	1.7	2
2343	*SGSH*	6448	N-sulfoglucosamine sulfohydrolase [Source:HGNC Symbol;Acc:HGNC:10818]	1.7	2
2344	*ARHGDIA*	396	Rho GDP dissociation inhibitor alpha [Source:HGNC Symbol;Acc:HGNC:678]	1.7	2
2345	*PARP2*	10038	poly(ADP-ribose) polymerase 2 [Source:HGNC Symbol;Acc:HGNC:272]	1.7	2
2346	*BST1*	683	bone marrow stromal cell antigen 1 [Source:HGNC Symbol;Acc:HGNC:1118]	1.7	2
2347	*WLS*	79971	wntless Wnt ligand secretion mediator [Source:HGNC Symbol;Acc:HGNC:30238]	1.7	2
2348	*CACNA2D3*	55799	calcium voltage-gated channel auxiliary subunit alpha2delta 3 [Source:HGNC Symbol;Acc:HGNC:15460]	1.7	2
2349	*SET*	6418	SET nuclear proto-oncogene [Source:HGNC Symbol;Acc:HGNC:10760]	1.7	2
2350	*ATP5C1*	509	ATP synthase, H+ transporting, mitochondrial F1 complex, gamma polypeptide 1 [Source:HGNC Symbol;Acc:HGNC:833]	1.7	2
2351	*KYNU*	8942	kynureninase [Source:HGNC Symbol;Acc:HGNC:6469]	1.7	2
2352	*RAD1*	5810	RAD1 checkpoint DNA exonuclease [Source:HGNC Symbol;Acc:HGNC:9806]	1.7	2
2353	*CNTRL*	11064	centriolin [Source:HGNC Symbol;Acc:HGNC:1858]	1.7	2
2354	*MAPK1IP1L*	93487	mitogen-activated protein kinase 1 interacting protein 1-like [Source:HGNC Symbol;Acc:HGNC:19840]	1.7	2
2355	*ACBD6*	84320	acyl-CoA binding domain containing 6 [Source:HGNC Symbol;Acc:HGNC:23339]	1.7	2
2356	*FBXO11*	80204	F-box protein 11 [Source:HGNC Symbol;Acc:HGNC:13590]	1.7	2
2357	*LMO2*	4005	LIM domain only 2 [Source:HGNC Symbol;Acc:HGNC:6642]	1.7	2
2358	*ARL11*	115761	ADP ribosylation factor like GTPase 11 [Source:HGNC Symbol;Acc:HGNC:24046]	1.7	2
2359	*MTMR9*	66036	myotubularin related protein 9 [Source:HGNC Symbol;Acc:HGNC:14596]	1.7	2
2360	*MKNK2*	2872	MAP kinase interacting serine/threonine kinase 2 [Source:HGNC Symbol;Acc:HGNC:7111]	1.7	2
2361	*SART1*	9092	squamous cell carcinoma antigen recognized by T-cells 1 [Source:HGNC Symbol;Acc:HGNC:10538]	1.7	2
2362	*RPS3*	6188	ribosomal protein S3 [Source:HGNC Symbol;Acc:HGNC:10420]	1.7	2
2363	*MAP2K4*	6416	mitogen-activated protein kinase kinase 4 [Source:HGNC Symbol;Acc:HGNC:6844]	1.7	2
2364	*SLC48A1*	55652	solute carrier family 48 member 1 [Source:HGNC Symbol;Acc:HGNC:26035]	1.7	2
2365	*PHC2*	1912	polyhomeotic homolog 2 [Source:HGNC Symbol;Acc:HGNC:3183]	1.7	2
2366	*TSC22D4*	81628	TSC22 domain family member 4 [Source:HGNC Symbol;Acc:HGNC:21696]	1.7	2
2367	*RPL27A*	6157	ribosomal protein L27a [Source:HGNC Symbol;Acc:HGNC:10329]	1.7	2
2368	*RPUSD1*	113000	RNA pseudouridylate synthase domain containing 1 [Source:HGNC Symbol;Acc:HGNC:14173]	1.7	2
2369	*TMEM165*	55858	transmembrane protein 165 [Source:HGNC Symbol;Acc:HGNC:30760]	1.7	2
2370	*DOLPP1*	57171	dolichyldiphosphatase 1 [Source:HGNC Symbol;Acc:HGNC:29565]	1.7	2
2371	*EOGT*	285203	EGF domain specific O-linked N-acetylglucosamine transferase [Source:HGNC Symbol;Acc:HGNC:28526]	1.7	2
2372	*MKRN1*	23608	makorin ring finger protein 1 [Source:HGNC Symbol;Acc:HGNC:7112]	1.7	2
2373	*MYO15B*	80022	myosin XVB [Source:HGNC Symbol;Acc:HGNC:14083]	1.7	2
2374	*DNAJC15*	29103	DnaJ heat shock protein family (Hsp40) member C15 [Source:HGNC Symbol;Acc:HGNC:20325]	1.7	2
2375	*IGFLR1*	79713	IGF like family receptor 1 [Source:HGNC Symbol;Acc:HGNC:23620]	1.7	2
2376	*PPM1M*	132160	protein phosphatase, Mg2+/Mn2+ dependent 1M [Source:HGNC Symbol;Acc:HGNC:26506]	1.7	2
2377	*LGALS9*	3965	galectin 9 [Source:HGNC Symbol;Acc:HGNC:6570]	1.7	2
2378	*RAB20*	55647	RAB20, member RAS oncogene family [Source:HGNC Symbol;Acc:HGNC:18260]	1.7	2
2379	*ZNF706*	51123	zinc finger protein 706 [Source:HGNC Symbol;Acc:HGNC:24992]	1.7	2
2380	*ALAS2*	212	5'-aminolevulinate synthase 2 [Source:HGNC Symbol;Acc:HGNC:397]	1.7	2
2381	*CORO7*	79585	coronin 7 [Source:HGNC Symbol;Acc:HGNC:26161]	1.7	2

2382	*SYK*	6850	spleen tyrosine kinase [Source:HGNC Symbol;Acc:HGNC:11491]	1.7	2
2383	*C2orf69*	205327	chromosome 2 open reading frame 69 [Source:HGNC Symbol;Acc:HGNC:26799]	1.7	2
2384	*CSNK1A1*	1452	casein kinase 1 alpha 1 [Source:HGNC Symbol;Acc:HGNC:2451]	1.7	2
2385	*LRRC8B*	23507	leucine rich repeat containing 8 family member B [Source:HGNC Symbol;Acc:HGNC:30692]	1.7	2
2386	*RPA1*	6117	replication protein A1 [Source:HGNC Symbol;Acc:HGNC:10289]	1.7	2
2387	*UPF2*	26019	UPF2 regulator of nonsense transcripts homolog (yeast) [Source:HGNC Symbol;Acc:HGNC:17854]	1.7	2
2388	*IER5*	51278	immediate early response 5 [Source:HGNC Symbol;Acc:HGNC:5393]	1.7	2
2389	*IFI27L2*	83982	interferon alpha inducible protein 27 like 2 [Source:HGNC Symbol;Acc:HGNC:19753]	1.7	2
2390	*PHAX*	51808	phosphorylated adaptor for RNA export [Source:HGNC Symbol;Acc:HGNC:10241]	1.7	2
2391	*RGS14*	10636	regulator of G-protein signaling 14 [Source:HGNC Symbol;Acc:HGNC:9996]	1.7	2
2392	*PRR14*	78994	proline rich 14 [Source:HGNC Symbol;Acc:HGNC:28458]	1.7	2
2393	*DBNL*	28988	drebrin like [Source:HGNC Symbol;Acc:HGNC:2696]	1.7	2
2394	*ATRN*	8455	attractin [Source:HGNC Symbol;Acc:HGNC:885]	1.7	2
2395	*SLC31A1*	1317	solute carrier family 31 member 1 [Source:HGNC Symbol;Acc:HGNC:11016]	1.7	2
2396	*TBC1D9B*	23061	TBC1 domain family member 9B [Source:HGNC Symbol;Acc:HGNC:29097]	1.7	2
2397	*C16orf54*	283897	chromosome 16 open reading frame 54 [Source:HGNC Symbol;Acc:HGNC:26649]	1.7	2
2398	*TIMM50*	92609	translocase of inner mitochondrial membrane 50 [Source:HGNC Symbol;Acc:HGNC:23656]	1.7	2
2399	*CD79A*	973	CD79a molecule [Source:HGNC Symbol;Acc:HGNC:1698]	1.7	2
2400	*PTPRE*	5791	protein tyrosine phosphatase, receptor type E [Source:HGNC Symbol;Acc:HGNC:9669]	1.7	2
2401	*KCNE3*	10008	potassium voltage-gated channel subfamily E regulatory subunit 3 [Source:HGNC Symbol;Acc:HGNC:6243]	1.7	2
2402	*NHLRC3*	387921	NHL repeat containing 3 [Source:HGNC Symbol;Acc:HGNC:33751]	1.7	2
2403	*IRF8*	3394	interferon regulatory factor 8 [Source:HGNC Symbol;Acc:HGNC:5358]	1.7	2
2404	*PKM*	5315	pyruvate kinase, muscle [Source:HGNC Symbol;Acc:HGNC:9021]	1.7	2
2405	*RREB1*	6239	ras responsive element binding protein 1 [Source:HGNC Symbol;Acc:HGNC:10449]	1.7	2
2406	*SAMSN1*	64092	SAM domain, SH3 domain and nuclear localization signals 1 [Source:HGNC Symbol;Acc:HGNC:10528]	1.7	2
2407	*COX20*	116228	COX20 cytochrome c oxidase assembly factor [Source:HGNC Symbol;Acc:HGNC:26970]	1.7	2
2408	*C1orf233*	643988	chromosome 1 open reading frame 233	1.7	2
2409	*HMGN3*	9324	high mobility group nucleosomal binding domain 3 [Source:HGNC Symbol;Acc:HGNC:12312]	1.7	2
2410	*YPEL2*	388403	yippee like 2 [Source:HGNC Symbol;Acc:HGNC:18326]	1.7	2
2411	*DEDD2*	162989	death effector domain containing 2 [Source:HGNC Symbol;Acc:HGNC:24450]	1.7	2
2412	*BIN3*	55909	bridging integrator 3 [Source:HGNC Symbol;Acc:HGNC:1054]	1.7	2
2413	*CCAR2*	57805	cell cycle and apoptosis regulator 2 [Source:HGNC Symbol;Acc:HGNC:23360]	1.7	2
2414	*SF3A2*	8175	splicing factor 3a subunit 2 [Source:HGNC Symbol;Acc:HGNC:10766]	1.7	2
2415	*NIPA1*	123606	non imprinted in Prader-Willi/Angelman syndrome 1 [Source:HGNC Symbol;Acc:HGNC:17043]	1.7	2
2416	*TRPM2*	7226	transient receptor potential cation channel subfamily M member 2 [Source:HGNC Symbol;Acc:HGNC:12339]	1.7	2
2417	*HINT1*	3094	histidine triad nucleotide binding protein 1 [Source:HGNC Symbol;Acc:HGNC:4912]	1.7	2
2418	*UBTD2*	92181	ubiquitin domain containing 2 [Source:HGNC Symbol;Acc:HGNC:24463]	1.7	2
2419	*IFFO1*	25900	intermediate filament family orphan 1 [Source:HGNC Symbol;Acc:HGNC:24970]	1.7	2
2420	*RAB7A*	7879	RAB7A, member RAS oncogene family [Source:HGNC Symbol;Acc:HGNC:9788]	1.7	2
2421	*CDK11A*	728642	cyclin dependent kinase 11A [Source:HGNC Symbol;Acc:HGNC:1730]	1.7	2
2422	*VPS8*	23355	VPS8, CORVET complex subunit [Source:HGNC Symbol;Acc:HGNC:29122]	1.7	2
2423	*NOL7*	51406	nucleolar protein 7 [Source:HGNC Symbol;Acc:HGNC:21040]	1.7	2
2424	*GDI2*	2665	GDP dissociation inhibitor 2 [Source:HGNC Symbol;Acc:HGNC:4227]	1.7	2
2425	*ARVCF*	421	armadillo repeat gene deleted in velocardiofacial syndrome [Source:HGNC Symbol;Acc:HGNC:728]	1.7	2
2426	*COMT*	1312	catechol-O-methyltransferase [Source:HGNC Symbol;Acc:HGNC:2228]	1.7	2
2427	*TXNRD2*	10587	thioredoxin reductase 2 [Source:HGNC Symbol;Acc:HGNC:18155]	1.7	2
2428	*GDA*	9615	guanine deaminase [Source:HGNC Symbol;Acc:HGNC:4212]	1.7	2
2429	*TMEM234*	56063	transmembrane protein 234 [Source:HGNC Symbol;Acc:HGNC:28837]	1.7	2
2430	*XRCC2*	7516	X-ray repair cross complementing 2 [Source:HGNC Symbol;Acc:HGNC:12829]	1.7	2
2431	*NDST2*	8509	N-deacetylase/N-sulfotransferase 2 [Source:HGNC Symbol;Acc:HGNC:7681]	1.7	2
2432	*TYROBP*	7305	TYRO protein tyrosine kinase binding protein [Source:HGNC Symbol;Acc:HGNC:12449]	1.7	2
2433	*POLR2E*	5434	polymerase (RNA) II subunit E [Source:HGNC Symbol;Acc:HGNC:9192]	1.7	2
2434	*FCMR*	9214	Fc fragment of IgM receptor [Source:HGNC Symbol;Acc:HGNC:14315]	1.7	2
2435	*MSRB1*	51734	methionine sulfoxide reductase B1 [Source:HGNC Symbol;Acc:HGNC:14133]	1.7	2
2436	*EIF2B3*	8891	eukaryotic translation initiation factor 2B subunit gamma [Source:HGNC Symbol;Acc:HGNC:3259]	1.7	2
2437	*ATG13*	9776	autophagy related 13 [Source:HGNC Symbol;Acc:HGNC:29091]	1.7	2
2438	*CD22*	933	CD22 molecule [Source:HGNC Symbol;Acc:HGNC:1643]	1.7	2
2439	*NDNL2*	56160	NSE3 Homolog, SMC5-SMC6 Complex Component	1.7	2
2440	*NUTF2*	10204	nuclear transport factor 2 [Source:HGNC Symbol;Acc:HGNC:13722]	1.7	2
2441	*TAGAP*	117289	T-cell activation RhoGTPase activating protein [Source:HGNC Symbol;Acc:HGNC:15669]	1.7	2
2442	*THG1L*	54974	tRNA-histidine guanylyltransferase 1 like [Source:HGNC Symbol;Acc:HGNC:26053]	1.7	2
2443	*GRN*	2896	granulin [Source:HGNC Symbol;Acc:HGNC:4601]	1.7	2
2444	*MYO19*	80179	myosin XIX [Source:HGNC Symbol;Acc:HGNC:26234]	1.7	2
2445	*WRB*	7485	tryptophan rich basic protein [Source:HGNC Symbol;Acc:HGNC:12790]	1.7	2
2446	*PRPF38A*	84950	pre-mRNA processing factor 38A [Source:HGNC Symbol;Acc:HGNC:25930]	1.7	2
2447	*TOMM40L*	84134	translocase of outer mitochondrial membrane 40 like [Source:HGNC Symbol;Acc:HGNC:25756]	1.7	2
2448	*NUP214*	8021	nucleoporin 214 [Source:HGNC Symbol;Acc:HGNC:8064]	1.7	2
2449	*RNASET2*	8635	ribonuclease T2 [Source:HGNC Symbol;Acc:HGNC:21686]	1.7	2
2450	*EPB41L3*	23136	erythrocyte membrane protein band 4.1 like 3 [Source:HGNC Symbol;Acc:HGNC:3380]	1.7	2
2451	*PCDH1*	5097	protocadherin 1 [Source:HGNC Symbol;Acc:HGNC:8655]	1.7	2
2452	*DUSP7*	1849	dual specificity phosphatase 7 [Source:HGNC Symbol;Acc:HGNC:3073]	1.7	2
2453	*PIK3CB*	5291	phosphatidylinositol-4,5-bisphosphate 3-kinase catalytic subunit beta [Source:HGNC Symbol;Acc:HGNC:8976]	1.6	2

2454	*MAPK3*	5595	mitogen-activated protein kinase 3 [Source:HGNC Symbol;Acc:HGNC:6877]	1.6	2
2455	*ODF3B*	440836	outer dense fiber of sperm tails 3B [Source:HGNC Symbol;Acc:HGNC:34388]	1.6	2
2456	*JMJD8*	339123	jumonji domain containing 8 [Source:HGNC Symbol;Acc:HGNC:14148]	1.6	2
2457	*VSTM1*	284415	V-set and transmembrane domain containing 1 [Source:HGNC Symbol;Acc:HGNC:29455]	1.6	2
2458	*MFSD7*	84179	major facilitator superfamily domain containing 7 [Source:HGNC Symbol;Acc:HGNC:26177]	1.6	2
2459	*C4BPA*	722	complement component 4 binding protein alpha [Source:HGNC Symbol;Acc:HGNC:1325]	1.6	2
2460	*SCAF11*	9169	SR-related CTD associated factor 11 [Source:HGNC Symbol;Acc:HGNC:10784]	1.6	2
2461	*CCM2*	83605	CCM2 scaffolding protein [Source:HGNC Symbol;Acc:HGNC:21708]	1.6	2
2462	*ZNF319*	57567	zinc finger protein 319 [Source:HGNC Symbol;Acc:HGNC:13644]	1.6	2
2463	*SPATA5*	166378	spermatogenesis associated 5 [Source:HGNC Symbol;Acc:HGNC:18119]	1.6	2
2464	*CHEK1*	1111	checkpoint kinase 1 [Source:HGNC Symbol;Acc:HGNC:1925]	1.6	2
2465	*TALDO1*	6888	transaldolase 1 [Source:HGNC Symbol;Acc:HGNC:11559]	1.6	2
2466	*ATOX1*	475	antioxidant 1 copper chaperone [Source:HGNC Symbol;Acc:HGNC:798]	1.6	2
2467	*PPME1*	51400	protein phosphatase methylesterase 1 [Source:HGNC Symbol;Acc:HGNC:30178]	1.6	2
2468	*CD83*	9308	CD83 molecule [Source:HGNC Symbol;Acc:HGNC:1703]	1.6	2
2469	*TAPBP*	6892	TAP binding protein (tapasin) [Source:HGNC Symbol;Acc:HGNC:11566]	1.6	2
2470	*ST7*	7982	suppression of tumorigenicity 7 [Source:HGNC Symbol;Acc:HGNC:11351]	1.6	2
2471	*PRPF6*	24148	pre-mRNA processing factor 6 [Source:HGNC Symbol;Acc:HGNC:15860]	1.6	2
2472	*SLC9A3R1*	9368	SLC9A3 regulator 1 [Source:HGNC Symbol;Acc:HGNC:11075]	1.6	2
2473	*NOL8*	55035	nucleolar protein 8 [Source:HGNC Symbol;Acc:HGNC:23387]	1.6	2
2474	*TRIM34*	53840	tripartite motif containing 34 [Source:HGNC Symbol;Acc:HGNC:10063]	1.6	2
2475	*TRIM5*	85363	tripartite motif containing 5 [Source:HGNC Symbol;Acc:HGNC:16276]	1.6	2
2476	*TRIM6*	117854	tripartite motif containing 6 [Source:HGNC Symbol;Acc:HGNC:16277]	1.6	2
2477	*CDK2AP1*	8099	cyclin dependent kinase 2 associated protein 1 [Source:HGNC Symbol;Acc:HGNC:14002]	1.6	2
2478	*TRAF7*	84231	TNF receptor associated factor 7 [Source:HGNC Symbol;Acc:HGNC:20456]	1.6	2
2479	*SH2B2*	10603	SH2B adaptor protein 2 [Source:HGNC Symbol;Acc:HGNC:17381]	1.6	2
2480	*PWWP2A*	114825	PWWP domain containing 2A [Source:HGNC Symbol;Acc:HGNC:29406]	1.6	2
2481	*DUS1L*	64118	dihydrouridine synthase 1 like [Source:HGNC Symbol;Acc:HGNC:30086]	1.6	2
2482	*PTPRO*	5800	protein tyrosine phosphatase, receptor type O [Source:HGNC Symbol;Acc:HGNC:9678]	1.6	2
2483	*METTL22*	79091	methyltransferase like 22 [Source:HGNC Symbol;Acc:HGNC:28368]	1.6	2
2484	*TMEM104*	54868	transmembrane protein 104 [Source:HGNC Symbol;Acc:HGNC:25984]	1.6	2
2485	*SEMA3A*	10371	semaphorin 3A [Source:HGNC Symbol;Acc:HGNC:10723]	1.6	2
2486	*RHCE*	6006	Rh blood group CcEe antigens [Source:HGNC Symbol;Acc:HGNC:10008]	1.6	2
2487	*DCUN1D5*	84259	defective in cullin neddylation 1 domain containing 5 [Source:HGNC Symbol;Acc:HGNC:28409]	1.6	2
2488	*IGSF21*	84966	immunoglobin superfamily member 21 [Source:HGNC Symbol;Acc:HGNC:28246]	1.6	2
2489	*CIT*	11113	citron rho-interacting serine/threonine kinase [Source:HGNC Symbol;Acc:HGNC:1985]	1.6	2
2490	*G3BP1*	10146	G3BP stress granule assembly factor 1 [Source:HGNC Symbol;Acc:HGNC:30292]	1.6	2
2491	*WDR37*	22884	WD repeat domain 37 [Source:HGNC Symbol;Acc:HGNC:31406]	1.6	2
2492	*CTDSP1*	58190	CTD small phosphatase 1 [Source:HGNC Symbol;Acc:HGNC:21614]	1.6	2
2493	*TM6SF1*	53346	transmembrane 6 superfamily member 1 [Source:HGNC Symbol;Acc:HGNC:11860]	1.6	2
2494	*ZBTB34*	403341	zinc finger and BTB domain containing 34 [Source:HGNC Symbol;Acc:HGNC:31446]	1.6	2
2495	*ZNF394*	84124	zinc finger protein 394 [Source:HGNC Symbol;Acc:HGNC:18832]	1.6	2
2496	*TBC1D1*	23216	TBC1 domain family member 1 [Source:HGNC Symbol;Acc:HGNC:11578]	1.6	2
2497	*PLCL2*	23228	phospholipase C like 2 [Source:HGNC Symbol;Acc:HGNC:9064]	1.6	2
2498	*CYP2U1*	113612	cytochrome P450 family 2 subfamily U member 1 [Source:HGNC Symbol;Acc:HGNC:20582]	1.6	2
2499	*CDC37*	11140	cell division cycle 37 [Source:HGNC Symbol;Acc:HGNC:1735]	1.6	2
2500	*RAB11A*	8766	RAB11A, member RAS oncogene family [Source:HGNC Symbol;Acc:HGNC:9760]	1.6	2
2501	*RNF115*	27246	ring finger protein 115 [Source:HGNC Symbol;Acc:HGNC:18154]	1.6	2
2502	*OAZ2*	4947	ornithine decarboxylase antizyme 2 [Source:HGNC Symbol;Acc:HGNC:8096]	1.6	2
2503	*ABHD17A*	81926	abhydrolase domain containing 17A [Source:HGNC Symbol;Acc:HGNC:28756]	1.6	2
2504	*SLC9A8*	23315	solute carrier family 9 member A8 [Source:HGNC Symbol;Acc:HGNC:20728]	1.6	2
2505	*SLC12A6*	9990	solute carrier family 12 member 6 [Source:HGNC Symbol;Acc:HGNC:10914]	1.6	2
2506	*CTBS*	1486	chitobiase [Source:HGNC Symbol;Acc:HGNC:2496]	1.6	2
2507	*SLC31A2*	1318	solute carrier family 31 member 2 [Source:HGNC Symbol;Acc:HGNC:11017]	1.6	2
2508	*HIST1H2AM*	8336	histone cluster 1, H2am [Source:HGNC Symbol;Acc:HGNC:4735]	1.6	2
2509	*PDPK1*	5170	3-phosphoinositide dependent protein kinase 1 [Source:HGNC Symbol;Acc:HGNC:8816]	1.6	2
2510	*SF3A1*	10291	splicing factor 3a subunit 1 [Source:HGNC Symbol;Acc:HGNC:10765]	1.6	2
2511	*HIST1H2AA*	221613	histone cluster 1, H2aa [Source:HGNC Symbol;Acc:HGNC:18729]	1.6	2
2512	*RASSF5*	83593	Ras association domain family member 5 [Source:HGNC Symbol;Acc:HGNC:17609]	1.6	2
2513	*LHFPL2*	10184	lipoma HMGIC fusion partner-like 2 [Source:HGNC Symbol;Acc:HGNC:6588]	1.6	2
2514	*SAMHD1*	25939	SAM and HD domain containing deoxynucleoside triphosphate triphosphohydrolase 1 [Source:HGNC Symbol;Acc:HGNC:15925]	1.6	2
2515	*CD163*	9332	CD163 molecule [Source:HGNC Symbol;Acc:HGNC:1631]	1.6	2
2516	*MRPL41*	64975	mitochondrial ribosomal protein L41 [Source:HGNC Symbol;Acc:HGNC:14492]	1.6	2
2517	*TPM3*	7170	tropomyosin 3 [Source:HGNC Symbol;Acc:HGNC:12012]	1.6	2
2518	*UBASH3B*	84959	ubiquitin associated and SH3 domain containing B [Source:HGNC Symbol;Acc:HGNC:29884]	1.6	2
2519	*CXorf21*	80231	chromosome X open reading frame 21 [Source:HGNC Symbol;Acc:HGNC:25667]	1.6	2
2520	*C20orf194*	25943	chromosome 20 open reading frame 194 [Source:HGNC Symbol;Acc:HGNC:17721]	1.6	2
2521	*TUBB*	203068	tubulin beta class I [Source:HGNC Symbol;Acc:HGNC:20778]	1.6	2
2522	*PTRF*	284119	polymerase I and transcript release factor [Source:HGNC Symbol;Acc:HGNC:9688]	1.6	2
2523	*EIF3H*	8667	eukaryotic translation initiation factor 3 subunit H [Source:HGNC Symbol;Acc:HGNC:3273]	1.6	2
2524	*TPTE*	7179	transmembrane phosphatase with tensin homology [Source:HGNC Symbol;Acc:HGNC:12023]	1.6	2
2525	*WAS*	7454	Wiskott-Aldrich syndrome [Source:HGNC Symbol;Acc:HGNC:12731]	1.6	2
2526	*GMNN*	51053	geminin, DNA replication inhibitor [Source:HGNC Symbol;Acc:HGNC:17493]	1.6	2
2527	*SPINK2*	6691	serine peptidase inhibitor, Kazal type 2 [Source:HGNC Symbol;Acc:HGNC:11245]	1.6	2
2528	*DDX18*	8886	DEAD-box helicase 18 [Source:HGNC Symbol;Acc:HGNC:2741]	1.6	2
2529	*ATP8B1*	5205	ATPase phospholipid transporting 8B1 [Source:HGNC Symbol;Acc:HGNC:3706]	1.6	2
2530	*SMIM7*	79086	small integral membrane protein 7 [Source:HGNC Symbol;Acc:HGNC:28419]	1.6	2

2531	*NFATC2IP*	84901	nuclear factor of activated T-cells 2 interacting protein [Source:HGNC Symbol;Acc:HGNC:25906]	1.6	2
2532	*OCIAD1*	54940	OCIA domain containing 1 [Source:HGNC Symbol;Acc:HGNC:16074]	1.6	2
2533	*TDRD9*	122402	tudor domain containing 9 [Source:HGNC Symbol;Acc:HGNC:20122]	1.6	2
2534	*TLK1*	9874	tousled like kinase 1 [Source:HGNC Symbol;Acc:HGNC:11841]	1.6	2
2535	*CNN2*	1265	calponin 2 [Source:HGNC Symbol;Acc:HGNC:2156]	1.6	2
2536	*UBQLN4*	56893	ubiquilin 4 [Source:HGNC Symbol;Acc:HGNC:1237]	1.6	2
2537	*TRAPPC9*	83696	trafficking protein particle complex 9 [Source:HGNC Symbol;Acc:HGNC:30832]	1.6	2
2538	*TOMM40*	10452	translocase of outer mitochondrial membrane 40 [Source:HGNC Symbol;Acc:HGNC:18001]	1.6	2
2539	*OGFR*	11054	opioid growth factor receptor [Source:HGNC Symbol;Acc:HGNC:15768]	1.6	2
2540	*TRIM73*	375593	tripartite motif containing 73 [Source:HGNC Symbol;Acc:HGNC:18162]	1.6	2
2541	*TRAPPC10*	7109	trafficking protein particle complex 10 [Source:HGNC Symbol;Acc:HGNC:11868]	1.6	2
2542	*MPPE1*	65258	metallophosphoesterase 1 [Source:HGNC Symbol;Acc:HGNC:15988]	1.6	2
2543	*MDP1*	145553	magnesium dependent phosphatase 1 [Source:HGNC Symbol;Acc:HGNC:28781]	1.6	2
2544	*NEDD8*	4738	neural precursor cell expressed, developmentally down-regulated 8 [Source:HGNC Symbol;Acc:HGNC:7732]	1.6	2
2545	*NEDD8-MDP1*	100528064	NEDD8-MDP1 readthrough [Source:HGNC Symbol;Acc:HGNC:39551]	1.6	2
2546	*RNH1*	6050	ribonuclease/angiogenin inhibitor 1 [Source:HGNC Symbol;Acc:HGNC:10074]	1.6	2
2547	*FAM166A*	401565	family with sequence similarity 166 member A [Source:HGNC Symbol;Acc:HGNC:33818]	1.6	2
2548	*TUBB4B*	10383	tubulin beta 4B class IVb [Source:HGNC Symbol;Acc:HGNC:20771]	1.6	2
2549	*FEZ1*	9638	fasciculation and elongation protein zeta 1 [Source:HGNC Symbol;Acc:HGNC:3659]	1.6	2
2550	*DENND1A*	57706	DENN domain containing 1A [Source:HGNC Symbol;Acc:HGNC:29324]	1.6	2
2551	*CD40*	958	CD40 molecule [Source:HGNC Symbol;Acc:HGNC:11919]	1.6	2
2552	*NBPF14*	25832	neuroblastoma breakpoint family member 14 [Source:HGNC Symbol;Acc:HGNC:25232]	1.6	2
2553	*KCTD2*	23510	potassium channel tetramerization domain containing 2 [Source:HGNC Symbol;Acc:HGNC:21294]	1.6	2
2554	*BOLA1*	51027	bolA family member 1 [Source:HGNC Symbol;Acc:HGNC:24263]	1.6	2
2555	*SPRED1*	161742	sprouty related EVH1 domain containing 1 [Source:HGNC Symbol;Acc:HGNC:20249]	1.6	2
2556	*CD68*	968	CD68 molecule [Source:HGNC Symbol;Acc:HGNC:1693]	1.6	2
2557	*CHKB*	1120	choline kinase beta [Source:HGNC Symbol;Acc:HGNC:1938]	1.6	2
2558	*ZNF512*	84450	zinc finger protein 512 [Source:HGNC Symbol;Acc:HGNC:29380]	1.6	2
2559	*CDIP1*	29965	cell death-inducing p53 target 1 [Source:HGNC Symbol;Acc:HGNC:13234]	1.6	2
2560	*CCNK*	8812	cyclin K [Source:HGNC Symbol;Acc:HGNC:1596]	1.6	2
2561	*KDM3B*	51780	lysine demethylase 3B [Source:HGNC Symbol;Acc:HGNC:1337]	1.6	2
2562	*PURB*	5814	purine rich element binding protein B [Source:HGNC Symbol;Acc:HGNC:9702]	1.6	2
2563	*DCAF6*	55827	DDB1 and CUL4 associated factor 6 [Source:HGNC Symbol;Acc:HGNC:30002]	1.6	2
2564	*MPC2*	25874	mitochondrial pyruvate carrier 2 [Source:HGNC Symbol;Acc:HGNC:24515]	1.6	2
2565	*GNAS*	2778	GNAS complex locus [Source:HGNC Symbol;Acc:HGNC:4392]	1.6	2
2566	*SORL1*	6653	sortilin-related receptor, L(DLR class) A repeats containing [Source:HGNC Symbol;Acc:HGNC:11185]	1.6	2
2567	*PCK1*	5105	phosphoenolpyruvate carboxykinase 1 [Source:HGNC Symbol;Acc:HGNC:8724]	1.6	2
2568	*CINP*	51550	cyclin dependent kinase 2 interacting protein [Source:HGNC Symbol;Acc:HGNC:23789]	1.6	2
2569	*WFDC2*	10406	WAP four-disulfide core domain 2 [Source:HGNC Symbol;Acc:HGNC:15939]	1.6	2
2570	*MAFB*	9935	MAF bZIP transcription factor B [Source:HGNC Symbol;Acc:HGNC:6408]	1.6	2
2571	*RPL38*	6169	ribosomal protein L38 [Source:HGNC Symbol;Acc:HGNC:10349]	1.6	2
2572	*TUBA1B*	10376	tubulin alpha 1b [Source:HGNC Symbol;Acc:HGNC:18809]	1.6	2
2573	*AKAP13*	11214	A-kinase anchoring protein 13 [Source:HGNC Symbol;Acc:HGNC:371]	1.6	2
2574	*CAPNS1*	826	calpain small subunit 1 [Source:HGNC Symbol;Acc:HGNC:1481]	1.6	2
2575	*MRPS24*	64951	mitochondrial ribosomal protein S24 [Source:HGNC Symbol;Acc:HGNC:14510]	1.6	2
2576	*URGCP*	55665	upregulator of cell proliferation [Source:HGNC Symbol;Acc:HGNC:30890]	1.6	2
2577	*URGCP-MRPS24*	100534592	URGCP-MRPS24 readthrough [Source:HGNC Symbol;Acc:HGNC:49188]	1.6	2
2578	*PFKP*	5214	phosphofructokinase, platelet [Source:HGNC Symbol;Acc:HGNC:8878]	1.6	2
2579	*MGA*	23269	MGA, MAX dimerization protein [Source:HGNC Symbol;Acc:HGNC:14010]	1.6	2
2580	*ACTR3B*	57180	ARP3 actin-related protein 3 homolog B (yeast) [Source:HGNC Symbol;Acc:HGNC:17256]	1.6	2
2581	*CCNY*	219771	cyclin Y [Source:HGNC Symbol;Acc:HGNC:23354]	1.6	2
2582	*DNAJB6*	10049	DnaJ heat shock protein family (Hsp40) member B6 [Source:HGNC Symbol;Acc:HGNC:14888]	1.6	2
2583	*HNRPDL*	9987	Heterogeneous Nuclear Ribonucleoprotein D Like	1.6	2
2584	*WWC3*	55841	WWC family member 3 [Source:HGNC Symbol;Acc:HGNC:29237]	1.6	2
2585	*MYCN*	4613	v-myc avian myelocytomatosis viral oncogene neuroblastoma derived homolog [Source:HGNC Symbol;Acc:HGNC:7559]	1.6	2
2586	*RNF213*	57674	ring finger protein 213 [Source:HGNC Symbol;Acc:HGNC:14539]	1.6	2
2587	*OST4*	100128731	oligosaccharyltransferase complex subunit 4, non-catalytic [Source:HGNC Symbol;Acc:HGNC:32483]	1.6	2
2588	*IP6K2*	51447	inositol hexakisphosphate kinase 2 [Source:HGNC Symbol;Acc:HGNC:17313]	1.6	2
2589	*RAPGEF1*	2889	Rap guanine nucleotide exchange factor 1 [Source:HGNC Symbol;Acc:HGNC:4568]	1.6	2
2590	*SMURF2*	64750	SMAD specific E3 ubiquitin protein ligase 2 [Source:HGNC Symbol;Acc:HGNC:16809]	1.6	2
2591	*PRKG1*	5592	protein kinase, cGMP-dependent, type I [Source:HGNC Symbol;Acc:HGNC:9414]	1.6	2
2592	*RPS27L*	51065	ribosomal protein S27 like [Source:HGNC Symbol;Acc:HGNC:18476]	1.5	2
2593	*ACTB*	60	actin beta [Source:HGNC Symbol;Acc:HGNC:132]	1.5	2
2594	*FUT2*	2524	fucosyltransferase 2 [Source:HGNC Symbol;Acc:HGNC:4013]	1.5	2
2595	*PHC1*	1911	polyhomeotic homolog 1 [Source:HGNC Symbol;Acc:HGNC:3182]	1.5	2
2596	*NRAS*	4893	neuroblastoma RAS viral oncogene homolog [Source:HGNC Symbol;Acc:HGNC:7989]	1.5	2
2597	*USB1*	79650	U6 snRNA biogenesis phosphodiesterase 1 [Source:HGNC Symbol;Acc:HGNC:25792]	1.5	2
2598	*HDGF*	3068	hepatoma-derived growth factor [Source:HGNC Symbol;Acc:HGNC:4856]	1.5	2
2599	*NCKIPSD*	51517	NCK interacting protein with SH3 domain [Source:HGNC Symbol;Acc:HGNC:15486]	1.5	2
2600	*C15orf40*	123207	chromosome 15 open reading frame 40 [Source:HGNC Symbol;Acc:HGNC:28443]	1.5	2
2601	*FAM103A1*	83640	family with sequence similarity 103 member A1 [Source:HGNC Symbol;Acc:HGNC:31022]	1.5	2
2602	*TMEM65*	157378	transmembrane protein 65 [Source:HGNC Symbol;Acc:HGNC:25203]	1.5	2
2603	*SYTL3*	94120	synaptotagmin like 3 [Source:HGNC Symbol;Acc:HGNC:15587]	1.5	2
2604	*SUSD1*	64420	sushi domain containing 1 [Source:HGNC Symbol;Acc:HGNC:25413]	1.5	2
2605	*MFAP3*	4238	microfibrillar associated protein 3 [Source:HGNC Symbol;Acc:HGNC:7034]	1.5	2

2606	*PLCG2*	5336	phospholipase C gamma 2 [Source:HGNC Symbol;Acc:HGNC:9066]	1.5	2
2607	*FOSB*	2354	FosB proto-oncogene, AP-1 transcription factor subunit [Source:HGNC Symbol;Acc:HGNC:3797]	1.5	2
2608	*NKRF*	55922	NFKB repressing factor [Source:HGNC Symbol;Acc:HGNC:19374]	1.5	2
2609	*SFTPB*	6439	surfactant protein B [Source:HGNC Symbol;Acc:HGNC:10801]	1.5	2
2610	*GRAMD1A*	57655	GRAM domain containing 1A [Source:HGNC Symbol;Acc:HGNC:29305]	1.5	2
2611	*RRAGA*	10670	Ras related GTP binding A [Source:HGNC Symbol;Acc:HGNC:16963]	1.5	2
2612	*GFM1*	85476	G elongation factor, mitochondrial 1 [Source:HGNC Symbol;Acc:HGNC:13780]	1.5	2
2613	*KATNB1*	10300	katanin regulatory subunit B1 [Source:HGNC Symbol;Acc:HGNC:6217]	1.5	2
2614	*INPPL1*	3636	inositol polyphosphate phosphatase like 1 [Source:HGNC Symbol;Acc:HGNC:6080]	1.5	2
2615	*NPL*	80896	N-acetylneuraminate pyruvate lyase [Source:HGNC Symbol;Acc:HGNC:16781]	1.5	2
2616	*CEBPD*	1052	CCAAT/enhancer binding protein delta [Source:HGNC Symbol;Acc:HGNC:1835]	1.5	2
2617	*NAA15*	80155	N(alpha)-acetyltransferase 15, NatA auxiliary subunit [Source:HGNC Symbol;Acc:HGNC:30782]	1.5	2
2618	*HUS1*	3364	HUS1 checkpoint clamp component [Source:HGNC Symbol;Acc:HGNC:5309]	1.5	2
2619	*ZNF843*	283933	zinc finger protein 843 [Source:HGNC Symbol;Acc:HGNC:28710]	1.5	2
2620	*SKP1*	6500	S-phase kinase-associated protein 1 [Source:HGNC Symbol;Acc:HGNC:10899]	1.5	2
2621	*DEF6*	50619	DEF6, guanine nucleotide exchange factor [Source:HGNC Symbol;Acc:HGNC:2760]	1.5	2
2622	*MAFG*	4097	MAF bZIP transcription factor G [Source:HGNC Symbol;Acc:HGNC:6781]	1.5	2
2623	*HK1*	3098	hexokinase 1 [Source:HGNC Symbol;Acc:HGNC:4922]	1.5	2
2624	*TUBA1A*	7846	tubulin alpha 1a [Source:HGNC Symbol;Acc:HGNC:20766]	1.5	2
2625	*TWF2*	11344	twinfilin actin binding protein 2 [Source:HGNC Symbol;Acc:HGNC:9621]	1.5	2
2626	*ABI2*	10152	abl interactor 2 [Source:HGNC Symbol;Acc:HGNC:24011]	1.5	2
2627	*DYNLRB1*	83658	dynein light chain roadblock-type 1 [Source:HGNC Symbol;Acc:HGNC:15468]	1.5	2
2628	*RGL1*	23179	ral guanine nucleotide dissociation stimulator like 1 [Source:HGNC Symbol;Acc:HGNC:30281]	1.5	2
2629	*GPR107*	57720	G protein-coupled receptor 107 [Source:HGNC Symbol;Acc:HGNC:17830]	1.5	2
2630	*HIST1H2BB*	3018	histone cluster 1, H2bb [Source:HGNC Symbol;Acc:HGNC:4751]	1.5	2
2631	*IFT122*	55764	intraflagellar transport 122 [Source:HGNC Symbol;Acc:HGNC:13556]	1.5	2
2632	*CCDC112*	153733	coiled-coil domain containing 112 [Source:HGNC Symbol;Acc:HGNC:28599]	1.5	2
2633	*B4GALT4*	8702	beta-1,4-galactosyltransferase 4 [Source:HGNC Symbol;Acc:HGNC:927]	1.5	2
2634	*ZFR*	51663	zinc finger RNA binding protein [Source:HGNC Symbol;Acc:HGNC:17277]	1.5	2
2635	*APOM*	55937	apolipoprotein M [Source:HGNC Symbol;Acc:HGNC:13916]	1.5	2
2636	*ACTN3*	89	actinin alpha 3 (gene/pseudogene) [Source:HGNC Symbol;Acc:HGNC:165]	1.5	2
2637	*HHEX*	3087	hematopoietically expressed homeobox [Source:HGNC Symbol;Acc:HGNC:4901]	1.5	2
2638	*CDKN1A*	1026	cyclin dependent kinase inhibitor 1A [Source:HGNC Symbol;Acc:HGNC:1784]	1.5	2
2639	*LAPTM4B*	55353	lysosomal protein transmembrane 4 beta [Source:HGNC Symbol;Acc:HGNC:13646]	1.5	2
2640	*LY75*	4065	lymphocyte antigen 75 [Source:HGNC Symbol;Acc:HGNC:6729]	1.5	2
2641	*INHBC*	3626	inhibin beta C subunit [Source:HGNC Symbol;Acc:HGNC:6068]	1.5	2
2642	*HSP90AA1*	3320	heat shock protein 90kDa alpha family class A member 1 [Source:HGNC Symbol;Acc:HGNC:5253]	1.5	2
2643	*FANCG*	2189	Fanconi anemia complementation group G [Source:HGNC Symbol;Acc:HGNC:3588]	1.5	2
2644	*TUBB3*	10381	tubulin beta 3 class III [Source:HGNC Symbol;Acc:HGNC:20772]	1.5	2
2645	*IFNAR2*	3455	interferon alpha and beta receptor subunit 2 [Source:HGNC Symbol;Acc:HGNC:5433]	1.5	2
2646	*SMYD3*	64754	SET and MYND domain containing 3 [Source:HGNC Symbol;Acc:HGNC:15513]	1.5	2
2647	*KIAA0226L*	80183	KIAA0226-like [Source:HGNC Symbol;Acc:HGNC:20420]	1.5	2
2648	*CHPT1*	56994	choline phosphotransferase 1 [Source:HGNC Symbol;Acc:HGNC:17852]	1.5	2
2649	*FAM20A*	54757	family with sequence similarity 20 member A [Source:HGNC Symbol;Acc:HGNC:23015]	1.5	2
2650	*ATP6V0D1*	9114	ATPase H+ transporting V0 subunit d1 [Source:HGNC Symbol;Acc:HGNC:13724]	1.5	2
2651	*TAOK1*	57551	TAO kinase 1 [Source:HGNC Symbol;Acc:HGNC:29259]	1.5	2
2652	*MED25*	81857	mediator complex subunit 25 [Source:HGNC Symbol;Acc:HGNC:28845]	1.5	2
2653	*PLS3*	5358	plastin 3 [Source:HGNC Symbol;Acc:HGNC:9091]		2
2654	*DCLK1*	9201	doublecortin like kinase 1 [Source:HGNC Symbol;Acc:HGNC:2700]		2
2655	*LGR5*	8549	leucine rich repeat containing G protein-coupled receptor 5 [Source:HGNC Symbol;Acc:HGNC:4504]		2
2656	*PLAU*	5328	plasminogen activator, urokinase [Source:HGNC Symbol;Acc:HGNC:9052]		2
2657	*PLAUR*	5329	plasminogen activator, urokinase receptor [Source:HGNC Symbol;Acc:HGNC:9053]		2
2658	*ALAS1*	211	5'-aminolevulinate synthase 1 [Source:HGNC Symbol;Acc:HGNC:396]		2
2659	*GAPDH*	2597	glyceraldehyde-3-phosphate dehydrogenase [Source:HGNC Symbol;Acc:HGNC:4141]		2
2660	*TIMP2*	7077	TIMP metallopeptidase inhibitor 2 [Source:HGNC Symbol;Acc:HGNC:11821]		2
2661	*LOXL4*	84171	lysyl oxidase like 4 [Source:HGNC Symbol;Acc:HGNC:17171]		2
2662	*CD44*	960	CD44 molecule (Indian blood group) [Source:HGNC Symbol;Acc:HGNC:1681]		2
2663	*FOLH1*	2346	folate hydrolase (prostate-specific membrane antigen) 1 [Source:HGNC Symbol;Acc:HGNC:3788]		2
2664	*FAP*	2191	fibroblast activation protein alpha [Source:HGNC Symbol;Acc:HGNC:3590]		2
2665	*A4GALT*	53947	alpha 1,4-galactosyltransferase [Source:HGNC Symbol;Acc:HGNC:18149]		2
2666	*ADAM15*	8751	ADAM metallopeptidase domain 15 [Source:HGNC Symbol;Acc:HGNC:193]		2
2667	*ADAMTS1*	9510	ADAM metallopeptidase with thrombospondin type 1 motif 1 [Source:HGNC Symbol;Acc:HGNC:217]		2
2668	*AGRN*	375790	agrin [Source:HGNC Symbol;Acc:HGNC:329]		2
2669	*AKT2*	208	AKT serine/threonine kinase 2 [Source:HGNC Symbol;Acc:HGNC:392]		2
2670	*ALDH1A1*	216	aldehyde dehydrogenase 1 family member A1 [Source:HGNC Symbol;Acc:HGNC:402]		2
2671	*ANGPTL2*	23452	angiopoietin like 2 [Source:HGNC Symbol;Acc:HGNC:490]		2
2672	*ANO1*	55107	anoctamin 1 [Source:HGNC Symbol;Acc:HGNC:21625]		2
2673	*ANXA2*	302	annexin A2 [Source:HGNC Symbol;Acc:HGNC:537]		2
2674	*APCDD1*	147495	adenomatosis polyposis coli down-regulated 1 [Source:HGNC Symbol;Acc:HGNC:15718]		2
2675	*AQP1*	358	aquaporin 1 (Colton blood group) [Source:HGNC Symbol;Acc:HGNC:633]		2
2676	*ARHGAP23*	57636	Rho GTPase activating protein 23 [Source:HGNC Symbol;Acc:HGNC:29293]		2
2677	*ATP1A1*	476	ATPase Na+/K+ transporting subunit alpha 1 [Source:HGNC Symbol;Acc:HGNC:799]		2
2678	*BCAM*	4059	basal cell adhesion molecule (Lutheran blood group) [Source:HGNC Symbol;Acc:HGNC:6722]		2
2679	*BGN*	633	biglycan [Source:HGNC Symbol;Acc:HGNC:1044]		2
2680	*BMPER*	168667	BMP binding endothelial regulator [Source:HGNC Symbol;Acc:HGNC:24154]		2
2681	*CAV1*	857	caveolin 1 [Source:HGNC Symbol;Acc:HGNC:1527]		2

2682	*CCDC3*	83643	coiled-coil domain containing 3 [Source:HGNC Symbol;Acc:HGNC:23813]		2
2683	*PROM1*	8842	prominin 1 [Source:HGNC Symbol;Acc:HGNC:9454]		2
2684	*CD34*	947	CD34 molecule [Source:HGNC Symbol;Acc:HGNC:1662]		2
2685	*CDC27*	996	cell division cycle 27 [Source:HGNC Symbol;Acc:HGNC:1728]		2
2686	*CDH1*	999	cadherin 1 [Source:HGNC Symbol;Acc:HGNC:1748]		2
2687	*CDH11*	1009	cadherin 11 [Source:HGNC Symbol;Acc:HGNC:1750]		2
2688	*CDH12*	1010	cadherin 12 [Source:HGNC Symbol;Acc:HGNC:1751]		2
2689	*CDH13*	1012	cadherin 13 [Source:HGNC Symbol;Acc:HGNC:1753]		2
2690	*CDH5*	1003	cadherin 5 [Source:HGNC Symbol;Acc:HGNC:1764]		2
2691	*CFH*	3075	complement factor H [Source:HGNC Symbol;Acc:HGNC:4883]		2
2692	*CGNL1*	84952	cingulin-like 1 [Source:HGNC Symbol;Acc:HGNC:25931]		2
2693	*PIK3CA*	5290	phosphatidylinositol-4,5-bisphosphate 3-kinase catalytic subunit alpha [Source:HGNC Symbol;Acc:HGNC:8975]		2
2694	*TGFBR1*	7046	transforming growth factor beta receptor 1 [Source:HGNC Symbol;Acc:HGNC:11772]		2
2695	*TFF1*	7031	trefoil factor 1 [Source:HGNC Symbol;Acc:HGNC:11755]		2
2696	*KRT19*	3880	keratin 19 [Source:HGNC Symbol;Acc:HGNC:6436]		2
2697	*SCGB2A2*	4250	secretoglobin family 2A member 2 [Source:HGNC Symbol;Acc:HGNC:7050]		2
2698	*TFF3*	7033	trefoil factor 3 [Source:HGNC Symbol;Acc:HGNC:11757]		2
2699	*SLC39A6*	25800	solute carrier family 39 member 6 [Source:HGNC Symbol;Acc:HGNC:18607]		2
2700	*MGP*	4256	matrix Gla protein [Source:HGNC Symbol;Acc:HGNC:7060]		2
2701	*MUCL1*	118430	mucin like 1 [Source:HGNC Symbol;Acc:HGNC:30588]		2
2702	*XPOT*	11260	exportin for tRNA [Source:HGNC Symbol;Acc:HGNC:12826]		2
2703	*MRPL9*	65005	mitochondrial ribosomal protein L9 [Source:HGNC Symbol;Acc:HGNC:14277]		2
2704	*SCGB2A1*	4246	secretoglobin family 2A member 1 [Source:HGNC Symbol;Acc:HGNC:7051]		2
2705	*SCGB1D2*	10647	secretoglobin, family 1D member 2 [Source:HGNC Symbol;Acc:HGNC:18396]		2
2706	*CRABP2*	1382	cellular retinoic acid binding protein 2 [Source:HGNC Symbol;Acc:HGNC:2339]		2
2707	*MYO18A*	399687	myosin XVIIIA [Source:HGNC Symbol;Acc:HGNC:31104]		2
2708	*CRNKL1*	51340	crooked neck pre-mRNA splicing factor 1 [Source:HGNC Symbol;Acc:HGNC:15762]		2
2709	*TRIAP1*	51499	TP53 regulated inhibitor of apoptosis 1 [Source:HGNC Symbol;Acc:HGNC:26937]		2
2710	*IMP3*	55272	IMP3, U3 small nucleolar ribonucleoprotein [Source:HGNC Symbol;Acc:HGNC:14497]		2
2711	*DERL3*	91319	derlin 3 [Source:HGNC Symbol;Acc:HGNC:14236]		2
2712	*NKD2*	85409	naked cuticle homolog 2 [Source:HGNC Symbol;Acc:HGNC:17046]		2
2713	*HYAL1*	3373	hyaluronoglucosaminidase 1 [Source:HGNC Symbol;Acc:HGNC:5320]		2
2714	*KRT8*	3856	keratin 8 [Source:HGNC Symbol;Acc:HGNC:6446]		2
2715	*SLC7A6*	9057	solute carrier family 7 member 6 [Source:HGNC Symbol;Acc:HGNC:11064]		2
2716	*FSD1*	79187	fibronectin type III and SPRY domain containing 1 [Source:HGNC Symbol;Acc:HGNC:13745]		2
2717	*KAT7*	11143	lysine acetyltransferase 7 [Source:HGNC Symbol;Acc:HGNC:17016]		2
2718	*ZNF497*	162968	zinc finger protein 497 [Source:HGNC Symbol;Acc:HGNC:23714]		2
2719	*LYPLA1*	10434	lysophospholipase I [Source:HGNC Symbol;Acc:HGNC:6737]		2
2720	*ADGRF3*	165082	adhesion G protein-coupled receptor F3 [Source:HGNC Symbol;Acc:HGNC:18989]		2
2721	*NBPF1*	55672	neuroblastoma breakpoint family member 1 [Source:HGNC Symbol;Acc:HGNC:26088]		2
2722	*SPTA1*	6708	spectrin alpha, erythrocytic 1 [Source:HGNC Symbol;Acc:HGNC:11272]		2
2723	*PIGW*	284098	phosphatidylinositol glycan anchor biosynthesis class W [Source:HGNC Symbol;Acc:HGNC:23213]		2
2724	*CLDN4*	1364	claudin 4 [Source:HGNC Symbol;Acc:HGNC:2046]		2
2725	*DIP2A*	23181	disco interacting protein 2 homolog A [Source:HGNC Symbol;Acc:HGNC:17217]		2
2726	*HSPE1*	3336	heat shock protein family E (Hsp10) member 1 [Source:HGNC Symbol;Acc:HGNC:5269]		2
2727	*DTX2*	113878	deltex 2, E3 ubiquitin ligase [Source:HGNC Symbol;Acc:HGNC:15973]		2
2728	*MTG1*	92170	mitochondrial ribosome associated GTPase 1 [Source:HGNC Symbol;Acc:HGNC:32159]		2
2729	*NSUN5*	55695	NOP2/Sun RNA methyltransferase family member 5 [Source:HGNC Symbol;Acc:HGNC:16385]		2
2730	*TAF1C*	9013	TATA-box binding protein associated factor, RNA polymerase I subunit C [Source:HGNC Symbol;Acc:HGNC:11534]		2
2731	*ZBTB48*	3104	zinc finger and BTB domain containing 48 [Source:HGNC Symbol;Acc:HGNC:4930]		2
2732	*ZNF70*	7621	zinc finger protein 70 [Source:HGNC Symbol;Acc:HGNC:13140]		2
2733	*TNFAIP8*	25816	TNF alpha induced protein 8 [Source:HGNC Symbol;Acc:HGNC:17260]		2
2734	*PCTP*	58488	phosphatidylcholine transfer protein [Source:HGNC Symbol;Acc:HGNC:8752]		2
2735	*CTGF*	1490	connective tissue growth factor [Source:HGNC Symbol;Acc:HGNC:2500]		2
2736	*FN1*	2335	fibronectin 1 [Source:HGNC Symbol;Acc:HGNC:3778]		2
2737	*ELN*	2006	elastin [Source:HGNC Symbol;Acc:HGNC:3327]		2
2738	*PODXL*	5420	podocalyxin like [Source:HGNC Symbol;Acc:HGNC:9171]		2
2739	*IGFBP7*	3490	insulin like growth factor binding protein 7 [Source:HGNC Symbol;Acc:HGNC:5476]		2
2740	*HSPG2*	3339	heparan sulfate proteoglycan 2 [Source:HGNC Symbol;Acc:HGNC:5273]		2
2741	*POSTN*	10631	periostin [Source:HGNC Symbol;Acc:HGNC:16953]		2
2742	*COL3A1*	1281	collagen type III alpha 1 chain [Source:HGNC Symbol;Acc:HGNC:2201]		2
2743	*ACKR3*	57007	atypical chemokine receptor 3 [Source:HGNC Symbol;Acc:HGNC:23692]		2
2744	*GJA1*	2697	gap junction protein alpha 1 [Source:HGNC Symbol;Acc:HGNC:4274]		2
2745	*HEG1*	57493	heart development protein with EGF like domains 1 [Source:HGNC Symbol;Acc:HGNC:29227]		2
2746	*HMCN1*	83872	hemicentin 1 [Source:HGNC Symbol;Acc:HGNC:19194]		2
2747	*COL6A1*	1291	collagen type VI alpha 1 [Source:HGNC Symbol;Acc:HGNC:2211]		2
2748	*SLCO2A1*	6578	solute carrier organic anion transporter family member 2A1 [Source:HGNC Symbol;Acc:HGNC:10955]		2
2749	*ENG*	2022	endoglin [Source:HGNC Symbol;Acc:HGNC:3349]		2
2750	*MMP2*	4313	matrix metallopeptidase 2 [Source:HGNC Symbol;Acc:HGNC:7166]		2
2751	*EFEMP1*	2202	EGF containing fibulin like extracellular matrix protein 1 [Source:HGNC Symbol;Acc:HGNC:3218]		2
2752	*SULF1*	23213	sulfatase 1 [Source:HGNC Symbol;Acc:HGNC:20391]		2
2753	*ITGB4*	3691	integrin subunit beta 4 [Source:HGNC Symbol;Acc:HGNC:6158]		2
2754	*DPYSL3*	1809	dihydropyrimidinase like 3 [Source:HGNC Symbol;Acc:HGNC:3015]		2
2755	*LTBP4*	8425	latent transforming growth factor beta binding protein 4 [Source:HGNC Symbol;Acc:HGNC:6717]		2

2756	*WWTR1*	25937	WW domain containing transcription regulator 1 [Source:HGNC Symbol;Acc:HGNC:24042]		2
2757	*RHOJ*	57381	ras homolog family member J [Source:HGNC Symbol;Acc:HGNC:688]		2
2758	*HYAL2*	8692	hyaluronoglucosaminidase 2 [Source:HGNC Symbol;Acc:HGNC:5321]		2
2759	*PLA2G2A*	5320	phospholipase A2 group IIA [Source:HGNC Symbol;Acc:HGNC:9031]		2
2760	*EMP1*	2012	epithelial membrane protein 1 [Source:HGNC Symbol;Acc:HGNC:3333]		2
2761	*ITGA9*	3680	integrin subunit alpha 9 [Source:HGNC Symbol;Acc:HGNC:6145]		2
2762	*LAMB2*	3913	laminin subunit beta 2 [Source:HGNC Symbol;Acc:HGNC:6487]		2
2763	*COL1A2*	1278	collagen type I alpha 2 chain [Source:HGNC Symbol;Acc:HGNC:2198]		2
2764	*ECM1*	1893	extracellular matrix protein 1 [Source:HGNC Symbol;Acc:HGNC:3153]		2
2765	*DKK3*	27122	dickkopf WNT signaling pathway inhibitor 3 [Source:HGNC Symbol;Acc:HGNC:2893]		2
2766	*CRIP2*	1397	cysteine rich protein 2 [Source:HGNC Symbol;Acc:HGNC:2361]		2
2767	*TNXB*	7148	tenascin XB [Source:HGNC Symbol;Acc:HGNC:11976]		2
2768	*SCARA3*	51435	scavenger receptor class A member 3 [Source:HGNC Symbol;Acc:HGNC:19000]		2
2769	*FSTL1*	11167	follistatin like 1 [Source:HGNC Symbol;Acc:HGNC:3972]		2
2770	*MMRN2*	79812	multimerin 2 [Source:HGNC Symbol;Acc:HGNC:19888]		2
2771	*PTPRB*	5787	protein tyrosine phosphatase, receptor type B [Source:HGNC Symbol;Acc:HGNC:9665]		2
2772	*TIE1*	7075	tyrosine kinase with immunoglobulin like and EGF like domains 1 [Source:HGNC Symbol;Acc:HGNC:11809]		2
2773	*NOS3*	4846	nitric oxide synthase 3 [Source:HGNC Symbol;Acc:HGNC:7876]		2
2774	*LRIG1*	26018	leucine rich repeats and immunoglobulin like domains 1 [Source:HGNC Symbol;Acc:HGNC:17360]		2
2775	*TPPP3*	51673	tubulin polymerization promoting protein family member 3 [Source:HGNC Symbol;Acc:HGNC:24162]		2
2776	*GPRC5B*	51704	G protein-coupled receptor class C group 5 member B [Source:HGNC Symbol;Acc:HGNC:13308]		2
2777	*RNASE1*	6035	ribonuclease A family member 1, pancreatic [Source:HGNC Symbol;Acc:HGNC:10044]		2
2778	*PLXND1*	23129	plexin D1 [Source:HGNC Symbol;Acc:HGNC:9107]		2
2779	*COL4A2*	1284	collagen type IV alpha 2 [Source:HGNC Symbol;Acc:HGNC:2203]		2
2780	*FLNC*	2318	filamin C [Source:HGNC Symbol;Acc:HGNC:3756]		2
2781	*SLC29A1*	2030	solute carrier family 29 member 1 (Augustine blood group) [Source:HGNC Symbol;Acc:HGNC:11003]		2
2782	*LOXL2*	4017	lysyl oxidase like 2 [Source:HGNC Symbol;Acc:HGNC:6666]		2
2783	*DKK2*	27123	dickkopf WNT signaling pathway inhibitor 2 [Source:HGNC Symbol;Acc:HGNC:2892]		2
2784	*PLTP*	5360	phospholipid transfer protein [Source:HGNC Symbol;Acc:HGNC:9093]		2
2785	*EHD2*	30846	EH domain containing 2 [Source:HGNC Symbol;Acc:HGNC:3243]		2
2786	*TEK*	7010	TEK receptor tyrosine kinase [Source:HGNC Symbol;Acc:HGNC:11724]		2
2787	*ITGAV*	3685	integrin subunit alpha V [Source:HGNC Symbol;Acc:HGNC:6150]		2
2788	*SERPINE2*	5270	serpin family E member 2 [Source:HGNC Symbol;Acc:HGNC:8951]		2
2789	*LAMA4*	3910	laminin subunit alpha 4 [Source:HGNC Symbol;Acc:HGNC:6484]		2
2790	*TXNDC5*	81567	thioredoxin domain containing 5 [Source:HGNC Symbol;Acc:HGNC:21073]		2
2791	*COL6A2*	1292	collagen type VI alpha 2 [Source:HGNC Symbol;Acc:HGNC:2212]		2
2792	*ADGRF5*	221395	adhesion G protein-coupled receptor F5 [Source:HGNC Symbol;Acc:HGNC:19030]		2
2793	*PLXNA4*	91584	plexin A4 [Source:HGNC Symbol;Acc:HGNC:9102]		2
2794	*PKHD1L1*	93035	polycystic kidney and hepatic disease 1 (autosomal recessive)-like 1 [Source:HGNC Symbol;Acc:HGNC:20313]		2
2795	*IL1R1*	3554	interleukin 1 receptor type 1 [Source:HGNC Symbol;Acc:HGNC:5993]		2
2796	*PERP*	64065	PERP, TP53 apoptosis effector [Source:HGNC Symbol;Acc:HGNC:17637]		2
2797	*CLEC3B*	7123	C-type lectin domain family 3 member B [Source:HGNC Symbol;Acc:HGNC:11891]		2
2798	*FSCN1*	6624	fascin actin-bundling protein 1 [Source:HGNC Symbol;Acc:HGNC:11148]		2
2799	*TNFRSF21*	27242	tumor necrosis factor receptor superfamily member 21 [Source:HGNC Symbol;Acc:HGNC:13469]		2
2800	*DOCK6*	57572	dedicator of cytokinesis 6 [Source:HGNC Symbol;Acc:HGNC:19189]		2
2801	*DSTN*	11034	destrin, actin depolymerizing factor [Source:HGNC Symbol;Acc:HGNC:15750]		2
2802	*LTBP3*	4054	latent transforming growth factor beta binding protein 3 [Source:HGNC Symbol;Acc:HGNC:6716]		2
2803	*COL5A2*	1290	collagen type V alpha 2 chain [Source:HGNC Symbol;Acc:HGNC:2210]		2
2804	*GOLM1*	51280	golgi membrane protein 1 [Source:HGNC Symbol;Acc:HGNC:15451]		2
2805	*MDK*	4192	midkine (neurite growth-promoting factor 2) [Source:HGNC Symbol;Acc:HGNC:6972]		2
2806	*RHOC*	389	ras homolog family member C [Source:HGNC Symbol;Acc:HGNC:669]		2
2807	*LIMS2*	55679	LIM zinc finger domain containing 2 [Source:HGNC Symbol;Acc:HGNC:16084]		2
2808	*FBLN2*	2199	fibulin 2 [Source:HGNC Symbol;Acc:HGNC:3601]		2
2809	*PTMS*	5763	parathymosin [Source:HGNC Symbol;Acc:HGNC:9629]		2
2810	*SYNPO*	11346	synaptopodin [Source:HGNC Symbol;Acc:HGNC:30672]		2
2811	*IL33*	90865	interleukin 33 [Source:HGNC Symbol;Acc:HGNC:16028]		2
2812	*EPHB4*	2050	EPH receptor B4 [Source:HGNC Symbol;Acc:HGNC:3395]		2
2813	*VCAN*	1462	versican [Source:HGNC Symbol;Acc:HGNC:2464]		2
2814	*NPDC1*	56654	neural proliferation, differentiation and control, 1 [Source:HGNC Symbol;Acc:HGNC:7899]		2
2815	*LRRC32*	2615	leucine rich repeat containing 32 [Source:HGNC Symbol;Acc:HGNC:4161]		2
2816	*MRC2*	9902	mannose receptor C type 2 [Source:HGNC Symbol;Acc:HGNC:16875]		2
2817	*FBXL7*	23194	F-box and leucine rich repeat protein 7 [Source:HGNC Symbol;Acc:HGNC:13604]		2
2818	*MEST*	4232	mesoderm specific transcript [Source:HGNC Symbol;Acc:HGNC:7028]		2
2819	*GATA6*	2627	GATA binding protein 6 [Source:HGNC Symbol;Acc:HGNC:4174]		2
2820	*MYOF*	26509	myoferlin [Source:HGNC Symbol;Acc:HGNC:3656]		2
2821	*FOXC1*	2296	forkhead box C1 [Source:HGNC Symbol;Acc:HGNC:3800]		2
2822	*MUC1*	4582	mucin 1, cell surface associated [Source:HGNC Symbol;Acc:HGNC:7508]		2
2823	*CRIP1*	1396	cysteine rich protein 1 [Source:HGNC Symbol;Acc:HGNC:2360]		2
2824	*SEMA3F*	6405	semaphorin 3F [Source:HGNC Symbol;Acc:HGNC:10728]		2
2825	*PIEZO1*	9780	piezo type mechanosensitive ion channel component 1 [Source:HGNC Symbol;Acc:HGNC:28993]		2
2826	*NRG1*	3084	neuregulin 1 [Source:HGNC Symbol;Acc:HGNC:7997]		2
2827	*SLC9A3R2*	9351	SLC9A3 regulator 2 [Source:HGNC Symbol;Acc:HGNC:11076]		2
2828	*DCHS1*	8642	dachsous cadherin-related 1 [Source:HGNC Symbol;Acc:HGNC:13681]		2

2829	*COL5A1*	1289	collagen type V alpha 1 [Source:HGNC Symbol;Acc:HGNC:2209]		2
2830	*PTGIS*	5740	prostaglandin I2 (prostacyclin) synthase [Source:HGNC Symbol;Acc:HGNC:9603]		2
2831	*ROBO4*	54538	roundabout guidance receptor 4 [Source:HGNC Symbol;Acc:HGNC:17985]		2
2832	*TJP1*	7082	tight junction protein 1 [Source:HGNC Symbol;Acc:HGNC:11827]		2
2833	*MYH10*	4628	myosin, heavy chain 10, non-muscle [Source:HGNC Symbol;Acc:HGNC:7568]		2
2834	*FBN1*	2200	fibrillin 1 [Source:HGNC Symbol;Acc:HGNC:3603]		2
2835	*KIAA1522*	57648	KIAA1522 [Source:HGNC Symbol;Acc:HGNC:29301]		2
2836	*FAM234A*	83986	family with sequence similarity 234 member A [Source:HGNC Symbol;Acc:HGNC:14163]		2
2837	*SPTAN1*	6709	spectrin alpha, non-erythrocytic 1 [Source:HGNC Symbol;Acc:HGNC:11273]		2
2838	*PLVAP*	83483	plasmalemma vesicle associated protein [Source:HGNC Symbol;Acc:HGNC:13635]		2
2839	*IL1RL1*	9173	interleukin 1 receptor like 1 [Source:HGNC Symbol;Acc:HGNC:5998]		2
2840	*GATA4*	2626	GATA binding protein 4 [Source:HGNC Symbol;Acc:HGNC:4173]		2
2841	*ADGRL2*	23266	adhesion G protein-coupled receptor L2 [Source:HGNC Symbol;Acc:HGNC:18582]		2
2842	*SFRP5*	6425	secreted frizzled related protein 5 [Source:HGNC Symbol;Acc:HGNC:10779]		2
2843	*NTN1*	9423	netrin 1 [Source:HGNC Symbol;Acc:HGNC:8029]		2
2844	*PALM*	5064	paralemmin [Source:HGNC Symbol;Acc:HGNC:8594]		2
2845	*MAOA*	4128	monoamine oxidase A [Source:HGNC Symbol;Acc:HGNC:6833]		2
2846	*RBMS2*	5939	RNA binding motif single stranded interacting protein 2 [Source:HGNC Symbol;Acc:HGNC:9909]		2
2847	*NFE2L1*	4779	nuclear factor, erythroid 2 like 1 [Source:HGNC Symbol;Acc:HGNC:7781]		2
2848	*MMP28*	79148	matrix metallopeptidase 28 [Source:HGNC Symbol;Acc:HGNC:14366]		2
2849	*FGD5*	152273	FYVE, RhoGEF and PH domain containing 5 [Source:HGNC Symbol;Acc:HGNC:19117]		2
2850	*EPS8*	2059	epidermal growth factor receptor pathway substrate 8 [Source:HGNC Symbol;Acc:HGNC:3420]		2
2851	*MAGED1*	9500	MAGE family member D1 [Source:HGNC Symbol;Acc:HGNC:6813]		2
2852	*PPP2R5B*	5526	protein phosphatase 2 regulatory subunit B'beta [Source:HGNC Symbol;Acc:HGNC:9310]		2
2853	*KIF1C*	10749	kinesin family member 1C [Source:HGNC Symbol;Acc:HGNC:6317]		2
2854	*MARVELD1*	83742	MARVEL domain containing 1 [Source:HGNC Symbol;Acc:HGNC:28674]		2
2855	*IFI27*	3429	interferon alpha inducible protein 27 [Source:HGNC Symbol;Acc:HGNC:5397]		2
2856	*EDN1*	1906	endothelin 1 [Source:HGNC Symbol;Acc:HGNC:3176]		2
2857	*PPIC*	5480	peptidylprolyl isomerase C [Source:HGNC Symbol;Acc:HGNC:9256]		2
2858	*GPX8*	493869	glutathione peroxidase 8 (putative) [Source:HGNC Symbol;Acc:HGNC:33100]		2
2859	*CDH3*	1001	cadherin 3 [Source:HGNC Symbol;Acc:HGNC:1762]		2
2860	*TUSC3*	7991	tumor suppressor candidate 3 [Source:HGNC Symbol;Acc:HGNC:30242]		2
2861	*ESRP2*	80004	epithelial splicing regulatory protein 2 [Source:HGNC Symbol;Acc:HGNC:26152]		2
2862	*AGR2*	10551	anterior gradient 2, protein disulphide isomerase family member [Source:HGNC Symbol;Acc:HGNC:328]		2
2863	*BAIAP2L1*	55971	BAI1 associated protein 2 like 1 [Source:HGNC Symbol;Acc:HGNC:21649]		2
2864	*ERBB2*	2064	erb-b2 receptor tyrosine kinase 2 [Source:HGNC Symbol;Acc:HGNC:3430]		2
2865	*EGFR*	1956	epidermal growth factor receptor [Source:HGNC Symbol;Acc:HGNC:3236]		2
2866	*MLANA*	2315	melan-A [Source:HGNC Symbol;Acc:HGNC:7124]		2
2867	*ABCB5*	340273	ATP binding cassette subfamily B member 5 [Source:HGNC Symbol;Acc:HGNC:46]		2
2868	*MCAM*	4162	melanoma cell adhesion molecule [Source:HGNC Symbol;Acc:HGNC:6934]		2
2869	*TGFB2*	7042	transforming growth factor beta 2 [Source:HGNC Symbol;Acc:HGNC:11768]		2
2870	*PAX3*	5077	paired box 3 [Source:HGNC Symbol;Acc:HGNC:8617]		2
2871	*CEACAM5*	1048	carcinoembryonic antigen related cell adhesion molecule 5 [Source:HGNC Symbol;Acc:HGNC:1817]		2
2872	*KRT7*	3855	keratin 7 [Source:HGNC Symbol;Acc:HGNC:6445]		2
2873	*KRT4*	3851	keratin 4 [Source:HGNC Symbol;Acc:HGNC:6441]		2
2874	*KRT5*	3852	keratin 5 [Source:HGNC Symbol;Acc:HGNC:6442]		2
2875	*KRT6A*	3853	keratin 6A [Source:HGNC Symbol;Acc:HGNC:6443]		2
2876	*KRT13*	3860	keratin 13 [Source:HGNC Symbol;Acc:HGNC:6415]		2
2877	*KRT18*	3875	keratin 18 [Source:HGNC Symbol;Acc:HGNC:6430]		2
2878	*CDX2*	1045	caudal type homeobox 2 [Source:HGNC Symbol;Acc:HGNC:1806]		2
2879	*KRT17*	3872	keratin 17 [Source:HGNC Symbol;Acc:HGNC:6427]		2
2880	*KRAS*	3845	KRAS proto-oncogene, GTPase [Source:HGNC Symbol;Acc:HGNC:6407]		2
2881	*ACSM4*	341392	acyl-CoA synthetase medium-chain family member 4 [Source:HGNC Symbol;Acc:HGNC:32016]		2
2882	*ADAM2*	2515	ADAM metallopeptidase domain 2 [Source:HGNC Symbol;Acc:HGNC:198]		2
2883	*ADCY8*	114	adenylate cyclase 8 (brain) [Source:HGNC Symbol;Acc:HGNC:239]		2
2884	*AGBL4*	84871	ATP/GTP binding protein-like 4 [Source:HGNC Symbol;Acc:HGNC:25892]		2
2885	*AICDA*	57379	activation-induced cytidine deaminase [Source:HGNC Symbol;Acc:HGNC:13203]		2
2886	*AKR1D1*	6718	aldo-keto reductase family 1 member D1 [Source:HGNC Symbol;Acc:HGNC:388]		2
2887	*ALB*	213	albumin [Source:HGNC Symbol;Acc:HGNC:399]		2
2888	*ALG1L*	200810	ALG1, chitobiosyldiphosphodolichol beta-mannosyltransferase-like [Source:HGNC Symbol;Acc:HGNC:33721]		2
2889	*ANKRD34B*	340120	ankyrin repeat domain 34B [Source:HGNC Symbol;Acc:HGNC:33736]		2
2890	*B3GNT4*	79369	UDP-GlcNAc:betaGal beta-1,3-N-acetylglucosaminyltransferase 4 [Source:HGNC Symbol;Acc:HGNC:15683]		2
2891	*BAGE3*	85318	B Melanoma antigen family member 3		2
2892	*BAGE*	574	B Melanoma antigen		2
2893	*C12orf56*	115749	chromosome 12 open reading frame 56 [Source:HGNC Symbol;Acc:HGNC:26967]		2
2894	*C5orf64*	285668	chromosome 5 open reading frame 64 [Source:HGNC Symbol;Acc:HGNC:26744]		2
2895	*CFAP45*	25790	cilia and flagella associated protein 45 [Source:HGNC Symbol;Acc:HGNC:17229]		2
2896	*CCNA2*	890	cyclin A2 [Source:HGNC Symbol;Acc:HGNC:1578]		2
2897	*CDCA3*	83461	cell division cycle associated 3 [Source:HGNC Symbol;Acc:HGNC:14624]		2
2898	*CDK1*	983	cyclin dependent kinase 1 [Source:HGNC Symbol;Acc:HGNC:1722]		2
2899	*CENPE*	1062	centromere protein E [Source:HGNC Symbol;Acc:HGNC:1856]		2
2900	*CENPF*	1063	centromere protein F [Source:HGNC Symbol;Acc:HGNC:1857]		2
2901	*CENPI*	2491	centromere protein I [Source:HGNC Symbol;Acc:HGNC:3968]		2
2902	*CGB2*	114336	chorionic gonadotropin beta subunit 2 [Source:HGNC Symbol;Acc:HGNC:16722]		2
2903	*CHAC2*	494143	ChaC cation transport regulator homolog 2 [Source:HGNC Symbol;Acc:HGNC:32363]		2
2904	*CLEC3A*	10143	C-type lectin domain family 3 member A [Source:HGNC Symbol;Acc:HGNC:2052]		2
2905	*CSAG1*	158511	chondrosarcoma associated gene 1 [Source:HGNC Symbol;Acc:HGNC:24294]		2

2906	*CTAG1B*	1485	cancer/testis antigen 1B [Source:HGNC Symbol;Acc:HGNC:2491]		2
2907	*CTAG2*	30848	cancer/testis antigen 2 [Source:HGNC Symbol;Acc:HGNC:2492]		2
2908	*CYP2J2*	1573	cytochrome P450 family 2 subfamily J member 2 [Source:HGNC Symbol;Acc:HGNC:2634]		2
2909	*DEFB126*	81623	defensin beta 126 [Source:HGNC Symbol;Acc:HGNC:15900]		2
2910	*DSC1*	1823	desmocollin 1 [Source:HGNC Symbol;Acc:HGNC:3035]		2
2911	*ERCC6L*	54821	ERCC excision repair 6 like, spindle assembly checkpoint helicase [Source:HGNC Symbol;Acc:HGNC:20794]		2
2912	*FADS6*	283985	fatty acid desaturase 6 [Source:HGNC Symbol;Acc:HGNC:30459]		2
2913	*FAM177B*	400823	family with sequence similarity 177 member B [Source:HGNC Symbol;Acc:HGNC:34395]		2
2914	*FAM57B*	83723	family with sequence similarity 57 member B [Source:HGNC Symbol;Acc:HGNC:25295]		2
2915	*FAM64A*	54478	family with sequence similarity 64 member A [Source:HGNC Symbol;Acc:HGNC:25483]		2
2916	*GALNT8*	26290	polypeptide N-acetylgalactosaminyltransferase 8 [Source:HGNC Symbol;Acc:HGNC:4130]		2
2917	*GNG13*	51764	G protein subunit gamma 13 [Source:HGNC Symbol;Acc:HGNC:14131]		2
2918	*GOLGA6L1*	283767	golgin A6 family-like 1 [Source:HGNC Symbol;Acc:HGNC:37444]		2
2919	*GOLGA6L6*	727832	golgin A6 family-like 6 [Source:HGNC Symbol;Acc:HGNC:37225]		2
2920	*GRIN2A*	2903	glutamate ionotropic receptor NMDA type subunit 2A [Source:HGNC Symbol;Acc:HGNC:4585]		2
2921	*GTSE1*	51512	G2 and S-phase expressed 1 [Source:HGNC Symbol;Acc:HGNC:13698]		2
2922	*HIST1H4C*	8364	histone cluster 1, H4c [Source:HGNC Symbol;Acc:HGNC:4787]		2
2923	*HIST1H4E*	8367	histone cluster 1, H4e [Source:HGNC Symbol;Acc:HGNC:4790]		2
2924	*HOXB7*	3217	homeobox B7 [Source:HGNC Symbol;Acc:HGNC:5118]		2
2925	*HOXB8*	3218	homeobox B8 [Source:HGNC Symbol;Acc:HGNC:5119]		2
2926	*HOXB9*	3219	homeobox B9 [Source:HGNC Symbol;Acc:HGNC:5120]		2
2927	*HTR1D*	3352	5-hydroxytryptamine receptor 1D [Source:HGNC Symbol;Acc:HGNC:5289]		2
2928	*KCNA6*	3742	potassium voltage-gated channel subfamily A member 6 [Source:HGNC Symbol;Acc:HGNC:6225]		2
2929	*KIAA0101*	9768	KIAA0101 [Source:HGNC Symbol;Acc:HGNC:28961]		2
2930	*KIF14*	9928	kinesin family member 14 [Source:HGNC Symbol;Acc:HGNC:19181]		2
2931	*KIF2C*	11004	kinesin family member 2C [Source:HGNC Symbol;Acc:HGNC:6393]		2
2932	*LDHC*	3948	lactate dehydrogenase C [Source:HGNC Symbol;Acc:HGNC:6544]		2
2933	*MAGEA12*	4111	MAGE family member A12 [Source:HGNC Symbol;Acc:HGNC:6799]		2
2934	*MAGEA1*	4100	MAGE family member A1 [Source:HGNC Symbol;Acc:HGNC:6796]		2
2935	*MAGEA3*	4102	MAGE family member A3 [Source:HGNC Symbol;Acc:HGNC:6801]		2
2936	*MAGEA6*	4105	MAGE family member A6 [Source:HGNC Symbol;Acc:HGNC:6804]		2
2937	*MAGEC1*	9947	MAGE family member C1 [Source:HGNC Symbol;Acc:HGNC:6812]		2
2938	*MELK*	9833	maternal embryonic leucine zipper kinase [Source:HGNC Symbol;Acc:HGNC:16870]		2
2939	*MTRNR2L10*	100463488	MT-RNR2-like 10 [Source:HGNC Symbol;Acc:HGNC:37167]		2
2940	*NANOGNB*	360030	NANOG neighbor homeobox [Source:HGNC Symbol;Acc:HGNC:24958]		2
2941	*NEK2*	4751	NIMA related kinase 2 [Source:HGNC Symbol;Acc:HGNC:7745]		2
2942	*NUF2*	83540	NUF2, NDC80 kinetochore complex component [Source:HGNC Symbol;Acc:HGNC:14621]		2
2943	*OR4K2*	390431	olfactory receptor family 4 subfamily K member 2 [Source:HGNC Symbol;Acc:HGNC:14728]		2
2944	*OR8A1*	390275	olfactory receptor family 8 subfamily A member 1 [Source:HGNC Symbol;Acc:HGNC:8469]		2
2945	*ORC1*	4998	origin recognition complex subunit 1 [Source:HGNC Symbol;Acc:HGNC:8487]		2
2946	*PGA3*	643834	pepsinogen 3, group I (pepsinogen A) [Source:HGNC Symbol;Acc:HGNC:8885]		2
2947	*PGA4*	643847	pepsinogen 4, group I (pepsinogen A) [Source:HGNC Symbol;Acc:HGNC:8886]		2
2948	*PGA5*	5222	pepsinogen 5, group I (pepsinogen A) [Source:HGNC Symbol;Acc:HGNC:8887]		2
2949	*RFX6*	222546	regulatory factor X6 [Source:HGNC Symbol;Acc:HGNC:21478]		2
2950	*RPS10-NUDT3*	100529239	RPS10-NUDT3 readthrough [Source:HGNC Symbol;Acc:HGNC:49181]		2
2951	*SGOL1*	151648	Shugoshin 1		2
2952	*SHISA2*	387914	shisa family member 2 [Source:HGNC Symbol;Acc:HGNC:20366]		2
2953	*SKA3*	221150	spindle and kinetochore associated complex subunit 3 [Source:HGNC Symbol;Acc:HGNC:20262]		2
2954	*SLC13A1*	6561	solute carrier family 13 member 1 [Source:HGNC Symbol;Acc:HGNC:10916]		2
2955	*SLC26A3*	1811	solute carrier family 26 member 3 [Source:HGNC Symbol;Acc:HGNC:3018]		2
2956	*SLC45A2*	51151	solute carrier family 45 member 2 [Source:HGNC Symbol;Acc:HGNC:16472]		2
2957	*SLURP1*	57152	secreted LY6/PLAUR domain containing 1 [Source:HGNC Symbol;Acc:HGNC:18746]		2
2958	*SPINK1*	6690	serine peptidase inhibitor, Kazal type 1 [Source:HGNC Symbol;Acc:HGNC:11244]		2
2959	*STMN4*	81551	stathmin 4 [Source:HGNC Symbol;Acc:HGNC:16078]		2
2960	*SYNGR3*	9143	synaptogyrin 3 [Source:HGNC Symbol;Acc:HGNC:11501]		2
2961	*TEX101*	83639	testis expressed 101 [Source:HGNC Symbol;Acc:HGNC:30722]		2
2962	*TGIF2-C20ORF24*	100527943	TGIF2-C20orf24 readthrough [Source:HGNC Symbol;Acc:HGNC:44664]		2
2963	*TK1*	7083	thymidine kinase 1 [Source:HGNC Symbol;Acc:HGNC:11830]		2
2964	*TM4SF5*	9032	transmembrane 4 L six family member 5 [Source:HGNC Symbol;Acc:HGNC:11857]		2
2965	*TNNI3*	7137	troponin I3, cardiac type [Source:HGNC Symbol;Acc:HGNC:11947]		2
2966	*TNNT1*	7138	troponin T1, slow skeletal type [Source:HGNC Symbol;Acc:HGNC:11948]		2
2967	*TOP2A*	7153	topoisomerase (DNA) II alpha [Source:HGNC Symbol;Acc:HGNC:11989]		2
2968	*TPX2*	22974	TPX2, microtubule nucleation factor [Source:HGNC Symbol;Acc:HGNC:1249]		2
2969	*UBE2T*	29089	ubiquitin conjugating enzyme E2 T [Source:HGNC Symbol;Acc:HGNC:25009]		2
2970	*UGT1A10*	54575	UDP glucuronosyltransferase family 1 member A10 [Source:HGNC Symbol;Acc:HGNC:12531]		2
2971	*UGT2B4*	7363	UDP glucuronosyltransferase family 2 member B4 [Source:HGNC Symbol;Acc:HGNC:12553]		2
2972	*UNC13A*	23025	unc-13 homolog A (C. elegans) [Source:HGNC Symbol;Acc:HGNC:23150]		2
2973	*VTN*	7448	vitronectin [Source:HGNC Symbol;Acc:HGNC:12724]		2
2974	*PTPRN2*	5799	protein tyrosine phosphatase, receptor type N2 [Source:HGNC Symbol;Acc:HGNC:9677]		2
2975	*CXCL13*	10563	C-X-C motif chemokine ligand 13 [Source:HGNC Symbol;Acc:HGNC:10639]		2
2976	*ESR2*	2100	estrogen receptor 2 [Source:HGNC Symbol;Acc:HGNC:3468]		2
2977	*IGF1*	3479	insulin like growth factor 1 [Source:HGNC Symbol;Acc:HGNC:5464]		2
2978	*IGF2*	3481	insulin like growth factor 2 [Source:HGNC Symbol;Acc:HGNC:5466]		2
2979	*WNT5A*	7474	Wnt family member 5A [Source:HGNC Symbol;Acc:HGNC:12784]		2
2980	*IGF1R*	3480	insulin like growth factor 1 receptor [Source:HGNC Symbol;Acc:HGNC:5465]		2

2981	*FZD4*	8322	frizzled class receptor 4 [Source:HGNC Symbol;Acc:HGNC:4042]		2
2982	*WNT11*	7481	Wnt family member 11 [Source:HGNC Symbol;Acc:HGNC:12776]		2
2983	*PTCH1*	5727	patched 1 [Source:HGNC Symbol;Acc:HGNC:9585]		2
2984	*PTCH2*	8643	patched 2 [Source:HGNC Symbol;Acc:HGNC:9586]		2
2985	*FOXP3*	50943	forkhead box P3 [Source:HGNC Symbol;Acc:HGNC:6106]		2
2986	*TGFB3*	7043	transforming growth factor beta 3 [Source:HGNC Symbol;Acc:HGNC:11769]		2
2987	*ZEB1*	6935	zinc finger E-box binding homeobox 1 [Source:HGNC Symbol;Acc:HGNC:11642]		2
2988	*ASPA*	443	aspartoacylase [Source:HGNC Symbol;Acc:HGNC:756]		2
2989	*DAB2IP*	153090	DAB2 interacting protein [Source:HGNC Symbol;Acc:HGNC:17294]		2
2990	*MMP14*	4323	matrix metallopeptidase 14 [Source:HGNC Symbol;Acc:HGNC:7160]		2
2991	*WNT5B*	81029	Wnt family member 5B [Source:HGNC Symbol;Acc:HGNC:16265]		2
2992	*SNAI2*	6591	snail family transcriptional repressor 2 [Source:HGNC Symbol;Acc:HGNC:11094]		2
2993	*MMP7*	4316	matrix metallopeptidase 7 [Source:HGNC Symbol;Acc:HGNC:7174]		2
2994	*SOX9*	6662	SRY-box 9 [Source:HGNC Symbol;Acc:HGNC:11204]		2
2995	*TCF3*	6929	transcription factor 3 [Source:HGNC Symbol;Acc:HGNC:11633]		2
2996	*CTNNB1*	1499	catenin beta 1 [Source:HGNC Symbol;Acc:HGNC:2514]		2
2997	*FZD7*	8324	frizzled class receptor 7 [Source:HGNC Symbol;Acc:HGNC:4045]		2
2998	*GLI3*	2737	GLI family zinc finger 3 [Source:HGNC Symbol;Acc:HGNC:4319]		2
2999	*SHH*	6469	sonic hedgehog [Source:HGNC Symbol;Acc:HGNC:10848]		2
3000	*BMP7*	655	bone morphogenetic protein 7 [Source:HGNC Symbol;Acc:HGNC:1074]		2
3001	*FOXC2*	2303	forkhead box C2 [Source:HGNC Symbol;Acc:HGNC:3801]		2
3002	*FOXA2*	3170	forkhead box A2 [Source:HGNC Symbol;Acc:HGNC:5022]		2
3003	*SRC*	6714	SRC proto-oncogene, non-receptor tyrosine kinase [Source:HGNC Symbol;Acc:HGNC:11283]		2
3004	*ABCG2*	9429	ATP binding cassette subfamily G member 2 (Junior blood group) [Source:HGNC Symbol;Acc:HGNC:74]		2
3005	*THY1*	7070	Thy-1 cell surface antigen [Source:HGNC Symbol;Acc:HGNC:11801]		2
3006	*GPX1*	2876	glutathione peroxidase 1 [Source:HGNC Symbol;Acc:HGNC:4553]		2
3007	*FGF2*	2247	fibroblast growth factor 2 [Source:HGNC Symbol;Acc:HGNC:3676]		2
3008	*BMI1*	648	BMI1 proto-oncogene, polycomb ring finger [Source:HGNC Symbol;Acc:HGNC:1066]		2
3009	*KRT20*	54474	keratin 20 [Source:HGNC Symbol;Acc:HGNC:20412]		2
3010	*PDGFRB*	5159	platelet derived growth factor receptor beta [Source:HGNC Symbol;Acc:HGNC:8804]		2
3011	*PPIA*	5478	Peptidylprolyl Isomerase A		2
3012	*UBB*	7314	ubiquitin B [Source:HGNC Symbol;Acc:HGNC:12463]		2
3013	*CXCR4*	7852	C-X-C motif chemokine receptor 4 [Source:HGNC Symbol;Acc:HGNC:2561]		2
3014	*PIK3R1*	5295	phosphoinositide-3-kinase regulatory subunit 1 [Source:HGNC Symbol;Acc:HGNC:8979]		2
3015	*BAX*	581	BCL2 associated X protein [Source:HGNC Symbol;Acc:HGNC:959]		2
3016	*CASP3*	836	caspase 3 [Source:HGNC Symbol;Acc:HGNC:1504]		2
3017	*RRM1*	6240	ribonucleotide reductase catalytic subunit M1 [Source:HGNC Symbol;Acc:HGNC:10451]		2
3018	*MAPK14*	1432	mitogen-activated protein kinase 14 [Source:HGNC Symbol;Acc:HGNC:6876]		2
3019	*PARP1*	142	poly(ADP-ribose) polymerase 1 [Source:HGNC Symbol;Acc:HGNC:270]		2
3020	*SLC2A1*	6513	solute carrier family 2 member 1 [Source:HGNC Symbol;Acc:HGNC:11005]		2
3021	*TFRC*	7037	transferrin receptor [Source:HGNC Symbol;Acc:HGNC:11763]		2
3022	*CD24*	100133941	CD24 molecule [Source:HGNC Symbol;Acc:HGNC:1645]		2
3023	*ANPEP*	290	alanyl aminopeptidase, membrane [Source:HGNC Symbol;Acc:HGNC:500]		2
3024	*PSCA*	8000	prostate stem cell antigen [Source:HGNC Symbol;Acc:HGNC:9500]		2
3025	*BRAF*	673	B-Raf proto-oncogene, serine/threonine kinase [Source:HGNC Symbol;Acc:HGNC:1097]		2
3026	*GDF15*	9518	growth differentiation factor 15 [Source:HGNC Symbol;Acc:HGNC:30142]		2
3027	*RELA*	5970	RELA proto-oncogene, NF-kB subunit [Source:HGNC Symbol;Acc:HGNC:9955]		2
3028	*RUNX1*	861	runt related transcription factor 1 [Source:HGNC Symbol;Acc:HGNC:10471]		2
3029	*STS*	412	steroid sulfatase (microsomal), isozyme S [Source:HGNC Symbol;Acc:HGNC:11425]		2
3030	*ETV5*	2119	ETS variant 5 [Source:HGNC Symbol;Acc:HGNC:3494]		2
3031	*SNAI1*	6615	snail family transcriptional repressor 1 [Source:HGNC Symbol;Acc:HGNC:11128]		2
3032	*KRT14*	3861	keratin 14 [Source:HGNC Symbol;Acc:HGNC:6416]		2
3033	*GABPB1*	2553	GA binding protein transcription factor beta subunit 1 [Source:HGNC Symbol;Acc:HGNC:4074]		2
3034	*YWHAB*	7529	tyrosine 3-monooxygenase/tryptophan 5-monooxygenase activation protein beta [Source:HGNC Symbol;Acc:HGNC:12849]		2
3035	*PRDX1*	5052	peroxiredoxin 1 [Source:HGNC Symbol;Acc:HGNC:9352]		2
3036	*TMEM70*	54968	transmembrane protein 70 [Source:HGNC Symbol;Acc:HGNC:26050]		2
3037	*CDKN2D*	1032	cyclin dependent kinase inhibitor 2D [Source:HGNC Symbol;Acc:HGNC:1790]		2
3038	*NFYA*	4800	nuclear transcription factor Y subunit alpha [Source:HGNC Symbol;Acc:HGNC:7804]		2
3039	*PTBP3*	9991	polypyrimidine tract binding protein 3 [Source:HGNC Symbol;Acc:HGNC:10253]		2
3040	*HMGN2*	3151	high mobility group nucleosomal binding domain 2 [Source:HGNC Symbol;Acc:HGNC:4986]		2
3041	*TMED10*	10972	transmembrane p24 trafficking protein 10 [Source:HGNC Symbol;Acc:HGNC:16998]		2
3042	*KPNA4*	3840	karyopherin subunit alpha 4 [Source:HGNC Symbol;Acc:HGNC:6397]		2
3043	*SRSF6*	6431	serine and arginine rich splicing factor 6 [Source:HGNC Symbol;Acc:HGNC:10788]		2
3044	*JUNB*	3726	JunB proto-oncogene, AP-1 transcription factor subunit [Source:HGNC Symbol;Acc:HGNC:6205]		2
3045	*DHX58*	79132	DEXH-box helicase 58 [Source:HGNC Symbol;Acc:HGNC:29517]		2
3046	*GLO1*	2739	glyoxalase I [Source:HGNC Symbol;Acc:HGNC:4323]		2
3047	*EIF6*	3692	eukaryotic translation initiation factor 6 [Source:HGNC Symbol;Acc:HGNC:6159]		2
3048	*WDR83OS*	51398	WD repeat domain 83 opposite strand [Source:HGNC Symbol;Acc:HGNC:30203]		2
3049	*BECN1*	8678	beclin 1 [Source:HGNC Symbol;Acc:HGNC:1034]		2
3050	*HNRNPU*	3192	heterogeneous nuclear ribonucleoprotein U [Source:HGNC Symbol;Acc:HGNC:5048]		2
3051	*PRKAR1A*	5573	protein kinase cAMP-dependent type I regulatory subunit alpha [Source:HGNC Symbol;Acc:HGNC:9388]		2
3052	*TERT*	7015	telomerase reverse transcriptase [Source:HGNC Symbol;Acc:HGNC:11730]		2
3053	*SKP2*	6502	S-phase kinase-associated protein 2, E3 ubiquitin protein ligase [Source:HGNC Symbol;Acc:HGNC:10901]		2
3054	*CD274*	29126	CD274 molecule [Source:HGNC Symbol;Acc:HGNC:17635]		2
3055	*KLK3*	354	kallikrein related peptidase 3 [Source:HGNC Symbol;Acc:HGNC:6364]		2
3056	*FAM83A*	84985	family with sequence similarity 83 member A [Source:HGNC Symbol;Acc:HGNC:28210]		2

3057	*NPY1R*	4886	neuropeptide Y receptor Y1 [Source:HGNC Symbol;Acc:HGNC:7956]		2
3058	*ERBB4*	2066	erb-b2 receptor tyrosine kinase 4 [Source:HGNC Symbol;Acc:HGNC:3432]		2
3059	*IGFBP5*	3488	insulin like growth factor binding protein 5 [Source:HGNC Symbol;Acc:HGNC:5474]		2
3060	*TOX3*	27324	TOX high mobility group box family member 3 [Source:HGNC Symbol;Acc:HGNC:11972]		2
3061	*PIP*	5304	prolactin induced protein [Source:HGNC Symbol;Acc:HGNC:8993]		2
3062	*PGR*	5241	progesterone receptor [Source:HGNC Symbol;Acc:HGNC:8910]		2
3063	*SERPINB5*	5268	serpin family B member 5 [Source:HGNC Symbol;Acc:HGNC:8949]		2
3064	*CD47*	961	CD47 molecule [Source:HGNC Symbol;Acc:HGNC:1682]		2
3065	*A1CF*	29974	APOBEC1 complementation factor [Source:HGNC Symbol;Acc:HGNC:24086]		2
3066	*ABCC2*	1244	ATP binding cassette subfamily C member 2 [Source:HGNC Symbol;Acc:HGNC:53]		2
3067	*ACSM2B*	348158	acyl-CoA synthetase medium-chain family member 2B [Source:HGNC Symbol;Acc:HGNC:30931]		2
3068	*ACSM3*	6296	acyl-CoA synthetase medium-chain family member 3 [Source:HGNC Symbol;Acc:HGNC:10522]		2
3069	*AKAP2*	11217	A-kinase anchoring protein 2 [Source:HGNC Symbol;Acc:HGNC:372]		2
3070	*ANKRD1*	27063	ankyrin repeat domain 1 [Source:HGNC Symbol;Acc:HGNC:15819]		2
3071	*C8B*	732	complement component 8, beta polypeptide [Source:HGNC Symbol;Acc:HGNC:1353]		2
3072	*CDH4*	1002	cadherin 4 [Source:HGNC Symbol;Acc:HGNC:1763]		2
3073	*DLL3*	10683	delta like canonical Notch ligand 3 [Source:HGNC Symbol;Acc:HGNC:2909]		2
3074	*DSCAM*	1826	DS cell adhesion molecule [Source:HGNC Symbol;Acc:HGNC:3039]		2
3075	*FAM81A*	145773	family with sequence similarity 81 member A [Source:HGNC Symbol;Acc:HGNC:28379]		2
3076	*GBX2*	2637	gastrulation brain homeobox 2 [Source:HGNC Symbol;Acc:HGNC:4186]		2
3077	*GCKR*	2646	glucokinase (hexokinase 4) regulator [Source:HGNC Symbol;Acc:HGNC:4196]		2
3078	*ICAM5*	7087	intercellular adhesion molecule 5 [Source:HGNC Symbol;Acc:HGNC:5348]		2
3079	*NMRK2*	27231	nicotinamide riboside kinase 2 [Source:HGNC Symbol;Acc:HGNC:17871]		2
3080	*MYH6*	4624	myosin, heavy chain 6, cardiac muscle, alpha [Source:HGNC Symbol;Acc:HGNC:7576]		2
3081	*NOG*	9241	noggin [Source:HGNC Symbol;Acc:HGNC:7866]		2
3082	*NR2E1*	7101	nuclear receptor subfamily 2 group E member 1 [Source:HGNC Symbol;Acc:HGNC:7973]		2
3083	*NRN1*	51299	neuritin 1 [Source:HGNC Symbol;Acc:HGNC:17972]		2
3084	*PDE3B*	5140	phosphodiesterase 3B [Source:HGNC Symbol;Acc:HGNC:8779]		2
3085	*TMPRSS15*	5651	transmembrane protease, serine 15 [Source:HGNC Symbol;Acc:HGNC:9490]		2
3086	*PTPRZ1*	5803	protein tyrosine phosphatase, receptor type Z1 [Source:HGNC Symbol;Acc:HGNC:9685]		2
3087	*RBFOX1*	54715	RNA binding protein, fox-1 homolog 1 [Source:HGNC Symbol;Acc:HGNC:18222]		2
3088	*TEAD1*	7003	TEA domain transcription factor 1 [Source:HGNC Symbol;Acc:HGNC:11714]		2
3089	*TLX2*	3196	T-cell leukemia homeobox 2 [Source:HGNC Symbol;Acc:HGNC:5057]		2
3090	*TMEM121*	80757	transmembrane protein 121 [Source:HGNC Symbol;Acc:HGNC:20511]		2
3091	*VRTN*	55237	vertebrae development associated [Source:HGNC Symbol;Acc:HGNC:20223]		2
3092	*CGB*	1082	chorionic gonadotropin beta subunit 1		2
3093	*GNRH1*	2796	gonadotropin releasing hormone 1 [Source:HGNC Symbol;Acc:HGNC:4419]		2
3094	*CCNE2*	9134	cyclin E2 [Source:HGNC Symbol;Acc:HGNC:1590]		2
3095	*MAL2*	114569	mal, T-cell differentiation protein 2 (gene/pseudogene) [Source:HGNC Symbol;Acc:HGNC:13634]		2
3096	*SLC6A8*	6535	Solute Carrier Family 6 Member 8		2
3097	*CCND1*	595	cyclin D1 [Source:HGNC Symbol;Acc:HGNC:1582]		2
3098	*CLDN3*	1365	claudin 3 [Source:HGNC Symbol;Acc:HGNC:2045]		2
3099	*CXCL14*	9547	C-X-C motif chemokine ligand 14 [Source:HGNC Symbol;Acc:HGNC:10640]		2
3100	*DTL*	51514	denticleless E3 ubiquitin protein ligase homolog [Source:HGNC Symbol;Acc:HGNC:30288]		2
3101	*DTX3*	196403	deltex 3, E3 ubiquitin ligase [Source:HGNC Symbol;Acc:HGNC:24457]		2
3102	*DUSP4*	1846	dual specificity phosphatase 4 [Source:HGNC Symbol;Acc:HGNC:3070]		2
3103	*EEF1A2*	1917	eukaryotic translation elongation factor 1 alpha 2 [Source:HGNC Symbol;Acc:HGNC:3192]		2
3104	*ERBB3*	2065	erb-b2 receptor tyrosine kinase 3 [Source:HGNC Symbol;Acc:HGNC:3431]		2
3105	*ESR1*	2099	estrogen receptor 1 [Source:HGNC Symbol;Acc:HGNC:3467]		2
3106	*FEN1*	2237	flap structure-specific endonuclease 1 [Source:HGNC Symbol;Acc:HGNC:3650]		2
3107	*FGFR3*	2261	fibroblast growth factor receptor 3 [Source:HGNC Symbol;Acc:HGNC:3690]		2
3108	*FGFR4*	2264	fibroblast growth factor receptor 4 [Source:HGNC Symbol;Acc:HGNC:3691]		2
3109	*FKBP10*	60681	FK506 binding protein 10 [Source:HGNC Symbol;Acc:HGNC:18169]		2
3110	*FOXA1*	3169	forkhead box A1 [Source:HGNC Symbol;Acc:HGNC:5021]		2
3111	*IGFBP2*	3485	insulin like growth factor binding protein 2 [Source:HGNC Symbol;Acc:HGNC:5471]		2
3112	*IGFBP4*	3487	insulin like growth factor binding protein 4 [Source:HGNC Symbol;Acc:HGNC:5473]		2
3113	*KIF11*	3832	kinesin family member 11 [Source:HGNC Symbol;Acc:HGNC:6388]		2
3114	*KPNA2*	3838	karyopherin subunit alpha 2 [Source:HGNC Symbol;Acc:HGNC:6395]		2
3115	*LAD1*	3898	ladinin 1 [Source:HGNC Symbol;Acc:HGNC:6472]		2
3116	*PKP3*	11187	plakophilin 3 [Source:HGNC Symbol;Acc:HGNC:9025]		2
3117	*PLOD2*	5352	procollagen-lysine,2-oxoglutarate 5-dioxygenase 2 [Source:HGNC Symbol;Acc:HGNC:9082]		2
3118	*S100A7*	6278	S100 calcium binding protein A7 [Source:HGNC Symbol;Acc:HGNC:10497]		2
3119	*SPDEF*	25803	SAM pointed domain containing ETS transcription factor [Source:HGNC Symbol;Acc:HGNC:17257]		2
3120	*TIMP3*	7078	TIMP metallopeptidase inhibitor 3 [Source:HGNC Symbol;Acc:HGNC:11822]		2
3121	*TSPAN13*	27075	tetraspanin 13 [Source:HGNC Symbol;Acc:HGNC:21643]		2
3122	*HMBS*	3145	hydroxymethylbilane synthase [Source:HGNC Symbol;Acc:HGNC:4982]		2
3123	*GABRP*	2568	gamma-aminobutyric acid type A receptor pi subunit [Source:HGNC Symbol;Acc:HGNC:4089]		2
3124	*TST*	7263	thiosulfate sulfurtransferase [Source:HGNC Symbol;Acc:HGNC:12388]		2
3125	*S100A13*	6284	S100 calcium binding protein A13 [Source:HGNC Symbol;Acc:HGNC:10490]		2
3126	*S100A14*	57402	S100 calcium binding protein A14 [Source:HGNC Symbol;Acc:HGNC:18901]		2
3127	*FABP1*	2168	fatty acid binding protein 1 [Source:HGNC Symbol;Acc:HGNC:3555]		2
3128	*SIL1*	64374	SIL1 nucleotide exchange factor [Source:HGNC Symbol;Acc:HGNC:24624]		2
3129	*HPN*	3249	hepsin [Source:HGNC Symbol;Acc:HGNC:5155]		2
3130	*KLK2*	3817	kallikrein related peptidase 2 [Source:HGNC Symbol;Acc:HGNC:6363]		2
3131	*SLC2A10*	81031	solute carrier family 2 member 10 [Source:HGNC Symbol;Acc:HGNC:13444]		2
3132	*MSMB*	4477	microseminoprotein, beta- [Source:HGNC Symbol;Acc:HGNC:7372]		2
3133	*MAOB*	4129	monoamine oxidase B [Source:HGNC Symbol;Acc:HGNC:6834]		2
3134	*BCL2*	596	B-cell CLL/lymphoma 2 [Source:HGNC Symbol;Acc:HGNC:990]		2

3135	*RYK*	6259	receptor-like tyrosine kinase [Source:HGNC Symbol;Acc:HGNC:10481]		2
3136	*TYRO3*	7301	TYRO3 protein tyrosine kinase [Source:HGNC Symbol;Acc:HGNC:12446]		2
3137	*ROR1*	4919	receptor tyrosine kinase-like orphan receptor 1 [Source:HGNC Symbol;Acc:HGNC:10256]		2
3138	*ROR2*	4920	receptor tyrosine kinase like orphan receptor 2 [Source:HGNC Symbol;Acc:HGNC:10257]		2
3139	*EPHA1*	2041	EPH receptor A1 [Source:HGNC Symbol;Acc:HGNC:3385]		2
3140	*EPHB2*	2048	EPH receptor B2 [Source:HGNC Symbol;Acc:HGNC:3393]		2
3141	*FGFR2*	2263	fibroblast growth factor receptor 2 [Source:HGNC Symbol;Acc:HGNC:3689]		2
3142	*CSNK2A1*	1457	casein kinase 2 alpha 1 [Source:HGNC Symbol;Acc:HGNC:2457]		2
3143	*KRT9*	3857	keratin 9 [Source:HGNC Symbol;Acc:HGNC:6447]		2
3144	*KRT16*	3868	keratin 16 [Source:HGNC Symbol;Acc:HGNC:6423]		2
3145	*COL24A1*	255631	collagen type XXIV alpha 1 [Source:HGNC Symbol;Acc:HGNC:20821]		2
3146	*LGALS3*	3958	galectin 3 [Source:HGNC Symbol;Acc:HGNC:6563]		2
3147	*SMC3*	9126	structural maintenance of chromosomes 3 [Source:HGNC Symbol;Acc:HGNC:2468]		2
3148	*ADAMTSL3*	57188	ADAMTS like 3 [Source:HGNC Symbol;Acc:HGNC:14633]		2
3149	*LTBP2*	4053	latent transforming growth factor beta binding protein 2 [Source:HGNC Symbol;Acc:HGNC:6715]		2
3150	*SLC1A3*	6507	solute carrier family 1 member 3 [Source:HGNC Symbol;Acc:HGNC:10941]		2
3151	*COL15A1*	1306	collagen type XV alpha 1 chain [Source:HGNC Symbol;Acc:HGNC:2192]		2
3152	*ERBB2IP*	55914	erb-B2 Receptor Tyrosine Kinase 2		2
3153	*VWF*	7450	von Willebrand factor [Source:HGNC Symbol;Acc:HGNC:12726]		2
3154	*SPOCK1*	6695	sparc/osteonectin, cwcv and kazal-like domains proteoglycan (testican) 1 [Source:HGNC Symbol;Acc:HGNC:11251]		2
3155	*TFIP11*	24144	tuftelin interacting protein 11 [Source:HGNC Symbol;Acc:HGNC:17165]		2
3156	*LAMA1*	284217	laminin subunit alpha 1 [Source:HGNC Symbol;Acc:HGNC:6481]		2
3157	*FRAS1*	80144	Fraser extracellular matrix complex subunit 1 [Source:HGNC Symbol;Acc:HGNC:19185]		2
3158	*GFOD2*	81577	glucose-fructose oxidoreductase domain containing 2 [Source:HGNC Symbol;Acc:HGNC:28159]		2
3159	*LAMA3*	3909	laminin subunit alpha 3 [Source:HGNC Symbol;Acc:HGNC:6483]		2
3160	*NID2*	22795	nidogen 2 [Source:HGNC Symbol;Acc:HGNC:13389]		2
3161	*TECTA*	7007	tectorin alpha [Source:HGNC Symbol;Acc:HGNC:11720]		2
3162	*USH2A*	7399	usherin [Source:HGNC Symbol;Acc:HGNC:12601]		2
3163	*COL4A3*	1285	collagen type IV alpha 3 chain [Source:HGNC Symbol;Acc:HGNC:2204]		2
3164	*LAMC2*	3918	laminin subunit gamma 2 [Source:HGNC Symbol;Acc:HGNC:6493]		2
3165	*ADAMTS3*	9508	ADAM metallopeptidase with thrombospondin type 1 motif 3 [Source:HGNC Symbol;Acc:HGNC:219]		2
3166	*ADAMTS5*	11096	ADAM metallopeptidase with thrombospondin type 1 motif 5 [Source:HGNC Symbol;Acc:HGNC:221]		2
3167	*EGFLAM*	133584	EGF like, fibronectin type III and laminin G domains [Source:HGNC Symbol;Acc:HGNC:26810]		2
3168	*MAMDC2*	256691	MAM domain containing 2 [Source:HGNC Symbol;Acc:HGNC:23673]		2
3169	*COL6A6*	131873	collagen type VI alpha 6 [Source:HGNC Symbol;Acc:HGNC:27023]		2
3170	*COL4A1*	1282	collagen type IV alpha 1 chain [Source:HGNC Symbol;Acc:HGNC:2202]		2
3171	*COL8A1*	1295	collagen type VIII alpha 1 [Source:HGNC Symbol;Acc:HGNC:2215]		2
3172	*COL12A1*	1303	collagen type XII alpha 1 chain [Source:HGNC Symbol;Acc:HGNC:2188]		2
3173	*COL14A1*	7373	collagen type XIV alpha 1 chain [Source:HGNC Symbol;Acc:HGNC:2191]		2
3174	*COL19A1*	1310	collagen type XIX alpha 1 chain [Source:HGNC Symbol;Acc:HGNC:2196]		2
3175	*COL17A1*	1308	collagen type XVII alpha 1 [Source:HGNC Symbol;Acc:HGNC:2194]		2
3176	*COL22A1*	169044	collagen type XXII alpha 1 [Source:HGNC Symbol;Acc:HGNC:22989]		2
3177	*ENTPD1*	953	ectonucleoside triphosphate diphosphohydrolase 1 [Source:HGNC Symbol;Acc:HGNC:3363]		2
3178	*FBLN1*	2192	fibulin 1 [Source:HGNC Symbol;Acc:HGNC:3600]		2
3179	*IMPG1*	3617	interphotoreceptor matrix proteoglycan 1 [Source:HGNC Symbol;Acc:HGNC:6055]		2
3180	*LAMB3*	3914	laminin subunit beta 3 [Source:HGNC Symbol;Acc:HGNC:6490]		2
3181	*MMP1*	4312	matrix metallopeptidase 1 [Source:HGNC Symbol;Acc:HGNC:7155]		2
3182	*OGN*	4969	osteoglycin [Source:HGNC Symbol;Acc:HGNC:8126]		2
3183	*PRELP*	5549	proline and arginine rich end leucine rich repeat protein [Source:HGNC Symbol;Acc:HGNC:9357]		2
3184	*WNT10A*	80326	Wnt family member 10A [Source:HGNC Symbol;Acc:HGNC:13829]		2
3185	*ZP2*	7783	zona pellucida glycoprotein 2 [Source:HGNC Symbol;Acc:HGNC:13188]		2
3186	*LGALS3BP*	3959	galectin 3 binding protein [Source:HGNC Symbol;Acc:HGNC:6564]		2
3187	*DAG1*	1605	dystroglycan 1 [Source:HGNC Symbol;Acc:HGNC:2666]		2
3188	*MMP24*	10893	matrix metallopeptidase 24 [Source:HGNC Symbol;Acc:HGNC:7172]		2
3189	*MMP19*	4327	matrix metallopeptidase 19 [Source:HGNC Symbol;Acc:HGNC:7165]		2
3190	*CALR*	811	calreticulin [Source:HGNC Symbol;Acc:HGNC:1455]		2
3191	*CRTAP*	10491	cartilage associated protein [Source:HGNC Symbol;Acc:HGNC:2379]		2
3192	*MFAP1*	4236	microfibrillar associated protein 1 [Source:HGNC Symbol;Acc:HGNC:7032]		2
3193	*DST*	667	dystonin [Source:HGNC Symbol;Acc:HGNC:1090]		2
3194	*THSD4*	79875	thrombospondin type 1 domain containing 4 [Source:HGNC Symbol;Acc:HGNC:25835]		2
3195	*ADAMTS15*	170689	ADAM metallopeptidase with thrombospondin type 1 motif 15 [Source:HGNC Symbol;Acc:HGNC:16305]		2
3196	*COL11A1*	1301	collagen type XI alpha 1 chain [Source:HGNC Symbol;Acc:HGNC:2186]		2
3197	*NTN4*	59277	netrin 4 [Source:HGNC Symbol;Acc:HGNC:13658]		2
3198	*NAV2*	89797	neuron navigator 2 [Source:HGNC Symbol;Acc:HGNC:15997]		2
3199	*SPON2*	10417	spondin 2 [Source:HGNC Symbol;Acc:HGNC:11253]		2
3200	*VWA1*	64856	von Willebrand factor A domain containing 1 [Source:HGNC Symbol;Acc:HGNC:30910]		2
3201	*NPNT*	255743	nephronectin [Source:HGNC Symbol;Acc:HGNC:27405]		2
3202	*COL7A1*	1294	collagen type VII alpha 1 [Source:HGNC Symbol;Acc:HGNC:2214]		2
3203	*FMOD*	2331	fibromodulin [Source:HGNC Symbol;Acc:HGNC:3774]		2
3204	*LAMA5*	3911	laminin subunit alpha 5 [Source:HGNC Symbol;Acc:HGNC:6485]		2
3205	*MMP10*	4319	matrix metallopeptidase 10 [Source:HGNC Symbol;Acc:HGNC:7156]		2
3206	*PAPLN*	89932	papilin, proteoglycan like sulfated glycoprotein [Source:HGNC Symbol;Acc:HGNC:19262]		2
3207	*ADAMTS13*	11093	ADAM metallopeptidase with thrombospondin type 1 motif 13 [Source:HGNC Symbol;Acc:HGNC:1366]		2
3208	*AMTN*	401138	amelotin [Source:HGNC Symbol;Acc:HGNC:33188]		2

3209	*CPZ*	8532	carboxypeptidase Z [Source:HGNC Symbol;Acc:HGNC:2333]		2
3210	*FBN3*	84467	fibrillin 3 [Source:HGNC Symbol;Acc:HGNC:18794]		2
3211	*GPC4*	2239	glypican 4 [Source:HGNC Symbol;Acc:HGNC:4452]		2
3212	*IMPG2*	50939	interphotoreceptor matrix proteoglycan 2 [Source:HGNC Symbol;Acc:HGNC:18362]		2
3213	*LAMA2*	3908	laminin subunit alpha 2 [Source:HGNC Symbol;Acc:HGNC:6482]		2
3214	*MEPE*	56955	matrix extracellular phosphoglycoprotein [Source:HGNC Symbol;Acc:HGNC:13361]		2
3215	*MMP3*	4314	matrix metallopeptidase 3 [Source:HGNC Symbol;Acc:HGNC:7173]		2
3216	*RELN*	5649	reelin [Source:HGNC Symbol;Acc:HGNC:9957]		2
3217	*TGFBI*	7045	transforming growth factor beta induced [Source:HGNC Symbol;Acc:HGNC:11771]		2
3218	*UCMA*	221044	upper zone of growth plate and cartilage matrix associated [Source:HGNC Symbol;Acc:HGNC:25205]		2
3219	*VIT*	5212	vitrin [Source:HGNC Symbol;Acc:HGNC:12697]		2
3220	*COL9A2*	1298	collagen type IX alpha 2 [Source:HGNC Symbol;Acc:HGNC:2218]		2
3221	*ZP3*	7784	zona pellucida glycoprotein 3 (sperm receptor) [Source:HGNC Symbol;Acc:HGNC:13189]		2
3222	*BMP4*	652	bone morphogenetic protein 4 [Source:HGNC Symbol;Acc:HGNC:1071]		2
3223	*P3H1*	64175	prolyl 3-hydroxylase 1 [Source:HGNC Symbol;Acc:HGNC:19316]		2
3224	*LUM*	4060	lumican [Source:HGNC Symbol;Acc:HGNC:6724]		2
3225	*APLP1*	333	amyloid beta precursor like protein 1 [Source:HGNC Symbol;Acc:HGNC:597]		2
3226	*COL11A2*	1302	collagen type XI alpha 2 [Source:HGNC Symbol;Acc:HGNC:2187]		2
3227	*CRISP3*	10321	cysteine rich secretory protein 3 [Source:HGNC Symbol;Acc:HGNC:16904]		2
3228	*DCN*	1634	decorin [Source:HGNC Symbol;Acc:HGNC:2705]		2
3229	*ENTPD2*	954	ectonucleoside triphosphate diphosphohydrolase 2 [Source:HGNC Symbol;Acc:HGNC:3364]		2
3230	*GPC1*	2817	glypican 1 [Source:HGNC Symbol;Acc:HGNC:4449]		2
3231	*MMP12*	4321	matrix metallopeptidase 12 [Source:HGNC Symbol;Acc:HGNC:7158]		2
3232	*SFTPA2*	729238	surfactant protein A2 [Source:HGNC Symbol;Acc:HGNC:10799]		2
3233	*ADAMTSL4*	54507	ADAMTS like 4 [Source:HGNC Symbol;Acc:HGNC:19706]		2
3234	*ANOS1*	3730	anosmin 1 [Source:HGNC Symbol;Acc:HGNC:6211]		2
3235	*KAZALD1*	81621	Kazal type serine peptidase inhibitor domain 1 [Source:HGNC Symbol;Acc:HGNC:25460]		2
3236	*SMOC1*	64093	SPARC related modular calcium binding 1 [Source:HGNC Symbol;Acc:HGNC:20318]		2
3237	*SMOC2*	64094	SPARC related modular calcium binding 2 [Source:HGNC Symbol;Acc:HGNC:20323]		2
3238	*ACHE*	43	acetylcholinesterase (Cartwright blood group) [Source:HGNC Symbol;Acc:HGNC:108]		2
3239	*CHADL*	150356	chondroadherin like [Source:HGNC Symbol;Acc:HGNC:25165]		2
3240	*COCH*	1690	cochlin [Source:HGNC Symbol;Acc:HGNC:2180]		2
3241	*COL2A1*	1280	collagen type II alpha 1 chain [Source:HGNC Symbol;Acc:HGNC:2200]		2
3242	*COL4A6*	1288	collagen type IV alpha 6 chain [Source:HGNC Symbol;Acc:HGNC:2208]		2
3243	*FBLN7*	129804	fibulin 7 [Source:HGNC Symbol;Acc:HGNC:26740]		2
3244	*PI3*	5266	peptidase inhibitor 3 [Source:HGNC Symbol;Acc:HGNC:8947]		2
3245	*ADAMTSL2*	9719	ADAMTS like 2 [Source:HGNC Symbol;Acc:HGNC:14631]		2
3246	*WNT10B*	7480	Wnt family member 10B [Source:HGNC Symbol;Acc:HGNC:12775]		2
3247	*WNT4*	54361	Wnt family member 4 [Source:HGNC Symbol;Acc:HGNC:12783]		2
3248	*MYC*	4609	v-myc avian myelocytomatosis viral oncogene homolog [Source:HGNC Symbol;Acc:HGNC:7553]		2
3249	*ZNF217*	7764	zinc finger protein 217 [Source:HGNC Symbol;Acc:HGNC:13009]		2
3250	*GATA3*	2625	GATA binding protein 3 [Source:HGNC Symbol;Acc:HGNC:4172]		2
3251	*PTX3*	5806	pentraxin 3 [Source:HGNC Symbol;Acc:HGNC:9692]		2
3252	*COPS6*	10980	COP9 signalosome subunit 6 [Source:HGNC Symbol;Acc:HGNC:21749]	-1.5	-2
3253	*DCP2*	167227	decapping mRNA 2 [Source:HGNC Symbol;Acc:HGNC:24452]	-1.5	-2
3254	*ARHGEF6*	9459	Rac/Cdc42 guanine nucleotide exchange factor 6 [Source:HGNC Symbol;Acc:HGNC:685]	-1.5	-2
3255	*PDZD7*	79955	PDZ domain containing 7 [Source:HGNC Symbol;Acc:HGNC:26257]	-1.5	-2
3256	*ENTPD4*	9583	ectonucleoside triphosphate diphosphohydrolase 4 [Source:HGNC Symbol;Acc:HGNC:14573]	-1.5	-2
3257	*LRP5*	4041	LDL receptor related protein 5 [Source:HGNC Symbol;Acc:HGNC:6697]	-1.5	-2
3258	*PAFAH1B1*	5048	platelet activating factor acetylhydrolase 1b regulatory subunit 1 [Source:HGNC Symbol;Acc:HGNC:8574]	-1.5	-2
3259	*PDE4B*	5142	phosphodiesterase 4B [Source:HGNC Symbol;Acc:HGNC:8781]	-1.5	-2
3260	*PFDN2*	5202	prefoldin subunit 2 [Source:HGNC Symbol;Acc:HGNC:8867]	-1.5	-2
3261	*FAM91A1*	157769	family with sequence similarity 91 member A1 [Source:HGNC Symbol;Acc:HGNC:26306]	-1.5	-2
3262	*TARM1*	441864	T cell-interacting, activating receptor on myeloid cells 1 [Source:HGNC Symbol;Acc:HGNC:37250]	-1.5	-2
3263	*RPL5*	6125	ribosomal protein L5 [Source:HGNC Symbol;Acc:HGNC:10360]	-1.5	-2
3264	*ATP5G1*	516	ATP synthase, H+ transporting, mitochondrial Fo complex subunit C1 (subunit 9) [Source:HGNC Symbol;Acc:HGNC:841]	-1.5	-2
3265	*JMJD1C*	221037	jumonji domain containing 1C [Source:HGNC Symbol;Acc:HGNC:12313]	-1.5	-2
3266	*DDOST*	1650	dolichyl-diphosphooligosaccharide--protein glycosyltransferase non-catalytic subunit [Source:HGNC Symbol;Acc:HGNC:2728]	-1.5	-2
3267	*LDLRAD4*	753	low density lipoprotein receptor class A domain containing 4 [Source:HGNC Symbol;Acc:HGNC:1224]	-1.5	-2
3268	*INPP5B*	3633	inositol polyphosphate-5-phosphatase B [Source:HGNC Symbol;Acc:HGNC:6077]	-1.5	-2
3269	*TMIE*	259236	transmembrane inner ear [Source:HGNC Symbol;Acc:HGNC:30800]	-1.5	-2
3270	*CIZ1*	25792	CDKN1A interacting zinc finger protein 1 [Source:HGNC Symbol;Acc:HGNC:16744]	-1.5	-2
3271	*TMEM229B*	161145	transmembrane protein 229B [Source:HGNC Symbol;Acc:HGNC:20130]	-1.5	-2
3272	*EXOSC9*	5393	exosome component 9 [Source:HGNC Symbol;Acc:HGNC:9137]	-1.5	-2
3273	*AKR1A1*	10327	aldo-keto reductase family 1 member A1 [Source:HGNC Symbol;Acc:HGNC:380]	-1.5	-2
3274	*SCNN1G*	6340	sodium channel epithelial 1 gamma subunit [Source:HGNC Symbol;Acc:HGNC:10602]	-1.5	-2
3275	*KRTAP15-1*	254950	keratin associated protein 15-1 [Source:HGNC Symbol;Acc:HGNC:18927]	-1.5	-2
3276	*CUTA*	51596	cutA divalent cation tolerance homolog (E. coli) [Source:HGNC Symbol;Acc:HGNC:21101]	-1.5	-2
3277	*UTRN*	7402	utrophin [Source:HGNC Symbol;Acc:HGNC:12635]	-1.5	-2
3278	*NBEAL1*	65065	neurobeachin like 1 [Source:HGNC Symbol;Acc:HGNC:20681]	-1.5	-2
3279	*MRPS15*	64960	mitochondrial ribosomal protein S15 [Source:HGNC Symbol;Acc:HGNC:14504]	-1.5	-2
3280	*FAM228A*	653140	family with sequence similarity 228 member A [Source:HGNC Symbol;Acc:HGNC:34418]	-1.5	-2
3281	*ZNF583*	147949	zinc finger protein 583 [Source:HGNC Symbol;Acc:HGNC:26427]	-1.5	-2
3282	*ZNF12*	7559	zinc finger protein 12 [Source:HGNC Symbol;Acc:HGNC:12902]	-1.5	-2

3283	*ZFYVE21*	79038	zinc finger FYVE-type containing 21 [Source:HGNC Symbol;Acc:HGNC:20760]	-1.5	-2
3284	*CHMP7*	91782	charged multivesicular body protein 7 [Source:HGNC Symbol;Acc:HGNC:28439]	-1.5	-2
3285	*FAM195A*	84331	family with sequence similarity 195 member A	-1.5	-2
3286	*INPP4A*	3631	inositol polyphosphate-4-phosphatase type I A [Source:HGNC Symbol;Acc:HGNC:6074]	-1.5	-2
3287	*ZNF436*	80818	zinc finger protein 436 [Source:HGNC Symbol;Acc:HGNC:20814]	-1.5	-2
3288	*MAN1B1*	11253	mannosidase alpha class 1B member 1 [Source:HGNC Symbol;Acc:HGNC:6823]	-1.5	-2
3289	*FGA*	2243	fibrinogen alpha chain [Source:HGNC Symbol;Acc:HGNC:3661]	-1.5	-2
3290	*FOSL1*	8061	FOS like 1, AP-1 transcription factor subunit [Source:HGNC Symbol;Acc:HGNC:13718]	-1.5	-2
3291	*TOMM7*	54543	translocase of outer mitochondrial membrane 7 [Source:HGNC Symbol;Acc:HGNC:21648]	-1.5	-2
3292	*PCSK2*	5126	proprotein convertase subtilisin/kexin type 2 [Source:HGNC Symbol;Acc:HGNC:8744]	-1.5	-2
3293	*GPBP1*	65056	GC-rich promoter binding protein 1 [Source:HGNC Symbol;Acc:HGNC:29520]	-1.5	-2
3294	*ZC3H15*	55854	zinc finger CCCH-type containing 15 [Source:HGNC Symbol;Acc:HGNC:29528]	-1.5	-2
3295	*IFITM3*	10410	interferon induced transmembrane protein 3 [Source:HGNC Symbol;Acc:HGNC:5414]	-1.5	-2
3296	*NUFIP1*	26747	NUFIP1, FMR1 interacting protein 1 [Source:HGNC Symbol;Acc:HGNC:8057]	-1.5	-2
3297	*RPL21*	6144	ribosomal protein L21 [Source:HGNC Symbol;Acc:HGNC:10313]	-1.5	-2
3298	*APMAP*	57136	adipocyte plasma membrane associated protein [Source:HGNC Symbol;Acc:HGNC:13238]	-1.5	-2
3299	*CNIH4*	29097	cornichon family AMPA receptor auxiliary protein 4 [Source:HGNC Symbol;Acc:HGNC:25013]	-1.5	-2
3300	*C17orf89*	284184	chromosome 17 open reading frame 89 [Source:HGNC Symbol;Acc:HGNC:33551]	-1.5	-2
3301	*FOXP1*	27086	forkhead box P1 [Source:HGNC Symbol;Acc:HGNC:3823]	-1.5	-2
3302	*LSM12*	124801	LSM12 homolog [Source:HGNC Symbol;Acc:HGNC:26407]	-1.5	-2
3303	*IFT52*	51098	intraflagellar transport 52 [Source:HGNC Symbol;Acc:HGNC:15901]	-1.5	-2
3304	*CSRP1*	1465	cysteine and glycine rich protein 1 [Source:HGNC Symbol;Acc:HGNC:2469]	-1.5	-2
3305	*VIMP*	55829	VCP interacting membrane selenoprotein [Source:HGNC Symbol;Acc:HGNC:30396]	-1.5	-2
3306	*DERL1*	79139	derlin 1 [Source:HGNC Symbol;Acc:HGNC:28454]	-1.5	-2
3307	*FLOT2*	2319	flotillin 2 [Source:HGNC Symbol;Acc:HGNC:3758]	-1.5	-2
3308	*C14orf166*	51637	chromosome 14 open reading frame 166 [Source:HGNC Symbol;Acc:HGNC:23169]	-1.5	-2
3309	*ORC4*	5000	origin recognition complex subunit 4 [Source:HGNC Symbol;Acc:HGNC:8490]	-1.5	-2
3310	*FAM21C*	253725	family with sequence similarity 21 member C [Source:HGNC Symbol;Acc:HGNC:23414]	-1.5	-2
3311	*TSPYL4*	23270	TSPY-like 4 [Source:HGNC Symbol;Acc:HGNC:21559]	-1.5	-2
3312	*ACAP3*	116983	ArfGAP with coiled-coil, ankyrin repeat and PH domains 3 [Source:HGNC Symbol;Acc:HGNC:16754]	-1.5	-2
3313	*COX7C*	1350	cytochrome c oxidase subunit 7C [Source:HGNC Symbol;Acc:HGNC:2292]	-1.5	-2
3314	*DFNB31*	25861	Deafness, Autosomal Recessive 31	-1.5	-2
3315	*KIAA0430*	9665	KIAA0430 [Source:HGNC Symbol;Acc:HGNC:29562]	-1.5	-2
3316	*NOSTRIN*	115677	nitric oxide synthase trafficking [Source:HGNC Symbol;Acc:HGNC:20203]	-1.5	-2
3317	*SNX5*	27131	sorting nexin 5 [Source:HGNC Symbol;Acc:HGNC:14969]	-1.5	-2
3318	*HSPA8*	3312	heat shock protein family A (Hsp70) member 8 [Source:HGNC Symbol;Acc:HGNC:5241]	-1.5	-2
3319	*LUC7L*	55692	LUC7 like [Source:HGNC Symbol;Acc:HGNC:6723]	-1.5	-2
3320	*EEF1A1*	1915	eukaryotic translation elongation factor 1 alpha 1 [Source:HGNC Symbol;Acc:HGNC:3189]	-1.5	-2
3321	*SNRPD1*	6632	small nuclear ribonucleoprotein D1 polypeptide [Source:HGNC Symbol;Acc:HGNC:11158]	-1.5	-2
3322	*MGMT*	4255	O-6-methylguanine-DNA methyltransferase [Source:HGNC Symbol;Acc:HGNC:7059]	-1.5	-2
3323	*SLIRP*	81892	SRA stem-loop interacting RNA binding protein [Source:HGNC Symbol;Acc:HGNC:20495]	-1.5	-2
3324	*ARFIP1*	27236	ADP ribosylation factor interacting protein 1 [Source:HGNC Symbol;Acc:HGNC:21496]	-1.5	-2
3325	*PTRH2*	51651	peptidyl-tRNA hydrolase 2 [Source:HGNC Symbol;Acc:HGNC:24265]	-1.5	-2
3326	*TXNRD1*	7296	thioredoxin reductase 1 [Source:HGNC Symbol;Acc:HGNC:12437]	-1.5	-2
3327	*EFR3A*	23167	EFR3 homolog A [Source:HGNC Symbol;Acc:HGNC:28970]	-1.5	-2
3328	*UHRF2*	115426	ubiquitin like with PHD and ring finger domains 2 [Source:HGNC Symbol;Acc:HGNC:12557]	-1.5	-2
3329	*RHEB*	6009	Ras homolog enriched in brain [Source:HGNC Symbol;Acc:HGNC:10011]	-1.5	-2
3330	*ZNF596*	169270	zinc finger protein 596 [Source:HGNC Symbol;Acc:HGNC:27268]	-1.5	-2
3331	*PRKAG1*	5571	protein kinase AMP-activated non-catalytic subunit gamma 1 [Source:HGNC Symbol;Acc:HGNC:9385]	-1.5	-2
3332	*SYF2*	25949	SYF2 pre-mRNA splicing factor [Source:HGNC Symbol;Acc:HGNC:19824]	-1.5	-2
3333	*PCSK1*	5122	proprotein convertase subtilisin/kexin type 1 [Source:HGNC Symbol;Acc:HGNC:8743]	-1.5	-2
3334	*PIGC*	5279	phosphatidylinositol glycan anchor biosynthesis class C [Source:HGNC Symbol;Acc:HGNC:8960]	-1.5	-2
3335	*PPA1*	5464	pyrophosphatase (inorganic) 1 [Source:HGNC Symbol;Acc:HGNC:9226]	-1.5	-2
3336	*IQCA1*	79781	IQ motif containing with AAA domain 1 [Source:HGNC Symbol;Acc:HGNC:26195]	-1.5	-2
3337	*RPL13A*	23521	ribosomal protein L13a [Source:HGNC Symbol;Acc:HGNC:10304]	-1.5	-2
3338	*TIA1*	7072	TIA1 cytotoxic granule-associated RNA binding protein [Source:HGNC Symbol;Acc:HGNC:11802]	-1.5	-2
3339	*CYTL1*	54360	cytokine like 1 [Source:HGNC Symbol;Acc:HGNC:24435]	-1.5	-2
3340	*CCDC6*	8030	coiled-coil domain containing 6 [Source:HGNC Symbol;Acc:HGNC:18782]	-1.5	-2
3341	*CLIC5*	53405	chloride intracellular channel 5 [Source:HGNC Symbol;Acc:HGNC:13517]	-1.5	-2
3342	*SFT2D1*	113402	SFT2 domain containing 1 [Source:HGNC Symbol;Acc:HGNC:21102]	-1.5	-2
3343	*CAND1*	55832	cullin associated and neddylation dissociated 1 [Source:HGNC Symbol;Acc:HGNC:30688]	-1.5	-2
3344	*RYR2*	6262	ryanodine receptor 2 [Source:HGNC Symbol;Acc:HGNC:10484]	-1.5	-2
3345	*NDUFB5*	4711	NADH:ubiquinone oxidoreductase subunit B5 [Source:HGNC Symbol;Acc:HGNC:7700]	-1.5	-2
3346	*DRAM2*	128338	DNA damage regulated autophagy modulator 2 [Source:HGNC Symbol;Acc:HGNC:28769]	-1.5	-2
3347	*NDUFA8*	4702	NADH:ubiquinone oxidoreductase subunit A8 [Source:HGNC Symbol;Acc:HGNC:7692]	-1.5	-2
3348	*ZKSCAN1*	7586	zinc finger with KRAB and SCAN domains 1 [Source:HGNC Symbol;Acc:HGNC:13101]	-1.5	-2
3349	*PPP1CC*	5501	protein phosphatase 1 catalytic subunit gamma [Source:HGNC Symbol;Acc:HGNC:9283]	-1.5	-2
3350	*WDR88*	126248	WD repeat domain 88 [Source:HGNC Symbol;Acc:HGNC:26999]	-1.5	-2
3351	*TMF1*	7110	TATA element modulatory factor 1 [Source:HGNC Symbol;Acc:HGNC:11870]	-1.5	-2
3352	*TICAM1*	148022	toll like receptor adaptor molecule 1 [Source:HGNC Symbol;Acc:HGNC:18348]	-1.5	-2
3353	*MLH1*	4292	mutL homolog 1 [Source:HGNC Symbol;Acc:HGNC:7127]	-1.5	-2
3354	*ZMYM2*	7750	zinc finger MYM-type containing 2 [Source:HGNC Symbol;Acc:HGNC:12989]	-1.5	-2
3355	*UTP14A*	10813	UTP14A small subunit processome component [Source:HGNC Symbol;Acc:HGNC:10665]	-1.5	-2
3356	*BBS7*	55212	Bardet-Biedl syndrome 7 [Source:HGNC Symbol;Acc:HGNC:18758]	-1.5	-2
3357	*GRIP1*	23426	glutamate receptor interacting protein 1 [Source:HGNC Symbol;Acc:HGNC:18708]	-1.5	-2
3358	*CENPB*	1059	centromere protein B [Source:HGNC Symbol;Acc:HGNC:1852]	-1.5	-2
3359	*AHCYL1*	10768	adenosylhomocysteinase like 1 [Source:HGNC Symbol;Acc:HGNC:344]	-1.5	-2

3360	*ANAPC11*	51529	anaphase promoting complex subunit 11 [Source:HGNC Symbol;Acc:HGNC:14452]	-1.5	-2
3361	*ANAPC16*	119504	anaphase promoting complex subunit 16 [Source:HGNC Symbol;Acc:HGNC:26976]	-1.5	-2
3362	*POLB*	5423	polymerase (DNA) beta [Source:HGNC Symbol;Acc:HGNC:9174]	-1.5	-2
3363	*ITGA11*	22801	integrin subunit alpha 11 [Source:HGNC Symbol;Acc:HGNC:6136]	-1.5	-2
3364	*CNOT7*	29883	CCR4-NOT transcription complex subunit 7 [Source:HGNC Symbol;Acc:HGNC:14101]	-1.5	-2
3365	*CD164*	8763	CD164 molecule [Source:HGNC Symbol;Acc:HGNC:1632]	-1.5	-2
3366	*UQCC2*	84300	ubiquinol-cytochrome c reductase complex assembly factor 2 [Source:HGNC Symbol;Acc:HGNC:21237]	-1.5	-2
3367	*UBA52*	7311	ubiquitin A-52 residue ribosomal protein fusion product 1 [Source:HGNC Symbol;Acc:HGNC:12458]	-1.5	-2
3368	*RPS23*	6228	ribosomal protein S23 [Source:HGNC Symbol;Acc:HGNC:10410]	-1.5	-2
3369	*SEMA3D*	223117	semaphorin 3D [Source:HGNC Symbol;Acc:HGNC:10726]	-1.5	-2
3370	*TEDDM1*	127670	transmembrane epididymal protein 1 [Source:HGNC Symbol;Acc:HGNC:30233]	-1.5	-2
3371	*C1GALT1*	56913	core 1 synthase, glycoprotein-N-acetylgalactosamine 3-beta-galactosyltransferase 1 [Source:HGNC Symbol;Acc:HGNC:24337]	-1.5	-2
3372	*IER3*	8870	immediate early response 3 [Source:HGNC Symbol;Acc:HGNC:5392]	-1.5	-2
3373	*HAX1*	10456	HCLS1 associated protein X-1 [Source:HGNC Symbol;Acc:HGNC:16915]	-1.5	-2
3374	*ZNF267*	10308	zinc finger protein 267 [Source:HGNC Symbol;Acc:HGNC:13060]	-1.5	-2
3375	*TBCK*	93627	TBC1 domain containing kinase [Source:HGNC Symbol;Acc:HGNC:28261]	-1.5	-2
3376	*RPS14*	6208	ribosomal protein S14 [Source:HGNC Symbol;Acc:HGNC:10387]	-1.5	-2
3377	*PSMG1*	8624	proteasome assembly chaperone 1 [Source:HGNC Symbol;Acc:HGNC:3043]	-1.5	-2
3378	*C1QBP*	708	complement component 1, q subcomponent binding protein [Source:HGNC Symbol;Acc:HGNC:1243]	-1.5	-2
3379	*ARID4B*	51742	AT-rich interaction domain 4B [Source:HGNC Symbol;Acc:HGNC:15550]	-1.5	-2
3380	*GLT8D1*	55830	glycosyltransferase 8 domain containing 1 [Source:HGNC Symbol;Acc:HGNC:24870]	-1.5	-2
3381	*ERGIC2*	51290	ERGIC and golgi 2 [Source:HGNC Symbol;Acc:HGNC:30208]	-1.5	-2
3382	*SEC24A*	10802	SEC24 homolog A, COPII coat complex component [Source:HGNC Symbol;Acc:HGNC:10703]	-1.5	-2
3383	*NR3C1*	2908	nuclear receptor subfamily 3 group C member 1 [Source:HGNC Symbol;Acc:HGNC:7978]	-1.5	-2
3384	*DYNC1I2*	1781	dynein cytoplasmic 1 intermediate chain 2 [Source:HGNC Symbol;Acc:HGNC:2964]	-1.5	-2
3385	*RAPGEF6*	51735	Rap guanine nucleotide exchange factor 6 [Source:HGNC Symbol;Acc:HGNC:20655]	-1.5	-2
3386	*TTC17*	55761	tetratricopeptide repeat domain 17 [Source:HGNC Symbol;Acc:HGNC:25596]	-1.5	-2
3387	*SMARCE1*	6605	SWI/SNF related, matrix associated, actin dependent regulator of chromatin, subfamily e, member 1 [Source:HGNC Symbol;Acc:HGNC:11109]	-1.5	-2
3388	*EI24*	9538	EI24, autophagy associated transmembrane protein [Source:HGNC Symbol;Acc:HGNC:13276]	-1.5	-2
3389	*SEC11A*	23478	SEC11 homolog A, signal peptidase complex subunit [Source:HGNC Symbol;Acc:HGNC:17718]	-1.5	-2
3390	*RNF7*	9616	ring finger protein 7 [Source:HGNC Symbol;Acc:HGNC:10070]	-1.5	-2
3391	*C4orf27*	54969	chromosome 4 open reading frame 27	-1.5	-2
3392	*TTC37*	9652	tetratricopeptide repeat domain 37 [Source:HGNC Symbol;Acc:HGNC:23639]	-1.5	-2
3393	*EEF1B2*	1933	eukaryotic translation elongation factor 1 beta 2 [Source:HGNC Symbol;Acc:HGNC:3208]	-1.5	-2
3394	*MZT2B*	80097	mitotic spindle organizing protein 2B [Source:HGNC Symbol;Acc:HGNC:25886]	-1.5	-2
3395	*TRIB3*	57761	tribbles pseudokinase 3 [Source:HGNC Symbol;Acc:HGNC:16228]	-1.5	-2
3396	*HS6ST1*	9394	heparan sulfate 6-O-sulfotransferase 1 [Source:HGNC Symbol;Acc:HGNC:5201]	-1.5	-2
3397	*GPR137B*	7107	G protein-coupled receptor 137B [Source:HGNC Symbol;Acc:HGNC:11862]	-1.5	-2
3398	*FOXJ3*	22887	forkhead box J3 [Source:HGNC Symbol;Acc:HGNC:29178]	-1.5	-2
3399	*VKORC1L1*	154807	vitamin K epoxide reductase complex subunit 1 like 1 [Source:HGNC Symbol;Acc:HGNC:21492]	-1.5	-2
3400	*TMEM42*	131616	transmembrane protein 42 [Source:HGNC Symbol;Acc:HGNC:28444]	-1.5	-2
3401	*DSG2*	1829	desmoglein 2 [Source:HGNC Symbol;Acc:HGNC:3049]	-1.5	-2
3402	*ZFHX4*	79776	zinc finger homeobox 4 [Source:HGNC Symbol;Acc:HGNC:30939]	-1.5	-2
3403	*AHCY*	191	adenosylhomocysteinase [Source:HGNC Symbol;Acc:HGNC:343]	-1.5	-2
3404	*ADA*	100	adenosine deaminase [Source:HGNC Symbol;Acc:HGNC:186]	-1.5	-2
3405	*RBM4*	5936	RNA binding motif protein 4 [Source:HGNC Symbol;Acc:HGNC:9901]	-1.5	-2
3406	*ZSCAN30*	100101467	zinc finger and SCAN domain containing 30 [Source:HGNC Symbol;Acc:HGNC:33517]	-1.5	-2
3407	*GPR162*	27239	G protein-coupled receptor 162 [Source:HGNC Symbol;Acc:HGNC:16693]	-1.5	-2
3408	*SLAMF7*	57823	SLAM family member 7 [Source:HGNC Symbol;Acc:HGNC:21394]	-1.5	-2
3409	*SMARCA2*	6595	SWI/SNF related, matrix associated, actin dependent regulator of chromatin, subfamily a, member 2 [Source:HGNC Symbol;Acc:HGNC:11098]	-1.5	-2
3410	*SCP2*	6342	sterol carrier protein 2 [Source:HGNC Symbol;Acc:HGNC:10606]	-1.5	-2
3411	*DAD1*	1603	defender against cell death 1 [Source:HGNC Symbol;Acc:HGNC:2664]	-1.5	-2
3412	*FKBP1A*	2280	FK506 binding protein 1A [Source:HGNC Symbol;Acc:HGNC:3711]	-1.5	-2
3413	*SDCBP2*	27111	syndecan binding protein 2 [Source:HGNC Symbol;Acc:HGNC:15756]	-1.5	-2
3414	*PRKD3*	23683	protein kinase D3 [Source:HGNC Symbol;Acc:HGNC:9408]	-1.5	-2
3415	*USP16*	10600	ubiquitin specific peptidase 16 [Source:HGNC Symbol;Acc:HGNC:12614]	-1.5	-2
3416	*EIF3K*	27335	eukaryotic translation initiation factor 3 subunit K [Source:HGNC Symbol;Acc:HGNC:24656]	-1.5	-2
3417	*ATXN1*	6310	ataxin 1 [Source:HGNC Symbol;Acc:HGNC:10548]	-1.5	-2
3418	*DYNC1LI2*	1783	dynein cytoplasmic 1 light intermediate chain 2 [Source:HGNC Symbol;Acc:HGNC:2966]	-1.5	-2
3419	*FIBCD1*	84929	fibrinogen C domain containing 1 [Source:HGNC Symbol;Acc:HGNC:25922]	-1.5	-2
3420	*CTNNBIP1*	56998	catenin beta interacting protein 1 [Source:HGNC Symbol;Acc:HGNC:16913]	-1.5	-2
3421	*NECAB2*	54550	N-terminal EF-hand calcium binding protein 2 [Source:HGNC Symbol;Acc:HGNC:23746]	-1.5	-2
3422	*ZBTB11*	27107	zinc finger and BTB domain containing 11 [Source:HGNC Symbol;Acc:HGNC:16740]	-1.5	-2
3423	*ATF6*	22926	activating transcription factor 6 [Source:HGNC Symbol;Acc:HGNC:791]	-1.5	-2
3424	*RPL27*	6155	ribosomal protein L27 [Source:HGNC Symbol;Acc:HGNC:10328]	-1.5	-2
3425	*PPP2CB*	5516	protein phosphatase 2 catalytic subunit beta [Source:HGNC Symbol;Acc:HGNC:9300]	-1.5	-2
3426	*ADRB1*	153	adrenoceptor beta 1 [Source:HGNC Symbol;Acc:HGNC:285]	-1.5	-2
3427	*SCAF1*	58506	SR-related CTD associated factor 1 [Source:HGNC Symbol;Acc:HGNC:30403]	-1.5	-2
3428	*CYSLTR2*	57105	cysteinyl leukotriene receptor 2 [Source:HGNC Symbol;Acc:HGNC:18274]	-1.5	-2
3429	*RPSA*	3921	ribosomal protein SA [Source:HGNC Symbol;Acc:HGNC:6502]	-1.5	-2
3430	*PPP6R3*	55291	protein phosphatase 6 regulatory subunit 3 [Source:HGNC Symbol;Acc:HGNC:1173]	-1.5	-2
3431	*CHRNA3*	1136	cholinergic receptor nicotinic alpha 3 subunit [Source:HGNC Symbol;Acc:HGNC:1957]	-1.5	-2
3432	*MRPS18B*	28973	mitochondrial ribosomal protein S18B [Source:HGNC Symbol;Acc:HGNC:14516]	-1.5	-2

3433	*ATM*	472	ATM serine/threonine kinase [Source:HGNC Symbol;Acc:HGNC:795]	-1.5	-2
3434	*NACA*	4666	nascent polypeptide-associated complex alpha subunit [Source:HGNC Symbol;Acc:HGNC:7629]	-1.5	-2
3435	*GCLC*	2729	glutamate-cysteine ligase catalytic subunit [Source:HGNC Symbol;Acc:HGNC:4311]	-1.5	-2
3436	*RPS2*	6187	ribosomal protein S2 [Source:HGNC Symbol;Acc:HGNC:10404]	-1.5	-2
3437	*C1QC*	714	complement component 1, q subcomponent, C chain [Source:HGNC Symbol;Acc:HGNC:1245]	-1.5	-2
3438	*SERINC5*	256987	serine incorporator 5 [Source:HGNC Symbol;Acc:HGNC:18825]	-1.5	-2
3439	*ZBTB21*	49854	zinc finger and BTB domain containing 21 [Source:HGNC Symbol;Acc:HGNC:13083]	-1.5	-2
3440	*RPL35*	11224	ribosomal protein L35 [Source:HGNC Symbol;Acc:HGNC:10344]	-1.5	-2
3441	*FBXO25*	26260	F-box protein 25 [Source:HGNC Symbol;Acc:HGNC:13596]	-1.5	-2
3442	*CANX*	821	calnexin [Source:HGNC Symbol;Acc:HGNC:1473]	-1.5	-2
3443	*CHMP4B*	128866	charged multivesicular body protein 4B [Source:HGNC Symbol;Acc:HGNC:16171]	-1.5	-2
3444	*NFIX*	4784	nuclear factor I X [Source:HGNC Symbol;Acc:HGNC:7788]	-1.5	-2
3445	*MESDC2*	23184	mesoderm development candidate 2 [Source:HGNC Symbol;Acc:HGNC:13520]	-1.5	-2
3446	*RGS6*	9628	regulator of G-protein signaling 6 [Source:HGNC Symbol;Acc:HGNC:10002]	-1.5	-2
3447	*CAPRIN1*	4076	cell cycle associated protein 1 [Source:HGNC Symbol;Acc:HGNC:6743]	-1.5	-2
3448	*RPS9*	6203	ribosomal protein S9 [Source:HGNC Symbol;Acc:HGNC:10442]	-1.5	-2
3449	*TEX9*	374618	testis expressed 9 [Source:HGNC Symbol;Acc:HGNC:29585]	-1.5	-2
3450	*IL24*	11009	interleukin 24 [Source:HGNC Symbol;Acc:HGNC:11346]	-1.5	-2
3451	*ZNF546*	339327	zinc finger protein 546 [Source:HGNC Symbol;Acc:HGNC:28671]	-1.5	-2
3452	*FAM127A*	8933	family with sequence similarity 127 member A [Source:HGNC Symbol;Acc:HGNC:2569]	-1.5	-2
3453	*HNRNPM*	4670	heterogeneous nuclear ribonucleoprotein M [Source:HGNC Symbol;Acc:HGNC:5046]	-1.5	-2
3454	*SUMF1*	285362	sulfatase modifying factor 1 [Source:HGNC Symbol;Acc:HGNC:20376]	-1.5	-2
3455	*SMN2*	6607	survival of motor neuron 2, centromeric [Source:HGNC Symbol;Acc:HGNC:11118]	-1.5	-2
3456	*CETN2*	1069	centrin 2 [Source:HGNC Symbol;Acc:HGNC:1867]	-1.5	-2
3457	*C1orf122*	127687	chromosome 1 open reading frame 122 [Source:HGNC Symbol;Acc:HGNC:24789]	-1.5	-2
3458	*PLEKHO2*	80301	pleckstrin homology domain containing O2 [Source:HGNC Symbol;Acc:HGNC:30026]	-1.5	-2
3459	*NIPSNAP3A*	25934	nipsnap homolog 3A [Source:HGNC Symbol;Acc:HGNC:23619]	-1.5	-2
3460	*PDCD4*	27250	programmed cell death 4 (neoplastic transformation inhibitor) [Source:HGNC Symbol;Acc:HGNC:8763]	-1.5	-2
3461	*RPL4*	6124	ribosomal protein L4 [Source:HGNC Symbol;Acc:HGNC:10353]	-1.5	-2
3462	*RPP14*	11102	ribonuclease P/MRP subunit p14 [Source:HGNC Symbol;Acc:HGNC:30327]	-1.5	-2
3463	*TXNDC17*	84817	thioredoxin domain containing 17 [Source:HGNC Symbol;Acc:HGNC:28218]	-1.5	-2
3464	*RASGRP1*	10125	RAS guanyl releasing protein 1 [Source:HGNC Symbol;Acc:HGNC:9878]	-1.5	-2
3465	*LDHB*	3945	lactate dehydrogenase B [Source:HGNC Symbol;Acc:HGNC:6541]	-1.5	-2
3466	*UNC119*	9094	unc-119 lipid binding chaperone [Source:HGNC Symbol;Acc:HGNC:12565]	-1.5	-2
3467	*EIF2AK2*	5610	eukaryotic translation initiation factor 2 alpha kinase 2 [Source:HGNC Symbol;Acc:HGNC:9437]	-1.5	-2
3468	*ATXN10*	25814	ataxin 10 [Source:HGNC Symbol;Acc:HGNC:10549]	-1.5	-2
3469	*TMEM205*	374882	transmembrane protein 205 [Source:HGNC Symbol;Acc:HGNC:29631]	-1.5	-2
3470	*EPS15*	2060	epidermal growth factor receptor pathway substrate 15 [Source:HGNC Symbol;Acc:HGNC:3419]	-1.5	-2
3471	*YTHDF2*	51441	YTH N6-methyladenosine RNA binding protein 2 [Source:HGNC Symbol;Acc:HGNC:31675]	-1.6	-2
3472	*SRM*	6723	spermidine synthase [Source:HGNC Symbol;Acc:HGNC:11296]	-1.6	-2
3473	*DPY30*	84661	dpy-30, histone methyltransferase complex regulatory subunit [Source:HGNC Symbol;Acc:HGNC:24590]	-1.6	-2
3474	*C1D*	10438	C1D nuclear receptor corepressor [Source:HGNC Symbol;Acc:HGNC:29911]	-1.6	-2
3475	*SLC39A9*	55334	solute carrier family 39 member 9 [Source:HGNC Symbol;Acc:HGNC:20182]	-1.6	-2
3476	*TP53INP1*	94241	tumor protein p53 inducible nuclear protein 1 [Source:HGNC Symbol;Acc:HGNC:18022]	-1.6	-2
3477	*STX17*	55014	syntaxin 17 [Source:HGNC Symbol;Acc:HGNC:11432]	-1.6	-2
3478	*EIF4E3*	317649	eukaryotic translation initiation factor 4E family member 3 [Source:HGNC Symbol;Acc:HGNC:31837]	-1.6	-2
3479	*R3HDM1*	23518	R3H domain containing 1 [Source:HGNC Symbol;Acc:HGNC:9757]	-1.6	-2
3480	*RPL7*	6129	ribosomal protein L7 [Source:HGNC Symbol;Acc:HGNC:10363]	-1.6	-2
3481	*RPL23A*	6147	ribosomal protein L23a [Source:HGNC Symbol;Acc:HGNC:10317]	-1.6	-2
3482	*PSMA3*	5684	proteasome subunit alpha 3 [Source:HGNC Symbol;Acc:HGNC:9532]	-1.6	-2
3483	*GMPPA*	29926	GDP-mannose pyrophosphorylase A [Source:HGNC Symbol;Acc:HGNC:22923]	-1.6	-2
3484	*IL11RA*	3590	interleukin 11 receptor subunit alpha [Source:HGNC Symbol;Acc:HGNC:5967]	-1.6	-2
3485	*PINK1*	65018	PTEN induced putative kinase 1 [Source:HGNC Symbol;Acc:HGNC:14581]	-1.6	-2
3486	*RPL10A*	4736	ribosomal protein L10a [Source:HGNC Symbol;Acc:HGNC:10299]	-1.6	-2
3487	*THEM4*	117145	thioesterase superfamily member 4 [Source:HGNC Symbol;Acc:HGNC:17947]	-1.6	-2
3488	*ITGA7*	3679	integrin subunit alpha 7 [Source:HGNC Symbol;Acc:HGNC:6143]	-1.6	-2
3489	*METTL7B*	196410	methyltransferase like 7B [Source:HGNC Symbol;Acc:HGNC:28276]	-1.6	-2
3490	*LSM14A*	26065	LSM14A mRNA processing body assembly factor [Source:HGNC Symbol;Acc:HGNC:24489]	-1.6	-2
3491	*PDCD7*	10081	programmed cell death 7 [Source:HGNC Symbol;Acc:HGNC:8767]	-1.6	-2
3492	*TMX3*	54495	thioredoxin related transmembrane protein 3 [Source:HGNC Symbol;Acc:HGNC:24718]	-1.6	-2
3493	*DDX5*	1655	DEAD-box helicase 5 [Source:HGNC Symbol;Acc:HGNC:2746]	-1.6	-2
3494	*TRIM31*	11074	tripartite motif containing 31 [Source:HGNC Symbol;Acc:HGNC:16289]	-1.6	-2
3495	*CSDE1*	7812	cold shock domain containing E1 [Source:HGNC Symbol;Acc:HGNC:29905]	-1.6	-2
3496	*RQCD1*	9125	CCR4-NOT Transcription Complex Subunit 9	-1.6	-2
3497	*KCTD10*	83892	potassium channel tetramerization domain containing 10 [Source:HGNC Symbol;Acc:HGNC:23236]	-1.6	-2
3498	*FAM19A5*	25817	family with sequence similarity 19 member A5, C-C motif chemokine like [Source:HGNC Symbol;Acc:HGNC:21592]	-1.6	-2
3499	*TTC19*	54902	tetratricopeptide repeat domain 19 [Source:HGNC Symbol;Acc:HGNC:26006]	-1.6	-2
3500	*FH*	2271	fumarate hydratase [Source:HGNC Symbol;Acc:HGNC:3700]	-1.6	-2
3501	*IMPAD1*	54928	inositol monophosphatase domain containing 1 [Source:HGNC Symbol;Acc:HGNC:26019]	-1.6	-2
3502	*STAC*	6769	SH3 and cysteine rich domain [Source:HGNC Symbol;Acc:HGNC:11353]	-1.6	-2
3503	*SYNGR2*	9144	synaptogyrin 2 [Source:HGNC Symbol;Acc:HGNC:11499]	-1.6	-2
3504	*BAZ2B*	29994	bromodomain adjacent to zinc finger domain 2B [Source:HGNC Symbol;Acc:HGNC:963]	-1.6	-2
3505	*KMO*	8564	kynurenine 3-monooxygenase [Source:HGNC Symbol;Acc:HGNC:6381]	-1.6	-2

3506	*IREB2*	3658	iron responsive element binding protein 2 [Source:HGNC Symbol;Acc:HGNC:6115]	-1.6	-2
3507	*SRSF7*	6432	serine and arginine rich splicing factor 7 [Source:HGNC Symbol;Acc:HGNC:10789]	-1.6	-2
3508	*TMEM167A*	153339	transmembrane protein 167A [Source:HGNC Symbol;Acc:HGNC:28330]	-1.6	-2
3509	*DHRS4*	10901	dehydrogenase/reductase 4 [Source:HGNC Symbol;Acc:HGNC:16985]	-1.6	-2
3510	*SARM1*	23098	sterile alpha and TIR motif containing 1 [Source:HGNC Symbol;Acc:HGNC:17074]	-1.6	-2
3511	*TRIM11*	81559	tripartite motif containing 11 [Source:HGNC Symbol;Acc:HGNC:16281]	-1.6	-2
3512	*PLP1*	5354	proteolipid protein 1 [Source:HGNC Symbol;Acc:HGNC:9086]	-1.6	-2
3513	*PNPLA8*	50640	patatin like phospholipase domain containing 8 [Source:HGNC Symbol;Acc:HGNC:28900]	-1.6	-2
3514	*ARHGEF12*	23365	Rho guanine nucleotide exchange factor 12 [Source:HGNC Symbol;Acc:HGNC:14193]	-1.6	-2
3515	*ABCB7*	22	ATP binding cassette subfamily B member 7 [Source:HGNC Symbol;Acc:HGNC:48]	-1.6	-2
3516	*SLC1A4*	6509	solute carrier family 1 member 4 [Source:HGNC Symbol;Acc:HGNC:10942]	-1.6	-2
3517	*ABCA5*	23461	ATP binding cassette subfamily A member 5 [Source:HGNC Symbol;Acc:HGNC:35]	-1.6	-2
3518	*GNB2L1*	10399	Guanine Nucleotide Binding Protein (G Protein), Beta Polypeptide 2-Like 1	-1.6	-2
3519	*TAF7*	6879	TATA-box binding protein associated factor 7 [Source:HGNC Symbol;Acc:HGNC:11541]	-1.6	-2
3520	*NUBPL*	80224	nucleotide binding protein like [Source:HGNC Symbol;Acc:HGNC:20278]	-1.6	-2
3521	*NCL*	4691	nucleolin [Source:HGNC Symbol;Acc:HGNC:7667]	-1.6	-2
3522	*KIAA0141*	9812	KIAA0141 [Source:HGNC Symbol;Acc:HGNC:28969]	-1.6	-2
3523	*PUM1*	9698	pumilio RNA binding family member 1 [Source:HGNC Symbol;Acc:HGNC:14957]	-1.6	-2
3524	*IRAK1*	3654	interleukin 1 receptor associated kinase 1 [Source:HGNC Symbol;Acc:HGNC:6112]	-1.6	-2
3525	*RPA2*	6118	replication protein A2 [Source:HGNC Symbol;Acc:HGNC:10290]	-1.6	-2
3526	*RPL18A*	6142	ribosomal protein L18a [Source:HGNC Symbol;Acc:HGNC:10311]	-1.6	-2
3527	*ZNF24*	7572	zinc finger protein 24 [Source:HGNC Symbol;Acc:HGNC:13032]	-1.6	-2
3528	*SLC25A28*	81894	solute carrier family 25 member 28 [Source:HGNC Symbol;Acc:HGNC:23472]	-1.6	-2
3529	*RCC2*	55920	regulator of chromosome condensation 2 [Source:HGNC Symbol;Acc:HGNC:30297]	-1.6	-2
3530	*ZNF550*	162972	zinc finger protein 550 [Source:HGNC Symbol;Acc:HGNC:28643]	-1.6	-2
3531	*CRK*	1398	v-crk avian sarcoma virus CT10 oncogene homolog [Source:HGNC Symbol;Acc:HGNC:2362]	-1.6	-2
3532	*FRY*	10129	FRY microtubule binding protein [Source:HGNC Symbol;Acc:HGNC:20367]	-1.6	-2
3533	*SFRP4*	6424	secreted frizzled related protein 4 [Source:HGNC Symbol;Acc:HGNC:10778]	-1.6	-2
3534	*SSFA2*	6744	sperm specific antigen 2 [Source:HGNC Symbol;Acc:HGNC:11319]	-1.6	-2
3535	*AKAP3*	10566	A-kinase anchoring protein 3 [Source:HGNC Symbol;Acc:HGNC:373]	-1.6	-2
3536	*PPRC1*	23082	peroxisome proliferator-activated receptor gamma, coactivator-related 1 [Source:HGNC Symbol;Acc:HGNC:30025]	-1.6	-2
3537	*KIAA0368*	23392	KIAA0368 [Source:HGNC Symbol;Acc:HGNC:29020]	-1.6	-2
3538	*CAT*	847	catalase [Source:HGNC Symbol;Acc:HGNC:1516]	-1.6	-2
3539	*SEC22B*	9554	SEC22 homolog B, vesicle trafficking protein (gene/pseudogene) [Source:HGNC Symbol;Acc:HGNC:10700]	-1.6	-2
3540	*WDR43*	23160	WD repeat domain 43 [Source:HGNC Symbol;Acc:HGNC:28945]	-1.6	-2
3541	*MST1L*	11223	macrophage stimulating 1-like [Source:HGNC Symbol;Acc:HGNC:7390]	-1.6	-2
3542	*UGCG*	7357	UDP-glucose ceramide glucosyltransferase [Source:HGNC Symbol;Acc:HGNC:12524]	-1.6	-2
3543	*ABRACL*	58527	ABRA C-terminal like [Source:HGNC Symbol;Acc:HGNC:21230]	-1.6	-2
3544	*PNLIPRP3*	119548	pancreatic lipase related protein 3 [Source:HGNC Symbol;Acc:HGNC:23492]	-1.6	-2
3545	*RPL12*	6136	ribosomal protein L12 [Source:HGNC Symbol;Acc:HGNC:10302]	-1.6	-2
3546	*NEMF*	9147	nuclear export mediator factor [Source:HGNC Symbol;Acc:HGNC:10663]	-1.6	-2
3547	*FYCO1*	79443	FYVE and coiled-coil domain containing 1 [Source:HGNC Symbol;Acc:HGNC:14673]	-1.6	-2
3548	*SLC25A6*	293	solute carrier family 25 member 6 [Source:HGNC Symbol;Acc:HGNC:10992]	-1.6	-2
3549	*PRDM2*	7799	PR domain 2 [Source:HGNC Symbol;Acc:HGNC:9347]	-1.6	-2
3550	*HSPA9*	3313	heat shock protein family A (Hsp70) member 9 [Source:HGNC Symbol;Acc:HGNC:5244]	-1.6	-2
3551	*NUP107*	57122	nucleoporin 107 [Source:HGNC Symbol;Acc:HGNC:29914]	-1.6	-2
3552	*PRKAR2A*	5576	protein kinase cAMP-dependent type II regulatory subunit alpha [Source:HGNC Symbol;Acc:HGNC:9391]	-1.6	-2
3553	*KRCC1*	51315	lysine rich coiled-coil 1 [Source:HGNC Symbol;Acc:HGNC:28039]	-1.6	-2
3554	*ATAD3A*	55210	ATPase family, AAA domain containing 3A [Source:HGNC Symbol;Acc:HGNC:25567]	-1.6	-2
3555	*WARS*	7453	tryptophanyl-tRNA synthetase [Source:HGNC Symbol;Acc:HGNC:12729]	-1.6	-2
3556	*PRDX5*	25824	peroxiredoxin 5 [Source:HGNC Symbol;Acc:HGNC:9355]	-1.6	-2
3557	*DCTN4*	51164	dynactin subunit 4 [Source:HGNC Symbol;Acc:HGNC:15518]	-1.6	-2
3558	*PRKACA*	5566	protein kinase cAMP-activated catalytic subunit alpha [Source:HGNC Symbol;Acc:HGNC:9380]	-1.6	-2
3559	*SEPT9*	10801	septin 9 [Source:HGNC Symbol;Acc:HGNC:7323]	-1.6	-2
3560	*RAP2C*	57826	RAP2C, member of RAS oncogene family [Source:HGNC Symbol;Acc:HGNC:21165]	-1.6	-2
3561	*SPAST*	6683	spastin [Source:HGNC Symbol;Acc:HGNC:11233]	-1.6	-2
3562	*CCDC47*	57003	coiled-coil domain containing 47 [Source:HGNC Symbol;Acc:HGNC:24856]	-1.6	-2
3563	*TBL1X*	6907	transducin (beta)-like 1X-linked [Source:HGNC Symbol;Acc:HGNC:11585]	-1.6	-2
3564	*PANK3*	79646	pantothenate kinase 3 [Source:HGNC Symbol;Acc:HGNC:19365]	-1.6	-2
3565	*WIPF3*	644150	WAS/WASL interacting protein family member 3 [Source:HGNC Symbol;Acc:HGNC:22004]	-1.6	-2
3566	*IPO9*	55705	importin 9 [Source:HGNC Symbol;Acc:HGNC:19425]	-1.6	-2
3567	*MCM5*	4174	minichromosome maintenance complex component 5 [Source:HGNC Symbol;Acc:HGNC:6948]	-1.6	-2
3568	*EIF4B*	1975	eukaryotic translation initiation factor 4B [Source:HGNC Symbol;Acc:HGNC:3285]	-1.6	-2
3569	*ZBED4*	9889	zinc finger BED-type containing 4 [Source:HGNC Symbol;Acc:HGNC:20721]	-1.6	-2
3570	*CD300E*	342510	CD300e molecule [Source:HGNC Symbol;Acc:HGNC:28874]	-1.6	-2
3571	*NIFK*	84365	nucleolar protein interacting with the FHA domain of MKI67 [Source:HGNC Symbol;Acc:HGNC:17838]	-1.6	-2
3572	*MORC2*	22880	MORC family CW-type zinc finger 2 [Source:HGNC Symbol;Acc:HGNC:23573]	-1.6	-2
3573	*ZNF746*	155061	zinc finger protein 746 [Source:HGNC Symbol;Acc:HGNC:21948]	-1.6	-2
3574	*VTI1B*	10490	vesicle transport through interaction with t-SNAREs 1B [Source:HGNC Symbol;Acc:HGNC:17793]	-1.6	-2
3575	*AKAP6*	9472	A-kinase anchoring protein 6 [Source:HGNC Symbol;Acc:HGNC:376]	-1.6	-2
3576	*RPL8*	6132	ribosomal protein L8 [Source:HGNC Symbol;Acc:HGNC:10368]	-1.6	-2
3577	*RPL36*	25873	ribosomal protein L36 [Source:HGNC Symbol;Acc:HGNC:13631]	-1.6	-2
3578	*POLE3*	54107	polymerase (DNA) epsilon 3, accessory subunit [Source:HGNC Symbol;Acc:HGNC:13546]	-1.6	-2
3579	*KAZN*	23254	kazrin, periplakin interacting protein [Source:HGNC Symbol;Acc:HGNC:29173]	-1.6	-2
3580	*CD4*	920	CD4 molecule [Source:HGNC Symbol;Acc:HGNC:1678]	-1.6	-2

3581	*POP7*	10248	POP7 homolog, ribonuclease P/MRP subunit [Source:HGNC Symbol;Acc:HGNC:19949]	-1.6	-2
3582	*TRIM14*	9830	tripartite motif containing 14 [Source:HGNC Symbol;Acc:HGNC:16283]	-1.6	-2
3583	*MT1F*	4494	metallothionein 1F [Source:HGNC Symbol;Acc:HGNC:7398]	-1.6	-2
3584	*UQCRC2*	7385	ubiquinol-cytochrome c reductase core protein II [Source:HGNC Symbol;Acc:HGNC:12586]	-1.6	-2
3585	*PTGES3*	10728	prostaglandin E synthase 3 [Source:HGNC Symbol;Acc:HGNC:16049]	-1.6	-2
3586	*TMEM230*	29058	transmembrane protein 230 [Source:HGNC Symbol;Acc:HGNC:15876]	-1.6	-2
3587	*HBEGF*	1839	heparin binding EGF like growth factor [Source:HGNC Symbol;Acc:HGNC:3059]	-1.6	-2
3588	*RGS1*	5996	regulator of G-protein signaling 1 [Source:HGNC Symbol;Acc:HGNC:9991]	-1.6	-2
3589	*FEM1B*	10116	fem-1 homolog B [Source:HGNC Symbol;Acc:HGNC:3649]	-1.6	-2
3590	*DHRS7*	51635	dehydrogenase/reductase 7 [Source:HGNC Symbol;Acc:HGNC:21524]	-1.6	-2
3591	*RPS10*	6204	ribosomal protein S10 [Source:HGNC Symbol;Acc:HGNC:10383]	-1.6	-2
3592	*ANXA4*	307	annexin A4 [Source:HGNC Symbol;Acc:HGNC:542]	-1.6	-2
3593	*HLA-DQB1*	3119	major histocompatibility complex, class II, DQ beta 1 [Source:HGNC Symbol;Acc:HGNC:4944]	-1.6	-2
3594	*MINOS1*	440574	mitochondrial inner membrane organizing system 1 [Source:HGNC Symbol;Acc:HGNC:32068]	-1.6	-2
3595	*NDUFA3*	4696	NADH:ubiquinone oxidoreductase subunit A3 [Source:HGNC Symbol;Acc:HGNC:7686]	-1.6	-2
3596	*HSPD1*	3329	heat shock protein family D (Hsp60) member 1 [Source:HGNC Symbol;Acc:HGNC:5261]	-1.6	-2
3597	*AFF4*	27125	AF4/FMR2 family member 4 [Source:HGNC Symbol;Acc:HGNC:17869]	-1.6	-2
3598	*NDUFV2*	4729	NADH:ubiquinone oxidoreductase core subunit V2 [Source:HGNC Symbol;Acc:HGNC:7717]	-1.6	-2
3599	*HSH2D*	84941	hematopoietic SH2 domain containing [Source:HGNC Symbol;Acc:HGNC:24920]	-1.6	-2
3600	*MTDH*	92140	metadherin [Source:HGNC Symbol;Acc:HGNC:29608]	-1.6	-2
3601	*THOC3*	84321	THO complex 3 [Source:HGNC Symbol;Acc:HGNC:19072]	-1.6	-2
3602	*TAC3*	6866	tachykinin 3 [Source:HGNC Symbol;Acc:HGNC:11521]	-1.6	-2
3603	*PQLC3*	130814	PQ loop repeat containing 3 [Source:HGNC Symbol;Acc:HGNC:28503]	-1.6	-2
3604	*TRPV2*	51393	transient receptor potential cation channel subfamily V member 2 [Source:HGNC Symbol;Acc:HGNC:18082]	-1.6	-2
3605	*CYB5R4*	51167	cytochrome b5 reductase 4 [Source:HGNC Symbol;Acc:HGNC:20147]	-1.6	-2
3606	*ANXA5*	308	annexin A5 [Source:HGNC Symbol;Acc:HGNC:543]	-1.6	-2
3607	*MKNK1*	8569	MAP kinase interacting serine/threonine kinase 1 [Source:HGNC Symbol;Acc:HGNC:7110]	-1.6	-2
3608	*PHPT1*	29085	phosphohistidine phosphatase 1 [Source:HGNC Symbol;Acc:HGNC:30033]	-1.6	-2
3609	*KLHDC4*	54758	kelch domain containing 4 [Source:HGNC Symbol;Acc:HGNC:25272]	-1.6	-2
3610	*SURF4*	6836	surfeit 4 [Source:HGNC Symbol;Acc:HGNC:11476]	-1.6	-2
3611	*RPS6*	6194	ribosomal protein S6 [Source:HGNC Symbol;Acc:HGNC:10429]	-1.6	-2
3612	*RNF146*	81847	ring finger protein 146 [Source:HGNC Symbol;Acc:HGNC:21336]	-1.6	-2
3613	*RBM8A*	9939	RNA binding motif protein 8A [Source:HGNC Symbol;Acc:HGNC:9905]	-1.6	-2
3614	*MRPL30*	51263	mitochondrial ribosomal protein L30 [Source:HGNC Symbol;Acc:HGNC:14036]	-1.6	-2
3615	*SPCS1*	28972	signal peptidase complex subunit 1 [Source:HGNC Symbol;Acc:HGNC:23401]	-1.6	-2
3616	*UTP15*	84135	UTP15, small subunit processome component [Source:HGNC Symbol;Acc:HGNC:25758]	-1.6	-2
3617	*SEC24D*	9871	SEC24 homolog D, COPII coat complex component [Source:HGNC Symbol;Acc:HGNC:10706]	-1.6	-2
3618	*RHOQ*	23433	ras homolog family member Q [Source:HGNC Symbol;Acc:HGNC:17736]	-1.6	-2
3619	*SNRPD2*	6633	small nuclear ribonucleoprotein D2 polypeptide [Source:HGNC Symbol;Acc:HGNC:11159]	-1.6	-2
3620	*GOLGB1*	2804	golgin B1 [Source:HGNC Symbol;Acc:HGNC:4429]	-1.6	-2
3621	*FLOT1*	10211	flotillin 1 [Source:HGNC Symbol;Acc:HGNC:3757]	-1.6	-2
3622	*RPL26*	6154	ribosomal protein L26 [Source:HGNC Symbol;Acc:HGNC:10327]	-1.6	-2
3623	*NMD3*	51068	NMD3 ribosome export adaptor [Source:HGNC Symbol;Acc:HGNC:24250]	-1.6	-2
3624	*CNOT2*	4848	CCR4-NOT transcription complex subunit 2 [Source:HGNC Symbol;Acc:HGNC:7878]	-1.6	-2
3625	*PAPL*	390928	Acid Phosphatase 7, Tartrate Resistant	-1.6	-2
3626	*CCL14*	6358	C-C motif chemokine ligand 14 [Source:HGNC Symbol;Acc:HGNC:10612]	-1.6	-2
3627	*HSPB8*	26353	heat shock protein family B (small) member 8 [Source:HGNC Symbol;Acc:HGNC:30171]	-1.6	-2
3628	*TMEM52B*	120939	transmembrane protein 52B [Source:HGNC Symbol;Acc:HGNC:26438]	-1.6	-2
3629	*EXOSC6*	118460	exosome component 6 [Source:HGNC Symbol;Acc:HGNC:19055]	-1.6	-2
3630	*SMAP1*	60682	small ArfGAP 1 [Source:HGNC Symbol;Acc:HGNC:19651]	-1.6	-2
3631	*PDZD11*	51248	PDZ domain containing 11 [Source:HGNC Symbol;Acc:HGNC:28034]	-1.6	-2
3632	*PIK3C2A*	5286	phosphatidylinositol-4-phosphate 3-kinase catalytic subunit type 2 alpha [Source:HGNC Symbol;Acc:HGNC:8971]	-1.6	-2
3633	*SLK*	9748	STE20 like kinase [Source:HGNC Symbol;Acc:HGNC:11088]	-1.6	-2
3634	*OR8S1*	341568	olfactory receptor family 8 subfamily S member 1 [Source:HGNC Symbol;Acc:HGNC:19628]	-1.6	-2
3635	*DEAF1*	10522	DEAF1, transcription factor [Source:HGNC Symbol;Acc:HGNC:14677]	-1.6	-2
3636	*C3orf67*	200844	chromosome 3 open reading frame 67 [Source:HGNC Symbol;Acc:HGNC:24763]	-1.6	-2
3637	*RFK*	55312	riboflavin kinase [Source:HGNC Symbol;Acc:HGNC:30324]	-1.6	-2
3638	*C11orf85*	283129	chromosome 11 open reading frame 85	-1.6	-2
3639	*RAB1A*	5861	RAB1A, member RAS oncogene family [Source:HGNC Symbol;Acc:HGNC:9758]	-1.6	-2
3640	*PSMA2*	5683	proteasome subunit alpha 2 [Source:HGNC Symbol;Acc:HGNC:9531]	-1.6	-2
3641	*SMG1*	23049	SMG1, nonsense mediated mRNA decay associated PI3K related kinase [Source:HGNC Symbol;Acc:HGNC:30045]	-1.6	-2
3642	*PLEKHA1*	59338	pleckstrin homology domain containing A1 [Source:HGNC Symbol;Acc:HGNC:14335]	-1.6	-2
3643	*ANKRD20A2*	441430	ankyrin repeat domain 20 family member A2 [Source:HGNC Symbol;Acc:HGNC:31979]	-1.6	-2
3644	*KHK*	3795	ketohexokinase [Source:HGNC Symbol;Acc:HGNC:6315]	-1.6	-2
3645	*PXYLP1*	92370	2-phosphoxylose phosphatase 1 [Source:HGNC Symbol;Acc:HGNC:26303]	-1.6	-2
3646	*AP5B1*	91056	adaptor related protein complex 5 beta 1 subunit [Source:HGNC Symbol;Acc:HGNC:25104]	-1.6	-2
3647	*SMAD3*	4088	SMAD family member 3 [Source:HGNC Symbol;Acc:HGNC:6769]	-1.6	-2
3648	*EEF1G*	1937	eukaryotic translation elongation factor 1 gamma [Source:HGNC Symbol;Acc:HGNC:3213]	-1.6	-2
3649	*RPS13*	6207	ribosomal protein S13 [Source:HGNC Symbol;Acc:HGNC:10386]	-1.6	-2
3650	*BRD3*	8019	bromodomain containing 3 [Source:HGNC Symbol;Acc:HGNC:1104]	-1.6	-2
3651	*NDUFA13*	51079	NADH:ubiquinone oxidoreductase subunit A13 [Source:HGNC Symbol;Acc:HGNC:17194]	-1.6	-2
3652	*HLA-DPB1*	3115	major histocompatibility complex, class II, DP beta 1 [Source:HGNC Symbol;Acc:HGNC:4940]	-1.6	-2

3653	*NDRG4*	65009	NDRG family member 4 [Source:HGNC Symbol;Acc:HGNC:14466]	-1.6	-2
3654	*KRTCAP2*	200185	keratinocyte associated protein 2 [Source:HGNC Symbol;Acc:HGNC:28942]	-1.6	-2
3655	*ATP5G3*	518	ATP synthase, H+ transporting, mitochondrial Fo complex subunit C3 (subunit 9) [Source:HGNC Symbol;Acc:HGNC:843]	-1.6	-2
3656	*IRF2BP2*	359948	interferon regulatory factor 2 binding protein 2 [Source:HGNC Symbol;Acc:HGNC:21729]	-1.6	-2
3657	*OLA1*	29789	Obg-like ATPase 1 [Source:HGNC Symbol;Acc:HGNC:28833]	-1.6	-2
3658	*BTBD7*	55727	BTB domain containing 7 [Source:HGNC Symbol;Acc:HGNC:18269]	-1.6	-2
3659	*POLR1E*	64425	polymerase (RNA) I subunit E [Source:HGNC Symbol;Acc:HGNC:17631]	-1.6	-2
3660	*KIDINS220*	57498	kinase D-interacting substrate 220kDa [Source:HGNC Symbol;Acc:HGNC:29508]	-1.6	-2
3661	*RPS8*	6202	ribosomal protein S8 [Source:HGNC Symbol;Acc:HGNC:10441]	-1.6	-2
3662	*LY86*	9450	lymphocyte antigen 86 [Source:HGNC Symbol;Acc:HGNC:16837]	-1.6	-2
3663	*IPO7*	10527	importin 7 [Source:HGNC Symbol;Acc:HGNC:9852]	-1.6	-2
3664	*ZNF541*	84215	zinc finger protein 541 [Source:HGNC Symbol;Acc:HGNC:25294]	-1.6	-2
3665	*VBP1*	7411	VHL binding protein 1 [Source:HGNC Symbol;Acc:HGNC:12662]	-1.6	-2
3666	*VEZT*	55591	vezatin, adherens junctions transmembrane protein [Source:HGNC Symbol;Acc:HGNC:18258]	-1.6	-2
3667	*CACNB4*	785	calcium voltage-gated channel auxiliary subunit beta 4 [Source:HGNC Symbol;Acc:HGNC:1404]	-1.6	-2
3668	*RPL23*	9349	ribosomal protein L23 [Source:HGNC Symbol;Acc:HGNC:10316]	-1.6	-2
3669	*KAT5*	10524	lysine acetyltransferase 5 [Source:HGNC Symbol;Acc:HGNC:5275]	-1.6	-2
3670	*RNASEH2C*	84153	ribonuclease H2 subunit C [Source:HGNC Symbol;Acc:HGNC:24116]	-1.6	-2
3671	*RECK*	8434	reversion inducing cysteine rich protein with kazal motifs [Source:HGNC Symbol;Acc:HGNC:11345]	-1.6	-2
3672	*CERKL*	375298	ceramide kinase like [Source:HGNC Symbol;Acc:HGNC:21699]	-1.6	-2
3673	*CCS*	9973	copper chaperone for superoxide dismutase [Source:HGNC Symbol;Acc:HGNC:1613]	-1.6	-2
3674	*TRIM23*	373	tripartite motif containing 23 [Source:HGNC Symbol;Acc:HGNC:660]	-1.6	-2
3675	*PCK2*	5106	phosphoenolpyruvate carboxykinase 2, mitochondrial [Source:HGNC Symbol;Acc:HGNC:8725]	-1.6	-2
3676	*OAT*	4942	ornithine aminotransferase [Source:HGNC Symbol;Acc:HGNC:8091]	-1.6	-2
3677	*ACADM*	34	acyl-CoA dehydrogenase, C-4 to C-12 straight chain [Source:HGNC Symbol;Acc:HGNC:89]	-1.6	-2
3678	*FXYD1*	5348	FXYD domain containing ion transport regulator 1 [Source:HGNC Symbol;Acc:HGNC:4025]	-1.6	-2
3679	*NDUFV1*	4723	NADH:ubiquinone oxidoreductase core subunit V1 [Source:HGNC Symbol;Acc:HGNC:7716]	-1.6	-2
3680	*ZNF503*	84858	zinc finger protein 503 [Source:HGNC Symbol;Acc:HGNC:23589]	-1.6	-2
3681	*HNMT*	3176	histamine N-methyltransferase [Source:HGNC Symbol;Acc:HGNC:5028]	-1.6	-2
3682	*ETNK1*	55500	ethanolamine kinase 1 [Source:HGNC Symbol;Acc:HGNC:24649]	-1.6	-2
3683	*ECT2*	1894	epithelial cell transforming 2 [Source:HGNC Symbol;Acc:HGNC:3155]	-1.6	-2
3684	*RPL24*	6152	ribosomal protein L24 [Source:HGNC Symbol;Acc:HGNC:10325]	-1.6	-2
3685	*PITPNC1*	26207	phosphatidylinositol transfer protein, cytoplasmic 1 [Source:HGNC Symbol;Acc:HGNC:21045]	-1.6	-2
3686	*CUL2*	8453	cullin 2 [Source:HGNC Symbol;Acc:HGNC:2552]	-1.6	-2
3687	*ZFC3H1*	196441	zinc finger C3H1-type containing [Source:HGNC Symbol;Acc:HGNC:28328]	-1.6	-2
3688	*XPO1*	7514	exportin 1 [Source:HGNC Symbol;Acc:HGNC:12825]	-1.6	-2
3689	*C8orf44*	56260	chromosome 8 open reading frame 44 [Source:HGNC Symbol;Acc:HGNC:25646]	-1.6	-2
3690	*TC2N*	123036	tandem C2 domains, nuclear [Source:HGNC Symbol;Acc:HGNC:19859]	-1.6	-2
3691	*TTC14*	151613	tetratricopeptide repeat domain 14 [Source:HGNC Symbol;Acc:HGNC:24697]	-1.6	-2
3692	*PXMP2*	5827	peroxisomal membrane protein 2 [Source:HGNC Symbol;Acc:HGNC:9716]	-1.6	-2
3693	*RPL13*	6137	ribosomal protein L13 [Source:HGNC Symbol;Acc:HGNC:10303]	-1.6	-2
3694	*SH3GLB1*	51100	SH3 domain containing GRB2 like endophilin B1 [Source:HGNC Symbol;Acc:HGNC:10833]	-1.6	-2
3695	*OR11A1*	26531	olfactory receptor family 11 subfamily A member 1 [Source:HGNC Symbol;Acc:HGNC:8176]	-1.6	-2
3696	*NSA2*	10412	NSA2, ribosome biogenesis homolog [Source:HGNC Symbol;Acc:HGNC:30728]	-1.6	-2
3697	*PEA15*	8682	phosphoprotein enriched in astrocytes 15 [Source:HGNC Symbol;Acc:HGNC:8822]	-1.6	-2
3698	*SPAG9*	9043	sperm associated antigen 9 [Source:HGNC Symbol;Acc:HGNC:14524]	-1.6	-2
3699	*DSC2*	1824	desmocollin 2 [Source:HGNC Symbol;Acc:HGNC:3036]	-1.6	-2
3700	*CSTF3*	1479	cleavage stimulation factor subunit 3 [Source:HGNC Symbol;Acc:HGNC:2485]	-1.6	-2
3701	*KCNH4*	23415	potassium voltage-gated channel subfamily H member 4 [Source:HGNC Symbol;Acc:HGNC:6253]	-1.6	-2
3702	*EID1*	23741	EP300 interacting inhibitor of differentiation 1 [Source:HGNC Symbol;Acc:HGNC:1191]	-1.6	-2
3703	*FYTTD1*	84248	forty-two-three domain containing 1 [Source:HGNC Symbol;Acc:HGNC:25407]	-1.6	-2
3704	*FXYD4*	53828	FXYD domain containing ion transport regulator 4 [Source:HGNC Symbol;Acc:HGNC:4028]	-1.6	-2
3705	*SEC62*	7095	SEC62 homolog, preprotein translocation factor [Source:HGNC Symbol;Acc:HGNC:11846]	-1.6	-2
3706	*TRAPPC2L*	51693	trafficking protein particle complex 2-like [Source:HGNC Symbol;Acc:HGNC:30887]	-1.6	-2
3707	*JUP*	3728	junction plakoglobin [Source:HGNC Symbol;Acc:HGNC:6207]	-1.6	-2
3708	*HDLBP*	3069	high density lipoprotein binding protein [Source:HGNC Symbol;Acc:HGNC:4857]	-1.6	-2
3709	*LIX1L*	128077	limb and CNS expressed 1 like [Source:HGNC Symbol;Acc:HGNC:28715]	-1.6	-2
3710	*COG4*	25839	component of oligomeric golgi complex 4 [Source:HGNC Symbol;Acc:HGNC:18620]	-1.6	-2
3711	*TMEM126B*	55863	transmembrane protein 126B [Source:HGNC Symbol;Acc:HGNC:30883]	-1.6	-2
3712	*EPC1*	80314	enhancer of polycomb homolog 1 [Source:HGNC Symbol;Acc:HGNC:19876]	-1.6	-2
3713	*ATP5A1*	498	ATP synthase, H+ transporting, mitochondrial F1 complex, alpha subunit 1, cardiac muscle [Source:HGNC Symbol;Acc:HGNC:823]	-1.6	-2
3714	*RPL10*	6134	ribosomal protein L10 [Source:HGNC Symbol;Acc:HGNC:10298]	-1.6	-2
3715	*F2RL3*	347745	F2R like thrombin/trypsin receptor 3 [Source:HGNC Symbol;Acc:HGNC:3540]	-1.6	-2
3716	*MRPS35*	60488	mitochondrial ribosomal protein S35 [Source:HGNC Symbol;Acc:HGNC:16635]	-1.6	-2
3717	*EIF3B*	8662	eukaryotic translation initiation factor 3 subunit B [Source:HGNC Symbol;Acc:HGNC:3280]	-1.6	-2
3718	*SNRK*	54861	SNF related kinase [Source:HGNC Symbol;Acc:HGNC:30598]	-1.6	-2
3719	*STX7*	8417	syntaxin 7 [Source:HGNC Symbol;Acc:HGNC:11442]	-1.6	-2
3720	*ITGAD*	3681	integrin subunit alpha D [Source:HGNC Symbol;Acc:HGNC:6146]	-1.6	-2
3721	*ATP13A3*	79572	ATPase 13A3 [Source:HGNC Symbol;Acc:HGNC:24113]	-1.6	-2

3722	*RPL32*	6161	ribosomal protein L32 [Source:HGNC Symbol;Acc:HGNC:10336]	-1.6	-2
3723	*ELP2*	55250	elongator acetyltransferase complex subunit 2 [Source:HGNC Symbol;Acc:HGNC:18248]	-1.6	-2
3724	*RAN*	5901	RAN, member RAS oncogene family [Source:HGNC Symbol;Acc:HGNC:9846]	-1.6	-2
3725	*MAPK6*	5597	mitogen-activated protein kinase 6 [Source:HGNC Symbol;Acc:HGNC:6879]	-1.6	-2
3726	*RPLP0*	6175	ribosomal protein lateral stalk subunit P0 [Source:HGNC Symbol;Acc:HGNC:10371]	-1.6	-2
3727	*ARID5B*	84159	AT-rich interaction domain 5B [Source:HGNC Symbol;Acc:HGNC:17362]	-1.6	-2
3728	*RPS16*	6217	ribosomal protein S16 [Source:HGNC Symbol;Acc:HGNC:10396]	-1.6	-2
3729	*BORCS7*	119032	BLOC-1 related complex subunit 7 [Source:HGNC Symbol;Acc:HGNC:23516]	-1.6	-2
3730	*PUM2*	23369	pumilio RNA binding family member 2 [Source:HGNC Symbol;Acc:HGNC:14958]	-1.6	-2
3731	*COPS4*	51138	COP9 signalosome subunit 4 [Source:HGNC Symbol;Acc:HGNC:16702]	-1.6	-2
3732	*EIF2S3*	1968	eukaryotic translation initiation factor 2 subunit gamma [Source:HGNC Symbol;Acc:HGNC:3267]	-1.6	-2
3733	*DNLZ*	728489	DNL-type zinc finger [Source:HGNC Symbol;Acc:HGNC:33879]	-1.6	-2
3734	*CCT8*	10694	chaperonin containing TCP1 subunit 8 [Source:HGNC Symbol;Acc:HGNC:1623]	-1.6	-2
3735	*SMIM19*	114926	small integral membrane protein 19 [Source:HGNC Symbol;Acc:HGNC:25166]	-1.6	-2
3736	*JKAMP*	51528	JNK1/MAPK8-associated membrane protein [Source:HGNC Symbol;Acc:HGNC:20184]	-1.6	-2
3737	*ACAN*	176	aggrecan [Source:HGNC Symbol;Acc:HGNC:319]	-1.6	-2
3738	*RPL14*	9045	ribosomal protein L14 [Source:HGNC Symbol;Acc:HGNC:10305]	-1.6	-2
3739	*PSKH1*	5681	protein serine kinase H1 [Source:HGNC Symbol;Acc:HGNC:9529]	-1.6	-2
3740	*NEDD9*	4739	neural precursor cell expressed, developmentally down-regulated 9 [Source:HGNC Symbol;Acc:HGNC:7733]	-1.6	-2
3741	*BFAR*	51283	bifunctional apoptosis regulator [Source:HGNC Symbol;Acc:HGNC:17613]	-1.6	-2
3742	*SRSF5*	6430	serine and arginine rich splicing factor 5 [Source:HGNC Symbol;Acc:HGNC:10787]	-1.6	-2
3743	*NDUFB7*	4713	NADH:ubiquinone oxidoreductase subunit B7 [Source:HGNC Symbol;Acc:HGNC:7702]	-1.6	-2
3744	*KHDC1*	80759	KH homology domain containing 1 [Source:HGNC Symbol;Acc:HGNC:21366]	-1.6	-2
3745	*PPOX*	5498	protoporphyrinogen oxidase [Source:HGNC Symbol;Acc:HGNC:9280]	-1.6	-2
3746	*PFKFB1*	5207	6-phosphofructo-2-kinase/fructose-2,6-biphosphatase 1 [Source:HGNC Symbol;Acc:HGNC:8872]	-1.6	-2
3747	*PFDN5*	5204	prefoldin subunit 5 [Source:HGNC Symbol;Acc:HGNC:8869]	-1.6	-2
3748	*OR51E1*	143503	olfactory receptor family 51 subfamily E member 1 [Source:HGNC Symbol;Acc:HGNC:15194]	-1.6	-2
3749	*FREM2*	341640	FRAS1 related extracellular matrix protein 2 [Source:HGNC Symbol;Acc:HGNC:25396]	-1.6	-2
3750	*YPEL1*	29799	yippee like 1 [Source:HGNC Symbol;Acc:HGNC:12845]	-1.6	-2
3751	*DDX50*	79009	DEAD-box helicase 50 [Source:HGNC Symbol;Acc:HGNC:17906]	-1.6	-2
3752	*YTHDC2*	64848	YTH domain containing 2 [Source:HGNC Symbol;Acc:HGNC:24721]	-1.6	-2
3753	*STXBP3*	6814	syntaxin binding protein 3 [Source:HGNC Symbol;Acc:HGNC:11446]	-1.6	-2
3754	*NFYB*	4801	nuclear transcription factor Y subunit beta [Source:HGNC Symbol;Acc:HGNC:7805]	-1.6	-2
3755	*RPS17*	6218	ribosomal protein S17 [Source:HGNC Symbol;Acc:HGNC:10397]	-1.6	-2
3756	*KDELR2*	11014	KDEL endoplasmic reticulum protein retention receptor 2 [Source:HGNC Symbol;Acc:HGNC:6305]	-1.6	-2
3757	*TARDBP*	23435	TAR DNA binding protein [Source:HGNC Symbol;Acc:HGNC:11571]	-1.6	-2
3758	*PJA2*	9867	praja ring finger ubiquitin ligase 2 [Source:HGNC Symbol;Acc:HGNC:17481]	-1.6	-2
3759	*ADAR*	103	adenosine deaminase, RNA specific [Source:HGNC Symbol;Acc:HGNC:225]	-1.6	-2
3760	*C9orf64*	84267	chromosome 9 open reading frame 64 [Source:HGNC Symbol;Acc:HGNC:28144]	-1.6	-2
3761	*HLA-DRB3*	3125	major histocompatibility complex, class II, DR beta 3 [Source:HGNC Symbol;Acc:HGNC:4951]	-1.6	-2
3762	*PDHB*	5162	pyruvate dehydrogenase (lipoamide) beta [Source:HGNC Symbol;Acc:HGNC:8808]	-1.6	-2
3763	*LARS*	51520	leucyl-tRNA synthetase [Source:HGNC Symbol;Acc:HGNC:6512]	-1.6	-2
3764	*GOLGA2*	2801	golgin A2 [Source:HGNC Symbol;Acc:HGNC:4425]	-1.6	-2
3765	*DNAJB11*	51726	DnaJ heat shock protein family (Hsp40) member B11 [Source:HGNC Symbol;Acc:HGNC:14889]	-1.6	-2
3766	*GRP*	2922	gastrin releasing peptide [Source:HGNC Symbol;Acc:HGNC:4605]	-1.6	-2
3767	*GALP*	85569	galanin like peptide [Source:HGNC Symbol;Acc:HGNC:24840]	-1.6	-2
3768	*CLPTM1L*	81037	CLPTM1-like [Source:HGNC Symbol;Acc:HGNC:24308]	-1.6	-2
3769	*SLC2A6*	11182	solute carrier family 2 member 6 [Source:HGNC Symbol;Acc:HGNC:11011]	-1.6	-2
3770	*MRGPRF*	116535	MAS related GPR family member F [Source:HGNC Symbol;Acc:HGNC:24828]	-1.6	-2
3771	*ATP5B*	506	ATP synthase, H+ transporting, mitochondrial F1 complex, beta polypeptide [Source:HGNC Symbol;Acc:HGNC:830]	-1.6	-2
3772	*RPS18*	6222	ribosomal protein S18 [Source:HGNC Symbol;Acc:HGNC:10401]	-1.6	-2
3773	*KCTD18*	130535	potassium channel tetramerization domain containing 18 [Source:HGNC Symbol;Acc:HGNC:26446]	-1.6	-2
3774	*BAZ1A*	11177	bromodomain adjacent to zinc finger domain 1A [Source:HGNC Symbol;Acc:HGNC:960]	-1.6	-2
3775	*PPP1R21*	129285	protein phosphatase 1 regulatory subunit 21 [Source:HGNC Symbol;Acc:HGNC:30595]	-1.6	-2
3776	*RGS4*	5999	regulator of G-protein signaling 4 [Source:HGNC Symbol;Acc:HGNC:10000]	-1.6	-2
3777	*UBXN1*	51035	UBX domain protein 1 [Source:HGNC Symbol;Acc:HGNC:18402]	-1.6	-2
3778	*DNAJA1*	3301	DnaJ heat shock protein family (Hsp40) member A1 [Source:HGNC Symbol;Acc:HGNC:5229]	-1.6	-2
3779	*SHTN1*	57698	shootin 1 [Source:HGNC Symbol;Acc:HGNC:29319]	-1.6	-2
3780	*TRMT44*	152992	tRNA methyltransferase 44 homolog (S. cerevisiae) [Source:HGNC Symbol;Acc:HGNC:26653]	-1.6	-2
3781	*MAGT1*	84061	magnesium transporter 1 [Source:HGNC Symbol;Acc:HGNC:28880]	-1.6	-2
3782	*CPNE3*	8895	copine 3 [Source:HGNC Symbol;Acc:HGNC:2316]	-1.6	-2
3783	*SNU13*	4809	SNU13 homolog, small nuclear ribonucleoprotein (U4/U6.U5) [Source:HGNC Symbol;Acc:HGNC:7819]	-1.6	-2
3784	*CCT4*	10575	chaperonin containing TCP1 subunit 4 [Source:HGNC Symbol;Acc:HGNC:1617]	-1.6	-2
3785	*TOPBP1*	11073	topoisomerase (DNA) II binding protein 1 [Source:HGNC Symbol;Acc:HGNC:17008]	-1.6	-2
3786	*RPL22*	6146	ribosomal protein L22 [Source:HGNC Symbol;Acc:HGNC:10315]	-1.6	-2
3787	*LFNG*	3955	LFNG O-fucosylpeptide 3-beta-N-acetylglucosaminyltransferase [Source:HGNC Symbol;Acc:HGNC:6560]	-1.6	-2
3788	*ZDHHC7*	55625	zinc finger DHHC-type containing 7 [Source:HGNC Symbol;Acc:HGNC:18459]	-1.6	-2
3789	*GALNT1*	2589	polypeptide N-acetylgalactosaminyltransferase 1 [Source:HGNC Symbol;Acc:HGNC:4123]	-1.6	-2
3790	*RPLP2*	6181	ribosomal protein lateral stalk subunit P2 [Source:HGNC Symbol;Acc:HGNC:10377]	-1.6	-2
3791	*PMVK*	10654	phosphomevalonate kinase [Source:HGNC Symbol;Acc:HGNC:9141]	-1.6	-2
3792	*NDUFS6*	4726	NADH:ubiquinone oxidoreductase subunit S6 [Source:HGNC Symbol;Acc:HGNC:7713]	-1.6	-2

3793	*SEC23IP*	11196	SEC23 interacting protein [Source:HGNC Symbol;Acc:HGNC:17018]	-1.6	-2
3794	*HIST2H2AC*	8338	histone cluster 2, H2ac [Source:HGNC Symbol;Acc:HGNC:4738]	-1.6	-2
3795	*UBL3*	5412	ubiquitin like 3 [Source:HGNC Symbol;Acc:HGNC:12504]	-1.6	-2
3796	*MCF2L*	23263	MCF.2 cell line derived transforming sequence like [Source:HGNC Symbol;Acc:HGNC:14576]	-1.6	-2
3797	*TRIM51*	84767	tripartite motif-containing 51 [Source:HGNC Symbol;Acc:HGNC:19023]	-1.6	-2
3798	*RPL6*	6128	ribosomal protein L6 [Source:HGNC Symbol;Acc:HGNC:10362]	-1.6	-2
3799	*PIGF*	5281	phosphatidylinositol glycan anchor biosynthesis class F [Source:HGNC Symbol;Acc:HGNC:8962]	-1.6	-2
3800	*TTC3*	7267	tetratricopeptide repeat domain 3 [Source:HGNC Symbol;Acc:HGNC:12393]	-1.6	-2
3801	*DNAJC1*	64215	DnaJ heat shock protein family (Hsp40) member C1 [Source:HGNC Symbol;Acc:HGNC:20090]	-1.6	-2
3802	*DDB2*	1643	damage specific DNA binding protein 2 [Source:HGNC Symbol;Acc:HGNC:2718]	-1.6	-2
3803	*CACUL1*	143384	CDK2 associated cullin domain 1 [Source:HGNC Symbol;Acc:HGNC:23727]	-1.6	-2
3804	*GATM*	2628	glycine amidinotransferase [Source:HGNC Symbol;Acc:HGNC:4175]	-1.6	-2
3805	*RAB3D*	9545	RAB3D, member RAS oncogene family [Source:HGNC Symbol;Acc:HGNC:9779]	-1.6	-2
3806	*TRIM33*	51592	tripartite motif containing 33 [Source:HGNC Symbol;Acc:HGNC:16290]	-1.6	-2
3807	*TMLHE*	55217	trimethyllysine hydroxylase, epsilon [Source:HGNC Symbol;Acc:HGNC:18308]	-1.6	-2
3808	*FOXO3*	2309	forkhead box O3 [Source:HGNC Symbol;Acc:HGNC:3821]	-1.6	-2
3809	*CCDC77*	84318	coiled-coil domain containing 77 [Source:HGNC Symbol;Acc:HGNC:28203]	-1.6	-2
3810	*CARKD*	55739	NAD(P)HX Dehydratase	-1.6	-2
3811	*CRELD2*	79174	cysteine rich with EGF like domains 2 [Source:HGNC Symbol;Acc:HGNC:28150]	-1.6	-2
3812	*GMPS*	8833	guanine monophosphate synthase [Source:HGNC Symbol;Acc:HGNC:4378]	-1.6	-2
3813	*ZNF668*	79759	zinc finger protein 668 [Source:HGNC Symbol;Acc:HGNC:25821]	-1.6	-2
3814	*ARHGAP10*	79658	Rho GTPase activating protein 10 [Source:HGNC Symbol;Acc:HGNC:26099]	-1.6	-2
3815	*RHOB*	388	ras homolog family member B [Source:HGNC Symbol;Acc:HGNC:668]	-1.6	-2
3816	*ATOH8*	84913	atonal bHLH transcription factor 8 [Source:HGNC Symbol;Acc:HGNC:24126]	-1.6	-2
3817	*IMMT*	10989	inner membrane mitochondrial protein [Source:HGNC Symbol;Acc:HGNC:6047]	-1.6	-2
3818	*PPA2*	27068	pyrophosphatase (inorganic) 2 [Source:HGNC Symbol;Acc:HGNC:28883]	-1.6	-2
3819	*THYN1*	29087	thymocyte nuclear protein 1 [Source:HGNC Symbol;Acc:HGNC:29560]	-1.6	-2
3820	*FAM96B*	51647	family with sequence similarity 96 member B [Source:HGNC Symbol;Acc:HGNC:24261]	-1.6	-2
3821	*TAF1D*	79101	TATA-box binding protein associated factor, RNA polymerase I subunit D [Source:HGNC Symbol;Acc:HGNC:28759]	-1.6	-2
3822	*SPG21*	51324	spastic paraplegia 21 (autosomal recessive, Mast syndrome) [Source:HGNC Symbol;Acc:HGNC:20373]	-1.6	-2
3823	*MSTO1*	55154	misato 1, mitochondrial distribution and morphology regulator [Source:HGNC Symbol;Acc:HGNC:29678]	-1.6	-2
3824	*SRXN1*	140809	sulfiredoxin 1 [Source:HGNC Symbol;Acc:HGNC:16132]	-1.6	-2
3825	*RNF222*	643904	ring finger protein 222 [Source:HGNC Symbol;Acc:HGNC:34517]	-1.6	-2
3826	*PNPO*	55163	pyridoxamine 5'-phosphate oxidase [Source:HGNC Symbol;Acc:HGNC:30260]	-1.6	-2
3827	*USP36*	57602	ubiquitin specific peptidase 36 [Source:HGNC Symbol;Acc:HGNC:20062]	-1.6	-2
3828	*CCDC84*	338657	coiled-coil domain containing 84 [Source:HGNC Symbol;Acc:HGNC:30460]	-1.7	-2
3829	*FCN3*	8547	ficolin 3 [Source:HGNC Symbol;Acc:HGNC:3625]	-1.7	-2
3830	*DDX21*	9188	DEAD-box helicase 21 [Source:HGNC Symbol;Acc:HGNC:2744]	-1.7	-2
3831	*SLC38A2*	54407	solute carrier family 38 member 2 [Source:HGNC Symbol;Acc:HGNC:13448]	-1.7	-2
3832	*FBLN5*	10516	fibulin 5 [Source:HGNC Symbol;Acc:HGNC:3602]	-1.7	-2
3833	*TSPAN10*	83882	tetraspanin 10 [Source:HGNC Symbol;Acc:HGNC:29942]	-1.7	-2
3834	*LIPT1*	51601	lipoyltransferase 1 [Source:HGNC Symbol;Acc:HGNC:29569]	-1.7	-2
3835	*N4BP2L2*	10443	NEDD4 binding protein 2-like 2 [Source:HGNC Symbol;Acc:HGNC:26916]	-1.7	-2
3836	*POLR2K*	5440	polymerase (RNA) II subunit K [Source:HGNC Symbol;Acc:HGNC:9198]	-1.7	-2
3837	*HSP90B1*	7184	heat shock protein 90kDa beta family member 1 [Source:HGNC Symbol;Acc:HGNC:12028]	-1.7	-2
3838	*RPL17*	6139	ribosomal protein L17 [Source:HGNC Symbol;Acc:HGNC:10307]	-1.7	-2
3839	*USP14*	9097	ubiquitin specific peptidase 14 [Source:HGNC Symbol;Acc:HGNC:12612]	-1.7	-2
3840	*TMEM14B*	81853	transmembrane protein 14B [Source:HGNC Symbol;Acc:HGNC:21384]	-1.7	-2
3841	*TBCA*	6902	tubulin folding cofactor A [Source:HGNC Symbol;Acc:HGNC:11579]	-1.7	-2
3842	*CXXC5*	51523	CXXC finger protein 5 [Source:HGNC Symbol;Acc:HGNC:26943]	-1.7	-2
3843	*TMEM242*	729515	transmembrane protein 242 [Source:HGNC Symbol;Acc:HGNC:17206]	-1.7	-2
3844	*ZNF365*	22891	zinc finger protein 365 [Source:HGNC Symbol;Acc:HGNC:18194]	-1.7	-2
3845	*SLC2A4*	6517	solute carrier family 2 member 4 [Source:HGNC Symbol;Acc:HGNC:11009]	-1.7	-2
3846	*SUMO1*	7341	small ubiquitin-like modifier 1 [Source:HGNC Symbol;Acc:HGNC:12502]	-1.7	-2
3847	*SULT2B1*	6820	sulfotransferase family 2B member 1 [Source:HGNC Symbol;Acc:HGNC:11459]	-1.7	-2
3848	*VASH1*	22846	vasohibin 1 [Source:HGNC Symbol;Acc:HGNC:19964]	-1.7	-2
3849	*DNAJC8*	22826	DnaJ heat shock protein family (Hsp40) member C8 [Source:HGNC Symbol;Acc:HGNC:15470]	-1.7	-2
3850	*RPS25*	6230	ribosomal protein S25 [Source:HGNC Symbol;Acc:HGNC:10413]	-1.7	-2
3851	*TXNL1*	9352	thioredoxin like 1 [Source:HGNC Symbol;Acc:HGNC:12436]	-1.7	-2
3852	*ZNF189*	7743	zinc finger protein 189 [Source:HGNC Symbol;Acc:HGNC:12980]	-1.7	-2
3853	*TSTA3*	7264	tissue specific transplantation antigen P35B [Source:HGNC Symbol;Acc:HGNC:12390]	-1.7	-2
3854	*WFDC8*	90199	WAP four-disulfide core domain 8 [Source:HGNC Symbol;Acc:HGNC:16163]	-1.7	-2
3855	*TYW1B*	441250	tRNA-yW synthesizing protein 1 homolog B [Source:HGNC Symbol;Acc:HGNC:33908]	-1.7	-2
3856	*ZCRB1*	85437	zinc finger CCHC-type and RNA binding motif containing 1 [Source:HGNC Symbol;Acc:HGNC:29620]	-1.7	-2
3857	*CHRNA6*	8973	cholinergic receptor nicotinic alpha 6 subunit [Source:HGNC Symbol;Acc:HGNC:15963]	-1.7	-2
3858	*LAMP2*	3920	lysosomal associated membrane protein 2 [Source:HGNC Symbol;Acc:HGNC:6501]	-1.7	-2
3859	*SGMS2*	166929	sphingomyelin synthase 2 [Source:HGNC Symbol;Acc:HGNC:28395]	-1.7	-2
3860	*GLTSCR1L*	23506	GLTSCR1 like [Source:HGNC Symbol;Acc:HGNC:21111]	-1.7	-2
3861	*CYB561A3*	220002	cytochrome b561 family member A3 [Source:HGNC Symbol;Acc:HGNC:23014]	-1.7	-2
3862	*MZT2A*	653784	mitotic spindle organizing protein 2A [Source:HGNC Symbol;Acc:HGNC:33187]	-1.7	-2
3863	*LDB1*	8861	LIM domain binding 1 [Source:HGNC Symbol;Acc:HGNC:6532]	-1.7	-2
3864	*TMEM39B*	55116	transmembrane protein 39B [Source:HGNC Symbol;Acc:HGNC:25510]	-1.7	-2
3865	*COMMD6*	170622	COMM domain containing 6 [Source:HGNC Symbol;Acc:HGNC:24015]	-1.7	-2
3866	*WDR6*	11180	WD repeat domain 6 [Source:HGNC Symbol;Acc:HGNC:12758]	-1.7	-2
3867	*MDH1*	4190	malate dehydrogenase 1 [Source:HGNC Symbol;Acc:HGNC:6970]	-1.7	-2

3868	*PMS2*	5395	PMS1 homolog 2, mismatch repair system component [Source:HGNC Symbol;Acc:HGNC:9122]	-1.7	-2
3869	*GLS*	2744	glutaminase [Source:HGNC Symbol;Acc:HGNC:4331]	-1.7	-2
3870	*ARMC3*	219681	armadillo repeat containing 3 [Source:HGNC Symbol;Acc:HGNC:30964]	-1.7	-2
3871	*MAN2A1*	4124	mannosidase alpha class 2A member 1 [Source:HGNC Symbol;Acc:HGNC:6824]	-1.7	-2
3872	*INSIG2*	51141	insulin induced gene 2 [Source:HGNC Symbol;Acc:HGNC:20452]	-1.7	-2
3873	*GABRA4*	2557	gamma-aminobutyric acid type A receptor alpha4 subunit [Source:HGNC Symbol;Acc:HGNC:4078]	-1.7	-2
3874	*HSPA4*	3308	heat shock protein family A (Hsp70) member 4 [Source:HGNC Symbol;Acc:HGNC:5237]	-1.7	-2
3875	*U2SURP*	23350	U2 snRNP associated SURP domain containing [Source:HGNC Symbol;Acc:HGNC:30855]	-1.7	-2
3876	*PDE8A*	5151	phosphodiesterase 8A [Source:HGNC Symbol;Acc:HGNC:8793]	-1.7	-2
3877	*CNR2*	1269	cannabinoid receptor 2 [Source:HGNC Symbol;Acc:HGNC:2160]	-1.7	-2
3878	*NOP58*	51602	NOP58 ribonucleoprotein [Source:HGNC Symbol;Acc:HGNC:29926]	-1.7	-2
3879	*SEC31A*	22872	SEC31 homolog A, COPII coat complex component [Source:HGNC Symbol;Acc:HGNC:17052]	-1.7	-2
3880	*ACTR6*	64431	ARP6 actin-related protein 6 homolog [Source:HGNC Symbol;Acc:HGNC:24025]	-1.7	-2
3881	*USP11*	8237	ubiquitin specific peptidase 11 [Source:HGNC Symbol;Acc:HGNC:12609]	-1.7	-2
3882	*ASH1L*	55870	ASH1 like histone lysine methyltransferase [Source:HGNC Symbol;Acc:HGNC:19088]	-1.7	-2
3883	*C1R*	715	complement C1r subcomponent [Source:HGNC Symbol;Acc:HGNC:1246]	-1.7	-2
3884	*EBAG9*	9166	estrogen receptor binding site associated, antigen, 9 [Source:HGNC Symbol;Acc:HGNC:3123]	-1.7	-2
3885	*EIF2A*	83939	eukaryotic translation initiation factor 2A [Source:HGNC Symbol;Acc:HGNC:3254]	-1.7	-2
3886	*CSNK1E*	1454	casein kinase 1 epsilon [Source:HGNC Symbol;Acc:HGNC:2453]	-1.7	-2
3887	*ZNHIT1*	10467	zinc finger HIT-type containing 1 [Source:HGNC Symbol;Acc:HGNC:21688]	-1.7	-2
3888	*LSM7*	51690	LSM7 homolog, U6 small nuclear RNA and mRNA degradation associated [Source:HGNC Symbol;Acc:HGNC:20470]	-1.7	-2
3889	*TMED9*	54732	transmembrane p24 trafficking protein 9 [Source:HGNC Symbol;Acc:HGNC:24878]	-1.7	-2
3890	*ZNF462*	58499	zinc finger protein 462 [Source:HGNC Symbol;Acc:HGNC:21684]	-1.7	-2
3891	*AGA*	175	aspartylglucosaminidase [Source:HGNC Symbol;Acc:HGNC:318]	-1.7	-2
3892	*SPRTN*	83932	SprT-like N-terminal domain [Source:HGNC Symbol;Acc:HGNC:25356]	-1.7	-2
3893	*EIF3D*	8664	eukaryotic translation initiation factor 3 subunit D [Source:HGNC Symbol;Acc:HGNC:3278]	-1.7	-2
3894	*HSPA5*	3309	heat shock protein family A (Hsp70) member 5 [Source:HGNC Symbol;Acc:HGNC:5238]	-1.7	-2
3895	*AP2B1*	163	adaptor related protein complex 2 beta 1 subunit [Source:HGNC Symbol;Acc:HGNC:563]	-1.7	-2
3896	*DNHD1*	144132	dynein heavy chain domain 1 [Source:HGNC Symbol;Acc:HGNC:26532]	-1.7	-2
3897	*HAT1*	8520	histone acetyltransferase 1 [Source:HGNC Symbol;Acc:HGNC:4821]	-1.7	-2
3898	*TPI1*	7167	triosephosphate isomerase 1 [Source:HGNC Symbol;Acc:HGNC:12009]	-1.7	-2
3899	*ARL6IP4*	51329	ADP ribosylation factor like GTPase 6 interacting protein 4 [Source:HGNC Symbol;Acc:HGNC:18076]	-1.7	-2
3900	*CAAP1*	79886	caspase activity and apoptosis inhibitor 1 [Source:HGNC Symbol;Acc:HGNC:25834]	-1.7	-2
3901	*FAM222B*	55731	family with sequence similarity 222 member B [Source:HGNC Symbol;Acc:HGNC:25563]	-1.7	-2
3902	*PAK2*	5062	p21 (RAC1) activated kinase 2 [Source:HGNC Symbol;Acc:HGNC:8591]	-1.7	-2
3903	*RPS26*	6231	ribosomal protein S26 [Source:HGNC Symbol;Acc:HGNC:10414]	-1.7	-2
3904	*MBNL2*	10150	muscleblind like splicing regulator 2 [Source:HGNC Symbol;Acc:HGNC:16746]	-1.7	-2
3905	*CRISPLD2*	83716	cysteine rich secretory protein LCCL domain containing 2 [Source:HGNC Symbol;Acc:HGNC:25248]	-1.7	-2
3906	*MICB*	4277	MHC class I polypeptide-related sequence B [Source:HGNC Symbol;Acc:HGNC:7091]	-1.7	-2
3907	*LAT*	27040	linker for activation of T-cells [Source:HGNC Symbol;Acc:HGNC:18874]	-1.7	-2
3908	*SGK1*	6446	serum/glucocorticoid regulated kinase 1 [Source:HGNC Symbol;Acc:HGNC:10810]	-1.7	-2
3909	*PELO*	53918	pelota homolog (Drosophila) [Source:HGNC Symbol;Acc:HGNC:8829]	-1.7	-2
3910	*IL15RA*	3601	interleukin 15 receptor subunit alpha [Source:HGNC Symbol;Acc:HGNC:5978]	-1.7	-2
3911	*ZNF600*	162966	zinc finger protein 600 [Source:HGNC Symbol;Acc:HGNC:30951]	-1.7	-2
3912	*C17orf51*	339263	chromosome 17 open reading frame 51 [Source:HGNC Symbol;Acc:HGNC:27904]	-1.7	-2
3913	*ZBTB1*	22890	zinc finger and BTB domain containing 1 [Source:HGNC Symbol;Acc:HGNC:20259]	-1.7	-2
3914	*BACE1*	23621	beta-secretase 1 [Source:HGNC Symbol;Acc:HGNC:933]	-1.7	-2
3915	*RNF214*	257160	ring finger protein 214 [Source:HGNC Symbol;Acc:HGNC:25335]	-1.7	-2
3916	*CTSO*	1519	cathepsin O [Source:HGNC Symbol;Acc:HGNC:2542]	-1.7	-2
3917	*FAM160B1*	57700	family with sequence similarity 160 member B1 [Source:HGNC Symbol;Acc:HGNC:29320]	-1.7	-2
3918	*DUT*	1854	deoxyuridine triphosphatase [Source:HGNC Symbol;Acc:HGNC:3078]	-1.7	-2
3919	*INPP1*	3628	inositol polyphosphate-1-phosphatase [Source:HGNC Symbol;Acc:HGNC:6071]	-1.7	-2
3920	*TOB1*	10140	transducer of ERBB2, 1 [Source:HGNC Symbol;Acc:HGNC:11979]	-1.7	-2
3921	*CARD9*	64170	caspase recruitment domain family member 9 [Source:HGNC Symbol;Acc:HGNC:16391]	-1.7	-2
3922	*RAB34*	83871	RAB34, member RAS oncogene family [Source:HGNC Symbol;Acc:HGNC:16519]	-1.7	-2
3923	*COQ3*	51805	coenzyme Q3, methyltransferase [Source:HGNC Symbol;Acc:HGNC:18175]	-1.7	-2
3924	*MORC3*	23515	MORC family CW-type zinc finger 3 [Source:HGNC Symbol;Acc:HGNC:23572]	-1.7	-2
3925	*TXNIP*	10628	thioredoxin interacting protein [Source:HGNC Symbol;Acc:HGNC:16952]	-1.7	-2
3926	*HSP90AB1*	3326	heat shock protein 90kDa alpha family class B member 1 [Source:HGNC Symbol;Acc:HGNC:5258]	-1.7	-2
3927	*GABARAP*	11337	GABA type A receptor-associated protein [Source:HGNC Symbol;Acc:HGNC:4067]	-1.7	-2
3928	*AHI1*	54806	Abelson helper integration site 1 [Source:HGNC Symbol;Acc:HGNC:21575]	-1.7	-2
3929	*RBM38*	55544	RNA binding motif protein 38 [Source:HGNC Symbol;Acc:HGNC:15818]	-1.7	-2
3930	*ASRGL1*	80150	asparaginase like 1 [Source:HGNC Symbol;Acc:HGNC:16448]	-1.7	-2
3931	*CFAP43*	80217	cilia and flagella associated protein 43 [Source:HGNC Symbol;Acc:HGNC:26684]	-1.7	-2
3932	*PHB*	5245	prohibitin [Source:HGNC Symbol;Acc:HGNC:8912]	-1.7	-2
3933	*MTIF2*	4528	mitochondrial translational initiation factor 2 [Source:HGNC Symbol;Acc:HGNC:7441]	-1.7	-2
3934	*IGSF9B*	22997	immunoglobulin superfamily member 9B [Source:HGNC Symbol;Acc:HGNC:32326]	-1.7	-2
3935	*APOBEC3G*	60489	apolipoprotein B mRNA editing enzyme catalytic subunit 3G [Source:HGNC Symbol;Acc:HGNC:17357]	-1.7	-2
3936	*USP10*	9100	ubiquitin specific peptidase 10 [Source:HGNC Symbol;Acc:HGNC:12608]	-1.7	-2
3937	*RPS28*	6234	ribosomal protein S28 [Source:HGNC Symbol;Acc:HGNC:10418]	-1.7	-2
3938	*ZDHHC2*	51201	zinc finger DHHC-type containing 2 [Source:HGNC Symbol;Acc:HGNC:18469]	-1.7	-2
3939	*PRKCE*	5581	protein kinase C epsilon [Source:HGNC Symbol;Acc:HGNC:9401]	-1.7	-2
3940	*MYOM1*	8736	myomesin 1 [Source:HGNC Symbol;Acc:HGNC:7613]	-1.7	-2
3941	*RANBP2*	5903	RAN binding protein 2 [Source:HGNC Symbol;Acc:HGNC:9848]	-1.7	-2

3942	*MAPKAPK2*	9261	mitogen-activated protein kinase-activated protein kinase 2 [Source:HGNC Symbol;Acc:HGNC:6887]	-1.7	-2
3943	*CTSL*	1514	cathepsin L [Source:HGNC Symbol;Acc:HGNC:2537]	-1.7	-2
3944	*DYNLL1*	8655	dynein light chain LC8-type 1 [Source:HGNC Symbol;Acc:HGNC:15476]	-1.7	-2
3945	*RD3*	343035	retinal degeneration 3 [Source:HGNC Symbol;Acc:HGNC:19689]	-1.7	-2
3946	*KLHL7*	55975	kelch like family member 7 [Source:HGNC Symbol;Acc:HGNC:15646]	-1.7	-2
3947	*SRI*	6717	sorcin [Source:HGNC Symbol;Acc:HGNC:11292]	-1.7	-2
3948	*STRBP*	55342	spermatid perinuclear RNA binding protein [Source:HGNC Symbol;Acc:HGNC:16462]	-1.7	-2
3949	*CSNK1G2*	1455	casein kinase 1 gamma 2 [Source:HGNC Symbol;Acc:HGNC:2455]	-1.7	-2
3950	*OR5P3*	120066	olfactory receptor family 5 subfamily P member 3 [Source:HGNC Symbol;Acc:HGNC:14784]	-1.7	-2
3951	*CD69*	969	CD69 molecule [Source:HGNC Symbol;Acc:HGNC:1694]	-1.7	-2
3952	*ATP6V1D*	51382	ATPase H+ transporting V1 subunit D [Source:HGNC Symbol;Acc:HGNC:13527]	-1.7	-2
3953	*CTBP2*	1488	C-terminal binding protein 2 [Source:HGNC Symbol;Acc:HGNC:2495]	-1.7	-2
3954	*AK3*	50808	adenylate kinase 3 [Source:HGNC Symbol;Acc:HGNC:17376]	-1.7	-2
3955	*ZNF470*	388566	zinc finger protein 470 [Source:HGNC Symbol;Acc:HGNC:22220]	-1.7	-2
3956	*RPL35A*	6165	ribosomal protein L35a [Source:HGNC Symbol;Acc:HGNC:10345]	-1.7	-2
3957	*ADAM9*	8754	ADAM metallopeptidase domain 9 [Source:HGNC Symbol;Acc:HGNC:216]	-1.7	-2
3958	*XIAP*	331	X-linked inhibitor of apoptosis [Source:HGNC Symbol;Acc:HGNC:592]	-1.7	-2
3959	*FUT9*	10690	fucosyltransferase 9 [Source:HGNC Symbol;Acc:HGNC:4020]	-1.7	-2
3960	*PLPP1*	8611	phospholipid phosphatase 1 [Source:HGNC Symbol;Acc:HGNC:9228]	-1.7	-2
3961	*FADS1*	3992	fatty acid desaturase 1 [Source:HGNC Symbol;Acc:HGNC:3574]	-1.7	-2
3962	*RPS7*	6201	ribosomal protein S7 [Source:HGNC Symbol;Acc:HGNC:10440]	-1.7	-2
3963	*TCF7*	6932	transcription factor 7 (T-cell specific, HMG-box) [Source:HGNC Symbol;Acc:HGNC:11639]	-1.7	-2
3964	*GOLGA6A*	342096	golgin A6 family member A [Source:HGNC Symbol;Acc:HGNC:13567]	-1.7	-2
3965	*GLTSCR2*	29997	glioma tumor suppressor candidate region gene 2 [Source:HGNC Symbol;Acc:HGNC:4333]	-1.7	-2
3966	*FAM13A*	10144	family with sequence similarity 13 member A [Source:HGNC Symbol;Acc:HGNC:19367]	-1.7	-2
3967	*TBC1D15*	64786	TBC1 domain family member 15 [Source:HGNC Symbol;Acc:HGNC:25694]	-1.7	-2
3968	*ATP11C*	286410	ATPase phospholipid transporting 11C [Source:HGNC Symbol;Acc:HGNC:13554]	-1.7	-2
3969	*SORD*	6652	sorbitol dehydrogenase [Source:HGNC Symbol;Acc:HGNC:11184]	-1.7	-2
3970	*SMC1A*	8243	structural maintenance of chromosomes 1A [Source:HGNC Symbol;Acc:HGNC:11111]	-1.7	-2
3971	*MAGIX*	79917	MAGI family member, X-linked [Source:HGNC Symbol;Acc:HGNC:30006]	-1.7	-2
3972	*RPL36AL*	6166	ribosomal protein L36a like [Source:HGNC Symbol;Acc:HGNC:10346]	-1.7	-2
3973	*RPS21*	6227	ribosomal protein S21 [Source:HGNC Symbol;Acc:HGNC:10409]	-1.7	-2
3974	*TNPO1*	3842	transportin 1 [Source:HGNC Symbol;Acc:HGNC:6401]	-1.7	-2
3975	*SLC44A1*	23446	solute carrier family 44 member 1 [Source:HGNC Symbol;Acc:HGNC:18798]	-1.7	-2
3976	*FAM58A*	92002	family with sequence similarity 58 member A [Source:HGNC Symbol;Acc:HGNC:28434]	-1.7	-2
3977	*DDX47*	51202	DEAD-box helicase 47 [Source:HGNC Symbol;Acc:HGNC:18682]	-1.7	-2
3978	*FAM102A*	399665	family with sequence similarity 102 member A [Source:HGNC Symbol;Acc:HGNC:31419]	-1.7	-2
3979	*SUCO*	51430	SUN domain containing ossification factor [Source:HGNC Symbol;Acc:HGNC:1240]	-1.7	-2
3980	*C17orf49*	124944	chromosome 17 open reading frame 49 [Source:HGNC Symbol;Acc:HGNC:28737]	-1.7	-2
3981	*RNASEK*	440400	ribonuclease K [Source:HGNC Symbol;Acc:HGNC:33911]	-1.7	-2
3982	*PSEN2*	5664	presenilin 2 [Source:HGNC Symbol;Acc:HGNC:9509]	-1.7	-2
3983	*DEFB123*	245936	defensin beta 123 [Source:HGNC Symbol;Acc:HGNC:18103]	-1.7	-2
3984	*P2RX4*	5025	purinergic receptor P2X 4 [Source:HGNC Symbol;Acc:HGNC:8535]	-1.7	-2
3985	*CDK8*	1024	cyclin dependent kinase 8 [Source:HGNC Symbol;Acc:HGNC:1779]	-1.7	-2
3986	*DHX15*	1665	DEAH-box helicase 15 [Source:HGNC Symbol;Acc:HGNC:2738]	-1.7	-2
3987	*NDUFC2*	4718	NADH:ubiquinone oxidoreductase subunit C2 [Source:HGNC Symbol;Acc:HGNC:7706]	-1.7	-2
3988	*PABPC4*	8761	poly(A) binding protein cytoplasmic 4 [Source:HGNC Symbol;Acc:HGNC:8557]	-1.7	-2
3989	*CSMD1*	64478	CUB and Sushi multiple domains 1 [Source:HGNC Symbol;Acc:HGNC:14026]	-1.7	-2
3990	*ICA1*	3382	islet cell autoantigen 1 [Source:HGNC Symbol;Acc:HGNC:5343]	-1.7	-2
3991	*KANK1*	23189	KN motif and ankyrin repeat domains 1 [Source:HGNC Symbol;Acc:HGNC:19309]	-1.7	-2
3992	*SMG8*	55181	SMG8, nonsense mediated mRNA decay factor [Source:HGNC Symbol;Acc:HGNC:25551]	-1.7	-2
3993	*UNC93B1*	81622	unc-93 homolog B1 (C. elegans) [Source:HGNC Symbol;Acc:HGNC:13481]	-1.7	-2
3994	*NSMCE1*	197370	NSE1 homolog, SMC5-SMC6 complex component [Source:HGNC Symbol;Acc:HGNC:29897]	-1.7	-2
3995	*PSMB10*	5699	proteasome subunit beta 10 [Source:HGNC Symbol;Acc:HGNC:9538]	-1.7	-2
3996	*MIB1*	57534	mindbomb E3 ubiquitin protein ligase 1 [Source:HGNC Symbol;Acc:HGNC:21086]	-1.7	-2
3997	*MBD2*	8932	methyl-CpG binding domain protein 2 [Source:HGNC Symbol;Acc:HGNC:6917]	-1.7	-2
3998	*SSR2*	6746	signal sequence receptor subunit 2 [Source:HGNC Symbol;Acc:HGNC:11324]	-1.7	-2
3999	*RPL31*	6160	ribosomal protein L31 [Source:HGNC Symbol;Acc:HGNC:10334]	-1.7	-2
4000	*LMBRD2*	92255	LMBR1 domain containing 2 [Source:HGNC Symbol;Acc:HGNC:25287]	-1.7	-2
4001	*CEBPA*	1050	CCAAT/enhancer binding protein alpha [Source:HGNC Symbol;Acc:HGNC:1833]	-1.7	-2
4002	*SERBP1*	26135	SERPINE1 mRNA binding protein 1 [Source:HGNC Symbol;Acc:HGNC:17860]	-1.7	-2
4003	*TMCC1*	23023	transmembrane and coiled-coil domain family 1 [Source:HGNC Symbol;Acc:HGNC:29116]	-1.7	-2
4004	*PDCD6*	10016	programmed cell death 6 [Source:HGNC Symbol;Acc:HGNC:8765]	-1.7	-2
4005	*GID8*	54994	GID complex subunit 8 homolog [Source:HGNC Symbol;Acc:HGNC:15857]	-1.7	-2
4006	*TM9SF2*	9375	transmembrane 9 superfamily member 2 [Source:HGNC Symbol;Acc:HGNC:11865]	-1.7	-2
4007	*NT5DC3*	51559	5'-nucleotidase domain containing 3 [Source:HGNC Symbol;Acc:HGNC:30826]	-1.7	-2
4008	*MED1*	5469	mediator complex subunit 1 [Source:HGNC Symbol;Acc:HGNC:9234]	-1.7	-2
4009	*ATP2A2*	488	ATPase sarcoplasmic/endoplasmic reticulum Ca2+ transporting 2 [Source:HGNC Symbol;Acc:HGNC:812]	-1.7	-2
4010	*SF3B1*	23451	splicing factor 3b subunit 1 [Source:HGNC Symbol;Acc:HGNC:10768]	-1.7	-2
4011	*FAM35A*	54537	family with sequence similarity 35 member A [Source:HGNC Symbol;Acc:HGNC:28773]	-1.7	-2
4012	*SAMM50*	25813	SAMM50 sorting and assembly machinery component [Source:HGNC Symbol;Acc:HGNC:24276]	-1.7	-2
4013	*TEX14*	56155	testis expressed 14, intercellular bridge forming factor [Source:HGNC Symbol;Acc:HGNC:11737]	-1.7	-2
4014	*TBCD*	6904	tubulin folding cofactor D [Source:HGNC Symbol;Acc:HGNC:11581]	-1.7	-2
4015	*RPL37*	6167	ribosomal protein L37 [Source:HGNC Symbol;Acc:HGNC:10347]	-1.7	-2
4016	*MRPS26*	64949	mitochondrial ribosomal protein S26 [Source:HGNC Symbol;Acc:HGNC:14045]	-1.7	-2
4017	*HRH4*	59340	histamine receptor H4 [Source:HGNC Symbol;Acc:HGNC:17383]	-1.7	-2
4018	*ZNF461*	92283	zinc finger protein 461 [Source:HGNC Symbol;Acc:HGNC:21629]	-1.7	-2

4019	*NME1*	4830	NME/NM23 nucleoside diphosphate kinase 1 [Source:HGNC Symbol;Acc:HGNC:7849]	-1.7	-2
4020	*SDF2*	6388	stromal cell derived factor 2 [Source:HGNC Symbol;Acc:HGNC:10675]	-1.7	-2
4021	*NID1*	4811	nidogen 1 [Source:HGNC Symbol;Acc:HGNC:7821]	-1.7	-2
4022	*RCOR1*	23186	REST corepressor 1 [Source:HGNC Symbol;Acc:HGNC:17441]	-1.7	-2
4023	*ZFP90*	146198	ZFP90 zinc finger protein [Source:HGNC Symbol;Acc:HGNC:23329]	-1.7	-2
4024	*APOC1*	341	apolipoprotein C1 [Source:HGNC Symbol;Acc:HGNC:607]	-1.7	-2
4025	*LRP1B*	53353	LDL receptor related protein 1B [Source:HGNC Symbol;Acc:HGNC:6693]	-1.7	-2
4026	*WDFY3*	23001	WD repeat and FYVE domain containing 3 [Source:HGNC Symbol;Acc:HGNC:20751]	-1.7	-2
4027	*HTATSF1*	27336	HIV-1 Tat specific factor 1 [Source:HGNC Symbol;Acc:HGNC:5276]	-1.7	-2
4028	*RPL3*	6122	ribosomal protein L3 [Source:HGNC Symbol;Acc:HGNC:10332]	-1.7	-2
4029	*MAP3K13*	9175	mitogen-activated protein kinase kinase kinase 13 [Source:HGNC Symbol;Acc:HGNC:6852]	-1.7	-2
4030	*ALDH3A1*	218	aldehyde dehydrogenase 3 family member A1 [Source:HGNC Symbol;Acc:HGNC:405]	-1.7	-2
4031	*ZNF460*	10794	zinc finger protein 460 [Source:HGNC Symbol;Acc:HGNC:21628]	-1.7	-2
4032	*TMEM126A*	84233	transmembrane protein 126A [Source:HGNC Symbol;Acc:HGNC:25382]	-1.7	-2
4033	*RPS5*	6193	ribosomal protein S5 [Source:HGNC Symbol;Acc:HGNC:10426]	-1.7	-2
4034	*PHC3*	80012	polyhomeotic homolog 3 [Source:HGNC Symbol;Acc:HGNC:15682]	-1.7	-2
4035	*FAM135A*	57579	family with sequence similarity 135 member A [Source:HGNC Symbol;Acc:HGNC:21084]	-1.7	-2
4036	*GPM6A*	2823	glycoprotein M6A [Source:HGNC Symbol;Acc:HGNC:4460]	-1.7	-2
4037	*EPRS*	2058	glutamyl-prolyl-tRNA synthetase [Source:HGNC Symbol;Acc:HGNC:3418]	-1.7	-2
4038	*PRDX3*	10935	peroxiredoxin 3 [Source:HGNC Symbol;Acc:HGNC:9354]	-1.7	-2
4039	*PSMC6*	5706	proteasome 26S subunit, ATPase 6 [Source:HGNC Symbol;Acc:HGNC:9553]	-1.7	-2
4040	*ANTXR2*	118429	anthrax toxin receptor 2 [Source:HGNC Symbol;Acc:HGNC:21732]	-1.7	-2
4041	*CREB3L2*	64764	cAMP responsive element binding protein 3 like 2 [Source:HGNC Symbol;Acc:HGNC:23720]	-1.7	-2
4042	*MROH8*	140699	maestro heat like repeat family member 8 [Source:HGNC Symbol;Acc:HGNC:16125]	-1.7	-2
4043	*FOSL2*	2355	FOS like 2, AP-1 transcription factor subunit [Source:HGNC Symbol;Acc:HGNC:3798]	-1.7	-2
4044	*PAIP2B*	400961	poly(A) binding protein interacting protein 2B [Source:HGNC Symbol;Acc:HGNC:29200]	-1.7	-2
4045	*TMEM9*	252839	transmembrane protein 9 [Source:HGNC Symbol;Acc:HGNC:18823]	-1.7	-2
4046	*YIF1A*	10897	Yip1 interacting factor homolog A, membrane trafficking protein [Source:HGNC Symbol;Acc:HGNC:16688]	-1.7	-2
4047	*TFAP2A*	7020	transcription factor AP-2 alpha [Source:HGNC Symbol;Acc:HGNC:11742]	-1.7	-2
4048	*MPDU1*	9526	mannose-P-dolichol utilization defect 1 [Source:HGNC Symbol;Acc:HGNC:7207]	-1.7	-2
4049	*THNSL1*	79896	threonine synthase like 1 [Source:HGNC Symbol;Acc:HGNC:26160]	-1.7	-2
4050	*SLITRK2*	84631	SLIT and NTRK like family member 2 [Source:HGNC Symbol;Acc:HGNC:13449]	-1.7	-2
4051	*ATG14*	22863	autophagy related 14 [Source:HGNC Symbol;Acc:HGNC:19962]	-1.7	-2
4052	*ZNF22*	7570	zinc finger protein 22 [Source:HGNC Symbol;Acc:HGNC:13012]	-1.7	-2
4053	*GAPT*	202309	GRB2-binding adaptor protein, transmembrane [Source:HGNC Symbol;Acc:HGNC:26588]	-1.7	-2
4054	*ISOC2*	79763	isochorismatase domain containing 2 [Source:HGNC Symbol;Acc:HGNC:26278]	-1.7	-2
4055	*NOP14*	8602	NOP14 nucleolar protein [Source:HGNC Symbol;Acc:HGNC:16821]	-1.7	-2
4056	*ETNK2*	55224	ethanolamine kinase 2 [Source:HGNC Symbol;Acc:HGNC:25575]	-1.7	-2
4057	*SIGMAR1*	10280	sigma non-opioid intracellular receptor 1 [Source:HGNC Symbol;Acc:HGNC:8157]	-1.7	-2
4058	*PLSCR1*	5359	phospholipid scramblase 1 [Source:HGNC Symbol;Acc:HGNC:9092]	-1.7	-2
4059	*ZBTB33*	10009	zinc finger and BTB domain containing 33 [Source:HGNC Symbol;Acc:HGNC:16682]	-1.7	-2
4060	*RPL29*	6159	ribosomal protein L29 [Source:HGNC Symbol;Acc:HGNC:10331]	-1.7	-2
4061	*PKN1*	5585	protein kinase N1 [Source:HGNC Symbol;Acc:HGNC:9405]	-1.7	-2
4062	*RSBN1*	54665	round spermatid basic protein 1 [Source:HGNC Symbol;Acc:HGNC:25642]	-1.7	-2
4063	*PER2*	8864	period circadian clock 2 [Source:HGNC Symbol;Acc:HGNC:8846]	-1.7	-2
4064	*RCAN1*	1827	regulator of calcineurin 1 [Source:HGNC Symbol;Acc:HGNC:3040]	-1.7	-2
4065	*HNRNPC*	3183	heterogeneous nuclear ribonucleoprotein C (C1/C2) [Source:HGNC Symbol;Acc:HGNC:5035]	-1.7	-2
4066	*UPF3A*	65110	UPF3 regulator of nonsense transcripts homolog A (yeast) [Source:HGNC Symbol;Acc:HGNC:20332]	-1.7	-2
4067	*UQCRFS1*	7386	ubiquinol-cytochrome c reductase, Rieske iron-sulfur polypeptide 1 [Source:HGNC Symbol;Acc:HGNC:12587]	-1.7	-2
4068	*F2RL1*	2150	F2R like trypsin receptor 1 [Source:HGNC Symbol;Acc:HGNC:3538]	-1.7	-2
4069	*DAZAP2*	9802	DAZ associated protein 2 [Source:HGNC Symbol;Acc:HGNC:2684]	-1.7	-2
4070	*IFI16*	3428	interferon gamma inducible protein 16 [Source:HGNC Symbol;Acc:HGNC:5395]	-1.7	-2
4071	*MDFIC*	29969	MyoD family inhibitor domain containing [Source:HGNC Symbol;Acc:HGNC:28870]	-1.7	-2
4072	*ATP1B3*	483	ATPase Na+/K+ transporting subunit beta 3 [Source:HGNC Symbol;Acc:HGNC:806]	-1.7	-2
4073	*GLI2*	2736	GLI family zinc finger 2 [Source:HGNC Symbol;Acc:HGNC:4318]	-1.7	-2
4074	*EBF1*	1879	early B-cell factor 1 [Source:HGNC Symbol;Acc:HGNC:3126]	-1.7	-2
4075	*PPP1R13L*	10848	protein phosphatase 1 regulatory subunit 13 like [Source:HGNC Symbol;Acc:HGNC:18838]	-1.7	-2
4076	*HNRNPA1L2*	144983	heterogeneous nuclear ribonucleoprotein A1-like 2 [Source:HGNC Symbol;Acc:HGNC:27067]	-1.7	-2
4077	*NDUFA10*	4705	NADH:ubiquinone oxidoreductase subunit A10 [Source:HGNC Symbol;Acc:HGNC:7684]	-1.7	-2
4078	*SOD1*	6647	superoxide dismutase 1, soluble [Source:HGNC Symbol;Acc:HGNC:11179]	-1.7	-2
4079	*BCORL1*	63035	BCL6 corepressor-like 1 [Source:HGNC Symbol;Acc:HGNC:25657]	-1.7	-2
4080	*FARP1*	10160	FERM, ARH/RhoGEF and pleckstrin domain protein 1 [Source:HGNC Symbol;Acc:HGNC:3591]	-1.7	-2
4081	*B2M*	567	beta-2-microglobulin [Source:HGNC Symbol;Acc:HGNC:914]	-1.7	-2
4082	*HTR2B*	3357	5-hydroxytryptamine receptor 2B [Source:HGNC Symbol;Acc:HGNC:5294]	-1.7	-2
4083	*PIGY*	84992	phosphatidylinositol glycan anchor biosynthesis class Y [Source:HGNC Symbol;Acc:HGNC:28213]	-1.7	-2
4084	*ZFP62*	643836	ZFP62 zinc finger protein [Source:HGNC Symbol;Acc:HGNC:23241]	-1.7	-2
4085	*UBR5*	51366	ubiquitin protein ligase E3 component n-recognin 5 [Source:HGNC Symbol;Acc:HGNC:16806]	-1.7	-2
4086	*TVP23B*	51030	trans-golgi network vesicle protein 23 homolog B (S. cerevisiae) [Source:HGNC Symbol;Acc:HGNC:20399]	-1.7	-2
4087	*EIF4A1*	1973	eukaryotic translation initiation factor 4A1 [Source:HGNC Symbol;Acc:HGNC:3282]	-1.7	-2
4088	*CCNDBP1*	23582	cyclin D1 binding protein 1 [Source:HGNC Symbol;Acc:HGNC:1587]	-1.7	-2
4089	*ATP6V1G1*	9550	ATPase H+ transporting V1 subunit G1 [Source:HGNC Symbol;Acc:HGNC:864]	-1.7	-2
4090	*VPS13D*	55187	vacuolar protein sorting 13 homolog D [Source:HGNC Symbol;Acc:HGNC:23595]	-1.7	-2

4091	*BTN2A1*	11120	butyrophilin subfamily 2 member A1 [Source:HGNC Symbol;Acc:HGNC:1136]	-1.7	-2
4092	*ZNF655*	79027	zinc finger protein 655 [Source:HGNC Symbol;Acc:HGNC:30899]	-1.7	-2
4093	*CPNE6*	9362	copine 6 [Source:HGNC Symbol;Acc:HGNC:2319]	-1.7	-2
4094	*ZNF416*	55659	zinc finger protein 416 [Source:HGNC Symbol;Acc:HGNC:20645]	-1.7	-2
4095	*SHE*	126669	Src homology 2 domain containing E [Source:HGNC Symbol;Acc:HGNC:27004]	-1.7	-2
4096	*CDR1*	1038	cerebellar degeneration related protein 1 [Source:HGNC Symbol;Acc:HGNC:1798]	-1.7	-2
4097	*RPS19*	6223	ribosomal protein S19 [Source:HGNC Symbol;Acc:HGNC:10402]	-1.7	-2
4098	*MED4*	29079	mediator complex subunit 4 [Source:HGNC Symbol;Acc:HGNC:17903]	-1.7	-2
4099	*CHMP3*	51652	charged multivesicular body protein 3 [Source:HGNC Symbol;Acc:HGNC:29865]	-1.7	-2
4100	*DDT*	1652	D-dopachrome tautomerase [Source:HGNC Symbol;Acc:HGNC:2732]	-1.7	-2
4101	*UCHL3*	7347	ubiquitin C-terminal hydrolase L3 [Source:HGNC Symbol;Acc:HGNC:12515]	-1.7	-2
4102	*DRP2*	1821	dystrophin related protein 2 [Source:HGNC Symbol;Acc:HGNC:3032]	-1.7	-2
4103	*PURA*	5813	purine rich element binding protein A [Source:HGNC Symbol;Acc:HGNC:9701]	-1.8	-2
4104	*IDE*	3416	insulin degrading enzyme [Source:HGNC Symbol;Acc:HGNC:5381]	-1.8	-2
4105	*ZHX2*	22882	zinc fingers and homeoboxes 2 [Source:HGNC Symbol;Acc:HGNC:18513]	-1.8	-2
4106	*PPP2R5C*	5527	protein phosphatase 2 regulatory subunit B'gamma [Source:HGNC Symbol;Acc:HGNC:9311]	-1.8	-2
4107	*C11orf31*	280636	chromosome 11 open reading frame 31 [Source:HGNC Symbol;Acc:HGNC:18251]	-1.8	-2
4108	*TMEM127*	55654	transmembrane protein 127 [Source:HGNC Symbol;Acc:HGNC:26038]	-1.8	-2
4109	*NME2*	4831	NME/NM23 nucleoside diphosphate kinase 2 [Source:HGNC Symbol;Acc:HGNC:7850]	-1.8	-2
4110	*TBC1D4*	9882	TBC1 domain family member 4 [Source:HGNC Symbol;Acc:HGNC:19165]	-1.8	-2
4111	*NUP62*	23636	nucleoporin 62 [Source:HGNC Symbol;Acc:HGNC:8066]	-1.8	-2
4112	*TM9SF3*	56889	transmembrane 9 superfamily member 3 [Source:HGNC Symbol;Acc:HGNC:21529]	-1.8	-2
4113	*ZNF131*	7690	zinc finger protein 131 [Source:HGNC Symbol;Acc:HGNC:12915]	-1.8	-2
4114	*COPB2*	9276	coatomer protein complex subunit beta 2 [Source:HGNC Symbol;Acc:HGNC:2232]	-1.8	-2
4115	*SMARCD2*	6603	SWI/SNF related, matrix associated, actin dependent regulator of chromatin, subfamily d, member 2 [Source:HGNC Symbol;Acc:HGNC:11107]	-1.8	-2
4116	*KEAP1*	9817	kelch like ECH associated protein 1 [Source:HGNC Symbol;Acc:HGNC:23177]	-1.8	-2
4117	*SERTAD1*	29950	SERTA domain containing 1 [Source:HGNC Symbol;Acc:HGNC:17932]	-1.8	-2
4118	*FAM3C*	10447	family with sequence similarity 3 member C [Source:HGNC Symbol;Acc:HGNC:18664]	-1.8	-2
4119	*GSPT1*	2935	G1 to S phase transition 1 [Source:HGNC Symbol;Acc:HGNC:4621]	-1.8	-2
4120	*BANP*	54971	BTG3 associated nuclear protein [Source:HGNC Symbol;Acc:HGNC:13450]	-1.8	-2
4121	*DHRS1*	115817	dehydrogenase/reductase 1 [Source:HGNC Symbol;Acc:HGNC:16445]	-1.8	-2
4122	*COQ9*	57017	coenzyme Q9 [Source:HGNC Symbol;Acc:HGNC:25302]	-1.8	-2
4123	*LYNX1*	66004	Ly6/neurotoxin 1 [Source:HGNC Symbol;Acc:HGNC:29604]	-1.8	-2
4124	*TRIP4*	9325	thyroid hormone receptor interactor 4 [Source:HGNC Symbol;Acc:HGNC:12310]	-1.8	-2
4125	*NFKBID*	84807	NFKB inhibitor delta [Source:HGNC Symbol;Acc:HGNC:15671]	-1.8	-2
4126	*MRPS10*	55173	mitochondrial ribosomal protein S10 [Source:HGNC Symbol;Acc:HGNC:14502]	-1.8	-2
4127	*DYNLT1*	6993	dynein light chain Tctex-type 1 [Source:HGNC Symbol;Acc:HGNC:11697]	-1.8	-2
4128	*RPGR*	6103	retinitis pigmentosa GTPase regulator [Source:HGNC Symbol;Acc:HGNC:10295]	-1.8	-2
4129	*GRPEL2*	134266	GrpE like 2, mitochondrial [Source:HGNC Symbol;Acc:HGNC:21060]	-1.8	-2
4130	*SURF1*	6834	surfeit 1 [Source:HGNC Symbol;Acc:HGNC:11474]	-1.8	-2
4131	*MTHFD2L*	441024	methylenetetrahydrofolate dehydrogenase (NADP+ dependent) 2-like [Source:HGNC Symbol;Acc:HGNC:31865]	-1.8	-2
4132	*SUCLG1*	8802	succinate-CoA ligase alpha subunit [Source:HGNC Symbol;Acc:HGNC:11449]	-1.8	-2
4133	*ALOX5AP*	241	arachidonate 5-lipoxygenase activating protein [Source:HGNC Symbol;Acc:HGNC:436]	-1.8	-2
4134	*TMEM258*	746	transmembrane protein 258 [Source:HGNC Symbol;Acc:HGNC:1164]	-1.8	-2
4135	*CALD1*	800	caldesmon 1 [Source:HGNC Symbol;Acc:HGNC:1441]	-1.8	-2
4136	*ARHGAP22*	58504	Rho GTPase activating protein 22 [Source:HGNC Symbol;Acc:HGNC:30320]	-1.8	-2
4137	*EMC4*	51234	ER membrane protein complex subunit 4 [Source:HGNC Symbol;Acc:HGNC:28032]	-1.8	-2
4138	*KIF13B*	23303	kinesin family member 13B [Source:HGNC Symbol;Acc:HGNC:14405]	-1.8	-2
4139	*MARCH3*	64844	membrane associated ring-CH-type finger 3 [Source:HGNC Symbol;Acc:HGNC:28728]	-1.8	-2
4140	*GPR83*	10888	G protein-coupled receptor 83 [Source:HGNC Symbol;Acc:HGNC:4523]	-1.8	-2
4141	*RPL7A*	6130	ribosomal protein L7a [Source:HGNC Symbol;Acc:HGNC:10364]	-1.8	-2
4142	*SUOX*	6821	sulfite oxidase [Source:HGNC Symbol;Acc:HGNC:11460]	-1.8	-2
4143	*CHP1*	11261	calcineurin like EF-hand protein 1 [Source:HGNC Symbol;Acc:HGNC:17433]	-1.8	-2
4144	*GNPAT*	8443	glyceronephosphate O-acyltransferase [Source:HGNC Symbol;Acc:HGNC:4416]	-1.8	-2
4145	*ANAPC13*	25847	anaphase promoting complex subunit 13 [Source:HGNC Symbol;Acc:HGNC:24540]	-1.8	-2
4146	*SP140*	11262	SP140 nuclear body protein [Source:HGNC Symbol;Acc:HGNC:17133]	-1.8	-2
4147	*C11orf88*	399949	chromosome 11 open reading frame 88 [Source:HGNC Symbol;Acc:HGNC:25061]	-1.8	-2
4148	*C9orf135*	138255	chromosome 9 open reading frame 135 [Source:HGNC Symbol;Acc:HGNC:31422]	-1.8	-2
4149	*VDAC1*	7416	voltage dependent anion channel 1 [Source:HGNC Symbol;Acc:HGNC:12669]	-1.8	-2
4150	*CXCL12*	6387	C-X-C motif chemokine ligand 12 [Source:HGNC Symbol;Acc:HGNC:10672]	-1.8	-2
4151	*PDGFC*	56034	platelet derived growth factor C [Source:HGNC Symbol;Acc:HGNC:8801]	-1.8	-2
4152	*OSBPL10*	114884	oxysterol binding protein like 10 [Source:HGNC Symbol;Acc:HGNC:16395]	-1.8	-2
4153	*ZNF860*	344787	zinc finger protein 860 [Source:HGNC Symbol;Acc:HGNC:34513]	-1.8	-2
4154	*CACNB3*	784	calcium voltage-gated channel auxiliary subunit beta 3 [Source:HGNC Symbol;Acc:HGNC:1403]	-1.8	-2
4155	*MGST2*	4258	microsomal glutathione S-transferase 2 [Source:HGNC Symbol;Acc:HGNC:7063]	-1.8	-2
4156	*MYO1D*	4642	myosin ID [Source:HGNC Symbol;Acc:HGNC:7598]	-1.8	-2
4157	*PIP5K1A*	8394	phosphatidylinositol-4-phosphate 5-kinase type 1 alpha [Source:HGNC Symbol;Acc:HGNC:8994]	-1.8	-2
4158	*ZNHIT3*	9326	zinc finger HIT-type containing 3 [Source:HGNC Symbol;Acc:HGNC:12309]	-1.8	-2
4159	*TTYH3*	80727	tweety family member 3 [Source:HGNC Symbol;Acc:HGNC:22222]	-1.8	-2
4160	*PAX8*	7849	paired box 8 [Source:HGNC Symbol;Acc:HGNC:8622]	-1.8	-2
4161	*FGFBP1*	9982	fibroblast growth factor binding protein 1 [Source:HGNC Symbol;Acc:HGNC:19695]	-1.8	-2
4162	*HYOU1*	10525	hypoxia up-regulated 1 [Source:HGNC Symbol;Acc:HGNC:16931]	-1.8	-2
4163	*MACROD2*	140733	MACRO domain containing 2 [Source:HGNC Symbol;Acc:HGNC:16126]	-1.8	-2
4164	*CTSC*	1075	cathepsin C [Source:HGNC Symbol;Acc:HGNC:2528]	-1.8	-2
4165	*IFITM2*	10581	interferon induced transmembrane protein 2 [Source:HGNC Symbol;Acc:HGNC:5413]	-1.8	-2
4166	*RAB18*	22931	RAB18, member RAS oncogene family [Source:HGNC Symbol;Acc:HGNC:14244]	-1.8	-2
4167	*ZNF878*	729747	zinc finger protein 878 [Source:HGNC Symbol;Acc:HGNC:37246]	-1.8	-2
4168	*SLAMF6*	114836	SLAM family member 6 [Source:HGNC Symbol;Acc:HGNC:21392]	-1.8	-2
4169	*PIFO*	128344	primary cilia formation [Source:HGNC Symbol;Acc:HGNC:27009]	-1.8	-2

4170	*HEXIM1*	10614	hexamethylene bis-acetamide inducible 1 [Source:HGNC Symbol;Acc:HGNC:24953]	-1.8	-2
4171	*CNIH1*	10175	cornichon family AMPA receptor auxiliary protein 1 [Source:HGNC Symbol;Acc:HGNC:19431]	-1.8	-2
4172	*STX12*	23673	syntaxin 12 [Source:HGNC Symbol;Acc:HGNC:11430]	-1.8	-2
4173	*TPRN*	286262	taperin [Source:HGNC Symbol;Acc:HGNC:26894]	-1.8	-2
4174	*ZNF37A*	7587	zinc finger protein 37A [Source:HGNC Symbol;Acc:HGNC:13102]	-1.8	-2
4175	*SNRNP27*	11017	small nuclear ribonucleoprotein U4/U6.U5 subunit 27 [Source:HGNC Symbol;Acc:HGNC:30240]	-1.8	-2
4176	*KIAA1143*	57456	KIAA1143 [Source:HGNC Symbol;Acc:HGNC:29198]	-1.8	-2
4177	*NKX3-1*	4824	NK3 homeobox 1 [Source:HGNC Symbol;Acc:HGNC:7838]	-1.8	-2
4178	*PLAC8*	51316	placenta specific 8 [Source:HGNC Symbol;Acc:HGNC:19254]	-1.8	-2
4179	*CYP2E1*	1571	cytochrome P450 family 2 subfamily E member 1 [Source:HGNC Symbol;Acc:HGNC:2631]	-1.8	-2
4180	*PHF3*	23469	PHD finger protein 3 [Source:HGNC Symbol;Acc:HGNC:8921]	-1.8	-2
4181	*ZNF354B*	117608	zinc finger protein 354B [Source:HGNC Symbol;Acc:HGNC:17197]	-1.8	-2
4182	*RPL18*	6141	ribosomal protein L18 [Source:HGNC Symbol;Acc:HGNC:10310]	-1.8	-2
4183	*KLKB1*	3818	kallikrein B1 [Source:HGNC Symbol;Acc:HGNC:6371]	-1.8	-2
4184	*MCM10*	55388	minichromosome maintenance 10 replication initiation factor [Source:HGNC Symbol;Acc:HGNC:18043]	-1.8	-2
4185	*HARBI1*	283254	harbinger transposase derived 1 [Source:HGNC Symbol;Acc:HGNC:26522]	-1.8	-2
4186	*TRIM32*	22954	tripartite motif containing 32 [Source:HGNC Symbol;Acc:HGNC:16380]	-1.8	-2
4187	*CDK10*	8558	cyclin dependent kinase 10 [Source:HGNC Symbol;Acc:HGNC:1770]	-1.8	-2
4188	*S100A11*	6282	S100 calcium binding protein A11 [Source:HGNC Symbol;Acc:HGNC:10488]	-1.8	-2
4189	*UNC45A*	55898	unc-45 myosin chaperone A [Source:HGNC Symbol;Acc:HGNC:30594]	-1.8	-2
4190	*PPP1CA*	5499	protein phosphatase 1 catalytic subunit alpha [Source:HGNC Symbol;Acc:HGNC:9281]	-1.8	-2
4191	*DHRS9*	10170	dehydrogenase/reductase 9 [Source:HGNC Symbol;Acc:HGNC:16888]	-1.8	-2
4192	*BRSK1*	84446	BR serine/threonine kinase 1 [Source:HGNC Symbol;Acc:HGNC:18994]	-1.8	-2
4193	*PPARD*	5467	peroxisome proliferator activated receptor delta [Source:HGNC Symbol;Acc:HGNC:9235]	-1.8	-2
4194	*IFT88*	8100	intraflagellar transport 88 [Source:HGNC Symbol;Acc:HGNC:20606]	-1.8	-2
4195	*ARRDC3*	57561	arrestin domain containing 3 [Source:HGNC Symbol;Acc:HGNC:29263]	-1.8	-2
4196	*EPHB6*	2051	EPH receptor B6 [Source:HGNC Symbol;Acc:HGNC:3396]	-1.8	-2
4197	*RILPL1*	353116	Rab interacting lysosomal protein-like 1 [Source:HGNC Symbol;Acc:HGNC:26814]	-1.8	-2
4198	*WDR82*	80335	WD repeat domain 82 [Source:HGNC Symbol;Acc:HGNC:28826]	-1.8	-2
4199	*TRIM26*	7726	tripartite motif containing 26 [Source:HGNC Symbol;Acc:HGNC:12962]	-1.8	-2
4200	*SSR4*	6748	signal sequence receptor subunit 4 [Source:HGNC Symbol;Acc:HGNC:11326]	-1.8	-2
4201	*ZNF391*	346157	zinc finger protein 391 [Source:HGNC Symbol;Acc:HGNC:18779]	-1.8	-2
4202	*NUDT21*	11051	nudix hydrolase 21 [Source:HGNC Symbol;Acc:HGNC:13870]	-1.8	-2
4203	*API5*	8539	apoptosis inhibitor 5 [Source:HGNC Symbol;Acc:HGNC:594]	-1.8	-2
4204	*OXR1*	55074	oxidation resistance 1 [Source:HGNC Symbol;Acc:HGNC:15822]	-1.8	-2
4205	*RNLS*	55328	renalase, FAD dependent amine oxidase [Source:HGNC Symbol;Acc:HGNC:25641]	-1.8	-2
4206	*SNX2*	6643	sorting nexin 2 [Source:HGNC Symbol;Acc:HGNC:11173]	-1.8	-2
4207	*GALNT18*	374378	polypeptide N-acetylgalactosaminyltransferase 18 [Source:HGNC Symbol;Acc:HGNC:30488]	-1.8	-2
4208	*INTU*	27152	inturned planar cell polarity protein [Source:HGNC Symbol;Acc:HGNC:29239]	-1.8	-2
4209	*OXNAD1*	92106	oxidoreductase NAD binding domain containing 1 [Source:HGNC Symbol;Acc:HGNC:25128]	-1.8	-2
4210	*ZDHHC3*	51304	zinc finger DHHC-type containing 3 [Source:HGNC Symbol;Acc:HGNC:18470]	-1.8	-2
4211	*MAP3K1*	4214	mitogen-activated protein kinase kinase kinase 1 [Source:HGNC Symbol;Acc:HGNC:6848]	-1.8	-2
4212	*FAM150B*	285016	family with sequence similarity 150 member B [Source:HGNC Symbol;Acc:HGNC:27683]	-1.8	-2
4213	*CCT7*	10574	chaperonin containing TCP1 subunit 7 [Source:HGNC Symbol;Acc:HGNC:1622]	-1.8	-2
4214	*RASAL1*	8437	RAS protein activator like 1 [Source:HGNC Symbol;Acc:HGNC:9873]	-1.8	-2
4215	*NCK1*	4690	NCK adaptor protein 1 [Source:HGNC Symbol;Acc:HGNC:7664]	-1.8	-2
4216	*TIPARP*	25976	TCDD inducible poly(ADP-ribose) polymerase [Source:HGNC Symbol;Acc:HGNC:23696]	-1.8	-2
4217	*LCK*	3932	LCK proto-oncogene, Src family tyrosine kinase [Source:HGNC Symbol;Acc:HGNC:6524]	-1.8	-2
4218	*BCAT1*	586	branched chain amino acid transaminase 1 [Source:HGNC Symbol;Acc:HGNC:976]	-1.8	-2
4219	*ACSS1*	84532	acyl-CoA synthetase short-chain family member 1 [Source:HGNC Symbol;Acc:HGNC:16091]	-1.8	-2
4220	*NTN5*	126147	netrin 5 [Source:HGNC Symbol;Acc:HGNC:25208]	-1.8	-2
4221	*FAM49B*	51571	family with sequence similarity 49 member B [Source:HGNC Symbol;Acc:HGNC:25216]	-1.8	-2
4222	*CDC25B*	994	cell division cycle 25B [Source:HGNC Symbol;Acc:HGNC:1726]	-1.8	-2
4223	*MOGS*	7841	mannosyl-oligosaccharide glucosidase [Source:HGNC Symbol;Acc:HGNC:24862]	-1.8	-2
4224	*C1orf228*	339541	chromosome 1 open reading frame 228 [Source:HGNC Symbol;Acc:HGNC:34345]	-1.8	-2
4225	*TNFRSF8*	943	tumor necrosis factor receptor superfamily member 8 [Source:HGNC Symbol;Acc:HGNC:11923]	-1.8	-2
4226	*CCL3L3*	414062	C-C motif chemokine ligand 3 like 3 [Source:HGNC Symbol;Acc:HGNC:30554]	-1.8	-2
4227	*LYRM1*	57149	LYR motif containing 1 [Source:HGNC Symbol;Acc:HGNC:25074]	-1.8	-2
4228	*TUBGCP5*	114791	tubulin gamma complex associated protein 5 [Source:HGNC Symbol;Acc:HGNC:18600]	-1.8	-2
4229	*CD28*	940	CD28 molecule [Source:HGNC Symbol;Acc:HGNC:1653]	-1.8	-2
4230	*PTGDR2*	11251	prostaglandin D2 receptor 2 [Source:HGNC Symbol;Acc:HGNC:4502]	-1.8	-2
4231	*ADAMTS2*	9509	ADAM metallopeptidase with thrombospondin type 1 motif 2 [Source:HGNC Symbol;Acc:HGNC:218]	-1.8	-2
4232	*C1orf43*	25912	chromosome 1 open reading frame 43 [Source:HGNC Symbol;Acc:HGNC:29876]	-1.8	-2
4233	*ZNF420*	147923	zinc finger protein 420 [Source:HGNC Symbol;Acc:HGNC:20649]	-1.8	-2
4234	*IDNK*	414328	IDNK, gluconokinase [Source:HGNC Symbol;Acc:HGNC:31367]	-1.8	-2
4235	*MRPL3*	11222	mitochondrial ribosomal protein L3 [Source:HGNC Symbol;Acc:HGNC:10379]	-1.8	-2
4236	*ARFGAP3*	26286	ADP ribosylation factor GTPase activating protein 3 [Source:HGNC Symbol;Acc:HGNC:661]	-1.8	-2
4237	*MAP3K2*	10746	mitogen-activated protein kinase kinase kinase 2 [Source:HGNC Symbol;Acc:HGNC:6854]	-1.8	-2
4238	*TAGLN*	6876	transgelin [Source:HGNC Symbol;Acc:HGNC:11553]	-1.8	-2
4239	*CLTCL1*	8218	clathrin heavy chain like 1 [Source:HGNC Symbol;Acc:HGNC:2093]	-1.8	-2
4240	*SYNCRIP*	10492	synaptotagmin binding cytoplasmic RNA interacting protein [Source:HGNC Symbol;Acc:HGNC:16918]	-1.8	-2
4241	*TAOK2*	9344	TAO kinase 2 [Source:HGNC Symbol;Acc:HGNC:16835]	-1.8	-2
4242	*TMPRSS13*	84000	transmembrane protease, serine 13 [Source:HGNC Symbol;Acc:HGNC:29808]	-1.8	-2

4243	*SETD7*	80854	SET domain containing lysine methyltransferase 7 [Source:HGNC Symbol;Acc:HGNC:30412]	-1.8	-2
4244	*ATP5G2*	517	ATP synthase, H+ transporting, mitochondrial Fo complex subunit C2 (subunit 9) [Source:HGNC Symbol;Acc:HGNC:842]	-1.8	-2
4245	*HDDC2*	51020	HD domain containing 2 [Source:HGNC Symbol;Acc:HGNC:21078]	-1.8	-2
4246	*HLA-DRB1*	3123	major histocompatibility complex, class II, DR beta 1 [Source:HGNC Symbol;Acc:HGNC:4948]	-1.8	-2
4247	*RASA1*	5921	RAS p21 protein activator 1 [Source:HGNC Symbol;Acc:HGNC:9871]	-1.8	-2
4248	*CHRDL1*	91851	chordin-like 1 [Source:HGNC Symbol;Acc:HGNC:29861]	-1.8	-2
4249	*ALG2*	85365	ALG2, alpha-1,3/1,6-mannosyltransferase [Source:HGNC Symbol;Acc:HGNC:23159]	-1.8	-2
4250	*SLC17A7*	57030	solute carrier family 17 member 7 [Source:HGNC Symbol;Acc:HGNC:16704]	-1.8	-2
4251	*KIAA0391*	9692	KIAA0391 [Source:HGNC Symbol;Acc:HGNC:19958]	-1.8	-2
4252	*HIST4H4*	121504	histone cluster 4, H4 [Source:HGNC Symbol;Acc:HGNC:20510]	-1.8	-2
4253	*EPPIN*	57119	epididymal peptidase inhibitor [Source:HGNC Symbol;Acc:HGNC:15932]	-1.8	-2
4254	*ZP4*	57829	zona pellucida glycoprotein 4 [Source:HGNC Symbol;Acc:HGNC:15770]	-1.8	-2
4255	*CENPP*	401541	centromere protein P [Source:HGNC Symbol;Acc:HGNC:32933]	-1.8	-2
4256	*IPPK*	64768	inositol-pentakisphosphate 2-kinase [Source:HGNC Symbol;Acc:HGNC:14645]	-1.8	-2
4257	*TFDP2*	7029	transcription factor Dp-2 [Source:HGNC Symbol;Acc:HGNC:11751]	-1.8	-2
4258	*TMEM117*	84216	transmembrane protein 117 [Source:HGNC Symbol;Acc:HGNC:25308]	-1.8	-2
4259	*SAR1B*	51128	secretion associated Ras related GTPase 1B [Source:HGNC Symbol;Acc:HGNC:10535]	-1.8	-2
4260	*AKAP11*	11215	A-kinase anchoring protein 11 [Source:HGNC Symbol;Acc:HGNC:369]	-1.8	-2
4261	*C21orf2*	755	chromosome 21 open reading frame 2 [Source:HGNC Symbol;Acc:HGNC:1260]	-1.8	-2
4262	*SUN3*	256979	Sad1 and UNC84 domain containing 3 [Source:HGNC Symbol;Acc:HGNC:22429]	-1.8	-2
4263	*PPP4R2*	151987	protein phosphatase 4 regulatory subunit 2 [Source:HGNC Symbol;Acc:HGNC:18296]	-1.8	-2
4264	*ESD*	2098	esterase D [Source:HGNC Symbol;Acc:HGNC:3465]	-1.8	-2
4265	*EXOSC8*	11340	exosome component 8 [Source:HGNC Symbol;Acc:HGNC:17035]	-1.8	-2
4266	*SUPT20H*	55578	SPT20 homolog, SAGA complex component [Source:HGNC Symbol;Acc:HGNC:20596]	-1.8	-2
4267	*CFL1*	1072	cofilin 1 [Source:HGNC Symbol;Acc:HGNC:1874]	-1.8	-2
4268	*NF2*	4771	neurofibromin 2 (merlin) [Source:HGNC Symbol;Acc:HGNC:7773]	-1.8	-2
4269	*BDP1*	55814	B double prime 1, subunit of RNA polymerase III transcription initiation factor IIIB [Source:HGNC Symbol;Acc:HGNC:13652]	-1.8	-2
4270	*QARS*	5859	glutaminyl-tRNA synthetase [Source:HGNC Symbol;Acc:HGNC:9751]	-1.8	-2
4271	*SNX13*	23161	sorting nexin 13 [Source:HGNC Symbol;Acc:HGNC:21335]	-1.8	-2
4272	*DLG1*	1739	discs large MAGUK scaffold protein 1 [Source:HGNC Symbol;Acc:HGNC:2900]	-1.8	-2
4273	*PAM16*	51025	presequence translocase-associated motor 16 homolog (S. cerevisiae) [Source:HGNC Symbol;Acc:HGNC:29679]	-1.8	-2
4274	*NPHP4*	261734	nephrocystin 4 [Source:HGNC Symbol;Acc:HGNC:19104]	-1.8	-2
4275	*DDX58*	23586	DEXD/H-box helicase 58 [Source:HGNC Symbol;Acc:HGNC:19102]	-1.8	-2
4276	*MAN1A1*	4121	mannosidase alpha class 1A member 1 [Source:HGNC Symbol;Acc:HGNC:6821]	-1.8	-2
4277	*SEMA6A*	57556	semaphorin 6A [Source:HGNC Symbol;Acc:HGNC:10738]	-1.8	-2
4278	*HECTD1*	25831	HECT domain E3 ubiquitin protein ligase 1 [Source:HGNC Symbol;Acc:HGNC:20157]	-1.8	-2
4279	*KTI12*	112970	KTI12 chromatin associated homolog [Source:HGNC Symbol;Acc:HGNC:25160]	-1.8	-2
4280	*MYO6*	4646	myosin VI [Source:HGNC Symbol;Acc:HGNC:7605]	-1.8	-2
4281	*STK17A*	9263	serine/threonine kinase 17a [Source:HGNC Symbol;Acc:HGNC:11395]	-1.8	-2
4282	*SLC35B3*	51000	solute carrier family 35 member B3 [Source:HGNC Symbol;Acc:HGNC:21601]	-1.8	-2
4283	*ADH5*	128	alcohol dehydrogenase 5 (class III), chi polypeptide [Source:HGNC Symbol;Acc:HGNC:253]	-1.8	-2
4284	*ATP6V1C1*	528	ATPase H+ transporting V1 subunit C1 [Source:HGNC Symbol;Acc:HGNC:856]	-1.8	-2
4285	*MTHFD2*	10797	methylenetetrahydrofolate dehydrogenase (NADP+ dependent) 2, methenyltetrahydrofolate cyclohydrolase [Source:HGNC Symbol;Acc:HGNC:7434]	-1.8	-2
4286	*UBE2D2*	7322	ubiquitin conjugating enzyme E2 D2 [Source:HGNC Symbol;Acc:HGNC:12475]	-1.8	-2
4287	*DNAH7*	56171	dynein axonemal heavy chain 7 [Source:HGNC Symbol;Acc:HGNC:18661]	-1.8	-2
4288	*CST4*	1472	cystatin S [Source:HGNC Symbol;Acc:HGNC:2476]	-1.8	-2
4289	*CCNT2*	905	cyclin T2 [Source:HGNC Symbol;Acc:HGNC:1600]	-1.8	-2
4290	*GABBR1*	2550	gamma-aminobutyric acid type B receptor subunit 1 [Source:HGNC Symbol;Acc:HGNC:4070]	-1.8	-2
4291	*STAT2*	6773	signal transducer and activator of transcription 2 [Source:HGNC Symbol;Acc:HGNC:11363]	-1.8	-2
4292	*SLC25A11*	8402	solute carrier family 25 member 11 [Source:HGNC Symbol;Acc:HGNC:10981]	-1.8	-2
4293	*TXNDC9*	10190	thioredoxin domain containing 9 [Source:HGNC Symbol;Acc:HGNC:24110]	-1.8	-2
4294	*PALMD*	54873	palmdelphin [Source:HGNC Symbol;Acc:HGNC:15846]	-1.8	-2
4295	*ZDHHC5*	25921	zinc finger DHHC-type containing 5 [Source:HGNC Symbol;Acc:HGNC:18472]	-1.8	-2
4296	*ARHGEF35*	445328	Rho guanine nucleotide exchange factor 35 [Source:HGNC Symbol;Acc:HGNC:33846]	-1.8	-2
4297	*SDF2L1*	23753	stromal cell derived factor 2 like 1 [Source:HGNC Symbol;Acc:HGNC:10676]	-1.8	-2
4298	*SPATA18*	132671	spermatogenesis associated 18 [Source:HGNC Symbol;Acc:HGNC:29579]	-1.8	-2
4299	*SOAT1*	6646	sterol O-acyltransferase 1 [Source:HGNC Symbol;Acc:HGNC:11177]	-1.8	-2
4300	*SSBP1*	6742	single stranded DNA binding protein 1 [Source:HGNC Symbol;Acc:HGNC:11317]	-1.8	-2
4301	*SCIMP*	388325	SLP adaptor and CSK interacting membrane protein [Source:HGNC Symbol;Acc:HGNC:33504]	-1.8	-2
4302	*MPV17L*	255027	MPV17 mitochondrial inner membrane protein like [Source:HGNC Symbol;Acc:HGNC:26827]	-1.8	-2
4303	*NDUFA12*	55967	NADH:ubiquinone oxidoreductase subunit A12 [Source:HGNC Symbol;Acc:HGNC:23987]	-1.8	-2
4304	*PPIB*	5479	peptidylprolyl isomerase B [Source:HGNC Symbol;Acc:HGNC:9255]	-1.8	-2
4305	*PAPPA*	5069	pappalysin 1 [Source:HGNC Symbol;Acc:HGNC:8602]	-1.8	-2
4306	*PABPC1L*	80336	poly(A) binding protein cytoplasmic 1 like [Source:HGNC Symbol;Acc:HGNC:15797]	-1.8	-2
4307	*IFT46*	738	intraflagellar transport 46 [Source:HGNC Symbol;Acc:HGNC:26146]	-1.8	-2
4308	*ABR*	29	active BCR-related [Source:HGNC Symbol;Acc:HGNC:81]	-1.8	-2
4309	*NPC2*	10577	NPC intracellular cholesterol transporter 2 [Source:HGNC Symbol;Acc:HGNC:14537]	-1.8	-2
4310	*CIITA*	4261	class II, major histocompatibility complex, transactivator [Source:HGNC Symbol;Acc:HGNC:7067]	-1.8	-2
4311	*PRKCH*	5583	protein kinase C eta [Source:HGNC Symbol;Acc:HGNC:9403]	-1.8	-2
4312	*SON*	6651	SON DNA binding protein [Source:HGNC Symbol;Acc:HGNC:11183]	-1.8	-2
4313	*PAFAH1B2*	5049	platelet activating factor acetylhydrolase 1b catalytic subunit 2 [Source:HGNC Symbol;Acc:HGNC:8575]	-1.8	-2

4314	*DAZAP1*	26528	DAZ associated protein 1 [Source:HGNC Symbol;Acc:HGNC:2683]	-1.8	-2
4315	*WDR91*	29062	WD repeat domain 91 [Source:HGNC Symbol;Acc:HGNC:24997]	-1.8	-2
4316	*AMPH*	273	amphiphysin [Source:HGNC Symbol;Acc:HGNC:471]	-1.8	-2
4317	*FOXN2*	3344	forkhead box N2 [Source:HGNC Symbol;Acc:HGNC:5281]	-1.8	-2
4318	*SMO*	6608	smoothened, frizzled class receptor [Source:HGNC Symbol;Acc:HGNC:11119]	-1.8	-2
4319	*TCERG1L*	256536	transcription elongation regulator 1 like [Source:HGNC Symbol;Acc:HGNC:23533]	-1.8	-2
4320	*CA5B*	11238	carbonic anhydrase 5B [Source:HGNC Symbol;Acc:HGNC:1378]	-1.8	-2
4321	*NLRP11*	204801	NLR family pyrin domain containing 11 [Source:HGNC Symbol;Acc:HGNC:22945]	-1.8	-2
4322	*GTF2B*	2959	general transcription factor IIB [Source:HGNC Symbol;Acc:HGNC:4648]	-1.8	-2
4323	*CAMK2A*	815	calcium/calmodulin dependent protein kinase II alpha [Source:HGNC Symbol;Acc:HGNC:1460]	-1.9	-2
4324	*CRLS1*	54675	cardiolipin synthase 1 [Source:HGNC Symbol;Acc:HGNC:16148]	-1.9	-2
4325	*TYK2*	7297	tyrosine kinase 2 [Source:HGNC Symbol;Acc:HGNC:12440]	-1.9	-2
4326	*CDK17*	5128	cyclin dependent kinase 17 [Source:HGNC Symbol;Acc:HGNC:8750]	-1.9	-2
4327	*PDCD2L*	84306	programmed cell death 2-like [Source:HGNC Symbol;Acc:HGNC:28194]	-1.9	-2
4328	*XBP1*	7494	X-box binding protein 1 [Source:HGNC Symbol;Acc:HGNC:12801]	-1.9	-2
4329	*UBXN2B*	137886	UBX domain protein 2B [Source:HGNC Symbol;Acc:HGNC:27035]	-1.9	-2
4330	*ANXA6*	309	annexin A6 [Source:HGNC Symbol;Acc:HGNC:544]	-1.9	-2
4331	*LRRC40*	55631	leucine rich repeat containing 40 [Source:HGNC Symbol;Acc:HGNC:26004]	-1.9	-2
4332	*NDUFA9*	4704	NADH:ubiquinone oxidoreductase subunit A9 [Source:HGNC Symbol;Acc:HGNC:7693]	-1.9	-2
4333	*GFRA3*	2676	GDNF family receptor alpha 3 [Source:HGNC Symbol;Acc:HGNC:4245]	-1.9	-2
4334	*GABARAPL2*	11345	GABA type A receptor associated protein like 2 [Source:HGNC Symbol;Acc:HGNC:13291]	-1.9	-2
4335	*GPRASP1*	9737	G protein-coupled receptor associated sorting protein 1 [Source:HGNC Symbol;Acc:HGNC:24834]	-1.9	-2
4336	*MAP3K19*	80122	mitogen-activated protein kinase kinase kinase 19 [Source:HGNC Symbol;Acc:HGNC:26249]	-1.9	-2
4337	*AJUBA*	84962	ajuba LIM protein [Source:HGNC Symbol;Acc:HGNC:20250]	-1.9	-2
4338	*MCHR2*	84539	melanin concentrating hormone receptor 2 [Source:HGNC Symbol;Acc:HGNC:20867]	-1.9	-2
4339	*ARF1*	375	ADP ribosylation factor 1 [Source:HGNC Symbol;Acc:HGNC:652]	-1.9	-2
4340	*EEF2*	1938	eukaryotic translation elongation factor 2 [Source:HGNC Symbol;Acc:HGNC:3214]	-1.9	-2
4341	*TPT1*	7178	tumor protein, translationally-controlled 1 [Source:HGNC Symbol;Acc:HGNC:12022]	-1.9	-2
4342	*ADAM33*	80332	ADAM metallopeptidase domain 33 [Source:HGNC Symbol;Acc:HGNC:15478]	-1.9	-2
4343	*IDH3A*	3419	isocitrate dehydrogenase 3 (NAD(+)) alpha [Source:HGNC Symbol;Acc:HGNC:5384]	-1.9	-2
4344	*OR52B6*	340980	olfactory receptor family 52 subfamily B member 6 [Source:HGNC Symbol;Acc:HGNC:15211]	-1.9	-2
4345	*DNAH17*	8632	dynein axonemal heavy chain 17 [Source:HGNC Symbol;Acc:HGNC:2946]	-1.9	-2
4346	*EPHA3*	2042	EPH receptor A3 [Source:HGNC Symbol;Acc:HGNC:3387]	-1.9	-2
4347	*NCEH1*	57552	neutral cholesterol ester hydrolase 1 [Source:HGNC Symbol;Acc:HGNC:29260]	-1.9	-2
4348	*CXCL1*	2919	C-X-C motif chemokine ligand 1 [Source:HGNC Symbol;Acc:HGNC:4602]	-1.9	-2
4349	*UBE2E1*	7324	ubiquitin conjugating enzyme E2 E1 [Source:HGNC Symbol;Acc:HGNC:12477]	-1.9	-2
4350	*BMS1*	9790	BMS1, ribosome biogenesis factor [Source:HGNC Symbol;Acc:HGNC:23505]	-1.9	-2
4351	*HIPK1*	204851	homeodomain interacting protein kinase 1 [Source:HGNC Symbol;Acc:HGNC:19006]	-1.9	-2
4352	*OR52M1*	119772	olfactory receptor family 52 subfamily M member 1 [Source:HGNC Symbol;Acc:HGNC:15225]	-1.9	-2
4353	*LETM1*	3954	leucine zipper and EF-hand containing transmembrane protein 1 [Source:HGNC Symbol;Acc:HGNC:6556]	-1.9	-2
4354	*DUSP1*	1843	dual specificity phosphatase 1 [Source:HGNC Symbol;Acc:HGNC:3064]	-1.9	-2
4355	*ITPRIPL2*	162073	inositol 1,4,5-trisphosphate receptor interacting protein-like 2 [Source:HGNC Symbol;Acc:HGNC:27257]	-1.9	-2
4356	*GSTO2*	119391	glutathione S-transferase omega 2 [Source:HGNC Symbol;Acc:HGNC:23064]	-1.9	-2
4357	*BPIFB1*	92747	BPI fold containing family B member 1 [Source:HGNC Symbol;Acc:HGNC:16108]	-1.9	-2
4358	*HLA-DMA*	3108	major histocompatibility complex, class II, DM alpha [Source:HGNC Symbol;Acc:HGNC:4934]	-1.9	-2
4359	*TXN2*	25828	thioredoxin 2 [Source:HGNC Symbol;Acc:HGNC:17772]	-1.9	-2
4360	*ABCC6*	368	ATP binding cassette subfamily C member 6 [Source:HGNC Symbol;Acc:HGNC:57]	-1.9	-2
4361	*UBA2*	10054	ubiquitin like modifier activating enzyme 2 [Source:HGNC Symbol;Acc:HGNC:30661]	-1.9	-2
4362	*ADH1C*	126	alcohol dehydrogenase 1C (class I), gamma polypeptide [Source:HGNC Symbol;Acc:HGNC:251]	-1.9	-2
4363	*FAM114A1*	92689	family with sequence similarity 114 member A1 [Source:HGNC Symbol;Acc:HGNC:25087]	-1.9	-2
4364	*SLC7A8*	23428	solute carrier family 7 member 8 [Source:HGNC Symbol;Acc:HGNC:11066]	-1.9	-2
4365	*ZBTB4*	57659	zinc finger and BTB domain containing 4 [Source:HGNC Symbol;Acc:HGNC:23847]	-1.9	-2
4366	*C14orf37*	145407	chromosome 14 open reading frame 37 [Source:HGNC Symbol;Acc:HGNC:19846]	-1.9	-2
4367	*AKTIP*	64400	AKT interacting protein [Source:HGNC Symbol;Acc:HGNC:16710]	-1.9	-2
4368	*KDR*	3791	kinase insert domain receptor [Source:HGNC Symbol;Acc:HGNC:6307]	-1.9	-2
4369	*NCMAP*	400746	non-compact myelin associated protein [Source:HGNC Symbol;Acc:HGNC:29332]	-1.9	-2
4370	*DHRS4L2*	317749	dehydrogenase/reductase 4 like 2 [Source:HGNC Symbol;Acc:HGNC:19731]	-1.9	-2
4371	*CD81*	975	CD81 molecule [Source:HGNC Symbol;Acc:HGNC:1701]	-1.9	-2
4372	*LMO4*	8543	LIM domain only 4 [Source:HGNC Symbol;Acc:HGNC:6644]	-1.9	-2
4373	*CMKLR1*	1240	chemerin chemokine-like receptor 1 [Source:HGNC Symbol;Acc:HGNC:2121]	-1.9	-2
4374	*DDX6*	1656	DEAD-box helicase 6 [Source:HGNC Symbol;Acc:HGNC:2747]	-1.9	-2
4375	*VNN1*	8876	vanin 1 [Source:HGNC Symbol;Acc:HGNC:12705]	-1.9	-2
4376	*B4GAT1*	11041	beta-1,4-glucuronyltransferase 1 [Source:HGNC Symbol;Acc:HGNC:15685]	-1.9	-2
4377	*PLEKHG3*	26030	pleckstrin homology and RhoGEF domain containing G3 [Source:HGNC Symbol;Acc:HGNC:20364]	-1.9	-2
4378	*IDH1*	3417	isocitrate dehydrogenase (NADP(+)) 1, cytosolic [Source:HGNC Symbol;Acc:HGNC:5382]	-1.9	-2
4379	*HLA-DPA1*	3113	major histocompatibility complex, class II, DP alpha 1 [Source:HGNC Symbol;Acc:HGNC:4938]	-1.9	-2
4380	*GLIS3*	169792	GLIS family zinc finger 3 [Source:HGNC Symbol;Acc:HGNC:28510]	-1.9	-2
4381	*ARHGEF19*	128272	Rho guanine nucleotide exchange factor 19 [Source:HGNC Symbol;Acc:HGNC:26604]	-1.9	-2
4382	*ACLY*	47	ATP citrate lyase [Source:HGNC Symbol;Acc:HGNC:115]	-1.9	-2
4383	*APOA1BP*	128240	apolipoprotein A-1-bindingprotein	-1.9	-2
4384	*SNRPE*	6635	small nuclear ribonucleoprotein polypeptide E [Source:HGNC Symbol;Acc:HGNC:11161]	-1.9	-2
4385	*RPL11*	6135	ribosomal protein L11 [Source:HGNC Symbol;Acc:HGNC:10301]	-1.9	-2

4386	*PRR15*	222171	proline rich 15 [Source:HGNC Symbol;Acc:HGNC:22310]	-1.9	-2
4387	*TMEM217*	221468	transmembrane protein 217 [Source:HGNC Symbol;Acc:HGNC:21238]	-1.9	-2
4388	*RPL19*	6143	ribosomal protein L19 [Source:HGNC Symbol;Acc:HGNC:10312]	-1.9	-2
4389	*FERMT1*	55612	fermitin family member 1 [Source:HGNC Symbol;Acc:HGNC:15889]	-1.9	-2
4390	*BTG2*	7832	BTG family member 2 [Source:HGNC Symbol;Acc:HGNC:1131]	-1.9	-2
4391	*ATP6V0A1*	535	ATPase H+ transporting V0 subunit a1 [Source:HGNC Symbol;Acc:HGNC:865]	-1.9	-2
4392	*TRABD2A*	129293	TraB domain containing 2A [Source:HGNC Symbol;Acc:HGNC:27013]	-1.9	-2
4393	*DNPH1*	10591	2'-deoxynucleoside 5'-phosphate N-hydrolase 1 [Source:HGNC Symbol;Acc:HGNC:21218]	-1.9	-2
4394	*ARL5B*	221079	ADP ribosylation factor like GTPase 5B [Source:HGNC Symbol;Acc:HGNC:23052]	-1.9	-2
4395	*TBC1D22A*	25771	TBC1 domain family member 22A [Source:HGNC Symbol;Acc:HGNC:1309]	-1.9	-2
4396	*NPM1*	4869	nucleophosmin (nucleolar phosphoprotein B23, numatrin) [Source:HGNC Symbol;Acc:HGNC:7910]	-1.9	-2
4397	*MCFD2*	90411	multiple coagulation factor deficiency 2 [Source:HGNC Symbol;Acc:HGNC:18451]	-1.9	-2
4398	*GTPBP4*	23560	GTP binding protein 4 [Source:HGNC Symbol;Acc:HGNC:21535]	-1.9	-2
4399	*MARCKS*	4082	myristoylated alanine rich protein kinase C substrate [Source:HGNC Symbol;Acc:HGNC:6759]	-1.9	-2
4400	*BCL7B*	9275	B-cell CLL/lymphoma 7B [Source:HGNC Symbol;Acc:HGNC:1005]	-1.9	-2
4401	*HCAR2*	338442	hydroxycarboxylic acid receptor 2 [Source:HGNC Symbol;Acc:HGNC:24827]	-1.9	-2
4402	*WDR12*	55759	WD repeat domain 12 [Source:HGNC Symbol;Acc:HGNC:14098]	-1.9	-2
4403	*SPINT2*	10653	serine peptidase inhibitor, Kunitz type, 2 [Source:HGNC Symbol;Acc:HGNC:11247]	-1.9	-2
4404	*YTHDC1*	91746	YTH domain containing 1 [Source:HGNC Symbol;Acc:HGNC:30626]	-1.9	-2
4405	*SRSF12*	135295	serine and arginine rich splicing factor 12 [Source:HGNC Symbol;Acc:HGNC:21220]	-1.9	-2
4406	*GOLGA8A*	23015	golgin A8 family member A [Source:HGNC Symbol;Acc:HGNC:31972]	-1.9	-2
4407	*EIF3A*	8661	eukaryotic translation initiation factor 3 subunit A [Source:HGNC Symbol;Acc:HGNC:3271]	-1.9	-2
4408	*UTP6*	55813	UTP6, small subunit processome component [Source:HGNC Symbol;Acc:HGNC:18279]	-1.9	-2
4409	*MTSS1*	9788	metastasis suppressor 1 [Source:HGNC Symbol;Acc:HGNC:20443]	-1.9	-2
4410	*LACE1*	246269	lactation elevated 1 [Source:HGNC Symbol;Acc:HGNC:16411]	-1.9	-2
4411	*RGS5*	8490	regulator of G-protein signaling 5 [Source:HGNC Symbol;Acc:HGNC:10001]	-1.9	-2
4412	*ACAD9*	28976	acyl-CoA dehydrogenase family member 9 [Source:HGNC Symbol;Acc:HGNC:21497]	-1.9	-2
4413	*PTPMT1*	114971	protein tyrosine phosphatase, mitochondrial 1 [Source:HGNC Symbol;Acc:HGNC:26965]	-1.9	-2
4414	*BDH2*	56898	3-hydroxybutyrate dehydrogenase, type 2 [Source:HGNC Symbol;Acc:HGNC:32389]	-1.9	-2
4415	*GBP3*	2635	guanylate binding protein 3 [Source:HGNC Symbol;Acc:HGNC:4184]	-1.9	-2
4416	*KIAA0408*	9729	KIAA0408 [Source:HGNC Symbol;Acc:HGNC:21636]	-1.9	-2
4417	*SOGA3*	387104	SOGA family member 3 [Source:HGNC Symbol;Acc:HGNC:21494]	-1.9	-2
4418	*KLHL3*	26249	kelch like family member 3 [Source:HGNC Symbol;Acc:HGNC:6354]	-1.9	-2
4419	*MICAL2*	9645	microtubule associated monooxygenase, calponin and LIM domain containing 2 [Source:HGNC Symbol;Acc:HGNC:24693]	-1.9	-2
4420	*RPS27A*	6233	ribosomal protein S27a [Source:HGNC Symbol;Acc:HGNC:10417]	-1.9	-2
4421	*CCZ1*	51622	CCZ1 homolog, vacuolar protein trafficking and biogenesis associated [Source:HGNC Symbol;Acc:HGNC:21691]	-1.9	-2
4422	*EIF3F*	8665	eukaryotic translation initiation factor 3 subunit F [Source:HGNC Symbol;Acc:HGNC:3275]	-1.9	-2
4423	*SOX4*	6659	SRY-box 4 [Source:HGNC Symbol;Acc:HGNC:11200]	-1.9	-2
4424	*TBC1D3*	729873	TBC1 domain family member 3 [Source:HGNC Symbol;Acc:HGNC:19031]	-1.9	-2
4425	*GFM2*	84340	G elongation factor, mitochondrial 2 [Source:HGNC Symbol;Acc:HGNC:29682]	-1.9	-2
4426	*PRB3*	5544	proline rich protein BstNI subfamily 3 [Source:HGNC Symbol;Acc:HGNC:9339]	-1.9	-2
4427	*SAA1*	6288	serum amyloid A1 [Source:HGNC Symbol;Acc:HGNC:10513]	-1.9	-2
4428	*OAZ1*	4946	ornithine decarboxylase antizyme 1 [Source:HGNC Symbol;Acc:HGNC:8095]	-1.9	-2
4429	*EML4*	27436	echinoderm microtubule associated protein like 4 [Source:HGNC Symbol;Acc:HGNC:1316]	-1.9	-2
4430	*FAM122C*	159091	family with sequence similarity 122C [Source:HGNC Symbol;Acc:HGNC:25202]	-1.9	-2
4431	*TMEM47*	83604	transmembrane protein 47 [Source:HGNC Symbol;Acc:HGNC:18515]	-1.9	-2
4432	*CHST10*	9486	carbohydrate sulfotransferase 10 [Source:HGNC Symbol;Acc:HGNC:19650]	-1.9	-2
4433	*METTL23*	124512	methyltransferase like 23 [Source:HGNC Symbol;Acc:HGNC:26988]	-1.9	-2
4434	*DCK*	1633	deoxycytidine kinase [Source:HGNC Symbol;Acc:HGNC:2704]	-1.9	-2
4435	*PIBF1*	10464	progesterone immunomodulatory binding factor 1 [Source:HGNC Symbol;Acc:HGNC:23352]	-1.9	-2
4436	*HLA-A*	3105	major histocompatibility complex, class I, A [Source:HGNC Symbol;Acc:HGNC:4931]	-1.9	-2
4437	*ERO1A*	30001	endoplasmic reticulum oxidoreductase alpha [Source:HGNC Symbol;Acc:HGNC:13280]	-1.9	-2
4438	*KIAA2013*	90231	KIAA2013 [Source:HGNC Symbol;Acc:HGNC:28513]	-1.9	-2
4439	*CD46*	4179	CD46 molecule [Source:HGNC Symbol;Acc:HGNC:6953]	-1.9	-2
4440	*PON3*	5446	paraoxonase 3 [Source:HGNC Symbol;Acc:HGNC:9206]	-1.9	-2
4441	*OR2C3*	81472	olfactory receptor family 2 subfamily C member 3 [Source:HGNC Symbol;Acc:HGNC:15005]	-1.9	-2
4442	*HRH2*	3274	histamine receptor H2 [Source:HGNC Symbol;Acc:HGNC:5183]	-1.9	-2
4443	*TNIP3*	79931	TNFAIP3 interacting protein 3 [Source:HGNC Symbol;Acc:HGNC:19315]	-1.9	-2
4444	*PLN*	5350	phospholamban [Source:HGNC Symbol;Acc:HGNC:9080]	-1.9	-2
4445	*MITF*	4286	melanogenesis associated transcription factor [Source:HGNC Symbol;Acc:HGNC:7105]	-1.9	-2
4446	*DDX60L*	91351	DEAD-box helicase 60-like [Source:HGNC Symbol;Acc:HGNC:26429]	-1.9	-2
4447	*RALA*	5898	RALA Ras like proto-oncogene A [Source:HGNC Symbol;Acc:HGNC:9839]	-1.9	-2
4448	*DARS*	1615	aspartyl-tRNA synthetase [Source:HGNC Symbol;Acc:HGNC:2678]	-1.9	-2
4449	*TNFSF13*	8741	tumor necrosis factor superfamily member 13 [Source:HGNC Symbol;Acc:HGNC:11928]	-1.9	-2
4450	*LMBRD1*	55788	LMBR1 domain containing 1 [Source:HGNC Symbol;Acc:HGNC:23038]	-1.9	-2
4451	*TPD52*	7163	tumor protein D52 [Source:HGNC Symbol;Acc:HGNC:12005]	-1.9	-2
4452	*ATG12*	9140	autophagy related 12 [Source:HGNC Symbol;Acc:HGNC:588]	-1.9	-2
4453	*PAPSS2*	9060	3'-phosphoadenosine 5'-phosphosulfate synthase 2 [Source:HGNC Symbol;Acc:HGNC:8604]	-1.9	-2
4454	*TRIM16L*	147166	tripartite motif containing 16-like [Source:HGNC Symbol;Acc:HGNC:32670]	-1.9	-2
4455	*APBA1*	320	amyloid beta precursor protein binding family A member 1 [Source:HGNC Symbol;Acc:HGNC:578]	-1.9	-2
4456	*REV3L*	5980	REV3 like, DNA directed polymerase zeta catalytic subunit [Source:HGNC Symbol;Acc:HGNC:9968]	-1.9	-2
4457	*CREM*	1390	cAMP responsive element modulator [Source:HGNC Symbol;Acc:HGNC:2352]	-1.9	-2
4458	*TMEM53*	79639	transmembrane protein 53 [Source:HGNC Symbol;Acc:HGNC:26186]	-1.9	-2

4459	*CNTNAP1*	8506	contactin associated protein 1 [Source:HGNC Symbol;Acc:HGNC:8011]	-1.9	-2
4460	*RAB31*	11031	RAB31, member RAS oncogene family [Source:HGNC Symbol;Acc:HGNC:9771]	-1.9	-2
4461	*CCDC157*	550631	coiled-coil domain containing 157 [Source:HGNC Symbol;Acc:HGNC:33854]	-1.9	-2
4462	*ZNF418*	147686	zinc finger protein 418 [Source:HGNC Symbol;Acc:HGNC:20647]	-1.9	-2
4463	*FTL*	2512	ferritin, light polypeptide [Source:HGNC Symbol;Acc:HGNC:3999]	-1.9	-2
4464	*GTF2I*	2969	general transcription factor IIi [Source:HGNC Symbol;Acc:HGNC:4659]	-1.9	-2
4465	*RAF1*	5894	Raf-1 proto-oncogene, serine/threonine kinase [Source:HGNC Symbol;Acc:HGNC:9829]	-1.9	-2
4466	*KARS*	3735	lysyl-tRNA synthetase [Source:HGNC Symbol;Acc:HGNC:6215]	-1.9	-2
4467	*FAM129C*	199786	family with sequence similarity 129 member C [Source:HGNC Symbol;Acc:HGNC:24130]	-1.9	-2
4468	*TSPAN16*	26526	tetraspanin 16 [Source:HGNC Symbol;Acc:HGNC:30725]	-1.9	-2
4469	*NR4A2*	4929	nuclear receptor subfamily 4 group A member 2 [Source:HGNC Symbol;Acc:HGNC:7981]	-1.9	-2
4470	*PLA2G10*	8399	phospholipase A2 group X [Source:HGNC Symbol;Acc:HGNC:9029]	-1.9	-2
4471	*ETS1*	2113	ETS proto-oncogene 1, transcription factor [Source:HGNC Symbol;Acc:HGNC:3488]	-1.9	-2
4472	*MEF2B*	100271849	myocyte enhancer factor 2B [Source:HGNC Symbol;Acc:HGNC:6995]	-1.9	-2
4473	*EMCN*	51705	endomucin [Source:HGNC Symbol;Acc:HGNC:16041]	-1.9	-2
4474	*SLC27A4*	10999	solute carrier family 27 member 4 [Source:HGNC Symbol;Acc:HGNC:10998]	-1.9	-2
4475	*SYPL1*	6856	synaptophysin like 1 [Source:HGNC Symbol;Acc:HGNC:11507]	-1.9	-2
4476	*LMCD1*	29995	LIM and cysteine rich domains 1 [Source:HGNC Symbol;Acc:HGNC:6633]	-1.9	-2
4477	*GEMIN8*	54960	gem nuclear organelle associated protein 8 [Source:HGNC Symbol;Acc:HGNC:26044]	-1.9	-2
4478	*CD8A*	925	CD8a molecule [Source:HGNC Symbol;Acc:HGNC:1706]	-1.9	-2
4479	*CYP27C1*	339761	cytochrome P450 family 27 subfamily C member 1 [Source:HGNC Symbol;Acc:HGNC:33480]	-1.9	-2
4480	*CRYAB*	1410	crystallin alpha B [Source:HGNC Symbol;Acc:HGNC:2389]	-1.9	-2
4481	*LPAR6*	10161	lysophosphatidic acid receptor 6 [Source:HGNC Symbol;Acc:HGNC:15520]	-1.9	-2
4482	*RHCG*	51458	Rh family C glycoprotein [Source:HGNC Symbol;Acc:HGNC:18140]	-1.9	-2
4483	*ARPC5*	10092	actin related protein 2/3 complex subunit 5 [Source:HGNC Symbol;Acc:HGNC:708]	-1.9	-2
4484	*OSTF1*	26578	osteoclast stimulating factor 1 [Source:HGNC Symbol;Acc:HGNC:8510]	-1.9	-2
4485	*CDIPT*	10423	CDP-diacylglycerol--inositol 3-phosphatidyltransferase [Source:HGNC Symbol;Acc:HGNC:1769]	-1.9	-2
4486	*RING1*	6015	ring finger protein 1 [Source:HGNC Symbol;Acc:HGNC:10018]	-1.9	-2
4487	*SAP18*	10284	Sin3A associated protein 18 [Source:HGNC Symbol;Acc:HGNC:10530]	-1.9	-2
4488	*KCNE4*	23704	potassium voltage-gated channel subfamily E regulatory subunit 4 [Source:HGNC Symbol;Acc:HGNC:6244]	-1.9	-2
4489	*TCEANC2*	127428	transcription elongation factor A N-terminal and central domain containing 2 [Source:HGNC Symbol;Acc:HGNC:26494]	-1.9	-2
4490	*CXorf23*	256643	chromosome X open reading frame 23 [Source:HGNC Symbol;Acc:HGNC:27413]	-1.9	-2
4491	*ADGRA2*	25960	adhesion G protein-coupled receptor A2 [Source:HGNC Symbol;Acc:HGNC:17849]	-1.9	-2
4492	*BRF2*	55290	BRF2, RNA polymerase III transcription initiation factor 50 kDa subunit [Source:HGNC Symbol;Acc:HGNC:17298]	-1.9	-2
4493	*PSMD8*	5714	proteasome 26S subunit, non-ATPase 8 [Source:HGNC Symbol;Acc:HGNC:9566]	-1.9	-2
4494	*FUT11*	170384	fucosyltransferase 11 [Source:HGNC Symbol;Acc:HGNC:19233]	-1.9	-2
4495	*TXNDC15*	79770	thioredoxin domain containing 15 [Source:HGNC Symbol;Acc:HGNC:20652]	-1.9	-2
4496	*RPS3A*	6189	ribosomal protein S3A [Source:HGNC Symbol;Acc:HGNC:10421]	-1.9	-2
4497	*ARSG*	22901	arylsulfatase G [Source:HGNC Symbol;Acc:HGNC:24102]	-1.9	-2
4498	*MS4A7*	58475	membrane spanning 4-domains A7 [Source:HGNC Symbol;Acc:HGNC:13378]	-1.9	-2
4499	*NEUROD2*	4761	neuronal differentiation 2 [Source:HGNC Symbol;Acc:HGNC:7763]	-1.9	-2
4500	*CNTNAP5*	129684	contactin associated protein like 5 [Source:HGNC Symbol;Acc:HGNC:18748]	-1.9	-2
4501	*E2F2*	1870	E2F transcription factor 2 [Source:HGNC Symbol;Acc:HGNC:3114]	-1.9	-2
4502	*PCID2*	55795	PCI domain containing 2 [Source:HGNC Symbol;Acc:HGNC:25653]	-1.9	-2
4503	*CAPZA1*	829	capping actin protein of muscle Z-line alpha subunit 1 [Source:HGNC Symbol;Acc:HGNC:1488]	-1.9	-2
4504	*RAB15*	376267	RAB15, member RAS oncogene family [Source:HGNC Symbol;Acc:HGNC:20150]	-1.9	-2
4505	*CETN3*	1070	centrin 3 [Source:HGNC Symbol;Acc:HGNC:1868]	-1.9	-2
4506	*GSTCD*	79807	glutathione S-transferase C-terminal domain containing [Source:HGNC Symbol;Acc:HGNC:25806]	-1.9	-2
4507	*TGFBR2*	7048	transforming growth factor beta receptor 2 [Source:HGNC Symbol;Acc:HGNC:11773]	-1.9	-2
4508	*KLK7*	5650	kallikrein related peptidase 7 [Source:HGNC Symbol;Acc:HGNC:6368]	-1.9	-2
4509	*KDM3A*	55818	lysine demethylase 3A [Source:HGNC Symbol;Acc:HGNC:20815]	-1.9	-2
4510	*PDE3A*	5139	phosphodiesterase 3A [Source:HGNC Symbol;Acc:HGNC:8778]	-1.9	-2
4511	*ELK4*	2005	ELK4, ETS transcription factor [Source:HGNC Symbol;Acc:HGNC:3326]	-1.9	-2
4512	*RDM1*	201299	RAD52 motif containing 1 [Source:HGNC Symbol;Acc:HGNC:19950]	-1.9	-2
4513	*FBL*	2091	fibrillarin [Source:HGNC Symbol;Acc:HGNC:3599]	-1.9	-2
4514	*HYAL4*	23553	hyaluronoglucosaminidase 4 [Source:HGNC Symbol;Acc:HGNC:5323]	-1.9	-2
4515	*DNAJC19*	131118	DnaJ heat shock protein family (Hsp40) member C19 [Source:HGNC Symbol;Acc:HGNC:30528]	-1.9	-2
4516	*RPRML*	388394	reprimo like [Source:HGNC Symbol;Acc:HGNC:32422]	-1.9	-2
4517	*KIAA1671*	85379	KIAA1671 [Source:HGNC Symbol;Acc:HGNC:29345]	-1.9	-2
4518	*AMER3*	205147	APC membrane recruitment protein 3 [Source:HGNC Symbol;Acc:HGNC:26771]	-1.9	-2
4519	*P2RY2*	5029	purinergic receptor P2Y2 [Source:HGNC Symbol;Acc:HGNC:8541]	-2.0	-2
4520	*AKT3*	10000	AKT serine/threonine kinase 3 [Source:HGNC Symbol;Acc:HGNC:393]	-2.0	-2
4521	*SRSF10*	10772	serine and arginine rich splicing factor 10 [Source:HGNC Symbol;Acc:HGNC:16713]	-2.0	-2
4522	*DEGS1*	8560	delta(4)-desaturase, sphingolipid 1 [Source:HGNC Symbol;Acc:HGNC:13709]	-2.0	-2
4523	*RSAD1*	55316	radical S-adenosyl methionine domain containing 1 [Source:HGNC Symbol;Acc:HGNC:25634]	-2.0	-2
4524	*OR2M3*	127062	olfactory receptor family 2 subfamily M member 3 [Source:HGNC Symbol;Acc:HGNC:8269]	-2.0	-2
4525	*STK38L*	23012	serine/threonine kinase 38 like [Source:HGNC Symbol;Acc:HGNC:17848]	-2.0	-2
4526	*ZBTB2*	57621	zinc finger and BTB domain containing 2 [Source:HGNC Symbol;Acc:HGNC:20868]	-2.0	-2
4527	*SIDT2*	51092	SID1 transmembrane family member 2 [Source:HGNC Symbol;Acc:HGNC:24272]	-2.0	-2
4528	*FXYD3*	5349	FXYD domain containing ion transport regulator 3 [Source:HGNC Symbol;Acc:HGNC:4027]	-2.0	-2
4529	*KPNA6*	23633	karyopherin subunit alpha 6 [Source:HGNC Symbol;Acc:HGNC:6399]	-2.0	-2
4530	*PTGR1*	22949	prostaglandin reductase 1 [Source:HGNC Symbol;Acc:HGNC:18429]	-2.0	-2
4531	*SEPW1*	6415	selenoprotein W, 1 [Source:HGNC Symbol;Acc:HGNC:10752]	-2.0	-2

4532	*UBAC1*	10422	UBA domain containing 1 [Source:HGNC Symbol;Acc:HGNC:30221]	-2.0	-2
4533	*FUT3*	2525	fucosyltransferase 3 (Lewis blood group) [Source:HGNC Symbol;Acc:HGNC:4014]	-2.0	-2
4534	*PRX*	57716	periaxin [Source:HGNC Symbol;Acc:HGNC:13797]	-2.0	-2
4535	*GNAI3*	2773	G protein subunit alpha i3 [Source:HGNC Symbol;Acc:HGNC:4387]	-2.0	-2
4536	*CADPS*	8618	calcium dependent secretion activator [Source:HGNC Symbol;Acc:HGNC:1426]	-2.0	-2
4537	*C9orf24*	84688	chromosome 9 open reading frame 24 [Source:HGNC Symbol;Acc:HGNC:19919]	-2.0	-2
4538	*LAMTOR4*	389541	late endosomal/lysosomal adaptor, MAPK and MTOR activator 4 [Source:HGNC Symbol;Acc:HGNC:33772]	-2.0	-2
4539	*MYCL*	4610	v-myc avian myelocytomatosis viral oncogene lung carcinoma derived homolog [Source:HGNC Symbol;Acc:HGNC:7555]	-2.0	-2
4540	*KCNH8*	131096	potassium voltage-gated channel subfamily H member 8 [Source:HGNC Symbol;Acc:HGNC:18864]	-2.0	-2
4541	*CNTN1*	1272	contactin 1 [Source:HGNC Symbol;Acc:HGNC:2171]	-2.0	-2
4542	*SLC39A13*	91252	solute carrier family 39 member 13 [Source:HGNC Symbol;Acc:HGNC:20859]	-2.0	-2
4543	*HLA-DQA1*	3117	major histocompatibility complex, class II, DQ alpha 1 [Source:HGNC Symbol;Acc:HGNC:4942]	-2.0	-2
4544	*PLEKHB2*	55041	pleckstrin homology domain containing B2 [Source:HGNC Symbol;Acc:HGNC:19236]	-2.0	-2
4545	*ZNF219*	51222	zinc finger protein 219 [Source:HGNC Symbol;Acc:HGNC:13011]	-2.0	-2
4546	*OR4Q3*	441669	olfactory receptor family 4 subfamily Q member 3 [Source:HGNC Symbol;Acc:HGNC:15426]	-2.0	-2
4547	*SYNGR1*	9145	synaptogyrin 1 [Source:HGNC Symbol;Acc:HGNC:11498]	-2.0	-2
4548	*PTPRH*	5794	protein tyrosine phosphatase, receptor type H [Source:HGNC Symbol;Acc:HGNC:9672]	-2.0	-2
4549	*ABLIM1*	3983	actin binding LIM protein 1 [Source:HGNC Symbol;Acc:HGNC:78]	-2.0	-2
4550	*GORASP2*	26003	golgi reassembly stacking protein 2 [Source:HGNC Symbol;Acc:HGNC:17500]	-2.0	-2
4551	*SRF*	6722	serum response factor [Source:HGNC Symbol;Acc:HGNC:11291]	-2.0	-2
4552	*LAX1*	54900	lymphocyte transmembrane adaptor 1 [Source:HGNC Symbol;Acc:HGNC:26005]	-2.0	-2
4553	*METTL1*	4234	methyltransferase like 1 [Source:HGNC Symbol;Acc:HGNC:7030]	-2.0	-2
4554	*OSBPL11*	114885	oxysterol binding protein like 11 [Source:HGNC Symbol;Acc:HGNC:16397]	-2.0	-2
4555	*FNDC3A*	22862	fibronectin type III domain containing 3A [Source:HGNC Symbol;Acc:HGNC:20296]	-2.0	-2
4556	*PSMB2*	5690	proteasome subunit beta 2 [Source:HGNC Symbol;Acc:HGNC:9539]	-2.0	-2
4557	*PPT2*	9374	palmitoyl-protein thioesterase 2 [Source:HGNC Symbol;Acc:HGNC:9326]	-2.0	-2
4558	*PAXBP1*	94104	PAX3 and PAX7 binding protein 1 [Source:HGNC Symbol;Acc:HGNC:13579]	-2.0	-2
4559	*C21orf59*	56683	chromosome 21 open reading frame 59 [Source:HGNC Symbol;Acc:HGNC:1301]	-2.0	-2
4560	*PLAG1*	5324	PLAG1 zinc finger [Source:HGNC Symbol;Acc:HGNC:9045]	-2.0	-2
4561	*RTBDN*	83546	retbindin [Source:HGNC Symbol;Acc:HGNC:30310]	-2.0	-2
4562	*ESYT1*	23344	extended synaptotagmin 1 [Source:HGNC Symbol;Acc:HGNC:29534]	-2.0	-2
4563	*ALG11*	440138	ALG11, alpha-1,2-mannosyltransferase [Source:HGNC Symbol;Acc:HGNC:32456]	-2.0	-2
4564	*SPATS1*	221409	spermatogenesis associated serine rich 1 [Source:HGNC Symbol;Acc:HGNC:22957]	-2.0	-2
4565	*INO80*	54617	INO80 complex subunit [Source:HGNC Symbol;Acc:HGNC:26956]	-2.0	-2
4566	*SLC35A2*	7355	solute carrier family 35 member A2 [Source:HGNC Symbol;Acc:HGNC:11022]	-2.0	-2
4567	*ASUN*	55726	asunder, spermatogenesis regulator [Source:HGNC Symbol;Acc:HGNC:20174]	-2.0	-2
4568	*TPRKB*	51002	TP53RK binding protein [Source:HGNC Symbol;Acc:HGNC:24259]	-2.0	-2
4569	*SLC2A5*	6518	solute carrier family 2 member 5 [Source:HGNC Symbol;Acc:HGNC:11010]	-2.0	-2
4570	*GNG12*	55970	G protein subunit gamma 12 [Source:HGNC Symbol;Acc:HGNC:19663]	-2.0	-2
4571	*PARP4*	143	poly(ADP-ribose) polymerase family member 4 [Source:HGNC Symbol;Acc:HGNC:271]	-2.0	-2
4572	*ZSWIM4*	65249	zinc finger SWIM-type containing 4 [Source:HGNC Symbol;Acc:HGNC:25704]	-2.0	-2
4573	*YME1L1*	10730	YME1 like 1 ATPase [Source:HGNC Symbol;Acc:HGNC:12843]	-2.0	-2
4574	*TDP2*	51567	tyrosyl-DNA phosphodiesterase 2 [Source:HGNC Symbol;Acc:HGNC:17768]	-2.0	-2
4575	*FAM96A*	84191	family with sequence similarity 96 member A [Source:HGNC Symbol;Acc:HGNC:26235]	-2.0	-2
4576	*TGM5*	9333	transglutaminase 5 [Source:HGNC Symbol;Acc:HGNC:11781]	-2.0	-2
4577	*TMSB15B*	286527	thymosin beta 15B [Source:HGNC Symbol;Acc:HGNC:28612]	-2.0	-2
4578	*NOLC1*	9221	nucleolar and coiled-body phosphoprotein 1 [Source:HGNC Symbol;Acc:HGNC:15608]	-2.0	-2
4579	*LSM2*	57819	LSM2 homolog, U6 small nuclear RNA and mRNA degradation associated [Source:HGNC Symbol;Acc:HGNC:13940]	-2.0	-2
4580	*SLC1A6*	6511	solute carrier family 1 member 6 [Source:HGNC Symbol;Acc:HGNC:10944]	-2.0	-2
4581	*DDIT4*	54541	DNA damage inducible transcript 4 [Source:HGNC Symbol;Acc:HGNC:24944]	-2.0	-2
4582	*GPLD1*	2822	glycosylphosphatidylinositol specific phospholipase D1 [Source:HGNC Symbol;Acc:HGNC:4459]	-2.0	-2
4583	*CDK2*	1017	cyclin dependent kinase 2 [Source:HGNC Symbol;Acc:HGNC:1771]	-2.0	-2
4584	*SLC35E4*	339665	solute carrier family 35 member E4 [Source:HGNC Symbol;Acc:HGNC:17058]	-2.0	-2
4585	*RPL30*	6156	ribosomal protein L30 [Source:HGNC Symbol;Acc:HGNC:10333]	-2.0	-2
4586	*PITPNM3*	83394	PITPNM family member 3 [Source:HGNC Symbol;Acc:HGNC:21043]	-2.0	-2
4587	*MRPL48*	51642	mitochondrial ribosomal protein L48 [Source:HGNC Symbol;Acc:HGNC:16653]	-2.0	-2
4588	*PARP14*	54625	poly(ADP-ribose) polymerase family member 14 [Source:HGNC Symbol;Acc:HGNC:29232]	-2.0	-2
4589	*HIBCH*	26275	3-hydroxyisobutyryl-CoA hydrolase [Source:HGNC Symbol;Acc:HGNC:4908]	-2.0	-2
4590	*NEK4*	6787	NIMA related kinase 4 [Source:HGNC Symbol;Acc:HGNC:11399]	-2.0	-2
4591	*IL6*	3569	interleukin 6 [Source:HGNC Symbol;Acc:HGNC:6018]	-2.0	-2
4592	*FAM83H*	286077	family with sequence similarity 83 member H [Source:HGNC Symbol;Acc:HGNC:24797]	-2.0	-2
4593	*SC5D*	6309	sterol-C5-desaturase [Source:HGNC Symbol;Acc:HGNC:10547]	-2.0	-2
4594	*ELANE*	1991	elastase, neutrophil expressed [Source:HGNC Symbol;Acc:HGNC:3309]	-2.0	-2
4595	*FBXL13*	222235	F-box and leucine rich repeat protein 13 [Source:HGNC Symbol;Acc:HGNC:21658]	-2.0	-2
4596	*SCN3B*	55800	sodium voltage-gated channel beta subunit 3 [Source:HGNC Symbol;Acc:HGNC:20665]	-2.0	-2
4597	*RNF126*	55658	ring finger protein 126 [Source:HGNC Symbol;Acc:HGNC:21151]	-2.0	-2
4598	*ALG5*	29880	ALG5, dolichyl-phosphate beta-glucosyltransferase [Source:HGNC Symbol;Acc:HGNC:20266]	-2.0	-2
4599	*SLC16A3*	9123	solute carrier family 16 member 3 [Source:HGNC Symbol;Acc:HGNC:10924]	-2.0	-2
4600	*HLA-DRA*	3122	major histocompatibility complex, class II, DR alpha [Source:HGNC Symbol;Acc:HGNC:4947]	-2.0	-2
4601	*FNDC1*	84624	fibronectin type III domain containing 1 [Source:HGNC Symbol;Acc:HGNC:21184]	-2.0	-2
4602	*NAT10*	55226	N-acetyltransferase 10 [Source:HGNC Symbol;Acc:HGNC:29830]	-2.0	-2
4603	*SYNE1*	23345	spectrin repeat containing nuclear envelope protein 1 [Source:HGNC Symbol;Acc:HGNC:17089]	-2.0	-2
4604	*RUNX1T1*	862	RUNX1 translocation partner 1 [Source:HGNC Symbol;Acc:HGNC:1535]	-2.0	-2

4605	*KATNBL1*	79768	katanin regulatory subunit B1 like 1 [Source:HGNC Symbol;Acc:HGNC:26199]	-2.0	-2
4606	*FAM151A*	338094	family with sequence similarity 151 member A [Source:HGNC Symbol;Acc:HGNC:25032]	-2.0	-2
4607	*EXOSC2*	23404	exosome component 2 [Source:HGNC Symbol;Acc:HGNC:17097]	-2.0	-2
4608	*COMMD3*	23412	COMM domain containing 3 [Source:HGNC Symbol;Acc:HGNC:23332]	-2.0	-2
4609	*ACSBG2*	81616	acyl-CoA synthetase bubblegum family member 2 [Source:HGNC Symbol;Acc:HGNC:24174]	-2.0	-2
4610	*OSBPL9*	114883	oxysterol binding protein like 9 [Source:HGNC Symbol;Acc:HGNC:16386]	-2.0	-2
4611	*SRP9*	6726	signal recognition particle 9kDa [Source:HGNC Symbol;Acc:HGNC:11304]	-2.0	-2
4612	*NOS2*	4843	nitric oxide synthase 2 [Source:HGNC Symbol;Acc:HGNC:7873]	-2.0	-2
4613	*RHEBL1*	121268	Ras homolog enriched in brain like 1 [Source:HGNC Symbol;Acc:HGNC:21166]	-2.0	-2
4614	*HGF*	3082	hepatocyte growth factor [Source:HGNC Symbol;Acc:HGNC:4893]	-2.0	-2
4615	*RANBP17*	64901	RAN binding protein 17 [Source:HGNC Symbol;Acc:HGNC:14428]	-2.0	-2
4616	*MRPL52*	122704	mitochondrial ribosomal protein L52 [Source:HGNC Symbol;Acc:HGNC:16655]	-2.0	-2
4617	*BEX4*	56271	brain expressed X-linked 4 [Source:HGNC Symbol;Acc:HGNC:25475]	-2.0	-2
4618	*PLEKHA6*	22874	pleckstrin homology domain containing A6 [Source:HGNC Symbol;Acc:HGNC:17053]	-2.0	-2
4619	*NUPR1L*	389493	Nuclear Protein 2, Transcriptional Regulator	-2.0	-2
4620	*SH2D4B*	387694	SH2 domain containing 4B [Source:HGNC Symbol;Acc:HGNC:31440]	-2.0	-2
4621	*SENP7*	57337	SUMO1/sentrin specific peptidase 7 [Source:HGNC Symbol;Acc:HGNC:30402]	-2.0	-2
4622	*PPP1R2*	5504	protein phosphatase 1 regulatory inhibitor subunit 2 [Source:HGNC Symbol;Acc:HGNC:9288]	-2.0	-2
4623	*GAGE10*	102724473	G antigen 10 [Source:HGNC Symbol;Acc:HGNC:30968]	-2.0	-2
4624	*GCC2*	9648	GRIP and coiled-coil domain containing 2 [Source:HGNC Symbol;Acc:HGNC:23218]	-2.0	-2
4625	*GTF2H1*	2965	general transcription factor IIH subunit 1 [Source:HGNC Symbol;Acc:HGNC:4655]	-2.0	-2
4626	*FAM126A*	84668	family with sequence similarity 126 member A [Source:HGNC Symbol;Acc:HGNC:24587]	-2.0	-2
4627	*TRAF4*	9618	TNF receptor associated factor 4 [Source:HGNC Symbol;Acc:HGNC:12034]	-2.0	-2
4628	*CCDC126*	90693	coiled-coil domain containing 126 [Source:HGNC Symbol;Acc:HGNC:22398]	-2.0	-2
4629	*MTRNR2L8*	100463486	MT-RNR2-like 8 [Source:HGNC Symbol;Acc:HGNC:37165]	-2.0	-2
4630	*SALL1*	6299	spalt like transcription factor 1 [Source:HGNC Symbol;Acc:HGNC:10524]	-2.0	-2
4631	*SNX12*	29934	sorting nexin 12 [Source:HGNC Symbol;Acc:HGNC:14976]	-2.0	-2
4632	*HNRNPA3*	220988	heterogeneous nuclear ribonucleoprotein A3 [Source:HGNC Symbol;Acc:HGNC:24941]	-2.0	-2
4633	*TNN*	63923	tenascin N [Source:HGNC Symbol;Acc:HGNC:22942]	-2.0	-2
4634	*OSBPL6*	114880	oxysterol binding protein like 6 [Source:HGNC Symbol;Acc:HGNC:16388]	-2.0	-2
4635	*MOV10L1*	54456	Mov10 RISC complex RNA helicase like 1 [Source:HGNC Symbol;Acc:HGNC:7201]	-2.0	-2
4636	*CXCL9*	4283	C-X-C motif chemokine ligand 9 [Source:HGNC Symbol;Acc:HGNC:7098]	-2.0	-2
4637	*C14orf178*	283579	chromosome 14 open reading frame 178 [Source:HGNC Symbol;Acc:HGNC:26385]	-2.0	-2
4638	*GSTM5*	2949	glutathione S-transferase mu 5 [Source:HGNC Symbol;Acc:HGNC:4637]	-2.0	-2
4639	*OTUD6B*	51633	OTU domain containing 6B [Source:HGNC Symbol;Acc:HGNC:24281]	-2.0	-2
4640	*F3*	2152	coagulation factor III, tissue factor [Source:HGNC Symbol;Acc:HGNC:3541]	-2.0	-2
4641	*FAM60A*	58516	family with sequence similarity 60 member A [Source:HGNC Symbol;Acc:HGNC:30702]	-2.0	-2
4642	*GOLIM4*	27333	golgi integral membrane protein 4 [Source:HGNC Symbol;Acc:HGNC:15448]	-2.0	-2
4643	*MAP4K5*	11183	mitogen-activated protein kinase kinase kinase kinase 5 [Source:HGNC Symbol;Acc:HGNC:6867]	-2.0	-2
4644	*STRN3*	29966	striatin 3 [Source:HGNC Symbol;Acc:HGNC:15720]	-2.0	-2
4645	*TYRP1*	7306	tyrosinase related protein 1 [Source:HGNC Symbol;Acc:HGNC:12450]	-2.0	-2
4646	*MAT2A*	4144	methionine adenosyltransferase 2A [Source:HGNC Symbol;Acc:HGNC:6904]	-2.0	-2
4647	*DNAJC2*	27000	DnaJ heat shock protein family (Hsp40) member C2 [Source:HGNC Symbol;Acc:HGNC:13192]	-2.0	-2
4648	*SOX6*	55553	SRY-box 6 [Source:HGNC Symbol;Acc:HGNC:16421]	-2.0	-2
4649	*AHNAK*	79026	AHNAK nucleoprotein [Source:HGNC Symbol;Acc:HGNC:347]	-2.0	-2
4650	*STOX2*	56977	storkhead box 2 [Source:HGNC Symbol;Acc:HGNC:25450]	-2.0	-2
4651	*KIAA1755*	85449	KIAA1755 [Source:HGNC Symbol;Acc:HGNC:29372]	-2.0	-2
4652	*BMP1*	649	bone morphogenetic protein 1 [Source:HGNC Symbol;Acc:HGNC:1067]	-2.0	-2
4653	*GNPNAT1*	64841	glucosamine-phosphate N-acetyltransferase 1 [Source:HGNC Symbol;Acc:HGNC:19980]	-2.0	-2
4654	*CTBP1*	1487	C-terminal binding protein 1 [Source:HGNC Symbol;Acc:HGNC:2494]	-2.0	-2
4655	*RBBP8*	5932	RB binding protein 8, endonuclease [Source:HGNC Symbol;Acc:HGNC:9891]	-2.0	-2
4656	*TRADD*	8717	TNFRSF1A associated via death domain [Source:HGNC Symbol;Acc:HGNC:12030]	-2.0	-2
4657	*RBMS1*	5937	RNA binding motif single stranded interacting protein 1 [Source:HGNC Symbol;Acc:HGNC:9907]	-2.0	-2
4658	*CNPY2*	10330	canopy FGF signaling regulator 2 [Source:HGNC Symbol;Acc:HGNC:13529]	-2.0	-2
4659	*EML3*	256364	echinoderm microtubule associated protein like 3 [Source:HGNC Symbol;Acc:HGNC:26666]	-2.0	-2
4660	*SORBS3*	10174	sorbin and SH3 domain containing 3 [Source:HGNC Symbol;Acc:HGNC:30907]	-2.0	-2
4661	*ANKRD50*	57182	ankyrin repeat domain 50 [Source:HGNC Symbol;Acc:HGNC:29223]	-2.0	-2
4662	*BBS9*	27241	Bardet-Biedl syndrome 9 [Source:HGNC Symbol;Acc:HGNC:30000]	-2.0	-2
4663	*RNF141*	50862	ring finger protein 141 [Source:HGNC Symbol;Acc:HGNC:21159]	-2.0	-2
4664	*DHX37*	57647	DEAH-box helicase 37 [Source:HGNC Symbol;Acc:HGNC:17210]	-2.0	-2
4665	*HACD1*	9200	3-hydroxyacyl-CoA dehydratase 1 [Source:HGNC Symbol;Acc:HGNC:9639]	-2.0	-2
4666	*KCNK2*	3776	potassium two pore domain channel subfamily K member 2 [Source:HGNC Symbol;Acc:HGNC:6277]	-2.0	-2
4667	*IL18BP*	10068	interleukin 18 binding protein [Source:HGNC Symbol;Acc:HGNC:5987]	-2.0	-2
4668	*HMGXB4*	10042	HMG-box containing 4 [Source:HGNC Symbol;Acc:HGNC:5003]	-2.0	-2
4669	*KIAA0907*	22889	KIAA0907 [Source:HGNC Symbol;Acc:HGNC:29145]	-2.0	-2
4670	*CACNA2D1*	781	calcium voltage-gated channel auxiliary subunit alpha2delta 1 [Source:HGNC Symbol;Acc:HGNC:1399]	-2.0	-2
4671	*CACNA1C*	775	calcium voltage-gated channel subunit alpha1 C [Source:HGNC Symbol;Acc:HGNC:1390]	-2.0	-2
4672	*ATIC*	471	5-aminoimidazole-4-carboxamide ribonucleotide formyltransferase/IMP cyclohydrolase [Source:HGNC Symbol;Acc:HGNC:794]	-2.1	-2
4673	*TNFRSF11A*	8792	tumor necrosis factor receptor superfamily member 11a [Source:HGNC Symbol;Acc:HGNC:11908]	-2.1	-2
4674	*BNIP3L*	665	BCL2/adenovirus E1B 19kDa interacting protein 3-like [Source:HGNC Symbol;Acc:HGNC:1085]	-2.1	-2
4675	*GIMAP5*	55340	GTPase, IMAP family member 5 [Source:HGNC Symbol;Acc:HGNC:18005]	-2.1	-2
4676	*ME1*	4199	malic enzyme 1 [Source:HGNC Symbol;Acc:HGNC:6983]	-2.1	-2
4677	*KPNA7*	402569	karyopherin subunit alpha 7 [Source:HGNC Symbol;Acc:HGNC:21839]	-2.1	-2

4678	*ZNF517*	340385	zinc finger protein 517 [Source:HGNC Symbol;Acc:HGNC:27984]	-2.1	-2
4679	*TESC*	54997	tescalcin [Source:HGNC Symbol;Acc:HGNC:26065]	-2.1	-2
4680	*GANAB*	23193	glucosidase II alpha subunit [Source:HGNC Symbol;Acc:HGNC:4138]	-2.1	-2
4681	*MGAT4C*	25834	MGAT4 family member C [Source:HGNC Symbol;Acc:HGNC:30871]	-2.1	-2
4682	*CD3EAP*	10849	CD3e molecule associated protein [Source:HGNC Symbol;Acc:HGNC:24219]	-2.1	-2
4683	*ERCC1*	2067	ERCC excision repair 1, endonuclease non-catalytic subunit [Source:HGNC Symbol;Acc:HGNC:3433]	-2.1	-2
4684	*CEP19*	84984	centrosomal protein 19 [Source:HGNC Symbol;Acc:HGNC:28209]	-2.1	-2
4685	*ACACB*	32	acetyl-CoA carboxylase beta [Source:HGNC Symbol;Acc:HGNC:85]	-2.1	-2
4686	*CLSTN3*	9746	calsyntenin 3 [Source:HGNC Symbol;Acc:HGNC:18371]	-2.1	-2
4687	*RAB17*	64284	RAB17, member RAS oncogene family [Source:HGNC Symbol;Acc:HGNC:16523]	-2.1	-2
4688	*ABI3*	51225	ABI family member 3 [Source:HGNC Symbol;Acc:HGNC:29859]	-2.1	-2
4689	*LTA4H*	4048	leukotriene A4 hydrolase [Source:HGNC Symbol;Acc:HGNC:6710]	-2.1	-2
4690	*BCKDK*	10295	branched chain ketoacid dehydrogenase kinase [Source:HGNC Symbol;Acc:HGNC:16902]	-2.1	-2
4691	*ZRANB2*	9406	zinc finger RANBP2-type containing 2 [Source:HGNC Symbol;Acc:HGNC:13058]	-2.1	-2
4692	*NELFCD*	51497	negative elongation factor complex member C/D [Source:HGNC Symbol;Acc:HGNC:15934]	-2.1	-2
4693	*HLA-DMB*	3109	major histocompatibility complex, class II, DM beta [Source:HGNC Symbol;Acc:HGNC:4935]	-2.1	-2
4694	*ZMYM3*	9203	zinc finger MYM-type containing 3 [Source:HGNC Symbol;Acc:HGNC:13054]	-2.1	-2
4695	*CXCR5*	643	C-X-C motif chemokine receptor 5 [Source:HGNC Symbol;Acc:HGNC:1060]	-2.1	-2
4696	*YBX1*	4904	Y-box binding protein 1 [Source:HGNC Symbol;Acc:HGNC:8014]	-2.1	-2
4697	*CTAG1A*	246100	cancer/testis antigen 1A [Source:HGNC Symbol;Acc:HGNC:24198]	-2.1	-2
4698	*HECA*	51696	hdc homolog, cell cycle regulator [Source:HGNC Symbol;Acc:HGNC:21041]	-2.1	-2
4699	*ICOSLG*	23308	inducible T-cell costimulator ligand [Source:HGNC Symbol;Acc:HGNC:17087]	-2.1	-2
4700	*ZNF674*	641339	zinc finger protein 674 [Source:HGNC Symbol;Acc:HGNC:17625]	-2.1	-2
4701	*PRUNE2*	158471	prune homolog 2 [Source:HGNC Symbol;Acc:HGNC:25209]	-2.1	-2
4702	*MYBPC3*	4607	myosin binding protein C, cardiac [Source:HGNC Symbol;Acc:HGNC:7551]	-2.1	-2
4703	*HSD17B12*	51144	hydroxysteroid 17-beta dehydrogenase 12 [Source:HGNC Symbol;Acc:HGNC:18646]	-2.1	-2
4704	*TMEM176A*	55365	transmembrane protein 176A [Source:HGNC Symbol;Acc:HGNC:24930]	-2.1	-2
4705	*WISP1*	8840	WNT1 inducible signaling pathway protein 1 [Source:HGNC Symbol;Acc:HGNC:12769]	-2.1	-2
4706	*SLCO1A2*	6579	solute carrier organic anion transporter family member 1A2 [Source:HGNC Symbol;Acc:HGNC:10956]	-2.1	-2
4707	*KHDC1L*	100129128	KH Homology Domain Containing 1-Like	-2.1	-2
4708	*ZFR2*	23217	zinc finger RNA binding protein 2 [Source:HGNC Symbol;Acc:HGNC:29189]	-2.1	-2
4709	*ZSCAN9*	7746	zinc finger and SCAN domain containing 9 [Source:HGNC Symbol;Acc:HGNC:12984]	-2.1	-2
4710	*PGLYRP2*	114770	peptidoglycan recognition protein 2 [Source:HGNC Symbol;Acc:HGNC:30013]	-2.1	-2
4711	*MRPS21*	54460	mitochondrial ribosomal protein S21 [Source:HGNC Symbol;Acc:HGNC:14046]	-2.1	-2
4712	*EPHA5*	2044	EPH receptor A5 [Source:HGNC Symbol;Acc:HGNC:3389]	-2.1	-2
4713	*ABCA1*	19	ATP binding cassette subfamily A member 1 [Source:HGNC Symbol;Acc:HGNC:29]	-2.1	-2
4714	*BTNL3*	10917	butyrophilin like 3 [Source:HGNC Symbol;Acc:HGNC:1143]	-2.1	-2
4715	*ZNF619*	285267	zinc finger protein 619 [Source:HGNC Symbol;Acc:HGNC:26910]	-2.1	-2
4716	*CRHR1*	1394	corticotropin releasing hormone receptor 1 [Source:HGNC Symbol;Acc:HGNC:2357]	-2.1	-2
4717	*RIMS2*	9699	regulating synaptic membrane exocytosis 2 [Source:HGNC Symbol;Acc:HGNC:17283]	-2.1	-2
4718	*NDRG1*	10397	N-myc downstream regulated 1 [Source:HGNC Symbol;Acc:HGNC:7679]	-2.1	-2
4719	*IL7R*	3575	interleukin 7 receptor [Source:HGNC Symbol;Acc:HGNC:6024]	-2.1	-2
4720	*NOL11*	25926	nucleolar protein 11 [Source:HGNC Symbol;Acc:HGNC:24557]	-2.1	-2
4721	*ESYT2*	57488	extended synaptotagmin 2 [Source:HGNC Symbol;Acc:HGNC:22211]	-2.1	-2
4722	*ARSD*	414	arylsulfatase D [Source:HGNC Symbol;Acc:HGNC:717]	-2.1	-2
4723	*HOXD1*	3231	homeobox D1 [Source:HGNC Symbol;Acc:HGNC:5132]	-2.1	-2
4724	*TXNL4B*	54957	thioredoxin like 4B [Source:HGNC Symbol;Acc:HGNC:26041]	-2.1	-2
4725	*PAIP1*	10605	poly(A) binding protein interacting protein 1 [Source:HGNC Symbol;Acc:HGNC:16945]	-2.1	-2
4726	*MGST1*	4257	microsomal glutathione S-transferase 1 [Source:HGNC Symbol;Acc:HGNC:7061]	-2.1	-2
4727	*MTPN*	136319	myotrophin [Source:HGNC Symbol;Acc:HGNC:15667]	-2.1	-2
4728	*LDOC1L*	84247	leucine zipper, down-regulated in cancer 1-like [Source:HGNC Symbol;Acc:HGNC:13343]	-2.1	-2
4729	*RHBDL2*	54933	rhomboid like 2 [Source:HGNC Symbol;Acc:HGNC:16083]	-2.1	-2
4730	*EVA1A*	84141	eva-1 homolog A, regulator of programmed cell death [Source:HGNC Symbol;Acc:HGNC:25816]	-2.1	-2
4731	*SLC20A2*	6575	solute carrier family 20 member 2 [Source:HGNC Symbol;Acc:HGNC:10947]	-2.1	-2
4732	*ITPRIP*	85450	inositol 1,4,5-trisphosphate receptor interacting protein [Source:HGNC Symbol;Acc:HGNC:29370]	-2.1	-2
4733	*PNISR*	25957	PNN interacting serine and arginine rich protein [Source:HGNC Symbol;Acc:HGNC:21222]	-2.1	-2
4734	*CATSPER2*	117155	cation channel sperm associated 2 [Source:HGNC Symbol;Acc:HGNC:18810]	-2.1	-2
4735	*BLMH*	642	bleomycin hydrolase [Source:HGNC Symbol;Acc:HGNC:1059]	-2.1	-2
4736	*BLCAP*	10904	bladder cancer associated protein [Source:HGNC Symbol;Acc:HGNC:1055]	-2.1	-2
4737	*DBX2*	440097	developing brain homeobox 2 [Source:HGNC Symbol;Acc:HGNC:33186]	-2.1	-2
4738	*DOCK9*	23348	dedicator of cytokinesis 9 [Source:HGNC Symbol;Acc:HGNC:14132]	-2.1	-2
4739	*CBX5*	23468	chromobox 5 [Source:HGNC Symbol;Acc:HGNC:1555]	-2.1	-2
4740	*DPYD*	1806	dihydropyrimidine dehydrogenase [Source:HGNC Symbol;Acc:HGNC:3012]	-2.1	-2
4741	*SORBS1*	10580	sorbin and SH3 domain containing 1 [Source:HGNC Symbol;Acc:HGNC:14565]	-2.1	-2
4742	*ITIH4*	3700	inter-alpha-trypsin inhibitor heavy chain family member 4 [Source:HGNC Symbol;Acc:HGNC:6169]	-2.1	-2
4743	*CCNG2*	901	cyclin G2 [Source:HGNC Symbol;Acc:HGNC:1593]	-2.1	-2
4744	*HSD3B2*	3284	hydroxy-delta-5-steroid dehydrogenase, 3 beta- and steroid delta-isomerase 2 [Source:HGNC Symbol;Acc:HGNC:5218]	-2.1	-2
4745	*ZCCHC4*	29063	zinc finger CCHC-type containing 4 [Source:HGNC Symbol;Acc:HGNC:22917]	-2.1	-2
4746	*PPP1R14B*	26472	protein phosphatase 1 regulatory inhibitor subunit 14B [Source:HGNC Symbol;Acc:HGNC:9057]	-2.1	-2
4747	*CLNK*	116449	cytokine dependent hematopoietic cell linker [Source:HGNC Symbol;Acc:HGNC:17438]	-2.1	-2
4748	*ZNF718*	255403	zinc finger protein 718 [Source:HGNC Symbol;Acc:HGNC:26889]	-2.1	-2
4749	*ADCY1*	107	adenylate cyclase 1 (brain) [Source:HGNC Symbol;Acc:HGNC:232]	-2.1	-2
4750	*SYNPO2L*	79933	synaptopodin 2 like [Source:HGNC Symbol;Acc:HGNC:23532]	-2.1	-2
4751	*PRSS55*	203074	protease, serine 55 [Source:HGNC Symbol;Acc:HGNC:30824]	-2.1	-2
4752	*CCM2L*	140706	CCM2 like scaffolding protein [Source:HGNC Symbol;Acc:HGNC:16153]	-2.1	-2

4753	*DNAJC10*	54431	DnaJ heat shock protein family (Hsp40) member C10 [Source:HGNC Symbol;Acc:HGNC:24637]	-2.1	-2
4754	*NUPR1*	26471	nuclear protein 1, transcriptional regulator [Source:HGNC Symbol;Acc:HGNC:29990]	-2.1	-2
4755	*MYO5A*	4644	myosin VA [Source:HGNC Symbol;Acc:HGNC:7602]	-2.1	-2
4756	*C18orf32*	497661	chromosome 18 open reading frame 32 [Source:HGNC Symbol;Acc:HGNC:31690]	-2.1	-2
4757	*PDE4DIP*	9659	phosphodiesterase 4D interacting protein [Source:HGNC Symbol;Acc:HGNC:15580]	-2.1	-2
4758	*PLAC8L1*	153770	PLAC8-like 1 [Source:HGNC Symbol;Acc:HGNC:31746]	-2.1	-2
4759	*FBXO15*	201456	F-box protein 15 [Source:HGNC Symbol;Acc:HGNC:13617]	-2.1	-2
4760	*STK33*	65975	serine/threonine kinase 33 [Source:HGNC Symbol;Acc:HGNC:14568]	-2.1	-2
4761	*PIK3AP1*	118788	phosphoinositide-3-kinase adaptor protein 1 [Source:HGNC Symbol;Acc:HGNC:30034]	-2.1	-2
4762	*LRRC1*	55227	leucine rich repeat containing 1 [Source:HGNC Symbol;Acc:HGNC:14307]	-2.1	-2
4763	*TMEM120B*	144404	transmembrane protein 120B [Source:HGNC Symbol;Acc:HGNC:32008]	-2.1	-2
4764	*SLC25A30*	253512	solute carrier family 25 member 30 [Source:HGNC Symbol;Acc:HGNC:27371]	-2.1	-2
4765	*GAA*	2548	glucosidase alpha, acid [Source:HGNC Symbol;Acc:HGNC:4065]	-2.1	-2
4766	*BATF2*	116071	basic leucine zipper ATF-like transcription factor 2 [Source:HGNC Symbol;Acc:HGNC:25163]	-2.1	-2
4767	*NME3*	4832	NME/NM23 nucleoside diphosphate kinase 3 [Source:HGNC Symbol;Acc:HGNC:7851]	-2.1	-2
4768	*CD19*	930	CD19 molecule [Source:HGNC Symbol;Acc:HGNC:1633]	-2.1	-2
4769	*RPS4X*	6191	ribosomal protein S4, X-linked [Source:HGNC Symbol;Acc:HGNC:10424]	-2.1	-2
4770	*SCD*	6319	stearoyl-CoA desaturase [Source:HGNC Symbol;Acc:HGNC:10571]	-2.1	-2
4771	*SFRP1*	6422	secreted frizzled related protein 1 [Source:HGNC Symbol;Acc:HGNC:10776]	-2.1	-2
4772	*ODF2L*	57489	outer dense fiber of sperm tails 2 like [Source:HGNC Symbol;Acc:HGNC:29225]	-2.1	-2
4773	*RTN3*	10313	reticulon 3 [Source:HGNC Symbol;Acc:HGNC:10469]	-2.1	-2
4774	*ZNF528*	84436	zinc finger protein 528 [Source:HGNC Symbol;Acc:HGNC:29384]	-2.1	-2
4775	*IGBP1*	3476	immunoglobulin (CD79A) binding protein 1 [Source:HGNC Symbol;Acc:HGNC:5461]	-2.1	-2
4776	*STON1-GTF2A1L*	286749	STON1-GTF2A1L readthrough [Source:HGNC Symbol;Acc:HGNC:30651]	-2.1	-2
4777	*GPR37L1*	9283	G protein-coupled receptor 37 like 1 [Source:HGNC Symbol;Acc:HGNC:14923]	-2.1	-2
4778	*ALS2CR11*	151254	amyotrophic lateral sclerosis 2 chromosome region candidate 11 [Source:HGNC Symbol;Acc:HGNC:14438]	-2.1	-2
4779	*GPS1*	2873	G protein pathway suppressor 1 [Source:HGNC Symbol;Acc:HGNC:4549]	-2.1	-2
4780	*GRIK3*	2899	glutamate ionotropic receptor kainate type subunit 3 [Source:HGNC Symbol;Acc:HGNC:4581]	-2.1	-2
4781	*COA4*	51287	cytochrome c oxidase assembly factor 4 homolog [Source:HGNC Symbol;Acc:HGNC:24604]	-2.1	-2
4782	*ZMYND11*	10771	zinc finger MYND-type containing 11 [Source:HGNC Symbol;Acc:HGNC:16966]	-2.1	-2
4783	*MAP1LC3C*	440738	microtubule associated protein 1 light chain 3 gamma [Source:HGNC Symbol;Acc:HGNC:13353]	-2.1	-2
4784	*CLEC19A*	728276	C-type lectin domain family 19 member A [Source:HGNC Symbol;Acc:HGNC:34522]	-2.1	-2
4785	*ERCC2*	2068	ERCC excision repair 2, TFIIH core complex helicase subunit [Source:HGNC Symbol;Acc:HGNC:3434]	-2.1	-2
4786	*MOGAT3*	346606	monoacylglycerol O-acyltransferase 3 [Source:HGNC Symbol;Acc:HGNC:23249]	-2.1	-2
4787	*CYB5R2*	51700	cytochrome b5 reductase 2 [Source:HGNC Symbol;Acc:HGNC:24376]	-2.1	-2
4788	*GGA2*	23062	golgi associated, gamma adaptin ear containing, ARF binding protein 2 [Source:HGNC Symbol;Acc:HGNC:16064]	-2.1	-2
4789	*C2CD4A*	145741	C2 calcium dependent domain containing 4A [Source:HGNC Symbol;Acc:HGNC:33627]	-2.1	-2
4790	*TNFRSF10A*	8797	tumor necrosis factor receptor superfamily member 10a [Source:HGNC Symbol;Acc:HGNC:11904]	-2.1	-2
4791	*RAB2A*	5862	RAB2A, member RAS oncogene family [Source:HGNC Symbol;Acc:HGNC:9763]	-2.1	-2
4792	*RC3H2*	54542	ring finger and CCCH-type domains 2 [Source:HGNC Symbol;Acc:HGNC:21461]	-2.1	-2
4793	*IFNGR1*	3459	interferon gamma receptor 1 [Source:HGNC Symbol;Acc:HGNC:5439]	-2.1	-2
4794	*SYNPO2*	171024	synaptopodin 2 [Source:HGNC Symbol;Acc:HGNC:17732]	-2.1	-2
4795	*POP4*	10775	POP4 homolog, ribonuclease P/MRP subunit [Source:HGNC Symbol;Acc:HGNC:30081]	-2.1	-2
4796	*APOBEC3H*	164668	apolipoprotein B mRNA editing enzyme catalytic subunit 3H [Source:HGNC Symbol;Acc:HGNC:24100]	-2.1	-2
4797	*TMEM143*	55260	transmembrane protein 143 [Source:HGNC Symbol;Acc:HGNC:25603]	-2.1	-2
4798	*UTS2*	10911	urotensin 2 [Source:HGNC Symbol;Acc:HGNC:12636]	-2.1	-2
4799	*NPY4R*	5540	neuropeptide Y receptor Y4 [Source:HGNC Symbol;Acc:HGNC:9329]	-2.1	-2
4800	*MRPL47*	57129	mitochondrial ribosomal protein L47 [Source:HGNC Symbol;Acc:HGNC:16652]	-2.1	-2
4801	*MSLN*	10232	mesothelin [Source:HGNC Symbol;Acc:HGNC:7371]	-2.2	-2
4802	*TXLNA*	200081	taxilin alpha [Source:HGNC Symbol;Acc:HGNC:30685]	-2.2	-2
4803	*PIK3IP1*	113791	phosphoinositide-3-kinase interacting protein 1 [Source:HGNC Symbol;Acc:HGNC:24942]	-2.2	-2
4804	*MEF2BNB-MEF2B*	4207	BORCS8-MEF2B Readthrough	-2.2	-2
4805	*PSAT1*	29968	phosphoserine aminotransferase 1 [Source:HGNC Symbol;Acc:HGNC:19129]	-2.2	-2
4806	*LCT*	3938	lactase [Source:HGNC Symbol;Acc:HGNC:6530]	-2.2	-2
4807	*KIAA1468*	57614	KIAA1468 [Source:HGNC Symbol;Acc:HGNC:29289]	-2.2	-2
4808	*CYSTM1*	84418	cysteine rich transmembrane module containing 1 [Source:HGNC Symbol;Acc:HGNC:30239]	-2.2	-2
4809	*NAPSA*	9476	napsin A aspartic peptidase [Source:HGNC Symbol;Acc:HGNC:13395]	-2.2	-2
4810	*TMEM176B*	28959	transmembrane protein 176B [Source:HGNC Symbol;Acc:HGNC:29596]	-2.2	-2
4811	*CNEP1R1*	255919	CTD nuclear envelope phosphatase 1 regulatory subunit 1 [Source:HGNC Symbol;Acc:HGNC:26759]	-2.2	-2
4812	*CELF4*	56853	CUGBP, Elav-like family member 4 [Source:HGNC Symbol;Acc:HGNC:14015]	-2.2	-2
4813	*CD8B*	926	CD8b molecule [Source:HGNC Symbol;Acc:HGNC:1707]	-2.2	-2
4814	*POU2F3*	25833	POU class 2 homeobox 3 [Source:HGNC Symbol;Acc:HGNC:19864]	-2.2	-2
4815	*CA5A*	763	carbonic anhydrase 5A [Source:HGNC Symbol;Acc:HGNC:1377]	-2.2	-2
4816	*CLN5*	1203	ceroid-lipofuscinosis, neuronal 5 [Source:HGNC Symbol;Acc:HGNC:2076]	-2.2	-2
4817	*SORBS2*	8470	sorbin and SH3 domain containing 2 [Source:HGNC Symbol;Acc:HGNC:24098]	-2.2	-2
4818	*ZNF212*	7988	zinc finger protein 212 [Source:HGNC Symbol;Acc:HGNC:13004]	-2.2	-2
4819	*AES*	166	amino-terminal enhancer of split [Source:HGNC Symbol;Acc:HGNC:307]	-2.2	-2
4820	*GSTP1*	2950	glutathione S-transferase pi 1 [Source:HGNC Symbol;Acc:HGNC:4638]	-2.2	-2
4821	*PGK1*	5230	phosphoglycerate kinase 1 [Source:HGNC Symbol;Acc:HGNC:8896]	-2.2	-2
4822	*CASC4*	113201	cancer susceptibility candidate 4 [Source:HGNC Symbol;Acc:HGNC:24892]	-2.2	-2
4823	*NIPBL*	25836	NIPBL, cohesin loading factor [Source:HGNC Symbol;Acc:HGNC:28862]	-2.2	-2

4824	*MLLT11*	10962	myeloid/lymphoid or mixed-lineage leukemia; translocated to, 11 [Source:HGNC Symbol;Acc:HGNC:16997]	-2.2	-2
4825	*ATRAID*	51374	all-trans retinoic acid induced differentiation factor [Source:HGNC Symbol;Acc:HGNC:24090]	-2.2	-2
4826	*MESP2*	145873	mesoderm posterior bHLH transcription factor 2 [Source:HGNC Symbol;Acc:HGNC:29659]	-2.2	-2
4827	*NLRP5*	126206	NLR family pyrin domain containing 5 [Source:HGNC Symbol;Acc:HGNC:21269]	-2.2	-2
4828	*SEPT3*	55964	septin 3 [Source:HGNC Symbol;Acc:HGNC:10750]	-2.2	-2
4829	*CDKN2C*	1031	cyclin dependent kinase inhibitor 2C [Source:HGNC Symbol;Acc:HGNC:1789]	-2.2	-2
4830	*GOLT1B*	51026	golgi transport 1B [Source:HGNC Symbol;Acc:HGNC:20175]	-2.2	-2
4831	*FBXO28*	23219	F-box protein 28 [Source:HGNC Symbol;Acc:HGNC:29046]	-2.2	-2
4832	*CYP17A1*	1586	cytochrome P450 family 17 subfamily A member 1 [Source:HGNC Symbol;Acc:HGNC:2593]	-2.2	-2
4833	*AZIN2*	113451	antizyme inhibitor 2 [Source:HGNC Symbol;Acc:HGNC:29957]	-2.2	-2
4834	*SRFBP1*	153443	serum response factor binding protein 1 [Source:HGNC Symbol;Acc:HGNC:26333]	-2.2	-2
4835	*HEYL*	26508	hes related family bHLH transcription factor with YRPW motif-like [Source:HGNC Symbol;Acc:HGNC:4882]	-2.2	-2
4836	*SAMD4A*	23034	sterile alpha motif domain containing 4A [Source:HGNC Symbol;Acc:HGNC:23023]	-2.2	-2
4837	*CD79B*	974	CD79b molecule [Source:HGNC Symbol;Acc:HGNC:1699]	-2.2	-2
4838	*AKAP7*	9465	A-kinase anchoring protein 7 [Source:HGNC Symbol;Acc:HGNC:377]	-2.2	-2
4839	*METRN*	79006	meteorin, glial cell differentiation regulator [Source:HGNC Symbol;Acc:HGNC:14151]	-2.2	-2
4840	*RPP25*	54913	ribonuclease P/MRP subunit p25 [Source:HGNC Symbol;Acc:HGNC:30361]	-2.2	-2
4841	*ACTA2*	59	actin, alpha 2, smooth muscle, aorta [Source:HGNC Symbol;Acc:HGNC:130]	-2.2	-2
4842	*BCKDHB*	594	branched chain keto acid dehydrogenase E1, beta polypeptide [Source:HGNC Symbol;Acc:HGNC:987]	-2.2	-2
4843	*SERPINB9*	5272	serpin family B member 9 [Source:HGNC Symbol;Acc:HGNC:8955]	-2.2	-2
4844	*C4orf50*	389197	chromosome 4 open reading frame 50 [Source:HGNC Symbol;Acc:HGNC:33766]	-2.2	-2
4845	*OTOP1*	133060	otopetrin 1 [Source:HGNC Symbol;Acc:HGNC:19656]	-2.2	-2
4846	*CPN1*	1369	carboxypeptidase N subunit 1 [Source:HGNC Symbol;Acc:HGNC:2312]	-2.2	-2
4847	*APBB2*	323	amyloid beta precursor protein binding family B member 2 [Source:HGNC Symbol;Acc:HGNC:582]	-2.2	-2
4848	*BCKDHA*	593	branched chain keto acid dehydrogenase E1, alpha polypeptide [Source:HGNC Symbol;Acc:HGNC:986]	-2.2	-2
4849	*FAM228B*	375190	family with sequence similarity 228 member B [Source:HGNC Symbol;Acc:HGNC:24736]	-2.2	-2
4850	*FIBIN*	387758	fin bud initiation factor homolog (zebrafish) [Source:HGNC Symbol;Acc:HGNC:33747]	-2.2	-2
4851	*ZNF253*	56242	zinc finger protein 253 [Source:HGNC Symbol;Acc:HGNC:13497]	-2.2	-2
4852	*RGSL1*	353299	regulator of G-protein signaling like 1 [Source:HGNC Symbol;Acc:HGNC:18636]	-2.2	-2
4853	*CACNG7*	59284	calcium voltage-gated channel auxiliary subunit gamma 7 [Source:HGNC Symbol;Acc:HGNC:13626]	-2.2	-2
4854	*HSD17B10*	3028	hydroxysteroid 17-beta dehydrogenase 10 [Source:HGNC Symbol;Acc:HGNC:4800]	-2.2	-2
4855	*TDRD12*	91646	tudor domain containing 12 [Source:HGNC Symbol;Acc:HGNC:25044]	-2.2	-2
4856	*OR4S1*	256148	olfactory receptor family 4 subfamily S member 1 [Source:HGNC Symbol;Acc:HGNC:14705]	-2.2	-2
4857	*PADI3*	51702	peptidyl arginine deiminase 3 [Source:HGNC Symbol;Acc:HGNC:18337]	-2.2	-2
4858	*DCAF16*	54876	DDB1 and CUL4 associated factor 16 [Source:HGNC Symbol;Acc:HGNC:25987]	-2.2	-2
4859	*MST1*	4485	macrophage stimulating 1 [Source:HGNC Symbol;Acc:HGNC:7380]	-2.2	-2
4860	*QSER1*	79832	glutamine and serine rich 1 [Source:HGNC Symbol;Acc:HGNC:26154]	-2.2	-2
4861	*CDC42BPG*	55561	CDC42 binding protein kinase gamma [Source:HGNC Symbol;Acc:HGNC:29829]	-2.2	-2
4862	*ANKS6*	203286	ankyrin repeat and sterile alpha motif domain containing 6 [Source:HGNC Symbol;Acc:HGNC:26724]	-2.2	-2
4863	*FAM214A*	56204	family with sequence similarity 214 member A [Source:HGNC Symbol;Acc:HGNC:25609]	-2.2	-2
4864	*KRT81*	3887	keratin 81 [Source:HGNC Symbol;Acc:HGNC:6458]	-2.2	-2
4865	*PDE4D*	5144	phosphodiesterase 4D [Source:HGNC Symbol;Acc:HGNC:8783]	-2.2	-2
4866	*ZNF629*	23361	zinc finger protein 629 [Source:HGNC Symbol;Acc:HGNC:29008]	-2.2	-2
4867	*CCDC40*	55036	coiled-coil domain containing 40 [Source:HGNC Symbol;Acc:HGNC:26090]	-2.2	-2
4868	*CHMP1B*	57132	charged multivesicular body protein 1B [Source:HGNC Symbol;Acc:HGNC:24287]	-2.2	-2
4869	*ZNF215*	7762	zinc finger protein 215 [Source:HGNC Symbol;Acc:HGNC:13007]	-2.2	-2
4870	*TLCD1*	116238	TLC domain containing 1 [Source:HGNC Symbol;Acc:HGNC:25177]	-2.2	-2
4871	*NSMAF*	8439	neutral sphingomyelinase activation associated factor [Source:HGNC Symbol;Acc:HGNC:8017]	-2.2	-2
4872	*KRT71*	112802	keratin 71 [Source:HGNC Symbol;Acc:HGNC:28927]	-2.2	-2
4873	*EPDR1*	54749	ependymin related 1 [Source:HGNC Symbol;Acc:HGNC:17572]	-2.2	-2
4874	*SEPT12*	124404	septin 12 [Source:HGNC Symbol;Acc:HGNC:26348]	-2.2	-2
4875	*C14orf159*	80017	chromosome 14 open reading frame 159 [Source:HGNC Symbol;Acc:HGNC:20498]	-2.2	-2
4876	*EPS8L1*	54869	EPS8 like 1 [Source:HGNC Symbol;Acc:HGNC:21295]	-2.2	-2
4877	*NEB*	4703	nebulin [Source:HGNC Symbol;Acc:HGNC:7720]	-2.2	-2
4878	*PPIH*	10465	peptidylprolyl isomerase H [Source:HGNC Symbol;Acc:HGNC:14651]	-2.2	-2
4879	*SPIN1*	10927	spindlin 1 [Source:HGNC Symbol;Acc:HGNC:11243]	-2.2	-2
4880	*ALCAM*	214	activated leukocyte cell adhesion molecule [Source:HGNC Symbol;Acc:HGNC:400]	-2.2	-2
4881	*PTPN12*	5782	protein tyrosine phosphatase, non-receptor type 12 [Source:HGNC Symbol;Acc:HGNC:9645]	-2.2	-2
4882	*ZC3H11A*	9877	zinc finger CCCH-type containing 11A [Source:HGNC Symbol;Acc:HGNC:29093]	-2.2	-2
4883	*CKMT1A*	548596	creatine kinase, mitochondrial 1A [Source:HGNC Symbol;Acc:HGNC:31736]	-2.2	-2
4884	*VMAC*	400673	vimentin-type intermediate filament associated coiled-coil protein [Source:HGNC Symbol;Acc:HGNC:33803]	-2.2	-2
4885	*LRPPRC*	10128	leucine rich pentatricopeptide repeat containing [Source:HGNC Symbol;Acc:HGNC:15714]	-2.2	-2
4886	*SFXN3*	81855	sideroflexin 3 [Source:HGNC Symbol;Acc:HGNC:16087]	-2.2	-2
4887	*PHLPP2*	23035	PH domain and leucine rich repeat protein phosphatase 2 [Source:HGNC Symbol;Acc:HGNC:29149]	-2.2	-2
4888	*ESCO1*	114799	establishment of sister chromatid cohesion N-acetyltransferase 1 [Source:HGNC Symbol;Acc:HGNC:24645]	-2.2	-2
4889	*ZNF565*	147929	zinc finger protein 565 [Source:HGNC Symbol;Acc:HGNC:26726]	-2.2	-2
4890	*IFNLR1*	163702	interferon lambda receptor 1 [Source:HGNC Symbol;Acc:HGNC:18584]	-2.2	-2
4891	*BORA*	79866	bora, aurora kinase A activator [Source:HGNC Symbol;Acc:HGNC:24724]	-2.2	-2

4892	*PRKCSH*	5589	protein kinase C substrate 80K-H [Source:HGNC Symbol;Acc:HGNC:9411]	-2.2	-2
4893	*SLC25A12*	8604	solute carrier family 25 member 12 [Source:HGNC Symbol;Acc:HGNC:10982]	-2.2	-2
4894	*ZNF362*	149076	zinc finger protein 362 [Source:HGNC Symbol;Acc:HGNC:18079]	-2.2	-2
4895	*C9orf66*	157983	chromosome 9 open reading frame 66 [Source:HGNC Symbol;Acc:HGNC:26436]	-2.2	-2
4896	*NPHS1*	4868	NPHS1 nephrin [Source:HGNC Symbol;Acc:HGNC:7908]	-2.2	-2
4897	*ARHGEF10L*	55160	Rho guanine nucleotide exchange factor 10 like [Source:HGNC Symbol;Acc:HGNC:25540]	-2.2	-2
4898	*UQCRQ*	27089	ubiquinol-cytochrome c reductase complex III subunit VII [Source:HGNC Symbol;Acc:HGNC:29594]	-2.2	-2
4899	*FAAH2*	158584	fatty acid amide hydrolase 2 [Source:HGNC Symbol;Acc:HGNC:26440]	-2.2	-2
4900	*SLC25A34*	284723	solute carrier family 25 member 34 [Source:HGNC Symbol;Acc:HGNC:27653]	-2.2	-2
4901	*PDE6D*	5147	phosphodiesterase 6D [Source:HGNC Symbol;Acc:HGNC:8788]	-2.2	-2
4902	*IRF4*	3662	interferon regulatory factor 4 [Source:HGNC Symbol;Acc:HGNC:6119]	-2.2	-2
4903	*GUCA1A*	2978	guanylate cyclase activator 1A [Source:HGNC Symbol;Acc:HGNC:4678]	-2.2	-2
4904	*ACIN1*	22985	apoptotic chromatin condensation inducer 1 [Source:HGNC Symbol;Acc:HGNC:17066]	-2.2	-2
4905	*PARM1*	25849	prostate androgen-regulated mucin-like protein 1 [Source:HGNC Symbol;Acc:HGNC:24536]	-2.2	-2
4906	*TMEM254*	80195	transmembrane protein 254 [Source:HGNC Symbol;Acc:HGNC:25804]	-2.2	-2
4907	*CTTNBP2NL*	55917	CTTNBP2 N-terminal like [Source:HGNC Symbol;Acc:HGNC:25330]	-2.2	-2
4908	*TEX264*	51368	testis expressed 264 [Source:HGNC Symbol;Acc:HGNC:30247]	-2.2	-2
4909	*CYP2W1*	54905	cytochrome P450 family 2 subfamily W member 1 [Source:HGNC Symbol;Acc:HGNC:20243]	-2.2	-2
4910	*ZNF208*	7757	zinc finger protein 208 [Source:HGNC Symbol;Acc:HGNC:12999]	-2.2	-2
4911	*FZD1*	8321	frizzled class receptor 1 [Source:HGNC Symbol;Acc:HGNC:4038]	-2.2	-2
4912	*MYOC*	4653	myocilin [Source:HGNC Symbol;Acc:HGNC:7610]	-2.2	-2
4913	*PRR12*	57479	proline rich 12 [Source:HGNC Symbol;Acc:HGNC:29217]	-2.2	-2
4914	*KLK5*	25818	kallikrein related peptidase 5 [Source:HGNC Symbol;Acc:HGNC:6366]	-2.2	-2
4915	*MRPL32*	64983	mitochondrial ribosomal protein L32 [Source:HGNC Symbol;Acc:HGNC:14035]	-2.2	-2
4916	*TMEM51*	55092	transmembrane protein 51 [Source:HGNC Symbol;Acc:HGNC:25488]	-2.2	-2
4917	*ADAL*	161823	adenosine deaminase-like [Source:HGNC Symbol;Acc:HGNC:31853]	-2.2	-2
4918	*RHOA*	387	ras homolog family member A [Source:HGNC Symbol;Acc:HGNC:667]	-2.2	-2
4919	*ANGPT2*	285	angiopoietin 2 [Source:HGNC Symbol;Acc:HGNC:485]	-2.2	-2
4920	*PRELID2*	153768	PRELI domain containing 2 [Source:HGNC Symbol;Acc:HGNC:28306]	-2.3	-2
4921	*OS9*	10956	OS9, endoplasmic reticulum lectin [Source:HGNC Symbol;Acc:HGNC:16994]	-2.3	-2
4922	*ECI1*	1632	enoyl-CoA delta isomerase 1 [Source:HGNC Symbol;Acc:HGNC:2703]	-2.3	-2
4923	*MASP2*	10747	mannan binding lectin serine peptidase 2 [Source:HGNC Symbol;Acc:HGNC:6902]	-2.3	-2
4924	*PNRC1*	10957	proline rich nuclear receptor coactivator 1 [Source:HGNC Symbol;Acc:HGNC:17278]	-2.3	-2
4925	*TMEM156*	80008	transmembrane protein 156 [Source:HGNC Symbol;Acc:HGNC:26260]	-2.3	-2
4926	*PAQR8*	85315	progestin and adipoQ receptor family member 8 [Source:HGNC Symbol;Acc:HGNC:15708]	-2.3	-2
4927	*NOS1*	4842	nitric oxide synthase 1 [Source:HGNC Symbol;Acc:HGNC:7872]	-2.3	-2
4928	*IFNGR2*	3460	interferon gamma receptor 2 (interferon gamma transducer 1) [Source:HGNC Symbol;Acc:HGNC:5440]	-2.3	-2
4929	*C1orf56*	54964	chromosome 1 open reading frame 56 [Source:HGNC Symbol;Acc:HGNC:26045]	-2.3	-2
4930	*TATDN3*	128387	TatD DNase domain containing 3 [Source:HGNC Symbol;Acc:HGNC:27010]	-2.3	-2
4931	*MTERF3*	51001	mitochondrial transcription termination factor 3 [Source:HGNC Symbol;Acc:HGNC:24258]	-2.3	-2
4932	*RAPGEFL1*	51195	Rap guanine nucleotide exchange factor like 1 [Source:HGNC Symbol;Acc:HGNC:17428]	-2.3	-2
4933	*LTBR*	4055	lymphotoxin beta receptor [Source:HGNC Symbol;Acc:HGNC:6718]	-2.3	-2
4934	*OPA1*	4976	OPA1, mitochondrial dynamin like GTPase [Source:HGNC Symbol;Acc:HGNC:8140]	-2.3	-2
4935	*EFR3B*	22979	EFR3 homolog B [Source:HGNC Symbol;Acc:HGNC:29155]	-2.3	-2
4936	*CARD6*	84674	caspase recruitment domain family member 6 [Source:HGNC Symbol;Acc:HGNC:16394]	-2.3	-2
4937	*ATL3*	25923	atlastin GTPase 3 [Source:HGNC Symbol;Acc:HGNC:24526]	-2.3	-2
4938	*DCAF8L2*	347442	DDB1 and CUL4 associated factor 8-like 2 [Source:HGNC Symbol;Acc:HGNC:31811]	-2.3	-2
4939	*GNG7*	2788	G protein subunit gamma 7 [Source:HGNC Symbol;Acc:HGNC:4410]	-2.3	-2
4940	*BNIPL*	149428	BCL2/adenovirus E1B 19kD interacting protein like [Source:HGNC Symbol;Acc:HGNC:16976]	-2.3	-2
4941	*RBM48*	84060	RNA binding motif protein 48 [Source:HGNC Symbol;Acc:HGNC:21785]	-2.3	-2
4942	*JCHAIN*	3512	joining chain of multimeric IgA and IgM [Source:HGNC Symbol;Acc:HGNC:5713]	-2.3	-2
4943	*USP29*	57663	ubiquitin specific peptidase 29 [Source:HGNC Symbol;Acc:HGNC:18563]	-2.3	-2
4944	*SLC8B1*	80024	solute carrier family 8 member B1 [Source:HGNC Symbol;Acc:HGNC:26175]	-2.3	-2
4945	*ZBTB20*	26137	zinc finger and BTB domain containing 20 [Source:HGNC Symbol;Acc:HGNC:13503]	-2.3	-2
4946	*SETD9*	133383	SET domain containing 9 [Source:HGNC Symbol;Acc:HGNC:28508]	-2.3	-2
4947	*CTSK*	1513	cathepsin K [Source:HGNC Symbol;Acc:HGNC:2536]	-2.3	-2
4948	*FAM84A*	151354	family with sequence similarity 84 member A [Source:HGNC Symbol;Acc:HGNC:20743]	-2.3	-2
4949	*SFT2D2*	375035	SFT2 domain containing 2 [Source:HGNC Symbol;Acc:HGNC:25140]	-2.3	-2
4950	*FAM124A*	220108	family with sequence similarity 124 member A [Source:HGNC Symbol;Acc:HGNC:26413]	-2.3	-2
4951	*ZCCHC7*	84186	zinc finger CCHC-type containing 7 [Source:HGNC Symbol;Acc:HGNC:26209]	-2.3	-2
4952	*METTL16*	79066	methyltransferase like 16 [Source:HGNC Symbol;Acc:HGNC:28484]	-2.3	-2
4953	*FRMD8*	83786	FERM domain containing 8 [Source:HGNC Symbol;Acc:HGNC:25462]	-2.3	-2
4954	*SLC16A7*	9194	solute carrier family 16 member 7 [Source:HGNC Symbol;Acc:HGNC:10928]	-2.3	-2
4955	*LPO*	4025	lactoperoxidase [Source:HGNC Symbol;Acc:HGNC:6678]	-2.3	-2
4956	*RNASEL*	6041	ribonuclease L [Source:HGNC Symbol;Acc:HGNC:10050]	-2.3	-2
4957	*KIR3DX1*	90011	killer cell immunoglobulin like receptor, three Ig domains X1 [Source:HGNC Symbol;Acc:HGNC:25043]	-2.3	-2
4958	*TINAGL1*	64129	tubulointerstitial nephritis antigen like 1 [Source:HGNC Symbol;Acc:HGNC:19168]	-2.3	-2
4959	*SDS*	10993	serine dehydratase [Source:HGNC Symbol;Acc:HGNC:10691]	-2.3	-2
4960	*SLC6A20*	54716	solute carrier family 6 member 20 [Source:HGNC Symbol;Acc:HGNC:30927]	-2.3	-2
4961	*PAN2*	9924	PAN2 poly(A) specific ribonuclease subunit [Source:HGNC Symbol;Acc:HGNC:20074]	-2.3	-2
4962	*MXRA5*	25878	matrix remodeling associated 5 [Source:HGNC Symbol;Acc:HGNC:7539]	-2.3	-2
4963	*F2RL2*	2151	coagulation factor II thrombin receptor like 2 [Source:HGNC Symbol;Acc:HGNC:3539]	-2.3	-2
4964	*CD3G*	917	CD3g molecule [Source:HGNC Symbol;Acc:HGNC:1675]	-2.3	-2
4965	*ZNF184*	7738	zinc finger protein 184 [Source:HGNC Symbol;Acc:HGNC:12975]	-2.3	-2
4966	*ZFP30*	22835	ZFP30 zinc finger protein [Source:HGNC Symbol;Acc:HGNC:29555]	-2.3	-2
4967	*COL28A1*	340267	collagen type XXVIII alpha 1 chain [Source:HGNC Symbol;Acc:HGNC:22442]	-2.3	-2
4968	*RAB33B*	83452	RAB33B, member RAS oncogene family [Source:HGNC Symbol;Acc:HGNC:16075]	-2.3	-2
4969	*CRP*	1401	C-reactive protein, pentraxin-related [Source:HGNC Symbol;Acc:HGNC:2367]	-2.3	-2

4970	*SCML2*	10389	sex comb on midleg-like 2 (Drosophila) [Source:HGNC Symbol;Acc:HGNC:10581]	-2.3	-2
4971	*CMTM8*	152189	CKLF like MARVEL transmembrane domain containing 8 [Source:HGNC Symbol;Acc:HGNC:19179]	-2.3	-2
4972	*ATP5J2*	9551	ATP synthase, H+ transporting, mitochondrial Fo complex subunit F2 [Source:HGNC Symbol;Acc:HGNC:848]	-2.3	-2
4973	*AHR*	196	aryl hydrocarbon receptor [Source:HGNC Symbol;Acc:HGNC:348]	-2.3	-2
4974	*CDH23*	64072	cadherin related 23 [Source:HGNC Symbol;Acc:HGNC:13733]	-2.3	-2
4975	*ACSM5*	54988	acyl-CoA synthetase medium-chain family member 5 [Source:HGNC Symbol;Acc:HGNC:26060]	-2.3	-2
4976	*UEVLD*	55293	UEV and lactate/malate dehyrogenase domains [Source:HGNC Symbol;Acc:HGNC:30866]	-2.3	-2
4977	*PNPLA3*	80339	patatin like phospholipase domain containing 3 [Source:HGNC Symbol;Acc:HGNC:18590]	-2.3	-2
4978	*SOS2*	6655	SOS Ras/Rho guanine nucleotide exchange factor 2 [Source:HGNC Symbol;Acc:HGNC:11188]	-2.3	-2
4979	*VEGFB*	7423	vascular endothelial growth factor B [Source:HGNC Symbol;Acc:HGNC:12681]	-2.3	-2
4980	*A1BG*	1	alpha-1-B glycoprotein [Source:HGNC Symbol;Acc:HGNC:5]	-2.3	-2
4981	*GZF1*	64412	GDNF inducible zinc finger protein 1 [Source:HGNC Symbol;Acc:HGNC:15808]	-2.3	-2
4982	*CSPG5*	10675	chondroitin sulfate proteoglycan 5 [Source:HGNC Symbol;Acc:HGNC:2467]	-2.3	-2
4983	*PRIM2*	5558	primase (DNA) subunit 2 [Source:HGNC Symbol;Acc:HGNC:9370]	-2.3	-2
4984	*VPS11*	55823	VPS11, CORVET/HOPS core subunit [Source:HGNC Symbol;Acc:HGNC:14583]	-2.3	-2
4985	*VPS35*	55737	VPS35, retromer complex component [Source:HGNC Symbol;Acc:HGNC:13487]	-2.3	-2
4986	*ENTPD6*	955	ectonucleoside triphosphate diphosphohydrolase 6 (putative) [Source:HGNC Symbol;Acc:HGNC:3368]	-2.3	-2
4987	*TRIM45*	80263	tripartite motif containing 45 [Source:HGNC Symbol;Acc:HGNC:19018]	-2.3	-2
4988	*SESN1*	27244	sestrin 1 [Source:HGNC Symbol;Acc:HGNC:21595]	-2.3	-2
4989	*CD109*	135228	CD109 molecule [Source:HGNC Symbol;Acc:HGNC:21685]	-2.3	-2
4990	*ACTL6A*	86	actin like 6A [Source:HGNC Symbol;Acc:HGNC:24124]	-2.3	-2
4991	*GRIPAP1*	56850	GRIP1 associated protein 1 [Source:HGNC Symbol;Acc:HGNC:18706]	-2.3	-2
4992	*ENTPD3*	956	ectonucleoside triphosphate diphosphohydrolase 3 [Source:HGNC Symbol;Acc:HGNC:3365]	-2.3	-2
4993	*NRDE2*	55051	NRDE-2, necessary for RNA interference, domain containing [Source:HGNC Symbol;Acc:HGNC:20186]	-2.3	-2
4994	*PAGE4*	9506	PAGE family member 4 [Source:HGNC Symbol;Acc:HGNC:4108]	-2.3	-2
4995	*CLEC17A*	388512	C-type lectin domain family 17 member A [Source:HGNC Symbol;Acc:HGNC:34520]	-2.3	-2
4996	*LPIN3*	64900	lipin 3 [Source:HGNC Symbol;Acc:HGNC:14451]	-2.3	-2
4997	*OOEP*	441161	oocyte expressed protein [Source:HGNC Symbol;Acc:HGNC:21382]	-2.3	-2
4998	*MPPED2*	744	metallophosphoesterase domain containing 2 [Source:HGNC Symbol;Acc:HGNC:1180]	-2.3	-2
4999	*ACVR2B*	93	activin A receptor type 2B [Source:HGNC Symbol;Acc:HGNC:174]	-2.3	-2
5000	*MOB2*	81532	MOB kinase activator 2 [Source:HGNC Symbol;Acc:HGNC:24904]	-2.3	-2
5001	*PCNA*	5111	proliferating cell nuclear antigen [Source:HGNC Symbol;Acc:HGNC:8729]	-2.3	-2
5002	*FHIT*	2272	fragile histidine triad [Source:HGNC Symbol;Acc:HGNC:3701]	-2.3	-2
5003	*ACSF2*	80221	acyl-CoA synthetase family member 2 [Source:HGNC Symbol;Acc:HGNC:26101]	-2.3	-2
5004	*NINL*	22981	ninein like [Source:HGNC Symbol;Acc:HGNC:29163]	-2.3	-2
5005	*ZFYVE28*	57732	zinc finger FYVE-type containing 28 [Source:HGNC Symbol;Acc:HGNC:29334]	-2.3	-2
5006	*NBL1*	4681	neuroblastoma 1, DAN family BMP antagonist [Source:HGNC Symbol;Acc:HGNC:7650]	-2.3	-2
5007	*RETSAT*	54884	retinol saturase [Source:HGNC Symbol;Acc:HGNC:25991]	-2.3	-2
5008	*CREBL2*	1389	cAMP responsive element binding protein like 2 [Source:HGNC Symbol;Acc:HGNC:2350]	-2.3	-2
5009	*RPS15A*	6210	ribosomal protein S15a [Source:HGNC Symbol;Acc:HGNC:10389]	-2.3	-2
5010	*SMAD5*	4090	SMAD family member 5 [Source:HGNC Symbol;Acc:HGNC:6771]	-2.3	-2
5011	*ATF7IP2*	80063	activating transcription factor 7 interacting protein 2 [Source:HGNC Symbol;Acc:HGNC:20397]	-2.3	-2
5012	*TMED7*	51014	transmembrane p24 trafficking protein 7 [Source:HGNC Symbol;Acc:HGNC:24253]	-2.3	-2
5013	*FPGT*	8790	fucose-1-phosphate guanylyltransferase [Source:HGNC Symbol;Acc:HGNC:3825]	-2.3	-2
5014	*PTGS2*	5743	prostaglandin-endoperoxide synthase 2 [Source:HGNC Symbol;Acc:HGNC:9605]	-2.3	-2
5015	*ZNF576*	79177	zinc finger protein 576 [Source:HGNC Symbol;Acc:HGNC:28357]	-2.3	-2
5016	*PGLS*	25796	6-phosphogluconolactonase [Source:HGNC Symbol;Acc:HGNC:8903]	-2.3	-2
5017	*CFAP157*	286207	cilia and flagella associated protein 157 [Source:HGNC Symbol;Acc:HGNC:27843]	-2.3	-2
5018	*PTRH1*	138428	peptidyl-tRNA hydrolase 1 homolog [Source:HGNC Symbol;Acc:HGNC:27039]	-2.3	-2
5019	*ST5*	6764	suppression of tumorigenicity 5 [Source:HGNC Symbol;Acc:HGNC:11350]	-2.3	-2
5020	*APOE*	348	apolipoprotein E [Source:HGNC Symbol;Acc:HGNC:613]	-2.3	-2
5021	*NTRK1*	4914	neurotrophic receptor tyrosine kinase 1 [Source:HGNC Symbol;Acc:HGNC:8031]	-2.3	-2
5022	*RNF6*	6049	ring finger protein 6 [Source:HGNC Symbol;Acc:HGNC:10069]	-2.3	-2
5023	*KCTD19*	146212	potassium channel tetramerization domain containing 19 [Source:HGNC Symbol;Acc:HGNC:24753]	-2.3	-2
5024	*FAM155A*	728215	family with sequence similarity 155 member A [Source:HGNC Symbol;Acc:HGNC:33877]	-2.3	-2
5025	*GAGE1*	2543	G antigen 1 [Source:HGNC Symbol;Acc:HGNC:4098]	-2.3	-2
5026	*LCAT*	3931	lecithin-cholesterol acyltransferase [Source:HGNC Symbol;Acc:HGNC:6522]	-2.3	-2
5027	*TCTN2*	79867	tectonic family member 2 [Source:HGNC Symbol;Acc:HGNC:25774]	-2.3	-2
5028	*SAMD9L*	219285	sterile alpha motif domain containing 9 like [Source:HGNC Symbol;Acc:HGNC:1349]	-2.3	-2
5029	*VWA3B*	200403	von Willebrand factor A domain containing 3B [Source:HGNC Symbol;Acc:HGNC:28385]	-2.3	-2
5030	*ZSCAN12*	9753	zinc finger and SCAN domain containing 12 [Source:HGNC Symbol;Acc:HGNC:13172]	-2.3	-2
5031	*SLC22A8*	9376	solute carrier family 22 member 8 [Source:HGNC Symbol;Acc:HGNC:10972]	-2.4	-2
5032	*PSPH*	5723	phosphoserine phosphatase [Source:HGNC Symbol;Acc:HGNC:9577]	-2.4	-2
5033	*CCNL2*	81669	cyclin L2 [Source:HGNC Symbol;Acc:HGNC:20570]	-2.4	-2
5034	*TNFSF4*	7292	tumor necrosis factor superfamily member 4 [Source:HGNC Symbol;Acc:HGNC:11934]	-2.4	-2
5035	*SLC25A14*	9016	solute carrier family 25 member 14 [Source:HGNC Symbol;Acc:HGNC:10984]	-2.4	-2
5036	*NANP*	140838	N-acetylneuraminic acid phosphatase [Source:HGNC Symbol;Acc:HGNC:16140]	-2.4	-2
5037	*COG7*	91949	component of oligomeric golgi complex 7 [Source:HGNC Symbol;Acc:HGNC:18622]	-2.4	-2
5038	*OTUB1*	55611	OTU deubiquitinase, ubiquitin aldehyde binding 1 [Source:HGNC Symbol;Acc:HGNC:23077]	-2.4	-2
5039	*PGM5*	5239	phosphoglucomutase 5 [Source:HGNC Symbol;Acc:HGNC:8908]	-2.4	-2
5040	*LRRC36*	55282	leucine rich repeat containing 36 [Source:HGNC Symbol;Acc:HGNC:25615]	-2.4	-2
5041	*HTRA3*	94031	HtrA serine peptidase 3 [Source:HGNC Symbol;Acc:HGNC:30406]	-2.4	-2
5042	*MRPL1*	65008	mitochondrial ribosomal protein L1 [Source:HGNC Symbol;Acc:HGNC:14275]	-2.4	-2
5043	*KIRREL3*	84623	kin of IRRE like 3 (Drosophila) [Source:HGNC Symbol;Acc:HGNC:23204]	-2.4	-2

5044	*CUL5*	8065	cullin 5 [Source:HGNC Symbol;Acc:HGNC:2556]	-2.4	-2
5045	*RMI2*	116028	RecQ mediated genome instability 2 [Source:HGNC Symbol;Acc:HGNC:28349]	-2.4	-2
5046	*ABCA13*	154664	ATP binding cassette subfamily A member 13 [Source:HGNC Symbol;Acc:HGNC:14638]	-2.4	-2
5047	*MAP7*	9053	microtubule associated protein 7 [Source:HGNC Symbol;Acc:HGNC:6869]	-2.4	-2
5048	*STPG1*	90529	sperm tail PG-rich repeat containing 1 [Source:HGNC Symbol;Acc:HGNC:28070]	-2.4	-2
5049	*RFPL1*	5988	ret finger protein like 1 [Source:HGNC Symbol;Acc:HGNC:9977]	-2.4	-2
5050	*SLC25A42*	284439	solute carrier family 25 member 42 [Source:HGNC Symbol;Acc:HGNC:28380]	-2.4	-2
5051	*OLFML2A*	169611	olfactomedin like 2A [Source:HGNC Symbol;Acc:HGNC:27270]	-2.4	-2
5052	*NLRP8*	126205	NLR family pyrin domain containing 8 [Source:HGNC Symbol;Acc:HGNC:22940]	-2.4	-2
5053	*DYX1C1*	161582	dyslexia susceptibility 1 candidate 1 [Source:HGNC Symbol;Acc:HGNC:21493]	-2.4	-2
5054	*TMEM204*	79652	transmembrane protein 204 [Source:HGNC Symbol;Acc:HGNC:14158]	-2.4	-2
5055	*MRPL21*	219927	mitochondrial ribosomal protein L21 [Source:HGNC Symbol;Acc:HGNC:14479]	-2.4	-2
5056	*ITM2B*	9445	integral membrane protein 2B [Source:HGNC Symbol;Acc:HGNC:6174]	-2.4	-2
5057	*CYBRD1*	79901	cytochrome b reductase 1 [Source:HGNC Symbol;Acc:HGNC:20797]	-2.4	-2
5058	*ZNF853*	54753	zinc finger protein 853 [Source:HGNC Symbol;Acc:HGNC:21767]	-2.4	-2
5059	*AAK1*	22848	AP2 associated kinase 1 [Source:HGNC Symbol;Acc:HGNC:19679]	-2.4	-2
5060	*ANXA3*	306	annexin A3 [Source:HGNC Symbol;Acc:HGNC:541]	-2.4	-2
5061	*SRRT*	51593	serrate, RNA effector molecule [Source:HGNC Symbol;Acc:HGNC:24101]	-2.4	-2
5062	*LRP2*	4036	LDL receptor related protein 2 [Source:HGNC Symbol;Acc:HGNC:6694]	-2.4	-2
5063	*CTNNA1*	1495	catenin alpha 1 [Source:HGNC Symbol;Acc:HGNC:2509]	-2.4	-2
5064	*FAM169B*	283777	family with sequence similarity 169 member B [Source:HGNC Symbol;Acc:HGNC:26835]	-2.4	-2
5065	*ZNF124*	7678	zinc finger protein 124 [Source:HGNC Symbol;Acc:HGNC:12907]	-2.4	-2
5066	*PTGER3*	5733	prostaglandin E receptor 3 [Source:HGNC Symbol;Acc:HGNC:9595]	-2.4	-2
5067	*HSPB11*	51668	heat shock protein family B (small) member 11 [Source:HGNC Symbol;Acc:HGNC:25019]	-2.4	-2
5068	*SPRN*	503542	shadow of prion protein homolog (zebrafish) [Source:HGNC Symbol;Acc:HGNC:16871]	-2.4	-2
5069	*DFNA5*	1687	DFNA5, deafness associated tumor suppressor [Source:HGNC Symbol;Acc:HGNC:2810]	-2.4	-2
5070	*CSMD2*	114784	CUB and Sushi multiple domains 2 [Source:HGNC Symbol;Acc:HGNC:19290]	-2.4	-2
5071	*CDKN2AIPNL*	91368	CDKN2A interacting protein N-terminal like [Source:HGNC Symbol;Acc:HGNC:30545]	-2.4	-2
5072	*FAM159A*	348378	family with sequence similarity 159 member A [Source:HGNC Symbol;Acc:HGNC:28757]	-2.4	-2
5073	*RALGPS1*	9649	Ral GEF with PH domain and SH3 binding motif 1 [Source:HGNC Symbol;Acc:HGNC:16851]	-2.4	-2
5074	*PRDX2*	7001	peroxiredoxin 2 [Source:HGNC Symbol;Acc:HGNC:9353]	-2.4	-2
5075	*COA7*	65260	cytochrome c oxidase assembly factor 7 (putative) [Source:HGNC Symbol;Acc:HGNC:25716]	-2.4	-2
5076	*HDAC9*	9734	histone deacetylase 9 [Source:HGNC Symbol;Acc:HGNC:14065]	-2.4	-2
5077	*ART3*	419	ADP-ribosyltransferase 3 [Source:HGNC Symbol;Acc:HGNC:725]	-2.4	-2
5078	*NFIB*	4781	nuclear factor I B [Source:HGNC Symbol;Acc:HGNC:7785]	-2.4	-2
5079	*TMA16*	55319	translation machinery associated 16 homolog [Source:HGNC Symbol;Acc:HGNC:25638]	-2.4	-2
5080	*HPS3*	84343	HPS3, biogenesis of lysosomal organelles complex 2 subunit 1 [Source:HGNC Symbol;Acc:HGNC:15597]	-2.4	-2
5081	*EPB41L1*	2036	erythrocyte membrane protein band 4.1 like 1 [Source:HGNC Symbol;Acc:HGNC:3378]	-2.4	-2
5082	*ITPR3*	3710	inositol 1,4,5-trisphosphate receptor type 3 [Source:HGNC Symbol;Acc:HGNC:6182]	-2.4	-2
5083	*PKNOX2*	63876	PBX/knotted 1 homeobox 2 [Source:HGNC Symbol;Acc:HGNC:16714]	-2.4	-2
5084	*WDR5B*	54554	WD repeat domain 5B [Source:HGNC Symbol;Acc:HGNC:17826]	-2.4	-2
5085	*NT5E*	4907	5'-nucleotidase ecto [Source:HGNC Symbol;Acc:HGNC:8021]	-2.4	-2
5086	*PSMB5*	5693	proteasome subunit beta 5 [Source:HGNC Symbol;Acc:HGNC:9542]	-2.4	-2
5087	*CTSZ*	1522	cathepsin Z [Source:HGNC Symbol;Acc:HGNC:2547]	-2.4	-2
5088	*A2M*	2	alpha-2-macroglobulin [Source:HGNC Symbol;Acc:HGNC:7]	-2.4	-2
5089	*SSR3*	6747	signal sequence receptor subunit 3 [Source:HGNC Symbol;Acc:HGNC:11325]	-2.4	-2
5090	*ASNSD1*	54529	asparagine synthetase domain containing 1 [Source:HGNC Symbol;Acc:HGNC:24910]	-2.4	-2
5091	*CPVL*	54504	carboxypeptidase, vitellogenic like [Source:HGNC Symbol;Acc:HGNC:14399]	-2.4	-2
5092	*CYP27A1*	1593	cytochrome P450 family 27 subfamily A member 1 [Source:HGNC Symbol;Acc:HGNC:2605]	-2.4	-2
5093	*RFPL2*	10739	ret finger protein like 2 [Source:HGNC Symbol;Acc:HGNC:9979]	-2.4	-2
5094	*DCX*	1641	doublecortin [Source:HGNC Symbol;Acc:HGNC:2714]	-2.4	-2
5095	*CC2D1B*	200014	coiled-coil and C2 domain containing 1B [Source:HGNC Symbol;Acc:HGNC:29386]	-2.4	-2
5096	*ZNF233*	353355	zinc finger protein 233 [Source:HGNC Symbol;Acc:HGNC:30946]	-2.4	-2
5097	*GRK7*	131890	G protein-coupled receptor kinase 7 [Source:HGNC Symbol;Acc:HGNC:17031]	-2.4	-2
5098	*C11orf68*	83638	chromosome 11 open reading frame 68 [Source:HGNC Symbol;Acc:HGNC:28801]	-2.4	-2
5099	*MT1G*	4495	metallothionein 1G [Source:HGNC Symbol;Acc:HGNC:7399]	-2.4	-2
5100	*LIG1*	3978	DNA ligase 1 [Source:HGNC Symbol;Acc:HGNC:6598]	-2.4	-2
5101	*USP53*	54532	ubiquitin specific peptidase 53 [Source:HGNC Symbol;Acc:HGNC:29255]	-2.4	-2
5102	*PMAIP1*	5366	phorbol-12-myristate-13-acetate-induced protein 1 [Source:HGNC Symbol;Acc:HGNC:9108]	-2.4	-2
5103	*RIMS1*	22999	regulating synaptic membrane exocytosis 1 [Source:HGNC Symbol;Acc:HGNC:17282]	-2.4	-2
5104	*TULP2*	7288	tubby like protein 2 [Source:HGNC Symbol;Acc:HGNC:12424]	-2.4	-2
5105	*RRP9*	9136	ribosomal RNA processing 9, small subunit (SSU) processome component, homolog (yeast) [Source:HGNC Symbol;Acc:HGNC:16829]	-2.4	-2
5106	*MOCOS*	55034	molybdenum cofactor sulfurase [Source:HGNC Symbol;Acc:HGNC:18234]	-2.4	-2
5107	*CPQ*	10404	carboxypeptidase Q [Source:HGNC Symbol;Acc:HGNC:16910]	-2.4	-2
5108	*EHBP1*	23301	EH domain binding protein 1 [Source:HGNC Symbol;Acc:HGNC:29144]	-2.4	-2
5109	*SERINC1*	57515	serine incorporator 1 [Source:HGNC Symbol;Acc:HGNC:13464]	-2.4	-2
5110	*IL2RG*	3561	interleukin 2 receptor subunit gamma [Source:HGNC Symbol;Acc:HGNC:6010]	-2.4	-2
5111	*SEL1L3*	23231	SEL1L family member 3 [Source:HGNC Symbol;Acc:HGNC:29108]	-2.4	-2
5112	*GUF1*	60558	GUF1 homolog, GTPase [Source:HGNC Symbol;Acc:HGNC:25799]	-2.4	-2
5113	*COPS8*	10920	COP9 signalosome subunit 8 [Source:HGNC Symbol;Acc:HGNC:24335]	-2.4	-2
5114	*C3orf62*	375341	chromosome 3 open reading frame 62 [Source:HGNC Symbol;Acc:HGNC:24771]	-2.5	-2
5115	*CP*	1356	ceruloplasmin [Source:HGNC Symbol;Acc:HGNC:2295]	-2.5	-2
5116	*ACSL5*	51703	acyl-CoA synthetase long-chain family member 5 [Source:HGNC Symbol;Acc:HGNC:16526]	-2.5	-2
5117	*C20orf24*	55969	chromosome 20 open reading frame 24 [Source:HGNC Symbol;Acc:HGNC:15870]	-2.5	-2
5118	*ISG20*	3669	interferon stimulated exonuclease gene 20 [Source:HGNC Symbol;Acc:HGNC:6130]	-2.5	-2
5119	*NR2F6*	2063	nuclear receptor subfamily 2 group F member 6 [Source:HGNC Symbol;Acc:HGNC:7977]	-2.5	-2
5120	*TBC1D2B*	23102	TBC1 domain family member 2B [Source:HGNC Symbol;Acc:HGNC:29183]	-2.5	-2

5121	*CHCHD2*	51142	coiled-coil-helix-coiled-coil-helix domain containing 2 [Source:HGNC Symbol;Acc:HGNC:21645]	-2.5	-2
5122	*TWF1*	5756	twinfilin actin binding protein 1 [Source:HGNC Symbol;Acc:HGNC:9620]	-2.5	-2
5123	*ARL5A*	26225	ADP ribosylation factor like GTPase 5A [Source:HGNC Symbol;Acc:HGNC:696]	-2.5	-2
5124	*C12orf10*	60314	chromosome 12 open reading frame 10 [Source:HGNC Symbol;Acc:HGNC:17590]	-2.5	-2
5125	*PAAF1*	80227	proteasomal ATPase associated factor 1 [Source:HGNC Symbol;Acc:HGNC:25687]	-2.5	-2
5126	*XKR5*	389610	XK related 5 [Source:HGNC Symbol;Acc:HGNC:20782]	-2.5	-2
5127	*ZNFX1*	57169	zinc finger NFX1-type containing 1 [Source:HGNC Symbol;Acc:HGNC:29271]	-2.5	-2
5128	*PIEZO2*	63895	piezo type mechanosensitive ion channel component 2 [Source:HGNC Symbol;Acc:HGNC:26270]	-2.5	-2
5129	*COL16A1*	1307	collagen type XVI alpha 1 chain [Source:HGNC Symbol;Acc:HGNC:2193]	-2.5	-2
5130	*PSMD3*	5709	proteasome 26S subunit, non-ATPase 3 [Source:HGNC Symbol;Acc:HGNC:9560]	-2.5	-2
5131	*INPP5K*	51763	inositol polyphosphate-5-phosphatase K [Source:HGNC Symbol;Acc:HGNC:33882]	-2.5	-2
5132	*IL17RE*	132014	interleukin 17 receptor E [Source:HGNC Symbol;Acc:HGNC:18439]	-2.5	-2
5133	*DCP1A*	55802	decapping mRNA 1A [Source:HGNC Symbol;Acc:HGNC:18714]	-2.5	-2
5134	*ZG16*	653808	zymogen granule protein 16 [Source:HGNC Symbol;Acc:HGNC:30961]	-2.5	-2
5135	*SFR1*	119392	SWI5 dependent homologous recombination repair protein 1 [Source:HGNC Symbol;Acc:HGNC:29574]	-2.5	-2
5136	*BCL6B*	255877	B-cell CLL/lymphoma 6B [Source:HGNC Symbol;Acc:HGNC:1002]	-2.5	-2
5137	*TMEM116*	89894	transmembrane protein 116 [Source:HGNC Symbol;Acc:HGNC:25084]	-2.5	-2
5138	*ALPP*	250	alkaline phosphatase, placental [Source:HGNC Symbol;Acc:HGNC:439]	-2.5	-2
5139	*WIPF2*	147179	WAS/WASL interacting protein family member 2 [Source:HGNC Symbol;Acc:HGNC:30923]	-2.5	-2
5140	*GREB1*	9687	growth regulation by estrogen in breast cancer 1 [Source:HGNC Symbol;Acc:HGNC:24885]	-2.5	-2
5141	*PHF8*	23133	PHD finger protein 8 [Source:HGNC Symbol;Acc:HGNC:20672]	-2.5	-2
5142	*BTN3A3*	10384	butyrophilin subfamily 3 member A3 [Source:HGNC Symbol;Acc:HGNC:1140]	-2.5	-2
5143	*NFKBIB*	4793	NFKB inhibitor beta [Source:HGNC Symbol;Acc:HGNC:7798]	-2.5	-2
5144	*SIRT2*	22933	sirtuin 2 [Source:HGNC Symbol;Acc:HGNC:10886]	-2.5	-2
5145	*ZNF578*	147660	zinc finger protein 578 [Source:HGNC Symbol;Acc:HGNC:26449]	-2.5	-2
5146	*GGT5*	2687	gamma-glutamyltransferase 5 [Source:HGNC Symbol;Acc:HGNC:4260]	-2.5	-2
5147	*PPP1R3F*	89801	protein phosphatase 1 regulatory subunit 3F [Source:HGNC Symbol;Acc:HGNC:14944]	-2.5	-2
5148	*EEA1*	8411	early endosome antigen 1 [Source:HGNC Symbol;Acc:HGNC:3185]	-2.5	-2
5149	*ZC3HC1*	51530	zinc finger C3HC-type containing 1 [Source:HGNC Symbol;Acc:HGNC:29913]	-2.5	-2
5150	*IFI6*	2537	interferon alpha inducible protein 6 [Source:HGNC Symbol;Acc:HGNC:4054]	-2.5	-2
5151	*NPAT*	4863	nuclear protein, coactivator of histone transcription [Source:HGNC Symbol;Acc:HGNC:7896]	-2.5	-2
5152	*TWSG1*	57045	twisted gastrulation BMP signaling modulator 1 [Source:HGNC Symbol;Acc:HGNC:12429]	-2.5	-2
5153	*ANK3*	288	ankyrin 3, node of Ranvier (ankyrin G) [Source:HGNC Symbol;Acc:HGNC:494]	-2.5	-2
5154	*ODC1*	4953	ornithine decarboxylase 1 [Source:HGNC Symbol;Acc:HGNC:8109]	-2.5	-2
5155	*SLC3A1*	6519	solute carrier family 3 member 1 [Source:HGNC Symbol;Acc:HGNC:11025]	-2.5	-2
5156	*MESP1*	55897	mesoderm posterior bHLH transcription factor 1 [Source:HGNC Symbol;Acc:HGNC:29658]	-2.5	-2
5157	*ZNF417*	147687	zinc finger protein 417 [Source:HGNC Symbol;Acc:HGNC:20646]	-2.5	-2
5158	*POLR2J2*	246721	polymerase (RNA) II subunit J2 [Source:HGNC Symbol;Acc:HGNC:23208]	-2.5	-2
5159	*CD58*	965	CD58 molecule [Source:HGNC Symbol;Acc:HGNC:1688]	-2.5	-2
5160	*CCT3*	7203	chaperonin containing TCP1 subunit 3 [Source:HGNC Symbol;Acc:HGNC:1616]	-2.5	-2
5161	*IKZF5*	64376	IKAROS family zinc finger 5 [Source:HGNC Symbol;Acc:HGNC:14283]	-2.5	-2
5162	*ZNF563*	147837	zinc finger protein 563 [Source:HGNC Symbol;Acc:HGNC:30498]	-2.5	-2
5163	*SALL4*	57167	spalt like transcription factor 4 [Source:HGNC Symbol;Acc:HGNC:15924]	-2.5	-2
5164	*C11orf96*	387763	chromosome 11 open reading frame 96 [Source:HGNC Symbol;Acc:HGNC:38675]	-2.5	-2
5165	*TRMT1L*	81627	tRNA methyltransferase 1 like [Source:HGNC Symbol;Acc:HGNC:16782]	-2.5	-2
5166	*SRCAP*	10847	Snf2-related CREBBP activator protein [Source:HGNC Symbol;Acc:HGNC:16974]	-2.5	-2
5167	*CCDC153*	283152	coiled-coil domain containing 153 [Source:HGNC Symbol;Acc:HGNC:27446]	-2.5	-2
5168	*PAFAH2*	5051	platelet activating factor acetylhydrolase 2 [Source:HGNC Symbol;Acc:HGNC:8579]	-2.5	-2
5169	*NSG1*	27065	Neuron Specific Gene Family Member 1	-2.5	-2
5170	*STX18*	53407	syntaxin 18 [Source:HGNC Symbol;Acc:HGNC:15942]	-2.5	-2
5171	*CHI3L2*	1117	chitinase 3 like 2 [Source:HGNC Symbol;Acc:HGNC:1933]	-2.5	-2
5172	*GDF10*	2662	growth differentiation factor 10 [Source:HGNC Symbol;Acc:HGNC:4215]	-2.5	-2
5173	*RPGRIP1*	57096	retinitis pigmentosa GTPase regulator interacting protein 1 [Source:HGNC Symbol;Acc:HGNC:13436]	-2.5	-2
5174	*ANGPTL6*	83854	angiopoietin like 6 [Source:HGNC Symbol;Acc:HGNC:23140]	-2.5	-2
5175	*C19orf66*	55337	chromosome 19 open reading frame 66 [Source:HGNC Symbol;Acc:HGNC:25649]	-2.5	-2
5176	*C2CD3*	26005	C2 calcium dependent domain containing 3 [Source:HGNC Symbol;Acc:HGNC:24564]	-2.5	-2
5177	*GRIN3B*	116444	glutamate ionotropic receptor NMDA type subunit 3B [Source:HGNC Symbol;Acc:HGNC:16768]	-2.5	-2
5178	*LRAT*	9227	lecithin retinol acyltransferase (phosphatidylcholine--retinol O-acyltransferase) [Source:HGNC Symbol;Acc:HGNC:6685]	-2.5	-2
5179	*CD302*	9936	CD302 molecule [Source:HGNC Symbol;Acc:HGNC:30843]	-2.5	-2
5180	*CTDSPL2*	51496	CTD small phosphatase like 2 [Source:HGNC Symbol;Acc:HGNC:26936]	-2.5	-2
5181	*CRLF3*	51379	cytokine receptor like factor 3 [Source:HGNC Symbol;Acc:HGNC:17177]	-2.5	-2
5182	*UGT8*	7368	UDP glycosyltransferase 8 [Source:HGNC Symbol;Acc:HGNC:12555]	-2.5	-2
5183	*CCDC80*	151887	coiled-coil domain containing 80 [Source:HGNC Symbol;Acc:HGNC:30649]	-2.5	-2
5184	*TRIM16*	10626	tripartite motif containing 16 [Source:HGNC Symbol;Acc:HGNC:17241]	-2.5	-2
5185	*ENDOU*	8909	endonuclease, poly(U) specific [Source:HGNC Symbol;Acc:HGNC:14369]	-2.5	-2
5186	*PLEKHA4*	57664	pleckstrin homology domain containing A4 [Source:HGNC Symbol;Acc:HGNC:14339]	-2.5	-2
5187	*MLF1*	4291	myeloid leukemia factor 1 [Source:HGNC Symbol;Acc:HGNC:7125]	-2.5	-2
5188	*TMEM108*	66000	transmembrane protein 108 [Source:HGNC Symbol;Acc:HGNC:28451]	-2.5	-2
5189	*SEC23A*	10484	Sec23 homolog A, coat complex II component [Source:HGNC Symbol;Acc:HGNC:10701]	-2.5	-2
5190	*MMD2*	221938	monocyte to macrophage differentiation associated 2 [Source:HGNC Symbol;Acc:HGNC:30133]	-2.5	-2
5191	*LAMP1*	3916	lysosomal associated membrane protein 1 [Source:HGNC Symbol;Acc:HGNC:6499]	-2.5	-2
5192	*GGA3*	23163	golgi associated, gamma adaptin ear containing, ARF binding protein 3 [Source:HGNC Symbol;Acc:HGNC:17079]	-2.5	-2

5193	*SLC35E1*	79939	solute carrier family 35 member E1 [Source:HGNC Symbol;Acc:HGNC:20803]	-2.5	-2
5194	*ASF1A*	25842	anti-silencing function 1A histone chaperone [Source:HGNC Symbol;Acc:HGNC:20995]	-2.5	-2
5195	*BACH2*	60468	BTB domain and CNC homolog 2 [Source:HGNC Symbol;Acc:HGNC:14078]	-2.5	-2
5196	*DYNLL2*	140735	dynein light chain LC8-type 2 [Source:HGNC Symbol;Acc:HGNC:24596]	-2.5	-2
5197	*IFITM1*	8519	interferon induced transmembrane protein 1 [Source:HGNC Symbol;Acc:HGNC:5412]	-2.5	-2
5198	*MED7*	9443	mediator complex subunit 7 [Source:HGNC Symbol;Acc:HGNC:2378]	-2.5	-2
5199	*ERLEC1*	27248	endoplasmic reticulum lectin 1 [Source:HGNC Symbol;Acc:HGNC:25222]	-2.5	-2
5200	*VPS13B*	157680	vacuolar protein sorting 13 homolog B [Source:HGNC Symbol;Acc:HGNC:2183]	-2.5	-2
5201	*SEPT7*	989	septin 7 [Source:HGNC Symbol;Acc:HGNC:1717]	-2.5	-2
5202	*SOX7*	83595	SRY-box 7 [Source:HGNC Symbol;Acc:HGNC:18196]	-2.5	-2
5203	*NPAP1*	23742	nuclear pore associated protein 1 [Source:HGNC Symbol;Acc:HGNC:1190]	-2.5	-2
5204	*TMEM45B*	120224	transmembrane protein 45B [Source:HGNC Symbol;Acc:HGNC:25194]	-2.5	-2
5205	*IKBKE*	9641	inhibitor of kappa light polypeptide gene enhancer in B-cells, kinase epsilon [Source:HGNC Symbol;Acc:HGNC:14552]	-2.5	-2
5206	*RAET1E*	135250	retinoic acid early transcript 1E [Source:HGNC Symbol;Acc:HGNC:16793]	-2.5	-2
5207	*ABCA9*	10350	ATP binding cassette subfamily A member 9 [Source:HGNC Symbol;Acc:HGNC:39]	-2.5	-2
5208	*FSTL3*	10272	follistatin like 3 [Source:HGNC Symbol;Acc:HGNC:3973]	-2.5	-2
5209	*FAM210A*	125228	family with sequence similarity 210 member A [Source:HGNC Symbol;Acc:HGNC:28346]	-2.5	-2
5210	*MARK2*	2011	microtubule affinity regulating kinase 2 [Source:HGNC Symbol;Acc:HGNC:3332]	-2.5	-2
5211	*SNX9*	51429	sorting nexin 9 [Source:HGNC Symbol;Acc:HGNC:14973]	-2.5	-2
5212	*PKIB*	5570	protein kinase (cAMP-dependent, catalytic) inhibitor beta [Source:HGNC Symbol;Acc:HGNC:9018]	-2.6	-2
5213	*SLC35F1*	222553	solute carrier family 35 member F1 [Source:HGNC Symbol;Acc:HGNC:21483]	-2.6	-2
5214	*COBL*	23242	cordon-bleu WH2 repeat protein [Source:HGNC Symbol;Acc:HGNC:22199]	-2.6	-2
5215	*CHD1*	1105	chromodomain helicase DNA binding protein 1 [Source:HGNC Symbol;Acc:HGNC:1915]	-2.6	-2
5216	*TMEM56*	148534	transmembrane protein 56 [Source:HGNC Symbol;Acc:HGNC:26477]	-2.6	-2
5217	*TAF6L*	10629	TATA-box binding protein associated factor 6 like [Source:HGNC Symbol;Acc:HGNC:17305]	-2.6	-2
5218	*CEP44*	80817	centrosomal protein 44 [Source:HGNC Symbol;Acc:HGNC:29356]	-2.6	-2
5219	*POFUT1*	23509	protein O-fucosyltransferase 1 [Source:HGNC Symbol;Acc:HGNC:14988]	-2.6	-2
5220	*PKD1L3*	342372	polycystin 1 like 3, transient receptor potential channel interacting [Source:HGNC Symbol;Acc:HGNC:21716]	-2.6	-2
5221	*GSK3A*	2931	glycogen synthase kinase 3 alpha [Source:HGNC Symbol;Acc:HGNC:4616]	-2.6	-2
5222	*ZNF519*	162655	zinc finger protein 519 [Source:HGNC Symbol;Acc:HGNC:30574]	-2.6	-2
5223	*PIGB*	9488	phosphatidylinositol glycan anchor biosynthesis class B [Source:HGNC Symbol;Acc:HGNC:8959]	-2.6	-2
5224	*PPARGC1A*	10891	PPARG coactivator 1 alpha [Source:HGNC Symbol;Acc:HGNC:9237]	-2.6	-2
5225	*GALM*	130589	galactose mutarotase [Source:HGNC Symbol;Acc:HGNC:24063]	-2.6	-2
5226	*HLA-DQB2*	3120	major histocompatibility complex, class II, DQ beta 2 [Source:HGNC Symbol;Acc:HGNC:4945]	-2.6	-2
5227	*BNIP2*	663	BCL2/adenovirus E1B 19kDa interacting protein 2 [Source:HGNC Symbol;Acc:HGNC:1083]	-2.6	-2
5228	*WDR78*	79819	WD repeat domain 78 [Source:HGNC Symbol;Acc:HGNC:26252]	-2.6	-2
5229	*PIKFYVE*	200576	phosphoinositide kinase, FYVE-type zinc finger containing [Source:HGNC Symbol;Acc:HGNC:23785]	-2.6	-2
5230	*LETM2*	137994	leucine zipper and EF-hand containing transmembrane protein 2 [Source:HGNC Symbol;Acc:HGNC:14648]	-2.6	-2
5231	*NREP*	9315	neuronal regeneration related protein [Source:HGNC Symbol;Acc:HGNC:16834]	-2.6	-2
5232	*POU6F1*	5463	POU class 6 homeobox 1 [Source:HGNC Symbol;Acc:HGNC:9224]	-2.6	-2
5233	*SPATA6L*	55064	spermatogenesis associated 6 like [Source:HGNC Symbol;Acc:HGNC:25472]	-2.6	-2
5234	*SLC6A4*	6532	solute carrier family 6 member 4 [Source:HGNC Symbol;Acc:HGNC:11050]	-2.6	-2
5235	*WNT7B*	7477	Wnt family member 7B [Source:HGNC Symbol;Acc:HGNC:12787]	-2.6	-2
5236	*NPC1L1*	29881	NPC1 like intracellular cholesterol transporter 1 [Source:HGNC Symbol;Acc:HGNC:7898]	-2.6	-2
5237	*GAS2*	2620	growth arrest specific 2 [Source:HGNC Symbol;Acc:HGNC:4167]	-2.6	-2
5238	*FAM234B*	57613	family with sequence similarity 234 member B [Source:HGNC Symbol;Acc:HGNC:29288]	-2.6	-2
5239	*LHFP*	10186	lipoma HMGIC fusion partner [Source:HGNC Symbol;Acc:HGNC:6586]	-2.6	-2
5240	*ZNF496*	84838	zinc finger protein 496 [Source:HGNC Symbol;Acc:HGNC:23713]	-2.6	-2
5241	*ABHD18*	80167	abhydrolase domain containing 18 [Source:HGNC Symbol;Acc:HGNC:26111]	-2.6	-2
5242	*CASP4*	837	caspase 4 [Source:HGNC Symbol;Acc:HGNC:1505]	-2.6	-2
5243	*DDR2*	4921	discoidin domain receptor tyrosine kinase 2 [Source:HGNC Symbol;Acc:HGNC:2731]	-2.6	-2
5244	*CCRL2*	9034	C-C motif chemokine receptor like 2 [Source:HGNC Symbol;Acc:HGNC:1612]	-2.6	-2
5245	*GRID1*	2894	glutamate ionotropic receptor delta type subunit 1 [Source:HGNC Symbol;Acc:HGNC:4575]	-2.6	-2
5246	*AARD*	441376	alanine and arginine rich domain containing protein [Source:HGNC Symbol;Acc:HGNC:33842]	-2.6	-2
5247	*LMNTD1*	160492	lamin tail domain containing 1 [Source:HGNC Symbol;Acc:HGNC:26683]	-2.6	-2
5248	*SOCS7*	30837	suppressor of cytokine signaling 7 [Source:HGNC Symbol;Acc:HGNC:29846]	-2.6	-2
5249	*MED26*	9441	mediator complex subunit 26 [Source:HGNC Symbol;Acc:HGNC:2376]	-2.6	-2
5250	*TMEM159*	57146	transmembrane protein 159 [Source:HGNC Symbol;Acc:HGNC:30136]	-2.6	-2
5251	*PRND*	23627	prion protein 2 (dublet) [Source:HGNC Symbol;Acc:HGNC:15748]	-2.6	-2
5252	*ADRA1A*	148	adrenoceptor alpha 1A [Source:HGNC Symbol;Acc:HGNC:277]	-2.6	-2
5253	*OMA1*	115209	OMA1 zinc metallopeptidase [Source:HGNC Symbol;Acc:HGNC:29661]	-2.6	-2
5254	*UBIAD1*	29914	UbiA prenyltransferase domain containing 1 [Source:HGNC Symbol;Acc:HGNC:30791]	-2.6	-2
5255	*CNN1*	1264	calponin 1 [Source:HGNC Symbol;Acc:HGNC:2155]	-2.6	-2
5256	*TSNAXIP1*	55815	translin associated factor X interacting protein 1 [Source:HGNC Symbol;Acc:HGNC:18586]	-2.6	-2
5257	*ENOX2*	10495	ecto-NOX disulfide-thiol exchanger 2 [Source:HGNC Symbol;Acc:HGNC:2259]	-2.6	-2
5258	*DKC1*	1736	dyskerin pseudouridine synthase 1 [Source:HGNC Symbol;Acc:HGNC:2890]	-2.6	-2
5259	*NR4A3*	8013	nuclear receptor subfamily 4 group A member 3 [Source:HGNC Symbol;Acc:HGNC:7982]	-2.6	-2
5260	*RIPK4*	54101	receptor interacting serine/threonine kinase 4 [Source:HGNC Symbol;Acc:HGNC:496]	-2.6	-2
5261	*CXCL3*	2921	C-X-C motif chemokine ligand 3 [Source:HGNC Symbol;Acc:HGNC:4604]	-2.6	-2
5262	*NCAN*	1463	neurocan [Source:HGNC Symbol;Acc:HGNC:2465]	-2.6	-2
5263	*CYP1A1*	1543	cytochrome P450 family 1 subfamily A member 1 [Source:HGNC Symbol;Acc:HGNC:2595]	-2.6	-2

5264	*THRA*	7067	thyroid hormone receptor, alpha [Source:HGNC Symbol;Acc:HGNC:11796]	-2.6	-2
5265	*CEBPG*	1054	CCAAT/enhancer binding protein gamma [Source:HGNC Symbol;Acc:HGNC:1837]	-2.6	-2
5266	*ZGRF1*	55345	zinc finger GRF-type containing 1 [Source:HGNC Symbol;Acc:HGNC:25654]	-2.6	-2
5267	*HRH1*	3269	histamine receptor H1 [Source:HGNC Symbol;Acc:HGNC:5182]	-2.6	-2
5268	*FAM50A*	9130	family with sequence similarity 50 member A [Source:HGNC Symbol;Acc:HGNC:18786]	-2.6	-2
5269	*EPHX4*	253152	epoxide hydrolase 4 [Source:HGNC Symbol;Acc:HGNC:23758]	-2.6	-2
5270	*LRRC27*	80313	leucine rich repeat containing 27 [Source:HGNC Symbol;Acc:HGNC:29346]	-2.6	-2
5271	*RFFL*	117584	ring finger and FYVE-like domain containing E3 ubiquitin protein ligase [Source:HGNC Symbol;Acc:HGNC:24821]	-2.6	-2
5272	*ARL2*	402	ADP ribosylation factor like GTPase 2 [Source:HGNC Symbol;Acc:HGNC:693]	-2.6	-2
5273	*SNX15*	29907	sorting nexin 15 [Source:HGNC Symbol;Acc:HGNC:14978]	-2.6	-2
5274	*C16orf70*	80262	chromosome 16 open reading frame 70 [Source:HGNC Symbol;Acc:HGNC:29564]	-2.6	-2
5275	*YIPF5*	81555	Yip1 domain family member 5 [Source:HGNC Symbol;Acc:HGNC:24877]	-2.6	-2
5276	*SLC4A5*	57835	solute carrier family 4 member 5 [Source:HGNC Symbol;Acc:HGNC:18168]	-2.6	-2
5277	*IL4R*	3566	interleukin 4 receptor [Source:HGNC Symbol;Acc:HGNC:6015]	-2.6	-2
5278	*TRIM65*	201292	tripartite motif containing 65 [Source:HGNC Symbol;Acc:HGNC:27316]	-2.6	-2
5279	*CLCN6*	1185	chloride voltage-gated channel 6 [Source:HGNC Symbol;Acc:HGNC:2024]	-2.6	-2
5280	*FAS*	355	Fas cell surface death receptor [Source:HGNC Symbol;Acc:HGNC:11920]	-2.6	-2
5281	*USP47*	55031	ubiquitin specific peptidase 47 [Source:HGNC Symbol;Acc:HGNC:20076]	-2.6	-2
5282	*MUC21*	394263	mucin 21, cell surface associated [Source:HGNC Symbol;Acc:HGNC:21661]	-2.6	-2
5283	*CASQ1*	844	calsequestrin 1 [Source:HGNC Symbol;Acc:HGNC:1512]	-2.6	-2
5284	*AACS*	65985	acetoacetyl-CoA synthetase [Source:HGNC Symbol;Acc:HGNC:21298]	-2.6	-2
5285	*ZNRF3*	84133	zinc and ring finger 3 [Source:HGNC Symbol;Acc:HGNC:18126]	-2.6	-2
5286	*NUDT14*	256281	nudix hydrolase 14 [Source:HGNC Symbol;Acc:HGNC:20141]	-2.6	-2
5287	*WDR19*	57728	WD repeat domain 19 [Source:HGNC Symbol;Acc:HGNC:18340]	-2.6	-2
5288	*SPRR2A*	6700	small proline rich protein 2A [Source:HGNC Symbol;Acc:HGNC:11261]	-2.6	-2
5289	*PHYHIP*	9796	phytanoyl-CoA 2-hydroxylase interacting protein [Source:HGNC Symbol;Acc:HGNC:16865]	-2.6	-2
5290	*HSD17B8*	7923	hydroxysteroid 17-beta dehydrogenase 8 [Source:HGNC Symbol;Acc:HGNC:3554]	-2.6	-2
5291	*FIS1*	51024	fission, mitochondrial 1 [Source:HGNC Symbol;Acc:HGNC:21689]	-2.6	-2
5292	*MOB3C*	148932	MOB kinase activator 3C [Source:HGNC Symbol;Acc:HGNC:29800]	-2.6	-2
5293	*NEK1*	4750	NIMA related kinase 1 [Source:HGNC Symbol;Acc:HGNC:7744]	-2.6	-2
5294	*SLC36A3*	285641	solute carrier family 36 member 3 [Source:HGNC Symbol;Acc:HGNC:19659]	-2.6	-2
5295	*OPTN*	10133	optineurin [Source:HGNC Symbol;Acc:HGNC:17142]	-2.6	-2
5296	*GPR132*	29933	G protein-coupled receptor 132 [Source:HGNC Symbol;Acc:HGNC:17482]	-2.6	-2
5297	*GPNMB*	10457	glycoprotein nmb [Source:HGNC Symbol;Acc:HGNC:4462]	-2.6	-2
5298	*DCAF11*	80344	DDB1 and CUL4 associated factor 11 [Source:HGNC Symbol;Acc:HGNC:20258]	-2.6	-2
5299	*TRIM59*	286827	tripartite motif containing 59 [Source:HGNC Symbol;Acc:HGNC:30834]	-2.6	-2
5300	*ZNF700*	90592	zinc finger protein 700 [Source:HGNC Symbol;Acc:HGNC:25292]	-2.6	-2
5301	*TFPI2*	7980	tissue factor pathway inhibitor 2 [Source:HGNC Symbol;Acc:HGNC:11761]	-2.6	-2
5302	*SEC22A*	26984	SEC22 homolog A, vesicle trafficking protein [Source:HGNC Symbol;Acc:HGNC:20260]	-2.6	-2
5303	*STC1*	6781	stanniocalcin 1 [Source:HGNC Symbol;Acc:HGNC:11373]	-2.6	-2
5304	*C6orf48*	50854	chromosome 6 open reading frame 48 [Source:HGNC Symbol;Acc:HGNC:19078]	-2.6	-2
5305	*KLF9*	687	Kruppel like factor 9 [Source:HGNC Symbol;Acc:HGNC:1123]	-2.6	-2
5306	*TMEM38A*	79041	transmembrane protein 38A [Source:HGNC Symbol;Acc:HGNC:28462]	-2.6	-2
5307	*AOC3*	8639	amine oxidase, copper containing 3 [Source:HGNC Symbol;Acc:HGNC:550]	-2.6	-2
5308	*DESI2*	51029	desumoylating isopeptidase 2 [Source:HGNC Symbol;Acc:HGNC:24264]	-2.6	-2
5309	*RUNDC3B*	154661	RUN domain containing 3B [Source:HGNC Symbol;Acc:HGNC:30286]	-2.6	-2
5310	*MOBP*	4336	myelin-associated oligodendrocyte basic protein [Source:HGNC Symbol;Acc:HGNC:7189]	-2.6	-2
5311	*DHX40*	79665	DEAH-box helicase 40 [Source:HGNC Symbol;Acc:HGNC:18018]	-2.7	-2
5312	*PIR*	8544	pirin [Source:HGNC Symbol;Acc:HGNC:30048]	-2.7	-2
5313	*MEX3C*	51320	mex-3 RNA binding family member C [Source:HGNC Symbol;Acc:HGNC:28040]	-2.7	-2
5314	*STXBP1*	6812	syntaxin binding protein 1 [Source:HGNC Symbol;Acc:HGNC:11444]	-2.7	-2
5315	*DCT*	1638	dopachrome tautomerase [Source:HGNC Symbol;Acc:HGNC:2709]	-2.7	-2
5316	*BBS2*	583	Bardet-Biedl syndrome 2 [Source:HGNC Symbol;Acc:HGNC:967]	-2.7	-2
5317	*SNAP47*	116841	synaptosome associated protein 47 [Source:HGNC Symbol;Acc:HGNC:30669]	-2.7	-2
5318	*CYP4Z1*	199974	cytochrome P450 family 4 subfamily Z member 1 [Source:HGNC Symbol;Acc:HGNC:20583]	-2.7	-2
5319	*FBXO32*	114907	F-box protein 32 [Source:HGNC Symbol;Acc:HGNC:16731]	-2.7	-2
5320	*CDK13*	8621	cyclin dependent kinase 13 [Source:HGNC Symbol;Acc:HGNC:1733]	-2.7	-2
5321	*DNAJB4*	11080	DnaJ heat shock protein family (Hsp40) member B4 [Source:HGNC Symbol;Acc:HGNC:14886]	-2.7	-2
5322	*CBFA2T3*	863	CBFA2/RUNX1 translocation partner 3 [Source:HGNC Symbol;Acc:HGNC:1537]	-2.7	-2
5323	*ERO1B*	56605	endoplasmic reticulum oxidoreductase beta [Source:HGNC Symbol;Acc:HGNC:14355]	-2.7	-2
5324	*MEGF6*	1953	multiple EGF like domains 6 [Source:HGNC Symbol;Acc:HGNC:3232]	-2.7	-2
5325	*CRYBB2*	1415	crystallin beta B2 [Source:HGNC Symbol;Acc:HGNC:2398]	-2.7	-2
5326	*POLR2J3*	548644	polymerase (RNA) II subunit J3 [Source:HGNC Symbol;Acc:HGNC:33853]	-2.7	-2
5327	*HEATR4*	399671	HEAT repeat containing 4 [Source:HGNC Symbol;Acc:HGNC:16761]	-2.7	-2
5328	*PRRC1*	133619	proline rich coiled-coil 1 [Source:HGNC Symbol;Acc:HGNC:28164]	-2.7	-2
5329	*SGCB*	6443	sarcoglycan beta [Source:HGNC Symbol;Acc:HGNC:10806]	-2.7	-2
5330	*UNC79*	57578	unc-79 homolog (C. elegans) [Source:HGNC Symbol;Acc:HGNC:19966]	-2.7	-2
5331	*MLIP*	90523	muscular LMNA-interacting protein [Source:HGNC Symbol;Acc:HGNC:21355]	-2.7	-2
5332	*TMEM14C*	51522	transmembrane protein 14C [Source:HGNC Symbol;Acc:HGNC:20952]	-2.7	-2
5333	*MYBBP1A*	10514	MYB binding protein 1a [Source:HGNC Symbol;Acc:HGNC:7546]	-2.7	-2
5334	*CCDC88B*	283234	coiled-coil domain containing 88B [Source:HGNC Symbol;Acc:HGNC:26757]	-2.7	-2
5335	*OSGIN1*	29948	oxidative stress induced growth inhibitor 1 [Source:HGNC Symbol;Acc:HGNC:30093]	-2.7	-2
5336	*GAB2*	9846	GRB2 associated binding protein 2 [Source:HGNC Symbol;Acc:HGNC:14458]	-2.7	-2
5337	*FRMPD1*	22844	FERM and PDZ domain containing 1 [Source:HGNC Symbol;Acc:HGNC:29159]	-2.7	-2
5338	*DHCR7*	1717	7-dehydrocholesterol reductase [Source:HGNC Symbol;Acc:HGNC:2860]	-2.7	-2
5339	*SPRR2D*	6703	small proline rich protein 2D [Source:HGNC Symbol;Acc:HGNC:11264]	-2.7	-2
5340	*MYOCD*	93649	myocardin [Source:HGNC Symbol;Acc:HGNC:16067]	-2.7	-2
5341	*CKMT1B*	1159	creatine kinase, mitochondrial 1B [Source:HGNC Symbol;Acc:HGNC:1995]	-2.7	-2
5342	*FBXO34*	55030	F-box protein 34 [Source:HGNC Symbol;Acc:HGNC:20201]	-2.7	-2
5343	*PTPRU*	10076	protein tyrosine phosphatase, receptor type U [Source:HGNC Symbol;Acc:HGNC:9683]	-2.7	-2

5344	*SLC25A18*	83733	solute carrier family 25 member 18 [Source:HGNC Symbol;Acc:HGNC:10988]	-2.7	-2
5345	*ZNF540*	163255	zinc finger protein 540 [Source:HGNC Symbol;Acc:HGNC:25331]	-2.7	-2
5346	*ANGEL2*	90806	angel homolog 2 (Drosophila) [Source:HGNC Symbol;Acc:HGNC:30534]	-2.7	-2
5347	*DHX57*	90957	DEAH-box helicase 57 [Source:HGNC Symbol;Acc:HGNC:20086]	-2.7	-2
5348	*NSRP1*	84081	nuclear speckle splicing regulatory protein 1 [Source:HGNC Symbol;Acc:HGNC:25305]	-2.7	-2
5349	*DAPK2*	23604	death associated protein kinase 2 [Source:HGNC Symbol;Acc:HGNC:2675]	-2.7	-2
5350	*GID4*	79018	GID complex subunit 4 homolog [Source:HGNC Symbol;Acc:HGNC:28453]	-2.7	-2
5351	*PPP1R14C*	81706	protein phosphatase 1 regulatory inhibitor subunit 14C [Source:HGNC Symbol;Acc:HGNC:14952]	-2.7	-2
5352	*ZNF611*	81856	zinc finger protein 611 [Source:HGNC Symbol;Acc:HGNC:28766]	-2.7	-2
5353	*ZNF691*	51058	zinc finger protein 691 [Source:HGNC Symbol;Acc:HGNC:28028]	-2.7	-2
5354	*FTO*	79068	fat mass and obesity associated [Source:HGNC Symbol;Acc:HGNC:24678]	-2.7	-2
5355	*NCOA4*	8031	nuclear receptor coactivator 4 [Source:HGNC Symbol;Acc:HGNC:7671]	-2.7	-2
5356	*STK16*	8576	serine/threonine kinase 16 [Source:HGNC Symbol;Acc:HGNC:11394]	-2.7	-2
5357	*OR7D2*	162998	olfactory receptor family 7 subfamily D member 2 [Source:HGNC Symbol;Acc:HGNC:8378]	-2.7	-2
5358	*PMEPA1*	56937	prostate transmembrane protein, androgen induced 1 [Source:HGNC Symbol;Acc:HGNC:14107]	-2.7	-2
5359	*PIGX*	54965	phosphatidylinositol glycan anchor biosynthesis class X [Source:HGNC Symbol;Acc:HGNC:26046]	-2.7	-2
5360	*HLA-B*	3106	major histocompatibility complex, class I, B [Source:HGNC Symbol;Acc:HGNC:4932]	-2.7	-2
5361	*THADA*	63892	THADA, armadillo repeat containing [Source:HGNC Symbol;Acc:HGNC:19217]	-2.7	-2
5362	*FAM200B*	285550	family with sequence similarity 200 member B [Source:HGNC Symbol;Acc:HGNC:27740]	-2.7	-2
5363	*LY6K*	54742	lymphocyte antigen 6 complex, locus K [Source:HGNC Symbol;Acc:HGNC:24225]	-2.7	-2
5364	*RBAK*	57786	RB associated KRAB zinc finger [Source:HGNC Symbol;Acc:HGNC:17680]	-2.7	-2
5365	*RBAK-RBAKDN*	100533952	RBAK-RBAKDN readthrough [Source:HGNC Symbol;Acc:HGNC:42971]	-2.7	-2
5366	*ZNF141*	7700	zinc finger protein 141 [Source:HGNC Symbol;Acc:HGNC:12926]	-2.7	-2
5367	*RAB6A*	5870	RAB6A, member RAS oncogene family [Source:HGNC Symbol;Acc:HGNC:9786]	-2.7	-2
5368	*GPN3*	51184	GPN-loop GTPase 3 [Source:HGNC Symbol;Acc:HGNC:30186]	-2.7	-2
5369	*PKNOX1*	5316	PBX/knotted 1 homeobox 1 [Source:HGNC Symbol;Acc:HGNC:9022]	-2.7	-2
5370	*CASP6*	839	caspase 6 [Source:HGNC Symbol;Acc:HGNC:1507]	-2.7	-2
5371	*AARS*	16	alanyl-tRNA synthetase [Source:HGNC Symbol;Acc:HGNC:20]	-2.7	-2
5372	*SASH1*	23328	SAM and SH3 domain containing 1 [Source:HGNC Symbol;Acc:HGNC:19182]	-2.7	-2
5373	*CALCOCO2*	10241	calcium binding and coiled-coil domain 2 [Source:HGNC Symbol;Acc:HGNC:29912]	-2.7	-2
5374	*RORA*	6095	RAR related orphan receptor A [Source:HGNC Symbol;Acc:HGNC:10258]	-2.7	-2
5375	*GDE1*	51573	glycerophosphodiester phosphodiesterase 1 [Source:HGNC Symbol;Acc:HGNC:29644]	-2.7	-2
5376	*DTX4*	23220	deltex 4, E3 ubiquitin ligase [Source:HGNC Symbol;Acc:HGNC:29151]	-2.7	-2
5377	*EEPD1*	80820	endonuclease/exonuclease/phosphatase family domain containing 1 [Source:HGNC Symbol;Acc:HGNC:22223]	-2.7	-2
5378	*DOCK1*	1793	dedicator of cytokinesis 1 [Source:HGNC Symbol;Acc:HGNC:2987]	-2.7	-2
5379	*HPD*	3242	4-hydroxyphenylpyruvate dioxygenase [Source:HGNC Symbol;Acc:HGNC:5147]	-2.7	-2
5380	*ANKK1*	255239	ankyrin repeat and kinase domain containing 1 [Source:HGNC Symbol;Acc:HGNC:21027]	-2.7	-2
5381	*MACF1*	23499	microtubule-actin crosslinking factor 1 [Source:HGNC Symbol;Acc:HGNC:13664]	-2.7	-2
5382	*CNTLN*	54875	centlein [Source:HGNC Symbol;Acc:HGNC:23432]	-2.7	-2
5383	*PLEKHG5*	57449	pleckstrin homology and RhoGEF domain containing G5 [Source:HGNC Symbol;Acc:HGNC:29105]	-2.7	-2
5384	*TSC22D3*	1831	TSC22 domain family member 3 [Source:HGNC Symbol;Acc:HGNC:3051]	-2.7	-2
5385	*PLEKHA2*	59339	pleckstrin homology domain containing A2 [Source:HGNC Symbol;Acc:HGNC:14336]	-2.7	-2
5386	*TRIP11*	9321	thyroid hormone receptor interactor 11 [Source:HGNC Symbol;Acc:HGNC:12305]	-2.7	-2
5387	*VEGFA*	7422	vascular endothelial growth factor A [Source:HGNC Symbol;Acc:HGNC:12680]	-2.7	-2
5388	*UFM1*	51569	ubiquitin-fold modifier 1 [Source:HGNC Symbol;Acc:HGNC:20597]	-2.7	-2
5389	*LRR1*	122769	leucine rich repeat protein 1 [Source:HGNC Symbol;Acc:HGNC:19742]	-2.7	-2
5390	*FEM1A*	55527	fem-1 homolog A [Source:HGNC Symbol;Acc:HGNC:16934]	-2.7	-2
5391	*NUP210L*	91181	nucleoporin 210 like [Source:HGNC Symbol;Acc:HGNC:29915]	-2.7	-2
5392	*NXN*	64359	nucleoredoxin [Source:HGNC Symbol;Acc:HGNC:18008]	-2.7	-2
5393	*ZCWPW1*	55063	zinc finger CW-type and PWWP domain containing 1 [Source:HGNC Symbol;Acc:HGNC:23486]	-2.7	-2
5394	*WDR55*	54853	WD repeat domain 55 [Source:HGNC Symbol;Acc:HGNC:25971]	-2.7	-2
5395	*UBE2B*	7320	ubiquitin conjugating enzyme E2 B [Source:HGNC Symbol;Acc:HGNC:12473]	-2.7	-2
5396	*LMX1B*	4010	LIM homeobox transcription factor 1 beta [Source:HGNC Symbol;Acc:HGNC:6654]	-2.7	-2
5397	*IL18*	3606	interleukin 18 [Source:HGNC Symbol;Acc:HGNC:5986]	-2.7	-2
5398	*UPB1*	51733	beta-ureidopropionase 1 [Source:HGNC Symbol;Acc:HGNC:16297]	-2.7	-2
5399	*PTCHD4*	442213	patched domain containing 4 [Source:HGNC Symbol;Acc:HGNC:21345]	-2.7	-2
5400	*FAM118A*	55007	family with sequence similarity 118 member A [Source:HGNC Symbol;Acc:HGNC:1313]	-2.7	-2
5401	*CHFR*	55743	checkpoint with forkhead and ring finger domains, E3 ubiquitin protein ligase [Source:HGNC Symbol;Acc:HGNC:20455]	-2.7	-2
5402	*TCF12*	6938	transcription factor 12 [Source:HGNC Symbol;Acc:HGNC:11623]	-2.7	-2
5403	*ELP4*	26610	elongator acetyltransferase complex subunit 4 [Source:HGNC Symbol;Acc:HGNC:1171]	-2.7	-2
5404	*ACBD4*	79777	acyl-CoA binding domain containing 4 [Source:HGNC Symbol;Acc:HGNC:23337]	-2.7	-2
5405	*HLA-DRB5*	3127	major histocompatibility complex, class II, DR beta 5 [Source:HGNC Symbol;Acc:HGNC:4953]	-2.7	-2
5406	*SNX25*	83891	sorting nexin 25 [Source:HGNC Symbol;Acc:HGNC:21883]	-2.7	-2
5407	*RAB38*	23682	RAB38, member RAS oncogene family [Source:HGNC Symbol;Acc:HGNC:9776]	-2.8	-2
5408	*FAM131A*	131408	family with sequence similarity 131 member A [Source:HGNC Symbol;Acc:HGNC:28308]	-2.8	-2
5409	*IFT172*	26160	intraflagellar transport 172 [Source:HGNC Symbol;Acc:HGNC:30391]	-2.8	-2
5410	*PTCD2*	79810	pentatricopeptide repeat domain 2 [Source:HGNC Symbol;Acc:HGNC:25734]	-2.8	-2
5411	*ZKSCAN7*	55888	zinc finger with KRAB and SCAN domains 7 [Source:HGNC Symbol;Acc:HGNC:12955]	-2.8	-2
5412	*PAIP2*	51247	poly(A) binding protein interacting protein 2 [Source:HGNC Symbol;Acc:HGNC:17970]	-2.8	-2
5413	*STKLD1*	169436	serine/threonine kinase-like domain containing 1 [Source:HGNC Symbol;Acc:HGNC:28669]	-2.8	-2
5414	*THBS2*	7058	thrombospondin 2 [Source:HGNC Symbol;Acc:HGNC:11786]	-2.8	-2
5415	*STXBP5*	134957	syntaxin binding protein 5 [Source:HGNC Symbol;Acc:HGNC:19665]	-2.8	-2
5416	*PROSER1*	80209	proline and serine rich 1 [Source:HGNC Symbol;Acc:HGNC:20291]	-2.8	-2
5417	*ZNF2*	7549	zinc finger protein 2 [Source:HGNC Symbol;Acc:HGNC:12991]	-2.8	-2

5418	*PRSS12*	8492	protease, serine 12 [Source:HGNC Symbol;Acc:HGNC:9477]	-2.8	-2
5419	*CERK*	64781	ceramide kinase [Source:HGNC Symbol;Acc:HGNC:19256]	-2.8	-2
5420	*ADAMTS4*	9507	ADAM metallopeptidase with thrombospondin type 1 motif 4 [Source:HGNC Symbol;Acc:HGNC:220]	-2.8	-2
5421	*ETFDH*	2110	electron transfer flavoprotein dehydrogenase [Source:HGNC Symbol;Acc:HGNC:3483]	-2.8	-2
5422	*TMEM106B*	54664	transmembrane protein 106B [Source:HGNC Symbol;Acc:HGNC:22407]	-2.8	-2
5423	*PARP8*	79668	poly(ADP-ribose) polymerase family member 8 [Source:HGNC Symbol;Acc:HGNC:26124]	-2.8	-2
5424	*WFDC3*	140686	WAP four-disulfide core domain 3 [Source:HGNC Symbol;Acc:HGNC:15957]	-2.8	-2
5425	*SWAP70*	23075	SWAP switching B-cell complex 70kDa subunit [Source:HGNC Symbol;Acc:HGNC:17070]	-2.8	-2
5426	*C4orf32*	132720	chromosome 4 open reading frame 32 [Source:HGNC Symbol;Acc:HGNC:26813]	-2.8	-2
5427	*KLK12*	43849	kallikrein related peptidase 12 [Source:HGNC Symbol;Acc:HGNC:6360]	-2.8	-2
5428	*HLA-C*	3107	major histocompatibility complex, class I, C [Source:HGNC Symbol;Acc:HGNC:4933]	-2.8	-2
5429	*FMN1*	342184	formin 1 [Source:HGNC Symbol;Acc:HGNC:3768]	-2.8	-2
5430	*CDK19*	23097	cyclin dependent kinase 19 [Source:HGNC Symbol;Acc:HGNC:19338]	-2.8	-2
5431	*GTF2IRD1*	9569	GTF2I repeat domain containing 1 [Source:HGNC Symbol;Acc:HGNC:4661]	-2.8	-2
5432	*TSKU*	25987	tsukushi, small leucine rich proteoglycan [Source:HGNC Symbol;Acc:HGNC:28850]	-2.8	-2
5433	*FAM20B*	9917	family with sequence similarity 20 member B [Source:HGNC Symbol;Acc:HGNC:23017]	-2.8	-2
5434	*LIG4*	3981	DNA ligase 4 [Source:HGNC Symbol;Acc:HGNC:6601]	-2.8	-2
5435	*ABCB10*	23456	ATP binding cassette subfamily B member 10 [Source:HGNC Symbol;Acc:HGNC:41]	-2.8	-2
5436	*TMEM192*	201931	transmembrane protein 192 [Source:HGNC Symbol;Acc:HGNC:26775]	-2.8	-2
5437	*ALDH4A1*	8659	aldehyde dehydrogenase 4 family member A1 [Source:HGNC Symbol;Acc:HGNC:406]	-2.8	-2
5438	*CHIT1*	1118	chitinase 1 [Source:HGNC Symbol;Acc:HGNC:1936]	-2.8	-2
5439	*ACAP2*	23527	ArfGAP with coiled-coil, ankyrin repeat and PH domains 2 [Source:HGNC Symbol;Acc:HGNC:16469]	-2.8	-2
5440	*ARPIN*	348110	actin-related protein 2/3 complex inhibitor [Source:HGNC Symbol;Acc:HGNC:28782]	-2.8	-2
5441	*SYCE2*	256126	synaptonemal complex central element protein 2 [Source:HGNC Symbol;Acc:HGNC:27411]	-2.8	-2
5442	*LCMT2*	9836	leucine carboxyl methyltransferase 2 [Source:HGNC Symbol;Acc:HGNC:17558]	-2.8	-2
5443	*ZNF69*	7620	zinc finger protein 69 [Source:HGNC Symbol;Acc:HGNC:13138]	-2.8	-2
5444	*KLHL24*	54800	kelch like family member 24 [Source:HGNC Symbol;Acc:HGNC:25947]	-2.8	-2
5445	*WDYHV1*	55093	WDYHV motif containing 1 [Source:HGNC Symbol;Acc:HGNC:25490]	-2.8	-2
5446	*MERTK*	10461	MER proto-oncogene, tyrosine kinase [Source:HGNC Symbol;Acc:HGNC:7027]	-2.8	-2
5447	*OCA2*	4948	OCA2 melanosomal transmembrane protein [Source:HGNC Symbol;Acc:HGNC:8101]	-2.8	-2
5448	*RNMT*	8731	RNA guanine-7 methyltransferase [Source:HGNC Symbol;Acc:HGNC:10075]	-2.8	-2
5449	*ZSCAN20*	7579	zinc finger and SCAN domain containing 20 [Source:HGNC Symbol;Acc:HGNC:13093]	-2.8	-2
5450	*CST3*	1471	cystatin C [Source:HGNC Symbol;Acc:HGNC:2475]	-2.8	-2
5451	*TMEM119*	338773	transmembrane protein 119 [Source:HGNC Symbol;Acc:HGNC:27884]	-2.8	-2
5452	*PARP6*	56965	poly(ADP-ribose) polymerase family member 6 [Source:HGNC Symbol;Acc:HGNC:26921]	-2.8	-2
5453	*CD82*	3732	CD82 molecule [Source:HGNC Symbol;Acc:HGNC:6210]	-2.8	-2
5454	*ATAD1*	84896	ATPase family, AAA domain containing 1 [Source:HGNC Symbol;Acc:HGNC:25903]	-2.8	-2
5455	*ATP1A2*	477	ATPase Na+/K+ transporting subunit alpha 2 [Source:HGNC Symbol;Acc:HGNC:800]	-2.8	-2
5456	*CITED2*	10370	Cbp/p300 interacting transactivator with Glu/Asp rich carboxy-terminal domain 2 [Source:HGNC Symbol;Acc:HGNC:1987]	-2.8	-2
5457	*BTBD3*	22903	BTB domain containing 3 [Source:HGNC Symbol;Acc:HGNC:15854]	-2.8	-2
5458	*FAHD2A*	51011	fumarylacetoacetate hydrolase domain containing 2A [Source:HGNC Symbol;Acc:HGNC:24252]	-2.8	-2
5459	*OGDH*	4967	oxoglutarate dehydrogenase [Source:HGNC Symbol;Acc:HGNC:8124]	-2.8	-2
5460	*IFNL1*	282618	interferon, lambda 1 [Source:HGNC Symbol;Acc:HGNC:18363]	-2.8	-2
5461	*FA2H*	79152	fatty acid 2-hydroxylase [Source:HGNC Symbol;Acc:HGNC:21197]	-2.8	-2
5462	*GJB2*	2706	gap junction protein beta 2 [Source:HGNC Symbol;Acc:HGNC:4284]	-2.8	-2
5463	*C22orf39*	128977	chromosome 22 open reading frame 39 [Source:HGNC Symbol;Acc:HGNC:27012]	-2.8	-2
5464	*MSC*	9242	musculin [Source:HGNC Symbol;Acc:HGNC:7321]	-2.8	-2
5465	*TRPA1*	8989	transient receptor potential cation channel subfamily A member 1 [Source:HGNC Symbol;Acc:HGNC:497]	-2.8	-2
5466	*USP40*	55230	ubiquitin specific peptidase 40 [Source:HGNC Symbol;Acc:HGNC:20069]	-2.8	-2
5467	*CRIP3*	401262	cysteine rich protein 3 [Source:HGNC Symbol;Acc:HGNC:17751]	-2.8	-2
5468	*CCNB1IP1*	57820	cyclin B1 interacting protein 1 [Source:HGNC Symbol;Acc:HGNC:19437]	-2.8	-2
5469	*ACBD7*	414149	acyl-CoA binding domain containing 7 [Source:HGNC Symbol;Acc:HGNC:17715]	-2.8	-2
5470	*IL17RC*	84818	interleukin 17 receptor C [Source:HGNC Symbol;Acc:HGNC:18358]	-2.8	-2
5471	*STIL*	6491	SCL/TAL1 interrupting locus [Source:HGNC Symbol;Acc:HGNC:10879]	-2.8	-2
5472	*CAPNS2*	84290	calpain small subunit 2 [Source:HGNC Symbol;Acc:HGNC:16371]	-2.8	-2
5473	*PICALM*	8301	phosphatidylinositol binding clathrin assembly protein [Source:HGNC Symbol;Acc:HGNC:15514]	-2.8	-2
5474	*SCOC*	60592	short coiled-coil protein [Source:HGNC Symbol;Acc:HGNC:20335]	-2.8	-2
5475	*PHYH*	5264	phytanoyl-CoA 2-hydroxylase [Source:HGNC Symbol;Acc:HGNC:8940]	-2.8	-2
5476	*SENP2*	59343	SUMO1/sentrin/SMT3 specific peptidase 2 [Source:HGNC Symbol;Acc:HGNC:23116]	-2.8	-2
5477	*ATP6V0D2*	245972	ATPase H+ transporting V0 subunit d2 [Source:HGNC Symbol;Acc:HGNC:18266]	-2.8	-2
5478	*NARF*	26502	nuclear prelamin A recognition factor [Source:HGNC Symbol;Acc:HGNC:29916]	-2.8	-2
5479	*SYBU*	55638	syntabulin [Source:HGNC Symbol;Acc:HGNC:26011]	-2.8	-2
5480	*ACP5*	54	acid phosphatase 5, tartrate resistant [Source:HGNC Symbol;Acc:HGNC:124]	-2.8	-2
5481	*HEBP2*	23593	heme binding protein 2 [Source:HGNC Symbol;Acc:HGNC:15716]	-2.8	-2
5482	*TSPO*	706	translocator protein [Source:HGNC Symbol;Acc:HGNC:1158]	-2.8	-2
5483	*CENPBD1*	92806	CENPB DNA-binding domain containing 1 [Source:HGNC Symbol;Acc:HGNC:28272]	-2.8	-2
5484	*DCAKD*	79877	dephospho-CoA kinase domain containing [Source:HGNC Symbol;Acc:HGNC:26238]	-2.8	-2
5485	*HERC1*	8925	HECT and RLD domain containing E3 ubiquitin protein ligase family member 1 [Source:HGNC Symbol;Acc:HGNC:4867]	-2.8	-2
5486	*ASB16*	92591	ankyrin repeat and SOCS box containing 16 [Source:HGNC Symbol;Acc:HGNC:19768]	-2.8	-2
5487	*PLA2G4A*	5321	phospholipase A2 group IVA [Source:HGNC Symbol;Acc:HGNC:9035]	-2.8	-2
5488	*NCAPD3*	23310	non-SMC condensin II complex subunit D3 [Source:HGNC Symbol;Acc:HGNC:28952]	-2.8	-2
5489	*NCKAP5*	344148	NCK associated protein 5 [Source:HGNC Symbol;Acc:HGNC:29847]	-2.8	-2
5490	*C11orf74*	119710	chromosome 11 open reading frame 74 [Source:HGNC Symbol;Acc:HGNC:25142]	-2.8	-2
5491	*ASCC1*	51008	activating signal cointegrator 1 complex subunit 1 [Source:HGNC Symbol;Acc:HGNC:24268]	-2.8	-2

5492	*CCR6*	1235	C-C motif chemokine receptor 6 [Source:HGNC Symbol;Acc:HGNC:1607]	-2.8	-2
5493	*SRGAP1*	57522	SLIT-ROBO Rho GTPase activating protein 1 [Source:HGNC Symbol;Acc:HGNC:17382]	-2.8	-2
5494	*C12orf4*	57102	chromosome 12 open reading frame 4 [Source:HGNC Symbol;Acc:HGNC:1184]	-2.8	-2
5495	*ANKRD35*	148741	ankyrin repeat domain 35 [Source:HGNC Symbol;Acc:HGNC:26323]	-2.8	-2
5496	*MS4A4A*	51338	membrane spanning 4-domains A4A [Source:HGNC Symbol;Acc:HGNC:13371]	-2.8	-2
5497	*MCC*	4163	mutated in colorectal cancers [Source:HGNC Symbol;Acc:HGNC:6935]	-2.8	-2
5498	*RRM2B*	50484	ribonucleotide reductase regulatory TP53 inducible subunit M2B [Source:HGNC Symbol;Acc:HGNC:17296]	-2.8	-2
5499	*FCGBP*	8857	Fc fragment of IgG binding protein [Source:HGNC Symbol;Acc:HGNC:13572]	-2.8	-2
5500	*STON1*	11037	stonin 1 [Source:HGNC Symbol;Acc:HGNC:17003]	-2.8	-2
5501	*ZNF606*	80095	zinc finger protein 606 [Source:HGNC Symbol;Acc:HGNC:25879]	-2.8	-2
5502	*CRTAM*	56253	cytotoxic and regulatory T-cell molecule [Source:HGNC Symbol;Acc:HGNC:24313]	-2.9	-2
5503	*KIF5A*	3798	kinesin family member 5A [Source:HGNC Symbol;Acc:HGNC:6323]	-2.9	-2
5504	*DEFA4*	1669	defensin alpha 4 [Source:HGNC Symbol;Acc:HGNC:2763]	-2.9	-2
5505	*CMC4*	100272147	C-X9-C motif containing 4 [Source:HGNC Symbol;Acc:HGNC:35428]	-2.9	-2
5506	*ERRFI1*	54206	ERBB receptor feedback inhibitor 1 [Source:HGNC Symbol;Acc:HGNC:18185]	-2.9	-2
5507	*BBS1*	582	Bardet-Biedl syndrome 1 [Source:HGNC Symbol;Acc:HGNC:966]	-2.9	-2
5508	*TMEM131*	23505	transmembrane protein 131 [Source:HGNC Symbol;Acc:HGNC:30366]	-2.9	-2
5509	*MGAT3*	4248	mannosyl (beta-1,4-)-glycoprotein beta-1,4-N-acetylglucosaminyltransferase [Source:HGNC Symbol;Acc:HGNC:7046]	-2.9	-2
5510	*AKIP1*	56672	A-kinase interacting protein 1 [Source:HGNC Symbol;Acc:HGNC:1170]	-2.9	-2
5511	*GNAL*	2774	G protein subunit alpha L [Source:HGNC Symbol;Acc:HGNC:4388]	-2.9	-2
5512	*RAP1B*	5908	RAP1B, member of RAS oncogene family [Source:HGNC Symbol;Acc:HGNC:9857]	-2.9	-2
5513	*METAP1*	23173	methionyl aminopeptidase 1 [Source:HGNC Symbol;Acc:HGNC:15789]	-2.9	-2
5514	*C22orf15*	150248	chromosome 22 open reading frame 15 [Source:HGNC Symbol;Acc:HGNC:15558]	-2.9	-2
5515	*CHCHD10*	400916	coiled-coil-helix-coiled-coil-helix domain containing 10 [Source:HGNC Symbol;Acc:HGNC:15559]	-2.9	-2
5516	*EMC1*	23065	ER membrane protein complex subunit 1 [Source:HGNC Symbol;Acc:HGNC:28957]	-2.9	-2
5517	*ICK*	22858	intestinal cell (MAK-like) kinase [Source:HGNC Symbol;Acc:HGNC:21219]	-2.9	-2
5518	*CALCRL*	10203	calcitonin receptor like receptor [Source:HGNC Symbol;Acc:HGNC:16709]	-2.9	-2
5519	*PNMAL1*	55228	paraneoplastic Ma antigen family-like 1 [Source:HGNC Symbol;Acc:HGNC:25578]	-2.9	-2
5520	*NBR1*	4077	NBR1, autophagy cargo receptor [Source:HGNC Symbol;Acc:HGNC:6746]	-2.9	-2
5521	*MMAB*	326625	methylmalonic aciduria (cobalamin deficiency) cblB type [Source:HGNC Symbol;Acc:HGNC:19331]	-2.9	-2
5522	*LDLRAD2*	401944	low density lipoprotein receptor class A domain containing 2 [Source:HGNC Symbol;Acc:HGNC:32071]	-2.9	-2
5523	*GPR157*	80045	G protein-coupled receptor 157 [Source:HGNC Symbol;Acc:HGNC:23687]	-2.9	-2
5524	*ALPPL2*	251	alkaline phosphatase, placental like 2 [Source:HGNC Symbol;Acc:HGNC:441]	-2.9	-2
5525	*SPIB*	6689	Spi-B transcription factor [Source:HGNC Symbol;Acc:HGNC:11242]	-2.9	-2
5526	*TMEM30A*	55754	transmembrane protein 30A [Source:HGNC Symbol;Acc:HGNC:16667]	-2.9	-2
5527	*KRTDAP*	388533	keratinocyte differentiation associated protein [Source:HGNC Symbol;Acc:HGNC:16313]	-2.9	-2
5528	*ATP6V0A2*	23545	ATPase H+ transporting V0 subunit a2 [Source:HGNC Symbol;Acc:HGNC:18481]	-2.9	-2
5529	*LRRC49*	54839	leucine rich repeat containing 49 [Source:HGNC Symbol;Acc:HGNC:25965]	-2.9	-2
5530	*C19orf38*	255809	chromosome 19 open reading frame 38 [Source:HGNC Symbol;Acc:HGNC:34073]	-2.9	-2
5531	*PHLDA2*	7262	pleckstrin homology like domain family A member 2 [Source:HGNC Symbol;Acc:HGNC:12385]	-2.9	-2
5532	*PLD6*	201164	phospholipase D family member 6 [Source:HGNC Symbol;Acc:HGNC:30447]	-2.9	-2
5533	*RNF103*	7844	ring finger protein 103 [Source:HGNC Symbol;Acc:HGNC:12859]	-2.9	-2
5534	*HIST1H1C*	3006	histone cluster 1, H1c [Source:HGNC Symbol;Acc:HGNC:4716]	-2.9	-2
5535	*IQCC*	55721	IQ motif containing C [Source:HGNC Symbol;Acc:HGNC:25545]	-2.9	-2
5536	*ELOVL1*	64834	ELOVL fatty acid elongase 1 [Source:HGNC Symbol;Acc:HGNC:14418]	-2.9	-2
5537	*TCN2*	6948	transcobalamin 2 [Source:HGNC Symbol;Acc:HGNC:11653]	-2.9	-2
5538	*SDCCAG3*	10807	serologically defined colon cancer antigen 3 [Source:HGNC Symbol;Acc:HGNC:10667]	-2.9	-2
5539	*PMEL*	6490	premelanosome protein [Source:HGNC Symbol;Acc:HGNC:10880]	-2.9	-2
5540	*TRPC6*	7225	transient receptor potential cation channel subfamily C member 6 [Source:HGNC Symbol;Acc:HGNC:12338]	-2.9	-2
5541	*MPC1*	51660	mitochondrial pyruvate carrier 1 [Source:HGNC Symbol;Acc:HGNC:21606]	-2.9	-2
5542	*SNRNP48*	154007	small nuclear ribonucleoprotein U11/U12 subunit 48 [Source:HGNC Symbol;Acc:HGNC:21368]	-2.9	-2
5543	*URI1*	8725	URI1, prefoldin like chaperone [Source:HGNC Symbol;Acc:HGNC:13236]	-2.9	-2
5544	*PAPOLG*	64895	poly(A) polymerase gamma [Source:HGNC Symbol;Acc:HGNC:14982]	-2.9	-2
5545	*GUCA1B*	2979	guanylate cyclase activator 1B [Source:HGNC Symbol;Acc:HGNC:4679]	-2.9	-2
5546	*PQLC2*	54896	PQ loop repeat containing 2 [Source:HGNC Symbol;Acc:HGNC:26001]	-2.9	-2
5547	*ZNF707*	286075	zinc finger protein 707 [Source:HGNC Symbol;Acc:HGNC:27815]	-2.9	-2
5548	*TNFSF14*	8740	tumor necrosis factor superfamily member 14 [Source:HGNC Symbol;Acc:HGNC:11930]	-2.9	-2
5549	*B3GALT6*	126792	beta-1,3-galactosyltransferase 6 [Source:HGNC Symbol;Acc:HGNC:17978]	-2.9	-2
5550	*NCAM1*	4684	neural cell adhesion molecule 1 [Source:HGNC Symbol;Acc:HGNC:7656]	-2.9	-2
5551	*CYP2F1*	1572	cytochrome P450 family 2 subfamily F member 1 [Source:HGNC Symbol;Acc:HGNC:2632]	-2.9	-2
5552	*BTBD19*	149478	BTB domain containing 19 [Source:HGNC Symbol;Acc:HGNC:27145]	-2.9	-2
5553	*TP53BP2*	7159	tumor protein p53 binding protein 2 [Source:HGNC Symbol;Acc:HGNC:12000]	-2.9	-2
5554	*QPRT*	23475	quinolinate phosphoribosyltransferase [Source:HGNC Symbol;Acc:HGNC:9755]	-2.9	-2
5555	*NXT1*	29107	nuclear transport factor 2 like export factor 1 [Source:HGNC Symbol;Acc:HGNC:15913]	-2.9	-2
5556	*CASD1*	64921	CAS1 domain containing 1 [Source:HGNC Symbol;Acc:HGNC:16014]	-2.9	-2
5557	*SSRP1*	6749	structure specific recognition protein 1 [Source:HGNC Symbol;Acc:HGNC:11327]	-2.9	-2
5558	*ZSCAN18*	65982	zinc finger and SCAN domain containing 18 [Source:HGNC Symbol;Acc:HGNC:21037]	-2.9	-2
5559	*EIF4A3*	9775	eukaryotic translation initiation factor 4A3 [Source:HGNC Symbol;Acc:HGNC:18683]	-2.9	-2
5560	*OXTR*	5021	oxytocin receptor [Source:HGNC Symbol;Acc:HGNC:8529]	-2.9	-2
5561	*C1orf194*	127003	chromosome 1 open reading frame 194 [Source:HGNC Symbol;Acc:HGNC:32331]	-2.9	-2
5562	*STX2*	2054	syntaxin 2 [Source:HGNC Symbol;Acc:HGNC:3403]	-2.9	-2
5563	*C11orf71*	54494	chromosome 11 open reading frame 71 [Source:HGNC Symbol;Acc:HGNC:25937]	-2.9	-2
5564	*TULP4*	56995	tubby like protein 4 [Source:HGNC Symbol;Acc:HGNC:15530]	-2.9	-2
5565	*NUDT22*	84304	nudix hydrolase 22 [Source:HGNC Symbol;Acc:HGNC:28189]	-2.9	-2
5566	*EFS*	10278	embryonal Fyn-associated substrate [Source:HGNC Symbol;Acc:HGNC:16898]	-2.9	-2

5567	*USP5*	8078	ubiquitin specific peptidase 5 [Source:HGNC Symbol;Acc:HGNC:12628]	-2.9	-2
5568	*PTK6*	5753	protein tyrosine kinase 6 [Source:HGNC Symbol;Acc:HGNC:9617]	-2.9	-2
5569	*ABCF2*	10061	ATP binding cassette subfamily F member 2 [Source:HGNC Symbol;Acc:HGNC:71]	-2.9	-2
5570	*MPV17L2*	84769	MPV17 mitochondrial inner membrane protein like 2 [Source:HGNC Symbol;Acc:HGNC:28177]	-2.9	-2
5571	*THBD*	7056	thrombomodulin [Source:HGNC Symbol;Acc:HGNC:11784]	-2.9	-2
5572	*ERG*	2078	ERG, ETS transcription factor [Source:HGNC Symbol;Acc:HGNC:3446]	-2.9	-2
5573	*ZNF283*	284349	zinc finger protein 283 [Source:HGNC Symbol;Acc:HGNC:13077]	-2.9	-2
5574	*NMI*	9111	N-myc and STAT interactor [Source:HGNC Symbol;Acc:HGNC:7854]	-2.9	-2
5575	*SNX14*	57231	sorting nexin 14 [Source:HGNC Symbol;Acc:HGNC:14977]	-2.9	-2
5576	*IDH3B*	3420	isocitrate dehydrogenase 3 (NAD(+)) beta [Source:HGNC Symbol;Acc:HGNC:5385]	-2.9	-2
5577	*NEBL*	10529	nebulette [Source:HGNC Symbol;Acc:HGNC:16932]	-2.9	-2
5578	*INPP5A*	3632	inositol polyphosphate-5-phosphatase A [Source:HGNC Symbol;Acc:HGNC:6076]	-2.9	-2
5579	*HSCB*	150274	HscB mitochondrial iron-sulfur cluster cochaperone [Source:HGNC Symbol;Acc:HGNC:28913]	-2.9	-2
5580	*ZNF672*	79894	zinc finger protein 672 [Source:HGNC Symbol;Acc:HGNC:26179]	-2.9	-2
5581	*MICALL1*	85377	MICAL like 1 [Source:HGNC Symbol;Acc:HGNC:29804]	-2.9	-2
5582	*AQP6*	363	aquaporin 6 [Source:HGNC Symbol;Acc:HGNC:639]	-2.9	-2
5583	*ATP8A1*	10396	ATPase phospholipid transporting 8A1 [Source:HGNC Symbol;Acc:HGNC:13531]	-2.9	-2
5584	*KIAA0196*	9897	KIAA0196 [Source:HGNC Symbol;Acc:HGNC:28984]	-2.9	-2
5585	*VWA5A*	4013	von Willebrand factor A domain containing 5A [Source:HGNC Symbol;Acc:HGNC:6658]	-2.9	-2
5586	*FAM174B*	400451	family with sequence similarity 174 member B [Source:HGNC Symbol;Acc:HGNC:34339]	-2.9	-2
5587	*INTS10*	55174	integrator complex subunit 10 [Source:HGNC Symbol;Acc:HGNC:25548]	-2.9	-2
5588	*PRPF8*	10594	pre-mRNA processing factor 8 [Source:HGNC Symbol;Acc:HGNC:17340]	-2.9	-2
5589	*KCNN4*	3783	potassium calcium-activated channel subfamily N member 4 [Source:HGNC Symbol;Acc:HGNC:6293]	-2.9	-2
5590	*GP2*	2813	glycoprotein 2 [Source:HGNC Symbol;Acc:HGNC:4441]	-2.9	-2
5591	*NATD1*	256302	N-acetyltransferase domain containing 1 [Source:HGNC Symbol;Acc:HGNC:30770]	-2.9	-2
5592	*DNAJC5G*	285126	DnaJ heat shock protein family (Hsp40) member C5 gamma [Source:HGNC Symbol;Acc:HGNC:24844]	-2.9	-2
5593	*CDKN1C*	1028	cyclin dependent kinase inhibitor 1C [Source:HGNC Symbol;Acc:HGNC:1786]	-2.9	-2
5594	*RUNDC3A*	10900	RUN domain containing 3A [Source:HGNC Symbol;Acc:HGNC:16984]	-2.9	-2
5595	*OR6M1*	390261	olfactory receptor family 6 subfamily M member 1 [Source:HGNC Symbol;Acc:HGNC:14711]	-2.9	-2
5596	*S100PBP*	64766	S100P binding protein [Source:HGNC Symbol;Acc:HGNC:25768]	-2.9	-2
5597	*YARS*	8565	tyrosyl-tRNA synthetase [Source:HGNC Symbol;Acc:HGNC:12840]	-2.9	-2
5598	*GAGE12B*	729428	G antigen 12B [Source:HGNC Symbol;Acc:HGNC:26779]	-2.9	-2
5599	*GNG2*	54331	G protein subunit gamma 2 [Source:HGNC Symbol;Acc:HGNC:4404]	-2.9	-2
5600	*FZD2*	2535	frizzled class receptor 2 [Source:HGNC Symbol;Acc:HGNC:4040]	-2.9	-2
5601	*NTRK3*	4916	neurotrophic receptor tyrosine kinase 3 [Source:HGNC Symbol;Acc:HGNC:8033]	-2.9	-2
5602	*UTY*	7404	ubiquitously transcribed tetratricopeptide repeat containing, Y-linked [Source:HGNC Symbol;Acc:HGNC:12638]	-2.9	-2
5603	*TNFRSF19*	55504	tumor necrosis factor receptor superfamily member 19 [Source:HGNC Symbol;Acc:HGNC:11915]	-2.9	-2
5604	*FANCA*	2175	Fanconi anemia complementation group A [Source:HGNC Symbol;Acc:HGNC:3582]	-3.0	-2
5605	*PSENEN*	55851	presenilin enhancer gamma-secretase subunit [Source:HGNC Symbol;Acc:HGNC:30100]	-3.0	-2
5606	*ZIM3*	114026	zinc finger imprinted 3 [Source:HGNC Symbol;Acc:HGNC:16366]	-3.0	-2
5607	*SDHA*	6389	succinate dehydrogenase complex flavoprotein subunit A [Source:HGNC Symbol;Acc:HGNC:10680]	-3.0	-2
5608	*ANKZF1*	55139	ankyrin repeat and zinc finger domain containing 1 [Source:HGNC Symbol;Acc:HGNC:25527]	-3.0	-2
5609	*DIRC2*	84925	disrupted in renal carcinoma 2 [Source:HGNC Symbol;Acc:HGNC:16628]	-3.0	-2
5610	*FNDC3B*	64778	fibronectin type III domain containing 3B [Source:HGNC Symbol;Acc:HGNC:24670]	-3.0	-2
5611	*FAM129B*	64855	family with sequence similarity 129 member B [Source:HGNC Symbol;Acc:HGNC:25282]	-3.0	-2
5612	*PTPN7*	5778	protein tyrosine phosphatase, non-receptor type 7 [Source:HGNC Symbol;Acc:HGNC:9659]	-3.0	-2
5613	*SMAD9*	4093	SMAD family member 9 [Source:HGNC Symbol;Acc:HGNC:6774]	-3.0	-2
5614	*TTC39A*	22996	tetratricopeptide repeat domain 39A [Source:HGNC Symbol;Acc:HGNC:18657]	-3.0	-2
5615	*TNFSF8*	944	tumor necrosis factor superfamily member 8 [Source:HGNC Symbol;Acc:HGNC:11938]	-3.0	-2
5616	*CCDC115*	84317	coiled-coil domain containing 115 [Source:HGNC Symbol;Acc:HGNC:28178]	-3.0	-2
5617	*C6orf141*	135398	chromosome 6 open reading frame 141 [Source:HGNC Symbol;Acc:HGNC:21351]	-3.0	-2
5618	*THAP9*	79725	THAP domain containing 9 [Source:HGNC Symbol;Acc:HGNC:23192]	-3.0	-2
5619	*NDUFA2*	4695	NADH:ubiquinone oxidoreductase subunit A2 [Source:HGNC Symbol;Acc:HGNC:7685]	-3.0	-2
5620	*C16orf45*	89927	chromosome 16 open reading frame 45 [Source:HGNC Symbol;Acc:HGNC:19213]	-3.0	-2
5621	*MPND*	84954	MPN domain containing [Source:HGNC Symbol;Acc:HGNC:25934]	-3.0	-2
5622	*SPTY2D1*	144108	SPT2 chromatin protein domain containing 1 [Source:HGNC Symbol;Acc:HGNC:26818]	-3.0	-2
5623	*C17orf104*	284071	chromosome 17 open reading frame 104	-3.0	-2
5624	*PPP1CB*	5500	protein phosphatase 1 catalytic subunit beta [Source:HGNC Symbol;Acc:HGNC:9282]	-3.0	-2
5625	*LURAP1L*	286343	leucine rich adaptor protein 1 like [Source:HGNC Symbol;Acc:HGNC:31452]	-3.0	-2
5626	*SQLE*	6713	squalene epoxidase [Source:HGNC Symbol;Acc:HGNC:11279]	-3.0	-2
5627	*LEF1*	51176	lymphoid enhancer binding factor 1 [Source:HGNC Symbol;Acc:HGNC:6551]	-3.0	-2
5628	*TLK2*	11011	tousled like kinase 2 [Source:HGNC Symbol;Acc:HGNC:11842]	-3.0	-2
5629	*SLC12A3*	6559	solute carrier family 12 member 3 [Source:HGNC Symbol;Acc:HGNC:10912]	-3.0	-2
5630	*OBSL1*	23363	obscurin-like 1 [Source:HGNC Symbol;Acc:HGNC:29092]	-3.0	-2
5631	*DDX43*	55510	DEAD-box helicase 43 [Source:HGNC Symbol;Acc:HGNC:18677]	-3.0	-2
5632	*PPP1R8*	5511	protein phosphatase 1 regulatory subunit 8 [Source:HGNC Symbol;Acc:HGNC:9296]	-3.0	-2
5633	*LIN7B*	64130	lin-7 homolog B, crumbs cell polarity complex component [Source:HGNC Symbol;Acc:HGNC:17788]	-3.0	-2
5634	*TP53*	7157	tumor protein p53 [Source:HGNC Symbol;Acc:HGNC:11998]	-3.0	-2
5635	*HACE1*	57531	HECT domain and ankyrin repeat containing E3 ubiquitin protein ligase 1 [Source:HGNC Symbol;Acc:HGNC:21033]	-3.0	-2
5636	*GLRX*	2745	glutaredoxin [Source:HGNC Symbol;Acc:HGNC:4330]	-3.0	-2
5637	*PRIM1*	5557	primase (DNA) subunit 1 [Source:HGNC Symbol;Acc:HGNC:9369]	-3.0	-2
5638	*MYD88*	4615	myeloid differentiation primary response 88 [Source:HGNC Symbol;Acc:HGNC:7562]	-3.0	-2

5639	*UBAC2*	337867	UBA domain containing 2 [Source:HGNC Symbol;Acc:HGNC:20486]	-3.0	-2
5640	*MB*	4151	myoglobin [Source:HGNC Symbol;Acc:HGNC:6915]	-3.0	-2
5641	*NANOS1*	340719	nanos C2HC-type zinc finger 1 [Source:HGNC Symbol;Acc:HGNC:23044]	-3.0	-2
5642	*DRG2*	1819	developmentally regulated GTP binding protein 2 [Source:HGNC Symbol;Acc:HGNC:3030]	-3.0	-2
5643	*FAM8A1*	51439	family with sequence similarity 8 member A1 [Source:HGNC Symbol;Acc:HGNC:16372]	-3.0	-2
5644	*ASB4*	51666	ankyrin repeat and SOCS box containing 4 [Source:HGNC Symbol;Acc:HGNC:16009]	-3.0	-2
5645	*EML5*	161436	echinoderm microtubule associated protein like 5 [Source:HGNC Symbol;Acc:HGNC:18197]	-3.0	-2
5646	*KIAA1210*	57481	KIAA1210 [Source:HGNC Symbol;Acc:HGNC:29218]	-3.0	-2
5647	*HDDC3*	374659	HD domain containing 3 [Source:HGNC Symbol;Acc:HGNC:30522]	-3.0	-2
5648	*SLBP*	7884	stem-loop binding protein [Source:HGNC Symbol;Acc:HGNC:10904]	-3.0	-2
5649	*UFD1L*	7353	ubiquitin fusion degradation 1 like (yeast) [Source:HGNC Symbol;Acc:HGNC:12520]	-3.0	-2
5650	*RHBDD2*	57414	rhomboid domain containing 2 [Source:HGNC Symbol;Acc:HGNC:23082]	-3.0	-2
5651	*ITM2C*	81618	integral membrane protein 2C [Source:HGNC Symbol;Acc:HGNC:6175]	-3.0	-2
5652	*SLC6A14*	11254	solute carrier family 6 member 14 [Source:HGNC Symbol;Acc:HGNC:11047]	-3.0	-2
5653	*NSDHL*	50814	NAD(P) dependent steroid dehydrogenase-like [Source:HGNC Symbol;Acc:HGNC:13398]	-3.0	-2
5654	*NDUFAF7*	55471	NADH:ubiquinone oxidoreductase complex assembly factor 7 [Source:HGNC Symbol;Acc:HGNC:28816]	-3.0	-2
5655	*ZNF345*	25850	zinc finger protein 345 [Source:HGNC Symbol;Acc:HGNC:16367]	-3.0	-2
5656	*RAB3GAP1*	22930	RAB3 GTPase activating protein catalytic subunit 1 [Source:HGNC Symbol;Acc:HGNC:17063]	-3.0	-2
5657	*LATS1*	9113	large tumor suppressor kinase 1 [Source:HGNC Symbol;Acc:HGNC:6514]	-3.0	-2
5658	*SLC14A1*	6563	solute carrier family 14 member 1 (Kidd blood group) [Source:HGNC Symbol;Acc:HGNC:10918]	-3.0	-2
5659	*DGKE*	8526	diacylglycerol kinase epsilon [Source:HGNC Symbol;Acc:HGNC:2852]	-3.0	-2
5660	*HCCS*	3052	holocytochrome c synthase [Source:HGNC Symbol;Acc:HGNC:4837]	-3.0	-2
5661	*LGSN*	51557	lengsin, lens protein with glutamine synthetase domain [Source:HGNC Symbol;Acc:HGNC:21016]	-3.0	-2
5662	*POLE4*	56655	polymerase (DNA) epsilon 4, accessory subunit [Source:HGNC Symbol;Acc:HGNC:18755]	-3.0	-2
5663	*VSIG1*	340547	V-set and immunoglobulin domain containing 1 [Source:HGNC Symbol;Acc:HGNC:28675]	-3.0	-2
5664	*ZNF320*	162967	zinc finger protein 320 [Source:HGNC Symbol;Acc:HGNC:13842]	-3.0	-2
5665	*MAP2K2*	5605	mitogen-activated protein kinase kinase 2 [Source:HGNC Symbol;Acc:HGNC:6842]	-3.0	-2
5666	*MSI2*	124540	musashi RNA binding protein 2 [Source:HGNC Symbol;Acc:HGNC:18585]	-3.0	-2
5667	*MIEF1*	54471	mitochondrial elongation factor 1 [Source:HGNC Symbol;Acc:HGNC:25979]	-3.0	-2
5668	*FAM134A*	79137	family with sequence similarity 134 member A [Source:HGNC Symbol;Acc:HGNC:28450]	-3.0	-2
5669	*TTC8*	123016	tetratricopeptide repeat domain 8 [Source:HGNC Symbol;Acc:HGNC:20087]	-3.0	-2
5670	*GADD45GIP1*	90480	GADD45G interacting protein 1 [Source:HGNC Symbol;Acc:HGNC:29996]	-3.0	-2
5671	*MSS51*	118490	MSS51 mitochondrial translational activator [Source:HGNC Symbol;Acc:HGNC:21000]	-3.0	-2
5672	*PDE6B*	5158	phosphodiesterase 6B [Source:HGNC Symbol;Acc:HGNC:8786]	-3.0	-2
5673	*FER1L5*	90342	fer-1 like family member 5 [Source:HGNC Symbol;Acc:HGNC:19044]	-3.0	-2
5674	*ALG10*	84920	ALG10, alpha-1,2-glucosyltransferase [Source:HGNC Symbol;Acc:HGNC:23162]	-3.0	-2
5675	*TMEM55A*	55529	transmembrane protein 55A [Source:HGNC Symbol;Acc:HGNC:25452]	-3.0	-2
5676	*VRK1*	7443	vaccinia related kinase 1 [Source:HGNC Symbol;Acc:HGNC:12718]	-3.0	-2
5677	*TNNT2*	7139	troponin T2, cardiac type [Source:HGNC Symbol;Acc:HGNC:11949]	-3.0	-2
5678	*BRWD1*	54014	bromodomain and WD repeat domain containing 1 [Source:HGNC Symbol;Acc:HGNC:12760]	-3.0	-2
5679	*TMEM60*	85025	transmembrane protein 60 [Source:HGNC Symbol;Acc:HGNC:21754]	-3.0	-2
5680	*STAG1*	10274	stromal antigen 1 [Source:HGNC Symbol;Acc:HGNC:11354]	-3.0	-2
5681	*CADM1*	23705	cell adhesion molecule 1 [Source:HGNC Symbol;Acc:HGNC:5951]	-3.0	-2
5682	*TSPAN4*	7106	tetraspanin 4 [Source:HGNC Symbol;Acc:HGNC:11859]	-3.0	-2
5683	*SVEP1*	79987	sushi, von Willebrand factor type A, EGF and pentraxin domain containing 1 [Source:HGNC Symbol;Acc:HGNC:15985]	-3.0	-2
5684	*CCDC36*	339834	coiled-coil domain containing 36 [Source:HGNC Symbol;Acc:HGNC:27945]	-3.0	-2
5685	*DGCR6L*	85359	DiGeorge syndrome critical region gene 6-like [Source:HGNC Symbol;Acc:HGNC:18551]	-3.0	-2
5686	*RINL*	126432	Ras and Rab interactor like [Source:HGNC Symbol;Acc:HGNC:24795]	-3.0	-2
5687	*HTRA1*	5654	HtrA serine peptidase 1 [Source:HGNC Symbol;Acc:HGNC:9476]	-3.0	-2
5688	*HSD17B1*	3292	hydroxysteroid 17-beta dehydrogenase 1 [Source:HGNC Symbol;Acc:HGNC:5210]	-3.0	-2
5689	*SGTB*	54557	small glutamine rich tetratricopeptide repeat containing beta [Source:HGNC Symbol;Acc:HGNC:23567]	-3.0	-2
5690	*ITGA3*	3675	integrin subunit alpha 3 [Source:HGNC Symbol;Acc:HGNC:6139]	-3.0	-2
5691	*USP50*	373509	ubiquitin specific peptidase 50 [Source:HGNC Symbol;Acc:HGNC:20079]	-3.0	-2
5692	*ZNF358*	140467	zinc finger protein 358 [Source:HGNC Symbol;Acc:HGNC:16838]	-3.0	-2
5693	*SIDT1*	54847	SID1 transmembrane family member 1 [Source:HGNC Symbol;Acc:HGNC:25967]	-3.0	-2
5694	*SEL1L2*	80343	SEL1L2 ERAD E3 ligase adaptor subunit [Source:HGNC Symbol;Acc:HGNC:15897]	-3.0	-2
5695	*CDSN*	1041	corneodesmosin [Source:HGNC Symbol;Acc:HGNC:1802]	-3.0	-2
5696	*CLDN10*	9071	claudin 10 [Source:HGNC Symbol;Acc:HGNC:2033]	-3.0	-2
5697	*DZIP1*	22873	DAZ interacting zinc finger protein 1 [Source:HGNC Symbol;Acc:HGNC:20908]	-3.0	-2
5698	*CFI*	3426	complement factor I [Source:HGNC Symbol;Acc:HGNC:5394]	-3.0	-2
5699	*DESI1*	27351	desumoylating isopeptidase 1 [Source:HGNC Symbol;Acc:HGNC:24577]	-3.0	-2
5700	*ANKMY2*	57037	ankyrin repeat and MYND domain containing 2 [Source:HGNC Symbol;Acc:HGNC:25370]	-3.0	-2
5701	*IFT80*	57560	intraflagellar transport 80 [Source:HGNC Symbol;Acc:HGNC:29262]	-3.0	-2
5702	*INTS4*	92105	integrator complex subunit 4 [Source:HGNC Symbol;Acc:HGNC:25048]	-3.0	-2
5703	*ZSCAN21*	7589	zinc finger and SCAN domain containing 21 [Source:HGNC Symbol;Acc:HGNC:13104]	-3.0	-2
5704	*CARD14*	79092	caspase recruitment domain family member 14 [Source:HGNC Symbol;Acc:HGNC:16446]	-3.0	-2
5705	*ETV4*	2118	ETS variant 4 [Source:HGNC Symbol;Acc:HGNC:3493]	-3.0	-2
5706	*KLHL12*	59349	kelch like family member 12 [Source:HGNC Symbol;Acc:HGNC:19360]	-3.0	-2
5707	*MDFI*	4188	MyoD family inhibitor [Source:HGNC Symbol;Acc:HGNC:6967]	-3.1	-2
5708	*EVI5*	7813	ecotropic viral integration site 5 [Source:HGNC Symbol;Acc:HGNC:3501]	-3.1	-2
5709	*PYM1*	84305	PYM homolog 1, exon junction complex associated factor [Source:HGNC Symbol;Acc:HGNC:30258]	-3.1	-2
5710	*SPTBN2*	6712	spectrin beta, non-erythrocytic 2 [Source:HGNC Symbol;Acc:HGNC:11276]	-3.1	-2
5711	*TTLL9*	164395	tubulin tyrosine ligase like 9 [Source:HGNC Symbol;Acc:HGNC:16118]	-3.1	-2

5712	*PDPR*	55066	pyruvate dehydrogenase phosphatase regulatory subunit [Source:HGNC Symbol;Acc:HGNC:30264]	-3.1	-2
5713	*BDH1*	622	3-hydroxybutyrate dehydrogenase, type 1 [Source:HGNC Symbol;Acc:HGNC:1027]	-3.1	-2
5714	*ASB8*	140461	ankyrin repeat and SOCS box containing 8 [Source:HGNC Symbol;Acc:HGNC:17183]	-3.1	-2
5715	*CDCP1*	64866	CUB domain containing protein 1 [Source:HGNC Symbol;Acc:HGNC:24357]	-3.1	-2
5716	*RAMP1*	10267	receptor activity modifying protein 1 [Source:HGNC Symbol;Acc:HGNC:9843]	-3.1	-2
5717	*ZBP1*	81030	Z-DNA binding protein 1 [Source:HGNC Symbol;Acc:HGNC:16176]	-3.1	-2
5718	*CDADC1*	81602	cytidine and dCMP deaminase domain containing 1 [Source:HGNC Symbol;Acc:HGNC:20299]	-3.1	-2
5719	*PCGF2*	7703	polycomb group ring finger 2 [Source:HGNC Symbol;Acc:HGNC:12929]	-3.1	-2
5720	*RDH5*	5959	retinol dehydrogenase 5 [Source:HGNC Symbol;Acc:HGNC:9940]	-3.1	-2
5721	*POP5*	51367	POP5 homolog, ribonuclease P/MRP subunit [Source:HGNC Symbol;Acc:HGNC:17689]	-3.1	-2
5722	*AFAP1*	60312	actin filament associated protein 1 [Source:HGNC Symbol;Acc:HGNC:24017]	-3.1	-2
5723	*JPH2*	57158	junctophilin 2 [Source:HGNC Symbol;Acc:HGNC:14202]	-3.1	-2
5724	*OR9Q2*	219957	olfactory receptor family 9 subfamily Q member 2 [Source:HGNC Symbol;Acc:HGNC:15328]	-3.1	-2
5725	*SLC38A8*	146167	solute carrier family 38 member 8 [Source:HGNC Symbol;Acc:HGNC:32434]	-3.1	-2
5726	*PCMTD1*	115294	protein-L-isoaspartate (D-aspartate) O-methyltransferase domain containing 1 [Source:HGNC Symbol;Acc:HGNC:30483]	-3.1	-2
5727	*FLAD1*	80308	flavin adenine dinucleotide synthetase 1 [Source:HGNC Symbol;Acc:HGNC:24671]	-3.1	-2
5728	*DPH6*	89978	diphthamine biosynthesis 6 [Source:HGNC Symbol;Acc:HGNC:30543]	-3.1	-2
5729	*MLPH*	79083	melanophilin [Source:HGNC Symbol;Acc:HGNC:29643]	-3.1	-2
5730	*IFIH1*	64135	interferon induced with helicase C domain 1 [Source:HGNC Symbol;Acc:HGNC:18873]	-3.1	-2
5731	*CHAF1A*	10036	chromatin assembly factor 1 subunit A [Source:HGNC Symbol;Acc:HGNC:1910]	-3.1	-2
5732	*PTPRF*	5792	protein tyrosine phosphatase, receptor type F [Source:HGNC Symbol;Acc:HGNC:9670]	-3.1	-2
5733	*ZNF304*	57343	zinc finger protein 304 [Source:HGNC Symbol;Acc:HGNC:13505]	-3.1	-2
5734	*ARRDC4*	91947	arrestin domain containing 4 [Source:HGNC Symbol;Acc:HGNC:28087]	-3.1	-2
5735	*VRK2*	7444	vaccinia related kinase 2 [Source:HGNC Symbol;Acc:HGNC:12719]	-3.1	-2
5736	*ITPKB*	3707	inositol-trisphosphate 3-kinase B [Source:HGNC Symbol;Acc:HGNC:6179]	-3.1	-2
5737	*INSIG1*	3638	insulin induced gene 1 [Source:HGNC Symbol;Acc:HGNC:6083]	-3.1	-2
5738	*BET1L*	51272	Bet1 golgi vesicular membrane trafficking protein like [Source:HGNC Symbol;Acc:HGNC:19348]	-3.1	-2
5739	*SIRT5*	23408	sirtuin 5 [Source:HGNC Symbol;Acc:HGNC:14933]	-3.1	-2
5740	*ANKLE1*	126549	ankyrin repeat and LEM domain containing 1 [Source:HGNC Symbol;Acc:HGNC:26812]	-3.1	-2
5741	*CRCP*	27297	CGRP receptor component [Source:HGNC Symbol;Acc:HGNC:17888]	-3.1	-2
5742	*MOSPD1*	56180	motile sperm domain containing 1 [Source:HGNC Symbol;Acc:HGNC:25235]	-3.1	-2
5743	*YKT6*	10652	YKT6 v-SNARE homolog (S. cerevisiae) [Source:HGNC Symbol;Acc:HGNC:16959]	-3.1	-2
5744	*C1orf210*	149466	chromosome 1 open reading frame 210 [Source:HGNC Symbol;Acc:HGNC:28755]	-3.1	-2
5745	*HRSP12*	10247	Reactive Intermediate Imine Deaminase A Homolog	-3.1	-2
5746	*RBM23*	55147	RNA binding motif protein 23 [Source:HGNC Symbol;Acc:HGNC:20155]	-3.1	-2
5747	*C19orf60*	55049	chromosome 19 open reading frame 60 [Source:HGNC Symbol;Acc:HGNC:26098]	-3.1	-2
5748	*MDM2*	4193	MDM2 proto-oncogene [Source:HGNC Symbol;Acc:HGNC:6973]	-3.1	-2
5749	*AVEN*	57099	apoptosis and caspase activation inhibitor [Source:HGNC Symbol;Acc:HGNC:13509]	-3.1	-2
5750	*CERCAM*	51148	cerebral endothelial cell adhesion molecule [Source:HGNC Symbol;Acc:HGNC:23723]	-3.1	-2
5751	*SLC5A4*	6527	solute carrier family 5 member 4 [Source:HGNC Symbol;Acc:HGNC:11039]	-3.1	-2
5752	*DSC3*	1825	desmocollin 3 [Source:HGNC Symbol;Acc:HGNC:3037]	-3.1	-2
5753	*CARD17*	440068	caspase recruitment domain family member 17 [Source:HGNC Symbol;Acc:HGNC:33827]	-3.1	-2
5754	*YAF2*	10138	YY1 associated factor 2 [Source:HGNC Symbol;Acc:HGNC:17363]	-3.1	-2
5755	*DOCK4*	9732	dedicator of cytokinesis 4 [Source:HGNC Symbol;Acc:HGNC:19192]	-3.1	-2
5756	*PLEKHM3*	389072	pleckstrin homology domain containing M3 [Source:HGNC Symbol;Acc:HGNC:34006]	-3.1	-2
5757	*AATF*	26574	apoptosis antagonizing transcription factor [Source:HGNC Symbol;Acc:HGNC:19235]	-3.1	-2
5758	*ANKRD45*	339416	ankyrin repeat domain 45 [Source:HGNC Symbol;Acc:HGNC:24786]	-3.1	-2
5759	*ARHGEF15*	22899	Rho guanine nucleotide exchange factor 15 [Source:HGNC Symbol;Acc:HGNC:15590]	-3.1	-2
5760	*RBM43*	375287	RNA binding motif protein 43 [Source:HGNC Symbol;Acc:HGNC:24790]	-3.1	-2
5761	*TTC28*	23331	tetratricopeptide repeat domain 28 [Source:HGNC Symbol;Acc:HGNC:29179]	-3.1	-2
5762	*IBA57*	200205	IBA57 homolog, iron-sulfur cluster assembly [Source:HGNC Symbol;Acc:HGNC:27302]	-3.1	-2
5763	*DUXA*	503835	double homeobox A [Source:HGNC Symbol;Acc:HGNC:32179]	-3.1	-2
5764	*HPCAL1*	3241	hippocalcin like 1 [Source:HGNC Symbol;Acc:HGNC:5145]	-3.1	-2
5765	*SLMAP*	7871	sarcolemma associated protein [Source:HGNC Symbol;Acc:HGNC:16643]	-3.1	-2
5766	*VAC14*	55697	Vac14, PIKFYVE complex component [Source:HGNC Symbol;Acc:HGNC:25507]	-3.1	-2
5767	*ATP5D*	513	ATP synthase, H+ transporting, mitochondrial F1 complex, delta subunit [Source:HGNC Symbol;Acc:HGNC:837]	-3.1	-2
5768	*PLEKHA5*	54477	pleckstrin homology domain containing A5 [Source:HGNC Symbol;Acc:HGNC:30036]	-3.1	-2
5769	*GULP1*	51454	GULP, engulfment adaptor PTB domain containing 1 [Source:HGNC Symbol;Acc:HGNC:18649]	-3.1	-2
5770	*SLC26A2*	1836	solute carrier family 26 member 2 [Source:HGNC Symbol;Acc:HGNC:10994]	-3.1	-2
5771	*NRSN1*	140767	neurensin 1 [Source:HGNC Symbol;Acc:HGNC:17881]	-3.1	-2
5772	*NOX5*	79400	NADPH oxidase, EF-hand calcium binding domain 5 [Source:HGNC Symbol;Acc:HGNC:14874]	-3.1	-2
5773	*SPESP1*	246777	sperm equatorial segment protein 1 [Source:HGNC Symbol;Acc:HGNC:15570]	-3.1	-2
5774	*IFT22*	64792	intraflagellar transport 22 [Source:HGNC Symbol;Acc:HGNC:21895]	-3.1	-2
5775	*UPK3BL*	100134938	uroplakin 3B-like [Source:HGNC Symbol;Acc:HGNC:37278]	-3.1	-2
5776	*ZFP42*	132625	ZFP42 zinc finger protein [Source:HGNC Symbol;Acc:HGNC:30949]	-3.1	-2
5777	*STAC2*	342667	SH3 and cysteine rich domain 2 [Source:HGNC Symbol;Acc:HGNC:23990]	-3.1	-2
5778	*ORMDL1*	94101	ORMDL sphingolipid biosynthesis regulator 1 [Source:HGNC Symbol;Acc:HGNC:16036]	-3.1	-2
5779	*CSDC2*	27254	cold shock domain containing C2 [Source:HGNC Symbol;Acc:HGNC:30359]	-3.1	-2
5780	*DMBX1*	127343	diencephalon/mesencephalon homeobox 1 [Source:HGNC Symbol;Acc:HGNC:19026]	-3.1	-2
5781	*SAMD8*	142891	sterile alpha motif domain containing 8 [Source:HGNC Symbol;Acc:HGNC:26320]	-3.1	-2
5782	*CTSV*	1515	cathepsin V [Source:HGNC Symbol;Acc:HGNC:2538]	-3.1	-2
5783	*MEF2C*	4208	myocyte enhancer factor 2C [Source:HGNC Symbol;Acc:HGNC:6996]	-3.1	-2
5784	*HEATR1*	55127	HEAT repeat containing 1 [Source:HGNC Symbol;Acc:HGNC:25517]	-3.1	-2
5785	*LGALS8*	3964	galectin 8 [Source:HGNC Symbol;Acc:HGNC:6569]	-3.1	-2
5786	*VPS33A*	65082	VPS33A, CORVET/HOPS core subunit [Source:HGNC Symbol;Acc:HGNC:18179]	-3.1	-2
5787	*DTX3L*	151636	deltex 3 like, E3 ubiquitin ligase [Source:HGNC Symbol;Acc:HGNC:30323]	-3.1	-2

5788	*HAUS2*	55142	HAUS augmin like complex subunit 2 [Source:HGNC Symbol;Acc:HGNC:25530]	-3.1	-2
5789	*RAD54B*	25788	RAD54 homolog B (S. cerevisiae) [Source:HGNC Symbol;Acc:HGNC:17228]	-3.1	-2
5790	*G6PC*	2538	glucose-6-phosphatase catalytic subunit [Source:HGNC Symbol;Acc:HGNC:4056]	-3.1	-2
5791	*HSPA1A*	3303	heat shock protein family A (Hsp70) member 1A [Source:HGNC Symbol;Acc:HGNC:5232]	-3.1	-2
5792	*DDAH2*	23564	dimethylarginine dimethylaminohydrolase 2 [Source:HGNC Symbol;Acc:HGNC:2716]	-3.1	-2
5793	*BEST1*	7439	bestrophin 1 [Source:HGNC Symbol;Acc:HGNC:12703]	-3.1	-2
5794	*PRKAG2*	51422	protein kinase AMP-activated non-catalytic subunit gamma 2 [Source:HGNC Symbol;Acc:HGNC:9386]	-3.1	-2
5795	*RXRB*	6257	retinoid X receptor beta [Source:HGNC Symbol;Acc:HGNC:10478]	-3.1	-2
5796	*USP33*	23032	ubiquitin specific peptidase 33 [Source:HGNC Symbol;Acc:HGNC:20059]	-3.1	-2
5797	*RAB11FIP1*	80223	RAB11 family interacting protein 1 [Source:HGNC Symbol;Acc:HGNC:30265]	-3.1	-2
5798	*ZNF207*	7756	zinc finger protein 207 [Source:HGNC Symbol;Acc:HGNC:12998]	-3.1	-2
5799	*PLA2G16*	11145	phospholipase A2 group XVI [Source:HGNC Symbol;Acc:HGNC:17825]	-3.1	-2
5800	*MAEL*	84944	maelstrom spermatogenic transposon silencer [Source:HGNC Symbol;Acc:HGNC:25929]	-3.1	-2
5801	*ITFG2*	55846	integrin alpha FG-GAP repeat containing 2 [Source:HGNC Symbol;Acc:HGNC:30879]	-3.1	-2
5802	*PRR27*	401137	proline rich 27 [Source:HGNC Symbol;Acc:HGNC:33193]	-3.1	-2
5803	*STAC3*	246329	SH3 and cysteine rich domain 3 [Source:HGNC Symbol;Acc:HGNC:28423]	-3.1	-2
5804	*COX6B2*	125965	cytochrome c oxidase subunit 6B2 [Source:HGNC Symbol;Acc:HGNC:24380]	-3.2	-2
5805	*STK25*	10494	serine/threonine kinase 25 [Source:HGNC Symbol;Acc:HGNC:11404]	-3.2	-2
5806	*MRO*	83876	maestro [Source:HGNC Symbol;Acc:HGNC:24121]	-3.2	-2
5807	*AP4B1*	10717	adaptor related protein complex 4 beta 1 subunit [Source:HGNC Symbol;Acc:HGNC:572]	-3.2	-2
5808	*ACSM1*	116285	acyl-CoA synthetase medium-chain family member 1 [Source:HGNC Symbol;Acc:HGNC:18049]	-3.2	-2
5809	*TAC4*	255061	tachykinin 4 (hemokinin) [Source:HGNC Symbol;Acc:HGNC:16641]	-3.2	-2
5810	*LDB3*	11155	LIM domain binding 3 [Source:HGNC Symbol;Acc:HGNC:15710]	-3.2	-2
5811	*UNK*	85451	unkempt family zinc finger [Source:HGNC Symbol;Acc:HGNC:29369]	-3.2	-2
5812	*PDXP*	57026	pyridoxal phosphatase [Source:HGNC Symbol;Acc:HGNC:30259]	-3.2	-2
5813	*B3GNTL1*	146712	UDP-GlcNAc:betaGal beta-1,3-N-acetylglucosaminyltransferase-like 1 [Source:HGNC Symbol;Acc:HGNC:21727]	-3.2	-2
5814	*MT2A*	4502	metallothionein 2A [Source:HGNC Symbol;Acc:HGNC:7406]	-3.2	-2
5815	*MANEAL*	149175	mannosidase endo-alpha like [Source:HGNC Symbol;Acc:HGNC:26452]	-3.2	-2
5816	*FBXO42*	54455	F-box protein 42 [Source:HGNC Symbol;Acc:HGNC:29249]	-3.2	-2
5817	*FAAP24*	91442	Fanconi anemia core complex associated protein 24 [Source:HGNC Symbol;Acc:HGNC:28467]	-3.2	-2
5818	*SEPT10*	151011	septin 10 [Source:HGNC Symbol;Acc:HGNC:14349]	-3.2	-2
5819	*ATF6B*	1388	activating transcription factor 6 beta [Source:HGNC Symbol;Acc:HGNC:2349]	-3.2	-2
5820	*CUL4A*	8451	cullin 4A [Source:HGNC Symbol;Acc:HGNC:2554]	-3.2	-2
5821	*PCYOX1*	51449	prenylcysteine oxidase 1 [Source:HGNC Symbol;Acc:HGNC:20588]	-3.2	-2
5822	*CD74*	972	CD74 molecule [Source:HGNC Symbol;Acc:HGNC:1697]	-3.2	-2
5823	*ZNF277*	11179	zinc finger protein 277 [Source:HGNC Symbol;Acc:HGNC:13070]	-3.2	-2
5824	*TMTC1*	83857	transmembrane and tetratricopeptide repeat containing 1 [Source:HGNC Symbol;Acc:HGNC:24099]	-3.2	-2
5825	*AARSD1*	80755	alanyl-tRNA synthetase domain containing 1 [Source:HGNC Symbol;Acc:HGNC:28417]	-3.2	-2
5826	*B3GNT6*	192134	UDP-GlcNAc:betaGal beta-1,3-N-acetylglucosaminyltransferase 6 [Source:HGNC Symbol;Acc:HGNC:24141]	-3.2	-2
5827	*MR1*	3140	major histocompatibility complex, class I-related [Source:HGNC Symbol;Acc:HGNC:4975]	-3.2	-2
5828	*PLEKHH1*	57475	pleckstrin homology, MyTH4 and FERM domain containing H1 [Source:HGNC Symbol;Acc:HGNC:17733]	-3.2	-2
5829	*PPP2R5A*	5525	protein phosphatase 2 regulatory subunit B'alpha [Source:HGNC Symbol;Acc:HGNC:9309]	-3.2	-2
5830	*AMPD3*	272	adenosine monophosphate deaminase 3 [Source:HGNC Symbol;Acc:HGNC:470]	-3.2	-2
5831	*PHKG2*	5261	phosphorylase kinase catalytic subunit gamma 2 [Source:HGNC Symbol;Acc:HGNC:8931]	-3.2	-2
5832	*PALB2*	79728	partner and localizer of BRCA2 [Source:HGNC Symbol;Acc:HGNC:26144]	-3.2	-2
5833	*ZNF695*	57116	zinc finger protein 695 [Source:HGNC Symbol;Acc:HGNC:30954]	-3.2	-2
5834	*C2orf68*	388969	chromosome 2 open reading frame 68 [Source:HGNC Symbol;Acc:HGNC:34353]	-3.2	-2
5835	*PTOV1*	53635	prostate tumor overexpressed 1 [Source:HGNC Symbol;Acc:HGNC:9632]	-3.2	-2
5836	*VMA21*	203547	VMA21 vacuolar H+-ATPase homolog (S. cerevisiae) [Source:HGNC Symbol;Acc:HGNC:22082]	-3.2	-2
5837	*DDHD1*	80821	DDHD domain containing 1 [Source:HGNC Symbol;Acc:HGNC:19714]	-3.2	-2
5838	*LSG1*	55341	large 60S subunit nuclear export GTPase 1 [Source:HGNC Symbol;Acc:HGNC:25652]	-3.2	-2
5839	*QPCT*	25797	glutaminyl-peptide cyclotransferase [Source:HGNC Symbol;Acc:HGNC:9753]	-3.2	-2
5840	*RAD51*	5888	RAD51 recombinase [Source:HGNC Symbol;Acc:HGNC:9817]	-3.2	-2
5841	*CDCA4*	55038	cell division cycle associated 4 [Source:HGNC Symbol;Acc:HGNC:14625]	-3.2	-2
5842	*SLC41A2*	84102	solute carrier family 41 member 2 [Source:HGNC Symbol;Acc:HGNC:31045]	-3.2	-2
5843	*CAP2*	10486	CAP, adenylate cyclase-associated protein, 2 (yeast) [Source:HGNC Symbol;Acc:HGNC:20039]	-3.2	-2
5844	*TBC1D7*	51256	TBC1 domain family member 7 [Source:HGNC Symbol;Acc:HGNC:21066]	-3.2	-2
5845	*SYNRG*	11276	synergin, gamma [Source:HGNC Symbol;Acc:HGNC:557]	-3.2	-2
5846	*RNMTL1*	55178	Mitochondrial RRNA Methyltransferase 3	-3.2	-2
5847	*UXT*	8409	ubiquitously expressed prefoldin like chaperone [Source:HGNC Symbol;Acc:HGNC:12641]	-3.2	-2
5848	*B4GALT5*	9334	beta-1,4-galactosyltransferase 5 [Source:HGNC Symbol;Acc:HGNC:928]	-3.2	-2
5849	*RDX*	5962	radixin [Source:HGNC Symbol;Acc:HGNC:9944]	-3.2	-2
5850	*SRRM4*	84530	serine/arginine repetitive matrix 4 [Source:HGNC Symbol;Acc:HGNC:29389]	-3.2	-2
5851	*SCAMP1*	9522	secretory carrier membrane protein 1 [Source:HGNC Symbol;Acc:HGNC:10563]	-3.2	-2
5852	*INIP*	58493	INTS3 and NABP interacting protein [Source:HGNC Symbol;Acc:HGNC:24994]	-3.2	-2
5853	*CERS5*	91012	ceramide synthase 5 [Source:HGNC Symbol;Acc:HGNC:23749]	-3.2	-2
5854	*DUSP26*	78986	dual specificity phosphatase 26 (putative) [Source:HGNC Symbol;Acc:HGNC:28161]	-3.2	-2
5855	*KCNA7*	3743	potassium voltage-gated channel subfamily A member 7 [Source:HGNC Symbol;Acc:HGNC:6226]	-3.2	-2
5856	*FAM173B*	134145	family with sequence similarity 173 member B [Source:HGNC Symbol;Acc:HGNC:27029]	-3.2	-2
5857	*HNRNPUL1*	11100	heterogeneous nuclear ribonucleoprotein U like 1 [Source:HGNC Symbol;Acc:HGNC:17011]	-3.2	-2
5858	*SPRYD3*	84926	SPRY domain containing 3 [Source:HGNC Symbol;Acc:HGNC:25920]	-3.2	-2
5859	*SCN2B*	6327	sodium voltage-gated channel beta subunit 2 [Source:HGNC Symbol;Acc:HGNC:10589]	-3.2	-2
5860	*GBA*	2629	glucosylceramidase beta [Source:HGNC Symbol;Acc:HGNC:4177]	-3.2	-2

5861	*CHPF*	79586	chondroitin polymerizing factor [Source:HGNC Symbol;Acc:HGNC:24291]	-3.2	-2
5862	*CPT2*	1376	carnitine palmitoyltransferase 2 [Source:HGNC Symbol;Acc:HGNC:2330]	-3.2	-2
5863	*TNRC6B*	23112	trinucleotide repeat containing 6B [Source:HGNC Symbol;Acc:HGNC:29190]	-3.2	-2
5864	*TAF1A*	9015	TATA-box binding protein associated factor, RNA polymerase I subunit A [Source:HGNC Symbol;Acc:HGNC:11532]	-3.2	-2
5865	*SVOP*	55530	SV2 related protein [Source:HGNC Symbol;Acc:HGNC:25417]	-3.2	-2
5866	*FASTKD2*	22868	FAST kinase domains 2 [Source:HGNC Symbol;Acc:HGNC:29160]	-3.2	-2
5867	*CFB*	629	complement factor B [Source:HGNC Symbol;Acc:HGNC:1037]	-3.2	-2
5868	*UPK1B*	7348	uroplakin 1B [Source:HGNC Symbol;Acc:HGNC:12578]	-3.2	-2
5869	*OAS3*	4940	2'-5'-oligoadenylate synthetase 3 [Source:HGNC Symbol;Acc:HGNC:8088]	-3.2	-2
5870	*MIPOL1*	145282	mirror-image polydactyly 1 [Source:HGNC Symbol;Acc:HGNC:21460]	-3.2	-2
5871	*RASSF8*	11228	Ras association domain family member 8 [Source:HGNC Symbol;Acc:HGNC:13232]	-3.2	-2
5872	*SUPT7L*	9913	SPT7-like STAGA complex gamma subunit [Source:HGNC Symbol;Acc:HGNC:30632]	-3.2	-2
5873	*GUCD1*	83606	guanylyl cyclase domain containing 1 [Source:HGNC Symbol;Acc:HGNC:14237]	-3.2	-2
5874	*ATPAF1*	64756	ATP synthase mitochondrial F1 complex assembly factor 1 [Source:HGNC Symbol;Acc:HGNC:18803]	-3.2	-2
5875	*PLPP2*	8612	phospholipid phosphatase 2 [Source:HGNC Symbol;Acc:HGNC:9230]	-3.2	-2
5876	*OSTM1*	28962	osteopetrosis associated transmembrane protein 1 [Source:HGNC Symbol;Acc:HGNC:21652]	-3.2	-2
5877	*ALDH5A1*	7915	aldehyde dehydrogenase 5 family member A1 [Source:HGNC Symbol;Acc:HGNC:408]	-3.2	-2
5878	*RYR1*	6261	ryanodine receptor 1 [Source:HGNC Symbol;Acc:HGNC:10483]	-3.2	-2
5879	*NUDT7*	283927	nudix hydrolase 7 [Source:HGNC Symbol;Acc:HGNC:8054]	-3.2	-2
5880	*TMEM14A*	28978	transmembrane protein 14A [Source:HGNC Symbol;Acc:HGNC:21076]	-3.3	-2
5881	*DNAJC3*	5611	DnaJ heat shock protein family (Hsp40) member C3 [Source:HGNC Symbol;Acc:HGNC:9439]	-3.3	-2
5882	*MTHFD1L*	25902	methylenetetrahydrofolate dehydrogenase (NADP+ dependent) 1-like [Source:HGNC Symbol;Acc:HGNC:21055]	-3.3	-2
5883	*SCLT1*	132320	sodium channel and clathrin linker 1 [Source:HGNC Symbol;Acc:HGNC:26406]	-3.3	-2
5884	*ARPP19*	10776	cAMP regulated phosphoprotein 19 [Source:HGNC Symbol;Acc:HGNC:16967]	-3.3	-2
5885	*EMC2*	9694	ER membrane protein complex subunit 2 [Source:HGNC Symbol;Acc:HGNC:28963]	-3.3	-2
5886	*ADK*	132	adenosine kinase [Source:HGNC Symbol;Acc:HGNC:257]	-3.3	-2
5887	*RRAGD*	58528	Ras related GTP binding D [Source:HGNC Symbol;Acc:HGNC:19903]	-3.3	-2
5888	*FRMD4B*	23150	FERM domain containing 4B [Source:HGNC Symbol;Acc:HGNC:24886]	-3.3	-2
5889	*ISLR*	3671	immunoglobulin superfamily containing leucine rich repeat [Source:HGNC Symbol;Acc:HGNC:6133]	-3.3	-2
5890	*ZNF786*	136051	zinc finger protein 786 [Source:HGNC Symbol;Acc:HGNC:21806]	-3.3	-2
5891	*APRT*	353	adenine phosphoribosyltransferase [Source:HGNC Symbol;Acc:HGNC:626]	-3.3	-2
5892	*RNF168*	165918	ring finger protein 168 [Source:HGNC Symbol;Acc:HGNC:26661]	-3.3	-2
5893	*TMEM219*	124446	transmembrane protein 219 [Source:HGNC Symbol;Acc:HGNC:25201]	-3.3	-2
5894	*ATP9B*	374868	ATPase phospholipid transporting 9B (putative) [Source:HGNC Symbol;Acc:HGNC:13541]	-3.3	-2
5895	*FN3KRP*	79672	fructosamine 3 kinase related protein [Source:HGNC Symbol;Acc:HGNC:25700]	-3.3	-2
5896	*SMTN*	6525	smoothelin [Source:HGNC Symbol;Acc:HGNC:11126]	-3.3	-2
5897	*YES1*	7525	YES proto-oncogene 1, Src family tyrosine kinase [Source:HGNC Symbol;Acc:HGNC:12841]	-3.3	-2
5898	*GATA1*	2623	GATA binding protein 1 [Source:HGNC Symbol;Acc:HGNC:4170]	-3.3	-2
5899	*NUP153*	9972	nucleoporin 153 [Source:HGNC Symbol;Acc:HGNC:8062]	-3.3	-2
5900	*USP13*	8975	ubiquitin specific peptidase 13 (isopeptidase T-3) [Source:HGNC Symbol;Acc:HGNC:12611]	-3.3	-2
5901	*WHSC1*	7468	Wolf-Hirschhorn syndrome candidate 1 [Source:HGNC Symbol;Acc:HGNC:12766]	-3.3	-2
5902	*SDK2*	54549	sidekick cell adhesion molecule 2 [Source:HGNC Symbol;Acc:HGNC:19308]	-3.3	-2
5903	*PHKA1*	5255	phosphorylase kinase regulatory subunit alpha 1 [Source:HGNC Symbol;Acc:HGNC:8925]	-3.3	-2
5904	*PM20D2*	135293	peptidase M20 domain containing 2 [Source:HGNC Symbol;Acc:HGNC:21408]	-3.3	-2
5905	*ABCC1*	4363	ATP binding cassette subfamily C member 1 [Source:HGNC Symbol;Acc:HGNC:51]	-3.3	-2
5906	*SLC26A8*	116369	solute carrier family 26 member 8 [Source:HGNC Symbol;Acc:HGNC:14468]	-3.3	-2
5907	*TPM2*	7169	tropomyosin 2 (beta) [Source:HGNC Symbol;Acc:HGNC:12011]	-3.3	-2
5908	*C5orf28*	64417	chromosome 5 open reading frame 28	-3.3	-2
5909	*EIF3G*	8666	eukaryotic translation initiation factor 3 subunit G [Source:HGNC Symbol;Acc:HGNC:3274]	-3.3	-2
5910	*PIWIL4*	143689	piwi like RNA-mediated gene silencing 4 [Source:HGNC Symbol;Acc:HGNC:18444]	-3.3	-2
5911	*ALG1*	56052	ALG1, chitobiosyldiphosphodolichol beta-mannosyltransferase [Source:HGNC Symbol;Acc:HGNC:18294]	-3.3	-2
5912	*ANKS3*	124401	ankyrin repeat and sterile alpha motif domain containing 3 [Source:HGNC Symbol;Acc:HGNC:29422]	-3.3	-2
5913	*ARSA*	410	arylsulfatase A [Source:HGNC Symbol;Acc:HGNC:713]	-3.3	-2
5914	*AFG3L2*	10939	AFG3 like matrix AAA peptidase subunit 2 [Source:HGNC Symbol;Acc:HGNC:315]	-3.3	-2
5915	*HSPH1*	10808	heat shock protein family H (Hsp110) member 1 [Source:HGNC Symbol;Acc:HGNC:16969]	-3.3	-2
5916	*VGLL3*	389136	vestigial like family member 3 [Source:HGNC Symbol;Acc:HGNC:24327]	-3.3	-2
5917	*RUVBL2*	10856	RuvB like AAA ATPase 2 [Source:HGNC Symbol;Acc:HGNC:10475]	-3.3	-2
5918	*DNAH12*	201625	dynein axonemal heavy chain 12 [Source:HGNC Symbol;Acc:HGNC:2943]	-3.3	-2
5919	*YIPF1*	54432	Yip1 domain family member 1 [Source:HGNC Symbol;Acc:HGNC:25231]	-3.3	-2
5920	*ZNF84*	7637	zinc finger protein 84 [Source:HGNC Symbol;Acc:HGNC:13159]	-3.3	-2
5921	*GDPD3*	79153	glycerophosphodiester phosphodiesterase domain containing 3 [Source:HGNC Symbol;Acc:HGNC:28638]	-3.3	-2
5922	*RNF170*	81790	ring finger protein 170 [Source:HGNC Symbol;Acc:HGNC:25358]	-3.3	-2
5923	*FAM175A*	84142	family with sequence similarity 175 member A [Source:HGNC Symbol;Acc:HGNC:25829]	-3.3	-2
5924	*CWF19L1*	55280	CWF19-like 1, cell cycle control (S. pombe) [Source:HGNC Symbol;Acc:HGNC:25613]	-3.3	-2
5925	*SYT2*	127833	synaptotagmin 2 [Source:HGNC Symbol;Acc:HGNC:11510]	-3.3	-2
5926	*UBE4B*	10277	ubiquitination factor E4B [Source:HGNC Symbol;Acc:HGNC:12500]	-3.3	-2
5927	*C12orf57*	113246	chromosome 12 open reading frame 57 [Source:HGNC Symbol;Acc:HGNC:29521]	-3.3	-2
5928	*ZNF292*	23036	zinc finger protein 292 [Source:HGNC Symbol;Acc:HGNC:18410]	-3.3	-2
5929	*DUSP19*	142679	dual specificity phosphatase 19 [Source:HGNC Symbol;Acc:HGNC:18894]	-3.3	-2
5930	*STK11*	6794	serine/threonine kinase 11 [Source:HGNC Symbol;Acc:HGNC:11389]	-3.3	-2
5931	*HIST2H2AA3*	8337	histone cluster 2, H2aa3 [Source:HGNC Symbol;Acc:HGNC:4736]	-3.3	-2

5932	*HIST2H2AA4*	723790	histone cluster 2, H2aa4 [Source:HGNC Symbol;Acc:HGNC:29668]	-3.3	-2
5933	*DGKA*	1606	diacylglycerol kinase alpha [Source:HGNC Symbol;Acc:HGNC:2849]	-3.3	-2
5934	*RHOT1*	55288	ras homolog family member T1 [Source:HGNC Symbol;Acc:HGNC:21168]	-3.3	-2
5935	*MAN2C1*	4123	mannosidase alpha class 2C member 1 [Source:HGNC Symbol;Acc:HGNC:6827]	-3.3	-2
5936	*HAVCR2*	84868	hepatitis A virus cellular receptor 2 [Source:HGNC Symbol;Acc:HGNC:18437]	-3.3	-2
5937	*ARHGEF39*	84904	Rho guanine nucleotide exchange factor 39 [Source:HGNC Symbol;Acc:HGNC:25909]	-3.3	-2
5938	*VPS41*	27072	VPS41, HOPS complex subunit [Source:HGNC Symbol;Acc:HGNC:12713]	-3.3	-2
5939	*EID2B*	126272	EP300 interacting inhibitor of differentiation 2B [Source:HGNC Symbol;Acc:HGNC:26796]	-3.3	-2
5940	*UIMC1*	51720	ubiquitin interaction motif containing 1 [Source:HGNC Symbol;Acc:HGNC:30298]	-3.3	-2
5941	*TCL1B*	9623	T-cell leukemia/lymphoma 1B [Source:HGNC Symbol;Acc:HGNC:11649]	-3.3	-2
5942	*KIFC3*	3801	kinesin family member C3 [Source:HGNC Symbol;Acc:HGNC:6326]	-3.3	-2
5943	*ZNF763*	284390	zinc finger protein 763 [Source:HGNC Symbol;Acc:HGNC:27614]	-3.3	-2
5944	*FAM129A*	116496	family with sequence similarity 129 member A [Source:HGNC Symbol;Acc:HGNC:16784]	-3.3	-2
5945	*KLB*	152831	klotho beta [Source:HGNC Symbol;Acc:HGNC:15527]	-3.3	-2
5946	*HDGFRP2*	84717	Hepatoma-Derived Growth Factor-Related Protein 2	-3.3	-2
5947	*PLXNB2*	23654	plexin B2 [Source:HGNC Symbol;Acc:HGNC:9104]	-3.3	-2
5948	*CD300LG*	146894	CD300 molecule like family member g [Source:HGNC Symbol;Acc:HGNC:30455]	-3.3	-2
5949	*AVL9*	23080	AVL9 cell migration associated [Source:HGNC Symbol;Acc:HGNC:28994]	-3.3	-2
5950	*ALKBH4*	54784	alkB homolog 4, lysine demethylase [Source:HGNC Symbol;Acc:HGNC:21900]	-3.3	-2
5951	*SELM*	140606	Selenoprotein M	-3.3	-2
5952	*NASP*	4678	nuclear autoantigenic sperm protein [Source:HGNC Symbol;Acc:HGNC:7644]	-3.3	-2
5953	*MAGOH*	4116	mago homolog, exon junction complex core component [Source:HGNC Symbol;Acc:HGNC:6815]	-3.3	-2
5954	*BCL7C*	9274	B-cell CLL/lymphoma 7C [Source:HGNC Symbol;Acc:HGNC:1006]	-3.3	-2
5955	*PRKAB2*	5565	protein kinase AMP-activated non-catalytic subunit beta 2 [Source:HGNC Symbol;Acc:HGNC:9379]	-3.3	-2
5956	*WFDC1*	58189	WAP four-disulfide core domain 1 [Source:HGNC Symbol;Acc:HGNC:15466]	-3.3	-2
5957	*ST20*	400410	suppressor of tumorigenicity 20 [Source:HGNC Symbol;Acc:HGNC:33520]	-3.3	-2
5958	*PLD1*	5337	phospholipase D1 [Source:HGNC Symbol;Acc:HGNC:9067]	-3.3	-2
5959	*TMEM136*	219902	transmembrane protein 136 [Source:HGNC Symbol;Acc:HGNC:28280]	-3.3	-2
5960	*SMIM10L2B*	644596	small integral membrane protein 10 like 2B [Source:HGNC Symbol;Acc:HGNC:34500]	-3.3	-2
5961	*GZMA*	3001	granzyme A [Source:HGNC Symbol;Acc:HGNC:4708]	-3.3	-2
5962	*ZNF169*	169841	zinc finger protein 169 [Source:HGNC Symbol;Acc:HGNC:12957]	-3.3	-2
5963	*TLR7*	51284	toll like receptor 7 [Source:HGNC Symbol;Acc:HGNC:15631]	-3.3	-2
5964	*CEP192*	55125	centrosomal protein 192 [Source:HGNC Symbol;Acc:HGNC:25515]	-3.3	-2
5965	*EXT1*	2131	exostosin glycosyltransferase 1 [Source:HGNC Symbol;Acc:HGNC:3512]	-3.3	-2
5966	*FAM126B*	285172	family with sequence similarity 126 member B [Source:HGNC Symbol;Acc:HGNC:28593]	-3.3	-2
5967	*GRHPR*	9380	glyoxylate reductase/hydroxypyruvate reductase [Source:HGNC Symbol;Acc:HGNC:4570]	-3.3	-2
5968	*SLX4*	84464	SLX4 structure-specific endonuclease subunit [Source:HGNC Symbol;Acc:HGNC:23845]	-3.3	-2
5969	*FAM92A1*	137392	family with sequence similarity 92 member A1 [Source:HGNC Symbol;Acc:HGNC:30452]	-3.3	-2
5970	*FAM161A*	84140	family with sequence similarity 161 member A [Source:HGNC Symbol;Acc:HGNC:25808]	-3.3	-2
5971	*TMOD2*	29767	tropomodulin 2 [Source:HGNC Symbol;Acc:HGNC:11872]	-3.3	-2
5972	*RNF207*	388591	ring finger protein 207 [Source:HGNC Symbol;Acc:HGNC:32947]	-3.3	-2
5973	*ADM*	133	adrenomedullin [Source:HGNC Symbol;Acc:HGNC:259]	-3.3	-2
5974	*ATG7*	10533	autophagy related 7 [Source:HGNC Symbol;Acc:HGNC:16935]	-3.3	-2
5975	*ARHGAP29*	9411	Rho GTPase activating protein 29 [Source:HGNC Symbol;Acc:HGNC:30207]	-3.3	-2
5976	*POLR2B*	5431	polymerase (RNA) II subunit B [Source:HGNC Symbol;Acc:HGNC:9188]	-3.3	-2
5977	*RBBP9*	10741	RB binding protein 9, serine hydrolase [Source:HGNC Symbol;Acc:HGNC:9892]	-3.4	-2
5978	*STK38*	11329	serine/threonine kinase 38 [Source:HGNC Symbol;Acc:HGNC:17847]	-3.4	-2
5979	*IL12RB2*	3595	interleukin 12 receptor subunit beta 2 [Source:HGNC Symbol;Acc:HGNC:5972]	-3.4	-2
5980	*ACO1*	48	aconitase 1 [Source:HGNC Symbol;Acc:HGNC:117]	-3.4	-2
5981	*ECT2L*	345930	epithelial cell transforming 2 like [Source:HGNC Symbol;Acc:HGNC:21118]	-3.4	-2
5982	*PSMG4*	389362	proteasome assembly chaperone 4 [Source:HGNC Symbol;Acc:HGNC:21108]	-3.4	-2
5983	*NGRN*	51335	neugrin, neurite outgrowth associated [Source:HGNC Symbol;Acc:HGNC:18077]	-3.4	-2
5984	*SUSD6*	9766	sushi domain containing 6 [Source:HGNC Symbol;Acc:HGNC:19956]	-3.4	-2
5985	*KCNK3*	3777	potassium two pore domain channel subfamily K member 3 [Source:HGNC Symbol;Acc:HGNC:6278]	-3.4	-2
5986	*NOX4*	50507	NADPH oxidase 4 [Source:HGNC Symbol;Acc:HGNC:7891]	-3.4	-2
5987	*DYRK4*	8798	dual specificity tyrosine phosphorylation regulated kinase 4 [Source:HGNC Symbol;Acc:HGNC:3095]	-3.4	-2
5988	*ATP8B4*	79895	ATPase phospholipid transporting 8B4 (putative) [Source:HGNC Symbol;Acc:HGNC:13536]	-3.4	-2
5989	*DCDC2*	51473	doublecortin domain containing 2 [Source:HGNC Symbol;Acc:HGNC:18141]	-3.4	-2
5990	*C19orf54*	284325	chromosome 19 open reading frame 54 [Source:HGNC Symbol;Acc:HGNC:24758]	-3.4	-2
5991	*NDN*	4692	necdin, MAGE family member [Source:HGNC Symbol;Acc:HGNC:7675]	-3.4	-2
5992	*CD40LG*	959	CD40 ligand [Source:HGNC Symbol;Acc:HGNC:11935]	-3.4	-2
5993	*ZNF808*	388558	zinc finger protein 808 [Source:HGNC Symbol;Acc:HGNC:33230]	-3.4	-2
5994	*L1CAM*	3897	L1 cell adhesion molecule [Source:HGNC Symbol;Acc:HGNC:6470]	-3.4	-2
5995	*CLN3*	1201	ceroid-lipofuscinosis, neuronal 3 [Source:HGNC Symbol;Acc:HGNC:2074]	-3.4	-2
5996	*C8orf37*	157657	chromosome 8 open reading frame 37 [Source:HGNC Symbol;Acc:HGNC:27232]	-3.4	-2
5997	*NDST3*	9348	N-deacetylase/N-sulfotransferase 3 [Source:HGNC Symbol;Acc:HGNC:7682]	-3.4	-2
5998	*SEPSECS*	51091	Sep (O-phosphoserine) tRNA:Sec (selenocysteine) tRNA synthase [Source:HGNC Symbol;Acc:HGNC:30605]	-3.4	-2
5999	*PVRL1*	5818	Nectin Cell Adhesion Molecule 1	-3.4	-2
6000	*GOT2*	2806	glutamic-oxaloacetic transaminase 2 [Source:HGNC Symbol;Acc:HGNC:4433]	-3.4	-2
6001	*ATP6AP1L*	92270	ATPase H+ transporting accessory protein 1 like [Source:HGNC Symbol;Acc:HGNC:28091]	-3.4	-2
6002	*TRAF5*	7188	TNF receptor associated factor 5 [Source:HGNC Symbol;Acc:HGNC:12035]	-3.4	-2
6003	*ZNF770*	54989	zinc finger protein 770 [Source:HGNC Symbol;Acc:HGNC:26061]	-3.4	-2
6004	*ITPK1*	3705	inositol-tetrakisphosphate 1-kinase [Source:HGNC Symbol;Acc:HGNC:6177]	-3.4	-2
6005	*SERPINF1*	5176	serpin family F member 1 [Source:HGNC Symbol;Acc:HGNC:8824]	-3.4	-2
6006	*ST6GALNAC2*	10610	ST6 N-acetylgalactosaminide alpha-2,6-sialyltransferase 2 [Source:HGNC Symbol;Acc:HGNC:10867]	-3.4	-2

6007	*SEC22C*	9117	SEC22 homolog C, vesicle trafficking protein [Source:HGNC Symbol;Acc:HGNC:16828]	-3.4	-2
6008	*ZSWIM7*	125150	zinc finger SWIM-type containing 7 [Source:HGNC Symbol;Acc:HGNC:26993]	-3.4	-2
6009	*OTUD6A*	139562	OTU deubiquitinase 6A [Source:HGNC Symbol;Acc:HGNC:32312]	-3.4	-2
6010	*ABHD1*	84696	abhydrolase domain containing 1 [Source:HGNC Symbol;Acc:HGNC:17553]	-3.4	-2
6011	*PREB*	10113	prolactin regulatory element binding [Source:HGNC Symbol;Acc:HGNC:9356]	-3.4	-2
6012	*CORO2A*	7464	coronin 2A [Source:HGNC Symbol;Acc:HGNC:2255]	-3.4	-2
6013	*GRM7*	2917	glutamate metabotropic receptor 7 [Source:HGNC Symbol;Acc:HGNC:4599]	-3.4	-2
6014	*C14orf105*	55195	chromosome 14 open reading frame 105 [Source:HGNC Symbol;Acc:HGNC:20189]	-3.4	-2
6015	*FBXO22*	26263	F-box protein 22 [Source:HGNC Symbol;Acc:HGNC:13593]	-3.4	-2
6016	*KIAA0825*	285600	KIAA0825 [Source:HGNC Symbol;Acc:HGNC:28532]	-3.4	-2
6017	*MYO15A*	51168	myosin XVA [Source:HGNC Symbol;Acc:HGNC:7594]	-3.4	-2
6018	*DNAJB2*	3300	DnaJ heat shock protein family (Hsp40) member B2 [Source:HGNC Symbol;Acc:HGNC:5228]	-3.4	-2
6019	*FAM186A*	121006	family with sequence similarity 186 member A [Source:HGNC Symbol;Acc:HGNC:26980]	-3.4	-2
6020	*USP42*	84132	ubiquitin specific peptidase 42 [Source:HGNC Symbol;Acc:HGNC:20068]	-3.4	-2
6021	*VKORC1*	79001	vitamin K epoxide reductase complex subunit 1 [Source:HGNC Symbol;Acc:HGNC:23663]	-3.4	-2
6022	*NUDT15*	55270	nudix hydrolase 15 [Source:HGNC Symbol;Acc:HGNC:23063]	-3.4	-2
6023	*SAE1*	10055	SUMO1 activating enzyme subunit 1 [Source:HGNC Symbol;Acc:HGNC:30660]	-3.4	-2
6024	*ALDH1L2*	160428	aldehyde dehydrogenase 1 family member L2 [Source:HGNC Symbol;Acc:HGNC:26777]	-3.4	-2
6025	*PUDP*	8226	pseudouridine 5'-phosphatase [Source:HGNC Symbol;Acc:HGNC:16818]	-3.4	-2
6026	*TMEM87B*	84910	transmembrane protein 87B [Source:HGNC Symbol;Acc:HGNC:25913]	-3.4	-2
6027	*ZNF81*	347344	zinc finger protein 81 [Source:HGNC Symbol;Acc:HGNC:13156]	-3.4	-2
6028	*SLC25A20*	788	solute carrier family 25 member 20 [Source:HGNC Symbol;Acc:HGNC:1421]	-3.4	-2
6029	*TOR1A*	1861	torsin family 1 member A [Source:HGNC Symbol;Acc:HGNC:3098]	-3.4	-2
6030	*RANBP9*	10048	RAN binding protein 9 [Source:HGNC Symbol;Acc:HGNC:13727]	-3.4	-2
6031	*TMPRSS11B*	132724	transmembrane protease, serine 11B [Source:HGNC Symbol;Acc:HGNC:25398]	-3.4	-2
6032	*MANSC1*	54682	MANSC domain containing 1 [Source:HGNC Symbol;Acc:HGNC:25505]	-3.4	-2
6033	*TMEM181*	57583	transmembrane protein 181 [Source:HGNC Symbol;Acc:HGNC:20958]	-3.4	-2
6034	*NRCAM*	4897	neuronal cell adhesion molecule [Source:HGNC Symbol;Acc:HGNC:7994]	-3.4	-2
6035	*GLTP*	51228	glycolipid transfer protein [Source:HGNC Symbol;Acc:HGNC:24867]	-3.4	-2
6036	*NXF3*	56000	nuclear RNA export factor 3 [Source:HGNC Symbol;Acc:HGNC:8073]	-3.4	-2
6037	*FDXR*	2232	ferredoxin reductase [Source:HGNC Symbol;Acc:HGNC:3642]	-3.4	-2
6038	*NKPD1*	284353	NTPase, KAP family P-loop domain containing 1 [Source:HGNC Symbol;Acc:HGNC:24739]	-3.4	-2
6039	*MCMBP*	79892	minichromosome maintenance complex binding protein [Source:HGNC Symbol;Acc:HGNC:25782]	-3.4	-2
6040	*HERC5*	51191	HECT and RLD domain containing E3 ubiquitin protein ligase 5 [Source:HGNC Symbol;Acc:HGNC:24368]	-3.4	-2
6041	*ZNF557*	79230	zinc finger protein 557 [Source:HGNC Symbol;Acc:HGNC:28632]	-3.4	-2
6042	*SSR1*	6745	signal sequence receptor subunit 1 [Source:HGNC Symbol;Acc:HGNC:11323]	-3.4	-2
6043	*C10orf76*	79591	chromosome 10 open reading frame 76 [Source:HGNC Symbol;Acc:HGNC:25788]	-3.4	-2
6044	*TMEM64*	169200	transmembrane protein 64 [Source:HGNC Symbol;Acc:HGNC:25441]	-3.4	-2
6045	*GSTM3*	2947	glutathione S-transferase mu 3 (brain) [Source:HGNC Symbol;Acc:HGNC:4635]	-3.4	-2
6046	*QPCTL*	54814	glutaminyl-peptide cyclotransferase-like [Source:HGNC Symbol;Acc:HGNC:25952]	-3.4	-2
6047	*S1PR3*	1903	sphingosine-1-phosphate receptor 3 [Source:HGNC Symbol;Acc:HGNC:3167]	-3.4	-2
6048	*PRDM7*	11105	PR domain 7 [Source:HGNC Symbol;Acc:HGNC:9351]	-3.4	-2
6049	*RNASEH2B*	79621	ribonuclease H2 subunit B [Source:HGNC Symbol;Acc:HGNC:25671]	-3.4	-2
6050	*AP4M1*	9179	adaptor related protein complex 4 mu 1 subunit [Source:HGNC Symbol;Acc:HGNC:574]	-3.4	-2
6051	*CREB3L4*	148327	cAMP responsive element binding protein 3 like 4 [Source:HGNC Symbol;Acc:HGNC:18854]	-3.4	-2
6052	*IARS2*	55699	isoleucyl-tRNA synthetase 2, mitochondrial [Source:HGNC Symbol;Acc:HGNC:29685]	-3.4	-2
6053	*PTBP2*	58155	polypyrimidine tract binding protein 2 [Source:HGNC Symbol;Acc:HGNC:17662]	-3.4	-2
6054	*ZNF235*	9310	zinc finger protein 235 [Source:HGNC Symbol;Acc:HGNC:12866]	-3.4	-2
6055	*MARVELD2*	153562	MARVEL domain containing 2 [Source:HGNC Symbol;Acc:HGNC:26401]	-3.5	-2
6056	*IKBIP*	121457	IKBKB interacting protein [Source:HGNC Symbol;Acc:HGNC:26430]	-3.5	-2
6057	*DDTL*	100037417	D-dopachrome tautomerase-like [Source:HGNC Symbol;Acc:HGNC:33446]	-3.5	-2
6058	*MXRA7*	439921	matrix remodeling associated 7 [Source:HGNC Symbol;Acc:HGNC:7541]	-3.5	-2
6059	*DUOX1*	53905	dual oxidase 1 [Source:HGNC Symbol;Acc:HGNC:3062]	-3.5	-2
6060	*TFR2*	7036	transferrin receptor 2 [Source:HGNC Symbol;Acc:HGNC:11762]	-3.5	-2
6061	*MOB1A*	55233	MOB kinase activator 1A [Source:HGNC Symbol;Acc:HGNC:16015]	-3.5	-2
6062	*TBC1D20*	128637	TBC1 domain family member 20 [Source:HGNC Symbol;Acc:HGNC:16133]	-3.5	-2
6063	*CRKL*	1399	v-crk avian sarcoma virus CT10 oncogene homolog-like [Source:HGNC Symbol;Acc:HGNC:2363]	-3.5	-2
6064	*PTGES*	9536	prostaglandin E synthase [Source:HGNC Symbol;Acc:HGNC:9599]	-3.5	-2
6065	*DBNDD1*	79007	dysbindin (dystrobrevin binding protein 1) domain containing 1 [Source:HGNC Symbol;Acc:HGNC:28455]	-3.5	-2
6066	*KIAA1328*	57536	KIAA1328 [Source:HGNC Symbol;Acc:HGNC:29248]	-3.5	-2
6067	*ABHD4*	63874	abhydrolase domain containing 4 [Source:HGNC Symbol;Acc:HGNC:20154]	-3.5	-2
6068	*FABP7*	2173	fatty acid binding protein 7 [Source:HGNC Symbol;Acc:HGNC:3562]	-3.5	-2
6069	*SLC30A7*	148867	solute carrier family 30 member 7 [Source:HGNC Symbol;Acc:HGNC:19306]	-3.5	-2
6070	*NXPH4*	11247	neurexophilin 4 [Source:HGNC Symbol;Acc:HGNC:8078]	-3.5	-2
6071	*CCDC66*	285331	coiled-coil domain containing 66 [Source:HGNC Symbol;Acc:HGNC:27709]	-3.5	-2
6072	*PPFIA3*	8541	PTPRF interacting protein alpha 3 [Source:HGNC Symbol;Acc:HGNC:9247]	-3.5	-2
6073	*HINT2*	84681	histidine triad nucleotide binding protein 2 [Source:HGNC Symbol;Acc:HGNC:18344]	-3.5	-2
6074	*KIF1B*	23095	kinesin family member 1B [Source:HGNC Symbol;Acc:HGNC:16636]	-3.5	-2
6075	*STX1B*	112755	syntaxin 1B [Source:HGNC Symbol;Acc:HGNC:18539]	-3.5	-2
6076	*SBNO2*	22904	strawberry notch homolog 2 (Drosophila) [Source:HGNC Symbol;Acc:HGNC:29158]	-3.5	-2
6077	*SIVA1*	10572	SIVA1 apoptosis inducing factor [Source:HGNC Symbol;Acc:HGNC:17712]	-3.5	-2
6078	*FUT6*	2528	fucosyltransferase 6 [Source:HGNC Symbol;Acc:HGNC:4017]	-3.5	-2
6079	*VHL*	7428	von Hippel-Lindau tumor suppressor [Source:HGNC Symbol;Acc:HGNC:12687]	-3.5	-2
6080	*HPS4*	89781	HPS4, biogenesis of lysosomal organelles complex 3 subunit 2 [Source:HGNC Symbol;Acc:HGNC:15844]	-3.5	-2
6081	*YAE1D1*	57002	Yae1 domain containing 1 [Source:HGNC Symbol;Acc:HGNC:24857]	-3.5	-2
6082	*PHTF1*	10745	putative homeodomain transcription factor 1 [Source:HGNC Symbol;Acc:HGNC:8939]	-3.5	-2

6083	*INTS2*	57508	integrator complex subunit 2 [Source:HGNC Symbol;Acc:HGNC:29241]	-3.5	-2
6084	*CCL28*	56477	C-C motif chemokine ligand 28 [Source:HGNC Symbol;Acc:HGNC:17700]	-3.5	-2
6085	*GNB2*	2783	G protein subunit beta 2 [Source:HGNC Symbol;Acc:HGNC:4398]	-3.5	-2
6086	*MBNL1*	4154	muscleblind like splicing regulator 1 [Source:HGNC Symbol;Acc:HGNC:6923]	-3.5	-2
6087	*ATG9A*	79065	autophagy related 9A [Source:HGNC Symbol;Acc:HGNC:22408]	-3.5	-2
6088	*PES1*	23481	pescadillo ribosomal biogenesis factor 1 [Source:HGNC Symbol;Acc:HGNC:8848]	-3.5	-2
6089	*FAM217B*	63939	family with sequence similarity 217 member B [Source:HGNC Symbol;Acc:HGNC:16170]	-3.5	-2
6090	*DNM2*	1785	dynamin 2 [Source:HGNC Symbol;Acc:HGNC:2974]	-3.5	-2
6091	*SETDB2*	83852	SET domain bifurcated 2 [Source:HGNC Symbol;Acc:HGNC:20263]	-3.5	-2
6092	*SLC35B4*	84912	solute carrier family 35 member B4 [Source:HGNC Symbol;Acc:HGNC:20584]	-3.5	-2
6093	*AGAP2*	116986	ArfGAP with GTPase domain, ankyrin repeat and PH domain 2 [Source:HGNC Symbol;Acc:HGNC:16921]	-3.5	-2
6094	*SLC28A2*	9153	solute carrier family 28 member 2 [Source:HGNC Symbol;Acc:HGNC:11002]	-3.5	-2
6095	*LGR6*	59352	leucine rich repeat containing G protein-coupled receptor 6 [Source:HGNC Symbol;Acc:HGNC:19719]	-3.5	-2
6096	*PTPDC1*	138639	protein tyrosine phosphatase domain containing 1 [Source:HGNC Symbol;Acc:HGNC:30184]	-3.5	-2
6097	*CXorf36*	79742	chromosome X open reading frame 36 [Source:HGNC Symbol;Acc:HGNC:25866]	-3.5	-2
6098	*HPS1*	3257	HPS1, biogenesis of lysosomal organelles complex 3 subunit 1 [Source:HGNC Symbol;Acc:HGNC:5163]	-3.5	-2
6099	*IFT140*	9742	intraflagellar transport 140 [Source:HGNC Symbol;Acc:HGNC:29077]	-3.5	-2
6100	*FBXW8*	26259	F-box and WD repeat domain containing 8 [Source:HGNC Symbol;Acc:HGNC:13597]	-3.5	-2
6101	*ALDH1L1*	10840	aldehyde dehydrogenase 1 family member L1 [Source:HGNC Symbol;Acc:HGNC:3978]	-3.5	-2
6102	*LRRC69*	100130742	leucine rich repeat containing 69 [Source:HGNC Symbol;Acc:HGNC:34303]	-3.5	-2
6103	*BPNT1*	10380	3'(2'), 5'-bisphosphate nucleotidase 1 [Source:HGNC Symbol;Acc:HGNC:1096]	-3.5	-2
6104	*POLN*	353497	polymerase (DNA) nu [Source:HGNC Symbol;Acc:HGNC:18870]	-3.5	-2
6105	*ARMC7*	79637	armadillo repeat containing 7 [Source:HGNC Symbol;Acc:HGNC:26168]	-3.5	-2
6106	*NT5C*	30833	5', 3'-nucleotidase, cytosolic [Source:HGNC Symbol;Acc:HGNC:17144]	-3.5	-2
6107	*TRIM63*	84676	tripartite motif containing 63 [Source:HGNC Symbol;Acc:HGNC:16007]	-3.5	-2
6108	*PRODH*	5625	proline dehydrogenase 1 [Source:HGNC Symbol;Acc:HGNC:9453]	-3.5	-2
6109	*COL10A1*	1300	collagen type X alpha 1 chain [Source:HGNC Symbol;Acc:HGNC:2185]	-3.5	-2
6110	*POLE2*	5427	polymerase (DNA) epsilon 2, accessory subunit [Source:HGNC Symbol;Acc:HGNC:9178]	-3.5	-2
6111	*IFIT2*	3433	interferon induced protein with tetratricopeptide repeats 2 [Source:HGNC Symbol;Acc:HGNC:5409]	-3.5	-2
6112	*COMMD9*	29099	COMM domain containing 9 [Source:HGNC Symbol;Acc:HGNC:25014]	-3.5	-2
6113	*SLC39A1*	27173	solute carrier family 39 member 1 [Source:HGNC Symbol;Acc:HGNC:12876]	-3.5	-2
6114	*PHF20*	51230	PHD finger protein 20 [Source:HGNC Symbol;Acc:HGNC:16098]	-3.5	-2
6115	*FTH1*	2495	ferritin heavy chain 1 [Source:HGNC Symbol;Acc:HGNC:3976]	-3.5	-2
6116	*TFPT*	29844	TCF3 (E2A) fusion partner (in childhood Leukemia) [Source:HGNC Symbol;Acc:HGNC:13630]	-3.5	-2
6117	*CACNB2*	783	calcium voltage-gated channel auxiliary subunit beta 2 [Source:HGNC Symbol;Acc:HGNC:1402]	-3.5	-2
6118	*COG6*	57511	component of oligomeric golgi complex 6 [Source:HGNC Symbol;Acc:HGNC:18621]	-3.5	-2
6119	*GSS*	2937	glutathione synthetase [Source:HGNC Symbol;Acc:HGNC:4624]	-3.5	-2
6120	*JUN*	3725	Jun proto-oncogene, AP-1 transcription factor subunit [Source:HGNC Symbol;Acc:HGNC:6204]	-3.5	-2
6121	*DNAAF3*	352909	dynein (axonemal) assembly factor 3 [Source:HGNC Symbol;Acc:HGNC:30492]	-3.5	-2
6122	*AP3M1*	26985	adaptor related protein complex 3 mu 1 subunit [Source:HGNC Symbol;Acc:HGNC:569]	-3.5	-2
6123	*MTMR6*	9107	myotubularin related protein 6 [Source:HGNC Symbol;Acc:HGNC:7453]	-3.5	-2
6124	*ATXN7L3B*	552889	ataxin 7 like 3B [Source:HGNC Symbol;Acc:HGNC:37931]	-3.5	-2
6125	*DAW1*	164781	dynein assembly factor with WD repeats 1 [Source:HGNC Symbol;Acc:HGNC:26383]	-3.5	-2
6126	*MYBPC2*	4606	myosin binding protein C, fast type [Source:HGNC Symbol;Acc:HGNC:7550]	-3.5	-2
6127	*SKIDA1*	387640	SKI/DACH domain containing 1 [Source:HGNC Symbol;Acc:HGNC:32697]	-3.5	-2
6128	*IARS*	3376	isoleucyl-tRNA synthetase [Source:HGNC Symbol;Acc:HGNC:5330]	-3.6	-2
6129	*ZFAND6*	54469	zinc finger AN1-type containing 6 [Source:HGNC Symbol;Acc:HGNC:30164]	-3.6	-2
6130	*PDSS2*	57107	prenyl (decaprenyl) diphosphate synthase, subunit 2 [Source:HGNC Symbol;Acc:HGNC:23041]	-3.6	-2
6131	*ACTR1B*	10120	ARP1 actin-related protein 1 homolog B, centractin beta [Source:HGNC Symbol;Acc:HGNC:168]	-3.6	-2
6132	*PRDX4*	10549	peroxiredoxin 4 [Source:HGNC Symbol;Acc:HGNC:17169]	-3.6	-2
6133	*DDR1*	780	discoidin domain receptor tyrosine kinase 1 [Source:HGNC Symbol;Acc:HGNC:2730]	-3.6	-2
6134	*GK5*	256356	glycerol kinase 5 (putative) [Source:HGNC Symbol;Acc:HGNC:28635]	-3.6	-2
6135	*DDHD2*	23259	DDHD domain containing 2 [Source:HGNC Symbol;Acc:HGNC:29106]	-3.6	-2
6136	*USP39*	10713	ubiquitin specific peptidase 39 [Source:HGNC Symbol;Acc:HGNC:20071]	-3.6	-2
6137	*RNF157*	114804	ring finger protein 157 [Source:HGNC Symbol;Acc:HGNC:29402]	-3.6	-2
6138	*DENND4A*	10260	DENN domain containing 4A [Source:HGNC Symbol;Acc:HGNC:24321]	-3.6	-2
6139	*SLFN5*	162394	schlafen family member 5 [Source:HGNC Symbol;Acc:HGNC:28286]	-3.6	-2
6140	*ERCC3*	2071	ERCC excision repair 3, TFIIH core complex helicase subunit [Source:HGNC Symbol;Acc:HGNC:3435]	-3.6	-2
6141	*GIGYF1*	64599	GRB10 interacting GYF protein 1 [Source:HGNC Symbol;Acc:HGNC:9126]	-3.6	-2
6142	*CNOT6L*	246175	CCR4-NOT transcription complex subunit 6 like [Source:HGNC Symbol;Acc:HGNC:18042]	-3.6	-2
6143	*MED11*	400569	mediator complex subunit 11 [Source:HGNC Symbol;Acc:HGNC:32687]	-3.6	-2
6144	*TBC1D8B*	54885	TBC1 domain family member 8B [Source:HGNC Symbol;Acc:HGNC:24715]	-3.6	-2
6145	*RPUSD3*	285367	RNA pseudouridylate synthase domain containing 3 [Source:HGNC Symbol;Acc:HGNC:28437]	-3.6	-2
6146	*AMBRA1*	55626	autophagy and beclin 1 regulator 1 [Source:HGNC Symbol;Acc:HGNC:25990]	-3.6	-2
6147	*RASGEF1B*	153020	RasGEF domain family member 1B [Source:HGNC Symbol;Acc:HGNC:24881]	-3.6	-2
6148	*CYP46A1*	10858	cytochrome P450 family 46 subfamily A member 1 [Source:HGNC Symbol;Acc:HGNC:2641]	-3.6	-2
6149	*EEFSEC*	60678	eukaryotic elongation factor, selenocysteine-tRNA specific [Source:HGNC Symbol;Acc:HGNC:24614]	-3.6	-2
6150	*KLC1*	3831	kinesin light chain 1 [Source:HGNC Symbol;Acc:HGNC:6387]	-3.6	-2
6151	*MRPL38*	64978	mitochondrial ribosomal protein L38 [Source:HGNC Symbol;Acc:HGNC:14033]	-3.6	-2

6152	*PHGDH*	26227	phosphoglycerate dehydrogenase [Source:HGNC Symbol;Acc:HGNC:8923]	-3.6	-2
6153	*PDE12*	201626	phosphodiesterase 12 [Source:HGNC Symbol;Acc:HGNC:25386]	-3.6	-2
6154	*EMC10*	284361	ER membrane protein complex subunit 10 [Source:HGNC Symbol;Acc:HGNC:27609]	-3.6	-2
6155	*FAM71E1*	112703	family with sequence similarity 71 member E1 [Source:HGNC Symbol;Acc:HGNC:25107]	-3.6	-2
6156	*RARS*	5917	arginyl-tRNA synthetase [Source:HGNC Symbol;Acc:HGNC:9870]	-3.6	-2
6157	*RRBP1*	6238	ribosome binding protein 1 [Source:HGNC Symbol;Acc:HGNC:10448]	-3.6	-2
6158	*RPP30*	10556	ribonuclease P/MRP subunit p30 [Source:HGNC Symbol;Acc:HGNC:17688]	-3.6	-2
6159	*C1orf53*	388722	chromosome 1 open reading frame 53 [Source:HGNC Symbol;Acc:HGNC:30003]	-3.6	-2
6160	*WDR17*	116966	WD repeat domain 17 [Source:HGNC Symbol;Acc:HGNC:16661]	-3.6	-2
6161	*ING1*	3621	inhibitor of growth family member 1 [Source:HGNC Symbol;Acc:HGNC:6062]	-3.6	-2
6162	*CARD11*	84433	caspase recruitment domain family member 11 [Source:HGNC Symbol;Acc:HGNC:16393]	-3.6	-2
6163	*MS4A10*	341116	membrane spanning 4-domains A10 [Source:HGNC Symbol;Acc:HGNC:13368]	-3.6	-2
6164	*TMC7*	79905	transmembrane channel like 7 [Source:HGNC Symbol;Acc:HGNC:23000]	-3.6	-2
6165	*FBXL5*	26234	F-box and leucine rich repeat protein 5 [Source:HGNC Symbol;Acc:HGNC:13602]	-3.6	-2
6166	*CHRNA5*	1138	cholinergic receptor nicotinic alpha 5 subunit [Source:HGNC Symbol;Acc:HGNC:1959]	-3.6	-2
6167	*MCCC1*	56922	methylcrotonoyl-CoA carboxylase 1 [Source:HGNC Symbol;Acc:HGNC:6936]	-3.6	-2
6168	*P3H2*	55214	prolyl 3-hydroxylase 2 [Source:HGNC Symbol;Acc:HGNC:19317]	-3.6	-2
6169	*ZAN*	7455	zonadhesin (gene/pseudogene) [Source:HGNC Symbol;Acc:HGNC:12857]	-3.6	-2
6170	*DHX8*	1659	DEAH-box helicase 8 [Source:HGNC Symbol;Acc:HGNC:2749]	-3.6	-2
6171	*OSBPL1A*	114876	oxysterol binding protein like 1A [Source:HGNC Symbol;Acc:HGNC:16398]	-3.6	-2
6172	*FAM189B*	10712	family with sequence similarity 189 member B [Source:HGNC Symbol;Acc:HGNC:1233]	-3.6	-2
6173	*MROH1*	727957	maestro heat like repeat family member 1 [Source:HGNC Symbol;Acc:HGNC:26958]	-3.6	-2
6174	*ZNF585B*	92285	zinc finger protein 585B [Source:HGNC Symbol;Acc:HGNC:30948]	-3.6	-2
6175	*NAV1*	89796	neuron navigator 1 [Source:HGNC Symbol;Acc:HGNC:15989]	-3.6	-2
6176	*SNRPG*	6637	small nuclear ribonucleoprotein polypeptide G [Source:HGNC Symbol;Acc:HGNC:11163]	-3.6	-2
6177	*ZNF136*	7695	zinc finger protein 136 [Source:HGNC Symbol;Acc:HGNC:12920]	-3.6	-2
6178	*LIN52*	91750	lin-52 DREAM MuvB core complex component [Source:HGNC Symbol;Acc:HGNC:19856]	-3.6	-2
6179	*TTLL1*	25809	tubulin tyrosine ligase like 1 [Source:HGNC Symbol;Acc:HGNC:1312]	-3.6	-2
6180	*ACSL6*	23305	acyl-CoA synthetase long-chain family member 6 [Source:HGNC Symbol;Acc:HGNC:16496]	-3.6	-2
6181	*ZNF713*	349075	zinc finger protein 713 [Source:HGNC Symbol;Acc:HGNC:22043]	-3.6	-2
6182	*IDO2*	169355	indoleamine 2,3-dioxygenase 2 [Source:HGNC Symbol;Acc:HGNC:27269]	-3.6	-2
6183	*ZNF835*	90485	zinc finger protein 835 [Source:HGNC Symbol;Acc:HGNC:34332]	-3.6	-2
6184	*HIST1H4H*	8365	histone cluster 1, H4h [Source:HGNC Symbol;Acc:HGNC:4788]	-3.6	-2
6185	*EIF5A2*	56648	eukaryotic translation initiation factor 5A2 [Source:HGNC Symbol;Acc:HGNC:3301]	-3.6	-2
6186	*GSTT2*	2953	glutathione S-transferase theta 2 (gene/pseudogene) [Source:HGNC Symbol;Acc:HGNC:4642]	-3.6	-2
6187	*GSTT2B*	653689	glutathione S-transferase theta 2B (gene/pseudogene) [Source:HGNC Symbol;Acc:HGNC:33437]	-3.6	-2
6188	*CCDC146*	57639	coiled-coil domain containing 146 [Source:HGNC Symbol;Acc:HGNC:29296]	-3.6	-2
6189	*PIN1*	5300	peptidylprolyl cis/trans isomerase, NIMA-interacting 1 [Source:HGNC Symbol;Acc:HGNC:8988]	-3.6	-2
6190	*CCDC28A*	25901	coiled-coil domain containing 28A [Source:HGNC Symbol;Acc:HGNC:21098]	-3.6	-2
6191	*TFEB*	7942	transcription factor EB [Source:HGNC Symbol;Acc:HGNC:11753]	-3.6	-2
6192	*GLG1*	2734	golgi glycoprotein 1 [Source:HGNC Symbol;Acc:HGNC:4316]	-3.6	-2
6193	*CHCHD6*	84303	coiled-coil-helix-coiled-coil-helix domain containing 6 [Source:HGNC Symbol;Acc:HGNC:28184]	-3.6	-2
6194	*ZPR1*	8882	ZPR1 zinc finger [Source:HGNC Symbol;Acc:HGNC:13051]	-3.6	-2
6195	*DIS3L*	115752	DIS3 like exosome 3'-5' exoribonuclease [Source:HGNC Symbol;Acc:HGNC:28698]	-3.6	-2
6196	*SIGLEC8*	27181	sialic acid binding Ig like lectin 8 [Source:HGNC Symbol;Acc:HGNC:10877]	-3.6	-2
6197	*ZSCAN32*	54925	zinc finger and SCAN domain containing 32 [Source:HGNC Symbol;Acc:HGNC:20812]	-3.6	-2
6198	*EXD1*	161829	exonuclease 3'-5' domain containing 1 [Source:HGNC Symbol;Acc:HGNC:28507]	-3.6	-2
6199	*ERCC8*	1161	ERCC excision repair 8, CSA ubiquitin ligase complex subunit [Source:HGNC Symbol;Acc:HGNC:3439]	-3.6	-2
6200	*NDUFS7*	374291	NADH:ubiquinone oxidoreductase core subunit S7 [Source:HGNC Symbol;Acc:HGNC:7714]	-3.6	-2
6201	*MRAS*	22808	muscle RAS oncogene homolog [Source:HGNC Symbol;Acc:HGNC:7227]	-3.6	-2
6202	*LRRN4CL*	221091	LRRN4 C-terminal like [Source:HGNC Symbol;Acc:HGNC:33724]	-3.6	-2
6203	*SLC1A2*	6506	solute carrier family 1 member 2 [Source:HGNC Symbol;Acc:HGNC:10940]	-3.6	-2
6204	*FMO5*	2330	flavin containing monooxygenase 5 [Source:HGNC Symbol;Acc:HGNC:3773]	-3.6	-2
6205	*N4BP2*	55728	NEDD4 binding protein 2 [Source:HGNC Symbol;Acc:HGNC:29851]	-3.6	-2
6206	*BLZF1*	8548	basic leucine zipper nuclear factor 1 [Source:HGNC Symbol;Acc:HGNC:1065]	-3.6	-2
6207	*DMKN*	93099	dermokine [Source:HGNC Symbol;Acc:HGNC:25063]	-3.6	-2
6208	*ARID3A*	1820	AT-rich interaction domain 3A [Source:HGNC Symbol;Acc:HGNC:3031]	-3.6	-2
6209	*ULK2*	9706	unc-51 like autophagy activating kinase 2 [Source:HGNC Symbol;Acc:HGNC:13480]	-3.6	-2
6210	*VRK3*	51231	vaccinia related kinase 3 [Source:HGNC Symbol;Acc:HGNC:18996]	-3.6	-2
6211	*C1orf61*	10485	chromosome 1 open reading frame 61 [Source:HGNC Symbol;Acc:HGNC:30780]	-3.6	-2
6212	*FAM105A*	54491	family with sequence similarity 105 member A [Source:HGNC Symbol;Acc:HGNC:25629]	-3.6	-2
6213	*DNAH10*	196385	dynein axonemal heavy chain 10 [Source:HGNC Symbol;Acc:HGNC:2941]	-3.6	-2
6214	*NME7*	29922	NME/NM23 family member 7 [Source:HGNC Symbol;Acc:HGNC:20461]	-3.6	-2
6215	*ZNF799*	90576	zinc finger protein 799 [Source:HGNC Symbol;Acc:HGNC:28071]	-3.6	-2
6216	*CSRP2BP*	57325	Cysteine Rich Protein 2 Binding Protein	-3.6	-2
6217	*SLC25A19*	60386	solute carrier family 25 member 19 [Source:HGNC Symbol;Acc:HGNC:14409]	-3.6	-2
6218	*CNOT10*	25904	CCR4-NOT transcription complex subunit 10 [Source:HGNC Symbol;Acc:HGNC:23817]	-3.6	-2
6219	*ZNF621*	285268	zinc finger protein 621 [Source:HGNC Symbol;Acc:HGNC:24787]	-3.6	-2
6220	*SPICE1*	152185	spindle and centriole associated protein 1 [Source:HGNC Symbol;Acc:HGNC:25083]	-3.6	-2
6221	*MITD1*	129531	microtubule interacting and trafficking domain containing 1 [Source:HGNC Symbol;Acc:HGNC:25207]	-3.6	-2
6222	*MRPL57*	78988	mitochondrial ribosomal protein L57 [Source:HGNC Symbol;Acc:HGNC:14514]	-3.7	-2
6223	*AQP3*	360	aquaporin 3 (Gill blood group) [Source:HGNC Symbol;Acc:HGNC:636]	-3.7	-2
6224	*SDCCAG8*	10806	serologically defined colon cancer antigen 8 [Source:HGNC Symbol;Acc:HGNC:10671]	-3.7	-2
6225	*TBC1D16*	125058	TBC1 domain family member 16 [Source:HGNC Symbol;Acc:HGNC:28356]	-3.7	-2
6226	*LILRA6*	79168	leukocyte immunoglobulin like receptor A6 [Source:HGNC Symbol;Acc:HGNC:15495]	-3.7	-2

6227	*GNS*	2799	glucosamine (N-acetyl)-6-sulfatase [Source:HGNC Symbol;Acc:HGNC:4422]	-3.7	-2
6228	*GBP6*	163351	guanylate binding protein family member 6 [Source:HGNC Symbol;Acc:HGNC:25395]	-3.7	-2
6229	*RWDD2B*	10069	RWD domain containing 2B [Source:HGNC Symbol;Acc:HGNC:1302]	-3.7	-2
6230	*IMPA2*	3613	inositol monophosphatase 2 [Source:HGNC Symbol;Acc:HGNC:6051]	-3.7	-2
6231	*SLC46A1*	113235	solute carrier family 46 member 1 [Source:HGNC Symbol;Acc:HGNC:30521]	-3.7	-2
6232	*SP4*	6671	Sp4 transcription factor [Source:HGNC Symbol;Acc:HGNC:11209]	-3.7	-2
6233	*GRASP*	160622	GRP1 (general receptor for phosphoinositides 1)-associated scaffold protein [Source:HGNC Symbol;Acc:HGNC:18707]	-3.7	-2
6234	*SCAMP4*	113178	secretory carrier membrane protein 4 [Source:HGNC Symbol;Acc:HGNC:30385]	-3.7	-2
6235	*MTMR12*	54545	myotubularin related protein 12 [Source:HGNC Symbol;Acc:HGNC:18191]	-3.7	-2
6236	*DPH1*	1801	diphthamide biosynthesis 1 [Source:HGNC Symbol;Acc:HGNC:3003]	-3.7	-2
6237	*TMEM123*	114908	transmembrane protein 123 [Source:HGNC Symbol;Acc:HGNC:30138]	-3.7	-2
6238	*FAM111A*	63901	family with sequence similarity 111 member A [Source:HGNC Symbol;Acc:HGNC:24725]	-3.7	-2
6239	*FAM161B*	145483	family with sequence similarity 161 member B [Source:HGNC Symbol;Acc:HGNC:19854]	-3.7	-2
6240	*RFT1*	91869	RFT1 homolog [Source:HGNC Symbol;Acc:HGNC:30220]	-3.7	-2
6241	*TMOD3*	29766	tropomodulin 3 [Source:HGNC Symbol;Acc:HGNC:11873]	-3.7	-2
6242	*ALKBH5*	54890	alkB homolog 5, RNA demethylase [Source:HGNC Symbol;Acc:HGNC:25996]	-3.7	-2
6243	*RBM10*	8241	RNA binding motif protein 10 [Source:HGNC Symbol;Acc:HGNC:9896]	-3.7	-2
6244	*ADIPOR1*	51094	adiponectin receptor 1 [Source:HGNC Symbol;Acc:HGNC:24040]	-3.7	-2
6245	*TTN*	7273	titin [Source:HGNC Symbol;Acc:HGNC:12403]	-3.7	-2
6246	*C17orf58*	284018	chromosome 17 open reading frame 58 [Source:HGNC Symbol;Acc:HGNC:27568]	-3.7	-2
6247	*POU2AF1*	5450	POU class 2 associating factor 1 [Source:HGNC Symbol;Acc:HGNC:9211]	-3.7	-2
6248	*BRE*	9577	brain and reproductive organ-expressed (TNFRSF1A modulator) [Source:HGNC Symbol;Acc:HGNC:1106]	-3.7	-2
6249	*JUND*	3727	JunD proto-oncogene, AP-1 transcription factor subunit [Source:HGNC Symbol;Acc:HGNC:6206]	-3.7	-2
6250	*DENND5B*	160518	DENN domain containing 5B [Source:HGNC Symbol;Acc:HGNC:28338]	-3.7	-2
6251	*PATL1*	219988	PAT1 homolog 1, processing body mRNA decay factor [Source:HGNC Symbol;Acc:HGNC:26721]	-3.7	-2
6252	*SERPING1*	710	serpin family G member 1 [Source:HGNC Symbol;Acc:HGNC:1228]	-3.7	-2
6253	*ZNF622*	90441	zinc finger protein 622 [Source:HGNC Symbol;Acc:HGNC:30958]	-3.7	-2
6254	*NDUFA4L2*	56901	NADH dehydrogenase (ubiquinone) 1 alpha subcomplex, 4-like 2 [Source:HGNC Symbol;Acc:HGNC:29836]	-3.7	-2
6255	*NUP133*	55746	nucleoporin 133 [Source:HGNC Symbol;Acc:HGNC:18016]	-3.7	-2
6256	*TSNAX*	7257	translin associated factor X [Source:HGNC Symbol;Acc:HGNC:12380]	-3.7	-2
6257	*ERLIN2*	11160	ER lipid raft associated 2 [Source:HGNC Symbol;Acc:HGNC:1356]	-3.7	-2
6258	*TTC33*	23548	tetratricopeptide repeat domain 33 [Source:HGNC Symbol;Acc:HGNC:29959]	-3.7	-2
6259	*HOMEZ*	57594	homeobox and leucine zipper encoding [Source:HGNC Symbol;Acc:HGNC:20164]	-3.7	-2
6260	*MFSD2A*	84879	major facilitator superfamily domain containing 2A [Source:HGNC Symbol;Acc:HGNC:25897]	-3.7	-2
6261	*PKP1*	5317	plakophilin 1 [Source:HGNC Symbol;Acc:HGNC:9023]	-3.7	-2
6262	*RBL1*	5933	RB transcriptional corepressor like 1 [Source:HGNC Symbol;Acc:HGNC:9893]	-3.7	-2
6263	*TMEM105*	284186	transmembrane protein 105 [Source:HGNC Symbol;Acc:HGNC:26794]	-3.7	-2
6264	*TRAPPC5*	126003	trafficking protein particle complex 5 [Source:HGNC Symbol;Acc:HGNC:23067]	-3.7	-2
6265	*CX3CL1*	6376	C-X3-C motif chemokine ligand 1 [Source:HGNC Symbol;Acc:HGNC:10647]	-3.7	-2
6266	*A2ML1*	144568	alpha-2-macroglobulin like 1 [Source:HGNC Symbol;Acc:HGNC:23336]	-3.7	-2
6267	*AK9*	221264	adenylate kinase 9 [Source:HGNC Symbol;Acc:HGNC:33814]	-3.7	-2
6268	*COL5A3*	50509	collagen type V alpha 3 [Source:HGNC Symbol;Acc:HGNC:14864]	-3.7	-2
6269	*CEACAM1*	634	carcinoembryonic antigen related cell adhesion molecule 1 [Source:HGNC Symbol;Acc:HGNC:1814]	-3.7	-2
6270	*FOPNL*	123811	FGFR1OP N-terminal like [Source:HGNC Symbol;Acc:HGNC:26435]	-3.7	-2
6271	*SYDE2*	84144	synapse defective Rho GTPase homolog 2 [Source:HGNC Symbol;Acc:HGNC:25841]	-3.7	-2
6272	*DUSP11*	8446	dual specificity phosphatase 11 [Source:HGNC Symbol;Acc:HGNC:3066]	-3.7	-2
6273	*MPHOSPH6*	10200	M-phase phosphoprotein 6 [Source:HGNC Symbol;Acc:HGNC:7214]	-3.7	-2
6274	*RAB22A*	57403	RAB22A, member RAS oncogene family [Source:HGNC Symbol;Acc:HGNC:9764]	-3.7	-2
6275	*ADAT1*	23536	adenosine deaminase, tRNA specific 1 [Source:HGNC Symbol;Acc:HGNC:228]	-3.7	-2
6276	*C11orf54*	28970	chromosome 11 open reading frame 54 [Source:HGNC Symbol;Acc:HGNC:30204]	-3.7	-2
6277	*BIRC6*	57448	baculoviral IAP repeat containing 6 [Source:HGNC Symbol;Acc:HGNC:13516]	-3.7	-2
6278	*UTP14C*	9724	UTP14, small subunit processome component homolog C (S. cerevisiae) [Source:HGNC Symbol;Acc:HGNC:20321]	-3.7	-2
6279	*GSTZ1*	2954	glutathione S-transferase zeta 1 [Source:HGNC Symbol;Acc:HGNC:4643]	-3.7	-2
6280	*ADPRM*	56985	ADP-ribose/CDP-alcohol diphosphatase, manganese dependent [Source:HGNC Symbol;Acc:HGNC:30925]	-3.7	-2
6281	*SAA2*	6289	serum amyloid A2 [Source:HGNC Symbol;Acc:HGNC:10514]	-3.7	-2
6282	*MIOS*	54468	meiosis regulator for oocyte development [Source:HGNC Symbol;Acc:HGNC:21905]	-3.7	-2
6283	*BEX2*	84707	brain expressed X-linked 2 [Source:HGNC Symbol;Acc:HGNC:30933]	-3.7	-2
6284	*FANCF*	2188	Fanconi anemia complementation group F [Source:HGNC Symbol;Acc:HGNC:3587]	-3.7	-2
6285	*RHPN2*	85415	rhophilin, Rho GTPase binding protein 2 [Source:HGNC Symbol;Acc:HGNC:19974]	-3.7	-2
6286	*GAGE6*	4103	G antigen 6	-3.7	-2
6287	*LPAR1*	1902	lysophosphatidic acid receptor 1 [Source:HGNC Symbol;Acc:HGNC:3166]	-3.7	-2
6288	*DTD1*	92675	D-tyrosyl-tRNA deacylase 1 [Source:HGNC Symbol;Acc:HGNC:16219]	-3.7	-2
6289	*TOR1AIP2*	163590	torsin 1A interacting protein 2 [Source:HGNC Symbol;Acc:HGNC:24055]	-3.7	-2
6290	*FBF1*	85302	Fas binding factor 1 [Source:HGNC Symbol;Acc:HGNC:24674]	-3.7	-2
6291	*TUBD1*	51174	tubulin delta 1 [Source:HGNC Symbol;Acc:HGNC:16811]	-3.8	-2
6292	*TMEM120A*	83862	transmembrane protein 120A [Source:HGNC Symbol;Acc:HGNC:21697]	-3.8	-2
6293	*LRTOMT*	220074	leucine rich transmembrane and O-methyltransferase domain containing [Source:HGNC Symbol;Acc:HGNC:25033]	-3.8	-2
6294	*UBE3C*	9690	ubiquitin protein ligase E3C [Source:HGNC Symbol;Acc:HGNC:16803]	-3.8	-2
6295	*DENND6A*	201627	DENN domain containing 6A [Source:HGNC Symbol;Acc:HGNC:26635]	-3.8	-2
6296	*TMPRSS3*	64699	transmembrane protease, serine 3 [Source:HGNC Symbol;Acc:HGNC:11877]	-3.8	-2
6297	*GLOD5*	392465	glyoxalase domain containing 5 [Source:HGNC Symbol;Acc:HGNC:33358]	-3.8	-2
6298	*C12orf43*	64897	chromosome 12 open reading frame 43 [Source:HGNC Symbol;Acc:HGNC:25719]	-3.8	-2
6299	*HDAC11*	79885	histone deacetylase 11 [Source:HGNC Symbol;Acc:HGNC:19086]	-3.8	-2
6300	*LDOC1*	23641	leucine zipper, down-regulated in cancer 1 [Source:HGNC Symbol;Acc:HGNC:6548]	-3.8	-2

6301	*TMEM50A*	23585	transmembrane protein 50A [Source:HGNC Symbol;Acc:HGNC:30590]	-3.8	-2
6302	*PNMA2*	10687	paraneoplastic Ma antigen 2 [Source:HGNC Symbol;Acc:HGNC:9159]	-3.8	-2
6303	*STX5*	6811	syntaxin 5 [Source:HGNC Symbol;Acc:HGNC:11440]	-3.8	-2
6304	*COG2*	22796	component of oligomeric golgi complex 2 [Source:HGNC Symbol;Acc:HGNC:6546]	-3.8	-2
6305	*ARHGEF5*	7984	Rho guanine nucleotide exchange factor 5 [Source:HGNC Symbol;Acc:HGNC:13209]	-3.8	-2
6306	*EBF2*	64641	early B-cell factor 2 [Source:HGNC Symbol;Acc:HGNC:19090]	-3.8	-2
6307	*DLST*	1743	dihydrolipoamide S-succinyltransferase [Source:HGNC Symbol;Acc:HGNC:2911]	-3.8	-2
6308	*ZNF609*	23060	zinc finger protein 609 [Source:HGNC Symbol;Acc:HGNC:29003]	-3.8	-2
6309	*DNAL1*	83544	dynein axonemal light chain 1 [Source:HGNC Symbol;Acc:HGNC:23247]	-3.8	-2
6310	*TRIM68*	55128	tripartite motif containing 68 [Source:HGNC Symbol;Acc:HGNC:21161]	-3.8	-2
6311	*TRAPPC2*	6399	trafficking protein particle complex 2 [Source:HGNC Symbol;Acc:HGNC:23068]	-3.8	-2
6312	*EHD4*	30844	EH domain containing 4 [Source:HGNC Symbol;Acc:HGNC:3245]	-3.8	-2
6313	*C9orf9*	11092	chromosome 9 open reading frame 9 [Source:HGNC Symbol;Acc:HGNC:24513]	-3.8	-2
6314	*VSIG2*	23584	V-set and immunoglobulin domain containing 2 [Source:HGNC Symbol;Acc:HGNC:17149]	-3.8	-2
6315	*XKR9*	389668	XK related 9 [Source:HGNC Symbol;Acc:HGNC:20937]	-3.8	-2
6316	*PNPT1*	87178	polyribonucleotide nucleotidyltransferase 1 [Source:HGNC Symbol;Acc:HGNC:23166]	-3.8	-2
6317	*ALKBH3*	221120	alkB homolog 3, alpha-ketoglutaratedependent dioxygenase [Source:HGNC Symbol;Acc:HGNC:30141]	-3.8	-2
6318	*ZNF652*	22834	zinc finger protein 652 [Source:HGNC Symbol;Acc:HGNC:29147]	-3.8	-2
6319	*ALPL*	249	alkaline phosphatase, liver/bone/kidney [Source:HGNC Symbol;Acc:HGNC:438]	-3.8	-2
6320	*UBXN7*	26043	UBX domain protein 7 [Source:HGNC Symbol;Acc:HGNC:29119]	-3.8	-2
6321	*RNF144A*	9781	ring finger protein 144A [Source:HGNC Symbol;Acc:HGNC:20457]	-3.8	-2
6322	*STRADB*	55437	STE20-related kinase adaptor beta [Source:HGNC Symbol;Acc:HGNC:13205]	-3.8	-2
6323	*MOG*	4340	myelin oligodendrocyte glycoprotein [Source:HGNC Symbol;Acc:HGNC:7197]	-3.8	-2
6324	*C5*	727	complement component 5 [Source:HGNC Symbol;Acc:HGNC:1331]	-3.8	-2
6325	*PRKCG*	5582	protein kinase C gamma [Source:HGNC Symbol;Acc:HGNC:9402]	-3.8	-2
6326	*MED29*	55588	mediator complex subunit 29 [Source:HGNC Symbol;Acc:HGNC:23074]	-3.8	-2
6327	*CCDC136*	64753	coiled-coil domain containing 136 [Source:HGNC Symbol;Acc:HGNC:22225]	-3.8	-2
6328	*PRICKLE1*	144165	prickle planar cell polarity protein 1 [Source:HGNC Symbol;Acc:HGNC:17019]	-3.8	-2
6329	*KLC3*	147700	kinesin light chain 3 [Source:HGNC Symbol;Acc:HGNC:20717]	-3.8	-2
6330	*SLC13A5*	284111	solute carrier family 13 member 5 [Source:HGNC Symbol;Acc:HGNC:23089]	-3.8	-2
6331	*TRIM6-TRIM34*	445372	TRIM6-TRIM34 Readthrough	-3.8	-2
6332	*MBTPS1*	8720	membrane bound transcription factor peptidase, site 1 [Source:HGNC Symbol;Acc:HGNC:15456]	-3.8	-2
6333	*INTS6*	26512	integrator complex subunit 6 [Source:HGNC Symbol;Acc:HGNC:14879]	-3.8	-2
6334	*SERPINE3*	647174	serpin family E member 3 [Source:HGNC Symbol;Acc:HGNC:24774]	-3.8	-2
6335	*ZNF197*	10168	zinc finger protein 197 [Source:HGNC Symbol;Acc:HGNC:12988]	-3.8	-2
6336	*IDO1*	3620	indoleamine 2,3-dioxygenase 1 [Source:HGNC Symbol;Acc:HGNC:6059]	-3.8	-2
6337	*CHMP4C*	92421	charged multivesicular body protein 4C [Source:HGNC Symbol;Acc:HGNC:30599]	-3.8	-2
6338	*SULT2A1*	6822	sulfotransferase family 2A member 1 [Source:HGNC Symbol;Acc:HGNC:11458]	-3.8	-2
6339	*DUSP16*	80824	dual specificity phosphatase 16 [Source:HGNC Symbol;Acc:HGNC:17909]	-3.8	-2
6340	*ERGIC1*	57222	endoplasmic reticulum-golgi intermediate compartment 1 [Source:HGNC Symbol;Acc:HGNC:29205]	-3.8	-2
6341	*KANK4*	163782	KN motif and ankyrin repeat domains 4 [Source:HGNC Symbol;Acc:HGNC:27263]	-3.8	-2
6342	*SPTSSA*	171546	serine palmitoyltransferase small subunit A [Source:HGNC Symbol;Acc:HGNC:20361]	-3.8	-2
6343	*ZYG11A*	440590	zyg-11 family member A, cell cycle regulator [Source:HGNC Symbol;Acc:HGNC:32058]	-3.8	-2
6344	*ISG15*	9636	ISG15 ubiquitin-like modifier [Source:HGNC Symbol;Acc:HGNC:4053]	-3.8	-2
6345	*BMP8B*	656	bone morphogenetic protein 8b [Source:HGNC Symbol;Acc:HGNC:1075]	-3.8	-2
6346	*RIPPLY3*	53820	ripply transcriptional repressor 3 [Source:HGNC Symbol;Acc:HGNC:3047]	-3.8	-2
6347	*FOXM1*	2305	forkhead box M1 [Source:HGNC Symbol;Acc:HGNC:3818]	-3.8	-2
6348	*SLC12A8*	84561	solute carrier family 12 member 8 [Source:HGNC Symbol;Acc:HGNC:15595]	-3.8	-2
6349	*GSG1*	83445	germ cell associated 1 [Source:HGNC Symbol;Acc:HGNC:19716]	-3.8	-2
6350	*RAB21*	23011	RAB21, member RAS oncogene family [Source:HGNC Symbol;Acc:HGNC:18263]	-3.8	-2
6351	*BRD1*	23774	bromodomain containing 1 [Source:HGNC Symbol;Acc:HGNC:1102]	-3.8	-2
6352	*RAB3B*	5865	RAB3B, member RAS oncogene family [Source:HGNC Symbol;Acc:HGNC:9778]	-3.8	-2
6353	*BRDT*	676	bromodomain testis associated [Source:HGNC Symbol;Acc:HGNC:1105]	-3.8	-2
6354	*LYPD3*	27076	LY6/PLAUR domain containing 3 [Source:HGNC Symbol;Acc:HGNC:24880]	-3.8	-2
6355	*METTL8*	79828	methyltransferase like 8 [Source:HGNC Symbol;Acc:HGNC:25856]	-3.8	-2
6356	*MAP3K15*	389840	mitogen-activated protein kinase kinase kinase 15 [Source:HGNC Symbol;Acc:HGNC:31689]	-3.8	-2
6357	*AMDHD2*	51005	amidohydrolase domain containing 2 [Source:HGNC Symbol;Acc:HGNC:24262]	-3.8	-2
6358	*CEMP1*	752014	cementum protein 1 [Source:HGNC Symbol;Acc:HGNC:32553]	-3.8	-2
6359	*COMP*	1311	cartilage oligomeric matrix protein [Source:HGNC Symbol;Acc:HGNC:2227]	-3.8	-2
6360	*FAM81B*	153643	family with sequence similarity 81 member B [Source:HGNC Symbol;Acc:HGNC:26335]	-3.8	-2
6361	*TTLL5*	23093	tubulin tyrosine ligase like 5 [Source:HGNC Symbol;Acc:HGNC:19963]	-3.8	-2
6362	*CUL3*	8452	cullin 3 [Source:HGNC Symbol;Acc:HGNC:2553]	-3.8	-2
6363	*GHDC*	84514	GH3 domain containing [Source:HGNC Symbol;Acc:HGNC:24438]	-3.8	-2
6364	*PHLDB1*	23187	pleckstrin homology like domain family B member 1 [Source:HGNC Symbol;Acc:HGNC:23697]	-3.8	-2
6365	*KCNJ5*	3762	potassium voltage-gated channel subfamily J member 5 [Source:HGNC Symbol;Acc:HGNC:6266]	-3.8	-2
6366	*AFMID*	125061	arylformamidase [Source:HGNC Symbol;Acc:HGNC:20910]	-3.8	-2
6367	*ZNF639*	51193	zinc finger protein 639 [Source:HGNC Symbol;Acc:HGNC:30950]	-3.8	-2
6368	*ZNF43*	7594	zinc finger protein 43 [Source:HGNC Symbol;Acc:HGNC:13109]	-3.8	-2
6369	*REEP3*	221035	receptor accessory protein 3 [Source:HGNC Symbol;Acc:HGNC:23711]	-3.8	-2
6370	*SPRYD4*	283377	SPRY domain containing 4 [Source:HGNC Symbol;Acc:HGNC:27468]	-3.8	-2
6371	*ZNF791*	163049	zinc finger protein 791 [Source:HGNC Symbol;Acc:HGNC:26895]	-3.8	-2
6372	*LRRIQ1*	84125	leucine rich repeats and IQ motif containing 1 [Source:HGNC Symbol;Acc:HGNC:25708]	-3.8	-2
6373	*C7orf31*	136895	chromosome 7 open reading frame 31 [Source:HGNC Symbol;Acc:HGNC:21722]	-3.8	-2
6374	*TAF8*	129685	TATA-box binding protein associated factor 8 [Source:HGNC Symbol;Acc:HGNC:17300]	-3.8	-2
6375	*LRP2BP*	55805	LRP2 binding protein [Source:HGNC Symbol;Acc:HGNC:25434]	-3.8	-2
6376	*ZSCAN25*	221785	zinc finger and SCAN domain containing 25 [Source:HGNC Symbol;Acc:HGNC:21961]	-3.8	-2
6377	*LRP6*	4040	LDL receptor related protein 6 [Source:HGNC Symbol;Acc:HGNC:6698]	-3.8	-2
6378	*AASS*	10157	aminoadipate-semialdehyde synthase [Source:HGNC Symbol;Acc:HGNC:17366]	-3.9	-2

6379	*PCNXL4*	64430	Pecanex Homolog 4	-3.9	-2
6380	*FBXO36*	130888	F-box protein 36 [Source:HGNC Symbol;Acc:HGNC:27020]	-3.9	-2
6381	*ESCO2*	157570	establishment of sister chromatid cohesion N-acetyltransferase 2 [Source:HGNC Symbol;Acc:HGNC:27230]	-3.9	-2
6382	*CXCL8*	3576	C-X-C motif chemokine ligand 8 [Source:HGNC Symbol;Acc:HGNC:6025]	-3.9	-2
6383	*IRF9*	10379	interferon regulatory factor 9 [Source:HGNC Symbol;Acc:HGNC:6131]	-3.9	-2
6384	*ACE*	1636	angiotensin I converting enzyme [Source:HGNC Symbol;Acc:HGNC:2707]	-3.9	-2
6385	*ITGAE*	3682	integrin subunit alpha E [Source:HGNC Symbol;Acc:HGNC:6147]	-3.9	-2
6386	*DDX52*	11056	DEAD-box helicase 52 [Source:HGNC Symbol;Acc:HGNC:20038]	-3.9	-2
6387	*RUBCN*	9711	RUN and cysteine rich domain containing beclin 1 interacting protein [Source:HGNC Symbol;Acc:HGNC:28991]	-3.9	-2
6388	*NANOG*	79923	Nanog homeobox [Source:HGNC Symbol;Acc:HGNC:20857]	-3.9	-2
6389	*TNFSF15*	9966	tumor necrosis factor superfamily member 15 [Source:HGNC Symbol;Acc:HGNC:11931]	-3.9	-2
6390	*SART3*	9733	squamous cell carcinoma antigen recognized by T-cells 3 [Source:HGNC Symbol;Acc:HGNC:16860]	-3.9	-2
6391	*AKR1B10*	57016	aldo-keto reductase family 1 member B10 [Source:HGNC Symbol;Acc:HGNC:382]	-3.9	-2
6392	*FAM151B*	167555	family with sequence similarity 151 member B [Source:HGNC Symbol;Acc:HGNC:33716]	-3.9	-2
6393	*PHACTR1*	221692	phosphatase and actin regulator 1 [Source:HGNC Symbol;Acc:HGNC:20990]	-3.9	-2
6394	*CLASP2*	23122	cytoplasmic linker associated protein 2 [Source:HGNC Symbol;Acc:HGNC:17078]	-3.9	-2
6395	*IFNAR1*	3454	interferon alpha and beta receptor subunit 1 [Source:HGNC Symbol;Acc:HGNC:5432]	-3.9	-2
6396	*MISP*	126353	mitotic spindle positioning [Source:HGNC Symbol;Acc:HGNC:27000]	-3.9	-2
6397	*ABHD2*	11057	abhydrolase domain containing 2 [Source:HGNC Symbol;Acc:HGNC:18717]	-3.9	-2
6398	*PLA2G4E*	123745	phospholipase A2 group IVE [Source:HGNC Symbol;Acc:HGNC:24791]	-3.9	-2
6399	*PRSS27*	83886	protease, serine 27 [Source:HGNC Symbol;Acc:HGNC:15475]	-3.9	-2
6400	*ALDH3B2*	222	aldehyde dehydrogenase 3 family member B2 [Source:HGNC Symbol;Acc:HGNC:411]	-3.9	-2
6401	*HIF3A*	64344	hypoxia inducible factor 3 alpha subunit [Source:HGNC Symbol;Acc:HGNC:15825]	-3.9	-2
6402	*PGAP3*	93210	post-GPI attachment to proteins 3 [Source:HGNC Symbol;Acc:HGNC:23719]	-3.9	-2
6403	*OSR2*	116039	odd-skipped related transciption factor 2 [Source:HGNC Symbol;Acc:HGNC:15830]	-3.9	-2
6404	*AARS2*	57505	alanyl-tRNA synthetase 2, mitochondrial [Source:HGNC Symbol;Acc:HGNC:21022]	-3.9	-2
6405	*SPATS2L*	26010	spermatogenesis associated serine rich 2 like [Source:HGNC Symbol;Acc:HGNC:24574]	-3.9	-2
6406	*EGR1*	1958	early growth response 1 [Source:HGNC Symbol;Acc:HGNC:3238]	-3.9	-2
6407	*DNAJC18*	202052	DnaJ heat shock protein family (Hsp40) member C18 [Source:HGNC Symbol;Acc:HGNC:28429]	-3.9	-2
6408	*SAP30L*	79685	SAP30-like [Source:HGNC Symbol;Acc:HGNC:25663]	-3.9	-2
6409	*C21orf58*	54058	chromosome 21 open reading frame 58 [Source:HGNC Symbol;Acc:HGNC:1300]	-3.9	-2
6410	*NQO1*	1728	NAD(P)H quinone dehydrogenase 1 [Source:HGNC Symbol;Acc:HGNC:2874]	-3.9	-2
6411	*LENEP*	55891	lens epithelial protein [Source:HGNC Symbol;Acc:HGNC:14429]	-3.9	-2
6412	*BHLHE41*	79365	basic helix-loop-helix family member e41 [Source:HGNC Symbol;Acc:HGNC:16617]	-3.9	-2
6413	*CPAMD8*	27151	C3 and PZP like, alpha-2-macroglobulin domain containing 8 [Source:HGNC Symbol;Acc:HGNC:23228]	-3.9	-2
6414	*SSTR2*	6752	somatostatin receptor 2 [Source:HGNC Symbol;Acc:HGNC:11331]	-3.9	-2
6415	*SHISA5*	51246	shisa family member 5 [Source:HGNC Symbol;Acc:HGNC:30376]	-3.9	-2
6416	*GRK4*	2868	G protein-coupled receptor kinase 4 [Source:HGNC Symbol;Acc:HGNC:4543]	-3.9	-2
6417	*NOL9*	79707	nucleolar protein 9 [Source:HGNC Symbol;Acc:HGNC:26265]	-3.9	-2
6418	*IYD*	389434	iodotyrosine deiodinase [Source:HGNC Symbol;Acc:HGNC:21071]	-3.9	-2
6419	*PUS10*	150962	pseudouridylate synthase 10 [Source:HGNC Symbol;Acc:HGNC:26505]	-3.9	-2
6420	*RRP36*	88745	ribosomal RNA processing 36 [Source:HGNC Symbol;Acc:HGNC:21374]	-3.9	-2
6421	*LIMD1*	8994	LIM domains containing 1 [Source:HGNC Symbol;Acc:HGNC:6612]	-3.9	-2
6422	*PPIE*	10450	peptidylprolyl isomerase E [Source:HGNC Symbol;Acc:HGNC:9258]	-3.9	-2
6423	*TMEM41A*	90407	transmembrane protein 41A [Source:HGNC Symbol;Acc:HGNC:30544]	-3.9	-2
6424	*SEC14L3*	266629	SEC14 like lipid binding 3 [Source:HGNC Symbol;Acc:HGNC:18655]	-3.9	-2
6425	*ALG8*	79053	ALG8, alpha-1,3-glucosyltransferase [Source:HGNC Symbol;Acc:HGNC:23161]	-3.9	-2
6426	*CPSF3L*	54973	cleavage and polyadenylation specific factor 3-like [Source:HGNC Symbol;Acc:HGNC:26052]	-3.9	-2
6427	*AKR1C2*	1646	aldo-keto reductase family 1 member C2 [Source:HGNC Symbol;Acc:HGNC:385]	-3.9	-2
6428	*ARL8B*	55207	ADP ribosylation factor like GTPase 8B [Source:HGNC Symbol;Acc:HGNC:25564]	-3.9	-2
6429	*C1QTNF6*	114904	C1q and tumor necrosis factor related protein 6 [Source:HGNC Symbol;Acc:HGNC:14343]	-3.9	-2
6430	*CAMK1D*	57118	calcium/calmodulin dependent protein kinase ID [Source:HGNC Symbol;Acc:HGNC:19341]	-3.9	-2
6431	*DCAF4*	26094	DDB1 and CUL4 associated factor 4 [Source:HGNC Symbol;Acc:HGNC:20229]	-3.9	-2
6432	*CDHR3*	222256	cadherin related family member 3 [Source:HGNC Symbol;Acc:HGNC:26308]	-3.9	-2
6433	*ANP32E*	81611	acidic nuclear phosphoprotein 32 family member E [Source:HGNC Symbol;Acc:HGNC:16673]	-3.9	-2
6434	*TMEM175*	84286	transmembrane protein 175 [Source:HGNC Symbol;Acc:HGNC:28709]	-3.9	-2
6435	*OSMR*	9180	oncostatin M receptor [Source:HGNC Symbol;Acc:HGNC:8507]	-3.9	-2
6436	*GTF3C1*	2975	general transcription factor IIIC subunit 1 [Source:HGNC Symbol;Acc:HGNC:4664]	-3.9	-2
6437	*NICN1*	84276	nicolin 1 [Source:HGNC Symbol;Acc:HGNC:18317]	-3.9	-2
6438	*ZBTB47*	92999	zinc finger and BTB domain containing 47 [Source:HGNC Symbol;Acc:HGNC:26955]	-3.9	-2
6439	*PGPEP1*	54858	pyroglutamyl-peptidase I [Source:HGNC Symbol;Acc:HGNC:13568]	-3.9	-2
6440	*SCNM1*	79005	sodium channel modifier 1 [Source:HGNC Symbol;Acc:HGNC:23136]	-3.9	-2
6441	*SH3BGR*	6450	SH3 domain binding glutamate rich protein [Source:HGNC Symbol;Acc:HGNC:10822]	-3.9	-2
6442	*ZNF721*	170960	zinc finger protein 721 [Source:HGNC Symbol;Acc:HGNC:29425]	-3.9	-2
6443	*NARS2*	79731	asparaginyl-tRNA synthetase 2, mitochondrial (putative) [Source:HGNC Symbol;Acc:HGNC:26274]	-4.0	-2
6444	*ATL1*	51062	atlastin GTPase 1 [Source:HGNC Symbol;Acc:HGNC:11231]	-4.0	-2
6445	*TRPM1*	4308	transient receptor potential cation channel subfamily M member 1 [Source:HGNC Symbol;Acc:HGNC:7146]	-4.0	-2
6446	*PGAP1*	80055	post-GPI attachment to proteins 1 [Source:HGNC Symbol;Acc:HGNC:25712]	-4.0	-2
6447	*PIGV*	55650	phosphatidylinositol glycan anchor biosynthesis class V [Source:HGNC Symbol;Acc:HGNC:26031]	-4.0	-2
6448	*TVP23C*	201158	trans-golgi network vesicle protein 23 homolog C (S. cerevisiae) [Source:HGNC Symbol;Acc:HGNC:30453]	-4.0	-2
6449	*SLC2A11*	66035	solute carrier family 2 member 11 [Source:HGNC Symbol;Acc:HGNC:14239]	-4.0	-2
6450	*RARRES2*	5919	retinoic acid receptor responder 2 [Source:HGNC Symbol;Acc:HGNC:9868]	-4.0	-2

6451	*JMJD7-PLA2G4B*	8681	JMJD7-PLA2G4B readthrough [Source:HGNC Symbol;Acc:HGNC:34449]	-4.0	-2
6452	*ARL10*	285598	ADP ribosylation factor like GTPase 10 [Source:HGNC Symbol;Acc:HGNC:22042]	-4.0	-2
6453	*DXO*	1797	decapping exoribonuclease [Source:HGNC Symbol;Acc:HGNC:2992]	-4.0	-2
6454	*KANK2*	25959	KN motif and ankyrin repeat domains 2 [Source:HGNC Symbol;Acc:HGNC:29300]	-4.0	-2
6455	*ALDH2*	217	aldehyde dehydrogenase 2 family (mitochondrial) [Source:HGNC Symbol;Acc:HGNC:404]	-4.0	-2
6456	*FKBP11*	51303	FK506 binding protein 11 [Source:HGNC Symbol;Acc:HGNC:18624]	-4.0	-2
6457	*NOM1*	64434	nucleolar protein with MIF4G domain 1 [Source:HGNC Symbol;Acc:HGNC:13244]	-4.0	-2
6458	*LNX2*	222484	ligand of numb-protein X 2 [Source:HGNC Symbol;Acc:HGNC:20421]	-4.0	-2
6459	*NFIA*	4774	nuclear factor I A [Source:HGNC Symbol;Acc:HGNC:7784]	-4.0	-2
6460	*AP2A1*	160	adaptor related protein complex 2 alpha 1 subunit [Source:HGNC Symbol;Acc:HGNC:561]	-4.0	-2
6461	*ATAD3C*	219293	ATPase family, AAA domain containing 3C [Source:HGNC Symbol;Acc:HGNC:32151]	-4.0	-2
6462	*DPYSL5*	56896	dihydropyrimidinase like 5 [Source:HGNC Symbol;Acc:HGNC:20637]	-4.0	-2
6463	*PCBD2*	84105	pterin-4 alpha-carbinolamine dehydratase 2 [Source:HGNC Symbol;Acc:HGNC:24474]	-4.0	-2
6464	*RHD*	6007	Rh blood group D antigen [Source:HGNC Symbol;Acc:HGNC:10009]	-4.0	-2
6465	*NSL1*	25936	NSL1, MIS12 kinetochore complex component [Source:HGNC Symbol;Acc:HGNC:24548]	-4.0	-2
6466	*TMEM138*	51524	transmembrane protein 138 [Source:HGNC Symbol;Acc:HGNC:26944]	-4.0	-2
6467	*CCDC62*	84660	coiled-coil domain containing 62 [Source:HGNC Symbol;Acc:HGNC:30723]	-4.0	-2
6468	*CA1*	759	carbonic anhydrase 1 [Source:HGNC Symbol;Acc:HGNC:1368]	-4.0	-2
6469	*IRAK2*	3656	interleukin 1 receptor associated kinase 2 [Source:HGNC Symbol;Acc:HGNC:6113]	-4.0	-2
6470	*ZFYVE16*	9765	zinc finger FYVE-type containing 16 [Source:HGNC Symbol;Acc:HGNC:20756]	-4.0	-2
6471	*SLC11A2*	4891	solute carrier family 11 member 2 [Source:HGNC Symbol;Acc:HGNC:10908]	-4.0	-2
6472	*ITGB1BP1*	9270	integrin subunit beta 1 binding protein 1 [Source:HGNC Symbol;Acc:HGNC:23927]	-4.0	-2
6473	*SNAPC1*	6617	small nuclear RNA activating complex polypeptide 1 [Source:HGNC Symbol;Acc:HGNC:11134]	-4.0	-2
6474	*MS4A8*	83661	membrane spanning 4-domains A8 [Source:HGNC Symbol;Acc:HGNC:13380]	-4.0	-2
6475	*KCNN3*	3782	potassium calcium-activated channel subfamily N member 3 [Source:HGNC Symbol;Acc:HGNC:6292]	-4.0	-2
6476	*FAM3A*	60343	family with sequence similarity 3 member A [Source:HGNC Symbol;Acc:HGNC:13749]	-4.0	-2
6477	*DSG3*	1830	desmoglein 3 [Source:HGNC Symbol;Acc:HGNC:3050]	-4.0	-2
6478	*WDR33*	55339	WD repeat domain 33 [Source:HGNC Symbol;Acc:HGNC:25651]	-4.0	-2
6479	*CCDC79*	283847	coiled-coil domain containing 79	-4.0	-2
6480	*CYP4V2*	285440	cytochrome P450 family 4 subfamily V member 2 [Source:HGNC Symbol;Acc:HGNC:23198]	-4.0	-2
6481	*ZMIZ1*	57178	zinc finger MIZ-type containing 1 [Source:HGNC Symbol;Acc:HGNC:16493]	-4.0	-2
6482	*CYP1B1*	1545	cytochrome P450 family 1 subfamily B member 1 [Source:HGNC Symbol;Acc:HGNC:2597]	-4.0	-2
6483	*INTS3*	65123	integrator complex subunit 3 [Source:HGNC Symbol;Acc:HGNC:26153]	-4.0	-2
6484	*ARID4A*	5926	AT-rich interaction domain 4A [Source:HGNC Symbol;Acc:HGNC:9885]	-4.0	-2
6485	*RASGRP4*	115727	RAS guanyl releasing protein 4 [Source:HGNC Symbol;Acc:HGNC:18958]	-4.0	-2
6486	*CCDC142*	84865	coiled-coil domain containing 142 [Source:HGNC Symbol;Acc:HGNC:25889]	-4.0	-2
6487	*GALNT16*	57452	polypeptide N-acetylgalactosaminyltransferase 16 [Source:HGNC Symbol;Acc:HGNC:23233]	-4.0	-2
6488	*PAG1*	55824	phosphoprotein membrane anchor with glycosphingolipid microdomains 1 [Source:HGNC Symbol;Acc:HGNC:30043]	-4.0	-2
6489	*MANBAL*	63905	mannosidase beta like [Source:HGNC Symbol;Acc:HGNC:15799]	-4.0	-2
6490	*AHRR*	57491	aryl-hydrocarbon receptor repressor [Source:HGNC Symbol;Acc:HGNC:346]	-4.0	-2
6491	*KIAA0922*	23240	KIAA0922 [Source:HGNC Symbol;Acc:HGNC:29146]	-4.0	-2
6492	*LAMP3*	27074	lysosomal associated membrane protein 3 [Source:HGNC Symbol;Acc:HGNC:14582]	-4.0	-2
6493	*TAF10*	6881	TATA-box binding protein associated factor 10 [Source:HGNC Symbol;Acc:HGNC:11543]	-4.0	-2
6494	*SLAMF1*	6504	signaling lymphocytic activation molecule family member 1 [Source:HGNC Symbol;Acc:HGNC:10903]	-4.0	-2
6495	*ATP1B4*	23439	ATPase Na+/K+ transporting family member beta 4 [Source:HGNC Symbol;Acc:HGNC:808]	-4.0	-2
6496	*IL11*	3589	interleukin 11 [Source:HGNC Symbol;Acc:HGNC:5966]	-4.0	-2
6497	*CA14*	23632	carbonic anhydrase 14 [Source:HGNC Symbol;Acc:HGNC:1372]	-4.0	-2
6498	*GPR1*	2825	G protein-coupled receptor 1 [Source:HGNC Symbol;Acc:HGNC:4463]	-4.0	-2
6499	*PRKCI*	5584	protein kinase C iota [Source:HGNC Symbol;Acc:HGNC:9404]	-4.0	-2
6500	*ABI3BP*	25890	ABI family member 3 binding protein [Source:HGNC Symbol;Acc:HGNC:17265]	-4.1	-2
6501	*CDH18*	1016	cadherin 18 [Source:HGNC Symbol;Acc:HGNC:1757]	-4.1	-2
6502	*AKAP5*	9495	A-kinase anchoring protein 5 [Source:HGNC Symbol;Acc:HGNC:375]	-4.1	-2
6503	*DPP9*	91039	dipeptidyl peptidase 9 [Source:HGNC Symbol;Acc:HGNC:18648]	-4.1	-2
6504	*NDUFAF5*	79133	NADH:ubiquinone oxidoreductase complex assembly factor 5 [Source:HGNC Symbol;Acc:HGNC:15899]	-4.1	-2
6505	*TRPM6*	140803	transient receptor potential cation channel subfamily M member 6 [Source:HGNC Symbol;Acc:HGNC:17995]	-4.1	-2
6506	*PRR23A*	729627	proline rich 23A [Source:HGNC Symbol;Acc:HGNC:37172]	-4.1	-2
6507	*SOCS2*	8835	suppressor of cytokine signaling 2 [Source:HGNC Symbol;Acc:HGNC:19382]	-4.1	-2
6508	*TRPM4*	54795	transient receptor potential cation channel subfamily M member 4 [Source:HGNC Symbol;Acc:HGNC:17993]	-4.1	-2
6509	*CEP63*	80254	centrosomal protein 63 [Source:HGNC Symbol;Acc:HGNC:25815]	-4.1	-2
6510	*SLC25A15*	10166	solute carrier family 25 member 15 [Source:HGNC Symbol;Acc:HGNC:10985]	-4.1	-2
6511	*FPR2*	2358	formyl peptide receptor 2 [Source:HGNC Symbol;Acc:HGNC:3827]	-4.1	-2
6512	*FOXO1*	2308	forkhead box O1 [Source:HGNC Symbol;Acc:HGNC:3819]	-4.1	-2
6513	*SFN*	2810	stratifin [Source:HGNC Symbol;Acc:HGNC:10773]	-4.1	-2
6514	*USHBP1*	83878	USH1 protein network component harmonin binding protein 1 [Source:HGNC Symbol;Acc:HGNC:24058]	-4.1	-2
6515	*ZHX3*	23051	zinc fingers and homeoboxes 3 [Source:HGNC Symbol;Acc:HGNC:15935]	-4.1	-2
6516	*OLFML3*	56944	olfactomedin like 3 [Source:HGNC Symbol;Acc:HGNC:24956]	-4.1	-2
6517	*DISP2*	85455	dispatched RND transporter family member 2 [Source:HGNC Symbol;Acc:HGNC:19712]	-4.1	-2
6518	*RNF216*	54476	ring finger protein 216 [Source:HGNC Symbol;Acc:HGNC:21698]	-4.1	-2
6519	*ZNF716*	441234	zinc finger protein 716 [Source:HGNC Symbol;Acc:HGNC:32458]	-4.1	-2
6520	*RNF185*	91445	ring finger protein 185 [Source:HGNC Symbol;Acc:HGNC:26783]	-4.1	-2
6521	*MRPL49*	740	mitochondrial ribosomal protein L49 [Source:HGNC Symbol;Acc:HGNC:1176]	-4.1	-2
6522	*AAR2*	25980	AAR2 splicing factor homolog [Source:HGNC Symbol;Acc:HGNC:15886]	-4.1	-2

6523	*THNSL2*	55258	threonine synthase like 2 [Source:HGNC Symbol;Acc:HGNC:25602]	-4.1	-2
6524	*TCN1*	6947	transcobalamin 1 [Source:HGNC Symbol;Acc:HGNC:11652]	-4.1	-2
6525	*MVB12A*	93343	multivesicular body subunit 12A [Source:HGNC Symbol;Acc:HGNC:25153]	-4.1	-2
6526	*IL32*	9235	interleukin 32 [Source:HGNC Symbol;Acc:HGNC:16830]	-4.1	-2
6527	*FZD3*	7976	frizzled class receptor 3 [Source:HGNC Symbol;Acc:HGNC:4041]	-4.1	-2
6528	*CYP2B6*	1555	cytochrome P450 family 2 subfamily B member 6 [Source:HGNC Symbol;Acc:HGNC:2615]	-4.1	-2
6529	*GTF2IRD2B*	389524	GTF2I repeat domain containing 2B [Source:HGNC Symbol;Acc:HGNC:33125]	-4.1	-2
6530	*PLIN2*	123	perilipin 2 [Source:HGNC Symbol;Acc:HGNC:248]	-4.1	-2
6531	*ZNF555*	148254	zinc finger protein 555 [Source:HGNC Symbol;Acc:HGNC:28382]	-4.1	-2
6532	*MRI1*	84245	methylthioribose-1-phosphate isomerase 1 [Source:HGNC Symbol;Acc:HGNC:28469]	-4.1	-2
6533	*LRRC47*	57470	leucine rich repeat containing 47 [Source:HGNC Symbol;Acc:HGNC:29207]	-4.1	-2
6534	*PDLIM7*	9260	PDZ and LIM domain 7 [Source:HGNC Symbol;Acc:HGNC:22958]	-4.1	-2
6535	*SLC25A26*	115286	solute carrier family 25 member 26 [Source:HGNC Symbol;Acc:HGNC:20661]	-4.1	-2
6536	*SLC5A10*	125206	solute carrier family 5 member 10 [Source:HGNC Symbol;Acc:HGNC:23155]	-4.1	-2
6537	*PPP1R27*	116729	protein phosphatase 1 regulatory subunit 27 [Source:HGNC Symbol;Acc:HGNC:16813]	-4.1	-2
6538	*SYNJ2BP*	55333	synaptojanin 2 binding protein [Source:HGNC Symbol;Acc:HGNC:18955]	-4.1	-2
6539	*SRCIN1*	80725	SRC kinase signaling inhibitor 1 [Source:HGNC Symbol;Acc:HGNC:29506]	-4.1	-2
6540	*OTULIN*	90268	OTU deubiquitinase with linear linkage specificity [Source:HGNC Symbol;Acc:HGNC:25118]	-4.1	-2
6541	*SYNJ2*	8871	synaptojanin 2 [Source:HGNC Symbol;Acc:HGNC:11504]	-4.1	-2
6542	*DSTYK*	25778	dual serine/threonine and tyrosine protein kinase [Source:HGNC Symbol;Acc:HGNC:29043]	-4.1	-2
6543	*TSPAN31*	6302	tetraspanin 31 [Source:HGNC Symbol;Acc:HGNC:10539]	-4.1	-2
6544	*ANXA1*	301	annexin A1 [Source:HGNC Symbol;Acc:HGNC:533]	-4.1	-2
6545	*ARL1*	400	ADP ribosylation factor like GTPase 1 [Source:HGNC Symbol;Acc:HGNC:692]	-4.1	-2
6546	*MFN2*	9927	mitofusin 2 [Source:HGNC Symbol;Acc:HGNC:16877]	-4.1	-2
6547	*C2orf72*	257407	chromosome 2 open reading frame 72 [Source:HGNC Symbol;Acc:HGNC:27418]	-4.1	-2
6548	*EFCAB7*	84455	EF-hand calcium binding domain 7 [Source:HGNC Symbol;Acc:HGNC:29379]	-4.1	-2
6549	*DCUN1D2*	55208	defective in cullin neddylation 1 domain containing 2 [Source:HGNC Symbol;Acc:HGNC:20328]	-4.1	-2
6550	*CCBL1*	883	Cysteine Conjugate-Beta Lyase 1	-4.1	-2
6551	*AGO1*	26523	argonaute 1, RISC catalytic component [Source:HGNC Symbol;Acc:HGNC:3262]	-4.1	-2
6552	*SKAP1*	8631	src kinase associated phosphoprotein 1 [Source:HGNC Symbol;Acc:HGNC:15605]	-4.1	-2
6553	*FCHO2*	115548	FCH domain only 2 [Source:HGNC Symbol;Acc:HGNC:25180]	-4.1	-2
6554	*NUDCD3*	23386	NudC domain containing 3 [Source:HGNC Symbol;Acc:HGNC:22208]	-4.2	-2
6555	*LYRM4*	57128	LYR motif containing 4 [Source:HGNC Symbol;Acc:HGNC:21365]	-4.2	-2
6556	*ZMAT3*	64393	zinc finger matrin-type 3 [Source:HGNC Symbol;Acc:HGNC:29983]	-4.2	-2
6557	*HSPA12A*	259217	heat shock protein family A (Hsp70) member 12A [Source:HGNC Symbol;Acc:HGNC:19022]	-4.2	-2
6558	*FBXO27*	126433	F-box protein 27 [Source:HGNC Symbol;Acc:HGNC:18753]	-4.2	-2
6559	*SH3BP1*	23616	SH3 domain binding protein 1 [Source:HGNC Symbol;Acc:HGNC:10824]	-4.2	-2
6560	*ZCCHC11*	23318	zinc finger CCHC-type containing 11 [Source:HGNC Symbol;Acc:HGNC:28981]	-4.2	-2
6561	*PKN3*	29941	protein kinase N3 [Source:HGNC Symbol;Acc:HGNC:17999]	-4.2	-2
6562	*ZDHHC12*	84885	zinc finger DHHC-type containing 12 [Source:HGNC Symbol;Acc:HGNC:19159]	-4.2	-2
6563	*C11orf70*	85016	chromosome 11 open reading frame 70 [Source:HGNC Symbol;Acc:HGNC:28188]	-4.2	-2
6564	*SAMD4B*	55095	sterile alpha motif domain containing 4B [Source:HGNC Symbol;Acc:HGNC:25492]	-4.2	-2
6565	*HSD17B7*	51478	hydroxysteroid 17-beta dehydrogenase 7 [Source:HGNC Symbol;Acc:HGNC:5215]	-4.2	-2
6566	*SLC36A2*	153201	solute carrier family 36 member 2 [Source:HGNC Symbol;Acc:HGNC:18762]	-4.2	-2
6567	*LRP10*	26020	LDL receptor related protein 10 [Source:HGNC Symbol;Acc:HGNC:14553]	-4.2	-2
6568	*PON2*	5445	paraoxonase 2 [Source:HGNC Symbol;Acc:HGNC:9205]	-4.2	-2
6569	*DUSP23*	54935	dual specificity phosphatase 23 [Source:HGNC Symbol;Acc:HGNC:21480]	-4.2	-2
6570	*ACKR2*	1238	atypical chemokine receptor 2 [Source:HGNC Symbol;Acc:HGNC:1565]	-4.2	-2
6571	*NEU1*	4758	neuraminidase 1 (lysosomal sialidase) [Source:HGNC Symbol;Acc:HGNC:7758]	-4.2	-2
6572	*ZNF554*	115196	zinc finger protein 554 [Source:HGNC Symbol;Acc:HGNC:26629]	-4.2	-2
6573	*FLVCR1*	28982	feline leukemia virus subgroup C cellular receptor 1 [Source:HGNC Symbol;Acc:HGNC:24682]	-4.2	-2
6574	*BLOC1S1*	2647	biogenesis of lysosomal organelles complex 1 subunit 1 [Source:HGNC Symbol;Acc:HGNC:4200]	-4.2	-2
6575	*KIAA0355*	9710	KIAA0355 [Source:HGNC Symbol;Acc:HGNC:29016]	-4.2	-2
6576	*STK32A*	202374	serine/threonine kinase 32A [Source:HGNC Symbol;Acc:HGNC:28317]	-4.2	-2
6577	*CEP104*	9731	centrosomal protein 104 [Source:HGNC Symbol;Acc:HGNC:24866]	-4.2	-2
6578	*OSBPL2*	9885	oxysterol binding protein like 2 [Source:HGNC Symbol;Acc:HGNC:15761]	-4.2	-2
6579	*C3orf70*	285382	chromosome 3 open reading frame 70 [Source:HGNC Symbol;Acc:HGNC:33731]	-4.2	-2
6580	*UBE3D*	90025	ubiquitin protein ligase E3D [Source:HGNC Symbol;Acc:HGNC:21381]	-4.2	-2
6581	*TUBGCP6*	85378	tubulin gamma complex associated protein 6 [Source:HGNC Symbol;Acc:HGNC:18127]	-4.2	-2
6582	*ICMT*	23463	isoprenylcysteine carboxyl methyltransferase [Source:HGNC Symbol;Acc:HGNC:5350]	-4.2	-2
6583	*ADTRP*	84830	androgen dependent TFPI regulating protein [Source:HGNC Symbol;Acc:HGNC:21214]	-4.2	-2
6584	*CDK11B*	984	cyclin dependent kinase 11B [Source:HGNC Symbol;Acc:HGNC:1729]	-4.2	-2
6585	*RGL3*	57139	ral guanine nucleotide dissociation stimulator like 3 [Source:HGNC Symbol;Acc:HGNC:30282]	-4.2	-2
6586	*KDELC2*	143888	KDEL motif containing 2 [Source:HGNC Symbol;Acc:HGNC:28496]	-4.2	-2
6587	*ATP10B*	23120	ATPase phospholipid transporting 10B (putative) [Source:HGNC Symbol;Acc:HGNC:13543]	-4.2	-2
6588	*C8orf33*	65265	chromosome 8 open reading frame 33 [Source:HGNC Symbol;Acc:HGNC:26104]	-4.2	-2
6589	*CLEC4M*	10332	C-type lectin domain family 4 member M [Source:HGNC Symbol;Acc:HGNC:13523]	-4.2	-2
6590	*ZNF561*	93134	zinc finger protein 561 [Source:HGNC Symbol;Acc:HGNC:28684]	-4.2	-2
6591	*POLH*	5429	polymerase (DNA) eta [Source:HGNC Symbol;Acc:HGNC:9181]	-4.2	-2
6592	*EEF2K*	29904	eukaryotic elongation factor 2 kinase [Source:HGNC Symbol;Acc:HGNC:24615]	-4.2	-2
6593	*ZNF398*	57541	zinc finger protein 398 [Source:HGNC Symbol;Acc:HGNC:18373]	-4.2	-2
6594	*CBFA2T2*	9139	CBFA2/RUNX1 translocation partner 2 [Source:HGNC Symbol;Acc:HGNC:1536]	-4.2	-2
6595	*TBC1D24*	57465	TBC1 domain family member 24 [Source:HGNC Symbol;Acc:HGNC:29203]	-4.2	-2
6596	*UBXN4*	23190	UBX domain protein 4 [Source:HGNC Symbol;Acc:HGNC:14860]	-4.2	-2
6597	*PDK4*	5166	pyruvate dehydrogenase kinase 4 [Source:HGNC Symbol;Acc:HGNC:8812]	-4.2	-2

6598	*RIPK1*	8737	receptor interacting serine/threonine kinase 1 [Source:HGNC Symbol;Acc:HGNC:10019]	-4.2	-2
6599	*ATP7A*	538	ATPase copper transporting alpha [Source:HGNC Symbol;Acc:HGNC:869]	-4.2	-2
6600	*PTCHD1*	139411	patched domain containing 1 [Source:HGNC Symbol;Acc:HGNC:26392]	-4.2	-2
6601	*LPCAT2*	54947	lysophosphatidylcholine acyltransferase 2 [Source:HGNC Symbol;Acc:HGNC:26032]	-4.2	-2
6602	*DIS3*	22894	DIS3 homolog, exosome endoribonuclease and 3'-5' exoribonuclease [Source:HGNC Symbol;Acc:HGNC:20604]	-4.2	-2
6603	*PHLDB2*	90102	pleckstrin homology like domain family B member 2 [Source:HGNC Symbol;Acc:HGNC:29573]	-4.2	-2
6604	*TMEM17*	200728	transmembrane protein 17 [Source:HGNC Symbol;Acc:HGNC:26623]	-4.2	-2
6605	*IGFBP6*	3489	insulin like growth factor binding protein 6 [Source:HGNC Symbol;Acc:HGNC:5475]	-4.2	-2
6606	*APPBP2*	10513	amyloid beta precursor protein binding protein 2 [Source:HGNC Symbol;Acc:HGNC:622]	-4.2	-2
6607	*GJB3*	2707	gap junction protein beta 3 [Source:HGNC Symbol;Acc:HGNC:4285]	-4.2	-2
6608	*ZNF607*	84775	zinc finger protein 607 [Source:HGNC Symbol;Acc:HGNC:28192]	-4.2	-2
6609	*FAT3*	120114	FAT atypical cadherin 3 [Source:HGNC Symbol;Acc:HGNC:23112]	-4.3	-2
6610	*SYNE2*	23224	spectrin repeat containing nuclear envelope protein 2 [Source:HGNC Symbol;Acc:HGNC:17084]	-4.3	-2
6611	*M6PR*	4074	mannose-6-phosphate receptor, cation dependent [Source:HGNC Symbol;Acc:HGNC:6752]	-4.3	-2
6612	*PPCS*	79717	phosphopantothenoylcysteine synthetase [Source:HGNC Symbol;Acc:HGNC:25686]	-4.3	-2
6613	*ANKRD40*	91369	ankyrin repeat domain 40 [Source:HGNC Symbol;Acc:HGNC:28233]	-4.3	-2
6614	*ZNF773*	374928	zinc finger protein 773 [Source:HGNC Symbol;Acc:HGNC:30487]	-4.3	-2
6615	*KCNRG*	283518	potassium channel regulator [Source:HGNC Symbol;Acc:HGNC:18893]	-4.3	-2
6616	*ZNF234*	10780	zinc finger protein 234 [Source:HGNC Symbol;Acc:HGNC:13027]	-4.3	-2
6617	*SLC22A15*	55356	solute carrier family 22 member 15 [Source:HGNC Symbol;Acc:HGNC:20301]	-4.3	-2
6618	*ZNF346*	23567	zinc finger protein 346 [Source:HGNC Symbol;Acc:HGNC:16403]	-4.3	-2
6619	*TNS4*	84951	tensin 4 [Source:HGNC Symbol;Acc:HGNC:24352]	-4.3	-2
6620	*C5orf22*	55322	chromosome 5 open reading frame 22 [Source:HGNC Symbol;Acc:HGNC:25639]	-4.3	-2
6621	*NIP7*	51388	NIP7, nucleolar pre-rRNA processing protein [Source:HGNC Symbol;Acc:HGNC:24328]	-4.3	-2
6622	*SIK3*	23387	SIK family kinase 3 [Source:HGNC Symbol;Acc:HGNC:29165]	-4.3	-2
6623	*GINS4*	84296	GINS complex subunit 4 [Source:HGNC Symbol;Acc:HGNC:28226]	-4.3	-2
6624	*WDR62*	284403	WD repeat domain 62 [Source:HGNC Symbol;Acc:HGNC:24502]	-4.3	-2
6625	*NMRAL1*	57407	NmrA-like family domain containing 1 [Source:HGNC Symbol;Acc:HGNC:24987]	-4.3	-2
6626	*TMTC4*	84899	transmembrane and tetratricopeptide repeat containing 4 [Source:HGNC Symbol;Acc:HGNC:25904]	-4.3	-2
6627	*SUSD5*	26032	sushi domain containing 5 [Source:HGNC Symbol;Acc:HGNC:29061]	-4.3	-2
6628	*AGPS*	8540	alkylglycerone phosphate synthase [Source:HGNC Symbol;Acc:HGNC:327]	-4.3	-2
6629	*CENPT*	80152	centromere protein T [Source:HGNC Symbol;Acc:HGNC:25787]	-4.3	-2
6630	*ACSM2A*	123876	acyl-CoA synthetase medium-chain family member 2A [Source:HGNC Symbol;Acc:HGNC:32017]	-4.3	-2
6631	*ACOT11*	26027	acyl-CoA thioesterase 11 [Source:HGNC Symbol;Acc:HGNC:18156]	-4.3	-2
6632	*TMX1*	81542	thioredoxin related transmembrane protein 1 [Source:HGNC Symbol;Acc:HGNC:15487]	-4.3	-2
6633	*ACVR1*	90	activin A receptor type 1 [Source:HGNC Symbol;Acc:HGNC:171]	-4.3	-2
6634	*KLK11*	11012	kallikrein related peptidase 11 [Source:HGNC Symbol;Acc:HGNC:6359]	-4.3	-2
6635	*CNNM3*	26505	cyclin and CBS domain divalent metal cation transport mediator 3 [Source:HGNC Symbol;Acc:HGNC:104]	-4.3	-2
6636	*BLVRA*	644	biliverdin reductase A [Source:HGNC Symbol;Acc:HGNC:1062]	-4.3	-2
6637	*MTRF1*	9617	mitochondrial translational release factor 1 [Source:HGNC Symbol;Acc:HGNC:7469]	-4.3	-2
6638	*AHSA2*	130872	AHA1, activator of heat shock 90kDa protein ATPase homolog 2 (yeast) [Source:HGNC Symbol;Acc:HGNC:20437]	-4.3	-2
6639	*DNAJC12*	56521	DnaJ heat shock protein family (Hsp40) member C12 [Source:HGNC Symbol;Acc:HGNC:28908]	-4.3	-2
6640	*LLGL1*	3996	LLGL1, scribble cell polarity complex component [Source:HGNC Symbol;Acc:HGNC:6628]	-4.3	-2
6641	*TRIM60*	166655	tripartite motif containing 60 [Source:HGNC Symbol;Acc:HGNC:21162]	-4.3	-2
6642	*DNAJC4*	3338	DnaJ heat shock protein family (Hsp40) member C4 [Source:HGNC Symbol;Acc:HGNC:5271]	-4.3	-2
6643	*PI4K2A*	55361	phosphatidylinositol 4-kinase type 2 alpha [Source:HGNC Symbol;Acc:HGNC:30031]	-4.3	-2
6644	*AOC1*	26	amine oxidase, copper containing 1 [Source:HGNC Symbol;Acc:HGNC:80]	-4.3	-2
6645	*SUV39H2*	79723	suppressor of variegation 3-9 homolog 2 [Source:HGNC Symbol;Acc:HGNC:17287]	-4.3	-2
6646	*SACM1L*	22908	SAC1 suppressor of actin mutations 1-like (yeast) [Source:HGNC Symbol;Acc:HGNC:17059]	-4.3	-2
6647	*ZC3H12B*	340554	zinc finger CCCH-type containing 12B [Source:HGNC Symbol;Acc:HGNC:17407]	-4.3	-2
6648	*ABHD10*	55347	abhydrolase domain containing 10 [Source:HGNC Symbol;Acc:HGNC:25656]	-4.3	-2
6649	*ZMYM1*	79830	zinc finger MYM-type containing 1 [Source:HGNC Symbol;Acc:HGNC:26253]	-4.3	-2
6650	*APOBEC3F*	200316	apolipoprotein B mRNA editing enzyme catalytic subunit 3F [Source:HGNC Symbol;Acc:HGNC:17356]	-4.3	-2
6651	*LGI3*	203190	leucine rich repeat LGI family member 3 [Source:HGNC Symbol;Acc:HGNC:18711]	-4.3	-2
6652	*NALCN*	259232	sodium leak channel, non-selective [Source:HGNC Symbol;Acc:HGNC:19082]	-4.3	-2
6653	*C12orf50*	160419	chromosome 12 open reading frame 50 [Source:HGNC Symbol;Acc:HGNC:26665]	-4.3	-2
6654	*DCST2*	127579	DC-STAMP domain containing 2 [Source:HGNC Symbol;Acc:HGNC:26562]	-4.3	-2
6655	*CLSPN*	63967	claspin [Source:HGNC Symbol;Acc:HGNC:19715]	-4.3	-2
6656	*TRNAU1AP*	54952	tRNA selenocysteine 1 associated protein 1 [Source:HGNC Symbol;Acc:HGNC:30813]	-4.3	-2
6657	*RND2*	8153	Rho family GTPase 2 [Source:HGNC Symbol;Acc:HGNC:18315]	-4.3	-2
6658	*HKR1*	284459	HKR1, GLI-Kruppel zinc finger family member [Source:HGNC Symbol;Acc:HGNC:4928]	-4.3	-2
6659	*PARK2*	5071	parkin RBR E3 ubiquitin protein ligase [Source:HGNC Symbol;Acc:HGNC:8607]	-4.3	-2
6660	*GTPBP10*	85865	GTP binding protein 10 [Source:HGNC Symbol;Acc:HGNC:25106]	-4.3	-2
6661	*NUCB2*	4925	nucleobindin 2 [Source:HGNC Symbol;Acc:HGNC:8044]	-4.3	-2
6662	*YRDC*	79693	yrdC N6-threonylcarbamoyltransferase domain containing [Source:HGNC Symbol;Acc:HGNC:28905]	-4.3	-2
6663	*S1PR2*	9294	sphingosine-1-phosphate receptor 2 [Source:HGNC Symbol;Acc:HGNC:3169]	-4.3	-2
6664	*KRT6B*	3854	keratin 6B [Source:HGNC Symbol;Acc:HGNC:6444]	-4.3	-2
6665	*ZNF681*	148213	zinc finger protein 681 [Source:HGNC Symbol;Acc:HGNC:26457]	-4.3	-2
6666	*CYBA*	1535	cytochrome b-245 alpha chain [Source:HGNC Symbol;Acc:HGNC:2577]	-4.3	-2
6667	*TNFAIP8L3*	388121	TNF alpha induced protein 8 like 3 [Source:HGNC Symbol;Acc:HGNC:20620]	-4.4	-2

6668	*MPP7*	143098	membrane palmitoylated protein 7 [Source:HGNC Symbol;Acc:HGNC:26542]	-4.4	-2
6669	*TCTN1*	79600	tectonic family member 1 [Source:HGNC Symbol;Acc:HGNC:26113]	-4.4	-2
6670	*FAM117B*	150864	family with sequence similarity 117 member B [Source:HGNC Symbol;Acc:HGNC:14440]	-4.4	-2
6671	*TTC23*	64927	tetratricopeptide repeat domain 23 [Source:HGNC Symbol;Acc:HGNC:25730]	-4.4	-2
6672	*CHRD*	8646	chordin [Source:HGNC Symbol;Acc:HGNC:1949]	-4.4	-2
6673	*ST8SIA1*	6489	ST8 alpha-N-acetyl-neuraminide alpha-2,8-sialyltransferase 1 [Source:HGNC Symbol;Acc:HGNC:10869]	-4.4	-2
6674	*LYRM7*	90624	LYR motif containing 7 [Source:HGNC Symbol;Acc:HGNC:28072]	-4.4	-2
6675	*PBRM1*	55193	polybromo 1 [Source:HGNC Symbol;Acc:HGNC:30064]	-4.4	-2
6676	*ATXN2*	6311	ataxin 2 [Source:HGNC Symbol;Acc:HGNC:10555]	-4.4	-2
6677	*DNAH2*	146754	dynein axonemal heavy chain 2 [Source:HGNC Symbol;Acc:HGNC:2948]	-4.4	-2
6678	*CCDC150*	284992	coiled-coil domain containing 150 [Source:HGNC Symbol;Acc:HGNC:26834]	-4.4	-2
6679	*FRRS1*	391059	ferric chelate reductase 1 [Source:HGNC Symbol;Acc:HGNC:27622]	-4.4	-2
6680	*TMED4*	222068	transmembrane p24 trafficking protein 4 [Source:HGNC Symbol;Acc:HGNC:22301]	-4.4	-2
6681	*GSDMA*	284110	gasdermin A [Source:HGNC Symbol;Acc:HGNC:13311]	-4.4	-2
6682	*PDK1*	5163	pyruvate dehydrogenase kinase 1 [Source:HGNC Symbol;Acc:HGNC:8809]	-4.4	-2
6683	*RGS12*	6002	regulator of G-protein signaling 12 [Source:HGNC Symbol;Acc:HGNC:9994]	-4.4	-2
6684	*ZNF765*	91661	zinc finger protein 765 [Source:HGNC Symbol;Acc:HGNC:25092]	-4.4	-2
6685	*TRIM66*	9866	tripartite motif containing 66 [Source:HGNC Symbol;Acc:HGNC:29005]	-4.4	-2
6686	*HOGA1*	112817	4-hydroxy-2-oxoglutarate aldolase 1 [Source:HGNC Symbol;Acc:HGNC:25155]	-4.4	-2
6687	*ZNF536*	9745	zinc finger protein 536 [Source:HGNC Symbol;Acc:HGNC:29025]	-4.4	-2
6688	*CDHR4*	389118	cadherin related family member 4 [Source:HGNC Symbol;Acc:HGNC:34527]	-4.4	-2
6689	*TPMT*	7172	thiopurine S-methyltransferase [Source:HGNC Symbol;Acc:HGNC:12014]	-4.4	-2
6690	*EGLN1*	54583	egl-9 family hypoxia inducible factor 1 [Source:HGNC Symbol;Acc:HGNC:1232]	-4.4	-2
6691	*FGB*	2244	fibrinogen beta chain [Source:HGNC Symbol;Acc:HGNC:3662]	-4.4	-2
6692	*CEP126*	57562	centrosomal protein 126 [Source:HGNC Symbol;Acc:HGNC:29264]	-4.4	-2
6693	*CA12*	771	carbonic anhydrase 12 [Source:HGNC Symbol;Acc:HGNC:1371]	-4.4	-2
6694	*HCAR1*	27198	hydroxycarboxylic acid receptor 1 [Source:HGNC Symbol;Acc:HGNC:4532]	-4.4	-2
6695	*SPRR1A*	6698	small proline rich protein 1A [Source:HGNC Symbol;Acc:HGNC:11259]	-4.4	-2
6696	*SERTAD2*	9792	SERTA domain containing 2 [Source:HGNC Symbol;Acc:HGNC:30784]	-4.4	-2
6697	*ENC1*	8507	ectodermal-neural cortex 1 [Source:HGNC Symbol;Acc:HGNC:3345]	-4.4	-2
6698	*CYP3A5*	1577	cytochrome P450 family 3 subfamily A member 5 [Source:HGNC Symbol;Acc:HGNC:2638]	-4.4	-2
6699	*PLIN3*	10226	perilipin 3 [Source:HGNC Symbol;Acc:HGNC:16893]	-4.4	-2
6700	*SWSAP1*	126074	SWIM-type zinc finger 7 associated protein 1 [Source:HGNC Symbol;Acc:HGNC:26638]	-4.4	-2
6701	*ZNF329*	79673	zinc finger protein 329 [Source:HGNC Symbol;Acc:HGNC:14209]	-4.4	-2
6702	*TMIGD2*	126259	transmembrane and immunoglobulin domain containing 2 [Source:HGNC Symbol;Acc:HGNC:28324]	-4.4	-2
6703	*APBB1*	322	amyloid beta precursor protein binding family B member 1 [Source:HGNC Symbol;Acc:HGNC:581]	-4.4	-2
6704	*METTL7A*	25840	methyltransferase like 7A [Source:HGNC Symbol;Acc:HGNC:24550]	-4.4	-2
6705	*PRKCA*	5578	protein kinase C alpha [Source:HGNC Symbol;Acc:HGNC:9393]	-4.4	-2
6706	*CAB39*	51719	calcium binding protein 39 [Source:HGNC Symbol;Acc:HGNC:20292]	-4.4	-2
6707	*FHAD1*	114827	forkhead-associated (FHA) phosphopeptide binding domain 1 [Source:HGNC Symbol;Acc:HGNC:29408]	-4.4	-2
6708	*ARMC9*	80210	armadillo repeat containing 9 [Source:HGNC Symbol;Acc:HGNC:20730]	-4.4	-2
6709	*EPHA10*	284656	EPH receptor A10 [Source:HGNC Symbol;Acc:HGNC:19987]	-4.4	-2
6710	*VIPR2*	7434	vasoactive intestinal peptide receptor 2 [Source:HGNC Symbol;Acc:HGNC:12695]	-4.4	-2
6711	*ARHGAP11A*	9824	Rho GTPase activating protein 11A [Source:HGNC Symbol;Acc:HGNC:15783]	-4.4	-2
6712	*PDE7A*	5150	phosphodiesterase 7A [Source:HGNC Symbol;Acc:HGNC:8791]	-4.4	-2
6713	*HDAC6*	10013	histone deacetylase 6 [Source:HGNC Symbol;Acc:HGNC:14064]	-4.4	-2
6714	*FUT10*	84750	fucosyltransferase 10 [Source:HGNC Symbol;Acc:HGNC:19234]	-4.4	-2
6715	*TMEM50B*	757	transmembrane protein 50B [Source:HGNC Symbol;Acc:HGNC:1280]	-4.4	-2
6716	*CC2D1A*	54862	coiled-coil and C2 domain containing 1A [Source:HGNC Symbol;Acc:HGNC:30237]	-4.4	-2
6717	*CAMKK2*	10645	calcium/calmodulin dependent protein kinase kinase 2 [Source:HGNC Symbol;Acc:HGNC:1470]	-4.4	-2
6718	*ADAMTS17*	170691	ADAM metallopeptidase with thrombospondin type 1 motif 17 [Source:HGNC Symbol;Acc:HGNC:17109]	-4.4	-2
6719	*LXN*	56925	latexin [Source:HGNC Symbol;Acc:HGNC:13347]	-4.5	-2
6720	*SRGAP3*	9901	SLIT-ROBO Rho GTPase activating protein 3 [Source:HGNC Symbol;Acc:HGNC:19744]	-4.5	-2
6721	*ANKS1A*	23294	ankyrin repeat and sterile alpha motif domain containing 1A [Source:HGNC Symbol;Acc:HGNC:20961]	-4.5	-2
6722	*DBT*	1629	dihydrolipoamide branched chain transacylase E2 [Source:HGNC Symbol;Acc:HGNC:2698]	-4.5	-2
6723	*AADAT*	51166	aminoadipate aminotransferase [Source:HGNC Symbol;Acc:HGNC:17929]	-4.5	-2
6724	*PDE11A*	50940	phosphodiesterase 11A [Source:HGNC Symbol;Acc:HGNC:8773]	-4.5	-2
6725	*MRPS34*	65993	mitochondrial ribosomal protein S34 [Source:HGNC Symbol;Acc:HGNC:16618]	-4.5	-2
6726	*ANXA9*	8416	annexin A9 [Source:HGNC Symbol;Acc:HGNC:547]	-4.5	-2
6727	*RAB42*	115273	RAB42, member RAS oncogene family [Source:HGNC Symbol;Acc:HGNC:28702]	-4.5	-2
6728	*C2orf83*	56918	chromosome 2 open reading frame 83 [Source:HGNC Symbol;Acc:HGNC:25344]	-4.5	-2
6729	*ZNF471*	57573	zinc finger protein 471 [Source:HGNC Symbol;Acc:HGNC:23226]	-4.5	-2
6730	*VPS53*	55275	VPS53, GARP complex subunit [Source:HGNC Symbol;Acc:HGNC:25608]	-4.5	-2
6731	*PADI1*	29943	peptidyl arginine deiminase 1 [Source:HGNC Symbol;Acc:HGNC:18367]	-4.5	-2
6732	*PPP4C*	5531	protein phosphatase 4 catalytic subunit [Source:HGNC Symbol;Acc:HGNC:9319]	-4.5	-2
6733	*ZNF669*	79862	zinc finger protein 669 [Source:HGNC Symbol;Acc:HGNC:25736]	-4.5	-2
6734	*TMEM33*	55161	transmembrane protein 33 [Source:HGNC Symbol;Acc:HGNC:25541]	-4.5	-2
6735	*WDHD1*	11169	WD repeat and HMG-box DNA binding protein 1 [Source:HGNC Symbol;Acc:HGNC:23170]	-4.5	-2
6736	*MLYCD*	23417	malonyl-CoA decarboxylase [Source:HGNC Symbol;Acc:HGNC:7150]	-4.5	-2
6737	*SLC5A1*	6523	solute carrier family 5 member 1 [Source:HGNC Symbol;Acc:HGNC:11036]	-4.5	-2
6738	*SPRYD7*	57213	SPRY domain containing 7 [Source:HGNC Symbol;Acc:HGNC:14297]	-4.5	-2
6739	*PHLDB3*	653583	pleckstrin homology like domain family B member 3 [Source:HGNC Symbol;Acc:HGNC:30499]	-4.5	-2
6740	*NOL12*	79159	nucleolar protein 12 [Source:HGNC Symbol;Acc:HGNC:28585]	-4.5	-2

6741	*KSR1*	8844	kinase suppressor of ras 1 [Source:HGNC Symbol;Acc:HGNC:6465]	-4.5	-2
6742	*DGKZ*	8525	diacylglycerol kinase zeta [Source:HGNC Symbol;Acc:HGNC:2857]	-4.5	-2
6743	*SMAD4*	4089	SMAD family member 4 [Source:HGNC Symbol;Acc:HGNC:6770]	-4.5	-2
6744	*LMOD1*	25802	leiomodin 1 [Source:HGNC Symbol;Acc:HGNC:6647]	-4.5	-2
6745	*C15orf43*	145645	chromosome 15 open reading frame 43	-4.5	-2
6746	*CDC40*	51362	cell division cycle 40 [Source:HGNC Symbol;Acc:HGNC:17350]	-4.5	-2
6747	*CATSPERD*	257062	cation channel sperm associated auxiliary subunit delta [Source:HGNC Symbol;Acc:HGNC:28598]	-4.5	-2
6748	*WDR76*	79968	WD repeat domain 76 [Source:HGNC Symbol;Acc:HGNC:25773]	-4.5	-2
6749	*HMGCS2*	3158	3-hydroxy-3-methylglutaryl-CoA synthase 2 [Source:HGNC Symbol;Acc:HGNC:5008]	-4.5	-2
6750	*APAF1*	317	apoptotic peptidase activating factor 1 [Source:HGNC Symbol;Acc:HGNC:576]	-4.5	-2
6751	*THAP5*	168451	THAP domain containing 5 [Source:HGNC Symbol;Acc:HGNC:23188]	-4.5	-2
6752	*SLC39A5*	283375	solute carrier family 39 member 5 [Source:HGNC Symbol;Acc:HGNC:20502]	-4.5	-2
6753	*ALDH7A1*	501	aldehyde dehydrogenase 7 family member A1 [Source:HGNC Symbol;Acc:HGNC:877]	-4.5	-2
6754	*DNPEP*	23549	aspartyl aminopeptidase [Source:HGNC Symbol;Acc:HGNC:2981]	-4.5	-2
6755	*C17orf99*	100141515	chromosome 17 open reading frame 99 [Source:HGNC Symbol;Acc:HGNC:34490]	-4.5	-2
6756	*STAMBPL1*	57559	STAM binding protein like 1 [Source:HGNC Symbol;Acc:HGNC:24105]	-4.5	-2
6757	*CENPN*	55839	centromere protein N [Source:HGNC Symbol;Acc:HGNC:30873]	-4.5	-2
6758	*KLHL23*	151230	kelch like family member 23 [Source:HGNC Symbol;Acc:HGNC:27506]	-4.5	-2
6759	*ANAPC15*	25906	anaphase promoting complex subunit 15 [Source:HGNC Symbol;Acc:HGNC:24531]	-4.5	-2
6760	*GPRC5A*	9052	G protein-coupled receptor class C group 5 member A [Source:HGNC Symbol;Acc:HGNC:9836]	-4.5	-2
6761	*CTSH*	1512	cathepsin H [Source:HGNC Symbol;Acc:HGNC:2535]	-4.5	-2
6762	*PLA1A*	51365	phospholipase A1 member A [Source:HGNC Symbol;Acc:HGNC:17661]	-4.5	-2
6763	*PTGDS*	5730	prostaglandin D2 synthase [Source:HGNC Symbol;Acc:HGNC:9592]	-4.5	-2
6764	*C3orf33*	285315	chromosome 3 open reading frame 33 [Source:HGNC Symbol;Acc:HGNC:26434]	-4.6	-2
6765	*ATPAF2*	91647	ATP synthase mitochondrial F1 complex assembly factor 2 [Source:HGNC Symbol;Acc:HGNC:18802]	-4.6	-2
6766	*UMPS*	7372	uridine monophosphate synthetase [Source:HGNC Symbol;Acc:HGNC:12563]	-4.6	-2
6767	*ABCA8*	10351	ATP binding cassette subfamily A member 8 [Source:HGNC Symbol;Acc:HGNC:38]	-4.6	-2
6768	*COX18*	285521	COX18 cytochrome c oxidase assembly factor [Source:HGNC Symbol;Acc:HGNC:26801]	-4.6	-2
6769	*CCDC30*	728621	coiled-coil domain containing 30 [Source:HGNC Symbol;Acc:HGNC:26103]	-4.6	-2
6770	*DCST1*	149095	DC-STAMP domain containing 1 [Source:HGNC Symbol;Acc:HGNC:26539]	-4.6	-2
6771	*TBX6*	6911	T-box 6 [Source:HGNC Symbol;Acc:HGNC:11605]	-4.6	-2
6772	*UTP20*	27340	UTP20, small subunit processome component [Source:HGNC Symbol;Acc:HGNC:17897]	-4.6	-2
6773	*EXD3*	54932	exonuclease 3'-5' domain containing 3 [Source:HGNC Symbol;Acc:HGNC:26023]	-4.6	-2
6774	*SLC7A14*	57709	solute carrier family 7 member 14 [Source:HGNC Symbol;Acc:HGNC:29326]	-4.6	-2
6775	*KIAA1147*	57189	KIAA1147 [Source:HGNC Symbol;Acc:HGNC:29472]	-4.6	-2
6776	*DBP*	1628	D-box binding PAR bZIP transcription factor [Source:HGNC Symbol;Acc:HGNC:2697]	-4.6	-2
6777	*SLC30A5*	64924	solute carrier family 30 member 5 [Source:HGNC Symbol;Acc:HGNC:19089]	-4.6	-2
6778	*RABEPK*	10244	Rab9 effector protein with kelch motifs [Source:HGNC Symbol;Acc:HGNC:16896]	-4.6	-2
6779	*GGCX*	2677	gamma-glutamyl carboxylase [Source:HGNC Symbol;Acc:HGNC:4247]	-4.6	-2
6780	*KANSL3*	55683	KAT8 regulatory NSL complex subunit 3 [Source:HGNC Symbol;Acc:HGNC:25473]	-4.6	-2
6781	*IL17RD*	54756	interleukin 17 receptor D [Source:HGNC Symbol;Acc:HGNC:17616]	-4.6	-2
6782	*SLC35F6*	54978	solute carrier family 35 member F6 [Source:HGNC Symbol;Acc:HGNC:26055]	-4.6	-2
6783	*TMCO4*	255104	transmembrane and coiled-coil domains 4 [Source:HGNC Symbol;Acc:HGNC:27393]	-4.6	-2
6784	*CASP2*	835	caspase 2 [Source:HGNC Symbol;Acc:HGNC:1503]	-4.6	-2
6785	*NAA20*	51126	N(alpha)-acetyltransferase 20, NatB catalytic subunit [Source:HGNC Symbol;Acc:HGNC:15908]	-4.6	-2
6786	*ARNT2*	9915	aryl hydrocarbon receptor nuclear translocator 2 [Source:HGNC Symbol;Acc:HGNC:16876]	-4.6	-2
6787	*TRIM39*	56658	tripartite motif containing 39 [Source:HGNC Symbol;Acc:HGNC:10065]	-4.6	-2
6788	*HPGD*	3248	hydroxyprostaglandin dehydrogenase 15-(NAD) [Source:HGNC Symbol;Acc:HGNC:5154]	-4.6	-2
6789	*CCND2*	894	cyclin D2 [Source:HGNC Symbol;Acc:HGNC:1583]	-4.6	-2
6790	*CYP20A1*	57404	cytochrome P450 family 20 subfamily A member 1 [Source:HGNC Symbol;Acc:HGNC:20576]	-4.6	-2
6791	*PDDC1*	347862	Parkinson disease 7 domain containing 1 [Source:HGNC Symbol;Acc:HGNC:26616]	-4.6	-2
6792	*MYEF2*	50804	myelin expression factor 2 [Source:HGNC Symbol;Acc:HGNC:17940]	-4.6	-2
6793	*SLC24A5*	283652	solute carrier family 24 member 5 [Source:HGNC Symbol;Acc:HGNC:20611]	-4.6	-2
6794	*MTX3*	345778	metaxin 3 [Source:HGNC Symbol;Acc:HGNC:24812]	-4.6	-2
6795	*NIT2*	56954	nitrilase family member 2 [Source:HGNC Symbol;Acc:HGNC:29878]	-4.6	-2
6796	*COQ10B*	80219	coenzyme Q10B [Source:HGNC Symbol;Acc:HGNC:25819]	-4.6	-2
6797	*GTF2F2*	2963	general transcription factor IIF subunit 2 [Source:HGNC Symbol;Acc:HGNC:4653]	-4.6	-2
6798	*PIK3C2B*	5287	phosphatidylinositol-4-phosphate 3-kinase catalytic subunit type 2 beta [Source:HGNC Symbol;Acc:HGNC:8972]	-4.6	-2
6799	*SBNO1*	55206	strawberry notch homolog 1 (Drosophila) [Source:HGNC Symbol;Acc:HGNC:22973]	-4.6	-2
6800	*TMEM170A*	124491	transmembrane protein 170A [Source:HGNC Symbol;Acc:HGNC:29577]	-4.6	-2
6801	*FXR2*	9513	FMR1 autosomal homolog 2 [Source:HGNC Symbol;Acc:HGNC:4024]	-4.6	-2
6802	*ARMCX5*	64860	armadillo repeat containing, X-linked 5 [Source:HGNC Symbol;Acc:HGNC:25772]	-4.6	-2
6803	*GPRASP2*	114928	G protein-coupled receptor associated sorting protein 2 [Source:HGNC Symbol;Acc:HGNC:25169]	-4.6	-2
6804	*XIRP2*	129446	xin actin binding repeat containing 2 [Source:HGNC Symbol;Acc:HGNC:14303]	-4.6	-2
6805	*CLASP1*	23332	cytoplasmic linker associated protein 1 [Source:HGNC Symbol;Acc:HGNC:17088]	-4.6	-2
6806	*ZNF587*	84914	zinc finger protein 587 [Source:HGNC Symbol;Acc:HGNC:30955]	-4.6	-2
6807	*HAUS8*	93323	HAUS augmin like complex subunit 8 [Source:HGNC Symbol;Acc:HGNC:30532]	-4.6	-2
6808	*DOPEY2*	9980	dopey family member 2 [Source:HGNC Symbol;Acc:HGNC:1291]	-4.6	-2
6809	*SPATA33*	124045	spermatogenesis associated 33 [Source:HGNC Symbol;Acc:HGNC:26463]	-4.6	-2
6810	*SLC25A45*	283130	solute carrier family 25 member 45 [Source:HGNC Symbol;Acc:HGNC:27442]	-4.6	-2
6811	*EXOC6*	54536	exocyst complex component 6 [Source:HGNC Symbol;Acc:HGNC:23196]	-4.6	-2
6812	*NDUFA5*	4698	NADH:ubiquinone oxidoreductase subunit A5 [Source:HGNC Symbol;Acc:HGNC:7688]	-4.6	-2
6813	*TCF19*	6941	transcription factor 19 [Source:HGNC Symbol;Acc:HGNC:11629]	-4.6	-2
6814	*DTD2*	112487	D-tyrosyl-tRNA deacylase 2 (putative) [Source:HGNC Symbol;Acc:HGNC:20277]	-4.6	-2
6815	*TRIM72*	493829	tripartite motif containing 72 [Source:HGNC Symbol;Acc:HGNC:32671]	-4.6	-2
6816	*LHFPL5*	222662	lipoma HMGIC fusion partner-like 5 [Source:HGNC Symbol;Acc:HGNC:21253]	-4.6	-2

6817	*C10orf67*	256815	chromosome 10 open reading frame 67 [Source:HGNC Symbol;Acc:HGNC:28716]	-4.6	-2
6818	*NPTXR*	23467	neuronal pentraxin receptor [Source:HGNC Symbol;Acc:HGNC:7954]	-4.6	-2
6819	*CDCA7*	83879	cell division cycle associated 7 [Source:HGNC Symbol;Acc:HGNC:14628]	-4.6	-2
6820	*LUZP2*	338645	leucine zipper protein 2 [Source:HGNC Symbol;Acc:HGNC:23206]	-4.6	-2
6821	*SLC6A11*	6538	solute carrier family 6 member 11 [Source:HGNC Symbol;Acc:HGNC:11044]	-4.6	-2
6822	*SLC4A4*	8671	solute carrier family 4 member 4 [Source:HGNC Symbol;Acc:HGNC:11030]	-4.6	-2
6823	*ZNF33B*	7582	zinc finger protein 33B [Source:HGNC Symbol;Acc:HGNC:13097]	-4.6	-2
6824	*TMEM184C*	55751	transmembrane protein 184C [Source:HGNC Symbol;Acc:HGNC:25587]	-4.7	-2
6825	*ZNF213*	7760	zinc finger protein 213 [Source:HGNC Symbol;Acc:HGNC:13005]	-4.7	-2
6826	*FGF5*	2250	fibroblast growth factor 5 [Source:HGNC Symbol;Acc:HGNC:3683]	-4.7	-2
6827	*PEX2*	5828	peroxisomal biogenesis factor 2 [Source:HGNC Symbol;Acc:HGNC:9717]	-4.7	-2
6828	*FKBP9*	11328	FK506 binding protein 9 [Source:HGNC Symbol;Acc:HGNC:3725]	-4.7	-2
6829	*FFAR4*	338557	free fatty acid receptor 4 [Source:HGNC Symbol;Acc:HGNC:19061]	-4.7	-2
6830	*ZNF397*	84307	zinc finger protein 397 [Source:HGNC Symbol;Acc:HGNC:18818]	-4.7	-2
6831	*RAB43*	339122	RAB43, member RAS oncogene family [Source:HGNC Symbol;Acc:HGNC:19983]	-4.7	-2
6832	*INCA1*	388324	inhibitor of CDK, cyclin A1 interacting protein 1 [Source:HGNC Symbol;Acc:HGNC:32224]	-4.7	-2
6833	*C1orf116*	79098	chromosome 1 open reading frame 116 [Source:HGNC Symbol;Acc:HGNC:28667]	-4.7	-2
6834	*TAMM41*	132001	TAM41 mitochondrial translocator assembly and maintenance homolog [Source:HGNC Symbol;Acc:HGNC:25187]	-4.7	-2
6835	*ZNF785*	146540	zinc finger protein 785 [Source:HGNC Symbol;Acc:HGNC:26496]	-4.7	-2
6836	*CCDC120*	90060	coiled-coil domain containing 120 [Source:HGNC Symbol;Acc:HGNC:28910]	-4.7	-2
6837	*PPIL4*	85313	peptidylprolyl isomerase like 4 [Source:HGNC Symbol;Acc:HGNC:15702]	-4.7	-2
6838	*GSDMC*	56169	gasdermin C [Source:HGNC Symbol;Acc:HGNC:7151]	-4.7	-2
6839	*FAM65C*	140876	family with sequence similarity 65 member C [Source:HGNC Symbol;Acc:HGNC:16168]	-4.7	-2
6840	*MRVI1*	10335	murine retrovirus integration site 1 homolog [Source:HGNC Symbol;Acc:HGNC:7237]	-4.7	-2
6841	*TBCCD1*	55171	TBCC domain containing 1 [Source:HGNC Symbol;Acc:HGNC:25546]	-4.7	-2
6842	*KATNAL2*	83473	katanin catalytic subunit A1 like 2 [Source:HGNC Symbol;Acc:HGNC:25387]	-4.7	-2
6843	*MILR1*	284021	mast cell immunoglobulin like receptor 1 [Source:HGNC Symbol;Acc:HGNC:27570]	-4.7	-2
6844	*FOXL2NB*	401089	FOXL2 neighbor [Source:HGNC Symbol;Acc:HGNC:34428]	-4.7	-2
6845	*MCM6*	4175	minichromosome maintenance complex component 6 [Source:HGNC Symbol;Acc:HGNC:6949]	-4.7	-2
6846	*ST6GALNAC3*	256435	ST6 N-acetylgalactosaminide alpha-2,6-sialyltransferase 3 [Source:HGNC Symbol;Acc:HGNC:19343]	-4.7	-2
6847	*BVES*	11149	blood vessel epicardial substance [Source:HGNC Symbol;Acc:HGNC:1152]	-4.7	-2
6848	*FUT1*	2523	fucosyltransferase 1 (H blood group) [Source:HGNC Symbol;Acc:HGNC:4012]	-4.7	-2
6849	*ASB11*	140456	ankyrin repeat and SOCS box containing 11 [Source:HGNC Symbol;Acc:HGNC:17186]	-4.7	-2
6850	*CRYZL1*	9946	crystallin zeta like 1 [Source:HGNC Symbol;Acc:HGNC:2420]	-4.7	-2
6851	*NME9*	347736	NME/NM23 family member 9 [Source:HGNC Symbol;Acc:HGNC:21343]	-4.7	-2
6852	*DISC1*	27185	disrupted in schizophrenia 1 [Source:HGNC Symbol;Acc:HGNC:2888]	-4.7	-2
6853	*MIER2*	54531	MIER family member 2 [Source:HGNC Symbol;Acc:HGNC:29210]	-4.7	-2
6854	*SEMA5A*	9037	semaphorin 5A [Source:HGNC Symbol;Acc:HGNC:10736]	-4.7	-2
6855	*ULBP3*	79465	UL16 binding protein 3 [Source:HGNC Symbol;Acc:HGNC:14895]	-4.7	-2
6856	*ZNF250*	58500	zinc finger protein 250 [Source:HGNC Symbol;Acc:HGNC:13044]	-4.7	-2
6857	*RSF1*	51773	remodeling and spacing factor 1 [Source:HGNC Symbol;Acc:HGNC:18118]	-4.7	-2
6858	*GBAS*	2631	glioblastoma amplified sequence [Source:HGNC Symbol;Acc:HGNC:4179]	-4.7	-2
6859	*CCDC141*	285025	coiled-coil domain containing 141 [Source:HGNC Symbol;Acc:HGNC:26821]	-4.7	-2
6860	*PDE6A*	5145	phosphodiesterase 6A [Source:HGNC Symbol;Acc:HGNC:8785]	-4.7	-2
6861	*KIAA1456*	57604	KIAA1456 [Source:HGNC Symbol;Acc:HGNC:26725]	-4.7	-2
6862	*PHYHD1*	254295	phytanoyl-CoA dioxygenase domain containing 1 [Source:HGNC Symbol;Acc:HGNC:23396]	-4.7	-2
6863	*AREL1*	9870	apoptosis resistant E3 ubiquitin protein ligase 1 [Source:HGNC Symbol;Acc:HGNC:20363]	-4.7	-2
6864	*SCAI*	286205	suppressor of cancer cell invasion [Source:HGNC Symbol;Acc:HGNC:26709]	-4.7	-2
6865	*PLCE1*	51196	phospholipase C epsilon 1 [Source:HGNC Symbol;Acc:HGNC:17175]	-4.7	-2
6866	*UNC80*	285175	unc-80 homolog, NALCN activator [Source:HGNC Symbol;Acc:HGNC:26582]	-4.7	-2
6867	*NAGLU*	4669	N-acetyl-alpha-glucosaminidase [Source:HGNC Symbol;Acc:HGNC:7632]	-4.7	-2
6868	*EXO5*	64789	exonuclease 5 [Source:HGNC Symbol;Acc:HGNC:26115]	-4.7	-2
6869	*AGMAT*	79814	agmatinase [Source:HGNC Symbol;Acc:HGNC:18407]	-4.7	-2
6870	*SFMBT2*	57713	Scm-like with four mbt domains 2 [Source:HGNC Symbol;Acc:HGNC:20256]	-4.8	-2
6871	*CSTL1*	128817	cystatin like 1 [Source:HGNC Symbol;Acc:HGNC:15958]	-4.8	-2
6872	*CRYBA2*	1412	crystallin beta A2 [Source:HGNC Symbol;Acc:HGNC:2395]	-4.8	-2
6873	*ZNF99*	7652	zinc finger protein 99 [Source:HGNC Symbol;Acc:HGNC:13175]	-4.8	-2
6874	*VIPR1*	7433	vasoactive intestinal peptide receptor 1 [Source:HGNC Symbol;Acc:HGNC:12694]	-4.8	-2
6875	*ZNF490*	57474	zinc finger protein 490 [Source:HGNC Symbol;Acc:HGNC:23705]	-4.8	-2
6876	*KLK4*	9622	kallikrein related peptidase 4 [Source:HGNC Symbol;Acc:HGNC:6365]	-4.8	-2
6877	*UGGT1*	56886	UDP-glucose glycoprotein glucosyltransferase 1 [Source:HGNC Symbol;Acc:HGNC:15663]	-4.8	-2
6878	*C4BPB*	725	complement component 4 binding protein beta [Source:HGNC Symbol;Acc:HGNC:1328]	-4.8	-2
6879	*INO80B*	83444	INO80 complex subunit B [Source:HGNC Symbol;Acc:HGNC:13324]	-4.8	-2
6880	*GINS1*	9837	GINS complex subunit 1 [Source:HGNC Symbol;Acc:HGNC:28980]	-4.8	-2
6881	*AKR1E2*	83592	aldo-keto reductase family 1 member E2 [Source:HGNC Symbol;Acc:HGNC:23437]	-4.8	-2
6882	*FAM83D*	81610	family with sequence similarity 83 member D [Source:HGNC Symbol;Acc:HGNC:16122]	-4.8	-2
6883	*HIP1*	3092	huntingtin interacting protein 1 [Source:HGNC Symbol;Acc:HGNC:4913]	-4.8	-2
6884	*SUSD4*	55061	sushi domain containing 4 [Source:HGNC Symbol;Acc:HGNC:25470]	-4.8	-2
6885	*MEMO1*	51072	mediator of cell motility 1 [Source:HGNC Symbol;Acc:HGNC:14014]	-4.8	-2
6886	*SCYL2*	55681	SCY1 like pseudokinase 2 [Source:HGNC Symbol;Acc:HGNC:19286]	-4.8	-2
6887	*PADI2*	11240	peptidyl arginine deiminase 2 [Source:HGNC Symbol;Acc:HGNC:18341]	-4.8	-2
6888	*NMT2*	9397	N-myristoyltransferase 2 [Source:HGNC Symbol;Acc:HGNC:7858]	-4.8	-2
6889	*ISPD*	729920	isoprenoid synthase domain containing [Source:HGNC Symbol;Acc:HGNC:37276]	-4.8	-2
6890	*ZNF529*	57711	zinc finger protein 529 [Source:HGNC Symbol;Acc:HGNC:29328]	-4.8	-2
6891	*ARFIP2*	23647	ADP ribosylation factor interacting protein 2 [Source:HGNC Symbol;Acc:HGNC:17160]	-4.8	-2
6892	*NUAK2*	81788	NUAK family kinase 2 [Source:HGNC Symbol;Acc:HGNC:29558]	-4.8	-2
6893	*IL23R*	149233	interleukin 23 receptor [Source:HGNC Symbol;Acc:HGNC:19100]	-4.8	-2
6894	*ZFAND4*	93550	zinc finger AN1-type containing 4 [Source:HGNC Symbol;Acc:HGNC:23504]	-4.8	-2

6895	*ZNF7*	7553	zinc finger protein 7 [Source:HGNC Symbol;Acc:HGNC:13139]	-4.8	-2
6896	*SYMPK*	8189	symplekin [Source:HGNC Symbol;Acc:HGNC:22935]	-4.8	-2
6897	*CLUAP1*	23059	clusterin associated protein 1 [Source:HGNC Symbol;Acc:HGNC:19009]	-4.8	-2
6898	*NRIP3*	56675	nuclear receptor interacting protein 3 [Source:HGNC Symbol;Acc:HGNC:1167]	-4.8	-2
6899	*DUSP28*	285193	dual specificity phosphatase 28 [Source:HGNC Symbol;Acc:HGNC:33237]	-4.8	-2
6900	*GPR12*	2835	G protein-coupled receptor 12 [Source:HGNC Symbol;Acc:HGNC:4466]	-4.8	-2
6901	*PRR23C*	389152	proline rich 23C [Source:HGNC Symbol;Acc:HGNC:37173]	-4.8	-2
6902	*C15orf48*	84419	chromosome 15 open reading frame 48 [Source:HGNC Symbol;Acc:HGNC:29898]	-4.8	-2
6903	*SOD3*	6649	superoxide dismutase 3, extracellular [Source:HGNC Symbol;Acc:HGNC:11181]	-4.8	-2
6904	*QTRTD1*	79691	Queuine TRNA-Ribosyltransferase Accessory Subunit 2	-4.8	-2
6905	*PYCRL*	65263	pyrroline-5-carboxylate reductase-like [Source:HGNC Symbol;Acc:HGNC:25846]	-4.8	-2
6906	*TNFRSF13B*	23495	tumor necrosis factor receptor superfamily member 13B [Source:HGNC Symbol;Acc:HGNC:18153]	-4.8	-2
6907	*MOCS3*	27304	molybdenum cofactor synthesis 3 [Source:HGNC Symbol;Acc:HGNC:15765]	-4.8	-2
6908	*TTC39C*	125488	tetratricopeptide repeat domain 39C [Source:HGNC Symbol;Acc:HGNC:26595]	-4.8	-2
6909	*VGF*	7425	VGF nerve growth factor inducible [Source:HGNC Symbol;Acc:HGNC:12684]	-4.8	-2
6910	*TAT*	6898	tyrosine aminotransferase [Source:HGNC Symbol;Acc:HGNC:11573]	-4.8	-2
6911	*ZNF264*	9422	zinc finger protein 264 [Source:HGNC Symbol;Acc:HGNC:13057]	-4.8	-2
6912	*SOWAHC*	65124	sosondowah ankyrin repeat domain family member C [Source:HGNC Symbol;Acc:HGNC:26149]	-4.8	-2
6913	*KIAA0930*	23313	KIAA0930 [Source:HGNC Symbol;Acc:HGNC:1314]	-4.8	-2
6914	*POMGNT1*	55624	protein O-linked mannose N-acetylglucosaminyltransferase 1 (beta 1,2-) [Source:HGNC Symbol;Acc:HGNC:19139]	-4.8	-2
6915	*RRP7A*	27341	ribosomal RNA processing 7 homolog A [Source:HGNC Symbol;Acc:HGNC:24286]	-4.8	-2
6916	*UBA5*	79876	ubiquitin like modifier activating enzyme 5 [Source:HGNC Symbol;Acc:HGNC:23230]	-4.8	-2
6917	*WRNIP1*	56897	Werner helicase interacting protein 1 [Source:HGNC Symbol;Acc:HGNC:20876]	-4.8	-2
6918	*PPFIBP1*	8496	PPFIA binding protein 1 [Source:HGNC Symbol;Acc:HGNC:9249]	-4.8	-2
6919	*FRMD4A*	55691	FERM domain containing 4A [Source:HGNC Symbol;Acc:HGNC:25491]	-4.8	-2
6920	*DCAF8*	50717	DDB1 and CUL4 associated factor 8 [Source:HGNC Symbol;Acc:HGNC:24891]	-4.8	-2
6921	*ZNF506*	440515	zinc finger protein 506 [Source:HGNC Symbol;Acc:HGNC:23780]	-4.8	-2
6922	*ZNF492*	57615	zinc finger protein 492 [Source:HGNC Symbol;Acc:HGNC:23707]	-4.9	-2
6923	*ZFY*	7544	zinc finger protein, Y-linked [Source:HGNC Symbol;Acc:HGNC:12870]	-4.9	-2
6924	*CYP4B1*	1580	cytochrome P450 family 4 subfamily B member 1 [Source:HGNC Symbol;Acc:HGNC:2644]	-4.9	-2
6925	*UBOX5*	22888	U-box domain containing 5 [Source:HGNC Symbol;Acc:HGNC:17777]	-4.9	-2
6926	*MORC4*	79710	MORC family CW-type zinc finger 4 [Source:HGNC Symbol;Acc:HGNC:23485]	-4.9	-2
6927	*SLC40A1*	30061	solute carrier family 40 member 1 [Source:HGNC Symbol;Acc:HGNC:10909]	-4.9	-2
6928	*MMS22L*	253714	MMS22 like, DNA repair protein [Source:HGNC Symbol;Acc:HGNC:21475]	-4.9	-2
6929	*PXMP4*	11264	peroxisomal membrane protein 4 [Source:HGNC Symbol;Acc:HGNC:15920]	-4.9	-2
6930	*DPP7*	29952	dipeptidyl peptidase 7 [Source:HGNC Symbol;Acc:HGNC:14892]	-4.9	-2
6931	*DNAH11*	8701	dynein axonemal heavy chain 11 [Source:HGNC Symbol;Acc:HGNC:2942]	-4.9	-2
6932	*NLRC3*	197358	NLR family CARD domain containing 3 [Source:HGNC Symbol;Acc:HGNC:29889]	-4.9	-2
6933	*ECHDC3*	79746	enoyl-CoA hydratase domain containing 3 [Source:HGNC Symbol;Acc:HGNC:23489]	-4.9	-2
6934	*NAA38*	84316	N(alpha)-acetyltransferase 38, NatC auxiliary subunit [Source:HGNC Symbol;Acc:HGNC:28212]	-4.9	-2
6935	*PIK3C3*	5289	phosphatidylinositol 3-kinase catalytic subunit type 3 [Source:HGNC Symbol;Acc:HGNC:8974]	-4.9	-2
6936	*TBPL2*	387332	TATA-box binding protein like 2 [Source:HGNC Symbol;Acc:HGNC:19841]	-4.9	-2
6937	*ORC3*	23595	origin recognition complex subunit 3 [Source:HGNC Symbol;Acc:HGNC:8489]	-4.9	-2
6938	*C7*	730	complement component 7 [Source:HGNC Symbol;Acc:HGNC:1346]	-4.9	-2
6939	*KANSL1L*	151050	KAT8 regulatory NSL complex subunit 1 like [Source:HGNC Symbol;Acc:HGNC:26310]	-4.9	-2
6940	*CLN6*	54982	ceroid-lipofuscinosis, neuronal 6, late infantile, variant [Source:HGNC Symbol;Acc:HGNC:2077]	-4.9	-2
6941	*SLC39A8*	64116	solute carrier family 39 member 8 [Source:HGNC Symbol;Acc:HGNC:20862]	-4.9	-2
6942	*FAM73A*	374986	family with sequence similarity 73 member A [Source:HGNC Symbol;Acc:HGNC:24741]	-4.9	-2
6943	*ZNF846*	162993	zinc finger protein 846 [Source:HGNC Symbol;Acc:HGNC:27260]	-4.9	-2
6944	*CXADR*	1525	coxsackie virus and adenovirus receptor [Source:HGNC Symbol;Acc:HGNC:2559]	-4.9	-2
6945	*ADM2*	79924	adrenomedullin 2 [Source:HGNC Symbol;Acc:HGNC:28898]	-4.9	-2
6946	*SLC16A12*	387700	solute carrier family 16 member 12 [Source:HGNC Symbol;Acc:HGNC:23094]	-4.9	-2
6947	*GSN*	2934	gelsolin [Source:HGNC Symbol;Acc:HGNC:4620]	-4.9	-2
6948	*NOTCH4*	4855	notch 4 [Source:HGNC Symbol;Acc:HGNC:7884]	-4.9	-2
6949	*SPTLC1*	10558	serine palmitoyltransferase long chain base subunit 1 [Source:HGNC Symbol;Acc:HGNC:11277]	-4.9	-2
6950	*SH3RF2*	153769	SH3 domain containing ring finger 2 [Source:HGNC Symbol;Acc:HGNC:26299]	-4.9	-2
6951	*ITGA10*	8515	integrin subunit alpha 10 [Source:HGNC Symbol;Acc:HGNC:6135]	-4.9	-2
6952	*SDE2*	163859	SDE2 telomere maintenance homolog [Source:HGNC Symbol;Acc:HGNC:26643]	-4.9	-2
6953	*FAM153A*	285596	family with sequence similarity 153 member A [Source:HGNC Symbol;Acc:HGNC:29940]	-4.9	-2
6954	*XPR1*	9213	xenotropic and polytropic retrovirus receptor 1 [Source:HGNC Symbol;Acc:HGNC:12827]	-4.9	-2
6955	*NDRG2*	57447	NDRG family member 2 [Source:HGNC Symbol;Acc:HGNC:14460]	-4.9	-2
6956	*PTK7*	5754	protein tyrosine kinase 7 (inactive) [Source:HGNC Symbol;Acc:HGNC:9618]	-4.9	-2
6957	*TMEM69*	51249	transmembrane protein 69 [Source:HGNC Symbol;Acc:HGNC:28035]	-4.9	-2
6958	*STRA6*	64220	stimulated by retinoic acid 6 [Source:HGNC Symbol;Acc:HGNC:30650]	-4.9	-2
6959	*LYPLAL1*	127018	lysophospholipase like 1 [Source:HGNC Symbol;Acc:HGNC:20440]	-4.9	-2
6960	*STYX*	6815	serine/threonine/tyrosine interacting protein [Source:HGNC Symbol;Acc:HGNC:11447]	-4.9	-2
6961	*IL10*	3586	interleukin 10 [Source:HGNC Symbol;Acc:HGNC:5962]	-4.9	-2
6962	*QRSL1*	55278	glutaminyl-tRNA synthase (glutamine-hydrolyzing)-like 1 [Source:HGNC Symbol;Acc:HGNC:21020]	-4.9	-2
6963	*CPA4*	51200	carboxypeptidase A4 [Source:HGNC Symbol;Acc:HGNC:15740]	-4.9	-2
6964	*OAF*	220323	out at first homolog [Source:HGNC Symbol;Acc:HGNC:28752]	-4.9	-2
6965	*FAM206A*	54942	family with sequence similarity 206 member A [Source:HGNC Symbol;Acc:HGNC:1364]	-4.9	-2
6966	*UBE2W*	55284	ubiquitin conjugating enzyme E2 W (putative) [Source:HGNC Symbol;Acc:HGNC:25616]	-4.9	-2
6967	*AMOTL1*	154810	angiomotin like 1 [Source:HGNC Symbol;Acc:HGNC:17811]	-4.9	-2
6968	*PBOV1*	59351	prostate and breast cancer overexpressed 1 [Source:HGNC Symbol;Acc:HGNC:21079]	-4.9	-2
6969	*CPT1A*	1374	carnitine palmitoyltransferase 1A [Source:HGNC Symbol;Acc:HGNC:2328]	-4.9	-2

6970	*RRAS*	6237	related RAS viral (r-ras) oncogene homolog [Source:HGNC Symbol;Acc:HGNC:10447]	-4.9	-2
6971	*C19orf18*	147685	chromosome 19 open reading frame 18 [Source:HGNC Symbol;Acc:HGNC:28642]	-4.9	-2
6972	*ACAA2*	10449	acetyl-CoA acyltransferase 2 [Source:HGNC Symbol;Acc:HGNC:83]	-4.9	-2
6973	*CCT6B*	10693	chaperonin containing TCP1 subunit 6B [Source:HGNC Symbol;Acc:HGNC:1621]	-4.9	-2
6974	*SP2*	6668	Sp2 transcription factor [Source:HGNC Symbol;Acc:HGNC:11207]	-4.9	-2
6975	*RTCA*	8634	RNA 3'-terminal phosphate cyclase [Source:HGNC Symbol;Acc:HGNC:17981]	-4.9	-2
6976	*MEIS3*	56917	Meis homeobox 3 [Source:HGNC Symbol;Acc:HGNC:29537]	-4.9	-2
6977	*SGK3*	23678	serum/glucocorticoid regulated kinase family member 3 [Source:HGNC Symbol;Acc:HGNC:10812]	-4.9	-2
6978	*BMPR1B*	658	bone morphogenetic protein receptor type 1B [Source:HGNC Symbol;Acc:HGNC:1077]	-4.9	-2
6979	*DTNB*	1838	dystrobrevin beta [Source:HGNC Symbol;Acc:HGNC:3058]	-4.9	-2
6980	*RPE*	6120	ribulose-5-phosphate-3-epimerase [Source:HGNC Symbol;Acc:HGNC:10293]	-5.0	-2
6981	*ZNF19*	7567	zinc finger protein 19 [Source:HGNC Symbol;Acc:HGNC:12981]	-5.0	-2
6982	*BHMT2*	23743	betaine--homocysteine S-methyltransferase 2 [Source:HGNC Symbol;Acc:HGNC:1048]	-5.0	-2
6983	*BAG5*	9529	BCL2 associated athanogene 5 [Source:HGNC Symbol;Acc:HGNC:941]	-5.0	-2
6984	*ZNF112*	7771	zinc finger protein 112 [Source:HGNC Symbol;Acc:HGNC:12892]	-5.0	-2
6985	*STX6*	10228	syntaxin 6 [Source:HGNC Symbol;Acc:HGNC:11441]	-5.0	-2
6986	*CLCC1*	23155	chloride channel CLIC like 1 [Source:HGNC Symbol;Acc:HGNC:29675]	-5.0	-2
6987	*SFMBT1*	51460	Scm-like with four mbt domains 1 [Source:HGNC Symbol;Acc:HGNC:20255]	-5.0	-2
6988	*FLT1*	2321	fms related tyrosine kinase 1 [Source:HGNC Symbol;Acc:HGNC:3763]	-5.0	-2
6989	*NT5DC2*	64943	5'-nucleotidase domain containing 2 [Source:HGNC Symbol;Acc:HGNC:25717]	-5.0	-2
6990	*TMEM266*	123591	transmembrane protein 266 [Source:HGNC Symbol;Acc:HGNC:26763]	-5.0	-2
6991	*SHROOM1*	134549	shroom family member 1 [Source:HGNC Symbol;Acc:HGNC:24084]	-5.0	-2
6992	*NOTCH2*	4853	notch 2 [Source:HGNC Symbol;Acc:HGNC:7882]	-5.0	-2
6993	*CORIN*	10699	corin, serine peptidase [Source:HGNC Symbol;Acc:HGNC:19012]	-5.0	-2
6994	*GJB5*	2709	gap junction protein beta 5 [Source:HGNC Symbol;Acc:HGNC:4287]	-5.0	-2
6995	*BCAS1*	8537	breast carcinoma amplified sequence 1 [Source:HGNC Symbol;Acc:HGNC:974]	-5.0	-2
6996	*GPSM2*	29899	G-protein signaling modulator 2 [Source:HGNC Symbol;Acc:HGNC:29501]	-5.0	-2
6997	*NWD1*	284434	NACHT and WD repeat domain containing 1 [Source:HGNC Symbol;Acc:HGNC:27619]	-5.0	-2
6998	*SPARCL1*	8404	SPARC like 1 [Source:HGNC Symbol;Acc:HGNC:11220]	-5.0	-2
6999	*RNF43*	54894	ring finger protein 43 [Source:HGNC Symbol;Acc:HGNC:18505]	-5.0	-2
7000	*C19orf35*	374872	chromosome 19 open reading frame 35 [Source:HGNC Symbol;Acc:HGNC:24793]	-5.0	-2
7001	*SLC25A53*	401612	solute carrier family 25 member 53 [Source:HGNC Symbol;Acc:HGNC:31894]	-5.0	-2
7002	*BPIFB2*	80341	BPI fold containing family B member 2 [Source:HGNC Symbol;Acc:HGNC:16177]	-5.0	-2
7003	*HTRA4*	203100	HtrA serine peptidase 4 [Source:HGNC Symbol;Acc:HGNC:26909]	-5.0	-2
7004	*DOCK5*	80005	dedicator of cytokinesis 5 [Source:HGNC Symbol;Acc:HGNC:23476]	-5.0	-2
7005	*ZDHHC15*	158866	zinc finger DHHC-type containing 15 [Source:HGNC Symbol;Acc:HGNC:20342]	-5.0	-2
7006	*HAUS3*	79441	HAUS augmin like complex subunit 3 [Source:HGNC Symbol;Acc:HGNC:28719]	-5.0	-2
7007	*ZAK*	51776	Sterile Alpha Motif And Leucine Zipper Containing Kinase AZK	-5.0	-2
7008	*GRTP1*	79774	growth hormone regulated TBC protein 1 [Source:HGNC Symbol;Acc:HGNC:20310]	-5.0	-2
7009	*TMEM209*	84928	transmembrane protein 209 [Source:HGNC Symbol;Acc:HGNC:21898]	-5.0	-2
7010	*PANK1*	53354	pantothenate kinase 1 [Source:HGNC Symbol;Acc:HGNC:8598]	-5.0	-2
7011	*METTL2A*	339175	methyltransferase like 2A [Source:HGNC Symbol;Acc:HGNC:25755]	-5.0	-2
7012	*SCD5*	79966	stearoyl-CoA desaturase 5 [Source:HGNC Symbol;Acc:HGNC:21088]	-5.0	-2
7013	*RALGAPA1*	253959	Ral GTPase activating protein catalytic alpha subunit 1 [Source:HGNC Symbol;Acc:HGNC:17770]	-5.0	-2
7014	*FBXO24*	26261	F-box protein 24 [Source:HGNC Symbol;Acc:HGNC:13595]	-5.0	-2
7015	*UNC13D*	201294	unc-13 homolog D [Source:HGNC Symbol;Acc:HGNC:23147]	-5.0	-2
7016	*LY6D*	8581	lymphocyte antigen 6 complex, locus D [Source:HGNC Symbol;Acc:HGNC:13348]	-5.0	-2
7017	*ZNF302*	55900	zinc finger protein 302 [Source:HGNC Symbol;Acc:HGNC:13848]	-5.0	-2
7018	*PTPN13*	5783	protein tyrosine phosphatase, non-receptor type 13 [Source:HGNC Symbol;Acc:HGNC:9646]	-5.0	-2
7019	*KLRD1*	3824	killer cell lectin like receptor D1 [Source:HGNC Symbol;Acc:HGNC:6378]	-5.0	-2
7020	*SHROOM4*	57477	shroom family member 4 [Source:HGNC Symbol;Acc:HGNC:29215]	-5.0	-2
7021	*VENTX*	27287	VENT homeobox [Source:HGNC Symbol;Acc:HGNC:13639]	-5.0	-2
7022	*MED6*	10001	mediator complex subunit 6 [Source:HGNC Symbol;Acc:HGNC:19970]	-5.0	-2
7023	*NAB1*	4664	NGFI-A binding protein 1 [Source:HGNC Symbol;Acc:HGNC:7626]	-5.0	-2
7024	*ATXN3*	4287	ataxin 3 [Source:HGNC Symbol;Acc:HGNC:7106]	-5.0	-2
7025	*ZSWIM8*	23053	zinc finger SWIM-type containing 8 [Source:HGNC Symbol;Acc:HGNC:23528]	-5.0	-2
7026	*KIF3A*	11127	kinesin family member 3A [Source:HGNC Symbol;Acc:HGNC:6319]	-5.0	-2
7027	*TEP1*	7011	telomerase associated protein 1 [Source:HGNC Symbol;Acc:HGNC:11726]	-5.1	-2
7028	*CDCA7L*	55536	cell division cycle associated 7 like [Source:HGNC Symbol;Acc:HGNC:30777]	-5.1	-2
7029	*CCDC125*	202243	coiled-coil domain containing 125 [Source:HGNC Symbol;Acc:HGNC:28924]	-5.1	-2
7030	*RBP1*	5947	retinol binding protein 1 [Source:HGNC Symbol;Acc:HGNC:9919]	-5.1	-2
7031	*SPIRE2*	84501	spire type actin nucleation factor 2 [Source:HGNC Symbol;Acc:HGNC:30623]	-5.1	-2
7032	*CCL22*	6367	C-C motif chemokine ligand 22 [Source:HGNC Symbol;Acc:HGNC:10621]	-5.1	-2
7033	*MTBP*	27085	MDM2 binding protein [Source:HGNC Symbol;Acc:HGNC:7417]	-5.1	-2
7034	*CXCL16*	58191	C-X-C motif chemokine ligand 16 [Source:HGNC Symbol;Acc:HGNC:16642]	-5.1	-2
7035	*SBF2*	81846	SET binding factor 2 [Source:HGNC Symbol;Acc:HGNC:2135]	-5.1	-2
7036	*AAMDC*	28971	adipogenesis associated, Mth938 domain containing [Source:HGNC Symbol;Acc:HGNC:30205]	-5.1	-2
7037	*WBP1*	23559	WW domain binding protein 1 [Source:HGNC Symbol;Acc:HGNC:12737]	-5.1	-2
7038	*SGSM1*	129049	small G protein signaling modulator 1 [Source:HGNC Symbol;Acc:HGNC:29410]	-5.1	-2
7039	*ZNF160*	90338	zinc finger protein 160 [Source:HGNC Symbol;Acc:HGNC:12948]	-5.1	-2
7040	*TMEM213*	155006	transmembrane protein 213 [Source:HGNC Symbol;Acc:HGNC:27220]	-5.1	-2
7041	*CHST5*	23563	carbohydrate sulfotransferase 5 [Source:HGNC Symbol;Acc:HGNC:1973]	-5.1	-2
7042	*ZNF114*	163071	zinc finger protein 114 [Source:HGNC Symbol;Acc:HGNC:12894]	-5.1	-2
7043	*C9orf85*	138241	chromosome 9 open reading frame 85 [Source:HGNC Symbol;Acc:HGNC:28784]	-5.1	-2
7044	*GPR82*	27197	G protein-coupled receptor 82 [Source:HGNC Symbol;Acc:HGNC:4533]	-5.1	-2
7045	*DYDC1*	143241	DPY30 domain containing 1 [Source:HGNC Symbol;Acc:HGNC:23460]	-5.1	-2
7046	*ZNF429*	353088	zinc finger protein 429 [Source:HGNC Symbol;Acc:HGNC:20817]	-5.1	-2
7047	*C17orf75*	64149	chromosome 17 open reading frame 75 [Source:HGNC Symbol;Acc:HGNC:30173]	-5.1	-2
7048	*NAPB*	63908	NSF attachment protein beta [Source:HGNC Symbol;Acc:HGNC:15751]	-5.1	-2
7049	*FXN*	2395	frataxin [Source:HGNC Symbol;Acc:HGNC:3951]	-5.1	-2

7050	*RPP21*	79897	ribonuclease P/MRP subunit p21 [Source:HGNC Symbol;Acc:HGNC:21300]	-5.1	-2
7051	*ATP1B2*	482	ATPase Na+/K+ transporting subunit beta 2 [Source:HGNC Symbol;Acc:HGNC:805]	-5.1	-2
7052	*ARIH2OS*	646450	ariadne homolog 2 opposite strand [Source:HGNC Symbol;Acc:HGNC:34425]	-5.1	-2
7053	*AP1S3*	130340	adaptor related protein complex 1 sigma 3 subunit [Source:HGNC Symbol;Acc:HGNC:18971]	-5.1	-2
7054	*TTLL3*	26140	tubulin tyrosine ligase like 3 [Source:HGNC Symbol;Acc:HGNC:24483]	-5.1	-2
7055	*SENP5*	205564	SUMO1/sentrin specific peptidase 5 [Source:HGNC Symbol;Acc:HGNC:28407]	-5.1	-2
7056	*GNG4*	2786	G protein subunit gamma 4 [Source:HGNC Symbol;Acc:HGNC:4407]	-5.1	-2
7057	*CEACAM8*	1088	carcinoembryonic antigen related cell adhesion molecule 8 [Source:HGNC Symbol;Acc:HGNC:1820]	-5.1	-2
7058	*DPY19L4*	286148	dpy-19 like 4 (C. elegans) [Source:HGNC Symbol;Acc:HGNC:27829]	-5.1	-2
7059	*BCL2L15*	440603	BCL2 like 15 [Source:HGNC Symbol;Acc:HGNC:33624]	-5.1	-2
7060	*SGSM2*	9905	small G protein signaling modulator 2 [Source:HGNC Symbol;Acc:HGNC:29026]	-5.1	-2
7061	*FCER1A*	2205	Fc fragment of IgE receptor Ia [Source:HGNC Symbol;Acc:HGNC:3609]	-5.1	-2
7062	*PKD1*	5310	polycystin 1, transient receptor potential channel interacting [Source:HGNC Symbol;Acc:HGNC:9008]	-5.1	-2
7063	*PEAK1*	79834	pseudopodium enriched atypical kinase 1 [Source:HGNC Symbol;Acc:HGNC:29431]	-5.1	-2
7064	*FCER2*	2208	Fc fragment of IgE receptor II [Source:HGNC Symbol;Acc:HGNC:3612]	-5.1	-2
7065	*APOLD1*	81575	apolipoprotein L domain containing 1 [Source:HGNC Symbol;Acc:HGNC:25268]	-5.1	-2
7066	*TERF2*	7014	telomeric repeat binding factor 2 [Source:HGNC Symbol;Acc:HGNC:11729]	-5.1	-2
7067	*TRPV1*	7442	transient receptor potential cation channel subfamily V member 1 [Source:HGNC Symbol;Acc:HGNC:12716]	-5.1	-2
7068	*SOX13*	9580	SRY-box 13 [Source:HGNC Symbol;Acc:HGNC:11192]	-5.1	-2
7069	*ZFP14*	57677	ZFP14 zinc finger protein [Source:HGNC Symbol;Acc:HGNC:29312]	-5.1	-2
7070	*KLF8*	11279	Kruppel like factor 8 [Source:HGNC Symbol;Acc:HGNC:6351]	-5.1	-2
7071	*KAT2A*	2648	lysine acetyltransferase 2A [Source:HGNC Symbol;Acc:HGNC:4201]	-5.1	-2
7072	*SMYD4*	114826	SET and MYND domain containing 4 [Source:HGNC Symbol;Acc:HGNC:21067]	-5.1	-2
7073	*TTC21B*	79809	tetratricopeptide repeat domain 21B [Source:HGNC Symbol;Acc:HGNC:25660]	-5.1	-2
7074	*ZNF527*	84503	zinc finger protein 527 [Source:HGNC Symbol;Acc:HGNC:29385]	-5.1	-2
7075	*CYP4F12*	66002	cytochrome P450 family 4 subfamily F member 12 [Source:HGNC Symbol;Acc:HGNC:18857]	-5.2	-2
7076	*PPEF2*	5470	protein phosphatase with EF-hand domain 2 [Source:HGNC Symbol;Acc:HGNC:9244]	-5.2	-2
7077	*GPATCH2L*	55668	G-patch domain containing 2 like [Source:HGNC Symbol;Acc:HGNC:20210]	-5.2	-2
7078	*ZNF737*	100129842	zinc finger protein 737 [Source:HGNC Symbol;Acc:HGNC:32468]	-5.2	-2
7079	*PRKACB*	5567	protein kinase cAMP-activated catalytic subunit beta [Source:HGNC Symbol;Acc:HGNC:9381]	-5.2	-2
7080	*MALL*	7851	mal, T-cell differentiation protein like [Source:HGNC Symbol;Acc:HGNC:6818]	-5.2	-2
7081	*NEK8*	284086	NIMA related kinase 8 [Source:HGNC Symbol;Acc:HGNC:13387]	-5.2	-2
7082	*TANGO6*	79613	transport and golgi organization 6 homolog [Source:HGNC Symbol;Acc:HGNC:25749]	-5.2	-2
7083	*GDPD1*	284161	glycerophosphodiester phosphodiesterase domain containing 1 [Source:HGNC Symbol;Acc:HGNC:20883]	-5.2	-2
7084	*TRAF3IP1*	26146	TRAF3 interacting protein 1 [Source:HGNC Symbol;Acc:HGNC:17861]	-5.2	-2
7085	*OTUD5*	55593	OTU deubiquitinase 5 [Source:HGNC Symbol;Acc:HGNC:25402]	-5.2	-2
7086	*GAGE12C*	729422	G antigen 12C [Source:HGNC Symbol;Acc:HGNC:28402]	-5.2	-2
7087	*GAGE12D*	100132399	G antigen 12D [Source:HGNC Symbol;Acc:HGNC:31904]	-5.2	-2
7088	*GAGE12E*	26748	G antigen 12E [Source:HGNC Symbol;Acc:HGNC:31905]	-5.2	-2
7089	*GAGE12F*	100008586	G antigen 12F [Source:HGNC Symbol;Acc:HGNC:31906]	-5.2	-2
7090	*GAGE12G*	645073	G antigen 12G [Source:HGNC Symbol;Acc:HGNC:31907]	-5.2	-2
7091	*GAGE12H*	729442	G antigen 12H [Source:HGNC Symbol;Acc:HGNC:31908]	-5.2	-2
7092	*GAGE12J*	729396	G antigen 12J [Source:HGNC Symbol;Acc:HGNC:17778]	-5.2	-2
7093	*GAGE13*	645051	G antigen 13 [Source:HGNC Symbol;Acc:HGNC:29081]	-5.2	-2
7094	*GAGE2A*	729447	G antigen 2A [Source:HGNC Symbol;Acc:HGNC:4099]	-5.2	-2
7095	*GAGE2B*	645037	G antigen 2B	-5.2	-2
7096	*GAGE2C*	2574	G antigen 2C	-5.2	-2
7097	*GAGE2D*	729408	G antigen 2D	-5.2	-2
7098	*GAGE2E*	26749	G antigen 2E [Source:HGNC Symbol;Acc:HGNC:31960]	-5.2	-2
7099	*GAGE4*	2576	G antigen 4	-5.2	-2
7100	*GAGE5*	2577	G antigen 5	-5.2	-2
7101	*GAGE7*	2579	G antigen 7	-5.2	-2
7102	*GAGE8*	100101629	G antigen 8	-5.2	-2
7103	*RNF41*	10193	ring finger protein 41 [Source:HGNC Symbol;Acc:HGNC:18401]	-5.2	-2
7104	*GTF2H2*	2966	general transcription factor IIH subunit 2 [Source:HGNC Symbol;Acc:HGNC:4656]	-5.2	-2
7105	*ZNF177*	7730	zinc finger protein 177 [Source:HGNC Symbol;Acc:HGNC:12966]	-5.2	-2
7106	*PTPN14*	5784	protein tyrosine phosphatase, non-receptor type 14 [Source:HGNC Symbol;Acc:HGNC:9647]	-5.2	-2
7107	*WNT9B*	7484	Wnt family member 9B [Source:HGNC Symbol;Acc:HGNC:12779]	-5.2	-2
7108	*TLDC1*	57707	TBC/LysM-associated domain containing 1 [Source:HGNC Symbol;Acc:HGNC:29325]	-5.2	-2
7109	*MED18*	54797	mediator complex subunit 18 [Source:HGNC Symbol;Acc:HGNC:25944]	-5.2	-2
7110	*C8orf86*	389649	chromosome 8 open reading frame 86 [Source:HGNC Symbol;Acc:HGNC:33774]	-5.2	-2
7111	*ZADH2*	284273	zinc binding alcohol dehydrogenase domain containing 2 [Source:HGNC Symbol;Acc:HGNC:28697]	-5.2	-2
7112	*LIMK1*	3984	LIM domain kinase 1 [Source:HGNC Symbol;Acc:HGNC:6613]	-5.2	-2
7113	*SLC16A4*	9122	solute carrier family 16 member 4 [Source:HGNC Symbol;Acc:HGNC:10925]	-5.2	-2
7114	*CCNT1*	904	cyclin T1 [Source:HGNC Symbol;Acc:HGNC:1599]	-5.2	-2
7115	*DDX51*	317781	DEAD-box helicase 51 [Source:HGNC Symbol;Acc:HGNC:20082]	-5.2	-2
7116	*ZNF665*	79788	zinc finger protein 665 [Source:HGNC Symbol;Acc:HGNC:25885]	-5.2	-2
7117	*C5orf24*	134553	chromosome 5 open reading frame 24 [Source:HGNC Symbol;Acc:HGNC:26746]	-5.2	-2
7118	*IRGQ*	126298	immunity-related GTPase Q [Source:HGNC Symbol;Acc:HGNC:24868]	-5.2	-2
7119	*C2orf15*	150590	chromosome 2 open reading frame 15 [Source:HGNC Symbol;Acc:HGNC:28436]	-5.2	-2
7120	*PEX26*	55670	peroxisomal biogenesis factor 26 [Source:HGNC Symbol;Acc:HGNC:22965]	-5.2	-2
7121	*DFFA*	1676	DNA fragmentation factor subunit alpha [Source:HGNC Symbol;Acc:HGNC:2772]	-5.2	-2
7122	*APOL2*	23780	apolipoprotein L2 [Source:HGNC Symbol;Acc:HGNC:619]	-5.2	-2
7123	*SLC52A1*	55065	solute carrier family 52 member 1 [Source:HGNC Symbol;Acc:HGNC:30225]	-5.2	-2
7124	*SLC25A51*	92014	solute carrier family 25 member 51 [Source:HGNC Symbol;Acc:HGNC:23323]	-5.2	-2

7125	*PAWR*	5074	pro-apoptotic WT1 regulator [Source:HGNC Symbol;Acc:HGNC:8614]	-5.2	-2
7126	*IFIT3*	3437	interferon induced protein with tetratricopeptide repeats 3 [Source:HGNC Symbol;Acc:HGNC:5411]	-5.2	-2
7127	*ZNF747*	65988	zinc finger protein 747 [Source:HGNC Symbol;Acc:HGNC:28350]	-5.2	-2
7128	*NDST4*	64579	N-deacetylase/N-sulfotransferase 4 [Source:HGNC Symbol;Acc:HGNC:20779]	-5.2	-2
7129	*STK35*	140901	serine/threonine kinase 35 [Source:HGNC Symbol;Acc:HGNC:16254]	-5.2	-2
7130	*CYP3A43*	64816	cytochrome P450 family 3 subfamily A member 43 [Source:HGNC Symbol;Acc:HGNC:17450]	-5.2	-2
7131	*PLPPR2*	64748	phospholipid phosphatase related 2 [Source:HGNC Symbol;Acc:HGNC:29566]	-5.2	-2
7132	*DMC1*	11144	DNA meiotic recombinase 1 [Source:HGNC Symbol;Acc:HGNC:2927]	-5.2	-2
7133	*SMC6*	79677	structural maintenance of chromosomes 6 [Source:HGNC Symbol;Acc:HGNC:20466]	-5.2	-2
7134	*WDR92*	116143	WD repeat domain 92 [Source:HGNC Symbol;Acc:HGNC:25176]	-5.2	-2
7135	*MED20*	9477	mediator complex subunit 20 [Source:HGNC Symbol;Acc:HGNC:16840]	-5.3	-2
7136	*MRPL40*	64976	mitochondrial ribosomal protein L40 [Source:HGNC Symbol;Acc:HGNC:14491]	-5.3	-2
7137	*GLS2*	27165	glutaminase 2 [Source:HGNC Symbol;Acc:HGNC:29570]	-5.3	-2
7138	*PPP1R12B*	4660	protein phosphatase 1 regulatory subunit 12B [Source:HGNC Symbol;Acc:HGNC:7619]	-5.3	-2
7139	*PCDH18*	54510	protocadherin 18 [Source:HGNC Symbol;Acc:HGNC:14268]	-5.3	-2
7140	*GALNT15*	117248	polypeptide N-acetylgalactosaminyltransferase 15 [Source:HGNC Symbol;Acc:HGNC:21531]	-5.3	-2
7141	*PHACTR4*	65979	phosphatase and actin regulator 4 [Source:HGNC Symbol;Acc:HGNC:25793]	-5.3	-2
7142	*PRR11*	55771	proline rich 11 [Source:HGNC Symbol;Acc:HGNC:25619]	-5.3	-2
7143	*HTR3E*	285242	5-hydroxytryptamine receptor 3E [Source:HGNC Symbol;Acc:HGNC:24005]	-5.3	-2
7144	*ZNF814*	730051	zinc finger protein 814 [Source:HGNC Symbol;Acc:HGNC:33258]	-5.3	-2
7145	*ZNF257*	113835	zinc finger protein 257 [Source:HGNC Symbol;Acc:HGNC:13498]	-5.3	-2
7146	*GJC1*	10052	gap junction protein gamma 1 [Source:HGNC Symbol;Acc:HGNC:4280]	-5.3	-2
7147	*CCBE1*	147372	collagen and calcium binding EGF domains 1 [Source:HGNC Symbol;Acc:HGNC:29426]	-5.3	-2
7148	*ARHGAP28*	79822	Rho GTPase activating protein 28 [Source:HGNC Symbol;Acc:HGNC:25509]	-5.3	-2
7149	*LIN54*	132660	lin-54 DREAM MuvB core complex component [Source:HGNC Symbol;Acc:HGNC:25397]	-5.3	-2
7150	*ELMOD1*	55531	ELMO domain containing 1 [Source:HGNC Symbol;Acc:HGNC:25334]	-5.3	-2
7151	*DYNC2LI1*	51626	dynein cytoplasmic 2 light intermediate chain 1 [Source:HGNC Symbol;Acc:HGNC:24595]	-5.3	-2
7152	*GLB1L*	79411	galactosidase beta 1 like [Source:HGNC Symbol;Acc:HGNC:28129]	-5.3	-2
7153	*SLC7A11*	23657	solute carrier family 7 member 11 [Source:HGNC Symbol;Acc:HGNC:11059]	-5.3	-2
7154	*DNAJC24*	120526	DnaJ heat shock protein family (Hsp40) member C24 [Source:HGNC Symbol;Acc:HGNC:26979]	-5.3	-2
7155	*NYAP2*	57624	neuronal tyrosine-phosphorylated phosphoinositide-3-kinase adaptor 2 [Source:HGNC Symbol;Acc:HGNC:29291]	-5.3	-2
7156	*CRAMP1*	57585	cramped chromatin regulator homolog 1 [Source:HGNC Symbol;Acc:HGNC:14122]	-5.3	-2
7157	*CDH6*	1004	cadherin 6 [Source:HGNC Symbol;Acc:HGNC:1765]	-5.3	-2
7158	*CMSS1*	84319	cms1 ribosomal small subunit homolog (yeast) [Source:HGNC Symbol;Acc:HGNC:28666]	-5.3	-2
7159	*TMEM38B*	55151	transmembrane protein 38B [Source:HGNC Symbol;Acc:HGNC:25535]	-5.3	-2
7160	*RBSN*	64145	rabenosyn, RAB effector [Source:HGNC Symbol;Acc:HGNC:20759]	-5.4	-2
7161	*ZNF543*	125919	zinc finger protein 543 [Source:HGNC Symbol;Acc:HGNC:25281]	-5.4	-2
7162	*FOXK1*	221937	forkhead box K1 [Source:HGNC Symbol;Acc:HGNC:23480]	-5.4	-2
7163	*NOTCH3*	4854	notch 3 [Source:HGNC Symbol;Acc:HGNC:7883]	-5.4	-2
7164	*ZC3H6*	376940	zinc finger CCCH-type containing 6 [Source:HGNC Symbol;Acc:HGNC:24762]	-5.4	-2
7165	*MUC20*	200958	mucin 20, cell surface associated [Source:HGNC Symbol;Acc:HGNC:23282]	-5.4	-2
7166	*ALDH3B1*	221	aldehyde dehydrogenase 3 family member B1 [Source:HGNC Symbol;Acc:HGNC:410]	-5.4	-2
7167	*RABAC1*	10567	Rab acceptor 1 [Source:HGNC Symbol;Acc:HGNC:9794]	-5.4	-2
7168	*NFE2L2*	4780	nuclear factor, erythroid 2 like 2 [Source:HGNC Symbol;Acc:HGNC:7782]	-5.4	-2
7169	*MAPK10*	5602	mitogen-activated protein kinase 10 [Source:HGNC Symbol;Acc:HGNC:6872]	-5.4	-2
7170	*SAMD5*	389432	sterile alpha motif domain containing 5 [Source:HGNC Symbol;Acc:HGNC:21180]	-5.4	-2
7171	*COL6A3*	1293	collagen type VI alpha 3 chain [Source:HGNC Symbol;Acc:HGNC:2213]	-5.4	-2
7172	*CSMD3*	114788	CUB and Sushi multiple domains 3 [Source:HGNC Symbol;Acc:HGNC:19291]	-5.4	-2
7173	*TNFRSF9*	3604	tumor necrosis factor receptor superfamily member 9 [Source:HGNC Symbol;Acc:HGNC:11924]	-5.4	-2
7174	*CPT1C*	126129	carnitine palmitoyltransferase 1C [Source:HGNC Symbol;Acc:HGNC:18540]	-5.4	-2
7175	*ARHGAP1*	392	Rho GTPase activating protein 1 [Source:HGNC Symbol;Acc:HGNC:673]	-5.4	-2
7176	*SPAG1*	6674	sperm associated antigen 1 [Source:HGNC Symbol;Acc:HGNC:11212]	-5.4	-2
7177	*LRRD1*	401387	leucine rich repeats and death domain containing 1 [Source:HGNC Symbol;Acc:HGNC:34300]	-5.4	-2
7178	*MAP3K6*	9064	mitogen-activated protein kinase kinase kinase 6 [Source:HGNC Symbol;Acc:HGNC:6858]	-5.4	-2
7179	*PCDH11Y*	83259	protocadherin 11 Y-linked [Source:HGNC Symbol;Acc:HGNC:15813]	-5.4	-2
7180	*SCEL*	8796	sciellin [Source:HGNC Symbol;Acc:HGNC:10573]	-5.4	-2
7181	*FKBP14*	55033	FK506 binding protein 14 [Source:HGNC Symbol;Acc:HGNC:18625]	-5.4	-2
7182	*ZNF341*	84905	zinc finger protein 341 [Source:HGNC Symbol;Acc:HGNC:15992]	-5.4	-2
7183	*PDP2*	57546	pyruvate dehyrogenase phosphatase catalytic subunit 2 [Source:HGNC Symbol;Acc:HGNC:30263]	-5.4	-2
7184	*CEP83*	51134	centrosomal protein 83 [Source:HGNC Symbol;Acc:HGNC:17966]	-5.4	-2
7185	*NUGGC*	389643	nuclear GTPase, germinal center associated [Source:HGNC Symbol;Acc:HGNC:33550]	-5.4	-2
7186	*ZBTB8A*	653121	zinc finger and BTB domain containing 8A [Source:HGNC Symbol;Acc:HGNC:24172]	-5.4	-2
7187	*GPC6*	10082	glypican 6 [Source:HGNC Symbol;Acc:HGNC:4454]	-5.4	-2
7188	*HERC4*	26091	HECT and RLD domain containing E3 ubiquitin protein ligase 4 [Source:HGNC Symbol;Acc:HGNC:24521]	-5.4	-2
7189	*CDKAL1*	54901	CDK5 regulatory subunit associated protein 1 like 1 [Source:HGNC Symbol;Acc:HGNC:21050]	-5.4	-2
7190	*ZNF766*	90321	zinc finger protein 766 [Source:HGNC Symbol;Acc:HGNC:28063]	-5.4	-2
7191	*PRR19*	284338	proline rich 19 [Source:HGNC Symbol;Acc:HGNC:33728]	-5.4	-2
7192	*TNKS*	8658	tankyrase [Source:HGNC Symbol;Acc:HGNC:11941]	-5.4	-2
7193	*CCL16*	6360	C-C motif chemokine ligand 16 [Source:HGNC Symbol;Acc:HGNC:10614]	-5.4	-2
7194	*CC2D2A*	57545	coiled-coil and C2 domain containing 2A [Source:HGNC Symbol;Acc:HGNC:29253]	-5.4	-2
7195	*CHMP1A*	5119	charged multivesicular body protein 1A [Source:HGNC Symbol;Acc:HGNC:8740]	-5.4	-2
7196	*AIPL1*	23746	aryl hydrocarbon receptor interacting protein like 1 [Source:HGNC Symbol;Acc:HGNC:359]	-5.5	-2

7197	*ITIH5*	80760	inter-alpha-trypsin inhibitor heavy chain family member 5 [Source:HGNC Symbol;Acc:HGNC:21449]	-5.5	-2
7198	*LRP12*	29967	LDL receptor related protein 12 [Source:HGNC Symbol;Acc:HGNC:31708]	-5.5	-2
7199	*PNPLA1*	285848	patatin like phospholipase domain containing 1 [Source:HGNC Symbol;Acc:HGNC:21246]	-5.5	-2
7200	*SBSPON*	157869	somatomedin B and thrombospondin type 1 domain containing [Source:HGNC Symbol;Acc:HGNC:30362]	-5.5	-2
7201	*SEC14L4*	284904	SEC14 like lipid binding 4 [Source:HGNC Symbol;Acc:HGNC:20627]	-5.5	-2
7202	*CEP41*	95681	centrosomal protein 41 [Source:HGNC Symbol;Acc:HGNC:12370]	-5.5	-2
7203	*GNL3L*	54552	G protein nucleolar 3 like [Source:HGNC Symbol;Acc:HGNC:25553]	-5.5	-2
7204	*CHRNB1*	1140	cholinergic receptor nicotinic beta 1 subunit [Source:HGNC Symbol;Acc:HGNC:1961]	-5.5	-2
7205	*PIWIL2*	55124	piwi like RNA-mediated gene silencing 2 [Source:HGNC Symbol;Acc:HGNC:17644]	-5.5	-2
7206	*PIF1*	80119	PIF1 5'-to-3' DNA helicase [Source:HGNC Symbol;Acc:HGNC:26220]	-5.5	-2
7207	*RDH10*	157506	retinol dehydrogenase 10 (all-trans) [Source:HGNC Symbol;Acc:HGNC:19975]	-5.5	-2
7208	*APOC2*	344	apolipoprotein C2 [Source:HGNC Symbol;Acc:HGNC:609]	-5.5	-2
7209	*APOC4*	346	apolipoprotein C4 [Source:HGNC Symbol;Acc:HGNC:611]	-5.5	-2
7210	*SRD5A3*	79644	steroid 5 alpha-reductase 3 [Source:HGNC Symbol;Acc:HGNC:25812]	-5.5	-2
7211	*CHRNB4*	1143	cholinergic receptor nicotinic beta 4 subunit [Source:HGNC Symbol;Acc:HGNC:1964]	-5.5	-2
7212	*AIG1*	51390	androgen-induced 1 [Source:HGNC Symbol;Acc:HGNC:21607]	-5.5	-2
7213	*YAP1*	10413	Yes associated protein 1 [Source:HGNC Symbol;Acc:HGNC:16262]	-5.5	-2
7214	*EXPH5*	23086	exophilin 5 [Source:HGNC Symbol;Acc:HGNC:30578]	-5.5	-2
7215	*SHPK*	23729	sedoheptulokinase [Source:HGNC Symbol;Acc:HGNC:1492]	-5.5	-2
7216	*MORN4*	118812	MORN repeat containing 4 [Source:HGNC Symbol;Acc:HGNC:24001]	-5.5	-2
7217	*RRP15*	51018	ribosomal RNA processing 15 homolog [Source:HGNC Symbol;Acc:HGNC:24255]	-5.5	-2
7218	*TMEM45A*	55076	transmembrane protein 45A [Source:HGNC Symbol;Acc:HGNC:25480]	-5.5	-2
7219	*NCOA7*	135112	nuclear receptor coactivator 7 [Source:HGNC Symbol;Acc:HGNC:21081]	-5.5	-2
7220	*LMOD3*	56203	leiomodin 3 [Source:HGNC Symbol;Acc:HGNC:6649]	-5.6	-2
7221	*DDX10*	1662	DEAD-box helicase 10 [Source:HGNC Symbol;Acc:HGNC:2735]	-5.6	-2
7222	*RALGAPA2*	57186	Ral GTPase activating protein catalytic alpha subunit 2 [Source:HGNC Symbol;Acc:HGNC:16207]	-5.6	-2
7223	*ZNF829*	374899	zinc finger protein 829 [Source:HGNC Symbol;Acc:HGNC:34032]	-5.6	-2
7224	*ACPP*	55	acid phosphatase, prostate [Source:HGNC Symbol;Acc:HGNC:125]	-5.6	-2
7225	*LGALS4*	3960	galectin 4 [Source:HGNC Symbol;Acc:HGNC:6565]	-5.6	-2
7226	*DGKB*	1607	diacylglycerol kinase beta [Source:HGNC Symbol;Acc:HGNC:2850]	-5.6	-2
7227	*LCMT1*	51451	leucine carboxyl methyltransferase 1 [Source:HGNC Symbol;Acc:HGNC:17557]	-5.6	-2
7228	*SLC39A2*	29986	solute carrier family 39 member 2 [Source:HGNC Symbol;Acc:HGNC:17127]	-5.6	-2
7229	*ADAMTS18*	170692	ADAM metallopeptidase with thrombospondin type 1 motif 18 [Source:HGNC Symbol;Acc:HGNC:17110]	-5.6	-2
7230	*DENND2A*	27147	DENN domain containing 2A [Source:HGNC Symbol;Acc:HGNC:22212]	-5.6	-2
7231	*ADCY10*	55811	adenylate cyclase 10 (soluble) [Source:HGNC Symbol;Acc:HGNC:21285]	-5.6	-2
7232	*GALNT4*	8693	polypeptide N-acetylgalactosaminyltransferase 4 [Source:HGNC Symbol;Acc:HGNC:4126]	-5.6	-2
7233	*TP53I3*	9540	tumor protein p53 inducible protein 3 [Source:HGNC Symbol;Acc:HGNC:19373]	-5.6	-2
7234	*SH3TC2*	79628	SH3 domain and tetratricopeptide repeats 2 [Source:HGNC Symbol;Acc:HGNC:29427]	-5.6	-2
7235	*CSNK1G1*	53944	casein kinase 1 gamma 1 [Source:HGNC Symbol;Acc:HGNC:2454]	-5.6	-2
7236	*DFFB*	1677	DNA fragmentation factor subunit beta [Source:HGNC Symbol;Acc:HGNC:2773]	-5.6	-2
7237	*MYO3B*	140469	myosin IIIB [Source:HGNC Symbol;Acc:HGNC:15576]	-5.6	-2
7238	*UBN2*	254048	ubinuclein 2 [Source:HGNC Symbol;Acc:HGNC:21931]	-5.6	-2
7239	*PIWIL3*	440822	piwi like RNA-mediated gene silencing 3 [Source:HGNC Symbol;Acc:HGNC:18443]	-5.6	-2
7240	*TLR6*	10333	toll like receptor 6 [Source:HGNC Symbol;Acc:HGNC:16711]	-5.6	-2
7241	*NCBP1*	4686	nuclear cap binding protein subunit 1 [Source:HGNC Symbol;Acc:HGNC:7658]	-5.6	-2
7242	*DIEXF*	27042	digestive organ expansion factor homolog (zebrafish) [Source:HGNC Symbol;Acc:HGNC:28440]	-5.6	-2
7243	*ZC3H7B*	23264	zinc finger CCCH-type containing 7B [Source:HGNC Symbol;Acc:HGNC:30869]	-5.6	-2
7244	*MLXIPL*	51085	MLX interacting protein like [Source:HGNC Symbol;Acc:HGNC:12744]	-5.6	-2
7245	*COLGALT1*	79709	collagen beta(1-O)galactosyltransferase 1 [Source:HGNC Symbol;Acc:HGNC:26182]	-5.6	-2
7246	*ZNF493*	284443	zinc finger protein 493 [Source:HGNC Symbol;Acc:HGNC:23708]	-5.7	-2
7247	*MFGE8*	4240	milk fat globule-EGF factor 8 protein [Source:HGNC Symbol;Acc:HGNC:7036]	-5.7	-2
7248	*GPR143*	4935	G protein-coupled receptor 143 [Source:HGNC Symbol;Acc:HGNC:20145]	-5.7	-2
7249	*FAM208A*	23272	family with sequence similarity 208 member A [Source:HGNC Symbol;Acc:HGNC:30314]	-5.7	-2
7250	*ZNF273*	10793	zinc finger protein 273 [Source:HGNC Symbol;Acc:HGNC:13067]	-5.7	-2
7251	*LRRC56*	115399	leucine rich repeat containing 56 [Source:HGNC Symbol;Acc:HGNC:25430]	-5.7	-2
7252	*ITGB6*	3694	integrin subunit beta 6 [Source:HGNC Symbol;Acc:HGNC:6161]	-5.7	-2
7253	*SYT17*	51760	synaptotagmin 17 [Source:HGNC Symbol;Acc:HGNC:24119]	-5.7	-2
7254	*KLHL21*	9903	kelch like family member 21 [Source:HGNC Symbol;Acc:HGNC:29041]	-5.7	-2
7255	*METTL20*	254013	Electron Transfer Flavoprotein Beta Subunit Lysine	-5.7	-2
7256	*C19orf12*	83636	chromosome 19 open reading frame 12 [Source:HGNC Symbol;Acc:HGNC:25443]	-5.7	-2
7257	*WDR13*	64743	WD repeat domain 13 [Source:HGNC Symbol;Acc:HGNC:14352]	-5.7	-2
7258	*MAGEF1*	64110	MAGE family member F1 [Source:HGNC Symbol;Acc:HGNC:29639]	-5.7	-2
7259	*TCEANC*	170082	transcription elongation factor A N-terminal and central domain containing [Source:HGNC Symbol;Acc:HGNC:28277]	-5.7	-2
7260	*ISY1*	57461	ISY1 splicing factor homolog [Source:HGNC Symbol;Acc:HGNC:29201]	-5.7	-2
7261	*PREX2*	80243	phosphatidylinositol-3,4,5-trisphosphate dependent Rac exchange factor 2 [Source:HGNC Symbol;Acc:HGNC:22950]	-5.7	-2
7262	*C19orf70*	125988	chromosome 19 open reading frame 70 [Source:HGNC Symbol;Acc:HGNC:33702]	-5.7	-2
7263	*TGFBR3*	7049	transforming growth factor beta receptor 3 [Source:HGNC Symbol;Acc:HGNC:11774]	-5.7	-2
7264	*ERCC4*	2072	ERCC excision repair 4, endonuclease catalytic subunit [Source:HGNC Symbol;Acc:HGNC:3436]	-5.7	-2
7265	*DAND5*	199699	DAN domain BMP antagonist family member 5 [Source:HGNC Symbol;Acc:HGNC:26780]	-5.7	-2
7266	*KCNQ3*	3786	potassium voltage-gated channel subfamily Q member 3 [Source:HGNC Symbol;Acc:HGNC:6297]	-5.7	-2
7267	*PPP2R3C*	55012	protein phosphatase 2 regulatory subunit B"gamma [Source:HGNC Symbol;Acc:HGNC:17485]	-5.7	-2
7268	*FBXO6*	26270	F-box protein 6 [Source:HGNC Symbol;Acc:HGNC:13585]	-5.7	-2
7269	*PARD6G*	84552	par-6 family cell polarity regulator gamma [Source:HGNC Symbol;Acc:HGNC:16076]	-5.7	-2
7270	*MARVELD3*	91862	MARVEL domain containing 3 [Source:HGNC Symbol;Acc:HGNC:30525]	-5.7	-2

7271	*MTFMT*	123263	mitochondrial methionyl-tRNA formyltransferase [Source:HGNC Symbol;Acc:HGNC:29666]	-5.7	-2
7272	*TRPM8*	79054	transient receptor potential cation channel subfamily M member 8 [Source:HGNC Symbol;Acc:HGNC:17961]	-5.7	-2
7273	*ATCAY*	85300	ataxia, cerebellar, Cayman type [Source:HGNC Symbol;Acc:HGNC:779]	-5.7	-2
7274	*SLC4A8*	9498	solute carrier family 4 member 8 [Source:HGNC Symbol;Acc:HGNC:11034]	-5.7	-2
7275	*AKAP8*	10270	A-kinase anchoring protein 8 [Source:HGNC Symbol;Acc:HGNC:378]	-5.7	-2
7276	*PLCXD2*	257068	phosphatidylinositol specific phospholipase C X domain containing 2 [Source:HGNC Symbol;Acc:HGNC:26462]	-5.7	-2
7277	*SLC33A1*	9197	solute carrier family 33 member 1 [Source:HGNC Symbol;Acc:HGNC:95]	-5.7	-2
7278	*RNF212*	285498	ring finger protein 212 [Source:HGNC Symbol;Acc:HGNC:27729]	-5.7	-2
7279	*ARSK*	153642	arylsulfatase family member K [Source:HGNC Symbol;Acc:HGNC:25239]	-5.7	-2
7280	*CCDC122*	160857	coiled-coil domain containing 122 [Source:HGNC Symbol;Acc:HGNC:26478]	-5.7	-2
7281	*SLC25A4*	291	solute carrier family 25 member 4 [Source:HGNC Symbol;Acc:HGNC:10990]	-5.8	-2
7282	*HOOK3*	84376	hook microtubule-tethering protein 3 [Source:HGNC Symbol;Acc:HGNC:23576]	-5.8	-2
7283	*FAM227A*	646851	family with sequence similarity 227 member A [Source:HGNC Symbol;Acc:HGNC:44197]	-5.8	-2
7284	*MUC4*	4585	mucin 4, cell surface associated [Source:HGNC Symbol;Acc:HGNC:7514]	-5.8	-2
7285	*CAMK2G*	818	calcium/calmodulin dependent protein kinase II gamma [Source:HGNC Symbol;Acc:HGNC:1463]	-5.8	-2
7286	*GEN1*	348654	GEN1, Holliday junction 5' flap endonuclease [Source:HGNC Symbol;Acc:HGNC:26881]	-5.8	-2
7287	*CABP4*	57010	calcium binding protein 4 [Source:HGNC Symbol;Acc:HGNC:1386]	-5.8	-2
7288	*ITSN1*	6453	intersectin 1 [Source:HGNC Symbol;Acc:HGNC:6183]	-5.8	-2
7289	*TMEM19*	55266	transmembrane protein 19 [Source:HGNC Symbol;Acc:HGNC:25605]	-5.8	-2
7290	*CEP135*	9662	centrosomal protein 135 [Source:HGNC Symbol;Acc:HGNC:29086]	-5.8	-2
7291	*LIAS*	11019	lipoic acid synthetase [Source:HGNC Symbol;Acc:HGNC:16429]	-5.8	-2
7292	*BCAT2*	587	branched chain amino acid transaminase 2 [Source:HGNC Symbol;Acc:HGNC:977]	-5.8	-2
7293	*ANKRD65*	441869	ankyrin repeat domain 65 [Source:HGNC Symbol;Acc:HGNC:42950]	-5.8	-2
7294	*PPARA*	5465	peroxisome proliferator activated receptor alpha [Source:HGNC Symbol;Acc:HGNC:9232]	-5.8	-2
7295	*PPP1R3B*	79660	protein phosphatase 1 regulatory subunit 3B [Source:HGNC Symbol;Acc:HGNC:14942]	-5.8	-2
7296	*GPATCH1*	55094	G-patch domain containing 1 [Source:HGNC Symbol;Acc:HGNC:24658]	-5.8	-2
7297	*SLC14A2*	8170	solute carrier family 14 member 2 [Source:HGNC Symbol;Acc:HGNC:10919]	-5.8	-2
7298	*ZNF805*	390980	zinc finger protein 805 [Source:HGNC Symbol;Acc:HGNC:23272]	-5.8	-2
7299	*TRMT2B*	79979	tRNA methyltransferase 2 homolog B [Source:HGNC Symbol;Acc:HGNC:25748]	-5.8	-2
7300	*TEX2*	55852	testis expressed 2 [Source:HGNC Symbol;Acc:HGNC:30884]	-5.8	-2
7301	*MYBPC1*	4604	myosin binding protein C, slow type [Source:HGNC Symbol;Acc:HGNC:7549]	-5.8	-2
7302	*CHDH*	55349	choline dehydrogenase [Source:HGNC Symbol;Acc:HGNC:24288]	-5.8	-2
7303	*NTHL1*	4913	nth-like DNA glycosylase 1 [Source:HGNC Symbol;Acc:HGNC:8028]	-5.8	-2
7304	*RSPH14*	27156	radial spoke head 14 homolog [Source:HGNC Symbol;Acc:HGNC:13437]	-5.8	-2
7305	*FBXL20*	84961	F-box and leucine rich repeat protein 20 [Source:HGNC Symbol;Acc:HGNC:24679]	-5.8	-2
7306	*FLG2*	388698	filaggrin family member 2 [Source:HGNC Symbol;Acc:HGNC:33276]	-5.8	-2
7307	*MPP4*	58538	membrane palmitoylated protein 4 [Source:HGNC Symbol;Acc:HGNC:13680]	-5.8	-2
7308	*NOCT*	25819	nocturnin [Source:HGNC Symbol;Acc:HGNC:14254]	-5.8	-2
7309	*EREG*	2069	epiregulin [Source:HGNC Symbol;Acc:HGNC:3443]	-5.8	-2
7310	*ZNF714*	148206	zinc finger protein 714 [Source:HGNC Symbol;Acc:HGNC:27124]	-5.8	-2
7311	*FBLIM1*	54751	filamin binding LIM protein 1 [Source:HGNC Symbol;Acc:HGNC:24686]	-5.8	-2
7312	*HEMK1*	51409	HemK methyltransferase family member 1 [Source:HGNC Symbol;Acc:HGNC:24923]	-5.9	-2
7313	*VSTM4*	196740	V-set and transmembrane domain containing 4 [Source:HGNC Symbol;Acc:HGNC:26470]	-5.9	-2
7314	*FBXL18*	80028	F-box and leucine rich repeat protein 18 [Source:HGNC Symbol;Acc:HGNC:21874]	-5.9	-2
7315	*WNK4*	65266	WNK lysine deficient protein kinase 4 [Source:HGNC Symbol;Acc:HGNC:14544]	-5.9	-2
7316	*DACT3*	147906	dishevelled binding antagonist of beta catenin 3 [Source:HGNC Symbol;Acc:HGNC:30745]	-5.9	-2
7317	*L2HGDH*	79944	L-2-hydroxyglutarate dehydrogenase [Source:HGNC Symbol;Acc:HGNC:20499]	-5.9	-2
7318	*ZBTB8OS*	339487	zinc finger and BTB domain containing 8 opposite strand [Source:HGNC Symbol;Acc:HGNC:24094]	-5.9	-2
7319	*PARD6B*	84612	par-6 family cell polarity regulator beta [Source:HGNC Symbol;Acc:HGNC:16245]	-5.9	-2
7320	*RASGRP3*	25780	RAS guanyl releasing protein 3 [Source:HGNC Symbol;Acc:HGNC:14545]	-5.9	-2
7321	*ADIPOQ*	9370	adiponectin, C1Q and collagen domain containing [Source:HGNC Symbol;Acc:HGNC:13633]	-5.9	-2
7322	*PCDH11X*	27328	protocadherin 11 X-linked [Source:HGNC Symbol;Acc:HGNC:8656]	-5.9	-2
7323	*MDS2*	259283	myelodysplastic syndrome 2 translocation associated [Source:HGNC Symbol;Acc:HGNC:29633]	-5.9	-2
7324	*FCF1*	51077	FCF1 rRNA-processing protein [Source:HGNC Symbol;Acc:HGNC:20220]	-5.9	-2
7325	*PLA2G4F*	255189	phospholipase A2 group IVF [Source:HGNC Symbol;Acc:HGNC:27396]	-5.9	-2
7326	*CYP1A2*	1544	cytochrome P450 family 1 subfamily A member 2 [Source:HGNC Symbol;Acc:HGNC:2596]	-5.9	-2
7327	*C12orf65*	91574	chromosome 12 open reading frame 65 [Source:HGNC Symbol;Acc:HGNC:26784]	-5.9	-2
7328	*C4orf26*	152816	chromosome 4 open reading frame 26 [Source:HGNC Symbol;Acc:HGNC:26300]	-5.9	-2
7329	*PROM2*	150696	prominin 2 [Source:HGNC Symbol;Acc:HGNC:20685]	-5.9	-2
7330	*KREMEN1*	83999	kringle containing transmembrane protein 1 [Source:HGNC Symbol;Acc:HGNC:17550]	-6.0	-2
7331	*DNASE2*	1777	deoxyribonuclease II, lysosomal [Source:HGNC Symbol;Acc:HGNC:2960]	-6.0	-2
7332	*MYO16*	23026	myosin XVI [Source:HGNC Symbol;Acc:HGNC:29822]	-6.0	-2
7333	*KIF18B*	146909	kinesin family member 18B [Source:HGNC Symbol;Acc:HGNC:27102]	-6.0	-2
7334	*APOL1*	8542	apolipoprotein L1 [Source:HGNC Symbol;Acc:HGNC:618]	-6.0	-2
7335	*MSH5*	4439	mutS homolog 5 [Source:HGNC Symbol;Acc:HGNC:7328]	-6.0	-2
7336	*AZGP1*	563	alpha-2-glycoprotein 1, zinc-binding [Source:HGNC Symbol;Acc:HGNC:910]	-6.0	-2
7337	*MACC1*	346389	metastasis associated in colon cancer 1 [Source:HGNC Symbol;Acc:HGNC:30215]	-6.0	-2
7338	*GGT6*	124975	gamma-glutamyltransferase 6 [Source:HGNC Symbol;Acc:HGNC:26891]	-6.0	-2
7339	*RASA4*	10156	RAS p21 protein activator 4 [Source:HGNC Symbol;Acc:HGNC:23181]	-6.0	-2
7340	*CLDN16*	10686	claudin 16 [Source:HGNC Symbol;Acc:HGNC:2037]	-6.0	-2
7341	*MPP5*	64398	membrane palmitoylated protein 5 [Source:HGNC Symbol;Acc:HGNC:18669]	-6.0	-2
7342	*HILPDA*	29923	hypoxia inducible lipid droplet associated [Source:HGNC Symbol;Acc:HGNC:28859]	-6.0	-2
7343	*EML2*	24139	echinoderm microtubule associated protein like 2 [Source:HGNC Symbol;Acc:HGNC:18035]	-6.0	-2
7344	*DNAH9*	1770	dynein axonemal heavy chain 9 [Source:HGNC Symbol;Acc:HGNC:2953]	-6.0	-2
7345	*PCGF6*	84108	polycomb group ring finger 6 [Source:HGNC Symbol;Acc:HGNC:21156]	-6.0	-2

7346	*PLBD1*	79887	phospholipase B domain containing 1 [Source:HGNC Symbol;Acc:HGNC:26215]	-6.0	-2
7347	*ATP1A4*	480	ATPase Na+/K+ transporting subunit alpha 4 [Source:HGNC Symbol;Acc:HGNC:14073]	-6.0	-2
7348	*LPGAT1*	9926	lysophosphatidylglycerol acyltransferase 1 [Source:HGNC Symbol;Acc:HGNC:28985]	-6.0	-2
7349	*FAM3B*	54097	family with sequence similarity 3 member B [Source:HGNC Symbol;Acc:HGNC:1253]	-6.0	-2
7350	*MFSD4*	148808	Major Facilitator Superfamily Domain Containing 4	-6.0	-2
7351	*PPP4R3B*	57223	protein phosphatase 4 regulatory subunit 3B [Source:HGNC Symbol;Acc:HGNC:29267]	-6.1	-2
7352	*UBXN2A*	165324	UBX domain protein 2A [Source:HGNC Symbol;Acc:HGNC:27265]	-6.1	-2
7353	*CEP170B*	283638	centrosomal protein 170B [Source:HGNC Symbol;Acc:HGNC:20362]	-6.1	-2
7354	*PIGN*	23556	phosphatidylinositol glycan anchor biosynthesis class N [Source:HGNC Symbol;Acc:HGNC:8967]	-6.1	-2
7355	*LRP4*	4038	LDL receptor related protein 4 [Source:HGNC Symbol;Acc:HGNC:6696]	-6.1	-2
7356	*HEATR5A*	25938	HEAT repeat containing 5A [Source:HGNC Symbol;Acc:HGNC:20276]	-6.1	-2
7357	*ZNF772*	400720	zinc finger protein 772 [Source:HGNC Symbol;Acc:HGNC:33106]	-6.1	-2
7358	*ZNF793*	390927	zinc finger protein 793 [Source:HGNC Symbol;Acc:HGNC:33115]	-6.1	-2
7359	*TTPAL*	79183	alpha tocopherol transfer protein like [Source:HGNC Symbol;Acc:HGNC:16114]	-6.1	-2
7360	*SLC44A3*	126969	solute carrier family 44 member 3 [Source:HGNC Symbol;Acc:HGNC:28689]	-6.1	-2
7361	*SLC27A1*	376497	solute carrier family 27 member 1 [Source:HGNC Symbol;Acc:HGNC:10995]	-6.1	-2
7362	*SLC5A5*	6528	solute carrier family 5 member 5 [Source:HGNC Symbol;Acc:HGNC:11040]	-6.1	-2
7363	*MAGEB10*	139422	MAGE family member B10 [Source:HGNC Symbol;Acc:HGNC:25377]	-6.1	-2
7364	*C10orf82*	143379	chromosome 10 open reading frame 82 [Source:HGNC Symbol;Acc:HGNC:28500]	-6.1	-2
7365	*ACVR2A*	92	activin A receptor type 2A [Source:HGNC Symbol;Acc:HGNC:173]	-6.1	-2
7366	*PITPNM1*	9600	phosphatidylinositol transfer protein membrane associated 1 [Source:HGNC Symbol;Acc:HGNC:9003]	-6.1	-2
7367	*FBXO45*	200933	F-box protein 45 [Source:HGNC Symbol;Acc:HGNC:29148]	-6.1	-2
7368	*HSD17B13*	345275	hydroxysteroid 17-beta dehydrogenase 13 [Source:HGNC Symbol;Acc:HGNC:18685]	-6.1	-2
7369	*MICAL3*	57553	microtubule associated monooxygenase, calponin and LIM domain containing 3 [Source:HGNC Symbol;Acc:HGNC:24694]	-6.1	-2
7370	*PLXDC1*	57125	plexin domain containing 1 [Source:HGNC Symbol;Acc:HGNC:20945]	-6.1	-2
7371	*SYT15*	83849	synaptotagmin 15 [Source:HGNC Symbol;Acc:HGNC:17167]	-6.1	-2
7372	*GRM6*	2916	glutamate metabotropic receptor 6 [Source:HGNC Symbol;Acc:HGNC:4598]	-6.1	-2
7373	*GBP4*	115361	guanylate binding protein 4 [Source:HGNC Symbol;Acc:HGNC:20480]	-6.1	-2
7374	*HES2*	54626	hes family bHLH transcription factor 2 [Source:HGNC Symbol;Acc:HGNC:16005]	-6.1	-2
7375	*CD2*	914	CD2 molecule [Source:HGNC Symbol;Acc:HGNC:1639]	-6.2	-2
7376	*QSOX1*	5768	quiescin sulfhydryl oxidase 1 [Source:HGNC Symbol;Acc:HGNC:9756]	-6.2	-2
7377	*OLAH*	55301	oleoyl-ACP hydrolase [Source:HGNC Symbol;Acc:HGNC:25625]	-6.2	-2
7378	*CARF*	79800	calcium responsive transcription factor [Source:HGNC Symbol;Acc:HGNC:14435]	-6.2	-2
7379	*SH3BP2*	6452	SH3 domain binding protein 2 [Source:HGNC Symbol;Acc:HGNC:10825]	-6.2	-2
7380	*MUC16*	94025	mucin 16, cell surface associated [Source:HGNC Symbol;Acc:HGNC:15582]	-6.2	-2
7381	*CHAC1*	79094	ChaC glutathione specific gamma-glutamylcyclotransferase 1 [Source:HGNC Symbol;Acc:HGNC:28680]	-6.2	-2
7382	*ARNTL2*	56938	aryl hydrocarbon receptor nuclear translocator like 2 [Source:HGNC Symbol;Acc:HGNC:18984]	-6.2	-2
7383	*TRIOBP*	11078	TRIO and F-actin binding protein [Source:HGNC Symbol;Acc:HGNC:17009]	-6.2	-2
7384	*ABCA4*	24	ATP binding cassette subfamily A member 4 [Source:HGNC Symbol;Acc:HGNC:34]	-6.2	-2
7385	*TRIM3*	10612	tripartite motif containing 3 [Source:HGNC Symbol;Acc:HGNC:10064]	-6.2	-2
7386	*OPA3*	80207	optic atrophy 3 (autosomal recessive, with chorea and spastic paraplegia) [Source:HGNC Symbol;Acc:HGNC:8142]	-6.2	-2
7387	*MYLK3*	91807	myosin light chain kinase 3 [Source:HGNC Symbol;Acc:HGNC:29826]	-6.2	-2
7388	*RBPMS2*	348093	RNA binding protein with multiple splicing 2 [Source:HGNC Symbol;Acc:HGNC:19098]	-6.2	-2
7389	*EPAS1*	2034	endothelial PAS domain protein 1 [Source:HGNC Symbol;Acc:HGNC:3374]	-6.2	-2
7390	*FLCN*	201163	folliculin [Source:HGNC Symbol;Acc:HGNC:27310]	-6.2	-2
7391	*BMP8A*	353500	bone morphogenetic protein 8a [Source:HGNC Symbol;Acc:HGNC:21650]	-6.2	-2
7392	*USE1*	55850	unconventional SNARE in the ER 1 [Source:HGNC Symbol;Acc:HGNC:30882]	-6.2	-2
7393	*PARP11*	57097	poly(ADP-ribose) polymerase family member 11 [Source:HGNC Symbol;Acc:HGNC:1186]	-6.2	-2
7394	*NMNAT1*	64802	nicotinamide nucleotide adenylyltransferase 1 [Source:HGNC Symbol;Acc:HGNC:17877]	-6.2	-2
7395	*AP4S1*	11154	adaptor related protein complex 4 sigma 1 subunit [Source:HGNC Symbol;Acc:HGNC:575]	-6.2	-2
7396	*KIAA1614*	57710	KIAA1614 [Source:HGNC Symbol;Acc:HGNC:29327]	-6.3	-2
7397	*VAMP8*	8673	vesicle associated membrane protein 8 [Source:HGNC Symbol;Acc:HGNC:12647]	-6.3	-2
7398	*ITGA1*	3672	integrin subunit alpha 1 [Source:HGNC Symbol;Acc:HGNC:6134]	-6.3	-2
7399	*BCO2*	83875	beta-carotene oxygenase 2 [Source:HGNC Symbol;Acc:HGNC:18503]	-6.3	-2
7400	*SIX4*	51804	SIX homeobox 4 [Source:HGNC Symbol;Acc:HGNC:10890]	-6.3	-2
7401	*CXorf38*	159013	chromosome X open reading frame 38 [Source:HGNC Symbol;Acc:HGNC:28589]	-6.3	-2
7402	*UCHL5*	51377	ubiquitin C-terminal hydrolase L5 [Source:HGNC Symbol;Acc:HGNC:19678]	-6.3	-2
7403	*ATG5*	9474	autophagy related 5 [Source:HGNC Symbol;Acc:HGNC:589]	-6.3	-2
7404	*SPOCK2*	9806	sparc/osteonectin, cwcv and kazal-like domains proteoglycan (testican) 2 [Source:HGNC Symbol;Acc:HGNC:13564]	-6.3	-2
7405	*MEFV*	4210	Mediterranean fever [Source:HGNC Symbol;Acc:HGNC:6998]	-6.3	-2
7406	*KLLN*	100144748	killin, p53-regulated DNA replication inhibitor [Source:HGNC Symbol;Acc:HGNC:37212]	-6.3	-2
7407	*CRX*	1406	cone-rod homeobox [Source:HGNC Symbol;Acc:HGNC:2383]	-6.3	-2
7408	*MSRB3*	253827	methionine sulfoxide reductase B3 [Source:HGNC Symbol;Acc:HGNC:27375]	-6.3	-2
7409	*L1TD1*	54596	LINE-1 type transposase domain containing 1 [Source:HGNC Symbol;Acc:HGNC:25595]	-6.3	-2
7410	*FTSJ2*	29960	Mitochondrial RRNA Methyltransferase 2	-6.4	-2
7411	*AVIL*	10677	advillin [Source:HGNC Symbol;Acc:HGNC:14188]	-6.4	-2
7412	*C1RL*	51279	complement C1r subcomponent like [Source:HGNC Symbol;Acc:HGNC:21265]	-6.4	-2
7413	*HIST2H2BF*	440689	histone cluster 2, H2bf [Source:HGNC Symbol;Acc:HGNC:24700]	-6.4	-2
7414	*TRPM7*	54822	transient receptor potential cation channel subfamily M member 7 [Source:HGNC Symbol;Acc:HGNC:17994]	-6.4	-2
7415	*ZSCAN22*	342945	zinc finger and SCAN domain containing 22 [Source:HGNC Symbol;Acc:HGNC:4929]	-6.4	-2
7416	*METTL6*	131965	methyltransferase like 6 [Source:HGNC Symbol;Acc:HGNC:28343]	-6.4	-2
7417	*ZNF788*	388507	zinc finger family member 788 [Source:HGNC Symbol;Acc:HGNC:33112]	-6.4	-2
7418	*FCRL2*	79368	Fc receptor like 2 [Source:HGNC Symbol;Acc:HGNC:14875]	-6.4	-2
7419	*TRPM3*	80036	transient receptor potential cation channel subfamily M member 3 [Source:HGNC Symbol;Acc:HGNC:17992]	-6.4	-2
7420	*MREG*	55686	melanoregulin [Source:HGNC Symbol;Acc:HGNC:25478]	-6.4	-2

7421	*MFAP2*	4237	microfibrillar associated protein 2 [Source:HGNC Symbol;Acc:HGNC:7033]	-6.4	-2
7422	*GMDS*	2762	GDP-mannose 4,6-dehydratase [Source:HGNC Symbol;Acc:HGNC:4369]	-6.4	-2
7423	*PDZD2*	23037	PDZ domain containing 2 [Source:HGNC Symbol;Acc:HGNC:18486]	-6.4	-2
7424	*TMEM212*	389177	transmembrane protein 212 [Source:HGNC Symbol;Acc:HGNC:34295]	-6.4	-2
7425	*BRIP1*	83990	BRCA1 interacting protein C-terminal helicase 1 [Source:HGNC Symbol;Acc:HGNC:20473]	-6.4	-2
7426	*KNOP1*	400506	lysine rich nucleolar protein 1 [Source:HGNC Symbol;Acc:HGNC:34404]	-6.4	-2
7427	*ZCCHC8*	55596	zinc finger CCHC-type containing 8 [Source:HGNC Symbol;Acc:HGNC:25265]	-6.5	-2
7428	*MYH14*	79784	myosin, heavy chain 14, non-muscle [Source:HGNC Symbol;Acc:HGNC:23212]	-6.5	-2
7429	*H6PD*	9563	hexose-6-phosphate dehydrogenase/glucose 1-dehydrogenase [Source:HGNC Symbol;Acc:HGNC:4795]	-6.5	-2
7430	*ECHDC1*	55862	ethylmalonyl-CoA decarboxylase 1 [Source:HGNC Symbol;Acc:HGNC:21489]	-6.5	-2
7431	*RAB36*	9609	RAB36, member RAS oncogene family [Source:HGNC Symbol;Acc:HGNC:9775]	-6.5	-2
7432	*ICA1L*	130026	islet cell autoantigen 1 like [Source:HGNC Symbol;Acc:HGNC:14442]	-6.5	-2
7433	*PTEN*	5728	phosphatase and tensin homolog [Source:HGNC Symbol;Acc:HGNC:9588]	-6.5	-2
7434	*SIMC1*	375484	SUMO interacting motifs containing 1 [Source:HGNC Symbol;Acc:HGNC:24779]	-6.5	-2
7435	*ADGRF1*	266977	adhesion G protein-coupled receptor F1 [Source:HGNC Symbol;Acc:HGNC:18990]	-6.5	-2
7436	*FAM221A*	340277	family with sequence similarity 221 member A [Source:HGNC Symbol;Acc:HGNC:27977]	-6.5	-2
7437	*ALDH1A3*	220	aldehyde dehydrogenase 1 family member A3 [Source:HGNC Symbol;Acc:HGNC:409]	-6.5	-2
7438	*CROT*	54677	carnitine O-octanoyltransferase [Source:HGNC Symbol;Acc:HGNC:2366]	-6.5	-2
7439	*SLC15A1*	6564	solute carrier family 15 member 1 [Source:HGNC Symbol;Acc:HGNC:10920]	-6.5	-2
7440	*STRIP2*	57464	striatin interacting protein 2 [Source:HGNC Symbol;Acc:HGNC:22209]	-6.5	-2
7441	*TTC39B*	158219	tetratricopeptide repeat domain 39B [Source:HGNC Symbol;Acc:HGNC:23704]	-6.5	-2
7442	*TRMT10B*	158234	tRNA methyltransferase 10B [Source:HGNC Symbol;Acc:HGNC:26454]	-6.5	-2
7443	*ATG10*	83734	autophagy related 10 [Source:HGNC Symbol;Acc:HGNC:20315]	-6.5	-2
7444	*GPKOW*	27238	G-patch domain and KOW motifs [Source:HGNC Symbol;Acc:HGNC:30677]	-6.6	-2
7445	*IAPP*	3375	islet amyloid polypeptide [Source:HGNC Symbol;Acc:HGNC:5329]	-6.6	-2
7446	*SLC46A3*	283537	solute carrier family 46 member 3 [Source:HGNC Symbol;Acc:HGNC:27501]	-6.6	-2
7447	*UBFD1*	56061	ubiquitin family domain containing 1 [Source:HGNC Symbol;Acc:HGNC:30565]	-6.6	-2
7448	*PGM2L1*	283209	phosphoglucomutase 2-like 1 [Source:HGNC Symbol;Acc:HGNC:20898]	-6.6	-2
7449	*NFS1*	9054	NFS1 cysteine desulfurase [Source:HGNC Symbol;Acc:HGNC:15910]	-6.6	-2
7450	*TMEM130*	222865	transmembrane protein 130 [Source:HGNC Symbol;Acc:HGNC:25429]	-6.6	-2
7451	*NCCRP1*	342897	non-specific cytotoxic cell receptor protein 1 homolog (zebrafish) [Source:HGNC Symbol;Acc:HGNC:33739]	-6.6	-2
7452	*AHNAK2*	113146	AHNAK nucleoprotein 2 [Source:HGNC Symbol;Acc:HGNC:20125]	-6.6	-2
7453	*ZNF568*	374900	zinc finger protein 568 [Source:HGNC Symbol;Acc:HGNC:25392]	-6.6	-2
7454	*CD320*	51293	CD320 molecule [Source:HGNC Symbol;Acc:HGNC:16692]	-6.7	-2
7455	*ZBTB3*	79842	zinc finger and BTB domain containing 3 [Source:HGNC Symbol;Acc:HGNC:22918]	-6.7	-2
7456	*SCARF1*	8578	scavenger receptor class F member 1 [Source:HGNC Symbol;Acc:HGNC:16820]	-6.7	-2
7457	*TMEM203*	94107	transmembrane protein 203 [Source:HGNC Symbol;Acc:HGNC:28217]	-6.7	-2
7458	*DZANK1*	55184	double zinc ribbon and ankyrin repeat domains 1 [Source:HGNC Symbol;Acc:HGNC:15858]	-6.7	-2
7459	*PDE4C*	5143	phosphodiesterase 4C [Source:HGNC Symbol;Acc:HGNC:8782]	-6.7	-2
7460	*SLC25A27*	9481	solute carrier family 25 member 27 [Source:HGNC Symbol;Acc:HGNC:21065]	-6.7	-2
7461	*PLEKHG4B*	153478	pleckstrin homology and RhoGEF domain containing G4B [Source:HGNC Symbol;Acc:HGNC:29399]	-6.7	-2
7462	*OGFOD3*	79701	2-oxoglutarate and iron dependent oxygenase domain containing 3 [Source:HGNC Symbol;Acc:HGNC:26174]	-6.7	-2
7463	*FCAR*	2204	Fc fragment of IgA receptor [Source:HGNC Symbol;Acc:HGNC:3608]	-6.7	-2
7464	*AMIGO2*	347902	adhesion molecule with Ig-like domain 2 [Source:HGNC Symbol;Acc:HGNC:24073]	-6.7	-2
7465	*PLEKHH3*	79990	pleckstrin homology, MyTH4 and FERM domain containing H3 [Source:HGNC Symbol;Acc:HGNC:26105]	-6.7	-2
7466	*CXCL17*	284340	C-X-C motif chemokine ligand 17 [Source:HGNC Symbol;Acc:HGNC:19232]	-6.7	-2
7467	*ZSWIM1*	90204	zinc finger SWIM-type containing 1 [Source:HGNC Symbol;Acc:HGNC:16155]	-6.7	-2
7468	*SRL*	6345	sarcalumenin [Source:HGNC Symbol;Acc:HGNC:11295]	-6.8	-2
7469	*ZNF616*	90317	zinc finger protein 616 [Source:HGNC Symbol;Acc:HGNC:28062]	-6.8	-2
7470	*MYH7B*	57644	myosin, heavy chain 7B, cardiac muscle, beta [Source:HGNC Symbol;Acc:HGNC:15906]	-6.8	-2
7471	*RAD51C*	5889	RAD51 paralog C [Source:HGNC Symbol;Acc:HGNC:9820]	-6.8	-2
7472	*RHBG*	57127	Rh family B glycoprotein (gene/pseudogene) [Source:HGNC Symbol;Acc:HGNC:14572]	-6.8	-2
7473	*TIGAR*	57103	TP53 induced glycolysis regulatory phosphatase [Source:HGNC Symbol;Acc:HGNC:1185]	-6.8	-2
7474	*ARGFX*	503582	arginine-fifty homeobox [Source:HGNC Symbol;Acc:HGNC:30146]	-6.8	-2
7475	*ZNF260*	339324	zinc finger protein 260 [Source:HGNC Symbol;Acc:HGNC:13499]	-6.8	-2
7476	*ZNF573*	126231	zinc finger protein 573 [Source:HGNC Symbol;Acc:HGNC:26420]	-6.8	-2
7477	*C21orf62*	56245	chromosome 21 open reading frame 62 [Source:HGNC Symbol;Acc:HGNC:1305]	-6.8	-2
7478	*ZNF780B*	163131	zinc finger protein 780B [Source:HGNC Symbol;Acc:HGNC:33109]	-6.8	-2
7479	*ZNF483*	158399	zinc finger protein 483 [Source:HGNC Symbol;Acc:HGNC:23384]	-6.8	-2
7480	*ST8SIA4*	7903	ST8 alpha-N-acetyl-neuraminide alpha-2,8-sialyltransferase 4 [Source:HGNC Symbol;Acc:HGNC:10871]	-6.8	-2
7481	*INMT*	11185	indolethylamine N-methyltransferase [Source:HGNC Symbol;Acc:HGNC:6069]	-6.8	-2
7482	*KBTBD4*	55709	kelch repeat and BTB domain containing 4 [Source:HGNC Symbol;Acc:HGNC:23761]	-6.8	-2
7483	*AP4E1*	23431	adaptor related protein complex 4 epsilon 1 subunit [Source:HGNC Symbol;Acc:HGNC:573]	-6.8	-2
7484	*MCF2L2*	23101	MCF.2 cell line derived transforming sequence-like 2 [Source:HGNC Symbol;Acc:HGNC:30319]	-6.9	-2
7485	*NRIP2*	83714	nuclear receptor interacting protein 2 [Source:HGNC Symbol;Acc:HGNC:23078]	-6.9	-2
7486	*SHISA9*	729993	shisa family member 9 [Source:HGNC Symbol;Acc:HGNC:37231]	-6.9	-2
7487	*TRIM29*	23650	tripartite motif containing 29 [Source:HGNC Symbol;Acc:HGNC:17274]	-6.9	-2
7488	*CDH7*	1005	cadherin 7 [Source:HGNC Symbol;Acc:HGNC:1766]	-6.9	-2
7489	*POLK*	51426	polymerase (DNA) kappa [Source:HGNC Symbol;Acc:HGNC:9183]	-6.9	-2
7490	*MAP7D3*	79649	MAP7 domain containing 3 [Source:HGNC Symbol;Acc:HGNC:25742]	-6.9	-2
7491	*ABCB9*	23457	ATP binding cassette subfamily B member 9 [Source:HGNC Symbol;Acc:HGNC:50]	-7.0	-2
7492	*TMPRSS4*	56649	transmembrane protease, serine 4 [Source:HGNC Symbol;Acc:HGNC:11878]	-7.0	-2
7493	*NLN*	57486	neurolysin [Source:HGNC Symbol;Acc:HGNC:16058]	-7.0	-2
7494	*TMEM180*	79847	Major Facilitator Superfamily Domain Containing 13A	-7.0	-2

7495	*CHST6*	4166	carbohydrate sulfotransferase 6 [Source:HGNC Symbol;Acc:HGNC:6938]	-7.0	-2
7496	*ZFP2*	80108	ZFP2 zinc finger protein [Source:HGNC Symbol;Acc:HGNC:26138]	-7.0	-2
7497	*LIN28A*	79727	lin-28 homolog A [Source:HGNC Symbol;Acc:HGNC:15986]	-7.0	-2
7498	*TGM2*	7052	transglutaminase 2 [Source:HGNC Symbol;Acc:HGNC:11778]	-7.1	-2
7499	*DEFA1B*	728358	defensin alpha 1B [Source:HGNC Symbol;Acc:HGNC:33596]	-7.1	-2
7500	*PPIL6*	285755	peptidylprolyl isomerase like 6 [Source:HGNC Symbol;Acc:HGNC:21557]	-7.1	-2
7501	*LRRC57*	255252	leucine rich repeat containing 57 [Source:HGNC Symbol;Acc:HGNC:26719]	-7.2	-2
7502	*DEPDC5*	9681	DEP domain containing 5 [Source:HGNC Symbol;Acc:HGNC:18423]	-7.2	-2
7503	*SEMA4C*	54910	semaphorin 4C [Source:HGNC Symbol;Acc:HGNC:10731]	-7.2	-2
7504	*POU5F1*	5460	POU class 5 homeobox 1 [Source:HGNC Symbol;Acc:HGNC:9221]	-7.2	-2
7505	*FASTKD1*	79675	FAST kinase domains 1 [Source:HGNC Symbol;Acc:HGNC:26150]	-7.2	-2
7506	*CLCN5*	1184	chloride voltage-gated channel 5 [Source:HGNC Symbol;Acc:HGNC:2023]	-7.2	-2
7507	*FDXACB1*	91893	ferredoxin-fold anticodon binding domain containing 1 [Source:HGNC Symbol;Acc:HGNC:25110]	-7.3	-2
7508	*CFAP74*	85452	cilia and flagella associated protein 74 [Source:HGNC Symbol;Acc:HGNC:29368]	-7.3	-2
7509	*LCA5L*	150082	LCA5L, lebercilin like [Source:HGNC Symbol;Acc:HGNC:1255]	-7.3	-2
7510	*PPP1R3C*	5507	protein phosphatase 1 regulatory subunit 3C [Source:HGNC Symbol;Acc:HGNC:9293]	-7.3	-2
7511	*ZNHIT6*	54680	zinc finger HIT-type containing 6 [Source:HGNC Symbol;Acc:HGNC:26089]	-7.3	-2
7512	*MRGBP*	55257	MRG/MORF4L binding protein [Source:HGNC Symbol;Acc:HGNC:15866]	-7.3	-2
7513	*PLEKHG1*	57480	pleckstrin homology and RhoGEF domain containing G1 [Source:HGNC Symbol;Acc:HGNC:20884]	-7.3	-2
7514	*ENPP1*	5167	ectonucleotide pyrophosphatase/phosphodiesterase 1 [Source:HGNC Symbol;Acc:HGNC:3356]	-7.4	-2
7515	*CACNG8*	59283	calcium voltage-gated channel auxiliary subunit gamma 8 [Source:HGNC Symbol;Acc:HGNC:13628]	-7.4	-2
7516	*INS-IGF2*	723961	INS-IGF2 readthrough [Source:HGNC Symbol;Acc:HGNC:33527]	-7.4	-2
7517	*ABCC9*	10060	ATP binding cassette subfamily C member 9 [Source:HGNC Symbol;Acc:HGNC:60]	-7.4	-2
7518	*FBXO43*	286151	F-box protein 43 [Source:HGNC Symbol;Acc:HGNC:28521]	-7.4	-2
7519	*ZNF383*	163087	zinc finger protein 383 [Source:HGNC Symbol;Acc:HGNC:18609]	-7.4	-2
7520	*MPO*	4353	myeloperoxidase [Source:HGNC Symbol;Acc:HGNC:7218]	-7.5	-2
7521	*RICTOR*	253260	RPTOR independent companion of MTOR complex 2 [Source:HGNC Symbol;Acc:HGNC:28611]	-7.5	-2
7522	*SETD1A*	9739	SET domain containing 1A [Source:HGNC Symbol;Acc:HGNC:29010]	-7.5	-2
7523	*HOXB5*	3215	homeobox B5 [Source:HGNC Symbol;Acc:HGNC:5116]	-7.5	-2
7524	*LAMB4*	22798	laminin subunit beta 4 [Source:HGNC Symbol;Acc:HGNC:6491]	-7.5	-2
7525	*ZNF662*	389114	zinc finger protein 662 [Source:HGNC Symbol;Acc:HGNC:31930]	-7.5	-2
7526	*RNF138*	51444	ring finger protein 138 [Source:HGNC Symbol;Acc:HGNC:17765]	-7.6	-2
7527	*PCSK5*	5125	proprotein convertase subtilisin/kexin type 5 [Source:HGNC Symbol;Acc:HGNC:8747]	-7.6	-2
7528	*SLC26A4*	5172	solute carrier family 26 member 4 [Source:HGNC Symbol;Acc:HGNC:8818]	-7.7	-2
7529	*DQX1*	165545	DEAQ-box RNA dependent ATPase 1 [Source:HGNC Symbol;Acc:HGNC:20410]	-7.7	-2
7530	*CNRIP1*	25927	cannabinoid receptor interacting protein 1 [Source:HGNC Symbol;Acc:HGNC:24546]	-7.7	-2
7531	*KIAA1919*	91749	Major Facilitator Superfamily Domain Containing 4B	-7.7	-2
7532	*LRRC2*	79442	leucine rich repeat containing 2 [Source:HGNC Symbol;Acc:HGNC:14676]	-7.7	-2
7533	*MRPL50*	54534	mitochondrial ribosomal protein L50 [Source:HGNC Symbol;Acc:HGNC:16654]	-7.8	-2
7534	*ORM1*	5004	orosomucoid 1 [Source:HGNC Symbol;Acc:HGNC:8498]	-7.8	-2
7535	*LRRK1*	79705	leucine rich repeat kinase 1 [Source:HGNC Symbol;Acc:HGNC:18608]	-7.8	-2
7536	*EFHD1*	80303	EF-hand domain family member D1 [Source:HGNC Symbol;Acc:HGNC:29556]	-7.9	-2
7537	*ZNF101*	94039	zinc finger protein 101 [Source:HGNC Symbol;Acc:HGNC:12881]	-7.9	-2
7538	*AQP7*	364	aquaporin 7 [Source:HGNC Symbol;Acc:HGNC:640]	-7.9	-2
7539	*CEACAM6*	4680	carcinoembryonic antigen related cell adhesion molecule 6 [Source:HGNC Symbol;Acc:HGNC:1818]	-7.9	-2
7540	*HSPB6*	126393	heat shock protein family B (small) member 6 [Source:HGNC Symbol;Acc:HGNC:26511]	-8.0	-2
7541	*DTWD2*	285605	DTW domain containing 2 [Source:HGNC Symbol;Acc:HGNC:19334]	-8.0	-2
7542	*C2orf50*	130813	chromosome 2 open reading frame 50 [Source:HGNC Symbol;Acc:HGNC:26324]	-8.0	-2
7543	*ONECUT2*	9480	one cut homeobox 2 [Source:HGNC Symbol;Acc:HGNC:8139]	-8.1	-2
7544	*ZCCHC24*	219654	zinc finger CCHC-type containing 24 [Source:HGNC Symbol;Acc:HGNC:26911]	-8.1	-2
7545	*S100A2*	6273	S100 calcium binding protein A2 [Source:HGNC Symbol;Acc:HGNC:10492]	-8.1	-2
7546	*SCGB1A1*	7356	secretoglobin family 1A member 1 [Source:HGNC Symbol;Acc:HGNC:12523]	-8.2	-2
7547	*POPDC2*	64091	popeye domain containing 2 [Source:HGNC Symbol;Acc:HGNC:17648]	-8.2	-2
7548	*SLPI*	6590	secretory leukocyte peptidase inhibitor [Source:HGNC Symbol;Acc:HGNC:11092]	-8.3	-2
7549	*ADIRF*	10974	adipogenesis regulatory factor [Source:HGNC Symbol;Acc:HGNC:24043]	-8.4	-2
7550	*WRN*	7486	Werner syndrome RecQ like helicase [Source:HGNC Symbol;Acc:HGNC:12791]	-8.5	-2
7551	*QRFPR*	84109	pyroglutamylated RFamide peptide receptor [Source:HGNC Symbol;Acc:HGNC:15565]	-8.5	-2
7552	*SPRR1B*	6699	small proline rich protein 1B [Source:HGNC Symbol;Acc:HGNC:11260]	-8.6	-2
7553	*KCTD12*	115207	potassium channel tetramerization domain containing 12 [Source:HGNC Symbol;Acc:HGNC:14678]	-8.6	-2
7554	*PLEKHH2*	130271	pleckstrin homology, MyTH4 and FERM domain containing H2 [Source:HGNC Symbol;Acc:HGNC:30506]	-8.6	-2
7555	*PLAT*	5327	plasminogen activator, tissue type [Source:HGNC Symbol;Acc:HGNC:9051]	-8.6	-2
7556	*GPX3*	2878	glutathione peroxidase 3 [Source:HGNC Symbol;Acc:HGNC:4555]	-8.9	-2
7557	*SPRR3*	6707	small proline rich protein 3 [Source:HGNC Symbol;Acc:HGNC:11268]	-9.0	-2
7558	*FAM189A2*	9413	family with sequence similarity 189 member A2 [Source:HGNC Symbol;Acc:HGNC:24820]	-9.1	-2
7559	*NRK*	203447	Nik related kinase [Source:HGNC Symbol;Acc:HGNC:25391]	-9.2	-2
7560	*MFAP4*	4239	microfibrillar associated protein 4 [Source:HGNC Symbol;Acc:HGNC:7035]	-9.2	-2
7561	*PCP4*	5121	Purkinje cell protein 4 [Source:HGNC Symbol;Acc:HGNC:8742]	-9.3	-2
7562	*KLK10*	5655	kallikrein related peptidase 10 [Source:HGNC Symbol;Acc:HGNC:6358]	-9.3	-2
7563	*DNAJC5B*	85479	DnaJ heat shock protein family (Hsp40) member C5 beta [Source:HGNC Symbol;Acc:HGNC:24138]	-9.8	-2
7564	*C3*	718	complement component 3 [Source:HGNC Symbol;Acc:HGNC:1318]	-9.9	-2
7565	*C1S*	716	complement component 1, s subcomponent [Source:HGNC Symbol;Acc:HGNC:1247]	-10.2	-2
7566	*MYH11*	4629	myosin, heavy chain 11, smooth muscle [Source:HGNC Symbol;Acc:HGNC:7569]	-10.3	-2
7567	*NPY*	4852	neuropeptide Y [Source:HGNC Symbol;Acc:HGNC:7955]	-10.6	-2
7568	*AEBP1*	165	AE binding protein 1 [Source:HGNC Symbol;Acc:HGNC:303]	-10.6	-2
7569	*ACTG2*	72	actin, gamma 2, smooth muscle, enteric [Source:HGNC Symbol;Acc:HGNC:145]	-11.3	-2

7570	*DES*	1674	desmin [Source:HGNC Symbol;Acc:HGNC:2770]	-11.8	-2
7571	*SPP1*	6696	secreted phosphoprotein 1 [Source:HGNC Symbol;Acc:HGNC:11255]		-2
7572	*ZDHHC21*	340481	zinc finger DHHC-type containing 21 [Source:HGNC Symbol;Acc:HGNC:20750]		-2

Yadavalli et al., 2016. Data-Driven Discovery of Extravasation Pathway in Circulating Tumor Cells

Supplementary Table S2: CTC_FC: Differentially expressed genes in circulating tumor cells when compared to primary tumors, PBMC, normal tissue and tumor cell lines. Data for a total number of 7,209 genes were obtained from reported literature of transcriptomics experiments from five different cancer studies of CTCs.

	Gene Symbol	Gene ID	Prostate CTC-Prostate tumors	Colorectal CTC-PBMC	Melanoma CTC-Melanoma cell lines	Melanoma CTC-Primary Melanocytes	Pancreatic CTC-Non-tumoral Pancreatic tissue	Pancreatic CTC-PBMC	Pancreatic CTC-Pancreatic tumor	Breast CTC-Breast epithelium	Breast CTC-PBMC	Breast CTC-Breast tumors	Chromosome	Gene Start (bp)	Gene End (bp)
1	*SPARC*	6678	-3.2	2.9	3.3		-3.1	3.3	-2.9	-2.6		-2.6	5	151661096	151687165
2	*HINT1*	3094		-1.5	1.7		-1.5		-1.5	-2.3	-1.7	-2.2	5	131159027	131171735
3	*MAP3K7CL*	56911	-3.9	2.3	2.8	2.7	4.4	2.6	4.8				21	29077471	29175889
4	*MFAP3L*	9848	-6.1	2.4			4.5	2.5	4.6	-2.4	-1.6		4	169986597	170033031
5	*PABPN1*	8106	4.6				2.7	2.8	2.7	-2.0	-2.2	-2.1	14	23321289	23326185
6	*ATP5E*	514		3.7			2.9	2.8	3.0		-2.5	-2.5	20	59025467	59032382
7	*BCLAF1*	9774	3.2				-2.8	-1.8	-2.4	-2.2		-1.6	6	136256863	136289851
8	*BTG1*	694	2.0				3.5		3.7	-3.6	-4.3	-2.9	12	92140278	92145897
9	*C9orf16*	79095		2.5	-1.6	-3.2	1.5	1.9	1.5				9	128160260	128163928
10	*DAB2*	1601	-2.7	2.0	-6.4	-4.7		3.6	2.1				5	39371675	39462300
11	*HIST2H2BE*	8349		3.3	-3.4		4.1	2.5	2.8		-1.9		1	149842204	149886652
12	*ITM2A*	9452	3.6	2.6	2.6	4.4				-1.9	-1.6		X	79360384	79367667
13	*LGALSL*	29094	-4.1	2.2			3.8	4.1	3.9	-2.3			2	64453969	64461381
14	*LUC7L3*	51747	4.7		2.6		-3.0	-1.9	-2.8			1.7	17	50719544	50756213
15	*MTURN*	222166	-3.5	2.6	-4.2		3.2	2.7	3.5				7	30134810	30162762
16	*PSMC2*	5701	4.6				-1.8		-1.9	-1.8	-2.0	-1.6	7	103344254	103369395
17	*RGS10*	6001		2.4			3.0	2.7	3.0		-3.0	-2.0	10	119499828	119542708
18	*SNCA*	6622	-3.7	2.0			3.2	3.1	3.7			1.7	4	89724099	89838315
19	*TPM1*	7168		3.7	4.1	1.7	-1.6	2.5	-1.6				15	63042632	63071915
20	*MARCH2*	51257	-3.0	2.0			3.8	2.7	4.0				19	8413270	8439017
21	*SEPT11*	55752			-1.9	-2.4	-2.1	2.0	-1.7				4	76949703	77040384
22	*ABCC4*	10257	-3.9				2.5	2.7	2.5			1.5	13	95019829	95301446
23	*ACTN1*	87		1.9	2.1		3.1	2.3	2.1				14	68874143	68979440
24	*AKIRIN2*	55122		1.7	2.0	2.4	2.0		1.6				6	87675072	87702209
25	*ALOX12*	239					5.8	3.4	5.7	-1.6	-2.0		17	6996065	7010736
26	*APP*	351	-6.2	2.4	-4.1	-6.3		1.8					21	25880550	26171128
27	*BCL2A1*	597			-6.9	-6.4	2.8		3.0		-2.2		15	79960889	79971446
28	*BEND2*	139105	-6.1	1.8			4.4	2.3	4.4				X	18162931	18220883
29	*C5orf15*	56951	2.6		1.9	4.3	-2.1		-2.2				5	133955502	133968787
30	*CARD16*	114769	-7.0		-3.0	2.1	1.6		1.7				11	105041326	105101431
31	*CD59*	966					-3.8	1.7	-3.7	-2.5		-2.6	11	33698261	33736445
32	*CD99*	4267		2.6		-1.8				-1.8	-2.0	-1.9	X	2691179	2741309
33	*CDC14B*	8555	-6.6	3.3			1.6	2.5	2.1				9	96490241	96619830
34	*CDS2*	8760	-3.4		-1.9	-2.2		1.8	1.9				20	5126786	5197887
35	*CLIP1*	6249		1.6			1.7	1.9	1.8	-2.3			12	122271432	122422632
36	*CSF1R*	1436	-4.8	-2.0			1.5		2.1		-2.2		5	150053291	150113372
37	*CYTH1*	9267	3.2	-1.5			2.0	2.1	2.1				17	78674048	78782297
38	*EEF1B2*	1933		-1.7			-1.5			-1.7	-1.8	-1.6	2	206159585	206162928
39	*ESAM*	90952	-4.8	2.3			3.0	4.0	3.2				11	124752583	124762290
40	*F13A1*	2162	-3.6	3.0			1.8	1.5	2.4				6	6144085	6321013
41	*FAM101B*	359845	-4.2		-4.1	-3.5	2.5		2.5				17	439978	445939
42	*FAM162A*	26355	4.0		-4.5	-3.4	-1.5		-1.7				3	122384176	122412334
43	*FAXDC2*	10826	-3.7	3.2			2.4	2.3	2.5				5	154818491	154859252
44	*FHL1*	2273	-7.9	2.9				2.3	1.6	-2.2			X	136146702	136211359
45	*GNAZ*	2781	-2.9	1.8			5.0	3.1	5.6				22	23070361	23125037
46	*GNG11*	2791		3.5		-2.3	1.7	2.5	2.2				7	93921699	93928610
47	*HIST1H2AC*	8334			-5.7		3.6	1.5	2.3		-2.9		6	26124145	26139116
48	*HLA-A*	3105			-2.9	-5.1				-1.9	-3.4	-1.9	6	29941260	29945884

49	*HLA-C*	3107			-3.6	-6.3				-3.3	-4.5	-2.8	6	31268749	31272130
50	*HLA-DPA1*	3113	-6.2	-2.5						-3.3	-2.4	-1.9	6	33064569	33080775
51	*HMGN4*	10473	5.6				-1.8	-1.6	-1.9		-1.6		6	26538405	26546254
52	*HRASLS*	57110	-4.0	1.8			1.8	1.8	1.8				3	193241125	193277738
53	*IGF2BP3*	10643	-2.9		-2.2		5.4	4.8	2.7				7	23310209	23470467
54	*KCNAB2*	8514			-1.8	-3.4	3.2		3.3		-2.5		1	5991466	6101193
55	*KIF2A*	3796	-3.9	2.3			3.8	3.0	3.6				5	62306162	62537249
56	*LAMP2*	3920	-2.7				-1.9		-1.7	-1.9	-2.2		X	120427827	120469365
57	*LGMN*	5641	-4.3		4.3	3.5		3.9				-2.0	14	92703807	92748702
58	*LIMS1*	3987		2.7	2.2		2.4		2.0		-1.8		2	110446640	110473075
59	*LRBA*	987	-4.1				1.9	3.2	2.0	-2.0			4	150264531	151015727
60	*LSM5*	23658	3.3		1.7	2.1	-2.7		-2.9				7	32485332	32495283
61	*LYST*	1130	-7.7		-2.6	-3.9	1.9		2.5				1	235661041	235883640
62	*MCUR1*	63933	-3.5	2.6			1.9	2.6	1.8				6	13786557	13814568
63	*MLH3*	27030	-3.3	1.9			3.6	3.3	3.9				14	75013764	75051532
64	*MRPS24*	64951		-1.6	1.6		-1.5		-1.6		-1.7		7	43866558	43869893
65	*MYOF*	26509	-7.4			-2.7			-1.7	-2.1		-2.1	10	93306429	93482317
66	*P2RY12*	64805	-2.8	2.5			2.8	2.8	4.2				3	151337380	151384812
67	*PBXIP1*	57326		2.0			2.1	2.5	1.9		-2.1		1	154944076	154956123
68	*PCSK6*	5046	-3.2	2.2			4.0	3.3	2.7				15	101297142	101525202
69	*PDE5A*	8654	-5.8	2.3			1.6	3.3	1.9				4	119494395	119628991
70	*PEBP1*	5037	2.3		-2.1	-1.6	-2.6		-2.1				12	118135858	118145584
71	*PF4V1*	5197	-2.9	2.5			6.0	2.5	5.9				4	73853189	73854155
72	*PLA2G12A*	81579	-3.2	1.8			1.7	3.0	2.6				4	109709989	109730077
73	*PPP1R12A*	4659				2.2	2.2	2.2	2.3		-2.1		12	79773563	79935460
74	*PRKAR2B*	5577	-3.3	3.5			2.7	1.8	4.9				7	107044649	107161811
75	*PRKD3*	23683	-4.3		-2.6		-2.5	-1.5	-1.9				2	37250502	37324808
76	*PTGS1*	5742	-3.4	2.8			4.7	2.5	4.4				9	122370530	122395703
77	*RBCK1*	10616	4.0				2.3	1.8	1.9		-2.5		20	407498	430966
78	*RELL1*	768211	-5.5		2.3	4.6	-2.2		-2.0				4	37590800	37686376
79	*RGS18*	64407	-3.7	2.3			4.3		5.7		-3.5		1	192158457	192185815
80	*RHOBTB1*	9886		3.2		-2.2	1.6	3.2	1.7				10	60869438	61001440
81	*RPL21*	6144		-1.5	-2.1					-2.7	-2.8	-1.8	13	27251309	27256691
82	*RPL22*	6146		-2.0	-1.8		-2.4	-1.6	-2.0				1	6181269	6209389
83	*RUNX3*	864		-1.7	-3.6	-4.0	2.7		2.5				1	24899511	24965121
84	*RYBP*	23429	3.1				2.2	1.7	2.5		-1.8		3	72371825	72446918
85	*SARS*	6301	3.2				-1.7		-1.5	2.0		2.2	1	109213918	109238169
86	*SELP*	6403	-4.5	2.7			2.6	2.6	3.8				1	169588849	169630193
87	*SET*	6418	1.7				-1.6	-1.8	-1.9	-1.7			9	128683424	128696400
88	*SIRPA*	140885			-4.9		1.7	1.9	1.8		-3.4		20	1894167	1940592
89	*TBXA2R*	6915	-3.9	1.7			3.8	4.1	4.2				19	3594506	3606840
90	*TCEA1*	6917	3.2		-1.6	2.1	-1.6		-1.7				8	53966552	54022529
91	*TCF4*	6925	-3.5	-1.6			-2.5	2.0	-2.1				18	55222331	55664787
92	*TMEM59*	9528	3.4				-1.9		-1.8	-1.8	-1.6		1	54026681	54053504
93	*TMSB4X*	7114			2.8	-2.8				-2.1	-3.6	-3.4	X	12975108	12977227
94	*TOB1*	10140			-5.0	-5.3	-1.7	-1.7	-2.4				17	50862223	50867978
95	*TRIM58*	25893	-3.0	3.6			5.0	2.8	5.1				1	247857199	247878205
96	*TRIP6*	7205	-8.4		-4.1			1.8	-1.6	-2.1			7	100867138	100873454
97	*UBL4A*	8266	-2.8	2.2			2.0	2.1	1.7				X	154483717	154486670
98	*XBP1*	7494			-5.6	-4.1	-3.0		-1.9		-1.9		22	28794555	28800597
99	*ABLIM3*	22885		3.5			3.2	3.5	2.7				5	149141483	149260542
100	*ACRBP*	84519		2.7			6.5	3.4	6.9				12	6638075	6647460
101	*ACSBG1*	23205	-1.9				3.7	3.3	3.7				15	78167468	78245688
102	*ACSL1*	2180				-4.0			1.8	-1.8	-2.9		4	184755595	184826818
103	*ACTA2*	59	-9.1		-2.2		-5.4		-5.5				10	88935074	88991390
104	*AK2*	204			-2.7		2.5	2.6	2.6				1	33007940	33080996
105	*AKIRIN1*	79647	4.3				1.6		2.0		-1.5		1	38991223	39006059
106	*ALOX5*	240	-4.0				3.2		2.8			-5.5	10	45374176	45446119
107	*AMFR*	267	2.8				1.8	1.8	1.8				16	56361452	56425538

108	*ANKRD55*	79722	-2.4				4.2	3.9	4.4				5	56099678	56233359
109	*APOBEC3A*	200315			4.5	4.7	4.9		5.0				22	38952741	38992778
110	*AQP10*	89872		4.2			3.4	3.0	3.4				1	154321090	154325325
111	*ARHGAP6*	395		3.1			3.5	3.2	4.0				X	11137543	11665701
112	*ARID3B*	10620	-3.5				2.6	1.5	2.8				15	74541177	74598131
113	*ARID5B*	84159			-2.0	-2.4	-2.7		-1.6				10	61901300	62096944
114	*ARL6IP1*	23204			3.6	4.9	-1.7		-2.0				16	18791667	18801678
115	*ARMCX3*	51566			2.1	1.7	-1.5	1.9					X	101622797	101627843
116	*ASAP1*	50807	3.4				1.5		1.5		-1.5		8	130052104	130443660
117	*ATG14*	22863	-3.9		-2.3	-2.2				-1.7			14	55366392	55411858
118	*ATL2*	64225		-1.7	2.8		-2.3		-2.5				2	38294880	38377285
119	*C10orf54*	64115			-2.2		4.6		4.3			1.9	10	71747559	71773498
120	*C12orf76*	400073		2.2			2.8	2.9	3.2				12	110027028	110073686
121	*C19orf33*	64073	-2.4	3.0			2.6	4.5					19	38304161	38305009
122	*C2orf88*	84281		3.3			4.9	3.1	5.1				2	189879609	190203484
123	*C9orf89*	84270		1.6			2.5	1.8	2.0				9	93096218	93113283
124	*CALM3*	808		2.9			2.6	2.3	2.3				19	46601074	46610793
125	*CAPN3*	825	-2.4			3.0	1.9		2.3				15	42359500	42412318
126	*CASP1*	834			-3.5	-3.1	1.8		2.0				11	105025443	105035250
127	*CAV2*	858	-6.9				-1.8	3.9	-2.4				7	116287380	116508541
128	*CCDC88A*	55704	-3.9				2.2	2.0	2.3				2	55287842	55419921
129	*CCDC92*	80212		1.5			2.3	2.5	2.2				12	123918660	123972831
130	*CCL5*	6352		1.6			3.5	2.2	4.0				17	35871491	35880793
131	*CCT2*	10576	4.8				-2.9	-1.6	-2.9				12	69585334	69601570
132	*CD83*	9308		-1.5	-1.8			1.6	1.6				6	14117256	14136918
133	*CD9*	928		2.8				2.1		-2.6		-2.8	12	6199715	6238271
134	*CDC5L*	988	-2.9				2.9	3.5	3.2				6	44387525	44450426
135	*CEP350*	9857	2.9				3.2	3.4	3.3				1	179954738	180114880
136	*CHMP4B*	128866	-3.5							-1.7	-2.2	-1.5	20	33811304	33854366
137	*CLN8*	2055	-3.0				2.3	1.8	2.3				8	1755778	1786572
138	*CMIP*	80790		1.7			2.7	2.9	2.6				16	81445170	81711762
139	*CNST*	163882		2.1			1.9	2.1	2.4				1	246566444	246668584
140	*CRAT*	1384		1.6				2.3	1.9	-1.8			9	129094810	129111189
141	*CREBRF*	153222			-2.3		2.6	2.1	2.6				5	173056352	173139284
142	*CRIM1*	51232			4.1		-2.0	2.4	-1.6				2	36355926	36551135
143	*CRISPLD2*	83716	-4.8				-1.7				-2.0	-1.7	16	84819984	84920768
144	*CSF2RB*	1439	-1.8				2.0		2.4		-3.6		22	36913628	36940449
145	*CST7*	8530	5.4	3.7			1.8		1.9				20	24949230	24959928
146	*CSTA*	1475	-6.9				2.0			-4.2	-2.5		3	122325244	122341972
147	*CTBS*	1486	-3.9	1.6	-2.3	-2.3							1	84549606	84574480
148	*CTDSP2*	10106	3.3							-2.3	-2.5	-1.7	12	57819927	57846739
149	*CTNND1*	1500	2.8				-1.5	1.5	-1.5				11	57753243	57819546
150	*CTSA*	5476		2.5			2.8		2.3		-1.8		20	45890144	45898820
151	*CTSO*	1519					-2.8	-2.0	-2.3			-1.7	4	155924118	155953917
152	*CTTN*	2017		3.2			4.3	4.6	3.7				11	70398404	70436584
153	*CXCR2*	3579	3.1		-1.5		3.6		4.1				2	218125289	218137253
154	*CYB5A*	1528	4.0		2.5	1.7						-2.3	18	74250847	74292016
155	*CYFIP2*	26999	5.8				2.1		2.8		-1.7		5	157266079	157395595
156	*DAPP1*	27071	-2.7				4.6	1.9	4.5				4	99816833	99870154
157	*DDIT4*	54541			-3.6		-2.0	-2.1	-3.2				12.73239	75013220.2	75087135
158	*DDX11*	1663		2.8			3.0	2.8	2.7				12	31073845	31104791
159	*DENR*	8562				2.1	-1.8	-1.6	-1.7				12	122752774	122771064
160	*DLG1*	1739	-6.2		-3.8		-1.8		-1.9				3	197042560	197299300
161	*DNAH3*	55567	-2.2				1.6	2.0	1.8				16	20933111	21159441
162	*DNAJB6*	10049		1.6						-2.0	-2.2	-1.6	7	157335381	157417439
163	*DNAJC22*	79962	-2.6				1.6	2.6	1.9				12	49346917	49357546
164	*DNM3*	26052		3.2			3.4	3.7	3.9				1	171841498	172418466
165	*DOCK8*	81704	-5.4				1.9		2.6		-3.0		9	214854	465259
166	*ECE1*	1889	-5.9				1.8	1.7	1.8				1	21217247	21345504

167	*EEF1A1*	1915		-1.5	-1.7		-2.5	-1.5					6	73515750	73523797
168	*EFEMP2*	30008	-3.4				-1.6	2.0	-1.9				11	65866441	65873592
169	*EIF2S3*	1968						-1.6		-2.4	-2.2	-2.5	X	24054716	24077971
170	*EIF5*	1983	4.5			2.8	-1.6		-1.7				14	103333544	103345025
171	*ELOVL7*	79993	-5.2				2.6	2.0	1.9				5	60751791	60844389
172	*EPB41L3*	23136	-3.3		-2.7	-1.9			1.7				18	5392381	5630700
173	*EPRS*	2058	-3.1		-2.7		-1.8		-1.7				1	219968601	220046658
174	*FAM107B*	83641			2.5		-1.7	-1.9	-1.7				10	14518557	14774897
175	*FAM209B*	388799	-3.6				3.1	3.1	3.2				20	56533246	56536520
176	*FAM63A*	55793	-4.1				2.2	1.6	2.5				1	150996086	151008375
177	*FBXW2*	26190					2.1	2.3	2.2		-1.5		9	120751978	120793412
178	*FCMR*	9214		-1.8		-8.9		-1.9	1.7				1	206904386	206923247
179	*FDFT1*	2222			1.9	2.2	-1.7		-1.9				8	11795573	11839309
180	*FGD2*	221472	-1.5				2.7	2.1	2.9				6	37005646	37029070
181	*FGFR1*	2260	-5.9				2.0	2.2	2.2				8	38411138	38468834
182	*FLNA*	2316	-5.5		1.6		2.4		2.0				X	154348524	154374638
183	*FRMD3*	257019		3.2			4.2	3.1	4.3				9	83242990	83538546
184	*FXYD5*	53827		-1.8	2.6	2.1	2.2						19	35154730	35169883
185	*GALNT1*	2589			-1.9	-4.1	-1.6		-1.7				18	35581117	35711834
186	*GAS2L1*	10634	-3.1				4.2	3.1	3.3				22	29306582	29312785
187	*GAS7*	8522	-3.9			2.7	1.7		1.8				17	9910609	10198551
188	*GCC2*	9648			-2.4	-2.4	-2.0		-2.0				2	108448561	108509415
189	*GDAP2*	54834	-7.0				2.0	2.4	2.3				1.113757	75028982.8	75102972.76
190	*GFI1B*	8328		3.3			5.1	3.5	5.2				9	132945533	132991687
191	*GFM1*	85476			-1.9	1.5	-2.4		-2.2				3	158644278	158692575
192	*GMPR*	2766	3.5	3.3	-3.4	-4.2							6	16238580	16295549
193	*GNB5*	10681	-4.9				2.5	2.5	2.2				15	52115105	52191369
194	*GP1BA*	2811	-3.3				4.2	3.6	4.4				17	4932297	4935030
195	*GPCPD1*	56261					2.2	1.5	2.1		-1.7		20	5544404	5611026
196	*GPS2*	2874			2.2	2.1	1.7		1.7				17	7311324	7315564
197	*GPSM3*	63940					2.2	1.6	2.6		-1.6		6	32190766	32195523
198	*GPX2*	2877	-2.7				-2.5	2.1	-3.9				14	64939152	64942905
199	*GTF2E2*	2961			2.3	3.7					-1.8	-1.6	8	30578318	30658251
200	*H2AFY*	9555			2.2		1.7		1.7		-1.6		5	135333900	135399914
201	*HACD4*	401494	-2.9	2.4			1.6		2.1				9	20999515	21031636
202	*HAX1*	10456		-1.6			-1.7		-1.5			-2.0	1	154272511	154275875
203	*HIST1H2BC*	8347			-2.8		2.6	2.2	1.9				6	26114873	26123926
204	*HIST1H2BF*	8343		2.0			2.6	2.7	1.5				6	26199520	26200715
205	*HIST1H2BK*	85236		3.3	-5.1		2.2	1.7					6	27146418	27146798
206	*HIST1H3H*	8357		4.3			6.3	2.8	4.8				6	27810064	27811300
207	*HLA-DOA*	3111	-2.4				1.5	2.4	1.9				6	33004178	33009612
208	*HMOX1*	3162		-1.5	-1.6		3.0		4.2				22	35380361	35394214
209	*HSP90AA1*	3320	1.5	-1.7			-1.6		-1.6				14	102080738	102139699
210	*HSP90B1*	7184		-1.9			-1.7		-1.7		-2.8		12	103930107	103953645
211	*HSPA8*	3312		-1.5			-1.7	-1.8	-1.6				11	123057489	123063230
212	*HSPB1*	3315		1.8			-1.9	2.8	-2.4				7	76302544	76304295
213	*ICAM3*	3385	3.4				2.6		2.9		-4.1		19	10333776	10339823
214	*IFITM2*	10581			-3.5	-4.8					-3.6	-1.8	11	307631	315272
215	*IGFBP7*	3490	-7.5				-4.1		-4.1			-2.4	4	57030773	57110385
216	*IKZF1*	10320	3.7	-1.5			3.6		3.9				7	50304124	50405101
217	*IL16*	3603					2.2		2.7		-2.1	-1.6	15	81159575	81314058
218	*IL6R*	3570	-2.9		-3.1		3.2		3.8				1	154405193	154469450
219	*ITGA2B*	3674		3.5			6.4	3.0	6.5				17	44372180	44389505
220	*ITGAL*	3683	-3.1				3.4	-1.9	3.6				16	30472658	30523185
221	*ITGB3*	3690		3.5			4.1	3.6	4.1				17	47253846	47311816
222	*KLF6*	1316	3.0		-2.3	-3.9					-3.5		10	3775996	3785281
223	*LCN2*	3934	-8.5	4.1				2.2	-1.9				9	128149071	128153455
224	*LILRA5*	353514	-3.8				2.3	1.5	2.4				19	54307070	54313139
225	*LIX1L*	128077	-6.1				-1.7	-1.7	-1.6				1	145933423	145958001

226	*LMF1*	64788	-2.6				2.1	2.5	2.3				16	975761	981596
227	*LRP1*	4035	-3.0				2.2	2.0	2.1				12	57128493	57213351
228	*LSP1*	4046					2.7		2.9	-1.9	-2.4		11	1852970	1892267
229	*LYRM2*	57226	-3.3				1.9	2.2	2.1				6	89568144	89638753
230	*LYZ*	4069	-5.0		6.5			-1.5			-3.5		12	69348341	69354234
231	*MAP3K3*	4215	2.8				2.1		2.2		-2.1		17	63622415	63696303
232	*MAST4*	375449	-3.0				2.1	3.7	1.6				5	66596361	67169595
233	*MCL1*	4170	4.4				2.7	2.2	2.6				1	150574551	150579738
234	*MESDC1*	59274				2.3	1.8	1.7	1.6				15	81000944	81005788
235	*METTL22*	79091	-6.0	1.6	-1.7		1.6						16	8621683	8649654
236	*MGLL*	11343	3.6	2.1			3.0	2.9					3	127689062	127823250
237	*MINK1*	50488	3.0				2.1	1.7	1.7				17	4833388	4898061
238	*MMD*	23531		2.6			2.0	1.8	2.5				17	55392613	55421992
239	*MMRN1*	22915	-5.0				1.7	2.9	3.1				4	89879532	89954629
240	*MOB1B*	92597	-4.5	1.5				1.6	2.1				4	70902326	71022449
241	*MPL*	4352	-4.9				4.7	3.8	4.7				1	43337807	43352772
242	*MPP1*	4354		2.5			2.7		3.6		-2.2		X	154778684	154821007
243	*MRPL43*	84545	3.8		3.0	3.4						-1.6	10	100969458	100987515
244	*MRPL46*	26589	3.0			6.2	-2.2		-2.1				15	88459476	88467419
245	*MTG2*	26164	-3.3				2.1	2.0	1.7				20	62183029	62203568
246	*NAAA*	27163	-4.9	-1.8			2.0		2.1				4	75913657	75941051
247	*NAE1*	8883	-3.5		2.3		-2.9		-2.7				16	66802875	66873256
248	*NDST1*	3340	-4.2				1.8	1.6	1.5				5	150485818	150558211
249	*NEDD9*	4739	-3.9		-2.4		-1.9		-1.6				6	11183298	11382348
250	*NEXN*	91624	-3.7	2.1				2.9	1.8				1	77888513	77943895
251	*NKG7*	4818	3.6				2.0	-2.2	1.9				19	51371606	51372715
252	*NME1*	4830					-2.0		-2.2	-1.7		-2.4	17	51153536	51162428
253	*NOL7*	51406			-3.3	-3.9		1.7	1.5				6	13615327	13632739
254	*NOP16*	51491	3.8		3.4	1.6		1.6					5	176383938	176388975
255	*NOP56*	10528	4.0	-1.8			-1.7		-1.7				20	2652145	2658393
256	*NOTCH2NL*	388677		-1.8			1.8		1.9		-1.6		1	146146203	146229026
257	*NRGN*	4900		2.4			6.5	2.9	6.8				11	124739846	124747210
258	*NT5M*	56953		3.3			2.9	2.9	3.0				17	17303335	17347663
259	*NUCKS1*	64710	3.2				-2.1		-1.6			-1.7	1	205712819	205750276
260	*NUDT4*	11163	3.3	1.9	1.5	1.6							12	93377883	93408146
261	*NUP214*	8021	-3.1				1.7		1.7		-1.6		9	131125561	131234670
262	*ORAI2*	80228	-4.2				3.1	2.6	3.1				7	102433106	102456821
263	*PARVB*	29780		2.0			3.4	2.4	3.5				22	43999211	44172949
264	*PCNA*	5111			-3.1	-4.3	-2.3		-3.0				20	5114953	5126626
265	*PDLIM5*	10611		1.9	2.1		-2.1		-1.9				4	94451857	94668227
266	*PEAR1*	375033	-4.8				2.7	2.9	2.6				1	156893698	156916434
267	*PECAM1*	5175	-4.6				2.0	1.5	2.5				17	64319415	64413776
268	*PELI1*	57162	-2.9		-2.1		3.0		2.9				2	64092652	64144454
269	*PF4*	5196		1.9			7.4	1.7	7.6				4	73981077	73982124
270	*PFDN5*	5204		-1.6						-2.4	-2.4	-2.4	12	53295291	53299450
271	*PHC3*	80012	-4.1	-1.8			-1.7		-1.7				3	170086732	170181749
272	*PHF21A*	51317	-2.3				1.9	2.2	2.1				11	45929323	46121178
273	*PNKD*	25953		-1.6			3.0	1.6	2.2				2	218270392	218346793
274	*PPP1R13L*	10848	-3.9		-2.1	-3.6				-1.7			19	45379634	45406349
275	*PPP3CA*	5530			2.1		-1.7	-1.6	-1.8				4	101023409	101348278
276	*PRDX1*	5052		-2.0			-1.7		-1.9			-1.8	1	45511036	45523047
277	*PRF1*	5551	4.5				1.7	-2.8	1.7				10	70597348	70602775
278	*PROSER2*	254427	-5.2				3.5	3.3	3.1				10	11823339	11872277
279	*PSMA5*	5686	4.5	-1.6			-1.6		-2.0				1	109399031	109426427
280	*PSME2*	5721		-1.8	1.8						-1.9	-1.7	14	24143362	24147570
281	*PTCRA*	171558		3.9			3.7	3.8	4.1				6	42915989	42925835
282	*PTP4A1*	7803	3.6		-3.6		-2.7		-2.2				6	63521761	63583587
283	*PTPRC*	5788	2.8				1.9	-1.9	2.7				1	198638671	198757283
284	*PYGB*	5834			-4.1	-3.0	2.2	2.0					20	25248069	25298014

285	*PYGL*	5836	-3.3				1.8		1.6		-2.0		14	50857891	50944736
286	*RAB32*	10981		2.2		-2.9			1.7		-2.2		6	146543693	146554965
287	*RAB34*	83871	-2.9		-4.6				-1.7			-1.7	17	28714281	28718429
288	*RAB7A*	7879					1.6	1.7	1.6		-1.6		3	128726122	128814796
289	*RAD23A*	5886	4.2				2.3	1.8	1.9				19	12945855	12953642
290	*RASSF2*	9770	-3.2		-2.1		2.1		3.1				20	4780023	4823645
291	*RBBP7*	5931	-2.6			2.2	-1.5		-1.6				X	16839283	16870414
292	*RHOF*	54509				-2.1	3.2	2.7	2.4				12.21143	75010136.2	75084036.3
293	*RNF10*	9921					2.0		1.8	-1.9	-1.9		12	120533480	120577594
294	*RNF11*	26994		2.2	-3.1	-3.3			1.7				1	51236271	51273455
295	*RNF144B*	255488	-6.9		-2.8		1.8		2.4				6	18387350	18468874
296	*RNPEPL1*	57140	-5.9				2.0	1.6	1.9				2	240565804	240581372
297	*RPL18*	6141		-1.8						-3.0	-2.3	-2.3	19	48615328	48619536
298	*RPL7A*	6130		-1.8						-2.6	-2.1	-2.1	9	133348214	133351426
299	*RPS11*	6205		-1.6			2.0	2.2	2.1				19	49496365	49499689
300	*RPS14*	6208		-1.5						-3.1	-2.6	-2.6	5	150443190	150449756
301	*RPS16*	6217		-1.6						-1.7	-2.3	-2.1	19	39433207	39435948
302	*RPS19*	6223		-1.7						-1.8	-2.0	-2.1	19	41859918	41872926
303	*RPS4X*	6191		-2.2						-2.6	-2.2	-2.1	X	72255679	72277300
304	*RPS5*	6193		-2.0			-1.8		-1.7			-1.8	19	58386400	58394806
305	*RPS6*	6194		-1.7			-1.6			-2.5	-2.0		9	19375715	19380254
306	*RPSA*	3921		-1.6						-1.5	-1.6	-1.6	3	39406689	39412542
307	*RUFY1*	80230		2.9			4.5	2.7	4.0				5	179550558	179610026
308	*S100A8*	6279	-4.9				4.9	3.0	5.2				1	153390032	153391188
309	*SCARB2*	950	-2.9		2.0		-1.7		-1.5				4	76158737	76213893
310	*SCN11A*	11280	-5.5				2.7	2.7	2.7				3	38845769	38950561
311	*SDC4*	6385	-4.0				-2.7	2.1	-3.2				20	45325288	45348424
312	*SDPR*	8436		3.6			3.9	2.7	5.4				2	191834302	191847255
313	*SEC14L5*	9717	-2.2				3.5	3.5	3.6				16	4958317	5019158
314	*SH3BGRL2*	83699		3.4			3.1	3.7	3.1				6	79631283	79703659
315	*SH3BGRL3*	83442		2.0			2.8		2.3		-1.9		1	26279176	26281522
316	*SLC11A1*	6556	-6.3				4.1	2.1	4.1				2	218382029	218396894
317	*SLC2A3*	6515	-4.2				2.6		2.8		-1.8		12	7919230	7936275
318	*SLCO3A1*	28232	-3.9				2.6	2.1	2.7				15	91853695	92172435
319	*SMCHD1*	23347					2.1	-1.7	2.1		-2.6		18	2655738	2805017
320	*SMIM19*	114926			-2.1	-3.4	-1.8		-1.6				8	42541155	42555193
321	*SMIM5*	643008		4.0			3.3	3.4	2.8				17	75633434	75641404
322	*SMOX*	54498		3.8			3.3	2.7	3.0				12.03778	75009108.2	75083003.41
323	*SOD2*	6648					2.7	2.5	2.6		-3.2		6	159669057	159762529
324	*SPECC1*	92521					2.3	1.8	2.4		-2.4		17	20009344	20319026
325	*SPNS1*	83985	-3.5	-1.7			2.5		2.2				16	28974221	28984548
326	*SRGN*	5552	-3.3				2.6		2.8		-4.7		10	69088106	69104811
327	*SRSF5*	6430	-3.5	-1.6						-1.9	-2.2		14	69726900	69772005
328	*SSX2IP*	117178			-2.0		2.6	3.2	3.2				1	84643707	84690803
329	*ST13*	6767		-1.6		3.7	-1.6		-1.9				22	40824535	40857022
330	*STK17B*	9262	-2.7		2.9		2.6		2.4				2	196133566	196176503
331	*STON2*	85439		2.5			3.8	3.1	3.7				14	81260656	81436465
332	*STXBP2*	6813	4.2			-4.0	2.8		2.6				19	7636881	7647873
333	*TAF15*	8148					2.2	2.5	2.2		-1.9		17	35713791	35864615
334	*TAGLN2*	8407					2.1	2.3			-3.3	-2.2	1	159918107	159925732
335	*TARS*	6897	3.7		-4.2		-2.6		-2.5				5	33440696	33469539
336	*TCAF1*	9747	4.4	-1.8	2.0	2.2							7	143851375	143902198
337	*TECPR2*	9895	-2.8				2.9	2.1	3.0				14	102362963	102502481
338	*TGFB1*	7040		2.0			3.8	2.6	3.7				19	41301587	41353911
339	*THBS1*	7057		1.6			2.8	2.8	2.7				15	39581079	39599466
340	*TIMMDC1*	51300	5.9			4.3	-1.6		-1.9				3	119498532	119525090
341	*TLE3*	7090					3.6	2.1	3.3		-1.7		15	70047790	70098176
342	*TMED10*	10972					-2.0		-1.8	-2.2		-2.2	14	75131470	75176631
343	*TMED8*	283578		1.7			2.0	1.8	2.2				14	77335021	77377109

344	*TMEFF2*	23671	7.7				5.0	4.9	5.0				2	191949043	192195709
345	*TMEM140*	55281		2.1			2.6	2.2	2.5				7	135148072	135166215
346	*TMEM164*	84187	3.4				2.8	2.2	2.4				X	110002631	110182734
347	*TMEM176B*	28959	-3.4	-2.8			-2.2		-2.3				7	150791285	150801360
348	*TMEM40*	55287		2.5			5.3	3.1	3.8				3	12733525	12769457
349	*TMF1*	7110			-3.2	-4.1	-1.5		-2.5				3	69019827	69052303
350	*TNS1*	7145	-5.8				-2.2	2.4	-2.3				2	217799789	218002995
351	*TPP2*	7174		2.1			5.6	4.4	5.6				13	102597003	102679958
352	*TPRKB*	51002	-3.6				-2.0		-2.0		-2.7		2	73729104	73737400
353	*TRAPPC6A*	79090	2.7	-1.7	-2.1	-5.2							19	45162928	45178237
354	*TREML1*	340205		4.1			5.6	3.2	5.8				6	41149342	41154337
355	*TSPAN13*	27075	-3.5		-2.5		-1.7		-1.6				7	16753535	16784536
356	*TSPAN33*	340348	3.2				3.9	3.4	4.5				7	129144892	129169697
357	*TUBA1A*	7846			1.5					-1.7	-2.5	-2.3	12	49184796	49189324
358	*TUBA4A*	7277		2.2	-2.7		3.3		1.5				2	219249711	219278170
359	*TUBB1*	81027		3.5			8.1	2.8	8.3				20	59019254	59026654
360	*TUBB2A*	7280		2.5			-2.3	2.2	-2.1				6	3153669	3157526
361	*TUSC2*	11334				1.6	2.7	3.1	3.2				3	50320027	50328251
362	*TYROBP*	7305	-5.9						1.7		-3.7	-2.3	19	35904401	35908295
363	*UBE2C*	11065		2.0	3.5			1.6	-1.9				20	45812576	45816957
364	*UBL3*	5412	-3.5		-4.2	-4.0	-1.6						13	29764371	29850684
365	*UBR5*	51366	-2.8				-2.8	-1.7	-2.8				8	102253012	102412841
366	*UCHL3*	7347			-4.2	-4.6	-1.7		-1.8				13	75549480	75606020
367	*VEPH1*	79674		1.7			1.9	3.5	3.3				3	157259742	157533619
368	*VWA5B1*	127731	-3.3				2.9	3.1	3.1				1	20290919	20354894
369	*WARS*	7453	-3.4	-1.6	-5.1	-3.2							14	100333788	100376805
370	*WHAMM*	123720		1.8			2.4	1.6	2.0				15	82809628	82836108
371	*WWP2*	11060					2.0	2.1	2.2		-1.6		16	69762306	69941741
372	*XPO6*	23214				-3.6	2.6		2.0		-2.2		16	28097979	28211920
373	*YWHAH*	7533		2.2			1.8	2.3	1.7				22	31944461	31957603
374	*YWHAZ*	7534	1.5				2.2	2.0	1.6				8	100916525	100953388
375	*ZBTB44*	29068	4.1				-2.1	-1.9	-1.9				11	130226677	130314686
376	*ZNF135*	7694	-2.5				1.8	2.1	2.0				19	58059239	58086310
377	*ZNF185*	7739		1.7			4.7	1.9	2.7				X	152914442	152973480
378	*ZNF330*	27309	2.6				-2.2	-1.6	-1.7				4	141220887	141234697
379	*ZNF410*	57862	3.1				-2.7	-1.8	-2.6				14	73886617	73932511
380	*ZNF445*	353274	-4.8				2.3	2.7	2.5				3	44431720	44477670
381	*ZSCAN29*	146050	-5.6				2.1	2.0	2.1				15	43358172	43371025
382	*SEPT6*	23157			2.2		2.2		2.1				X	119615724	119693370
383	*ABCB5*	340273	-5.9		-5.4	-4.8							7	20615207	20777038
384	*ABCC3*	8714	-4.2	2.7				2.1					17	50634777	50692252
385	*ABHD12*	26090			-2.6	-1.7		2.2					20	25294743	25390983
386	*ABLIM1*	3983	-4.0							-2.8	-2.0		10	114431113	114685003
387	*ABTB1*	80325	3.4				2.5		2.4				3	127672935	127680920
388	*ADCY7*	113	4.0				2.5		2.0				16	50246137	50318135
389	*ADGRE2*	30817	-2.8				2.5		2.7				19	14732393	14778541
390	*ADH5*	128	-2.9				-2.1		-1.8				4	99070978	99088801
391	*ADRBK2*	157					3.0	1.8	3.2				22	25564849	25729294
392	*AGPAT1*	10554					3.2	2.9	2.7				6	32168212	32178096
393	*AGTRAP*	57085	-4.5				2.1		1.9				1	11736084	11754802
394	*AIMP1*	9255	2.9				-2.1		-2.2				4	106315544	106349226
395	*AIMP2*	7965					3.7	3.6	3.5				7	6009245	6023834
396	*AKAP12*	9590	-2.6		2.7							-2.3	6	151239999	151358557
397	*ALOX5AP*	241	-3.1								-2.8	-1.8	13	30713478	30764426
398	*AMPD2*	271					1.7		1.6		-2.1		1	109616104	109632051
399	*ANKRD28*	23243					2.7	4.1	3.8				3	15667236	15859771
400	*ANKRD33B*	651746					3.3	3.0	3.3				5	10564330	10657816
401	*ANKRD44*	91526	-4.2				1.6		2.4				2	196967017	197311173
402	*ANKRD9*	122416					3.3	3.4	2.3				14	102501760	102509799

403	*ANXA2*	302		-1.5			-1.6		-2.0				15	60347134	60402883
404	*ANXA3*	306			-3.0		-2.4		-2.9				4	78551519	78610451
405	*ANXA4*	307		-1.6			-3.3		-3.5				2	69644425	69827100
406	*ANXA5*	308					-1.6		-1.6			-1.8	4	121667955	121697113
407	*APMAP*	57136								-1.5	-2.7	-1.8	20	24962925	24992979
408	*APOPT1*	84334					2.0	2.3	2.0				14	103562962	103607523
409	*ARF4*	378	3.2				-1.8		-1.8				3	57571363	57598220
410	*ARFIP1*	27236	-6.3				-1.7		-1.5				4	152687727	152918463
411	*ARHGAP26*	23092	-4.1				1.9		1.7				5	142770384	143229011
412	*ARHGEF2*	9181					2.3	2.1	2.4				1	155946851	156007070
413	*ARL11*	115761	-4.9				1.7		1.7				13	49628299	49633872
414	*ARL2*	402	-3.3			-2.6					-3.9		11	65014113	65022184
415	*ARL4A*	10124			3.1	1.6				-2.3			7	12686856	12690934
416	*ARMC10*	83787	-3.2		-1.6	1.8							7	103074881	103099764
417	*ARRDC3*	57561					-2.2		-2.0		-1.8		5	91368724	91383359
418	*ASCC1*	51008	-2.9		-2.8	-2.9							10	72096032	72217134
419	*ASNS*	440	4.5		-5.2	2.5							7	97852118	97872542
420	*ATF3*	467	4.5	-2.1	-1.6								1	212565334	212620777
421	*ATF6*	22926	-4.7		-3.3		-1.5						1	161766294	161964070
422	*ATG12*	9140					-2.1	-1.9	-2.3				5	115828200	115841858
423	*ATG16L2*	89849	-4.0				3.7		3.9				11	72814308	72843674
424	*ATM*	472	-4.7	-1.8				-1.5					11	108222484	108369102
425	*ATP1A1*	476		-1.6			-2.0		-1.9				1	116372668	116410261
426	*ATP1B1*	481					-3.2	1.8	-3.7				1	169105697	169132722
427	*ATP2A2*	488			-1.7	-3.6			-2.0				12	110280756	110351093
428	*ATP2A3*	489					2.4	2.0	3.3				17	3923870	3964464
429	*ATP5G2*	517		-1.8						-1.9		-2.0	12	53632726	53677408
430	*ATP5G3*	518							-1.6	-2.3		-2.6	2	175176258	175184607
431	*ATP6V0E1*	8992					2.9	2.6	3.1				5	172983757	173035445
432	*ATP6V1D*	51382			-1.9	-2.0	-1.7						14	67294371	67360265
433	*ATP8B1*	5205	-2.7				1.6	1.6					18	57646426	57803101
434	*AURKA*	6790	-5.3		4.1	4.5							20	56369389	56392337
435	*AXIN1*	8312					2.1	1.8	2.0				16	287440	352673
436	*AXL*	558	-6.1		2.8	-2.1							19	41219203	41261766
437	*BANK1*	55024	-6.8				2.4		2.6				4	101411286	102074812
438	*BAZ2A*	11176					2.0	2.0	1.8				12	56595596	56636816
439	*BCAS3*	54828	-5.4		3.3	3.8							1.312169	75028640.1	75102628.46
440	*BCAT1*	586	-9.1				-2.5		-1.8				12	24810022	24949459
441	*BCL7B*	9275	-2.5		-3.8	-1.9							7	73536356	73558002
442	*BCR*	613					2.3	2.3	1.9				22	23179704	23318037
443	*BDP1*	55814					-2.7	-1.8	-2.6				5	71455615	71567820
444	*BEST1*	7439	-3.1		-3.5	-5.0							11	61949821	61965515
445	*BIN2*	51411					3.4		3.9		-3.9		12	51281038	51324668
446	*BMPR2*	659					-3.1	2.3	-2.9				2	202376936	202567751
447	*BOLA2*	552900					1.8	2.4	2.2				16	29443056	29454964
448	*BRCA1*	672	-4.5				1.9	2.0					17	43044295	43170245
449	*BTBD11*	121551	-3.5				2.4		1.6				12	107318413	107659642
450	*BZW1*	9689				3.1	-2.3		-2.3				2	200810594	200827338
451	*C11orf54*	28970	-4.6		-3.7	-4.7							11	93741591	93764749
452	*C12orf75*	387882	4.4	4.9				1.5					12	105235290	105396097
453	*C14orf119*	55017	3.7				-1.8		-1.9				14	23094765	23100462
454	*C15orf39*	56905		-1.9			2.8		2.4				15	75195643	75212169
455	*C17orf89*	284184		-1.5	-3.2	-2.9							17	81239239	81241281
456	*C18orf32*	497661								-2.1	-2.8	-2.3	18	49477250	49487252
457	*C19orf66*	55337	-3.3		-2.5	-3.2							19	10086122	10093252
458	*C1D*	10438				-3.3	-1.7		-1.6				2	68041130	68110948
459	*C1orf162*	128346	3.3				1.9		2.5				1	111473792	111478512
460	*C1QBP*	708					-1.6	-1.8	-1.5				17	5432777	5448830
461	*C2*	717	-2.6				1.8	2.7					6	31897785	31945672

462	*C2CD2L*	9854					2.1	1.9	2.1				11	119102198	119118544
463	*C4orf19*	55286	-3.0		2.3	4.6							4	37453941	37623495
464	*C5AR1*	728	-2.9				2.8		3.4				19	47290023	47322066
465	*C6orf62*	81688	5.0				1.8		1.8				6	24704861	24720836
466	*C9orf72*	203228	-1.6				1.8		2.4				9	27546545	27573866
467	*C9orf91*	203197			-3.0		1.8		1.8				9	114611206	114646422
468	*CABP5*	56344					2.7	2.5	2.8				19	48029953	48044053
469	*CACNG6*	59285					1.5	2.1	1.8				19	53992288	54012669
470	*CAMK1*	8536	-4.2				1.7		2.0				3	9757342	9769992
471	*CAMKK1*	84254					1.7	1.8	2.2				17	3860315	3894891
472	*CAMTA2*	23125					2.6	1.6	2.4				17	4967992	4987652
473	*CAPG*	822	-5.4		2.9	-1.5							2	85394748	85418432
474	*CAPRIN1*	4076					-1.6	-1.5	-1.6				11	34051683	34101156
475	*CARD8*	22900					3.3	2.1	3.2				19	48180770	48255946
476	*CBL*	867					2.3		1.9		-2.1		11	119206276	119308149
477	*CBX4*	8535	3.0		-2.9		1.6						17	79833156	79839429
478	*CCDC69*	26112	-6.1				2.6		3.4				5	151181052	151224145
479	*CCNB1IP1*	57820	-2.8		-3.9	-3.7							14	20311368	20333312
480	*CCND3*	896		1.6			2.7		2.4				6	41934933	42050357
481	*CCNI*	10983	2.5							-1.9	-2.4		4	77047158	77076005
482	*CCR1*	1230					2.3		2.6		-1.6		3	46201709	46208396
483	*CCT6A*	908				2.6	-2.0		-2.0				7	56051630	56063989
484	*CD33*	945	-5.1				2.4		2.9				19	51225064	51243860
485	*CD36*	948					3.5	3.4	4.2				7	80369575	80679277
486	*CD52*	1043		-2.5			1.7		2.4				1	26317957	26320523
487	*CD53*	963	3.4						1.6		-4.3		1	110873154	110899928
488	*CD81*	975			-4.5		-2.0		-1.9				11	2376177	2397419
489	*CDC25B*	994	-3.9	-1.8		-2.5							20	3786772	3806121
490	*CDC42EP3*	10602	-3.4				2.1		2.1				2	37641882	37738468
491	*CDIPT*	10423								-1.9	-2.3	-1.9	16	29858357	29863736
492	*CDKN1A*	1026		1.5	-4.4	-6.4							6	36676460	36687339
493	*CEACAM4*	1089					1.8	1.6	2.0				19	41618971	41627074
494	*CEBPB*	1051			-2.8		2.5		1.9				20	50190734	50192689
495	*CECR1*	51816		-1.9			1.9		2.6				22	17178790	17221989
496	*CES1*	1066			-1.6		1.6		1.8				16	55802851	55833337
497	*CFL2*	1073	-5.1		1.8	-2.1							14	34706769	34714823
498	*CHD9*	80205			2.0		-1.5	2.1					16	53055033	53329150
499	*CHMP1B*	57132	-6.0		-2.2	-3.3							18	11851396	11854449
500	*CHMP3*	51652					-2.0		-1.9		-1.7		2	86503431	86563479
501	*CHMP5*	51510	4.4				-2.0		-2.0				9	33264879	33282069
502	*CHPT1*	56994		1.5	-2.7	-3.3							12	101696947	101744140
503	*CIC*	23152					1.9	1.9	2.0				19	42268537	42295797
504	*CIRBP*	1153					2.9	1.7	3.0				19	1259384	1274880
505	*CKS2*	1164			3.6	2.3				2.2			9	89311198	89316703
506	*CLDN5*	7122					2.0	2.9	2.3				22	19523024	19527545
507	*CLDND1*	56650			2.1		-1.7		-1.7				3	98497912	98523066
508	*CLEC12A*	160364		-1.6			4.2		4.5				12	9951316	9995694
509	*CLEC2D*	29121		-1.9	1.6	2.1							12	9664969	9699555
510	*CLEC7A*	64581	-4.7				2.3		3.1				12	10116777	10130258
511	*CLPTM1L*	81037			-1.6	-2.1			-1.7				5	1317744	1345099
512	*CLU*	1191		4.2				2.7	1.9				8	27596917	27615031
513	*CMTM5*	116173					4.5	3.7	4.6				14	23376808	23379772
514	*CNIH1*	10175					-2.7	-1.8	-2.7				14	54423560	54441431
515	*CNOT7*	29883					-1.5	-1.6	-1.5				8	17224964	17246878
516	*CNPPD1*	27013	2.9							-1.7		-1.6	2	219171897	219178106
517	*COL1A1*	1277	-5.3					2.1	1.7				17	50183289	50201632
518	*COMMD10*	51397			2.2	2.0	-1.8						5	116084991	116412762
519	*COPS2*	9318	3.7				-2.0		-1.8				15	49106068	49155661
520	*COQ7*	10229	-2.4				1.6	2.1					16	19067599	19080095

521	*COTL1*	23406					3.1		2.4		-3.0		16	84565594	84618077
522	*COX7A2L*	9167				3.3	-1.6		-1.5				2	42333546	42425088
523	*COX7C*	1350		-1.8			-1.6		-1.5				5	86617904	86620962
524	*CPNE5*	57699					3.3	2.4	3.4				6	36740775	36840002
525	*CPNE8*	144402			2.4		1.6	1.9					12	38646822	38907430
526	*CR1*	1378	-3.8				3.2		4.0				1	207496147	207640647
527	*CRK*	1398	-3.0				-1.9		-1.6				17	1420689	1463162
528	*CRLS1*	54675	-2.8		-1.9	-2.9							13.71643	75019045.5	75092988.08
529	*CSE1L*	1434	3.8				-1.5		-1.6				20	49046246	49096960
530	*CST3*	1471	-5.0		-3.7	-2.8							20	23626706	23638473
531	*CTDSPL*	10217		3.3			2.0	3.0					3	37861960	37984469
532	*CTSK*	1513	-2.3		-2.7	-9.0							1	150796208	150808323
533	*CTSL*	1514			-1.7	-3.4						-1.7	9	87725519	87731393
534	*CTSS*	1520					1.6		2.0		-3.4		1	150730196	150765957
535	*CX3CR1*	1524						-1.8	2.2		-2.9		3	39263494	39281735
536	*CXCR4*	7852	-3.1	-2.0				-1.9					2	136114349	136118165
537	*CXorf56*	63932	-5.5		1.7	3.1							X	119538149	119565408
538	*CYB5R1*	51706					1.6	2.0	1.6				1	202961869	202967280
539	*CYB5R4*	51167			-1.7	-4.6		-1.6					6	83859643	83967424
540	*CYBRD1*	79901	-2.6				-2.8		-2.4				2	171522247	171558133
541	*DAD1*	1603					-1.5		-1.6			-1.6	14	22564905	22589269
542	*DAP*	1611	3.3		-3.0	-3.3							5	10679230	10761272
543	*DAZAP2*	9802								-1.8	-3.6	-1.7	12	51238292	51271362
544	*DBI*	1622	3.2		1.7		-1.5						2	119366921	119372560
545	*DCAF13*	25879				5.2	-3.1		-3.0				8	103414714	103443453
546	*DCAF5*	8816	3.8				2.0		1.6				14	69050881	69153150
547	*DCLRE1A*	9937					2.4	3.1	2.6				10	113834725	113854383
548	*DDX21*	9188					-1.7	-2.8	-2.9				10	68956128	68985073
549	*DDX39A*	10212		-1.5	3.0	1.6							19	14408819	14419383
550	*DDX50*	79009					-2.5	-1.6	-2.2				10	68901278	68946847
551	*DENND3*	22898	-4.3				2.4		2.2				8	141117278	141195808
552	*DESI2*	51029	-4.5				-2.6		-2.7				1	244652935	244709033
553	*DHRS4*	10901	-5.3		-1.6	-3.0							14	23953586	23969279
554	*DIAPH1*	1729			3.1	1.5	2.1						5	141515016	141619055
555	*DNAJC16*	23341					1.7	1.7	1.7				1	15526813	15592379
556	*DNAJC5*	80331	3.5				2.7		2.1				20	63895182	63936031
557	*DOCK2*	1794					2.7		3.3		-2.4		5	169637247	170083382
558	*DPEP2*	64174	-4.3				3.7		4.4				16	67987394	68000586
559	*DUS1L*	64118			-2.5	-1.6						1.6	17	82057506	82065887
560	*DUSP22*	56940	-3.1				1.7		2.2				6	291630	351355
561	*DUT*	1854	-2.9	-2.1					-1.7				15	48331011	48343373
562	*DYNC1I2*	1781					-2.1		-2.0			-1.5	2	171687409	171748420
563	*DYNC1LI2*	1783					-3.0		-3.0	-1.5			16	66720893	66751798
564	*E2F3*	1871	-3.0				2.4		2.0				6	20401906	20493715
565	*ECT2*	1894	-3.2				-1.6		-3.6				3	172750682	172821474
566	*EEF1D*	1936					2.0	2.4	2.3				8	143579697	143599541
567	*EHD3*	30845					3.8	2.8	3.9				2	31234337	31269447
568	*EI24*	9538		-1.6			-1.5		-1.7				11	125569216	125584687
569	*EIF2AK1*	27102		2.4			2.0	1.5					7	6022244	6059230
570	*EIF2S2*	8894	4.6				-1.6		-1.8				20	34088298	34112332
571	*EIF4G1*	1981	2.8				1.9	1.7					3	184314495	184335358
572	*ELMO1*	9844					2.1	2.0	2.2				7	36854361	37449249
573	*EMB*	133418	2.4			-3.9					-2.6		5	50396192	50443248
574	*EMC3*	55831		1.9				2.0			-1.7		3	9962537	10011116
575	*EMC7*	56851					2.3	2.5	2.3				15	34084017	34101948
576	*EMILIN2*	84034	-2.3				2.0		1.9				18	2847030	2915993
577	*ENDOD1*	23052					2.7	2.9	2.1				11	95089810	95132645
578	*ENGASE*	64772	-4.0				1.9		1.9				17	79074939	79088599
579	*EPOR*	2057					2.1	2.0	2.1				19	11377205	11384342

580	*EPS15L1*	58513					2.2	1.8	1.7				19	16355239	16472085
581	*ERICH1*	157697					2.4	1.6	2.1				8	614746	738106
582	*ESYT1*	23344	-4.0		-2.2	-2.0							12	56118250	56144671
583	*EZR*	7430		-1.9	2.5	3.2							6	158765741	158819412
584	*FAM110A*	83541					4.1	1.9	3.6				20	833715	857463
585	*FAM120A*	23196	3.6				1.7		1.6				9	93451722	93566107
586	*FAM134C*	162427	-3.6		2.4							-2.0	17	42579513	42610623
587	*FAM168B*	130074	4.2		-2.4	-3.9							2	131047876	131093460
588	*FAM177A1*	283635			3.0		-2.1		-2.2				14	35044907	35113130
589	*FAM26F*	441168	-2.9				2.4		2.4				6	116461370	116463779
590	*FAM35A*	54537		-1.8			-2.1		-1.7				12.67451	75012877.5	75086790.7
591	*FAM78A*	286336	-2.7				2.2		2.3				9	131258076	131276547
592	*FAM91A1*	157769	-4.5				-1.5		-1.6				8	123768456	123815452
593	*FBXO25*	26260		-1.9	-1.7	-1.5							8	406428	477967
594	*FCGR3B*	2215	-3.6				1.8		2.0				1	161623196	161631963
595	*FGD3*	89846	2.8				3.9		4.3				9	92947451	93036236
596	*FGL2*	10875	-3.5						2.3		-3.7		7	77193371	77199826
597	*FGR*	2268	-4.5				5.0		5.1				1	27612064	27635277
598	*FKBP1B*	2281	2.8	2.3				2.5					2	24049701	24063681
599	*FKBP3*	2287	4.2				-2.1		-2.1				14	45115600	45135319
600	*FKBP5*	2289	-2.9	2.2				-1.7					6	35573585	35728583
601	*FKSG49*	400949					2.3	2.4	2.2				5	_	_
602	*FN1*	2335	-8.8			-1.6						-4.3	2	215360440	215436172
603	*FNDC3A*	22862			-3.0		-2.5		-2.0				13	48975912	49209779
604	*FNTA*	2339	4.1						-1.8			1.7	8	43034194	43085788
605	*FOS*	2353	3.4		-3.1	-3.4							14	75278774	75282230
606	*FOXN3*	1112	3.5							-2.9	-1.7		14	89124871	89619149
607	*FRMD8*	83786	-3.3		-2.3	-3.9							11	65386599	65413525
608	*FRYL*	285527			2.8					-2.1	-1.8		4	48497361	48780322
609	*FYB*	2533					2.5		3.0		-1.7		5	39105236	39274528
610	*FYN*	2534					2.5		2.6		-2.2		6	111660332	111873452
611	*GAB3*	139716	-5.0				2.3		3.0				X	154675249	154751583
612	*GABARAP*	11337				-1.9					-2.8	-1.7	17	7240014	7242770
613	*GATAD1*	57798		-1.9	1.7	3.7							7	92447453	92458836
614	*GATAD2A*	54815					2.3	1.5	1.8				2.10582	75027269.4	75101251.26
615	*GCA*	25801					1.9		2.4		-3.4		2	162318840	162371595
616	*GCLM*	2730	-5.4					1.6	2.0				1	93885205	93909456
617	*GIMAP1*	170575					2.8	-1.6	3.1				7	150716557	150724284
618	*GK*	2710				-2.0	1.5		1.8				X	30653359	30731456
619	*GLRX2*	51022	3.3		-3.4	-3.6							1	193096468	193106114
620	*GMNN*	51053					-3.1	1.6	-2.1				6	24774931	24786099
621	*GNE*	10020	-7.0		1.6	2.8							9	36214441	36277056
622	*GNG10*	2790	4.6								-3.7	-1.5	9	111661335	111670246
623	*GOLGA4*	2803	2.9				-1.8		-1.6				3	37243177	37366751
624	*GOLIM4*	27333				-3.9	-2.1		-2.0				3	168008677	168095975
625	*GP5*	2814					3.4	3.1	3.4				3	194394821	194398354
626	*GP9*	2815					4.7	4.0	4.9				3	129060767	129062406
627	*GPR65*	8477	-3.3		2.3				1.5				14	88005124	88014811
628	*GRINA*	2907	4.0		-2.4	-3.8							8	143990058	143993415
629	*GRK5*	2869					2.5	1.7	2.3				10	119207589	119459742
630	*GSK3B*	2932	3.9				2.1			-1.6			3	119821323	120094417
631	*GSTM3*	2947	-3.4		-3.9	-4.2							1	109733932	109741038
632	*GTF2I*	2969								-2.0	-1.9	-1.9	7	74657667	74760692
633	*GTPBP2*	54676					2.1	1.5	1.8				13.77431	75019388.2	75093332.38
634	*GUCY1A3*	2982	2.9				-1.6	3.2					4	155666711	155732349
635	*GUCY1B3*	2983	-3.0	2.8				2.0					4	155758992	155807591
636	*GUK1*	2987			1.9				-1.8			-1.8	1	228139962	228148984
637	*GZMB*	3002	4.8				1.7	-2.2					14	24630954	24634267
638	*H1F0*	3005	-3.9		-3.3			2.8					22	37805093	37807436

639	*H2AFJ*	55766		2.0	-2.2			1.7					12	14774383	14778002
640	*HAT1*	8520	-3.6				-1.9		-1.7				2	171922448	171983682
641	*HCK*	3055	-3.8				3.0		3.3				20	32052188	32101856
642	*HCLS1*	3059					1.6		2.1		-4.4		3	121631399	121660927
643	*HDAC1*	3065	3.3	-1.5							-1.9		1	32292086	32333635
644	*HEXIM2*	124790					2.4	2.5	2.5				17	45160700	45170040
645	*HGD*	3081					2.9	4.0	2.9				3	120628173	120682571
646	*HINT3*	135114				2.3	1.6		2.3				6	125956781	125980244
647	*HIPK2*	28996					3.7	2.0	3.9				7	139561570	139777778
648	*HIST1H2AG*	8969					5.4	4.2	5.0				6	27133042	27135291
649	*HIST1H2BD*	3017			-5.0		3.0	3.1					6	26158146	26171349
650	*HIST1H2BG*	8339					3.6	1.7	3.5				6	26215159	26216692
651	*HJURP*	55355	-3.9		3.8	4.6							2	233833416	233854566
652	*HMG20B*	10362	2.7				1.8	1.8					19	3572777	3579088
653	*HMHA1*	23526	-2.9				4.2		4.0				19	1065923	1086628
654	*HMMR*	3161			5.3	3.0						1.7	5	163460203	163491945
655	*HNMT*	3176	-1.6				-2.3		-1.9				2	137964020	138016364
656	*HNRNPA1*	3178	2.7	-1.7						-1.7			12	54280193	54287088
657	*HNRPDL*	9987		-2.0	1.6		-1.8		-1.9				4	82422564	82430408
658	*HPSE*	10855	-4.7				2.2		2.4				4	83292461	83335153
659	*HRAS*	3265	3.1		-2.4	-4.6							11	532242	537287
660	*HSD17B11*	51170			3.0	1.8					-4.3		4	87336610	87391386
661	*HSP90AB1*	3326		-2.0			-1.7		-1.9				6	44246166	44253888
662	*HTATSF1*	27336	-3.5				-1.8		-1.7				X	136497079	136512346
663	*HVCN1*	84329		-1.9			1.7		2.0				12	110627841	110704950
664	*IDH1*	3417			-2.2		-1.9		-2.0				2	208236227	208266074
665	*IDH3A*	3419	-7.5				-2.0		-1.9				15	78131498	78171949
666	*IFRD1*	3475					2.2	1.8	2.5				7	112422968	112481017
667	*IGBP1*	3476	-3.9							-2.2	-2.1		X	70133449	70166324
668	*IGF2R*	3482	-2.0				2.7		2.4				6	159969099	160113507
669	*IGFBP5*	3488	-4.3								-1.8	-2.0	2	216672105	216695525
670	*IKBKG*	8517					2.0	1.8	2.3				X	154541199	154565046
671	*IL10RA*	3587		-2.1			2.3		2.8				11	117986348	118001483
672	*IL12RB1*	3594	-6.7				3.5		3.3				19	18058995	18098944
673	*IL15*	3600					2.0	1.8	2.1				4	141636599	141733987
674	*IL17RA*	23765					3.3		3.7		-1.7		22	17084954	17115694
675	*INF2*	64423					3.1	3.5	2.5				14	104689606	104722535
676	*IRAK3*	11213	-6.9				3.1		3.2				12	66188879	66254622
677	*IRAK4*	51135	-4.3				1.6		1.7				12	43758944	43789543
678	*IRF5*	3663		-2.0			2.3		2.2				7	128937612	128950035
679	*IRX3*	79191	2.5							-2.3		-2.4	16	54283304	54286763
680	*ITGAX*	3687					3.0		3.2		-1.9		16	31355134	31382997
681	*ITGB1*	3688			1.9		-1.7		-2.0				10	32900319	33005792
682	*ITGB2*	3689	5.1				3.1		3.3				21	44885953	44931989
683	*ITGB7*	3695		-1.8			2.7		2.3				12	53191318	53207307
684	*ITM2B*	9445								-3.7	-4.7	-2.4	13	48233158	48270357
685	*ITPKB*	3707	-3.5		-3.8	-3.1							1	226631690	226739323
686	*JAK3*	3718	-4.5				1.8		1.8				19	17824780	17848071
687	*JAM3*	83700	-8.1	2.4				3.1					11	134068925	134152001
688	*JARID2*	3720	-3.3				2.8		3.0				6	15246296	15522040
689	*JKAMP*	51528	-4.8				-1.6		-1.8				14	59484443	59505410
690	*JMJD1C*	221037					-2.1		-1.5		-2.0		10	63167221	63521850
691	*KARS*	3735					-1.9	-2.0	-2.2				16	75627474	75648643
692	*KDM2B*	84678	-6.6				1.9		1.8				12	121429097	121581015
693	*KDM4B*	23030					3.5	2.1	3.1				19	4969113	5153595
694	*KDM6B*	23135					2.4	1.9	2.8				17	7839904	7854796
695	*KIAA0513*	9764	-3.3				2.4		2.7				16	85027751	85094230
696	*KIAA1033*	23325	2.5		-2.5	-3.0							12	105107324	105169134
697	*KIF3C*	3797					2.3	2.8	2.4				2	25926596	25982749

698	*KLHL6*	89857					2.6	1.9	2.5				3	183487531	183555689
699	*KPNA3*	3839					1.8	1.6	1.9				13	49699307	49792921
700	*KTN1*	3895			1.9		-1.5		-1.7				14	55559072	55701526
701	*LAT2*	7462	-4.5				2.2		2.6				7	74199652	74229834
702	*LCP1*	3936	1.9						1.9		-4.5		13	46125920	46211871
703	*LDLRAP1*	26119		1.9			2.7		2.9				1	25543580	25568886
704	*LEPROT*	54741	-3.9	2.5								-1.7	5.280423	75021786.8	75095742.48
705	*LGALS1*	3956	-5.9		2.0				-1.6				22	37675608	37679806
706	*LGALS9*	3965	-3.3	-1.7			1.7						17	27629798	27649560
707	*LILRB2*	10288					4.6		4.8		-2.3		19	54216278	54223506
708	*LILRB4*	11006	-1.8				1.7		1.8				19	54643889	54670359
709	*LMNB1*	4001		2.4			2.2		1.8				5	126776623	126837020
710	*LOXL3*	84695					2.8	2.2	2.5				2	74532414	74555690
711	*LPAR5*	57121					2.9	2.8	2.2				12	6618835	6636447
712	*LRG1*	116844	-5.6				2.0		2.2				19	4536409	4540474
713	*LRRC40*	55631					-3.0	-1.9	-2.5				1	70144805	70205620
714	*LST1*	7940	-3.4				3.1		3.7				6	31586124	31588909
715	*LTBP1*	4052	-4.9	2.6				2.6					2	32946972	33399509
716	*LYN*	4067					2.9		2.7		-2.3		8	55879813	56014168
717	*LYPLA2*	11313	-6.5		2.9	-2.8							1	23790970	23795539
718	*MAD2L1*	4085	3.4		3.0	1.6							4	120055608	120067074
719	*MALSU1*	115416				3.6	-1.7		-1.7				7	23298739	23311729
720	*MAP1B*	4131	3.4		-1.9	-3.1							5	72107234	72209570
721	*MAP2K3*	5606	3.9				1.9		1.9				17	21284672	21315240
722	*MAP4*	4134					-1.5	1.8	-1.6				3	47850690	48089272
723	*MAP4K1*	11184	-4.1				1.9		2.0				19	38587641	38618882
724	*MAP4K2*	5871					2.3	1.8	2.3				11	64784914	64803241
725	*MAPK1*	5594					3.0		3.0	-2.2			22	21754500	21867680
726	*MAPK7*	5598	-2.9				2.2		2.0				17	19377721	19383544
727	*MAT2B*	27430	-2.7		2.2						-2.1		5	163503114	163519336
728	*MATR3*	9782				1.9	-1.7		-2.0				5	139273752	139331677
729	*MAX*	4149					3.3	3.1	3.3				14	65006174	65102695
730	*MBNL3*	55796					2.3	2.3	2.4				X	132369317	132489968
731	*MBP*	4155			2.1		2.2		2.3				18	76978827	77133683
732	*MCM3*	4172	2.9	-2.0	1.6								6	52264009	52284881
733	*MCTS1*	28985				2.1	-2.6		-2.6				X	120594010	120621074
734	*ME2*	4200	-3.4				1.9		2.1				18	50879049	50954257
735	*MED1*	5469	-7.4		-2.2	-1.7							17	39404285	39451286
736	*MED16*	10025					3.0	1.9	2.5				19	867630	893218
737	*MED4*	29079					-2.3	-1.7	-2.2				13	48053323	48095131
738	*MEF2D*	4209					1.6	2.0	1.9				1	156463727	156500828
739	*MEPCE*	56257					1.9	1.7	1.6				7	100428790	100434126
740	*MESDC2*	23184	-3.8				-1.5		-1.7				15	80946289	80989878
741	*METAP2*	10988	4.0				-2.1		-1.6				12	95473520	95515839
742	*METTL5*	29081	3.5				-2.1		-2.3				2	169810081	169824931
743	*MGAT4A*	11320	-3.6				2.7		2.8				2	98619106	98731126
744	*MGRN1*	23295					2.3	1.8	2.0				16	4616493	4690974
745	*MGST1*	4257				-3.6	-2.4		-2.1				12	16347142	16609259
746	*MGST2*	4258			-5.0					-2.3		-1.8	4	139665768	139740745
747	*MICAL1*	64780	-2.9				2.8		2.7				6	109444062	109465968
748	*MIDN*	90007					3.0	2.4	2.9				19	1248553	1259140
749	*MKNK2*	2872			-2.4		1.7		1.6				19	2037465	2051244
750	*MLANA*	2315	-4.7		-3.1	-3.5							9	5890802	5910606
751	*MME*	4311				-2.1	3.3		3.4				3	155024124	155183729
752	*MORF4L2*	9643	3.1				-2.1		-2.0				X	103675496	103688158
753	*MPC2*	25874			1.6		-1.9		-1.6				1	167916729	167937040
754	*MPEG1*	219972		-2.0			2.4		3.2				11	59208510	59212951
755	*MPZL3*	196264					3.3	2.3	2.9				11	118226690	118252350
756	*MRPL1*	65008	-3.9		-2.4	-3.1							4	77862520	77952790

757	*MRPL48*	51642	-5.7				-2.1		-2.0				11	73787316	73865133
758	*MRPS25*	64432	3.3							-2.3		-1.7	3	15042460	15065335
759	*MT1X*	4501		2.7	-4.9	-3.2							16	56682424	56684196
760	*MTAP*	4507	-4.2		3.6	3.4							9	21802543	21937651
761	*MT-ATP6*	4508					1.6	2.1	1.7				MT	8527	9207
762	*MTF1*	4520					1.8	1.5	1.8				1	37809567	37859620
763	*MTHFD2*	10797			-3.9		-2.0		-1.8				2	74198562	74217565
764	*MTHFR*	4524					2.7	1.5	2.8				1	11785723	11806920
765	*MTMR9*	66036	-6.4		1.7	-1.6							8	11284416	11328146
766	*MT-ND6*	4541					5.0	3.2	5.1				MT	14149	14673
767	*MVP*	9961	-2.7		2.2		1.8						16	29820394	29848039
768	*MYEF2*	50804	-4.9		-4.6	-5.2							15	48134631	48178517
769	*MYL12B*	103910	3.7		1.6						-1.8		18	3261909	3278284
770	*MYL9*	10398	-4.9	4.1				3.4					20	36541484	36551447
771	*MYLK*	4638	-4.8	3.5				2.0					3	123610049	123884331
772	*MZT2B*	80097			-1.6	-3.1			-1.5				2	130181737	130190729
773	*NADK*	65220					2.7		2.6		-2.8		1	1751232	1780457
774	*NADSYN1*	55191	3.0		-3.9		1.8						11	71453109	71524107
775	*NAGPA*	51172	-2.4				2.1		1.8				16	5024844	5034141
776	*NAP1L1*	4673					1.5	2.0	2.0				12	76036587	76085033
777	*NARF*	26502	-2.8		-3.7	-4.9							17	82458180	82490537
778	*NBPF10*	100132406		-2.0			2.1		2.2				1	146064699	146144942
779	*NBPF14*	25832		-1.9	1.6	1.6							1	148531385	148679751
780	*NCAPG*	64151	-2.8		3.4	2.5							4	17810902	17844862
781	*NDUFB5*	4711	-2.8				-1.7		-1.5				3	179604690	179627647
782	*NDUFS6*	4726								-1.6	-2.0	-1.7	5	1801400	1816605
783	*NDUFV1*	4723		-1.6	-2.7	-5.5							11	67606852	67612535
784	*NEB*	4703	-3.8							-2.2	-3.3		2	151485336	151734487
785	*NENF*	29937					1.9	2.4	2.1				1	212432887	212446379
786	*NFAM1*	150372	-2.1				1.7		1.9				22	42380410	42432395
787	*NFKBIB*	4793	-4.5		-2.5	-5.4							19	38899700	38908893
788	*NGFRAP1*	27018		3.4				1.7				-1.6	X	103376340	103378077
789	*NID1*	4811	-1.7				-2.3		-2.3				1	235975830	236065162
790	*NIN*	51199					2.8	1.6	2.9				14	50719763	50831121
791	*NIPAL1*	152519					2.2	2.4	2.3				4	47914142	48040173
792	*NIPSNAP1*	8508	2.6		-2.7	-4.2							22	29554808	29581337
793	*NIPSNAP3A*	25934					-2.6	-1.5	-2.0				9	104747688	104760122
794	*NLK*	51701					2.1	2.4	2.2				17	28041737	28196381
795	*NLRC4*	58484		2.9			1.6		1.9				2	32224453	32265854
796	*NLRC5*	84166	5.6				2.6		1.7				16	56989485	57083531
797	*NLRP12*	91662	-6.5				2.2		2.3				19	53793603	53824394
798	*NMD3*	51068					-3.1	-1.6	-2.9				3	161104696	161253532
799	*NOL11*	25926			-3.8		-2.4		-2.1				17	67717833	67744531
800	*NOLC1*	9221					-2.7	-2.0	-2.5				10	102152176	102163871
801	*NPRL3*	8131					1.5	1.7	2.0				16	84271	138860
802	*NR2C2*	7182	5.1							-1.7	-1.5		3	14947584	15053600
803	*NSG1*	27065	-3.5		-4.1	-2.5							4	4348140	4419058
804	*NTNG2*	84628					2.9	2.6	2.9				9	132161676	132244534
805	*NUBPL*	80224	-3.0	-1.6	-2.4								14	31489956	31861224
806	*OAS2*	4939	-3.0	-1.6			2.2						12	112978395	113011723
807	*OAT*	4942	-4.0				-1.8		-1.6				10	124397303	124418976
808	*OCIAD1*	54940					-1.9	1.6	1.5				6.824242	75042346.7	75116400.42
809	*ODF2L*	57489	-3.4		-3.0	-2.1							1	86346824	86396342
810	*OPHN1*	4983	-4.7					2.0	1.5				X	68042344	68433913
811	*ORC4*	5000	-3.1				-1.5		-1.7				2	147930397	148021604
812	*ORMDL2*	29095	3.5				-1.9		-2.3				12	55817919	55821879
813	*OSTC*	58505	4.4				-3.0		-2.8				4	108650584	108667820
814	*OTUD1*	220213					2.4	1.9	2.4				10	23439458	23442390
815	*OVOS2*	144203					3.0	4.2	3.6				12	31111652	31206154

816	*PAK2*	5062						-1.7			-2.6	-1.8	3	196739857	196832647
817	*PBK*	55872			4.4	3.6						1.5	8	27809620	27838095
818	*PBX2*	5089					1.8	1.8	1.8				6	32184741	32190186
819	*PCBP2*	5094	3.2							-1.5	-2.2		12	53452102	53481162
820	*PCED1A*	64773	2.8		-2.6	-2.0							20	2835314	2841190
821	*PCGF5*	84333	-3.1				2.7		2.8				10	91163012	91284331
822	*PCMT1*	5110	-2.7		3.0	1.6							6	149749443	149811420
823	*PDCD6*	10016			-4.0	-4.1			-1.7				5	271621	353856
824	*PDLIM1*	9124		3.2	-1.9			2.4					10	95237572	95291024
825	*PDZK1IP1*	10158		2.7			1.6	4.9					1	47183593	47191044
826	*PEA15*	8682		-1.8			-1.6		-1.6				1	160205337	160215376
827	*PFDN4*	5203				2.8	-2.3		-2.4				20	54207847	54228052
828	*PGS1*	9489			-1.9		1.8		1.6				17	78378640	78425114
829	*PHC1*	1911	-4.7	-1.8				1.5					12	8913896	8941467
830	*PIK3CD*	5293	-3.8				3.5		3.6				1	9651732	9729114
831	*PIK3R5*	23533	2.8				2.3		2.7				17	8878911	8965712
832	*PIN4*	5303		-1.8	1.6	4.2							X	72181353	72302926
833	*PIP4K2A*	5305					2.4		2.7		-2.2		10	22534849	22714555
834	*PITPNA*	5306					2.0	1.6	1.8				17	1517718	1562816
835	*PITPNC1*	26207	-3.4		-1.6	-1.9							17	67377281	67697261
836	*PJA2*	9867					-2.4	-1.6	-1.9				5	109334709	109409994
837	*PLAC8*	51316	-3.6	-1.8				-1.8					4	83090048	83137075
838	*PLEKHA5*	54477	-5.1		-3.1	-3.9							11.74836	75007394.9	75081281.91
839	*PLEKHG2*	64857	-4.3					1.8	1.6				19	39412585	39428415
840	*PLP2*	5355			3.7					-2.1	-1.9		X	49171926	49175239
841	*PMM1*	5372	3.6		3.3	4.7							22	41576894	41589890
842	*POLD3*	10714					2.2	2.6	2.2				11	74493851	74669117
843	*PPA1*	5464		-1.5			-2.3		-2.3				10	70202830	70233911
844	*PPDPF*	79144	2.9	1.5				1.9					20	63520724	63522206
845	*PPM1F*	9647	-3.2				1.8		1.8				22	21919420	21952837
846	*PPM1L*	151742					2.6	2.3	2.7				3	160755602	161078907
847	*PPP1R10*	5514	4.0		-3.0							-2.0	6	30600400	30618612
848	*PPP2R5A*	5525	-3.2		-4.1	-3.9							1	212285537	212361863
849	*PPP6R1*	22870	2.8				1.9		1.7				19	55229780	55258995
850	*PPP6R3*	55291								-1.7	-2.2	-1.5	11	68460731	68615334
851	*PRKCD*	5580					3.1		2.9		-2.0		3	53156009	53192717
852	*PRKX*	5613	-3.3				2.5		2.6				X	3604370	3713608
853	*PRPS2*	5634			2.4		-2.3		-2.1				X	12791355	12824222
854	*PRRC1*	133619	-5.2				-2.8		-2.7				5	127517609	127555089
855	*PRRC2B*	84726		-1.8			2.0		1.9				9	131394093	131500197
856	*PRRG4*	79056	-7.1		2.5					-1.7			11	32829943	32858123
857	*PRUNE2*	158471	-4.9		-2.1	-3.7							9	76611376	76906087
858	*PSEN2*	5664	-2.5							-1.7	-3.0		1	226870184	226896105
859	*PSMC6*	5706					-2.1		-2.0			-1.7	14	52707172	52728587
860	*PSMD10*	5716				2.2	-2.7		-2.7				X	108084207	108091618
861	*PSMD4*	5710	4.2					-1.8	-1.8				1	151254703	151267479
862	*PSMG2*	56984	3.1				-1.7		-1.6				18	12658043	12725740
863	*PTPN18*	26469					3.0	2.0	3.0				2	130356007	130375409
864	*PTPN21*	11099	-6.1		2.3						-3.1		14	88465778	88554733
865	*PTPRE*	5791					1.6		1.7		-2.5		10	127907061	128085855
866	*PTPRJ*	5795			-1.8		4.2		4.2				11	47980558	48170841
867	*PTPRS*	5802	-7.4					2.8	1.9				19	5158495	5340803
868	*PTRF*	284119	-3.0							-1.7	1.6		17	42402452	42423517
869	*PTRH2*	51651	-5.9	-1.5			-1.6						17	59674636	59707626
870	*PTS*	5805	2.9				-1.7		-1.7				11	112226365	112269955
871	*PTTG1*	9232	-3.2		2.5	1.6							5	160421822	160428744
872	*PWP1*	11137	3.2				-2.2		-2.3				12	107685732	107713167
873	*PXK*	54899					2.6	1.6	2.7				7.341176	75037206.7	75111235.94
874	*PYCR2*	29920					2.8	2.5	2.5				1	225919877	225924340

875	*R3HDM4*	91300	2.9				2.7		2.3				19	896503	913245
876	*RAB13*	5872					-2.1	1.9	-2.2				1	153981617	153986358
877	*RAB24*	53917					2.0		2.1			-1.5	5	177301198	177303744
878	*RAB27A*	5873	-5.1				2.0		2.3				15	55202966	55319113
879	*RAB2A*	5862					-2.2		-2.3		-2.1		8	60516857	60623627
880	*RAB37*	326624					5.4	2.6	5.4				17	74670578	74747335
881	*RAB3D*	9545	-4.9		-1.6	-2.2							19	11322046	11346270
882	*RAC2*	5880					2.8		2.3		-3.5		22	37225261	37244448
883	*RAD21*	5885	2.8				-1.5		-1.7				8	116845935	116874866
884	*RAN*	5901		-1.6			-2.0		-2.3				12	130871879	130877678
885	*RAP1A*	5906	-3.4					1.5	2.0				1	111542218	111716691
886	*RAP2B*	5912			-1.9		2.3		1.8				3	153162270	153167173
887	*RAPGEF2*	9693					1.9	2.1	1.9				4	159104178	159360169
888	*RARS2*	57038	3.1				-2.2		-1.9				6	87514378	87590003
889	*RASA3*	22821	-5.2				2.9		2.4				13	113977783	114132611
890	*RASAL3*	64926	2.4				1.6		1.9				19	15451624	15464571
891	*RASGRP2*	10235					4.6	1.6	4.8				11	64726911	64745456
892	*RBM28*	55131	-2.6		1.6	3.1							7	128297685	128343908
893	*RBPJ*	3516			3.0	1.9	-1.5						4	26163455	26435131
894	*RCAN1*	1827			-5.9	-4.5				-1.7			21	34513142	34615142
895	*RDH11*	51109					1.5	2.4	1.8				14	67676801	67695814
896	*REL*	5966	-5.2				1.7		1.9				2	60881521	60931610
897	*RGS1*	5996	-7.0				-1.8		-1.6				1	192575727	192580031
898	*RGS2*	5997	2.7			-5.5			1.7				1	192809039	192812283
899	*RHBDD1*	84236					2.2	1.7	2.0				2	226835581	226999215
900	*RHEB*	6009	-2.9				-1.6		-1.5				7	151466012	151520120
901	*RHOG*	391					2.1		1.9		-2.9		11	3826978	3840983
902	*RILPL2*	196383					2.1		2.2		-2.0		12	123410683	123436717
903	*RNASE4*	6038	3.1				-3.4		-3.2				14	20684100	20701215
904	*RNASET2*	8635	-3.6				1.7				-3.4		6	166929504	166957191
905	*RNF149*	284996				-1.5	2.4		1.9				2	101271219	101308701
906	*RNF24*	11237	-3.3				2.9		2.4				20	3927309	4015582
907	*RNH1*	6050			1.6	-1.5						-1.9	11	494512	507300
908	*RPL30*	6156								-2.2	-2.2	-2.0	8	98024851	98046469
909	*RPL35A*	6165								-2.0	-1.7	-1.9	3	197949987	197956610
910	*RPL6*	6128		-1.6						-2.2	-1.8		12	112405190	112418838
911	*RPS15A*	6210								-2.5	-2.3	-2.7	16	18781295	18790383
912	*RPS23*	6228		-1.5						-2.0	-1.9		5	82273358	82278577
913	*RPS25*	6230		-1.7						-2.1	-2.2		11	119015712	119018691
914	*RPS27A*	6233								-2.5	-2.3	-1.9	2	55231903	55235853
915	*RRAGA*	10670			1.5		-2.0		-1.9				9	19049395	19050983
916	*RSL24D1*	51187				4.1	-1.6		-1.7				15	55180806	55197067
917	*S100A16*	140576	-6.0		1.7	4.5							1	153606886	153613145
918	*S100A9*	6280	-3.6				4.2		4.6				1	153357854	153361027
919	*S100P*	6286	-4.9		-1.7		2.1						4	6693069	6697170
920	*S100Z*	170591					2.8	2.3	2.8				5	76850001	76921650
921	*SAMD14*	201191					4.9	5.1	4.9				17	50110040	50129882
922	*SAMHD1*	25939						-1.9	1.6		-1.6		20	36890229	36951843
923	*SAMM50*	25813			-3.5		-1.7		-1.9				22	43955421	44010531
924	*SAR1A*	56681			2.1		-1.9		-1.8				10	70147289	70170523
925	*SASH3*	54440	2.6				3.0		3.5				11.16951	75003968.3	75077838.92
926	*SAV1*	60485						1.9		-1.9	-2.1		14	50632058	50668331
927	*SCFD2*	152579					2.3	2.1	2.5				4	52872982	53366075
928	*SCN1B*	6324					2.5	3.2	3.0				19	35030684	35040448
929	*SCOC*	60592	-3.0				-3.2		-2.8				4	140257286	140385726
930	*SDC3*	9672					1.8	2.0	1.7				1	30869467	30908761
931	*SDF2*	6388								-1.7	-2.1	-1.9	17	28648356	28662189
932	*SEC11A*	23478		-2.0						-1.5	-2.0		15	84669538	84716716
933	*SEC23IP*	11196	-4.2				-1.7		-1.6				10	119892711	119944658

934	*SEC61G*	23480	3.3				-2.3		-2.4				7	54752250	54759974
935	*SELL*	6402	-3.8				2.9		3.9				1	169690667	169711698
936	*SERPINB6*	5269					5.5	5.4	5.6				6	2948159	2972165
937	*SERPING1*	710	-8.2				-3.8		-3.7				11	57597387	57614853
938	*SF3A1*	10291			-1.9	-3.5	1.6						22	30331988	30356947
939	*SFT2D1*	113402	-3.6				-1.5		-1.5				6	166319728	166342591
940	*SGMS2*	166929				-4.1	-1.8		-1.7				4	107824563	107915047
941	*SH3BGRL*	6451	-4.0	1.8					1.7				X	81201943	81298547
942	*SH3BP5*	9467			1.7	2.2			1.8				3	15254853	15341368
943	*SH3TC1*	54436	-1.7				2.9		2.3				11.05374	75003282.9	75077150.32
944	*SHKBP1*	92799					2.5		2.1		-2.1		19	40576851	40591399
945	*SHMT2*	6472	3.3	-1.8	-3.7								12	57229327	57234935
946	*SIGLEC10*	89790	-4.6				2.4		2.5				19	51410021	51417803
947	*SIRPB1*	10326		-1.8			3.5		3.4				20	1563521	1620061
948	*SIRPB2*	284759	-3.9				2.8		2.9				20	1470741	1491587
949	*SLA*	6503	-8.4				2.3		3.0				8	133036724	133103054
950	*SLA2*	84174					5.4	3.3	5.6				20	36612318	36646216
951	*SLC12A9*	56996	-4.1				2.1		2.1				7	100826820	100867009
952	*SLC16A6*	9120			-3.9		1.6		2.1				17	68267026	68291267
953	*SLC20A1*	6574		-1.6	2.9				-1.7				2	112645857	112663827
954	*SLC24A4*	123041	-7.9				3.8		3.9				14	92322581	92501483
955	*SLC25A16*	8034			-2.1	-2.9		1.8					10	68477999	68527474
956	*SLC25A37*	51312					2.7		2.2		-1.8		8	23528805	23575463
957	*SLC25A5*	292	4.2	-2.3	1.8								X	119468400	119471319
958	*SLC35B3*	51000				-3.7	-1.9		-1.8				6	8413068	8435483
959	*SLC38A2*	54407	-2.6				-1.7		-1.7				X	75001912.3	75075773.12
960	*SLIRP*	81892		-1.5			-1.5		-1.5				14	77708071	77761104
961	*SLK*	9748				-4.5	-1.7		-1.6				10	103967201	104029233
962	*SMAP2*	64744	2.9				2.2		2.6				1	40344850	40423326
963	*SMCR8*	140775	-4.8				2.1		2.1				17	18315310	18328055
964	*SMIM15*	643155			3.9		-1.8		-2.1				5	61157709	61162474
965	*SNRK*	54861								-2.5	-3.5	-1.6	7.181818	75031724.1	75105727.15
966	*SNX13*	23161				-3.5	-2.2		-1.8				7	17790761	17940501
967	*SNX5*	27131		-1.5	-4.6		-1.6						20	17941597	17968980
968	*SNX6*	58533	3.7				-1.7		-1.6				14	34561094	34630183
969	*SOX4*	6659	-4.6		-1.9	-2.3							6	21592769	21598619
970	*SP110*	3431	2.4				2.8		2.7				2	230176665	230221721
971	*SPARCL1*	8404	-5.2							-5.8		-5.0	4	87473335	87531061
972	*SPAST*	6683	-5.9				-1.6	-1.8					2	32063611	32157637
973	*SPATA24*	202051					1.7	2.2	2.1				5	139396563	139404088
974	*SPN*	6693	-5.3				2.4		2.3				16	29662979	29670876
975	*SPOCD1*	90853					2.7	3.0	2.5				1	31790422	31816051
976	*SPPL3*	121665					3.0	2.7	2.3				12	120762510	120904371
977	*SPRED1*	161742	-6.0					1.6			1.5		15	38252326	38357249
978	*SPTLC2*	9517	-4.2				2.6		2.6				14	77505997	77616773
979	*SPX*	80763					1.9	3.5	4.8				12	21526307	21537377
980	*SRP19*	6728				2.7	-2.5		-2.3				5	112861222	112869788
981	*SRSF7*	6432		-1.6			-2.0		-1.6				2	38743599	38751494
982	*SRXN1*	140809			-4.9	-2.4					-1.6		20	646615	653370
983	*SSH2*	85464	-3.2				2.2		2.4				17	29625938	29930276
984	*ST3GAL1*	6482					2.6	1.8	2.2				8	133454848	133571940
985	*ST6GALNAC2*	10610	-3.4		-5.4	-5.3							17	76563710	76586956
986	*STAT5B*	6777					1.7		1.9		-3.3		17	42199168	42276707
987	*STK10*	6793					2.8		2.3		-1.8		5	172042073	172188386
988	*STK4*	6789	-2.9				2.6		2.4				20	44966474	45079959
989	*STRN4*	29888					1.9	1.8	1.8				19	46719507	46746994
990	*STX11*	8676	-6.8				3.5		4.0				6	144150526	144188370
991	*SUB1*	10923	2.2				-2.9		-2.5				5	32531633	32604079
992	*SYAP1*	94056	-3.4		1.8	3.0							X	16719632	16765336

993	*SYCP3*	50511	4.6		-2.7	-3.3							12	101728648	101739472
994	*SYPL1*	6856	-2.9				-1.9		-1.9				7	106090503	106112576
995	*TAF7*	6879					-1.6		-1.6		-1.7		5	141260225	141320821
996	*TAL1*	6886					5.7	4.8	5.9				1	47216290	47232220
997	*TATDN3*	128387	-4.3		-3.0	-2.3							1	212791828	212816626
998	*TBCA*	6902								-2.3	-1.7	-1.8	5	77691166	77868780
999	*TCEAL8*	90843	4.5				-3.5		-3.0				X	103252995	103255203
1000	*TCF12*	6938	-3.0				-3.0		-2.7				15	56918623	57299281
1001	*TCIRG1*	10312	-2.3				2.2		1.7				11	68039016	68050895
1002	*TGFB1I1*	7041	-2.4			-3.9		3.1					16	31471585	31477960
1003	*TGFBR2*	7048					-2.3	-1.9	-2.1				3	30606502	30694142
1004	*THEMIS2*	9473			-2.9		3.3		3.6				1	27872543	27886685
1005	*THYN1*	29087	-3.3				-2.0		-1.6				11	134248279	134253370
1006	*TIMP3*	7078	-8.2		-2.7	-3.5							22	32801701	32863043
1007	*TKT*	7086	-5.6				2.0		2.0				3	53224707	53256052
1008	*TLN1*	7094					2.8	2.4	2.8				9	35696948	35732395
1009	*TLR5*	7100	-5.6	2.0					1.8				1	223109406	223143282
1010	*TM2D1*	83941	2.5				-2.4		-2.2				1	61681046	61725423
1011	*TMED2*	10959	4.6							-2.0		-2.2	12	123584531	123598577
1012	*TMEM104*	54868	-3.1				1.6		1.6				7.1	75032752.1	75106760.05
1013	*TMEM106C*	79022	5.1		1.6	2.0							12	47963569	47968878
1014	*TMEM127*	55654	-4.6		-1.8	-4.0							2	96248516	96265994
1015	*TMEM129*	92305					2.1	2.1	1.9				4	1715952	1721358
1016	*TMEM147*	10430	3.8						-1.6			-1.9	19	35545595	35547526
1017	*TMEM14B*	81853					-3.0	-1.7	-2.9				6	10747759	10852753
1018	*TMEM165*	55858		1.7			-1.9		-2.0				4	55395957	55453397
1019	*TMEM170B*	100113407	-2.6				3.4		4.3				6	11538278	11583524
1020	*TMEM258*	746	-3.5				-1.8		-1.8				11	61768501	61792802
1021	*TMEM87A*	25963				5.2	-1.9		-2.1				15	42210452	42273663
1022	*TMEM9*	252839					-1.7		-1.8			-1.7	1	201134772	201171574
1023	*TMEM91*	641649					4.6	3.2	4.7				19	41350911	41384083
1024	*TMSB15B*	286527	-2.4		-2.0	-4.0							X	103918896	103966712
1025	*TNC*	3371	-1.8		4.2						2.0		9	115019578	115118257
1026	*TNFAIP2*	7127					2.0	1.9	2.1				14	103123442	103137439
1027	*TNFAIP3*	7128	3.3		-2.8	-1.8							6	137867188	137883312
1028	*TNFAIP8L1*	126282	-4.0				2.3	2.0					19	4639518	4655568
1029	*TNFRSF10C*	8794	2.6				3.5		3.9				8	23084355	23117437
1030	*TNFRSF1A*	7132	-4.2				1.8		1.6				12	6328757	6342114
1031	*TNFRSF1B*	7133					4.1		4.3		-2.5		1	12167003	12209228
1032	*TNK2*	10188	-2.9				2.5		1.8				3	195863364	195911945
1033	*TOB2*	10766	3.4		1.8	3.3							22	41433492	41447023
1034	*TOM1*	10043	-3.5				2.0		1.8				22	35299275	35347994
1035	*TOMM5*	401505	3.5				-2.2		-2.0				9	37582646	37592642
1036	*TOR4A*	54863	-3.7				2.0		1.5				7.127273	75032409.4	75106415.75
1037	*TPGS1*	91978					2.2	2.4	2.3				19	507497	519654
1038	*TPM4*	7171		1.6			1.7	2.4					19	16067021	16103005
1039	*TRA2B*	6434					2.6	2.6	2.7				3	185915906	185938136
1040	*TRAF1*	7185					2.2	2.3	2.2				9	120902393	120929173
1041	*TRAFD1*	10906			-2.1		2.1		1.9				12	112125501	112153609
1042	*TRANK1*	9881					2.2	1.6	2.2				3	36826820	36945057
1043	*TRAPPC9*	83696	-3.3				1.5	1.6					8	139730343	140458579
1044	*TRIM33*	51592					-2.0	-1.6	-1.7				1	114392777	114511160
1045	*TRIP13*	9319	-3.4		2.3	2.4							5	892643	919357
1046	*TRPM2*	7226					1.6	1.7	1.7				21	44350163	44443081
1047	*TSG101*	7251	6.4				-1.6		-1.7				11	18468336	18527232
1048	*TSPAN32*	10077					3.3	1.6	3.6				11	2301997	2318200
1049	*TSPAN9*	10867					2.0	2.9	1.9				12	3077355	3286564
1050	*TSR1*	55720					1.5	1.9	1.9				17	2322503	2337507
1051	*TTC14*	151613					-2.4	-1.6	-2.3				3	180602130	180617828

1052	*TTC3*	7267			-2.8	-3.1	-1.6						21	37073226	37203112
1053	*TTC7A*	57217					2.1	1.8	1.8				2	46916157	47076137
1054	*TTC7B*	145567					2.7	2.7	2.9				14	90524564	90816479
1055	*TUBA3C*	7278		2.4			1.6	1.8					13	19173770	19181852
1056	*TXN2*	25828	-4.6		-2.6	-1.9							22	36467036	36482030
1057	*TXNDC15*	79770	-2.9				-2.2		-1.9				5	134873803	134901525
1058	*TXNIP*	10628			-2.0	-5.0		-1.7					1	145992435	145996600
1059	*UBE2J1*	51465					1.7	1.8	2.2				6	89326625	89352848
1060	*UBE2L6*	9246	4.1								-2.9	-2.1	11	57551656	57568284
1061	*UCP2*	7351					2.6		2.9		-2.5		11	73974667	73983307
1062	*UGP2*	7360	-3.6		2.6	4.5							2	63840940	63891562
1063	*UQCRFS1*	7386					-1.8	-1.7	-1.9				19	29205321	29213541
1064	*USO1*	8615	3.8				-2.1		-1.7				4	75724593	75814286
1065	*USP32*	84669	3.1				1.8		1.7				17	60179094	60422470
1066	*UTRN*	7402		-1.5						-2.0	-2.3		6	144285701	144853034
1067	*VAMP5*	10791	-5.6		3.5	-4.1							2	85584408	85593412
1068	*VBP1*	7411	-3.0				-1.7		-1.6				X	155197007	155239817
1069	*VDAC1*	7416		-1.9			-1.8		-2.3				5	133971915	134005133
1070	*VEGFA*	7422			-2.8		-2.7		-3.2				6	43770184	43786487
1071	*VIM*	7431	-2.9					2.6	1.5				10	17228259	17237593
1072	*VIMP*	55829					-2.1		-2.4			-1.5	15	101270817	101277500
1073	*VNN2*	8875	2.6				3.2		4.0				6	132743870	132763459
1074	*VSTM1*	284415	-1.6				1.6		1.6				19	54040825	54063953
1075	*WBP2*	23558			-3.6	-4.0	1.8						17	75845699	75856507
1076	*WBP5*	51186					-3.2	2.5	-3.1				X	103356445	103358469
1077	*WDR1*	9948					2.2	2.1	2.1				4	10074339	10116949
1078	*WIPI1*	55062	4.4		-3.2	-2.7							17	68420948	68457513
1079	*WWC3*	55841					1.6				-1.9	-1.5	X	10015562	10144478
1080	*XK*	7504					3.7	3.0	3.6				X	37685759	37732130
1081	*XPC*	7508	2.9		-1.5						-1.7		3	14145147	14178783
1082	*XRCC2*	7516	-2.8					1.7	1.5				7	152644779	152676165
1083	*XRCC6*	2547		-1.7	2.5	2.0							22	41621119	41664048
1084	*YME1L1*	10730	-3.2				-2.0		-2.0				10	27110112	27155266
1085	*YWHAE*	7531		1.8						-1.7		-1.7	17	1344272	1400378
1086	*YY1AP1*	55249	6.0		-2.7		1.6						1	155659443	155689000
1087	*ZBED3*	84327					1.9	2.7	2.4				5	77072072	77087323
1088	*ZBTB45*	84878					1.7	1.9	1.7				19	58513530	58538911
1089	*ZBTB7A*	51341					2.7	1.6	1.9				19	4044364	4066945
1090	*ZCCHC6*	79670					2.1		2.2		-1.9		9	86287733	86354454
1091	*ZDHHC18*	84243					3.8		3.7		-2.8		1	26826710	26857602
1092	*ZDHHC4*	55146			-2.3	2.6		1.6					7	6577434	6589374
1093	*ZEB2*	9839	-2.9				2.4		3.0				2	144384081	144524583
1094	*ZFP62*	643836		-1.7			-1.8		-1.8				5	180847611	180861285
1095	*ZFPM1*	161882					1.6	1.6	1.8				16	88453317	88537016
1096	*ZFYVE27*	118813					1.9	1.7	1.8				10	97737121	97760907
1097	*ZKSCAN1*	7586	-3.3				-1.7		-1.5				7	100015572	100041689
1098	*ZMIZ2*	83637					2.1	2.2	2.0				7	44748581	44769881
1099	*ZMYND11*	10771	-6.0				-2.7		-2.1				10	134465	254637
1100	*ZNF189*	7743	-7.5		-1.8		-1.7						9	101398873	101410660
1101	*ZNF221*	7638	-2.7		1.9	-1.7							19	43951223	43967709
1102	*ZNF319*	57567	-3.9				1.6		1.6				16	57994668	58000453
1103	*ZNF367*	195828					3.0	3.3	2.3				9	96385941	96418329
1104	*ZNF394*	84124	-3.2				1.6		1.6				7	99486519	99500324
1105	*ZNF576*	79177	-2.3		-2.6	-5.1							19	43596392	43601157
1106	*ZNHIT3*	9326		-1.9			-2.0		-1.8				17	36486629	36499310
1107	*ZXDC*	79364	-4.2				2.1		2.0				3	126437601	126475919
1108	*ZZEF1*	23140	-5.0				2.2		2.4				17	4004445	4143020
1109	*MARCH1*	55016					1.8		2.7				4	163524298	164384050
1110	*SEPT3*	55964	-3.4		-2.2								22	41976272	41998221

1111	*SEPT10*	151011					-3.2		-3.4				2	109542982	109614206
1112	*A1BG*	1	-2.3			-3.5							19	58345178	58353499
1113	*A2M*	2	-10.9									-2.4	12	9067664	9116229
1114	*AARS*	16	-2.7		-4.1								16	70252295	70289543
1115	*ABCA7*	10347					2.8		2.5				19	1040101	1065572
1116	*ABCB7*	22	-3.0	-1.6									X	75053172	75156732
1117	*ABCE1*	6059	-4.8			2.5							4	145097932	145129179
1118	*ABR*	29			-1.8	-2.9							17	1003518	1229021
1119	*ACADM*	34	-2.9				-1.6						1	75724347	75787575
1120	*ACBD6*	84320	-5.4		1.7								1	180269653	180502954
1121	*ACLY*	47	-3.4	-1.9									17	41866908	41930542
1122	*ACOT7*	11332					1.9	1.7					1	6264269	6394391
1123	*ACTL6A*	86					-2.3		-2.8				3	179562880	179588408
1124	*ACTN3*	89		1.5							-2.2		11	66546395	66563329
1125	*ACTR6*	64431					-1.8		-1.7				12	100199122	100241865
1126	*ADA*	100	-2.8	-1.5									20	44619522	44652233
1127	*ADAM19*	8728			3.0		1.5						5	157395534	157575775
1128	*ADAM9*	8754	-5.0						-1.7				8	38996869	39105144
1129	*ADAP2*	55803					1.6		2.1				17	30906344	30959322
1130	*ADCY3*	109		1.7	3.5								2	24819169	24919839
1131	*ADGRE1*	2015					3.2		3.6				19	6887566	6940459
1132	*ADGRE3*	84658					1.9		2.0				19	14619117	14690027
1133	*ADGRG3*	222487					2.5		2.8				16	57668187	57689378
1134	*ADH4*	127						1.9	1.5				4	99123657	99157792
1135	*ADPRM*	56985	-5.6		-3.7								17	10697594	10711233
1136	*AES*	166			-2.2	-4.4							19	3052910	3063107
1137	*AFAP1L1*	134265	-1.7		1.9								5	149271871	149341802
1138	*AFF4*	27125					-1.8		-1.6				5	132875379	132963634
1139	*AFTPH*	54812	5.2								-1.8		2.502646	75026584.1	75100562.66
1140	*AGFG1*	3267					1.8		1.7				2	227472152	227561214
1141	*AGPAT3*	56894	3.4			-2.8							21	43865186	43986536
1142	*AGTPBP1*	23287	-5.1						2.2				9	85546539	85742029
1143	*AHI1*	54806	-3.5								-1.7		2.701058	75026241.4	75100218.36
1144	*AHNAK*	79026	-3.7	-2.0									11	62433542	62556235
1145	*AHR*	196	-2.3		-3.0								7	17298622	17346152
1146	*AHRR*	57491			-4.0	-4.1							5	304176	438291
1147	*AIF1*	199							2.2		-3.6		6	31615184	31617021
1148	*AK4*	205	4.2		-2.4								1	65147549	65232145
1149	*AKAP13*	11214	-2.9				1.6						15	85380571	85749358
1150	*AKAP3*	10566	-1.6		-2.4								12	4615508	4649047
1151	*AKAP7*	9465	-2.7				-2.2						6	131135666	131283535
1152	*AKAP9*	10142	3.2				-1.7						7	91940867	92110673
1153	*AKNA*	80709					3.2		3.5				9	114334156	114394405
1154	*AKR1A1*	10327					-1.7		-1.5				1	45550543	45570049
1155	*AKT3*	10000	-5.6				-2.0						1	243488233	243851079
1156	*ALCAM*	214					-2.4		-2.2				3	105366909	105576900
1157	*ALDH2*	217	-4.0		-5.1								12	111766887	111817529
1158	*ALDH3A2*	224			2.9	4.6							17	19648136	19677598
1159	*ALDOA*	226					1.8					-2.3	16	30053090	30070457
1160	*ALG2*	85365					-2.0		-1.8				9	99216426	99221956
1161	*ALG5*	29880					-2.2		-2.0				13	36949775	37000261
1162	*ALG8*	79053			-3.9	-5.6							11	78100936	78139660
1163	*JAML*	120425					3.0		3.7				11	118193740	118225094
1164	*AMMECR1*	9949			1.9	2.2							X	110194186	110440233
1165	*ANAPC11*	51529					-1.5		-1.5				17	81890790	81900991
1166	*ANAPC13*	25847	-2.9				-1.8						3	134477706	134486716
1167	*ANAPC16*	119504	-2.7				-1.5						10	72216000	72235860
1168	*ANGPTL6*	83854			-2.5	-3.2							19	10092338	10102796
1169	*ANK3*	288	-4.2							-2.5			10	60026298	60733490

1170	*ANKHD1*	54882	7.4		-3.9								9.785294	75035150.7	75109170.14
1171	*ANKHD1-EIF4EBP3*	404734	7.4		-3.9								5	140401908	140549569
1172	*ANKRD10*	55608	5.2						-1.6				13	110878540	110915069
1173	*ANKRD11*	29123					2.5		1.9				16	89267627	89490561
1174	*ANKRD13D*	338692					2.3		1.7				11	67288547	67302485
1175	*ANLN*	54443			5.7	6.5							11.2274	75004310.9	75078183.22
1176	*AOAH*	313					2.1		2.7				7	36512949	36724549
1177	*AP1S2*	8905					1.8		2.7				X	15825806	15855014
1178	*AP5B1*	91056	-4.2								-1.6		11	65775893	65780802
1179	*APBB1IP*	54518					2.7		3.2				12.3272	75010821.6	75084724.9
1180	*API5*	8539					-2.1		-1.8				11	43311963	43344529
1181	*APLP2*	334	-3.1				1.8						11	130069837	130144811
1182	*APOBEC3B*	9582			4.5	4.7							22	38982347	38992804
1183	*APOBEC3D*	140564	-4.0		2.1								22	39014363	39033276
1184	*APOBEC3G*	60489		-2.0							-1.7		22	39040961	39087743
1185	*APOBR*	55911					3.0		3.0				16	28494649	28498970
1186	*APOC1*	341	-2.4		-1.7								19	44914247	44919349
1187	*APOD*	347	-8.0			4.3							3	195568702	195584205
1188	*AQP1*	358								-3.3		-1.9	7	30911855	30925516
1189	*AQP9*	366					3.7		3.7				15	58138169	58185911
1190	*ARAP1*	116985					2.8		2.8				11	72685069	72793599
1191	*ARFGAP3*	26286	-3.3				-1.8						22	42796502	42858106
1192	*ARG2*	384						1.8	1.6				14	67619798	67651720
1193	*ARHGAP18*	93663		3.4				3.3					6	129576132	129710225
1194	*ARHGAP25*	9938					2.0		2.5				2	68679601	68826833
1195	*ARHGAP27*	201176					3.3		2.3				17	45393902	45434421
1196	*ARHGAP30*	257106					3.4		3.7				1	161046946	161069970
1197	*ARHGAP9*	64333					3.4		3.9				12	57472255	57488814
1198	*ARHGDIA*	396				-2.1	1.7						17	81867721	81871406
1199	*ARHGEF1*	9138					2.3		2.4				19	41883161	41930150
1200	*ARHGEF18*	23370					1.9				-2.0		19	7395113	7472477
1201	*ARID4B*	51742					-1.6		-1.5				1	235131634	235328219
1202	*ARL5B*	221079					-2.2		-1.9				10	18659405	18681639
1203	*ARMC3*	219681	-1.7	-2.2									10	22928024	23038523
1204	*ARPC1B*	10095					2.0				-2.2		7	99374249	99394801
1205	*ARPC2*	10109	1.9								-2.0		2	218217094	218254356
1206	*ARRB1*	408					3.0		3.2				11	75264182	75351705
1207	*ARRB2*	409					2.4		2.6				17	4710489	4721499
1208	*ASAP2*	8853	-3.5					2.9					2	9206765	9405683
1209	*ASCL2*	430					2.0		2.2				11	2268495	2270952
1210	*ASGR2*	433					2.3		2.5				17	7101322	7115700
1211	*ASNSD1*	54529					-3.0		-2.4				12.38508	75011164.2	75085069.2
1212	*ASPM*	259266	-5.8		3.2								1	197084128	197146694
1213	*ASS1*	445	-4.1									2.2	9	130444929	130501274
1214	*ASUN*	55726					-2.0		-2.0				12	26905181	26938326
1215	*ATAD1*	84896					-2.8		-2.8				10	87751512	87841343
1216	*ATAD3B*	83858		-1.5				1.8					1	1471769	1497848
1217	*ATF4*	468			-3.2	2.0							22	39519695	39522685
1218	*ATIC*	471					-2.1		-2.6				2	215311817	215349773
1219	*ATP13A3*	79572					-2.0		-1.6				3	194402672	194498364
1220	*ATP1A2*	477	-5.2	-2.8									1	160115759	160143591
1221	*ATP5A1*	498		-1.9			-1.6						18	46080248	46104334
1222	*ATP5S*	27109			1.7	5.1							14	50312326	50335558
1223	*ATP6V1G1*	9550									-1.7	-1.8	9	114587746	114598373
1224	*ATRAID*	51374					-2.2		-2.2				2	27212027	27217178
1225	*B4GALT1*	2683	2.7				1.5						9	33104082	33167356
1226	*BACE1*	23621			-1.7	-4.0							11	117285207	117316259
1227	*BASP1*	10409					2.0				-3.1		5	17065598	17276843
1228	*BATF3*	55509						1.8	1.9				1	212686418	212699985

1229	*BAZ1B*	9031	3.9	-2.3									7	73440398	73522278
1230	*BCL10*	8915					1.8		-1.8				1	85266248	85277090
1231	*BCL2*	596								-2.3	-2.5		18	63123346	63320128
1232	*BCL6*	604					2.1		2.5				3	187721377	187745727
1233	*BEND7*	222389		2.2				1.7					10	13438484	13528974
1234	*BET1*	10282				3.9		2.1					7	93962762	94004382
1235	*BEX4*	56271			-2.8	-2.0							X	103215092	103217246
1236	*BICD2*	23299					2.6		2.6				9	92711363	92764812
1237	*BID*	637	-3.8				2.0						22	17734138	17774770
1238	*BIRC5*	332			3.9	4.0							17	78214186	78225636
1239	*BNIPL*	149428	-4.4							-2.3			1	151036570	151047600
1240	*BORCS8*	729991					2.3		1.8				19	19176903	19192591
1241	*BPTF*	2186	2.9									1.7	17	67825524	67984378
1242	*BRD2*	6046					1.8	1.5					6	32968660	32981505
1243	*BRI3*	25798	3.1			-1.5							7	98252379	98310441
1244	*BST1*	683					1.6		1.7				4	15702950	15738313
1245	*BST2*	684			4.2							-3.1	19	17402939	17405648
1246	*BTBD7*	55727	-3.4				-1.6						14	93237550	93333092
1247	*BTF3L4*	91408			-2.2	2.3							1	52056125	52090716
1248	*BTG2*	7832	-3.3	-1.9									1	203305491	203309602
1249	*BTK*	695					2.6		3.0				X	101349447	101390796
1250	*BUB1*	699			4.4	5.3							2	110637698	110678114
1251	*BUB1B*	701	-4.3		2.7								15	40161023	40221136
1252	*BUB3*	9184	3.2		2.7								10	123154277	123170467
1253	*C11orf21*	29125					4.5		4.6				11	2295645	2303049
1254	*C11orf96*	387763	-6.3		-2.5								11	43925342	43944338
1255	*C12orf57*	113246					-3.5		-3.3				12	6942978	6946003
1256	*C15orf40*	123207	-3.5			1.5							15	82988441	83011641
1257	*C15orf52*	388115	-2.3					2.8					15	40331452	40340967
1258	*C16orf54*	283897					1.7	-1.9					16	29742463	29746006
1259	*C17orf62*	79415					2.0		1.6				17	82442589	82450829
1260	*C1GALT1*	56913	-4.9						-1.5				7	7156934	7248651
1261	*C1orf106*	55765	-5.3								1.9		1	200891048	200915735
1262	*C1orf174*	339448	-4.1			2.8							1	3889125	3900293
1263	*C1orf198*	84886		2.5				3.0					1	230837119	230869589
1264	*C1QB*	713	-4.6					2.0					1	22652762	22661538
1265	*C1R*	715	-7.6									-1.7	12	7080209	7092607
1266	*C21orf33*	8209	3.8									-1.6	21	44133605	44145723
1267	*C22orf15*	150248			-3.5	-2.9							22	23763021	23765861
1268	*C6orf106*	64771			2.2	3.0							6	34587288	34696859
1269	*C8orf76*	84933	3.1			4.3							8	123219957	123241398
1270	*CA2*	760		2.5				2.4					8	85463852	85481493
1271	*CA5B*	11238	-6.8		-1.8								X	15688830	15788409
1272	*CAB39L*	81617						3.3	2.3				13	49308650	49444126
1273	*CACNA2D3*	55799	-2.6						1.7				3	54122547	55074557
1274	*CACUL1*	143384	-3.4								-1.6		10	118674167	118755249
1275	*CADPS*	8618	-3.1		-2.0								3	62398346	62875389
1276	*CALD1*	800	-4.6									-1.8	7	134744252	134970728
1277	*CALM2*	805			1.6	1.8							2	47160082	47176601
1278	*CALU*	813	3.1					2.9					7	128739292	128771807
1279	*CANX*	821					-1.9		-1.5				5	179678628	179730925
1280	*CAP2*	10486	-6.1		-3.2								6	17393216	17557792
1281	*CARD17*	440068			-3.5	-3.1							11	105092469	105101431
1282	*CASC4*	113201	-2.8								-2.2		15	44288729	44415758
1283	*CASC5*	57082	-2.5		2.2								15	40594020	40664342
1284	*CAV1*	857	-4.3							-1.9			7	116524785	116561184
1285	*CBR1*	873	3.9		2.3								21	36069941	36073166
1286	*CBS*	875			-1.6					2.4			21	43053191	43076943
1287	*CBX5*	23468	-3.0	-2.1									12	54230940	54280133

1288	*CBX6*	23466	3.1									-1.6	22	38861450	38872314
1289	*CCDC112*	153733			1.5	-2.3							5	115267188	115296831
1290	*CCDC14*	64770			3.0	1.6							3	123897305	123961408
1291	*CCDC152*	100129792			4.6	4.3							5	42756801	42802360
1292	*CCDC157*	550631			-1.9	-3.5							22	30356635	30378658
1293	*CCDC167*	154467	3.1	-1.6									6	37482920	37499922
1294	*CCDC28A*	25901			-3.6	-4.7							6	138773509	138793319
1295	*CCDC47*	57003					-1.7		-1.6				17	63745250	63776351
1296	*CCDC6*	8030					-1.6		-1.5				10	59788763	59906656
1297	*CCDC86*	79080	3.9		-1.8								11	60842071	60851081
1298	*CCNB1*	891			3.5	6.8							5	69167010	69178245
1299	*CCNB2*	9133			5.8	5.7							15	59105078	59125045
1300	*CCND1*	595			-3.1	-4.5							11	69641087	69654474
1301	*CCNT2*	905					-2.2		-1.8				2	134918235	134959342
1302	*CCNY*	219771					1.6				-1.9		10	35247025	35572669
1303	*CCR2*	729230					1.9		3.1				3	46353734	46360928
1304	*CCR7*	1236					3.8		4.1				17	40553769	40565472
1305	*CCT3*	7203					-2.5		-2.7				1	156308968	156367873
1306	*CCT4*	10575					-1.6		-1.7				2	61868089	61888804
1307	*CD109*	135228	-2.3			-3.2							6	73695785	73828316
1308	*CD14*	929					1.6		2.0				5	140631728	140633701
1309	*CD1D*	912					2.9		3.5				1	158179947	158184896
1310	*CD300A*	11314					2.6		2.6				17	74466416	74484796
1311	*CD300LF*	146722					2.4		2.9				17	74694311	74712978
1312	*CD37*	951					3.5		4.1				19	49335171	49343335
1313	*CD3EAP*	10849			-2.1	-3.6							19	45406209	45410766
1314	*CD48*	962					1.5		2.3				1	160678746	160711851
1315	*CD68*	968				-2.2			1.6				17	7579467	7582113
1316	*CD69*	969					-1.7	-2.1					12	9752486	9760901
1317	*CD86*	942					1.9		2.0				3	122055366	122121139
1318	*CD8B*	926		-2.9							-2.2		2	86815339	86861924
1319	*CD93*	22918					1.5		2.2				20	23079349	23086340
1320	*CD96*	10225	-2.8		3.8								3	111292719	111665750
1321	*CDC23*	8697	3.6		1.9								5	138187648	138213343
1322	*CDC37*	11140					1.6					-1.5	19	10391134	10420121
1323	*CDCA8*	55143			2.0	3.2							1	37692418	37709719
1324	*CDIP1*	29965	-2.9						1.6				16	4510675	4538828
1325	*CDK13*	8621	-3.3		-2.7								7	39950037	40097134
1326	*CDK5RAP2*	55755	-3.4	2.5									9	120388869	120580170
1327	*CDK6*	1021							-1.8			1.7	7	92604921	92836594
1328	*CDK8*	1024					-1.7		-1.7				13	26254104	26405238
1329	*CDKL1*	8814	2.4	1.8									14	50329404	50416461
1330	*CDKN3*	1033			5.3	5.5							14	54396849	54420218
1331	*CEACAM1*	634	-5.1		-3.7								19	42507304	42561234
1332	*CEBPD*	1052	-5.5						1.5				8	47736909	47739086
1333	*CELF1*	10658	5.7	-2.1									11	47465944	47565569
1334	*CELF2*	10659					2.5		3.1				10	11005321	11336675
1335	*CENPH*	64946			4.0	2.5							5	69189548	69210357
1336	*CENPM*	79019			6.0	6.6							22	41938721	41947164
1337	*CENPU*	79682			2.4				-1.6				4	184694618	184734133
1338	*CERK*	64781			-3.1	-2.8							22	46684411	46738261
1339	*CETN2*	1069					-1.5		-1.6				X	152826973	152830777
1340	*CETN3*	1070					-2.1		-1.9				5	90392261	90409786
1341	*CFAP157*	286207	-2.8		-2.3								9	127706989	127716002
1342	*CFD*	1675	-1.6						2.5				19	859643	863630
1343	*CFLAR*	8837					2.4		2.3				2	201116104	201176687
1344	*CFP*	5199					4.5		4.9				X	47624213	47630305
1345	*CHCHD10*	400916			-3.5	-2.9							22	23765834	23768443
1346	*CHEK1*	1111	-3.0							1.6			11	125625136	125676255

1347	*CHKB*	1120	-2.8				1.6						22	50578949	50601455
1348	*CHMP7*	91782	-2.3	-1.5									8	23243637	23262000
1349	*CHORDC1*	26973			1.9	4.5							11	90201160	90223364
1350	*CHP1*	11261	-2.6							-1.8			15	41230839	41281890
1351	*CHST2*	9435					2.0		1.9				3	143119331	143124014
1352	*CIAPIN1*	57019	5.4								-1.5		16	57428169	57447528
1353	*CIITA*	4261	-4.9	-1.8									16	10877198	10932281
1354	*CKAP2*	26586			2.8	4.9							13	52455429	52476628
1355	*CKAP4*	10970	6.5		-3.8								12	106237877	106304279
1356	*CKS1B*	1163		-1.8	2.0								1	154974653	154979249
1357	*CLCN3*	1182		1.8				1.6					4	169612633	169723673
1358	*CLCN7*	1186					1.7		1.9				16	1444934	1475580
1359	*CLDN7*	1366			1.7	2.4							17	7259903	7263983
1360	*CLEC1B*	51266	-3.3	3.2									12	9985642	10013424
1361	*CLEC2B*	9976			4.0						-1.7		12	9852984	9870136
1362	*CLEC4D*	338339					3.6		3.8				12	8509475	8522366
1363	*CLEC4E*	26253					2.9		3.4				12	8533305	8540963
1364	*CLK3*	1198					1.9		1.7				15	74598500	74645414
1365	*CLUL1*	27098	-2.9		4.3								18	596988	650334
1366	*CMPK1*	51727		2.4					-1.5				1	47333797	47378839
1367	*CMTM1*	113540	-3.7		2.2								16	66566393	66579137
1368	*CMTM2*	146225					3.5		3.9				16	66579448	66588275
1369	*CMTM8*	152189	-2.7		-2.3								3	32238679	32370325
1370	*CNDP1*	84735	-2.0		4.2								18	74534440	74587212
1371	*CNIH4*	29097					-1.5		-1.9				1	224356850	224379459
1372	*CNN1*	1264	-3.9								-2.6		19	11538717	11550323
1373	*CNN2*	1265	-4.9				1.6						19	1026581	1039068
1374	*CNOT2*	4848					-1.7		-1.6				12	70242994	70354993
1375	*CNPY3*	10695					2.2		2.4				6	42929192	42939287
1376	*COA1*	55744			2.2	4.8							7	43608456	43729717
1377	*COA4*	51287	-5.0									-2.1	11	73872667	73876988
1378	*COG4*	25839			-1.6	-2.3							16	70480568	70523565
1379	*COL19A1*	1310	-2.3		-1.9								6	69866571	70209976
1380	*COL1A2*	1278	-6.8									-2.3	7	94394561	94431232
1381	*COL4A2*	1284	-2.6									-2.5	13	110305812	110513027
1382	*COL6A1*	1291	-6.0									-1.7	21	45981737	46005050
1383	*COMMD6*	170622	-3.4								-1.7		13	75525219	75549439
1384	*COMT*	1312			1.7						-1.7		22	19941607	19969975
1385	*COPB2*	9276					-1.8		-1.8				3	139355600	139389732
1386	*COPS4*	51138					-2.2		-1.6				4	83034447	83075818
1387	*COPS8*	10920					-2.5		-2.4				2	237085312	237100466
1388	*CORO1A*	11151					3.9		4.2				16	30182827	30189076
1389	*CPM*	1368	-6.5					2.5					12	68842197	68971570
1390	*CPN1*	1369	-2.3		-2.2								10	100042193	100081877
1391	*CPNE3*	8895					-1.8		-1.6				8	86484830	86561498
1392	*CPPED1*	55313					2.6		2.6				16	12659799	12804017
1393	*CPQ*	10404			-2.4	-4.9							8	96645227	97149654
1394	*CPVL*	54504	-2.4		-4.9								12.15355	75009793.6	75083692
1395	*CREB5*	9586	-3.1						2.2				7	28299321	28825894
1396	*CRIP1*	1396		-2.3							-2.1		14	105486317	105488947
1397	*CRLF3*	51379	-2.7								-2.5		17	30769388	30824776
1398	*CSF3R*	1441					4.4		4.5				1	36466043	36483278
1399	*CSGALNACT1*	55790	-4.1		3.2								8	19404161	19758029
1400	*CSK*	1445					2.3		1.9				15	74782057	74803198
1401	*CSNK1A1*	1452						1.7	-1.6				5	149492197	149551552
1402	*CTNNAL1*	8727			4.3	2.5							9	108942569	109013529
1403	*CTNNBIP1*	56998			-1.5					-2.6			1	9848276	9910336
1404	*CTNS*	1497		1.8				1.7					17	3636468	3661542
1405	*CTSC*	1075	-2.7								-1.8		11	88293592	88337787

1406	*CTSZ*	1522	-7.9		-2.4								20	58995185	59007247
1407	*CUL4A*	8451			-3.2	-3.4							13	113208193	113267108
1408	*CUL5*	8065									-3.2	-2.4	11	108008733	108107776
1409	*CUTA*	51596		-1.5			-1.5						6	33416442	33418317
1410	*CUX1*	1523					2.3		1.9				7	101815904	102283957
1411	*CXCL1*	2919			-5.4						-1.9		4	73869393	73871242
1412	*CXCL14*	9547	-3.6							-5.3			5	135570679	135579279
1413	*CXCL5*	6374					1.7	3.1					4	73995642	73998779
1414	*CXCL9*	4283	-2.0									-2.9	4	76001275	76007488
1415	*CXorf21*	80231	-1.7						1.6				X	30558824	30577844
1416	*CXXC5*	51523					-1.7		-1.8				5	139647299	139683882
1417	*CYB5B*	80777	3.7		1.6								16	69424525	69466266
1418	*CYBB*	1536					2.7		3.2				X	37780011	37813461
1419	*CYP4F3*	4051					3.4		2.6				19	15615617	15662825
1420	*CYSTM1*	84418					-2.2		-2.7				5	140174642	140282052
1421	*CYTH2*	9266	3.7					1.7					19	48469032	48482314
1422	*CYTH4*	27128					2.4		2.7				22	37282027	37315345
1423	*CYTIP*	9595					1.6		1.9				2	157414619	157488961
1424	*DAZAP1*	26528			-2.3	-1.8							19	1407569	1435687
1425	*DCAF16*	54876	-5.7	-2.2									10.19265	75034808.1	75108825.84
1426	*DCAKD*	79877			-2.8	-3.4							17	45023340	45061109
1427	*DCTD*	1635	3.5			1.8							4	182890060	182917936
1428	*DCTN6*	10671			2.6	3.0							8	30156297	30183640
1429	*DDAH2*	23564	-3.1			-5.0							6	31727038	31730617
1430	*DDX17*	10521						1.9	1.6				22	38483440	38507660
1431	*DDX19A*	55308	-4.6			3.8							16	70346829	70373383
1432	*DDX47*	51202					-1.7		-2.0				12	12813316	12829981
1433	*DDX52*	11056	-5.1		-3.9								17	37609739	37643464
1434	*DENND1A*	57706					1.6		1.5				9	123379654	123930152
1435	*DENND1C*	79958					3.1		3.2				19	6467207	6482557
1436	*DENND2C*	163259	3.2					1.7					1	114582848	114670422
1437	*DENND4B*	9909					2.1		1.8				1	153929501	153946696
1438	*DEPDC1B*	55789			3.1	3.9							5	60596912	60700190
1439	*DGAT1*	8694	4.2				1.6						8	144314584	144326910
1440	*DGKD*	8527					2.4		2.4				2	233354507	233472104
1441	*DHRS13*	147015					1.8	1.6					17	28897781	28903071
1442	*DHRS4L2*	317749	-4.4	-1.9									14	23969874	24006408
1443	*DHRS9*	10170	-2.6						-1.8				2	169064789	169096167
1444	*DHX15*	1665					-1.9		-1.7				4	24517441	24584550
1445	*DHX38*	9785	2.4		-2.1								16	72093562	72112912
1446	*DKC1*	1736					-2.6		-2.8				X	154762742	154777689
1447	*DLD*	1738	4.7				-1.9						7	107890970	107931730
1448	*DLGAP5*	9787			4.0	4.7							14	55148112	55191678
1449	*DMTN*	2039	3.5	3.2									8	22048995	22082527
1450	*DNAH17*	8632	-3.1		-1.9								17	78423697	78577394
1451	*DNAJA1*	3301					-2.1		-1.6				9	33025211	33039907
1452	*DNAJB7*	150353			4.5	4.9							22	40859549	40862126
1453	*DNAJC1*	64215					-1.6		-1.9				10	21756537	22003769
1454	*DNAJC10*	54431					-2.1		-2.2				10.93797	75002597.6	75076461.72
1455	*DNAJC12*	56521	-5.3		-4.3								10	67796665	67838166
1456	*DNAJC15*	29103	-3.4		1.7								13	43023203	43114224
1457	*DNAJC2*	27000					-2.0		-2.2				7	103312474	103344873
1458	*DNAJC3*	5611			-3.3	-3.3							13	95677139	95794989
1459	*DNAJC8*	22826					-2.0		-1.7				1	28199456	28233025
1460	*DNPH1*	10591		-2.3								-1.9	6	43225629	43229484
1461	*DOK1*	1796					2.5		2.6				2	74549026	74557554
1462	*DOK2*	9046					3.9		4.0				8	21908873	21913860
1463	*DOK3*	79930					4.0		4.2				5	177501907	177511274
1464	*DOK4*	55715	-3.2	2.5									16	57471922	57487327

1465	*DPH6*	89978			-3.1	-4.6							15	35217345	35546193
1466	*DPPA4*	55211	-5.9									2.1	3	109326141	109337572
1467	*DPY30*	84661	-4.0						-1.6				2	31867809	32039812
1468	*DRAM2*	128338		-1.5			-1.5						1	111117333	111140216
1469	*DSC2*	1824					-1.6		-1.6				18	31058840	31102415
1470	*DSG2*	1829			-1.5	-4.3							18	31498043	31549008
1471	*DSP*	1832								-3.8	3.6		6	7541575	7586717
1472	*DSTN*	11034					-2.6		-2.4				20	17569863	17609919
1473	*DTD1*	92675			-3.7	-4.3							20	18567453	18763917
1474	*DTX4*	23220	-5.2	-2.7									11	59171430	59208587
1475	*DUSP6*	1848		-1.9			1.9						12	89347232	89353271
1476	*DYNLL1*	8655								-1.8		-1.7	12	120469850	120498493
1477	*DYNLT1*	6993					-1.8		-1.8				6	158636474	158644739
1478	*DYRK4*	8798			-3.4	-4.0							12	4562204	4615302
1479	*DYSF*	8291					3.7		3.6				2	71453722	71686768
1480	*DZIP1*	22873	-4.2		-3.0								13	95578202	95644703
1481	*DZIP3*	9666			2.9	4.6							3	108589682	108694846
1482	*EAF2*	55840					1.9		3.3				3	121835183	121886526
1483	*EEA1*	8411					-2.9		-2.5				12	92770637	92929331
1484	*EEF1G*	1937		-1.9	-1.6								11	62559601	62574086
1485	*EFEMP1*	2202	-3.4							-2.1			2	55865967	55924139
1486	*EGF*	1950						2.2	2.4				4	109912884	110012266
1487	*EGLN2*	112398	4.3			2.8							19	40798996	40808433
1488	*EGLN3*	112399					1.8	2.8					14	33924231	34462774
1489	*EHBP1*	23301					-2.6		-2.4				2	62673851	63046487
1490	*EHBP1L1*	254102					2.6		1.9				11	65576038	65592650
1491	*EHD1*	10938					2.5		1.9				11	64851642	64888296
1492	*EID1*	23741					-1.8		-1.6				15	48877886	48880183
1493	*EIF1AY*	9086			4.2	-3.2							Y	20575725	20593154
1494	*EIF2AK2*	5610	-3.1						-1.5				2	37099210	37157065
1495	*EIF3K*	27335		-1.6						-1.5			19	38619082	38636955
1496	*EIF4A3*	9775			-3.7	-2.9							17	80135214	80147183
1497	*EIF4EBP3*	8637	3.1		-3.9								5	140547666	140549578
1498	*EIF5A*	1984	3.8					2.0					17	7306999	7312463
1499	*ELF4*	2000	2.5								-1.5		X	130064874	130110716
1500	*ELOVL5*	60481	2.4								-2.4		6	53267398	53349179
1501	*ELP5*	23587			1.7	2.4							17	7251416	7259940
1502	*EMC2*	9694					-3.5		-3.3				8	108443601	108486918
1503	*EMC4*	51234					-1.8		-1.9				15	34224999	34230156
1504	*EMCN*	51705	-6.0									-1.9	4	100395341	100880126
1505	*EML4*	27436					-2.0		-1.9				2	42169350	42332548
1506	*ENKUR*	219670	-4.6	2.3									10	24981979	25062279
1507	*ENOSF1*	55556	-3.3		4.3								18	670324	712676
1508	*ENTPD4*	9583	-3.5	-1.5									8	23385783	23457695
1509	*ENTPD6*	955			-3.2	-2.3							20	25195693	25226729
1510	*EOGT*	285203	-2.6					1.7					3	68975214	69013961
1511	*EPB41*	2035					2.5		2.9				1	28887091	29120046
1512	*EPS8*	2059			-2.1	-2.0							12	15620158	15882329
1513	*ERAP2*	64167	-4.8		4.0								5	96875939	96919716
1514	*ERCC1*	2067			-2.1	-3.6							19	45407333	45478828
1515	*ERLEC1*	27248					-2.6		-2.5				2	53787044	53818819
1516	*ERN2*	10595			5.1	3.6							16	23690309	23713500
1517	*ERO1A*	30001	-3.6						-1.9				14	52639916	52695900
1518	*ESYT2*	57488	-3.8						-2.1				7	158730995	158830253
1519	*ETNK1*	55500					-1.9		-1.6				12	22625075	22690665
1520	*EVI2B*	2124					1.7		2.6				17	31303766	31314112
1521	*EXOSC2*	23404			-4.0	-2.0							9	130693721	130704894
1522	*EXOSC8*	11340	-3.0		-1.8								13	36998816	37009613
1523	*F2R*	2149	-5.0					2.1					5	76716043	76735781

1524	*F2RL1*	2150								-1.7	-2.0		5	76818933	76835315
1525	*F5*	2153					3.1		2.8				1	169514166	169586588
1526	*FAAH*	2166	-5.7	2.3									1	46394265	46413848
1527	*FABP5*	2171			2.1			2.5					8	81280363	81284777
1528	*FADD*	8772	-2.3			1.7							11	70203163	70207390
1529	*FAM102A*	399665	-3.1								-1.7		9	127940579	127980513
1530	*FAM110B*	90362	3.0		-2.5								8	57994509	58204279
1531	*FAM126A*	84668	-5.3		-2.0								7	22889371	23014130
1532	*FAM129C*	199786	-3.3	-1.9									19	17523301	17553839
1533	*FAM174B*	400451	-2.9			-3.9							15	92617443	92809884
1534	*FAM222B*	55731	-3.3		-1.7								17	28755978	28855232
1535	*FAM229B*	619208		-2.3				1.9					6	112087599	112102790
1536	*FAM3C*	10447					-1.8		-2.3				7	121348851	121396364
1537	*FAM49A*	81553					2.0		2.4				2	16549459	16666331
1538	*FAM49B*	51571						-1.8			-2.1		8	129839593	130017129
1539	*FAM60A*	58516					-2.0		-2.4				12	31280584	31327058
1540	*FAM65B*	9750					3.7		4.2				6	24804282	25042168
1541	*FAM96A*	84191									-2.0	-2.1	15	64072559	64094018
1542	*FANCD2*	2177	-6.2		3.5								3	10026414	10101930
1543	*FANCL*	55120	-2.7					2.4					2	58159243	58241372
1544	*FARP1*	10160		-1.7	-2.3								13	98142562	98455176
1545	*FBLN5*	10516	-8.3		-1.7								14	91869412	91947987
1546	*FBN1*	2200								-2.3		-1.8	15	48408306	48645849
1547	*FBN2*	2201					2.9		2.9				5	128257909	128659185
1548	*FBXO11*	80204						1.7	1.6				2	47789316	47905793
1549	*FBXO28*	23219					-2.2		-2.2				1	224114087	224162047
1550	*FBXO32*	114907			-2.9	-2.7							8	123497889	123541206
1551	*FCER1G*	2207					1.9		2.2				1	161215234	161220699
1552	*FCN1*	2219					6.1		6.9				9	134905890	134917963
1553	*FCRL1*	115350					1.8		2.0				1	157794403	157820105
1554	*FCRL3*	115352					1.8		2.0				1	157674321	157700857
1555	*FCRL5*	83416	-1.8	2.2									1	157513377	157552520
1556	*FCRLA*	84824					2.0		2.3				1	161706972	161714352
1557	*FEM1B*	10116					-2.1		-1.6				15	68277803	68295865
1558	*FERMT3*	83706					4.3		4.0				11	64206678	64223886
1559	*FES*	2242					2.9		3.0				15	90883695	90895776
1560	*FEZ1*	9638			1.6	-4.2							11	125445745	125496317
1561	*FGF13*	2258		1.6	2.2								X	138614731	139222777
1562	*FGGY*	55277	-6.2		2.9								1	59296638	59810647
1563	*FGL1*	2267								2.5		2.4	8	17864380	17910365
1564	*FHIT*	2272	-2.7		-2.3								3	59749310	61251459
1565	*FIP1L1*	81608			2.5	-1.7							4	53377643	53460861
1566	*FIS1*	51024					-2.7		-2.6				7	101239458	101252316
1567	*FKBP11*	51303			-4.0	-5.8							12	48921518	48926474
1568	*FKBP1A*	2280			-1.5	-1.8							20	1368978	1393172
1569	*FLI1*	2313					2.0		2.9				11	128686535	128813267
1570	*FLNB*	2317			2.5						-1.5		3	58008400	58172251
1571	*FLOT1*	10211									-1.9	-1.6	6	30727709	30742733
1572	*FLOT2*	2319	-2.9								-1.5		17	28879335	28897679
1573	*FNBP1*	23048					1.8		2.0				9	129887187	130043194
1574	*FNDC3B*	64778					-3.0		-3.1				3	172039628	172401665
1575	*FOLR3*	2352					3.0		3.1				11	72114869	72139892
1576	*FOSL1*	8061	-2.4		-1.5								11	65892049	65900573
1577	*FOSL2*	2355								-2.3	-1.7		2	28392448	28417312
1578	*FOXO3*	2309								-1.6	-1.7		6	108559835	108684774
1579	*FOXP1*	27086					-1.7		-1.5				3	70954693	71583989
1580	*FPR1*	2357					2.9		4.1				19	51745172	51804110
1581	*FRAT1*	10023					2.1		2.7				10	97319267	97321915
1582	*FRAT2*	23401	3.0					-1.6					10	97332497	97334709

1583	*FRY*	10129					-1.6				-2.6		13	32031300	32299122
1584	*FTH1*	2495			-3.5	-5.0							11	61959718	61967660
1585	*FUNDC2*	65991	5.7				-1.6						X	155025980	155060303
1586	*FUT2*	2524	-5.0					1.5					19	48695971	48705950
1587	*FUT8*	2530	-2.4		3.1								14	65410592	65744121
1588	*FXYD3*	5349	-5.1		-2.0								19	35115879	35124324
1589	*FYCO1*	79443	-6.7							-1.6			3	45917899	45995824
1590	*G3BP1*	10146						1.6	1.6				5	151771045	151812785
1591	*G6PD*	2539					3.8		3.6				X	154531391	154547572
1592	*GAB1*	2549						3.9	2.0				4	143336762	143474568
1593	*GABARAPL2*	11345								-1.9	-2.5		16	75566351	75577881
1594	*GADD45A*	1647		2.8	-3.0								1	67685061	67688338
1595	*GAK*	2580					2.2		1.8				4	849276	932373
1596	*GALK2*	2585	-3.9		2.8								15	49155656	49367869
1597	*GANAB*	23193									-2.1	-2.1	11	62624826	62646726
1598	*GARS*	2617			-3.3	2.1							7	30594681	30634033
1599	*GATM*	2628								-2.9		-1.6	15	45361124	45402327
1600	*GBP3*	2635					-1.9		-2.5				1	89006666	89022894
1601	*GCLC*	2729	-3.0							-1.5			6	53497341	53616970
1602	*GCOM1*	145781						3.2	2.6				15	57591908	57714745
1603	*GDA*	9615	-3.5									1.7	9	72114595	72257193
1604	*GEMIN8*	54960	-4.2		-1.9								20.14286	75044402.7	75118466.22
1605	*GFM2*	84340					-1.9		-2.0				5	74721204	74767371
1606	*GIMAP5*	55340									-3.4	-2.1	7	150722253	150750033
1607	*GIMAP8*	155038					2.1		2.7				7	150450630	150479392
1608	*GLIPR1*	11010							1.8		-1.5		12	75480680	75503853
1609	*GLIPR2*	152007					2.3		2.6				9	36136536	36163913
1610	*GLO1*	2739					-2.5		-2.5				6	38675925	38703141
1611	*GLS*	2744					-2.0		-1.7				2	190880827	190965552
1612	*GLT1D1*	144423					3.8		4.3				12	128853427	128984968
1613	*GLT8D1*	55830					-1.7		-1.5				3	52694485	52706083
1614	*GLTSCR1L*	23506	-3.6							-1.7			6	42746958	42868560
1615	*GLTSCR2*	29997	-6.1	-1.7									19	47745522	47757058
1616	*GMFG*	9535					1.7		2.5				19	39328353	39342372
1617	*GNA15*	2769					2.9		1.5				19	3136193	3163769
1618	*GNAI3*	2773					-2.0		-2.0				1	109548611	109618321
1619	*GNG7*	2788	-2.7	-2.3									19	2511219	2702709
1620	*GNPAT*	8443					-1.8		-1.8				1	231241207	231277973
1621	*GNPNAT1*	64841	-2.8	-2.0									14	52775194	52791668
1622	*GOLGA2*	2801			-1.6	-3.3							9	128255829	128275995
1623	*GOLGA8A*	23015					-1.9		-2.1				15	34525207	34588503
1624	*GOLT1B*	51026					-2.2		-2.2				12	21501781	21518408
1625	*GORASP2*	26003					-2.0		-2.5				2	170928464	170967129
1626	*GPBAR1*	151306					2.6		1.9				2	218259496	218263859
1627	*GPM6A*	2823	-3.0		-1.7								4	175632934	176002664
1628	*GPR137B*	7107			-1.5	-1.8							1	236142505	236221865
1629	*GPR143*	4935			-5.7	-6.4							X	9725346	9786297
1630	*GPR27*	2850					1.6		2.3				3	71754050	71756496
1631	*GPR34*	2857					-1.8	1.8					X	41688973	41697277
1632	*GRAMD1A*	57655	-3.1				1.5						19	34994784	35026471
1633	*GRB10*	2887	3.9		-2.5								7	50590063	50793462
1634	*GRK6*	2870					2.8		2.6				5	177403204	177442901
1635	*GRN*	2896				-2.6	1.7						17	44345086	44353102
1636	*GRPEL2*	134266			-3.8	-1.8							5	149345430	149354583
1637	*GSDMD*	79792					2.2		1.9				8	143553207	143563062
1638	*GSPT1*	2935					-1.8		-2.1				16	11868128	11916082
1639	*GSTP1*	2950	-4.4			-2.2							11	67583595	67586660
1640	*GTF2B*	2959	-3.2								-1.8		1	88852932	88891944
1641	*GTF2H1*	2965					-2.4		-2.0				11	18322295	18367044

1642	*GTPBP1*	9567					2.0				-2.7		22	38705723	38738299
1643	*GTPBP4*	23560					-1.9		-1.9				10	988019	1019936
1644	*GUF1*	60558	-2.4		-2.6								4	44678427	44700926
1645	*GUSB*	2990	3.6								-1.8		7	65960684	65982314
1646	*H2AFV*	94239			1.9	3.4							7	44826791	44848083
1647	*HAL*	3034					3.7		3.8				12	95972662	95996365
1648	*HARS*	3035	-2.8		3.0								5	140673173	140692024
1649	*HBB*	3043	1.7						1.8				11	5225464	5229395
1650	*HBE1*	3046	3.3		-2.1								11	5268345	5505617
1651	*HBS1L*	10767	-6.6			3.9							6	134960378	135103056
1652	*HCAR3*	8843					3.9		1.8				12	122714756	122716892
1653	*HCFC1R1*	54985	-3.4		2.9								16	3022620	3024286
1654	*HCST*	10870	3.1						1.8				19	35902480	35904377
1655	*HDAC2*	3066				1.9	-1.6						6	113933028	114011308
1656	*HDGF*	3068		1.5								-1.6	1	156742107	156766925
1657	*HEBP2*	23593	-2.8			-4.8							6	138403531	138422197
1658	*HECTD1*	25831					-1.9		-1.8				14	31100112	31207804
1659	*HEMGN*	55363								2.5		1.6	9	97926791	97944856
1660	*HES1*	3280	-5.5					2.6					3	194136145	194138732
1661	*HES6*	55502			-2.3	2.2							2	238238267	238240662
1662	*HEXIM1*	10614								-1.9	-1.8		17	45148502	45152101
1663	*HIPK1*	204851	-2.0								-1.9		1	113929192	113977869
1664	*HIST1H1C*	3006			-5.4	-2.9							6	26055787	26056428
1665	*HIST1H2BE*	8344					2.7	2.7					6	26172059	26184655
1666	*HIST1H4C*	8364					-1.7		-1.8				6	26103876	26104310
1667	*HK3*	3101					4.8		4.6				5	176880869	176899332
1668	*HLA-B*	3106			-2.7	-5.3							6	31353872	31357188
1669	*HLA-DMA*	3108	-3.6	-1.9									6	32948613	32969094
1670	*HLA-DMB*	3109	-5.1	-2.1									6	32934629	32941070
1671	*HLA-DQA1*	3117	-2.7	-2.0									6	32628179	32647062
1672	*HLA-DQB1*	3119		-2.4								-1.6	6	32659467	32668383
1673	*HLA-DRA*	3122	-4.7	-2.0									6	32439842	32445046
1674	*HLA-DRB1*	3123	-5.0	-1.8									6	32552990	32589848
1675	*HLA-E*	3133					2.1		2.0				6	30489467	30494205
1676	*HLX*	3142					3.0		2.7				1	220879400	220885059
1677	*HMGB2*	3148			4.3	1.8							4	173331695	173335125
1678	*HMGB3*	3149			2.5	1.5							X	150980509	150990775
1679	*HMGXB4*	10042			-2.0	-3.8							22	35257452	35295807
1680	*HNRNPA3*	220988					-2.4		-2.0				2	177212563	177223958
1681	*HNRNPC*	3183					-1.8		-1.7				14	21209136	21269494
1682	*HSCB*	150274	-2.9		-3.3								22	28742031	28757515
1683	*HSD17B12*	51144					-2.1		-2.1				11	43556436	43856617
1684	*HSPA6*	3310					3.0		3.2				1	161524540	161526910
1685	*HSPA9*	3313					-1.6		-1.7				5	138554882	138575444
1686	*HSPB11*	51668					-2.4		-2.4				1	53916574	53945929
1687	*HSPE1*	3336		-1.8					-1.8				2	197499994	197503457
1688	*HTRA1*	5654	-9.3		-3.0								10	122461525	122514908
1689	*ICAM2*	3384					2.0		2.2				17	64002594	64020634
1690	*ID1*	3397	-3.7	2.1									20	31605283	31606515
1691	*ID3*	3399			3.7	1.9							1	23557918	23559794
1692	*IER2*	9592					1.8				-2.3		19	13150415	13154908
1693	*IER5*	51278		-2.7			1.7						1	181088712	181092899
1694	*IFFO1*	25900					1.7		1.6				12	6538375	6556083
1695	*IFI16*	3428			-1.8						-1.7		1	158999968	159055155
1696	*IFI27L1*	122509			2.6	-2.4							14	94081282	94103846
1697	*IFI6*	2537	-2.5			-5.1							1	27666061	27672218
1698	*IFITM1*	8519	-3.0			-2.5							11	313506	315272
1699	*IFITM3*	10410	-4.2			-1.5							11	319669	327537
1700	*IFT88*	8100			-3.6	-1.8							13	20567069	20691437

1701	*IGF2BP2*	10644					2.3	2.4					3	185643739	185825056
1702	*IGFBP2*	3485	-3.2		-2.9								2	216632828	216664436
1703	*IGFBP4*	3487	-7.1		-2.4								17	40443461	40457731
1704	*IGFLR1*	79713					1.7		1.7				19	35738801	35742453
1705	*IGSF10*	285313	-2.3									1.8	3	151425384	151458709
1706	*IKZF5*	64376								-2.5		-2.9	10	122990806	123008817
1707	*IL18RAP*	8807					2.4		3.6				2	102418689	102452565
1708	*IL1R1*	3554	-3.0							-2.0			2	102064544	102179874
1709	*IL1R2*	7850					2.9		1.6				2	101991844	102028544
1710	*IL24*	11009		-1.5		-8.9							1	206897443	206904139
1711	*IL6ST*	3572		2.5			-1.9						5	55935095	55994993
1712	*IMMT*	10989					-1.7		-1.6				2	86143932	86195770
1713	*IMPAD1*	54928					-1.8		-1.6				7.333333	75040290.7	75114334.63
1714	*INPP1*	3628					-1.7		-2.1				2	190343470	190371665
1715	*INPP5B*	3633	-5.5		-1.5								1	37860697	37947057
1716	*INPP5D*	3635					2.8		2.8				2	233059967	233207903
1717	*INSIG2*	51141					-1.7		-2.0				2	118088452	118110997
1718	*INSR*	3643	-6.0								1.9		19	7112255	7294034
1719	*INTS4*	92105	-3.7		-3.0								11	77878720	77994678
1720	*IPCEF1*	26034					2.2		2.5				6	154154496	154356792
1721	*IPO13*	9670	-4.1					1.8					1	43946939	43968022
1722	*IQCJ*	654502		-1.9	4.2								3	158962928	159266307
1723	*IRF4*	3662			-3.1	-2.2							6	391739	411447
1724	*IRF8*	3394					1.5		1.7				16	85898803	85922609
1725	*IRS2*	8660					2.6		2.1				13	109752698	109786568
1726	*ISG15*	9636	-3.8			-7.8							1	1001138	1014541
1727	*ITGA4*	3676		-1.6					1.9				2	181457202	181536187
1728	*ITGA6*	3655	-3.9		2.6								2	172427354	172506282
1729	*ITGAM*	3684					3.0		3.1				16	31259990	31332892
1730	*ITGB5*	3693			3.4			2.4					3	124761948	124901418
1731	*ITSN2*	50618	3.4								-2.0		2	24202864	24360714
1732	*JMJD6*	23210	-4.8				1.9						17	76712832	76726799
1733	*KANK1*	23189	-6.2							-1.7			9	470291	746106
1734	*KAT5*	10524			-1.6	-3.8							11	65711996	65719604
1735	*KBTBD11*	9920					2.4		1.7				8	1973878	2006936
1736	*KCNMB4*	27345	-3.5									1.8	12	70366276	70434292
1737	*KCTD2*	23510		-1.7					1.6				17	75032575	75065889
1738	*KCTD20*	222658					1.5		2.6				6	36442767	36491143
1739	*KDELR2*	11014					-1.6		-1.8				7	6445953	6484242
1740	*KDM3B*	51780	-1.8				1.6						5	138352596	138437028
1741	*KHDRBS1*	10657	3.7	-2.1									1	32013829	32060850
1742	*KIAA0226L*	80183							1.5		-2.0		13	46342000	46438190
1743	*KIAA0391*	9692					-1.8		-2.0				14	35121846	35277614
1744	*KIAA0408*	9729	-3.5		-1.9								6	127440343	127459391
1745	*KIAA0895*	23366			5.7	6.5							7	36324221	36390125
1746	*KIAA0907*	22889					-2.0		-2.6				1	155913043	155934400
1747	*KIAA1524*	57650			2.9	4.6							3	108549869	108589644
1748	*KIAA1551*	55196					2.2		1.8				12	31959370	31993107
1749	*KIAA1841*	84542	-4.7	2.2									2	61065871	61138034
1750	*KIF13B*	23303	-6.4	-1.8									8	29067279	29263124
1751	*KIF20B*	9585			3.0	3.3							10	89701610	89774939
1752	*KIF22*	3835	3.7		3.4								16	29790719	29805385
1753	*KIF4A*	24137			3.5	3.0							X	70290090	70420832
1754	*KIFC1*	3833			3.0	3.0							6	33391536	33409924
1755	*KLF2*	10365					2.5		1.8				19	16324817	16327874
1756	*KLHL9*	55958	2.4				-1.8						9	21329671	21335380
1757	*KPNA4*	3840					-1.8		-1.9				3	160494995	160565588
1758	*KRCC1*	51315					-1.7		-1.6				2	88027205	88055729
1759	*KRT14*	3861	-2.2							-3.7			17	41582279	41586921

1760	*KRT15*	3866	-7.0		5.1								17	41513743	41522529
1761	*KRT4*	3851	-9.6								-2.8		12	52806549	52814551
1762	*KRTAP19-1*	337882			5.7	3.1							21	30479699	30480344
1763	*LAMP1*	3916			-2.5	-4.0							13	113297241	113323672
1764	*LAMTOR1*	55004					2.0		2.0				11	72085895	72103387
1765	*LAMTOR3*	8649			2.1	1.7							4	99878336	99894490
1766	*LBR*	3930			2.7						-3.1		1	225401502	225428925
1767	*LCORL*	254251			3.4	2.5							4	17841199	18021876
1768	*LCP2*	3937					2.3		2.7				5	170246237	170298227
1769	*LCTL*	197021			3.2	2.2							15	66547179	66565979
1770	*LDHB*	3945					-1.6		-1.5				12	21635342	21757857
1771	*LDOC1L*	84247	-3.9		-2.1								22	44492572	44498298
1772	*LEPR*	3953	-1.8	1.8									1	65420652	65641559
1773	*LETM1*	3954	-3.5							-1.9			4	1811479	1856247
1774	*LGALS3*	3958				-1.7				-1.5			14	55124110	55145413
1775	*LGALS3BP*	3959	-6.6									-3.2	17	78971238	78980109
1776	*LHFPL2*	10184	-4.2					1.6					5	78485215	78770021
1777	*LIG4*	3981			-3.1	-2.8							13	108207439	108218368
1778	*LILRA1*	11024					3.4		3.7				19	54573879	54602090
1779	*LILRA2*	11027					4.6		4.9				19	54572920	54590287
1780	*LILRA3*	11026					4.5		4.4				19	270735	280849
1781	*LILRB1*	10859					3.1		3.6				19	54617158	54637528
1782	*LILRB3*	11025					3.1		3.7				19	54216278	54223506
1783	*LIMS3*	96626						2.3	1.8				2	109898428	109924868
1784	*LIPT1*	51601	-1.7		-3.3								2	99154955	99163157
1785	*LITAF*	9516			1.8	2.0							16	11547722	11636381
1786	*LMBRD1*	55788					-2.4		-1.9				6	69675802	69797111
1787	*LMBRD2*	92255					-2.0		-1.7				5	36098412	36151961
1788	*LMO2*	4005	-2.5						1.7				11	33858576	33892289
1789	*LMO4*	8543				-3.6			-1.9				1	87328468	87348923
1790	*LPAR2*	9170					2.1		1.8				19	19623668	19628930
1791	*LPXN*	9404			5.7			-1.7					11	58526871	58578220
1792	*LRCH4*	4034					2.4		2.1				7	100574011	100586153
1793	*LRMP*	4033					1.7		2.6				12	25021002	25108334
1794	*LRP8*	7804					1.8	1.9					1	53242364	53328070
1795	*LRPPRC*	10128					-2.2		-2.2				2	43886508	43996005
1796	*LRRFIP1*	9208	3.1				1.7						2	237627576	237813682
1797	*LRRK2*	120892					2.3		3.2				12	40196744	40369285
1798	*LSM1*	27257	3.7		3.2								8	38163321	38176730
1799	*LSM12*	124801	-4.0						-1.5				17	44034635	44067619
1800	*LSM6*	11157			2.6	3.3							4	146175685	146200000
1801	*LSM7*	51690	-4.4	-1.7									19	2321517	2328620
1802	*LTB4R*	1241					4.3		4.3				14	24311450	24318036
1803	*LY75*	4065	-2.8				1.5						2	159803355	159904749
1804	*LYAR*	55646			1.6	5.9							4	4267701	4290169
1805	*LYL1*	4066					3.8		4.0				19	13099033	13103161
1806	*LYNX1*	66004	-4.0	-1.8									8	142764334	142778224
1807	*MACROD2*	140733	-3.2	-1.8									20	13995369	16053197
1808	*MADD*	8567					2.1		1.8				11	47269161	47330031
1809	*MAGEA10*	4109	-4.8		5.3								X	152133310	152138578
1810	*MAGT1*	84061					-1.7		-1.6				X	77826364	77895593
1811	*MAN1B1*	11253	-2.8	-1.5									9	137086927	137109187
1812	*MAN2A1*	4124					-1.9		-1.7				5	109689366	109869625
1813	*MAN2A2*	4122					2.6		2.8				15	90902218	90922584
1814	*MAN2B1*	4125					1.8		1.7				19	12646511	12666742
1815	*MANEAL*	149175	-4.8		-3.2								1	37793802	37801137
1816	*MAP1A*	4130	-4.2					2.2					15	43510958	43531620
1817	*MAP3K11*	4296					2.0		1.5				11	65597755	65615382
1818	*MAP3K13*	9175	-7.3				-1.7						3	185282941	185489097

1819	*MAP4K5*	11183	-4.6								-2.0		14	50418501	50561126
1820	*MAP7*	9053					-2.5		-2.4				6	136342281	136550819
1821	*MAPK3*	5595	-3.6					1.6					16	30114105	30123506
1822	*MAPK6*	5597			-3.6				-1.6				15	51952106	52067372
1823	*MAPKAPK3*	7867					2.6		2.3				3	50611520	50649297
1824	*MARCKSL1*	65108	3.5	-2.0									1	32333832	32336379
1825	*MAST3*	23031					2.2		2.4				19	18097793	18151692
1826	*MAT2A*	4144	-4.0				-2.0						2	85539165	85545280
1827	*MATN2*	4147	-7.1		2.4								8	97868840	98036716
1828	*MBD6*	114785	5.0				1.6						12	57520710	57530148
1829	*MBNL2*	10150					-2.1		-1.7				13	97221434	97394120
1830	*MBOAT7*	79143					2.3		1.6				19	54173412	54189882
1831	*MCEMP1*	199675					2.8		2.5				19	7676628	7679826
1832	*MCFD2*	90411					-2.1		-1.9				2	46901870	46941855
1833	*MCM4*	4173	-4.5							2.3			8	47960185	47978160
1834	*MCTP1*	79772						1.6	2.8				5	94703741	95284575
1835	*MDFIC*	29969	-1.7				-1.9						7	114922154	115019916
1836	*MDH1*	4190					-1.7		-1.7				2	63588609	63607197
1837	*MDM2*	4193	-4.5		-3.1								12	68808172	68850686
1838	*MEF2C*	4208	-3.1			-4.5							5	88717117	88904257
1839	*MEGF9*	1955					3.4		4.0				9	120600813	120714470
1840	*MEIS1*	4211		2.9				3.4					2	66433452	66573869
1841	*MEN1*	4221	3.4				1.7						11	64803510	64811294
1842	*METTL1*	4234			-4.9	-2.0							12	57768471	57772793
1843	*METTL21A*	151194	-3.1		2.9								2	207580631	207625928
1844	*METTL25*	84190	-2.8			2.1							12	82358497	82479236
1845	*METTL2B*	55798	-3.1		1.8								7	128476729	128506602
1846	*MFNG*	4242					2.5		2.7				22	37469063	37486401
1847	*MFSD7*	84179	-2.9					1.6					4	681829	689441
1848	*MGA*	23269		-1.6				1.6					15	41621224	41773081
1849	*MGAM*	8972					5.5		5.8				7	141907813	142106747
1850	*MGAT4B*	11282					2.7	2.4					5	179797597	179806952
1851	*MGME1*	92667	3.0							-1.6			20	17968913	17991122
1852	*MIB1*	57534					-1.9		-1.7				18	21704957	21870957
1853	*MIER1*	57708	2.8								-1.8		1	66924895	66988619
1854	*MITF*	4286			-1.9	-2.1							3	69739435	69968337
1855	*MLH1*	4292					-1.7		-1.5				3	36993332	37050918
1856	*MLPH*	79083			-3.1	-3.1							2	237485428	237555318
1857	*MMP25*	64386					3.0		3.1				16	3046681	3060726
1858	*MMP9*	4318					4.3		2.4				20	46008908	46016561
1859	*MND1*	84057	-1.7		2.4								4	153344649	153415118
1860	*MNDA*	4332					2.4		3.6				1	158831317	158849506
1861	*MOB1A*	55233	-4.2		-3.5								2	74152528	74178898
1862	*MOB2*	81532	-5.1			-2.3							11	1469457	1501247
1863	*MOB3A*	126308					3.6		3.2				19	2071038	2096673
1864	*MOCOS*	55034	-2.5		-2.4								18	36187519	36272157
1865	*MON1B*	22879	-3.1				1.9						16	77190835	77202405
1866	*MPC1*	51660	-3.4			-2.9							6	166364919	166383013
1867	*MRPL3*	11222					-1.8		-1.9				3	131462212	131502983
1868	*MRPL30*	51263					-1.6		-1.7				2	99181079	99197626
1869	*MRPL32*	64983					-2.5		-2.2				7	42932200	42948958
1870	*MRPL33*	9553	2.7			1.6							2	27771717	27988087
1871	*MRPL42*	28977	3.7		2.1								12	93467488	93516213
1872	*MRPL47*	57129					-2.1		-2.2				3	179588285	179604654
1873	*MRPL52*	122704	-4.8	-2.0									14	22829879	22835037
1874	*MRPL9*	65005					-1.5		-1.9				1	151759643	151763564
1875	*MRPS10*	55173					-1.8		-1.9				6	42206801	42217865
1876	*MRPS18B*	28973		-2.5					-1.5				6	30617709	30626395
1877	*MS4A1*	931					2.4		3.8				11	60455752	60470760

1878	*MSL2*	55167					2.0		2.0				3	136148922	136197241
1879	*MSMO1*	6307			4.3	4.0							4	165327623	165343160
1880	*MSN*	4478	4.7								-2.7		X	65588377	65741931
1881	*MT1G*	4495			-2.4	-2.8							16	56666731	56668065
1882	*MTA3*	57504		-1.5							1.8		2	42494569	42756947
1883	*MTERF3*	51001			-2.3	-5.2							8	96239398	96261610
1884	*MTFR1L*	56181	-4.7	1.8									1	25818640	25832942
1885	*MTIF2*	4528					-1.8		-1.7				2	55236595	55269347
1886	*MTPAP*	55149	-3.9		2.3								10	30309801	30374448
1887	*MTSS1*	9788								-2.7	-1.9		8	124550790	124728429
1888	*MX2*	4600					2.1				-1.9		21	41361943	41409390
1889	*MXD1*	4084					3.1		2.2				2	69897688	69942945
1890	*MXD4*	10608	2.8						1.9				4	2247432	2262294
1891	*MXRA5*	25878								-2.3		-2.9	X	3308565	3346641
1892	*MXRA7*	439921			-3.5	-3.5							17	76672551	76711016
1893	*MYC*	4609			-4.7	-2.2							8	127735434	127741434
1894	*MYCT1*	80177						2.2	1.8				6	152697895	152724567
1895	*MYL4*	4635						1.8	1.7				17	47200446	47223679
1896	*MYL6*	4637						1.9				-1.6	12	56158161	56163496
1897	*MYO15B*	80022	-4.4				1.7						17	75588058	75626849
1898	*MYO1F*	4542					3.4		3.8				19	8520790	8577577
1899	*MYO1G*	64005					3.5		3.7				7	44962662	44979098
1900	*MYO5A*	4644			-2.1	-4.3							15	52307283	52529050
1901	*MYO6*	4646			-2.3							-1.8	6	75749192	75919537
1902	*MYO9B*	4650					2.0		1.9				19	17075781	17214537
1903	*MZT2A*	653784	-3.3						-1.7				2	131464900	131492743
1904	*N4BP2L1*	90634	-6.5						1.8				13	32400723	32428311
1905	*N4BP2L2*	10443					-1.7		-1.7				13	32432417	32538885
1906	*NACA*	4666		-1.6							-1.5		12	56712428	56731628
1907	*NAIP*	4671					1.8		2.2				5	70968483	71025114
1908	*NANOS1*	340719	-5.0		-3.0								10	119029716	119033732
1909	*NAPEPLD*	222236			-1.6	1.8							7	103099776	103149560
1910	*NBEAL2*	23218					4.1		3.6				3	46979683	47009703
1911	*NCAPD2*	9918	-3.1		1.8								12	6493356	6531955
1912	*NCAPH*	23397			3.5	2.4							2	96335787	96373845
1913	*NCF2*	4688					2.3		2.5				1	183555563	183590876
1914	*NCF4*	4689					4.1		4.5				22	36860988	36878015
1915	*NCKIPSD*	51517	-3.5					1.5					3	48673844	48686364
1916	*NCOA1*	8648	3.6						1.6				2	24491914	24770702
1917	*NDN*	4692	-3.8		-3.4								15	23685400	23687330
1918	*NDNL2*	56160					1.6		1.7				15	29268149	29269829
1919	*NDST2*	8509					1.7		1.6				10	73801911	73811798
1920	*NDUFA12*	55967	-5.0	-1.8									12	94897055	95003770
1921	*NDUFA13*	51079					-1.7		-1.6				19	19515736	19529054
1922	*NDUFA3*	4696	-4.1			-1.6							19	54102728	54109257
1923	*NDUFA6*	4700			2.5	2.5							22	42085525	42090955
1924	*NDUFA8*	4702					-1.5		-1.6				9	122144058	122159819
1925	*NDUFA9*	4704					-1.9		-2.0				12	4649095	4694317
1926	*NDUFB2*	4708	2.5	-1.7									7	140690777	140722790
1927	*NDUFC2*	4718					-1.7		-1.9				11	78068304	78080219
1928	*NDUFS7*	374291			-3.6	-5.0							19	1383527	1395589
1929	*NDUFV2*	4729					-1.7		-1.6				18	9102630	9134345
1930	*NEBL*	10529	-3.4							-2.9			10	20779973	21174187
1931	*NECAP2*	55707	2.4	-1.7									1	16440672	16460078
1932	*NELFCD*	51497			-2.1	-2.6							20	58981208	58995133
1933	*NEMF*	9147					-1.7		-1.6				14	49782083	49853203
1934	*NF2*	4771			-1.8	-4.0							22	29603556	29698598
1935	*NFE2*	4778					4.7		4.7				12	54292111	54301121
1936	*NFKBIZ*	64332					2.1		2.2				3	101827991	101861022

1937	*NFYB*	4801					-2.1		-1.6				12	104117077	104138289
1938	*NGDN*	25983	-5.7			2.1							14	23469688	23509862
1939	*NGRN*	51335					-3.4		-3.5				15	90265659	90278141
1940	*NHLRC3*	387921					1.7		1.7				13	39038306	39050109
1941	*NHP2*	55651			2.0							-1.6	5	178149460	178153967
1942	*NIFK*	84365					-1.6		-1.7				2	121726945	121736923
1943	*NINJ1*	4814					2.0		2.2				9	93121489	93134288
1944	*NKIRAS1*	28512			3.0	4.3							3	23891660	23946591
1945	*NKIRAS2*	28511					2.1		1.6				17	42011382	42025644
1946	*NLRP3*	114548					2.0		2.8				1	247416156	247449108
1947	*NOD2*	64127					2.2		1.9				16	50693603	50733077
1948	*NOL8*	55035				-1.8		1.6					9	92297358	92325636
1949	*NOSIP*	51070			3.5		-1.6						19	49555711	49590262
1950	*NOX5*	79400	-5.5		-3.1								15	68930525	69062743
1951	*NPEPL1*	79716					2.1		1.9				20	58689131	58719238
1952	*NPFFR2*	10886	-2.0		3.9								4	72031804	72148067
1953	*NPHP4*	261734			-1.8	-3.4							1	5862811	5992473
1954	*NPIPB3*	23117	2.4								-1.8		16	21834569	21880827
1955	*NPLOC4*	55666					2.0		1.6				17	81556887	81648465
1956	*NPM1*	4869					-2.0		-1.9				5	171387116	171411137
1957	*NQO2*	4835			3.4	3.0							6	2987987	3028869
1958	*NR4A3*	8013	-3.9		-2.6								9	99821855	99866891
1959	*NRAS*	4893	-6.9					1.5					1	114704469	114716894
1960	*NRBP2*	340371	3.7		-1.6								8	143833594	143840974
1961	*NREP*	9315					-2.6		-2.9				5	111662621	111997464
1962	*NSRP1*	84081			-2.7	-4.0							17	30115521	30186475
1963	*NT5C3A*	51251	-2.9	2.5									7	33014114	33062797
1964	*NT5DC3*	51559	-2.9		-1.7								12	103770453	103841197
1965	*NUP107*	57122					-1.6		-1.8				12	68686734	68745809
1966	*NUP37*	79023			2.9	4.7							12	102073103	102120124
1967	*NUP43*	348995	-4.9		2.2								6	149724315	149749665
1968	*NUPR1*	26471			-6.6	-2.1							16	28532708	28539174
1969	*NUSAP1*	51203			5.1	4.2							15	41332694	41381050
1970	*OAS1*	4938	2.5				3.0						12	112906777	112933222
1971	*OASL*	8638					3.0	2.4					12	121019111	121039242
1972	*OCIAD2*	132299			3.9	-2.3							4	48885019	48906937
1973	*OGDH*	4967	-3.3			-2.8							7	44606572	44709066
1974	*OGFR*	11054					1.6		1.6				20	62804835	62814000
1975	*OIP5*	11339	-1.7		3.4								15	41309268	41332621
1976	*OLFML2A*	169611	-3.1							-2.4			9	124777158	124814885
1977	*OLIG1*	116448					2.9		3.0				21	33070144	33072420
1978	*OS9*	10956	-4.0			-2.3							12	57693955	57721557
1979	*OSBPL10*	114884	-5.9			-1.8							3	31657890	32077580
1980	*OSBPL9*	114883					-2.3		-2.0				1	51577179	51798427
1981	*OSCAR*	126014					2.5		2.4				19	54094668	54102692
1982	*OTUD6B*	51633			-2.0	-3.8							8	91070196	91087095
1983	*OXR1*	55074					-1.9		-1.8				8	106270178	106752694
1984	*P2RX1*	5023					2.5		3.8				17	3896592	3916500
1985	*P2RX4*	5025			-1.7	-5.4							12	121209857	121234106
1986	*P2RY13*	53829					1.6		2.8				3	151326312	151329548
1987	*P2RY8*	286530					3.0		3.4				X	1462572	1537107
1988	*PABPC4*	8761		-1.7	-2.2								1	39560816	39576790
1989	*PADI4*	23569					4.7		4.8				1	17308195	17364004
1990	*PAFAH1B1*	5048								-2.0	-1.5		17	2593210	2685615
1991	*PAFAH1B2*	5049	-3.3						-1.8				11	117144267	117176894
1992	*PAGR1*	79447	-3.6		2.2								16	29815952	29820117
1993	*PAIP1*	10605			-2.1	-2.2							5	43526267	43557758
1994	*PAPSS2*	9060			-2.1		-1.9						10	87659613	87747705
1995	*PARP9*	83666					2.2		1.6				3	122527924	122564577

1996	*PARVG*	64098					2.8		3.0				22	44172956	44219533
1997	*PAXBP1*	94104					-2.3		-2.0				21	32733899	32771858
1998	*PBLD*	64081					-1.5	2.2					10	68282660	68333049
1999	*PBX1*	5087		2.6				3.3					1	164555584	164899296
2000	*PCK2*	5106		-1.6	-4.7								14	24094053	24110598
2001	*PCSK1*	5122	-1.6								-1.5		5	96390415	96434143
2002	*PDCD2*	5134			2.2	3.6							6	170575295	170584692
2003	*PDCD4*	27250		-2.0			-1.5						10	110871795	110900006
2004	*PDCD7*	10081	-3.6	-1.6									15	65117379	65133836
2005	*PDE4B*	5142	-3.6								-1.5		1	65792514	66374579
2006	*PDE8A*	5151					-1.9		-1.7				15	84980440	85139145
2007	*PDGFA*	5154			-2.4			3.5					7	497258	520296
2008	*PDGFC*	56034					-1.8		-2.2				4	156760454	156971394
2009	*PDZD11*	51248							-1.8			-1.6	X	70286595	70290514
2010	*PER2*	8864	-4.2				-1.7						2	238244038	238290102
2011	*PFDN2*	5202					-1.5		-1.9				1	161100556	161118111
2012	*PFKFB4*	5210					2.3		2.0				3	48517684	48562015
2013	*PFKP*	5214	-3.1					1.6					10	3066333	3137712
2014	*PFN1*	5216					1.9		1.6				17	4945652	4949061
2015	*PGD*	5226					2.7		2.5				1	10398592	10420144
2016	*PGGT1B*	5229			1.9	2.5							5	115204012	115262872
2017	*PGLS*	25796			-2.3	-3.8							19	17511629	17521288
2018	*PGM2*	55276			4.5	1.9							4	37826633	37862937
2019	*PGRMC1*	10857		2.7				2.2					X	119236245	119244466
2020	*PHB*	5245		-1.8					-1.7				17	49404049	49414905
2021	*PHC2*	1912					1.7				-2.1		1	33323623	33431052
2022	*PHF6*	84295	-3.7			4.1							X	134373253	134428791
2023	*PHGDH*	26227			-5.2	-3.6							1	119659798	119744215
2024	*PHLDA2*	7262	-2.9			-4.4							11	2928273	2929455
2025	*PHYKPL*	85007					2.3		2.3				5	178208497	178232791
2026	*PIGC*	5279					-1.5		-1.7				1	172370189	172444086
2027	*PIGF*	5281					-1.9		-1.6				2	46580937	46617119
2028	*PIGY*	84992					-2.0		-1.7				4	88521573	88521789
2029	*PIK3AP1*	118788	-3.4								-2.1		10	96593312	96720514
2030	*PIK3C2A*	5286					-2.1		-1.6				11	17077730	17207983
2031	*PILRA*	29992					3.2		3.4				7	100367530	100400099
2032	*PILRB*	29990				4.4	1.5						7	100352176	100367733
2033	*PIM2*	11040				3.0					-2.4		X	48913182	48919024
2034	*PIM3*	415116	3.9		-1.9								22	49960513	49964080
2035	*PISD*	23761					1.7		2.0				22	31618491	31662432
2036	*PKP1*	5317	-7.0							-3.7			1	201283452	201332993
2037	*PLA1A*	51365	-4.5		-4.9								3	119597842	119629811
2038	*PLCG2*	5336							1.5		-2.1		16	81739097	81962693
2039	*PLEC*	5339	3.0									-1.5	8	143915147	143976734
2040	*PLEK*	5341					2.8		3.2				2	68365173	68397453
2041	*PLEKHA1*	59338	-5.8							-1.6			10	122374696	122442602
2042	*PLEKHO1*	51177					3.5		3.6				1	150149183	150164720
2043	*PLIN5*	440503	-3.5	3.0									19	4522531	4535224
2044	*PLK1*	5347			5.1	3.6							16	23677656	23690367
2045	*PLK2*	10769	2.6		-3.4								5	58453982	58460260
2046	*PLOD1*	5351			2.1	4.7							1	11934205	11975538
2047	*PLOD3*	8985	3.3			-1.7							7	101205977	101218420
2048	*PLPP4*	196051			2.4	-3.0							10	120456954	120589855
2049	*PMEL*	6490			-2.9	-3.3							12	55954105	55973317
2050	*PMS2*	5395	-6.0		-1.7								7	5973239	6009125
2051	*PNPLA3*	80339	-6.1	-2.3									22	43923739	43964488
2052	*PNPLA6*	10908					2.0		1.7				19	7534004	7561764
2053	*PNPO*	55163	-4.2	-1.6									17	47941506	47948288
2054	*POLR1B*	84172						2.0	1.6				2	112541915	112577150

2055	*POLR3C*	10623			-2.8	2.3							1	145824088	145842505
2056	*POLR3GL*	84265	2.7			-3.5							1	145964702	145978848
2057	*POLR3H*	171568	3.4								-1.9		22	41525804	41544606
2058	*POP4*	10775					-2.1		-2.3				19	29604017	29617237
2059	*POP7*	10248					-1.6		-2.1				7	100706053	100707495
2060	*POU2F2*	5452					2.0		2.1				19	42086110	42196585
2061	*PPA2*	27068					-1.6		-1.9				4	105369077	105474081
2062	*PPARD*	5467	-5.6	-1.8									6	35342558	35428191
2063	*PPBP*	5473					8.3		7.8				4	73987038	73988197
2064	*PPCS*	79717			-4.3	-6.3							1	42456117	42473385
2065	*PPIB*	5479					-1.8		-1.9				15	64155812	64163205
2066	*PPIC*	5480	-3.3		-4.4								5	123023250	123036741
2067	*PPIP5K1*	9677	-4.5					2.1					15	43533462	43590253
2068	*PPP1CA*	5499									-1.9	-1.8	11	67398183	67421183
2069	*PPP1CC*	5501		-2.0			-1.5						12	110719680	110742939
2070	*PPP1R14A*	94274	-6.2	2.2									19	38251237	38256591
2071	*PPP1R14B*	26472			-2.1	-4.7							11	64244480	64246941
2072	*PPP1R15A*	23645					2.7		2.4				19	48872392	48876057
2073	*PPP1R15B*	84919			1.7	3.0							1	204403387	204411791
2074	*PPP2R2A*	5520	4.9						-1.5				8	26291491	26372680
2075	*PPP6C*	5537	3.2			3.5							9	125146573	125189939
2076	*PPT2*	9374								-2.9		-2.0	6	32153441	32163680
2077	*PRAM1*	84106					4.8		5.0				19	8490056	8503112
2078	*PRDM2*	7799	-4.2								-1.6		1	13700198	13825079
2079	*PRDX2*	7001			-2.4	-8.6							19	12796820	12801859
2080	*PRDX4*	10549					-4.0		-3.6				X	23664262	23686399
2081	*PRDX5*	25824							-1.6			-1.6	11	64318088	64321811
2082	*PREPL*	9581	3.9		-2.5								2	44316281	44361862
2083	*PREX1*	57580					3.7		3.9				20	48624252	48827883
2084	*PRKACA*	5566				-4.1					-1.6		19	14091688	14118084
2085	*PRKAG2*	51422	-3.1		-3.2								7	151556111	151877125
2086	*PRKAR2A*	5576	-2.0				-1.6						3	48744597	48847846
2087	*PRKCB*	5579					4.2		4.9				16	23836001	24220611
2088	*PRKCH*	5583	-4.3		-1.8								14	61187559	61550976
2089	*PRKG1*	5592	-2.9								1.6		10	50991358	52298350
2090	*PROK2*	60675					4.0		4.4				3	71771656	71785206
2091	*PROS1*	5627	-4.2					2.9					3	93873033	93974066
2092	*PRPF18*	8559	3.4	-2.0									10	13586927	13630868
2093	*PRPF31*	26121	4.1			-3.5							19	54115410	54131719
2094	*PRPF38A*	84950	-5.7									1.7	1	52404564	52420839
2095	*PRRC2A*	7916						1.9	1.6				6	31620720	31637771
2096	*PRRC2C*	23215	4.8									-2.1	1	171485551	171593511
2097	*PRTFDC1*	56952	-2.5					3.8					10	24848607	24952604
2098	*PSMA2*	5683					-1.6		-1.6				7	42916857	42932223
2099	*PSMA3*	5684					-1.6		-1.6				14	58244831	58272012
2100	*PSMB10*	5699	-3.4			-1.7							16	67934502	67937087
2101	*PSMB2*	5690					-2.0		-2.5				1	35599544	35641844
2102	*PSMB4*	5692			2.2	1.6							1	151399534	151401944
2103	*PSMB5*	5693					-2.4		-2.5				14	23016543	23035230
2104	*PSMB8*	5696	5.4									-1.6	6	32840717	32844703
2105	*PSMD11*	5717	-5.0			2.6							17	32444261	32483318
2106	*PSMD13*	5719	3.1						-1.6				11	236546	252984
2107	*PSMG1*	8624					-1.5		-1.6				21	39174769	39183851
2108	*PSMG3*	84262			1.5	3.6							7	1567330	1571005
2109	*PSPH*	5723	-4.8		-2.4								7	56011051	56051604
2110	*PSTPIP1*	9051					3.3		3.3				15	76993359	77037332
2111	*PSTPIP2*	9050					3.3		3.2				18	45983536	46072272
2112	*PTAFR*	5724	-2.6						1.8				1	28147166	28193936
2113	*PTCHD4*	442213	-3.4		-2.7								6	47878028	48068689

2114	*PTGR1*	22949			-2.0	-4.8							9	111549722	111599855
2115	*PTK2*	5747			1.6			2.2					8	140657900	141002216
2116	*PTK2B*	2185					3.1		2.7				8	27311482	27459391
2117	*PTPMT1*	114971	-4.9	-1.9									11	47565430	47573461
2118	*PTPN6*	5777					2.6		2.6				12	6946468	6961316
2119	*PTPRB*	5787	-5.5							-2.7			12	70515866	70637440
2120	*PTPRO*	5800					1.5	1.6					12	15322397	15597399
2121	*PTRH1*	138428	-5.8		-2.3								9	127692978	127724873
2122	*PUM1*	9698					-1.6		-1.6				1	30931506	31065991
2123	*PUM3*	9933				4.3		1.9					9	2720469	2844241
2124	*PURB*	5814	-3.8				1.6						7	44876293	44885361
2125	*PXN*	5829				4.2	1.9						12	120210439	120265771
2126	*PYCARD*	29108			-5.2		1.9						16	31201485	31203450
2127	*PYCR1*	5831	3.2		-4.1								17	81932384	81942412
2128	*QARS*	5859		-2.2						-1.8			3	49095932	49105135
2129	*QPCT*	25797			-3.8	-3.2							2	37344574	37373322
2130	*QPRT*	23475	-3.6		-2.9								16	29679008	29698699
2131	*QSER1*	79832					-2.3		-2.2				11	32893178	32993316
2132	*RAB12*	201475	-2.2		2.1								18	8609445	8639381
2133	*RAB18*	22931					-2.0		-1.8				10	27504174	27542237
2134	*RAB1A*	5861					-1.8		-1.6				2	65070701	65130106
2135	*RAB27B*	5874	-5.2	2.3									18	54717860	54895516
2136	*RAB29*	8934					2.0		1.8				1	205767986	205775460
2137	*RAB4A*	5867		2.6				2.1					1	229271062	229305894
2138	*RAB4B*	53916	-3.3			2.8							19	40778216	40796938
2139	*RAB8A*	4218					2.8		2.5				19	16111629	16134234
2140	*RABGAP1L*	9910					2.2		2.6				1	174159410	174995308
2141	*RABIF*	5877			1.8	4.4							1	202878957	202889135
2142	*RACGAP1*	29127			4.5	3.5							12	49976923	50033136
2143	*RAD1*	5810								1.7	1.5		5	34905264	34918989
2144	*RALB*	5899			1.9						-2.7		2	120240064	120294713
2145	*RANBP2*	5903					-2.2		-1.7				2	108719481	108785811
2146	*RAP1GDS1*	5910	4.4		1.6								4	98261384	98443861
2147	*RAPGEF1*	2889					1.6		1.5				9	131576770	131740074
2148	*RAPGEF6*	51735					-1.5	-1.6					5	131423921	131635236
2149	*RARA*	5914					2.1		2.1				17	40309192	40357643
2150	*RASA1*	5921	-3.6						-1.8				5	87267888	87391931
2151	*RASGRP1*	10125					-1.5	-2.2					15	38488103	38565575
2152	*RASSF3*	283349					1.6		1.9				12	64610513	64697567
2153	*RASSF5*	83593					1.6		1.6				1	206507530	206589283
2154	*RBBP8*	5932					-2.0		-2.1				18	22798261	23026488
2155	*RBM34*	23029		-1.6		2.4							1	235131183	235161457
2156	*RBM4*	5936									-1.5	-2.3	11	66638617	66666682
2157	*RBP7*	116362	-3.4						3.1				1	9997206	10016020
2158	*RCSD1*	92241					2.1		2.7				1	167630093	167706249
2159	*RDX*	5962					-3.7		-3.2				11	110174880	110296722
2160	*REEP3*	221035	-5.5			-3.8							10	63521363	63625123
2161	*RER1*	11079	3.6						-1.8				1	2391775	2405444
2162	*RETSAT*	54884								-2.5		-2.3	9.377941	75035493.4	75109514.44
2163	*REV3L*	5980	-1.9			-2.6							6	111299028	111483715
2164	*RFC3*	5983			2.3	2.9							13	33818049	33966558
2165	*RFK*	55312					-2.2		-1.6				9	76385517	76394517
2166	*RGL1*	23179	-2.7					1.5					1	183636085	183928531
2167	*RGL2*	5863				-4.6	1.7						6	33291654	33299324
2168	*RGS19*	10287					2.5		2.1				20	64073181	64079988
2169	*RGS5*	8490	-2.8							-1.9			1	163111121	163321791
2170	*RGS6*	9628	-1.5	-1.6									14	71932439	72566529
2171	*RHBDL2*	54933	-4.3	-2.1									7.163636	75040976	75115023.23
2172	*RHCE*	6006	-4.2									1.6	1	25362249	25430192

2173	*RHOQ*	23433	-3.0				-1.6						2	46541806	46583121
2174	*RILPL1*	353116									-1.8	-2.0	12	123470054	123533718
2175	*RIN3*	79890					4.6		4.2				14	92513774	92688994
2176	*RIT1*	6016					1.7		1.9				1	155897808	155911404
2177	*RNASE2*	6036					2.2		2.8				14	20955452	20956436
2178	*RNASEH2A*	10535	3.0					1.6					19	12806580	12813638
2179	*RNASEH2C*	84153			-1.6	-3.8							11	65714896	65720947
2180	*RNF123*	63891					1.9		1.6				3	49689499	49721529
2181	*RNF125*	54941	-5.2		3.1								6.739394	75042689.3	75116744.72
2182	*RNF130*	55819					1.7		2.0				5	179911651	180072118
2183	*RNF141*	50862			-2.0	-3.7							11	10511678	10541230
2184	*RNF166*	115992					2.4		2.5				16	88696495	88706421
2185	*RNF17*	56163	-2.0		3.3								13	24764152	24879921
2186	*RNF213*	57674					1.6					-2.3	17	80260866	80398786
2187	*RNF214*	257160			-1.7	-4.0							11	117232625	117286445
2188	*RPA1*	6117					1.7		1.6				17	1829702	1900082
2189	*RPH3A*	22895					1.9		2.0				12	112570380	112898881
2190	*RPL10A*	4736		-1.6			-1.6						6	35468408	35470785
2191	*RPL10L*	140801		-2.0	5.8								14	46651010	46651821
2192	*RPL13*	6137		-1.8	-1.6								16	89560657	89566828
2193	*RPL13A*	23521		-1.6	-1.5								19	49487554	49492308
2194	*RPL19*	6143		-1.9	-2.4								17	39200283	39204727
2195	*RPL23A*	6147		-1.6	-2.2								17	28719393	28724359
2196	*RPL26L1*	51121			1.7				-1.5				5	172958729	172969771
2197	*RPL3*	6122		-1.8			-1.7						22	39312882	39320389
2198	*RPL5*	6125		-1.5						-1.8			1	92832025	92841924
2199	*RPP25*	54913	-2.2			-4.0							4.489706	75039605.4	75113646.03
2200	*RPS27L*	51065	-2.9					1.5					15	63125872	63158021
2201	*RPS28*	6234			-1.8	-1.7							19	8321158	8323340
2202	*RPS3*	6188		-1.6		1.7							11	75399486	75422280
2203	*RPS4Y1*	6192	3.6		9.5								Y	2841486	2932000
2204	*RPS6KA1*	6195					2.0		1.9				1	26529761	26575030
2205	*RPS6KB1*	6198	2.7		-2.3								17	59893046	59950564
2206	*RPS9*	6203	-3.6	-1.5									19	54200742	54249003
2207	*RPUSD1*	113000						1.7	1.6				16	784974	788397
2208	*RRM2*	6241								2.7		2.6	2	10122328	10131419
2209	*RSAD1*	55316			-2.2	-2.0							17	50478800	50485975
2210	*RSL1D1*	26156		-1.9		2.3							16	11833850	11851585
2211	*RSU1*	6251		2.3				1.6					10	16590611	16817528
2212	*RWDD2B*	10069			-3.8	-3.7							21	29004384	29019378
2213	*RXRA*	6256					2.8		2.6				9	134317098	134440585
2214	*RYK*	6259					-1.8		-2.0				3	134065303	134250744
2215	*S100A12*	6283					5.5		7.0				1	153373706	153375649
2216	*S100PBP*	64766	-4.5		-2.9								1	32816767	32858879
2217	*SAA1*	6288	-2.3									-1.9	11	18266174	18269977
2218	*SAMSN1*	64092							1.7		-2.5		21	14485228	14583402
2219	*SAP18*	10284								-2.2	-1.9		13	21140514	21149084
2220	*SAR1B*	51128					-1.9		-1.8				5	134601144	134649271
2221	*SARM1*	23098	-3.8	-1.6									17	28364356	28404049
2222	*SC5D*	6309					-2.2		-2.0				11	121292453	121308694
2223	*SCAF1*	58506								-1.5	-1.8		19	49642125	49658642
2224	*SCCPDH*	51097			2.5	2.1							1	246724047	246768137
2225	*SCD*	6319	-3.3			-2.1							10	100347124	100364834
2226	*SCIMP*	388325	-5.3	-1.8									17	5208961	5234860
2227	*SDCBP2*	27111			-1.5	-1.8							20	1309909	1329239
2228	*SDF2L1*	23753		-1.8	-2.9								22	21642261	21644298
2229	*SDHA*	6389			-3.9	-3.0							5	218241	256700
2230	*SEC13*	6396	3.7									-2.1	3	10293131	10321178
2231	*SEC14L1*	6397					2.1		2.4				17	77086716	77217101

2232	*SEC22B*	9554	-2.5				-1.6						1	120150898	120176515
2233	*SEC23A*	10484					-2.5		-2.5				14	39031919	39109646
2234	*SEC24D*	9871	-3.0				-1.6						4	118722823	118838683
2235	*SEC31A*	22872					-1.8		-1.7				4	82818661	82901166
2236	*SECTM1*	6398					2.0		1.8				17	82321024	82334074
2237	*SEL1L*	6400	3.5				-1.8						14	81471549	81533861
2238	*SEL1L3*	23231					-2.4		-2.5				4	25747427	25863760
2239	*SELENBP1*	8991	4.9	1.7									1	151364302	151372733
2240	*SELK*	58515	3.3				-1.8						3	53884410	53891988
2241	*SELPLG*	6404					3.1		2.7				12	108622277	108633959
2242	*SEMA4A*	64218					1.9		2.0				1	156147366	156177752
2243	*SEMA4D*	10507					4.0		4.1				9	89360787	89498130
2244	*SEMA4G*	57715			3.0	3.4							10	100969518	100985871
2245	*SEMA5A*	9037			-4.7	-5.1							5	9035026	9546075
2246	*SENP6*	26054					1.7		1.8				6	75601509	75718278
2247	*SEPP1*	6414			4.6	4.3							5	42799880	42887392
2248	*SEPW1*	6415					-2.0		-2.0				19	47778572	47784686
2249	*SERINC5*	256987				-2.3			-1.5				5	80111651	80256079
2250	*SERPINA3*	12	-4.2		5.6								14	94592058	94624646
2251	*SERPINB1*	1992	-4.4				1.8						6	2832332	2842006
2252	*SERPINB9*	5272	-6.2								-2.2		6	2887266	2903280
2253	*SERPINE1*	5054	-6.1					4.1					7	101127089	101139266
2254	*SERPINF1*	5176	-4.0		-3.4								17	1761959	1777574
2255	*SESN2*	83667	3.0		-3.3								1	28259527	28282491
2256	*SESTD1*	91404					1.5	2.6					2	179101692	179264790
2257	*SETD8*	387893			2.6	3.1							12	123383773	123409358
2258	*SETX*	23064					1.7		1.8				9	132261356	132354985
2259	*SF3B4*	10262			2.4	1.8							1	149923317	149928344
2260	*SFN*	2810	-6.3						-4.1				1	26863138	26864457
2261	*SFR1*	119392	-2.6		-2.5								10	104122058	104126385
2262	*SFT2D2*	375035		-2.3		-4.5							1	168225938	168253025
2263	*SFTPB*	6439	-5.4					1.5					2	85657314	85668741
2264	*SGSH*	6448					1.7		1.6				17	80206716	80220923
2265	*SH2B2*	10603					1.6		1.6				7	102285091	102321711
2266	*SH2B3*	10019					1.7		1.9				12	111405948	111451623
2267	*SH2D3C*	10044					2.2		2.3				9	127738317	127778741
2268	*SHCBP1*	79801			2.7	3.6							16	46580554	46621626
2269	*SHISA2*	387914	-2.9		-1.7								13	26044597	26051031
2270	*SIDT2*	51092	-3.3	-2.0									11	117178733	117197445
2271	*SIGLEC7*	27036					2.1		2.3				19	51142299	51153526
2272	*SIGMAR1*	10280	-2.8									-1.7	9	34634722	34637809
2273	*SIRT2*	22933			-2.5	-5.4							19	38878555	38899862
2274	*SIRT7*	51547					2.9		1.9				17	81911939	81921323
2275	*SKA1*	220134	-7.4		2.5								18	50374995	50394173
2276	*SKAP2*	8935					1.9		1.9				7	26667062	26995239
2277	*SLC10A3*	8273					2.4		1.7				X	154487306	154490690
2278	*SLC12A6*	9990	-3.0				1.6						15	34229996	34338060
2279	*SLC16A1*	6566	-3.7									1.8	1	112911847	112957013
2280	*SLC19A1*	6573					2.9		2.5				21	45493572	45544411
2281	*SLC20A2*	6575			-2.1	-3.4							8	42416475	42541926
2282	*SLC24A3*	57419	-7.7	2.3									20	19212646	19722937
2283	*SLC24A5*	283652			-4.6	-5.2							15	48120972	48142672
2284	*SLC25A17*	10478			2.1	2.4							22	40769630	40819399
2285	*SLC25A19*	60386			-3.6	-4.1							17	75272981	75289510
2286	*SLC25A23*	79085	2.9		-1.7								19	6436079	6465203
2287	*SLC25A30*	253512	-6.1		-2.1								13	45393316	45418455
2288	*SLC25A32*	81034	-2.7			5.2							8	103398635	103415189
2289	*SLC25A33*	84275	3.0		-2.6								1	9539482	9585179
2290	*SLC25A36*	55186	4.4				-1.8						3	140941830	140979933

2291	*SLC25A6*	293		-1.6								-1.6	X	1386152	1392724
2292	*SLC31A1*	1317	-3.9								1.7		9	113221562	113264492
2293	*SLC31A2*	1318							1.6		-2.0		9	113150942	113164137
2294	*SLC35F2*	54733			2.9	3.7							5.478836	75021444.1	75095398.18
2295	*SLC38A7*	55238		-1.6				1.9					16	58665109	58685104
2296	*SLC39A10*	57181			2.7	1.9							2	195575977	195737702
2297	*SLC39A6*	25800					-2.7		-2.4				18	36108532	36129385
2298	*SLC39A9*	55334					-1.6		-1.6				14	69398015	69462388
2299	*SLC3A1*	6519	-4.2		-2.5								2	44275458	44321494
2300	*SLC43A2*	124935					1.8		1.8				17	1569267	1628886
2301	*SLC43A3*	29015		-2.0	3.1								11	57406954	57427580
2302	*SLC44A1*	23446					-1.7		-1.8				9	105244622	105439171
2303	*SLC44A2*	57153					3.3		2.5				19	10602457	10644559
2304	*SLC48A1*	55652	-5.1					1.7					12	47753916	47782753
2305	*SMAD3*	4088		-1.6					-1.7				15	67063763	67195195
2306	*SMARCA1*	6594			1.7	2.6							X	129446501	129523500
2307	*SMARCA2*	6595								-2.5	-1.5		9	2015219	2193624
2308	*SMC4*	10051						1.8	-1.7				3	160399274	160434962
2309	*SMG1*	23049								-1.6	-1.6		16	18804853	18926454
2310	*SMIM3*	85027	-4.1	2.4									5	150777946	150796734
2311	*SMN2*	6607		-1.5		-3.1							5	70049612	70078522
2312	*SMPD4*	55627			-1.6	3.2							2	130151392	130182750
2313	*SMPDL3A*	10924				1.8				-2.2			6	122789049	122809720
2314	*SMU1*	55234	-3.8			4.9							9	33041764	33076659
2315	*SMUG1*	23583			2.2	9.1							12	54121277	54189008
2316	*SNN*	8303					3.8		4.3				16	11668414	11679159
2317	*SNRNP27*	11017					-1.8		-1.8				2	69893560	69905575
2318	*SNRPD2*	6633		-1.6					-1.7				19	45687454	45692569
2319	*SNURF*	8926	2.5	1.6									15	24954986	24977850
2320	*SNX10*	29887	-6.1			6.4							7	26291895	26374329
2321	*SNX15*	29907	-3.5			-2.6							11	65027408	65040572
2322	*SNX2*	6643					-1.8	-1.9					5	122774996	122830108
2323	*SNX20*	124460					2.4		2.5				16	50666300	50681353
2324	*SNX24*	28966	-2.2		2.0								5	122843439	123029354
2325	*SNX9*	51429	-5.0		-2.5								6	157700387	157945077
2326	*SOAT1*	6646	-4.1				-1.8						1	179293714	179358680
2327	*SOD1*	6647					-1.7		-1.8				21	31659622	31668931
2328	*SOGA3*	387104	-3.3		-1.9								6	127472794	127519191
2329	*SORBS2*	8470	-7.9		-2.2								4	185585444	185956652
2330	*SORD*	6652	-2.9						-1.7				15	45023104	45077185
2331	*SORL1*	6653							1.6		-2.5		11	121452203	121633693
2332	*SP1*	6667					1.9		1.8				12	53380176	53416446
2333	*SP140*	11262	-1.8	-1.9									2	230203110	230313215
2334	*SP3*	6670	2.5				1.9						2	173906459	173965702
2335	*SPAG7*	9552	3.6			-3.5							17	4959226	4967872
2336	*SPATA5*	166378	-4.6		1.6								4	122923074	123319450
2337	*SPDYE3*	441272		-1.7		4.4							7	100307702	100322196
2338	*SPIN1*	10927					-2.3		-2.2				9	88388419	88478694
2339	*SPINT2*	10653							-1.9			-2.5	19	38244035	38292614
2340	*SPRR2D*	6703	-2.7		-2.8								1	153039725	153041931
2341	*SPTAN1*	6709					-1.6		-1.9				9	128552558	128633662
2342	*SPTY2D1*	144108	-5.4		-3.0								11	18606401	18634791
2343	*SRI*	6717					-1.7		-2.0				7	88205118	88226993
2344	*SRP72*	6731	4.3				-1.5						4	56466915	56503680
2345	*SRSF10*	10772					-2.2		-2.0				1	23964804	23980927
2346	*SRSF3*	6428	2.7				-1.9						6	36594353	36605600
2347	*SSBP1*	6742					-2.0		-1.8				7	141738321	141787922
2348	*SSFA2*	6744					-1.6		-1.6				2	181891833	181930738
2349	*SSH3*	54961					1.9	2.5					21.28571	75044745.3	75118810.52

2350	*SSR2*	6746					-1.8		-1.7				1	156009048	156020959
2351	*SSR3*	6747					-2.5		-2.4				3	156540140	156555184
2352	*ST6GAL2*	84620	-6.4	2.0									2	106801600	106887108
2353	*STAB1*	23166				-5.0			1.9				3	52495338	52524495
2354	*STAG2*	10735					1.6		1.8				X	123960212	124422664
2355	*STARD3NL*	83930	3.0			2.4							7	38178222	38230671
2356	*STARD8*	9754						2.3	2.0				X	68647666	68725842
2357	*STAT2*	6773			-1.8	-2.4							12	56341597	56360155
2358	*STK38L*	23012					-2.3		-2.0				12	27243968	27325959
2359	*STK40*	83931					2.2		2.5				1	36339624	36385896
2360	*STOM*	2040	-2.6	3.0									9	121338988	121370304
2361	*STRADB*	55437			-4.1	-3.8							2	201387858	201480846
2362	*STRBP*	55342			-2.1						-1.7		9	123109500	123268576
2363	*STRN3*	29966					-2.0		-2.2				14	30893799	31026401
2364	*STX18*	53407			-4.1	-2.5							4	4415742	4542346
2365	*STXBP1*	6812			-2.7	-3.4							9	127611760	127692936
2366	*SUCO*	51430					-2.2		-1.7				1	172532349	172611833
2367	*SULT2B1*	6820	-3.0							-1.7			19	48552075	48599425
2368	*SUMF1*	285362	-2.9		-1.5								3	3700814	4467281
2369	*SUMO1*	7341					-1.7		-1.8				2	202206180	202238608
2370	*SUOX*	6821	-3.8	-1.8									12	55997180	56006641
2371	*SURF2*	6835	4.3		-1.6								9	133356552	133361169
2372	*SURF4*	6836					-1.6		-1.7				9	133361449	133376166
2373	*SVIP*	258010						2.8	1.7				11	22813799	22830299
2374	*SWT1*	54823						3.0	2.3				1.510582	75028297.4	75102284.16
2375	*SYF2*	25949								-1.5	-2.2		1	25222679	25232502
2376	*SYK*	6850	-4.5						1.7				9	90801787	90898549
2377	*SYNCRIP*	10492					-2.1		-1.8				6	85607785	85643792
2378	*SYNE1*	23345	-3.5			-2.0							6	152121684	152637801
2379	*SYNGR1*	9145			-2.0	-4.8							22	39349925	39385588
2380	*SYNM*	23336						3.2	2.2				15	99098217	99135593
2381	*SYNPO2*	171024	-4.9									-2.1	4	118850688	119061247
2382	*SYNPO2L*	79933	-4.9	-2.1									10	73644881	73663803
2383	*TACC3*	10460					3.1		1.8				4	1721490	1745176
2384	*TADA2B*	93624					2.1		2.4				4	7041899	7057952
2385	*TAF1D*	79101					-1.8		-1.6				11	93729948	93784391
2386	*TAGAP*	117289						-2.0	1.7				6	159034468	159045152
2387	*TAGLN*	6876	-9.8	-1.8									11	117199321	117204782
2388	*TAOK1*	57551	-3.4						1.5				17	29390464	29551904
2389	*TAP1*	6890	4.7								-1.9		6	32845209	32853978
2390	*TARBP2*	6895			2.2	2.6							12	53500921	53506431
2391	*TARDBP*	23435		-1.8						-1.6			1	11012344	11026420
2392	*TARM1*	441864	-1.5	-1.8									19	54069895	54081365
2393	*TAX1BP1*	8887	2.7			1.9							7	27739331	27844564
2394	*TAX1BP3*	30851		2.1				1.5					17	3662896	3668682
2395	*TBC1D1*	23216					1.5		1.6				4	37891087	38139175
2396	*TBC1D10C*	374403					2.7		3.1				11	67403915	67410089
2397	*TBC1D16*	125058			-3.7	-4.0							17	79932343	80035848
2398	*TBC1D20*	128637	-5.3		-3.5								20	435480	462553
2399	*TBC1D7*	51256			-3.8	-3.2							6	13266542	13328583
2400	*TBC1D9B*	23061					1.6	1.7					5	179862066	179907859
2401	*TBCD*	6904	-4.1	-1.7									17	82752064	82945922
2402	*TBXAS1*	6916					1.9		2.6				7	139777051	140020325
2403	*TCOF1*	6949	3.7	-1.6									5	150357639	150400308
2404	*TCTN2*	79867	-2.8			-2.3							12	123671113	123708403
2405	*TFAP2A*	7020			-3.5	-1.7							6	10393186	10419659
2406	*TFDP2*	7029	-3.8		-1.8								3	141944428	142149544
2407	*TGFBR1*	7046					-1.9		-1.6				9	99104038	99154192
2408	*THAP5*	168451			-4.5	-4.7							7	108554543	108569750

2409	*THBS2*	7058			-2.9	-2.8							6	169215780	169254044
2410	*THEM4*	117145	-4.0		-1.6								1	151873584	151909808
2411	*TIA1*	7072					-1.5		-1.5				2	70209444	70248660
2412	*TIMM50*	92609			-2.0	1.7							19	39480412	39493785
2413	*TIMP1*	7076	-2.9	2.7									X	47582313	47586789
2414	*TIMP2*	7077	-5.4						-1.7				17	78852977	78925387
2415	*TLR4*	7099					2.4		2.8				9	117704332	117716871
2416	*TLR8*	51311					2.1		2.9				X	12906620	12923169
2417	*TM4SF1*	4071			2.4						2.6		3	149369022	149377865
2418	*TM9SF2*	9375					-1.7		-1.8				13	99501417	99564006
2419	*TM9SF3*	56889					-1.8		-2.1				10	96518109	96587452
2420	*TMC6*	11322	-3.8				2.1						17	78110458	78132407
2421	*TMC8*	147138					3.1		3.2				17	78130770	78142968
2422	*TMCC3*	57458					2.4		2.1				12	94567124	94650562
2423	*TMCO1*	54499	4.1						-1.5				12.09566	75009450.9	75083347.71
2424	*TMED7*	51014					-2.3		-2.4				5	115613508	115632992
2425	*TMED9*	54732							-1.7			-1.7	5.677249	75021101.5	75095053.88
2426	*TMEM106B*	54664					-3.2		-2.8				13.60066	75018360.2	75092299.48
2427	*TMEM11*	8834			4.5	5.0							17	21197280	21214624
2428	*TMEM120B*	144404	-3.2			-2.1							12	121712752	121783001
2429	*TMEM126A*	84233					-1.9		-1.7				11	85647967	85656547
2430	*TMEM14C*	51522					-2.7		-2.8				6	10722915	10731129
2431	*TMEM154*	201799					2.6		2.0				4	152618632	152680165
2432	*TMEM158*	25907					2.9	3.5					3	45224466	45226278
2433	*TMEM167A*	153339	-3.0						-1.6				5	83052846	83077863
2434	*TMEM2*	23670			1.6	2.9							9	71683366	71816690
2435	*TMEM200B*	399474	4.2					2.3					1	29119428	29123935
2436	*TMEM204*	79652					-2.4		-2.8				16	1528688	1555580
2437	*TMEM222*	84065						2.2	1.6				1	27322145	27336400
2438	*TMEM229B*	161145	-5.4	-1.5									14	67447084	67533739
2439	*TMEM230*	29058	-4.3				-1.6						20	5068232	5113103
2440	*TMEM259*	91304	-2.8				2.1						19	1009648	1021179
2441	*TMEM261*	90871				2.7	-1.6						9	7796490	7888380
2442	*TMEM45B*	120224	-4.8								-2.5		11	129815819	129860003
2443	*TMEM71*	137835					2.0		2.8				8	132685007	132760712
2444	*TMPO*	7112					2.1		1.9				12	98515512	98550379
2445	*TMPRSS9*	360200	-2.2		2.1								19	2389771	2426239
2446	*TNFAIP8*	25816	-4.6					-1.5					5	119268692	119399688
2447	*TNFAIP8L2*	79626					1.9		2.3				1	151156629	151159749
2448	*TNFSF13B*	10673					1.8		1.7				13	108251240	108308484
2449	*TNPO3*	23534					2.0		1.6				7	128954180	129055173
2450	*TOMM40*	10452				-2.5		1.6					19	44890569	44903689
2451	*TP53INP1*	94241									-2.0	-1.6	8	94925972	94949411
2452	*TPD52*	7163					-1.9		-2.2				8	80034745	80231232
2453	*TPM2*	7169	-9.2			-3.3							9	35681992	35691020
2454	*TPM3*	7170			-1.7		1.6						1	154155304	154194648
2455	*TRADD*	8717	-4.3	-2.0									16	67154180	67160298
2456	*TRAF3IP3*	80342					2.2		2.5				1	209756032	209782320
2457	*TRAPPC10*	7109					1.6		1.5				21	44012319	44106552
2458	*TREM1*	54210					2.6		1.6				7.6	75000541.6	75074395.93
2459	*TRIB3*	57761			-6.3	-1.5							20	362835	397559
2460	*TRIM13*	10206	-3.7					1.8					13	49995888	50020481
2461	*TRIM16L*	147166	-2.1		-1.9								17	18697998	18736118
2462	*TRIM25*	7706					2.1		1.8				17	56887909	56914038
2463	*TRIM34*	53840	-5.2		1.6								11	5619764	5644398
2464	*TRIM38*	10475					1.8		2.0				6	25962802	25991226
2465	*TRIM73*	375593					1.6		1.6				7	75395063	75410996
2466	*TRMT112*	51504	3.6						-1.5				11	64316460	64318084
2467	*TROAP*	10024			4.7	4.0							12	49323236	49331731

2468	*TSC22D1*	8848		3.4				2.0					13	44432143	44577147
2469	*TSC22D4*	81628					1.7		1.6				7	100463359	100479279
2470	*TSPAN10*	83882	-6.4		-1.7								17	81637171	81648749
2471	*TSPAN14*	81619					2.5		2.0				10	80454166	80533123
2472	*TSPO*	706			-2.8	-5.9							22	43151514	43163242
2473	*TTC39A*	22996	-7.1		-3.0								1	51287258	51345116
2474	*TTC8*	123016			-3.0	-5.0							14	88824153	88881078
2475	*TTF1*	7270	2.9		2.2								9	132375548	132406851
2476	*TTYH2*	94015					2.2		2.6				17	74213514	74262020
2477	*TTYH3*	80727			-1.8	-3.9							7	2631951	2664802
2478	*TUBA1C*	84790	3.0		1.7								12	49188736	49274603
2479	*TUBB6*	84617			2.0			2.3					18	12307669	12344320
2480	*TVP23B*	51030					-1.7		-1.8				17	18780995	18806714
2481	*TWF1*	5756					-2.5		-2.5				12	43793723	43806375
2482	*TWSG1*	57045					-2.8		-2.5				18	9334767	9402420
2483	*TXNDC9*	10190					-1.8		-2.0				2	99318982	99340702
2484	*TXNL1*	9352	-3.0				-1.7						18	56597208	56651600
2485	*TYMP*	1890					2.6		2.2				22	50525752	50530056
2486	*TYMS*	7298	-4.4		4.3								18	657604	673578
2487	*TYRP1*	7306			-9.6					-2.0			9	12685439	12710290
2488	*U2SURP*	23350					-1.7		-1.9				3	142964497	143060546
2489	*UACA*	55075			1.8	-2.6							15	70654554	70763593
2490	*UBA2*	10054					-1.9		-1.9				19	34428352	34471251
2491	*UBA52*	7311									-1.5	-1.7	19	18571730	18577550
2492	*UBA6*	55236			2.5	2.2							4	67612652	67701179
2493	*UBA7*	7318					2.2		2.0				3	49805207	49813946
2494	*UBALD2*	283991	3.0		-1.5								17	76265202	76271299
2495	*UBE2E1*	7324					-1.9		-1.9				3	23805903	23891316
2496	*UBE2K*	3093	3.9		2.9								4	39698044	39782792
2497	*UBE2N*	7334	4.2		1.8								12	93405673	93442262
2498	*UBE2V2*	7336	4.9				-1.5						8	48008400	48064708
2499	*UBIAD1*	29914	-4.5		-2.6								1	11273206	11296049
2500	*UBN1*	29855					2.0		1.8				16	4846665	4882360
2501	*UBXN2B*	137886			-1.9						-1.9		8	58411264	58451501
2502	*UFC1*	51506	2.8				-1.5						1	161152776	161158856
2503	*UGCG*	7357			-3.9		-1.6						9	111896766	111935369
2504	*UHRF2*	115426					-1.6		-1.5				9	6413151	6507054
2505	*UNC119B*	84747	4.0				-1.5						12	120710435	120723640
2506	*UPF3A*	65110			-1.7	-3.1							13	114281584	114305817
2507	*UPP1*	7378				2.9	1.7						7	48088628	48108733
2508	*UQCC2*	84300	-3.5			-1.5							6	33694293	33711727
2509	*UQCRQ*	27089					-2.3		-2.2				5	132866560	132868031
2510	*USF3*	205717					1.6		1.9				3	113648385	113696646
2511	*USP11*	8237	-4.4	-1.7									X	47232690	47248328
2512	*USP15*	9958	5.7						1.8				12	62260338	62417431
2513	*VASH1*	22846		-1.7								-2.0	14	76762189	76783015
2514	*VAV1*	7409					3.1		3.0				19	6772714	6857366
2515	*VCAN*	1462									-2.3	-1.8	5	83471465	83582303
2516	*VCL*	7414		2.3	3.6								10	73995193	74121363
2517	*VDR*	7421	-6.9				2.0						12	47841537	47943048
2518	*VEGFB*	7423			-2.3	-4.0							11	64234538	64238793
2519	*VGLL4*	9686	-3.6					2.1					3	11556070	11771350
2520	*VPS13D*	55187	-2.8							-1.7			1	12230067	12512047
2521	*VPS35*	55737					-2.3		-2.4				16	46656132	46689518
2522	*VPS8*	23355	-3.4	1.7									3	184812143	185052614
2523	*VWA3B*	200403	-2.6	-2.3									2	98087116	98313299
2524	*WDR12*	55759			-2.5	-1.9							2	202874782	203014798
2525	*WDR37*	22884	-6.5						1.6				10	1049538	1132297
2526	*WDR43*	23160					-1.9		-1.6				2	28894643	28948222

2527	*WDR91*	29062	-2.9		-1.8								7	135183839	135211534
2528	*WFDC1*	58189	-3.7		-3.3								16	84294646	84329851
2529	*WFDC2*	10406	-6.8	1.6									20	45469706	45481532
2530	*WIPF1*	7456					3.2		3.0				2	174559572	174682916
2531	*WIPF2*	147179	-3.4			-2.5							17	40219304	40284136
2532	*WLS*	79971					-2.0	1.7					1	68098473	68233120
2533	*WRB*	7485	-3.7					1.7					21	39380244	39428528
2534	*WSB1*	26118	3.0		-1.6								17	27294076	27315926
2535	*WTAP*	9589			2.0		-1.6						6	159725585	159756319
2536	*XPNPEP3*	63929			4.5	4.9							22	40857077	40932815
2537	*XRCC4*	7518			2.9	4.1							5	83077498	83353787
2538	*XRN2*	22803	3.5								-1.7		20	21303304	21389827
2539	*YIF1B*	90522					2.1	1.7					19	38305104	38317273
2540	*YIPF5*	81555					-2.6		-2.6				5	144158159	144170714
2541	*YTHDC2*	64848	-2.9				-1.6						5	113513683	113595285
2542	*YTHDF2*	51441								-1.9	-1.6		1	28736621	28769775
2543	*ZBTB11*	27107					-1.6	-1.5					3	101648889	101677495
2544	*ZC3H11A*	9877			-2.2	-2.5							1	203795654	203854124
2545	*ZCRB1*	85437					-1.7		-1.7				12	42312078	42326118
2546	*ZFAND5*	7763					1.8		1.6				9	72351425	72365235
2547	*ZFC3H1*	196441					-1.6		-1.6				12	71609472	71667725
2548	*ZFP36L2*	678					1.8		1.6				2	43222402	43226609
2549	*ZFP90*	146198	-6.4		-1.7								16	68530090	68576072
2550	*ZFYVE21*	79038	-3.4						-1.5				14	103715730	103733668
2551	*ZFYVE9*	9372	-3.5	2.9									1	52142094	52346686
2552	*ZGPAT*	84619					2.2		1.7				20	63707465	63736142
2553	*ZMYM2*	7750				-3.4	-1.5						13	19958670	20091829
2554	*ZNF12*	7559					-1.5		-1.5				7	6688433	6706923
2555	*ZNF131*	7690			-2.0		-1.8						5	43065176	43192021
2556	*ZNF133*	7692			-2.3			2.3					20	18288283	18316996
2557	*ZNF207*	7756			-3.5	-3.1							17	32350117	32381886
2558	*ZNF345*	25850	-3.2								-3.0		19	36850361	36913029
2559	*ZNF426*	79088			3.2	1.7							19	9523224	9538627
2560	*ZNF461*	92283	-2.2	-1.7									19	36637192	36666853
2561	*ZNF467*	168544					4.3		4.4				7	149764182	149773479
2562	*ZNF512*	84450	-6.3			1.6							2	27582969	27623215
2563	*ZNF548*	147694	-4.9	2.1									19	57389850	57402992
2564	*ZNF550*	162972	-3.9	-1.6									19	57535257	57559863
2565	*ZNF600*	162966		-1.7	-1.9								19	52764195	52818004
2566	*ZNF655*	79027	-4.9								-1.7		7	99558406	99576453
2567	*ZNF674*	641339	-3.5		-2.1								X	46497727	46545457
2568	*ZNF706*	51123						1.7		-1.9			8	101177878	101206193
2569	*ZPR1*	8882			-3.6	-4.3							11	116773799	116788050
2570	*ZRANB2*	9406					-2.3		-2.1				1	71063291	71081297
2571	*ZRSR2*	8233					2.1		2.0				X	15790472	15823260
2572	*ZWILCH*	55055			3.2	2.2							15	66504959	66550128
2573	*ZYX*	7791					3.2		2.6				7	143381080	143391111
2574	*MARCH3*	64844									-1.8		2	159712457	159771027
2575	*SEPT5*	5413		3.8									22	19714464	19724772
2576	*SEPT7*	989								-2.5			7	35800932	35907105
2577	*SEPT8*	23176	2.4										5	132750817	132807241
2578	*SEPT9*	10801		-1.6									17	77280569	77500596
2579	*SEPT12*	124404	-2.2										16	4777669	4788521
2580	*SEP15*	9403	3.2										1	86862445	86914424
2581	*A2ML1*	144568	-3.7										12	8822472	8887001
2582	*AACS*	65985				-2.6							12	125065379	125143333
2583	*AADAT*	51166	-4.5										4	170060222	170091699
2584	*AAK1*	22848									-2.4		2	69457997	69674349
2585	*AAMDC*	28971	-5.1										11	77821109	77918432

2586	*AAR2*	25980				-4.1							20	36236459	36270918
2587	*AARD*	441376	-2.6										8	116938199	116944487
2588	*AARS2*	57505	-3.9										6	44299654	44313326
2589	*AARSD1*	80755	-3.2										17	42950526	42964498
2590	*AASS*	10157	-3.9										7	122075647	122144280
2591	*AATF*	26574	-3.1										17	36948875	37056871
2592	*ABCA1*	19	-2.1										9	104781002	104928237
2593	*ABCA13*	154664	-2.4										7	48171458	48647496
2594	*ABCA4*	24	-6.2										1	93992835	94121132
2595	*ABCA5*	23461								-1.6			17	69244311	69327244
2596	*ABCA8*	10351	-4.6										17	68867292	68955392
2597	*ABCA9*	10350	-2.5										17	68974488	69061064
2598	*ABCB10*	23456	-2.8										1	229516582	229558695
2599	*ABCB9*	23457	-7.0										12	122920951	122981649
2600	*ABCC1*	4363	-3.3										16	15949577	16143074
2601	*ABCC2*	1244			-2.4								10	99782732	99852192
2602	*ABCC6*	368	-1.9										16	16148928	16223522
2603	*ABCC9*	10060	-7.4										12	21797401	21941402
2604	*ABCF2*	10061	-2.9										7	151207837	151227230
2605	*ABCF3*	55324	3.3										3	184186023	184194012
2606	*ABCG5*	64240			3.3								2	43812472	43838865
2607	*ABHD1*	84696				-3.4							2	27123789	27130812
2608	*ABHD10*	55347	-4.3										3	111979010	111993363
2609	*ABHD17A*	81926					1.6						19	1876810	1885547
2610	*ABHD18*	80167	-2.6										4	127965306	128039711
2611	*ABHD2*	11057	-3.9										15	89087459	89202360
2612	*ABHD4*	63874			-3.5								14	22598237	22613215
2613	*ABI2*	10152						1.5					2	203328219	203447723
2614	*ABI3*	51225		-2.1									17	49210227	49223225
2615	*ABI3BP*	25890	-4.1										3	100749156	100993515
2616	*ABRACL*	58527		-1.6									6	139028682	139043302
2617	*ACAA2*	10449				-4.9							18	49782167	49813960
2618	*ACACB*	32	-2.1										12	109116595	109268226
2619	*ACAD9*	28976								-1.9			3	128879596	128916067
2620	*ACAN*	176	-1.6										15	88803443	88875354
2621	*ACAP2*	23527	-2.8										3	195274736	195443078
2622	*ACAP3*	116983		-1.5									1	1292376	1309609
2623	*ACAT2*	39			2.9								6	159760328	159779055
2624	*ACBD4*	79777	-2.7										17	45132600	45144181
2625	*ACBD7*	414149	-2.8										10	15077523	15088776
2626	*ACE*	1636	-3.9										17	63477061	63498380
2627	*ACER2*	340485		2.4									9	19409059	19452020
2628	*ACIN1*	22985	-2.2										14	23058564	23095614
2629	*ACKR2*	1238	-4.2										3	42804752	42887974
2630	*ACO1*	48	-3.4										9	32384603	32454769
2631	*ACO2*	50	3.5										22	41469125	41528989
2632	*ACOT11*	26027	-4.3										1	54542257	54639192
2633	*ACP1*	52	2.7										2	264140	278283
2634	*ACP5*	54	-2.8										19	11574660	11579008
2635	*ACPP*	55	-5.6										3	132317367	132368298
2636	*ACSBG2*	81616	-2.0										19	6135247	6193094
2637	*ACSF2*	80221		-2.3									17	50426158	50474845
2638	*ACSL5*	51703							-2.5				10	112374018	112428380
2639	*ACSL6*	23305	-3.6										5	131949973	132012243
2640	*ACSM1*	116285	-3.2										16	20623237	20698890
2641	*ACSM2A*	123876	-4.3										16	20451461	20487667
2642	*ACSM2B*	348158	-1.7										16	20536226	20576427
2643	*ACSM5*	54988	-2.3										16	20409534	20441336
2644	*ACSS1*	84532		-1.8									20	25006230	25058980

2645	*ACTB*	60			1.5								7	5527151	5563784
2646	*ACTG2*	72	-11.3										2	73892314	73919865
2647	*ACTR1A*	10121	3.7										10	102479229	102502711
2648	*ACTR1B*	10120	-3.6										2	97655963	97664107
2649	*ACTR2*	10097	3.6										2	65227753	65271253
2650	*ACTR3B*	57180		1.6									7	152759749	152855378
2651	*ACVR1*	90	-4.3										2	157736444	157875862
2652	*ACVR2A*	92	-6.1										2	147844517	147930826
2653	*ACVR2B*	93	-2.3										3	38453851	38493142
2654	*ADAL*	161823			-2.2								15	43330674	43354555
2655	*ADAM15*	8751	-4.9										1	155050566	155062775
2656	*ADAM33*	80332	-1.9										20	3667965	3682246
2657	*ADAM8*	101					3.0						10	133262403	133276868
2658	*ADAMTS13*	11093	-3.0										9	133414358	133459402
2659	*ADAMTS17*	170691	-4.4										15	99971589	100342005
2660	*ADAMTS18*	170692	-5.6										16	77247813	77435114
2661	*ADAMTS2*	9509	-1.8										5	179110851	179345430
2662	*ADAMTS4*	9507	-2.8										1	161184308	161199056
2663	*ADAMTSL2*	9719	-2.6										9	133532164	133575519
2664	*ADAMTSL3*	57188	-3.3										15	83654086	84039842
2665	*ADAR*	103				-1.6							1	154582062	154627999
2666	*ADAT1*	23536	-3.7										16	75596981	75623300
2667	*ADCY1*	107	-2.1										7	45574140	45723116
2668	*ADCY10*	55811	-5.6										1	167809388	167914215
2669	*ADD3*	120	4.7										10	109996368	110135565
2670	*ADGRA2*	25960			-1.9								8	37784191	37844896
2671	*ADGRE5*	976					2.3						19	14380501	14408725
2672	*ADGRF1*	266977	-6.5										6	46997703	47042363
2673	*ADGRF5*	221395	-2.0										6	46852512	46954943
2674	*ADH1C*	126		-1.9									4	99336492	99353027
2675	*ADI1*	55256		1.8									2	3497361	3519736
2676	*ADIPOQ*	9370	-5.9										3	186842690	186858463
2677	*ADIPOR1*	51094	-3.7										1	202940823	202958572
2678	*ADIRF*	10974	-8.4										10	86968192	86983934
2679	*ADK*	132	-3.3										10	74151185	74709303
2680	*ADM*	133	-3.3										11	10304680	10307397
2681	*ADM2*	79924	-4.9										22	50481556	50486440
2682	*ADPRHL2*	54936	3.5										6.993939	75041661.3	75115711.83
2683	*ADRA1A*	148	-2.6										8	26748150	26867273
2684	*ADRB1*	153		-1.5									10	114044056	114046908
2685	*ADRM1*	11047				4.4							20	62302093	62308862
2686	*ADTRP*	84830	-4.2										6	11712054	11807046
2687	*AEBP1*	165	-10.6										7	44104361	44114562
2688	*AEN*	64782	2.8										15	88621296	88632282
2689	*AFAP1*	60312	-3.1										4	7758714	7939926
2690	*AFG3L2*	10939	-3.3										18	12328944	12377314
2691	*AFMID*	125061	-3.8										17	78187317	78207701
2692	*AGA*	175					-1.7						4	177430770	177442503
2693	*AGAP2*	116986	-3.5										12	57723761	57742157
2694	*AGBL4*	84871	-3.6										1	48532855	50023913
2695	*AGBL5*	60509	3.7										2	27042364	27070622
2696	*AGER*	177			2.8								6	32180968	32184324
2697	*AGMAT*	79814	-4.7										1	15572353	15585110
2698	*AGO1*	26523	-4.1										1	35869808	35930528
2699	*AGPS*	8540	-4.3										2	177392644	177543836
2700	*AGR3*	155465	2.3										7	16859405	16881987
2701	*AHCY*	191							-1.5				20	34280268	34311802
2702	*AHCYL1*	10768					-1.5						1	109984686	110023741
2703	*AHNAK2*	113146	-6.6										14	104937244	104978357

2704	*AHSA2*	130872	-4.3										2	61177418	61191203
2705	*AHSP*	51327	5.7										16	31527864	31528803
2706	*AICDA*	57379	-3.9										12	8602166	8612871
2707	*AIG1*	51390	-5.5										6	143060496	143340304
2708	*AIPL1*	23746	-5.5										17	6393693	6435199
2709	*AJUBA*	84962	-1.9										14	22971174	22982642
2710	*AK3*	50808					-1.7						9	4709559	4742043
2711	*AK9*	221264	-3.7										6	109492856	109691217
2712	*AKAP1*	8165	4.0										17	57085092	57121349
2713	*AKAP11*	11215		-1.8									13	42272153	42323267
2714	*AKAP5*	9495	-4.1										14	64465499	64474503
2715	*AKAP6*	9472	-1.6										14	32329273	32837681
2716	*AKAP8*	10270	-5.7										19	15353385	15379798
2717	*AKIP1*	56672	-2.9										11	8911139	8920084
2718	*AKR1B1*	231	2.4										7	134442350	134459284
2719	*AKR1B10*	57016	-3.9										7	134527592	134541408
2720	*AKR1C2*	1646	-3.9										10	4922564	5135226
2721	*AKR1D1*	6718	-4.3										7	138002324	138117986
2722	*AKR1E2*	83592	-4.8										10	4786629	4848062
2723	*AKT1*	207	3.2										14	104769349	104795751
2724	*AKT2*	208	-2.9										19	40230317	40285536
2725	*AKTIP*	64400									-1.9		16	53491040	53504411
2726	*ALAS2*	212										1.7	X	55009055	55031064
2727	*ALDH18A1*	5832	4.1										10	95605929	95656706
2728	*ALDH1A2*	8854										1.8	15	57953424	58497866
2729	*ALDH1A3*	220	-6.5										15	100877714	100916626
2730	*ALDH1B1*	219				3.5							9	38392664	38398661
2731	*ALDH1L1*	10840	-3.5										3	126103562	126197994
2732	*ALDH1L2*	160428	-3.4										12	105019784	105084577
2733	*ALDH3A1*	218	-1.7										17	19737984	19748943
2734	*ALDH3B1*	221	-5.4										11	68008578	68029282
2735	*ALDH3B2*	222	-3.9										11	67662162	67681200
2736	*ALDH4A1*	8659	-2.8										1	18871430	18902781
2737	*ALDH5A1*	7915	-3.2										6	24494852	24537207
2738	*ALDH7A1*	501	-4.5										5	126541841	126595418
2739	*ALDH9A1*	223	4.9										1	165662216	165698863
2740	*ALG1*	56052	-3.3										16	5033960	5087379
2741	*ALG10*	84920				-3.0							12	34022281	34029694
2742	*ALG11*	440138	-2.0										13	52012398	52029664
2743	*ALKBH3*	221120	-3.8										11	43880811	43920266
2744	*ALKBH4*	54784	-3.3										3.693122	75024528.1	75098496.87
2745	*ALKBH5*	54890			-3.7								8.563235	75036178.7	75110203.04
2746	*ALPI*	248										2.5	2	232456123	232460032
2747	*ALPL*	249	-3.8										1	21509372	21578412
2748	*ALPP*	250	-2.5										2	232378534	232382889
2749	*ALPPL2*	251	-2.9										2	232406843	232410714
2750	*ALS2CR11*	151254	-2.1										2	201487425	201619178
2751	*AMBRA1*	55626	-3.6										11	46396414	46594125
2752	*AMDHD2*	51005				-3.8							16	2520357	2531422
2753	*AMER3*	205147	-1.9										2	130755435	130768134
2754	*AMIGO2*	347902	-6.7										12	47075707	47079951
2755	*AMOTL1*	154810	-4.9										11	94706431	94876753
2756	*AMPD3*	272	-3.2										11	10308313	10507579
2757	*AMPH*	273	-1.8										7	38383704	38631567
2758	*AMZ2*	51321	3.4										17	68247574	68257164
2759	*ANAPC10*	10393				2.5							4	144967112	145098541
2760	*ANAPC15*	25906	-4.5										11	72106378	72112780
2761	*ANG*	283	2.7										14	20684177	20698971
2762	*ANGEL1*	23357										2.0	14	76786178	76826246

2763	*ANGEL2*	90806	-2.7										1	212992182	213015826
2764	*ANGPT2*	285								-2.2			8	6499651	6563409
2765	*ANGPTL4*	51129	2.4										19	8363289	8374373
2766	*ANKK1*	255239	-2.7										11	113387791	113400418
2767	*ANKLE1*	126549	-3.1										19	17281645	17287646
2768	*ANKLE2*	23141	3.3										12	132725503	132761888
2769	*ANKMY2*	57037	-3.0										7	16599776	16645817
2770	*ANKRD17*	26057	5.3										4	73073376	73258798
2771	*ANKRD20A2*	441430		-1.6									9	40223285	40266392
2772	*ANKRD35*	148741	-2.8										1	145866560	145885866
2773	*ANKRD36*	375248			2.0								2	97113496	97264521
2774	*ANKRD36B*	57730			2.4								2	97492663	97589965
2775	*ANKRD40*	91369	-4.3										17	50693190	50707924
2776	*ANKRD45*	339416	-3.1										1	173609561	173669862
2777	*ANKRD50*	57182					-2.0						4	124664052	124712732
2778	*ANKRD65*	441869	-5.8										1	1418420	1421769
2779	*ANKS1A*	23294	-4.5										6	34889265	35091413
2780	*ANKS3*	124401	-3.3										16	4696510	4734378
2781	*ANKS6*	203286	-2.2										9	98731329	98796965
2782	*ANKZF1*	55139	-3.0										2	219229757	219236669
2783	*ANO1*	55107	-5.6										11	70078302	70189528
2784	*ANOS1*	3730									-2.0		X	8528874	8732187
2785	*ANP32E*	81611	-3.9										1	150218417	150236156
2786	*ANTXR2*	118429									-1.7		4	79901149	80125454
2787	*ANXA1*	301	-4.1										9	73151757	73170393
2788	*ANXA6*	309			-1.9								5	151100712	151157882
2789	*ANXA9*	8416	-4.5										1	150982017	150995634
2790	*AOC1*	26	-4.3										7	150824627	150861504
2791	*AOC3*	8639									-2.6		17	42851184	42858130
2792	*AP1M2*	10053	3.5										19	10572671	10587315
2793	*AP1S3*	130340	-5.1										2	223751686	223838027
2794	*AP2A1*	160	-4.0										19	49766968	49807113
2795	*AP2B1*	163							-1.7				17	35578046	35726409
2796	*AP3D1*	8943	5.4										19	2100988	2164465
2797	*AP3M1*	26985	-3.5										10	74120255	74151063
2798	*AP4B1*	10717	-3.2										1	113894748	113905201
2799	*AP4E1*	23431	-6.8										15	50908672	51005900
2800	*AP4M1*	9179	-3.4										7	100101549	100110345
2801	*AP4S1*	11154	-6.2										14	31025106	31096450
2802	*APAF1*	317	-4.5										12	98645141	98735433
2803	*APBA1*	320	-1.9										9	69427530	69672306
2804	*APBB1*	322				-4.4							11	6395124	6419414
2805	*APBB2*	323			-2.2								4	40810027	41216714
2806	*APOA1BP*	128240		-1.9									1	156591762	156594299
2807	*APOBEC3F*	200316	-4.3										22	39040604	39053910
2808	*APOBEC3H*	164668	-2.1										22	39097224	39104067
2809	*APOC2*	344			-5.5								19	44942238	44949565
2810	*APOC4*	346			-5.5								19	44942238	44945496
2811	*APOE*	348			-2.3								19	44905754	44909393
2812	*APOL1*	8542	-6.0										22	36253010	36267530
2813	*APOL2*	23780	-5.2										22	36226203	36239954
2814	*APOLD1*	81575	-5.1										12	12725917	12829975
2815	*APOM*	55937										1.5	6	31652416	31658210
2816	*APPBP2*	10513	-4.2										17	60443149	60526219
2817	*APRT*	353	-3.3										16	88809339	88811944
2818	*APTX*	54840				3.8							X	75030010.8	75104005.66
2819	*AQP3*	360								-3.7			9	33441154	33447611
2820	*AQP6*	363	-2.9										12	49967194	49977139
2821	*AQP7*	364	-7.9										9	33383179	33402682

2822	*AR*	367	2.4										X	67544032	67730619
2823	*AREL1*	9870	-4.7										14	74653437	74713115
2824	*ARF1*	375										-1.9	1	228082660	228099212
2825	*ARF3*	377		3.2									12	48935723	48957551
2826	*ARFIP2*	23647	-4.8										11	6474683	6481479
2827	*ARGFX*	503582	-6.8										3	121570704	121586634
2828	*ARHGAP1*	392	-5.4										11	46677080	46700615
2829	*ARHGAP10*	79658	-1.6										4	147732063	148072780
2830	*ARHGAP11A*	9824	-4.4										15	32615144	32639949
2831	*ARHGAP21*	57584						2.4					10	24583609	24723668
2832	*ARHGAP22*	58504	-1.8										10	48446034	48656265
2833	*ARHGAP23*	57636	-1.9										17	38419280	38512392
2834	*ARHGAP28*	79822	-5.3										18	6729718	6915716
2835	*ARHGAP29*	9411									-3.3		1	94148988	94275068
2836	*ARHGEF10L*	55160	-2.2										1	17539835	17697874
2837	*ARHGEF12*	23365			-1.6								11	120336914	120489936
2838	*ARHGEF15*	22899	-3.1										17	8310241	8322516
2839	*ARHGEF19*	128272	-1.9										1	16197854	16212609
2840	*ARHGEF35*	445328		-1.8									7	144186083	144195655
2841	*ARHGEF37*	389337	3.7										5	149551947	149634968
2842	*ARHGEF39*	84904	-3.3										9	35658875	35675866
2843	*ARHGEF5*	7984	-3.8										7	144355288	144380632
2844	*ARHGEF6*	9459						-1.5					X	136665547	136782088
2845	*ARID1A*	8289	3.8										1	26696033	26782104
2846	*ARID3A*	1820	-3.6										19	925781	975934
2847	*ARID4A*	5926				-4.0							14	58298385	58373887
2848	*ARIH2OS*	646450	-5.1										3	48917788	48919385
2849	*ARL1*	400	-4.1										12	101393120	101407820
2850	*ARL10*	285598	-4.0										5	176365468	176401865
2851	*ARL14EP*	120534	3.1										11	30323051	30338227
2852	*ARL4C*	10123	4.3										2	234493041	234497053
2853	*ARL5A*	26225			-2.5								2	151788984	151828492
2854	*ARL6IP4*	51329		-1.7									12	122980060	122982913
2855	*ARL8B*	55207	-3.9										3	5122220	5180912
2856	*ARMC7*	79637				-3.5							17	75109952	75130265
2857	*ARMC9*	80210	-4.4										2	231198546	231374837
2858	*ARMCX5*	64860				-4.6							X	102599168	102604159
2859	*ARMCX6*	54470		2.1									11.5747	75006366.9	75080249.01
2860	*ARNT2*	9915	-4.6										15	80404350	80597937
2861	*ARNTL2*	56938	-6.2										12	27332854	27425289
2862	*ARPC1A*	10552	3.5										7	99325898	99388164
2863	*ARPC5*	10092									-1.9		1	183620846	183635757
2864	*ARPIN*	348110	-2.8										15	89895006	89912956
2865	*ARPP19*	10776	-3.3										15	52547045	52569883
2866	*ARRDC4*	91947	-3.1										15	97960698	97973838
2867	*ARSA*	410	-3.3										22	50622754	50628173
2868	*ARSD*	414		-2.1									X	2903970	2929351
2869	*ARSG*	22901	-1.9										17	68259182	68422731
2870	*ARSK*	153642	-5.7										5	95555074	95605064
2871	*ART3*	419	-2.4										4	76011184	76112802
2872	*ARVCF*	421			1.7								22	19969896	20016808
2873	*ASB11*	140456	-4.7										X	15281697	15315656
2874	*ASB16*	92591	-2.8										17	44170447	44179083
2875	*ASB3*	51130	2.8										2	53532672	53787610
2876	*ASB4*	51666			-3.0								7	95478444	95540232
2877	*ASB8*	140461	-3.1										12	48147788	48181213
2878	*ASF1A*	25842				-2.5							6	118894220	118909167
2879	*ASH1L*	55870									-1.7		1	155335268	155562807
2880	*ASNA1*	439	3.2										19	12737139	12748323

2881	*ASRGL1*	80150		-1.7									11	62337448	62393412
2882	*ATAD3A*	55210		-1.6									1	1512151	1534687
2883	*ATAD3C*	219293	-4.0										1	1449689	1470158
2884	*ATCAY*	85300	-5.7										19	3879864	3928079
2885	*ATF6B*	1388	-3.2										6	32098176	32128253
2886	*ATF7IP2*	80063	-2.3										16	10326434	10483638
2887	*ATG10*	83734	-6.5										5	81972025	82276857
2888	*ATG13*	9776						1.7					11	46617527	46674818
2889	*ATG5*	9474	-6.3										6	106184476	106325820
2890	*ATG7*	10533	-3.3										3	11272309	11557665
2891	*ATG9A*	79065	-3.5										2	219209772	219229717
2892	*ATHL1*	80162					2.1						11	289135	296107
2893	*ATL1*	51062	-4.0										14	50532509	50633068
2894	*ATL3*	25923								-2.3			11	63624087	63671921
2895	*ATOH8*	84913	-1.6										2	85751344	85788066
2896	*ATOX1*	475						1.6					5	151742316	151772532
2897	*ATP10B*	23120	-4.2										5	160563120	160852214
2898	*ATP11C*	286410					-1.7						X	139726346	139945276
2899	*ATP13A1*	57130	2.8										19	19645198	19663693
2900	*ATP1A4*	480	-6.0										1	160151570	160186977
2901	*ATP1B2*	482	-5.1										17	7646627	7657768
2902	*ATP1B3*	483		-1.7									3	141876124	141926514
2903	*ATP1B4*	23439	-4.0										X	120362085	120383249
2904	*ATP2B1*	490	5.2										12	89588049	89709300
2905	*ATP2C1*	27032						2.0					3	130850595	131016712
2906	*ATP5B*	506		-1.6									12	56638175	56646068
2907	*ATP5C1*	509			1.7								10	7788129	7807815
2908	*ATP5D*	513				-3.1							19	1241746	1244826
2909	*ATP5F1*	515	3.0										1	111448864	111462773
2910	*ATP5G1*	516		-1.5									17	48892765	48895871
2911	*ATP5J2*	9551										-2.3	7	99448475	99466331
2912	*ATP5SL*	55101	2.4										19	41431318	41440717
2913	*ATP6AP1*	537	2.7										X	154428632	154436516
2914	*ATP6AP1L*	92270	-3.4										5	82279462	82386977
2915	*ATP6V0A1*	535			-1.9								17	42458844	42522611
2916	*ATP6V0A2*	23545	-2.9										12	123712318	123761755
2917	*ATP6V0D1*	9114							1.5				16	67438014	67481237
2918	*ATP6V0D2*	245972	-2.8										8	85987323	86154228
2919	*ATP6V1B2*	526	3.1										8	20197367	20226819
2920	*ATP6V1C1*	528			-1.8								8	103021063	103073051
2921	*ATP7A*	538	-4.2										X	77910656	78050395
2922	*ATP8A1*	10396	-2.9										4	42408373	42657105
2923	*ATP8B4*	79895	-3.4										15	49858238	50182817
2924	*ATP9A*	10079		3.4									20	51596514	51768634
2925	*ATP9B*	374868	-3.3										18	79069285	79378283
2926	*ATPAF1*	64756				-3.2							1	46632737	46673867
2927	*ATPAF2*	91647				-4.6							17	17977409	18039209
2928	*ATRN*	8455						1.7					20	3471040	3651122
2929	*ATRX*	546	3.1										X	77504878	77786269
2930	*ATXN1*	6310								-1.5			6	16299112	16761491
2931	*ATXN10*	25814		-1.5									22	45671798	45845307
2932	*ATXN2*	6311	-4.4										12	111452214	111599676
2933	*ATXN3*	4287	-5.0										14	92038652	92106621
2934	*ATXN7*	6314							2.0				3	63864557	64003462
2935	*ATXN7L3B*	552889	-3.5										12	74537827	74545430
2936	*AVEN*	57099	-3.1										15	33866227	34039176
2937	*AVIL*	10677	-6.4										12	57797376	57818704
2938	*AVL9*	23080	-3.3										7	32495426	32588721
2939	*AZGP1*	563	-6.0										7	99966720	99976157

2940	*AZI2*	64343	3.2										3	28315003	28349127
2941	*AZIN2*	113451		-2.2									1	33081104	33120530
2942	*B2M*	567				-1.7							15	44711477	44718877
2943	*B3GALT6*	126792				-2.9							1	1232265	1235041
2944	*B3GNT6*	192134	-3.2										11	77034398	77041973
2945	*B3GNTL1*	146712	-3.2										17	82942155	83051810
2946	*B4GALT4*	8702						1.5					3	119211732	119241103
2947	*B4GALT5*	9334	-3.2										20	49632945	49713878
2948	*B4GAT1*	11041			-1.9								11	66345372	66347692
2949	*BABAM1*	29086			2.9								19	17267350	17281249
2950	*BACH2*	60468	-2.5										6	89926529	90296908
2951	*BAG5*	9529	-5.0										14	103556544	103562831
2952	*BAHCC1*	57597						2.0					17	81395475	81466332
2953	*BAIAP2L1*	55971				-1.5							7	98291651	98401068
2954	*BANP*	54971		-1.8									X	75046116	75120187.72
2955	*BATF2*	116071	-2.1										11	64987943	64997045
2956	*BAZ1A*	11177						-1.6					14	34752731	34875647
2957	*BAZ2B*	29994					-1.6						2	159318979	159616692
2958	*BBS1*	582	-2.9										11	66510606	66533627
2959	*BBS2*	583	-2.7										16	56466836	56520283
2960	*BBS7*	55212	-1.5										4	121824440	121870497
2961	*BBS9*	27241								-2.0			7	33129244	33606068
2962	*BBX*	56987	3.5										3	107522936	107811324
2963	*BCAM*	4059	-3.6										19	44809059	44821420
2964	*BCAS1*	8537	-5.0										20	53936777	54070594
2965	*BCAS2*	10286	3.7										1	114567557	114581639
2966	*BCAT2*	587				-5.8							19	48795062	48811029
2967	*BCKDHA*	593			-2.2								19	41397460	41431345
2968	*BCKDHB*	594	-2.2										6	80106647	80346270
2969	*BCKDK*	10295			-2.1								16	31106107	31112791
2970	*BCL2L1*	598		1.8									20	31664452	31723989
2971	*BCL2L15*	440603	-5.1										1	113878168	113887547
2972	*BCL6B*	255877	-2.5										17	7023020	7030290
2973	*BCL7C*	9274				-3.3							16	30833626	30894960
2974	*BCO2*	83875	-6.3										11	112175467	112224699
2975	*BCORL1*	63035	-1.7										X	129981107	130058083
2976	*BDH1*	622	-3.1										3	197509783	197573323
2977	*BDH2*	56898	-1.9										4	103079435	103099883
2978	*BET1L*	51272			-3.1								11	167784	207428
2979	*BEX2*	84707			-3.7								X	103309346	103311046
2980	*BFAR*	51283							-1.6				16	14632815	14669236
2981	*BGN*	633	-5.8										X	153494939	153509554
2982	*BHLHE41*	79365			-3.9								12	26120026	26125127
2983	*BHMT2*	23743	-5.0										5	79069717	79089466
2984	*BIN1*	274	2.5										2	127048027	127107355
2985	*BIN3*	55909			1.7								8	22620418	22669148
2986	*BIRC6*	57448	-3.7										2	32357028	32618899
2987	*BLCAP*	10904							-2.1				20	37492472	37527931
2988	*BLMH*	642								-2.1			17	30248195	30292056
2989	*BLOC1S1*	2647	-4.2										12	55716037	55720087
2990	*BLVRA*	644	-4.3										7	43758680	43807342
2991	*BLZF1*	8548	-3.6										1	169367970	169396540
2992	*BMP1*	649	-2.0										8	22164736	22212326
2993	*BMP6*	654		1.9									6	7726797	7881422
2994	*BMP7*	655	-4.8										20	57168748	57266629
2995	*BMP8A*	353500	-6.2										1	39491646	39525935
2996	*BMP8B*	656	-3.8										1	39757182	39788861
2997	*BMPR1B*	658	-4.9										4	94757968	95158448
2998	*BMS1*	9790		-1.9									10	42782801	42834937

2999	*BMX*	660			6.3								X	15464246	15556529
3000	*BNIP2*	663									-2.6		15	59659146	59689534
3001	*BNIP3L*	665									-2.1		8	26382898	26505636
3002	*BOLA1*	51027						1.6					1	149887890	149900798
3003	*BORA*	79866	-2.2										13	72727749	72756198
3004	*BORCS7*	119032					-1.6						10	102854223	102864961
3005	*BPIFB1*	92747	-1.9										20	33273480	33309878
3006	*BPIFB2*	80341	-5.0										20	33007600	33023709
3007	*BPNT1*	10380	-3.5										1	220057482	220090462
3008	*BRD1*	23774				-3.8							22	49773283	49827512
3009	*BRD3*	8019			-1.6								9	134030305	134068535
3010	*BRD4*	23476	3.3										19	15235519	15332545
3011	*BRD7*	29117	2.9										16	50313487	50368934
3012	*BRD8*	10902	3.4										5	138139766	138178986
3013	*BRD9*	65980	3.9										5	850291	892824
3014	*BRDT*	676	-3.8										1	91949371	92014426
3015	*BRE*	9577	-3.7										2	27889941	28338901
3016	*BRF2*	55290			-1.9								8	37843268	37849904
3017	*BRIP1*	83990	-6.4										17	61681266	61863521
3018	*BRK1*	55845		2.0									3	10115592	10127190
3019	*BRMS1*	25855	2.4										11	66337333	66345125
3020	*BRSK1*	84446	-1.8										19	55282072	55312533
3021	*BRWD1*	54014				-3.0							21	39184176	39321559
3022	*BSPRY*	54836	3.0										0.915344	75029325.4	75103317.06
3023	*BTBD19*	149478	-2.9										1	44808482	44815585
3024	*BTBD3*	22903	-2.8										20	11890723	11926609
3025	*BTF3*	689				1.9							5	73498408	73505635
3026	*BTN2A1*	11120									-1.7		6	26457904	26476621
3027	*BTN3A3*	10384									-2.5		6	26440472	26453415
3028	*BTNL3*	10917	-2.1										5	180988845	181006727
3029	*BUD31*	8896	5.9										7	99408641	99419616
3030	*BVES*	11149	-4.7										6	105096822	105137174
3031	*C10orf67*	256815	-4.6										10	23267195	23344845
3032	*C10orf76*	79591	-3.4										10	101845599	102056193
3033	*C10orf82*	143379	-6.1										10	116663696	116670264
3034	*C11orf31*	280636		-1.8									11	57741250	57743554
3035	*C11orf68*	83638			-2.4								11	65916808	65919117
3036	*C11orf70*	85016	-4.2										11	102047443	102084560
3037	*C11orf71*	54494	-2.9										11.92201	75008422.9	75082314.81
3038	*C11orf74*	119710	-2.8										11	36594493	36659290
3039	*C11orf85*	283129	-1.6										11	64937517	64972085
3040	*C11orf88*	399949	-1.8										11	111514785	111537031
3041	*C12orf10*	60314									-2.5		12	53299686	53307177
3042	*C12orf4*	57102	-2.8										12	4487728	4538508
3043	*C12orf43*	64897	-3.8										12	121000486	121016502
3044	*C12orf50*	160419	-4.3										12	87980035	88034037
3045	*C12orf65*	91574	-5.9										12	123232916	123257959
3046	*C14orf1*	11161	3.2										14	75649791	75661189
3047	*C14orf105*	55195	-3.4										14	57469301	57493867
3048	*C14orf142*	84520			2.3								14	93202894	93207094
3049	*C14orf159*	80017									-2.2		14	91060333	91225632
3050	*C14orf166*	51637					-1.5						14	51989475	52010691
3051	*C14orf178*	283579	-2.0										14	77760830	77769742
3052	*C14orf37*	145407	-1.9										14	57999735	58298139
3053	*C15orf43*	145645	-4.5										15	44956702	44979229
3054	*C15orf48*	84419	-4.8										15	45430529	45448761
3055	*C16orf45*	89927	-3.0										16	15434295	15625028
3056	*C16orf70*	80262	-2.6										16	67109958	67148539
3057	*C16orf72*	29035	3.3										16	9091648	9121640

3058	*C17orf104*	284071	-3.0										17	44656404	44690308
3059	*C17orf49*	124944				-1.7							17	7014495	7017525
3060	*C17orf51*	339263	-1.7										17	21524790	21574458
3061	*C17orf58*	284018			-3.7								17	67991101	67993649
3062	*C17orf75*	64149	-5.1										17	32324565	32350023
3063	*C17orf99*	100141515	-4.5										17	78146353	78166177
3064	*C19orf12*	83636	-5.7										19	29698886	29715789
3065	*C19orf18*	147685	-4.9										19	57958437	57974534
3066	*C19orf35*	374872	-5.0										19	2274622	2282176
3067	*C19orf38*	255809	-2.9										19	10836575	10869790
3068	*C19orf43*	79002	2.3										19	12730640	12734775
3069	*C19orf54*	284325	-3.4										19	40740856	40751553
3070	*C19orf60*	55049	-3.1										19	18588685	18592336
3071	*C19orf70*	125988	-5.7										19	5678421	5680896
3072	*C1orf116*	79098	-4.7										1	207018521	207032756
3073	*C1orf122*	127687							-1.5				1	37806979	37809454
3074	*C1orf194*	127003	-2.9										1	109105951	109113857
3075	*C1orf210*	149466	-3.1										1	43281883	43285617
3076	*C1orf228*	339541	-1.8										1	44674692	44725591
3077	*C1orf233*	643988						1.7					1	1598012	1600096
3078	*C1orf43*	25912			-1.8								1	154206706	154220628
3079	*C1orf53*	388722	-3.6										1	197902647	197907367
3080	*C1orf56*	54964									-2.3		22.42857	75045088	75119154.82
3081	*C1orf61*	10485	-3.6										1	156404250	156430701
3082	*C1QC*	714										-1.5	1	22643630	22648110
3083	*C1QTNF6*	114904	-3.9										22	37180167	37199385
3084	*C1RL*	51279	-6.4										12	7089587	7109273
3085	*C1S*	716	-10.2										12	6988259	7071032
3086	*C20orf194*	25943						1.6					20	3249305	3407625
3087	*C20orf24*	55969									-2.5		20	36605734	36612557
3088	*C21orf2*	755		-1.8									21	44328944	44339402
3089	*C21orf58*	54058	-3.9										21	46300181	46323875
3090	*C21orf59*	56683		-2.0									21	32592079	32612866
3091	*C21orf62*	56245	-6.8										21	32790674	32813743
3092	*C22orf39*	128977	-2.8										22	19351368	19448232
3093	*C2CD3*	26005	-2.5										11	74012714	74171210
3094	*C2CD4A*	145741	-2.1										15	62066977	62070917
3095	*C2orf15*	150590	-5.2										2	99141485	99151487
3096	*C2orf50*	130813	-8.0										2	11133053	11146790
3097	*C2orf68*	388969	-3.2										2	85606654	85612066
3098	*C2orf69*	205327		1.7									2	199911256	199955935
3099	*C2orf72*	257407				-4.1							2	231037490	231049719
3100	*C2orf83*	56918	-4.5										2	227610090	227633320
3101	*C3*	718	-9.9										19	6677704	6730562
3102	*C3orf33*	285315	-4.6										3	155762617	155806351
3103	*C3orf62*	375341	-2.5										3	49268602	49277909
3104	*C3orf67*	200844	-1.6										3	58717365	59050084
3105	*C3orf70*	285382	-4.2										3	185078050	185153014
3106	*C4BPA*	722										1.6	1	207104262	207144972
3107	*C4BPB*	725	-4.8										1	207088842	207099993
3108	*C4orf26*	152816	-5.9										4	75556048	75565885
3109	*C4orf27*	54969									-1.5		24.71429	75045773.3	75119843.42
3110	*C4orf32*	132720	-2.8										4	112145397	112195256
3111	*C4orf50*	389197	-2.2										4	389197	389197
3112	*C5*	727	-3.8										9	120952335	121050276
3113	*C5orf22*	55322			-4.3								5	31532266	31555058
3114	*C5orf24*	134553	-5.2										5	134845680	134859737
3115	*C5orf28*	64417			-3.3								5	43444252	43483893
3116	*C6orf141*	135398	-3.0										6	49550646	49561907

3117	*C6orf201*	404220	2.8										6	4079209	4130951
3118	*C6orf25*	80739		3.8									6	31718594	31726714
3119	*C6orf48*	50854	-2.6										6	31834608	31839766
3120	*C7*	730	-4.9										5	40909252	40982939
3121	*C7orf25*	79020	3.0										7	42908726	42912305
3122	*C7orf26*	79034	2.5										7	6590017	6608726
3123	*C7orf31*	136895	-3.8										7	25134697	25180356
3124	*C8orf33*	65265	-4.2										8	145052378	145056030
3125	*C8orf37*	157657	-3.4										8	95244919	95269201
3126	*C8orf44*	56260	-1.6										8	66667615	66685564
3127	*C8orf82*	414919	2.9										8	144525733	144529132
3128	*C8orf86*	389649	-5.2										8	38510834	38528662
3129	*C9orf135*	138255	-1.8										9	69820793	69906232
3130	*C9orf24*	84688	-2.0										9	34379019	34397832
3131	*C9orf64*	84267	-1.6										9	83938311	83956986
3132	*C9orf66*	157983	-2.2										9	212824	215741
3133	*C9orf85*	138241	-5.1										9	71911510	71986054
3134	*C9orf9*	11092	-3.8										9	132878027	132890201
3135	*CA1*	759	-4.0										8	85327608	85379014
3136	*CA12*	771	-4.4										15	63321378	63382161
3137	*CA14*	23632	-4.0										1	150257159	150265078
3138	*CA3*	761									1.8		8	85373436	85449040
3139	*CA4*	762							1.9				17	60149936	60170899
3140	*CA5A*	763	-2.2										16	87881546	87936529
3141	*CAAP1*	79886	-1.7										9	26840685	26892804
3142	*CAB39*	51719				-4.4							2	230712845	230821075
3143	*CABP4*	57010	-5.8										11	67452406	67460313
3144	*CACNA1C*	775	-2.0										12	1970786	2697950
3145	*CACNA2D1*	781	-2.0										7	81946444	82443798
3146	*CACNA2D4*	93589			2.2								12	1791957	1918836
3147	*CACNB2*	783	-3.5										10	18140677	18541869
3148	*CACNB3*	784									-1.8		12	48813794	48828941
3149	*CACNB4*	785	-1.6										2	151832776	152099079
3150	*CACNG7*	59284	-2.2										19	53909335	53943941
3151	*CACNG8*	59283	-7.4										19	53963040	53990215
3152	*CACYBP*	27101			2.3								1	174999163	175011715
3153	*CADM1*	23705	-3.0										11	115169218	115504957
3154	*CADPS2*	93664	3.7										7	122318425	122886759
3155	*CALCOCO2*	10241	-2.7										17	48830988	48866522
3156	*CALCRL*	10203	-2.9										2	187343129	187448460
3157	*CALML3*	810		2.6									10	5524009	5526771
3158	*CALR*	811				-1.6							19	12938578	12944489
3159	*CAMK1D*	57118	-3.9										10	12349482	12835545
3160	*CAMK2A*	815	-1.9										5	150219491	150290291
3161	*CAMK2G*	818	-5.8										10	73812501	73874591
3162	*CAMKK2*	10645	-4.4										12	121237691	121298308
3163	*CAMSAP1*	157922	3.0										9	135808487	135907228
3164	*CAND1*	55832				-1.5							12	67269281	67319951
3165	*CAPNS1*	826	1.6										19	36139575	36150353
3166	*CAPNS2*	84290	-2.8										16	55566672	55567687
3167	*CAPZA1*	829									-1.9		1	112619173	112671619
3168	*CAPZA2*	830		1.8									7	116811070	116922049
3169	*CAPZB*	832	3.7										1	19338776	19485539
3170	*CARD11*	84433	-3.6										7	2906141	3043945
3171	*CARD14*	79092	-3.0										17	80169992	80209331
3172	*CARD6*	84674	-2.3										5	40841184	40860175
3173	*CARD9*	64170	-1.7										9	136361903	136373681
3174	*CARF*	79800	-6.2										2	202912214	202987063
3175	*CARKD*	55739		-1.6									13	110615460	110639993

3176	*CARS2*	79587	3.5										13	110641412	110713603
3177	*CASD1*	64921	-2.9										7	94509219	94557019
3178	*CASP2*	835	-4.6										7	143288215	143307696
3179	*CASP4*	837									-2.6		11	104942866	104969436
3180	*CASP6*	839	-2.7										4	109688622	109703583
3181	*CASP7*	840			3.7								10	113679162	113730907
3182	*CASQ1*	844	-2.6										1	160190556	160201886
3183	*CAT*	847						-1.6					11	34438925	34472062
3184	*CATSPER2*	117155		-2.1									15	43628503	43668118
3185	*CATSPERD*	257062	-4.5										19	5720677	5778734
3186	*CBFA2T2*	9139	-4.2										20	33490075	33650036
3187	*CBFA2T3*	863	-2.7										16	88874858	88977204
3188	*CBR4*	84869	3.9										4	168863770	169010275
3189	*CBX3*	11335	2.8										7	26201162	26213356
3190	*CC2D1A*	54862	-4.4										7.154545	75032066.7	75106071.45
3191	*CC2D1B*	200014	-2.4										1	52345723	52366193
3192	*CC2D2A*	57545	-5.4										4	15469865	15601557
3193	*CCAR1*	55749	2.5										10	68721012	68792377
3194	*CCAR2*	57805			1.7								8	22604632	22621514
3195	*CCBE1*	147372	-5.3										18	59430940	59697380
3196	*CCBL1*	883	-4.1										9	128832942	128882494
3197	*CCBL2*	56267	3.3										1	88935773	88992953
3198	*CCDC115*	84317	-3.0										2	130338241	130342349
3199	*CCDC120*	90060	-4.7										X	49053572	49069857
3200	*CCDC122*	160857	-5.7										13	43823909	43879727
3201	*CCDC125*	202243	-5.1										5	69280175	69332809
3202	*CCDC126*	90693			-2.0								7	23597379	23644708
3203	*CCDC127*	133957	3.3										5	196871	218215
3204	*CCDC136*	64753	-3.8										7	128790757	128822132
3205	*CCDC141*	285025	-4.7										2	178829757	179050086
3206	*CCDC142*	84865	-4.0										2	74471986	74483408
3207	*CCDC146*	57639	-3.6										7	77122434	77329533
3208	*CCDC150*	284992	-4.4										2	196639554	196763490
3209	*CCDC153*	283152	-2.5										11	119189638	119196769
3210	*CCDC169*	728591			4.5								13	36222008	36297840
3211	*CCDC169-SOHLH2*	100526761			4.5								13	36168794	36297842
3212	*CCDC174*	51244	4.3										3	14651746	14672659
3213	*CCDC30*	728621	-4.6										1	42463330	42654664
3214	*CCDC36*	339834	-3.0										3	49198428	49258104
3215	*CCDC40*	55036	-2.2										17	80036632	80100613
3216	*CCDC53*	51019				3.9							12	102012927	102062149
3217	*CCDC58*	131076				4.1							3	122359591	122383231
3218	*CCDC59*	29080				2.1							12	82223681	82358805
3219	*CCDC62*	84660	-4.0										12	122774327	122827528
3220	*CCDC66*	285331	-3.5										3	56557161	56621818
3221	*CCDC7*	221016		1.9									10	32446082	32882874
3222	*CCDC71L*	168455	2.7										7	106656765	106660996
3223	*CCDC73*	493860	4.3										11	32602246	32794658
3224	*CCDC77*	84318	-1.6										12	389273	442645
3225	*CCDC79*	283847	-4.0										16	66754976	66801620
3226	*CCDC80*	151887										-2.5	3	112596794	112649530
3227	*CCDC84*	338657		-1.7									11	118998142	119015791
3228	*CCDC88B*	283234	-2.7										11	64340223	64357534
3229	*CCDC91*	55297	2.7										12	28133249	28581511
3230	*CCL14*	6358	-1.6										17	35983291	35987004
3231	*CCL16*	6360	-5.4										17	35976493	35981496
3232	*CCL22*	6367	-5.1										16	57358772	57366190
3233	*CCL28*	56477	-3.5										5	43376645	43412391
3234	*CCL3L3*	414062		-1.8									17	36194869	36196758

3235	*CCM2*	83605					1.6						7	44999475	45076469
3236	*CCM2L*	140706	-2.1										20	32010450	32032180
3237	*CCNA2*	890									-1.5		4	121816444	121823933
3238	*CCND2*	894	-4.6										12	4273772	4305350
3239	*CCNDBP1*	23582									-1.7		15	43185118	43197176
3240	*CCNG2*	901									-2.1		4	77157151	77433388
3241	*CCNK*	8812					1.6						14	99481169	99535044
3242	*CCNL1*	57018	4.7										3	157146508	157160760
3243	*CCNL2*	81669			-2.4								1	1385711	1399328
3244	*CCNT1*	904	-5.2										12	48688458	48716998
3245	*CCR6*	1235	-2.8										6	167111807	167139696
3246	*CCRL2*	9034	-2.6										3	46407163	46412997
3247	*CCS*	9973			-1.6								11	66592821	66606019
3248	*CCT6B*	10693	-4.9										17	34927859	34981078
3249	*CCT7*	10574							-1.8				2	73233420	73253021
3250	*CCT8*	10694		-1.6									21	29055805	29073797
3251	*CCZ1*	51622										-1.9	7	5898725	5926550
3252	*CD151*	977						3.3					11	832843	839831
3253	*CD163*	9332							1.6				12	7470813	7503893
3254	*CD164*	8763							-1.5				6	109366514	109382559
3255	*CD19*	930	-2.1										16	28931939	28939346
3256	*CD2*	914	-6.2										1	116754385	116769228
3257	*CD22*	933							1.7				19	35319261	35347355
3258	*CD247*	919	5.1										1	167430640	167518610
3259	*CD276*	80381						2.0					15	73683966	73714518
3260	*CD28*	940	-1.8										2	203706475	203738912
3261	*CD300E*	342510		-1.6									17	74609887	74623738
3262	*CD300LG*	146894	-3.3										17	43847148	43863629
3263	*CD302*	9936									-2.5		2	159768630	159798255
3264	*CD320*	51293	-6.7										19	8302127	8308356
3265	*CD3G*	917	-2.3										11	118344344	118355161
3266	*CD4*	920										-1.6	12	6786858	6820808
3267	*CD40*	958						1.6					20	46118272	46129863
3268	*CD40LG*	959	-3.4										X	136648193	136660390
3269	*CD46*	4179									-1.9		1	207752057	207795513
3270	*CD47*	961							-1.6				3	108043298	108091025
3271	*CD55*	1604					2.2						1	207321508	207386804
3272	*CD58*	965	-2.5										1	116514535	116571039
3273	*CD74*	972	-3.2										5	150401637	150412929
3274	*CD79A*	973							1.7				19	41877120	41881372
3275	*CD79B*	974		-2.2									17	63928740	63932354
3276	*CD82*	3732	-2.8										11	44564427	44620363
3277	*CD8A*	925									-1.9		2	86784610	86808396
3278	*CDADC1*	81602			-3.1								13	49247925	49293485
3279	*CDC20*	991			4.8								1	43358955	43363203
3280	*CDC25C*	995			3.7								5	138285265	138338355
3281	*CDC37L1*	55664			2.9								9	4679559	4708398
3282	*CDC40*	51362	-4.5										6	110180141	110254275
3283	*CDC42*	998	3.0										1	22052627	22092946
3284	*CDC42BPB*	9578	3.0										14	102932379	103057462
3285	*CDC42BPG*	55561	-2.2										11	64823387	64844569
3286	*CDCA4*	55038	-3.2										14	105009573	105021148
3287	*CDCA7*	83879	-4.6										2	173354820	173368997
3288	*CDCA7L*	55536	-5.1										7	21900900	21946084
3289	*CDCP1*	64866	-3.1										3	45082278	45146422
3290	*CDH1*	999			-3.8								16	68737225	68835548
3291	*CDH11*	1009										-1.9	16	64943753	65126112
3292	*CDH18*	1016	-4.1										5	19472951	20575873
3293	*CDH2*	1000										1.8	18	27950966	28177446

3294	*CDH23*	64072	-2.3										10	71396934	71815947
3295	*CDH6*	1004	-5.3										5	31193750	31329146
3296	*CDH7*	1005	-6.9										18	65750252	65890341
3297	*CDHR3*	222256	-3.9										7	105876796	106033773
3298	*CDHR4*	389118	-4.4										3	49790732	49799835
3299	*CDK10*	8558		-1.8									16	89680737	89696364
3300	*CDK11A*	728642					1.7						1	1702730	1724324
3301	*CDK11B*	984	-4.2										1	1635227	1659012
3302	*CDK17*	5128					-1.9						12	96278261	96400560
3303	*CDK19*	23097	-2.8										6	110609978	110815958
3304	*CDK2*	1017			-2.0								12	55966769	55972784
3305	*CDK2AP1*	8099		1.6									12	123260971	123272334
3306	*CDKAL1*	54901	-5.4										6.526471	75037892	75111924.54
3307	*CDKL5*	6792		1.8									X	18425583	18653629
3308	*CDKN1C*	1028		-2.9									11	2883213	2885881
3309	*CDKN2AIPNL*	91368	-2.4										5	134402087	134411898
3310	*CDKN2C*	1031	-2.2										1	50960745	50974633
3311	*CDR1*	1038			-1.7								X	140782405	140784871
3312	*CDS1*	1040	3.0										4	84582979	84651338
3313	*CDSN*	1041								-3.0			6	31115090	31120446
3314	*CEACAM5*	1048	-4.7										19	41708585	41729798
3315	*CEACAM6*	4680	-7.9										19	41750977	41772208
3316	*CEACAM8*	1088	-5.1										19	42580241	42740384
3317	*CEBPA*	1050		-1.7									19	33299934	33302564
3318	*CEBPG*	1054			-2.6								19	33373330	33382686
3319	*CELF4*	56853	-2.2										18	37243047	37566037
3320	*CEMP1*	752014				-3.8							16	2530035	2531417
3321	*CENPB*	1059		-1.5									20	3783851	3786690
3322	*CENPBD1*	92806	-2.8										16	89969791	89972534
3323	*CENPI*	2491	-2.0										X	101098218	101163681
3324	*CENPK*	64105			4.3								5	65517766	65563171
3325	*CENPN*	55839	-4.5										16	81006498	81033114
3326	*CENPO*	79172			3.5								2	24793136	24822376
3327	*CENPP*	401541				-1.8							9	92325484	92620533
3328	*CENPT*	80152	-4.3										16	67828157	67847811
3329	*CEP104*	9731	-4.2										1	3812081	3857214
3330	*CEP126*	57562	-4.4										11	101915015	102001058
3331	*CEP135*	9662	-5.8										4	55948871	56033363
3332	*CEP170B*	283638	-6.1										14	104865280	104896770
3333	*CEP19*	84984	-2.1										3	196706277	196712293
3334	*CEP192*	55125	-3.3										18	12991362	13125052
3335	*CEP41*	95681	-5.5										7	130393771	130442433
3336	*CEP44*	80817	-2.6										4	174283677	174333380
3337	*CEP55*	55165			2.9								10	93496632	93529092
3338	*CEP57*	9702	4.1										11	95789965	95832693
3339	*CEP63*	80254	-4.1										3	134485743	134575017
3340	*CEP83*	51134	-5.4										12	94306449	94459988
3341	*CEP85L*	387119			1.8								6	118460772	118710075
3342	*CERCAM*	51148	-3.1										9	128411751	128437351
3343	*CERKL*	375298		-1.6									2	181536676	181680665
3344	*CERS1*	10715										2.0	19	18868545	18896727
3345	*CERS5*	91012	-3.2										12	50129306	50167533
3346	*CFAP20*	29105			2.6								16	58113588	58129450
3347	*CFAP43*	80217	-1.7										10	104129888	104232362
3348	*CFAP74*	85452	-7.3										1	1921951	2003837
3349	*CFB*	629	-3.2										6	31945650	31952084
3350	*CFH*	3075	-7.3										1	196651878	196747504
3351	*CFI*	3426	-3.0										4	109740694	109802179
3352	*CFL1*	1072				-1.8							11	65823022	65862026

3353	*CGNL1*	84952	-6.8										15	57375967	57550727
3354	*CHAC1*	79094			-6.2								15	40952962	40956519
3355	*CHAF1A*	10036	-3.1										19	4402662	4445018
3356	*CHCHD2*	51142										-2.5	7	56101569	56106576
3357	*CHCHD4*	131474	2.8										3	14112077	14124870
3358	*CHCHD6*	84303			-3.6								3	126704220	126960406
3359	*CHD1*	1105	-2.6										5	98853985	98928957
3360	*CHD1L*	9557						2.1					1	147242641	147295766
3361	*CHD4*	1108	4.4										12	6570083	6607476
3362	*CHDH*	55349	-5.8										3	53812335	53846390
3363	*CHFR*	55743	-2.7										12	132822187	132956304
3364	*CHI3L2*	1117	-2.5										1	111200771	111243440
3365	*CHIT1*	1118	-2.8										1	203212827	203273641
3366	*CHKA*	1119	3.1										11	68052859	68121444
3367	*CHMP1A*	5119	-5.4										16	89644431	89657845
3368	*CHMP4C*	92421	-3.8										8	81732434	81759515
3369	*CHMP6*	79643	2.4										17	80991598	81009517
3370	*CHPF*	79586	-3.2										2	219538947	219543787
3371	*CHRD*	8646	-4.4										3	184380073	184390736
3372	*CHRDL1*	91851	-1.8										X	110673856	110795819
3373	*CHRNA3*	1136	-1.5										15	78593052	78621295
3374	*CHRNA5*	1138	-3.6										15	78565520	78595269
3375	*CHRNA6*	8973	-1.7										8	42752620	42796392
3376	*CHRNB1*	1140	-5.5										17	7445061	7457707
3377	*CHRNB4*	1143	-5.5										15	78624119	78727754
3378	*CHRNE*	1145	3.4										17	4897774	4903074
3379	*CHST10*	9486	-1.9										2	100391860	100417656
3380	*CHST3*	9469						1.7					10	71964365	72013564
3381	*CHST5*	23563	-5.1										16	75528535	75535247
3382	*CHST6*	4166	-7.0										16	75472052	75495384
3383	*CINP*	51550			1.6								14	102341102	102362916
3384	*CIT*	11113										1.6	12	119685790	119877291
3385	*CITED2*	10370	-2.8										6	139371807	139374620
3386	*CIZ1*	25792		-1.5									9	128166064	128204383
3387	*CKLF*	51192			2.2								16	66552563	66566251
3388	*CKLF-CMTM1*	100529251			2.2								16	66552587	66579135
3389	*CKMT1A*	548596			-2.2								15	43692886	43699222
3390	*CKMT1B*	1159			-2.7								15	43593054	43604901
3391	*CLASP1*	23332	-4.6										2	121337776	121649587
3392	*CLASP2*	23122				-3.9							3	33496245	33718356
3393	*CLCC1*	23155	-5.0										1	108929508	108963457
3394	*CLCN5*	1184	-7.2										X	49922615	50099235
3395	*CLCN6*	1185	-2.6										1	11806096	11843144
3396	*CLDN10*	9071			-3.0								13	95433604	95579759
3397	*CLDN16*	10686	-6.0										3	190322541	190412143
3398	*CLDN4*	1364	-3.0										7	73799542	73832693
3399	*CLEC17A*	388512		-2.3									19	14583084	14611157
3400	*CLEC19A*	728276	-2.1										16	19285739	19324537
3401	*CLEC4M*	10332	-4.2										19	7763149	7769605
3402	*CLIC2*	1193						1.8					X	155276211	155334657
3403	*CLIC5*	53405		-1.5									6	45898451	46080395
3404	*CLMN*	79789	3.2										14	95181940	95319906
3405	*CLN3*	1201	-3.4										16	28477279	28495575
3406	*CLN5*	1203			-2.2								13	76990660	77002517
3407	*CLN6*	54982	-4.9										15	68206992	68257211
3408	*CLNK*	116449	-2.1										4	10486395	10684865
3409	*CLPP*	8192	3.2										19	6361452	6368908
3410	*CLSPN*	63967	-4.3										1	35720218	35769967
3411	*CLSTN3*	9746	-2.1										12	7129698	7158945

3412	*CLTCL1*	8218	-1.8										22	19179473	19291716
3413	*CLUAP1*	23059	-4.8										16	3500924	3539048
3414	*CMC2*	56942		1.9									16	80966448	81020270
3415	*CMC4*	100272147	-2.9										X	155061622	155071362
3416	*CMKLR1*	1240		-1.9									12	108288044	108339341
3417	*CMSS1*	84319	-5.3										3	99817834	100178603
3418	*CMTM4*	146223	2.9										16	66614750	66696707
3419	*CNDP2*	55748	3.4										18	74495816	74523454
3420	*CNEP1R1*	255919	-2.2										16	50024410	50037088
3421	*CNNM3*	26505	-4.3										2	96816245	96833911
3422	*CNOT10*	25904	-3.6										3	32685145	32773875
3423	*CNOT6L*	246175	-3.6										4	77713387	77819615
3424	*CNPY2*	10330		-2.0									12	56309842	56316336
3425	*CNR2*	1269		-1.7									1	23870526	23913362
3426	*CNRIP1*	25927	-7.7										2	68284171	68320051
3427	*CNTLN*	54875	-2.7										10.6	75034465.4	75108481.54
3428	*CNTN1*	1272								-2.0			12	40692442	41072418
3429	*CNTNAP1*	8506	-1.9										17	42682613	42699814
3430	*CNTNAP5*	129684	-1.9										2	124025287	124915287
3431	*CNTRL*	11064							1.7				9	121074863	121177610
3432	*COA7*	65260	-2.4										1	52684451	52698366
3433	*COBL*	23242	-2.6										7	51016212	51316818
3434	*COCH*	1690	-2.9										14	30874514	30895065
3435	*COG2*	22796	-3.8										1	230642489	230693982
3436	*COG6*	57511			-3.5								13	39655627	39791665
3437	*COG7*	91949	-2.4										16	23388493	23453180
3438	*COG8*	84342	3.2										16	69320140	69339667
3439	*COL10A1*	1300	-3.5										6	116118923	116158747
3440	*COL12A1*	1303	-1.8										6	75084326	75206051
3441	*COL14A1*	7373	-2.0										8	120059780	120372036
3442	*COL15A1*	1306	-4.5										9	98943179	99070792
3443	*COL16A1*	1307	-2.5										1	31652247	31704319
3444	*COL17A1*	1308	-2.0										10	104031286	104086002
3445	*COL24A1*	255631	-4.2										1	85729233	86156943
3446	*COL28A1*	340267	-2.3										7	7356203	7535853
3447	*COL3A1*	1281	-3.6										2	188974320	189012746
3448	*COL4A1*	1282	-6.4										13	110148963	110307149
3449	*COL5A1*	1289	-4.1										9	134641774	134844843
3450	*COL5A3*	50509	-3.7										19	9959561	10010471
3451	*COL6A2*	1292	-4.2										21	46098097	46132849
3452	*COL6A3*	1293	-5.4										2	237324003	237414375
3453	*COL7A1*	1294	-3.0										3	48564073	48595267
3454	*COL8A1*	1295	-3.1										3	99638475	99799226
3455	*COLGALT1*	79709	-5.6										19	17555594	17583162
3456	*COMMD3*	23412										-2.0	10	22315974	22320308
3457	*COMMD5*	28991	3.6										8	144841042	144853736
3458	*COMMD7*	149951	4.4										20	32702691	32743997
3459	*COMMD9*	29099	-3.5										11	36269284	36289449
3460	*COMP*	1311	-3.8										19	18782773	18791314
3461	*COPS6*	10980							-1.5				7	100088954	100092200
3462	*COPZ1*	22818	2.5										12	54301202	54351849
3463	*COQ10B*	80219	-4.6										2	197453423	197475308
3464	*COQ3*	51805									-1.7		6	99369400	99394204
3465	*COQ4*	51117		2.4									9	128322536	128334072
3466	*COQ9*	57017		-1.8									16	57447425	57461275
3467	*CORIN*	10699	-5.0										4	47593998	47838106
3468	*CORO2A*	7464	-3.4										9	98120975	98192640
3469	*CORO7*	79585							1.7				16	4354542	4425705
3470	*COX17*	10063	3.4										3	119654513	119677454

3471	*COX18*	285521	-4.6										4	73052362	73069755
3472	*COX20*	116228			1.7								1	244835322	244845057
3473	*COX6A1*	1337	2.9										12	120438090	120440742
3474	*COX6B2*	125965	-3.2										19	55349306	55354814
3475	*COX7B2*	170712			9.3								4	46734827	46909235
3476	*CP*	1356	-2.5										3	149162410	149222055
3477	*CPA4*	51200	-4.9										7	130293134	130324180
3478	*CPAMD8*	27151	-3.9										19	16892947	17026815
3479	*CPB2*	1361										1.8	13	46053186	46105076
3480	*CPD*	1362	2.8										17	30378905	30469989
3481	*CPEB4*	80315							1.8				5	173888280	173961976
3482	*CPED1*	79974						2.6					7	120988677	121297444
3483	*CPNE6*	9362		-1.7									14	24070837	24078100
3484	*CPSF3L*	54973	-3.9										10.63361	75046458.6	75120532.01
3485	*CPT1A*	1374	-4.9										11	68754620	68844410
3486	*CPT1C*	126129	-5.4										19	49690898	49713731
3487	*CPT2*	1376	-3.2										1	53196429	53214197
3488	*CRAMP1*	57585	-5.3										16	1612325	1677908
3489	*CRBN*	51185	3.2										3	3148992	3179710
3490	*CRCP*	27297	-3.1										7	66114604	66154568
3491	*CREB3L2*	64764					-1.7						7	137874979	138002067
3492	*CREB3L4*	148327	-3.4										1	153967534	153974363
3493	*CREBL2*	1389					-2.3						12	12611827	12645108
3494	*CRELD2*	79174			-1.6								22	49918167	49927540
3495	*CREM*	1390					-1.9						10	35126791	35212958
3496	*CRHR1*	1394	-2.1										17	45784280	45835828
3497	*CRIP3*	401262	-2.8										6	43299710	43308797
3498	*CRISPLD1*	83690	2.8										8	74984515	75034558
3499	*CRKL*	1399			-3.5								22	20917426	20953749
3500	*CROT*	54677	-6.5										13.83219	75019730.8	75093676.68
3501	*CRP*	1401									-2.3		1	159712289	159714589
3502	*CRTAM*	56253	-2.9										11	122838500	122872639
3503	*CRTAP*	10491	-5.8										3	33113979	33147773
3504	*CRTC3*	64784	2.7										15	90529925	90645345
3505	*CRX*	1406	-6.3										19	47819779	47843330
3506	*CRYAB*	1410	-1.9										11	111908565	111923722
3507	*CRYBA2*	1412	-4.8										2	218990189	218993421
3508	*CRYBB2*	1415				-2.7							22	25219522	25231869
3509	*CRYZL1*	9946	-4.7										21	33589341	33643926
3510	*CSDC2*	27254	-3.1										22	41560763	41577741
3511	*CSDE1*	7812					-1.6						1	114716913	114758676
3512	*CSMD1*	64478	-1.7										8	2935353	4994972
3513	*CSMD2*	114784	-2.4										1	33513999	34165842
3514	*CSMD3*	114788	-5.4										8	112222928	113437099
3515	*CSNK1A1L*	122011		2.9									13	37103259	37105664
3516	*CSNK1D*	1453	3.2										17	82239023	82273731
3517	*CSNK1E*	1454									-1.7		22	38290691	38398522
3518	*CSNK1G1*	53944	-5.6										15	64165517	64356259
3519	*CSNK1G2*	1455				-1.7							19	1941149	1981338
3520	*CSPG5*	10675	-2.3										3	47562239	47580792
3521	*CSRP1*	1465					-1.5						1	201483530	201509456
3522	*CSRP2BP*	57325	-3.6										20	18138118	18188387
3523	*CST4*	1472			-1.8								20	23685640	23689040
3524	*CSTF3*	1479					-1.6						11	33077188	33162371
3525	*CSTL1*	128817	-4.8										20	23439685	23444930
3526	*CT45A1*	541466			3.9								X	135713453	135723539
3527	*CT45A2*	728911			3.7								X	135811668	135820012
3528	*CT45A3*	441519			3.7								X	135759846	135768191
3529	*CT45A4*	441520			3.8								X	135811668	135820012

3530	*CT45A5*	441521			1.8								X	135777130	135785298
3531	*CT45A6*	541465			3.5								X	135794706	135802656
3532	*CTAG1A*	246100			-2.1								X	154585143	154586821
3533	*CTAG1B*	1485			-2.1								X	154617604	154619282
3534	*CTBP1*	1487				-2.0							4	1211448	1249953
3535	*CTBP2*	1488							-1.7				10	124984317	125161170
3536	*CTDSP1*	58190					1.6						2	218398256	218405941
3537	*CTDSPL2*	51496			-2.5								15	44427234	44529038
3538	*CTGF*	1490	-6.5										6	131948176	131951373
3539	*CTNNA1*	1495								-2.4			5	138610967	138935034
3540	*CTPS1*	1503	3.1										1	40979335	41012565
3541	*CTSH*	1512	-4.5										15	78921058	78949574
3542	*CTSV*	1515	-3.1										9	97029679	97039643
3543	*CTSW*	1521	3.6										11	65879809	65883741
3544	*CTTNBP2NL*	55917	-2.2										1	112396181	112463456
3545	*CUL2*	8453	-1.6										10	35008551	35090642
3546	*CUL3*	8452	-3.8										2	224470150	224585397
3547	*CWF19L1*	55280	-3.3										10	100232298	100267680
3548	*CX3CL1*	6376	-3.7										16	57372458	57385048
3549	*CXADR*	1525	-4.9										21	17512382	17593579
3550	*CXCL12*	6387										-1.8	10	44370165	44386493
3551	*CXCL16*	58191	-5.1										17	4733526	4739922
3552	*CXCL17*	284340	-6.7										19	42428288	42443048
3553	*CXCL3*	2921	-2.6										4	74036589	74038807
3554	*CXCL8*	3576			-3.9								4	73740506	73743716
3555	*CXCR5*	643		-2.1									11	118883766	118897799
3556	*CXorf23*	256643	-1.9										X	19912860	19970298
3557	*CXorf36*	79742	-3.5										X	45148374	45200901
3558	*CXorf38*	159013	-6.3										X	40626921	40647554
3559	*CYB561A3*	220002		-1.7									11	61348745	61362299
3560	*CYB5D1*	124637	3.3										17	7857746	7862282
3561	*CYB5R2*	51700	-2.1										11	7665100	7677222
3562	*CYB5R3*	1727		2.1									22	42617840	42649568
3563	*CYBA*	1535	-4.3										16	88643283	88651152
3564	*CYP17A1*	1586	-2.2										10	102830531	102837533
3565	*CYP1A1*	1543	-2.6										15	74719542	74725610
3566	*CYP1A2*	1544	-5.9										15	74748844	74756202
3567	*CYP1B1*	1545	-4.0										2	38066973	38109902
3568	*CYP20A1*	57404	-4.6										2	203238449	203305840
3569	*CYP27A1*	1593	-2.4										2	218781749	218815293
3570	*CYP27C1*	339761	-1.9										2	127184120	127220078
3571	*CYP2B6*	1555	-4.1										19	40991299	41018398
3572	*CYP2E1*	1571	-1.8										10	133520406	133561220
3573	*CYP2F1*	1572	-2.9										19	41114432	41128366
3574	*CYP2J2*	1573	-2.1										1	59893308	59926790
3575	*CYP2U1*	113612										1.6	4	107931369	107953457
3576	*CYP2W1*	54905	-2.2										5.711765	75038577.4	75112613.13
3577	*CYP3A43*	64816	-5.2										7	99828013	99866102
3578	*CYP3A5*	1577	-4.4										7	99648194	99679998
3579	*CYP46A1*	10858	-3.6										14	99684304	99727301
3580	*CYP4B1*	1580	-4.9										1	46757838	46819413
3581	*CYP4F12*	66002	-5.2										19	15672757	15697174
3582	*CYP4V2*	285440	-4.0										4	186191520	186213456
3583	*CYP4Z1*	199974	-2.7										1	47067488	47118319
3584	*CYSLTR2*	57105		-1.5									13	48653711	48711226
3585	*CYTL1*	54360	-1.5										2.8	75001569.6	75075428.82
3586	*DAAM1*	23002						2.5					14	59188646	59371405
3587	*DACT3*	147906	-5.9										19	46647612	46661138
3588	*DAND5*	199699	-5.7										19	12965159	12974762

3589	*DAPK2*	23604	-2.7										15	63907036	64072033
3590	*DARS*	1615			-1.9								2	135906677	135986100
3591	*DARS2*	55157				2.8							1	173824503	173858546
3592	*DAW1*	164781	-3.5										2	227871054	227924344
3593	*DBN1*	1627		1.9									5	177456608	177474401
3594	*DBNDD1*	79007	-3.5										16	90004865	90020128
3595	*DBNL*	28988					1.7						7	44044640	44069456
3596	*DBP*	1628	-4.6										19	48630030	48637438
3597	*DBT*	1629	-4.5										1	100186919	100249834
3598	*DBX2*	440097	-2.1										12	45014672	45051099
3599	*DCAF11*	80344			-2.6								14	24114195	24125242
3600	*DCAF12*	25853	3.1										9	34086387	34127399
3601	*DCAF4*	26094	-3.9										14	72926332	72959703
3602	*DCAF6*	55827			1.6								1	167935783	168075843
3603	*DCAF8*	50717	-4.8										1	160215715	160262531
3604	*DCAF8L2*	347442	-2.3										X	27590382	27748821
3605	*DCDC2*	51473	-3.4										6	24171756	24358052
3606	*DCK*	1633						-1.9					4	70992538	71030914
3607	*DCLK1*	9201			-3.4								13	35768652	36131306
3608	*DCN*	1634	-7.1										12	91140484	91183123
3609	*DCP1A*	55802				-2.5							3	53283428	53347610
3610	*DCP2*	167227									-1.5		5	112976702	113020970
3611	*DCST1*	149095	-4.6										1	155033824	155050930
3612	*DCST2*	127579	-4.3										1	155018520	155033781
3613	*DCT*	1638			-2.7								13	94436808	94479682
3614	*DCTN2*	10540	2.7										12	57530102	57547331
3615	*DCTN3*	11258	4.6										9	34613545	34620523
3616	*DCTN4*	51164					-1.6						5	150708440	150759109
3617	*DCUN1D2*	55208	-4.1										13	113455819	113490952
3618	*DCUN1D5*	84259										1.6	11	103062076	103092215
3619	*DCX*	1641	-2.4										X	111293780	111412375
3620	*DDB2*	1643		-1.6									11	47214465	47239240
3621	*DDC*	1644	3.2										7	50458436	50565457
3622	*DDHD1*	80821	-3.2										14	53036745	53153282
3623	*DDHD2*	23259	-3.6										8	38225218	38275558
3624	*DDIT3*	1649						2.2					12	57516588	57520517
3625	*DDOST*	1650					-1.5						1	20651767	20661544
3626	*DDR1*	780	-3.6										6	30876421	30900156
3627	*DDR2*	4921		-2.6									1	162631373	162787400
3628	*DDT*	1652		-1.7									22	23971365	23980469
3629	*DDTL*	100037417	-3.5										22	23966901	23972532
3630	*DDX10*	1662	-5.6										11	108665025	108940930
3631	*DDX18*	8886				1.6							2	117814650	117832379
3632	*DDX3Y*	8653	4.2										Y	12904108	12920478
3633	*DDX43*	55510	-3.0										6	73394748	73417569
3634	*DDX49*	54555	3.3										13.19547	75015961.5	75089889.39
3635	*DDX5*	1655									-1.6		17	64499616	64508199
3636	*DDX51*	317781	-5.2										12	132136594	132144335
3637	*DDX58*	23586		-1.8									9	32455705	32526324
3638	*DDX6*	1656									-1.9		11	118747766	118791149
3639	*DDX60L*	91351	-1.9										4	168356735	168537786
3640	*DEAF1*	10522									-1.6		11	644233	706715
3641	*DECR1*	1666	4.0										8	90001405	90052092
3642	*DEDD2*	162989					1.7						19	42198598	42220140
3643	*DEF6*	50619					1.5						6	35297852	35321771
3644	*DEF8*	54849	3.0										7.263636	75030696.1	75104694.25
3645	*DEFA1B*	728358	-7.1										8	6977649	6980080
3646	*DEFA4*	1669	-2.9										8	6935822	6938338
3647	*DEFB123*	245936	-1.7										20	31440519	31450257

3648	*DEGS1*	8560								-2.0			1	224175756	224193441
3649	*DENND2A*	27147	-5.6										7	140518420	140673993
3650	*DENND4A*	10260	-3.6										15	65658046	65792293
3651	*DENND5B*	160518	-3.7										12	31382223	31591097
3652	*DENND6A*	201627	-3.8										3	57625457	57693089
3653	*DEPDC1*	55635			3.4								1	68474152	68497221
3654	*DEPDC5*	9681	-7.2										22	31753951	31907034
3655	*DERL1*	79139							-1.5				8	123013164	123042423
3656	*DES*	1674	-11.8										2	219418377	219426739
3657	*DESI1*	27351	-3.0										22	41598028	41621096
3658	*DFFA*	1676	-5.2										1	10456522	10472526
3659	*DFFB*	1677	-5.6										1	3857267	3885429
3660	*DFNA5*	1687			-2.4								7	24698353	24758113
3661	*DFNB31*	25861		-1.5									9	114402080	114505450
3662	*DGAT2*	84649	4.2										11	75759512	75801535
3663	*DGCR6L*	85359	-3.0										22	20314276	20320080
3664	*DGKA*	1606	-3.3										12	55927319	55954027
3665	*DGKB*	1607	-5.6										7	14145049	14974777
3666	*DGKE*	8526	-3.0										17	56834099	56869567
3667	*DGKZ*	8525	-4.5										11	46332905	46380554
3668	*DHCR24*	1718			3.1								1	54849633	54887218
3669	*DHCR7*	1717			-2.7								11	71428193	71452868
3670	*DHRS1*	115817								-1.8			14	24290598	24299833
3671	*DHRS3*	9249						2.9					1	12567910	12617731
3672	*DHRS7*	51635									-1.6		14	60144120	60169856
3673	*DHX37*	57647	-2.0										12	124946825	124989122
3674	*DHX40*	79665	-2.7										17	59565525	59608345
3675	*DHX57*	90957	-2.7										2	38797729	38875934
3676	*DHX8*	1659	-3.6										17	43483865	43544463
3677	*DIEXF*	27042	-5.6										1	209828007	209857565
3678	*DIRC2*	84925	-3.0										3	122794795	122881139
3679	*DIS3*	22894	-4.2										13	72752169	72782096
3680	*DIS3L*	115752	-3.6										15	66293217	66333898
3681	*DISC1*	27185	-4.7										1	231626815	232041272
3682	*DISP2*	85455	-4.1										15	40358235	40378639
3683	*DKK1*	22943										2.5	10	52314296	52318042
3684	*DKK3*	27122	-3.2										11	11963106	12009769
3685	*DLC1*	10395			2.6								8	13083361	13604610
3686	*DLG3*	1741	3.6										X	70444861	70505490
3687	*DLST*	1743	-3.8										14	74881891	74903745
3688	*DMBX1*	127343	-3.1										1	46506996	46514226
3689	*DMC1*	11144	-5.2										22	38518949	38570286
3690	*DMKN*	93099	-3.6										19	35497220	35513658
3691	*DNAAF3*	352909	-3.5										19	55158661	55166722
3692	*DNAH10*	196385	-3.6										12	123762188	123936206
3693	*DNAH11*	8701	-4.9										7	21543215	21901839
3694	*DNAH12*	201625	-3.3										3	57293699	57544344
3695	*DNAH2*	146754	-4.4										17	7717354	7833744
3696	*DNAH7*	56171	-1.8										2	195737703	196068812
3697	*DNAH9*	1770	-6.0										17	11598431	11969748
3698	*DNAJA2*	10294	4.5										16	46955362	46973788
3699	*DNAJA4*	55466	5.8										15	78264086	78282196
3700	*DNAJB11*	51726							-1.6				3	186567403	186597203
3701	*DNAJB2*	3300	-3.4										2	219279267	219286900
3702	*DNAJB4*	11080	-2.7										1	77979175	78017964
3703	*DNAJC11*	55735	2.7										1	6634168	6701924
3704	*DNAJC18*	202052	-3.9										5	139408588	139444491
3705	*DNAJC19*	131118		-1.9									3	180983709	180989774
3706	*DNAJC24*	120526	-5.3										11	31369840	31431849

3707	*DNAJC25-GNG10*	552891	4.6										9	111631386	111670229
3708	*DNAJC4*	3338	-4.3										11	64230278	64234286
3709	*DNAJC5B*	85479	-9.8										8	66021560	66100526
3710	*DNAJC5G*	285126	-2.9										2	27275421	27281499
3711	*DNAL1*	83544	-3.8										14	73644875	73703732
3712	*DNASE2*	1777	-6.0										19	12875211	12881468
3713	*DNHD1*	144132		-1.7									11	6497260	6593758
3714	*DNLZ*	728489	-1.6										9	136359480	136363789
3715	*DNM1L*	10059				4.2							12	32679200	32745650
3716	*DNM2*	1785	-3.5										19	10718079	10833488
3717	*DNMT3A*	1788		1.7									2	25227855	25342590
3718	*DNPEP*	23549	-4.5										2	219373546	219400022
3719	*DOCK1*	1793	-2.7										10	126905409	127452517
3720	*DOCK4*	9732	-3.1										7	111726110	112206411
3721	*DOCK5*	80005	-5.0										8	25184723	25418082
3722	*DOCK9*	23348								-2.1			13	98793487	99086625
3723	*DOLPP1*	57171						1.7					9	129081100	129090438
3724	*DOPEY2*	9980	-4.6										21	36156782	36294274
3725	*DPH1*	1801	-3.7										17	2030110	2043430
3726	*DPM3*	54344	3.0										4.4	75001226.9	75075084.53
3727	*DPP7*	29952				-4.9							9	137110542	137115177
3728	*DPP9*	91039	-4.1										19	4675224	4724673
3729	*DPY19L4*	286148	-5.1										8	94719703	94793836
3730	*DPYD*	1806									-2.1		1	97077743	97921049
3731	*DPYSL2*	1808			2.7								8	26514275	26658178
3732	*DPYSL5*	56896	-4.0										2	26847747	26950351
3733	*DQX1*	165545	-7.7										2	74518131	74526336
3734	*DRAP1*	10589	4.4										11	65919257	65921561
3735	*DRG2*	1819									-3.0		17	18087886	18107971
3736	*DROSHA*	29102	3.6										5	31400497	31532196
3737	*DRP2*	1821	-1.7										X	101219769	101264497
3738	*DSC1*	1823	-2.6										18	31129236	31162856
3739	*DSC3*	1825	-3.1										18	30990008	31042815
3740	*DSG3*	1830	-4.0										18	31447795	31478702
3741	*DST*	667	-6.2										6	56457987	56954628
3742	*DSTYK*	25778	-4.1										1	205142505	205211566
3743	*DTD2*	112487	-4.6										14	31446036	31457510
3744	*DTL*	51514	-3.0										1	212035553	212107400
3745	*DTNB*	1838	-4.9										2	25377198	25673647
3746	*DTWD2*	285605	-8.0										5	118837322	118988545
3747	*DTX3L*	151636	-3.1										3	122564238	122575203
3748	*DUOX1*	53905	-3.5										15	45129933	45165576
3749	*DUSP1*	1843			-1.9								5	172768090	172771195
3750	*DUSP11*	8446	-3.7										2	73762184	73780157
3751	*DUSP16*	80824	-3.8										12	12474210	12562383
3752	*DUSP19*	142679	-3.3										2	183078559	183100005
3753	*DUSP23*	54935			-4.2								7.078788	75041318.7	75115367.53
3754	*DUSP26*	78986	-3.2										8	33591332	33600106
3755	*DUSP28*	285193	-4.8										2	240560054	240564014
3756	*DUSP7*	1849					1.7						3	52048919	52056550
3757	*DUXA*	503835	-3.1										19	57154021	57167443
3758	*DXO*	1797	-4.0										6	31969810	31972292
3759	*DYDC1*	143241	-5.1										10	80336105	80356755
3760	*DYNC2LI1*	51626	-5.3										2	43774039	43810010
3761	*DYNLL2*	140735	-2.5										17	58083415	58095536
3762	*DYNLRB1*	83658						1.5					20	34516409	34540958
3763	*DYNLT3*	6990			3.7								X	37836757	37847637
3764	*DYX1C1*	161582	-2.4										15	55410525	55508234
3765	*DZANK1*	55184	-6.7										20	18383367	18467281

3766	*E2F2*	1870	-1.9										1	23506430	23531220
3767	*E2F6*	1876	2.9										2	11444375	11466177
3768	*EAF1*	85403	2.7										3	15427355	15450635
3769	*EBAG9*	9166					-1.7						8	109539711	109565996
3770	*EBF1*	1879		-1.7									5	158695916	159099761
3771	*EBF2*	64641	-3.8										8	25841730	26045397
3772	*ECHDC1*	55862	-6.5										6	127288710	127343609
3773	*ECHDC2*	55268	3.7										1	52895910	52927212
3774	*ECHDC3*	79746	-4.9										10	11742366	11764070
3775	*ECI1*	1632			-2.3								16	2239395	2252300
3776	*ECI2*	10455	6.3										6	4115689	4135597
3777	*ECT2L*	345930	-3.4										6	138795926	138904070
3778	*EDARADD*	128178	2.9										1	236348262	236484914
3779	*EEF2*	1938		-1.9									19	3976056	3985469
3780	*EEF2K*	29904	-4.2										16	22206282	22288732
3781	*EEFSEC*	60678	-3.6										3	128153454	128408646
3782	*EEPD1*	80820	-2.7										7	36153149	36301543
3783	*EFCAB10*	100130771	2.7										7	105565120	105600875
3784	*EFCAB14*	9813				3.0							1	46675159	46719064
3785	*EFCAB7*	84455				-4.1							1	63523372	63572693
3786	*EFHD1*	80303	-7.9										2	232606057	232682781
3787	*EFHD2*	79180					2.2						1	15409895	15430343
3788	*EFR3A*	23167					-1.5						8	131904088	132013642
3789	*EFR3B*	22979	-2.3										2	25042130	25159137
3790	*EFS*	10278	-2.9										14	23356402	23365752
3791	*EGFLAM*	133584	-3.8										5	38258409	38465021
3792	*EGLN1*	54583	-4.4										13.31124	75016646.8	75090577.99
3793	*EGR1*	1958			-3.9								5	138465490	138469315
3794	*EHD2*	30846	-5.0										19	47713343	47743134
3795	*EHD4*	30844	-3.8										15	41895939	41972578
3796	*EID2B*	126272	-3.3										19	39530990	39532854
3797	*EIF2A*	83939					-1.7						3	150546678	150584242
3798	*EIF2B3*	8891								1.7			1	44850522	44986722
3799	*EIF3A*	8661		-1.9									10	119033670	119080823
3800	*EIF3B*	8662		-1.6									7	2354086	2380745
3801	*EIF3D*	8664		-1.7									22	36510850	36529436
3802	*EIF3F*	8665		-1.9									11	7970251	8001862
3803	*EIF3G*	8666	-3.3										19	10115017	10119918
3804	*EIF3H*	8667				1.6							8	116642130	116766925
3805	*EIF3M*	10480	3.4										11	32583798	32606262
3806	*EIF4A1*	1973		-1.7									17	7572706	7579005
3807	*EIF4B*	1975		-1.6									12	53006158	53042209
3808	*EIF4E2*	9470	2.9										2	232550052	232583644
3809	*EIF4E3*	317649									-1.6		3	71675416	71754773
3810	*EIF4EBP2*	1979			2.0								10	70404379	70428618
3811	*EIF4G3*	8672		1.8									1	20806292	21176888
3812	*EIF5A2*	56648	-3.6										3	170888415	170908693
3813	*EIF6*	3692							-1.9				20	35278907	35284985
3814	*ELANE*	1991	-2.0										19	851014	856247
3815	*ELK4*	2005			-1.9								1	205597556	205631962
3816	*ELMOD1*	55531	-5.3										11	107591091	107666779
3817	*ELOF1*	84337			3.1								19	11551147	11559236
3818	*ELOVL1*	64834	-2.9										1	43363397	43368074
3819	*ELP2*	55250					-1.6						18	36129444	36180556
3820	*ELP3*	55140	3.4										8	28089673	28191156
3821	*ELP4*	26610	-2.7										11	31509750	31783998
3822	*EMC1*	23065	-2.9										1	19215664	19251552
3823	*EMC10*	284361				-3.6							19	50476400	50490870
3824	*EMC9*	51016			2.2								14	24138959	24141588

3825	*EML2*	24139	-6.0										19	45606994	45645629
3826	*EML3*	256364	-2.0										11	62602218	62612765
3827	*EML5*	161436	-3.0										14	88612431	88792752
3828	*EMP1*	2012	-5.8										12	13196716	13219939
3829	*EMP2*	2013			1.8								16	10528422	10580698
3830	*ENC1*	8507				-4.4							5	74627406	74641424
3831	*ENDOU*	8909								-2.5			12	47709734	47725567
3832	*ENG*	2022	-9.3										9	127815012	127854756
3833	*ENOX2*	10495	-2.6										X	130623369	130903317
3834	*ENPP1*	5167	-7.4										6	131808016	131895155
3835	*ENSA*	2029						2.8					1	150600851	150629612
3836	*ENTPD1*	953	-5.1										10	95844477	95866383
3837	*ENTPD3*	956				-2.3							3	40387156	40428619
3838	*EPAS1*	2034	-6.2										2	46293667	46386703
3839	*EPB41L1*	2036	-2.4										20	36091504	36232799
3840	*EPB42*	2038	3.1										15	43106225	43221283
3841	*EPC1*	80314					-1.6						10	32267751	32378798
3842	*EPCAM*	4072	4.0										2	47345158	47387601
3843	*EPDR1*	54749			-2.2								4.883598	75022472.1	75096431.07
3844	*EPHA10*	284656	-4.4										1	37713880	37765133
3845	*EPHA3*	2042	-1.9										3	89107524	89482134
3846	*EPHA5*	2044	-2.1										4	65319563	65670495
3847	*EPHB4*	2050	-2.8										7	100802565	100827521
3848	*EPHB6*	2051								-1.8			7	142855061	142871094
3849	*EPHX4*	253152	-2.6										1	92029982	92063536
3850	*EPPIN*	57119	-1.8										20	45540626	45547752
3851	*EPS15*	2060		-1.5									1	51354263	51519328
3852	*EPS8L1*	54869	-2.2										7.072727	75033094.7	75107104.35
3853	*ERC1*	23085						1.9					12	990509	1495933
3854	*ERCC2*	2068	-2.1										19	45349837	45370918
3855	*ERCC3*	2071	-3.6										2	127257290	127294176
3856	*ERCC4*	2072	-5.7										16	13920157	13952345
3857	*ERCC8*	1161	-3.6										5	60873831	60945073
3858	*EREG*	2069	-5.8										4	74365143	74388751
3859	*ERG*	2078	-2.9										21	38380027	38661780
3860	*ERGIC1*	57222	-3.8										5	172834275	172952685
3861	*ERGIC2*	51290					-1.5						12	29337352	29381189
3862	*ERLIN2*	11160	-3.7										8	37736599	37759101
3863	*ERO1B*	56605	-2.7										1	236215555	236281985
3864	*ERP44*	23071	3.8										9	99979179	100099040
3865	*ERRFI1*	54206	-2.9										9.2	75000199	75074051.63
3866	*ESCO1*	114799			-2.2								18	21529281	21600884
3867	*ESCO2*	157570	-3.9										8	27771949	27812640
3868	*ESD*	2098					-1.8						13	46771256	46797232
3869	*ESRRA*	2101	3.4										11	64305572	64316743
3870	*ETF1*	2107				2.6							5	138506095	138543300
3871	*ETFA*	2108		2.2									15	76215355	76311472
3872	*ETFB*	2109			1.9								19	51345169	51366418
3873	*ETFDH*	2110	-2.8										4	158672125	158709623
3874	*ETNK2*	55224	-1.7										1	204131062	204152003
3875	*ETS1*	2113						-1.9					11	128458761	128587558
3876	*ETV1*	2115				2.6							7	13891228	13991425
3877	*ETV4*	2118	-3.0										17	43527844	43579620
3878	*EVA1A*	84141	-2.1										2	75469302	75569722
3879	*EVI2A*	2123							2.0				17	31317560	31321884
3880	*EVI5*	7813	-3.1										1	92508696	92792404
3881	*EWSR1*	2130	5.7										22	29268009	29300525
3882	*EXD1*	161829	-3.6										15	41182725	41230743
3883	*EXD3*	54932	-4.6										7.248485	75040633.3	75114678.93

3884	*EXO5*	64789	-4.7										1	40508741	40516556
3885	*EXOC6*	54536	-4.6										12.61662	75012534.9	75086446.4
3886	*EXOSC1*	51013	2.8										10	97436142	97446017
3887	*EXOSC6*	118460		-1.6									16	70246778	70251930
3888	*EXOSC9*	5393		-1.5									4	121801317	121817021
3889	*EXPH5*	23086	-5.5										11	108505431	108593738
3890	*EXT1*	2131	-3.3										8	117794490	118111853
3891	*EYA1*	2138			2.9								8	71197433	71362232
3892	*F11R*	50848						2.1					1	160995211	161021348
3893	*F2RL2*	2151	-2.3										5	76615482	76623434
3894	*F2RL3*	347745		-1.6									19	16888860	16892606
3895	*F3*	2152	-2.0										1	94529225	94541800
3896	*FA2H*	79152	-2.8										16	74712955	74774831
3897	*FAAH2*	158584	-2.2										X	57286706	57489196
3898	*FAAP24*	91442	-3.2										19	32972209	32978222
3899	*FABP7*	2173			-3.5								6	122779475	122784074
3900	*FADS1*	3992				-1.7							11	61799625	61829318
3901	*FADS6*	283985	-6.2										17	74877299	74893781
3902	*FAF1*	11124	2.8										1	50437028	50960263
3903	*FAF2*	23197	3.0										5	176447628	176510074
3904	*FAHD2A*	51011	-2.8										2	95402721	95416616
3905	*FAM103A1*	83640				1.5							15	82986207	82991057
3906	*FAM105A*	54491	-3.6										11.86413	75008080.2	75081970.51
3907	*FAM111A*	63901	-3.7										11	59142748	59155039
3908	*FAM114A1*	92689										-1.9	4	38867677	38945739
3909	*FAM117B*	150864	-4.4										2	202635188	202769757
3910	*FAM118A*	55007	-2.7										22	45308968	45341955
3911	*FAM122C*	159091	-1.9										X	134796789	134854610
3912	*FAM124A*	220108	-2.3										13	51222334	51284241
3913	*FAM126B*	285172	-3.3										2	200973718	201071671
3914	*FAM127A*	8933										-1.5	X	135032366	135033546
3915	*FAM129A*	116496									-3.3		1	184790724	184974550
3916	*FAM129B*	64855	-3.0										9	127505339	127578989
3917	*FAM131A*	131408	-2.8										3	184335926	184346275
3918	*FAM133A*	286499			4.8								X	93674013	93712274
3919	*FAM133B*	257415	5.0										7	92560793	92590394
3920	*FAM134A*	79137	-3.0										2	219176225	219185479
3921	*FAM135A*	57579								-1.7			6	70412941	70561174
3922	*FAM136A*	84908	4.6										2	70295975	70302090
3923	*FAM13A*	10144					-1.7						4	88725955	89111398
3924	*FAM150B*	285016	-1.8										2	279558	288851
3925	*FAM151A*	338094	-2.0										1	54609182	54623556
3926	*FAM151B*	167555	-3.9										5	80487969	80542563
3927	*FAM153A*	285596	-4.9										5	177707981	177783398
3928	*FAM155A*	728215		-2.3									13	107163510	107866735
3929	*FAM159A*	348378	-2.4										1	52633344	52669683
3930	*FAM160B1*	57700									-1.7		10	114821744	114899832
3931	*FAM161A*	84140	-3.3										2	61824854	61854143
3932	*FAM161B*	145483	-3.7										14	73931501	73950414
3933	*FAM166A*	401565			1.6								9	137243584	137247770
3934	*FAM169B*	283777	-2.4										15	98437162	98514382
3935	*FAM173B*	134145			-3.2								5	10226330	10249897
3936	*FAM175A*	84142	-3.3										4	83459517	83523348
3937	*FAM186A*	121006	-3.4										12	50326230	50396622
3938	*FAM188A*	80013						1.8					10	15778170	15860520
3939	*FAM189A2*	9413	-9.1										9	69324572	69392455
3940	*FAM189B*	10712	-3.6										1	155247205	155255483
3941	*FAM192A*	80011	4.5										16	57152466	57186116
3942	*FAM193A*	8603	3.8										4	2625261	2732565

3943	*FAM195A*	84331		-1.5									16	636817	648474
3944	*FAM19A5*	25817	-1.6										22	48489460	48850912
3945	*FAM200B*	285550	-2.7										4	15681662	15705565
3946	*FAM206A*	54942			-4.9								6.654545	75043032	75117089.02
3947	*FAM208A*	23272	-5.7										3	56620133	56683237
3948	*FAM208B*	54906			2.6								5.304412	75038920	75112957.43
3949	*FAM209A*	200232		2.4									20	56517187	56526142
3950	*FAM20A*	54757		1.5									4.089947	75023842.8	75097808.27
3951	*FAM20B*	9917	-2.8										1	179025804	179076562
3952	*FAM210A*	125228	-2.5										18	13663347	13726663
3953	*FAM213A*	84293			2.6								10	80407829	80437115
3954	*FAM214A*	56204			-2.2								15	52581317	52709817
3955	*FAM217B*	63939	-3.5										20	59933764	59948680
3956	*FAM21C*	253725		-1.5									10	45727200	45792961
3957	*FAM221A*	340277	-6.5										7	23680130	23703249
3958	*FAM227A*	646851	-5.8										22	38578120	38656629
3959	*FAM227B*	196951			2.8								15	49326962	49620931
3960	*FAM228A*	653140	-1.5										2	24175069	24200849
3961	*FAM228B*	375190	-2.2										2	24076526	24169640
3962	*FAM234B*	57613	-2.6										12	13044284	13142521
3963	*FAM3A*	60343	-4.0										X	154506159	154516242
3964	*FAM3B*	54097	-6.0										21	41304212	41357431
3965	*FAM50A*	9130			-2.6								X	154444126	154450654
3966	*FAM57A*	79850	2.5										17	732412	742972
3967	*FAM58A*	92002		-1.7									X	153587919	153600045
3968	*FAM64A*	54478	-3.1										11.80624	75007737.6	75081626.21
3969	*FAM65C*	140876	-4.7										20	50586108	50691528
3970	*FAM71E1*	112703				-3.6							19	50466785	50476753
3971	*FAM72A*	554282			1.8								1	206186179	206204414
3972	*FAM73A*	374986	-4.9										1	77779624	77879539
3973	*FAM81B*	153643	-3.8										5	95391344	95450454
3974	*FAM83A*	84985	-1.9										8	123178960	123210079
3975	*FAM83D*	81610	-4.8										20	38926312	38953106
3976	*FAM83H*	286077								-2.0			8	143723933	143733801
3977	*FAM84A*	151354									-2.3		2	14632686	14650814
3978	*FAM8A1*	51439	-3.0										6	17600355	17611719
3979	*FAM92A1*	137392	-3.3										8	93698561	93731527
3980	*FAM96B*	51647							-1.6				16	66932055	66934423
3981	*FANCA*	2175	-3.0										16	89737549	89816657
3982	*FANCD2OS*	115795			3.5								3	10081317	10108231
3983	*FANCF*	2188	-3.7										11	22622519	22626787
3984	*FANCG*	2189								1.5			9	35073835	35080016
3985	*FANCI*	55215				3.4							15	89243949	89317261
3986	*FAP*	2191										-2.5	2	162170684	162245151
3987	*FAS*	355	-2.6										10	88990531	89015785
3988	*FASN*	2194	3.0										17	82078333	82098332
3989	*FASTKD1*	79675	-7.2										2	169529749	169573875
3990	*FASTKD2*	22868	-3.2										2	206765357	206792509
3991	*FAT3*	120114	-4.3										11	92352096	92896470
3992	*FBF1*	85302	-3.7										17	75910723	75938149
3993	*FBL*	2091		-1.9									19	39834458	39846414
3994	*FBLIM1*	54751	-5.8										4.685185	75022814.8	75096775.37
3995	*FBLN1*	2192	-8.3										22	45502238	45601135
3996	*FBLN2*	2199										-1.8	3	13549131	13638422
3997	*FBRSL1*	57666					1.9						12	132489551	132585188
3998	*FBXL13*	222235	-2.0										7	102813230	103074843
3999	*FBXL18*	80028	-5.9										7	5431335	5513798
4000	*FBXL20*	84961	-5.8										17	39252644	39402523
4001	*FBXL5*	26234				-3.6							4	15604539	15681679

4002	*FBXO15*	201456	-2.1										18	74073353	74147865
4003	*FBXO22*	26263	-3.4										15	75903859	75942510
4004	*FBXO24*	26261	-5.0										7	100583982	100601117
4005	*FBXO27*	126433	-4.2										19	38990714	39032785
4006	*FBXO34*	55030	-2.7										14	55271303	55361918
4007	*FBXO36*	130888	-3.9										2	229922302	230013109
4008	*FBXO41*	150726	2.5										2	73254682	73284431
4009	*FBXO42*	54455	-3.2										11.28528	75004653.6	75078527.52
4010	*FBXO43*	286151	-7.4										8	100133360	100145800
4011	*FBXO45*	200933	-6.1										3	196568611	196589059
4012	*FBXO6*	26270	-5.7										1	11664124	11674354
4013	*FBXW8*	26259	-3.5										12	116910956	117031148
4014	*FCAR*	2204	-6.7										19	54874248	54890472
4015	*FCER1A*	2205	-5.1										1	159289714	159308224
4016	*FCER2*	2208	-5.1										19	7688758	7702146
4017	*FCF1*	51077	-5.9										14	74713144	74738620
4018	*FCGBP*	8857	-2.8										19	39863323	39934626
4019	*FCHO2*	115548	-4.1										5	72955981	73090522
4020	*FCN3*	8547		-1.7									1	27369112	27374824
4021	*FCRL2*	79368	-6.4										1	157745733	157777132
4022	*FDXACB1*	91893	-7.3										11	111874056	111881243
4023	*FDXR*	2232	-3.4										17	74862497	74873031
4024	*FEM1A*	55527	-2.7										19	4791681	4801273
4025	*FER1L5*	90342	-3.0										2	96642737	96704887
4026	*FERMT1*	55612	-1.9										20	6074845	6123544
4027	*FFAR4*	338557	-4.7										10	93566665	93604480
4028	*FGA*	2243										-1.5	4	154583126	154590766
4029	*FGB*	2244	-4.4										4	154562956	154571086
4030	*FGF5*	2250	-4.7										4	80266599	80336680
4031	*FGFBP1*	9982	-1.8										4	15935569	15938740
4032	*FGFR1OP2*	26127		2.1									12	26938383	26966650
4033	*FGFR2*	2263	-5.6										10	121478334	121598458
4034	*FH*	2271					-1.6						1	241497603	241519761
4035	*FHAD1*	114827	-4.4										1	15247272	15400283
4036	*FHL2*	2274		2.5									2	105357712	105438513
4037	*FIBCD1*	84929		-1.5									9	130902438	130939286
4038	*FIBIN*	387758	-2.2										11	26994184	26996121
4039	*FKBP10*	60681	-3.4										17	41812680	41823217
4040	*FKBP14*	55033	-5.4										7	30010587	30026684
4041	*FKBP9*	11328	-4.7										7	32957404	33006931
4042	*FLAD1*	80308	-3.1										1	154983338	154993111
4043	*FLCN*	201163	-6.2										17	17212212	17237188
4044	*FLG2*	388698	-5.8										1	152348735	152360006
4045	*FLII*	2314	4.4										17	18244836	18258916
4046	*FLT1*	2321	-5.0										13	28300344	28495128
4047	*FLVCR1*	28982	-4.2										1	212858255	212899363
4048	*FMN1*	342184	-2.8										15	32765545	33194733
4049	*FMO5*	2330	-3.6										1	147175351	147243050
4050	*FMOD*	2331	-3.7										1	203340628	203351489
4051	*FN3KRP*	79672	-3.3										17	82716683	82730328
4052	*FNBP1L*	54874									2.2		X	75034122.7	75108137.25
4053	*FNBP4*	23360	3.6										11	47716517	47767443
4054	*FNDC1*	84624	-2.0										6	159169397	159272109
4055	*FOPNL*	123811	-3.7										16	15865720	15888625
4056	*FOSB*	2354										1.5	19	45467995	45475179
4057	*FOXH1*	8928	3.0										8	144473412	144476335
4058	*FOXJ3*	22887					-1.5						1	42176539	42335877
4059	*FOXK1*	221937	-5.4										7	4682309	4771443
4060	*FOXK2*	3607	3.7										17	82519713	82644662

4061	*FOXL2NB*	401089	-4.7										3	138947234	138953451
4062	*FOXM1*	2305	-3.8										12	2857681	2877155
4063	*FOXN2*	3344									-1.8		2	48314637	48379294
4064	*FOXO1*	2308	-4.1										13	40555667	40666597
4065	*FPGT*	8790	-2.3										1	74198212	74234086
4066	*FPR2*	2358									-4.1		19	51752026	51770526
4067	*FREM2*	341640	-1.6										13	38687129	38887131
4068	*FRMD4A*	55691	-4.8										10	13643706	14462142
4069	*FRMD4B*	23150	-3.3										3	69169990	69542583
4070	*FRMPD1*	22844	-2.7										9	37651000	37746904
4071	*FRRS1*	391059	-4.4										1	99708703	99766631
4072	*FRS2*	10818	2.6										12	69470349	69579789
4073	*FSCN3*	29999	2.9										7	127591409	127602144
4074	*FSD1*	79187	-2.2										19	4304600	4323843
4075	*FSTL3*	10272				-2.5							19	676365	683399
4076	*FSTL5*	56884			2.9								4	161383897	162164035
4077	*FTL*	2512			-1.9								19	48965301	48966878
4078	*FTO*	79068	-2.7										16	53703963	54121941
4079	*FTSJ2*	29960	-6.4										7	2234231	2242198
4080	*FUT1*	2523	-4.7										19	48748011	48755390
4081	*FUT10*	84750	-4.4										8	33370824	33473422
4082	*FUT11*	170384	-1.9										10	73772291	73780251
4083	*FUT3*	2525	-2.0										19	5842888	5851474
4084	*FUT6*	2528	-3.5										19	5830610	5839731
4085	*FUT9*	10690	-1.7										6	96015984	96215612
4086	*FXN*	2395	-5.1										9	69035259	69100178
4087	*FXR2*	9513	-4.6										17	7591230	7614871
4088	*FXYD1*	5348	-1.6										19	35138808	35143109
4089	*FXYD4*	53828	-1.6										10	43371642	43376335
4090	*FYTTD1*	84248					-1.6						3	197737179	197787596
4091	*FZD1*	8321								-2.2			7	91264364	91271326
4092	*FZD2*	2535	-2.9										17	44557459	44559570
4093	*FZD3*	7976	-4.1										8	28494205	28574268
4094	*FZD4*	8322	-3.1										11	86945679	86955391
4095	*G6PC*	2538	-3.1										17	42900797	42913369
4096	*GAA*	2548									-2.1		17	80101556	80119879
4097	*GAB2*	9846	-2.7										11	78215297	78418348
4098	*GABBR1*	2550		-1.8									6	29555629	29633976
4099	*GABRA4*	2557	-1.7										4	46918900	46994407
4100	*GADD45GIP1*	90480				-3.0							19	12953119	12957236
4101	*GAGE1*	2543			-2.3								X	49589496	49608536
4102	*GAGE10*	102724473			-2.0								X	49303669	49319844
4103	*GAGE12B*	729428			-2.9								X	49551333	49558649
4104	*GAGE12C*	729422			-5.2								X	49532211	49539538
4105	*GAGE12D*	100132399			-5.2								X	49541767	49549094
4106	*GAGE12E*	26748			-5.2								X	49551333	49558649
4107	*GAGE12F*	100008586			-5.2								X	49589496	49608536
4108	*GAGE12G*	645073			-5.2								X	49570434	49577754
4109	*GAGE12H*	729442			-5.2								X	49579983	49587301
4110	*GAGE12J*	729396			-5.2								X	49322057	49329384
4111	*GAGE13*	645051			-5.2								X	49322057	49329384
4112	*GAGE2A*	729447			-5.2								X	49589529	49596827
4113	*GAGE2B*	645037			-5.2								X	49331616	49338952
4114	*GAGE2C*	2574			-5.2								X	49589529	49596827
4115	*GAGE2D*	729408			-5.2								X	49560876	49568205
4116	*GAGE2E*	26749			-5.2								X	49589529	49596827
4117	*GAGE4*	2576			-5.2								X	49560876	49568205
4118	*GAGE5*	2577			-5.2								X	49560876	49568205
4119	*GAGE6*	4103			-3.7								X	151912509	151925170

4120	*GAGE7*	2579			-5.2								X	49560876	49568205
4121	*GAGE8*	100101629			-5.2								X	49589529	49596827
4122	*GAL3ST4*	79690			4.6								7	100159244	100168750
4123	*GALM*	130589	-2.6										2	38665910	38741237
4124	*GALNT15*	117248	-5.3										3	16174649	16231992
4125	*GALNT16*	57452	-4.0										14	69259277	69357033
4126	*GALNT18*	374378	-1.8										11	11270876	11622005
4127	*GALNT3*	2591	2.5										2	165747591	165794682
4128	*GALNT4*	8693	-5.6										12	89519408	89524806
4129	*GALP*	85569	-1.6										19	56176020	56185775
4130	*GAPT*	202309	-1.7										5	58491435	58497090
4131	*GART*	2618	2.9										21	33503931	33543491
4132	*GAS2*	2620			-2.6								11	22625642	22813055
4133	*GAS2L3*	283431						2.8					12	100573683	100628286
4134	*GAS6*	2621						2.2					13	113820549	113864067
4135	*GATA1*	2623	-3.3										X	48786554	48794311
4136	*GATA3*	2625	-3.2										10	8053604	8075198
4137	*GBA*	2629	-3.2										1	155234452	155244699
4138	*GBAS*	2631	-4.7										7	55951793	56000181
4139	*GBP4*	115361	-6.1										1	89181148	89198932
4140	*GBP6*	163351	-3.7										1	89364058	89386461
4141	*GCH1*	2643	2.6										14	54842008	54902852
4142	*GDE1*	51573	-2.7										16	19501689	19522145
4143	*GDF10*	2662									-2.5		10	47300386	47313547
4144	*GDF11*	10220	3.0										12	55743280	55757278
4145	*GDF15*	9518			-6.6								19	18374731	18389176
4146	*GDI1*	2664	3.4										X	154436913	154443467
4147	*GDI2*	2665			1.7								10	5765223	5842132
4148	*GDPD1*	284161	-5.2										17	59220467	59275967
4149	*GDPD3*	79153	-3.3										16	30104810	30113856
4150	*GEN1*	348654	-5.8										2	17753858	17788941
4151	*GFOD1*	54438			4.5								11.11163	75003625.6	75077494.62
4152	*GFOD2*	81577	-5.2										16	67674531	67719421
4153	*GFRA3*	2676	-1.9										5	138252379	138274671
4154	*GGA2*	23062		-2.1									16	23463542	23521995
4155	*GGA3*	23163			-2.5								17	75236599	75262363
4156	*GGCX*	2677	-4.6										2	85544723	85561547
4157	*GGNBP2*	79893	3.3										17	36544888	36589848
4158	*GGT5*	2687	-2.5										22	24219654	24245142
4159	*GGT6*	124975	-6.0										17	4556927	4560818
4160	*GHDC*	84514	-3.8										17	42188799	42194532
4161	*GID4*	79018	-2.7										17	18039292	18068404
4162	*GID8*	54994			-1.7								20	62938119	62948475
4163	*GIGYF1*	64599	-3.6										7	100679507	100689448
4164	*GIMAP7*	168537							2.0				7	150514830	150521073
4165	*GINS1*	9837	-4.8										20	25407727	25452628
4166	*GINS2*	51659			2.9								16	85676198	85690073
4167	*GINS4*	84296	-4.3										8	41529206	41545046
4168	*GIT2*	9815	2.8										12	109929792	109996389
4169	*GJB2*	2706	-2.8										13	20187470	20192898
4170	*GJB3*	2707	-4.2										1	34781189	34786369
4171	*GJB5*	2709	-5.0										1	34755047	34758512
4172	*GJC1*	10052	-5.3										17	44798448	44830816
4173	*GK5*	256356	-3.6										3	142157527	142225607
4174	*GLA*	2717	2.9										X	101397803	101407925
4175	*GLB1L*	79411	-5.3										2	219236606	219245478
4176	*GLG1*	2734				-3.6							16	74447427	74607144
4177	*GLI2*	2736	-1.7										2	120735623	120992653
4178	*GLIS3*	169792	-1.9										9	3824127	4348392

4179	*GLOD5*	392465	-3.8										X	48761750	48773648
4180	*GLRX*	2745									-3.0		5	95751319	95823005
4181	*GLRX3*	10539						1.7					10	130136399	130184521
4182	*GLRX5*	51218	2.6										14	95533503	95544724
4183	*GLS2*	27165	-5.3										12	56470944	56488414
4184	*GLTP*	51228	-3.4										12	109850943	109880488
4185	*GMDS*	2762	-6.4										6	1623806	2245692
4186	*GMPPA*	29926		-1.6									2	219498867	219506989
4187	*GMPR2*	51292	5.0										14	24232422	24239242
4188	*GMPS*	8833							-1.6				3	155870536	155944026
4189	*GNAL*	2774	-2.9										18	11688956	11885685
4190	*GNAS*	2778		1.6									20	58839718	58911192
4191	*GNB2*	2783				-3.5							7	100673531	100679174
4192	*GNB2L1*	10399		-1.6									5	181236909	181248096
4193	*GNG12*	55970								-2.0			1	67701466	67833467
4194	*GNG2*	54331	-2.9										6	75000884.3	75074740.23
4195	*GNG4*	2786	-5.1										1	235547687	235650754
4196	*GNG8*	94235		2.4									19	46634076	46634685
4197	*GNL1*	2794	2.8										6	30541377	30557174
4198	*GNL3*	26354	3.0										3	52681156	52694492
4199	*GNL3L*	54552	-5.5										13.0797	75015276.2	75089200.79
4200	*GNLY*	10578	4.4										2	85685175	85698854
4201	*GNS*	2799	-3.7										12	64713445	64759447
4202	*GOLGA3*	2802				2.5							12	132768909	132828858
4203	*GOLGA6A*	342096		-1.7									15	74069857	74082550
4204	*GOLGB1*	2804					-1.6						3	121663199	121749767
4205	*GOLPH3*	64083	3.0										5	32124704	32174350
4206	*GOT2*	2806	-3.4										16	58707131	58734357
4207	*GP1BB*	2812		2.9									22	19722945	19724771
4208	*GP2*	2813	-2.9										16	20309572	20327808
4209	*GP6*	51206		3.9									19	55013705	55038264
4210	*GPATCH1*	55094	-5.8										19	33080880	33130542
4211	*GPATCH2L*	55668	-5.2										14	76151916	76254342
4212	*GPBP1*	65056					-1.5						5	57173948	57264679
4213	*GPC1*	2817									-2.4		2	240435671	240468078
4214	*GPC4*	2239	-2.9										X	133300103	133415490
4215	*GPC6*	10082	-5.4										13	93226842	94407401
4216	*GPKOW*	27238	-6.6										X	49113389	49123801
4217	*GPLD1*	2822	-2.0										6	24424565	24495205
4218	*GPN3*	51184	-2.7										12	110452484	110469268
4219	*GPNMB*	10457	-2.6										7	23235967	23275108
4220	*GPR1*	2825	-4.0										2	206175316	206218047
4221	*GPR107*	57720						1.5					9	130053426	130140169
4222	*GPR12*	2835	-4.8										13	26755200	26760785
4223	*GPR132*	29933	-2.6										14	105049389	105065445
4224	*GPR137C*	283554									2.0		14	52553148	52637713
4225	*GPR157*	80045	-2.9										1	9100305	9129170
4226	*GPR162*	27239		-1.5									12	6821545	6829972
4227	*GPR37L1*	9283	-2.1										1	202122858	202133592
4228	*GPR82*	27197	-5.1										X	41724155	41730135
4229	*GPR83*	10888	-1.8										11	94377311	94401419
4230	*GPRASP1*	9737								-1.9			X	102651366	102659083
4231	*GPRASP2*	114928				-4.6							X	102712176	102717733
4232	*GPRC5A*	9052	-4.5										12	12890782	12917937
4233	*GPRC5B*	51704	-2.6										16	19856691	19886167
4234	*GPS1*	2873			-2.1								17	82050691	82057470
4235	*GPSM2*	29899	-5.0										1	108875350	108934545
4236	*GPX3*	2878	-8.9										5	151020438	151028993
4237	*GRAP2*	9402		3.1									22	39901082	39973721

4238	*GRASP*	160622	-3.7										12	52006940	52015889
4239	*GRB14*	2888		1.7									2	164492812	164621848
4240	*GREB1*	9687			-2.5								2	11482341	11642788
4241	*GRHPR*	9380	-3.3										9	37422666	37436990
4242	*GRID1*	2894	-2.6										10	85599555	86366493
4243	*GRIK3*	2899	-2.1										1	36795527	37034129
4244	*GRIN3B*	116444	-2.5										19	1000419	1009732
4245	*GRIP1*	23426								-1.5			12	66347431	66804186
4246	*GRIPAP1*	56850	-2.3										X	48973720	49002264
4247	*GRK4*	2868	-3.9										4	2963608	3040747
4248	*GRK7*	131890	-2.4										3	141778148	141818490
4249	*GRM6*	2916	-6.1										5	178978327	178996206
4250	*GRM7*	2917	-3.4										3	6770001	7741533
4251	*GRP*	2922	-1.6										18	59220168	59230774
4252	*GRPEL1*	80273	3.1										4	7058906	7068197
4253	*GRTP1*	79774	-5.0										13	113324164	113364148
4254	*GSDMA*	284110	-4.4										17	39962973	39977766
4255	*GSDMC*	56169	-4.7										8	129748196	129786888
4256	*GSE1*	23199	3.3										16	85611409	85676204
4257	*GSG1*	83445	-3.8										12	13083560	13103683
4258	*GSK3A*	2931				-2.6							19	42230186	42242625
4259	*GSN*	2934	-4.9										9	121207794	121332843
4260	*GSS*	2937	-3.5										20	34928430	34955817
4261	*GSTCD*	79807	-1.9										4	105708778	105847728
4262	*GSTM5*	2949	-2.0										1	109712255	109775428
4263	*GSTO2*	119391									-1.9		10	104268873	104304945
4264	*GSTT2*	2953				-3.6							22	23980058	23983915
4265	*GSTT2B*	653689				-3.6							22	23957414	23961186
4266	*GSTZ1*	2954	-3.7										14	77320884	77331597
4267	*GTF2F2*	2963	-4.6										13	45120515	45284909
4268	*GTF2H2*	2966	-5.2										5	71032670	71067689
4269	*GTF2IRD1*	9569	-2.8										7	74453790	74602604
4270	*GTF2IRD2B*	389524	-4.1										7	75092573	75149817
4271	*GTF3C1*	2975	-3.9										16	27459555	27549913
4272	*GTF3C4*	9329	2.9										9	132670035	132694955
4273	*GTPBP10*	85865	-4.3										7	90335223	90391455
4274	*GTSF1*	121355			9.2								12	54455950	54473602
4275	*GUCA1A*	2978	-2.2										6	42155406	42180056
4276	*GUCA1B*	2979	-2.9										6	42184401	42194916
4277	*GUCD1*	83606	-3.2										22	24540423	24555935
4278	*GULP1*	51454	-3.1										2	188291669	188595931
4279	*GYG1*	2992			3.6								3	148991341	149027668
4280	*GYPA*	2993	2.9										4	144109303	144140751
4281	*GZF1*	64412	-2.3										20	23362182	23373063
4282	*GZMA*	3001									-3.3		5	55102648	55110252
4283	*GZMH*	2999	4.4										14	24606480	24609699
4284	*H2AFZ*	3015			2.0								4	99948086	99950388
4285	*H6PD*	9563	-6.5										1	9234775	9271337
4286	*HABP4*	22927	2.9										9	96450201	96491336
4287	*HACD1*	9200	-2.0										10	17589032	17617377
4288	*HACE1*	57531	-3.0										6	104728093	104859919
4289	*HARBI1*	283254	-1.8										11	46602861	46617909
4290	*HAUS2*	55142	-3.1										15	42548810	42569994
4291	*HAUS3*	79441	-5.0										4	2227464	2242164
4292	*HAUS6*	54801				2.5							2.899471	75025898.8	75099874.07
4293	*HAUS8*	93323	-4.6										19	17049729	17075625
4294	*HAVCR2*	84868	-3.3										5	157085832	157142869
4295	*HBA1*	3039	3.7										16	176680	177522
4296	*HBA2*	3040	4.4										16	172847	173710

4297	*HBEGF*	1839			-1.6								5	140332843	140346631
4298	*HBG1*	3047	5.8										11	5248083	5249892
4299	*HBG2*	3048	4.0										11	5248274	5645789
4300	*HCAR1*	27198	-4.4										12	122726076	122730843
4301	*HCAR2*	338442	-1.9										12	122701293	122703343
4302	*HCCS*	3052				-3.0							X	11111301	11123078
4303	*HDAC11*	79885	-3.8										3	13479724	13506424
4304	*HDAC3*	8841	3.9										5	141620876	141636870
4305	*HDAC6*	10013				-4.4							X	48801377	48824982
4306	*HDAC8*	55869	2.8										X	72329516	72573103
4307	*HDAC9*	9734	-2.4										7	18086949	19002416
4308	*HDDC2*	51020					-1.8						6	125219962	125302078
4309	*HDDC3*	374659	-3.0										15	90929964	90932569
4310	*HDGFRP2*	84717	-3.3										19	4472287	4502211
4311	*HDLBP*	3069							-1.6				2	241227264	241317061
4312	*HEATR1*	55127			-3.1								1	236549005	236604504
4313	*HEATR4*	399671	-2.7										14	73478484	73558947
4314	*HEATR5A*	25938	-6.1										14	31291791	31420582
4315	*HECA*	51696									-2.1		6	139135112	139180802
4316	*HEMK1*	51409	-5.9										3	50569152	50596168
4317	*HERC1*	8925	-2.8										15	63608618	63833942
4318	*HERC4*	26091	-5.4										10	67921899	68075348
4319	*HERC5*	51191				-3.4							4	88457117	88506163
4320	*HES2*	54626	-6.1										13.54277	75018017.5	75091955.18
4321	*HEXDC*	284004							1.7				17	82418318	82442645
4322	*HEYL*	26508	-2.2										1	39624153	39639945
4323	*HGF*	3082	-2.0										7	81699006	81770438
4324	*HHEX*	3087							1.5				10	92689951	92695646
4325	*HIBCH*	26275				-2.0							2	190189735	190344193
4326	*HIF1AN*	55662	3.0										10	100529072	100559998
4327	*HIF3A*	64344	-3.9										19	46297046	46343433
4328	*HILPDA*	29923	-6.0										7	128455849	128458418
4329	*HINT2*	84681	-3.5										9	35812960	35815354
4330	*HIP1*	3092	-4.8										7	75533300	75738962
4331	*HIST1H2AA*	221613		1.6									6	25726132	25726527
4332	*HIST1H2AE*	3012		2.0									6	26216975	26217483
4333	*HIST1H2AI*	8329		1.9									6	27808199	27808701
4334	*HIST1H2AM*	8336		1.6									6	27892757	27893149
4335	*HIST1H2BB*	3018		1.5									6	26043227	26123904
4336	*HIST1H3B*	8358		2.8									6	26031650	26032060
4337	*HIST1H4H*	8365	-3.6										6	26277609	26285638
4338	*HIST2H2AA3*	8337			-3.3								1	149842188	149842736
4339	*HIST2H2AA4*	723790			-3.3								1	149851061	149851624
4340	*HIST2H2AC*	8338	-1.6										1	149886975	149887364
4341	*HIST2H2BF*	440689	-6.4										1	149782689	149812373
4342	*HIST4H4*	121504	-1.8										12	14767999	14771131
4343	*HK1*	3098					1.5						10	69269984	69401882
4344	*HKR1*	284459	-4.3										19	37312837	37369365
4345	*HLA-DPB1*	3115		-1.6									6	33075926	33087201
4346	*HLA-DQB2*	3120		-2.6									6	32756098	32763534
4347	*HLA-DRB3*	3125		-1.6									6	3824511	3837575
4348	*HLA-DRB5*	3127		-2.7									6	32517343	32530287
4349	*HLA-F*	3134	3.4										6	29722775	29738528
4350	*HMBOX1*	79618	2.7										8	28890394	29064764
4351	*HMGCS1*	3157										1.8	5	43289395	43313512
4352	*HMGCS2*	3158	-4.5										1	119747996	119768905
4353	*HMGN2*	3151		-1.5									1	26472450	26475972
4354	*HMGN3*	9324										1.7	6	79201245	79234738
4355	*HN1*	51155	4.3										17	75135248	75168281

4356	*HNRNPA0*	10949				2.9							5	137745651	137754376
4357	*HNRNPA1L2*	144983		-1.7									13	52640786	52643796
4358	*HNRNPH3*	3189	4.6										10	68331174	68343191
4359	*HNRNPM*	4670										-1.5	19	8444767	8489114
4360	*HNRNPUL1*	11100	-3.2										19	41262496	41307598
4361	*HOGA1*	112817	-4.4										10	97584323	97612802
4362	*HOMER2*	9455	4.3										15	82836946	82986153
4363	*HOMEZ*	57594	-3.7										14	23272422	23299447
4364	*HOOK3*	84376	-5.8										8	42896932	43030539
4365	*HOXB5*	3215	-7.5										17	48591257	48593961
4366	*HOXD1*	3231			-2.1								2	176188579	176190907
4367	*HP*	3240					2.0						16	72054592	72061055
4368	*HPCAL1*	3241	-3.1										2	10302889	10427617
4369	*HPD*	3242	-2.7										12	121839527	121863596
4370	*HPGD*	3248	-4.6										4	174490177	174523154
4371	*HPS1*	3257										-3.5	10	98416198	98446947
4372	*HPS3*	84343	-2.4										3	149129584	149173732
4373	*HPS4*	89781	-3.5										22	26443423	26483837
4374	*HRH1*	3269	-2.6										3	11137093	11263557
4375	*HRH2*	3274	-1.9										5	175658030	175686242
4376	*HRH4*	59340	-1.7										18	24460629	24479957
4377	*HRSP12*	10247	-3.1										8	98102344	98117241
4378	*HS6ST1*	9394								-1.5			2	128236716	128318577
4379	*HSD17B1*	3292	-3.0										17	42549214	42555213
4380	*HSD17B10*	3028		-2.2									X	53431258	53434373
4381	*HSD17B13*	345275	-6.1										4	87303789	87322906
4382	*HSD17B7*	51478	-4.2										1	162790702	162812817
4383	*HSD17B8*	7923	-2.6										6	33204642	33206831
4384	*HSD3B2*	3284	-2.1										1	119414931	119423035
4385	*HSF1*	3297	3.5										8	144291591	144314722
4386	*HSH2D*	84941		-1.6									19	16134028	16158575
4387	*HSPA12A*	259217	-4.2										10	116671192	116742574
4388	*HSPA13*	6782	2.5										21	14371115	14383484
4389	*HSPA14*	51182	2.6										10	14838164	14871741
4390	*HSPA1A*	3303	-3.1										6	31815464	31817946
4391	*HSPA2*	3306	2.6										14	64535905	64546173
4392	*HSPA4*	3308							-1.7				5	133051962	133106449
4393	*HSPA5*	3309							-1.7				9	125234853	125241330
4394	*HSPB6*	126393	-8.0										19	35754568	35758079
4395	*HSPB8*	26353			-1.6								12	119178642	119221131
4396	*HSPD1*	3329		-1.6									2	197486581	197516737
4397	*HSPG2*	3339	-5.5										1	21822245	21937297
4398	*HSPH1*	10808		-3.3									13	31134974	31162388
4399	*HTATIP2*	10553	2.8										11	20363685	20383783
4400	*HTR2B*	3357			-1.7								2	231108230	231125118
4401	*HTR3E*	285242	-5.3										3	184097064	184106995
4402	*HTRA3*	94031	-2.4										4	8269765	8307111
4403	*HTRA4*	203100	-5.0										8	38974164	38988662
4404	*HUS1*	3364			1.5								7	47695730	47979581
4405	*HYAL4*	23553	-1.9										7	123828983	123877478
4406	*HYI*	81888						2.2					1	43451003	43453989
4407	*HYOU1*	10525			-1.8								11	119044189	119057202
4408	*IAPP*	3375	-6.6										12	21354959	21379978
4409	*IARS*	3376	-3.6										9	92210207	92293756
4410	*IARS2*	55699	-3.4										1	220094102	220148041
4411	*IBA57*	200205	-3.1										1	228165815	228182257
4412	*ICA1*	3382			-1.7								7	8113184	8262687
4413	*ICA1L*	130026	-6.5										2	202773150	202871985
4414	*ICK*	22858	-2.9										6	53001279	53061802

4415	*ICMT*	23463	-4.2										1	6221193	6235972
4416	*ICOSLG*	23308		-2.1									21	44222991	44240966
4417	*IDE*	3416								-1.8			10	92451684	92574076
4418	*IDH2*	3418	3.2										15	90083045	90102504
4419	*IDH3B*	3420			-2.9								20	2658395	2664219
4420	*IDI1*	3422			3.6								10	1039908	1049170
4421	*IDI2*	91734			3.6								10	1018907	1025859
4422	*IDNK*	414328			-1.8								9	83623049	83644130
4423	*IDO1*	3620	-3.8										8	39902275	39928444
4424	*IDO2*	169355	-3.6										8	39934614	40016391
4425	*IER3*	8870							-1.5				6	30743199	30744554
4426	*IFI27*	3429				-6.6							14	94104836	94116698
4427	*IFI27L2*	83982						1.7					14	94127779	94130253
4428	*IFIH1*	64135	-3.1										2	162267079	162318703
4429	*IFIT2*	3433									-3.5		10	89301955	89309276
4430	*IFIT3*	3437	-5.2										10	89327894	89340971
4431	*IFNAR1*	3454	-3.9										21	33324477	33359862
4432	*IFNAR2*	3455					1.5						21	33229901	33265675
4433	*IFNGR1*	3459									-2.1		6	137197484	137219449
4434	*IFNGR2*	3460									-2.3		21	33402896	33479348
4435	*IFNL1*	282618	-2.8										19	39296325	39298673
4436	*IFNLR1*	163702	-2.2										1	24154157	24187959
4437	*IFT122*	55764						1.5					3	129440036	129520507
4438	*IFT140*	9742	-3.5										16	1510427	1612110
4439	*IFT172*	26160	-2.8										2	27444371	27489789
4440	*IFT20*	90410	5.1										17	28328325	28335489
4441	*IFT22*	64792	-3.1										7	101313367	101321823
4442	*IFT46*	738		-1.8									11	65089324	65111860
4443	*IFT52*	51098					-1.5						20	43590931	43647296
4444	*IFT57*	55081	5.6										3	108160812	108222570
4445	*IFT74*	80173						2.1					9	26947039	27062930
4446	*IFT80*	57560	-3.0										3	160256986	160399880
4447	*IGF1*	3479	-7.3										12	102395867	102480645
4448	*IGF1R*	3480			-2.5								15	98648971	98964530
4449	*IGF2*	3481	-7.4										11	2129112	2141238
4450	*IGFBP6*	3489	-4.2										12	53097436	53102345
4451	*IGSF21*	84966						1.6					1	18107746	18378483
4452	*IGSF6*	10261							1.8				16	21639537	21652660
4453	*IGSF9B*	22997	-1.7										11	133908564	133956985
4454	*IKBIP*	121457	-3.5										12	98613405	98645113
4455	*IKBKE*	9641	-2.5										1	206470476	206496889
4456	*IL10*	3586	-4.9										1	206767602	206772494
4457	*IL11*	3589	-4.0										19	55364389	55370463
4458	*IL11RA*	3590								-1.6			9	34650702	34661892
4459	*IL12RB2*	3595	-3.4										1	67307364	67396900
4460	*IL15RA*	3601	-1.7										10	5937569	5978187
4461	*IL17RC*	84818	-2.8										3	9917074	9933630
4462	*IL17RD*	54756	-4.6										4.28836	75023500.1	75097463.97
4463	*IL17RE*	132014	-2.5										3	9902612	9916402
4464	*IL18*	3606	-2.7										11	112143251	112164117
4465	*IL18BP*	10068		-2.0									11	71998541	72005715
4466	*IL23R*	149233	-4.8										1	67166400	67259979
4467	*IL27RA*	9466	3.0										19	14031748	14053216
4468	*IL2RB*	3560	5.3										22	37125838	37175054
4469	*IL2RG*	3561									-2.4		X	71107404	71112108
4470	*IL32*	9235	-4.1										16	3065297	3082192
4471	*IL4R*	3566									-2.6		16	27313668	27364778
4472	*IL6*	3569			-2.0								7	22725884	22732002
4473	*IL7R*	3575						-2.1					5	35852695	35879603

4474	*ILF3*	3609	5.0										19	10654261	10692417
4475	*IMPA2*	3613	-3.7										18	11981025	12030883
4476	*IMPDH1*	3614					2.5						7	128392277	128410252
4477	*IMPG2*	50939	-2.4										3	101222546	101320560
4478	*INA*	9118										2.0	10	103277163	103290351
4479	*INCA1*	388324	-4.7										17	4988130	4997610
4480	*ING1*	3621	-3.6										13	110712736	110723339
4481	*INHBC*	3626						1.5					12	57434760	57450828
4482	*INIP*	58493	-3.2										9	112683926	112718236
4483	*INMT*	11185	-6.8										7	30697985	30757602
4484	*INO80*	54617		-2.0									13.427	75017332.2	75091266.59
4485	*INO80B*	83444	-4.8										2	74455023	74457960
4486	*INPP4A*	3631									-1.5		2	98444854	98594390
4487	*INPP5A*	3632	-2.9										10	132537820	132783480
4488	*INPP5F*	22876			1.8								10	119726097	119829278
4489	*INPP5K*	51763									-2.5		17	1494571	1516888
4490	*INPPL1*	3636					1.5						11	72223701	72239105
4491	*INSIG1*	3638	-3.1										7	155297776	155310235
4492	*INS-IGF2*	723961	-7.4										11	2132538	2161209
4493	*INTS10*	55174	-2.9										8	19817140	19852083
4494	*INTS2*	57508	-3.5										17	61865367	61928016
4495	*INTS3*	65123	-4.0										1	153728067	153774808
4496	*INTS6*	26512				-3.8							13	51354077	51454264
4497	*INTS9*	55756			1.8								8	28767661	28890242
4498	*INTU*	27152	-1.8										4	127623271	127726737
4499	*IP6K2*	51447			1.6								3	48688003	48740353
4500	*IPO7*	10527					-1.6						11	9384622	9448126
4501	*IPO9*	55705				-1.6							1	201829141	201884294
4502	*IPPK*	64768				-1.8							9	92613184	92670265
4503	*IQCA1*	79781		-1.5									2	236324147	236507542
4504	*IQCC*	55721	-2.9										1	32205661	32208687
4505	*IQCJ-SCHIP1*	100505385			4.2								3	158962235	159897366
4506	*IQGAP3*	128239			4.1								1	156525405	156572604
4507	*IQSEC1*	9922	3.9										3	12897220	13073117
4508	*IRAK1*	3654										-1.6	X	154010500	154019980
4509	*IRAK2*	3656	-4.0										3	10164865	10243743
4510	*IREB2*	3658									-1.6		15	78437431	78501456
4511	*IRF2BP2*	359948							-1.6				1	234604269	234609525
4512	*IRF7*	3665					2.6						11	612553	615999
4513	*IRF9*	10379				-3.9							14	24161053	24166565
4514	*IRGQ*	126298	-5.2										19	43584369	43596135
4515	*ISCA1*	81689						2.0					9	86264546	86282538
4516	*ISG20*	3669									-2.5		15	88636153	88656483
4517	*ISLR*	3671										-3.3	15	74173671	74176872
4518	*ISOC2*	79763	-1.7										19	55452985	55462343
4519	*ISPD*	729920	-4.8										7	16087527	16421322
4520	*IST1*	9798				4.3							16	71845996	71931199
4521	*ISY1*	57461	-5.7										3	129127415	129161293
4522	*ITFG2*	55846			-3.1								12	2812622	2859791
4523	*ITGA1*	3672	-6.3										5	52787896	52959210
4524	*ITGA10*	8515	-4.9										1	145891208	145910189
4525	*ITGA11*	22801	-1.5										15	68296533	68432162
4526	*ITGA3*	3675	-3.0										17	50055968	50090481
4527	*ITGA7*	3679			-1.6								12	55684568	55716043
4528	*ITGAD*	3681	-1.6										16	31393312	31426505
4529	*ITGAE*	3682	-3.9										17	3714628	3801243
4530	*ITGB1BP1*	9270	-4.0										2	9403475	9423547
4531	*ITGB4*	3691	-2.0										17	75721328	75757818
4532	*ITGB6*	3694	-5.7										2	160099666	160271888

4533	*ITIH2*	3698										2.2	10	7703269	7749520
4534	*ITIH4*	3700		-2.1									3	52812975	52831479
4535	*ITIH5*	80760	-5.5										10	7559270	7666998
4536	*ITM2C*	81618	-3.0										2	230864639	230879248
4537	*ITPK1*	3705	-3.4										14	92936914	93116320
4538	*ITPKC*	80271	2.7										19	40717103	40740860
4539	*ITPR3*	3710			-2.4								6	33620365	33696574
4540	*ITPRIP*	85450			-2.1								10	104309698	104338404
4541	*ITPRIPL2*	162073								-1.9			16	19113932	19121629
4542	*ITSN1*	6453	-5.8										21	33642400	33899861
4543	*IYD*	389434	-3.9										6	150368892	150405969
4544	*JCHAIN*	3512	-2.3										4	70655541	70681817
4545	*JMJD7-PLA2G4B*	8681	-4.0										15	41828095	41848155
4546	*JMJD8*	339123						1.6					16	681671	684528
4547	*JPH2*	57158	-3.1										20	44111695	44187578
4548	*JUN*	3725	-3.5										1	58780788	58784327
4549	*JUNB*	3726									-2.6		19	12791496	12793315
4550	*JUND*	3727	-3.7										19	18279760	18281622
4551	*JUP*	3728		-1.6									17	41754604	41786931
4552	*KANK2*	25959	-4.0										19	11164267	11197791
4553	*KANK4*	163782	-3.8										1	62236979	62319414
4554	*KANSL1L*	151050	-4.9										2	210021423	210171383
4555	*KANSL3*	55683	-4.6										2	96593170	96642787
4556	*KAT2A*	2648	-5.1										17	42113108	42121358
4557	*KATNAL2*	83473	-4.7										18	46917492	47102243
4558	*KATNB1*	10300					1.5						16	57735730	57757250
4559	*KATNBL1*	79768									-2.0		15	34140674	34210096
4560	*KAZN*	23254	-1.6										1	14598704	15118043
4561	*KBTBD4*	55709	-6.8										11	47572197	47579015
4562	*KCNA7*	3743	-3.2										19	49067418	49072941
4563	*KCNE3*	10008					1.7						11	74454841	74467729
4564	*KCNE4*	23704	-1.9										2	223051814	223198399
4565	*KCNH4*	23415	-1.6										17	42156891	42181278
4566	*KCNH8*	131096	-2.0										3	19148454	19535646
4567	*KCNJ5*	3762	-3.8										11	128891356	128921035
4568	*KCNK2*	3776	-2.0										1	215005775	215237093
4569	*KCNK3*	3777	-3.4										2	26692690	26733420
4570	*KCNN2*	3781			4.2								5	114360945	114496500
4571	*KCNN3*	3782	-4.0										1	154697455	154870280
4572	*KCNN4*	3783	-2.9										19	43766533	43781257
4573	*KCNQ3*	3786	-5.7										8	132120859	132480953
4574	*KCNRG*	283518	-4.3										13	50015254	50020922
4575	*KCTD10*	83892								-1.6			12	109448656	109477544
4576	*KCTD12*	115207	-8.6										13	76880166	76886390
4577	*KCTD18*	130535								-1.6			2	200488952	200519784
4578	*KCTD19*	146212	-2.3										16	67289428	67326763
4579	*KDELC1*	79070								1.8			13	102784281	102799007
4580	*KDELC2*	143888	-4.2										11	108472105	108498432
4581	*KDM3A*	55818							-1.9				2	86440647	86492716
4582	*KDR*	3791	-1.9										4	55078477	55125589
4583	*KEAP1*	9817			-1.8								19	10486120	10503741
4584	*KHDC1*	80759	-1.6										6	73241314	73310365
4585	*KHDC1L*	100129128	-2.1										6	73223544	73225770
4586	*KHK*	3795	-1.6										2	27086747	27100772
4587	*KIAA0101*	9768	-5.3										15	64364311	64387687
4588	*KIAA0141*	9812		-1.6									5	141923808	141942047
4589	*KIAA0196*	9897	-2.9										8	125024260	125091840
4590	*KIAA0232*	9778	3.7										4	6781375	6884170
4591	*KIAA0355*	9710	-4.2										19	34254537	34355586

4592	*KIAA0368*	23392					-1.6						9	111360692	111484745
4593	*KIAA0430*	9665									-1.5		16	15594386	15643166
4594	*KIAA0825*	285600	-3.4										5	94152966	94618604
4595	*KIAA0922*	23240	-4.0										4	153466346	153636711
4596	*KIAA0930*	23313	-4.8										22	45190338	45240769
4597	*KIAA1143*	57456					-1.8						3	44737661	44761662
4598	*KIAA1147*	57189	-4.6										7	141656728	141702153
4599	*KIAA1210*	57481	-3.0										X	119078635	119150579
4600	*KIAA1324*	57535		2.9									1	109113679	109206781
4601	*KIAA1328*	57536	-3.5										18	36829106	37232172
4602	*KIAA1456*	57604	-4.7										8	12945642	13031503
4603	*KIAA1468*	57614	-2.2										18	62187258	62307829
4604	*KIAA1614*	57710	-6.3										1	180913154	180951614
4605	*KIAA1671*	85379								-1.9			22	24952730	25197448
4606	*KIAA1755*	85449	-2.0										20	38210488	38260772
4607	*KIAA1919*	91749	-7.7										6	111259348	111271167
4608	*KIAA2013*	90231									-1.9		1	11919591	11926428
4609	*KIDINS220*	57498					-1.6						2	8725278	8837630
4610	*KIF14*	9928	-2.0										1	200551497	200620734
4611	*KIF18B*	146909	-6.0										17	44924709	44947711
4612	*KIF1B*	23095	-3.5										1	10210805	10381603
4613	*KIF20A*	10112			1.9								5	138178719	138187715
4614	*KIF3A*	11127	-5.0										5	132692628	132737638
4615	*KIF5A*	3798	-2.9										12	57549998	57586632
4616	*KIF5B*	3799	3.3										10	32009010	32056431
4617	*KIF5C*	3800	5.1										2	148875250	149026759
4618	*KIF9*	64147	2.9										3	47228026	47283451
4619	*KIFC3*	3801	-3.3										16	57758217	57863053
4620	*KIRREL3*	84623	-2.4										11	126423359	127003460
4621	*KLB*	152831	-3.3										4	39406853	39451536
4622	*KLC1*	3831	-3.6										14	103561896	103714249
4623	*KLC3*	147700	-3.8										19	45333434	45351520
4624	*KLF10*	7071	3.6										8	102648779	102655902
4625	*KLF8*	11279	-5.1										X	56232421	56287889
4626	*KLF9*	687	-2.6										9	70384597	70414624
4627	*KLHDC4*	54758		-1.6									3.891534	75024185.5	75098152.57
4628	*KLHDC8B*	200942						2.6					3	49171611	49176486
4629	*KLHL12*	59349	-3.0										1	202891100	202928636
4630	*KLHL18*	23276	3.6										3	47282917	47346816
4631	*KLHL21*	9903	-5.7										1	6590724	6614607
4632	*KLHL23*	151230	-4.5										2	169694488	169776989
4633	*KLHL24*	54800			-2.8								3.097884	75025556.1	75099529.77
4634	*KLHL3*	26249	-1.9										5	137617500	137736090
4635	*KLHL7*	55975	-1.7										7	23105758	23177914
4636	*KLK10*	5655	-9.3										19	51012739	51020175
4637	*KLK11*	11012	-4.3										19	51022216	51028039
4638	*KLK12*	43849	-2.8										19	51029092	51035230
4639	*KLK2*	3817	-2.0										19	50861568	50880567
4640	*KLK3*	354	-2.7										19	50854915	50860764
4641	*KLK4*	9622	-4.8										19	50906352	50910738
4642	*KLK5*	25818	-2.2										19	50943303	50953093
4643	*KLK7*	5650	-1.9										19	50976482	50984099
4644	*KLKB1*	3818	-1.8										4	186208979	186258471
4645	*KLLN*	100144748	-6.3										10	87859161	87863437
4646	*KLRD1*	3824	-5.0										12	10226058	10329600
4647	*KMO*	8564		-1.6									1	241532134	241595642
4648	*KMT2E*	55904	2.7										7	105014179	105114361
4649	*KNOP1*	400506	-6.4										16	19701934	19718235
4650	*KNSTRN*	90417			3.3								15	40382721	40394246

4651	*KPNA2*	3838							-1.5				17	68035519	68046842
4652	*KPNA6*	23633										-2.0	1	32108038	32176568
4653	*KPNA7*	402569	-2.1										7	99173574	99207506
4654	*KPNB1*	3837	2.8										17	47649476	47685505
4655	*KREMEN1*	83999	-6.0										22	29073078	29168333
4656	*KRI1*	65095	3.2										19	10553078	10566037
4657	*KRR1*	11103	2.9										12	75490861	75511636
4658	*KRT1*	3848	3.7										12	52674736	52680407
4659	*KRT10*	3858	3.0										17	40818117	40822595
4660	*KRT13*	3860	-10.0										17	41500981	41505705
4661	*KRT16*	3868	-2.5										17	41609778	41615899
4662	*KRT17*	3872	-7.6										17	41619437	41624842
4663	*KRT18*	3875									-3.4		12	52948871	52952901
4664	*KRT5*	3852	-6.7										12	52514575	52520687
4665	*KRT6A*	3853	-9.6										12	52487174	52493257
4666	*KRT6B*	3854	-4.3										12	52446651	52452126
4667	*KRT7*	3855	-5.9										12	52232520	52252186
4668	*KRT71*	112802								-2.2			12	52543909	52553147
4669	*KRT81*	3887	-2.2										12	52285913	52291534
4670	*KRTAP15-1*	254950	-1.5										21	30440275	30440945
4671	*KRTCAP2*	200185							-1.6				1	155169408	155173475
4672	*KRTCAP3*	200634	4.1										2	27442366	27446481
4673	*KRTDAP*	388533	-2.9										19	35487324	35495558
4674	*KSR1*	8844	-4.5										17	27456714	27626438
4675	*KTI12*	112970			-1.8								1	52032103	52033816
4676	*KXD1*	79036	4.2										19	18557762	18569387
4677	*KYNU*	8942					1.7						2	142877498	143055832
4678	*L1CAM*	3897	-3.4										X	153861514	153909223
4679	*L1TD1*	54596	-6.3										13.36912	75016989.5	75090922.29
4680	*L2HGDH*	79944	-5.9										14	50237563	50312548
4681	*LACE1*	246269	-1.9										6	108294894	108526796
4682	*LAD1*	3898	-2.4										1	201373244	201399915
4683	*LAMA1*	284217	-5.9										18	6941744	7117814
4684	*LAMA2*	3908	-3.3										6	128883141	129516569
4685	*LAMA5*	3911	-4.2										20	62307955	62367312
4686	*LAMB1*	3912			2.1								7	107923799	108003255
4687	*LAMB2*	3913	-2.9										3	49121114	49133118
4688	*LAMB3*	3914	-7.5										1	209614870	209652466
4689	*LAMB4*	22798	-7.5										7	108023548	108130356
4690	*LAMC1*	3915	3.2										1	183023460	183145592
4691	*LAMC2*	3918	-3.7										1	183186238	183244900
4692	*LAMP3*	27074	-4.0										3	183122213	183163839
4693	*LAMTOR4*	389541									-2.0		7	100148907	100155944
4694	*LAMTOR5*	10542	3.9										1	110401249	110407942
4695	*LAPTM4B*	55353				1.5							8	97775057	97853013
4696	*LARP1*	23367	4.2										5	154712902	154817607
4697	*LARP4*	113251	4.3										12	50392383	50480004
4698	*LARP7*	51574	3.2										4	112636964	112657592
4699	*LARS*	51520					-1.6						5	146113038	146182660
4700	*LAT*	27040									-1.7		16	28984826	28990783
4701	*LATS1*	9113	-3.0										6	149658153	149718256
4702	*LAX1*	54900	-2.0										6.933824	75037549.4	75111580.24
4703	*LBHD1*	79081	3.3										11	62662817	62672255
4704	*LCA5L*	150082	-7.3										21	39405844	39445805
4705	*LCAT*	3931	-2.3										16	67939750	67944131
4706	*LCK*	3932									-1.8		1	32251239	32286165
4707	*LCMT1*	51451	-5.6										16	25111729	25178231
4708	*LCMT2*	9836	-2.8										15	43323649	43330605
4709	*LCOR*	84458	3.4										10	96832260	96981043

4710	*LCT*	3938	-2.2										2	135787840	135837180
4711	*LDB1*	8861		-1.7									10	102107560	102120453
4712	*LDB3*	11155	-3.2										10	86668449	86736068
4713	*LDLRAD2*	401944	-2.9										1	21812265	21825221
4714	*LDLRAD4*	753					-1.5						18	13217498	13652755
4715	*LDOC1*	23641	-3.8										X	141175745	141177125
4716	*LEF1*	51176									-3.0		4	108047545	108168956
4717	*LENEP*	55891	-3.9										1	154993586	154994315
4718	*LETM2*	137994	-2.6										8	38386207	38409527
4719	*LFNG*	3955		-1.6									7	2512529	2529177
4720	*LGALS4*	3960	-5.6										19	38801671	38813364
4721	*LGALS8*	3964			-3.1								1	236518000	236552981
4722	*LGI3*	203190	-4.3										8	22146825	22157084
4723	*LGR6*	59352	-3.5										1	202193901	202319781
4724	*LGSN*	51557	-3.0										6	63275951	63319977
4725	*LHFP*	10186	-2.6										13	39342892	39603528
4726	*LHFPL5*	222662	-4.6										6	35805293	35833874
4727	*LIAS*	11019	-5.8										4	39459000	39477653
4728	*LIG1*	3978	-2.4										19	48115445	48170603
4729	*LILRA6*	79168	-3.7										19	54236592	54242791
4730	*LIMCH1*	22998	3.5										4	41359607	41700044
4731	*LIMD1*	8994	-3.9										3	45555394	45686338
4732	*LIMD2*	80774	3.1										17	63695902	63701172
4733	*LIMK1*	3984	-5.2										7	74082933	74122525
4734	*LIMS2*	55679	-1.7										2	127638381	127681786
4735	*LIN28A*	79727	-7.0										1	26410778	26429722
4736	*LIN52*	91750	-3.6										14	74084796	74201235
4737	*LIN54*	132660	-5.3										4	82909973	83012926
4738	*LIN7B*	64130	-3.0										19	49114324	49118460
4739	*LLGL1*	3996	-4.3										17	18225587	18244875
4740	*LMCD1*	29995	-1.9										3	8501707	8574673
4741	*LMNA*	4000						3.6					1	156082573	156140089
4742	*LMNTD1*	160492	-2.6										12	25409307	25648579
4743	*LMOD1*	25802	-4.5										1	201896452	201946588
4744	*LMOD3*	56203	-5.6										3	69106872	69123032
4745	*LMX1B*	4010	-2.7										9	126614443	126701032
4746	*LNX2*	222484	-4.0										13	27545911	27620404
4747	*LOXL2*	4017	-1.9										8	23297189	23425328
4748	*LPAR1*	1902	-3.7										9	110873263	111038458
4749	*LPAR6*	10161								-1.9			13	48389567	48444704
4750	*LPCAT2*	54947	-4.2										6.569697	75043374.7	75117433.32
4751	*LPGAT1*	9926	-6.0										1	211743457	211830772
4752	*LPIN2*	9663	3.7										18	2916994	3013315
4753	*LPIN3*	64900	-2.3										20	41340920	41360582
4754	*LPO*	4025	-2.3										17	58218548	58268518
4755	*LRAT*	9227	-2.5										4	154626945	154753118
4756	*LRIG1*	26018	-4.9										3	66378797	66501263
4757	*LRP10*	26020	-4.2										14	22871613	22881580
4758	*LRP12*	29967	-5.5										8	104489231	104589024
4759	*LRP1B*	53353	-1.7										2	140231423	142131701
4760	*LRP2*	4036	-2.4										2	169127109	169362685
4761	*LRP2BP*	55805	-3.8										4	185363879	185395899
4762	*LRP4*	4038	-6.1										11	46856868	46918642
4763	*LRP5*	4041									-1.5		11	68312609	68449275
4764	*LRP6*	4040	-3.8										12	12116025	12267012
4765	*LRR1*	122769				-2.7							14	49598697	49614672
4766	*LRRC1*	55227	-2.1										6	53794497	53924121
4767	*LRRC2*	79442	-7.7										3	46515423	46580099
4768	*LRRC27*	80313	-2.6										10	132332154	132379918

4769	*LRRC32*	2615	-3.5										11	76657524	76670747
4770	*LRRC36*	55282	-2.4										16	67326798	67385203
4771	*LRRC42*	115353	3.4										1	53946077	53968168
4772	*LRRC47*	57470	-4.1										1	3778558	3796504
4773	*LRRC49*	54839	-2.9										X	75029668.1	75103661.36
4774	*LRRC56*	115399	-5.7										11	537527	554916
4775	*LRRC57*	255252	-7.2										15	42537820	42548802
4776	*LRRC69*	100130742	-3.5										8	91101832	91219236
4777	*LRRC8B*	23507						1.7					1	89524836	89597864
4778	*LRRD1*	401387	-5.4										7	92112159	92179531
4779	*LRRIQ1*	84125	-3.8										12	85036314	85263224
4780	*LRRK1*	79705	-7.8										15	100919215	101078254
4781	*LRRN4CL*	221091	-3.6										11	62686402	62689899
4782	*LRTOMT*	220074	-3.8										11	72080331	72110782
4783	*LSG1*	55341	-3.2										3	194640788	194672477
4784	*LSM10*	84967	3.3										1	36391238	36397892
4785	*LSM14A*	26065						-1.6					19	34172504	34229515
4786	*LSM2*	57819		-2.0									6	31797396	31806984
4787	*LSMEM1*	286006		3.1									7	112480853	112491062
4788	*LTA4H*	4048				-2.1							12	96000828	96043520
4789	*LTBP4*	8425	-2.2										19	40592883	40629818
4790	*LTBR*	4055										-2.3	12	6375045	6391571
4791	*LUC7L*	55692		-1.5									16	188969	229463
4792	*LUM*	4060			-4.3								12	91102629	91111831
4793	*LURAP1L*	286343	-3.0										9	12775021	12822131
4794	*LUZP2*	338645	-4.6										11	24496970	25082631
4795	*LUZP4*	51213			5.0								X	115289727	115307556
4796	*LUZP6*	767558		1.8									7	135927274	135927450
4797	*LXN*	56925			-4.5								3	158645822	158672693
4798	*LY6D*	8581	-5.0										8	142784880	142786592
4799	*LY6G6F*	259215		3.1									6	31706885	31710595
4800	*LY6K*	54742			-2.7								5.082011	75022129.5	75096086.77
4801	*LY86*	9450		-1.6									6	6588108	6654983
4802	*LYPD3*	27076								-3.8			19	43460787	43465660
4803	*LYPLAL1*	127018	-4.9										1	219173844	219212865
4804	*LYRM1*	57149									-1.8		16	20899868	20925006
4805	*LYRM4*	57128	-4.2										6	5102593	5260939
4806	*LYRM7*	90624	-4.4										5	131170810	131205426
4807	*M6PR*	4074	-4.3										12	8940363	8949955
4808	*MACC1*	346389	-6.0										7	20134655	20217404
4809	*MACF1*	23499	-2.7										1	39081316	39487177
4810	*MAEA*	10296	4.2										4	1289851	1340147
4811	*MAEL*	84944	-3.1										1	166975582	167022214
4812	*MAF1*	84232	6.0										8	144104499	144107611
4813	*MAFB*	9935							1.6				20	40685848	40689240
4814	*MAFG*	4097					1.5						17	81918270	81927714
4815	*MAGEA1*	4100			-3.4								X	153179285	153183880
4816	*MAGEA10-MAGEA5*	100533997			5.3								X	152114049	152138578
4817	*MAGEB1*	4112			5.1								X	30243730	30252038
4818	*MAGEB10*	139422	-6.1										X	27807990	27823014
4819	*MAGEB2*	4113			4.9								X	30215560	30220089
4820	*MAGEB4*	4115			5.1								X	30242052	30244193
4821	*MAGEC2*	51438			4.9								X	142202345	142205290
4822	*MAGED2*	10916				4.8							X	54807599	54816012
4823	*MAGEF1*	64110	-5.7										3	184710367	184712002
4824	*MAGIX*	79917	-1.7										X	49162564	49168483
4825	*MAGOH*	4116	-3.3										1	53226892	53238610
4826	*MALL*	7851	-5.2										2	110083870	110116566
4827	*MAN1A1*	4121					-1.8						6	119177209	119349761

4828	*MAN1A2*	10905	3.4										1	117367449	117528872
4829	*MAN2C1*	4123				-3.3							15	75355207	75368630
4830	*MANBAL*	63905	-4.0										20	37289638	37317260
4831	*MANSC1*	54682	-3.4										6.074074	75020416.2	75094365.28
4832	*MAOA*	4128	-3.0										X	43654907	43746824
4833	*MAP1LC3B*	81631		1.8									16	87383995	87404779
4834	*MAP1LC3C*	440738	-2.1										1	241995490	241999073
4835	*MAP1S*	55201	2.4										19	17719242	17734516
4836	*MAP2K2*	5605				-3.0							19	4090321	4124129
4837	*MAP2K4*	6416							1.7				17	12020824	12143830
4838	*MAP3K1*	4214		-1.8									5	56815574	56896152
4839	*MAP3K15*	389840	-3.8										X	19360056	19515261
4840	*MAP3K19*	80122	-1.9										2	134964491	135047468
4841	*MAP3K2*	10746								-1.8			2	127298730	127388465
4842	*MAP3K6*	9064	-5.4										1	27355184	27366892
4843	*MAP7D3*	79649	-6.9										X	136213220	136256482
4844	*MAPK10*	5602	-5.4										4	86015123	86594131
4845	*MAPK13*	5603	2.8										6	36127809	36144524
4846	*MAPK14*	1432									-2.4		6	36027677	36111236
4847	*MAPK1IP1L*	93487		1.7									14	55051631	55070192
4848	*MAPKAPK2*	9261										-1.7	1	206684944	206734283
4849	*MAPRE2*	10982		1.9									18	34976928	35143470
4850	*MARCKS*	4082										-1.9	6	113857362	113863471
4851	*MARCO*	8685					2.4						2	118942166	118994660
4852	*MARK2*	2011	-2.5										11	63838928	63911019
4853	*MARVELD2*	153562	-3.5										5	69415112	69444330
4854	*MARVELD3*	91862	-5.7										16	71626161	71642114
4855	*MASP2*	10747	-2.3										1	11026523	11047233
4856	*MB*	4151	-3.0										22	35606764	35637951
4857	*MBD2*	8932									-1.7		18	54151601	54224788
4858	*MBD4*	8930	3.0										3	129430944	129440179
4859	*MBNL1*	4154	-3.5										3	152243828	152465780
4860	*MBOAT1*	154141			3.2								6	20102145	20212399
4861	*MBTPS1*	8720				-3.8							16	84053761	84116906
4862	*MCAM*	4162	-2.3										11	119308529	119321521
4863	*MCC*	4163	-2.8										5	113022099	113488830
4864	*MCCC1*	56922			-3.6								3	183015218	183116075
4865	*MCF2L*	23263			-1.6								13	112894378	113099739
4866	*MCF2L2*	23101	-6.9										3	183178043	183428778
4867	*MCHR2*	84539	-1.9										6	99919910	99994247
4868	*MCM10*	55388	-1.8										10	13161554	13211104
4869	*MCM5*	4174		-1.6									22	35400063	35425430
4870	*MCM6*	4175				-4.7							2	135839626	135876426
4871	*MCMBP*	79892	-3.4										10	119829404	119892556
4872	*MDFI*	4188	-3.1										6	41636882	41654246
4873	*MDH2*	4191	2.7										7	76048051	76067508
4874	*MDK*	4192	-3.8										11	46380756	46383837
4875	*MDP1*	145553			1.6								14	24213937	24216070
4876	*MDS2*	259283	-5.9										1	23581495	23640568
4877	*ME1*	4199	-2.1										6	83210389	83431071
4878	*MEAF6*	64769	3.6										1	37492575	37514774
4879	*MED11*	400569	-3.6										17	4731428	4733610
4880	*MED15*	51586					1.9						22	20495913	20587632
4881	*MED18*	54797	-5.2										3.296296	75025213.5	75099185.47
4882	*MED20*	9477	-5.3										6	41905354	41921139
4883	*MED21*	9412	2.8										12	27022546	27066343
4884	*MED22*	6837	2.8										9	133338323	133348131
4885	*MED24*	9862	3.1										17	40019097	40061215
4886	*MED25*	81857					1.5						19	49818279	49838816

4887	*MED26*	9441	-2.6										19	16574907	16629062
4888	*MED27*	9442			3.0								9	131860107	132079908
4889	*MED29*	55588	-3.8										19	39391303	39400637
4890	*MED6*	10001	-5.0										14	70581257	70600690
4891	*MED7*	9443	-2.5										5	157137412	157159019
4892	*MEF2B*	100271849	-1.9										19	19145568	19170289
4893	*MEF2BNB-MEF2B*	4207	-2.2										19	19145567	19192158
4894	*MEFV*	4210	-6.3										16	3242028	3256627
4895	*MEGF6*	1953	-2.7										1	3489920	3611495
4896	*MEIS3*	56917	-4.9										19	47403124	47419523
4897	*MELK*	9833	-5.1										9	36572862	36677683
4898	*MEMO1*	51072	-4.8										2	31865060	32011230
4899	*MERTK*	10461			-2.8								2	111898479	112029561
4900	*MESP1*	55897	-2.5										15	89748661	89751310
4901	*MESP2*	145873	-2.2										15	89760591	89778754
4902	*METAP1*	23173	-2.9										4	98995620	99062813
4903	*METRN*	79006	-2.2										16	715115	719655
4904	*METTL16*	79066			-2.3								17	2405562	2511891
4905	*METTL20*	254013	-5.7										12	31647160	31673114
4906	*METTL23*	124512			-1.9								17	76726830	76733936
4907	*METTL2A*	339175	-5.0										17	62423867	62450822
4908	*METTL6*	131965	-6.4										3	15381275	15440566
4909	*METTL7A*	25840	-4.4										12	50923472	50932517
4910	*METTL7B*	196410			-1.6								12	55681546	55684611
4911	*METTL8*	79828	-3.8										2	171317405	171434802
4912	*MEX3C*	51320	-2.7										18	51174550	51218304
4913	*MFAP2*	4237				-6.4							1	16974502	16980835
4914	*MFAP3*	4238						1.5					5	154038906	154220478
4915	*MFAP4*	4239	-9.2										17	19383442	19387240
4916	*MFGE8*	4240	-5.7										15	88898683	88913411
4917	*MFN2*	9927	-4.1										1	11980181	12013514
4918	*MFSD2A*	84879	-3.7										1	39955112	39969968
4919	*MFSD3*	113655	2.5										8	144509074	144511213
4920	*MFSD4*	148808	-6.0										1	205568885	205602918
4921	*MFSD5*	84975	2.8										12	53251251	53254405
4922	*MGAT3*	4248		-2.9									22	39457344	39492194
4923	*MGAT4C*	25834	-2.1										12	85955666	86838904
4924	*MGMT*	4255			-1.5								10	129467184	129768007
4925	*MGP*	4256	-7.0										12	14881181	14885926
4926	*MIA*	8190				2.8							19	40771648	40777490
4927	*MICAL2*	9645							-1.9				11	12094008	12263789
4928	*MICAL3*	57553	-6.1										22	17787649	18024559
4929	*MICALL1*	85377	-2.9										22	37905657	37942822
4930	*MICB*	4277						-1.7					6	31494881	31511124
4931	*MIEF1*	54471	-3.0										11.63259	75006709.6	75080593.31
4932	*MIER2*	54531	-4.7										12.44297	75011506.9	75085413.5
4933	*MILR1*	284021	-4.7										17	64449037	64468643
4934	*MINA*	84864	3.0										3	97941818	97972457
4935	*MINOS1*	440574					-1.6						1	19596977	19629821
4936	*MIOS*	54468	-3.7										11.45893	75005681.6	75079560.41
4937	*MIPEP*	4285	2.7										13	23730189	23889419
4938	*MIPOL1*	145282	-3.2										14	37197913	37552361
4939	*MISP*	126353	-3.9										19	751126	764318
4940	*MITD1*	129531			-3.6								2	99161427	99181058
4941	*MKI67*	4288			2.7								10	128096659	128126385
4942	*MKKS*	8195	5.1										20	10401009	10434222
4943	*MKNK1*	8569									-1.6		1	46557408	46616843
4944	*MKRN1*	23608					1.7						7	140453040	140479569
4945	*MLEC*	9761	7.3										12	120686869	120701864

4946	*MLF1*	4291			-2.5								3	158571163	158607252
4947	*MLIP*	90523			-2.7								6	53929982	54266280
4948	*MLLT11*	10962			-2.2								1	151057758	151068497
4949	*MLX*	6945	2.8										17	42567068	42573239
4950	*MLXIP*	22877	3.8										12	122078722	122147347
4951	*MLXIPL*	51085	-5.6										7	73593194	73624543
4952	*MLYCD*	23417	-4.5										16	83899126	83927026
4953	*MMAB*	326625	-2.9										12	109553737	109573874
4954	*MMD2*	221938	-2.5										7	4905989	4959213
4955	*MMP16*	4325			3.3								8	88032009	88328025
4956	*MMP2*	4313	-4.1										16	55389700	55506691
4957	*MMP28*	79148	-5.3										17	35756249	35795707
4958	*MMS22L*	253714	-4.9										6	97142161	97283217
4959	*MOB3C*	148932	-2.6										1	46607715	46616891
4960	*MOBP*	4336		-2.6									3	39467198	39529479
4961	*MOCS3*	27304	-4.8										20	50958826	50963931
4962	*MOG*	4340	-3.8										6	29656981	29672372
4963	*MOGAT3*	346606	-2.1										7	101195007	101201021
4964	*MOGS*	7841		-1.8									2	74461057	74465410
4965	*MON1A*	84315	2.6										3	49908862	49930173
4966	*MORC2*	22880							-1.6				22	30925130	30968298
4967	*MORC3*	23515					-1.7						21	36320189	36386148
4968	*MORC4*	79710	-4.9										X	106813871	107000244
4969	*MORN2*	729967	3.2										2	38875962	38929072
4970	*MORN4*	118812	-5.5										10	97614553	97633500
4971	*MOSPD1*	56180	-3.1										X	134887626	134915267
4972	*MOV10L1*	54456	-2.0										11.34317	75004996.3	75078871.82
4973	*MPDU1*	9526										-1.7	17	7583529	7592789
4974	*MPHOSPH6*	10200	-3.7										16	82147798	82170226
4975	*MPND*	84954	-3.0										19	4343527	4360086
4976	*MPO*	4353	-7.5										17	58269856	58280935
4977	*MPP4*	58538	-5.8										2	201644870	201698694
4978	*MPP5*	64398	-6.0										14	67241109	67335819
4979	*MPP7*	143098	-4.4										10	28050993	28334486
4980	*MPPE1*	65258							1.6				18	11882622	11909223
4981	*MPPED2*	744	-2.3										11	30384493	30586872
4982	*MPV17L*	255027	-1.8										16	15395754	15413268
4983	*MPV17L2*	84769			-2.9								19	18193182	18196948
4984	*MR1*	3140	-3.2										1	181033425	181061938
4985	*MRAS*	22808	-3.6										3	138347648	138405534
4986	*MRC2*	9902	-1.9										17	62627401	62693597
4987	*MREG*	55686	-6.4										2	215942805	216034096
4988	*MRGBP*	55257	-7.3										20	62796453	62801738
4989	*MRGPRF*	116535	-1.6										11	69004395	69013409
4990	*MRI1*	84245	-4.1										19	13764532	13774282
4991	*MRO*	83876	-3.2										18	50795120	50825402
4992	*MROH1*	727957	-3.6										8	144148016	144261940
4993	*MROH8*	140699	-1.7										20	37101226	37179588
4994	*MRPL12*	6182	3.7										17	81703357	81707526
4995	*MRPL15*	29088	3.4										8	54135210	54147901
4996	*MRPL17*	63875	3.2										11	6680782	6683401
4997	*MRPL21*	219927			-2.4								11	68891276	68903835
4998	*MRPL22*	29093	4.6										5	154941070	154969411
4999	*MRPL35*	51318	2.8										2	86199355	86213794
5000	*MRPL36*	64979	3.4										5	1798386	1801366
5001	*MRPL37*	51253	3.3										1	54184041	54225464
5002	*MRPL38*	64978	-3.6										17	75898643	75905413
5003	*MRPL40*	64976	-5.3										22	19431902	19436075
5004	*MRPL41*	64975			1.6								9	137551199	137552555

5005	*MRPL49*	740	-4.1										11	65122183	65127371
5006	*MRPL50*	54534	-7.8										12.55874	75012192.2	75086102.1
5007	*MRPL57*	78988	-3.7										13	21176645	21179084
5008	*MRPS15*	64960							-1.5				1	36455718	36464437
5009	*MRPS21*	54460		-2.1									11.40105	75005338.9	75079216.12
5010	*MRPS26*	64949		-1.7									20	3045945	3048254
5011	*MRPS27*	23107	3.4										5	72219409	72320646
5012	*MRPS30*	10884	3.7										5	44808925	44820428
5013	*MRPS34*	65993	-4.5										16	1771890	1773155
5014	*MRPS35*	60488							-1.6				12	27710773	27756295
5015	*MRRF*	92399	4.5										9	122264603	122331343
5016	*MRTO4*	51154						1.9					1	19251539	19260128
5017	*MRVI1*	10335	-4.7										11	10573091	10693988
5018	*MS4A10*	341116	-3.6										11	60785348	60801305
5019	*MS4A4A*	51338	-2.8										11	60280541	60308972
5020	*MS4A6A*	64231							1.9				11	60172014	60184666
5021	*MS4A7*	58475		-1.9									11	60378482	60395951
5022	*MS4A8*	83661	-4.0										11	60699574	60715811
5023	*MSANTD3*	91283						2.2					9	100427156	100451711
5024	*MSC*	9242			-2.8								8	71841549	71844468
5025	*MSH5*	4439	-6.0										6	31739948	31762834
5026	*MSI2*	124540	-3.0										17	57255851	57684685
5027	*MSL1*	339287	5.4										17	40122298	40136916
5028	*MSLN*	10232	-2.2										16	643262	768865
5029	*MSMB*	4477	-10.7										10	46033307	46046269
5030	*MSRB1*	51734							1.7				16	1938210	1943326
5031	*MSRB3*	253827	-6.3										12	65278643	65488244
5032	*MSS51*	118490	-3.0										10	73423579	73433561
5033	*MST1*	4485										-2.2	3	49683947	49689501
5034	*MST1L*	11223									-1.6		1	16754634	16770237
5035	*MSTO1*	55154		-1.6									1	155610205	155614967
5036	*MT1F*	4494					-1.6						16	56657694	56660698
5037	*MT2A*	4502				-3.2							16	56608199	56609497
5038	*MTBP*	27085	-5.1										8	120445400	120542133
5039	*MTCH2*	23788			2.2								11	47617315	47642623
5040	*MTDH*	92140							-1.6				8	97644179	97728770
5041	*MTERF2*	80298	4.3										12	106977291	106987166
5042	*MTFMT*	123263	-5.7										15	65001512	65029639
5043	*MTG1*	92170				-2.4							10	133394094	133421307
5044	*MTHFD1L*	25902	-3.3										6	150865549	151101887
5045	*MTHFD2L*	441024		-1.8									4	74114174	74303099
5046	*MTMR12*	54545	-3.7										12.90604	75014248.2	75088167.89
5047	*MTMR6*	9107			-3.5								13	25246201	25288009
5048	*MTPN*	136319									-2.1		7	135926761	135977353
5049	*MTRF1*	9617	-4.3										13	41216369	41263577
5050	*MTRNR2L8*	100463486				-2.0							11	10507887	10509189
5051	*MTX3*	345778	-4.6										5	79976731	79991262
5052	*MUC1*	4582	-6.2										1	155185824	155192916
5053	*MUC16*	94025	-6.2										19	8848844	8981342
5054	*MUC20*	200958	-5.4										3	195720882	195741123
5055	*MUC21*	394263	-2.6										6	30983718	30989903
5056	*MUC4*	4585	-5.8										3	195746765	195812277
5057	*MVB12A*	93343	-4.1										19	17405722	17433724
5058	*MXD3*	83463			2.1								5	177301461	177312757
5059	*MYBBP1A*	10514	-2.7										17	4538897	4555631
5060	*MYBPC1*	4604	-5.8										12	101568353	101686018
5061	*MYBPC2*	4606	-3.5										19	50432903	50466321
5062	*MYBPC3*	4607	-2.1										11	47331397	47352702
5063	*MYCL*	4610								-2.0			1	39895426	39902256

5064	*MYCN*	4613										1.6	2	15940564	15947007
5065	*MYD88*	4615									-3.0		3	38138478	38143022
5066	*MYH11*	4629	-10.3										16	15703135	15857033
5067	*MYH14*	79784	-6.5										19	50188186	50310545
5068	*MYH7B*	57644	-6.8										20	34975403	35002437
5069	*MYH9*	4627					1.8						22	36281281	36388018
5070	*MYL5*	4636		2.9									4	673580	682033
5071	*MYL6B*	140465						2.9					12	56152256	56159647
5072	*MYLK3*	91807	-6.2										16	46703369	46790407
5073	*MYO15A*	51168	-3.4										17	18108706	18179802
5074	*MYO16*	23026	-6.0										13	108596152	109208007
5075	*MYO19*	80179						1.7					17	36495633	36543435
5076	*MYO1D*	4642			-1.8								17	32492522	32877177
5077	*MYO3B*	140469	-5.6										2	170178145	170655171
5078	*MYOC*	4653	-2.2										1	171635417	171652683
5079	*MYOCD*	93649	-2.7										17	12665890	12768949
5080	*MYOM1*	8736	-1.7										18	3066807	3220108
5081	*MYZAP*	100820829		3.8									15	57591941	57685364
5082	*N4BP2*	55728	-3.6										4	40056826	40158252
5083	*NAA10*	8260				3.2							X	153929242	153935223
5084	*NAA15*	80155						1.5					4	139301455	139420033
5085	*NAA20*	51126	-4.6										20	20017116	20033655
5086	*NAA38*	84316	-4.9										17	7856685	7885238
5087	*NAA50*	80218	3.9										3	113716460	113746300
5088	*NAALADL2*	254827	2.4										3	174438573	175810552
5089	*NAB1*	4664	-5.0										2	190646746	190692766
5090	*NAGA*	4668			3.2								22	42058354	42070842
5091	*NAGK*	55577	2.9										2	71064344	71079805
5092	*NAGLU*	4669	-4.7										17	42536172	42544449
5093	*NALCN*	259232	-4.3										13	101053776	101416492
5094	*NAMPT*	10135	5.7										7	106248285	106286326
5095	*NANOG*	79923	-3.9										12	7787794	7799141
5096	*NANOGNB*	360030	-1.7										12	7765216	7774121
5097	*NANP*	140838			-2.4								20	25612935	25624175
5098	*NAPB*	63908	-5.1										20	23374519	23421519
5099	*NAPSA*	9476		-2.2									19	50358477	50365830
5100	*NARS2*	79731			-4.0								11	78435961	78574874
5101	*NASP*	4678	-3.3										1	45583846	45618904
5102	*NAT10*	55226			-2.0								11	34105602	34147670
5103	*NAT9*	26151	2.7										17	74770547	74776367
5104	*NATD1*	256302	-2.9										17	21238870	21253410
5105	*NAV1*	89796	-3.6										1	201622885	201826969
5106	*NBEAL1*	65065		-1.5									2	203014879	203226378
5107	*NBL1*	4681										-2.3	1	19640554	19658456
5108	*NBPF1*	55672	-3.2										1	16562319	16613605
5109	*NBR1*	4077	-2.9										17	43170481	43211689
5110	*NCAM1*	4684	-2.9										11	112961247	113278436
5111	*NCAM2*	4685	2.8										21	20998315	21543329
5112	*NCAN*	1463	-2.6										19	19211973	19252233
5113	*NCAPD3*	23310	-2.8										11	134150119	134225454
5114	*NCBP1*	4686	-5.6										9	97633626	97673748
5115	*NCCRP1*	342897	-6.6										19	39196961	39201884
5116	*NCEH1*	57552							-1.9				3	172630249	172711218
5117	*NCK1*	4690							-1.8				3	136862208	136949823
5118	*NCK2*	8440		2.5									2	105744897	105894274
5119	*NCKAP5*	344148	-2.8										2	132671799	133568463
5120	*NCL*	4691								-1.6			2	231453531	231483641
5121	*NCMAP*	400746	-1.9										1	24556111	24609328
5122	*NCOA4*	8031									-2.7		10	46005088	46030714

5123	*NCOA7*	135112	-5.5										6	125781161	125932030
5124	*NDE1*	54820					1.9						1.907407	75027612.1	75101595.56
5125	*NDRG1*	10397							-2.1				8	133237171	133302022
5126	*NDRG2*	57447	-4.9										14	21016763	21070872
5127	*NDRG4*	65009								-1.6			16	58462846	58513628
5128	*NDST3*	9348	-3.4										4	118033618	118258648
5129	*NDST4*	64579	-5.2										4	114827763	115113876
5130	*NDUFA10*	4705		-1.7									2	239892450	240025402
5131	*NDUFA2*	4695	-3.0										5	140638740	140647785
5132	*NDUFA4L2*	56901			-3.7								12	57234903	57240715
5133	*NDUFA5*	4698	-4.6										7	123536997	123558255
5134	*NDUFAF2*	91942	3.4										5	60945129	61153037
5135	*NDUFAF5*	79133				-4.1							20	13784950	13821582
5136	*NDUFAF7*	55471	-3.0										2	37231631	37253403
5137	*NDUFB10*	4716				3.1							16	1959508	1961975
5138	*NDUFB7*	4713							-1.6				19	14566078	14572062
5139	*NECAB2*	54550	-1.5										13.02181	75014933.5	75088856.49
5140	*NECAP1*	25977			2.4								12	8082211	8097771
5141	*NEDD8*	4738			1.6								14	24216852	24232454
5142	*NEDD8-MDP1*	100528064			1.6								14	24213937	24232370
5143	*NEK1*	4750	-2.6										4	169393275	169612629
5144	*NEK2*	4751	-4.4										1	211658657	211675630
5145	*NEK4*	6787									-2.0		3	52708449	52770949
5146	*NEK8*	284086	-5.2										17	28725897	28743455
5147	*NET1*	10276						2.5					10	5412551	5458463
5148	*NEU1*	4758			-4.2								6	31857659	31862906
5149	*NEUROD2*	4761	-1.9										17	39603536	39609777
5150	*NFATC2IP*	84901					1.6						16	28950807	28967097
5151	*NFE2L2*	4780	-5.4										2	177227595	177392697
5152	*NFIA*	4774	-4.0										1	60865259	61462793
5153	*NFIB*	4781								-2.4			9	14081843	14398983
5154	*NFIC*	4782	4.0										19	3359563	3469217
5155	*NFIL3*	4783	4.7										9	91409045	91423862
5156	*NFIX*	4784								-1.5			19	12995608	13098796
5157	*NFKB1*	4790			2.7								4	102501329	102617302
5158	*NFKBIA*	4792	3.6										14	35401511	35404749
5159	*NFKBID*	84807	-1.8										19	35887653	35902303
5160	*NFKBIE*	4794	2.5										6	44258166	44265788
5161	*NFS1*	9054	-6.6										20	35668055	35699359
5162	*NHLRC2*	374354						2.0					10	113854661	113917194
5163	*NICN1*	84276	-3.9										3	49422946	49429326
5164	*NINL*	22981	-2.3										20	25452705	25585517
5165	*NIP7*	51388	-4.3										16	69339430	69343111
5166	*NIPA1*	123606						1.7					15	22773063	22829791
5167	*NIPBL*	25836			-2.2								5	36876759	37066413
5168	*NIT2*	56954	-4.6										3	100334701	100361635
5169	*NKPD1*	284353	-3.4										19	45149750	45160150
5170	*NKRF*	55922										1.5	X	119588337	119605895
5171	*NKX3-1*	4824			-1.8								8	23678693	23682927
5172	*NLN*	57486	-7.0										5	65722196	65871725
5173	*NLRC3*	197358	-4.9										16	3539033	3577400
5174	*NLRP11*	204801	-1.8										19	55785397	55836800
5175	*NLRP5*	126206	-2.2										19	55999726	56061813
5176	*NLRP8*	126205	-2.4										19	55947832	55988629
5177	*NME2*	4831		-1.8									17	51165435	51171747
5178	*NME3*	4832			-2.1								16	1770286	1771730
5179	*NME7*	29922	-3.6										1	169132531	169367967
5180	*NME9*	347736	-4.7										3	138261437	138329886
5181	*NMI*	9111									-2.9		2	151270465	151290057

5182	*NMNAT1*	64802	-6.2										1	9943428	9985501
5183	*NMRAL1*	57407	-4.3										16	4461680	4495763
5184	*NMRK2*	27231	-9.5										19	3933103	3942416
5185	*NMT2*	9397	-4.8										10	15102584	15168693
5186	*NOB1*	28987	2.8										16	69741867	69754940
5187	*NOCT*	25819	-5.8										4	139015789	139045939
5188	*NOL12*	79159	-4.5										22	37681673	37693478
5189	*NOL6*	65083	3.1										9	33461441	33473930
5190	*NOL9*	79707	-3.9										1	6521347	6554535
5191	*NOM1*	64434	-4.0										7	156949723	156973182
5192	*NOMO1*	23420	2.7										16	14833681	14896160
5193	*NONO*	4841	3.3										X	71283192	71301168
5194	*NOP14*	8602									-1.7		4	2937933	2963385
5195	*NOP58*	51602							-1.7				2	202265716	202303666
5196	*NOS1*	4842	-2.3										12	117208142	117452170
5197	*NOS2*	4843	-2.0										17	27756766	27800499
5198	*NOSTRIN*	115677	-1.5										2	168786539	168865514
5199	*NOTCH1*	4851	2.7										9	136494444	136545862
5200	*NOTCH2*	4853	-5.0										1	119911553	120069626
5201	*NOTCH3*	4854	-5.4										19	15159038	15200981
5202	*NOTCH4*	4855	-4.9										6	32194843	32224067
5203	*NOV*	4856			4.5								8	119416306	119424353
5204	*NOX4*	50507	-3.4										11	89324356	89498187
5205	*NPAP1*	23742	-2.5										15	24675868	24683393
5206	*NPAT*	4863			-2.5								11	108157215	108222642
5207	*NPC1L1*	29881	-2.6										7	44512535	44541315
5208	*NPC2*	10577				-1.8							14	74476192	74494177
5209	*NPDC1*	56654				-2.1							9	137039470	137046203
5210	*NPHS1*	4868	-2.2										19	35825964	35869287
5211	*NPL*	80896							1.5				1	182789293	182830384
5212	*NPNT*	255743	-5.1										4	105894775	106004027
5213	*NPTN*	27020		1.8									15	73560014	73634134
5214	*NPTXR*	23467	-4.6										22	38818452	38843982
5215	*NPY*	4852	-10.6										7	24284163	24291865
5216	*NPY4R*	5540	-2.1										10	46461099	46465881
5217	*NQO1*	1728	-3.9										16	69706996	69726951
5218	*NR2F2*	7026	3.2										15	96325938	96340263
5219	*NR2F6*	2063			-2.5								19	17231883	17245940
5220	*NR3C1*	2908					-1.5						5	143277931	143435512
5221	*NR4A2*	4929			-1.9								2	156324432	156342348
5222	*NRBF2*	29982	2.7										10	63133247	63155031
5223	*NRCAM*	4897	-3.4										7	108147623	108456717
5224	*NRDE2*	55051	-2.3										14	90267856	90332137
5225	*NRG1*	3084	-2.0										8	31639386	32767959
5226	*NRIP2*	83714	-6.9										12	2825348	2835544
5227	*NRIP3*	56675	-4.8										11	8980576	9004049
5228	*NRK*	203447	-9.2										X	105822543	105958610
5229	*NRSN1*	140767	-3.1										6	24126122	24154900
5230	*NSA2*	10412		-1.6									5	74766991	74780113
5231	*NSDHL*	50814	-3.0										X	152830967	152869729
5232	*NSL1*	25936	-4.0										1	212726153	212791782
5233	*NSMAF*	8439	-2.2										8	58583504	58659844
5234	*NSMCE1*	197370										-1.7	16	27224991	27268794
5235	*NSUN6*	221078			3.5								10	18545561	18659285
5236	*NT5C*	30833				-3.5							17	75130225	75131795
5237	*NT5DC2*	64943				-5.0							3	52524385	52535054
5238	*NT5E*	4907				-2.4							6	85449584	85495791
5239	*NTHL1*	4913				-5.8							16	2039815	2047866
5240	*NTN5*	126147	-1.8										19	48661407	48673081

5241	*NTRK1*	4914	-2.3										1	156815640	156881850
5242	*NTRK3*	4916		-2.9									15	87859751	88256768
5243	*NUAK2*	81788	-4.8										1	205302059	205321791
5244	*NUCB2*	4925	-4.3										11	17208153	17349974
5245	*NUDCD2*	134492			2.6								5	163446526	163460140
5246	*NUDCD3*	23386	-4.2										7	44379121	44490880
5247	*NUDT1*	4521			4.2								7	2242222	2251146
5248	*NUDT14*	256281	-2.6										14	105172938	105181323
5249	*NUDT15*	55270	-3.4										13	48037567	48047222
5250	*NUDT19*	390916				1.9							19	32691961	32713796
5251	*NUDT21*	11051					-1.8						16	56429133	56452199
5252	*NUDT22*	84304			-2.9								11	64225941	64230686
5253	*NUDT5*	11164	3.9										10	12165325	12196144
5254	*NUDT7*	283927	-3.2										16	77722492	77742260
5255	*NUFIP1*	26747	-1.5										13	44939249	44989483
5256	*NUFIP2*	57532	3.8										17	29255836	29294118
5257	*NUGGC*	389643	-5.4										8	28021964	28083871
5258	*NUMB*	8650										2.5	14	73275107	73463642
5259	*NUP133*	55746	-3.7										1	229440260	229508341
5260	*NUP153*	9972	-3.3										6	17615035	17706834
5261	*NUP210*	23225	3.4										3	13316235	13420309
5262	*NUP210L*	91181	-2.7										1	153992685	154155116
5263	*NUP35*	129401				5.0							2	183117513	183161680
5264	*NUP62*	23636		-1.8									19	49906825	49929763
5265	*NUP93*	9688	5.1										16	56730105	56850286
5266	*NUP98*	4928			1.8								11	3671083	3797792
5267	*NUPR1L*	389493	-2.0										7	56114681	56116400
5268	*NUTF2*	10204		1.7									16	67846732	67872567
5269	*NWD1*	284434	-5.0										19	16719976	16817963
5270	*NXF3*	56000	-3.4										X	103075810	103093125
5271	*NXN*	64359	-2.7										17	799313	979770
5272	*NXPH4*	11247		-3.5									12	57216795	57226449
5273	*NXT1*	29107			-2.9								20	23350736	23354777
5274	*NYAP2*	57624	-5.3										2	225400648	225654018
5275	*OAF*	220323				-4.9							11	120210767	120230332
5276	*OAS3*	4940	-3.2										12	112938352	112973249
5277	*OAZ1*	4946									-1.9		19	2269509	2273490
5278	*OAZ2*	4947							1.6				15	64687573	64703281
5279	*OBSL1*	23363								-3.0			2	219550729	219571859
5280	*OCA2*	4948			-2.8								15	27754875	28099358
5281	*ODC1*	4953			-2.5								2	10439968	10448504
5282	*ODF3B*	440836					1.6						22	50529710	50532580
5283	*OGFOD3*	79701	-6.7										17	82389223	82418637
5284	*OGFRL1*	79627							1.9				6	71288803	71308950
5285	*OGT*	8473	4.4										X	71533083	71575897
5286	*OLA1*	29789							-1.6				2	174072447	174248698
5287	*OLAH*	55301	-6.2										10	15032227	15073852
5288	*OLFML3*	56944	-4.1										1	113979391	114035572
5289	*OMA1*	115209			-2.6								1	58415384	58546802
5290	*ONECUT2*	9480	-8.1										18	57435685	57491297
5291	*OOEP*	441161	-2.3										6	73368555	73395133
5292	*OPA1*	4976			-2.3								3	193593144	193697823
5293	*OPA3*	80207	-6.2										19	45527427	45602212
5294	*OPTN*	10133	-2.6										10	13099449	13138308
5295	*OR11A1*	26531		-1.6									6	29425504	29457071
5296	*OR2C3*	81472			-1.9								1	247530132	247533839
5297	*OR2M3*	127062		-2.0									1	248203068	248204006
5298	*OR4Q3*	441669		-2.0									14	19747428	19748369
5299	*OR4S1*	256148		-2.2									11	48306223	48307152

5300	*OR51E1*	143503	-1.6										11	4643420	4655488
5301	*OR52B6*	340980		-1.9									11	5580877	5581884
5302	*OR52M1*	119772		-1.9									11	4545191	4546144
5303	*OR5P3*	120066		-1.7									11	7825037	7825972
5304	*OR6M1*	390261		-2.9									11	123805335	123806387
5305	*OR7D2*	162998	-2.7										19	9185594	9188817
5306	*OR8S1*	341568	-1.6										12	48507354	48529897
5307	*OR9Q2*	219957		-3.1									11	58190434	58191518
5308	*ORC3*	23595	-4.9										6	87590067	87667453
5309	*ORC5*	5001			2.1								7	104126341	104208047
5310	*ORC6*	23594						1.8					16	46689643	46698394
5311	*ORM1*	5004	-7.8										9	114323056	114326475
5312	*ORMDL1*	94101	-3.1										2	189770323	189784371
5313	*OSBP2*	23762		2.4									22	30693782	30907824
5314	*OSBPL11*	114885									-2.0		3	125528858	125595090
5315	*OSBPL1A*	114876	-3.6										18	24162044	24397880
5316	*OSBPL2*	9885	-4.2										20	62231922	62296213
5317	*OSBPL6*	114880	-2.0										2	178194481	178402891
5318	*OSER1*	51526	3.1										20	44196496	44210791
5319	*OSGIN1*	29948			-2.7								16	83948282	83966332
5320	*OSMR*	9180	-3.9										5	38845858	38945596
5321	*OSR2*	116039	-3.9										8	98944403	98952104
5322	*OST4*	100128731						1.6					2	27070472	27071773
5323	*OSTF1*	26578									-1.9		9	75088543	75147265
5324	*OSTM1*	28962	-3.2										6	108041409	108165854
5325	*OTOP1*	133060		-2.2									4	4188803	4226889
5326	*OTUB1*	55611				-2.4							11	63985853	64001811
5327	*OTUD5*	55593	-5.2										X	48922028	48958386
5328	*OTUD6A*	139562	-3.4										X	70062491	70064179
5329	*OTULIN*	90268	-4.1										5	14664664	14699711
5330	*OXNAD1*	92106			-1.8								3	16265160	16350299
5331	*OXTR*	5021	-2.9										3	8750408	8769628
5332	*P2RY1*	5028		1.8									3	152834693	152841439
5333	*P2RY2*	5029		-2.0									11	73218298	73236352
5334	*P3H1*	64175	-4.2										1	42746335	42767084
5335	*P3H2*	55214	-3.6										3	189956728	190122437
5336	*PAAF1*	80227	-2.5										11	73876699	73931124
5337	*PABPC1L*	80336									-1.8		20	44910062	44959035
5338	*PABPC3*	5042	4.2										13	25095868	25099254
5339	*PADI1*	29943	-4.5										1	17205126	17246005
5340	*PADI2*	11240	-4.8										1	17066761	17119435
5341	*PADI3*	51702	-2.2										1	17249098	17284233
5342	*PAEP*	5047				5.1							9	135561756	135566955
5343	*PAFAH2*	5051	-2.5										1	25959767	25998157
5344	*PAG1*	55824			-4.0								8	80967810	81112068
5345	*PAGE2*	203569			4.1								X	55089008	55092842
5346	*PAGE2B*	389860			4.4								X	55075063	55078909
5347	*PAGE4*	9506	-2.3										X	49829260	49833973
5348	*PAGE5*	90737			8.4								X	55220355	55224108
5349	*PAIP2*	51247	-2.8										5	139341587	139369720
5350	*PAIP2B*	400961								-1.7			2	71182739	71227083
5351	*PAK6*	56924			2.7								15	40238759	40277487
5352	*PALB2*	79728	-3.2										16	23603160	23641310
5353	*PALM*	5064	-4.7										19	708939	748329
5354	*PALMD*	54873		-1.8									7.018182	75033780.1	75107792.95
5355	*PAM16*	51025								-1.8			16	4331549	4355607
5356	*PAN2*	9924	-2.3										12	56316223	56334053
5357	*PANK1*	53354	-5.0										10	89579497	89645572
5358	*PANK3*	79646					-1.6						5	168548495	168579600

5359	*PAPL*	390928		-1.6									19	39083913	39111493
5360	*PAPLN*	89932	-5.5										14	73237497	73274640
5361	*PAPOLA*	10914				2.1							14	96501433	96567111
5362	*PAPOLG*	64895	-2.9										2	60756230	60802085
5363	*PAPPA*	5069	-1.8										9	116153804	116402322
5364	*PAPSS1*	9061	2.6										4	107590276	107720452
5365	*PAQR8*	85315			-2.3								6	52361421	52407777
5366	*PARD6B*	84612	-5.9										20	50731544	50756795
5367	*PARD6G*	84552	-5.7										18	80157232	80247546
5368	*PARK2*	5071	-4.3										6	161347420	162727771
5369	*PARM1*	25849	-2.2										4	74933095	75050115
5370	*PARP11*	57097	-6.2										12	3791047	3873448
5371	*PARP14*	54625									-2.0		13.48489	75017674.8	75091610.89
5372	*PARP2*	10038			1.7								14	20343582	20357905
5373	*PARP4*	143									-2.0		13	24420926	24512810
5374	*PARP6*	56965			-2.8								15	72241181	72272999
5375	*PARP8*	79668									-2.8		5	50665899	50846522
5376	*PATL1*	219988	-3.7										11	59636716	59668980
5377	*PAWR*	5074	-5.2										12	79574979	79691097
5378	*PAX8*	7849	-1.8										2	113215997	113278950
5379	*PAXIP1*	22976	2.6										7	154943687	155003084
5380	*PBOV1*	59351	-4.9										6	138215986	138218491
5381	*PBRM1*	55193	-4.4										3	52545352	52685917
5382	*PCBD2*	84105	-4.0										5	134904906	135007959
5383	*PCDH1*	5097			1.7								5	141853111	141879246
5384	*PCDH11X*	27328	-5.9										X	91779261	92623230
5385	*PCDH11Y*	83259	-5.4										Y	5000226	5742224
5386	*PCDH18*	54510	-5.3										12.26932	75010478.9	75084380.6
5387	*PCDHB16*	57717			1.8								5	141181399	141186399
5388	*PCF11*	51585	3.2										11	83156988	83187451
5389	*PCGF2*	7703	-3.1										17	38733897	38749817
5390	*PCGF3*	10336	4.2										4	705748	770640
5391	*PCGF6*	84108	-6.0										10	103302796	103351134
5392	*PCID2*	55795		-1.9									13	113177539	113208715
5393	*PCK1*	5105										1.6	20	57561080	57568112
5394	*PCMTD1*	115294	-3.1										8	51817575	51899186
5395	*PCNXL4*	64430	-3.9										14	60091911	60169133
5396	*PCP4*	5121	-9.3										21	39867317	39929397
5397	*PCSK2*	5126									-1.5		20	17226107	17484578
5398	*PCSK5*	5125	-7.6										9	75890644	76362339
5399	*PCYOX1*	51449	-3.2										2	70257386	70281191
5400	*PCYOX1L*	78991	3.0										5	149358007	149369653
5401	*PDCD11*	22984	2.9										10	103396648	103446292
5402	*PDCD2L*	84306	-1.9										19	34404384	34426168
5403	*PDDC1*	347862	-4.6										11	767220	777488
5404	*PDE11A*	50940	-4.5										2	177623252	178108339
5405	*PDE12*	201626	-3.6										3	57556276	57566844
5406	*PDE3A*	5139	-1.9										12	20369245	20684381
5407	*PDE3B*	5140	-5.3										11	14643723	14872044
5408	*PDE4C*	5143	-6.7										19	18207961	18255419
5409	*PDE4D*	5144			-2.2								5	58969038	60522120
5410	*PDE4DIP*	9659			-2.1								1	148808181	149048286
5411	*PDE6A*	5145	-4.7										5	149857955	149944793
5412	*PDE6B*	5158	-3.0										4	625584	670782
5413	*PDE6D*	5147	-2.2										2	231732425	231786272
5414	*PDE7A*	5150	-4.4										8	65717510	65842322
5415	*PDGFRB*	5159	-2.2										5	150113837	150155872
5416	*PDHB*	5162					-1.6						3	58427630	58433857
5417	*PDIA5*	10954	3.2										3	123067062	123225227

5418	*PDK1*	5163	-4.4										2	172555373	172608669
5419	*PDK4*	5166			-4.2								7	95583499	95596491
5420	*PDLIM7*	9260	-4.1										5	177483394	177497606
5421	*PDP2*	57546	-5.4										16	66878589	66895754
5422	*PDPK1*	5170					1.6						16	2537964	2603188
5423	*PDPR*	55066	-3.1										16	70113626	70162537
5424	*PDSS1*	23590					2.0						10	26697659	26746798
5425	*PDSS2*	57107	-3.6										6	107152557	107459564
5426	*PDXP*	57026	-3.2										22	37658727	37666934
5427	*PDZD2*	23037	-6.4										5	31639410	32110931
5428	*PDZD7*	79955	-1.5										10	101007683	101031157
5429	*PEAK1*	79834	-5.1										15	77100656	77420144
5430	*PEF1*	553115	4.9										1	31629862	31644896
5431	*PELO*	53918							-1.7				5	52787940	52804046
5432	*PERP*	64065								-1.9			6	138088505	138107511
5433	*PES1*	23481	-3.5										22	30576625	30607083
5434	*PEX2*	5828	-4.7										8	76980258	77001044
5435	*PEX26*	55670	-5.2										22	18077920	18131138
5436	*PFDN6*	10471						1.9					6	33289302	33298401
5437	*PFKFB1*	5207									-1.6		X	54932961	54998534
5438	*PFKM*	5213						2.0					12	48105139	48146404
5439	*PGAP1*	80055	-4.0										2	196833004	196927796
5440	*PGAP2*	27315			1.8								11	3797724	3826371
5441	*PGAP3*	93210	-3.9										17	39671122	39696797
5442	*PGK1*	5230							-2.2				X	78065188	78129296
5443	*PGLYRP2*	114770	-2.1										19	15468645	15498956
5444	*PGM1*	5236				3.4							1	63593276	63660245
5445	*PGM2L1*	283209	-6.6										11	74330318	74398473
5446	*PGM5*	5239	-2.4										9	68328308	68531061
5447	*PGPEP1*	54858	-3.9										7.209091	75031381.4	75105382.85
5448	*PHACTR1*	221692	-3.9										6	12716805	13290484
5449	*PHACTR4*	65979	-5.3										1	28369582	28500369
5450	*PHAX*	51808						1.7					5	126600268	126627252
5451	*PHB2*	11331	2.9										12	6965327	6970825
5452	*PHF19*	26147	3.0										9	120855652	120894896
5453	*PHF20*	51230	-3.5										20	35771974	35950381
5454	*PHF3*	23469					-1.8						6	63635820	63779336
5455	*PHF5A*	84844			2.1								22	41459717	41468725
5456	*PHF8*	23133	-2.5										X	53936676	54048958
5457	*PHKA1*	5255	-3.3										X	72578814	72714319
5458	*PHKG2*	5261	-3.2										16	30748270	30761176
5459	*PHLDB1*	23187	-3.8										11	118606440	118658038
5460	*PHLDB2*	90102	-4.2										3	111732497	111976517
5461	*PHLDB3*	653583	-4.5										19	43474954	43504935
5462	*PHLPP2*	23035	-2.2										16	71637835	71724701
5463	*PHPT1*	29085		-1.6									9	136848724	136851027
5464	*PHTF1*	10745	-3.5										1	113696831	113759489
5465	*PHTF2*	57157	3.5										7	77798792	77957503
5466	*PHYH*	5264	-2.8										10	13277796	13302412
5467	*PHYHD1*	254295	-4.7										9	128920895	128942041
5468	*PHYHIP*	9796	-2.6										8	22219704	22232341
5469	*PI3*	5266	-5.9										20	45174876	45176544
5470	*PI4K2A*	55361	-4.3										10	97640686	97676434
5471	*PIBF1*	10464	-1.9										13	72782059	73016461
5472	*PICALM*	8301									-2.8		11	85957684	86069882
5473	*PIEZO2*	63895	-2.5										18	10666483	11148762
5474	*PIF1*	80119	-5.5										15	64815632	64825668
5475	*PIFO*	128344	-1.8										1	111346288	111353013
5476	*PIGB*	9488	-2.6										15	55318960	55355648

5477	*PIGG*	54872	2.5										7.045455	75033437.4	75107448.65
5478	*PIGK*	10026						2.4					1	77088990	77219430
5479	*PIGN*	23556	-6.1										18	62043567	62187118
5480	*PIGS*	94005	3.4										17	28553383	28571872
5481	*PIGV*	55650	-4.0										1	26787472	26798398
5482	*PIGX*	54965				-2.7							23.57143	75045430.6	75119499.12
5483	*PIK3C2B*	5287	-4.6										1	204422628	204494724
5484	*PIK3C3*	5289	-4.9										18	41955206	42087830
5485	*PIK3CB*	5291										1.6	3	138652699	138834938
5486	*PIK3CG*	5294							1.9				7	106865278	106907145
5487	*PIK3IP1*	113791									-2.2		22	31281593	31292534
5488	*PIK3R3*	8503			2.5								1	46043661	46176488
5489	*PIKFYVE*	200576	-2.6										2	208266267	208358751
5490	*PIN1*	5300	-3.6										19	9835257	9849682
5491	*PINK1*	65018			-1.6								1	20633455	20651511
5492	*PIP*	5304	-9.1										7	143132077	143139746
5493	*PIP4K2C*	79837	2.7										12	57591174	57603418
5494	*PIP5K1A*	8394			-1.8								1	151197949	151249536
5495	*PIR*	8544	-2.7										X	15384799	15493564
5496	*PITHD1*	57095	3.0										1	23778405	23788232
5497	*PITPNM1*	9600	-6.1										11	67491768	67506263
5498	*PITPNM3*	83394	-2.0										17	6451264	6556494
5499	*PIWIL2*	55124	-5.5										8	22275297	22357563
5500	*PIWIL3*	440822	-5.6										22	24719034	24774720
5501	*PIWIL4*	143689	-3.3										11	94543840	94621421
5502	*PKD1*	5310	-5.1										16	2088710	2135898
5503	*PKD1L3*	342372	-2.6										16	71929538	71999978
5504	*PKHD1L1*	93035	-4.2										8	109362477	109530330
5505	*PKIB*	5570	-2.6										6	122471917	122726373
5506	*PKM*	5315					1.7						15	72199029	72231822
5507	*PKN1*	5585		-1.7									19	14433053	14471867
5508	*PKN3*	29941				-4.2							9	128702523	128720918
5509	*PKNOX1*	5316	-2.7										21	42974510	43033931
5510	*PKNOX2*	63876	-2.4										11	125164687	125433389
5511	*PKP2*	5318						1.9					12	32790745	32896840
5512	*PKP4*	8502						1.9					2	158456964	158682879
5513	*PLA2G10*	8399	-1.9										16	14672545	14694669
5514	*PLA2G16*	11145	-3.1										11	63573195	63616883
5515	*PLA2G2A*	5320	-3.7										1	19975431	19980416
5516	*PLA2G4A*	5321			-2.8								1	186828953	186988981
5517	*PLA2G4E*	123745	-3.9										15	41981582	42051190
5518	*PLA2G4F*	255189	-5.9										15	42139034	42156636
5519	*PLA2G7*	7941	2.5										6	46704201	46735693
5520	*PLAA*	9373	4.0										9	26904083	26947463
5521	*PLAC8L1*	153770	-2.1										5	146084386	146104369
5522	*PLAG1*	5324	-2.0										8	56160904	56211324
5523	*PLAT*	5327	-8.6										8	42175233	42207724
5524	*PLAUR*	5329	-4.6										19	43646095	43670547
5525	*PLBD1*	79887	-6.0										12	14503661	14568349
5526	*PLCE1*	51196	-4.7										10	93993989	94332823
5527	*PLCL2*	23228							1.6				3	16802651	17090594
5528	*PLCXD2*	257068	-5.7										3	111674676	111846447
5529	*PLD1*	5337	-3.3										3	171600405	171810950
5530	*PLD6*	201164	-2.9										17	17200995	17206315
5531	*PLEKHA2*	59339	-2.7										8	38901235	38973909
5532	*PLEKHA4*	57664				-2.5							19	48837097	48868632
5533	*PLEKHA6*	22874	-2.0										1	204218851	204377665
5534	*PLEKHB2*	55041									-2.0		2	131104847	131353709
5535	*PLEKHG1*	57480	-7.3										6	150599863	150843665

5536	*PLEKHG3*	26030									-1.9		14	64704102	64750247
5537	*PLEKHG4B*	153478	-6.7										5	140258	189970
5538	*PLEKHG5*	57449	-2.7										1	6466092	6520061
5539	*PLEKHH1*	57475	-3.2										14	67533301	67589612
5540	*PLEKHH2*	130271	-8.6										2	43637273	43767987
5541	*PLEKHH3*	79990	-6.7										17	42667914	42676994
5542	*PLEKHM3*	389072	-3.1										2	207828303	208025560
5543	*PLEKHO2*	80301									-1.5		15	64841883	64868007
5544	*PLIN2*	123			-4.1								9	19108375	19149290
5545	*PLIN3*	10226	-4.4										19	4838341	4867768
5546	*PLLP*	51090			3.3								16	57248547	57284687
5547	*PLN*	5350	-1.9										6	118548298	118560730
5548	*PLP1*	5354								-1.6			X	103773718	103792619
5549	*PLPP1*	8611		-1.7									5	55424854	55535050
5550	*PLPP2*	8612	-3.2										19	281040	291504
5551	*PLPPR2*	64748	-5.2										19	11355386	11365698
5552	*PLRG1*	5356				3.5							4	154535006	154550435
5553	*PLS3*	5358	-6.2										X	115561174	115650861
5554	*PLSCR1*	5359									-1.7		3	146515180	146544864
5555	*PLXDC1*	57125	-6.1										17	39063303	39154394
5556	*PLXNA1*	5361						1.8					3	126988594	127037392
5557	*PLXNA4*	91584	-2.5										7	132123332	132648688
5558	*PLXNB2*	23654	-3.3										22	50274979	50307627
5559	*PM20D2*	135293	-3.3										6	89146050	89165565
5560	*PMAIP1*	5366		-2.4									18	59899948	59904306
5561	*PMEPA1*	56937	-2.7										20	57648392	57711536
5562	*PMPCB*	9512	2.9										7	103297422	103329511
5563	*PMVK*	10654		-1.6									1	154924734	154936991
5564	*PNISR*	25957					-2.1						6	99398051	99425331
5565	*PNLIPRP3*	119548			-1.6								10	116427867	116477957
5566	*PNMA1*	9240		2.4									14	73711783	73714372
5567	*PNMA2*	10687	-3.8										8	26504686	26514092
5568	*PNMAL1*	55228	-2.9										19	46466491	46471563
5569	*PNN*	5411	5.5										14	39175183	39183218
5570	*PNPLA1*	285848	-5.5										6	36243203	36308595
5571	*PNPLA8*	50640					-1.6						7	108470422	108569666
5572	*PNPT1*	87178	-3.8										2	55634265	55693910
5573	*PNRC1*	10957			-2.3								6	89080751	89085160
5574	*PNRC2*	55629	3.8										1	23959109	23963462
5575	*PODXL*	5420	-7.1										7	131500262	131558217
5576	*POFUT1*	23509	-2.6										20	32207880	32238667
5577	*POLB*	5423							-1.5				8	42338454	42371808
5578	*POLE2*	5427	-3.5										14	49643555	49688422
5579	*POLE3*	54107										-1.6	9	113407235	113410672
5580	*POLE4*	56655	-3.0										2	74958492	74970128
5581	*POLG*	5428				3.4							15	89316305	89334861
5582	*POLH*	5429	-4.2										6	43576150	43615660
5583	*POLK*	51426	-6.9										5	75511756	75601144
5584	*POLN*	353497	-3.5										4	2071918	2242121
5585	*POLR1C*	9533	3.0										6	43509702	43529585
5586	*POLR1D*	51082	3.1										13	27620742	27667415
5587	*POLR1E*	64425			-1.6								9	37485935	37503697
5588	*POLR2A*	5430	3.0										17	7484366	7514618
5589	*POLR2B*	5431	-3.3										4	56977722	57031168
5590	*POLR2E*	5434					1.7						19	1086579	1095380
5591	*POLR2J2*	246721	-2.5										7	102665368	102671629
5592	*POLR2J3*	548644	-2.7										7	102537918	102572656
5593	*POLR2K*	5440					-1.7						8	100150584	100154002
5594	*POM121*	9883	5.0										7	72879365	72951440

5595	*POM121C*	100101267	3.4										7	75416787	75486271
5596	*POMGNT1*	55624	-4.8										1	46188682	46220305
5597	*PON2*	5445	-4.2										7	95404863	95435329
5598	*PON3*	5446	-1.9										7	95359944	95396368
5599	*POP5*	51367	-3.1										12	120578764	120581398
5600	*POPDC2*	64091	-8.2										3	119636457	119665324
5601	*POU2AF1*	5450	-3.7										11	111352252	111455630
5602	*POU2F3*	25833	-2.2										11	120236640	120319944
5603	*POU5F1*	5460	-7.2										6	31164337	31180731
5604	*POU6F1*	5463	-2.6										12	51186936	51217693
5605	*PPARA*	5465	-5.8										22	46150521	46243756
5606	*PPARGC1A*	10891	-2.6										4	23755041	23904089
5607	*PPEF2*	5470	-5.2										4	75859864	75902571
5608	*PPFIA1*	8500	3.9										11	70270700	70384403
5609	*PPFIA3*	8541	-3.5										19	49119389	49151026
5610	*PPFIBP1*	8496	-4.8										12	27523431	27695564
5611	*PPIE*	10450	-3.9										1	39692182	39763914
5612	*PPIG*	9360	3.1										2	169584340	169641406
5613	*PPIH*	10465		-2.2									1	42658425	42676758
5614	*PPIL4*	85313	-4.7										6	149504733	149546038
5615	*PPIL6*	285755	-7.1										6	109390215	109441171
5616	*PPM1A*	5494	4.4										14	60245752	60299087
5617	*PPM1M*	132160							1.7				3	52245793	52250597
5618	*PPME1*	51400						1.6					11	74171099	74254703
5619	*PPOX*	5498		-1.6									1	161166410	161178013
5620	*PPP1CB*	5500	-3.0										2	28751640	28802940
5621	*PPP1R12B*	4660	-5.3										1	202348699	202592706
5622	*PPP1R14C*	81706								-2.7			6	150143076	150250357
5623	*PPP1R18*	170954					1.8						6	30676389	30687895
5624	*PPP1R2*	5504									-2.0		3	195514425	195543386
5625	*PPP1R21*	129285									-1.6		2	48440598	48515391
5626	*PPP1R27*	116729	-4.1										17	81833492	81835050
5627	*PPP1R3B*	79660	-5.8										8	9136255	9151574
5628	*PPP1R3C*	5507	-7.3										10	91628442	91633054
5629	*PPP1R3F*	89801	-2.5										X	49269843	49301461
5630	*PPP1R8*	5511	-3.0										1	27830778	27851676
5631	*PPP1R9A*	55607			2.4								7	94907202	95296415
5632	*PPP2CB*	5516							-1.5				8	30774457	30814314
5633	*PPP2R3C*	55012	-5.7										14	35085467	35122517
5634	*PPP2R5B*	5526	-5.8										11	64917553	64934473
5635	*PPP2R5C*	5527						-1.8					14	101761798	101927989
5636	*PPP2R5E*	5529	3.4										14	63371357	63543374
5637	*PPP4C*	5531				-4.5							16	30075978	30085377
5638	*PPP4R2*	151987									-1.8		3	72996785	73069199
5639	*PPP4R3B*	57223	-6.1										2	55547292	55618880
5640	*PPRC1*	23082		-1.6									10	102132994	102150331
5641	*PQBP1*	10084	3.6										X	48897912	48903143
5642	*PQLC2*	54896	-2.9										7.748529	75036864	75110891.64
5643	*PQLC3*	130814					-1.6						2	11155198	11178874
5644	*PRAC2*	360205	2.5										17	48723168	48724758
5645	*PRAF2*	11230						2.0					X	49071156	49074071
5646	*PRB3*	5544		-1.9									12	11265924	11269805
5647	*PRCC*	5546	2.4										1	156750610	156800817
5648	*PRDM4*	11108	3.0										12	107732866	107761272
5649	*PRDM7*	11105	-3.4										16	90056566	90092072
5650	*PRDX3*	10935					-1.7						10	119167703	119178833
5651	*PREB*	10113				-3.4							2	27130756	27134675
5652	*PRELID1*	27166			2.1								5	177303774	177306959
5653	*PRELID2*	153768	-2.3										5	145471799	145835369

5654	*PRELP*	5549	-5.6										1	203475828	203491352
5655	*PREX2*	80243	-5.7										8	67952118	68237030
5656	*PRICKLE1*	144165	-3.8										12	42456754	42590355
5657	*PRIM1*	5557	-3.0										12	56731596	56752373
5658	*PRIM2*	5558	-2.3										6	57314805	57646849
5659	*PRIMPOL*	201973			2.4								4	184649613	184694963
5660	*PRKAB2*	5565			-3.3								1	147155106	147172550
5661	*PRKACB*	5567	-5.2										1	84078062	84238498
5662	*PRKAG1*	5571									-1.5		12	49002274	49019197
5663	*PRKAR1A*	5573										-1.6	17	68511780	68551319
5664	*PRKAR1B*	5575		1.9									7	549197	727650
5665	*PRKCA*	5578	-4.4										17	66302636	66810743
5666	*PRKCDBP*	112464			3.1								11	6318946	6320647
5667	*PRKCE*	5581	-1.7										2	45651345	46187990
5668	*PRKCG*	5582	-3.8										19	53879190	53907652
5669	*PRKCI*	5584	-4.0										3	170222365	170305981
5670	*PRKCSH*	5589									-2.2		19	11435288	11450968
5671	*PRKRIP1*	79706		2.1									7	102363872	102426676
5672	*PRMT5*	10419	3.1										14	22920511	22929585
5673	*PRND*	23627	-2.6										20	4721910	4728460
5674	*PRODH*	5625	-3.5										22	18912777	18936553
5675	*PROM1*	8842	-1.8										4	15963076	16084378
5676	*PROM2*	150696	-5.9										2	95274453	95291308
5677	*PROSER1*	80209			-2.8								13	39009866	39038076
5678	*PRPF40A*	55660	4.0										2	152651593	152717997
5679	*PRPF6*	24148				1.6							20	63981135	64033100
5680	*PRPF8*	10594	-2.9										17	1650629	1684882
5681	*PRR11*	55771	-5.3										17	59155499	59204705
5682	*PRR12*	57479	-2.2										19	49591643	49626439
5683	*PRR14*	78994					1.7						16	30650717	30656440
5684	*PRR15*	222171	-1.9										7	29563811	29567295
5685	*PRR19*	284338	-5.4										19	42302098	42310821
5686	*PRR23A*	729627	-4.1										3	139005468	139006268
5687	*PRR23C*	389152	-4.8										3	139042102	139044892
5688	*PRR27*	401137	-3.1										4	70133616	70176799
5689	*PRSS12*	8492	-2.8										4	118280038	118353003
5690	*PRSS27*	83886	-3.9										16	2712418	2720551
5691	*PRSS55*	203074	-2.1										8	10525546	10554166
5692	*PRX*	57716	-2.0										19	40393768	40413366
5693	*PSAT1*	29968			-2.2								9	78297143	78330093
5694	*PSCA*	8000	-5.8										8	142670308	142682724
5695	*PSENEN*	55851	-3.0										19	35745114	35747519
5696	*PSIP1*	11168				1.9							9	15464066	15511019
5697	*PSKH1*	5681								-1.6			16	67893272	67929678
5698	*PSMB3*	5691				1.9							17	38752736	38764231
5699	*PSMC4*	5704			1.8								19	39971005	39981441
5700	*PSMD1*	5707	2.7										2	231056864	231172827
5701	*PSMD3*	5709				-2.5							17	39980797	39997960
5702	*PSMD5*	5711	3.3										9	120815496	120842984
5703	*PSMD8*	5714										-1.9	19	38374536	38383824
5704	*PSME1*	5720			2.2								14	24136158	24138967
5705	*PSME4*	23198	2.5										2	53864067	53970840
5706	*PSMG4*	389362	-3.4										6	3231403	3303373
5707	*PTBP2*	58155	-3.4										1	96721665	96823738
5708	*PTBP3*	9991									-1.8		9	112217716	112333667
5709	*PTCD2*	79810	-2.8										5	72320367	72368395
5710	*PTCHD1*	139411	-4.2										X	23334015	23404372
5711	*PTEN*	5728	-6.5										10	87863113	87971930
5712	*PTGDR2*	11251			-1.8								11	60850940	60855971

5713	*PTGDS*	5730	-4.5										9	136977505	136985435
5714	*PTGER3*	5733	-2.4										1	70852353	71047808
5715	*PTGES*	9536	-3.5										9	129738331	129753047
5716	*PTGES3*	10728							-1.6				12	56663341	56688408
5717	*PTGIS*	5740	-5.4										20	49503874	49568146
5718	*PTGS2*	5743									-2.3		1	186671791	186680427
5719	*PTK6*	5753	-2.9										20	63528001	63537370
5720	*PTK7*	5754	-4.9										6	43076268	43161719
5721	*PTMA*	5757	2.1										2	231706895	231713541
5722	*PTN*	5764			5.2								7	137227341	137343865
5723	*PTOV1*	53635				-3.2							19	49850735	49860744
5724	*PTPDC1*	138639				-3.5							9	94030794	94109856
5725	*PTPN12*	5782									-2.2		7	77537275	77640071
5726	*PTPN13*	5783	-5.0										4	86594315	86815171
5727	*PTPN14*	5784	-5.2										1	214348696	214552449
5728	*PTPN7*	5778	-3.0										1	202147013	202161588
5729	*PTPRF*	5792	-3.1										1	43525187	43623666
5730	*PTPRH*	5794	-2.0										19	55181248	55209506
5731	*PTPRU*	10076	-2.7										1	29236516	29326813
5732	*PTRHD1*	391356			3.5								2	24789734	24793382
5733	*PUDP*	8226	-3.4										X	7048920	7148190
5734	*PUM2*	23369									-1.6		2	20248691	20352234
5735	*PURA*	5813								-1.8			5	140107777	140125619
5736	*PUS10*	150962	-3.9										2	60940222	61018259
5737	*PVRL1*	5818	-3.4										11	119623408	119729084
5738	*PVRL2*	5819		3.0									19	44846175	44889228
5739	*PWP2*	5822	2.9										21	44107290	44131181
5740	*PWWP2A*	114825				1.6							5	160061801	160119423
5741	*PXMP2*	5827			-1.6								12	132687606	132704991
5742	*PXMP4*	11264	-4.9										20	33702754	33720319
5743	*PXYLP1*	92370		-1.6									3	141228726	141294906
5744	*PYCRL*	65263	-4.8										8	143603913	143609773
5745	*PYM1*	84305			-3.1								12	55901413	55932618
5746	*QPCTL*	54814	-3.4										2.304233	75026926.8	75100906.96
5747	*QRFPR*	84109	-8.5										4	121329312	121381059
5748	*QRSL1*	55278	-4.9										6	106629578	106668417
5749	*QSOX1*	5768	-6.2										1	180154834	180204030
5750	*QTRTD1*	79691	-4.8										3	114005833	114088422
5751	*R3HDM1*	23518		-1.6									2	135531455	135725270
5752	*RAB11A*	8766			1.6								15	65726054	65891991
5753	*RAB11FIP1*	80223	-3.1										8	37858618	37899467
5754	*RAB14*	51552	3.4										9	121178137	121223014
5755	*RAB15*	376267			-1.9								14	64945814	64972776
5756	*RAB17*	64284			-2.1								2	237574322	237601614
5757	*RAB20*	55647					1.7						13	110523066	110561733
5758	*RAB21*	23011	-3.8										12	71754874	71800285
5759	*RAB22A*	57403	-3.7										20	58309696	58367507
5760	*RAB31*	11031									-1.9		18	9708165	9862551
5761	*RAB33B*	83452	-2.3										4	139453232	139476609
5762	*RAB36*	9609	-6.5										22	23145326	23164350
5763	*RAB38*	23682	-2.8										11	88113242	88175467
5764	*RAB3B*	5865	-3.8										1	51907956	51990764
5765	*RAB3GAP1*	22930	-3.0										2	135052265	135176394
5766	*RAB40B*	10966	3.6										17	82654973	82698728
5767	*RAB42*	115273	-4.5										1	28592200	28595443
5768	*RAB43*	339122	-4.7										3	129087569	129122801
5769	*RAB5C*	5878						1.8					17	42124976	42155044
5770	*RAB6A*	5870									-2.7		11	73675638	73761137
5771	*RABAC1*	10567				-5.4							19	41956681	41959390

5772	*RABEPK*	10244	-4.6										9	125200542	125234158
5773	*RAD51*	5888	-3.2										15	40694774	40732339
5774	*RAD51C*	5889	-6.8										17	58692573	58735611
5775	*RAD54B*	25788	-3.1										8	94371960	94475109
5776	*RAET1E*	135250	-2.5										6	149883375	149898102
5777	*RAF1*	5894									-1.9		3	12583601	12664226
5778	*RALA*	5898							-1.9				7	39623483	39708124
5779	*RALGAPA1*	253959	-5.0										14	35538352	35809304
5780	*RALGAPA2*	57186	-5.6										20	20389552	20712488
5781	*RALGPS1*	9649	-2.4										9	126914774	127223166
5782	*RAMP1*	10267	-3.1										2	237858893	237912114
5783	*RANBP17*	64901	-2.0										5	170861870	171300015
5784	*RANBP9*	10048	-3.4										6	13621498	13711564
5785	*RAP1B*	5908									-2.9		12	68610839	68671901
5786	*RAP1GAP*	5909	2.5										1	21596215	21669363
5787	*RAP2C*	57826						-1.6					X	132203024	132219480
5788	*RAPGEFL1*	51195	-2.3										17	40177010	40195656
5789	*RARRES2*	5919	-4.0										7	150338317	150341674
5790	*RARS*	5917	-3.6										5	168486445	168519299
5791	*RASA4*	10156	-6.0										7	102573807	102616757
5792	*RASAL1*	8437	-1.8										12	113098819	113136239
5793	*RASGEF1B*	153020	-3.6										4	81426393	82044244
5794	*RASGRP3*	25780	-5.9										2	33436324	33564750
5795	*RASGRP4*	115727	-4.0										19	38409051	38426305
5796	*RASSF4*	83937							1.8				10	44959407	44995891
5797	*RASSF8*	11228	-3.2										12	25959029	26079892
5798	*RBAK*	57786			-2.7								7	5045821	5069488
5799	*RBAK-RBAKDN*	100533952			-2.7								7	4983718	5073221
5800	*RBBP9*	10741	-3.4										20	18486540	18497243
5801	*RBFOX2*	23543	3.6										22	35738736	36028425
5802	*RBL1*	5933	-3.7										20	36996349	37095995
5803	*RBM10*	8241				-3.7							X	47144869	47186813
5804	*RBM18*	92400	5.3										9	122237624	122264839
5805	*RBM22*	55696				4.4							5	150690794	150701107
5806	*RBM23*	55147	-3.1										14	22893206	22919184
5807	*RBM25*	58517	3.8										14	73058436	73123898
5808	*RBM26*	64062	2.5										13	79311824	79406477
5809	*RBM38*	55544									-1.7		20	57391407	57409333
5810	*RBM43*	375287	-3.1										2	151247940	151261879
5811	*RBM48*	84060	-2.3										7	92528773	92538005
5812	*RBM8A*	9939							-1.6				1	145917714	145927678
5813	*RBMS1*	5937									-2.0		2	160272151	160493794
5814	*RBMS2*	5939	-3.8										12	56521929	56596196
5815	*RBMX*	27316	2.3										X	136848004	136880764
5816	*RBP1*	5947	-5.1										3	139517434	139539829
5817	*RBPMS2*	348093	-6.2										15	64739892	64775587
5818	*RBSN*	64145	-5.4										3	15070073	15099163
5819	*RBX1*	9978			2.0								22	40951347	40973309
5820	*RC3H2*	54542	-2.1										12.79028	75013562.9	75087479.3
5821	*RCC1*	1104				3.3							1	28505943	28539300
5822	*RCC2*	55920										-1.6	1	17406760	17439724
5823	*RCOR1*	23186									-1.7		14	102592661	102730576
5824	*RD3*	343035	-1.7										1	211476522	211492917
5825	*RDH10*	157506	-5.5										8	73294612	73325281
5826	*RDH5*	5959	-3.1										12	55720367	55724705
5827	*RDM1*	201299	-1.9										17	35918066	35930773
5828	*RECK*	8434	-1.6										9	36036433	36124451
5829	*RELA*	5970			-2.7								11	65653596	65663094
5830	*REV1*	51455	3.3										2	99400475	99490035

5831	*RFFL*	117584									-2.6		17	35005990	35089319
5832	*RFPL1*	5988	-2.4										22	29438583	29442455
5833	*RFPL2*	10739			-2.4								22	32190435	32203477
5834	*RFT1*	91869	-3.7										3	53088483	53130462
5835	*RFX5*	5993	2.7										1	151340640	151347357
5836	*RGL3*	57139	-4.2										19	11384341	11419342
5837	*RGS12*	6002	-4.4										4	3293028	3439913
5838	*RGS14*	10636					1.7						5	177357837	177372601
5839	*RGS4*	5999		-1.6									1	163068775	163076802
5840	*RGSL1*	353299	-2.2										1	182409192	182560599
5841	*RHAG*	6005										1.9	6	49605158	49636839
5842	*RHBDD2*	57414	-3.0										7	75842602	75888926
5843	*RHBDF2*	79651					2.0						17	76470891	76501790
5844	*RHBG*	57127	-6.8										1	156369212	156385219
5845	*RHCG*	51458	-1.9										15	89471398	89496613
5846	*RHD*	6007	-4.0										1	25272393	25330445
5847	*RHEBL1*	121268			-2.0								12	49064685	49070025
5848	*RHOA*	387									-2.2		3	49359145	49412998
5849	*RHOB*	388		-1.6									2	20447074	20449445
5850	*RHOBTB3*	22836										1.8	5	95713522	95824383
5851	*RHOT1*	55288			-3.3								17	32142454	32253374
5852	*RHOT2*	89941	3.4										16	668086	674174
5853	*RHPN2*	85415	-3.7										19	32978593	33064888
5854	*RICTOR*	253260	-7.5										5	38937919	39074408
5855	*RIMS1*	22999	-2.4										6	71886703	72403143
5856	*RIMS2*	9699	-2.1										8	103500748	104256094
5857	*RIN1*	9610						1.9					11	66330242	66336840
5858	*RING1*	6015		-1.9									6	33208495	33212722
5859	*RINL*	126432	-3.0										19	38867834	38878279
5860	*RINT1*	60561	3.0										7	105532085	105567677
5861	*RIPK1*	8737	-4.2										6	3063991	3115187
5862	*RIPK4*	54101			-2.6								21	41739369	41767106
5863	*RIPPLY3*	53820	-3.8										21	37006150	37019659
5864	*RMI2*	116028	-2.4										16	11249619	11351762
5865	*RNASE1*	6035	-8.2										14	20801228	20803278
5866	*RNASEH1*	246243	3.0										2	3544793	3558616
5867	*RNASEH2B*	79621	-3.4										13	50909678	50973745
5868	*RNASEK*	440400				-1.7							17	7012417	7014532
5869	*RNASEL*	6041	-2.3										1	182573634	182589256
5870	*RND2*	8153	-4.3										17	43025241	43032036
5871	*RNF103*	7844	-2.9										2	86603393	86623866
5872	*RNF115*	27246						1.6					1	145738868	145824077
5873	*RNF126*	55658		-2.0									19	647526	663277
5874	*RNF138*	51444	-7.6										18	32091855	32131561
5875	*RNF144A*	9781	-3.8										2	6917392	7068286
5876	*RNF146*	81847					-1.6						6	127266610	127288567
5877	*RNF157*	114804	-3.6										17	76142453	76240373
5878	*RNF168*	165918	-3.3										3	196468783	196503768
5879	*RNF169*	254225	3.6										11	74748868	74842413
5880	*RNF170*	81790	-3.3										8	42849637	42897290
5881	*RNF182*	221687			2.2								6	13924446	13980302
5882	*RNF185*	91445			-4.1								22	31160183	31207019
5883	*RNF207*	388591	-3.3										1	6205475	6221299
5884	*RNF212*	285498	-5.7										4	1056250	1113562
5885	*RNF216*	54476	-4.1										11.69047	75007052.2	75080937.61
5886	*RNF222*	643904	-1.6										17	8390704	8397826
5887	*RNF34*	80196	4.0										12	121400041	121430623
5888	*RNF41*	10193	-5.2										12	56202175	56221933
5889	*RNF43*	54894	-5.0										8.155882	75036521.4	75110547.34

5890	*RNF44*	22838					2.2						5	176526697	176538025
5891	*RNF5*	6048				4.1							6	32178354	32180793
5892	*RNF6*	6049									-2.3		13	26132115	26222493
5893	*RNF7*	9616							-1.5				3	141738204	141747560
5894	*RNLS*	55328			-1.8								10	88273864	88584530
5895	*RNMT*	8731	-2.8										18	13726660	13764558
5896	*RNMTL1*	55178	-3.2										17	782273	792509
5897	*ROR2*	4920	-2.3										9	91563091	91950162
5898	*RORA*	6095	-2.7										15	60488284	61229319
5899	*RPA2*	6118									-1.6		1	27891524	27914746
5900	*RPE*	6120	-5.0										2	210002565	210022259
5901	*RPGR*	6103			-1.8								X	38269163	38327564
5902	*RPGRIP1*	57096	-2.5										14	21287939	21351301
5903	*RPL10*	6134		-1.6									X	154389955	154409168
5904	*RPL11*	6135		-1.9									1	23691779	23696425
5905	*RPL12*	6136		-1.6									9	127447674	127451405
5906	*RPL14*	9045		-1.6									3	40457292	40468587
5907	*RPL17*	6139		-1.7									18	49488453	49492523
5908	*RPL18A*	6142		-1.6									19	17859876	17864153
5909	*RPL22L1*	200916			2.6								3	170864875	170870483
5910	*RPL23*	9349		-1.6									17	38847865	38853843
5911	*RPL24*	6152		-1.6									3	101681091	101686782
5912	*RPL26*	6154		-1.6									17	8377520	8383213
5913	*RPL27*	6155		-1.5									17	42998273	43002959
5914	*RPL27A*	6157						1.7					11	8682411	8714759
5915	*RPL29*	6159		-1.7									3	51993600	51995942
5916	*RPL31*	6160								-1.7			2	101001715	101024032
5917	*RPL32*	6161		-1.6									3	12834485	12841588
5918	*RPL35*	11224		-1.5									9	124857880	124861981
5919	*RPL36*	25873		-1.6									19	5674947	5691876
5920	*RPL36AL*	6166									-1.7		14	49618519	49620685
5921	*RPL37*	6167					-1.7						5	40825262	40835335
5922	*RPL38*	6169						1.6					17	74203582	74210655
5923	*RPL39L*	116832			2.6								3	187120948	187180908
5924	*RPL4*	6124		-1.5									15	66498015	66524532
5925	*RPL7*	6129		-1.6									8	73290267	73295789
5926	*RPL8*	6132		-1.6									8	144789765	144792587
5927	*RPLP0*	6175		-1.6									12	120196686	120201235
5928	*RPLP2*	6181		-1.6									11	809647	812880
5929	*RPP14*	11102			-1.5								3	58306247	58324695
5930	*RPP21*	79897	-5.1										6	30345131	30346884
5931	*RPP30*	10556	-3.6										10	90871716	90908553
5932	*RPRML*	388394		-1.9									17	46978156	46979248
5933	*RPS10*	6204		-1.6									6	34417454	34426125
5934	*RPS13*	6207		-1.6									11	17074389	17077787
5935	*RPS17*	6218		-1.6									15	82536753	82540564
5936	*RPS18*	6222		-1.6									6	33272010	33276510
5937	*RPS2*	6187		-1.5									16	1962052	1964860
5938	*RPS21*	6227		-1.7									20	62387116	62388520
5939	*RPS26*	6231		-1.7									12	56041853	56044675
5940	*RPS3A*	6189		-1.9									4	151099573	151104652
5941	*RPS4Y2*	140032			2.2								Y	20756164	20781032
5942	*RPS7*	6201		-1.7									2	3575205	3580919
5943	*RPS8*	6202		-1.6									1	44775251	44778779
5944	*RPUSD3*	285367	-3.6										3	9837849	9844602
5945	*RQCD1*	9125	-1.6										2	218568580	218597080
5946	*RRAGD*	58528			-3.3								6	89364636	89412270
5947	*RRAS*	6237	-4.9										19	49635292	49640201
5948	*RRBP1*	6238				-3.6							20	17613678	17682295

5949	*RREB1*	6239						1.7					6	7107597	7251980
5950	*RRM2B*	50484			-2.8								8	102204502	102239118
5951	*RRP15*	51018	-5.5										1	218285287	218337983
5952	*RRP36*	88745	-3.9										6	43021645	43034156
5953	*RRP7A*	27341	-4.8										22	42509968	42519802
5954	*RRP9*	9136			-2.4								3	51933430	51941941
5955	*RRS1*	23212	2.5										8	66429028	66430733
5956	*RSBN1*	54665					-1.7						13.65854	75018702.8	75092643.78
5957	*RSF1*	51773	-4.7										11	77659996	77821017
5958	*RSPH14*	27156	-5.8										22	23059415	23145021
5959	*RSRP1*	57035	3.1										1	25242237	25338213
5960	*RTBDN*	83546	-2.0										19	12825478	12835428
5961	*RTCA*	8634	-4.9										1	100266207	100292769
5962	*RTCB*	51493	3.6										22	32387582	32412255
5963	*RTN3*	10313									-2.1		11	63681446	63759891
5964	*RUBCN*	9711	-3.9										3	197671393	197749727
5965	*RUNDC3A*	10900								-2.9			17	44308413	44318671
5966	*RUNDC3B*	154661	-2.6										7	87627548	87832296
5967	*RUNX1T1*	862	-2.0										8	91954967	92103286
5968	*RUVBL1*	8607	6.3										3	128064778	128153914
5969	*RUVBL2*	10856	-3.3										19	48993448	49015995
5970	*RXRB*	6257			-3.1								6	33193588	33200688
5971	*RYR1*	6261	-3.2										19	38433699	38587564
5972	*RYR2*	6262	-1.5										1	237042205	237833988
5973	*S100A10*	6281			2.2								1	151982915	151994390
5974	*S100A11*	6282				-1.8							1	152032506	152047907
5975	*S100A13*	6284	-7.1										1	153618787	153631360
5976	*S100A14*	57402	-5.2										1	153614255	153616986
5977	*S100A2*	6273	-8.1										1	153561108	153567890
5978	*S100A4*	6275					1.9						1	153543613	153550136
5979	*S100A7*	6278	-6.0										1	153457744	153460701
5980	*S100B*	6285				3.7							21	46598962	46605208
5981	*S1PR2*	9294	-4.3										19	10221435	10231272
5982	*S1PR3*	1903	-3.4										9	88991447	89005010
5983	*SAA2*	6289	-3.7										11	18239223	18248643
5984	*SAC3D1*	29901	2.6										11	65040901	65044828
5985	*SACM1L*	22908	-4.3										3	45689056	45745424
5986	*SAE1*	10055	-3.4										19	47113274	47210636
5987	*SALL1*	6299	-2.0										16	51135975	51151367
5988	*SALL4*	57167		-2.5									20	51782331	51802520
5989	*SAMD4A*	23034			-2.2								14	54567097	54793315
5990	*SAMD4B*	55095	-4.2										19	39342396	39385710
5991	*SAMD5*	389432	-5.4										6	147508927	147737547
5992	*SAMD8*	142891	-3.1										10	75099586	75182123
5993	*SAMD9L*	219285	-2.3										7	93130055	93148369
5994	*SAP30L*	79685	-3.9										5	154445957	154461054
5995	*SAPCD2*	89958	3.3										9	137062124	137070588
5996	*SART1*	9092					1.7						11	65961689	65979828
5997	*SART3*	9733	-3.9										12	108522580	108561400
5998	*SASH1*	23328	-2.7										6	148272304	148552050
5999	*SBF2*	81846	-5.1										11	9778667	10294207
6000	*SBNO1*	55206	-4.6										12	123289109	123364843
6001	*SBNO2*	22904	-3.5										19	1107636	1174283
6002	*SBSPON*	157869	-5.5										8	73064540	73124088
6003	*SCAF11*	9169							1.6				12	45919131	45992120
6004	*SCAF8*	22828	2.4										6	154733325	154834244
6005	*SCAI*	286205	-4.7										9	124942608	125143506
6006	*SCAMP1*	9522	-3.2										5	78360583	78479071
6007	*SCAMP4*	113178	-3.7										19	1905214	1926013

6008	*SCARF1*	8578	-6.7										17	1633858	1645747
6009	*SCD5*	79966	-5.0										4	82629539	82798857
6010	*SCEL*	8796	-5.4										13	77535674	77645263
6011	*SCGB1A1*	7356	-8.2										11	62405103	62423195
6012	*SCHIP1*	29970			4.2								3	159839861	159897360
6013	*SCLT1*	132320	-3.3										4	128864921	129093607
6014	*SCLY*	51540	4.1										2	238060889	238099413
6015	*SCML2*	10389	-2.3										X	18239314	18354727
6016	*SCN2B*	6327	-3.2										11	118161951	118176673
6017	*SCN3B*	55800	-2.0										11	123629187	123655244
6018	*SCNM1*	79005	-3.9										1	151156664	151170297
6019	*SCNN1G*	6340	-1.5										16	23182715	23216883
6020	*SCO2*	9997	3.2										22	50523568	50526439
6021	*SCP2*	6342					-1.5						1	52927229	53051703
6022	*SCRIB*	23513	3.4										8	143790920	143815379
6023	*SCYL1*	57410	3.5										11	65525077	65538704
6024	*SCYL2*	55681	-4.8										12	100267140	100341724
6025	*SDCCAG3*	10807	-2.9										9	136401922	136410609
6026	*SDCCAG8*	10806	-3.7										1	243256034	243500092
6027	*SDE2*	163859	-4.9										1	225982702	225999331
6028	*SDHB*	6390	4.2										1	17018722	17054170
6029	*SDK2*	54549	-3.3										12.96393	75014590.9	75088512.19
6030	*SDR39U1*	56948	3.1										14	24439766	24442905
6031	*SDS*	10993	-2.3										12	113392445	113426301
6032	*SEC14L3*	266629	-3.9										22	30447959	30472049
6033	*SEC14L4*	284904	-5.5										22	30488913	30505711
6034	*SEC22A*	26984	-2.6										3	123201927	123274130
6035	*SEC22C*	9117	-3.4										3	42547969	42601080
6036	*SEC24A*	10802					-1.5						5	134648789	134727823
6037	*SEC24C*	9632					2.2						10	73744384	73772161
6038	*SEC61B*	10952	4.9										9	99222064	99230615
6039	*SEC62*	7095					-1.6						3	169966635	169998373
6040	*SEL1L2*	80343	-3.0										20	13849247	13996443
6041	*SELM*	140606	-3.3										22	31104772	31120069
6042	*SEMA3A*	10371										1.6	7	83955777	84492724
6043	*SEMA3D*	223117	-1.5										7	84995553	85186855
6044	*SEMA4C*	54910	-7.2										4.897059	75039262.7	75113301.73
6045	*SEMA6A*	57556		-1.8									5	116443616	116574934
6046	*SENP2*	59343	-2.8										3	185582496	185633551
6047	*SENP5*	205564	-5.1										3	196867856	196934714
6048	*SENP7*	57337									-2.0		3	101324205	101513241
6049	*SEPHS1*	22929	3.2										10	13317424	13348298
6050	*SEPSECS*	51091	-3.4										4	25120014	25160442
6051	*SERBP1*	26135					-1.7						1	67407810	67430415
6052	*SERINC1*	57515									-2.4		6	122443354	122471822
6053	*SERPINA1*	5265	3.5										14	94376747	94390693
6054	*SERPINB5*	5268	-5.7										18	63476761	63505085
6055	*SERPINC1*	462										2.1	1	173903804	173917378
6056	*SERPIND1*	3053										2.9	22	20773879	20787720
6057	*SERPINE3*	647174				-3.8							13	51335773	51364735
6058	*SERTAD1*	29950			-1.8								19	40421592	40426025
6059	*SERTAD2*	9792	-4.4										2	64631621	64751005
6060	*SESN1*	27244			-2.3								6	108986437	109094819
6061	*SETD1A*	9739	-7.5										16	30957294	30985116
6062	*SETD7*	80854					-1.8						4	139495941	139606699
6063	*SETD9*	133383			-2.3								5	56909260	56925532
6064	*SETDB2*	83852				-3.5							13	49444374	49495003
6065	*SF3A2*	8175					1.7						19	2236504	2248679
6066	*SF3B1*	23451									-1.7		2	197389784	197435091

6067	*SF3B2*	10992	3.1										11	66050729	66069308
6068	*SF3B6*	51639	5.9										2	24067584	24076443
6069	*SFMBT1*	51460	-5.0										3	52903572	53046750
6070	*SFMBT2*	57713	-4.8										10	7158624	7411486
6071	*SFRP1*	6422	-2.1										8	41261958	41309497
6072	*SFRP4*	6424	-1.6										7	37905932	38025695
6073	*SFSWAP*	6433	4.8										12	131711081	131799737
6074	*SFTPA2*	729238	-5.9										10	79555852	79560397
6075	*SFXN3*	81855	-2.2										10	101031234	101041244
6076	*SGCB*	6443	-2.7										4	52020706	52038482
6077	*SGK1*	6446					-1.7						6	134169246	134318112
6078	*SGK3*	23678	-4.9										8	66712418	66862022
6079	*SGOL1*	151648	-5.7										3	20160593	20186292
6080	*SGOL2*	151246			2.7								2	200510008	200583782
6081	*SGSM1*	129049	-5.1										22	24806169	24927578
6082	*SGSM2*	9905	-5.1										17	2337498	2381058
6083	*SGTB*	54557	-3.0										13.25335	75016304.2	75090233.69
6084	*SH2D4B*	387694	-2.0										10	80537902	80646560
6085	*SH3BGR*	6450			-3.9								21	39445855	39515506
6086	*SH3BP1*	23616	-4.2										22	37634654	37666932
6087	*SH3BP2*	6452	-6.2										4	2793023	2841098
6088	*SH3GLB1*	51100									-1.6		1	86704570	86748184
6089	*SH3KBP1*	30011				3.0							X	19533975	19887601
6090	*SH3RF2*	153769	-4.9										5	145936579	146081791
6091	*SH3TC2*	79628	-5.6										5	148923639	149063163
6092	*SHE*	126669	-1.7										1	154469772	154502113
6093	*SHISA5*	51246	-3.9										3	48467798	48504826
6094	*SHISA9*	729993	-6.9										16	12901620	13240413
6095	*SHOC2*	8036			1.9								10	110919547	111013667
6096	*SHPK*	23729	-5.5										17	3608262	3636322
6097	*SHQ1*	55164	3.5										3	72749277	72861914
6098	*SHROOM1*	134549	-5.0										5	132822141	132830898
6099	*SHROOM4*	57477	-5.0										X	50591647	50814302
6100	*SHTN1*	57698										-1.6	10	116881482	117126586
6101	*SIAE*	54414						1.8					10.88009	75002254.9	75076117.42
6102	*SIAH2*	6478					1.8						3	150741127	150763477
6103	*SIDT1*	54847	-3.0										7.290909	75030353.4	75104349.95
6104	*SIGLEC8*	27181	-3.6										19	51450847	51458456
6105	*SIK2*	23235	3.0										11	111602391	111730853
6106	*SIK3*	23387	-4.3										11	116843402	117098437
6107	*SIMC1*	375484	-6.5										5	176238367	176345991
6108	*SIPA1L2*	57568		2.1									1	232397965	232561558
6109	*SIRT5*	23408	-3.1										6	13574529	13615158
6110	*SIVA1*	10572	-3.5										14	104753100	104768494
6111	*SIX4*	51804	-6.3										14	60709528	60724348
6112	*SKA2*	348235			2.2								17	59109951	59155269
6113	*SKAP1*	8631	-4.1										17	48133440	48430275
6114	*SKIDA1*	387640	-3.5										10	21513478	21526368
6115	*SKP1*	6500						1.5					5	134148935	134177038
6116	*SKP2*	6502				-3.0							5	36151989	36184319
6117	*SLAIN2*	57606	3.3										4	48341322	48426212
6118	*SLAMF1*	6504	-4.0										1	160608100	160647295
6119	*SLAMF6*	114836		-1.8									1	160485030	160523262
6120	*SLAMF7*	57823		-1.5									1	160739057	160754821
6121	*SLBP*	7884	-3.0										4	1692800	1712555
6122	*SLC11A2*	4891	-4.0										12	50979401	51028566
6123	*SLC12A2*	6558			2.2								5	128083766	128189688
6124	*SLC12A3*	6559	-3.0										16	56865207	56915850
6125	*SLC12A8*	84561	-3.8										3	125082636	125212864

6126	*SLC13A5*	284111	-3.8										17	6684713	6713567
6127	*SLC14A1*	6563	-3.0										18	45724127	45752520
6128	*SLC14A2*	8170	-5.8										18	45212995	45683686
6129	*SLC15A1*	6564	-6.5										13	98683801	98752654
6130	*SLC16A12*	387700	-4.9										10	89430299	89556641
6131	*SLC16A3*	9123	-2.0										17	82228397	82261129
6132	*SLC16A4*	9122	-5.2										1	110362848	110391082
6133	*SLC16A7*	9194					-2.3						12	59596067	59789855
6134	*SLC17A7*	57030	-1.8										19	49429401	49442360
6135	*SLC1A2*	6506	-3.6										11	35251206	35420063
6136	*SLC1A4*	6509				-1.6							2	64988477	65023865
6137	*SLC1A6*	6511	-2.0										19	14950034	15022990
6138	*SLC22A15*	55356	-4.3										1	115976498	116070054
6139	*SLC22A18*	5002					1.9						11	2899721	2925246
6140	*SLC22A8*	9376	-2.4										11	62989154	63015839
6141	*SLC25A10*	1468	4.1										17	81712236	81721016
6142	*SLC25A11*	8402				-1.8							17	4937130	4940251
6143	*SLC25A12*	8604	-2.2										2	171784370	171999859
6144	*SLC25A14*	9016	-2.4										X	130339888	130373361
6145	*SLC25A15*	10166	-4.1										13	40789412	40810111
6146	*SLC25A18*	83733	-2.7										22	17563439	17590994
6147	*SLC25A20*	788	-3.4										3	48856931	48898993
6148	*SLC25A26*	115286	-4.1										3	66133610	66388116
6149	*SLC25A27*	9481	-6.7										6	46652915	46678193
6150	*SLC25A28*	81894		-1.6									10	99610522	99620609
6151	*SLC25A34*	284723	-2.2										1	15736405	15741396
6152	*SLC25A38*	54977	4.7										10.71989	75047144	75121220.61
6153	*SLC25A39*	51629	1.8										17	44319625	44324870
6154	*SLC25A4*	291	-5.8										4	185143241	185150382
6155	*SLC25A40*	55972		1.9									7	87833568	87876357
6156	*SLC25A42*	284439	-2.4										19	19063999	19112888
6157	*SLC25A45*	283130	-4.6										11	65375192	65383701
6158	*SLC25A51*	92014	-5.2										9	37879400	37904353
6159	*SLC25A53*	401612	-5.0										X	104099214	104157027
6160	*SLC26A2*	1836	-3.1										5	149960737	149993455
6161	*SLC26A4*	5172	-7.7										7	107660635	107717809
6162	*SLC26A8*	116369	-3.3										6	35943514	36024868
6163	*SLC27A1*	376497	-6.1										19	17468769	17506168
6164	*SLC27A4*	10999	-1.9										9	128340646	128361470
6165	*SLC28A2*	9153	-3.5										15	45252230	45277845
6166	*SLC2A1*	6513								-1.8			1	42925375	42959173
6167	*SLC2A10*	81031	-3.7										20	46709487	46736347
6168	*SLC2A11*	66035	-4.0										22	23856703	23886309
6169	*SLC2A4*	6517	-1.7										17	7281667	7288257
6170	*SLC2A5*	6518	-2.0										1	9035107	9088478
6171	*SLC2A6*	11182		-1.6									9	133471095	133479137
6172	*SLC2A8*	29988	2.9										9	127397138	127408424
6173	*SLC30A5*	64924	-4.6										5	69093646	69131069
6174	*SLC30A7*	148867	-3.5										1	100896076	100981753
6175	*SLC33A1*	9197	-5.7										3	155821024	155854429
6176	*SLC35A2*	7355							-2.0				X	48903180	48911958
6177	*SLC35A4*	113829			2.0								5	140564456	140569103
6178	*SLC35B1*	10237	5.7										17	49700943	49709014
6179	*SLC35B4*	84912	-3.5										7	134289332	134317051
6180	*SLC35C1*	55343	2.6										11	45804072	45813015
6181	*SLC35E1*	79939	-2.5										19	16549831	16572382
6182	*SLC35E4*	339665	-2.0										22	30635652	30669016
6183	*SLC35F1*	222553	-2.6										6	117907526	118317676
6184	*SLC35F6*	54978	-4.6										10.76303	75047486.6	75121564.91

6185	*SLC36A2*	153201	-4.2										5	151314978	151347590
6186	*SLC36A3*	285641	-2.6										5	151276762	151303766
6187	*SLC37A4*	2542	2.8										11	119024114	119030906
6188	*SLC38A8*	146167	-3.1										16	84009667	84042636
6189	*SLC38A9*	153129	2.5										5	55625845	55773194
6190	*SLC39A1*	27173	-3.5										1	153959099	153968184
6191	*SLC39A13*	91252	-2.0										11	47407132	47416501
6192	*SLC39A2*	29986	-5.6										14	20999255	21001871
6193	*SLC39A3*	29985		1.9									19	2732204	2740152
6194	*SLC39A5*	283375	-4.5										12	56230049	56237846
6195	*SLC39A8*	64116	-4.9										4	102251041	102431258
6196	*SLC40A1*	30061	-4.9										2	189560579	189583758
6197	*SLC41A2*	84102	-3.2										12	104802553	104958744
6198	*SLC44A3*	126969	-6.1										1	94820342	94895246
6199	*SLC45A2*	51151			-4.7								5	33944616	33984730
6200	*SLC46A1*	113235	-3.7										17	28394756	28407197
6201	*SLC46A3*	283537	-6.6										13	28700064	28718970
6202	*SLC4A4*	8671	-4.6										4	71187286	71572087
6203	*SLC4A5*	57835			-2.6								2	74216242	74343414
6204	*SLC4A8*	9498	-5.7										12	51391317	51515763
6205	*SLC50A1*	55974						1.8					1	155135344	155138857
6206	*SLC52A1*	55065	-5.2										17	5032600	5052009
6207	*SLC5A1*	6523	-4.5										22	32043032	32113029
6208	*SLC5A10*	125206	-4.1										17	18950345	19022595
6209	*SLC5A3*	6526	2.7										21	34073570	34106260
6210	*SLC5A4*	6527			-3.1								22	32218476	32255341
6211	*SLC5A5*	6528	-6.1										19	17871973	17895174
6212	*SLC6A11*	6538	-4.6										3	10816200	10940733
6213	*SLC6A14*	11254	-3.0										X	116436622	116461458
6214	*SLC6A20*	54716	-2.3										5.875661	75020758.8	75094709.58
6215	*SLC6A4*	6532	-2.6										17	30194319	30236002
6216	*SLC7A11*	23657	-5.3										4	138164097	138242349
6217	*SLC7A14*	57709	-4.6										3	170459584	170586074
6218	*SLC7A7*	9056					1.9						14	22773222	22829820
6219	*SLC7A8*	23428			-1.9								14	23125295	23183674
6220	*SLC8B1*	80024	-2.3										12	113298759	113359493
6221	*SLC9A3R1*	9368					1.6						17	74748652	74769353
6222	*SLC9A3R2*	9351	-2.9										16	2025356	2039026
6223	*SLC9A8*	23315					1.6						20	49812713	49892242
6224	*SLCO1A2*	6579	-2.1										12	21264600	21419594
6225	*SLCO4A1*	28231			2.8								20	62642445	62685785
6226	*SLFN5*	162394			-3.6								17	35243036	35273655
6227	*SLITRK2*	84631			-1.7								X	145817832	145825842
6228	*SLMAP*	7871	-3.1										3	57755450	57929168
6229	*SLPI*	6590	-8.3										20	45252239	45254564
6230	*SLX4*	84464	-3.3										16	3581181	3611598
6231	*SMAD2*	4087						1.7					18	47808957	47931146
6232	*SMAD4*	4089	-4.5										18	51028394	51085045
6233	*SMAD5*	4090								-2.3			5	136132845	136188747
6234	*SMAD9*	4093	-3.0										13	36844831	36920765
6235	*SMAP1*	60682					-1.6						6	70667776	70862015
6236	*SMARCD2*	6603								-1.8			17	63832081	63843065
6237	*SMARCE1*	6605							-1.5				17	40624962	40648508
6238	*SMC1A*	8243		-1.7									X	53374149	53422728
6239	*SMC2*	10592			2.7								9	104094260	104141417
6240	*SMC6*	79677	-5.2										2	17663812	17800242
6241	*SMDT1*	91689			2.3								22	42079691	42084284
6242	*SMG7*	9887	3.2										1	183472216	183598246
6243	*SMG8*	55181			-1.7								17	59209400	59215247

6244	*SMIM10L2B*	644596	-3.3										X	135094985	135098703
6245	*SMIM7*	79086				1.6							19	16630751	16660442
6246	*SMN1*	6606				3.4							5	70049612	70078522
6247	*SMO*	6608									-1.8		7	129188872	129213545
6248	*SMPD1*	6609	5.0										11	6390431	6394998
6249	*SMTN*	6525	-3.3										22	31064105	31104757
6250	*SMURF2*	64750						1.6					17	64542295	64662068
6251	*SMYD3*	64754		1.5									1	245749342	246507312
6252	*SMYD4*	114826	-5.1										17	1779485	1830634
6253	*SNAI2*	6591			-3.9								8	48917690	48921740
6254	*SNAP47*	116841	-2.7										1	227728539	227781231
6255	*SNAPC1*	6617	-4.0										14	61762357	61796428
6256	*SNRNP25*	79622	5.0										16	53010	57669
6257	*SNRNP48*	154007	-2.9										6	7590199	7611967
6258	*SNRPA1*	6627	4.8										15	101281510	101295282
6259	*SNRPD1*	6632		-1.5									18	21612267	21633524
6260	*SNRPE*	6635										-1.9	1	203861603	203870550
6261	*SNRPF*	6636	3.6										12	95858928	95903828
6262	*SNRPG*	6637	-3.6										2	70281362	70293771
6263	*SNU13*	4809		-1.6									22	41673930	41690504
6264	*SNX12*	29934	-2.0										X	71056332	71073426
6265	*SNX14*	57231	-2.9										6	85505496	85594156
6266	*SNX25*	83891	-2.7										4	185204237	185370185
6267	*SOCS2*	8835	-4.1										12	93569814	93583487
6268	*SOCS7*	30837	-2.6										17	38352228	38405593
6269	*SOD3*	6649	-4.8										4	24789912	24800842
6270	*SOHLH2*	54937			4.5								6.909091	75042004	75116056.13
6271	*SON*	6651		-1.8									21	33542618	33577481
6272	*SORBS1*	10580			-2.1								10	95311771	95561414
6273	*SORBS3*	10174	-2.0										8	22544986	22575788
6274	*SOS1*	6654	2.3										2	38981396	39124345
6275	*SOS2*	6655									-2.3		14	50117120	50231558
6276	*SOWAHC*	65124	-4.8										2	109614334	109618990
6277	*SOX13*	9580	-5.1										1	204073115	204127743
6278	*SOX6*	55553			-2.0								11	15966449	16739591
6279	*SOX7*	83595	-2.5										8	10723768	10730512
6280	*SP2*	6668	-4.9										17	47896150	47928957
6281	*SP4*	6671	-3.7										7	21428034	21514822
6282	*SPAG1*	6674	-5.4										8	100157906	100259278
6283	*SPAG9*	9043					-1.6						17	50962174	51120865
6284	*SPATA18*	132671	-1.8										4	52051331	52097292
6285	*SPATA20*	64847	4.6										17	50543058	50555852
6286	*SPATA33*	124045	-4.6										16	89657802	89671272
6287	*SPATA6L*	55064	-2.6										9	4553386	4666674
6288	*SPATS1*	221409	-2.0										6	44342660	44377167
6289	*SPATS2L*	26010	-3.9										2	200305881	200482263
6290	*SPCS1*	28972									-1.6		3	52704955	52711146
6291	*SPCS2*	9789	3.2										11	74949247	74979031
6292	*SPEN*	23013	2.3										1	15847864	15940460
6293	*SPESP1*	246777			-3.1								15	68818221	68946811
6294	*SPG21*	51324			-1.6								15	64963021	64990310
6295	*SPIB*	6689	-2.9										19	50418938	50431313
6296	*SPICE1*	152185	-3.6										3	113442718	113515187
6297	*SPIDR*	23514							2.1				8	47260575	47736306
6298	*SPINK2*	6691								1.6			4	56809860	56821742
6299	*SPIRE2*	84501	-5.1										16	89818179	89871319
6300	*SPOCK1*	6695	-6.1										5	136975298	137598379
6301	*SPOCK2*	9806	-6.3										10	72059035	72089032
6302	*SPPL2B*	56928	5.1										19	2328615	2354806

6303	*SPRN*	503542				-2.4							10	133420666	133424572
6304	*SPRR1A*	6698	-4.4										1	152985231	152985500
6305	*SPRR1B*	6699	-8.6										1	153031202	153032900
6306	*SPRR2A*	6700	-2.6										1	153056113	153057537
6307	*SPRR3*	6707	-9.0										1	153001747	153003856
6308	*SPRTN*	83932			-1.7								1	231337104	231355023
6309	*SPRY4*	81848			2.5								5	142310427	142326455
6310	*SPRYD3*	84926	-3.2										12	53064316	53079420
6311	*SPRYD4*	283377	-3.8										12	56468567	56479707
6312	*SPRYD7*	57213	-4.5										13	49912706	49936490
6313	*SPTBN1*	6711						2.4					2	54456285	54671445
6314	*SPTBN2*	6712	-3.1										11	66685248	66729226
6315	*SPTLC1*	10558	-4.9										9	92031999	92115384
6316	*SPTSSA*	171546	-3.8										14	34432789	34462356
6317	*SQLE*	6713	-3.0										8	124998497	125022283
6318	*SRCAP*	10847	-2.5										16	30698209	30741409
6319	*SRCIN1*	80725	-4.1										17	38530016	38605930
6320	*SRD5A3*	79644	-5.5										4	55346109	55373096
6321	*SREK1*	140890	6.7										5	66139971	66183615
6322	*SRF*	6722			-2.0								6	43171299	43181507
6323	*SRFBP1*	153443	-2.2										5	121961961	122075570
6324	*SRGAP1*	57522	-2.8										12	63844293	64162221
6325	*SRGAP3*	9901	-4.5										3	8980591	9363053
6326	*SRL*	6345	-6.8										16	4189374	4242080
6327	*SRM*	6723									-1.6		1	11054584	11060024
6328	*SRP68*	6730	2.8										17	76038775	76072653
6329	*SRP9*	6726										-2.0	1	225777813	225790466
6330	*SRPRB*	58477	4.7										3	133784033	133825772
6331	*SRPX*	8406				4.4							X	38149336	38220924
6332	*SRRD*	402055	2.5										22	26483877	26494658
6333	*SRRM1*	10250	4.2										1	24631716	24673267
6334	*SRRM2*	23524					2.0						16	2752329	2772538
6335	*SRRM4*	84530	-3.2										12	118981495	119163051
6336	*SRRT*	51593									-2.4		7	100875111	100888664
6337	*SRSF1*	6426	3.0										17	58003360	58007346
6338	*SRSF11*	9295	6.4										1	70205682	70253052
6339	*SRSF12*	135295	-1.9										6	89095959	89118081
6340	*SRSF4*	6429	2.8										1	29147743	29181987
6341	*SRSF8*	10929		1.9									11	95067197	95071224
6342	*SRSF9*	8683	3.1										12	120461668	120469793
6343	*SSB*	6741	4.4										2	169791933	169812064
6344	*SSR1*	6745	-3.4										6	7268306	7347446
6345	*SSR4*	6748					-1.8						X	153793516	153798505
6346	*SSRP1*	6749	-2.9										11	57325985	57335877
6347	*SSSCA1*	10534	2.8										11	65570430	65573942
6348	*SSTR2*	6752	-3.9										17	73165012	73176633
6349	*SSX1*	6756			3.5								X	48255317	48267444
6350	*SSX2*	6757			3.8								X	52644567	52707189
6351	*SSX2B*	727837			3.8								X	52751132	52790305
6352	*ST20*	400410									-3.3		15	79898840	79923702
6353	*ST3GAL6*	10402										1.8	3	98732236	98821201
6354	*ST5*	6764	-2.3										11	8693351	8910951
6355	*ST6GAL1*	6480	2.9										3	186930485	187078553
6356	*ST6GALNAC3*	256435			-4.7								1	76074719	76634601
6357	*ST7*	7982		1.6									7	116953238	117230103
6358	*ST8SIA1*	6489	-4.4										12	22063773	22437041
6359	*ST8SIA4*	7903	-6.8										5	100806935	100903266
6360	*STAC*	6769	-1.6										3	36380344	36548007
6361	*STAC2*	342667	-3.1										17	39210536	39225872

6362	*STAC3*	246329	-3.1										12	57243453	57251193
6363	*STAG1*	10274	-3.0										3	136336233	136752403
6364	*STAMBPL1*	57559	-4.5										10	88879734	88975153
6365	*STBD1*	8987	2.7										4	76306026	76311599
6366	*STC1*	6781									-2.6		8	23841915	23854807
6367	*STIL*	6491	-2.8										1	47250139	47314147
6368	*STK11*	6794	-3.3										19	1177558	1228435
6369	*STK16*	8576			-2.7								2	219245455	219250337
6370	*STK17A*	9263									-1.8		7	43582758	43626786
6371	*STK25*	10494	-3.2										2	241492674	241509730
6372	*STK32A*	202374			-4.2								5	147234963	147387852
6373	*STK33*	65975	-2.1										11	8391868	8594289
6374	*STK35*	140901	-5.2										20	2101611	2177038
6375	*STK38*	11329	-3.4										6	36493892	36547470
6376	*STKLD1*	169436	-2.8										9	133376367	133406096
6377	*STMN1*	3925			2.2								1	25884181	25906991
6378	*STON1*	11037	-2.8										2	48529383	48598513
6379	*STON1-GTF2A1L*	286749	-2.1										2	48529925	48776517
6380	*STOX2*	56977	-2.0										4	183853431	184023526
6381	*STPG1*	90529	-2.4										1	24356999	24416934
6382	*STRA6*	64220	-4.9										15	74179466	74212267
6383	*STRAP*	11171	3.4										12	15882391	15903478
6384	*STRIP2*	57464	-6.5										7	129434433	129488399
6385	*STX12*	23673					-1.8						1	27773183	27824452
6386	*STX17*	55014		-1.6									9	99906633	99970341
6387	*STX1B*	112755	-3.5										16	30989256	31010628
6388	*STX2*	2054	-2.9										12	130789600	130839266
6389	*STX5*	6811				-3.8							11	62806897	62832088
6390	*STX6*	10228	-5.0										1	180972712	181023121
6391	*STX7*	8417									-1.6		6	132445867	132513198
6392	*STX8*	9482	3.0										17	9250471	9576591
6393	*STXBP3*	6814									-1.6		1	108746674	108809526
6394	*STXBP5*	134957				-2.8							6	147204425	147390476
6395	*STYX*	6815	-4.9										14	52730180	52774998
6396	*SUCLG1*	8802					-1.8						2	84423523	84460045
6397	*SULF1*	23213	-1.9										8	69466624	69660915
6398	*SULT2A1*	6822	-3.8										19	47870466	47886397
6399	*SUMO2*	6613	4.9										17	75165586	75182983
6400	*SUMO3*	6612			2.5								21	44805617	44818779
6401	*SUN3*	256979	-1.8										7	47987148	48029119
6402	*SUPT16H*	11198	3.8										14	21351472	21384266
6403	*SUPT20H*	55578			-1.8								13	37009312	37059713
6404	*SUPT7L*	9913	-3.2										2	27650812	27663840
6405	*SURF1*	6834		-1.8									9	133351755	133356676
6406	*SUSD1*	64420							1.5				9	112040785	112175408
6407	*SUSD4*	55061	-4.8										1	223220819	223364202
6408	*SUSD5*	26032	-4.3										3	33150042	33219215
6409	*SUSD6*	9766				-3.4							14	69611596	69715142
6410	*SUV39H2*	79723	-4.3										10	14878820	14904315
6411	*SUV420H1*	51111	4.1										11	68154863	68213828
6412	*SUZ12*	23512	2.9										17	31937018	32001045
6413	*SVBP*	374969	2.5										1	42807052	42817252
6414	*SVEP1*	79987	-3.0										9	110365251	110579880
6415	*SVOP*	55530	-3.2										12	108907741	109021240
6416	*SWAP70*	23075	-2.8										11	9664077	9752991
6417	*SWI5*	375757						2.1					9	128275379	128288990
6418	*SWSAP1*	126074	-4.4										19	11374685	11376951
6419	*SYBU*	55638	-2.8										8	109573978	109691791
6420	*SYCE2*	256126	-2.8										19	12898786	12919276

6421	*SYCP2L*	221711										1.8	6	10886831	10979320
6422	*SYDE1*	85360						1.9					19	15107403	15114988
6423	*SYDE2*	84144	-3.7										1	85156873	85201046
6424	*SYMPK*	8189	-4.8										19	45815410	45863290
6425	*SYNE2*	23224	-4.3										14	63852983	64226433
6426	*SYNGR2*	9144		-1.6									17	78168558	78173527
6427	*SYNJ2*	8871	-4.1										6	157981887	158099176
6428	*SYNJ2BP*	55333	-4.1										14	70366496	70417061
6429	*SYNPO*	11346	-3.0										5	150601080	150659220
6430	*SYNRG*	11276	-3.2										17	37514797	37609496
6431	*SYT15*	83849	-6.1										10	47750864	47763592
6432	*SYT16*	83851			3.6								14	61995823	62112550
6433	*SYT17*	51760	-5.7										16	19167971	19268334
6434	*SYT2*	127833	-3.3										1	202590596	202710417
6435	*SYTL1*	84958					2.1						1	27342020	27353937
6436	*SYTL3*	94120							1.5				6	158650014	158764876
6437	*SYTL5*	94122			4.6								X	38006582	38128819
6438	*TAC3*	6866		-1.6									12	57010000	57028883
6439	*TAC4*	255061	-3.2										17	49838309	49848017
6440	*TADA1*	117143	3.6										1	166856510	166876327
6441	*TAF10*	6881	-4.0										11	6606296	6612667
6442	*TAF1A*	9015	-3.2										1	222557902	222589933
6443	*TAF3*	83860	2.5										10	7818504	8016627
6444	*TAF6L*	10629	-2.6										11	62771303	62787342
6445	*TAF8*	129685	-3.8										6	42050513	42087461
6446	*TALDO1*	6888					1.6						11	747329	765024
6447	*TAMM41*	132001	-4.7										3	11790442	11846919
6448	*TANGO6*	79613	-5.2										16	68843604	69085180
6449	*TAOK2*	9344	-1.8										16	29973641	29992261
6450	*TAPBP*	6892					1.6						6	33299694	33314387
6451	*TAT*	6898	-4.8										16	71565660	71577130
6452	*TATDN1*	83940	3.0										8	124488485	124539458
6453	*TBC1D15*	64786					-1.7						12	71839707	71927248
6454	*TBC1D2*	55357					1.7						9	98198999	98255721
6455	*TBC1D22A*	25771		-1.9									22	46762617	47175699
6456	*TBC1D24*	57465	-4.2										16	2475118	2505734
6457	*TBC1D2B*	23102				-2.5							15	77984036	78077724
6458	*TBC1D3*	729873	-1.9										17	38181659	38192541
6459	*TBC1D4*	9882			-1.8								13	75284665	75482114
6460	*TBC1D8B*	54885	-3.6										8.970588	75035836	75109858.74
6461	*TBCCD1*	55171	-4.7										3	186546073	186570543
6462	*TBCK*	93627		-1.5									4	106041599	106321495
6463	*TBL1X*	6907				-1.6							X	9463295	9719743
6464	*TBPL2*	387332	-4.9										14	55413541	55456726
6465	*TBX6*	6911	-4.6										16	30085793	30091887
6466	*TC2N*	123036								-1.6			14	91779751	91867536
6467	*TCEANC*	170082	-5.7										X	13653189	13681964
6468	*TCEANC2*	127428	-1.9										1	54053587	54112519
6469	*TCERG1*	10915	3.9										5	146447311	146511961
6470	*TCERG1L*	256536	-1.8										10	131092391	131311721
6471	*TCF19*	6941	-4.6										6	31158542	31167159
6472	*TCF7*	6932	-1.7										5	134114711	134151865
6473	*TCHP*	84260	3.6										12	109900264	109983841
6474	*TCL1B*	9623	-3.3										14	95686417	95692643
6475	*TCN1*	6947	-4.1										11	59852800	59866575
6476	*TCN2*	6948	-2.9										22	30606838	30627278
6477	*TCP1*	6950			2.9								6	159778498	159789749
6478	*TCTN1*	79600	-4.4										12	110614027	110649430
6479	*TCTN3*	26123	4.1										10	95663396	95694143

6480	*TDP2*	51567									-2.0		6	24649977	24667033
6481	*TDRD12*	91646	-2.2										19	32719753	32829580
6482	*TDRD9*	122402							1.6				14	103928462	104052667
6483	*TEDDM1*	127670	-1.5										1	182398117	182400616
6484	*TEP1*	7011	-5.1										14	20365667	20413429
6485	*TERF2*	7014	-5.1										16	69355561	69408571
6486	*TESC*	54997			-2.1								12	117038923	117099479
6487	*TEX101*	83639	-5.9										19	43401496	43418597
6488	*TEX14*	56155	-1.7										17	58556678	58692055
6489	*TEX2*	55852	-5.8										17	64147227	64263301
6490	*TEX264*	51368				-2.2							3	51662693	51704323
6491	*TEX9*	374618	-1.5										15	56244009	56445997
6492	*TFAP2C*	7022			2.6								20	56629302	56639283
6493	*TFEB*	7942	-3.6										6	41683978	41736259
6494	*TFF1*	7031	-6.0										21	42362282	42366594
6495	*TFF3*	7033	-8.3										21	42311667	42315651
6496	*TFPI*	7035		2.7									2	187464230	187565760
6497	*TFPI2*	7980	-2.6										7	93885397	93890991
6498	*TFPT*	29844				-3.5							19	54107013	54115675
6499	*TFR2*	7036	-3.5										7	100620416	100642779
6500	*TGFB2*	7042	-2.3										1	218346235	218444619
6501	*TGFB3*	7043	-3.5										14	75958099	75982991
6502	*TGFBI*	7045		-2.0									5	136028895	136063818
6503	*TGFBR3*	7049	-5.7										1	91680343	91906335
6504	*TGM2*	7052	-7.1										20	38127387	38166578
6505	*TGM5*	9333	-2.0										15	43232595	43266857
6506	*THADA*	63892	-2.7										2	43230836	43596046
6507	*THAP3*	90326	3.1										1	6624866	6635586
6508	*THAP9*	79725	-3.0										4	82900684	82919969
6509	*THBD*	7056	-2.9										20	23045633	23049741
6510	*THEM5*	284486		2.0									1	151847263	151853697
6511	*THG1L*	54974			1.7								10.67675	75046801.3	75120876.31
6512	*THNSL1*	79896								-1.7			10	25016658	25026664
6513	*THNSL2*	55258	-4.1										2	88170295	88186636
6514	*THOC3*	84321		-1.6									5	175917873	176034680
6515	*THOC6*	79228			2.9								16	3024027	3027755
6516	*THRA*	7067	-2.6										17	40058290	40093867
6517	*THUMPD3*	25917	3.6										3	9362842	9386791
6518	*THY1*	7070	-1.6										11	119417378	119424985
6519	*TICAM1*	148022		-1.5									19	4815932	4831704
6520	*TIGAR*	57103	-6.8										12	4307763	4354593
6521	*TIMM10*	26519	3.8										11	57528463	57530803
6522	*TIMM13*	26517			2.1								19	2425624	2427894
6523	*TIMM17A*	10440	4.7										1	201955491	201970661
6524	*TIMM21*	29090	2.8										18	74148511	74160530
6525	*TIMM23*	100287932			2.6								10	45972449	46003734
6526	*TIMM8A*	1678			1.9								X	101345661	101349196
6527	*TIMM8B*	26521	5.0										11	112084800	112086798
6528	*TINAGL1*	64129	-2.3										1	31576485	31587686
6529	*TIPARP*	25976					-1.8						3	156673235	156706770
6530	*TLCD1*	116238			-2.2								17	28724348	28727935
6531	*TLDC1*	57707	-5.2										16	84476421	84554033
6532	*TLK1*	9874		1.6									2	170990823	171231314
6533	*TLK2*	11011	-3.0										17	62458658	62615481
6534	*TLR1*	7096							1.8				4	38790677	38856817
6535	*TLR2*	7097							1.9				4	153701500	153705699
6536	*TLR6*	10333	-5.6										4	38823715	38856817
6537	*TLR7*	51284	-3.3										X	12867083	12890380
6538	*TM4SF18*	116441			1.9								3	149318498	149334414

6539	*TM6SF1*	53346							1.6				15	83107407	83144854
6540	*TMA16*	55319	-2.4										4	163494442	163520539
6541	*TMC7*	79905	-3.6										16	18983934	19063942
6542	*TMCC1*	23023									-1.7		3	129647792	129893576
6543	*TMCO4*	255104	-4.6										1	19682213	19799945
6544	*TMED4*	222068	-4.4										7	44577894	44582287
6545	*TMEM105*	284186	-3.7										17	81311270	81330674
6546	*TMEM108*	66000	-2.5										3	133038391	133397792
6547	*TMEM116*	89894	-2.5										12	111931282	112013185
6548	*TMEM117*	84216			-1.8								12	43835967	44389762
6549	*TMEM119*	338773	-2.8										12	108589846	108598320
6550	*TMEM120A*	83862	-3.8										7	75986837	75994659
6551	*TMEM123*	114908	-3.7										11	102396332	102470384
6552	*TMEM126B*	55863					-1.6						11	85628573	85636539
6553	*TMEM130*	222865	-6.6										7	98846488	98870771
6554	*TMEM131*	23505				-2.9							2	97756333	97995891
6555	*TMEM136*	219902	-3.3										11	120325129	120333682
6556	*TMEM138*	51524	-4.0										11	61362001	61369509
6557	*TMEM143*	55260	-2.1										19	48332356	48364237
6558	*TMEM14A*	28978	-3.3										6	52671109	52686588
6559	*TMEM150C*	441027						3.1					4	82483170	82562357
6560	*TMEM156*	80008	-2.3										4	38966744	39032922
6561	*TMEM159*	57146	-2.6										16	21158377	21180616
6562	*TMEM17*	200728	-4.2										2	62500221	62511894
6563	*TMEM170A*	124491	-4.6										16	75443054	75465497
6564	*TMEM173*	340061										1.9	5	139475534	139482935
6565	*TMEM175*	84286	-3.9										4	932387	958656
6566	*TMEM176A*	55365		-2.1									7	150800403	150805120
6567	*TMEM179B*	374395	3.0										11	62787415	62790405
6568	*TMEM180*	79847	-7.0										10	102461395	102477045
6569	*TMEM181*	57583	-3.4										6	158536436	158635428
6570	*TMEM184C*	55751	-4.7										4	147617383	147672044
6571	*TMEM189-UBE2V1*	387522	2.9										20	50081124	50153637
6572	*TMEM19*	55266	-5.8										12	71686087	71705046
6573	*TMEM192*	201931	-2.8										4	165070608	165208549
6574	*TMEM203*	94107	-6.7										9	137204082	137205638
6575	*TMEM205*	374882					-1.5						19	11342776	11346518
6576	*TMEM209*	84928	-5.0										7	130164715	130207770
6577	*TMEM212*	389177	-6.4										3	171843349	171938715
6578	*TMEM213*	155006	-5.1										7	138797952	138838101
6579	*TMEM217*	221468	-1.9										6	37212180	37258155
6580	*TMEM219*	124446	-3.3										16	29940885	29973052
6581	*TMEM234*	56063						1.7					1	32214472	32222359
6582	*TMEM241*	85019						2.0					18	23197144	23437961
6583	*TMEM242*	729515	-1.7										6	157289386	157323601
6584	*TMEM248*	55069				3.6							7	66921225	66958551
6585	*TMEM254*	80195								-2.2			10	80078646	80092557
6586	*TMEM266*	123591	-5.0										15	76059837	76229121
6587	*TMEM30A*	55754	-2.9										6	75252924	75284968
6588	*TMEM33*	55161	-4.5										4	41935120	41960572
6589	*TMEM38A*	79041	-2.6										19	16661127	16690029
6590	*TMEM38B*	55151	-5.3										9	105694544	105776612
6591	*TMEM39B*	55116			-1.7								1	32072031	32102866
6592	*TMEM41A*	90407	-3.9										3	185476496	185499057
6593	*TMEM42*	131616		-1.5									3	44861869	44865670
6594	*TMEM45A*	55076	-5.5										3	100492619	100577444
6595	*TMEM47*	83604	-1.9										X	34627064	34657288
6596	*TMEM50A*	23585	-3.8										1	25337917	25362361
6597	*TMEM50B*	757	-4.4										21	33432485	33480011

6598	*TMEM51*	55092		-2.2									1	15152532	15220480
6599	*TMEM52B*	120939		-1.6									12	10170542	10191801
6600	*TMEM53*	79639	-1.9										1	44635238	44674555
6601	*TMEM55A*	55529	-3.0										8	90993796	91041064
6602	*TMEM56*	148534	-2.6										1	95117338	95197607
6603	*TMEM60*	85025	-3.0										7	77793728	77798580
6604	*TMEM64*	169200	-3.4										8	90621995	90791632
6605	*TMEM65*	157378						1.5					8	124306189	124372692
6606	*TMEM69*	51249	-4.9										1	45687214	45694443
6607	*TMEM80*	283232	3.5										11	695428	705028
6608	*TMEM87B*	84910	-3.4										2	112055223	112119318
6609	*TMEM8A*	58986	3.1										16	370773	387113
6610	*TMEM97*	27346	4.2										17	28319095	28328685
6611	*TMIE*	259236	-1.5										3	46701333	46710886
6612	*TMIGD2*	126259	-4.4										19	4292232	4302431
6613	*TMLHE*	55217	-1.6										X	155490115	155669944
6614	*TMOD2*	29767	-3.3										15	51751561	51816368
6615	*TMOD3*	29766	-3.7										15	51829628	51947295
6616	*TMPRSS11B*	132724	-3.4										4	68226653	68245720
6617	*TMPRSS13*	84000	-1.8										11	117900643	117929459
6618	*TMPRSS3*	64699	-3.8										21	42371890	42396846
6619	*TMPRSS4*	56649	-7.0										11	118077012	118121890
6620	*TMTC1*	83857	-3.2										12	29500840	29784759
6621	*TMTC4*	84899	-4.3										13	100603927	100675093
6622	*TMX1*	81542	-4.3										14	51240162	51257546
6623	*TMX2*	51075	3.0										11	57712600	57740973
6624	*TMX3*	54495					-1.6						11.97989	75008765.6	75082659.11
6625	*TNFAIP8L3*	388121	-4.4										15	51056598	51105276
6626	*TNFRSF10A*	8797	-2.1										8	23190452	23225126
6627	*TNFRSF11A*	8792	-2.1										18	62325287	62391292
6628	*TNFRSF13B*	23495	-4.8										17	16929816	16972118
6629	*TNFRSF14*	8764					1.9						1	2555639	2565382
6630	*TNFRSF19*	55504			-2.9								13	23570370	23676104
6631	*TNFRSF8*	943		-1.8									1	12063377	12144207
6632	*TNFRSF9*	3604	-5.4										1	7915894	7943165
6633	*TNFSF13*	8741									-1.9		17	7558292	7561608
6634	*TNFSF14*	8740	-2.9										19	6661253	6670588
6635	*TNFSF15*	9966	-3.9										9	114784635	114806126
6636	*TNFSF4*	7292	-2.4										1	173183734	173207313
6637	*TNFSF8*	944	-3.0										9	114893343	114930595
6638	*TNIK*	23043	3.4										3	171061339	171460408
6639	*TNIP3*	79931	-1.9										4	121131408	121227466
6640	*TNKS*	8658	-5.4										8	9555914	9782346
6641	*TNN*	63923	-2.0										1	175067858	175148066
6642	*TNNC2*	7125		2.3									20	45823214	45833745
6643	*TNNT1*	7138	-1.8										19	55132794	55149354
6644	*TNNT2*	7139	-3.0										1	201359008	201377762
6645	*TNPO1*	3842					-1.7						5	72816312	72916733
6646	*TNRC6B*	23112	-3.2										22	40044817	40335808
6647	*TNS4*	84951	-4.3										17	40475828	40501597
6648	*TOMM40L*	84134						1.7					1	161225939	161230744
6649	*TOMM7*	54543		-1.5									12.84816	75013905.5	75087823.59
6650	*TOP1*	7150	4.5										20	41028818	41124487
6651	*TOP2B*	7155	4.8										3	25597905	25664907
6652	*TOPBP1*	11073				-1.6							3	133598175	133661893
6653	*TOR1A*	1861	-3.4										9	129812944	129824134
6654	*TOR1AIP2*	163590	-3.7										1	179839967	179877803
6655	*TOX3*	27324	-2.7										16	52438005	52547802
6656	*TP53*	7157	-3.0										17	7661779	7687550

6657	*TP53BP1*	7158	3.7										15	43403061	43510728
6658	*TP53BP2*	7159	-2.9										1	223779899	223845972
6659	*TP53I3*	9540	-5.6										2	24077433	24085861
6660	*TP53INP2*	58476	2.5										20	34704290	34713439
6661	*TPI1*	7167							-1.7				12	6867119	6870948
6662	*TPMT*	7172	-4.4										6	18128311	18155074
6663	*TPRN*	286262		-1.8									9	137191617	137204193
6664	*TPST2*	8459		1.8									22	26521983	26596717
6665	*TPT1*	7178			-1.9								13	45333471	45341370
6666	*TPTE*	7179		1.6									21	10521553	10606140
6667	*TRA2A*	29896			5.1								7	23504780	23532041
6668	*TRABD2A*	129293	-1.9										2	84821650	84907008
6669	*TRAF3IP1*	26146	-5.2										2	238320441	238400900
6670	*TRAF4*	9618		-2.0									17	28743984	28750958
6671	*TRAF5*	7188	-3.4										1	211326615	211374946
6672	*TRAF7*	84231						1.6					16	2155698	2178129
6673	*TRAM1*	23471	2.9										8	70573442	70608387
6674	*TRAPPC1*	58485		1.8									17	7930345	7932123
6675	*TRAPPC2*	6399	-3.8										X	13712244	13734635
6676	*TRAPPC2L*	51693		-1.6									16	88856220	88862686
6677	*TRAPPC5*	126003				-3.7							19	7680843	7687703
6678	*TRAPPC6B*	122553	3.8										14	39147811	39170532
6679	*TRIAP1*	51499					-1.9						12	120443961	120446412
6680	*TRIB1*	10221	3.8										8	125430321	125438405
6681	*TRIM11*	81559			-1.6								1	228393673	228406840
6682	*TRIM14*	9830						-1.6					9	98069275	98119212
6683	*TRIM16*	10626	-2.5										17	15627960	15684311
6684	*TRIM23*	373	-1.6										5	65589680	65625975
6685	*TRIM26*	7726			-1.8								6	30184455	30213427
6686	*TRIM28*	10155	3.1										19	58544091	58550722
6687	*TRIM29*	23650	-6.9										11	120111275	120185529
6688	*TRIM3*	10612	-6.2										11	6448613	6474459
6689	*TRIM31*	11074									-1.6		6	30102897	30113106
6690	*TRIM32*	22954			-1.8								9	116687302	116701300
6691	*TRIM39*	56658	-4.6										6	30326479	30343729
6692	*TRIM45*	80263	-2.3										1	117111060	117122587
6693	*TRIM5*	85363			1.6								11	5663557	5938619
6694	*TRIM51*	84767		-1.6									11	55883297	55891810
6695	*TRIM59*	286827	-2.6										3	160432445	160485773
6696	*TRIM6*	117854			1.6								11	5596109	5612958
6697	*TRIM60*	166655	-4.3										4	165031953	165041744
6698	*TRIM63*	84676			-3.5								1	26051304	26068436
6699	*TRIM65*	201292	-2.6										17	75880335	75897003
6700	*TRIM66*	9866	-4.4										11	8612037	8671866
6701	*TRIM68*	55128	-3.8										11	4598672	4608259
6702	*TRIM6-TRIM34*	445372	-3.8										11	5596725	5644398
6703	*TRIM72*	493829	-4.6										16	31214021	31231537
6704	*TRIOBP*	11078	-6.2										22	37697004	37776556
6705	*TRIP11*	9321	-2.7										14	91965991	92040896
6706	*TRIP12*	9320	5.1										2	229763838	229923239
6707	*TRIP4*	9325								-1.8			15	64387748	64455303
6708	*TRMT10B*	158234	-6.5										9	37753803	37778972
6709	*TRMT1L*	81627	-2.5										1	185118098	185157072
6710	*TRMT2B*	79979	-5.8										X	101009346	101052116
6711	*TRMT44*	152992									-1.6		4	8436140	8493531
6712	*TRNAU1AP*	54952	-4.3										X	75043717.3	75117777.62
6713	*TRPA1*	8989			-2.8								8	72019917	72075617
6714	*TRPC6*	7225	-2.9										11	101451564	101872562
6715	*TRPM1*	4308			-4.0								15	31001061	31161273

6716	*TRPM3*	80036	-6.4										9	70529063	71446904
6717	*TRPM4*	54795	-4.1										3.494709	75024870.8	75098841.17
6718	*TRPM6*	140803	-4.1										9	74722495	74888094
6719	*TRPM7*	54822	-6.4										1.708995	75027954.8	75101939.86
6720	*TRPM8*	79054	-5.7										2	233917398	234019522
6721	*TRPV1*	7442	-5.1										17	3565444	3609411
6722	*TRPV2*	51393		-1.6									17	16415542	16437003
6723	*TSC22D3*	1831									-2.7		X	107713221	107777342
6724	*TSKU*	25987	-2.8										11	76782251	76798154
6725	*TSNAX*	7257	-3.7										1	231528653	231566524
6726	*TSNAXIP1*	55815	-2.6										16	67806765	67832148
6727	*TSPAN16*	26526	-1.9										19	11296139	11326996
6728	*TSPAN31*	6302	-4.1										12	57738013	57750211
6729	*TSPAN4*	7106	-3.0										11	842808	867116
6730	*TSPAN5*	10098	3.4										4	98470367	98658629
6731	*TSPAN8*	7103	3.5										12	71125085	71441898
6732	*TSPYL4*	23270					-1.5						6	116249961	116254140
6733	*TSTA3*	7264							-1.7				8	143612618	143618048
6734	*TSTD1*	100131187	5.5										1	161037631	161038990
6735	*TTC1*	7265				4.3							5	160009113	160065543
6736	*TTC17*	55761		-1.5									11	43358932	43494933
6737	*TTC19*	54902					-1.6						6.119118	75038234.7	75112268.84
6738	*TTC21B*	79809	-5.1										2	165857475	165953843
6739	*TTC23*	64927	-4.4										15	99136323	99251223
6740	*TTC28*	23331	-3.1										22	27978014	28679865
6741	*TTC33*	23548	-3.7										5	40714475	40755975
6742	*TTC37*	9652		-1.5									5	95463895	95555007
6743	*TTC39B*	158219	-6.5										9	15163622	15307360
6744	*TTC39C*	125488	-4.8										18	23992773	24135610
6745	*TTK*	7272			4.3								6	80003887	80042527
6746	*TTLL1*	25809	-3.6										22	43039644	43089428
6747	*TTLL3*	26140	-5.1										3	9808086	9855138
6748	*TTLL5*	23093	-3.8										14	75633625	75955078
6749	*TTLL9*	164395	-3.1										20	31870702	31944963
6750	*TTN*	7273	-3.7										2	178525989	178830802
6751	*TTPAL*	79183	-6.1										20	44475886	44494603
6752	*TUBA1B*	10376			1.6								12	49127782	49131397
6753	*TUBA8*	51807		3.2									22	18110331	18146554
6754	*TUBB*	203068								1.6			6	30720201	30725426
6755	*TUBB3*	10381						1.5					16	89921392	89938761
6756	*TUBB4B*	10383			1.6								9	137241213	137243707
6757	*TUBD1*	51174	-3.8										17	59859482	59892945
6758	*TUBG1*	7283			2.4								17	42609676	42615234
6759	*TUBGCP5*	114791	-1.8										15	22983192	23039673
6760	*TUBGCP6*	85378	-4.2										22	50217689	50244992
6761	*TULP2*	7288	-2.4										19	48880965	48898733
6762	*TULP4*	56995	-2.9										6	158232236	158511828
6763	*TUSC1*	286319			2.5								9	25676389	25678440
6764	*TVP23C*	201158	-4.0										17	15502264	15563595
6765	*TWF2*	11344					1.5						3	52228610	52239260
6766	*TWIST1*	7291			2.9								7	19020991	19117672
6767	*TWISTNB*	221830	3.8										7	19695462	19709087
6768	*TXLNA*	200081			-2.2								1	32179686	32198285
6769	*TXNDC11*	51061	3.0										16	11679080	11742878
6770	*TXNDC16*	57544	2.7										14	52430590	52552522
6771	*TXNDC17*	84817							-1.5				17	6640758	6644541
6772	*TXNL4B*	54957			-2.1								19	75044060	75118121.92
6773	*TXNRD1*	7296							-1.5				12	104215779	104350305
6774	*TXNRD2*	10587			1.7								22	19875517	19941992

6775	*TYK2*	7297									-1.9		19	10350529	10380676
6776	*TYMSOS*	494514			4.3								18	641320	658340
6777	*TYW1B*	441250		-1.7									7	72558744	72828198
6778	*UBA3*	9039	2.6										3	69054730	69080408
6779	*UBA5*	79876	-4.8										3	132654446	132678097
6780	*UBAC1*	10422								-2.0			9	135932969	135961380
6781	*UBAC2*	337867			-3.0								13	99200774	99386434
6782	*UBASH3B*	84959		1.6									11	122655675	122814473
6783	*UBE2A*	7319	3.7										X	119574467	119591083
6784	*UBE2B*	7320									-2.7		5	134371179	134391992
6785	*UBE2D2*	7322							-1.8				5	139526431	139628433
6786	*UBE2E3*	10477	4.4										2	180967248	181076585
6787	*UBE2H*	7328	2.9										7	129830732	129952949
6788	*UBE2S*	27338	3.2										19	55399745	55407777
6789	*UBE2V1*	7335	3.6										20	50081124	50115959
6790	*UBE2W*	55284	-4.9										8	73780097	73878910
6791	*UBE3C*	9690	-3.8										7	157138913	157269372
6792	*UBE3D*	90025	-4.2										6	82892398	83065841
6793	*UBE4B*	10277	-3.3										1	10032832	10181239
6794	*UBFD1*	56061	-6.6										16	23557362	23574389
6795	*UBL7*	84993			2.6								15	74445977	74461182
6796	*UBN2*	254048	-5.6										7	139230356	139308236
6797	*UBOX5*	22888	-4.9										20	3107573	3160196
6798	*UBP1*	7342	3.9										3	33388336	33441371
6799	*UBQLN4*	56893						1.6					1	156035301	156053794
6800	*UBR7*	55148			2.3								14	93207056	93229215
6801	*UBTD2*	92181						1.7					5	172209640	172284071
6802	*UBXN1*	51035		-1.6									11	62676498	62679117
6803	*UBXN2A*	165324	-6.1										2	23927285	24004909
6804	*UBXN4*	23190	-4.2										2	135741619	135785055
6805	*UBXN7*	26043	-3.8										3	196347662	196432474
6806	*UCHL5*	51377	-6.3										1	193012250	193060080
6807	*UEVLD*	55293	-2.3										11	18529609	18588747
6808	*UFD1L*	7353	-3.0										22	19449910	19479215
6809	*UFM1*	51569	-2.7										13	38349849	38363619
6810	*UGGT1*	56886	-4.8										2	128091200	128195677
6811	*UGT2B4*	7363	-5.6										4	69480165	69526014
6812	*UGT8*	7368	-2.5										4	114598455	114678224
6813	*UIMC1*	51720	-3.3										5	176905005	177022633
6814	*ULBP3*	79465	-4.7										6	150063150	150069095
6815	*ULK2*	9706	-3.6										17	19770829	19867936
6816	*ULK4*	54986			2.6								3	41246599	41962430
6817	*UMPS*	7372	-4.6										3	124730366	124749273
6818	*UNC119*	9094		-1.5									17	28546707	28552668
6819	*UNC13A*	23025	-5.1										19	17601328	17688365
6820	*UNC13B*	10497	5.0										9	35161992	35405338
6821	*UNC13D*	201294	-5.0										17	75827225	75844717
6822	*UNC45A*	55898			-1.8								15	90930180	90954093
6823	*UNC79*	57578	-2.7										14	93333219	93707876
6824	*UNC80*	285175	-4.7										2	209771993	209999300
6825	*UNC93B1*	81622		-1.7									11	67991104	68004982
6826	*UNK*	85451			-3.2								17	75784771	75825799
6827	*UPB1*	51733	-2.7										22	24494107	24528390
6828	*UPF2*	26019			1.7								10	11920022	12043170
6829	*UPK1B*	7348	-3.2										3	119173517	119205153
6830	*UPK3BL*	100134938	-3.1										7	102637025	102642791
6831	*UQCRC2*	7385					-1.6						16	21952660	21983660
6832	*URGCP*	55665			1.6								7	43875894	43926411
6833	*URGCP-MRPS24*	100534592			1.6								7	43866558	43906589

6834	*URI1*	8725	-2.9										19	29923644	30016608
6835	*USB1*	79650					1.5						16	57999546	58021618
6836	*USE1*	55850	-6.2										19	17215346	17219829
6837	*USH2A*	7399	-2.6										1	215622894	216423396
6838	*USHBP1*	83878	-4.1										19	17249176	17282786
6839	*USP1*	7398	4.7										1	62436297	62451804
6840	*USP10*	9100		-1.7									16	84699978	84779922
6841	*USP12*	219333						1.9					13	27066142	27171896
6842	*USP13*	8975	-3.3										3	179652755	179789401
6843	*USP14*	9097							-1.7				18	158383	214629
6844	*USP16*	10600					-1.5						21	29024629	29054488
6845	*USP29*	57663	-2.3										19	57119138	57131926
6846	*USP31*	57478						2.6					16	23061406	23149270
6847	*USP33*	23032	-3.1										1	77695987	77759852
6848	*USP36*	57602			-1.6								17	78787381	78841441
6849	*USP39*	10713	-3.6										2	85602856	85649282
6850	*USP40*	55230			-2.8								2	233475520	233566782
6851	*USP42*	84132	-3.4										7	6104884	6161564
6852	*USP47*	55031	-2.6										11	11841423	11959323
6853	*USP48*	84196	4.2										1	21678298	21783606
6854	*USP5*	8078	-2.9										12	6852128	6866632
6855	*USP50*	373509	-3.0										15	50500562	50546708
6856	*USP53*	54532	-2.4										12.50085	75011849.5	75085757.8
6857	*USP8*	9101	3.1										15	50424380	50514419
6858	*UTP14A*	10813							-1.5				X	129906121	129929761
6859	*UTP14C*	9724	-3.7										13	52024691	52033600
6860	*UTP15*	84135								-1.6			5	73565443	73583377
6861	*UTP20*	27340	-4.6										12	101280109	101386616
6862	*UTP6*	55813		-1.9									17	31860899	31901765
6863	*UTS2*	10911	-2.1										1	7843083	7853512
6864	*UTY*	7404	-2.9										Y	13248379	13480673
6865	*UXT*	8409	-3.2										X	47651798	47659161
6866	*VAC14*	55697	-3.1										16	70687439	70801161
6867	*VAMP1*	6843	4.2										12	6462237	6470987
6868	*VAMP8*	8673			-6.3								2	85561562	85582031
6869	*VAPB*	9217	4.0										20	58389122	58451101
6870	*VARS*	7407	3.1										6	31777518	31795953
6871	*VASP*	7408					2.0						19	45506579	45526983
6872	*VAV3*	10451							2.1				1	107571160	107965144
6873	*VDAC2*	7417	2.0										10	75210154	75231448
6874	*VDAC3*	7419			2.0								8	42391624	42405897
6875	*VENTX*	27287	-5.0										10	133237404	133241929
6876	*VEZF1*	7716	2.8										17	57971547	57988259
6877	*VEZT*	55591							-1.6				12	95217746	95302790
6878	*VGF*	7425			-4.8								7	101162509	101165593
6879	*VGLL3*	389136	-3.3										3	86937969	86991119
6880	*VHL*	7428	-3.5										3	10141008	10152220
6881	*VIL1*	7429						2.4					2	218419092	218453295
6882	*VIPR1*	7433	-4.8										3	42489299	42537573
6883	*VIPR2*	7434	-4.4										7	159028175	159144957
6884	*VKORC1*	79001	-3.4										16	31090842	31095980
6885	*VKORC1L1*	154807			-1.5								7	65873267	65959563
6886	*VMA21*	203547	-3.2										X	151396515	151409364
6887	*VMAC*	400673	-2.2										19	5904858	5910853
6888	*VNN1*	8876	-1.9										6	132681590	132714049
6889	*VPS11*	55823			-2.3								11	119067692	119081978
6890	*VPS13A*	23230	3.8										9	77177353	77421541
6891	*VPS13B*	157680				-2.5							8	99013266	99877580
6892	*VPS28*	51160	4.6										8	144423601	144428563

6893	*VPS33A*	65082	-3.1										12	122229564	122266521
6894	*VPS37A*	137492		1.9									8	17246571	17302427
6895	*VPS37B*	79720					2.0						12	122865328	122896444
6896	*VPS37C*	55048	4.0										11	61130256	61161617
6897	*VPS41*	27072	-3.3										7	38722963	38932394
6898	*VPS53*	55275	-4.5										17	508668	721717
6899	*VRK1*	7443	-3.0										14	96797304	96931722
6900	*VRK2*	7444	-3.1										2	57907651	58159920
6901	*VRK3*	51231	-3.6										19	49976467	50025946
6902	*VSIG1*	340547	-3.0										X	108044970	108079184
6903	*VSIG2*	23584	-3.8										11	124747472	124752238
6904	*VSTM4*	196740	-5.9										10	49014245	49115509
6905	*VTI1B*	10490										-1.6	14	67647075	67674831
6906	*VWA1*	64856	-4.5										1	1434861	1442882
6907	*VWA5A*	4013	-2.9										11	124115362	124147721
6908	*VWF*	7450	-4.1										12	5948874	6124770
6909	*WAS*	7454							1.6				X	48676596	48691427
6910	*WASL*	8976	3.1										7	123681935	123749067
6911	*WBP1*	23559	-5.1										2	74458329	74460891
6912	*WDFY3*	23001									-1.7		4	84669610	84966391
6913	*WDHD1*	11169	-4.5										14	54938950	55027105
6914	*WDR13*	64743	-5.7										X	48590042	48608867
6915	*WDR17*	116966	-3.6										4	176065834	176182818
6916	*WDR19*	57728	-2.6										4	39182404	39285810
6917	*WDR33*	55339	-4.0										2	127701022	127811187
6918	*WDR41*	55255	2.7										5	77425970	77620611
6919	*WDR45B*	56270	5.1										17	82614562	82648553
6920	*WDR55*	54853	-2.7										7.236364	75031038.7	75105038.55
6921	*WDR5B*	54554	-2.4										13.13758	75015618.9	75089545.09
6922	*WDR6*	11180		-1.7									3	49007062	49015953
6923	*WDR62*	284403	-4.3										19	36054881	36105106
6924	*WDR76*	79968	-4.5										15	43826963	43868419
6925	*WDR78*	79819	-2.6										1	66812885	66924887
6926	*WDR82*	80335		-1.8									3	52254421	52288020
6927	*WDR88*	126248	-1.5										19	33132090	33175795
6928	*WDR92*	116143	-5.2										2	68122936	68157560
6929	*WDYHV1*	55093	-2.8										8	123416725	123467230
6930	*WEE1*	7465	3.0										11	9573681	9593457
6931	*WFDC3*	140686	-2.8										20	45747944	45791932
6932	*WFDC8*	90199	-1.7										20	45551153	45579326
6933	*WHSC1*	7468	-3.3										4	1871424	1982207
6934	*WIPF3*	644150		-1.6									7	29806486	29917066
6935	*WISP1*	8840	-2.1										8	133191039	133230344
6936	*WNK2*	65268	3.2										9	93184916	93320572
6937	*WNK4*	65266	-5.9										17	42780678	42796936
6938	*WNT7B*	7477	-2.6										22	45920362	45977129
6939	*WNT9B*	7484	-5.2										17	46833201	46886730
6940	*WRN*	7486	-8.5										8	31033801	31173769
6941	*WRNIP1*	56897	-4.8										6	2765414	2786952
6942	*WWTR1*	25937	-4.3										3	149517235	149736714
6943	*XIAP*	331							-1.7				X	123859724	123913979
6944	*XIRP2*	129446	-4.6										2	166888487	167259753
6945	*XKR5*	389610	-2.5										8	6808517	6835644
6946	*XKR9*	389668	-3.8										8	70669365	70790371
6947	*XPO1*	7514							-1.6				2	61477849	61538626
6948	*XPOT*	11260			-2.7								12	64404350	64451127
6949	*XPR1*	9213	-4.9										1	180632004	180890251
6950	*XRCC1*	7515	2.6										19	43543040	43580473
6951	*XRRA1*	143570	4.3										11	74807739	74949200

6952	*YAE1D1*	57002	-3.5										7	39566376	39610320
6953	*YAF2*	10138	-3.1										12	42157104	42238349
6954	*YAP1*	10413	-5.5										11	102110461	102233423
6955	*YARS*	8565			-2.9								1	32775237	32818153
6956	*YBX1*	4904			-2.1								1	42682427	42702349
6957	*YES1*	7525	-3.3										18	721588	812546
6958	*YIF1A*	10897							-1.7				11	66284580	66289170
6959	*YIPF1*	54432	-3.3										10.99586	75002940.3	75076806.02
6960	*YKT6*	10652	-3.1										7	44200968	44214294
6961	*YPEL1*	29799		-1.6									22	21697544	21735834
6962	*YPEL2*	388403							1.7				17	59331689	59401729
6963	*YRDC*	79693	-4.3										1	37802944	37808185
6964	*YTHDC1*	91746									-1.9		4	68310387	68350089
6965	*YWHAG*	7532	5.0										7	76326794	76359031
6966	*YY1*	7528	4.5										14	100238298	100282792
6967	*ZADH2*	284273	-5.2										18	75195108	75209348
6968	*ZAK*	51776	-5.0										2	173075435	173268010
6969	*ZAN*	7455	-3.6										7	100733626	100797797
6970	*ZAP70*	7535	2.6										2	97713560	97739862
6971	*ZBED4*	9889			-1.6								22	49853842	49890078
6972	*ZBP1*	81030	-3.1										20	57603846	57620576
6973	*ZBTB1*	22890			-1.7								14	64503712	64533690
6974	*ZBTB2*	57621	-2.0										6	151364117	151391548
6975	*ZBTB20*	26137	-2.3										3	114338094	115147271
6976	*ZBTB21*	49854		-1.5									21	41986831	42010387
6977	*ZBTB3*	79842	-6.7										11	62748319	62754188
6978	*ZBTB33*	10009								-1.7			X	120250752	120258398
6979	*ZBTB34*	403341							1.6				9	126860665	126885878
6980	*ZBTB4*	57659								-1.9			17	7459366	7484263
6981	*ZBTB47*	92999	-3.9										3	42653684	42665854
6982	*ZBTB8A*	653121	-5.4										1	32539427	32605939
6983	*ZBTB8OS*	339487	-5.9										1	32600172	32650903
6984	*ZC3H12A*	80149	2.6										1	37474552	37484379
6985	*ZC3H12B*	340554	-4.3										X	65366638	65507887
6986	*ZC3H13*	23091	3.7										13	45954465	46052759
6987	*ZC3H15*	55854					-1.5						2	186486156	186509363
6988	*ZC3H6*	376940	-5.4										2	112275594	112340063
6989	*ZC3H7B*	23264	-5.6										22	41301522	41360147
6990	*ZC3HAV1L*	92092						2.8					7	139025706	139036029
6991	*ZC3HC1*	51530	-2.5										7	130018286	130051451
6992	*ZCCHC11*	23318	-4.2										1	52408282	52553487
6993	*ZCCHC24*	219654	-8.1										10	79382325	79445627
6994	*ZCCHC4*	29063	-2.1										4	25312785	25370383
6995	*ZCCHC7*	84186		-2.3									9	37120539	37358149
6996	*ZCCHC8*	55596	-6.5										12	122471600	122501073
6997	*ZCWPW1*	55063	-2.7										7	100400826	100428992
6998	*ZDHHC12*	84885				-4.2							9	128720869	128724127
6999	*ZDHHC15*	158866	-5.0										X	75368427	75523502
7000	*ZDHHC16*	84287	3.8										10	97446131	97457370
7001	*ZDHHC2*	51201					-1.7						8	17156029	17224799
7002	*ZDHHC3*	51304								-1.8			3	44915257	44976185
7003	*ZDHHC5*	25921		-1.8									11	57667747	57701187
7004	*ZDHHC7*	55625		-1.6									16	84974181	85011535
7005	*ZDHHC9*	51114	4.0										X	129803288	129843909
7006	*ZFAND4*	93550	-4.8										10	45615501	45672780
7007	*ZFAND6*	54469	-3.6										11.51682	75006024.2	75079904.71
7008	*ZFHX4*	79776			-1.5								8	76681219	76867285
7009	*ZFP14*	57677	-5.1										19	36334453	36379199
7010	*ZFP2*	80108	-7.0										5	178895894	178933212

7011	*ZFP30*	22835	-2.3										19	37613749	37692337
7012	*ZFP42*	132625	-3.1										4	187995771	188005050
7013	*ZFR*	51663				1.5							5	32354350	32444761
7014	*ZFR2*	23217	-2.1										19	3804024	3869032
7015	*ZFY*	7544	-4.9										Y	2935281	2982506
7016	*ZFYVE16*	9765	-4.0										5	80408013	80479350
7017	*ZFYVE28*	57732	-2.3										4	2269582	2418663
7018	*ZG16*	653808	-2.5										16	29778240	29782973
7019	*ZG16B*	124220	3.2										16	2830169	2839585
7020	*ZGRF1*	55345	-2.6										4	112539333	112636995
7021	*ZHX1*	11244				4.3							8	123248451	123275541
7022	*ZHX1-C8orf76*	100533106				4.3							8	123226189	123274284
7023	*ZHX2*	22882									-1.8		8	122781394	122974512
7024	*ZHX3*	23051	-4.1										20	41178448	41317672
7025	*ZIC2*	7546										2.0	13	99981772	99986773
7026	*ZIM3*	114026	-3.0										19	57134096	57145202
7027	*ZKSCAN7*	55888	-2.8										3	44555193	44594173
7028	*ZMAT2*	153527	3.3										5	140698680	140706676
7029	*ZMAT3*	64393	-4.2										3	179017223	179072279
7030	*ZMIZ1*	57178	-4.0										10	79069035	79316528
7031	*ZMYM1*	79830	-4.3										1	35059786	35115859
7032	*ZMYM3*	9203									-2.1		X	71239624	71255146
7033	*ZNF101*	94039	-7.9										19	19668796	19683509
7034	*ZNF112*	7771	-5.0										19	44326555	44367217
7035	*ZNF114*	163071	-5.1										19	48172318	48287608
7036	*ZNF124*	7678	-2.4										1	247121975	247172016
7037	*ZNF136*	7695	-3.6										19	12163064	12189881
7038	*ZNF141*	7700	-2.7										4	337814	384864
7039	*ZNF160*	90338	-5.1										19	53066606	53103436
7040	*ZNF169*	169841	-3.3										9	94259311	94301454
7041	*ZNF177*	7730	-5.2										19	9363020	9382617
7042	*ZNF184*	7738			-2.3								6	27450743	27473118
7043	*ZNF19*	7567	-5.0										16	71464555	71565089
7044	*ZNF197*	10168	-3.8										3	44584888	44648471
7045	*ZNF2*	7549	-2.8										2	95165432	95184317
7046	*ZNF208*	7757	-2.2										19	21932958	22010949
7047	*ZNF212*	7988	-2.2										7	149239651	149255609
7048	*ZNF213*	7760	-4.7										16	3129777	3142805
7049	*ZNF215*	7762	-2.2										11	6926404	6984632
7050	*ZNF217*	7764									-1.8		20	53567065	53609907
7051	*ZNF219*	51222	-2.0										14	21090046	21104722
7052	*ZNF22*	7570			-1.7								10	45000475	45005326
7053	*ZNF233*	353355	-2.4										19	44259880	44275317
7054	*ZNF234*	10780	-4.3										19	44141557	44160309
7055	*ZNF235*	9310	-3.4										19	44228729	44305046
7056	*ZNF24*	7572					-1.6						18	35332212	35345482
7057	*ZNF250*	58500	-4.7										8	144876497	144902168
7058	*ZNF253*	56242	-2.2										19	19865886	19894674
7059	*ZNF257*	113835	-5.3										19	22052452	22091480
7060	*ZNF260*	339324	-6.8										19	36510695	36528660
7061	*ZNF263*	10127	4.1										16	3263800	3301401
7062	*ZNF264*	9422	-4.8										19	57191500	57222846
7063	*ZNF267*	10308					-1.5						16	31873758	31917357
7064	*ZNF268*	10795	3.4										12	133181409	133214831
7065	*ZNF273*	10793	-5.7										7	64870172	64930966
7066	*ZNF277*	11179	-3.2										7	112206588	112343096
7067	*ZNF283*	284349	-2.9										19	43827292	43852017
7068	*ZNF292*	23036	-3.3										6	87152833	87264196
7069	*ZNF302*	55900	-5.0										19	34677639	34686397

7070	*ZNF304*	57343	-3.1										19	57351307	57359898
7071	*ZNF320*	162967	-3.0										19	52863790	52897693
7072	*ZNF329*	79673	-4.4										19	58126252	58155110
7073	*ZNF33B*	7582	-4.6										10	42574185	42638568
7074	*ZNF341*	84905	-5.4										20	33731657	33792269
7075	*ZNF346*	23567	-4.3										5	177022696	177081189
7076	*ZNF354B*	117608	-1.8										5	178859953	178888122
7077	*ZNF358*	140467				-3.0							19	7515292	7521026
7078	*ZNF362*	149076			-2.2								1	33256545	33300719
7079	*ZNF365*	22891		-1.7									10	62374192	62672011
7080	*ZNF37A*	7587		-1.8									10	38094336	38125544
7081	*ZNF383*	163087	-7.4										19	37217926	37248738
7082	*ZNF391*	346157	-1.8										6	27374615	27403904
7083	*ZNF397*	84307	-4.7										18	35241030	35267133
7084	*ZNF398*	57541	-4.2										7	149126416	149182802
7085	*ZNF416*	55659		-1.7									19	57571566	57578927
7086	*ZNF417*	147687	-2.5										19	57900296	57916610
7087	*ZNF418*	147686	-1.9										19	57921884	57935393
7088	*ZNF420*	147923	-1.8										19	37007857	37130314
7089	*ZNF429*	353088	-5.1										19	21496682	21556270
7090	*ZNF43*	7594	-3.8										19	21804949	21852125
7091	*ZNF436*	80818								-1.5			1	23359448	23369442
7092	*ZNF460*	10794		-1.7									19	57280051	57293569
7093	*ZNF462*	58499		-1.7									9	106863097	107013634
7094	*ZNF470*	388566	-1.7										19	56567511	56588911
7095	*ZNF471*	57573	-4.5										19	56507843	56530221
7096	*ZNF473*	25888	2.6										19	50025714	50053414
7097	*ZNF483*	158399	-6.8										9	111525159	111577844
7098	*ZNF490*	57474	-4.8										19	12577961	12640098
7099	*ZNF492*	57615	-4.9										19	22634324	22667670
7100	*ZNF493*	284443	-5.7										19	21397119	21427573
7101	*ZNF496*	84838	-2.6										1	247297412	247331846
7102	*ZNF497*	162968				-3.5							19	58354357	58362848
7103	*ZNF503*	84858									-1.6		10	75397830	75401906
7104	*ZNF506*	440515	-4.8										19	19785839	19821751
7105	*ZNF517*	340385	-2.1										8	144798876	144811169
7106	*ZNF519*	162655	-2.6										18	14057457	14132490
7107	*ZNF527*	84503	-5.1										19	37371061	37393066
7108	*ZNF528*	84436	-2.1										19	52397849	52418412
7109	*ZNF529*	57711	-4.8										19	36534774	36605276
7110	*ZNF536*	9745	-4.4										19	30228290	30713538
7111	*ZNF540*	163255	-2.7										19	37551406	37614097
7112	*ZNF541*	84215	-1.6										19	47520685	47555856
7113	*ZNF543*	125919	-5.4										19	57320509	57330776
7114	*ZNF546*	339327		-1.5									19	39984134	40021041
7115	*ZNF554*	115196	-4.2										19	2819874	2835773
7116	*ZNF555*	148254	-4.1										19	2841435	2860484
7117	*ZNF557*	79230	-3.4										19	7069444	7087968
7118	*ZNF561*	93134	-4.2										19	9604680	9621399
7119	*ZNF563*	147837	-2.5										19	12317477	12333720
7120	*ZNF565*	147929	-2.2										19	36182060	36246257
7121	*ZNF568*	374900	-6.6										19	36916329	36998700
7122	*ZNF573*	126231	-6.8										19	37735833	37817300
7123	*ZNF578*	147660	-2.5										19	52453576	52512154
7124	*ZNF583*	147949		-1.5									19	56397966	56436035
7125	*ZNF585B*	92285	-3.6										19	37181579	37218153
7126	*ZNF587*	84914	-4.6										19	57849857	57865112
7127	*ZNF592*	9640	2.8										15	84748635	84806432
7128	*ZNF593*	51042						2.1					1	26169871	26172060

7129	*ZNF596*	169270		-1.5									8	232137	247342
7130	*ZNF606*	80095	-2.8										19	57977053	58003349
7131	*ZNF607*	84775	-4.2										19	37696363	37719790
7132	*ZNF609*	23060	-3.8										15	64460742	64686068
7133	*ZNF611*	81856	-2.7										19	52702813	52735073
7134	*ZNF616*	90317	-6.8										19	52113091	52139922
7135	*ZNF619*	285267	-2.1										3	40477113	40490236
7136	*ZNF621*	285268	-3.6										3	40524878	40574685
7137	*ZNF622*	90441	-3.7										5	16451519	16465792
7138	*ZNF629*	23361	-2.2										16	30778449	30787202
7139	*ZNF639*	51193	-3.8										3	179322991	179338583
7140	*ZNF644*	84146	3.7										1	90915298	91022272
7141	*ZNF652*	22834	-3.8										17	49289206	49362473
7142	*ZNF662*	389114	-7.5										3	42905731	42917641
7143	*ZNF665*	79788	-5.2										19	53159213	53193386
7144	*ZNF668*	79759	-1.6										16	31060843	31074320
7145	*ZNF669*	79862	-4.5										1	247099962	247104372
7146	*ZNF672*	79894	-2.9										1	248838210	248849517
7147	*ZNF681*	148213	-4.3										19	23739195	23758891
7148	*ZNF69*	7620	-2.8										19	11887784	11914329
7149	*ZNF691*	51058	-2.7										1	42846573	42852477
7150	*ZNF695*	57116	-3.2										1	246945547	247008093
7151	*ZNF7*	7553	-4.8										8	144827464	144847509
7152	*ZNF70*	7621	-2.1										22	23738678	23751092
7153	*ZNF700*	90592	-2.6										19	11925068	11950773
7154	*ZNF707*	286075	-2.9										8	143684452	143713898
7155	*ZNF713*	349075	-3.6										7	55887475	55942225
7156	*ZNF714*	148206	-5.8										19	21082159	21125270
7157	*ZNF716*	441234	-4.1										7	57450177	57473546
7158	*ZNF718*	255403	-2.1										4	124480	163989
7159	*ZNF721*	170960	-3.9										4	425815	499156
7160	*ZNF737*	100129842	-5.2										19	20535825	20565809
7161	*ZNF746*	155061									-1.6		7	149472794	149497817
7162	*ZNF747*	65988	-5.2										16	30530367	30535347
7163	*ZNF75D*	7626		2.2									X	135248920	135344087
7164	*ZNF763*	284390	-3.3										19	11965037	11980381
7165	*ZNF765*	91661	-4.4										19	53389793	53430413
7166	*ZNF766*	90321	-5.4										19	52269571	52296046
7167	*ZNF770*	54989			-3.4								15	34978341	34988287
7168	*ZNF772*	400720	-6.1										19	57466663	57477570
7169	*ZNF773*	374928	-4.3										19	57499915	57518404
7170	*ZNF780B*	163131	-6.8										19	40028260	40056209
7171	*ZNF785*	146540	-4.7										16	30573740	30585771
7172	*ZNF786*	136051	-3.3										7	149069643	149090782
7173	*ZNF788*	388507	-6.4										19	12092263	12137235
7174	*ZNF791*	163049	-3.8										19	12610918	12633840
7175	*ZNF793*	390927	-6.1										19	37506939	37548762
7176	*ZNF799*	90576	-3.6										19	12390016	12401271
7177	*ZNF805*	390980	-5.8										19	57240685	57255135
7178	*ZNF808*	388558	-3.4										19	52527652	52564464
7179	*ZNF81*	347344	-3.4										X	47836902	48002561
7180	*ZNF814*	730051	-5.3										19	57848731	57889074
7181	*ZNF829*	374899	-5.6										19	36888124	36916291
7182	*ZNF835*	90485	-3.6										19	56661981	56671783
7183	*ZNF84*	7637	-3.3										12	133037292	133063304
7184	*ZNF843*	283933						1.5					16	31432593	31443160
7185	*ZNF846*	162993	-4.9										19	9751993	9793180
7186	*ZNF853*	54753	-2.4										4.486772	75023157.5	75097119.67
7187	*ZNF860*	344787				-1.8							3	31981771	31991628

7188	*ZNF878*	729747	-1.8										19	12043805	12052939
7189	*ZNF99*	7652	-4.8										19	22752183	22784107
7190	*ZNFX1*	57169			-2.5								20	49237946	49278426
7191	*ZNHIT1*	10467				-1.7							7	101217668	101224190
7192	*ZNHIT6*	54680	-7.3										X	75020073.5	75094020.98
7193	*ZNRF3*	84133	-2.6										22	28883592	29057487
7194	*ZP4*	57829				-1.8							1	237877864	237890922
7195	*ZSCAN12*	9753	-2.3										6	28378955	28399734
7196	*ZSCAN18*	65982	-2.9										19	58083838	58118427
7197	*ZSCAN20*	7579	-2.8										1	33472645	33496507
7198	*ZSCAN21*	7589	-3.0										7	100049774	100065038
7199	*ZSCAN22*	342945	-6.4										19	58327019	58342332
7200	*ZSCAN25*	221785	-3.8										7	99616946	99632407
7201	*ZSCAN30*	100101467		-1.5									18	35251058	35290245
7202	*ZSCAN32*	54925	-3.6										X	75039948	75113990.33
7203	*ZSCAN9*	7746		-2.1									6	28224886	28233482
7204	*ZSWIM1*	90204	-6.7										20	45881227	45885266
7205	*ZSWIM4*	65249	-2.0										19	13795460	13832230
7206	*ZSWIM7*	125150	-3.4										17	15976560	15999717
7207	*ZSWIM8*	23053	-5.0										10	73785582	73801797
7208	*ZWINT*	11130			4.2								10	56357228	56361275
7209	*ZYG11A*	440590	-3.8										1	52842511	52894998

Supplementary Table S3: CTC isolation and purity confirmation techniques used by the five high-throughput experiments from which differentially expressed genes with fold change values (CTC_FC) was compiled for data analysis in our study.

Cancer	PubMed ID	CTC isolation methods
Prostate	26383955	Miyamoto et al., utilized the microfluidic CTC-iChip technique to isolate CTCs from fresh whole blood after depletion of leukocytes. To check for contamination of leukocytes in the CTC-enriched solution, after processing the whole blood with CTC-iChip, the CTC-enriched product was stained in solution with EpCAM and CDH11 to identify single CTCs and PE-CF594-conjugated antibody against CD45 was used to counterstain contaminating leukocytes, if any. After microscopic screening of blood samples, the stained CTCs were individually micromanipulated on an Eppendorf TransferMan NK2 micromanipulator, transferred into PCR tubes containing RNA protective lysis buffer and flash frozen in liquid nitrogen. Of the 221 single CTCs isolated, 133 single prostate CTCs were successfully processed for next-generation RNA sequencing. Bioinfomatically, stringent expression thresholds were used to define lineage-confirmed CTCs, in order to exclude specimens containing contaminating leukocytes and ensure analysis of bonafide prostate CTCs[1].
Melanoma	22820318	Ramskold et al., isolated single CTCs from peripheral blood of a male patient with recurrent, metastatic melanoma. After lysis and pelleting, the nucleated cells were stained with PE-conjugated anti-human CD45 IgG to label leukocytes. After biotinylation and streptavidin conjugation, the cells were captured based on magnetic sweeping to harvest the beads from cell suspension using the MagSweeper instrument (Illumina Inc.). The harvested cells were stained with 5 µg/ml Calcein AM (Life Technologies, Carlsbad, CA) in HBSS for 20 minutes to identify viable cells. Viable cells that showed desired Calcein-positive/CD45-negative/bead-attached profile were manually picked for molecular profiling. The individual cells were placed into 2.5 µl of Superblock (Thermo Scientific, Waltham, MA) containing 4000 Unit/ml RNase inhibitor (New England Biolabs, Ipswich, MA) and stored at −80 °C until subsequent RNA sequencing by Smart-Seq technique[2].
Breast	25432738	While major advances were seen in rare cell profiling, signatures from leukocytes that introduce potential for bias and false discovery needed to be addressed. Dr. Park's group had developed a FACS protocol that included an immune-magnetic enrichment step to collect rare populations of pure CTCs from peripheral blood for downstream molecular analyses[3]. Using this protocol in a pilot study, Lang et al. demonstrated the ability to isolate pure population of CTCs and profiled CTCs isolated from blood of five metastatic breast cancer patients without evidence of contaminating leukocyte RNA.

		Immunomagnetic separation using EpCAM mAb-coated magnetic beads followed by FACS with EpCAM mAb conjugated to phycoerythrin (PE), thioflavin nucleic acid dye, and CD45 (2D1) mAb conjugated to Cy5-PerCP. A threshold of a single cell meeting these criteria was qualified as a positive test result[4].
Colorectal	22811761	Isolation and molecular characterization of CTCs in metastatic colorectal cancer by performed by Barbazan et al. CTCs were isolated by using the CELLection Epithelial Enrich kit (Invitrogen). After washing, CTCs coupled to magnetic beads were directly resuspended in RNAlaterH solution and frozen until downstream RNA extraction and microarray analysis. Importantly, to ensure that the expression of the selected genes was characteristic of CTC and not an artifact due to variation in gene expression in lymphocytes in the patient samples, their expression was compared in the remaining non-isolated fraction upon CTC enrichment in a set of five mCRC patients and five healthy controls. No differences were found between both groups for eleven candidate genes, thus reinforcing specificity of expression in CTCs[5].
Pancreatic	23157946	Sergeant et al., aimed to develop a negative depletion strategy to enrich CTCs from whole blood without excluding any particular subset of CTCs. The procedure enriches for CTCs by combining gates containing CD45 negative and CD34 negative cells in addition to 7-amino-actinomycin D viability staining to exclude all hematological and non-viable cells. Microarray analysis was performed on all fractions of the isolate. Depletion of dead and non-epithelial cell types (leukocytes-CD45+) and other endothelial/leukocyte progenitor cells (CD34+) ensured unbiased enrichment of CTCs in their spiking and *in vivo* experiments[6].

1 Miyamoto, D. T. *et al.* RNA-Seq of single prostate CTCs implicates noncanonical Wnt signaling in antiandrogen resistance. *Science* **349**, 1351-1356, doi:10.1126/science.aab0917 (2015).

2 Ramskold, D. *et al.* Full-length mRNA-Seq from single-cell levels of RNA and individual circulating tumor cells. *Nature biotechnology* **30**, 777-782, doi:10.1038/nbt.2282 (2012).

3 Magbanua, M. J. & Park, J. W. Isolation of circulating tumor cells by immunomagnetic enrichment and fluorescence-activated cell sorting (IE/FACS) for molecular profiling. *Methods* **64**, 114-118, doi:10.1016/j.ymeth.2013.07.029 (2013).

4 Lang, J. E. *et al.* Expression profiling of circulating tumor cells in metastatic breast cancer. *Breast cancer research and treatment* **149**, 121-131, doi:10.1007/s10549-014-3215-0 (2015).

5 Barbazan, J. *et al.* Molecular characterization of circulating tumor cells in human metastatic colorectal cancer. *PloS one* **7**, e40476, doi:10.1371/journal.pone.0040476 (2012).

6 Sergeant, G., van Eijsden, R., Roskams, T., Van Duppen, V. & Topal, B. Pancreatic cancer circulating tumour cells express a cell motility gene signature that predicts survival after surgery. *BMC cancer* **12**, 527, doi:10.1186/1471-2407-12-527 (2012).

Yadavalli et al., 2016. Data-Driven Discovery of Extravasation Pathway in Circulating Tumor Cells				
**Supplementary Table S4: List of molecular interactions reported in literature (PubMed IDs included) and depicted in the leukocyte/CTC extravasation pathway**				
	**Molecule**	**Molecule**	**Interaction**	**PMID + Pathway Reference ID in parenthesis**
1	VAV	RHOA	Activation	PMID: 9822605 (Ref# 17)
2	VAV	CDC42	Activation	PMID: 12376551 (Ref# 46)
3	VAV	RAC1	Activation	PMID: 9438848 (Ref# 14); PMID: 16782872 (Ref# 21)
4	VAV	RAC2	Activation	PMID: 19858206 (Ref# 80); PMID: 15870290 (Ref# 78)
5	RHOA	ROCK1	Activation	PMID: 17673200 (Ref# 49)
6	ROCK	MYL6	Phosphorylation	PMID: 8702756 (Ref# 10)
7	TEC	PLCG1	Phosphorylation	PMID: 10823839 (Ref# 72)
8	TEC	VAV1	Protein-protein interaction	PMID: 11328862 (Ref# 84); PMID: 7651724 (Ref# 106)
9	PTK2B	VAV	Protein-protein interaction	PMID: 12626562 (Ref# 33)
10	PIK3CA	TEC	Phosphorylation and Activation	PMID: 11123316 (Ref# 76)
11	GNAL	PI3KCA	Activation	PMID: 21308712 (Ref# 23)
12	GNAL	CAMP	Inhibition	PMID: 2869405 (Ref# 88)
13	CAMP	EPAC	Activation	PMID: 9853756 (Ref# 30)
14	EPAC	RAP1	Activation	PMID: 9853756 (Ref# 30); PMID: 9856955 (Ref# 3)
15	DAG	RAP1	Activation	PMID: 9786933 (Ref# 24); PMID: 11043767 (Ref# 95)
16	RAPL	RAP1	Protein-protein interaction	PMID: 17716979 (Ref# 115); PMID: 15143186 (Ref# 58)
17	SIPA1	RAP1	Inhibition	PMID: 10373454 (Ref# 2)
18	RAP1GAP	RAP1	Inhibition	PMID: 15569673 (Ref# 42); PMID: 20124489 (Ref# 26)
19	PIP2	IP3 and DAG	Reaction catalysed by PLCG1	PMID: 1411571 (Ref# 12)
20	IP3	CALCIUM	Reaction catalysed by PLCG1	PMID: 7511641 (Ref# 94)
21	RASGRP1	RAP1	Activation	PMID: 10918068 (Ref# 6)
22	RAPL	RAP1	Protein-protein interaction	PMID: 15143186 (Ref# 58)
23	ITGAL-ITGB2	ICAM1	Protein-protein interaction	PMID: 9670961 (Ref# 9)
24	PECAM1	PECAM1	Protein-protein interaction	PMID: 12610627 (Ref# 11); PMID: 10425179 (Ref# 68)
25	PECAM1	FER	Activation	PMID: 10462517 (Ref# 65)
26	FER	SHP2	Phosphorylation	PMID: 10462517 (Ref# 65)
27	SHP2	CTNNB1	Phosphorylation	PMID: 22956765 (Ref# 114); PMID: 10681592 (Ref# 47)
28	PECAM1	CTNNB1	Protein-protein interaction	PMID: 10462517 (Ref# 65); PMID: 10801826 (Ref# 54)
29	ITGAL/ITGB2	JAM1	Protein-protein interaction	PMID: 11812992 (Ref# 34)
30	ITGAM/ITGB2	JAM3	Protein-protein interaction	PMID: 22415084 (Ref# 20); PMID: 17379836 (Ref# 89)
31	ITGA4/ITGB1	JAM2	Protein-protein interaction	PMID: 12070135 (Ref# 25)
32	ITGA4/ITGB1	VCAM	Protein-protein interaction	PMID: 10626664 (Ref# 44); PMID: 7504895 (Ref# 124)
33	VCAM1	ERM	Protein-protein interaction	PMID: 12082081 (Ref# 7); PMID: 10366600 (Ref# 53)
34	VCAM1	MAP2K2	Activation	PMID: 11123288 (Ref# 71)
35	VCAM1	CALCIUM	Activation	PMID: 14594451 (Ref# 18)
36	VCAM1	RAC1	Activation	PMID: 14594451 (Ref# 18)
37	VCAM1	PI3KCA	Activation	PMID: 11123288 (Ref# 71)
38	PI3K	RAC1	Activation	PMID: 15728722 (Ref# 59); PMID: 11803464 (Ref# 107)
39	CALCIUM	CYBA-NCF1C-NCF2-NOX-NCF4	Activation	PMID: 14594451 (Ref# 18)
40	RAC1	CYBA-NCF1C-NCF2-NOX-NCF4	Activation	PMID: 14594451 (Ref# 18)
41	CYBA-NCF1C-NCF2-NOX-NCF4	H2O2	Activation	PMID: 10843714 (Ref# 8)
42	H2O2	P38MAPK	Activation	PMID: 12700137 (Ref# 86)
43	H2O2	MMP	Activation	PMID: 15265790 (Ref# 87)
44	H2O2	SHP2	Inhibition	PMID: 23582017 (Ref# 69)
45	MMP	CDH5	Molecular cleavage	PMID: 21905018 (Ref# 51); PMID: 9614196 (Ref# 111)
46	CD99	CD99	Protein-protein interaction	PMID: 11812991 (Ref# 118); PMID: 23486730 (Ref# 102)
47	CDH5	CDH5	Protein-protein interaction	PMID: 10428027 (Ref# 28); PMID: 9614196 (Ref# 64)
48	CAM	CAM	Protein-protein interaction	PMID: 17761522 (Ref# 85)
49	CDH5	CTNNA1	Protein-protein interaction	PMID: 9886272 (Ref# 81)
50	CDH5	CTNNB1	Protein-protein interaction	PMID: 15778498 (Ref# 123)
51	CDH5	CTNND1	Protein-protein interaction	PMID: 9434630 (Ref# 117)
52	RAP1	ITGA4/ITGB1	Activation	PMID: 12171996 (Ref# 52)
53	ITGAL/ITGB2	ICAM1	Rho H/Catalyst-Inhibition	PMID: 15300248 (Ref# 37)
54	ICAM1	PLCG1	Activation	PMID: 7540845 (Ref# 31)
55	ICAM1	MAP3K4	Activation	PMID: 11359848 (Ref# 108)
56	IP3	CA 2+	Reaction catalysed by PLCG1	PMID: 7511641 (Ref# 94)

57	DAG	PRKCA	Activation	PMID: 3321305 (Ref# 27)
58	PRKCA	SRC	Activation	PMID: 11925438 (Ref# 104); PMID: 11083862 (Ref# 1)
59	SRC	CTTN	Phosphorylation	PMID: 11084024 (Ref# 57); PMID: 10956618 (Ref# 92)
60	ITGAM/ITGB2	THY1	Protein-protein interaction	PMID: 21264853 (Ref# 35)
61	THY1	RHOGAP	Inhibition	PMID: 15093746 (Ref# 13)
62	PTK2B	ITGAL/ITGB2	Activation	PMID: 12626562 (Ref# 33)
63	RHOGAP	RHOA	Inhibition	PMID: 15093746 (Ref# 13); PMID: 12842009 (Ref# 63)
64	RHOA	ROCK	Activation	PMID: 23029055 (Ref# 16); PMID: 10926873 (Ref# 77)
65	ROCK	MLC1	Phosphorylation	PMID: 10872457 (Ref# 19)
66	ICAM1	RHOA	Activation	PMID: 12097408 (Ref# 38); PMID: 9820557 (Ref# 29)
67	RHOA	PTK2	Phosphorylation	PMID: 17449913 (Ref# 100); PMID: 9603980 (Ref# 97)
68	RHOA	MAPK8	Activation	PMID: 15753034 (Ref# 99)
69	FAK	PXN	Phosphorylation	PMID: 9696039 (Ref# 110); PMID: 7537852 (Ref# 40)
70	RAP1GAP	RAP1	Inhibition	PMID: 15569673 (Ref# 42); PMID: 20124489 (Ref# 26)
71	SIPA1	RAP1	Inhibition	PMID: 10373454 (Ref# 2)
72	MLTT4	RAP1	Protein-protein interaction	PMID: 21429211 (Ref# 67)
73	RAP1	RHOA	Activation	PMID: 15972668 (Ref# 62)
74	CXCR4	SDF1	Protein-protein interaction	PMID: 22220212 (Ref# 48); PMID: 16267013 (Ref# 82);
75	CXCR4	GNAL	Activation	PMID: 10506573
76	CCL2	CCR2	Protein-protein interaction	PMID: 26806351 (Ref# 41); PMID: 15927208 (Ref# 98)
77	CCR2	JAK2	Phosphorylation	PMID: 22789541 (Ref# 121)
78	JAK2	STAT5	Phosphorylation	PMID: 22789541 (Ref# 121); PMID: 15856028 (Ref# 60)
79	JAK2	P38MAPK	Phosphorylation	PMID: 22789541 (Ref# 22)
80	JAK2	PIK3CA	Inhibition	
81	JAK2	STAT3	Inhibition	
82	JAK2	RAC1	Inhibition	
83	ANGPTL4	TGF B1	Protein-protein interaction	PMID: 18394990 (Ref# 36); PMID: 21624123 (Ref# 113)
84	ANGPTL4	ITGB1/ITGA4	Protein-protein interaction	PMID: 21841165 (Ref# 66)
85	RAC1	PAK	Activation	PMID: 9705280 (Ref# 96)
86	PAK	CDH5	Phosphorylation	PMID: 17060906 (Ref# 39)
87	ERM	SELPLG	Protein-protein interaction	PMID: 12115638 (Ref# 61)
88	ERM	ICAM3	Protein-protein interaction	PMID: 11784723 (Ref# 109)
89	ERM	CD44	Protein-protein interaction	PMID: 25979842 (Ref# 32); PMID: 12370738 (Ref# 91)
90	ERM	CD48	Protein-protein interaction	
91	ATP	P2Y2	Activation	PMID: 23810565 (Ref# 50)
92	F-ACTIN	VCL	Protein-protein interaction	PMID: 7816144 (Ref# 73)
93	F-ACTIN	ACTIN	Protein-protein interaction	PMID: 15111441 (Ref# 5)
94	ERM	RAC1	Activation	PMID: 12802084 (Ref# 43)
95	ERM	ICAM1	Protein-protein interaction	PMID: 10366600 (Ref# 53)
96	H202	TIMP	Inhibition	PMID: 15265790 (Ref# 125)
97	TIMP	MMP	Inhibition	PMID: 15265790 (Ref# 125)
98	ICAM1	LFA1	Protein-protein interaction	PMID: 23039186 (Ref# 126)
99	SHP2	CDH	Protein-protein interaction	PMID: 22956765 (Ref# 128)
100	DEL1	LFA1	Inhibition	PMID: 19008446 (Ref# 127)
101	CD44	LGALS3	Protein-protein interaction	PMID: 19055814 (Ref# 4)
102	RAC1	ITGB1/ITGA5	Activation	PMID: 9705280 (Ref# 96)

Yadavalli et al., 2016. Data-Driven Discovery of Extravasation Pathway in Circulating Tumor Cells			
**Supplementary Table S5: PubMed IDs for 98 papers from which a gene list was compiled for molecular characterization of CTCs. This list includes the five high-throughput transcriptomics experiment papers from which differentially expressed genes with fold change values were obtained (highlighted in yellow color).**			
	PubMed IDs	Cancer	Experiment type
1	25880010	Breast cancer	qRT-pCR
2	26571236	Squamous cell lung cancer	FISH and Immunocytochemistry
3	25631749	Colorectal	RT-PCR
4	25623304	Breast	IHC
5	25336717	Breast	Immunostaining with 84-I antibody
6	25218346	Gastrointestinal	Immunofloroscence
7	24752533	Colorectal	QRT-PCR
8	24300972	Hepatocellular carcinoma	qRT-PCR, immunoblotting, IHC
9	24166007	Prostate cancer	Flow cytometry, immunocytochemical (ICC) analyses
10	24091674	Hepatocellular carcinoma	Immunofluorescence staining
11	23372014	Breast cancer	RNA -ISH
12	22264265	Breast cancer	PCR
13	21665936	Prostate and breast cancer	IHC
14	23017820	Epithelial Ovarian cancer	Immunocytochemical analyses and qRT-PCR
15	22745585	Ovarian cancer	µNMR
16	23013138	Melanoma	qRT-PCR
17	23013139	Melanoma	qRT-PCR
18	23013140	Melanoma	qRT-PCR
19	19383356	Colorectal	Immunomagnetic enrichment
20	20156783	Colorectal	Immunomagnetic enrichment
21	20167419	Colorectal	Immunomagnetic enrichment
22	21364588	Colorectal	Immunomagnetic enrichment
23	23695297	Colorectal	Immunospot assay (Epispot)
24	18682708	Colorectal	Flourescence scanning microscope
25	16433139	Colorectal	Flourescence scanning microscope
26	19646840	Colorectal	Flow cytometry
27	12405286	Colorectal	Flow cytometry
28	18097410	Colorectal	Microdevice based, EpCAM
29	21378321	Colorectal	Microdevice based, Cell size 6.5-7.5 um
30	21732137	Colorectal	TCR (RT concerted reaction)
31	19937133	Colorectal	WCHMA (weighted chemiluminescent membrane array), qPCR
32	23145101	Prostate	RNA Seq
33	23280481	Prostate	Single-CellMicrofluidics-BasedRT-PCR
34	23175471	Hepatocellular carcinoma	qRT-PCR
35	23175472	Hepatocellular carcinoma	qRT-PCR
36	23175473	Hepatocellular carcinoma	qRT-PCR
37	23046102	Prostate cancer	qRT-PCR
38	22843916	Prostate cancer	qRT-PCR
39	17226905	Pancreatic cancer	qRT-PCR
40	16136352	Pancreatic cancer	qRT-PCR
41	22591372	Breast cancer	qRT-PCR
42	22586443	Breast cancer	qRT-PCR
43	22527498	Squamous cell carcinoma of the head and neck	Immunostaining/-ve depletion
44	22443102	Breast cancer	RT-PCR
45	26497689	Prostate cancer	RT-PCR
46	25516888	Colorectal cancer	Immunofloroscence and FISH
47	25450039	Prostate	Immunofloroscence
48	25261936	Endometrial cancer	qRT-PCR
49	25182808	Metastatic breast cancer	Immunoflorescence
50	23378342	Colorectal cancer	Fluorescence immunocytochemistry.
51	22538972	Metastatic breast cancer	RNA ISH
52	21663619	Breast cancer	triple-immunofluorescence
53	19589136	Metastatic Breast cancer	RT-PCR
54	21298334	Breast cancer	RT-PCR
55	24808221	Breast cancer	cDNA microarray
56	26505456	NSCLC	RT-PCR
57	26328005	Ovarian cancer	RT-PCR

58	26041877	OSCC	Immunofluorescence and Nanostring
59	26009151	Prostate	RT-PCR
60	25862542	Gastric cancer	RT-PCR
61	25474037	Lung cancer	RT-PCR
62	24932314	Breast cancer	RT-PCR
63	24649289	Metastatic Breast cancer	qRT-PCR
64	24599131	Colorectal cancer	Single cell aCGH
65	24058517	Metastatic Breast cancer	qRT-PCR
66	23686807	Breast cancer	RT-PCR
67	23136247	Colorectal cancer	WGA/FISH
68	22783565	Adenocarcinoma (hepatopancreatic, bile duct, breast, cardia, colon esophagus, pancreas, rectum, small intestine, stomach)	RT-PCR
69	22384245	Breast cancer	Microarray
70	21967632	Breast cancer	RT-PCR
71	21827674	Ovarian carcinoma, endometrial carcinoma, uterine-cervix carcinoma	RT-PCR
72	21129172	Breast, endometrial, cervical, and ovarian cancers	qRT-PCR
73	21505063	Metastatic Breast cancer	RT-PCR
74	21245367	Breast cancer	Liquid bead array hybridization
75	18949363	Colorectal cancer	RT-PCR
76	18262345	Metastatic Breast cancer and metastatic colorectal cancer	RT-PCR
77	17649779	Colorectal cancer	RT-PCR
78	17649771	Breast cancer	RT-PCR
79	16646576	Breast cancer	RT-PCR
80	15958538	Prostate cancer	qRT-PCR
81	23538216	Metastatic breast cancer	Immunostaining
82	23576814	Breast cancer	Immunoflorescence/FISH
83	26328272	Small cell lung cancer SCLC	Western blot
84	23845299	Prostate	Ferromagnetic antibody capture, floroscent imaging
85	22228641	Breast cancer	Immunostaining
86	25962645	Breast cancer	Single-cell triple immunofluorescence laser scanning microscopy and image analysis.
87	25719830	Prostate	Immunofluorescence (IF) staining and FISH
88	24917030	Breast cancer	Western blot
89	25242334	PDAC (pancreatic cancer)	RNA-seq
90	26008969	Breast cancer	DNA FISH
91	22553466	Metastatic breast cancer	RT-PCR
92	26967453	Metastatic pancreatic cancer	Immunomagnetic enrichment
93	26923772	Prostate cancer	Immunostaining
94	26383955	Prostate	RNA-Seq
95	22820318	Melanoma	RNA-Seq
96	23157946	Pancreatic	cDNA microarray
97	22811761	Colorectal	cDNA microarray
98	25432738	Breast	cDNA microarray

Yadavalli et al., 2016. Data-Driven Discovery of Extravasation Pathway in Circulating Tumor Cells							
**Supplementary Table S6: EMT scores computed from transcriptome datasets of CTCs in five cancers—GSE18670, GSE31023, GSE38495, GSE45965, and GSE67980. The pre-processed data GSE45965 was also used**							
	Sample	Cohort	Description	Label (T: original tumor, G: haematological cell, P: Non-tumoral pancreatic control tissue)	Patient code	EMT.p-value	EMT.score
1	GSM463723	GSE18670	T117	PDAC_T_117	117	0.00	0.22
2	GSM463724	GSE18670	P117	PDAC_P_117	117	0.32	0.12
3	GSM463725	GSE18670	G117	PDAC_G_117	117	0.16	0.16
4	GSM463726	GSE18670	CTC117	PDAC_CTC_117	117	0.02	0.23
5	GSM463727	GSE18670	T104	PDAC_T_104	104	0.24	0.10
6	GSM463728	GSE18670	P104	PDAC_P_104	104	0.02	0.24
7	GSM463729	GSE18670	G104	PDAC_G_104	104	0.33	0.12
8	GSM463730	GSE18670	CTC104	PDAC_CTC_104	104	0.25	0.14
9	GSM463731	GSE18670	T105	PDAC_T_105	105	0.00	0.20
10	GSM463732	GSE18670	P105	PDAC_P_105	105	0.00	0.55
11	GSM463733	GSE18670	G105	PDAC_G_105	105	0.07	0.19
12	GSM463734	GSE18670	CTC105	PDAC_CTC_105	105	0.24	0.14
13	GSM463735	GSE18670	T91	PDAC_T_91	91	0.07	0.13
14	GSM463736	GSE18670	P91	PDAC_P_91	91	0.00	0.34
15	GSM463737	GSE18670	G91	PDAC_G_91	91	0.23	0.14
16	GSM463738	GSE18670	CTC91	PDAC_CTC_91	91	0.05	0.20
17	GSM463739	GSE18670	T138	PDAC_T_138	138	0.00	0.25
18	GSM463740	GSE18670	P138	PDAC_P_138	138	0.00	0.65
19	GSM463741	GSE18670	G138	PDAC_G_138	138	0.40	0.11
20	GSM463742	GSE18670	CTC138	PDAC_CTC_138	138	0.23	-0.14
21	GSM463743	GSE18670	T123	PDAC_T_123	123	0.32	-0.08
22	GSM463744	GSE18670	P123	PDAC_P_123	123	0.00	0.31
23	GSM463745	GSE18670	G123	PDAC_G_123	123	0.56	0.09
24	GSM463746	GSE18670	CTC123	PDAC_CTC_123	123	0.02	0.23
